# Expedient Assembly of Multiantennary *N*-Glycans from Common *N*-Glycan Cores with Orthogonal Protection for the Profiling of Glycan-Binding Proteins

**DOI:** 10.1021/jacs.5c02356

**Published:** 2025-04-07

**Authors:** Ruofan Li, Pengxi Chen, Yi-Fang Zeng, Tzu-Hao Tseng, Veeranjaneyulu Gannedi, Larissa Krasnova, Chi-Huey Wong

**Affiliations:** 1 Department of Chemistry, The Scripps Research Institute, 10550 N. Torrey Pines Road, La Jolla, California 92037, United States; 2 Genomics Research Center, Academia Sinica, Taipei 11529, Taiwan

## Abstract

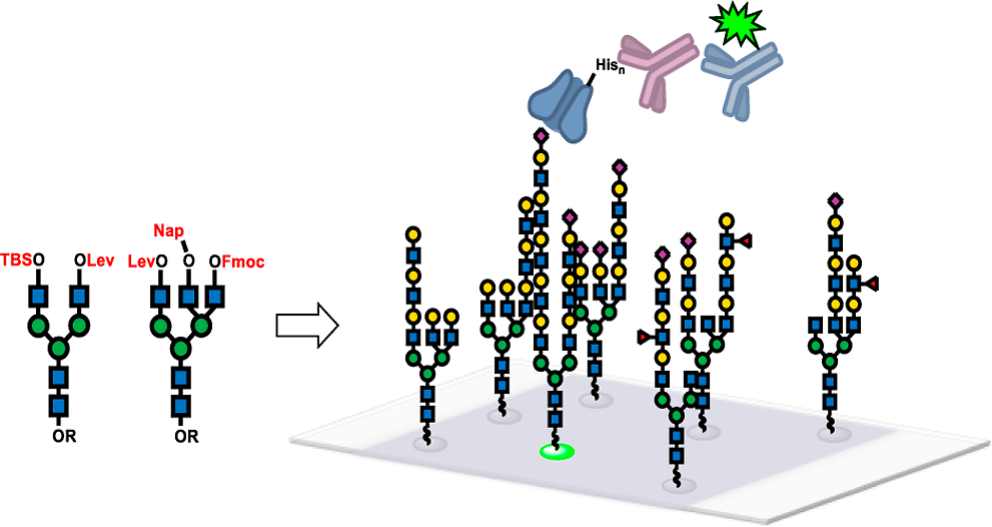

Complex-type *N*-glycans are structurally diverse molecules, responsible for many biological processes, yet the specific sequences of *N*-glycans involved in biological recognition remain largely unknown. Despite the recent development of many efficient chemoenzymatic approaches, it is still lacking a general approach to produce structurally diverse complex-type *N*-glycans. Here, we designed two common precursors equipped with orthogonal protecting groups for antennary differentiation and selective glycan elongation. The *N*-acetyllactosamine (LacNAc) repeat modules were synthesized separately based on iterative Au(I) promoted glycosylation and programmable one-pot strategy and were incorporated into the *N*-glycan core structure in a site-specific manner. The final removal of benzyl groups was cleanly achieved using pressurized flow chemistry. A total of 51 *N*-glycans were assembled and presented as an array to study the binding specificity toward a panel of influenza hemagglutinins and other lectins. The established method allows a rapid and previously infeasible synthesis of asymmetric bi- and triantennary *N*-glycans, especially with the LacNAc repeats residing at a specific arm, bringing in new opportunities to study carbohydrate–receptor interactions.

## Introduction

**Figure 1:**
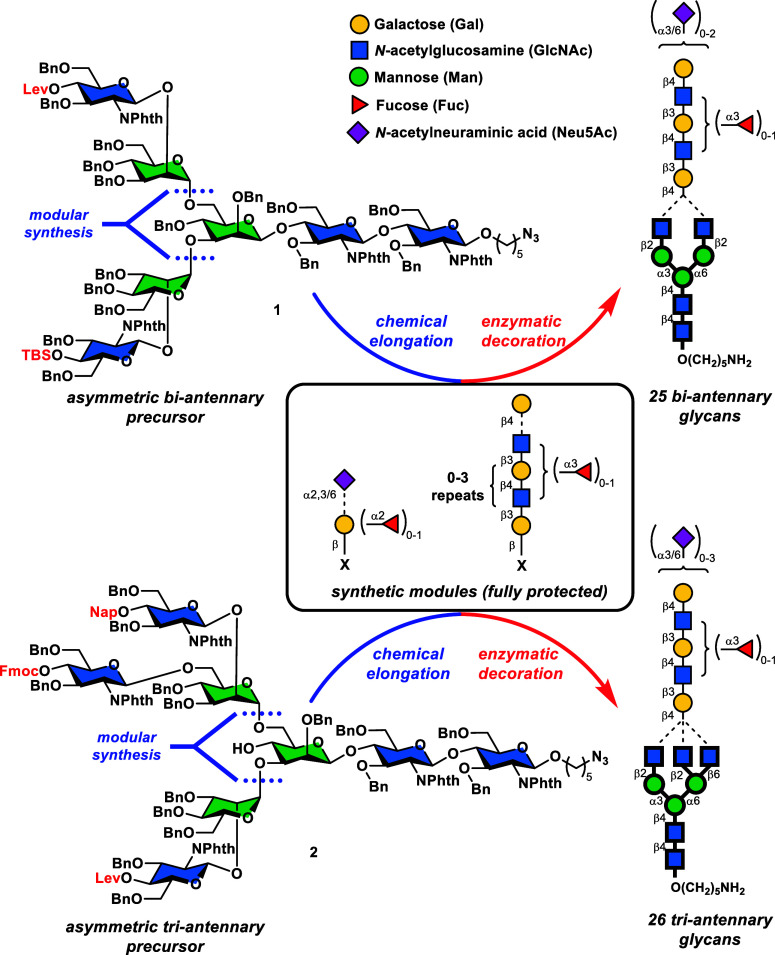
General approach to asymmetric bi- and triantennary *N*-glycans: synthetic precursors **1** and **2** with orthogonally protected GlcNAc units and modules are coupled to give a diverse array of *N*-glycan intermediates that are further elaborated by enzymatic synthesis.

Protein glycosylation is a complex post-translational process that affects the structure and function of a protein. Understanding the role of complex glycans in glycoproteins and their association with the development of diseases is of great importance to human health. However, glycoproteins exist as mixtures of glycoforms, and it is almost impossible to isolate homogeneous glycoproteins and the attached glycans, particularly the multiantennary *N*-glycans for the study of their functions. Specifically, sialylated and/or fucosylated multiantennary *N*-glycans containing one or several LacNAc repeats have been generally considered as epitopes and ligands for a wide variety of glycan-binding proteins, such as plant lectins, galectins, asialoglycoprotein receptors (ASGPRs), selectins, influenza hemagglutinins (HAs), sialic acid-binding immunoglobulin-type lectins (Siglecs), etc.^1^ However, the exact sequences of these epitopes and the location of the LacNAc repeats as well as the underlined detailed binding mechanisms remain largely unknown. This information is crucial for understanding binding specificities and the development of diagnostics and prophylactic measures. Despite the recent advances in glycomics and studies that have provided insightful knowledge about the structural diversity of *N*-glycans,^2^ the extreme heterogeneity of glycosylation patterns from tissue to tissue makes it impossible to use isolated samples for in vitro experiments.^3^ To advance the research on these topics, structurally well-defined and diverse *N*-glycan samples, as well as efficient and generalizable methods of *N*-glycan synthesis are greatly required. Previous endeavors in this area include the common precursor and the stop-and-go strategies developed by the Boons group,^4,5^ the programmable and modular synthesis methods reported by our group,^6^ and the automated solid-phase glycan assembly pursued by the Seeberger group.^7^ These methods generated many *N*-glycan molecules with varying degrees of complexity. Nonetheless, each method has certain limitations in producing a repertoire of highly elaborated, elongated *N*-glycan structures. For example, the overall efficiency of the modular synthesis decreased with elongated, complex mannosyl fluorides, making it difficult to produce relatively large *N*-glycan structures, the enzymatic elongation method was limited to the specificity and availability of enzymes, and the solid-phase method has not been well developed for the assembly of complex-type *N*-glycans. Herein, we report an optimized modular strategy that we believe to be a generalizable approach to complex-type *N*-glycans (Figure 1). Compared to the original report,^6^ practicality was significantly improved by redesigning the core precursors (**1**, **2** in Figure 1) with orthogonal protecting groups for modular chemical assembly, optimizing procedures for iterative synthesis of long LacNAc sequences and programmable one-pot synthesis of LacNAc repeats with or without fucosylation, as well as improving global deprotection procedure by utilizing a high-pressure flow reactor. Once a diverse set of chemically synthesized structures was obtained, subsequent enzymatic steps were used to increase diversity even further by introducing terminal Gal, Fuc, or Neu5Ac or removing internal Fuc residues. Our optimized method is an efficient and general approach toward highly complex structures, such as an icositrisaccharide with two sialylated polyLacNAc chains of different lengths and an asymmetric triantennary decaoctasaccharide with fucosylation and sialylation at specific sites. We believe that the method could be extended to even larger structures without a significant loss in yield. As highlighted in Figure 1, the synthesis of a total of 51 bi- and triantennary *N*-glycans is described in this report, a number that could certainly increase given the modular nature of our strategy.

## Results and Discussion

### Construction of Bi- and Triantennary Core Structures with Orthogonal Protecting Groups at Terminal *N*-Acetylglucosamine (GlcNAc) Residues

Biosynthetically, the complex-type *N*-glycans are mainly matured in trans-Golgi where galactosyltransferases, fucosyltransferases, and sialyltransferases are enriched.^8^ Regardless of the complexity of the final structures, their GlcNAc-terminated precursors, such as G0 and [G0 + GN] (Figure 1, glycan structures in bold), serve as common intermediates for bi- and triantennary *N*-glycans, respectively. Yet the natural elongation of G0 or [G0 + GN] by UDP-Gal and galactosyltransferases lack a mechanism for distinguishing different arms (i.e., MGAT1, 2, 4, and 5 arms, or GnT-1, 2, 4, 5 arms), leading to a mixture of antennary isomers.^9^ To prepare the structurally well-defined complex-type *N*-glycans, we employed a set of orthogonal protecting groups at the GlcNAc-4OH positions on the fully protected G0 (**1**) and [G0+GN] (**2**) structures. As illustrated in Figure 1, the design of biantennary heptasaccharide **1** and triantennary octasaccharide **2** took advantage of the orthogonal protecting groups at terminal GlcNAc-4OH and the use of highly reactive galactosyl donors as modules, allowing the pending glycosylation reactions to proceed smoothly.^10^ Further elaborations on the chemically assembled *N*-glycan intermediates by UDP-Gal, GDP-Fuc, CMP-Neu5Ac, and related glycosyltransferases efficiently furnished a larger variety of *N*-glycan products. Since the complexity of *N*-glycans mainly arises from terminal modification, this approach provides an efficient way for chemical or enzymatic elongation of glycan chains from a specific arm of the bi- and triantennary cores (**1** and **2**), leading to *N*-glycan structures with broad chemical space.

**Scheme 1:**
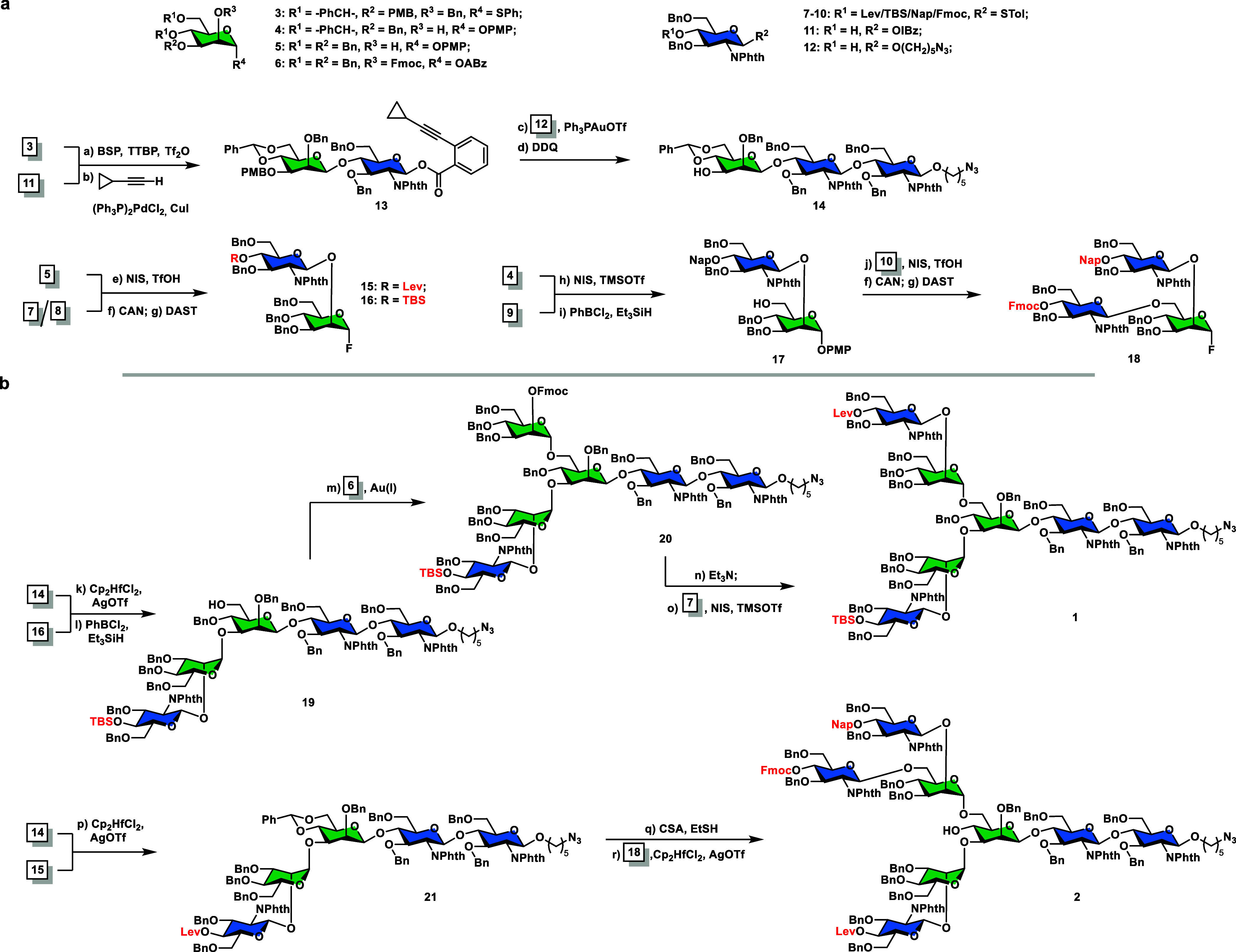
Rapid Assembly of Common Precursors **1** and **2** Using Mannosyl and GlcNPhth BBLs Reagents and conditions: (a) **3** (1.6 equiv), BSP (1.6 equiv), TTBP (3.2 equiv), Tf_2_O (1.6 equiv), CH_2_Cl_2_, 4 Å MS, −60 °C, 5 min; then **11** (1.0 equiv), −60 → −20 °C, 1 h, 57%; (b) cyclopropyl acetylene (5.0 equiv), (Ph_3_P)_2_PdCl_2_ (0.1 equiv), CuI (0.2 equiv), DMF/Et_3_N = 1:3 (v/v), r.t., 24 h, 90%; (c) **12** (1.1 equiv), Ph_3_PAuOTf (0.2 equiv), CH_2_Cl_2_, 4 Å MS, 0 °C, 30 min, 78%; (d) DDQ (1.2 equiv), CH_2_Cl_2_:H_2_O = 10:1 (v/v), 0 °C, 6 h, 82%; (e) **5** (1.0 equiv), **7** (1.05 equiv) or **8** (1.1 equiv), NIS (1.2 equiv), TfOH (0.1 equiv), 4 Å MS, CH_2_Cl_2_, −50 → 0 °C, 30 min, 64% for Lev-disaccharide, 83% for TBS-disaccharide; (f) CAN (3.0 equiv), PhMe:ACN:H_2_O (1:4:1, v/v/v), 0 °C, 1 h; (g) DAST (2.0 equiv), 4 Å MS, CH_2_Cl_2_, −40 °C, 30 min, 72% for **15** over the two steps, 75% for **16** over the two steps, 69% for **18** over the two steps; (h) **4** (1.0 equiv), **9** (1.1 equiv), NIS (1.2 equiv), TMSOTf (0.1 equiv), 4 Å MS, CH_2_Cl_2_, −40 → 0 °C, 30 min; (i) PhBCl_2_ (2.0 equiv), Et_3_SiH (5.0 equiv), 4 Å MS, CH_2_Cl_2_, −78 °C, 2 h, 70% over the two steps; (j) **17** (1.0 equiv), **10** (1.25 equiv), NIS (1.25 equiv), TfOH (0.15 equiv), 4 Å MS, CH_2_Cl_2_, −40 → 0 °C, 30 min, 82%; (k) **14** (1.0 equiv), **16** (1.2 equiv), Cp_2_HfCl_2_ (3.5 equiv), AgOTf (5.0 equiv), 4 Å MS, PhMe, −60 → −30 °C, 1 h, 70%; (l) PhBCl_2_ (2.0 equiv), Et_3_SiH (5.0 equiv), 4 Å MS, CH_2_Cl_2_, −78 → −60 °C, 2 h, 78%; (m) **19** (1.0 equiv), **6** (3.0 equiv), Ph_3_PAuOTf (0.5 equiv), 4 Å MS, CH_2_Cl_2_, 0 °C, 80%; (n) Et_3_N:CH_2_Cl_2_ = 1:10 (v/v), r.t., 12 h; (o) **20** (1.0 equiv), **7** (3.0 equiv), NIS (3.0 equiv), TMSOTf (0.6 equiv), 4 Å MS, CH_2_Cl_2_, −40 → 0 °C, 54% for the two steps; (p) **14** (1.0 equiv), **15** (1.1 equiv), Cp_2_HfCl_2_ (3.5 equiv), AgOTf (5.0 equiv), 4 Å MS, PhMe, −60 → −20 °C, 1 h, 53%; (q) CSA (5.0 equiv), EtSH (10.0 equiv), CH_2_Cl_2_:MeOH (20:1, v/v), r.t., 12 h, 59% (26% recovered S.M.); and (r) **18** (1.5 equiv), Cp_2_HfCl_2_ (3.5 equiv), AgOTf (5.0 equiv), 4 Å MS, PhMe, −20 °C, 1 h, 72% combined yield (α:β *ca*. 1.5:1). BSP = 1-(phenylsulfinyl)piperidine, TTBP = 2,4,6-tri*tert*-butylpyrimidine, DMF = *N*,*N*-dimethylformamide, DDQ = 2,3-dichloro-5,6-dicyano-*p*-benzoquinone, NIS = *N*-iodosuccinimide, CAN = ammonium cerium(IV)nitrate, DAST = (diethylamino)sulfur trifluoride, MS = molecular sieves, TMSOTf = trimethylsilyl trifluoromethanesulfonate, and CSA = camphor-10-sulfonic acid.

As summarized in Scheme 1, common precursors **1** and **2** are composed of only mannose (Man) and GlcNAc residues, and therefore, only two types of building blocks (BBLs) are needed for the construction of these two intermediates. Mannosyl BBLs **3–6** and *N*-phthalimido-glucosaminyl BBLs **7–12** were conveniently synthesized from readily available starting materials (see the Supporting Information for details). As shown in Scheme 1a, first, PMB-protected phenylthio mannoside **3** was coupled with *o*-iodobenzoate **11** under the Crich glycosylation conditions,^11^ followed by a Sonogashira coupling of cyclopropyl acetylene on the aryl iodide handle, affording disaccharide **13** that featured an *o*-alkynylbenzoate auxiliary for Yu’s glycosylation.^12^ Under the influence of the Au(I) catalyst, *o*-alkynylbenzoate **13** was activated at 0 °C and reacted with the linker attached GlcNPhth acceptor **12**, furnishing the chitobiose core trisaccharide **14** after PMB removal (DDQ) in 64% yield over the two steps. In parallel, the required GlcNPhth-Man-F and GlcNPhth_2_-Man-F modules with different protecting groups were assembled in a straightforward manner as depicted in Scheme 1a. As such, tribenzylated mannosyl acceptor **5** was glycosylated with levulinoyl (Lev) and *tert*-butyldimethylsilyl (TBS)-protected GlcNPhth-STol donors **7** and **8**, providing the corresponding GlcNPhth-Man-OPMP disaccharides, which were then elaborated to mannosyl fluorides **15** and **16** by a two-step sequence (CAN; DAST). Similarly, bifurcated trisaccharide donor (GlcNPhth)_2_-Man-F **18** was constructed from benzylidene acceptor **4** and GlcNPhth-STol donors **9** and **10** in 40% overall yield in five steps.

Inspired by the modular strategy developed by our group,^6,13^ we attempted to employ a tandem Man-Man coupling to form the key Man-α1,3-(Man-α1,6-)Man linkage empowered by Mukaiyama–Suzuki glycosylation.^14^ As outlined in Scheme 1b, chitobiose core **14** with exposed Man-3-OH was glycosylated with TBS-protected GlcNPhth-Man-F donor **16** in the presence of Cp_2_HfCl_2_/AgOTf, affording pentasaccharide in 70% yield. The C6-OH of the latter compound was then released by a regioselective benzylidene ring opening reaction mediated by Et_3_SiH/PhBCl_2_^15^ and the resulting alcohol **19** was subjected to the second Man-Man coupling with Fmoc-protected mannosyl *o*-alkynylbenzoate donor **6** in the presence of Au(I) species, producing hexasaccharide **20** in 80% yield with 100% α-selectivity. It was noted that direct coupling of **19** with Lev-GlcNPhth-Man-F donor **15** failed, presumably due to the poor nucleophilicity of primary alcohol **19** caused by the nearby steric hindrance. Finally, removal of the Fmoc group by Et_3_N followed by NIS/TMSOTf promoted glycosylation with Lev-protected GlcNPhth-STol donor **7** afforded biantennary heptasaccharide precursor **1** in 54% yield in the last two steps. The triantennary octasaccharide precursor **2** was synthesized in a similar fashion except that the benzylidene moiety (as shown in **21**, Scheme 1b) on the central mannose residue was cleaved by CSA/EtSH to release both C4- and C6-OHs followed by a regioselective glycosylation with trisaccharide donor (GlcNPhth)_2_-Man-F **18** under the Mukaiyama–Suzuki conditions to provide the desired (GlcNPhth)_3_(Man)_3_(GlcNPhth)_2_ octasaccharide **2** in 72% combined yield (α:β ca. 1.5:1). The unmasked C4–OH on the central Man remained inert during later-stage manipulations and was thus exempt from protection.

### Synthesis of LacNAc Repeats with or without Fucosylation

**Scheme 2:**
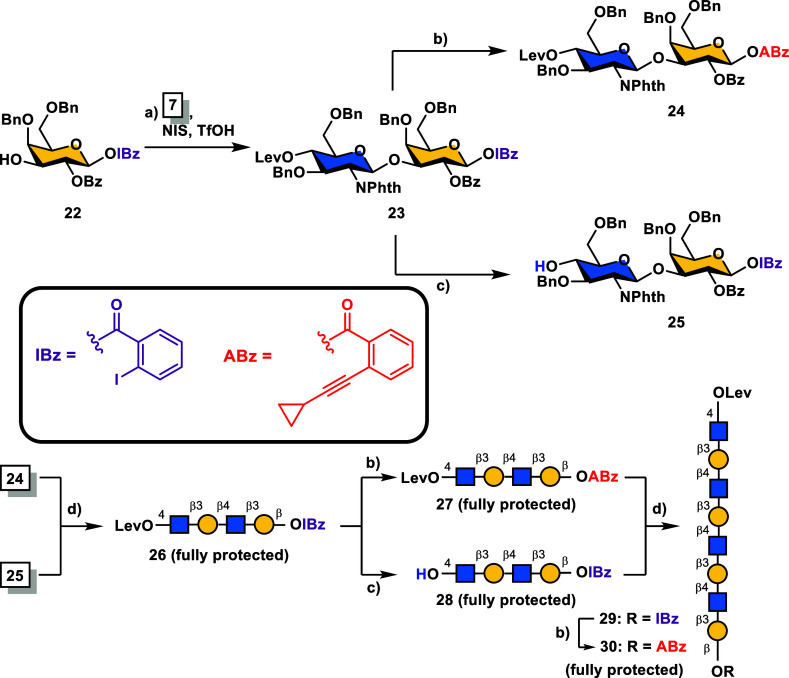
An Iterative Strategy for the Synthesis of LacNAc Repeats Reagents and conditions: (a) **7** (1.1 equiv), **22** (1.0 equiv), NIS (1.2 equiv), TMSOTf (0.1 equiv), 4 Å MS, CH_2_Cl_2_, −50 °C, 1 h, 72%; (b) cyclopropyl acetylene (5.0 equiv), (Ph_3_P)_2_PdCl_2_ (0.1 equiv), CuI (0.2 equiv), DMF:Et_3_N (1:4, v/v), r.t., 24 h, 90% for **24**, 82% for **27**, 94% for **30**; (c) N_2_H_4_·AcOH (2.0 equiv), CH_2_Cl_2_/MeOH (20:1, v/v), r.t., 12 h, 88% for **25**, 90% for **28**; and (d) Ph_3_PAuOTf (0.25 equiv), 4 Å MS, CH_2_Cl_2_, 0 °C, 82% for **26**, 82% for **29**.

With the success in the core constructions, we next moved forward to the assembly of (fucosylated) polyLacNAc modules (cf. Figure 1 in the middle box) for antennary elongations. Type-II LacNAc repeats are common structural motifs in various *O*- and *N*-glycans and glycolipids^8^ and are potent ligands for plant lectins and galectins and for influenza HAs when capped with sialylation.^16^ It was known in the early studies that different galectins responded differently to Type-II polyLacNAc ligands with variable lengths.^17^ Recent studies also revealed that the length of LacNAc repeats affected the HA binding processes.^5b,18,19^ In the biochemical pathway, the assembly of a polyLacNAc motif relies on the stepwise, alternating introduction of Gal and GlcNAc residues by UDP-Gal/β4GalT1 and UDP-GlcNAc/β3GnT2, resulting in heterogeneous mixtures with different lengths. Therefore, a concise and robust method for the generation of polyLacNAc motifs with a well-defined length and structure is highly desired. We envisaged to use a bifunctional “A-GlcNPhth-Gal-B” disaccharide unit to generate donor and acceptor disaccharide BBLs, through the cleavage and/or modification of groups A and B directly, followed by coupling of the donor and acceptor to give the dimeric LacNAc, A-(GlcNPhth-Gal)_2_-B. Levulinic ester served best for this purpose at the nonreducing end due to easy removal under mild conditions, while the *o*-iodobenzoate (IBz), which is a precursor for *o*-alkynylbenzoate (ABz) auxiliary,^12^ served as a versatile mask at the Gal reducing end. To minimize the undesired orthoester formation and acyl migration during glycosylation, benzoyl rather than the acetyl group was selected for C2–OH protection on the Gal residue.^20^ As shown in Scheme 2 (vide infra), the starting disaccharide Lev-GlcNPhth-Gal-IBz (**23**) was synthesized in 72% yield from GlcNPhth-STol (**7**) and galactosyl *o*-iodobenzoate (**22**, see the Supporting Information for the synthesis) using an NIS/TfOH promoter. The resulting product was split into two portions: Sonogashira coupling of **23** with cyclopropylacetylene in the presence of catalytic Pd(II)/Cu(I) gave donor Lev-GlcNPhth-Gal-ABz (**24**) in 90% yield and treatment of **23** with hydrazine acetate afforded acceptor HO-GlcNPhth-Gal-IBz (**25**) in 88% yield. Coupling of donor **24** and acceptor **25** by the Au(I)-catalyzed glycosylation reaction produced the Lev-(GlcNPhth-Gal)_2_-IBz tetrasaccharide fragment **26** in 82% yield. To our delight, tetrasaccharide **26** was smoothly elongated to octasaccharide **29** by applying another round of Sonogashira coupling (82% for **27**), Lev cleavage (90% for **28**), and Au(I)-catalyzed glycosylation (82% for **29**) procedure without significant loss of efficacy.

Fucose residues are commonly found on the LacNAc moiety of *N*-glycans. However, the relationship between their biological functions and their specific locations on the polyLacNAc chain is not clear. Such studies are largely hampered by the lack of well-defined fucose-containing glycan standards. Among the hitherto discovered seven noncore α1,3-fucosyltransferases (human FuTs 3, 4, 5, 6, 7, 9 and *H. pylori* FuT A), only human FuT 7 has a strict substrate specificity (i.e., it only uses α2,3-sialylated Type-II polyLacNAc glycans and catalyzes α1,3-fucosylation on the GlcNAc residue closest to the terminal α2,3-sialoside).^21^ But its nonsecretory, membrane-bound nature prevents its wide use for the enzymatic synthesis of fucosylated glycans.^22^ To selectively block enzymatic fucosylation at GlcNAc, the unnatural GlcNH_2_/GlcNHBoc^23^ analogues and Gal-*O*6-sialosides^24^ have been utilized. However, the required lengthy steps to ensure the fucosylation at desired positions make these procedures impractical at the late-stage modification of complex-type *N*-glycans, especially those with a low degree of fucosylation. Under these circumstances, chemical synthesis serves as an alternative way to generate specific and diverse fucosylated polyLacNAc structures.

**Scheme 3:**
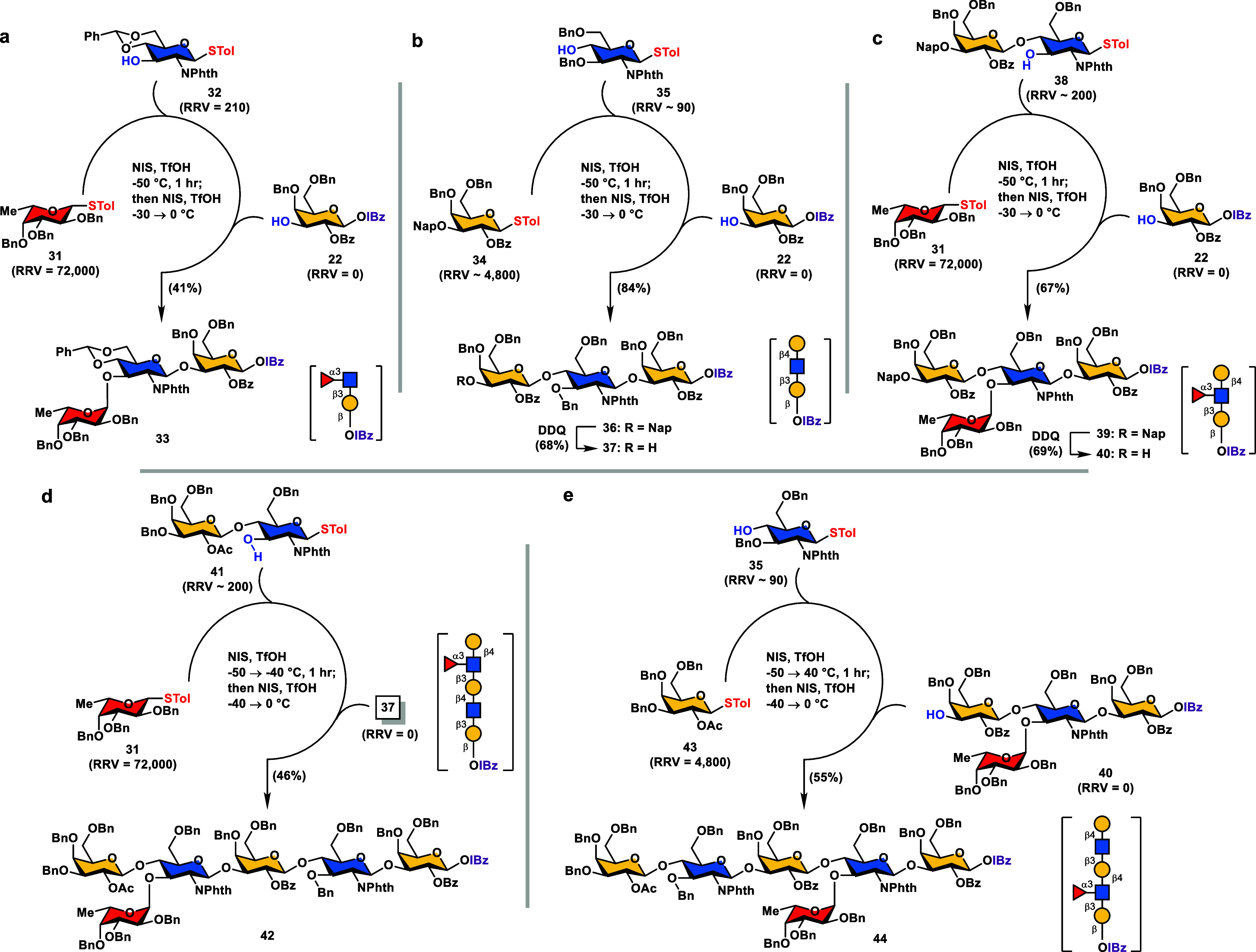
Programmable One-Pot Synthesis of (Fucosylated) LacNAc Modules **33**, **36**, **39**, **42**, and **44** Reagents and conditions: (a) **31** (1.1 equiv), **32** (1.0 equiv), NIS (1.08 equiv), TfOH (0.15 equiv), 4 Å MS, CH_2_Cl_2_, −50 → −40 °C, 1 h; then **22** (0.9 equiv), NIS (1.1 equiv), TfOH (0.15 equiv), −40 → 0 °C, 30 min, 41% overall. (b) (i) **34** (1.1 equiv), **35** (1.0 equiv), NIS (1.08 equiv), TfOH (0.15 equiv), 4 Å MS, CH_2_Cl_2_, −50 → −40 °C, 1 h; then **22** (0.9 equiv), NIS (1.1 equiv), TfOH (0.15 equiv), −40 → 0 °C, 30 min, 84% overall; (ii) DDQ (2.0 equiv), CH_2_Cl_2_/H_2_O (10:1, v/v), 0 °C, 4 h, 68%. (c) (i) **31** (1.2 equiv), **38** (1.0 equiv), NIS (1.18 equiv), TfOH (0.15 equiv), 4 Å MS, CH_2_Cl_2_, −50 → −40 °C, 1 h; then **22** (1.0 equiv), NIS (1.2 equiv), TfOH (0.15 equiv), −40 → 0 °C, 30 min, 67% overall; (ii) DDQ (1.2 equiv), CH_2_Cl_2_/H_2_O (10:1, v/v), 0 °C, 6 h, 69%. (d) **31** (1.1 equiv), **41** (1.0 equiv), NIS (1.1 equiv), TfOH (0.15 equiv), 4 Å MS, CH_2_Cl_2_, −50 → −40 °C, 1 h; then **37** (0.9 equiv), NIS (1.1 equiv), TfOH (0.15 equiv), −40 → 0 °C, 30 min, 46% overall. (e) **43** (1.1 equiv), **35** (1.0 equiv), NIS (1.1 equiv), TfOH (0.15 equiv), 4 Å MS, CH_2_Cl_2_, −50 → −40 °C, 1 h; then **40** (0.9 equiv), NIS (1.1 equiv), TfOH (0.15 equiv), −40 → 0 °C, 30 min, 55% overall.

The programmable one-pot oligosaccharide synthesis developed by our group^10,25^ was thus explored as a suitable method for this purpose. According to the published relative reactivity values (RRVs, either predicted or measured), the tribenzylfucosyl STol donor has the largest RRV (RRV = 72,000), followed by 2-acyl-3,4,6-tribenzylgalactosyl STol donor (RRVs ∼4000–5000) and then GlcNPhth-STol donor (RRVs ≈ 100–200).^10,26^ The remarkable gaps between the three different types of STol donors promise a successful implementation of a one-pot procedure into our synthesis. As illustrated in Scheme 3 (vide infra), five glyco-sequences were conveniently constructed using this approach, including Fuc-GlcNAc-Gal (**33**), LacNAc-Gal (**36**), Le^x^-Gal (**39**), Le^x^-LacNAc-Gal (**42**), and LacNAc-Le^x^-Gal (**44**), in moderate to good overall yields. The *o*-iodobenzoate handle in each synthetic module was then elaborated into the *o*-alkynylbenzoate glycosylation auxiliary using the same Sonogashira coupling procedures as those presented in Scheme 2.

### Elongation of Bi- and Triantennary Cores with Chemical and Enzymatic Approach

**Figure 2:**
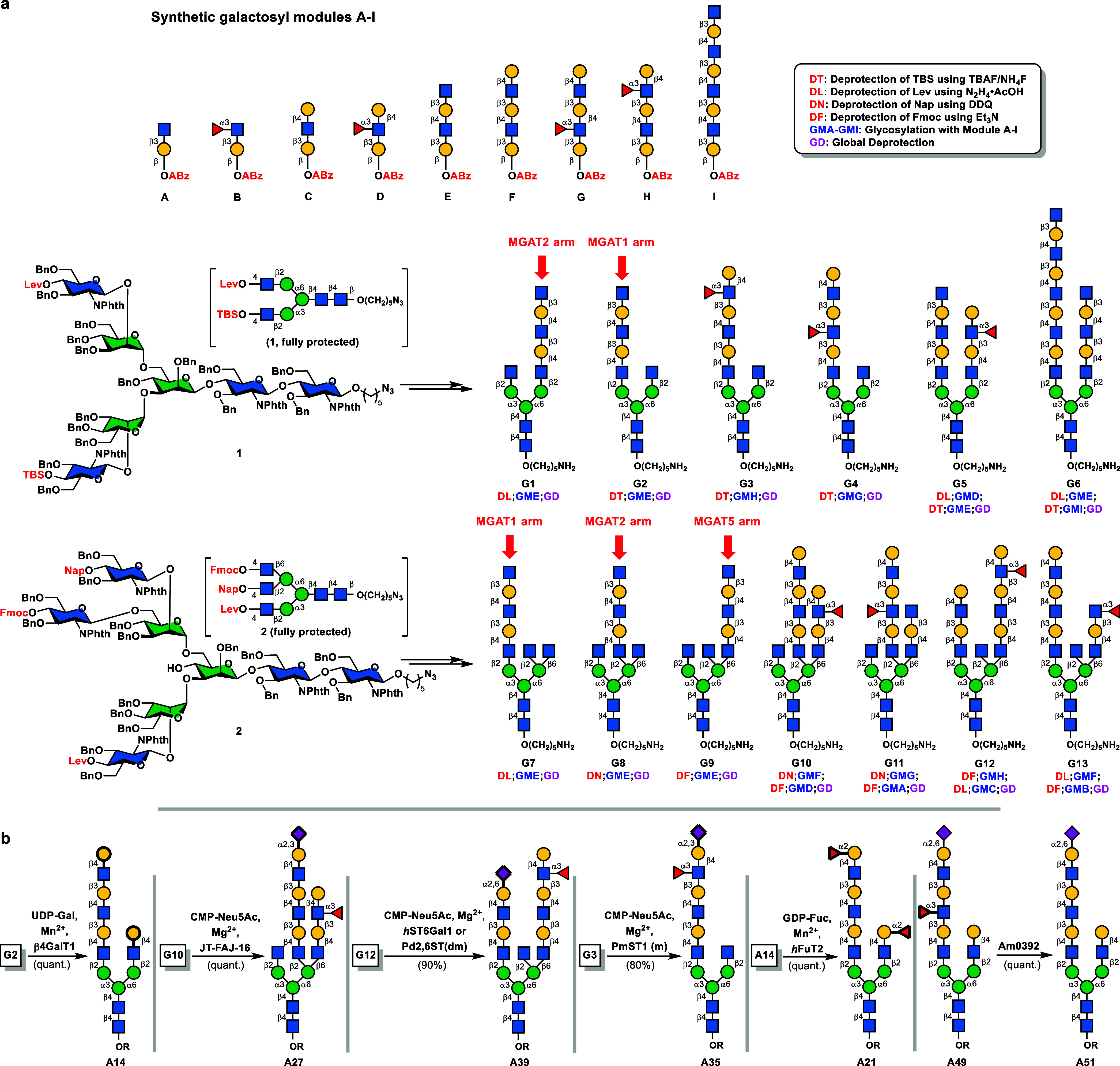
(a) Chemical elongation of common precursors **1** and **2** using galactosyl donors **A**-**I**. (b) Representative enzymatic derivatizations of synthetic bi- and triantennary *N*-glycans.

With biantennary and triantennary core structures **1** and **2**, and synthetic modules **A**–**I** in hand, our next task was to elongate the specific arm(s) with galactosyl donors and finalize the synthesis via global deprotection. In order to ensure the site-specificity for glycosylation, only one protecting group was removed at a time followed by the coupling of the exposed GlcNPhth-C4-OH with a proper galactosyl *o*-alkynylbenzoate donor. As summarized in Figure 2a, a total of 13 *N*-glycans were assembled using a deprotection-glycosylation protocol, each containing at least two LacNAc units at a specific antenna. The global deprotection of the resulting *N*-glycans were accomplished by a standard four-step sequence: (1) BuOH/EDA, (2) Ac_2_O/pyridine, (3) NaOMe, and (4) Pd(OH)_2_/C, H_2_,^27^ rendering the final protecting group free *N*-glycans (**G1**–**G13**) in 55–60% overall yield. The final debenzylation step presented as a major challenge toward the target compounds, as normal pressure hydrogenolysis using commercially available Pd catalysts always yielded a significant amount of oversaturated byproducts (i.e., the *O*-benzyl group was hydrogenated to *O*-cyclohexylmethyl group).^28^ It was reported by the Carson group that pretoxifying the Pd catalysts with acidified DMF solution could largely enhance the hydrogenolysis selectivity by suppressing the undesired hydrogenation (ring saturation) pathway.^27^ However, this procedure also resulted in longer reaction time (>7 days) as the overall catalyst activity was reduced. By deploying a pressurized flow setup enabled by an H-Cube Mini apparatus with a DMF/HCl pretreated Pd(OH)_2_/C cartridge,^29^ we were able to obtain the desired debenzylated product with a low oversaturation rate and a total reaction time <3 h on a 10 mg scale.

With diverse glycan modules installed on the bi/triantennary *N*-glycan cores as illustrated in Figure 2a, further enzymatic glycosylation of the deprotected *N*-glycans provided even larger structural diversity and more importantly, allowing the introduction of necessary functionality (i.e., terminal Gal, Fuc, or Neu5Ac) to study the binding specificities of lectins, influenza HAs, and other proteins of interest. Figure 2b exemplifies some terminal modifications on chemically synthesized *N*-glycan structures **G1**–**G13** by enzymes. The GlcNAc residues at both antennae of asymmetric biantennary glycan **G2** (or **A4** in the array) were capped by Gal via an enzymatic galactosylation (UDP-Gal, *B. taurus* β4GalT1, quantitative yield) and the asymmetric triantennary glycan **G10** (**A10**), bearing a tri-LacNAc moiety on its MGAT2 arm and a Le^x^ terminal on its MGAT5 arm, was site-specifically α2,3-sialylated at the Gal residue using an α2,3SiaT from *Vibrio* sp. bacterium JT-FAJ-16.^30^ It is noted that some Le^x^ was also sialylated under these conditions, albeit to a very limited extent (<5%). Such ambiguity was completely avoided when α2,6-sialyltransferases were employed. As such, triantennary glycan **G12** (**A20**) was cleanly converted into **A39** in the presence of CMP-Neu5Ac, Mg^2+^, and human ST6Gal1,^31^ with the Le^x^ motif remaining completely intact. The same result could also be obtained by using a recently engineered α2,6-sialyltransferase from *Photobacterium damselae* [Pd2, 6ST(A200Y/S232Y)], which only recognizes the terminal Gal residue on a polyLacNAc chain.^32^ As shown in Figure 2b, the Le^x^ end within the biantennary structure **G3** (**A18**) was specifically α2,3-sialylated by PmST1(M144D) and in this case sialyl-Le^x^**A35** was produced in 80% yield.^33^ Other than Neu5Ac, the Gal end could also be modified to a blood group H (Type II) antigen featuring a Fuc(α1,2)Gal linkage, as shown in the bis-α1,2-fucosylation of **A14** with GDP-Fucose/human FuT2.^34^ Last but not the least, the α1,3-fucose residue on the inner GlcNAc unit could be removed by an *endo* α1,3-fucosidase from *Akkermansia muciniphila*,^35^ which allows a direct comparison between a fucosylated glycan of interest and its afucosylated counterpart (e.g., **A49** vs **A51** in Figure 2b).

### Assembly of Glycan Microarrays to Study Binding Specificity

**Figure 3:**
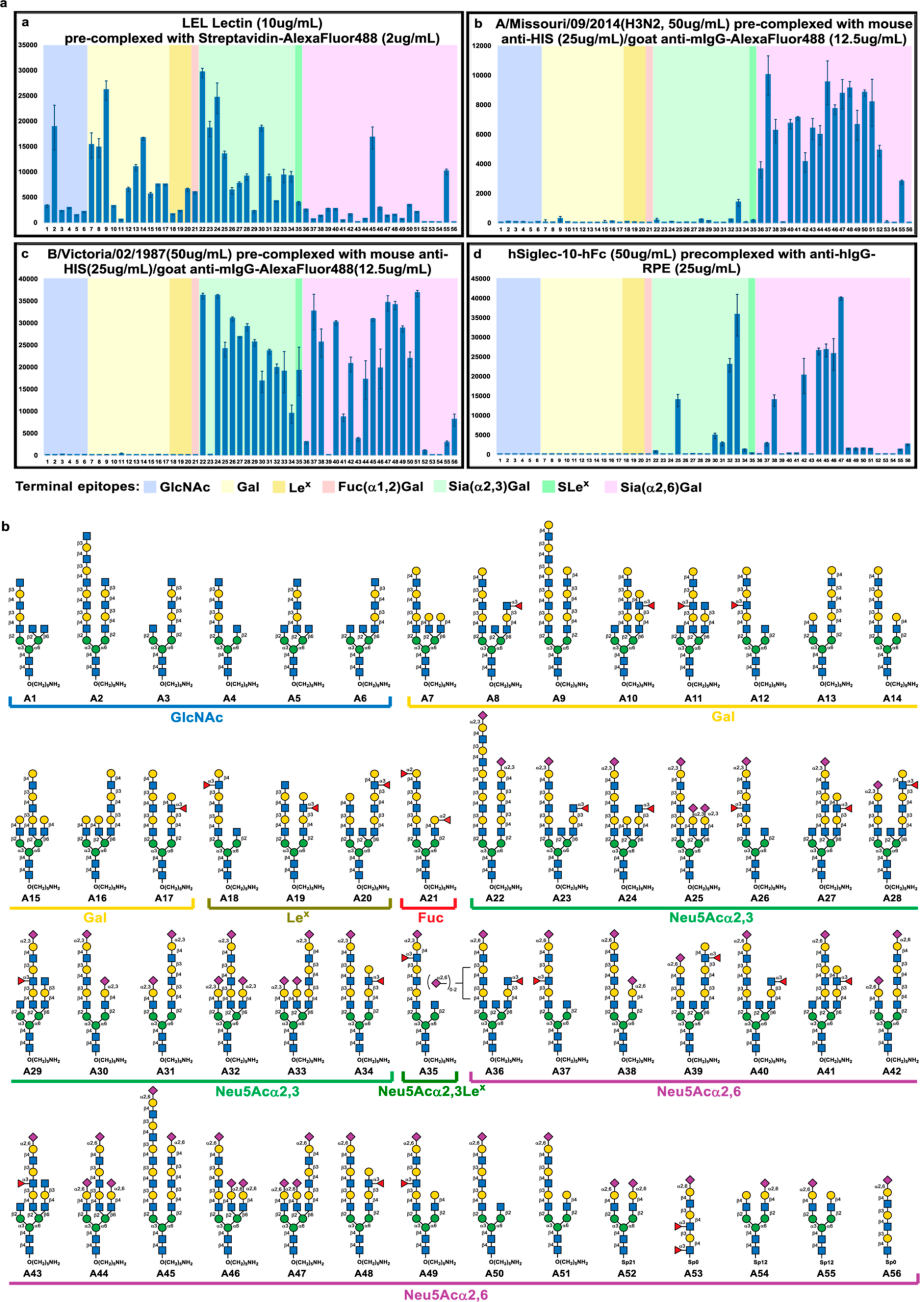
(a) Representative microarray binding results from a plant lectin, influenza Has, and human Siglec-10. (b) *N*-glycans used in the array.

Glycan microarrays have been widely used to study the multivalent interactions between glycans and proteins of interest. Herein, we prepared a glycan microarray of the newly synthesized *N*-glycans using the established arraying method to screen a panel of lectins to understand their binding specificity toward complex-type *N*-glycans with LacNAc repeat located at a specific antenna. A total of 51 polyLacNAc bearing *N*-glycans were synthesized (see Figure 3b and S1 in Supporting Information for the complete array)^36^ in this study and these *N*-glycans, with various epitopes [GlcNAc, Gal, Fuc(α1,2)Gal, Le^x^, Neu5Ac(α2,3)Gal, Neu5Ac(α2,3)Le^x^, and Neu5Ac(α2,6)Gal] at the terminal ends, were printed on NHS-coated glass slides together with another five short mono- and biantennary glycans from the Consortium for Functional Glycomics (the CFG).^37^ The resulting microarray was incubated with a series of precomplexed plant lectins (*Sambucus nigra* lectin, SNA; *Ricinus communis* agglutinin I, RCA-I; *Erythrina cristagalli* lectin, ECA; and *Lycopersicon esculentum* lectin, LEL), human influenza HAs (H1N1/California/04/2009, H1N1/Wisconsin/67/2022, H3N2/Missouri/09/2014, IVB/Victoria/02/1987, and IVB/Yamagata/16/1988), and human Siglec-10 and the incubated slides were washed, dried, and scanned at λ = 488 or 532 nm. As displayed in Figures S2–S5 (see Supporting Information), SNA specifically bound to α2,6-sialosides rather than any other functionalities at the nonreducing end.^37−39^ RCA-I bound to terminal Gal well, yet this affinity was compromised with a Neu5Ac(α2,3)- or a Neu5Ac(α2,6)-linkage to the Gal unit and was largely diminished by introducing a terminal GlcNAc(α1,3)- or a Fuc(α1,3)- residue to the penultimate GlcNAc (to form a Le^x^ epitope as seen in **A18** and **A19**).^37−40^ At this point, we felt necessary to link the structural features with the detected fluorescence using statistical tools to better extract (and at the same time not to overinterpret) detailed information from the massive data. This could be done by assigning each feature a numeric value followed by establishing a Pearson correlation between a certain feature and fluorescence intensity, if its *t*-test passes (see the Supporting Information for details). The major factors that significantly affect the binding (*p* < 0.1) were summarized in Table 1 (vide infra). Like RCA-I, ECA also preferred Gal at the non-reducing end (with *r* = 0.83 and *p* < 0.0001), yet all further terminal modifications, including Neu5Ac(α2,3)-, Neu5Ac(α2,6)-, Fuc(α1,2)Gal, and Le^x^, prevented ECA from binding to the glycan.^37−39^ In contrast, LEL seemed to recognize all available polyLacNAc motifs, with or without the terminal modifications [e.g., GlcNAc(α1,3)-, Fuc(α1,2)-, Neu5Ac(α2,3)-, and Neu5Ac(α2,6)-],^37,39^ with α2,6-sialosides having the weakest binding among all (*p* = 0.004). From Table 1, we also learned that LEL recognized MGAT1 antenna better on an asymmetric triantennary *N*-glycan (as seen from **A7** vs **A15**/**16**, **A25** vs **A32**/**33**, **A46** vs **A44**/**47** in Figure 3a, with *p* = 0.004), and it preferred long polyLacNAc chain (e.g., **A45** vs **A38**, **42** vs **A52**, with *p* < 0.0001), a similar trend also observed with RCA and ECA but not SNA. Among the four tested plant lectins, only LEL’s binding capability was significantly impaired by introducing an internal fucose residue (or an internal Le^x^) to a polyLacNAc chain, with *p* ≈ 0.05. It was reported that certain human influenza strains like H1N1 and H3N2 had adapted to recognize long LacNAc repeats instead of the short sequences over the last several decades,^18^ and such tendency was reversed in recent years.^5b,19^ This phenomenon was also noticed in our assay toward the two H1N1 HAs. As illustrated in Figures S6–S8 and summarized in Table 1, A/California/04/2009 (H1N1) had a much stronger proclivity to polyLacNAc-containing glycans (*p* < 0.01) than that of a more recent H1N1 strain, A/Wisconsin/67/2022 (*p* > 0.1). Interestingly, all the three pandemic-causing influenza HAs favored the MGAT1 arm for binding (e.g., **A38** vs **A42**, **A54** vs **A55**),^5b^ although the correlation for A/California/04/2009 HA was not significant judged by the relatively high *p*-value (*p* > 0.1). While α1,3-fucosylation on the internal GlcNAc unit negatively affected the HA binding, such impact was generally trivial (e.g., **A37** vs **A50**, **A49** vs **A51**, with *p*-values >0.1) for all three cases, indicating the structural modification only induced minor confirmational change which was also supported by GLYCAM force-field energy minimization.^41^

**Table 1:**
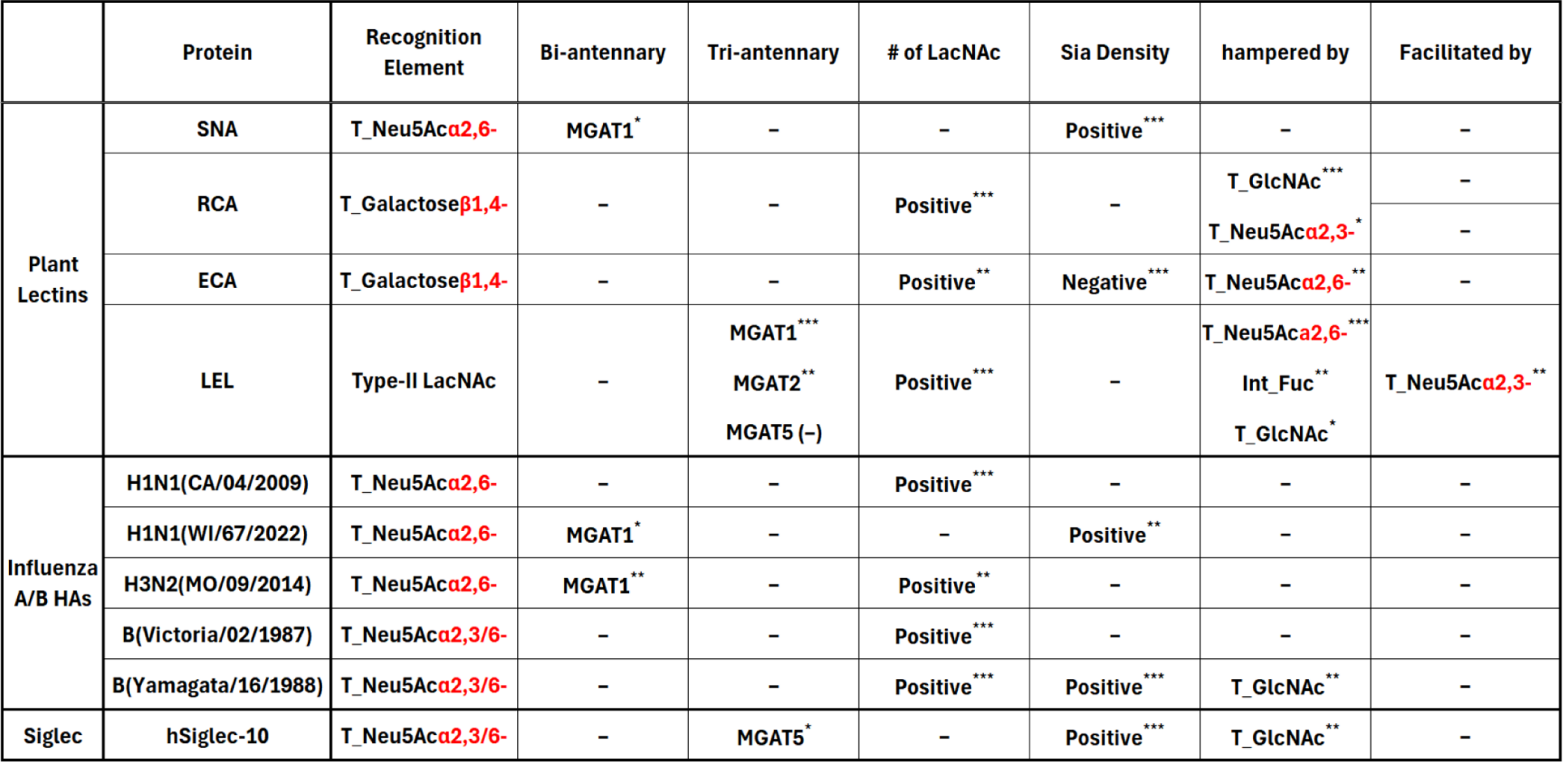
Summary of Factors That Affect the Protein Bindings toward *N*-Glycans^a^^b^

a Pearson correlation coefficients (*r*-values) were calculated between each factor and fluorescence intensities. The significance was judged by *p*-values generated from the *t*-tests.

b Abbreviations: T_: terminal; Int_: internal.

c *p* < 0.1; **p < 0.05; ***p < 0.01; “–”: no significance observed.

HAs from the two classified influenza B lineages, B/Victoria and B/Yamagata,^42^ were also tested using the same array. Both B/Victoria/02/1987 and B/Yamagata/16/1988 HAs displayed strong bindings to almost all of the sialosides in the array, while their binding profiles were quite different (Figures 3a, S9–S10). Unlike the H1N1 and H3N2 HAs, the influenza B HAs exhibited no apparent structural preferences toward human [Neu5Ac(α2,6)Gal] vs. avian [Neu5Ac(α2,3)Gal] ligands, or fucosylated vs afucosylated polyLacNAc chains, or bi-vs tri antennary structures as frequently noticed with influenza A HAs, LEL, and SNA.^43^ However, both influenza B HAs recognized long polyLacNAc sequence much better than the shorter ones (e.g., **A22** vs **A30**/**31**, **A45** vs **A38**/**42**/**52** in Figures 3a and S10, with *p* = 0.0016 and 0.0010, respectively), and as a distinct characteristic for B/Yamagata/16/1988 HA, its binding affinity seemed to be very relevant to the number of Neu5Ac residues on a glycan (*p* = 0.00016). Notably, compound **A39**, an asymmetric triantennary *N*-glycan featuring a Neu5Ac(α2,6)LacNAc_2_ on the MGAT1 antenna and a long asialo-Le^x^LacNAc_2_ chain on the MGAT5 branch, showed almost no binding to any of the five human influenza A/B HAs tested. This could be explained by the interfering effect where the real epitope was hindered by a longer and nonresponsive glycan sequence immobilized on a 2D glass slide. Nevertheless, the fact that **A39** was still well recognized by plant lectins SNA and RCA, and the fact that its α2,3-sialylated counterpart **A28** exhibited excellent binding only toward B/Victoria/02/1987 HA, implied possible distinctive binding modes of these proteins to the short sequences under our microarray assay conditions.^44^

Human Siglec-10 (paralog of murine Siglec-G), a sialic acid-binding Ig like protein, has been reported to have Neu5Ac(α2,6)LacNAc or the corresponding *N*-glycolyl motif (Neu5Gc(α2,6)LacNAc) as its primary epitope.^45^ In our assay, we found that it also accepted Neu5Ac/Gc(α2,3)LacNAc well (Figure 3a). Just like B/Yamagata/16/1988 HA, hSiglec-10 displayed a binding profile heavily dependent on the Neu5Ac density. As a result, bisiaylated and trisialylated glycans (e.g., **A22**, **A25**, **A30**, **A31–33**, **A38**, **A42**, **A44–47**) were generally good binders for hSiglec-10. Further data analysis revealed a minor but clear preference for MGAT5 antenna among the asymmetric triantennary trisialosides (i.e., **A33** and **A47**). As all human Siglec proteins discovered so far only have one active binding site in the V-type domain for one Neu5Ac (or Neu5Gc) residue, the observed unique binding preference of hSiglec-10 toward densely sialylated asymmetric multiantennary *N*-glycans may provide some novel understandings and insights into Siglec protein–sialoglycan interactions on a structural basis.

### Conclusions

In summary, we have described an efficient method for a rapid and previously infeasible synthesis of asymmetric complex-type *N*-glycans with a high degree of structural complexity, including fucosylation and sialylation modifications on the LacNAc and its repeats on different antennae. While enzymatic elongation is limited to the specificity and availability of enzymes, this approach is complementary to the enzymatic approach and more general than the existing methods with the following advantages: (1) the elongation modules (the donors) containing LacNAc repeats and fucose motifs can be efficiently assembled using programmable one-pot strategy; (2) elongation is site-specific and different branches will not interfere with each other; and (3) the elongation modules can be used repeatedly on different antennae. These advantages allowed us to rapidly generate a structurally diverse array of *N*-glycans that were previously inaccessible via other methods. The protecting strategy and the large chemical space described in this study have not been achieved otherwise and the new products are useful for efficiently profiling the proteins of interest. The exploitation of orthogonal protecting groups (in this case Lev–Nap–Fmoc) and programmable one-pot assembly of oligosaccharide modules, together with continuous flow hydrogenolysis for global debenzylation, allows batch production of advanced glycan structures with broad diversity. The resulting *N*-glycans were employed to probe various proteins of interest including plant lectins, influenza A/B HAs, and human Siglecs using the glycan microarray technology. The unique *N*-glycans structures synthesized in this work provided more detailed information on protein binding/recognition events for the first time as demonstrated in the glycan array analysis, which could be valuable for a better understanding of the underlying mechanisms of glycan–protein interactions in the recognition processes of many glycan-binding proteins.

## Supporting Information Available

Synthetic and experimental procedures, characterization of compounds, glycan microarray assays, and NMR data (PDF)
