# Supplementary material for: Expedient Assembly of Multiantennary N-Glycans from Common N-Glycan Cores with Orthogonal Protection for the Profiling of Glycan-Binding Proteins
Source: J Am Chem Soc. 2025 Apr 7;147(15):12937–48. doi: 10.1021/jacs.5c02356 (PMC12006998; doi:10.1021/jacs.5c02356)
Supplement: Supplementary file 1 — ja5c02356_si_001.pdf [file ja5c02356_si_001.pdf]

Expedient Assembly of Multi-antennary *N*-glycans from Common *N*-Glycan Cores with Orthogonal Protection for the Profiling of Glycan-binding Proteins

Ruofan Li,[1] Pengxi Chen,[1] Yi-Fang Zeng,[1] Tzu-Hao Tseng,[1] Veeranjaneyulu Gannedi,[1]

Larissa Krasnova,[1] Chi-Huey Wong[1,2]

[1]Department of Chemistry, The Scripps Research Institute, 10550 N. Torrey Pines Rd., La Jolla, CA 92037, United States; [2]Genomics Research Center, Academia Sinica, Taipei 11529, Taiwan

Table of Contents

General Information **2**

General Procedures **3**

Experimental Procedures and Characterization of Compounds **8**

Scheme S1. Synthesis of Mannoside Building Blocks **3**–**6** 8

Scheme S2. Synthesis of *N*-Phthalimido-glycosamine Building Blocks **7**–**12** 11

Scheme S3. Synthesis of $ManGlcNPhth_2$ Core 14

Scheme S4. Synthesis of Mannosyl Fluoride Donors **15**, **16** and **18** 17

Scheme S5. Assembly of Heptasaccharide Common Precursors **1** 22

Scheme S6. Assembly of Octasaccharide Common Precursors **2** 26

Scheme S7. Assembly of LacNAc Repeat Module **30** by an Iterative Strategy 30

Scheme S8. Assembly of Trisaccharide Modules Using Programmable One-pot Strategy 37

Scheme S9. Assembly of Tetrasaccharide and Hexasaccharide Modules 40

Scheme S10. Assembly of Asymmetric Bi-antennary *N*-Glycans from Precursor **1** and *O*-Alkynylbenzoate Modules A-I 48

Scheme S11. Assembly of Asymmetric Tri-antennary *N*-Glycans from Precursor **2** and *O*-Alkynylbenzoate Modules A-I 56

Figure S1. *N*-glycans used for microarray assays (sorted according to the terminal epitopes) 66

Table S1. Summary of preparation, ^{1}H NMR and LRMS data of **A1**–**A51** 67

Microarray Assays **86**

Table S2. Summary of proteins used in microarray assay 86

Figure S2–S11. SNA, RCA-I, ECA, LEL, HA (H1N1, A/California/04/2009), HA (H1N1, A/Wisconsin/67/2022), HA (H3N2, A/Missouri/09/2014), HA (B/Victoria/02/1987), HA (B/Yamagata/16/1988), Human Siglec-10 binding to the glycan array 88

Table S3. Summary of factors that affect the protein bindings towards *N*-glycans 98

Figure S12. Comparison between conformations of fucosylated/afucosylated *N*-glycan pairs **A26**/**A50** and **A49**/**A51** 99

Reference **100**

NMR Spectra **102**

General Information

All the organic reactions were performed under inert atmosphere (nitrogen or argon) using anhydrous solvents unless otherwise noted. All chemicals were purchased as reagent grade and used without further purification. Anhydrous dichloromethane (CH_2Cl_2), acetonitrile (CH_3CN), *N*,*N*-dimethyl formamide (DMF), toluene, methanol (MeOH), tetrahydrofuran (THF), *tert*-butanol (^{t}BuOH), *n*-butanol (^{n}BuOH) were purchased from commercial sources and were used without further distillation. Pulverized molecular sieves MS-3Å, MS-4Å and MS-5Å for glycosylation reactions were activated by flaming drying under high vacuum. All the reactions were monitored by analytical thin-layer chromatography (TLC) using silica gel 60 F254 plates and visualized under UV (254 nm) and/or by acidic ceric ammonium molybdate (CAM) or *p*-anisaldehyde. Flash chromatography was performed on silica gel (Fischer Chemical or Silicycle Inc.) of 40-63 µm particle size. Size exclusive chromatography was performed on P2 gel (Bio-rad, 45-90 µm) or Sephadex G-25 (Cytiva, superfine). ^{1}H NMR spectra were recorded on a Bruker AV NEO 500 MHz NMR and Bruker AVIII HD 600 MHz NMR spectrometers at 25 °C. All ^{1}H chemical shifts were calibrated using solvent residue peaks of $CDCl_3$ (δ = 7.26 ppm, s), MeOD (δ = 3.31 ppm, quint) and D_2O (δ = 4.79 ppm, s). ^{13}C NMR spectra were acquired on Bruker AVIII HD 600 MHz NMR spectrometer at 25 °C and all ^{13}C chemical shifts were calibrated using deuterated standards $CDCl_3$ (δ = 77.16 ppm, t) and MeOD (δ = 49.00 ppm, hept). Coupling constant values (*J* values) are reported in hertz (Hz). Splitting patterns are described using the following abbreviations: s, singlet; brs, broad singlet; d, doublet; t, triplet; q, quartet; quint, quintet; hept, heptet; dd, doublet of doublet; m, multiplet. High resolution ESI mass spectra were recorded on an Agilent 6230B time-of-flight LC/MS (LC/TOF) spectrometer.

All the enzymes used in this study, including *Bos taurus* galactosyltransferase (β4GalT1)[1], Neisseria meningitidis CMP-sialic acid synthetase (*Nm*CSS)[2], *Vibrio* sp. bacterium (JT-FAJ-16) α2,3-sialyltransferase[3], *Pasteurella multocida* α2,3-sialyltransferase M144D mutant (PmST1 M144D)[4], Photobacterium sp. (JT-ISH-224) α2,6-sialyltransferase[5], human α2,6-sialyltransferase (ST6Gal1)[6], *Photobacterium damselae* α2,6-sialyltransferase double mutant (Pd2,6ST A200Y/S232Y)[7], human α1,2-fucosyltransferase (FuT2)[8] and *Akkermansia muciniphila* α1,3-fucosidase (Am0392)[9] were expressed and purified as reported in the previous literatures.

General Procedures

I. **NIS/TfOH promoted glycosylation:** a mixture of tolyl thioglycoside donor (1.25 – 3.0 equiv., depending on the reaction), acceptor (1.0 equiv.) and flame-dried 4Å molecular sieves (100 mg/mL) in CH_2Cl_2 (final concentration of donor ~0.08 M) were stirred at −40 °C for 30 min before NIS (1.25 – 3.0 equiv., depending on the reaction) and TfOH (0.125 – 0.3 equiv., depending on the reaction) were sequentially added. The reaction mixture was slowly warmed up to 0 °C over the course of 30 min and then quenched by saturated aq. $NaHCO_3/Na_2S_2O_3$. The resulting mixture was then diluted with CH_2Cl_2 and H_2O and extracted with CH_2Cl_2 (20 mL × 3). The organic phases were combined, dried over Na_2SO_4, filtered and concentrated under vacuum. The resulting residue was purified by flash chromatography (silica gel) using proper eluent to obtain clean glycoside product.

II. **Sonogashira Coupling:** to a solution of glycosyl *o*-iodobenzoate (1.0 equiv.) in a mixed solvent of Et_3N:DMF (3:1 *v*/*v*, final concentration of substrate ~50 mg/mL) were sequentially added $(Ph_3P)_2PdCl_2$ (0.1 equiv.) and CuI (0.2 equiv.) at room temperature. The dark yellow-green solution was bubbled with argon continuously for 15 min while stirring. Cyclopropyl acetylene (5.0 equiv.) was added and the resulting dark red solution was stirred for another 24 hrs. All the volatiles were removed under vacuum and the black residue was purified by flash chromatography (silica gel) using proper eluent to acquire clean glycosyl *o*-alkynylbenzoate.

III. **Gold(I) promoted glycosylation:** to a flame-dried flask under argon were sequentially added AgOTf (51.4 mg, 0.200 mmol, 1.0 equiv.) and Ph_3PAuCl (99.0 mg, 0.200 mmol, 1.0 equiv.) and the white mixture was stirred under vacuum for 15 min before CH_2Cl_2 (4.0 mL) was added under argon. The flask was then covered with aluminum foil and the suspension continued stirring at room temperature for another 2 hrs. The resulting slightly purplish suspension was allowed to sediment for 15 min and the supernatant, which contained ~0.05 M $Ph_3PAuOTf$, was ready to be used for the pending Au(I)-promoted glycosylation reactions. The Au(I)-containing solution so acquired was active at room temperature for at least 4 hrs with avoidance of light. A mixture of *o*-alkynyl benzoate donor (1.2 to 3.0 equiv., depending on the reaction), acceptor (1.0 equiv.) and flame-dried 4Å molecular sieves (100 mg/mL) in CH_2Cl_2 (final concentration of acceptor: 0.02 M) were stirred at 0 °C for 30 min before a solution of $Ph_3PAuOTf$ (*ca.* 0.05 M in CH_2Cl_2, 0.2 to 1.0 equiv., depending on the reaction) was added. The resulting mixture was allowed to stir at 0 °C for another 30 min before it was directly subjected onto a flash column (silica gel). The desired product was purified using proper eluent.

IV. **PMP Ether Cleavage by CAN Oxidation:** to a solution of glycosyl PMP ether (1.0 equiv.) in a mixed solvent of toluene, acetonitrile and H_2O (1/4/1, *v*/*v*/*v*) at 0 °C was slowly added an aqueous solution of CAN (3.0 equiv., 1.0 M in ddH_2O) over 1 h. The orange reaction mixture was stirred at the same temperature for another 10 min before it was diluted with H_2O and EtOAc. The organic phase was separated, dried over Na_2SO_4 and concentrated under vacuum. The resulting residue was purified by flash chromatography using proper eluent.

V. **Deoxyfluorination of Lactol:** to a suspension of lactol (1.0 equiv.) and 4Å MS (100 mg per 1 mL solvent) in CH_2Cl_2 (final substrate concentration ~40-50 mg/mL) at −40 °C was slowly added DAST (2.0 equiv.). The reaction mixture was allowed to stir at the same temperature for 30 min and then filtered through Celite®. The filtrate was washed with saturated aqueous $NaHCO_3$, dried over Na_2SO_4 and concentrated under vacuum. The residue was purified on a short silica gel column using proper eluent to afford desired glycosyl fluoride.

VI. **Programmable One-pot Synthesis of Oligosaccharides:** to a mixture of the most reactive tolyl thioglycoside donor (with the largest RRV, 1.1 equiv.), hydroxy tolyl-thioglycoside (with middle RRV, 1.0 equiv.) and flame-dried 4Å molecular sieves (100 mg/mL) in CH_2Cl_2 (final concentration of acceptor: 0.05 M) at −50 °C were sequentially added NIS (1.08 equiv.) and TfOH (0.15 equiv.). The reaction mixture was allowed to warm to −40°C over 1 hr during which time it should turn dark red. The TLC analysis revealed full consumption of both starting materials. At this point, acceptor (0.9 equiv.) and another portion of NIS (1.1 equiv.) and TfOH (0.15 equiv.) were added at −40 °C. The reaction mixture was allowed to warm to 0 °C over 30 min before it was quenched by saturated aq. $NaHCO_3/Na_2S_2O_3$. The resulting mixture was then diluted with CH_2Cl_2 and H_2O and extracted with CH_2Cl_2 (20 mL × 3). The organic phases were combined, dried over Na_2SO_4, filtered and concentrated under vacuum. The resulting residue was purified by a flash chromatography using proper eluent to obtain clean product.

VII. **Removal of Lev Ester:** to a solution of levulinate substrate (1.0 equiv.) in CH_2Cl_2 (final concentration of the substrate: 40 mg/mL) at room temperature was added a solution of hydrazine acetate in MeOH (*ca*. 0.5 M, 2.0 equiv.). The reaction mixture was stirred at room temperature for 6-12 hrs before it was diluted with CH_2Cl_2 and H_2O. The organic phase was separated, and the aqueous phase was extracted with CH_2Cl_2 (three times). The organic phases were combined, dried over Na_2SO_4 and concentrated under vacuum. The residue was then purified by flash chromatography using proper eluents to afford desired alcohol.

VIII. **Removal of TBS Ether:** to a solution of TBS ether substrate (1.0 equiv.) in THF (final concentration of the substrate: 30-40 mg/mL) at 0 °C were sequentially added NH_4F (10.0 equiv.) and TBAF (1.0 M solution in THF, 5.0 equiv.). The reaction suspension was stirred at room temperature for 12 hrs before it was quenched by aqueous NH_4Cl (1.0 M) and was diluted by EtOAc. The organic phase was separated, and the aqueous phase was extracted by EtOAc (three times). The organic phases were combined, dried over Na_2SO_4 and concentrated under vacuum. The residue was then purified by flash chromatography using proper eluents to afford desired alcohol.

IX. **Removal of Nap Ether:** to a solution of 2-naphthylmethyl ether substrate (1.0 equiv.) in $CH_2Cl_2:H_2O$ (10:1, *v*/*v*, final concentration of the substrate: 30-40 mg/mL) at 0 °C was added DDQ (2.0 equiv.) in one portion. The reaction suspension was stirred at the same temperature for another 3 hrs (monitored by TLC) before it was quenched by saturated aqueous $NaHCO_3$ (same volume as CH_2Cl_2). The organic phase was separated, and the aqueous phase was extracted by CH_2Cl_2 (three times). The organic phases were combined, dried over Na_2SO_4 and concentrated under vacuum. The residue was then purified by flash chromatography using proper eluents to afford desired alcohol.

X. **Removal of Fmoc Group:** a solution of Fmoc carbonate substrate (1.0 equiv.) in CH_2Cl_2:Et_3N (10:1, *v*/*v*, final concentration of the substrate: 30-40 mg/mL) at room temperature was stirred for 12 hrs before all the volatiles were removed under vacuum. The residue was then purified by flash chromatography using proper eluents to afford desired alcohol.

XI. **Global Deprotection of *N*-glycans (per-debenzylation):** a solution of fully protected *N*-glycans in ^{n}BuOH/EDA (4:1, *v*/*v*, 25 mg/mL) was heated at 90 °C for 18 hrs. The reaction mixture was cooled to room temperature, concentrated under vacuum, and re-dissolved in pyridine/Ac_2O (2/1, *v*/*v*). The clear solution was stirred for another 24 hrs before it was concentrated under vacuum and azeotroped with toluene (10 mL × 3). The resulting residue was then dissolved in MeOH and NaOMe (*ca.* 5.4 M in MeOH) was added at room temperature to make the final concentration at 0.2 M. The de-acetylation process continued for 12 hrs and the reaction mixture was diluted with MeOH and neutralized with Amberlite™ IR-120 (washed, 1.0 g per 1 mmol NaOMe added). The suspension was filtered, concentrated and dried under vacuum. The reaction residue was purified by a flash column (silica gel, toluene:acetone = 7/3 → 1/1, *v*/*v*) to provide the corresponding multi-benzylated *N*-glycan as a glassy film.

XII. **Global Debenzylation by Hydrogenolysis:**

(A) Hydrogenlolysis by a routine set-up: to a solution of multi-benzylated *N*-glycan in THF/^{t}BuOH/H_2O (3/1/1, *v*/*v*/*v*, 10-20 mg/mL) under argon was added acidic DMF pretreated 20% *w*/*w* $Pd(OH)_2$/C (100% *w*/*w* to the substrate) at room temperature. Then the system was switched to hydrogen atmosphere using a hydrogen balloon on a three-way valve. The reaction mixture was vigorously but steadily stirred for 72-120 hrs until the TLC (siliga gel, MeCN/H_2O = 3:1, *v*/*v*) showed spot only on the baseline. The palladium catalyst was filtered and the filtrate was concentrated and subjected onto a P2 column. The factions containing the final product (judged by TLC or mass spec) were combined and lyophilized to afford the protection group free *N*-glycan as a white puffy solid.

(B) Hydrogenolysis using H-Cube® Mini: the 30 mm palladium catalyst (10% Pd/C, Cat# THS-01111 or 20% $Pd(OH)_2$/C, Cat# THS-01115) cartridge was placed into the cartridge holder and the system parameters were set as following: hydrogen mode ON (full mode), temperature OFF (room temperature), flow rate 1.0 mL/min. The system was primed using the reaction solvent THF/^{t}BuOH/H_2O (3/1/1, *v*/*v*/*v*) at a flow rate of 2 mL/min until stable. The catalyst was then pretreated with acidified DMF by passing DMF/H_2O/conc. HCl (12/3/1, *v*/*v*/*v*) through the catalyst cartridge at a flow rate of 1.0 mL/min with hydrogen mode ON. Dark yellow liquid might be observed from the outlet end if 20% $Pd(OH)_2$/C cartridge was used, but the color should cease within 5 min and the pretreatment process was allowed to continue for another 15 min as to exceed the dead volume of the system (approx. 10 mL/10 min running time). The inlet filter was then placed into the reaction solvent [THF/^{t}BuOH/H_2O (2/1/1, *v*/*v*/*v*)] and let the system run for another 20 min at 1 mL/min to wash out all the DMF and/or acid residue(s) in the line. The hydrogenolysis reaction was initiated by placing the inlet filter into the flask containing the substrate solution. The outlet end was also placed in the same flask so that the reaction solution was circulating through the catalyst cartridge. When running at flow rate of 1.0 mL/min, the system pressure was usually 35-40

bar. The reaction was closely monitored by TLC (siliga gel, MeCN/H_2O = 3:1, *v*/*v*, spot only on the baseline) or by mass spec. Upon completion, the inlet filter was switched into blank reaction solvent to wash out all the product. All the volatiles were evaporated under vacuum and the resulting residue was subjected onto a P2 column. The factions containing the final product (judged by TLC or mass spec) were combined and lyophilized to afford the protection group free *N*-glycan as a white puffy solid.

XIII. **Enzymatic β1,4-Galactosylation:** to a solution of terminal GlcNAc *N*-glycan substrate (1.0 equiv.) and UDP-Gal disodium salt (2.0 equiv. per equiv. of terminal GlcNAc) in ddH_2O (final substrate concentration = 4-5 mM) were sequentially added Tris-HCl buffer (pH = 7.5, 1.0 M, 1/20 *v*/*v*(ddH_2O)), $MnCl_2$ (200 mM in ddH_2O, 1/20 *v*/*v*(ddH_2O)) and bovine β4GalT1 (0.3 nmol protein per μmol galactosylation). The reaction mixture was incubated at 37 °C for 24 hrs before it was quenched by adding equal volume of EtOH and leaving on ice for 1 hr. The resulting suspension was centrifuged and the supernatant was concentrated under vacuum. The residue was purified using P2 (Bio-Gel®, superfine, particle size <45 μm) or Sephadex™ G-25 (Cytiva, superfine, particle size ~20 μm) gel to afford galactosylated glycan. It could be further purified on a short DEAE (Bio-Gel®, Cl^- form) column if the product was slightly contaminated with UDP-Gal.

XIV. **Enzymatic α2,3-Sialylation:** to a solution of terminal Gal *N*-glycan substrate (1.0 equiv.) and CMP-Neu5Ac (2.0 equiv. per equiv. of terminal Gal) in ddH_2O (final substrate concentration = 1 mM) were sequentially added Tris-HCl buffer (pH = 7.5, 1.0 M, 1/10 *v*/*v*(ddH_2O)), $MgCl_2$ (200 mM in ddH_2O, 1/10 *v*/*v*(ddH_2O)) and proper α2,3-sialyltransferase (e.g., *Vibrio sp.* JT-FAJ-16 (mutant) or PmST1 (M144D), 1% *w*/*w* of substrate per sialylation loci). The reaction mixture was incubated at 37 °C for 24 hrs before it was quenched by adding equal volume of EtOH and leaving on ice for 1 hr. The resulting suspension was centrifuged and the supernatant was concentrated under vacuum. The residue was purified using P2 (Bio-Gel®, superfine, particle size <45 μm) or Sephadex™ G-25 (Cytiva, superfine, particle size ~20 μm) gel to afford sialylated glycan. In some cases (especially when using PmST1 (M144D) to synthesize sialyl Le^x epitope), the product so isolated was mixed with unsialylated or partially sialylated glycans due to incomplete sialylation reaction. The mixture was subjected to the same reaction conditions to pursue the completion of sialylation.

XV. **Enzymatic α2,6-Sialylation:** to a solution of terminal Gal *N*-glycan substrate (1.0 equiv.) and CMP-Neu5Ac (2.0 equiv. per equiv. of terminal Gal) in ddH_2O (final substrate concentration = 1 mM) were sequentially added Tris-HCl buffer (pH = 7.5, 1.0 M, 1/10 *v*/*v*(ddH_2O)), $MgCl_2$ (200 mM in ddH_2O, 1/10 *v*/*v*(ddH_2O)) and proper α2,6-sialyltransferase (e.g., Pd2,6ST (A200Y/S232Y) or human ST6Gal1, 1% *w*/*w* of substrate per sialylation loci). The reaction mixture was incubated at 37 °C for 24 hrs before it was quenched by adding equal volume of EtOH and leaving on ice for 1 hr. The resulting suspension was centrifuged and the supernatant was concentrated under vacuum. The residue was purified using P2 (Bio-Gel®, superfine, particle size <45 μm) or Sephadex™ G-25 (Cytiva, superfine, particle size ~20 μm) gel to afford sialylated glycan. In some cases (especially when using tri-antennary tri-galactoside glycan as an acceptor), the product so isolated was mixed

with partially sialylated glycans due to incomplete sialylation reaction. The mixture was subjected to the same reaction conditions to pursue the completion of sialylation.

XVI. **Enzymatic α1,2-Fucosylation:** to a solution of terminal Gal *N*-glycan substrate (1.0 equiv.) and GDP-fucose (1.5 equiv. per equiv. of terminal Gal) in ddH_2O (final substrate concentration = 1 mM) were sequentially added Tris-HCl buffer (pH = 7.5, 1.0 M, 1/20 *v*/*v*(ddH_2O)), $MnCl_2$ (200 mM in ddH_2O, 1/20 *v*/*v*(ddH_2O)) and human FuT1 or FuT2 (1% *w*/*w* of substrate per terminal Gal). The reaction mixture was incubated at 37 °C for 24 hrs before it was quenched by adding equal volume of EtOH and leaving on ice for 1 hr. The resulting suspension was centrifuged and the supernatant was concentrated under vacuum. The residue was purified using P2 (Bio-Gel®, superfine, particle size <45 µm) or Sephadex™ G-25 (Cytiva, superfine, particle size ~20 µm) gel to afford fucosylated glycan. It could be further purified on a short DEAE (Bio-Gel®, Cl^- form) column if the product was slightly contaminated with GDP-fucose.

XVII. **Enzymatic Digestion of Internal Fucose Residue:** to a solution of fucosylated glycan (2 mM in ddH_2O) were sequentially added NaCl (1.0 M in ddH_2O, 1/10 *v*/*v*(substrate solution)), MES buffer (100 mM, pH = 6.5, 1/5 *v*/*v*(substrate solution)) and α1,3-fucosidase Am0392 (20% *w*/*w* of substrate). The reaction mixture was incubated at 37 °C for 24 hrs before it was quenched by adding equal volume of EtOH and leaving on ice for 1 hr. The resulting suspension was centrifuged and the supernatant was concentrated under vacuum. The residue was purified using P2 (Bio-Gel®, superfine, particle size <45 µm) or Sephadex™ G-25 (Cytiva, superfine, particle size ~20 µm) gel to afford de-fucosylated glycan.

Experimental Procedures

Scheme S1. Synthesis of Mannoside Building Blocks 3–6[a]

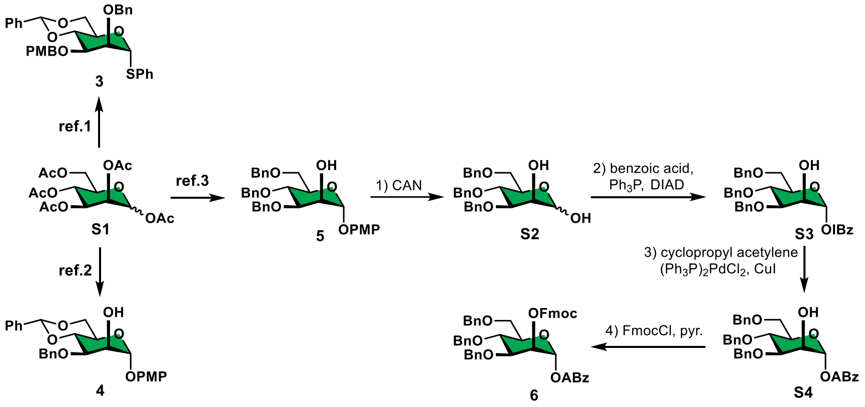

[a]Reagents and Conditions: 1) CAN (3.0 equiv.), toluene:ACN:H_2O 1:4:1 (*v*/*v*/*v*), 0 °C, 1 hr, 75%; 2) 2-iodobenzoic acid (2.0 equiv.), PPh_3 (2.0 equiv.), DIAD (2.0 equiv.), THF, −78 to 22 °C, 2 hrs, 88%; 3) cyclopropyl acetylene (5.0 equiv.), $(Ph_3P)_2PdCl_2$ (0.1 equiv.), CuI (0.2 equiv.), Et_3N:DMF = 3:1 (*v*/*v*), 22 °C, 24 hrs, 90%; 4) FmocCl (2.0 equiv.), pyridine (10.0 equiv.), CH_2Cl_2, 0 °C, 93%.

Phenylthio mannoside **3**, 4-methoxyphenyl mannosides **4** and **5** were synthesized from mannose pentaacetate **S1** according to reported literatures.[10–12]

3,4,6-Tri-*O*-benzyl-D-mannopyranose (S2): to a solution of **5** (500 mg, 0.898 mmol, 1.0 equiv.) in a mixed solvent (12 mL, toluene:ACN:H_2O 1:4:1 (*v*/*v*/*v*)) at 0 °C was slowly added a solution of CAN (2.7 mL, 1.0 M in ddH_2O). The reaction mixture was stirred at the same temperature for another 1 hr before it was diluted with EtOAc (20 mL) and H_2O (20 mL). The organic phase was separated, and the aqueous phase was extracted with EtOAc (10 mL × 3). The organic phases were combined, dried over Na_2SO_4 and concentrated *in vacuo*. The resulting residue was purified by flash chromatography (silica gel, toluene:EtOAc = 10:1 → 4:1) to afford diol **S2** as a pair of anomeric mixture (α:β *ca.* 5:1, 305 mg, 75%). **S2** (α-siomer): ^{1}H NMR (600 MHz, $CDCl_3$) δ = 7.53 – 7.27 (m, 13H), 7.24 – 7.16 (m, 2H), 5.29 (d, *J* = 1.8 Hz, 1H), 4.87 (d, *J* = 10.9 Hz, 1H), 4.72 (d, *J* = 11.5 Hz, 1H), 4.67 (d, *J* = 11.5 Hz, 1H), 4.59 (d, *J* = 12.2 Hz, 1H), 4.52 (d, *J* = 12.1 Hz, 1H), 4.51 (d, *J* = 11.0 Hz, 1H), 4.34 (brs, 1H), 4.10 (ddd, *J* = 10.0, 6.1, 2.2 Hz, 1H), 4.04 (dd, *J* = 3.2, 1.8 Hz, 1H), 3.97 (dd, *J* = 9.1, 3.2 Hz, 1H), 3.76 (apparent t, *J* = 9.6 Hz, 1H), 3.71 (dd, *J* = 10.4, 2.2 Hz, 1H), 3.67 (dd, *J* = 10.4, 6.1 Hz, 1H), 2.82 (br s, 1H) ppm; ^{13}C NMR (151 MHz, $CDCl_3$) δ = 138.3, 138.0, 137.8, 128.5, 128.43, 128.39, 128.2, 128.0, 127.9, 127.8, 127.7, 93.9, 79.7, 75.1, 74.6, 73.3, 71.9, 70.6, 69.3, 68.6 ppm. **S2** (β-siomer): δ = 7.53 – 7.27 (m, 13H), 7.24 – 7.16 (m, 2H), 4.89 (d, *J* = 10.9 Hz, 1H), 4.73 (d, *J* = 11.2 Hz, 1H), 4.63 (s, 1H), 4.62 (d, *J* = 11.5 Hz, 1H), 4.61 – 4.57 (m, 1H), 4.56 – 4.48 (m, 2H), 3.92 (d, *J* = 3.2 Hz, 1H), 3.85 (apparent t, *J* = 9.5 Hz, 1H), 3.75 – 3.72 (m, 2H), 3.53 (dd, *J* = 9.2, 3.2 Hz, 1H), 3.42 (apparent dt, *J* = 9.9, 3.3 Hz, 1H) ppm; ^{13}C NMR (151 MHz, $CDCl_3$) δ = 138.2, 137.9, 137.7, 128.5, 128.4, 128.2, 128.1, 127.92, 127.89, 127.8,

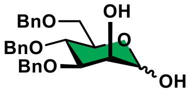

S2

94.4, 81.6, 75.2, 74.6, 73.8, 73.5, 71.4, 68.9, 68.8 ppm. HRMS (ESI-ToF) *m/z* calculated for $C_{27}H_{30}NaO_6^+$ $[M+Na]^+$: 473.1935, found: 473.1929.

3,4,6-Tri-*O*-benzyl-α-D-mannopyranosyl 2-iodobenzoate (S3): to a solution of **S2** (305 mg, 0.677 mmol, 1.0 equiv.), 2-iodobenzoic acid (337 mg, 1.35 mmol, 2.0 equiv.) and Ph_3P (355 mg, 1.35 mmol, 2.0 equiv.) in THF (8.0 mL) at −78 °C was added DIAD (274 mg, 266 μL, 1.35 mmol, 2.0 equiv.). The reaction mixture was allowed to warm to 22 °C and stir at the same temperature for another 2 hrs. All the volatiles were evaporated *in vacuo* and the resulting residue was purified by flash chromatography (silica gel, toluene:EtOAc = 10:1 → 6:1) to afford glycosyl 2-iodobenzoate **S3** (408 mg, 88%) as a white foam. **S3**: ^{1}H NMR (600 MHz, $CDCl_3$) δ = 7.99 (dd, *J* = 8.0, 1.1 Hz, 1H), 7.73 (dd, *J* = 7.8, 1.7 Hz, 1H), 7.43 – 7.26 (m, 14H), 7.24 –7.20 (m, 2H), 7.17 (td, *J* = 7.7, 1.7 Hz, 1H), 6.48 (d, *J* = 1.9 Hz, 1H), 4.88 (d, *J* = 10.6 Hz, 1H), 4.76 (s, 2H), 4.69 (d, *J* = 12.2 Hz, 1H), 4.59 (d, *J* = 10.7 Hz, 1H), 4.55 (d, *J* = 12.1 Hz, 1H), 4.20 (dd, *J* = 3.0, 1.9 Hz, 1H), 4.08 (apparent t, *J* = 9.2 Hz, 1H), 4.05 (dd, *J* = 9.1, 3.1 Hz, 1H), 3.97 (ddd, *J* = 9.4, 4.1, 1.9 Hz, 1H), 3.81 (dd, *J* = 11.1, 4.0 Hz, 1H), 3.73 (dd, *J* = 11.1, 2.0 Hz, 1H), 2.74 (brs, 1H) ppm; ^{13}C NMR (151 MHz, $CDCl_3$) δ = 164.4, 141.5, 138.20, 138.16, 137.8, 134.6, 133.2, 131.5, 128.7, 128.53, 128.46 128.24, 128.18, 128.09, 128.07, 127.9, 127.7, 94.6, 94.1, 79.2, 75.5, 74.3, 73.8, 73.7, 72.2, 68.5, 67.7 ppm. HRMS (ESI-ToF) *m/z* calculated for $C_{34}H_{33}INaO_7^+$ $[M+Na]^+$: 703.1163, found: 703.1160.

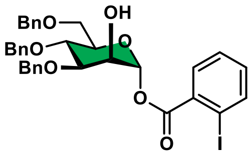

S3

3,4,6-Tri-*O*-benzyl-α-D-mannopyranosyl 2-(cyclopropylethynyl)benzoate (S4): to a solution of **S3** (405 mg, 0.595 mmol, 1.0 equiv.) in a mixed solvent (10.0 mL, Et_3N:DMF = 3:1, *v*/*v*) at 22 °C were sequentially added $(Ph_3P)_2PdCl_2$ (42.0 mg, 59.5 μmol, 0.1 equiv.) and CuI (23.0 mg, 0.119 mmol, 0.2 equiv.) and the resulting dark yellow solution was bubbled with argon for 20 min. After that, cyclopropyl acetylene (197 mg, 252 μL, 2.98 mmol, 5.0 equiv.) was added and the solution turned dark red instantly. The reaction mixture was stirred at 22 °C for 24 hrs before it was concentrated under vacuum and purified by flash chromatography (silica gel, hexanes:EtOAc = 4:1 → 2:1, *v*/*v*) to afford *o*-alkynylbenzoate **S4** (330 mg, 90%) as a yellow foam. **S4**: ^{1}H NMR (600 MHz, $CDCl_3$) δ = 7.87 (dd, *J* = 8.0, 1.3 Hz, 1H), 7.49 (dd, *J* = 7.8, 1.4 Hz, 1H), 7.44 (td, *J* = 7.6, 1.4 Hz, 1H), 7.42 – 7.26 (m, 14H), 7.21 – 7.15 (m, 2H), 6.51 (d, *J* = 1.9 Hz, 1H), 4.87 (d, *J* = 10.5 Hz, 1H), 4.76 (ABq, J_{AB} = 11.5 Hz, $\Delta\nu_{AB}$ = 10.0 Hz ,2H), 4.72 (d, *J* = 12.2 Hz, 1H), 4.58 (d, *J* = 10.6 Hz, 1H), 4.56 (d, *J* = 12.2 Hz, 1H), 4.17 (apparent t, *J* = 2.4 Hz, 1H), 4.15 – 4.08 (m, 3H), 3.85 (dd, *J* = 11.0, 2.7 Hz, 1H), 3.75 (dd, *J* = 11.0, 1.1 Hz, 1H), 2.77 (br s, 1H), 1.57 (tt, *J* = 8.3, 5.0 Hz, 1H), 0.86 – 0.79 (m, 2H), 0.79 – 0.71 (m, 2H) ppm; ^{13}C NMR (151 MHz, $CDCl_3$) δ = 164.2, 138.3, 138.2, 137.7, 134.7, 132.2, 130.9, 130.7, 128.7, 128.42, 128.40, 128.13, 128.08, 128.0, 127.8, 127.7, 127.2, 124.9, 100.1, 94.1, 79.6, 75.4, 74.9, 74.1, 73.7, 73.6, 72.3, 68.5, 67.8, 9.12, 9.11, 0.8 ppm. HRMS (ESI-ToF) *m/z* calculated for $C_{39}H_{38}NaO_7^+$ $[M+Na]^+$: 641.2510, found: 641.2512.

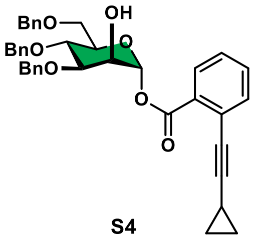

S4

3,4,6-Tri-*O*-benzyl-2-*O*-fluorenylmethoxycarbonyl-α-D-mannopyranosyl 2-(cyclopropylethynyl)benzoate (6): to a solution of **S4** (330 mg, 0.533 mmol, 1.0 equiv.) and pyridine (422 mg, 430 μL, 5.33 mmol, 10.0 equiv.) in CH_2Cl_2 (8.0 mL) at 0 °C was added portion-wise FmocCl (276 mg, 1.07 mmol, 2.0 equiv., two portions). The reaction mixture was stirred at the same temperature for another 1 hr until TLC showed full consumption of the starting material (**S4**). The reaction was then diluted with CH_2Cl_2 (10 mL) and quenched by 1 N HCl (10 mL). The organic phase was washed sequentially with 1 N HCl (20 mL), aqueous saturated $NaHCO_3$ (20 mL) and brine (20 mL), dried over Na_2SO_4, and concentrated under vacuum. The resulting residue was purified by flash chromatography (silica gel, toluene:EtOAc = 40:1 → 12:1, *v*/*v*) to afford *o*-alkynylbenzoate **6** (417 mg, 93%) as a white foam.

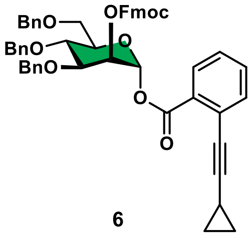

6

6: ^{1}H NMR (600 MHz, $CDCl_3$) δ = 7.90 (dd, *J* = 8.0, 1.3 Hz, 1H), 7.85 – 7.78 (m, 2H), 7.72 (d, *J* = 7.5 Hz, 1H), 7.68 (d, *J* = 7.5 Hz, 1H), 7.51 (dd, *J* = 7.9, 1.3 Hz, 1H), 7.48 (dd, *J* = 7.4, 1.4 Hz, 1H), 7.47 – 7.42 (m, 4H), 7.41 – 7.29 (m, 11H), 7.28 – 7.24 (m, 3H), 7.23 – 7.17 (m, 2H), 6.62 (d, *J* = 2.0 Hz, 1H), 5.44 (dd, *J* = 3.2, 2.1 Hz, 1H), 4.96 (d, *J* = 10.5 Hz, 1H), 4.88 (d, *J* = 11.2 Hz, 1H), 4.80 (d, *J* = 12.1 Hz, 1H), 4.70 (d, *J* = 11.3 Hz, 1H), 4.64 (d, *J* = 10.5 Hz, 1H), 4.62 (d, *J* = 12.1 Hz, 1H), 4.51 (dd, *J* = 10.4, 7.4 Hz, 1H), 4.42 (dd, *J* = 10.4, 8.0 Hz, 1H), 4.33 (t, *J* = 7.9 Hz, 1H), 4.30 (dd, *J* = 9.1, 3.2 Hz, 1H), 4.22 (t, *J* = 9.5 Hz, 1H), 4.18 (ddd, *J* = 9.9, 3.9, 1.7 Hz, 1H), 3.95 (dd, *J* = 11.3, 3.9 Hz, 1H), 3.83 (dd, *J* = 11.3, 1.7 Hz, 1H), 1.60 (tt, *J* = 8.3, 5.0 Hz, 1H), 0.90 – 0.81 (m, 2H), 0.80 – 0.72 (m, 2H) ppm; ^{13}C NMR (151 MHz, $CDCl_3$) δ = 164.0, 154.8, 143.6, 143.3, 141.4, 141.3, 138.3, 137.8, 134.7, 132.3, 130.9, 130.4, 128.5, 128.43, 128.39, 128.10, 128.08, 128.05, 127.98, 127.96, 127.9, 127.8, 127.7, 127.33, 127.28, 127.2, 125.5, 125.3, 125.0, 120.12, 120.08, 100.3, 92.1, 77.9, 75.5, 74.9, 74.6, 73.8, 73.7, 72.2, 71.8, 70.6, 68.6, 46.7, 9.16, 9.15, 0.8 ppm. HRMS (ESI-ToF) *m*/*z* calculated for $C_{54}H_{48}NaO_9^+$ [M+Na]$^+$: 863.3191, found: 863.3189.

Scheme S2. Synthesis of *N*-Phthalimido-glycosamine Building Blocks 7–12[a]

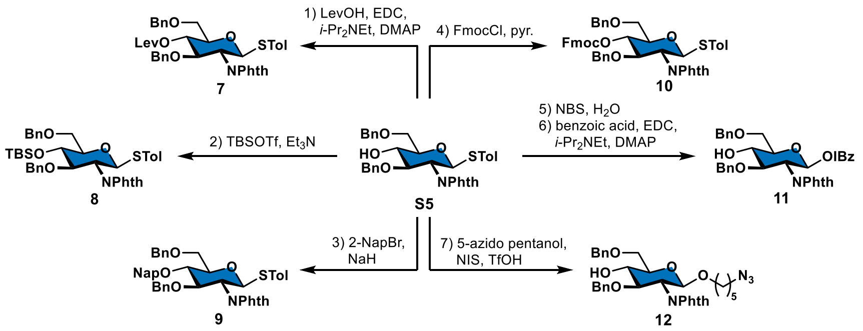

[a]Reagents and conditions: 1) LevOH (2.0 equiv.), EDC (2.0 equiv.), *i*-Pr_2NEt (2.0 equiv.), DMAP (0.1 equiv.), CH_2Cl_2, 22 °C, 12 hrs, 90%; 2) TBSOTf (2.0 equiv.), Et_3N (5.0 equiv.), CH_2Cl_2, 0 → 22 °C, 12 hrs, 93%; 3) 2-NapBr (1.5 equiv.), NaH (2.0 equiv.), DMF, 0 → 22 °C, 1 hr, 66%; 4) FmocCl (3.0 equiv.), pyridine (10.0 equiv.), CH_2Cl_2, 0 °C, 3 hrs, 86%; 5) NBS (3.0 equiv.), acetone:H_2O = 10:1 (*v*/*v*), 22 °C, 30 min; 6) 2-iodobenzoic acid (2.0 equiv.), EDC (2.0 equiv.), *i*-Pr_2NEt (2.0 equiv.), DMAP (0.1 equiv.), CH_2Cl_2, 0 → 22 °C, 3 hrs, 88% over the two steps; 7) 5-azido pentanol (3.0 equiv.), NIS (1.5 equiv.), TfOH (0.15 equiv.), CH_2Cl_2, 4Å MS, −40 → 0 °C, 30 min, 72%.

***p*-Tolyl 4-*O*-levulinoyl-3,6-di-*O*-benzyl-2-deoxy-2-phthalimido-1-thio-β-D-glucopyranoside (7)**: to a solution of **S5**[11] (1.80 g, 3.02 mmol, 1.0 equiv.) and levulinic acid (701 mg, 616 µL, 6.04 mmol, 2.0 equiv.) in CH_2Cl_2 (40 mL) at 22 °C were sequentially added EDC (1.16 g, 6.04 mmol, 2.0 equiv.), DIPEA (781 mg, 1.05 mL, 6.04 mmol, 2.0 equiv.) and 4-DMAP (36.9 mg, 0.302 mmol, 0.1 equiv.). The reaction mixture was stirred at 22 °C for 12 hrs before it was diluted with CH_2Cl_2 (50 mL) and quenched with H_2O (50 mL). The organic phase was separated and the aqueous phase was extracted with CH_2Cl_2 (20 mL × 3). The organic phases were combined, dried over Na_2SO_4 and concentrated under vacuum, and the resulting residue was purified by flash chromatography (silica gel, hexanes:EtOAc = 4:1 → 1:1, *v*/*v*) to afford levulinic ester **7** (1.88 g, 90%) as an amorphous foam. **7**: ^{1}H NMR (600 MHz, $CDCl_3$) δ = 7.80 (br d, *J* = 7.1 Hz, 1H), 7.73 – 7.62 (m, 3H), 7.40 – 7.32 (m, 4H), 7.31 – 7.26 (m, 3H), 7.02 – 6.94 (m, 4H), 6.93 – 6.87 (m, 2H), 6.87 – 6.83 (m, 1H), 5.48 (d, *J* = 10.5 Hz, 1H), 5.12 (dd, *J* = 10.1, 8.9 Hz, 1H), 4.64 (d, *J* = 12.2 Hz, 1H), 4.53 (ABq, J_{AB} = 11.8 Hz, $\Delta\nu_{AB}$ = 5.2 Hz, 2H), 4.44 (dd, *J* = 10.2, 8.9 Hz, 1H), 4.31 (d, *J* = 10.9 Hz, 1H), 4.29 (apparent t, *J* = 9.9 Hz, 1H), 3.80 (ddd, *J* = 9.8, 5.6, 3.5 Hz, 1H), 3.68 – 3.60 (m, 2H), 2.70 – 2.59 (m, 2H), 2.45 (t, *J* = 6.5 Hz, 2H), 2.26 (s, 3H), 2.14 (s, 3H) ppm; ^{13}C NMR (151 MHz, $CDCl_3$) δ = 206.3, 171.7, 168.2, 167.1, 138.3, 138.2, 137.8, 134.1, 133.9, 133.2, 131.7, 131.6, 129.7, 128.4, 128.23, 128.16, 128.0, 127.9, 127.7, 127.5, 123.7, 123.4, 83.8, 78.1, 77.8, 74.3, 73.6, 72.7, 69.8, 54.7, 37.8, 29.9, 28.0, 21.2 ppm. HRMS (ESI-ToF) *m*/*z* calculated for $C_{40}H_{39}NNaO_8S^+$ $[M+Na]^+$: 716.2289, found: 716.2275.

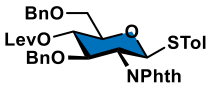

7

***p*-Tolyl 4-*O*-*tert*-butyldimethylsilyl-3,6-di-*O*-benzyl-2-deoxy-2-phthalimido-1-thio-β-D-glucopyranoside (8)**: to a solution of **S5** (1.79 g, 3.00 mmol, 1.0 equiv.) in CH_2Cl_2 (30 mL) at 0 °C were sequentially added Et_3N (911 mg, 1.25 mL, 9.00 mmol, 3.0 equiv.) and TBSOTf (1.19 g, 1.03 mL, 4.50 mmol, 1.5 equiv.). The reaction mixture was stirred at 22 °C for 12 hrs before

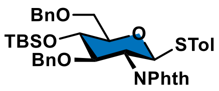

8

it was quenched with MeOH (0.1 mL) and concentrated under vacuum. The resulting residue was purified by flash chromatography (silica gel, hexanes:EtOAc = 10:1 → 4:1, *v*/*v*) to afford TBS silyl ether **8** (1.98 g, 93%) as a white foam. The spectral data of **8** were consistent with those reported in the literature.[13]

***p*-Tolyl 4-*O*-(2-naphthylmethyl)-3,6-di-*O*-benzyl-2-deoxy-2-phthalimido-1-thio-β-D-glucopyranoside (9)**: to a solution of **S5** (1.45 g, 2.43 mmol, 1.0 equiv.) and 2-NapBr (805 mg, 3.64 mmol, 1.5 equiv.) in DMF (20 mL) at 0 °C was added NaH (146 mg, 60% suspension in mineral oil, 3.64 mmol, 1.5 equiv.). The suspension was slowly warmed to 22 °C and stirred at the same temperature for another 1 hr before it was quenched with AcOH (0.250 mL, 263 mg, 4.38 mmol, 1.8 equiv.) and diluted with CH_2Cl_2 (30 mL). The organic phase was washed with $NaHCO_3$ (20 mL × 2) and brine (20 mL × 2). The organic phases were combined, dried over Na_2SO_4, and concentrated under vacuum, and the resulting residue was purified by flash chromatography (silica gel, hexanes:EtOAc = 4:1 → 2:1, *v*/*v*) to afford 2-Nap ether **9** (1.18 g, 66%). **9**: ^{1}H NMR (600 MHz, $CDCl_3$) δ = 7.89 – 7.78 (m, 4H), 7.74 – 7.63 (m, 4H), 7.54 – 7.47 (m, 2H), 7.43 – 7.31 (m, 8H), 7.06 – 6.99 (m, 4H), 6.95 – 6.86 (m, 3H), 5.55 (d, *J* = 10.4 Hz, 1H), 5.02 (d, *J* = 11.2 Hz, 1H), 4.88 – 4.82 (m, 2H), 4.67 (d, *J* = 11.9 Hz, 1H), 4.57 (d, *J* = 11.9 Hz, 1H), 4.50 (d, *J* = 12.1 Hz, 1H), 4.44 (dd, *J* = 10.2, 8.7 Hz, 1H), 4.30 (apparent t, *J* = 10.3 Hz, 1H), 3.92 – 3.84 (m, 3H), 3.76 (ddd, *J* = 10.0, 4.1, 2.2 Hz, 1H), 2.30 (s, 3H) ppm; ^{13}C NMR (151 MHz, $CDCl_3$) δ = 168.1, 167.4, 138.4, 138.2, 137.9, 135.6, 133.9, 133.8, 133.5, 133.3, 133.0, 131.8, 131.6, 129.6, 128.4, 128.3, 128.2, 128.1, 128.0, 127.79, 127.75, 127.6, 127.5, 126.7, 126.2, 126.1, 126.0, 123.5, 123.4, 83.4, 80.4, 79.5, 75.09, 75.07, 73.5, 69.0, 55.1, 21.2 ppm. HRMS (ESI-ToF) *m*/*z* calculated for $C_{46}H_{41}NNaO_6S^+$ $[M+Na]^+$: 758.2547, found: 758.2551.

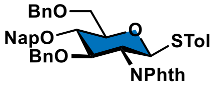

9

***p*-Tolyl 4-*O*-fluorenylmethoxycarbonyl-3,6-di-*O*-benzyl-2-deoxy-2-phthalimido-1-thio-β-D-glucopyranoside (10)**: to a solution of **S5** (1.79 g, 3.00 mmol, 1.0 equiv.) and pyridine (2.37 g, 2.42 mL, 30.0 mmol, 10.0 equiv.) in CH_2Cl_2 (40 mL) at 0 °C was added FmocCl (2.33 g, 9.00 mmol, 3.0 equiv.) in three portions. The reaction was monitored by TLC to ensure all the starting material was consumed. Then 1 N HCl (50 mL) was added to quench the reaction. The organic phase was separated and washed sequentially with 1 N HCl (20 mL × 2), saturated aqueous $NaHCO_3$ (20 mL) and brine (20 mL). The organic phase was then dried over Na_2SO_4 and concentrated under vacuum, and the residue was purified by flash chromatography (silica gel, toluene:EtOAc = 20:1 → 10:1) to afford Fmoc carbonate **10** (2.11 g, 86%) as a white foam. The spectral data of **10** were consistent with those reported in the literature.[14]

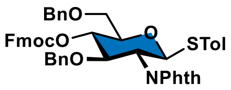

10

3,6-Di-*O*-benzyl-2-deoxy-2-phthalimido-β-D-glucopyranosyl 2-iodobenzoate (11): to a solution of **S5** (4.00 g, 6.71 mmol, 1.0 equiv.) in a mixed solvent (100 mL, acetone:H_2O = 10:1, *v*/*v*) at 22 °C was added NBS (3.59 g, 20.1 mmol, 3.0 equiv.). The reaction mixture was stirred at 22 °C for 30 min before it was quenched with saturated aqueous $NaHCO_3$ (30 mL). The resulting suspension was concentrated under vacuum to remove most of acetone and was then re-partitioned between CH_2Cl_2 (60 mL) and H_2O (60 mL). The organic phase was separated, dried over Na_2SO_4, and concentrated under vacuum. The crude material so-obtained was purified briefly by flash chromatography (silica gel, hexanes:EtOAc = 3:1 → 1:1, *v*/*v*) to provide 4-hydroxy lactol (3.30 g, 100%) as a white foam. To a solution of 4-hydroxy lactol (3.30 g, 6.71 mmol, 1.0 equiv.) and 2-iodobenzoic acid (3.34 g, 13.42 mmol, 2.0 equiv.) in CH_2Cl_2 (70 mL) were sequentially added EDC (2.57 g, 13.42 mmol, 2.0 equiv.), *i*-Pr_2NEt (1.73 g, 2.34 mL, 13.42 mmol, 2.0 equiv.) and 4-DMAP (82.0 mg, 671 μmol, 0.1 equiv.) at 0 °C. The reaction mixture was allowed to warm slowly to 22 °C and stirred at the same temperature for another 3 hrs before it was quenched with MeOH (1.0 mL). All the volatiles were removed under vacuum and the resulting residue was purified by flash chromatography (silica gel, hexanes:EtOAc = 4:1 → 2:1, *v*/*v*) to afford 2-iodobenzoate **11** (4.24 g, 88% for the two steps) as a white foam. **11**: ^{1}H NMR (600 MHz, $CDCl_3$) δ = 7.89 (dd, *J* = 7.9, 1.2 Hz, 1H), 7.76 (dd, *J* = 7.9, 1.7 Hz, 1H), 7.72 (br s, 2H), 7.68 – 7.64 (m, 2H), 7.40 – 7.34 (m, 4H), 7.34 – 7.29 (m, 2H), 7.11 – 7.04 (m, 3H), 6.99 – 6.91 (m, 3H), 6.55 – 6.49 (m, 1H), 4.80 (d, *J* = 12.2 Hz, 1H), 4.66 (d, *J* = 11.9 Hz, 1H), 4.61 – 4.54 (m, 2H), 4.50 – 4.42 (m, 2H), 3.99 – 3.94 (m, 1H), 3.89 (dd, *J* = 9.8, 4.1 Hz, 1H), 3.85 (apparent dt, *J* = 9.3, 4.4 Hz, 1H), 3.79 (dd, *J* = 9.8, 4.8 Hz, 1H), 2.92 (br s, 1H) ppm; ^{13}C NMR (151 MHz, $CDCl_3$) δ = 167.9, 163.8, 141.8, 138.2, 137.6, 134.1, 133.4, 132.8, 131.8, 131.7, 128.7, 128.3, 128.2, 128.1, 128.05, 128.00, 127.6, 123.6, 94.8, 91.3, 78.3, 74.7, 74.3, 74.2, 74.0, 70.1, 54.5 ppm. HRMS (ESI-ToF) *m*/*z* calculated for $C_{35}H_{30}INNaO_8^+$ $[M+Na]^+$: 742.0908, found: 742.0925.

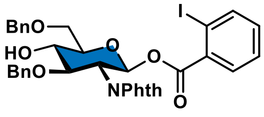

11

5-Azidopentyl 3,6-di-*O*-benzyl-2-deoxy-2-phthalimido-β-D-glucopyranoside (12): following General Procedure I, to a solution of **S5** (1.79 g, 3.00 mmol, 1.0 equiv.), 5-azidopentan-1-ol (1.16 g, 1.20 mL, 9.00 mmol, 3.0 equiv.) and flame-dried 4Å molecular sieves (4.0 g) in CH_2Cl_2 (40 mL) were sequentially added NIS (675 mg, 3.00 mmol, 1.0 equiv.) and TfOH (26.6 μL, 45.0 mg, 0.300 mmol, 0.1 equiv.) at −40 °C. Purification by flash chromatography (silica gel, hexanes:EtOAc = 4:1 → 2:1, *v*/*v*) afforded 5-azidopentyl glycoside **12** (1.30 g, 72%) as amorphous foam. The spectral data of **12** were consistent with those reported in the literature.[11,15]

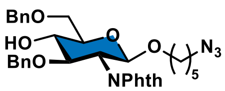

12

Scheme S3. Synthesis of ManGlcNPhth$_2$ Core[a]

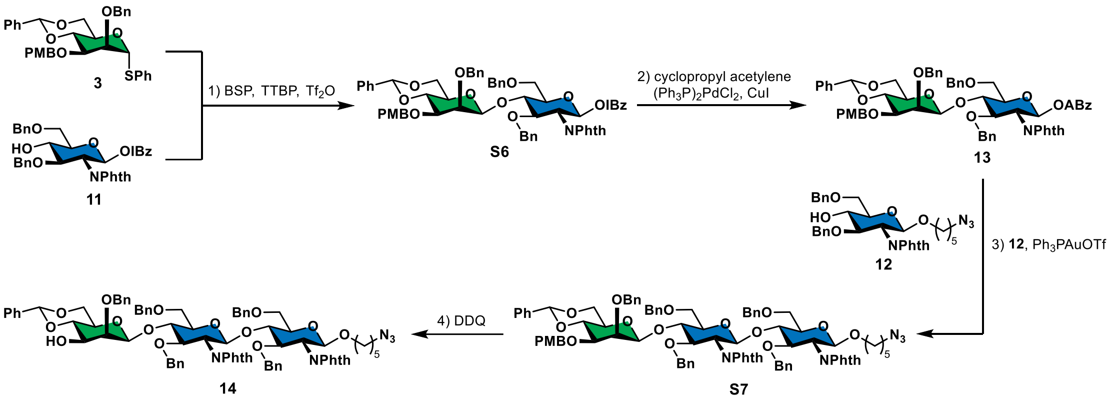

[a]Reagents and conditions: 1) **3** (1.6 equiv.), BSP (1.6 equiv.), TTBP (3.2 equiv.), Tf_2O (1.6 equiv.), CH_2Cl_2, 4Å MS, −60 °C, 5 min; then **11** (1.0 equiv.), −60 → −20°C, 1 hr, 57%; 2) cyclopropyl acetylene (5.0 equiv.), $(Ph_3P)_2PdCl_2$ (0.1 equiv.), CuI (0.2 equiv.), DMF:Et_3N = 1:3 (*v*/*v*), r.t., 24 hrs, 90%; 3) **12** (1.1 equiv.), $Ph_3PAuOTf$ (0.2 equiv.), CH_2Cl_2, 4Å MS, 0 °C, 30 min, 78%; 4) DDQ (1.2 equiv.), CH_2Cl_2:H_2O = 10:1 (*v*/*v*), 0 °C, 6 hrs, 82%.

2-*O*-benzyl-3-*O*-(4-methoxybenzyl)-4,6-benzylidene-β-D-mannopyranosyl-(1→4)-3,6-di-*O*-benzyl-2-deoxy-2-phthalimido-β-D-glucopyranosyl 2-iodobenzoate (S6): to a solution of phenylthiol glycoside **3** (1.00 g, 1.75 mmol, 1.6 equiv.), BSP (367 mg, 1.75 mmol, 1.6 equiv.), TTBP (870 mg, 3.50 mmol, 3.2 equiv.) and flame-dried 4Å molecular sieves (2.0 g) in CH_2Cl_2 (20 mL) was added Tf_2O (494 mg, 294 µL, 1.75 mmol, 1.6 equiv.) at −60 °C. The red to brown reaction mixture was stirred at the same temperature for 5 min before a solution of *o*-iodobenzoate **11** (788 mg, 1.1 mmol, 1.0 equiv.) in CH_2Cl_2 (4 mL) was added. The reaction mixture was allowed to slowly warmed to −20 °C over 1 hr and the resulting slightly decolored suspension was directly loaded onto a column. Purification by flash chromatography (silica gel, toluene:EtOAc = 88:12) afforded desired β-mannoside **S6** (735 mg, 0.623 mmol, 57%) as a white foam.

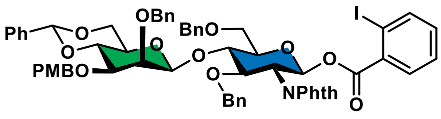

S6

S6: ^{1}H NMR (600 MHz, $CDCl_3$) δ = 7.91 (dd, *J* = 7.9, 1.2 Hz, 1H), 7.77 (dd, *J* = 7.9, 1.7 Hz, 1H), 7.73 – 7.61 (m, 4H), 7.51 – 7.44 (m, 4H), 7.41 – 7.23 (m, 14H), 7.09 (apparent td, *J* = 7.6, 1.7 Hz, 1H), 6.96 – 6.90 (m, 2H), 6.88 – 6.82 (m, 5H), 6.50 – 6.47 (m, 1H), 5.53 (s, 1H), 4.92 (d, *J* = 12.4 Hz, 1H), 4.85 (d, *J* = 12.0 Hz, 1H), 4.68 (d, *J* = 11.9 Hz, 1H), 4.66 (d, *J* = 12.0 Hz, 1H), 4.56 – 4.52 (m, 2H), 4.48 – 4.42 (m, 3H), 4.35 (d, *J* = 12.0 Hz, 1H), 4.19 (dd, *J* = 10.4, 4.8 Hz, 1H), 4.16 – 4.12 (m, 1H), 4.07 (apparent t, *J* = 9.6 Hz, 1H), 3.79 (s, 3H), 3.74 – 3.69 (m, 2H), 3.66 (apparent dt, *J* = 10.1, 2.5 Hz, 1H), 3.58 (apparent t, *J* = 10.3 Hz, 1H), 3.54 (dd, *J* = 11.2, 2.9 Hz, 1H), 3.39 (dd, *J* = 9.9, 3.1 Hz, 1H), 3.14 (apparent td, *J* = 9.7, 4.9 Hz, 1H) ppm; ^{13}C NMR (151 MHz, $CDCl_3$) δ = 167.8, 163.9, 159.3, 141.8, 138.8, 138.7, 137.8, 137.7, 134.0, 133.4, 132.7, 132.0, 131.7, 130.7, 129.2, 129.0, 128.7, 128.6, 128.34, 128.31, 128.22, 128.20, 128.1, 128.0, 127.9, 127.8, 127.1, 126.2, 123.6, 113.9, 101.8, 101.5, 94.8, 91.3, 78.8, 78.7, 78.2, 76.9, 75.5, 75.1, 75.0, 73.8, 72.5, 68.7, 68.1, 67.5, 55.4, 54.7 ppm. HRMS (ESI-ToF) *m*/*z* calculated for $C_{63}H_{58}INNaO_{14}^+$ [M+Na]$^+$: 1202.2794, found: 1202.2762.

2-*O*-benzyl-3-*O*-(4-methoxybenzyl)-4,6-benzylidene-β-D-mannopyranosyl-(1→4)-3,6-di-*O*-benzyl-2-deoxy-2-phthalimido-β-D-glucopyranosyl 2-(cyclopropylethynyl)benzoate (13): following General Procedure II, to a solution of glycosyl *o*-iodobenzoate **S6** (1.62 g, 1.37 mmol, 1.0 equiv.) in Et_3N:DMF (40 mL, 3:1 *v*/*v*) at room temperature were sequentially added $(Ph_3P)_2PdCl_2$ (96.4 mg, 0.137 mmol, 0.1 equiv.) and CuI (52.3 mg, 0.275 mmol, 0.2 equiv.), and the resulting dark yellow-green solution was bubbled with argon for 15 min before

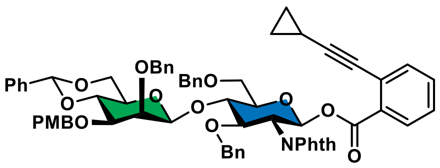

13

cyclopropyl acetylene (0.580 mL, 454 mg, 6.86 mmol, 5.0 equiv.) was added. The reaction mixture was stirred for another 24 hrs before it was concentrated and purified by flash chromatography (silica gel, EtOAc:hexanes = 1:4 → 1:2) to afford glycosyl *o*-alkynylbenzoate **13** (1.37 g, 90%) as a pale yellow foam. **13**: ^{1}H NMR (600 MHz, $CDCl_3$) δ = 7.82 (dd, *J* = 8.0, 1.4 Hz, 1H), 7.73 – 7.60 (m, 4H), 7.50 – 7.45 (m, 4H), 7.40 – 7.33 (m, 7H), 7.32 – 7.22 (m, 8H), 7.22 – 7.18 (m, 1H), 6.96 – 6.91 (m, 2H), 6.88 – 6.82 (m, 5H), 6.50 – 6.42 (m, 1H), 5.52 (s, 1H), 4.95 – 4.84 (m, 3H), 4.67 (d, *J* = 12.0 Hz, 1H), 4.65 (d, *J* = 12.0 Hz, 1H), 4.56 (s, 1H), 4.53 (d, *J* = 11.9 Hz, 1H), 4.49 – 4.44 (m, 3H), 4.34 (d, *J* = 12.0 Hz, 1H), 4.19 (dd, *J* = 10.4, 4.8 Hz, 1H), 4.15 (ddd, *J* = 9.8, 5.9, 2.4 Hz, 1H), 4.07 (apparent t, *J* = 9.6 Hz, 1H), 3.79 (s, 3H), 3.74 – 3.67 (m, 3H), 3.59 (apparent t, *J* = 10.3 Hz, 1H), 3.55 (dd, *J* = 11.1, 2.7 Hz, 1H), 3.39 (dd, *J* = 9.9, 3.1 Hz, 1H), 3.14 (apparent td, *J* = 9.7, 4.8 Hz, 1H), 1.48 (tt, *J* = 6.8, 5.4 Hz, 1H), 0.89 – 0.80 (m, 4H) ppm; ^{13}C NMR (151 MHz, $CDCl_3$) δ = 163.4, 159.3, 138.9, 138.7, 137.8, 137.7, 134.5, 134.0, 132.4, 131.1, 130.7, 129.7, 129.2, 129.0, 128.7, 128.5, 128.4, 128.3, 128.2, 128.1, 127.94, 127.89, 127.8, 127.2, 127.1, 126.2, 125.8, 123.5, 113.9, 101.9, 101.5, 100.5, 90.8, 78.9, 78.8, 78.1, 77.0, 76.9, 75.4, 75.1, 74.9, 74.5, 73.8, 72.4, 68.7, 68.2, 67.5, 55.4, 54.8, 9.1, 0.8 ppm. HRMS (ESI-ToF) *m*/*z* calculated for $C_{68}H_{63}NNaO_{14}^+$ $[M+Na]^+$: 1140.4141, found: 1140.4160.

5-Azidopentyl 2-*O*-benzyl-3-*O*-(4-methoxybenzyl)-4,6-benzylidene-β-D-mannopyranosyl-(1→4)-3,6-di-*O*-benzyl-2-deoxy-2-phthalimido-β-D-glucopyranosyl-(1→4)-3,6-di-*O*-benzyl-2-deoxy-2-phthalimido-β-D-glucopyranoside (S7): following General Procedure III, to a solution of glycosyl *o*-alkynylbenzoate **S7** (1.79 g, 1.60 mmol, 1.0 equiv.), secondary alcohol **12** (990 mg, 1.65 mmol, 1.03 equiv.) and 4Å MS (4.0 g, flame dried) in CH_2Cl_2 (30 mL) at 0 °C was added freshly prepared $Ph_3PAuOTf$ (6.4 mL, *ca.* 0.05 M in CH_2Cl_2). The reaction mixture was stirred at the same temperature for another 30 min before it was directly subjected onto a

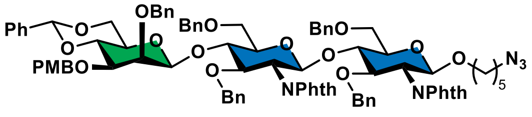

S7

flash column (silica gel, EtOAc:hexanes = 1:4 → 1:2 *v*/*v*) to afford chitobiose trisaccharide **S7** (1.90 g, 78%) as a pale yellow foam. **S7**: ^{1}H NMR (600 MHz, $CDCl_3$) δ = 7.87 (br d, *J* = 7.4 Hz, 1H), 7.82 – 7.61 (m, 6H), 7.56 (br s, 1H), 7.50 – 7.43 (m, 4H), 7.38 – 7.21 (m, 18H), 6.99 – 6.95 (m, 2H), 6.95 – 6.91 (m, 2H), 6.89 – 6.82 (m, 5H), 6.80 – 6.74 (m, 3H), 5.50 (s, 1H), 5.28 (d, *J* = 8.2 Hz, 1H), 4.97 – 4.92 (m, 1H), 4.89 (d, *J* = 12.4 Hz, 1H), 4.88 – 4.82 (m, 3H), 4.66 (d, *J* = 11.9 Hz, 1H), 4.57 (s, 1H), 4.54 (d, *J* = 11.9 Hz, 1H), 4.53 – 4.49 (m, 4H), 4.41 (d, *J* = 12.4 Hz, 1H), 4.38

(d, J = 12.0 Hz, 1H), 4.26 (dd, J = 10.7, 8.3 Hz, 1H), 4.23 – 4.17 (m, 2H), 4.15 – 4.09 (m, 3H), 4.09 – 4.02 (m, 2H), 3.79 (s, 3H), 3.73 (d, J = 3.2 Hz, 1H), 3.68 (apparent dt, J = 10.0, 6.1 Hz, 1H), 3.61 (dd, J = 11.3, 2.0 Hz, 1H), 3.57 – 3.49 (m, 2H), 3.46 – 3.37 (m, 3H), 3.32 (ddd, J = 9.8, 3.9, 1.6 Hz, 1H), 3.25 (ddd, J = 10.0, 7.2, 5.7 Hz, 1H), 3.20 (apparent dt, J = 10.0, 2.6 Hz, 1H), 3.12 (apparent td, J = 9.7, 4.8 Hz, 1H), 2.93 – 2.81 (m, 2H), 1.41 – 1.24 (m, 4H), 1.12 – 1.02 (m, 2H) ppm; ^{13}C NMR (151 MHz, $CDCl_3$) δ = 168.6, 167.7, 159.3, 139.0, 138.80, 138.78, 138.6, 138.0, 137.8, 134.1, 133.9, 133.8, 131.6, 130.7, 129.2, 128.9, 128.7, 128.4, 128.34, 128.30, 128.26, 128.2, 127.94, 127.91, 127.81, 127.76, 127.7, 127.5, 127.4, 127.02, 126.98, 126.2, 123.8, 123.2, 113.9, 102.0, 101.4, 98.2, 97.2, 79.4, 78.8, 78.1, 77.3, 77.1, 76.9, 76.0, 75.2, 74.8, 74.72, 74.66, 74.5, 73.4, 72.8, 72.4, 69.0, 68.7, 68.4, 68.1, 67.5, 56.7, 55.9, 55.4, 51.2, 28.8, 28.4, 23.1 ppm.

5-Azidopentyl 2-*O*-benzyl-4,6-benzylidene-β-D-mannopyranosyl-(1→4)-3,6-di-*O*-benzyl-2-deoxy-2-phthalimido-β-D-glucopyranosyl-(1→4)-3,6-di-*O*-benzyl-2-deoxy-2-phthalimido-β-D-glucopyranoside (14): to a solution of PMB ether **S7** (3.53 g, 2.30 mmol, 1.0 equiv.) in a mixture of CH_2Cl_2 (80 mL) and H_2O (8 mL) at 0 °C was added DDQ (627 mg, 2.76 mmol, 1.2 equiv.). The reaction suspension was vigorously stirred for another 6 hrs before it was quenched with saturated aqueous $NaHCO_3$ (30 mL). The organic phase was separated and the aqueous phase was extracted with CH_2Cl_2 (30 mL). The organic phases were combined, dried over Na_2SO_4 and concentrated under vacuum. The residue was purified by flash chromatography (silica gel, EtOAc:hexanes = 1:4 → 1:2, *v*/*v*) to afford secondary alcohol **14** (2.66 g, 82%) as a white foam.

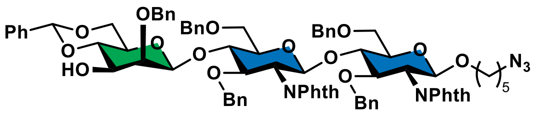

14

14: ^{1}H NMR (600 MHz, $CDCl_3$) δ = 7.88 (d, J = 7.4 Hz, 1H), 7.83 – 7.62 (m, 6H), 7.57 (br s, 1H), 7.48 – 7.43 (m, 2H), 7.42 – 7.26 (m, 18H), 7.04 – 6.97 (m, 2H), 6.97 – 6.92 (m, 2H), 6.91 – 6.85 (m, 3H), 6.84 – 6.76 (m, 3H), 5.43 (s, 1H), 5.29 (d, J = 7.3 Hz, 1H), 5.02 (d, J = 11.6 Hz, 1H), 4.95 (d, J = 6.9 Hz, 1H), 4.91 (d, J = 12.4 Hz, 1H), 4.88 (d, J = 13.1 Hz, 1H), 4.72 – 4.66 (m, 2H), 4.62 (d, J = 12.0 Hz, 1H), 4.58 – 4.50 (m, 3H), 4.47 (d, J = 12.1 Hz, 1H), 4.41 (d, J = 12.3 Hz, 1H), 4.30 – 4.20 (m, 3H), 4.18 – 4.09 (m, 4H), 3.75 – 3.63 (m, 4H), 3.61 – 3.54 (m, 2H), 3.54 – 3.39 (m, 3H), 3.34 (dd, J = 9.9, 3.7 Hz, 1H), 3.26 (apparent dt, J = 9.8, 6.4 Hz, 1H), 3.20 (d, J = 10.0 Hz, 1H), 3.12 (apparent td, J = 9.7, 4.9 Hz, 1H), 2.94 – 2.78 (m, 2H), 2.37 (d, J = 8.6 Hz, 1H), 1.43 – 1.20 (m, 4H), 1.14 – 1.00 (m, 2H) ppm; ^{13}C NMR (151 MHz, $CDCl_3$) δ = 168.6, 168.1, 167.7, 138.8, 138.7, 138.6, 138.2, 137.8, 137.3, 134.1, 133.9, 133.8, 131.8, 131.5, 129.2, 128.7, 128.6, 128.33, 128.27, 128.15, 128.08, 128.06, 128.04, 127.99, 127.9, 127.7, 127.39, 127.36, 127.1, 127.0, 126.4, 123.7, 123.2, 102.01, 102.00, 98.2, 97.1, 79.4, 79.2, 79.0, 77.1, 76.8, 75.90, 75.88, 74.8, 74.64, 74.55, 74.4, 73.5, 72.7, 71.0, 69.0, 68.5, 68.3, 67.8, 66.9, 56.6, 55.8, 51.1, 28.7, 28.3, 23.1 ppm.

Scheme S4. Synthesis of Mannosyl Fluoride Donors 15, 16 and 18[a]

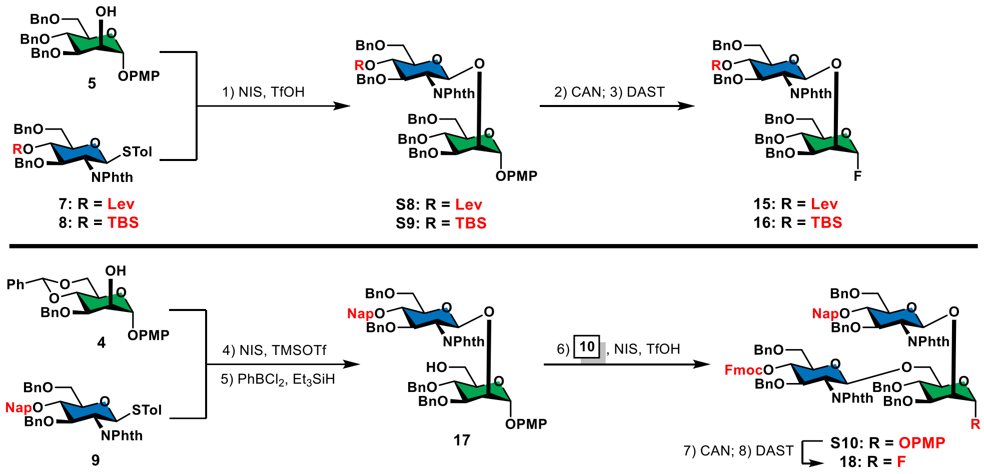

[a]Reagents and conditions: 1) **5** (1.0 equiv.), **7** (1.05 equiv.) or **8** (1.1 equiv.), NIS (1.2 equiv.), TfOH (0.1 equiv.), 4Å MS, CH_2Cl_2, −50 → 0 °C, 30 min, 64% for **S8**, 83% for **S9**; 2) CAN (3.0 equiv.), PhMe:ACN:H_2O (1:4:1, *v*/*v*/*v*), 0 °C, 70 min; 3) DAST (2.0 equiv.), 4Å MS, CH_2Cl_2, −40 °C, 30 min, 72% for **15** over the two steps, 75% for **16** over the two steps; 4) **4** (1.0 equiv.), **9** (1.1 equiv.), NIS (1.2 equiv.), TMSOTf (0.1 equiv.), 4Å MS, CH_2Cl_2, −40 → 0 °C, 30 min; 5) $PhBCl_2$ (2.0 equiv.), Et_3SiH (5.0 equiv.), 4Å MS, CH_2Cl_2, −78 °C, 2 hrs, 70% over the two steps; 6) **17** (1.0 equiv.), **10** (1.25 equiv.), NIS (1.25 equiv.), TfOH (0.15 equiv.), 4Å MS, CH_2Cl_2, −40 → 0 °C, 30 min, 82%; 7) CAN (3.0 equiv.), PhMe:ACN:H_2O (1:4:1, *v*/*v*/*v*), 0 °C, 1 hr; 8) DAST (2.0 equiv.), 4Å MS, CH_2Cl_2, −40 °C, 30 min, 69% over the two steps.

4-Methoxyphenyl 4-*O*-levulinoyl-3,6-di-*O*-benzyl-2-deoxy-2-phthalimido-β-D-glucopyranosyl-(1→2)-3,4,6-tri-*O*-benzyl-α-D-mannopyranoside (S8): following General Procedure I, levulinate **S8** was synthesized from **5** (748 mg, 1.34 mmol, 1.0 equiv.), **7** (980 mg, 1.41 mmol, 1.05 equiv.), NIS (362 mg, 1.61 mmol, 1.2 equiv.) and TfOH (20.1 mg, 11.9 µL, 0.134 mmol, 0.1 equiv.) in CH_2Cl_2 (25 mL) with 4Å MS (2.5 g, flame dried). Flash chromatography (silica gel, EtOAc:hexanes = 1:4 → 1:1, *v*/*v*) afforded pure compound **S8**

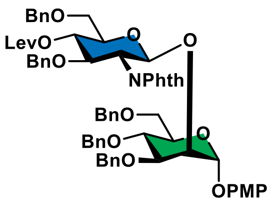

S8

(968 mg, 64%) as a white foam. **S8**: ^{1}H NMR (600 MHz, $CDCl_3$) δ = 7.61 (br s, 2H), 7.52 (br s, 2H), 7.41 – 7.36 (m, 2H), 7.33 – 7.20 (m, 14H), 7.17 – 7.13 (m, 2H), 7.05 – 7.00 (m, 4H), 6.94 – 6.89 (m, 2H), 6.89 – 6.85 (m, 1H), 6.74 – 6.69 (m, 2H), 6.69 – 6.65 (m, 2H), 5.35 (d, *J* = 8.1 Hz, 1H), 5.18 (dd, *J* = 10.1, 8.5 Hz, 1H), 5.07 (d, *J* = 2.3 Hz, 1H), 4.85 – 4.79 (m, 2H), 4.68 (d, *J* = 12.3 Hz, 1H), 4.56 (d, *J* = 11.5 Hz, 1H), 4.52 (ABq, *J* = 11.7 Hz, $\Delta\nu_{AB}$ = 9.2 Hz, 2H), 4.47 – 4.40 (m, 2H), 4.39 (d, *J* = 11.0 Hz, 1H), 4.36 (d, *J* = 12.3 Hz, 1H), 4.26 (dd, *J* = 3.2, 2.3 Hz, 1H), 3.99 – 3.93 (m, 3H), 3.83 (ddd, *J* = 9.9, 6.2, 3.4 Hz, 1H), 3.73 (s, 3H), 3.72 – 3.63 (m, 3H), 3.58 (dd, *J* = 9.9, 8.7 Hz, 1H), 3.41 (dd, *J* = 11.0, 1.9 Hz, 1H), 3.05 (dd, *J* = 11.0, 6.6 Hz, 1H), 2.71 – 2.59 (m, 2H), 2.47 (t, *J* = 6.3 Hz, 2H), 2.15 (s, 3H) ppm; ^{13}C NMR (151 MHz, $CDCl_3$) δ = 206.3, 171.8, 154.9, 150.2, 138.6, 138.5, 138.3, 138.1, 137.9, 133.8, 133.6, 131.9, 131.8, 123.4, 123.0, 117.7, 114.5, 97.4, 96.1, 77.6, 76.8, 75.0, 74.8, 74.2, 74.0, 73.8, 73.7, 72.9, 72.7, 72.1, 71.2, 70.3, 70.0, 55.7, 55.6, 37.8, 29.9, 28.1 ppm. HRMS (ESI-ToF) *m*/*z* calculated for $C_{67}H_{67}NNaO_{15}^+$ [M+Na]$^+$: 1148.4403, found: 1148.4392.

4-*O*-levulinoyl-3,6-di-*O*-benzyl-2-deoxy-2-phthalimido-β-D-glucopyranosyl-(1→2)-3,4,6-tri-*O*-benzyl-α-D-mannopyranosyl fluoride (15): following General Procedures IV and V, mannosyl fluoride **15** was synthesized from PMP ether **S8** (950 mg, 0.843 mmol, 1.0 equiv.), CAN (2.5 mL, *ca.* 1.0 M in H_2O, 2.5 mmol, 3.0 equiv.) and DAST (179 µL, 218 mg, 1.35 mmol, 2.0 equiv. of lactol intermediate). Flash chromatography (silica gel, EtOAc:hexanes = 1:4 → 1:2, *v*/*v*) afforded mannosyl fluoride **15** (620 mg, 72% over the two steps) as a white foam. **15**: ^{1}H NMR (600 MHz, $CDCl_3$) δ = 7.76 – 7.47 (m, 4H), 7.35 – 7.22 (m, 16H), 7.12 – 7.07 (m, 4H), 7.04 – 6.99 (m, 2H), 6.93 – 6.89 (m, 2H), 6.89 – 6.85 (m, 1H), 5.34 – 5.31 (m, 1H), 5.30 (dd, *J* = 50.2, 2.2 Hz, 1H), 5.20 – 5.15 (m, 1H), 4.78 (d, *J* = 11.3 Hz, 2H overlapped), 4.67 (d, *J* = 12.2 Hz, 1H), 4.53 – 4.47 (m, 3H), 4.43 – 4.37 (m, 2H), 4.35 (d, *J* = 1.5 Hz, 1H), 4.34 (d, *J* = 2.9 Hz, 1H), 4.21 (apparent t, *J* = 2.7 Hz, 1H), 4.11 (d, *J* = 12.2 Hz, 1H), 4.04 (d, *J* = 12.2 Hz, 1H), 3.83 (ddd, *J* = 9.9, 6.3, 3.3 Hz, 1H), 3.76 (apparent dt, *J* = 8.9, 2.6 Hz, 1H), 3.71 – 3.65 (m, 2H), 3.63 (dd, *J* = 10.5, 3.3 Hz, 1H), 3.59 (apparent t, *J* = 9.4 Hz, 1H), 3.36 (dd, *J* = 11.0, 1.7 Hz, 1H), 3.05 (dd, *J* = 11.0, 5.9 Hz, 1H), 2.66 (t, *J* = 6.5 Hz, 2H), 2.47 (td, *J* = 6.3, 2.1 Hz, 2H), 2.15 (s, 3H) ppm; ^{13}C NMR (151 MHz, $CDCl_3$) δ = 206.3, 171.8, 138.2, 138.1, 138.0, 138.0, 137.8, 133.9, 131.6, 128.5, 128.5, 128.5, 128.4, 128.4, 128.2, 128.2, 128.1, 128.1, 128.0, 127.8, 127.8, 127.7, 127.6, 127.5, 123.4, 105.5 (d, *J* = 222.9 Hz), 97.3, 76.9, 76.6, 75.1, 74.1, 74.0, 73.8, 73.7, 73.7, 73.0, 72.8, 72.5 (d, *J* = 34.8 Hz), 71.1, 70.1, 69.1, 55.4, 37.8, 29.9, 28.0 ppm.

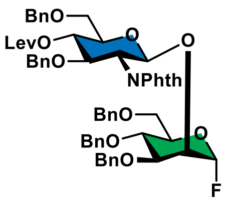

15

4-Methoxyphenyl 4-*O*-*tert*-butyldimethylsilyl-3,6-di-*O*-benzyl-2-deoxy-2-phthalimido-β-D-glucopyranosyl-(1→2)-3,4,6-tri-*O*-benzyl-α-D-mannopyranoside (S9): following General Procedure I, TBS ether **S9** was synthesized from **5** (720 g, 1.29 mmol, 1.0 equiv.), **8** (1.01 g, 1.42 mmol, 1.1 equiv.), NIS (349 mg, 1.55 mmol, 1.2 equiv.) and TMSOTf (35 µL, 43.0 mg, 0.194 mmol, 0.15 equiv.) in CH_2Cl_2 (20 mL) with 4Å MS (1.5 g, flame dried). Flash chromatography (silica gel, EtOAc:hexanes = 1:10 → 1:3, *v*/*v*) afforded pure compound **S9** (1.23 g, 83%) as a white foam. **S9**: ^{1}H NMR (600 MHz, $CDCl_3$) δ = 7.50 – 7.44 (m, 2H), 7.42 – 7.34 (m, 2H), 7.34 – 7.30 (m, 2H), 7.24 – 7.11 (m, 14H), 7.08 – 7.04 (m, 2H), 6.95 – 6.89 (m, 4H), 6.81 – 6.76 (m, 2H), 6.73 – 6.69 (m, 1H), 6.63 – 6.55 (m, 4H), 5.23 (d, *J* = 8.5 Hz, 1H), 4.95 (d, *J* = 2.3 Hz, 1H), 4.79 (d, *J* = 11.6 Hz, 1H), 4.74 (d, *J* = 10.9 Hz, 1H), 4.67 (d, *J* = 12.3 Hz, 1H), 4.54 (d, *J* = 12.1 Hz, 1H), 4.47 (d, *J* = 11.6 Hz, 1H), 4.38 (d, *J* = 12.1 Hz, 1H), 4.29 (d, *J* = 11.0 Hz, 1H), 4.27 – 4.20 (m, 2H), 4.19 (apparent t, *J* = 2.8 Hz, 1H), 4.16 – 4.10 (m, 1H), 3.91 – 3.83 (m, 3H), 3.72 (d, *J* = 10.2 Hz, 1H), 3.63 (s, 3H), 3.62 – 3.60 (m, 2H), 3.59 – 3.50 (m, 2H), 3.46 (apparent t, *J* = 9.3 Hz, 1H), 3.31 (dd, *J* = 11.0, 1.8 Hz, 1H), 2.92 (dd, *J* = 11.0, 6.7 Hz, 1H), 0.77 (s, 9H), −0.00 (s, 3H), −0.08 (s, 3H) ppm; ^{13}C NMR (151 MHz, $CDCl_3$) δ = 154.8, 150.1, 138.51, 138.47, 138.4, 138.2, 138.1, 133.5, 131.8, 128.4, 128.30, 128.29, 128.27, 128.1, 128.0, 127.72, 127.67, 127.6, 127.54, 127.50, 127.3, 127.2, 127.1, 123.0, 117.6, 114.4, 97.3, 96.1, 80.6, 77.4, 76.4, 75.4, 74.9,

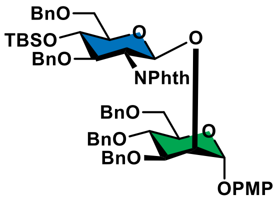

S9

74.7, 73.7, 73.5, 73.1, 72.6, 72.0, 70.7, 70.0, 69.9, 56.0, 55.6, 25.9, 18.0, −3.7, −4.4 ppm. HRMS (ESI-ToF) *m/z* calculated for $C_{68}H_{75}NNaO_{13}Si^+$ $[M+Na]^+$: 1164.4900, found: 1164.4859.

4-*O*-*tert*-butyldimethylsilyl-3,6-di-*O*-benzyl-2-deoxy-2-phthalimido-β-D-glucopyranosyl-(1→2)-3,4,6-tri-*O*-benzyl-α-D-mannopyranosyl fluoride (16): following General Procedures IV and V, mannosyl fluoride **16** was synthesized from PMP ether **S9** (1.23 g, 1.08 mmol, 1.0 equiv.), CAN (2.69 mL, *ca.* 1.0 M in H_2O, 2.69 mmol, 2.5 equiv.) and DAST (208 μL, 254 mg, 1.58 mmol, 2.0 equiv. of lactol intermediate). Flash chromatography (silica gel, EtOAc:hexanes = 1:4 → 1:3, *v/v*) afforded mannosyl fluoride **16** (838 mg, 75% over the two steps) as a white foam.

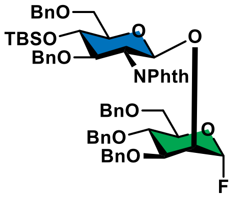

16

16: ^1H NMR (600 MHz, $CDCl_3$) δ = 7.69 – 7.47 (m, 4H), 7.38 – 7.33 (m, 2H), 7.31 – 7.21 (m, 14H), 7.13 – 7.06 (m, 4H), 7.01 – 6.97 (m, 2H), 6.91 – 6.86 (m, 2H), 6.84 – 6.79 (m, 1H), 5.30 (d, *J* = 8.5 Hz, 1H), 5.29 (dd, *J* = 50.3, 2.2 Hz, 1H), 4.83 (d, *J* = 11.7 Hz, 1H), 4.80 – 4.74 (m, 2H), 4.61 (d, *J* = 12.1 Hz, 1H), 4.51 (d, *J* = 11.8 Hz, 1H), 4.46 (d, *J* = 12.1 Hz, 1H), 4.35 (d, *J* = 10.9 Hz, 1H), 4.33 – 4.27 (m, 2H), 4.24 (apparent t, *J* = 2.7 Hz, 1H), 4.18 (ddd, *J* = 10.8, 5.5, 2.7 Hz, 1H), 4.10 (d, *J* = 12.2 Hz, 1H), 4.02 (d, *J* = 12.2 Hz, 1H), 3.80 (dd, *J* = 10.4, 1.2 Hz, 1H), 3.76 (apparent dt, *J* = 8.9, 2.7 Hz, 1H), 3.72 – 3.63 (m, 3H), 3.62 – 3.52 (m, 2H), 3.35 (dd, *J* = 10.9, 1.8 Hz, 1H), 2.99 (dd, *J* = 11.0, 6.1 Hz, 1H), 0.87 (s, 9H), 0.09 (s, 3H), 0.01 (s, 3H) ppm; ^{13}C NMR (151 MHz, $CDCl_3$) δ = 138.2, 138.10, 138.06, 138.05, 133.6, 131.6, 128.4, 128.30, 128.27, 128.2, 128.04, 127.98, 127.7, 127.63, 127.60, 127.56, 127.51, 127.46, 127.1, 123.2, 105.4 (d, *J* = 222.8 Hz), 97.1, 80.7, 76.43, 76.39, 75.4, 74.9, 73.8, 73.6, 73.5, 73.0, 72.9, 72.1, 71.9, 70.6, 69.8, 69.1, 55.8, 25.9, 18.0, −3.7, −4.4 ppm.

4-Methoxyphenyl 4-*O*-(2-naphthylmethyl)-3,6-di-*O*-benzyl-2-deoxy-2-phthalimido-β-D-glucopyranosyl-(1→2)-3,4-di-*O*-benzyl-α-D-mannopyranoside (17): following General Procedure I, 2-naphthylmethyl disaccharide was synthesized from benzylidene **4** (1.25 g, 2.69 mmol, 1.0 equiv.) and thioglycoside **9** (2.18 g, 2.96 mmol, 1.1 equiv.), NIS (727 mg, 3.23 mmol, 1.2 equiv.) and TMSOTf (73.0 μL, 90.0 mg, 0.405 mmol, 0.15 equiv.) in CH_2Cl_2 (40 mL) with 4Å MS (3 g, flame dried). Flash chromatography (silica gel, EtOAc:hexanes = 1:4 → 1:2, *v/v*) afforded disaccharide (1.80 g) as a pale yellow foam which was directly used for the next step.

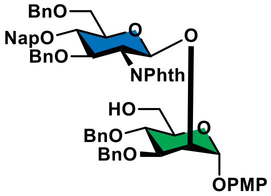

17

To a suspension of disaccharide (1.80 g, 1.67 mmol, 1.0 equiv.) and 4Å MS (1.8 g, flame dried) in CH_2Cl_2 (30 mL) at −78 °C were sequentially added Et_3SiH (1.34 mL, 972 mg, 8.36 mmol, 5.0 euqiv.) and $PhBCl_2$ (436 μL, 532 mg, 3.34 mmol, 2.0 equiv.). The reaction mixture was stirred at the same temperature for another 2 hrs before it was quenched carefully by dropwise addition of MeOH/Et_3N (0.5 mL, 1:1, *v/v*) at −78 °C. The resulting reaction mixture was filtered, and the filtrate was extracted with CH_2Cl_2 (20 mL × 3). The organic phases were combined, dried over Na_2SO_4, and concentrated under vacuum. The colorless slurry was purified by flash chromatography (silica gel, EtOAc:toluene = 1:10 → 1:7, *v/v*) to afford

primary alcohol **17** (1.55 g, 86%) as a white foam. **17**: ^{1}H NMR (600 MHz, $CDCl_3$) δ = 7.92 – 7.67 (m, 7H), 7.63 (br s, 1H), 7.54 – 7.46 (m, 2H), 7.44 – 7.39 (m, 2H), 7.36 (dd, *J* = 8.5, 1.7 Hz, 1H), 7.33 – 7.19 (m, 13H), 7.10 – 7.05 (m, 2H), 6.91 (apparent t, *J* = 7.4 Hz, 2H), 6.87 – 6.83 (m, 1H), 6.70 – 6.60 (m, 2H), 6.49 – 6.42 (m, 2H), 5.20 (d, *J* = 8.5 Hz, 1H), 5.03 (d, *J* = 11.1 Hz, 1H), 4.92 (d, *J* = 2.3 Hz, 1H), 4.90 – 4.80 (m, 4H), 4.62 – 4.55 (m, 2H), 4.55 – 4.48 (m, 4H), 4.38 (dd, *J* = 10.8, 8.5 Hz, 1H), 4.20 (apparent t, *J* = 2.7 Hz, 1H), 3.97 (dd, *J* = 9.2, 3.0 Hz, 1H), 3.85 – 3.74 (m, 5H), 3.74 (s, 3H), 3.41 (ddd, *J* = 10.0, 4.8, 2.5 Hz, 1H), 3.33 (dd, *J* = 12.0, 2.6 Hz, 1H), 3.22 (dd, *J* = 12.0, 4.7 Hz, 1H) ppm; ^{13}C NMR (151 MHz, $CDCl_3$) δ = 154.9, 149.8, 138.5, 138.33, 138.27, 138.1, 135.4, 134.3, 133.8, 133.4, 133.1, 132.1, 131.6, 128.53, 128.47, 128.42, 128.40, 128.35, 128.23, 128.21, 128.11, 128.07, 127.9, 127.82, 127.79, 127.75, 127.72, 127.5, 127.0, 126.3, 126.2, 126.2, 123.4, 123.1, 116.9, 114.5, 97.7, 95.8, 79.7, 79.2, 75.3, 74.9, 74.1, 74.0, 73.7, 72.4, 71.0, 69.5, 62.4, 56.3, 55.7 ppm. HRMS (ESI-ToF) *m/z* calculated for $C_{66}H_{63}NNaO_{13}^{+}$ $[M+Na]^{+}$: 1100.4192, found: 1100.4180.

4-Methoxyphenyl 4-*O*-(2-naphthylmethyl)-3,6-di-*O*-benzyl-2-deoxy-2-phthalimido-β-D-glucopyranosyl-(1→2)-[4-*O*-fluorenylmethoxycarbonyl-3,6-di-*O*-benzyl-2-deoxy-2-phthalimido-β-D-glucopyranosyl-(1→6)]-3,4-di-*O*-benzyl-mannopyranoside (S10): following General Procedure I, trisaccharyl PMP ether **S10** was synthesized from primary alcohol **17** (1.54 g, 1.43 mmol, 1.0 equiv.), Fmoc protected thioglycoside **10** (1.46 g, 1.78 mmol, 1.25 equiv.), NIS (402 mg, 1.78 mmol, 1.25 equiv.) and TfOH (32.2 mg, 19.0 µL, 0.214 mmol, 0.15 equiv.) in CH_2Cl_2 (30 mL) with 4Å MS (3.0 g, flame dried). Flash chromatography (silica gel, EtOAc:hexanes = 1:5 → 1:3, *v*/*v*) afforded trisaccharide **S10** (2.07 g, 82%) as a white amorphous foam.

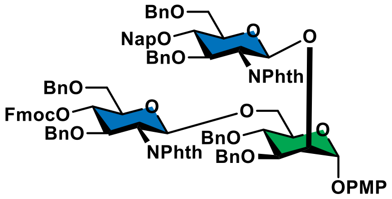

S10

S10: ^{1}H NMR (600 MHz, $CDCl_3$) δ = 7.85 – 7.67 (m, 8H), 7.66 – 7.42 (m, 11H), 7.41 – 7.28 (m, 6H), 7.28 – 7.18 (m, 16H), 7.08 – 7.02 (m, 4H), 6.96 – 6.89 (m, 4H), 6.88 – 6.82 (m, 4H), 6.63 – 6.59 (m, 2H), 6.59 – 6.56 (m, 2H), 5.24 (d, *J* = 8.4 Hz, 1H), 5.00 (d, *J* = 11.2 Hz, 1H), 4.92 (d, *J* = 2.5 Hz, 1H), 4.91 (d, *J* = 8.4 Hz, 1H), 4.84 – 4.69 (m, 4H), 4.58 – 4.54 (m, 2H), 4.53 – 4.44 (m, 4H), 4.42 (dd, *J* = 10.8, 8.6 Hz, 1H), 4.35 (ABq, *J* = 3.7 Hz, $\Delta\nu_{AB}$ = 0.2 Hz, 2H), 4.34 – 4.26 (m, 4H), 4.23 (d, *J* = 12.1 Hz, 1H), 4.14 (apparent t, *J* = 2.8 Hz, 1H), 4.13 – 4.09 (m, 2H), 3.93 (dd, *J* = 10.7, 8.5 Hz, 1H), 3.84 (dd, *J* = 8.5, 3.1 Hz, 1H), 3.79 (dd, *J* = 9.8, 8.5 Hz, 1H), 3.77 – 3.75 (m, 2H), 3.74 (s, 3H), 3.71 – 3.65 (m, 2H), 3.60 (ddd, *J* = 9.7, 5.4, 4.2 Hz, 1H), 3.55 (dd, *J* = 11.2, 2.3 Hz, 1H), 3.40 (dd, *J* = 10.5, 4.1 Hz, 1H), 3.34 – 3.26 (m, 2H), 3.05 (dd, *J* = 11.1, 6.6 Hz, 1H) ppm; ^{13}C NMR (151 MHz, $CDCl_3$) δ = 154.9, 154.5, 150.5, 143.5, 143.2, 141.5, 141.4, 138.41, 138.39, 138.21, 138.19, 137.7, 135.6, 133.8, 133.4, 133.1, 131.7, 128.5, 128.4, 128.32, 128.29, 128.2, 128.13, 128.10, 128.02, 128.00, 127.93, 127.90, 127.83, 127.80, 127.7, 127.61, 127.59, 127.54, 127.48, 127.4, 127.28, 127.27, 126.9, 126.3, 126.1, 125.2, 125.1, 120.2, 117.9, 114.4, 98.7, 97.4, 96.5, 79.8, 79.5, 77.61, 77.59, 76.6, 75.3, 75.2, 75.0, 74.9, 74.4, 74.2, 73.9, 73.7, 73.5, 72.7, 71.4, 71.1, 70.1, 70.0, 69.8, 69.5, 56.0, 55.7, 55.4, 46.8 ppm.

4-*O*-(2-naphthylmethyl)-3,6-di-*O*-benzyl-2-deoxy-2-phthalimido-β-D-glucopyranosyl-(1→2)-[4-*O*-fluorenylmethoxycarbonyl-3,6-di-*O*-benzyl-2-deoxy-2-phthalimido-β-D-glucopyranosyl-(1→6)]-3,4-di-*O*-benzyl-mannopyranosyl fluoride (18): following General Procedure IV and V, mannosyl fluoride **18** was synthesized from PMP ether **S10** (1.82 g, 1.03 mmol, 1.0 equiv.), CAN (2.57 mL, *ca.* 1.0 M in H_2O, 2.69 mmol, 2.5 equiv.) and DAST (193 µL, 235 mg, 1.46 mmol, 2.0 equiv. of lactol intermediate). Flash chromatography (silica gel, EtOAc:hexanes = 1:4 → 1:3, *v*/*v*) afforded mannosyl fluoride **18** (1.18 g, 69% over the two steps) as a white foam, which was directly used for the pending glycosylation reaction.

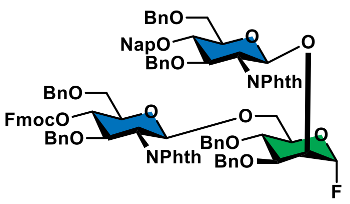

18

Scheme S5. Assembly of Heptasaccharide Common Precursors 1[a]

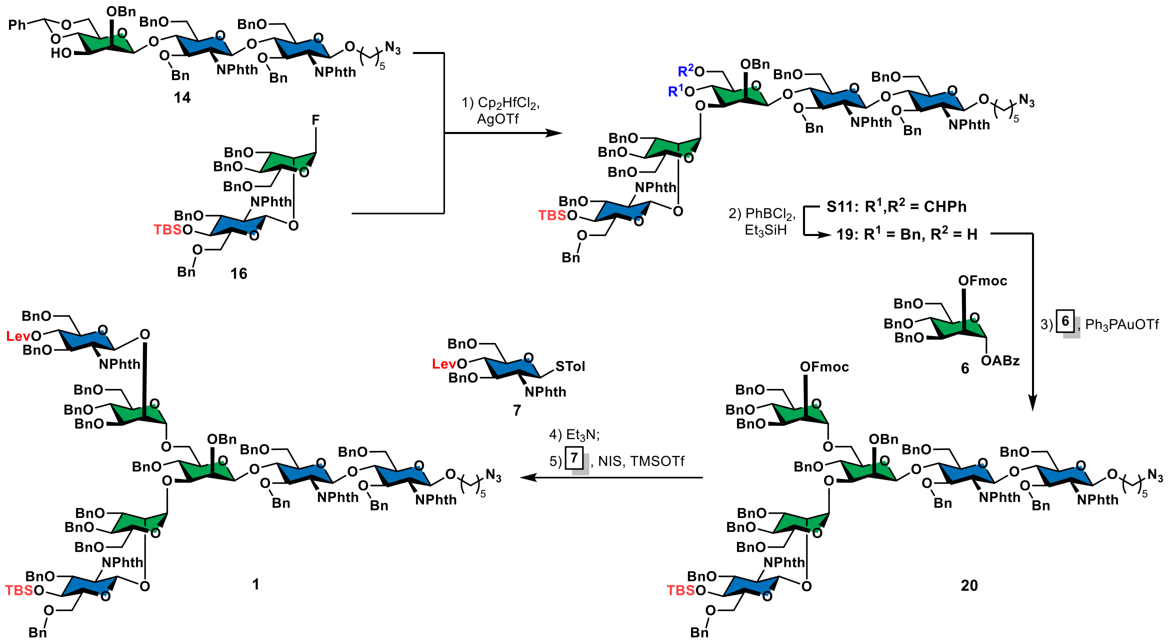

[a]Reagents and conditions: 1) **14** (1.0 equiv.), **16** (1.2 equiv.), Cp_2HfCl_2 (3.5 equiv.), AgOTf (5.0 equiv.), 4Å MS, PhMe, −60 → −30 °C, 1 hr, 70%; 2) $PhBCl_2$ (2.0 equiv.), Et_3SiH (5.0 equiv.), 4Å MS, CH_2Cl_2, −78 → −60 °C, 2 hrs, 78%; 3) **19** (1.0 equiv.), **6** (3.0 equiv.), $Ph_3PAuOTf$ (0.5 equiv.), 4Å MS, CH_2Cl_2, 0 °C, 30 min, 80%; 4) Et_3N:CH_2Cl_2 = 1:10 (*v*/*v*), r.t., 12 hrs; 5) **20** (1.0 equiv.), **7** (3.0 equiv.), NIS (3.0 equiv.), TMSOTf (0.6 equiv.), 4Å MS, CH_2Cl_2, −40 → 0 °C, 54% for the two steps.

5-Azidopentyl 4-*O*-*tert*-butyldimethylsilyl-3,6-di-*O*-benzyl-2-deoxy-2-phthalimido-β-D-glucopyranosyl-(1→2)-3,4,6-tri-*O*-benzyl-α-D-mannopyranosyl-(1→3)-2-*O*-benzyl-4,6-benzylidene-β-D-mannopyranosyl-(1→4)-3,6-di-*O*-benzyl-2-deoxy-2-phthalimido-β-D-glucopyranosyl-(1→4)-3,6-di-*O*-benzyl-2-deoxy-2-phthalimido-β-D-glucopyranoside (S11): to a mixture of Cp_2HfCl_2 (470 mg, 1.24 mmol, 3.5 equiv.), AgOTf (455 mg, 1.77 mmol, 5.0 equiv.) and 4Å MS (1.8 g, flame dried) with vigorous stirring was added PhMe (18 mL) at room temperature. The suspension was stirred for another 3 hrs before it was cooled to −60 °C, at which point a solution of trisaccharide **14** (500 mg, 0.354 mmol, 1.0 equiv.) and mannosyl fluoride **16** (441 mg, 0.425 mmol, 1.2 equiv.) in PhMe (4.0 mL) was slowly added into the suspension. The reaction mixture was slowly warmed up to −30 °C over 1 hr and then quenched by saturated aqueous $NaHCO_3$ (20 mL) and diluted with EtOAc (20 mL). The resulting suspension was filtered through Celite® and the organic layer of the filtrate was separated. The aqueous layer was extracted with EtOAc (20 mL × 3) and the organic layers were combined, dried over Na_2SO_4, and concentrated under vacuum. The residue was purified by flash chromatography (silica gel, EtOAc:hexanes = 1:4 → 2:3) to afford pentasaccharide **S11** (602 mg, 70%) as a white foam. **S11**: ^{1}H NMR (600 MHz, $CDCl_3$) δ = 7.82 – 6.64 (m, 67H), 5.18 (d, *J* = 7.8 Hz, 1H), 5.15 (s, 1H), 4.96 (d, *J* = 1.9 Hz, 1H), 4.86 (d, *J* = 7.9 Hz, 1H), 4.83 – 4.76 (m, 4H), 4.73 (d, *J* = 12.3 Hz, 1H), 4.63 (d, *J* = 11.9 Hz, 1H), 4.60 (d,

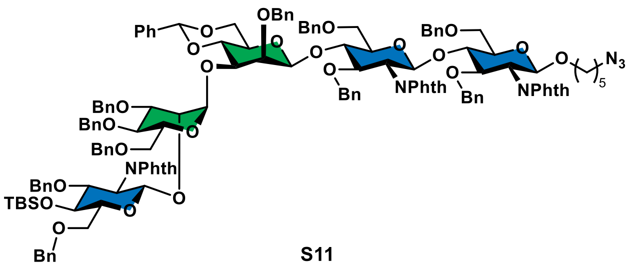

S11

J = 12.0 Hz, 1H), 4.57 (d, J = 11.8 Hz, 1H), 4.47 – 4.42 (m, 2H), 4.40 (d, J = 11.9 Hz, 1H), 4.35 – 4.22 (m, 6H), 4.19 (d, J = 11.9 Hz, 1H), 4.15 – 4.00 (m, 8H), 3.97 – 3.88 (m, 5H), 3.80 (d, J = 12.0 Hz, 1H), 3.74 – 3.67 (m, 2H), 3.60 (apparent dt, J = 10.2, 6.0 Hz, 1H), 3.54 – 3.39 (m, 7H), 3.37 – 3.31 (m, 2H), 3.30 – 3.20 (m, 4H), 3.20 – 3.15 (m, 1H), 3.13 (dd, J = 10.9, 2.7 Hz, 1H), 3.04 (apparent dt, J = 9.7, 2.3 Hz, 1H), 2.85 – 2.74 (m, 2H), 2.59 (apparent td, J = 9.7, 4.8 Hz, 1H), 2.45 = 2.36 (m, 2H), 1.34 – 1.16 (m, 4H), 1.04 – 0.95 (m, 2H), 0.89 (s, 9H), 0.00 (s, 3H), −0.06 (s, 3H) ppm.

5-Azidopentyl 4-*O*-*tert*-butyldimethylsilyl-3,6-di-*O*-benzyl-2-deoxy-2-phthalimido-β-D-glucopyranosyl-(1→2)-3,4,6-tri-*O*-benzyl-α-D-mannopyranosyl-(1→3)-2,4-di-*O*-benzyl-β-D-mannopyranosyl-(1→4)-3,6-di-*O*-benzyl-2-deoxy-2-phthalimido-β-D-glucopyranosyl-(1→4)-3,6-di-*O*-benzyl-2-deoxy-2-phthalimido-β-D-glucopyranoside (19): to a suspension of benzylidene **S11** (197 mg, 81.0 µmol, 1.0 equiv.), Et_3SiH (47.1 mg, 64.7 µL, 0.405 mmol, 5.0 equiv.) and 4Å MS (600 mg, flame dried) in CH_2Cl_2 (5.0 mL) at −78 °C was added $PhBCl_2$ (25.8 mg, 21.1 µL, 0.162 mmol, 2.0 equiv.). The reaction mixture was allowed to slowly warmed to −60 °C over 30 min and stirred at this temperature for another 2 hrs before it was carefully quenched by a mixture of Et_3N and MeOH (0.1 mL, 1:1, *v*/*v*) at −78 °C. The resulting white suspension was diluted with CH_2Cl_2 (10 mL), warmed to room temperature and further quenched by saturated aqueous $NaHCO_3$ (10 mL). The resulting suspension was filtered through Celite® and the organic layer of the filtrate was separated. The aqueous layer was extracted with CH_2Cl_2 (10 mL × 3) and the organic layers were combined, dried over Na_2SO_4, and concentrated under vacuum. The residue was purified by flash chromatography (silica gel, EtOAc:hexanes = 1:3 → 1:2) to afford primary alcohol **19** (154 mg, 78%) as a glassy film. **19**: ^{1}H NMR (600 MHz, $CDCl_3$) δ = 7.97 – 6.61 (m, 67H), 5.24 (d, J = 7.4 Hz, 1H), 5.03 – 4.96 (m, 2H), 4.94 (d, J = 7.7 Hz, 1H), 4.92 – 4.80 (m, 3H), 4.80 – 4.69 (m, 3H), 4.69 – 4.58 (m, 2H), 4.56 – 4.28 (m, 11H), 4.28 – 4.17 (m, 4H), 4.17 – 4.06 (m, 5H), 4.05 – 3.87 (m, 4H), 3.81 (dd, J = 9.2, 2.9 Hz, 1H), 3.77 – 3.55 (m, 5H), 3.54 – 3.45 (m, 4H), 3.45 – 3.19 (m, 9H), 3.12 (apparent dt, J = 9.8, 2.2 Hz, 1H), 2.92 – 2.80 (m, 2H), 2.74 – 2.70 (m, 1H), 2.70 – 2.61 (m, 1H), 2.61 – 2.55 (m, 1H), 1.40 – 1.22 (m, 4H), 1.12 – 1.00 (m, 2H), 0.90 (s, 9H), 0.05 (s, 3H), −0.02 (s, 3H) ppm; ^{13}C NMR (151 MHz, $CDCl_3$) δ = 168.6, 167.7, 138.9, 138.80, 138.78, 138.7, 138.5, 138.42, 138.37, 138.3, 137.9, 135.7, 134.3, 134.2, 133.9, 133.7, 133.4, 132.8, 131.9, 131.8, 131.5, 129.0, 128.6, 128.50, 128.47, 128.4, 128.34, 128.31, 128.29, 128.26, 128.24, 128.19, 128.17, 128.14, 128.10, 128.05, 128.04, 128.00, 127.93, 127.89, 127.7, 127.54, 127.47, 127.44, 127.40, 127.36, 127.14, 127.09, 127.07, 126.9, 126.8, 123.8, 123.2, 100.3, 98.6, 98.2, 97.4, 95.9, 80.7, 80.1, 78.2, 77.9, 77.3, 77.1, 76.8, 76.4, 75.7, 75.3, 75.2, 74.7, 74.6, 74.3, 74.2, 73.9, 73.4, 73.1, 72.9, 72.7, 72.4, 70.6, 70.4, 69.5, 69.0, 68.3, 67.6, 61.8, 56.6, 55.8, 51.2, 28.8, 28.4, 26.0, 23.1, 18.1, −3.6, −4.5 ppm.

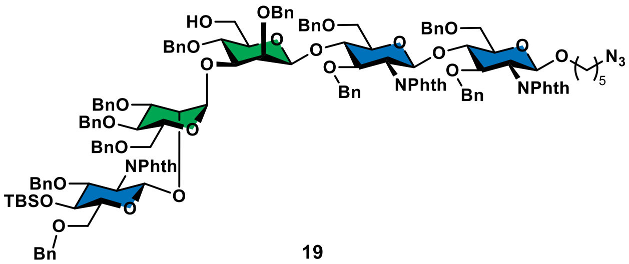

19

5-Azidopentyl 4-*O*-*tert*-butyldimethylsilyl-3,6-di-*O*-benzyl-2-deoxy-2-phthalimido-β-D-glucopyranosyl-(1→2)-3,4,6-tri-*O*-benzyl-α-D-mannopyranosyl-(1→3)-[3,4,6-tri-*O*-benzyl-2-*O*-fluorenylmethoxycarbonyl-α-D-mannopyranosyl-(1→6)]-2,4-di-*O*-benzyl-β-D-mannopyranosyl-(1→4)-3,6-di-*O*-benzyl-2-deoxy-2-phthalimido-β-D-glucopyranosyl-(1→4)-3,6-di-*O*-benzyl-2-deoxy-2-phthalimido-β-D-glucopyranoside (20): following General Procedure III, hexasaccharide **20** was synthesized from primary alcohol **19** (100 mg, 41.1 μmol, 1.0 equiv.), *o*-alkynylbenzoate donor **6** (52.0 mg, 61.7 mmol, 1.5 equiv.) and 4Å MS (250 mg, flame dried) in the presence of Ph_3AuOTf (412 μL, *ca.* 0.05 M, 20.6 μmol, 0.5 equiv.). Flash chromatography (silica gel, EtOAc:haxanes = 1:4 → 1:2, *v*/*v*) afforded hexasaccharide **20** (101 mg, 80%) as a glassy film. **20**: ^{1}H NMR (600 MHz, $CDCl_3$) δ = 7.83 – 6.79 (m, 84H), 6.79 – 6.67 (m, 3H), 6.62 – 6.47 (m, 3H), 5.15 (d, *J* = 7.9 Hz, 1H), 5.12 (apparent t, *J* = 2.1 Hz, 1H), 4.98 (d, *J* = 1.8 Hz, 1H), 4.95 – 4.91 (m, 2H), 4.91 – 4.88 (m, 1H), 4.87 – 4.81 (m, 4H), 4.80 (d, *J* = 13.0 Hz, 1H), 4.77 – 4.69 (m, 3H), 4.60 (d, *J* = 12.6 Hz, 1H), 4.56 (d, *J* = 12.1 Hz, 1H), 4.52 (d, *J* = 13.0 Hz, 1H), 4.50 – 4.46 (m, 2H), 4.46 – 4.39 (m, 5H), 4.39 – 4.36 (m, 4H), 4.35 – 4.32 (m, 2H), 4.31 – 4.28 (m, 2H), 4.20 (dd, *J* = 10.8, 8.4 Hz, 1H), 4.16 – 4.03 (m, 9H), 4.01 (d, *J* = 12.4 Hz, 1H), 3.99 – 3.93 (m, 2H), 3.90 – 3.80 (m, 5H), 3.77 – 3.59 (m, 8H), 3.57 (dd, *J* = 11.4, 2.2 Hz, 1H), 3.51 (d, *J* = 10.5 Hz, 1H), 3.48 – 3.33 (m, 6H), 3.32 – 3.27 (m, 2H), 3.27 – 3.17 (m, 3H), 3.06 – 3.00 (m, 1H), 2.91 – 2.80 (m, 2H), 2.78 (dd, *J* = 9.6, 2.9 Hz, 1H), 2.55 (dd, *J* = 10.4, 6.9 Hz, 1H), 2.52 – 2.47 (m, 1H), 1.40 – 1.21 (m, 4H), 1.12 – 0.99 (m, 2H), 0.90 (s, 9H), 0.03 (s, 3H), −0.03 (s, 3H) ppm; ^{13}C NMR (151 MHz, $CDCl_3$) δ = 168.2, 167.9, 167.8, 167.6, 154.4, 143.8, 143.5, 141.3, 141.2, 138.92, 138.85, 138.8, 138.7, 138.6, 138.50, 138.49, 138.47, 138.43, 138.41, 138.3, 138.1, 138.0, 133.8, 133.74, 133.72, 133.69, 133.6, 133.5, 132.0, 131.9, 131.6, 129.0, 128.6, 128.5, 128.42, 128.40, 128.38, 128.33, 128.32, 128.29, 128.27, 128.26, 128.24, 128.18, 128.13, 128.07, 128.06, 127.94, 127.91, 127.84, 127.82, 127.78, 127.76, 127.7, 127.57, 127.56, 127.54, 127.51, 127.47, 127.46, 127.42, 127.40, 127.39, 127.21, 127.16, 126.92, 126.90, 126.5, 125.7, 125.4, 123.6, 123.4, 123.33, 123.29, 123.27, 123.24, 123.20, 123.16, 120.0, 119.9, 101.4, 98.6, 98.2, 98.1, 97.3, 95.9, 80.8, 80.5, 79.2, 78.2, 77.8, 77.6, 76.4, 75.7, 75.4, 75.3, 74.9, 74.7, 74.63, 74.60, 74.5, 74.4, 74.28, 74.26, 74.2, 73.9, 73.4, 73.0, 72.7, 72.5, 72.2, 72.1, 71.8, 71.3, 70.6, 70.5, 69.9, 69.3, 69.0, 68.9, 68.2, 67.8, 66.8, 56.6, 55.9, 51.2, 46.5, 28.8, 28.4, 26.1, 23.1, 18.1, −3.6, −4.6 ppm.

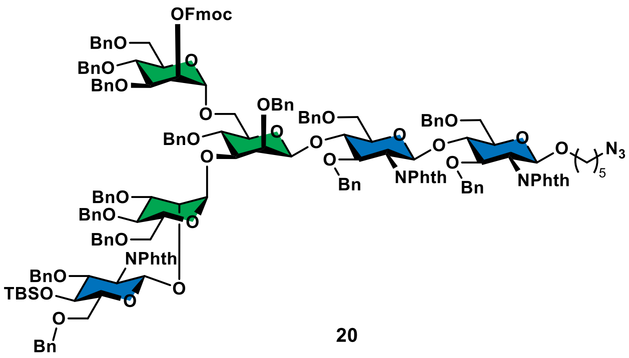

20

5-Azidopentyl 4-*O*-*tert*-butyldimethylsilyl-3,6-di-*O*-benzyl-2-deoxy-2-phthalimido-β-D-glucopyranosyl-(1→2)-3,4,6-tri-*O*-benzyl-α-D-mannopyranosyl-(1→3)-[4-*O*-levulinoyl-3,6-di-*O*-benzyl-2-deoxy-2-phthalimido-β-D-glucopyranosyl-(1→2)-3,4,6-tri-*O*-benzyl-2-*O*-fluorenylmethoxycarbonyl-α-D-mannopyranosyl-(1→6)]-2,4-di-*O*-benzyl-β-D-mannopyranosyl-(1→4)-3,6-di-*O*-benzyl-2-deoxy-2-phthalimido-β-D-glucopyranosyl-(1→4)-3,6-di-*O*-benzyl-2-deoxy-2-phthalimido-β-D-glucopyranoside (1): to a solution of hexasaccharide **20** (100 mg, 32.4 μmol, 1.0 equiv.) in CH_2Cl_2 (4.0 mL) at room temperature was added Et_3N (0.400 mL). The homogenous reaction solution was stirred at the same temperature for another 12 hrs before it was completely dried under vacuum. The resulting residue was briefly purified by flash chromatography (silica gel, EtOAc:hexanes = 1:10 → 1:1, *v*/*v*) to provide the corresponding secondary alcohol which was used directly for the pending glycosylation reaction. Following the General Procedure I,

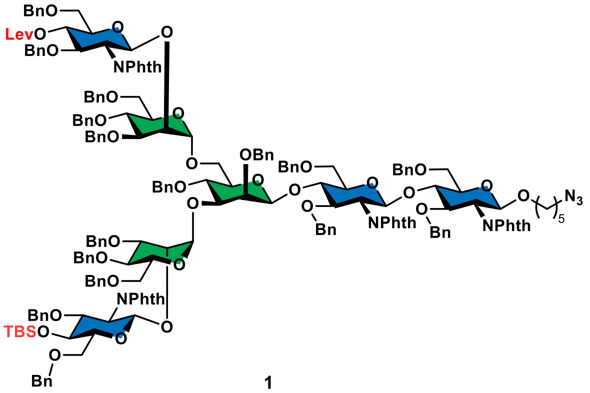

1

heptasaccharide **1** was synthesized from so-obtained secondary alcohol (86.0 mg, 30.0 μmol, 1.0 equiv.), Lev protected thioglycoside donor **7** (62.4 mg, 90.0 μmol, 3.0 equiv.), NIS (19.0 mg, 84.0 μmol, 2.8 equiv.), TMSOTf (2.2 mg, 1.8 μL, 10.0 μmol, 0.3equiv.) and 4Å MS (300 mg, flame dried) in CH_2Cl_2 (2.5 mL) from −40 → −20 °C. Flash chromatography (silica gel, EtOAc:hexanes = 1:4 → 2:3, *v*/*v*) afforded heptasaccharide **1** (60.0 mg, 54% for the two steps) as a glassy film. **1**: ^{1}H NMR (600 MHz, $CDCl_3$) δ = 7.86 – 6.61 (m, 96H), 5.17 (d, *J* = 8.1 Hz, 1H), 5.05 (apparent t, *J* = 9.5 Hz, 1H), 5.00 (d, *J* = 1.9 Hz, 1H), 4.93 – 4.87 (m, 2H), 4.86 – 4.71 (m, 8H), 4.63 (d, *J* = 13.2 Hz, 1H), 4.60 (d, *J* = 11.6 Hz, 1H), 4.53 (d, *J* = 12.0 Hz, 1H), 4.50 – 4.46 (m, 2H), 4.44 – 4.34 (m, 10H), 4.33 – 4.03 (m, 16H), 3.99 (d, *J* = 12.4 Hz, 1H), 3.95 – 3.83 (m, 5H), 3.73 (ABq, *J* = 12.2 Hz, $\Delta\nu_{AB}$ = 14.7 Hz, 2H), 3.70 – 3.59 (m, 6H), 3.52 (dd, *J* = 10.6, 2.5 Hz, 1H), 3.49 – 3.38 (m, 4H), 3.38 – 3.08 (m, 14H), 2.92 – 2.79 (m, 3H), 2.71 (d, *J* = 9.4 Hz, 1H), 2.68 – 2.63 (m, 1H), 2.63 – 2.52 (m, 2H), 2.48 (dd, *J* = 10.2, 7.3 Hz, 1H), 2.43 – 2.31 (m, 2H), 2.30 – 2.24 (m, 1H), 2.13 (s, 3H), 1.42 – 1.21 (m, 4H), 1.12 – 1.01 (m, 2H), 0.90 (s, 9H), 0.02 (s, 3H), −0.04 (s, 3H) ppm; ^{13}C NMR (151 MHz, $CDCl_3$) δ = 206.6, 171.5, 168.2, 167.6, 139.1, 139.0, 138.9, 138.8, 138.6, 138.52, 138.49, 138.4, 138.3, 138.04, 137.98, 133.9, 133.7, 133.5, 132.0, 131.8, 131.6, 129.0, 128.91, 128.89, 128.8, 128.5, 128.42, 128.39, 128.37, 128.32, 128.29, 128.26, 128.24, 128.16, 128.13, 128.09, 128.06, 128.03, 128.01, 127.9, 127.82, 127.77, 127.64, 127.62, 127.55, 127.52, 127.49, 127.47, 127.44, 127.42, 127.40, 127.37, 127.3, 127.2, 127.1, 127.0, 126.2, 123.6, 123.3, 101.9, 98.4, 98.2, 98.0, 97.2, 97.1, 95.6, 80.8, 80.1, 79.9, 78.2, 77.7, 77.0, 76.8, 76.71, 76.67, 76.1, 75.6, 75.24, 75.20, 74.9, 74.64, 74.57, 74.3, 73.9, 73.8, 73.5, 73.4, 73.0, 72.94, 72.90, 72.7, 72.5, 72.43, 72.39, 72.3, 72.0, 70.6, 70.4, 69.9, 69.8, 69.7, 69.02, 68.98, 68.3, 67.7, 66.9, 56.8, 55.89, 55.85, 55.2, 51.2, 37.9, 30.0, 28.8, 28.4, 28.0, 26.1, 23.1, 18.2, −3.6, −4.6 ppm.

Scheme S6. Assembly of Octasaccharide Common Precursors 2[a]

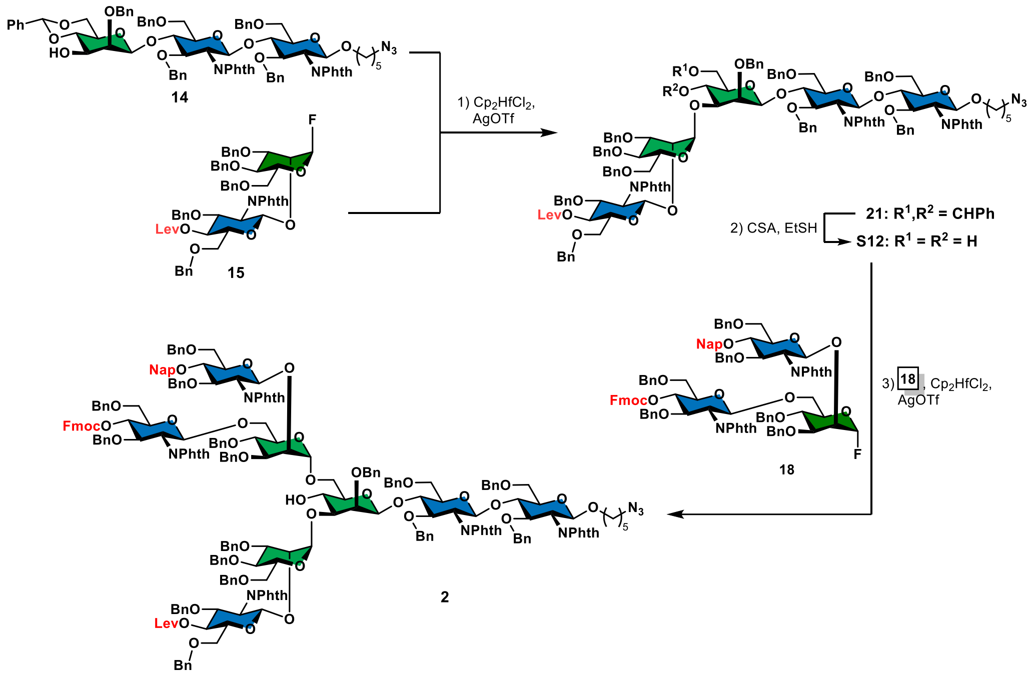

[a]Reagents and conditions: 1) **14** (1.0 equiv.), **15** (1.1 equiv.), Cp_2HfCl_2 (3.5 equiv.), AgOTf (5.0 equiv.), 4Å MS, PhMe, −60 → −20 °C, 1 hr, 53% **21** and 7% **S12**; 2) CSA (5.0 equiv.), EtSH (10.0 equiv.), CH_2Cl_2:MeOH (20:1, *v*/*v*), r.t., 12 hrs, 59% (26% recovered S.M.); 3) **S12** (1.0 equiv.), **18** (1.5 equiv.), Cp_2HfCl_2 (3.5 equiv.), AgOTf (5.0 equiv.), 4Å MS, PhMe, −20 °C, 1 hr, 72% combined yield (α:β *ca.* 1.5:1).

5-A zidopentyl 4-*O*-levulinoyl-3,6-di-*O*-benzyl-2-deoxy-2-phthalimido-β-D-glucopyranosyl-(1→2)-3,4,6-tri-*O*-benzyl-α-D-mannopyranosyl-(1→3)-2-*O*-benzyl-4,6-benzylidene-β-D-mannopyranosyl-(1→4)-3,6-di-*O*-benzyl-2-deoxy-2-phthalimido-β-D-glucopyranosyl-(1→4)-3,6-di-*O*-benzyl-2-deoxy-2-phthalimido-β-D-glucopyranoside (21): to a mixture of Cp_2HfCl_2 (242 mg, 0.637 mmol, 3.5 equiv.), AgOTf (234 mg, 0.910 mmol, 5.0 equiv.) and 4Å MS (0.8 g, flame dried) with vigorous stirring was added PhMe (8.0 mL) at room temperature. The suspension was stirred for another 3 hrs before it was cooled to −60 °C, at which point a solution of trisaccharide **14** (257 mg, 0.182 mmol, 1.0 equiv.) and mannosyl fluoride **15** (205 mg, 0.200 mmol, 1.1 equiv.) in PhMe (2.0 mL) was slowly added into the suspension. The reaction mixture was slowly warmed up to −20 °C over 1 hr and then quenched by saturated aqueous $NaHCO_3$ (10 mL) and diluted with EtOAc (10 mL). The resulting suspension was filtered through Celite® and the organic layer of the filtrate was separated. The aqueous layer was extracted with EtOAc (20 mL × 3) and the organic layers were combined, dried over Na_2SO_4, and concentrated under vacuum. The residue was purified by flash chromatography (silica gel, EtOAc:hexanes = 1:4 → 1:1 → 2:1) to afford pentasaccharide benzylidene **21** (235 mg, 53%) as a major product and pentasaccharide diol **S12** (30.0 mg, 7%) as minor product. **21**: ^{1}H NMR (500 MHz, $CDCl_3$) δ = 7.91 – 7.53 (m, 14H), 7.52 – 7.47 (m, 2H), 7.46 – 7.36 (m, 4H), 7.33 – 7.26 (m, 13H), 7.25 – 7.09 (m, 17H), 7.09 – 7.05 (m, 4H), 7.03 – 6.94 (m, 5H),

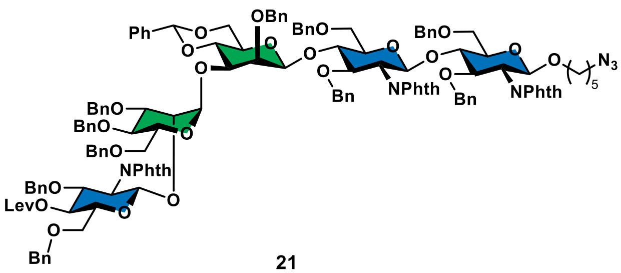

21

6.91 – 6.84 (m, 5H), 6.82 – 6.76 (m, 3H), 5.25 (d, *J* = 7.2 Hz, 1H), 5.24 (s, 1H), 5.01 (d, *J* = 2.0 Hz, 1H), 4.97 (dd, *J* = 10.1, 8.8 Hz, 1H), 4.93 (d, *J* = 7.9 Hz, 1H), 4.90 – 4.80 (m, 4H), 4.71 – 4.59 (m, 4H), 4.54 – 4.45 (m, 3H), 4.44 – 4.25 (m, 8H), 4.25 – 4.07 (m, 8H), 4.04 – 3.96 (m, 4H), 3.90 (d, *J* = 12.1 Hz, 1H), 3.80 – 3.73 (m, 2H), 3.67 (apparent dt, *J* = 9.9, 6.0 Hz, 1H), 3.59 (dd, *J* = 9.8, 7.0 Hz, 1H), 3.55 – 3.47 (m, 4H), 3.44 – 3.28 (m, 7H), 3.28 – 3.19 (m, 2H), 3.12 (br d, *J* = 9.9 Hz, 1H), 2.93 – 2.81 (m, 2H), 2.75 – 2.66 (m, 3H), 2.61 (dd, *J* = 10.4, 6.8 Hz, 1H), 2.56 – 2.42 (m, 2H), 2.39 – 2.32 (m, 1H), 2.17 (s, 3H), 1.50 – 1.18 (m, 4H), 1.17 – 1.00 (m, 2H) ppm; ^{13}C NMR (126 MHz, $CDCl_3$) δ = 206.3, 171.7, 139.1, 139.0, 138.8, 138.57, 138.55, 138.46, 138.394, 138.388, 138.1, 138.0, 137.9, 134.1, 133.9, 133.8, 133.71, 133.69, 133.67, 132.0, 129.8, 129.1, 128.7, 128.53, 128.50, 128.44, 128.35, 128.32, 128.28, 128.25, 128.21, 128.18, 128.17, 127.99, 127.96, 127.9, 127.8, 127.73, 127.70, 127.64, 127.62, 127.60, 127.58, 127.5, 127.4, 127.1, 127.00, 126.98, 123.8, 123.7, 123.6, 123.31, 123.28, 123.2, 102.1, 100.9, 98.2, 97.6, 97.4, 95.6, 79.0, 78.3, 78.2, 77.5, 77.4, 76.6, 76.3, 75.3, 75.0, 74.70, 74.65, 74.62, 74.60, 74.1, 73.7, 73.2, 73.0, 72.8, 72.64, 72.57, 72.2, 71.5, 70.6, 70.4, 69.8, 69.0, 68.7, 68.4, 67.7, 66.6, 56.7, 55.9, 55.4, 51.2, 37.9, 30.0, 28.8, 28.4, 28.0, 23.1 ppm. **S12**: ^{1}H NMR (600 MHz, $CDCl_3$) δ = 7.88 (br d, *J* = 7.4 Hz, 1H), 7.82 – 7.46 (m, 11H), 7.45 – 7.41 (m, 2H), 7.38 – 7.20 (m, 28H), 7.19 – 7.15 (m, 1H), 7.13 – 7.09 (m, 2H), 7.09 – 7.05 (m, 2H), 7.03 – 6.95 (m, 4H), 6.94 – 6.90 (m, 2H), 6.89 – 6.83 (m, 4H), 6.83 – 6.80 (m, 2H), 6.79 – 6.75 (m, 3H), 5.23 (d, *J* = 8.4 Hz, 1H), 5.21 (d, *J* = 2.3 Hz, 1H), 5.08 (d, *J* = 8.0 Hz, 1H), 5.03 (dd, *J* = 10.1, 8.3 Hz, 1H), 4.93 (d, *J* = 8.2 Hz, 1H), 4.89 – 4.81 (m, 3H), 4.76 (br d, *J* = 11.2 Hz, 1H), 4.69 – 4.58 (m, 3H), 4.56 – 4.44 (m, 8H), 4.40 (d, *J* = 12.1 Hz, 1H), 4.34 – 4.22 (m, 5H), 4.19 – 4.13 (m, 2H), 4.13 – 4.07 (m, 3H), 4.04 – 3.93 (m, 4H), 3.86 – 3.75 (m, 2H), 3.74 – 3.64 (m, 3H), 3.61 – 3.55 (m, 2H), 3.55 – 3.43 (m, 4H), 3.42 – 3.37 (m, 2H), 3.37 – 3.28 (m, 4H), 3.25 (ddd, *J* = 10.0, 7.2, 5.8 Hz, 1H), 3.15 (apparent dt, *J* = 10.0, 2.6 Hz, 1H), 3.04 – 2.96 (m, 2H), 2.93 – 2.80 (m, 2H), 2.64 (apparent t, *J* = 6.6 Hz, 2H), 2.51 – 2.39 (m, 2H), 2.15 (s, 3H), 1.41 – 1.22 (m, 4H), 1.12 – 1.01 (m, 2H) ppm; ^{13}C NMR (151 MHz, $CDCl_3$) δ = 206.3, 171.7, 168.5, 167.7, 138.82, 138.80, 138.7, 138.6, 138.42, 138.39, 138.2, 138.0, 137.85, 137.83, 134.1, 133.9, 133.7, 131.9, 131.8, 131.5, 128.7, 128.5, 128.38, 128.35, 128.3, 128.23, 128.16, 128.1, 128.01, 127.99, 127.96, 127.91, 127.87, 127.75, 127.68, 127.66, 127.6, 127.52, 127.46, 127.4, 127.2, 127.1, 126.9, 123.7, 123.3, 123.2, 123.1, 101.0, 98.2, 97.2, 96.4, 95.5, 80.6, 78.9, 78.7, 77.0, 76.9, 76.8, 76.1, 75.7, 74.8, 74.71, 74.67, 74.6, 74.3, 73.9, 73.6, 73.4, 73.1, 73.0, 72.8, 72.6, 71.4, 71.3, 70.2, 70.0, 69.0, 68.4, 67.6, 65.9, 62.9, 56.6, 55.9, 55.5, 51.2, 37.8, 29.9, 28.8, 28.4, 28.0, 23.1 ppm.

5-Azidopentyl 4-*O*-levulinoyl-3,6-di-*O*-benzyl-2-deoxy-2-phthalimido-β-D-glucopyranosyl-(1→2)-3,4,6-tri-*O*-benzyl-α-D-mannopyranosyl-(1→3)-2-*O*-benzyl-β-D-mannopyranosyl-(1→4)-3,6-di-*O*-benzyl-2-deoxy-2-phthalimido-β-D-glucopyranosyl-(1→4)-3,6-di-*O*-benzyl-2-deoxy-2-phthalimido-β-D-glucopyranoside (S12): to a solution of **21** (230 mg, 95.3 µmol, 1.0 equiv.) in CH_2Cl_2 (6.0 mL) at room temperature were sequentially added MeOH (0.3 mL), EtSH (59.2 mg, 68.6 µL, 0.952 mmol, 10.0 equiv.) and CSA (111 mg, 0.476 mmol, 5.0 equiv.). The reaction mixture was stirred at 40 °C for another 12 hrs before it was quenched by Et_3N (101 mg, 0.139 mL, 1.00 mmol, 10.5 equiv.) and concentrated under vacuum. The resulting residue was purified by flash chromatography (silica gel, EtOAc:hexanes = 1:2 → 1:1, *v*/*v*) to afford pentasaccharide diol **S12** (130 mg, 59%) and unreacted pentasaccharide benzylidene **21** (60.0 mg, 26%). The spectral data of **S12** was reported above.

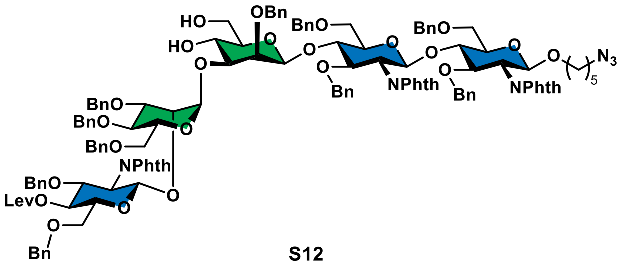

S12

5-Azidopentyl 4-*O*-levulinoyl-3,6-di-*O*-benzyl-2-deoxy-2-phthalimido-β-D-glucopyranosyl-(1→2)-3,4,6-tri-*O*-benzyl-α-D-mannopyranosyl-(1→3)-[4-*O*-(2-naphthylmethyl)-3,6-di-*O*-benzyl-2-deoxy-2-phthalimido-β-D-glucopyranosyl-(1→2)-[4-*O*-fluorenylmethoxycarbonyl-3,6-di-*O*-benzyl-2-deoxy-2-phthalimido-β-D-glucopyranosyl-(1→6)]-3,4-di-*O*-benzyl-α-D-mannopyranosyl-(1→6)]-2-*O*-benzyl-β-D-mannopyranosyl-(1→4)-3,6-di-*O*-benzyl-2-deoxy-2-phthalimido-β-D-glucopyranosyl-(1→4)-3,6-di-*O*-benzyl-2-deoxy-2-phthalimido-β-D-glucopyranoside (2): to a mixture of Cp_2HfCl_2 (708 mg, 1.87 mmol, 3.5 equiv.), AgOTf (685 mg, 2.66 mmol, 5.0 equiv.) and 4Å MS (2.0 g, flame dried) with vigorous stirring was added PhMe (20 mL) at room temperature. The suspension was stirred for another 3 hrs before it was cooled to −20 °C, at which point a solution of pentasaccharide diol **S12** (1.24 g, 0.533 mmol, 1.0 equiv.) and mannosyl fluoride **18** (1.16 g, 0.693 mmol, 1.3 equiv.) in PhMe (6.0 mL) was slowly added into the suspension. The reaction mixture was kept at the same temperature for another 1 hr and then quenched by saturated aqueous $NaHCO_3$ (10 mL) and diluted with EtOAc (10 mL). The resulting suspension was filtered through Celite® and the organic layer of the filtrate was separated. The aqueous layer was extracted with EtOAc (30 mL × 3) and the organic layers were combined, dried over Na_2SO_4, and concentrated under vacuum. The residue was purified by flash chromatography (silica gel, EtOAc:CH_2Cl_2 = 1:15 → 1:10) to afford octasaccharide **2** (915 mg, 43%) and its anomeric isomer **2-β** (610 mg, 29%) as white foams. **2**: ^{1}H NMR (600 MHz, $CDCl_3$) δ = 7.89 – 7.85 (m, 1H), 7.83 – 7.45 (m, 28H), 7.45 – 7.35 (m, 5H), 7.34 – 7.12 (m, 39H), 7.11 – 6.91 (m, 27H), 6.91 – 6.73 (m, 12H), 6.66 – 6.60 (m, 2H), 6.60 – 6.54 (m, 1H), 5.39 (d, *J* = 8.3 Hz, 1H), 5.20 – 5.09 (m, 2H), 5.04 – 4.96 (m, 1H),

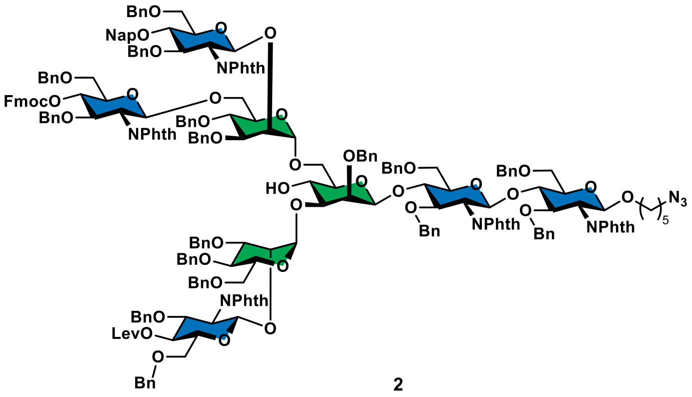

2

4.93 – 4.82 (m, 5H), 4.80 (d, J = 11.2 Hz, 1H), 4.77 – 4.69 (m, 3H), 4.64 (d, J = 12.3 Hz, 1H), 4.60 – 4.55 (m, 3H), 4.52 – 4.24 (m, 26H), 4.23 (d, J = 2.1 Hz, 1H), 4.18 – 4.05 (m, 9H), 4.04 (d, J = 12.3 Hz, 1H), 4.02 – 3.94 (m, 3H), 3.90 (dd, J = 3.2, 1.8 Hz, 1H), 3.87 – 3.51 (m, 17H), 3.51 – 3.41 (m, 3H), 3.37 – 3.29 (m, 4H), 3.29 – 3.21 (m, 3H), 3.21 – 3.10 (m, 3H), 3.08 (apparent dt, J = 9.9, 2.4 Hz, 1H), 2.93 – 2.81 (m, 3H), 2.80 – 2.74 (m, 2H), 2.60 (br d, J = 10.0 Hz, 1H), 2.55 – 2.46 (m, 3H), 2.41 – 2.28 (m, 3H), 2.07 (s, 3H), 1.42 – 1.23 (m, 4H), 1.12 – 1.00 (m, 2H) ppm; ^{13}C NMR (151 MHz, $CDCl_3$) δ = 206.3, 171.8, 168.1, 167.9, 167.7, 167.2, 154.5, 143.5, 143.2, 141.5, 141.4, 139.2, 138.94, 138.89, 138.8, 138.5, 138.37, 138.36, 138.3, 138.21, 138.18, 138.1, 138.05, 138.02, 137.7, 136.2, 133.9, 133.8, 133.7, 133.6, 133.4, 133.0, 131.9, 131.8, 131.6, 128.8, 128.55, 128.49, 128.44, 128.42, 128.39, 128.31, 128.29, 128.26, 128.2, 128.15, 128.11, 128.09, 128.05, 128.01, 127.98, 127.96, 127.93, 127.85, 127.84, 127.82, 127.71, 127.67, 127.65, 127.6, 127.54, 127.52, 127.49, 127.45, 127.40, 127.38, 127.31, 127.30, 127.26, 127.2, 127.0, 126.4, 126.2, 126.03, 125.99, 125.2, 125.1, 123.9, 123.5, 123.2, 120.2, 101.9, 98.8, 98.6, 98.2, 97.1, 96.6, 96.5, 96.3, 81.0, 79.6, 79.32, 79.27, 78.23, 78.17, 77.6, 77.5, 77.12, 77.06, 76.8, 76.1, 75.0, 74.73, 74.70, 74.68, 74.65, 74.6, 74.55, 74.51, 74.21, 74.19, 73.9, 73.75, 73.68, 73.54, 73.52, 73.3, 73.2, 73.0, 72.8, 72.7, 72.3, 72.0, 70.8, 70.7, 70.4, 70.2, 70.0, 69.9, 69.82, 69.77, 69.0, 68.8, 68.3, 67.6, 65.9, 65.6, 56.8, 55.8, 55.7, 55.6, 55.3, 51.2, 46.9, 37.8, 29.8, 28.8, 28.4, 28.0, 23.1 ppm.

Scheme S7. Assembly of LacNAc Repeat Module 30 by an Iterative Strategy[a]

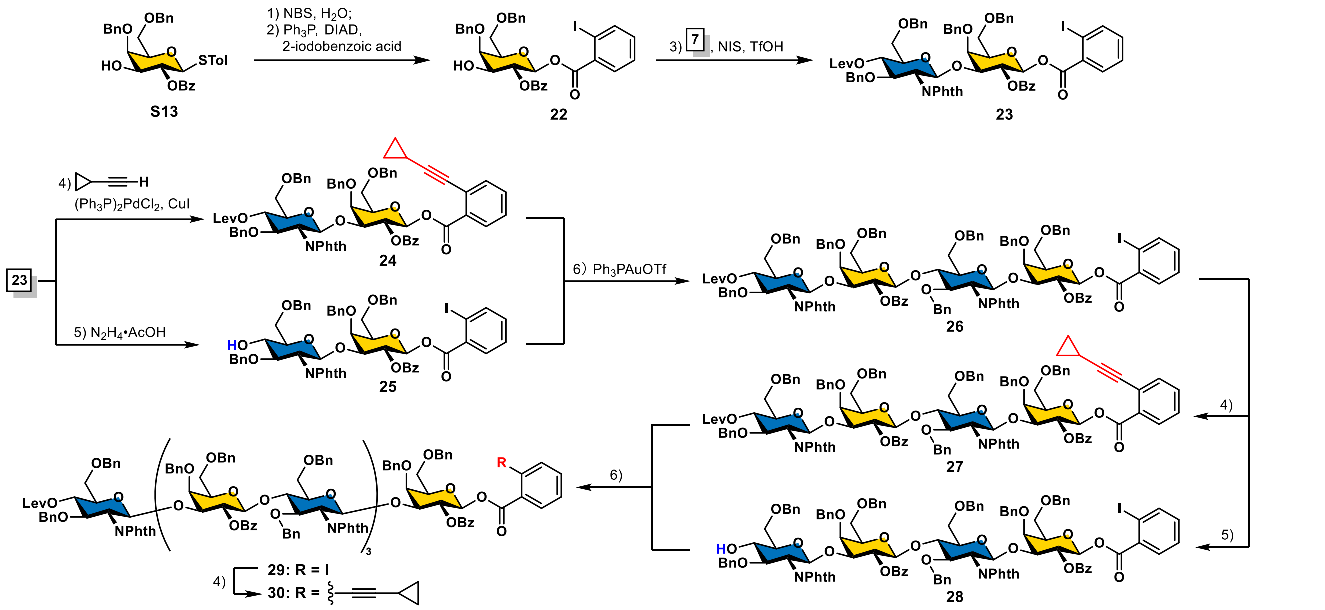

[a]Reagents and conditions: 1) NBS (3.0 equiv.), acetone:H_2O (10:1, *v*/*v*), r.t., 1 hr; 2) Ph_3P (2.0 equiv.), DIAD (2.0 equiv.), 2-iodobenzoic acid (2.0 equiv.), THF, −78 °C → r.t., 90% for the two steps; 3) **7** (1.1 equiv.), **22** (1.0 equiv.), NIS (1.2 equiv.), TMSOTf (0.1 equiv.), 4Å MS, CH_2Cl_2, −50 °C, 1 hr, 72%; 4) cyclopropyl acetylene (5.0 equiv.), $(Ph_3P)_2PdCl_2$ (0.1 equiv.), CuI (0.2 equiv.), DMF:Et_3N (1:4, *v*/*v*), r.t., 24 hrs, 90% for **24**, 82% for **27**, 94% for **30**; 5) N_2H_4·AcOH (2.0 equiv.), CH_2Cl_2:MeOH (20:1, *v*/*v*), r.t., 12 hrs, 88% for **25**, 90% for **28**; 6) $Ph_3PAuOTf$ (0.25 equiv.), 4Å MS, CH_2Cl_2, 0 °C, 82% for **26**, 82% for **29**.

2-*O*-benzoyl-4,6-di-*O*-benzyl-β-D-galactopyranosyl 2-iodobenzoate (22): to a solution of **S13**[16] (1.00 g, 1.75 mmol, 1.0 equiv.) in acetone:H_2O (22 mL, 10:1, *v*/*v*) at room temperature was added NBS (936 mg, 5.26 mmol, 3.0 equiv.) in one portion. The homogenous solution was stirred for another 30 min before it was quenched by saturated aqueous $NaHCO_3$ (20 mL). The organic phase was separated and the aqueous phase was extracted with CH_2Cl_2 (20 mL × 3). The organic phases were combined, dried over Na_2SO_4 and concentrated under vacuum. The resulting residue was briefly purified by flash chromatography (silica gel, EtOAc:hexanes = 1:1, *v*/*v*) to afford lactol intermediate (750 mg) which was used directly for the next step. To a solution of so-obtained lactol (750 mg, 1.61 mmol, 1.0 equiv.), Ph_3P (846 mg, 3.23 mmol, 2.0 equiv.) and 2-iodobenzoic acid (800 mg, 3.23 mmol, 2,0 equiv.) in THF (15 mL) at −78 °C was slowly added DIAD (653 mg, 634 μL, 2.0 equiv.). The reaction mixture was allowed to warm to room temperature and was stirred for another 1 hr. The reaction was then quenched by H_2O (20 mL) and was diluted with EtOAc (20 mL). The organic phase was separated, dried over Na_2SO_4 and concentrated under vacuum. The residue was purified by flash chromatography (silica gel, EtOAc:toluene = 1:20 → 1:7, *v*/*v*) to afford 2-iodobenzoate **22** (1.10 g, 90%, β:α *ca.* > 20:1) as a white foam. **22**: ^1H NMR (600 MHz, $CDCl_3$) δ = 8.04 – 7.99 (m, 2H), 7.94 (dd, *J* = 8.0, 1.2 Hz, 1H), 7.86 (dd, *J* = 7.9, 1.7 Hz, 1H), 7.56 – 7.51 (m, 1H), 7.44 – 7.29 (m, 13H), 7.11 (apparent td, *J* = 7.6, 1.7 Hz, 1H), 6.04 (d, *J* = 8.2 Hz, 1H), 5.54 (dd, *J* = 10.0, 8.2 Hz, 1H), 4.78 (ABq, *J* = 11.6 Hz, $\Delta\nu_{AB}$ = 14.7 Hz, 2H), 4.53 (ABq, *J* = 11.7 Hz,

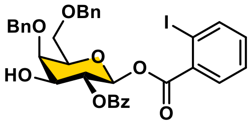

22

$\Delta\nu_{AB}$ = 27.0 Hz, 2H), 4.06 (dd, *J* = 3.6, 1.1 Hz, 1H), 3.97 (ddd, *J* = 7.8, 5.8, 1.1 Hz, 1H), 3.95 – 3.90 (m, 1H), 3.80 – 3.70 (m, 2H), 2.52 (br d, *J* = 9.4 Hz, 1H) ppm; ^{13}C NMR (151 MHz, $CDCl_3$) δ = 166.8, 164.2, 141.8, 138.0, 137.6, 133.6, 133.5, 132.5, 132.1, 130.1, 129.4, 128.8, 128.7, 128.6, 128.23 128.22, 128.15, 128.13, 128.11, 95.0, 93.1, 77.4, 77.2, 76.9, 76.3, 75.8, 74.6, 73.8, 73.3, 73.0, 67.6 ppm. HRMS (ESI-ToF) *m*/*z* calculated for $C_{34}H_{31}INaO_8^+$ $[M+Na]^+$: 717.0956, found: 717.0970.

4-*O*-levulinoyl-3,6-di-*O*-benzyl-2-deoxy-2-phthalimido-β-D-glucopyranosyl-(1→3)-2-*O*-benzoyl-4,6-di-*O*-benzyl-β-D-galactopyranosyl 2-iodobenzoate (23): following General Procedure I, disaccharide **23** was synthesized from 2-iodobenzoate **22** (1.23 g, 1.77 mmol, 1.0 equiv.), levulinate **7** (1.35 g, 1.95 mmol, 1.1 equiv.), NIS (478 mg, 2.13 mmol, 1.2 equiv.), TMSOTf (39.3 mg, 32.0 µL, 0.177 mmol, 0.1 equiv.) in CH_2Cl_2 (30 mL) in the presence of 4Å MS (3.0 g, flame dried) at −50 °C for 1 hr. Flash chromatography (silica gel, EtOAc:hexanes = 1:4 → 1:2, *v*/*v*) afforded disaccharide **23** (1.62 g, 72%) as a white foam. **23**: ^{1}H NMR (600 MHz, $CDCl_3$) δ = 7.84 (dd, *J* = 7.9, 1.1 Hz, 1H), 7.74 – 7.66 (m, 2H), 7.61 – 7.54 (m, 3H), 7.50 (apparent td, *J* = 7.4, 1.3 Hz, 1H), 7.44 – 7.40 (m, 2H), 7.38 – 7.24 (m, 16H), 7.19 (apparent td, *J* = 7.7, 1.2 Hz, 1H), 7.01 (apparent td, *J* = 7.7, 1.7 Hz, 1H), 6.95 – 6.90 (m, 2H), 6.89 – 6.74 (m, 4H), 5.83 (d, *J* = 8.2 Hz, 1H), 5.64 (dd, *J* = 10.1, 8.2 Hz, 1H), 5.33 (d, *J* = 8.3 Hz, 1H), 5.12 (dd, *J* = 10.1, 8.8 Hz, 1H), 5.06 (d, *J* = 11.5 Hz, 1H), 4.67 (d, *J* = 11.5 Hz, 1H), 4.57 (d, *J* = 12.2 Hz, 1H), 4.52 (s, 2H), 4.47 – 4.41 (m, 2H), 4.39 (d, *J* = 11.8 Hz, 1H), 4.29 (dd, *J* = 10.8, 8.4 Hz, 1H), 4.25 (d, *J* = 12.2 Hz, 1H), 4.23 (d, *J* = 2.9 Hz, 1H), 4.08 (dd, *J* = 10.1, 3.0 Hz, 1H), 3.86 (apparent t, *J* = 6.4 Hz, 1H), 3.82 (ddd, *J* = 9.7, 6.3, 3.0 Hz, 1H), 3.69 (dd, *J* = 10.6, 3.0 Hz, 1H), 3.64 – 3.57 (m, 3H), 2.70 – 2.59 (m, 2H), 2.47 (ddd, *J* = 17.3, 7.2, 5.9 Hz, 1H), 2.40 (apparent dt, *J* = 17.4, 6.4 Hz, 1H), 2.14 (s, 3H) ppm; ^{13}C NMR (151 MHz, $CDCl_3$) δ = 206.3, 171.7, 164.8, 163.8, 141.7, 138.6, 138.0, 137.9, 137.8, 133.6, 133.3, 133.0, 132.03, 131.99, 131.4, 130.9, 129.8, 129.0, 128.9, 128.53, 128.45, 128.32, 128.25, 128.0, 127.95, 127.87, 127.8, 127.7, 127.5, 127.3, 123.3, 122.9, 99.5, 95.0, 93.5, 80.1, 77.4, 77.2, 76.9, 76.7, 75.6, 75.0, 74.8, 74.1, 73.8, 73.53, 73.47, 72.6, 70.6, 69.8, 68.2, 55.8, 37.8, 29.9, 28.0 ppm. HRMS (ESI-ToF) *m*/*z* calculated for $C_{67}H_{62}INNaO_{16}^+$ $[M+Na]^+$: 1286.3006, found: 1286.2990.

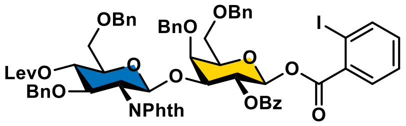

23

4-*O*-levulinoyl-3,6-di-*O*-benzyl-2-deoxy-2-phthalimido-β-D-glucopyranosyl-(1→3)-2-*O*-benzoyl-4,6-di-*O*-benzyl-β-D-galactopyranosyl 2-(cyclopropylethynyl)benzoate (24): following General Procedue II, *o*-alkynylbenzoate **24** was synthesized from 2-iodobenzoate **23** (872 mg, 0.690 mmol, 1.0 equiv.), cyclopropyl acetylene (228 mg, 0.292 mL, 3.45 mmol, 5.0 equiv.), $(Ph_3P)_2PdCl_2$ (48.4 mg, 69.0 µmol, 0.1 equiv.) and CuI (26.3 mg, 0.138 mmol, 0.2 equiv.) in Et_3N:DMF (15 mL, 4:1, *v*/*v*) at room temperature for 24 hrs. Flash chromatography (silica gel, EtOAc:hexanes = 1:4 → 1:2, *v*/*v*) afforded *o*-alkynylbenzoate **24** (745 mg, 90%) as a pale yellow foam. **24**: ^{1}H NMR (600 MHz, $CDCl_3$) δ = 7.76 – 7.66 (m, 2H), 7.60 – 7.52 (m, 3H), 7.48

(apparent tt, *J* = 7.4, 1.3 Hz, 1H), 7.41 (d, *J* = 7.1 Hz, 2H), 7.37 – 7.23 (m, 18H), 7.07 (apparent td, *J* = 7.7, 1.4 Hz, 1H), 6.93 – 6.88 (m, 2H), 6.87 – 6.83 (m, 2H), 6.83 – 6.80 (m, 1H), 6.79 – 6.72 (m, 1H), 5.81 (d, *J* = 8.2 Hz, 1H), 5.62 (dd, *J* = 10.1, 8.2 Hz, 1H), 5.31 (d, *J* = 8.3 Hz, 1H), 5.10 (dd, *J* = 10.1, 8.9 Hz, 1H), 5.05 (d, *J* = 11.4 Hz, 1H), 4.65 (d, *J* = 11.4 Hz, 1H), 4.55 (d, *J* = 12.2 Hz, 1H), 4.51 (s, 2H), 4.47 – 4.40 (m, 2H), 4.38 (d, *J* = 11.8 Hz, 1H), 4.27 (dd, *J* = 10.8, 8.3 Hz, 1H), 4.24 (d, *J* = 12.2 Hz, 1H), 4.20 (d, *J* = 3.0 Hz, 1H), 4.07 (dd, *J* = 10.1, 3.0 Hz, 1H), 3.85 (apparent t, *J* = 6.4 Hz, 1H), 3.80 (ddd, *J* = 9.7, 6.2, 3.0 Hz, 1H), 3.67 (dd, *J* = 10.6, 3.0 Hz, 1H), 3.63 – 3.56 (m, 3H), 2.70 – 2.58 (m, 2H), 2.45 (ddd, *J* = 17.4, 7.1, 6.0 Hz, 1H), 2.39 (apparent dt, *J* = 17.3, 6.4 Hz, 1H), 2.14 (s, 3H), 1.40 (tt, *J* = 7.7, 5.4 Hz, 1H), 0.83 – 0.72 (m, 4H) ppm; ^{13}C NMR (151 MHz, $CDCl_3$) δ = 206.4, 171.8, 168.1, 167.0, 164.7, 163.6, 138.6, 138.1, 137.9, 137.8, 134.2, 133.6, 133.4, 133.0, 132.2, 131.4, 131.1, 130.9, 129.8, 129.5, 129.1, 128.9, 128.6, 128.48, 128.45, 128.3, 128.2, 128.11, 128.06, 127.89, 127.86, 127.8, 127.7, 127.5, 127.4, 127.0, 125.6, 123.4, 122.9, 100.3, 99.5, 93.1, 80.1, 76.9, 76.7, 75.7, 75.1, 74.7, 74.5, 74.1, 73.8, 73.54, 73.47, 72.7, 70.7, 69.9, 68.2, 55.8, 37.8, 29.9, 28.0, 9.00, 8.98, 0.8 ppm. HRMS (ESI-ToF) *m*/*z* calculated for $C_{72}H_{67}NNaO_{16}^{+}$ [M+Na]$^{+}$: 1224.4352, found: 1224.4330.

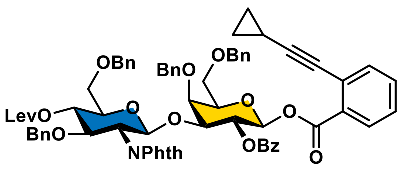

24

3,6-Di-*O*-benzyl-2-deoxy-2-phthalimido-β-D-glucopyranosyl-(1→3)-2-*O*-benzoyl-4,6-di-*O*-benzyl-β-D-galactopyranosyl 2-iodobenzoate (25): following General Procedure VII, secondary alcohol **25** was synthesized from levulinate **23** (802 mg, 0.634 mmol, 1.0 equiv.) and hydrazine acetate (1.27 mL, *ca.* 1.0 M in MeOH, 1.27 mmol, 2.0 equiv.) in CH_2Cl_2 (20 mL) at room temperature for 6 hrs. It was noted that prolonged reaction time might result in cleavage of glycosyl 2-iodobenzoate. Flash chromatography (silica gel, EtOAc:hexanes = 1:4 → 1:2, *v*/*v*) afforded secondary alcohol **25** (650 mg, 88%) as a white foam. **25**: ^{1}H NMR (600 MHz, $CDCl_3$) δ = 7.85 (dd, *J* = 7.9, 1.2 Hz, 1H), 7.73 (br d, *J* = 7.5 Hz, 1H), 7.68 (dd, *J* = 7.9, 1.7 Hz, 1H), 7.62 – 7.56 (m, 3H), 7.50 (apparent tt, *J* = 7.4, 1.3 Hz, 1H), 7.43 – 7.24 (m, 18H), 7.19 (apparent td, *J* = 7.7, 1.2 Hz, 1H), 7.01 (apparent td, *J* = 7.7, 1.7 Hz, 1H), 6.99 – 6.94 (m, 2H), 6.94 – 6.89 (m, 3H), 6.81 (br d, *J* = 7.4 Hz, 1H), 5.83 (d, *J* = 8.2 Hz, 1H), 5.64 (dd, *J* = 10.1, 8.2 Hz, 1H), 5.34 (d, *J* = 8.0 Hz, 1H), 5.06 (d, *J* = 11.4 Hz, 1H), 4.68 – 4.55 (m, 4H), 4.48 – 4.37 (m, 3H), 4.27 – 4.19 (m, 2H), 4.18 (d, *J* = 2.9 Hz, 1H), 4.06 (dd, *J* = 10.0, 2.9 Hz, 1H), 3.87 (apparent t, *J* = 6.4 Hz, 1H), 3.84 – 3.81 (m, 2H), 3.79 (dd, *J* = 9.6, 7.8 Hz, 1H), 3.70 (apparent dt, *J* = 9.7, 4.9 Hz, 1H), 3.64 – 3.58 (m, 2H), 2.80 (br s, 1H) ppm; ^{13}C NMR (151 MHz, $CDCl_3$) δ = 168.1, 167.3, 164.8, 163.8, 141.7, 138.6, 138.1, 137.9, 137.6, 133.6, 133.5, 133.4, 133.1, 132.1, 131.9, 131.4, 130.9, 129.8, 129.0, 128.8, 128.7, 128.5, 128.32, 128.29, 128.2, 128.12, 128.07, 128.04, 127.99, 127.9, 127.8, 127.7, 127.6, 127.5, 123.3, 122.9, 99.7, 95.1, 93.6, 80.1, 78.5, 75.6, 75.1, 74.7, 74.5, 74.1, 74.0, 73.7, 73.6, 70.7, 70.6, 68.1, 55.7 ppm. HRMS (ESI-ToF) *m*/*z* calculated for $C_{62}H_{56}INNaO_{14}^{+}$ [M+Na]$^{+}$: 1188.2638, found: 1188.2662.

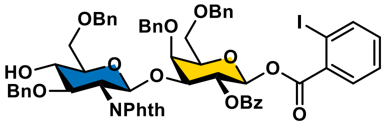

25

4-*O*-levulinoyl-3,6-di-*O*-benzyl-2-deoxy-2-phthalimido-β-D-glucopyranosyl-(1→3)-2-*O*-benzoyl-4,6-di-*O*-benzyl-β-D-galactopyranosyl-(1→4)-3,6-di-*O*-benzyl-2-deoxy-2-phthalimido-β-D-glucopyranosyl-(1→3)-2-*O*-benzoyl-4,6-di-*O*-benzyl-β-D-galactopyranosyl 2-iodobenzoate (26): following General Procedure III, tetrasaccharide **26** was synthesized from *o*-alkynylbenzoate **24** (496 mg, 0.412 mmol, 1.1 equiv.), secondary alcohol **25** (437 mg, 0.375 mmol, 1.0 equiv.) and $Ph_3PAuOTf$ (1.87 mL, *ca.* 0.05 M in CH_2Cl_2, 93.5 μmol, 0.25 equiv.) in CH_2Cl_2 (10 mL) at 0 °C in the presence of 4Å MS (1.5 g, flame dried). Flash chromatography (silica gel, EtOAc:hexanes = 1:4 → 1:2, *v*/*v*) afforded tetrasaccharide **26** (711 mg, 82%) as a white foam. **26**:

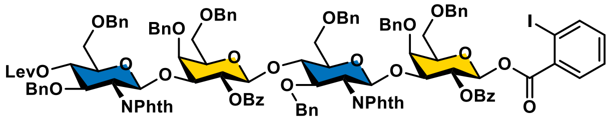

26

^{1}H NMR (600 MHz, $CDCl_3$) δ = 7.83 (dd, *J* = 8.0, 1.2 Hz, 1H), 7.73 – 7.67 (m, 2H), 7.65 (dd, *J* = 8.0, 1.7 Hz, 1H), 7.62 – 7.56 (m, 1H), 7.56 – 7.52 (m, 3H), 7.50 – 7.46 (m, 2H), 7.44 (apparent tt, *J* = 7.4, 1.3 Hz, 1H), 7.42 – 7.13 (m, 38H), 6.99 (apparent td, *J* = 7.6, 1.7 Hz, 1H), 6.93 – 6.90 (m, 2H), 6.89 – 6.85 (m, 2H), 6.85 – 6.81 (m, 1H), 6.81 – 6.77 (m, 3H), 6.76 – 6.68 (m, 4H), 5.73 (d, *J* = 8.3 Hz, 1H), 5.56 (dd, *J* = 10.1, 8.2 Hz, 1H), 5.32 – 5.28 (m, 1H), 5.22 (d, *J* = 8.4 Hz, 1H), 5.10 (d, *J* = 8.2 Hz, 1H), 5.09 – 5.04 (m, 2H), 5.00 (d, *J* = 11.8 Hz, 1H), 4.77 (d, *J* = 11.9 Hz, 1H), 4.58 – 4.49 (m, 6H), 4.45 (dd, *J* = 10.7, 8.9 Hz, 1H), 4.41 – 4.38 (m, 2H), 4.32 (d, *J* = 11.7 Hz, 1H), 4.30 – 4.22 (m, 3H), 4.18 – 4.03 (m, 7H), 3.91 – 3.80 (m, 3H), 3.76 (apparent t, *J* = 6.4 Hz, 1H), 3.71 (dd, *J* = 10.1, 3.1 Hz, 1H), 3.68 (dd, *J* = 10.5, 2.9 Hz, 1H), 3.60 (dd, *J* = 10.6, 6.7 Hz, 1H), 3.57 – 3.52 (m, 2H), 3.52 – 3.49 (m, 1H), 3.43 (dd, *J* = 10.7, 3.7 Hz, 1H), 3.38 – 3.33 (m, 2H), 3.27 (dd, *J* = 10.8, 1.8 Hz, 1H), 3.14 (ddd, *J* = 10.0, 3.7, 1.9 Hz, 1H), 2.71 – 2.60 (m, 2H), 2.49 (ddd, *J* = 17.4, 7.6, 5.8 Hz, 1H), 2.41 (apparent dt, *J* = 17.3, 6.4 Hz, 1H), 2.15 (s, 3H) ppm; ^{13}C NMR (151 MHz, $CDCl_3$) δ = 206.3, 171.8, 167.8, 167.2, 164.7, 164.3, 163.8, 141.7, 139.1, 138.85, 138.75, 138.4, 138.1, 138.04, 138.02, 137.9, 133.5, 133.4, 133.2, 133.0, 132.9, 132.1, 132.0, 131.5, 131.0, 129.83, 129.78, 129.5, 129.0, 128.8, 128.7, 128.55, 128.52, 128.50, 128.46, 128.4, 128.3, 128.22, 128.20, 128.15, 128.12, 128.06, 127.98, 127.95, 127.9, 127.83, 127.79, 127.7, 127.64, 127.60, 127.57, 127.5, 127.40, 127.35, 126.7, 123.4, 123.2, 122.8, 100.6, 99.7, 99.5, 95.0, 93.6, 80.3, 80.2, 77.2, 76.8, 76.4, 76.3, 75.8, 75.2, 74.9, 74.7, 74.4, 74.1, 73.6, 73.5, 72.7, 72.0, 70.4, 70.0, 68.6, 68.2, 67.6, 56.0, 55.8, 37.8, 29.9, 28.0 ppm.

4-*O*-levulinoyl-3,6-di-*O*-benzyl-2-deoxy-2-phthalimido-β-D-glucopyranosyl-(1→3)-2-*O*-benzoyl-4,6-di-*O*-benzyl-β-D-galactopyranosyl-(1→4)-3,6-di-*O*-benzyl-2-deoxy-2-phthalimido-β-D-glucopyranosyl-(1→3)-2-*O*-benzoyl-4,6-di-*O*-benzyl-β-D-galactopyranosyl 2-(cyclopropylethynyl)benzoate (27): following General Procedure II, *o*-alkynylbenzoate **27** was synthesized from 2-iodobenzoate **26** (1.56 g, 0.713 mmol, 1.0 equiv.), cyclopropyl acetylene (236 mg, 0.300 mL, 3.56 mmol, 5.0 equiv.), $(Ph_3P)_2PdCl_2$ (50.1 mg, 71.3 μmol,

0.1 equiv.) and CuI (27.2 mg, 0.143 mmol, 0.2 equiv.) in Et_3N:DMF (20 mL, 4:1, *v*/*v*) at room temperature. Flash chromatography (silica gel, EtOAc:hexanes = 1:4 → 1:1, *v*/*v*) afforded *o*-alkynylbenzoate **27** (1.24 g, 82%) as a pale yellow foam. **27**: ^{1}H NMR (600 MHz, $CDCl_3$) δ = 7.78 – 7.65 (m, 3H), 7.64 – 7.49 (m, 6H), 7.48 – 7.14 (m, 40H), 7.05 (apparent t, *J* = 7.7 Hz, 1H), 6.95 – 6.90 (m, 2H), 6.89 – 6.82 (m, 3H), 6.81 – 6.77 (m, 3H), 6.76 – 6.65 (m, 4H), 5.74 (d, *J* = 8.2 Hz, 1H), 5.56 (dd, *J* = 10.1, 8.3 Hz, 1H), 5.30 (dd, *J* = 10.2, 7.9 Hz, 1H), 5.22 (d, *J* = 8.4 Hz, 1H), 5.12 – 5.04 (m, 3H), 5.00 (d, *J* = 11.7 Hz, 1H), 4.78 (d, *J* = 11.9 Hz, 1H), 4.59 – 4.48 (m, 6H), 4.45 (dd, *J* = 10.7, 8.9 Hz, 1H), 4.43 – 4.36 (m, 2H), 4.35 – 4.20 (m, 4H), 4.20 – 4.02 (m, 7H), 3.92 – 3.81 (m, 3H), 3.77 (apparent t, *J* = 6.4 Hz, 1H), 3.72 (dd, *J* = 10.2, 3.1 Hz, 1H), 3.69 (dd, *J* = 10.8, 2.9 Hz, 1H), 3.60 (dd, *J* = 10.6, 6.6 Hz, 1H), 3.57 – 3.53 (m, 2H), 3.51 (apparent t, *J* = 6.4 Hz, 1H), 3.44 (dd, *J* = 10.9, 3.6 Hz, 1H), 3.39 – 3.34 (m, 2H), 3.27 (apparent d, *J* = 10.5 Hz, 1H), 3.15 (br d, *J* = 10.3 Hz, 1H), 2.72 – 2.59 (m, 2H), 2.50 (apparent dt, *J* = 17.2, 6.6 Hz, 1H), 2.41 (apparent dt, *J* = 17.3, 6.3 Hz, 1H), 2.15 (s, 3H), 1.43 – 1.35 (m, 1H), 0.79 – 0.72 (m, 4H) ppm; ^{13}C NMR (151 MHz, $CDCl_3$) δ = 206.3, 171.8, 167.8, 167.2, 164.7, 164.3, 163.6, 139.1, 138.84, 138.81, 138.3, 138.1, 138.0, 137.9, 134.2, 133.4, 133.2, 132.9, 132.8, 132.1, 131.4, 131.03, 130.95, 129.8, 129.7, 129.52, 129.50, 129.0, 128.8, 128.7, 128.54, 128.50, 128.44, 128.39, 128.3, 128.24, 128.21, 128.12, 128.05, 128.0, 127.94, 127.87, 127.82, 127.76, 127.7, 127.63, 127.59, 127.53, 127.49, 127.4, 127.3, 126.9, 126.7, 125.5, 123.1, 122.8, 100.6, 100.2, 99.7, 99.5, 93.1, 80.3, 80.2, 76.8, 76.4, 76.3, 75.9, 75.2, 74.9, 74.7, 74.5, 74.41, 74.40, 74.1, 73.9, 73.6, 73.51, 73.49, 72.7, 72.0, 70.5, 70.0, 68.6, 68.2, 67.6, 56.0, 55.8, 37.8, 29.9, 28.0, 8.94, 8.91, 0.7 ppm.

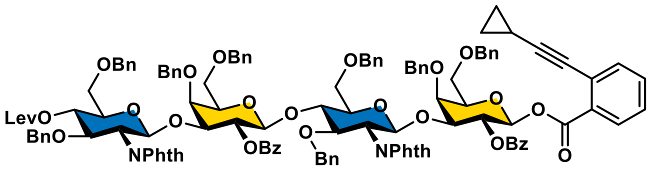

27

3,6-Di-*O*-benzyl-2-deoxy-2-phthalimido-β-D-glucopyranosyl-(1→3)-2-*O*-benzoyl-4,6-di-*O*-benzyl-β-D-galactopyranosyl-(1→4)-3,6-di-*O*-benzyl-2-deoxy-2-phthalimido-β-D-glucopyranosyl-(1→3)-2-*O*-benzoyl-4,6-di-*O*-benzyl-β-D-galactopyranosyl 2-iodobenzoate (28): following General Procedure VII, secondary alcohol **28** was synthesized from levulinate **26** (552 mg, 0.253 mmol, 1.0 equiv.) and hydrazine acetate (1.01 mL, *ca.* 0.5 M in MeOH, 0.505 mmol, 2.0 equiv.) in CH_2Cl_2 (15 mL) at room temperature for 6 hrs. It was noted that prolonged reaction time might result in cleavage of glycosyl 2-iodobenzoate. Flash chromatography (silica gel, EtOAc:hexanes = 1:4 → 1:2, *v*/*v*) afforded secondary alcohol **28** (475 mg, 90%) as a glassy film. **28**: ^{1}H NMR (600 MHz, $CDCl_3$) δ = 7.83 (d, *J* = 7.8 Hz, 1H), 7.78 – 7.51 (m, 8H), 7.50 – 7.43 (m, 3H), 7.42 – 7.14 (m, 38H), 7.02 – 6.91 (m, 6H), 6.84 – 6.75 (m, 3H), 6.75 – 6.67 (m, 3H), 5.74 (d, *J* = 8.2 Hz, 1H), 5.56 (dd, *J* = 10.1, 8.2 Hz, 1H), 5.32 (dd, *J* = 10.2, 7.9 Hz, 1H), 5.24 (d, *J* = 8.3 Hz, 1H), 5.11 (d, *J* = 8.2 Hz, 1H), 5.05 (d, *J* = 11.4 Hz, 1H), 5.01 (d, *J* = 11.7 Hz, 1H), 4.77 (d, *J* = 11.9 Hz, 1H), 4.64 – 4.49 (m, 6H), 4.46 – 4.37 (m, 3H), 4.34 – 4.23 (m, 3H), 4.20 – 4.05 (m, 7H), 4.02 (d, *J* = 3.1 Hz, 1H), 3.93 – 3.68 (m, 8H), 3.59 – 3.48 (m, 3H), 3.45 (dd,

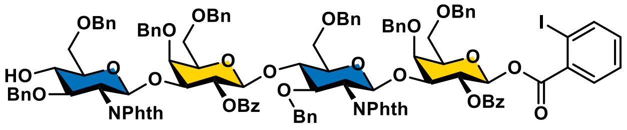

28

J = 10.8, 3.7 Hz, 1H), 3.40 – 3.31 (m, 2H), 3.29 (d, J = 10.5 Hz, 1H), 3.19 – 3.13 (m, 1H), 2.75 (d, J = 2.5 Hz, 1H) ppm; ^{13}C NMR (151 MHz, $CDCl_3$) δ = 168.2, 167.9, 167.2, 164.7, 164.4, 163.8, 141.7, 139.1, 138.8, 138.7, 138.4, 138.2, 138.0, 137.7, 133.6, 133.41, 133.36, 133.3, 133.0, 132.1, 132.0, 131.5, 131.0, 129.84, 129.78, 129.6, 129.0, 128.8, 128.69, 128.66, 128.5, 128.42, 128.41, 128.31, 128.28, 128.26, 128.21, 128.19, 128.1, 127.99, 127.97, 127.84, 127.79, 127.7, 127.63, 127.61, 127.57, 127.5, 127.4, 126.7, 123.4, 123.2, 122.9, 122.8, 100.7, 99.71, 99.69, 95.0, 93.6, 80.3, 80.2, 78.7, 77.2, 76.42, 76.39, 75.8, 75.2, 74.9, 74.70, 74.69, 74.5, 74.4, 74.02, 73.97, 73.9, 73.8, 73.53, 73.50, 73.48, 72.1, 70.9, 70.4, 68.6, 68.2, 67.7, 56.0, 55.7 ppm.

4-*O*-levulinoyl-3,6-di-*O*-benzyl-2-deoxy-2-phthalimido-β-D-glucopyranosyl-(1→3)-2-*O*-benzoyl-4,6-di-*O*-benzyl-β-D-galactopyranosyl-(1→4)-3,6-di-*O*-benzyl-2-deoxy-2-phthalimido-β-D-glucopyranosyl-(1→3)-2-*O*-benzoyl-4,6-di-*O*-benzyl-β-D-galactopyranosyl-(1→4)-3,6-di-*O*-benzyl-2-deoxy-2-phthalimido-β-D-glucopyranosyl-(1→3)-2-*O*-benzoyl-4,6-di-*O*-benzyl-β-D-galactopyranosyl-(1→4)-3,6-di-*O*-benzyl-2-deoxy-2-phthalimido-β-D-glucopyranosyl-(1→3)-2-*O*-benzoyl-4,6-di-*O*-benzyl-β-D-galactopyranosyl 2-iodobenzoate (29): following General Procedure III, octasaccharide **29** was synthesized from *o*-alkynylbenzoate **27** (542 mg, 0.256 mmol, 1.1 equiv.), secondary alcohol **28** (485 mg, 0.232 mmol, 1.0 equiv.) and $Ph_3PAuOTf$ (1.16 mL, *ca.* 0.05 M in CH_2Cl_2, 58.1 μmol, 0.25 equiv.) in CH_2Cl_2 (10 mL) at 0 °C in the presence of 4Å MS (1.5 g, flame dried). Flash chromatography (silica gel, EtOAc:hexanes = 1:4 → 1:2, *v*/*v*) afforded octasaccharide **29** (765 mg, 82%) as a white foam. **29**: ^{1}H NMR (600 MHz, $CDCl_3$) δ = 7.85 (d, J = 7.9 Hz, 1H), 7.80 – 7.06 (m, 93H), 7.01 (apparent t, J = 7.8 Hz, 1H), 6.98 – 6.63 (m, 25H), 5.75 (d, J = 8.2 Hz, 1H), 5.58 (apparent t, J = 9.1 Hz, 1H), 5.40 – 5.21 (m, 4H), 5.17 – 4.95 (m, 8H), 4.88 – 4.70 (m, 3H), 4.65 – 4.01 (m, 38H), 3.99 – 3.68 (m, 11H), 3.67 – 3.49 (m, 6H), 3.48 – 3.35 (m, 7H), 3.35 – 3.25 (m, 6H), 3.23 – 3.10 (m, 3H), 2.77 – 2.60 (m, 2H), 2.52 (apparent dt, J = 17.4, 6.6 Hz, 1H), 2.44 (apparent dt, J = 17.5, 6.5 Hz, 1H), 2.18 (s, 3H) ppm; ^{13}C NMR (151 MHz, $CDCl_3$) δ = 206.3, 171.8, 167.9, 167.84, 167.79, 167.1, 164.7, 164.35, 164.30, 164.2, 163.8, 141.7, 139.2, 139.1, 138.9, 138.83, 138.80, 138.7, 138.4, 138.34, 138.31, 138.09, 138.06, 138.01, 137.99, 137.9, 133.5, 133.3, 133.2, 133.0, 132.92, 132.85, 132.1, 132.0, 131.4, 130.9, 129.8, 129.7, 129.5, 129.43, 129.36, 128.9, 128.8, 128.6, 128.52, 128.49, 128.43, 128.39, 128.35, 128.30, 128.26, 128.24, 128.21, 128.18, 128.11, 128.09, 128.05, 127.95, 127.92, 127.86, 127.81, 127.76, 127.72, 127.70, 127.66, 127.63, 127.60, 127.56, 127.52, 127.49, 127.4, 127.3, 127.24, 127.19, 126.7, 126.6, 123.2, 122.8, 122.7, 100.8, 100.7, 99.7, 99.64, 99.62, 99.5, 95.0, 93.5, 80.40, 80.36, 80.2, 77.5, 77.4, 77.3, 77.2, 76.8, 76.6, 76.54, 76.47, 76.4, 76.2, 75.8, 75.2, 75.0, 74.95, 74.86, 74.7, 74.6, 74.5, 74.4, 74.3, 74.1, 73.9, 73.8, 73.71, 73.68, 73.50, 73.48, 73.41, 73.40, 73.37, 73.3, 72.7, 72.1, 72.0, 71.8, 70.4, 70.0, 68.64, 68.58, 68.55, 68.1, 67.82, 67.79, 67.7, 55.94, 55.91, 55.8, 37.8, 29.9, 28.0 ppm.

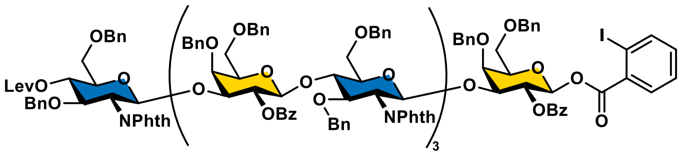

29

4-*O*-levulinoyl-3,6-di-*O*-benzyl-2-deoxy-2-phthalimido-β-D-glucopyranosyl-(1→3)-2-*O*-benzoyl-4,6-di-*O*-benzyl-β-D-galactopyranosyl-(1→4)-3,6-di-*O*-benzyl-2-deoxy-2-phthalimido-β-D-glucopyranosyl-(1→3)-2-*O*-benzoyl-4,6-di-*O*-benzyl-β-D-galactopyranosyl-(1→4)-3,6-di-*O*-benzyl-2-deoxy-2-phthalimido-β-D-glucopyranosyl-(1→3)-2-*O*-benzoyl-4,6-di-*O*-benzyl-β-D-galactopyranosyl-(1→4)-3,6-di-*O*-benzyl-2-deoxy-2-phthalimido-β-D-glucopyranosyl-(1→3)-2-*O*-benzoyl-4,6-di-*O*-benzyl-β-D-galactopyranosyl 2-(cyclopropylethynyl)benzoate (30): following General Procedure II, *o*-alkynylbenzoate **30** was synthesized from 2-iodobenzoate **29** (720 mg, 0.179 mmol, 1.0 equiv.), cyclopropyl acetylene (59.2 mg, 75.8 µL, 0.896 mmol, 5.0 equiv.), $(Ph_3P)_2PdCl_2$ (12.6 mg, 17.9 µmol, 0.1 equiv.) and CuI (6.8 mg, 35.8 mmol, 0.2 equiv.) in Et_3N:DMF (20 mL, 4:1, *v*/*v*) at room temperature. Flash chromatography (silica gel, EtOAc:hexanes = 1:4 → 1:1, *v*/*v*) afforded *o*-alkynylbenzoate **30** (665 mg, 94%) as a pale yellow foam. **30**: ^{1}H NMR (600 MHz, $CDCl_3$) δ = 7.76 – 7.63 (m, 5H), 7.62 – 7.07 (m, 90H), 7.04 (apparent t, *J* = 7.8 Hz, 1H), 6.93 – 6.89 (m, 2H), 6.89 – 6.82 (m, 3H), 6.82 – 6.60 (m, 19H), 5.72 (d, *J* = 7.8 Hz, 1H), 5.54 (apparent t, *J* = 9.2 Hz, 1H), 5.34 – 5.19 (m, 4H), 5.10 – 4.93 (m, 8H), 4.82 – 4.70 (m, 3H), 4.59 – 4.54 (m, 2H), 4.53 – 4.48 (m, 3H), 4.47 – 3.98 (m, 33H), 3.94 – 3.88 (m, 2H), 3.87 – 3.77 (m, 5H), 3.77 – 3.70 (m, 2H), 3.67 (br d, *J* = 10.3 Hz, 1H), 3.62 – 3.46 (m, 6H), 3.44 – 3.31 (m, 7H), 3.31 – 3.19 (m, 7H), 3.19 – 3.09 (m, 3H), 2.73 – 2.58 (m, 2H), 2.53 – 2.45 (m, 1H), 2.40 (apparent dt, *J* = 17.4, 6.4 Hz, 1H), 2.15 (s, 3H), 1.37 (apparent hept, *J* = 6.3 Hz, 1H), 0.77 – 0.71 (m, 4H) ppm; ^{13}C NMR (151 MHz, $CDCl_3$) δ = 206.3, 171.8, 167.89, 167.86, 167.20, 167.16, 164.7, 164.4, 164.33, 164.25, 163.6, 139.2, 139.1, 138.89, 138.87, 138.84, 138.81, 138.40, 138.36, 138.3, 138.14, 138.12, 138.08, 138.05, 138.03, 137.9, 134.2, 133.4, 133.2, 132.94, 132.86, 132.1, 131.5, 131.0, 130.9, 129.82, 129.76, 129.7, 129.6, 129.53, 129.46, 129.4, 129.0, 128.8, 128.6, 128.55, 128.53, 128.51, 128.44, 128.41, 128.37, 128.32, 128.28, 128.2, 128.14, 128.11, 128.08, 128.07, 128.0, 127.94, 127.91, 127.88, 127.84, 127.80, 127.78, 127.75, 127.73, 127.68, 127.65, 127.61, 127.58, 127.56, 127.54, 127.51, 127.42, 127.36, 127.3, 127.2, 126.9, 126.7, 126.6, 125.6, 123.2, 122.9, 122.7, 100.8, 100.68, 100.67, 100.2, 99.7, 99.5, 93.1, 80.42, 80.38, 80.24, 80.23, 77.5, 77.4, 77.3, 76.8, 76.59, 76.56, 76.50, 76.48, 76.4, 76.3, 75.9, 75.2, 74.99, 74.97, 74.9, 74.69, 74.67, 74.66, 74.51, 74.48, 74.44, 74.42, 74.36, 74.1, 73.91, 73.87, 73.72, 73.69, 73.6, 73.54, 73.53, 73.48, 73.44, 73.42, 73.39, 73.3, 72.7, 72.1, 72.0, 71.9, 70.5, 70.0, 68.7, 68.60, 68.57, 68.2, 67.84, 67.81, 67.7, 56.0, 55.9, 55.8, 37.8, 29.9, 28.0, 8.94, 8.92, 0.7 ppm.

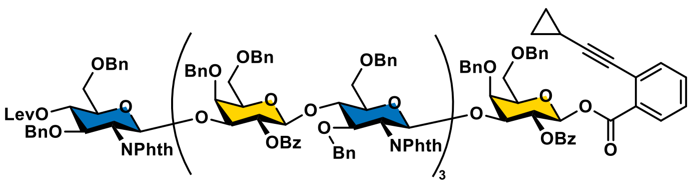

30

Scheme S8. Assembly of Trisaccharide Modules Using Programmable One-pot Strategy[a]

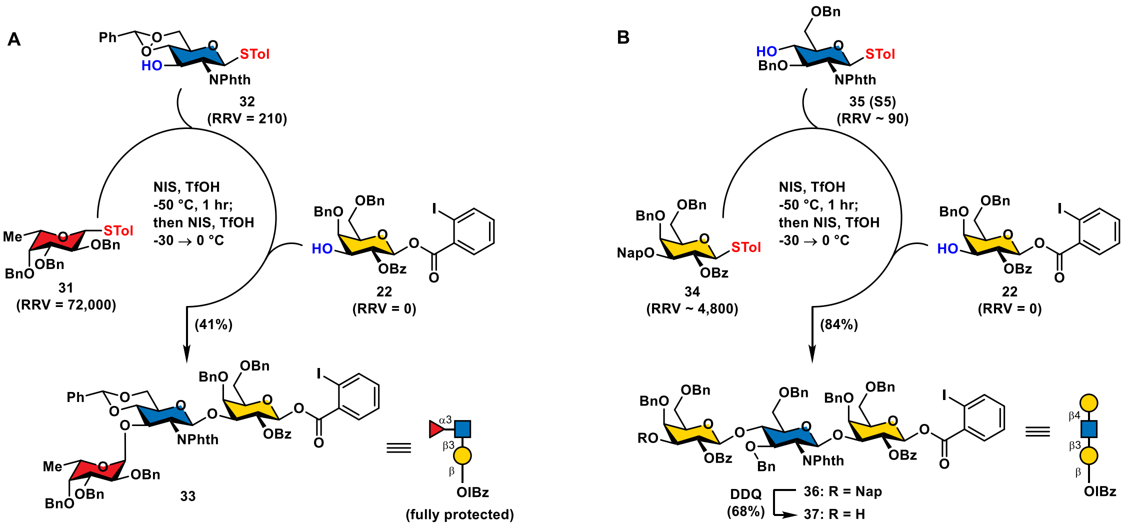

[a]Reagents and conditions: A) **31** (1.1 equiv.), **32** (1.0 equiv.), NIS (1.08 equiv.), TfOH (0.15 equiv.), 4Å MS, CH_2Cl_2, −50 → −40 °C, 1 hr; then **22** (0.9 equiv.), NIS (1.1 equiv.), TfOH (0.15 equiv.), −40 → 0 °C, 30 min, 41% overall. B) (i) **34** (1.1 equiv.), **35** (1.0 equiv.), NIS (1.08 equiv.), TfOH (0.15 equiv.), 4Å MS, CH_2Cl_2, −50 → −40 °C, 1 hr; then **22** (0.9 equiv.), NIS (1.1 equiv.), TfOH (0.15 equiv.), −40 → 0 °C, 30 min, 84% overall; (ii) DDQ (2.0 equiv.), CH_2Cl_2:H_2O (10:1, *v*/*v*), 0 °C, 4 hrs, 68%.

2,3,4-Tri-*O*-benzyl-α-L-fucopyranosyl-(1→3)-4,6-benzylidene-2-deoxy-2-phthalimido-β-D-glucopyranosyl-(1→3)-2-benzoyl-4,6-di-*O*-benzyl-β-D-galactopyranosyl 2-iodobenzoate (33): following General Procedure VI, trisaccharide **33** was synthesized from **31**[17] (128 mg, 0.237 mmol, 1.1 equiv.), **32**[18] (108 mg, 0.215 mmol, 1.0 equiv.) and **22** (135 mg, 0.194 mmol, 0.9 equiv.), with NIS (52.3 mg, 0.232 mmol, 1.08 equiv.) and TfOH (2.9 µL, 4.9 mg, 32.3 µmol, 0.15 equiv.) as promoters for the 1st stage and NIS (53.2 mg, 0.237 mmol,

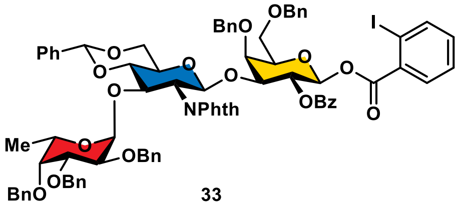

1.1 equiv.) and TfOH (2.9 µL, 4.9 mg, 32.3 µmol, 0.15 equiv.) for the 2nd stage. Flash chromatography (silica gel, EtOAc:toluene = 1:15, *v*/*v*; then EtOAc:CH_2Cl_2 = 1:40 → 1:20, *v*/*v*) afforded desired trisaccharide **33** (132 mg, 41% overall) as a white foam. **33**: ^{1}H NMR (600 MHz, $CDCl_3$) δ = 7.86 (d, *J* = 7.9 Hz, 1H), 7.73 – 7.67 (m, 2H), 7.63 – 7.58 (m, 2H), 7.54 – 7.44 (m, 6H), 7.43 – 7.16 (m, 25H), 7.16 – 7.10 (m, 3H), 7.02 (apparent td, *J* = 7.6, 1.6 Hz, 1H), 6.97 – 6.92 (m, 2H), 6.81 (d, *J* = 7.4 Hz, 1H), 5.87 (d, *J* = 8.2 Hz, 1H), 5.71 (dd, *J* = 10.0, 8.2 Hz, 1H), 5.57 (s, 1H), 5.54 (d, *J* = 8.4 Hz, 1H), 5.09 (d, *J* = 11.4 Hz, 1H), 4.77 (d, *J* = 11.6 Hz, 1H), 4.72 (d, *J* = 3.3 Hz, 1H), 4.67 (d, *J* = 11.4 Hz, 1H), 4.65 – 4.60 (m, 1H), 4.53 – 4.45 (m, 3H), 4.45 – 4.39 (m, 2H), 4.36 (d, *J* = 11.5 Hz, 1H), 4.30 (d, *J* = 11.5 Hz, 1H), 4.21 – 4.10 (m, 3H), 4.03 (q, *J* = 6.7 Hz, 1H), 3.92 (apparent t, *J* = 6.4 Hz, 1H), 3.88 – 3.83 (m, 1H), 3.74 – 3.68 (m, 3H), 3.68 – 3.63 (m, 3H), 3.61 (dd, *J* = 10.2, 3.3 Hz, 1H), 3.43 (d, *J* = 2.7 Hz, 1H), 0.85 (d, *J* = 6.5 Hz, 3H) ppm; ^{13}C NMR (151 MHz, $CDCl_3$) δ = 168.2, 167.5, 164.9, 163.8, 141.7, 138.9, 138.62, 138.61, 138.3, 137.9, 137.1, 133.5, 133.4, 133.1, 132.1, 132.0, 131.8, 131.5, 129.8, 129.01, 128.98, 128.95, 128.6, 128.5, 128.42, 128.36, 128.3, 128.23, 128.17, 128.01, 127.97, 127.8, 127.6,

127.53, 127.47, 127.4, 126.2, 123.0, 122.9, 101.3, 100.2, 99.4, 95.1, 93.6, 82.1, 80.3, 79.6, 78.1, 75.7, 75.3, 75.2, 75.0, 74.8, 74.6, 73.6, 73.2, 72.6, 70.7, 68.8, 68.0, 67.3, 66.3, 56.0, 16.5 ppm.

2-*O*-benzoyl-3-*O*-(2-naphthylmethyl)-4,6-di-*O*-benzyl-β-D-galactopyranosyl-(1→4)-3,6-di-*O*-benzyl-2-deoxy-2-phthalimido-β-D-glucopyranosyl-(1→3)-2-*O*-benzoyl-4,6-di-*O*-benzyl-β-D-galactopyranosyl 2-iodobenzoate (36): following General Procedure VI, trisaccharide **36** was synthesized from **34**[19] (195 mg, 0.275 mmol, 1.1 equiv.), **35**[11] (also see **S5** in Scheme S2, 149 mg, 0.250 mmol, 1.0 equiv.) and **22** (153 mg, 0.225 mmol, 0.9 equiv.), with NIS (60.7 mg, 0.270 mmol, 1.08 equiv.) and TfOH (3.3 µL, 5.6 mg, 37.5 µmol, 0.15 equiv.) as promoters for the 1st stage and NIS (56.2 mg, 0.250 mmol, 1.0 equiv.) and TfOH (3.3 µL, 5.6 mg, 37.5 µmol, 0.15 equiv.) for the 2nd stage. Flash chromatography (silica gel, EtOAc:toluene = 1:10, *v*/*v*) afforded desired trisaccharide **36** (330 mg, 84%) as a colorless amorphous foam.

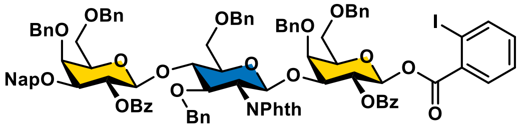

36

36: ^{1}H NMR (600 MHz, $CDCl_3$) δ = 7.96 – 7.91 (m, 2H), 7.83 (dd, *J* = 8.0, 1.2 Hz, 1H), 7.78 (d, *J* = 8.0 Hz, 1H), 7.71 (br d, *J* = 7.1 Hz, 1H), 7.68 (dd, *J* = 7.9, 1.7 Hz, 1H), 7.65 – 7.61 (m, 2H), 7.60 – 7.56 (m, 2H), 7.56 – 7.50 (m, 3H), 7.50 – 7.42 (m, 5H), 7.41 – 7.38 (m, 2H), 7.37 – 7.21 (m, 21H), 7.20 – 7.14 (m, 6H), 7.09 (apparent td, *J* = 6.7, 1.9 Hz, 1H), 6.98 (apparent td, *J* = 7.7, 1.7 Hz, 1H), 6.93 – 6.88 (m, 2H), 6.86 – 6.82 (m, 1H), 6.81 – 6.76 (m, 3H), 5.79 (d, *J* = 8.2 Hz, 1H), 5.68 (dd, *J* = 10.1, 7.9 Hz, 1H), 5.62 (dd, *J* = 10.0, 8.2 Hz, 1H), 5.24 (d, *J* = 8.0 Hz, 1H), 5.11 (d, *J* = 11.3 Hz, 1H), 5.03 (d, *J* = 11.6 Hz, 1H), 4.91 (d, *J* = 12.0 Hz, 1H), 4.81 (d, *J* = 12.7 Hz, 1H), 4.65 (d, *J* = 7.9 Hz, 1H), 4.63 – 4.57 (m, 3H), 4.54 (d, *J* = 12.0 Hz, 1H), 4.42 (d, *J* = 11.8 Hz, 1H), 4.38 – 4.32 (m, 3H), 4.30 – 4.20 (m, 4H), 4.17 (d, *J* = 2.9 Hz, 1H), 4.08 (d, *J* = 2.9 Hz, 1H), 4.04 (dd, *J* = 9.9, 8.1 Hz, 1H), 3.95 (dd, *J* = 10.1, 3.0 Hz, 1H), 3.82 (apparent t, *J* = 6.4 Hz, 1H), 3.66 (dd, *J* = 10.8, 3.8 Hz, 1H), 3.62 – 3.55 (m, 3H), 3.54 – 3.46 (m, 3H), 3.46 – 3.39 (m, 2H) ppm; ^{13}C NMR (151 MHz, $CDCl_3$) δ = 167.8, 167.2, 165.1, 164.7, 163.8, 141.6, 138.9, 138.8, 138.7, 138.1, 137.98, 137.97, 135.3, 133.3, 133.20, 133.16, 133.02, 132.98, 132.03, 131.95, 131.4, 131.0, 130.0, 129.9, 129.7, 128.9, 128.8, 128.52, 128.51, 128.45, 128.41, 128.3, 128.25, 128.21, 128.0, 127.94, 127.92, 127.84, 127.82, 127.7, 127.6, 127.54, 127.51, 127.47, 126.7, 126.5, 126.2, 126.0, 125.8, 123.1, 122.9, 100.8, 99.7, 95.0, 93.6, 80.2, 79.7, 77.6, 76.7, 75.8, 75.2, 74.7, 74.6, 74.5, 73.6, 73.49, 73.47, 73.4, 72.51, 72.47, 71.4, 70.4, 68.1, 56.0 ppm.

2-*O*-benzoyl-4,6-di-*O*-benzyl-β-D-galactopyranosyl-(1→4)-3,6-di-*O*-benzyl-2-deoxy-2-phthalimido-β-D-glucopyranosyl-(1→3)-2-*O*-benzoyl-4,6-di-*O*-benzyl-β-D-galactopyranosyl 2-iodobenzoate (37): following General Procedure IX, secondary alcohol **37** was synthesized from Nap ether **36** (320 mg, 0.183 mmol, 1.0 equiv.) and DDQ (82.9 mg, 0.365 mmol, 2.0 equiv.) in CH_2Cl_2:H_2O (11 mL, 10:1, *v*/*v*) at 0 °C. Flash chromatography (silica gel, EtOAc:hexanes = 1:10 → 2:3, *v*/*v*) afforded secondary alcohol **37** (200 mg, 68%) as

a glassy colorless film. **37**: ^{1}H NMR (600 MHz, $CDCl_3$) δ = 8.03 – 8.00 (m, 2H), 7.84 (dd, *J* = 7.8, 1.2 Hz, 1H), 7.71 (br s, 1H), 7.67 (dd, *J* = 7.9, 1.7 Hz, 1H), 7.64 – 7.60 (m, 1H), 7.59 – 7.51 (m, 3H), 7.50 – 7.44 (m, 3H), 7.42 – 7.38 (m, 2H), 7.38 – 7.22 (m, 26H), 7.19 (apparent td, *J* = 7.7, 1.2 Hz, 1H), 7.00 (apparent td, *J* = 7.7, 1.8 Hz, 1H), 6.94 – 6.90 (m, 2H), 6.87 – 6.72 (m, 4H), 5.79 (d, *J* = 8.2 Hz, 1H), 5.61 (dd, *J* = 10.0, 8.2 Hz, 1H), 5.28 – 5.22 (m, 2H), 5.11 (d, *J* = 11.4 Hz, 1H), 4.87 (d, *J* = 12.1 Hz, 1H), 4.71 – 4.63 (m, 4H), 4.60 (d, *J* = 11.4 Hz, 1H), 4.43 (d, *J* = 11.7 Hz, 1H), 4.41 – 4.31 (m, 5H), 4.29 – 4.21 (m, 2H), 4.19 (d, *J* = 2.9 Hz, 1H), 4.08 (dd, *J* = 9.9, 7.8 Hz, 1H), 3.97 (dd, *J* = 10.1, 2.9 Hz, 1H), 3.91 (d, *J* = 3.6 Hz, 1H), 3.83 (apparent t, *J* = 6.3 Hz, 1H), 3.78 (dd, *J* = 10.9, 3.7 Hz, 1H), 3.68 – 3.61 (m, 2H), 3.61 – 3.55 (m, 3H), 3.51 – 3.44 (m, 3H), 2.26 (br s, 1H) ppm; ^{13}C NMR (151 MHz, $CDCl_3$) δ = 167.8, 167.3, 166.3, 164.8, 163.8, 141.7, 138.8, 138.7, 138.2, 138.1, 138.0, 137.8, 133.42, 133.35, 133.0, 132.1, 132.0, 131.5, 131.0, 129.9, 129.8, 129.0, 128.8, 128.7, 128.63, 128.59, 128.56, 128.5, 128.3, 128.2, 128.1, 128.00, 127.97, 127.96, 127.9, 127.8, 127.74, 127.73, 127.6, 127.5, 126.9, 123.2, 122.9, 100.5, 99.7, 95.0, 93.6, 80.2, 77.8, 76.7, 76.6, 75.9, 75.4, 75.3, 74.7, 74.64, 74.58, 73.7, 73.57, 73.55, 73.3, 72.9, 70.5, 68.2, 68.1, 67.8, 56.1 ppm.

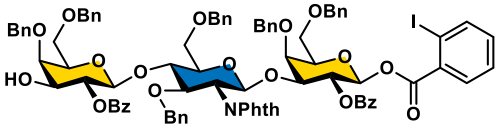

37

Scheme S9. Assembly of Tetrasaccharide and Hexasaccharide Modules[a]

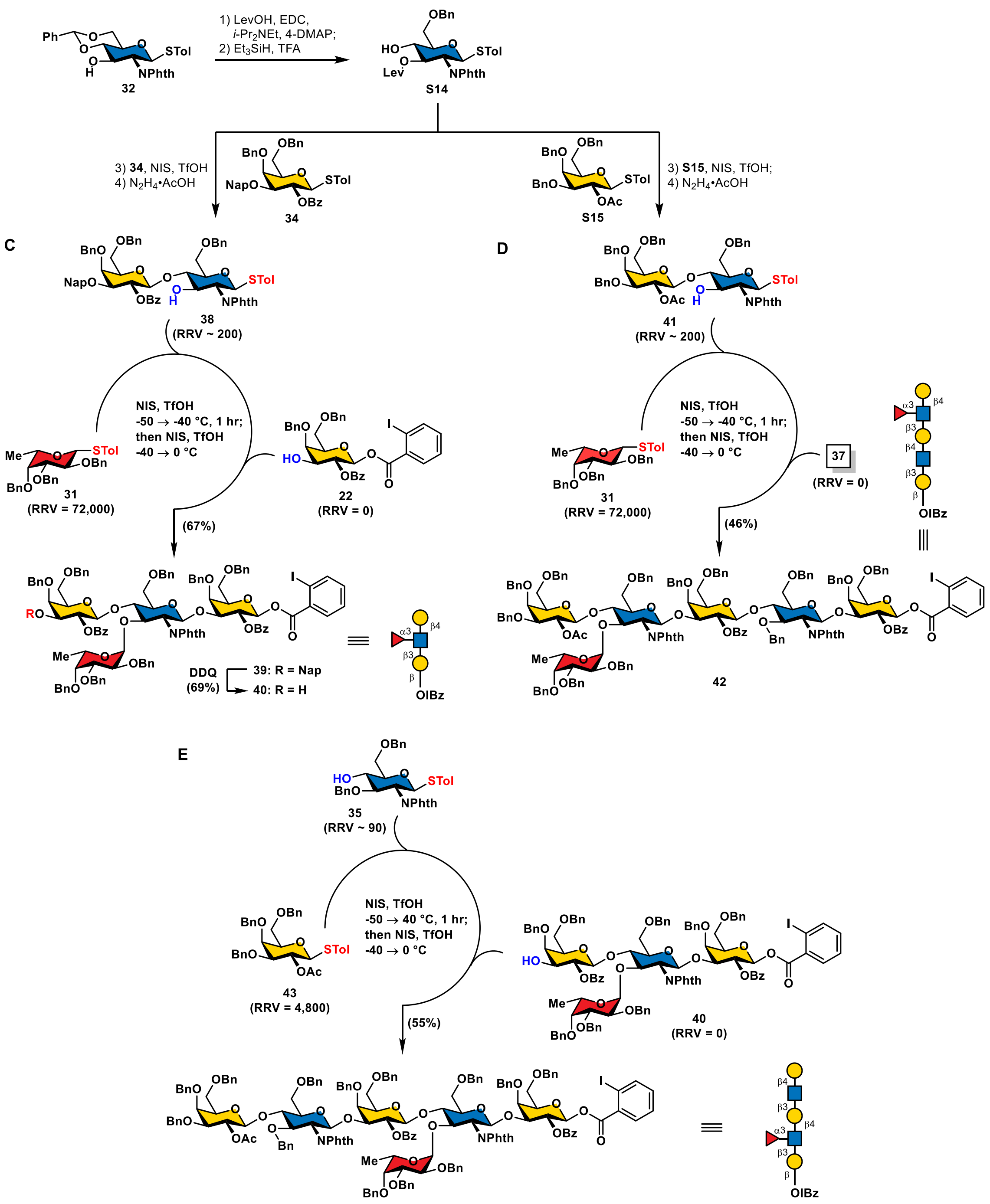

[a]Reagents and conditions: 1) LevOH (2.0 equiv.), EDC (2.0 equiv.), *i*-Pr_2NEt (2.0 equiv.), 4-DMAP (0.2 equiv.), CH_2Cl_2, r.t., 12 hrs; 2) Et_3SiH (10.0 equiv.), TFA (10.0 equiv.), 4Å MS, CH_2Cl_2, 0 °C, 3 hrs, 82% for the two steps; 3) **34** (1.1 equiv.) or **S15** (1.1 equiv.), **S14** (1.0 equiv.), NIS (1.1 equiv.), TfOH (0.15 equiv.), 4Å MS, CH_2Cl_2, −40 → −20 °C, 1 hr; 4) $N_2H_4{\cdot}AcOH$ (1.5 equiv.), CH_2Cl_2:MeOH (20:1, *v*/*v*), r.t., 3 hrs, 81% for **38**, 73% for **41** over the two steps; C) (i) **31** (1.2 equiv.), **38** (1.0 equiv.), NIS (1.18 equiv.), TfOH (0.15 equiv.), 4Å MS, CH_2Cl_2, −50 → −40 °C, 1 hr; then **22** (1.0 equiv.), NIS (1.2 equiv.), TfOH (0.15 equiv.), −40 → 0 °C, 30 min, 67% overall; (ii) DDQ (1.2 equiv.), CH_2Cl_2:H_2O (10:1, *v*/*v*), 0 °C, 6 hrs, 69%. D) **31** (1.1 equiv.), **41** (1.0 equiv.), NIS (1.1 equiv.), TfOH (0.15 equiv.), 4Å MS, CH_2Cl_2, −50 → −40 °C, 1 hr; then **37** (0.9 equiv.), NIS (1.1 equiv.), TfOH (0.15 equiv.), −40 → 0 °C, 30 min, 46% overall. E) **43** (1.1 equiv.), **35** (1.0 equiv.), NIS (1.1 equiv.), TfOH (0.15 equiv.), 4Å MS, CH_2Cl_2, −50 → −40 °C, 1 hr; then **40** (0.9 equiv.), NIS (1.1 equiv.), TfOH (0.15 equiv.), −40 → 0 °C, 30 min, 55% overall.

***p*-Tolyl 3-*O*-levulinoyl-6-*O*-benzyl-2-deoxy-2-phthalimido-1-thio-β-D-glucopyranoside (S14)**: to a solution of secondary alcohol **32**[18] (2.14 g, 4.00 mmol, 1.0 equiv.), levulinic acid (0.815 mL, 929 mg, 8.00 mmol, 2.0 equiv.), EDC (1.53 g, 8.00 mmol, 2.0 equiv.) and *i*-Pr_2NEt (1.39 mL, 1.03 g, 8.00 mmol, 2.0 equiv.) in CH_2Cl_2 (40 mL) was added 4-DMAP (97.7 mg, 0.800 mmol, 0.2 equiv.) at room temperature. The reaction mixture was stirred at

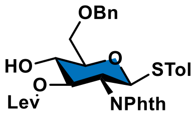

S14

the same temperature for another 12 hrs before all the volatiles were removed under vacuum. The resulting residue was briefly purified by flash chromatography (silica gel, EtOAc:hexanes = 1:3 → 2:3, *v*/*v*) to afford levulinate which was dried thoroughly under vacuum and was directly used for the next step. To a vigorously stirred suspension of so-obtained levulinate (601 mg, 1.00 mmol, 1.0 equiv.), Et_3SiH (1.60 mL, 1.16 g, 10.0 mmol, 10.0 equiv.) and 4Å MS (1.5 g, flame dried) in CH_2Cl_2 (15 mL) at 0 °C was slowly added TFA (0.765 mL, 1.14 g, 10.0 mmol, 10.0 equiv.). The reaction suspension was allowed to stir at the same temperature for another 3 hrs before it was poured into a mixture of cooled saturated aqueous $NaHCO_3$ (100 mL) and CH_2Cl_2 (50 mL). The milky mixture was filtered through Celite® and the organic phase of the filtrate was separated. The aqueous phase was extracted with CH_2Cl_2 (20 mL × 3) and the organic phases were combined, dried over Na_2SO_4 and concentrated under vacuum. The resulting residue was purified by flash chromatography (silica gel, EtOAc:hexanes = 1:2 → 1:1, *v*/*v*) to afford secondary alcohol **S14** (510 mg, 84%) as a white foam. **S14**: ^1H NMR (600 MHz, $CDCl_3$) δ = 7.87 (d, *J* = 6.8 Hz, 1H), 7.85 – 7.82 (m, 1H), 7.76 – 7.69 (m, 2H), 7.39 – 7.33 (m, 4H), 7.33 – 7.28 (m, 3H), 7.00 (d, *J* = 7.9 Hz, 2H), 5.71 (dd, *J* = 10.4, 8.2 Hz, 1H), 5.67 (d, *J* = 10.5 Hz, 1H), 4.66 – 4.55 (ABq, *J* = 11.9 Hz, $\Delta\nu_{AB}$ = 14.3 Hz, 2H), 4.30 (apparent t, *J* = 10.4 Hz, 1H), 3.88 (dd, *J* = 10.6, 3.2 Hz, 1H), 3.83 (dd, *J* = 10.7, 4.6 Hz, 1H), 3.81 – 3.74 (m, 2H), 3.25 (br s, 1H), 2.59 (apparent td, *J* = 6.3, 2.1 Hz, 2H), 2.46 – 2.38 (m, 1H), 2.37 – 2.30 (m, 1H), 2.28 (s, 3H), 1.98 (s, 3H) ppm; ^{13}C NMR (151 MHz, $CDCl_3$) δ = 207.0, 172.9, 168.0, 167.5, 138.4, 138.1, 134.3, 134.1, 133.5, 131.8, 131.6, 129.8, 128.5, 128.0, 127.8, 123.7, 83.5, 78.8, 74.9, 73.8, 70.9, 70.1, 53.6, 38.2, 29.6, 28.1, 21.3 ppm. HRMS (ESI-ToF) *m*/*z* calculated for $C_{33}H_{33}NNaO_8S^+$ $[M+Na]^+$: 626.1819, found: 626.1829.

***p*-Tolyl 2-*O*-benzoyl-3-*O*-(2-naphthylmethyl)-4,6-di-*O*-benzyl-β-D-galactopyranosyl-(1→4)-6-*O*-benzyl-2-deoxy-2-phthalimido-1-thio-β-D-glucopyranoside (38)**: following General Procedure I, **S14** (780 mg, 1.29 mmol, 1.0 equiv.) was glycosylated with **34**[19] (964 mg, 1.36 mmol, 1.05 equiv.) under the influence of NIS (302 mg, 1.34 mmol, 1.04 equiv.) and TfOH (17.2 μL, 29.1 mg, 0.194 mmol, 0.15 equiv.) at −40 °C for 30 min in the presence of 4Å MS (1.5 g, flame dried) in CH_2Cl_2 (20 mL). Flash chromatography (silica gel,

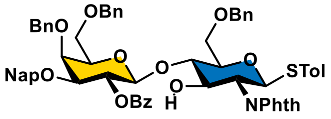

38

EtOAc:toluene = 1:5, *v*/*v*) afforded levulinoyl disaccharide intermediate (1.45 g, 94%, β:α *ca.* 8.3:1) as a white foam. ^1H NMR (600 MHz, $CDCl_3$, β anomer) δ = 7.89 – 7.86 (m, 2H), 7.86 – 7.84 (m, 1H), 7.83 – 7.80 (m, 1H), 7.74 (d, *J* = 7.9 Hz, 1H), 7.72 – 7.68 (m, 2H), 7.60 – 7.50 (m, 4H), 7.46 – 7.43 (m, 1H), 7.43 – 7.39 (m, 1H), 7.39 – 7.34 (m, 4H), 7.34 – 7.26 (m, 8H), 7.25 – 7.18 (m, 7H), 7.16 – 7.12 (m, 1H), 6.95 (d, *J* = 7.9 Hz, 2H), 5.69 (dd, *J* = 10.4, 9.0 Hz, 1H), 5.52 (d, *J* = 10.5 Hz, 1H), 5.48 (dd, *J* = 10.1, 7.9 Hz, 1H), 4.98 (d, *J* = 11.5 Hz, 1H),

4.76 (d, J = 12.6 Hz, 1H), 4.61 – 4.44 (m, 6H), 4.27 (d, J = 12.1 Hz, 1H), 4.23 (apparent t, J = 10.4 Hz, 1H), 4.04 (d, J = 2.2 Hz, 1H), 3.94 (apparent t, J = 9.5 Hz, 1H), 3.64 (apparent t, J = 8.5 Hz, 1H), 3.61 – 3.54 (m, 3H), 3.54 – 3.46 (m, 3H), 2.41 (apparent dt, J = 17.6, 7.1 Hz, 1H), 2.33 (apparent dt, J = 16.7, 7.2 Hz, 1H), 2.27 (apparent dt, J = 5.7, 4.8 Hz, 1H), 2.24 (s, 3H), 2.19 (ddd, J = 17.6, 7.0, 5.7 Hz, 1H), 1.75 (s, 3H) ppm; ^{13}C NMR (151 MHz, $CDCl_3$, β anomer) δ = 206.3, 171.9, 167.7, 167.3, 164.8, 138.6, 138.25, 138.19, 137.8, 135.1, 134.0, 133.8, 133.5, 133.1, 133.0, 132.9, 131.7, 131.7, 129.9, 129.8, 129.6, 128.6, 128.4, 128.29, 128.26, 128.2, 128.1, 128.05, 128.02, 127.9, 127.8, 127.65, 127.60, 127.5, 126.4, 126.1, 125.9, 125.7, 123.6, 100.6, 83.3, 79.7, 78.7, 74.7, 74.6, 73.6, 73.3, 73.2, 72.3, 72.15, 72.08, 71.4, 67.9, 67.8, 53.9, 37.7, 29.4, 27.9, 21.1 ppm. Following General Procedure VII, to a solution of aforementioned levulinoyl disaccharide (1.45 g, 1.22 mmol, 1.0 equiv.) in CH_2Cl_2 (30 mL) at room temperature was added a solution of hydrazine acetate in MeOH (1.8 mL, *ca.* 1.0 M, 1.8 mmol, 1.5 equiv.). The reaction solution was stirred at the same temperature for another 2 hrs before it was quenched by H_2O (30 mL). The organic phase was separated, and the aqueous phase was extracted with CH_2Cl_2 (15 mL × 3). The organic phases were combined, dried over Na_2SO_4 and concentrated under vacuum. The resulting residue was purified by flash chromatography (silica gel, EtOAc:toluene = 1:4, *v*/*v*) to afford secondary alcohol **38** (1.15 g, 81% for the two steps) as a white amorphous foam. **38**: ^{1}H NMR (600 MHz, $CDCl_3$) δ = 8.01 (d, J = 7.8 Hz, 2H), 7.93 – 7.86 (m, 1H), 7.85 – 7.80 (m, 1H), 7.76 (d, J = 7.8 Hz, 1H), 7.73 – 7.66 (m, 2H), 7.65 – 7.53 (m, 4H), 7.51 – 7.38 (m, 4H), 7.37 – 7.17 (m, 16H), 7.16 – 7.05 (m, 2H), 6.94 (d, J = 7.7 Hz, 2H), 5.72 (apparent t, J = 9.0 Hz, 1H), 5.49 (d, J = 10.5 Hz, 1H), 5.01 (d, J = 11.7 Hz, 1H), 4.81 (d, J = 12.4 Hz, 1H), 4.67 – 4.59 (m, 2H), 4.56 (d, J = 8.0 Hz, 1H), 4.48 (dd, J = 10.3, 7.7 Hz, 1H), 4.34 (ABq, J = 11.5 Hz, $\Delta\nu_{AB}$ = 20.8 Hz, 2H), 4.25 (apparent t, J = 10.4 Hz, 1H), 4.07 (ABq, J = 12.0 Hz, $\Delta\nu_{AB}$ = 45.0 Hz, 2H), 3.98 (d, J = 2.8 Hz, 1H), 3.72 – 3.56 (m, 5H), 3.51 – 3.40 (m, 3H), 2.25 (s, 3H) ppm; ^{13}C NMR (151 MHz, $CDCl_3$) δ = 168.1, 167.7, 165.1, 138.6, 138.1, 138.0, 137.3, 134.8, 134.1, 134.0, 133.4, 133.2, 133.1, 133.0, 131.9, 131.8, 130.1, 129.8, 129.6, 128.6, 128.5, 128.40, 128.36, 128.3, 128.2, 128.1, 127.94, 127.92, 127.85, 127.7, 127.4, 127.3, 126.8, 126.3, 126.1, 125.8, 123.7, 123.3, 102.0, 83.5, 82.1, 79.6, 77.9, 74.6, 73.9, 73.7, 72.8, 72.01, 72.00, 71.6, 71.0, 68.54, 68.46, 55.2, 21.2 ppm.

***p*-Tolyl 2-*O*-acetyl-3,4,6-tri-*O*-benzyl-β-D-galactopyranosyl-(1→4)-6-*O*-benzyl-2-deoxy-2-phthalimido-1-thio-β-D-glucopyranoside (41)**: following General Procedure I, **S14** (476 mg, 0.788 mmol, 1.0 equiv.) was glycosylated with **S15** (519 mg, 0.867 mmol, 1.1 equiv.) under the influence of NIS (195 mg, 0.867 mmol, 1.1 equiv.) and TfOH (10.5 μL, 17.8 mg, 0.118 mmol, 0.15 equiv.) at −40 °C for 30 min in the presence of 4Å MS (1.0 g, flame dried) in CH_2Cl_2 (12 mL). Flash chromatography (silica gel, EtOAc:toluene = 1:4, *v*/*v*) afforded levulinoyl disaccharide intermediate (900 mg, quantitative yield, β:α *ca.* 3.4:1) as a white foam. Careful chromatographic purification (silica gel, long column, EtOAc:toluene = 1:10 → 1:8, *v*/*v*) afforded relatively pure α-anomer (200 mg, 23%) and β-anomer (680 mg, 80%) as white amorphous foams. ^{1}H NMR (600 MHz, $CDCl_3$,

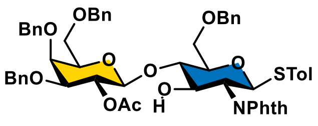

41

β anomer): δ = 7.90 – 7.84 (m, 1H), 7.84 – 7.80 (m, 1H), 7.76 – 7.67 (m, 2H), 7.39 – 7.17 (m, 22H), 7.01 (d, J = 7.7 Hz, 2H), 5.71 (apparent t, J = 9.7 Hz, 1H), 5.62 (d, J = 10.5 Hz, 1H), 5.17 (dd, J = 10.0, 8.0 Hz, 1H), 4.87 (d, J = 11.5 Hz, 1H), 4.69 (d, J = 12.0 Hz, 1H), 4.63 (d, J = 12.3 Hz, 1H), 4.53 – 4.37 (m, 6H), 4.25 (apparent t, J = 10.4 Hz, 1H), 3.98 – 3.88 (m, 2H), 3.78 (d, J = 2.6 Hz, 2H), 3.66 (apparent d, J = 9.9 Hz, 1H), 3.57 (apparent t, J = 8.5 Hz, 1H), 3.51 (dd, J = 8.8, 4.9 Hz, 1H), 3.43 – 3.38 (m, 1H), 3.36 (dd, J = 10.1, 2.8 Hz, 1H), 2.45 – 2.15 (m, 7H), 1.95 (s, 3H), 1.75 (s, 3H) ppm; ^{13}C NMR (151 MHz, $CDCl_3$) δ = 206.4, 172.0, 169.2, 167.9, 167.4, 138.7, 138.4, 138.3, 138.1, 137.9, 134.1, 134.0, 133.7, 131.83, 131.75, 129.7, 128.6, 128.52, 128.50, 128.3, 128.1, 128.0, 127.9, 127.82, 127.79, 127.7, 127.6, 127.4, 123.7, 100.7, 83.4, 80.5, 79.2, 74.9, 74.6, 73.63, 73.59, 73.2, 72.5, 72.2, 71.84, 71.78, 68.0, 67.9, 54.0, 37.8, 29.5, 28.0, 21.3, 21.1 ppm. Following General Procedure VII, to a solution of aforementioned levulinoyl disaccharide (680 mg, 0.631 mmol, 1.0 equiv.) in CH_2Cl_2 (16 mL) at room temperature was added a solution of hydrazine acetate in MeOH (0.946 mL, *ca.* 1.0 M, 0.946 mmol, 1.5 equiv.). The reaction solution was stirred at the same temperature for another 2 hrs before it was quenched by H_2O (30 mL). The organic phase was separated, and the aqueous phase was extracted with CH_2Cl_2 (15 mL × 3). The organic phases were combined, dried over Na_2SO_4 and concentrated under vacuum. The resulting residue was purified by flash chromatography (silica gel, acetone:toluene = 1:10, *v/v*) to afford secondary alcohol **41** (564 mg, 73% for the two steps) as a white amorphous foam. **41**: ^{1}H NMR (600 MHz, $CDCl_3$) δ = 7.92 – 7.87 (m, 1H), 7.84 – 7.80 (m, 1H), 7.74 – 7.67 (m, 2H), 7.39 – 7.21 (m, 18H), 7.22 – 7.15 (m, 4H), 7.00 (d, J = 7.9 Hz, 2H), 5.53 (d, J = 10.5 Hz, 1H), 5.37 (dd, J = 10.1, 8.0 Hz, 1H), 4.90 (d, J = 11.8 Hz, 1H), 4.69 – 4.62 (m, 2H), 4.55 – 4.48 (m, 3H), 4.46 (dd, J = 10.4, 7.6 Hz, 1H), 4.40 (d, J = 8.0 Hz, 1H), 4.38 (br s, 1H), 4.31 (ABq, J = 11.6 Hz, $\Delta\nu_{AB}$ = 26.5 Hz, 2H), 4.25 (apparent t, J = 10.4 Hz, 1H), 3.86 (d, J = 2.8 Hz, 1H), 3.76 (dd, J = 11.2, 1.4 Hz, 1H), 3.72 (dd, J = 11.2, 3.6 Hz, 1H), 3.69 – 3.63 (m, 2H), 3.60 – 3.54 (m, 2H), 3.45 (dd, J = 10.1, 2.8 Hz, 1H), 3.43 – 3.40 (m, 1H), 2.28 (s, 3H), 2.00 (s, 3H) ppm; ^{13}C NMR (151 MHz, $CDCl_3$) δ = 169.4, 168.1, 167.7, 138.6, 138.18, 138.15, 137.8, 137.4, 134.1, 134.0, 133.5, 132.0, 131.9, 129.7, 128.6, 128.5, 128.44, 128.36, 128.3, 128.2, 128.0, 127.9, 127.79, 127.75, 127.7, 127.6, 123.7, 123.3, 101.9, 83.6, 81.7, 80.3, 78.5, 74.6, 73.9, 73.7, 73.6, 72.31, 72.28, 71.3, 71.0, 68.6, 68.5, 55.3, 21.2, 21.1 ppm. HRMS (ESI-ToF) *m/z* calculated for $C_{66}H_{61}NNaO_{12}S^+$ $[M+Na]^+$: 1114.3807, found: 1114.3825.

2-*O*-benzoyl-3-*O*-(2-naphthylmethyl)-4,6-di-*O*-benzyl-β-D-galactopyranosyl-(1→4)-[2,3,4-tri-*O*-benzyl-α-L-fucopyranosyl-(1→3)]-6-*O*-benzyl-2-deoxy-2-phthalimido-β-D-glucopyranosyl-(1→3)-2-*O*-benzoyl-4,6-di-*O*-benzyl-β-D-galactopyranosyl 2-iodobenzoate (39): following General Procedure VI, tetrasaccharide **39** was synthesized from **31**[17] (195 mg, 0.360 mmol, 1.2 equiv.), **38** (328 mg, 0.300 mmol, 1.0 equiv.) and **22** (208 mg, 0.300 mmol, 1.0 equiv.), with NIS (79.0 mg, 0.354 mmol, 1.18 equiv.) and TfOH (4.0 μL, 6.7 mg, 45.0 μmol, 0.15 equiv.) as promoters for the 1st stage and NIS (81.0 mg, 0.360 mmol, 1.2 equiv.) and TfOH (4.0 μL, 6.7 mg, 45.0 μmol, 0.15 equiv.) for the 2nd stage. Flash chromatography (silica gel, EtOAc:toluene = 1:6, *v/v*; then EtOAc:hexanes = 1:3 → 1:2, *v/v*) afforded desired tetrasaccharide **39** (420 mg, 67% overall) as a white

foam. **39**: ^{1}H NMR (600 = MHz, $CDCl_3$) δ = 7.91 – 7.87 (m, 2H), 7.84 (dd, *J* = 7.9, 1.2 Hz, 1H), 7.78 (d, *J* = 8.1 Hz, 1H), 7.67 (dd, *J* = 7.9, 1.7 Hz, 1H), 7.66 – 7.55 (m, 5H), 7.54 – 7.35 (m, 14H), 7.34 – 7.12 (m, 31H), 7.11 – 7.04 (m, 4H), 7.02 – 6.96 (m, 3H), 6.90 – 6.86 (m, 2H), 6.77 (br s, 1H), 5.72 (d, *J* = 8.2 Hz, 1H),

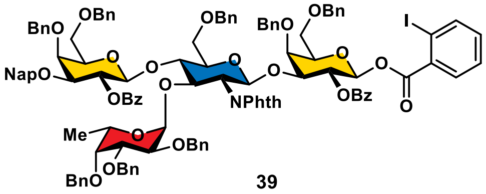

39

5.59 – 5.51 (m, 2H), 5.15 (d, *J* = 8.3 Hz, 1H), 5.12 (d, *J* = 11.6 Hz, 1H), 4.97 (d, *J* = 10.8 Hz, 1H), 4.87 (d, *J* = 12.6 Hz, 1H), 4.75 – 4.70 (m, 2H), 4.70 – 4.62 (m, 3H), 4.62 – 4.57 (m, 2H), 4.56 – 4.52 (m, 2H), 4.49 – 4.43 (m, 2H), 4.38 – 4.32 (m, 5H), 4.30 – 4.26 (m, 2H), 4.21 (apparent t, *J* = 9.4 Hz, 1H), 4.17 – 4.13 (m, 2H), 4.07 (d, *J* = 12.1 Hz, 1H), 3.91 (dd, *J* = 10.1, 2.9 Hz, 1H), 3.87 (d, *J* = 11.3 Hz, 1H), 3.84 – 3.77 (m, 4H), 3.75 (dd, *J* = 8.1, 4.2 Hz, 1H), 3.58 (dd, *J* = 10.2, 3.8 Hz, 1H), 3.57 – 3.50 (m, 4H), 3.45 (dd, *J* = 9.2, 4.5 Hz, 1H), 3.36 (apparent dt, *J* = 9.8, 2.1 Hz, 1H), 3.22 (apparent t, *J* = 2.0 Hz, 1H), 1.22 (d, *J* = 6.5 Hz, 3H) ppm; ^{13}C NMR (151 MHz, $CDCl_3$) δ = 164.8, 164.7, 163.8, 141.7, 139.4, 139.2, 138.79, 138.77, 138.2, 138.04, 137.99, 137.8, 135.3, 133.7, 133.4, 133.21, 133.20, 133.12, 133.09, 132.1, 131.8, 130.0, 129.91, 129.86, 129.1, 129.0, 128.9, 128.7, 128.6, 128.45, 128.37, 128.33, 128.29, 128.23, 128.22, 128.12, 128.09, 127.99, 127.96, 127.91, 127.88, 127.84, 127.78, 127.62, 127.59, 127.3, 127.2, 127.09, 127.06, 126.5, 126.2, 126.1, 125.7, 123.4, 100.0, 99.8, 96.8, 95.1, 93.7, 80.2, 80.1, 79.5, 78.7, 75.70, 75.66, 75.1, 74.94, 74.93, 74.7, 74.3, 73.8, 73.7, 73.6, 73.5, 72.92, 72.89, 72.6, 72.2, 72.0, 71.7, 70.7, 70.4, 68.2, 68.0, 67.7, 66.7, 56.8, 16.4 ppm.

2-*O*-benzoyl-4,6-di-*O*-benzyl-β-D-galactopyranosyl-(1→4)-[2,3,4-tri-*O*-benzyl-α-L-fucopyranosyl-(1→3)]-6-*O*-benzyl-2-deoxy-2-phthalimido-β-D-glucopyranosyl-(1→3)-2-*O*-benzoyl-4,6-di-*O*-benzyl-β-D-galactopyranosyl 2-iodobenzoate (40): following General Procedure IX, secondary alcohol **40** was synthesized from Nap ether **39** (410 mg, 0.197 mmol, 1.0 equiv.) and DDQ (90.0 mg, 0.394 mmol, 2.0 equiv.) in CH_2Cl_2:H_2O (10 mL, 10:1, *v*/*v*) at 0 °C. Flash chromatography (silica gel, EtOAc:hexanes = 1:10 → 2:3, *v*/*v*) afforded secondary alcohol **40** (265 mg, 69%) as a glassy colorless film. **40**: ^{1}H NMR (600 MHz, $CDCl_3$) δ = 8.02 – 7.96 (m, 2H), 7.84 (d, *J* = 7.9 Hz, 1H), 7.69 – 7.61 (m, 2H), 7.61 – 7.44 (m, 7H), 7.44 – 7.11 (m, 37H), 7.11 – 7.06 (m, 3H), 7.03 – 6.97 (m, 3H), 6.88 (d, *J* = 7.5 Hz, 2H), 6.77 (br s, 1H), 5.73 (d, *J* = 8.2 Hz, 1H), 5.56 (dd, *J* = 10.1, 8.2 Hz, 1H), 5.20 – 5.10 (m, 3H), 4.80 (d, *J* = 11.9 Hz, 1H), 4.76 (d,

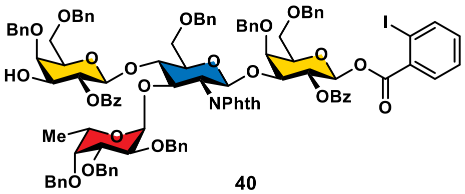

40

J = 8.1 Hz, 1H), 4.72 (d, *J* = 11.1 Hz, 1H), 4.66 (dd, *J* = 10.5, 8.9 Hz, 1H), 4.63 – 4.55 (m, 4H), 4.53 (d, *J* = 11.1 Hz, 1H), 4.49 – 4.45 (m, 3H), 4.43 (d, *J* = 11.9 Hz, 1H), 4.40 – 4.35 (m, 2H), 4.35 – 4.27 (m, 3H), 4.24 (apparent t, *J* = 9.4 Hz, 1H), 4.17 (d, *J* = 2.9 Hz, 1H), 4.06 (d, *J* = 12.1 Hz, 1H), 4.02 (d, *J* = 11.3 Hz, 1H), 3.99 – 3.92 (m, 3H), 3.84 – 3.78 (m, 2H), 3.75 – 3.67 (m, 3H), 3.65 – 3.58 (m, 2H), 3.57 – 3.51 (m, 2H), 3.49 – 3.42 (m, 2H), 3.29 – 3.26 (m, 1H), 2.31 (d, *J* = 9.2 Hz, 1H), 1.11 (d, *J* = 6.5 Hz, 3H) ppm; ^{13}C NMR (151 MHz, $CDCl_3$) δ = 166.4, 164.8, 163.8, 141.7, 139.2, 139.1, 138.8, 138.2, 138.11, 138.06, 138.0, 137.8, 133.7, 133.5, 133.4, 133.1, 132.2, 131.9, 129.94, 129.89, 129.7, 129.1, 129.0, 128.82, 128.78, 128.7, 128.6, 128.50,

128.47, 128.4, 128.34, 128.25, 128.2, 128.14, 128.08, 128.06, 128.03, 128.01, 127.98, 127.8, 127.70, 127.65, 127.4, 127.24, 127.18, 127.16, 123.4, 99.8, 99.4, 97.0, 95.1, 93.7, 80.1, 79.6, 78.6, 76.5, 76.3, 75.8, 75.2, 75.05, 74.96, 74.8, 74.4, 74.3, 74.1, 73.8, 73.7, 73.6, 73.5, 72.8, 72.7, 72.4, 71.1, 70.5, 68.22, 68.20, 67.5, 66.6, 56.8, 16.6 ppm.

2-*O*-acetyl-3,4,6-tri-*O*-benzyl-β-D-galactopyranosyl-(1→4)-[2,3,4-tri-*O*-benzyl-α-L-fucopyranosyl-(1→3)]-6-*O*-benzyl-2-deoxy-2-phthalimido-β-D-glucopyranosyl-(1→3)-2-*O*-benzoyl-4,6-di-*O*-benzyl-β-D-galactopyranosyl-(1→4)-3,6-di-*O*-benzyl-2-deoxy-2-phthalimido-β-D-glucopyranosyl-(1→3)-2-*O*-benzoyl-4,6-di-*O*-benzyl-β-D-galactopyranosyl 2-iodobenzoate (42): following General Procedure VI, hexasaccharide **42** was synthesized from **31**[17] (59.5 mg, 0.110 mmol, 1.1 equiv.), **41** (98.0 mg, 0.100 mmol, 1.0 equiv.) and **37** (145 mg, 90.0 µmol, 0.9 equiv.), with NIS (24.3 mg, 0.108 mmol, 1.08 equiv.) and TfOH (1.3 µL, 2.3 mg, 15.0 µmol, 0.15 equiv.) as promoters for the 1st stage and NIS (24.7 mg, 0.110 mmol, 1.1 equiv.) and TfOH (1.3 µL, 2.3 mg, 15.0 µmol, 0.15 equiv.) for the 2nd stage. Flash chromatography (silica gel, EtOAc:toluene = 1:6, *v*/*v*; then EtOAc:hexanes = 1:3 → 1:2, *v*/*v*) afforded desired tetrasaccharide **42** (119 mg, 46% overall) as a colorless glassy film. **42**: ^{1}H NMR (600 MHz, $CDCl_3$) δ = 7.84 (d, *J* = 7.8 Hz, 1H), 7.71 (br d, *J* = 7.3 Hz, 1H), 7.66 (dd, *J* = 8.0, 1.7 Hz, 1H), 7.64 – 7.59 (m, 2H), 7.58 – 7.52 (m, 4H), 7.49 (d, *J* = 7.7 Hz, 2H), 7.46 (apparent t, *J* = 7.4 Hz, 1H), 7.44 – 7.11 (m, 60H), 7.11 – 7.05 (m, 3H), 7.03 – 6.97 (m, 3H), 6.88 (d, *J* = 7.5 Hz, 2H), 6.83 – 6.79 (m, 3H), 6.79 – 6.63 (m, 4H), 5.75 (d, *J* = 8.2 Hz, 1H), 5.58 (dd, *J* = 10.0, 8.2 Hz, 1H), 5.36 – 5.25 (m, 2H), 5.21 (d, *J* = 8.4 Hz, 1H), 5.16 – 5.10 (m, 2H), 5.06 (d, *J* = 11.3 Hz, 1H), 4.87 (d, *J* = 10.6 Hz, 1H), 4.79 (d, *J* = 11.8 Hz, 1H), 4.76 – 4.69 (m, 3H), 4.63 – 4.49 (m, 7H), 4.49 – 4.38 (m, 7H), 4.37 – 4.29 (m, 5H), 4.26 – 4.12 (m, 5H), 4.12 – 4.00 (m, 6H), 3.96 – 3.82 (m, 5H), 3.81 – 3.71 (m, 4H), 3.68 (dd, *J* = 8.4, 4.4 Hz, 1H), 3.62 (br d, *J* = 9.7 Hz, 1H), 3.59 – 3.47 (m, 4H), 3.43 (dd, *J* = 11.0, 3.6 Hz, 1H), 3.40 – 3.29 (m, 4H), 3.27 (br d, *J* = 10.4 Hz, 1H), 3.18 – 3.12 (m, 2H), 2.07 (s, 3H), 1.12 (d, *J* = 6.4 Hz, 3H) ppm; ^{13}C NMR (151 MHz, $CDCl_3$) δ = 169.0, 168.2, 167.8, 167.2, 166.6, 164.7, 164.3, 163.8, 141.7, 139.3, 139.25, 139.18, 138.8, 138.7, 138.4, 138.2, 138.10, 138.06, 138.0, 137.8, 133.7, 133.6, 133.4, 133.33, 133.26, 133.01, 132.97, 132.1, 132.0, 131.5, 130.9, 129.83, 129.75, 129.4, 128.84, 128.78, 128.6, 128.55, 128.51, 128.50, 128.44, 128.36, 128.30, 128.28, 128.24, 128.20, 128.1, 128.0, 127.95, 127.93, 127.91, 127.89, 127.85, 127.83, 127.76, 127.74, 127.72, 127.71, 127.66, 127.62, 127.56, 127.5, 127.4, 127.34, 127.28, 127.2, 127.15, 127.07, 127.0, 126.6, 123.5, 123.1, 122.9, 100.9, 100.0, 99.9, 99.7, 96.9, 95.0, 93.5, 80.9, 80.4, 80.3, 79.4, 78.7, 77.3, 76.5, 76.4, 75.8, 75.5, 75.4, 75.2, 75.0, 74.9, 74.7, 74.6, 74.4, 74.2, 73.7, 73.6, 73.48, 73.47, 73.4, 73.3, 73.0, 72.9, 72.6, 72.1, 72.0, 71.9, 71.7, 70.7, 70.4, 68.6, 68.3, 68.1, 67.7, 67.6, 66.6, 56.8, 56.0, 21.1, 16.2 ppm. The 2-iodobenzoate was elaborated to the corresponding *o*-alkynylbenzoate following General Procedure II and the spectral data are as follows: ^{1}H NMR (600 MHz, $CDCl_3$) δ = 7.76 – 7.67 (m, 2H), 7.66 – 7.50 (m, 6H), 7.49 – 7.12 (m, 64H), 7.11 – 7.03 (m, 4H), 6.99

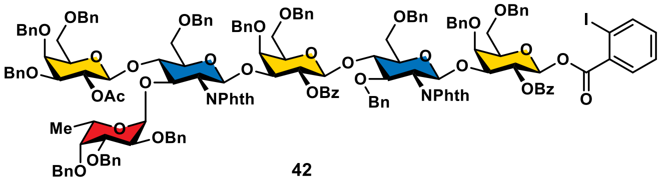

42

(apparent t, *J* = 7.5 Hz, 2H), 6.88 (d, *J* = 7.5 Hz, 2H), 6.84 – 6.66 (m, 7H), 5.75 (d, *J* = 8.2 Hz, 1H), 5.58 (dd, *J* = 10.1, 8.2 Hz, 1H), 5.39 –5.24 (m, 2H), 5.21 (d, *J* = 8.4 Hz, 1H), 5.13 (d, *J* = 3.7 Hz, 1H), 5.11 (s, 1H), 5.07 (d, *J* = 11.3 Hz, 1H), 4.88 (d, *J* = 10.6 Hz, 1H), 4.79 (d, *J* = 11.9 Hz, 1H), 4.77 – 4.69 (m, 3H), 4.64 – 4.38 (m, 14H), 4.36 – 4.28 (m, 5H), 4.25 – 3.99 (m, 11H), 3.97 – 3.81 (m, 5H), 3.81 – 3.65 (m, 5H), 3.62 (br d, *J* = 9.8 Hz, 1H), 3.59 – 3.53 (m, 3H), 3.51 (br t, *J* = 6.4 Hz, 1H), 3.43 (dd, *J* = 10.9, 3.6 Hz, 1H), 3.41 – 3.29 (m, 4H), 3.27 (d, *J* = 10.5 Hz, 1H), 3.20 – 3.11 (m, 2H), 2.07 (s, 3H), 1.39 (apparent tt, *J* = 7.9, 5.5 Hz, 1H), 1.12 (d, *J* = 6.4 Hz, 3H), 0.80 – 0.71 (m, 4H) ppm; ^{13}C NMR (151 MHz, $CDCl_3$) δ = 169.0, 168.1, 167.8, 167.2, 166.6, 164.6, 164.3, 163.6, 139.33, 139.26, 139.2, 138.9, 138.8, 138.7, 138.4, 138.3, 138.10, 138.07, 138.02, 137.98, 137.8, 134.1, 133.7, 133.5, 133.4, 133.2, 133.0, 132.8, 132.1, 131.6, 131.4, 131.0, 130.9, 130.7, 129.8, 129.7, 129.5, 129.4, 129.0, 128.83, 128.80, 128.61, 128.59, 128.55, 128.51, 128.49, 128.42, 128.36, 128.30, 128.28, 128.2, 128.13, 128.11, 128.00, 127.96, 127.93, 127.89, 127.8, 127.74, 127.71, 127.65, 127.61, 127.55, 127.5, 127.4, 127.34, 127.28, 127.2, 127.15, 127.06, 127.0, 126.9, 126.6, 125.5, 123.5, 123.1, 122.8, 100.9, 100.2, 100.0, 99.9, 99.7, 96.8, 93.1, 80.9, 80.4, 80.3, 79.4, 78.7, 77.3, 76.5, 76.4, 75.9, 75.5, 75.4, 75.2, 75.0, 74.9, 74.7, 74.5, 74.4, 74.2, 73.7, 73.6, 73.5, 73.4, 73.3, 73.0, 72.9, 72.6, 72.1, 72.0, 71.9, 71.7, 70.7, 70.5, 68.6, 68.3, 68.1, 67.7, 67.6, 66.6, 56.8, 56.0, 21.1, 16.2, 8.93, 8.91, 0.7 ppm.

2-*O*-acetyl-3,4,6-tri-*O*-benzyl-β-D-galactopyranosyl-(1→4)-3,6-di-*O*-benzyl-2-deoxy-2-phthalimido-β-D-glucopyranosyl-(1→3)-2-*O*-benzoyl-4,6-di-*O*-benzyl-β-D-galactopyranosyl-(1→4)-[2,3,4-tri-*O*-benzyl-α-L-fucopyranosyl-(1→3)]-6-*O*-benzyl-2-deoxy-2-phthalimido-β-D-glucopyranosyl-(1→3)-2-*O*-benzoyl-4,6-di-*O*-benzyl-β-D-galactopyranosyl 2-(cyclopropylethynyl)benzoate (44): following General Procedure VI, hexasaccharide **44** was synthesized from **43**[11] (65.9 mg, 0.110 mmol, 1.1 equiv.), **35**[11] (59.6 mg, 0.100 mmol, 1.0 equiv.) and **40** (175 mg, 90.0 µmol, 0.9 equiv.), with NIS (24.3 mg, 0.108 mmol, 1.08 equiv.) and TfOH (1.3 µL, 2.3 mg, 15.0 µmol, 0.15 equiv.) as promoters for the 1st stage and NIS (24.7 mg, 0.110 mmol, 1.1 equiv.) and TfOH (1.3 µL, 2.3 mg, 15.0 µmol, 0.15 equiv.) for the 2nd stage. Flash chromatography (silica gel, EtOAc:toluene = 1:6, *v*/*v*; then EtOAc:hexanes = 1:3 → 1:2, *v*/*v*) afforded desired tetrasaccharide **44** (142 mg, 55% overall) as a colorless glassy film. The 2-iodobenzoate was elaborated to the corresponding *o*-alkynylbenzoate following General Procedure II and the spectral data are as follows: ^{1}H NMR (600 MHz, $CDCl_3$) δ = 7.74 (br s, 1H), 7.66 (br d, *J* = 8.0 Hz, 1H), 7.61 – 7.45 (m, 7H), 7.45 – 7.10 (m, 64H), 7.08 – 7.01 (m, 4H), 6.97 – 6.90 (m, 2H), 6.85 – 6.78 (m, 5H), 6.77 – 6.73 (m, 2H), 6.69 (br s, 1H), 5.68 (d, *J* = 8.2 Hz, 1H), 5.50 (dd, *J* = 10.1, 8.2 Hz, 1H), 5.33 (dd, *J* = 10.1, 7.8 Hz, 1H), 5.27 – 5.18 (m, 2H), 5.10 – 5.04 (m, 2H), 5.03 (d, *J* = 8.3 Hz, 1H), 4.89 (d, *J* = 11.6 Hz, 1H), 4.80 (d, *J* = 11.9 Hz, 1H), 4.70 (d, *J* = 12.0 Hz, 1H), 4.64 (d, *J* = 12.3 Hz, 1H), 4.59 – 4.39 (m, 12H), 4.39 – 4.30 (m, 5H), 4.30 – 4.15 (m, 10H), 4.11 – 4.05 (m, 2H), 4.00 (d, *J* = 12.2 Hz, 1H), 3.94 – 3.87 (m, 2H), 3.83 (dd, *J* = 10.1, 2.8 Hz, 1H), 3.79 – 3.67

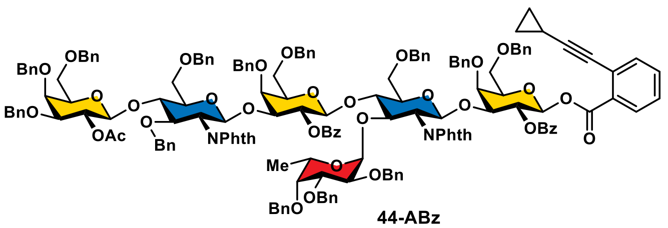

44-ABz

(m, 7H), 3.68 – 3.59 (m, 3H), 3.55 – 3.44 (m, 4H), 3.39 – 3.31 (m, 4H), 3.29 (dd, *J* = 7.4, 3.5 Hz, 1H), 3.13 – 3.04 (m, 2H), 1.99 (s, 3H), 1.36 (apparent quint, *J* = 6.7 Hz, 1H), 0.94 (d, *J* = 6.5 Hz, 3H), 0.76 – 0.72 (m, 4H) ppm; ^{13}C NMR (151 MHz, $CDCl_3$) δ = 169.5, 164.7, 163.7, 163.6, 139.5, 139.3, 139.2, 138.9, 138.8, 138.20, 138.16, 138.13, 138.10, 138.07, 138.06, 138.0, 134.2, 133.8, 133.5, 133.3, 133.0, 132.7, 132.1, 131.1, 129.8, 129.5, 129.4, 129.3, 129.1, 129.0, 128.8, 128.54, 128.53, 128.51, 128.45, 128.3, 128.24, 128.22, 128.1, 127.99, 127.95, 127.94, 127.91, 127.87, 127.84, 127.83, 127.79, 127.77, 127.7, 127.62, 127.55, 127.53, 127.48, 127.4, 127.3, 127.02, 126.97, 126.96, 126.8, 125.6, 123.4, 101.0, 100.2, 100.05, 99.98, 99.7, 96.8, 93.2, 81.3, 80.5, 80.1, 79.3, 78.8, 78.0, 76.7, 76.4, 75.9, 75.8, 75.2, 75.1, 75.0, 74.8, 74.7, 74.6, 74.5, 74.4, 74.1, 73.9, 73.6, 73.5, 73.44, 73.38, 72.9, 72.7, 72.5, 72.1, 72.0, 71.8, 71.3, 70.49, 70.46, 68.6, 68.2, 68.1, 67.8, 67.7, 66.5, 56.7, 56.1, 21.2, 16.1, 9.0, 8.9, 0.7 ppm.

Scheme S10. Assembly of Asymmetric Bi-antennary *N*-Glycans from Precursor 1 and *O*-Alkynylbenzoate Modules A-I[a]

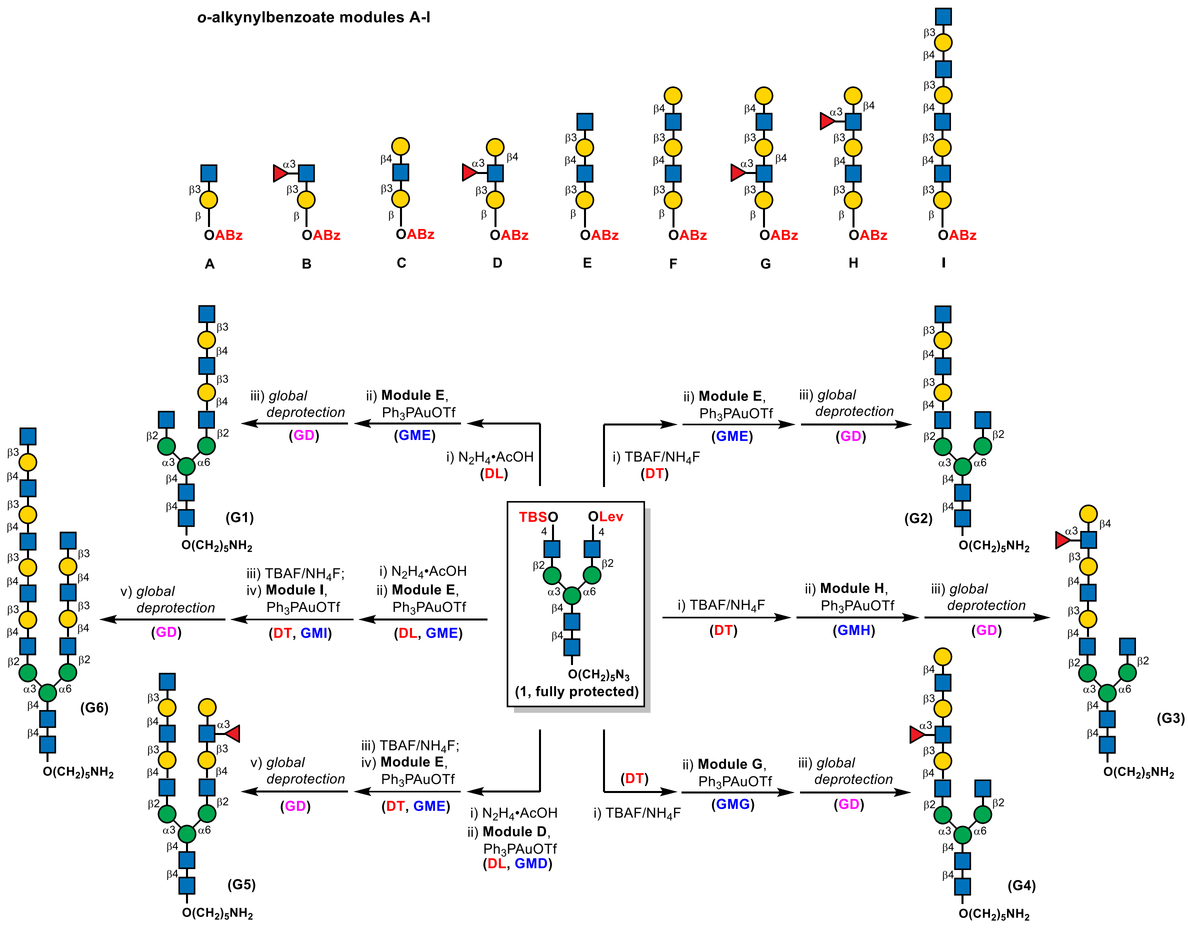

[a]Reagents and conditions: (**DL**) $N_2H_4 \cdot AcOH$ (3.0 equiv.), CH_2Cl_2:MeOH (20:1, *v*/*v*), r.t., 3 hrs; (**DT**) TBAF (5.0 equiv.), NH_4F (10.0 equiv.), THF, r.t., 12 hrs; (**GMX**) **Module X** (X = D, E, G, H, I) (2.0 equiv.), $Ph_3PAuOTf$ (1.0 equiv.), 4Å MS, CH_2Cl_2, 0 °C, 30 min; (**GD**) i) EDA:*n*-BuOH (1:4, *v*/*v*), 90 °C, 18 hrs; ii) pyridine:Ac_2O (2:1, *v*/*v*), r.t., 24 hrs; iii) NaOMe (final concentration: 0.2 M), MeOH, r.t., 12 hrs; iv) $Pd(OH)_2$/C, H_2, THF:H_2O:*t*-BuOH (6:3:1, *v*/*v*/*v*), 3-12 hrs.

5-Aminopentyl 2-acetamido-2-deoxy-β-D-glucopyranosyl-(1→2)-α-D-mannopyranosyl-(1→3)-[2-acetamido-2-deoxy-β-D-glucopyranosyl-(1→3)-β-D-galactopyranosyl-(1→4)-2-acetamido-2-deoxy-β-D-glucopyranosyl-(1→3)-β-D-galactopyranosyl-(1→4)-2-acetamido-2-deoxy-β-D-glucopyranosyl-(1→2)-α-D-mannopyranosyl-(1→6)]-β-D-mannopyranosyl-(1→4)-2-acetamido-2-deoxy-β-D-glucopyranosyl-(1→4)-2-acetamido-2-deoxy-β-D-glucopyranoside (G1): asymmetric bi-antennary *N*-glycan **G1** was synthesized from the bi-antennary precursor **1** and *o*-alkynylbenzoate **Module E** following General Procedures VII, III, VIII, XI and XII. An exemplified procedure was as follows: to a solution of fully protected heptasaccharide **1** (80.0 mg, 23.3 μmol, 1.0 equiv.) in CH_2Cl_2 (2.5 mL) at room temperature was added a solution of $N_2H_4 \cdot AcOH$ in MeOH (0.140 mL, *ca.* 0.5 M, 70.0 μmol, 3.0 equiv.). The reaction was stirred at the same temperature for another 3 hrs before it was quenched by H_2O (2.0 mL) and diluted by CH_2Cl_2 (2.0 mL). The organic phase was separated and the aqueous phase was extracted by CH_2Cl_2 (5.0 mL × 3). The organic phases were combined, dried over

Na_2SO_4, and concentrated under vacuum. The resulting residue was briefly purified by flash chromatography (silica gel, EtOAc:hexanes = 1:4 → 1:1, *v*/*v*) to afford secondary alcohol intermediate (68.0 mg, 88%) which was directly used for the pending glycosylation reactions. To a suspension of so-obtained acceptor (28.0 mg, 8.6 μmol, 1.0 equiv.), tetrasaccharide donor (**Module E**, 37.0 mg, 17.2 μmol, 2.0 equiv.) and 4Å MS (150 mg, flame dried) in CH_2Cl_2 (1.0 mL) at 0 °C was slowly added a solution of $Ph_3PAuOTf$ in CH_2Cl_2 (0.172 mL, *ca.* 0.05 M, 8.6 μmol, 1.0 equiv.). The reaction suspension was stirred at the same temperature for another 30 min before it was directly subjected to a flash column (silica gel, EtOAc:hexanes = 1:4 → 1:1, *v*/*v*), which provided the fully protected undecasaccharide intermediate (31.0 mg, 68%) as a colorless glassy film. ^{1}H NMR (600 MHz, $CDCl_3$) δ = 7.83 – 7.75 (m, 3H), 7.75 – 7.65 (m, 5H), 7.65 – 7.50 (m, 13H), 7.50 – 7.05 (m, 89H), 7.05 – 6.94 (m, 11H), 6.94 – 6.66 (m, 30H), 6.24 (t, *J* = 7.3 Hz, 1H), 6.17 (t, *J* = 7.4 Hz, 2H), 5.29 (dd, *J* = 10.2, 8.1 Hz, 1H), 5.25 (dd, *J* = 10.0, 8.1 Hz, 1H), 5.21 (d, *J* = 8.4 Hz, 1H), 5.14 (d, *J* = 8.0 Hz, 1H), 5.09 – 5.03 (m, 2H), 5.00 (d, *J* = 11.7 Hz, 1H), 4.97 – 4.93 (m, 2H), 4.91 (br d, *J* = 7.7 Hz, 1H), 4.88 (br d, *J* = 8.4 Hz, 1H), 4.85 – 4.65 (m, 8H), 4.61 – 4.52 (m, 5H), 4.52 – 4.48 (m, 3H), 4.48 – 4.33 (m, 15H), 4.33 – 4.21 (m, 10H), 4.20 – 3.95 (m, 21H), 3.95 – 3.89 (m, 2H), 3.89 – 3.79 (m, 5H), 3.78 – 3.52 (m, 15H), 3.52 – 3.40 (m, 6H), 3.40 – 3.30 (m, 9H), 3.29 – 3.15 (m, 7H), 3.15 – 3.08 (m, 3H), 3.06 (br d, *J* = 10.1 Hz, 1H), 2.93 – 2.78 (m, 4H), 2.70 – 2.61 (m, 3H), 2.53 – 2.44 (m, 2H), 2.40 (dt, *J* = 17.4, 6.4 Hz, 1H), 2.32 – 2.24 (m, 1H), 2.15 (s, 3H), 1.88 (br d, *J* = 9.9 Hz, 1H), 1.43 – 1.25 (m, 4H), 1.15 – 1.01 (m, 2H), 0.89 (s, 9H), 0.02 (s, 3H), −0.04 (s, 3H) ppm; ^{13}C NMR (151 MHz, $CDCl_3$) δ = 206.3, 171.8, 168.2, 168.1, 167.8, 167.6, 167.5, 167.2, 164.4, 164.3, 139.5, 139.2, 139.11, 139.10, 139.07, 138.93, 138.90, 138.8, 138.60, 138.56, 138.54, 138.51, 138.47, 138.41, 138.36, 138.28, 138.26, 138.2, 138.1, 138.0, 137.9, 134.0, 133.7, 133.6, 133.44, 133.37, 133.3, 133.2, 133.0, 132.0, 131.9, 131.80, 131.75, 131.4, 131.3, 129.84, 129.82, 129.7, 129.6, 129.53, 129.50, 128.9, 128.8, 128.70, 128.66, 128.6, 128.54, 128.50, 128.42, 128.39, 128.36, 128.29, 128.27, 128.23, 128.16, 128.14, 128.11, 128.10, 128.07, 128.04, 127.96, 127.93, 127.89, 127.86, 127.81, 127.78, 127.75, 127.72, 127.68, 127.62, 127.55, 127.51, 127.50, 127.48, 127.45, 127.41, 127.40, 127.36, 127.3, 127.22, 127.19, 127.12, 127.10, 127.06, 126.9, 126.7, 126.2, 123.6, 123.4, 123.29, 123.26, 123.0, 122.9, 101.8, 100.7, 100.6, 99.7, 99.6, 98.4, 98.2, 97.8, 97.1, 96.9, 95.6, 80.9, 80.8, 80.24, 80.16, 80.1, 79.9, 78.2, 77.75, 77.73, 77.3, 76.8, 76.6, 76.5, 76.3, 76.1, 75.6, 75.4, 75.2, 75.1, 75.0, 74.9, 74.8, 74.72, 74.68, 74.63, 74.55, 74.5, 74.4, 74.1, 74.0, 73.91, 73.88, 73.8, 73.72, 73.66, 73.62, 73.59, 73.56, 73.5, 73.41, 73.37, 73.3, 72.95, 72.94, 72.8, 72.7, 72.5, 72.4, 72.24, 72.18, 72.10, 72.07, 71.9, 70.6, 70.4, 70.0, 69.6, 69.2, 69.0, 68.7, 68.3, 67.73, 67.69, 67.6, 66.5, 56.7, 56.1, 55.9, 55.8, 55.5, 51.2, 37.8, 29.9, 28.8, 28.4, 28.0, 26.1, 23.1, 18.1, −3.6, −4.7 ppm. A solution of so-obtained fully protected undecasaccharide (31.0 mg, 4.7 μmol) in a mixed solvent *n*-BuOH/EDA (2.0 mL, 4:1, *v*/*v*) was stirred at 90 °C for 18 hrs before it was cooled back to room temperature. All the volatiles were azeotropically removed under vacuum, and the residue was dissolved in pyridine/Ac_2O (1.5 mL, 2:1, *v*/*v*). The reaction mixture was stirred at room temperature for 24 hrs and was then dried azeotropically with PhMe (10 mL × 3). The residue was

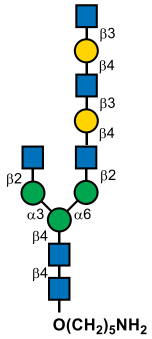

G1 (A3)

dissolved in MeOH (2.0 mL) and NaOMe (0.2 mL, *ca.* 5.4 M in MeOH, final concentration: 0.5 M) was added at room temperature. The reaction was stirred for another 24 hrs and was neutralized by Amberlite™ IR-120 (washed by MeOH). The suspension was filtered and concentrated under vacuum, and the residue was purified briefly by flash chromatography (silica gel, PhMe:acetone = 4:1 → 1:1, *v*/*v*). The resulting tetra-ol (14.0 mg) was subjected to the final hydrogenolysis using H-Cube® Mini (*cf.* General Procedure XII) and P2 purification followed by lyophilization afforded undecasaccharide **G1** (7.5 mg, 60% overall from the fully protected undecasaccharide intermediate) as a fluffy white solid. **G1**: ^{1}H NMR (600 MHz, D_2O) δ = 5.12 (br s, 1H), 4.93 (br s, 1H), 4.77 (br s, 1H), 4.71 (d, *J* = 9.6 Hz, 1H), 4.69 (d, *J* = 8.5 Hz, 1H), 4.60 (d, *J* = 8.3 Hz, 1H), 4.59 (d, *J* = 8.3 Hz, 1H), 4.56 (d, *J* = 8.4 Hz, 1H), 4.50 (d, *J* = 7.7 Hz, 1H), 4.49 – 4.45 (m, 2H), 4.25 (d, *J* = 2.8 Hz, 1H), 4.20 (d, *J* = 3.4 Hz, 1H), 4.16 (apparent t, *J* = 3.7 Hz, 2H), 4.11 (d, *J* = 3.7 Hz, 1H), 4.02 – 3.40 (m, 63H), 2.99 (apparent t, *J* = 7.6 Hz, 2H), 2.09 (s, 3H), 2.06 (s, 3H), 2.05 (s, 3H), 2.05 – 2.04 (m, 6H), 2.04 (s, 3H), 1.67 (apparent quint, *J* = 7.7 Hz, 2H), 1.59 (apparent hept, *J* = 6.5 Hz, 2H), 1.44 – 1.37 (m, 2H) ppm; ^{13}C NMR (151 MHz, D_2O) δ = 174.94, 174.89, 174.74, 174.70, 174.6, 174.4, 103.0, 102.9, 102.8, 102.7, 101.4, 101.0, 100.4, 99.61, 99.58, 99.4, 97.0, 82.1, 82.0, 80.4, 79.4, 79.3, 78.5, 78.1, 76.4, 76.3, 75.8, 75.6, 74.9, 74.7, 74.52, 74.49, 74.4, 73.5, 73.2, 72.8, 72.4, 72.2, 72.05, 71.98, 70.2, 70.1, 70.0, 69.93, 69.88, 69.7, 69.42, 69.37, 68.30, 68.26, 67.32, 67.27, 65.8, 65.6, 61.7, 61.6, 60.94, 60.91, 60.6, 60.4, 60.1, 59.94, 59.89, 59.8, 55.6, 55.3, 55.1, 55.0, 54.9, 54.8, 39.3, 28.0, 26.3, 22.31, 22.29, 22.21, 22.16, 22.14, 22.11, 22.07 ppm. LRMS (ESI-MS) *m*/*z* calculated for $C_{83}H_{139}N_7O_{56}^{2-}$ $[M-2H]^{2-}$: 1064.9, found: 1065.0.

5-Aminopentyl 2-acetamido-2-deoxy-β-D-glucopyranosyl-(1→3)-β-D-galactopyranosyl-(1→4)-2-acetamido-2-deoxy-β-D-glucopyranosyl-(1→3)-β-D-galactopyranosyl-(1→4)-2-acetamido-2-deoxy-β-D-glucopyranosyl-(1→2)-α-D-mannopyranosyl-(1→3)-[2-acetamido-2-deoxy-β-D-glucopyranosyl-(1→2)-α-D-mannopyranosyl-(1→6)]-β-D-mannopyranosyl-(1→4)-2-acetamido-2-deoxy-β-D-glucopyranosyl-(1→4)-2-acetamido-2-deoxy-β-D-glucopyranoside (G2): similar to **G1**, asymmetric bi-antennary *N*-glycan **G2** was synthesized from the bi-antennary precursor **1** and *o*-alkynylbenzoate **Module E** following General Procedures VIII, III, XI and XII sequentially. **G2** (9.0 mg, 55% overall from the undecasaccharide intermediate) was acquired from P2 purification and lyophilization as a fluffy white solid. **G2**: ^{1}H NMR (600 MHz, D_2O) δ = 5.12 (br s, 1H), 4.93 (br s, 1H), 4.78 (s, 1H), 4.72 – 4.67 (m, 2H), 4.60 (d, *J* = 7.9 Hz, 1H), 4.58 (br d, *J* = 7.6 Hz, 1H), 4.56 (d, *J* = 8.5 Hz, 1H), 4.50 (d, *J* = 7.6 Hz, 1H), 4.49 – 4.44 (m, 2H), 4.25 (d, *J* = 2.6 Hz, 1H), 4.20 (d, *J* = 3.3 Hz, 1H), 4.18 – 4.14 (m, 2H), 4.13 – 4.10 (m, 1H), 4.02 – 3.40 (m, 63H), 2.99 (apparent t, *J* = 7.7 Hz, 2H), 2.09 (s, 3H), 2.08 – 2.05 (m, 6H), 2.05 – 2.02 (m, 9H), 1.67 (apparent quint, *J* = 7.7 Hz, 2H), 1.60 (apparent quint, *J* = 6.6 Hz, 2H), 1.44 – 1.36 (m, 2H) ppm; ^{13}C NMR (151 MHz, D_2O) δ = 174.94, 174.89, 174.8, 174.7, 174.6, 174.4, 102.93, 102.86, 102.8, 102.7, 101.4, 101.0, 100.4, 99.6, 99.5, 99.4, 97.0, 82.04, 81.97, 80.4, 79.5, 79.3, 78.5, 78.1, 76.4, 76.3, 75.8, 75.6, 74.9, 74.8, 74.7, 74.52, 74.49, 74.34, 74.29, 73.5, 73.4, 72.8, 72.4, 72.2, 72.0,

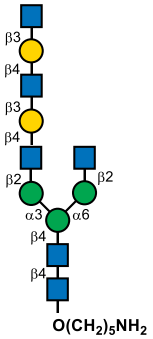

G2 (A4)

71.9, 70.2, 70.1, 70.0, 69.93, 69.87, 69.7, 69.43, 69.36, 68.3, 67.32, 67.26, 65.9, 65.7, 61.7, 61.6, 60.94, 60.92, 60.6, 60.4, 60.1, 59.95, 59.89, 59.8, 55.6, 55.3, 55.1, 55.0, 54.9, 54.8, 39.3, 28.0, 26.4, 22.31, 22.30, 22.18, 22.15, 22.14, 22.11, 22.07 ppm. LRMS (ESI-MS) *m/z* calculated for $C_{83}H_{139}N_7O_{56}^{2-}$ $[M-2H]^{2-}$: 1064.9, found: 1065.2.

5-Aminopentyl β-D-galactopyranosyl-(1→4)-[α-L-fucopyranosyl-(1→3)]-2-acetamido-2-deoxy-β-D-glucopyranosyl-(1→3)-β-D-galactopyranosyl-(1→4)-2-acetamido-2-deoxy-β-D-glucopyranosyl-(1→3)-β-D-galactopyranosyl-(1→4)-2-acetamido-2-deoxy-β-D-glucopyranosyl-(1→2)-α-D-mannopyranosyl-(1→3)-[2-acetamido-2-deoxy-β-D-glucopyranosyl-(1→2)-α-D-mannopyranosyl-(1→6)]-β-D-mannopyranosyl-(1→4)-2-acetamido-2-deoxy-β-D-glucopyranosyl-(1→4)-2-acetamido-2-deoxy-β-D-glucopyranoside (G3): similar to **G1**, asymmetric bi-antennary *N*-glycan **G3** was synthesized from the bi-antennary precursor **1** and *o*-alkynylbenzoate **Module H** following General Procedures VIII, III, XI and XII sequentially. **G3** (6.8 mg, 58% overall from 28.0 mg tridecasaccharide intermediate) was acquired from P2 purification and lyophilization as a fluffy white solid. **The tridecasaccharide intermediate**: ^1H NMR (600 MHz, $CDCl_3$) δ = 7.90 – 6.51 (m, 179H), 5.37 – 5.23 (m, 3H), 5.20 (d, *J* = 9.6 Hz, 1H), 5.16 (br d, *J* = 6.9 Hz, 1H), 5.11 (d, *J* = 12.0 Hz, 1H), 5.08 (apparent t, *J* = 9.5 Hz, 1H), 5.03 (d, *J* = 11.6 Hz, 1H), 4.97 (d, *J* = 7.1 Hz, 1H), 4.93 – 4.90 (m, 1H), 4.89 – 4.65 (m, 13H), 4.62 – 2.99 (m, 120H), 2.97 – 2.76 (m, 4H), 2.76 – 2.68 (m, 2H), 2.68 – 2.51 (m, 3H), 2.45 – 2.30 (m, 2H), 2.14 (s, 3H), 2.06 (s, 3H), 2.04 – 1.98 (m, 1H), 1.41 – 1.19 (m, 4H), 1.11 (d, *J* = 6.3 Hz, 3H), 1.09 – 1.01 (m, 2H) ppm; ^{13}C NMR (151 MHz, $CDCl_3$) δ = 206.5, 171.5, 169.0, 168.2, 168.1, 167.8, 167.6, 167.5, 167.1, 164.4, 164.2, 139.34, 139.26, 139.2, 139.14, 139.10, 139.07, 138.93, 138.90, 138.87, 138.85, 138.7, 138.6, 138.5, 138.44, 138.42, 138.37, 138.3, 138.25, 138.20, 138.11, 138.08, 137.99, 137.95, 137.8, 134.3, 134.2, 133.6, 133.3, 133.0, 132.9, 132.0, 131.7, 131.6, 129.8, 129.7, 129.5, 129.44, 129.39, 129.3, 128.9, 128.84, 128.80, 128.64, 128.61, 128.59, 128.55, 128.52, 128.50, 128.45, 128.40, 128.37, 128.34, 128.28, 128.24, 128.20, 128.15, 128.12, 128.09, 128.00, 127.96, 127.93, 127.89, 127.87, 127.84, 127.76, 127.74, 127.71, 127.67, 127.63, 127.58, 127.55, 127.51, 127.48, 127.42, 127.38, 127.35, 127.33, 127.28, 127.25, 127.21, 127.16, 127.1, 127.0, 126.9, 126.7, 126.6, 125.4, 123.5, 123.2, 101.8, 100.9, 100.2, 100.0, 99.9, 99.6, 98.9, 98.1, 97.8, 97.1, 96.9, 81.4, 80.9, 80.6, 80.4, 79.8, 79.4, 78.7, 78.2, 77.7, 77.4, 77.0, 76.65, 76.62, 76.5, 76.4, 76.3, 76.1, 75.5, 75.4, 75.04, 74.96, 74.93, 74.88, 74.7, 74.6, 74.54, 74.45, 74.2, 74.0, 73.7, 73.64, 73.60, 73.53, 73.49, 73.47, 73.42, 73.40, 73.38, 73.37, 73.3, 73.2, 73.1, 72.99, 72.97, 72.9, 72.8, 72.7, 72.6, 72.5, 72.42, 72.38, 72.1, 72.04, 71.99, 71.95, 71.90, 71.7, 70.74, 70.67, 70.5, 70.0, 69.8, 69.7, 68.9, 68.7, 68.6, 68.3, 68.2, 67.6, 66.9, 66.6, 56.8, 56.7, 56.0, 55.8, 55.7, 55.2, 51.2, 37.8, 29.9, 28.8, 28.4, 27.9, 23.1, 21.1, 16.3 ppm. **G3**: ^1H NMR (600 MHz, D_2O) δ = 5.13 (d, *J* = 4.1 Hz, 1H), 5.12 (s, 1H), 4.92 (s, 1H), 4.85 (q, *J* = 6.6 Hz, 1H), 4.78 (s, 1H), 4.74 – 4.69 (m, 2H), 4.62 – 4.54 (m, 3H), 4.52 – 4.44 (m, 4H), 4.26 (d, *J* = 2.7 Hz, 1H), 4.19 (d, *J* = 3.9 Hz, 1H), 4.18 – 4.15 (m, 2H), 4.11 (dd, *J* = 3.4, 1.6 Hz, 1H), 4.02 – 3.40 (m,

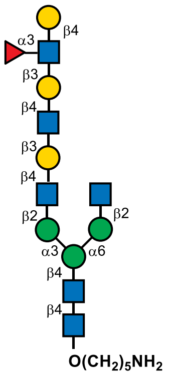

G3 (A18)

72H), 2.99 (apparent t, *J* = 7.7 Hz, 2H), 2.09 (s, 3H), 2.07 – 2.05 (m, 6H), 2.05 – 2.01 (m, 9H), 1.67 (apparent quint, *J* = 7.7 Hz, 2H), 1.62 – 1.56 (m, 2H), 1.43 – 1.37 (m, 2H), 1.18 (d, *J* = 6.6 Hz, 3H) ppm; ^{13}C NMR (151 MHz, D_2O) δ = 174.9, 174.8, 174.7, 174.6, 174.4, 102.93, 102.86, 102.7, 102.5, 101.7, 101.4, 101.0, 100.4, 99.5, 99.4, 98.6, 97.0, 82.1, 80.4, 79.5, 79.3, 78.5, 78.1, 76.4, 76.2, 75.8, 75.1, 74.9, 74.8, 74.7, 74.52, 74.49, 74.34, 74.29, 73.5, 73.4, 73.0, 72.8, 72.45, 72.37, 72.1, 71.95, 71.89, 71.0, 70.2, 70.1, 69.93, 69.87, 69.43, 69.36, 69.2, 68.33, 68.27, 67.7, 67.32, 67.26, 66.7, 65.9, 65.7, 61.7, 61.6, 61.5, 60.9, 60.6, 60.1, 59.94, 59.89, 59.8, 59.6, 55.9, 55.3, 55.1, 55.0, 54.9, 54.8, 39.3, 28.0, 26.4, 22.3, 22.22, 22.18, 22.15, 22.11, 22.07, 15.3 ppm. LRMS (ESI-MS) *m/z* calculated for $C_{95}H_{159}N_7O_{65}^{2-}$ [M-2H]$^{2-}$: 1219.5, found: 1219.5.

5-Aminopentyl β-D-galactopyranosyl-(1→4)-2-acetamido-2-deoxy-β-D-glucopyranosyl-(1→3)-β-D-galactopyranosyl-(1→4)-[α-L-fucopyranosyl-(1→3)]-2-acetamido-2-deoxy-β-D-glucopyranosyl-(1→3)-β-D-galactopyranosyl-(1→4)-2-acetamido-2-deoxy-β-D-glucopyranosyl-(1→2)-α-D-mannopyranosyl-(1→3)-[2-acetamido-2-deoxy-β-D-glucopyranosyl-(1→2)-α-D-mannopyranosyl-(1→6)]-β-D-mannopyranosyl-(1→4)-2-acetamido-2-deoxy-β-D-glucopyranosyl-(1→4)-2-acetamido-2-deoxy-β-D-glucopyranoside (G4): similar to **G1**, asymmetric bi-antennary *N*-glycan **G4** was synthesized from the bi-antennary precursor **1** and *o*-alkynylbenzoate **Module G** following General Procedures VIII, III, XI and XII sequentially. **G4** (4.7 mg, 60% overall from 18.0 mg tridecasaccharide intermediate) was acquired from P2 purification and lyophilization as a fluffy white solid. **The tridecasaccharide intermediate**: ^{1}H NMR (600 MHz, $CDCl_3$) δ = 7.86 – 6.50 (m, 179H), 5.34 (dd, *J* = 10.0, 7.7 Hz, 1H), 5.30 – 5.18 (m, 3H), 5.15 (br d, *J* = 6.8 Hz, 1H), 5.12 – 5.03 (m, 3H), 4.95 – 4.87 (m, 3H), 4.86 – 4.69 (m, 8H), 4.69 – 4.63 (m, 3H), 4.61 – 4.16 (m, 52H), 4.15 – 3.94 (m, 14H), 3.94 – 3.81 (m, 8H), 3.80 – 3.55 (m, 18H), 3.54 – 3.41 (m, 6H), 3.41 – 3.17 (m, 18H), 3.16 – 3.02 (m, 5H), 2.92 – 2.82 (m, 2H), 2.82 – 2.76 (m, 2H), 2.75 – 2.68 (m, 2H), 2.68 – 2.63 (m, 1H), 2.63 – 2.52 (m, 2H), 2.45 – 2.31 (m, 2H), 2.13 (s, 3H), 2.08 – 2.02 (m, 1H), 2.00 (s, 3H), 1.40 – 1.25 (m, 4H), 1.10 – 1.02 (m, 2H), 0.95 (d, *J* = 6.3 Hz, 3H) ppm; ^{13}C NMR (151 MHz, $CDCl_3$) δ = 206.5, 171.5, 169.4, 168.2, 168.1, 168.0, 167.6, 167.5, 167.3, 164.2, 163.8, 139.5, 139.4, 139.3, 139.19, 139.15, 139.09, 139.07, 138.93, 138.89, 138.85, 138.8, 138.6, 138.53, 138.46, 138.4, 138.35, 138.28, 138.25, 138.24, 138.16, 138.13, 138.10, 138.07, 138.02, 137.99, 137.9, 133.9, 133.6, 133.5, 133.3, 133.0, 132.7, 131.9, 131.7, 131.6, 129.8, 129.7, 129.44, 129.36, 129.2, 128.9, 128.8, 128.7, 128.6, 128.52, 128.50, 128.48, 128.45, 128.4, 128.3, 128.24, 128.21, 128.15, 128.12, 128.09, 128.07, 127.97, 127.95, 127.92, 127.90, 127.88, 127.84, 127.81, 127.80, 127.74, 127.73, 127.71, 127.69, 127.59, 127.56, 127.54, 127.52, 127.45, 127.43, 127.39, 127.34, 127.26, 127.23, 127.21, 127.18, 127.1, 127.0, 126.9, 126.8, 126.7, 125.4, 123.5, 123.4, 123.3, 123.2, 123.0, 101.8, 100.9, 100.4, 100.02, 99.99, 99.5, 99.0, 98.1, 97.8, 97.1, 96.7, 95.9, 81.6, 81.2, 80.5, 80.2, 79.8, 79.2, 78.9, 78.8, 78.2, 77.9, 77.7, 77.3, 77.0, 76.7, 76.65, 76.61, 76.4, 76.3, 76.1, 75.9, 75.1, 75.00, 74.97, 74.9, 74.8, 74.7, 74.6, 74.54, 74.51, 74.23, 74.19, 74.0, 73.9, 73.8, 73.71, 73.68, 73.65, 73.6, 73.53, 73.50, 73.41, 73.39, 73.35, 73.3, 73.03, 72.98, 72.9, 72.8, 72.7,

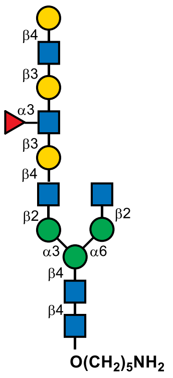

G4 (A12)

72.6, 72.5, 72.1, 72.0, 71.9, 71.8, 71.4, 70.7, 70.5, 70.4, 70.0, 69.80, 69.76, 68.9, 68.7, 68.5, 68.2, 68.0, 67.7, 67.6, 66.9, 66.5, 56.7, 56.1, 55.8, 55.7, 55.2, 51.2, 37.8, 29.9, 28.8, 28.4, 27.9, 23.1, 21.1, 16.1 ppm. **G4**: ^{1}H NMR (600 MHz, D_2O) δ = 5.15 – 5.08 (m, 2H), 4.92 (s, 1H), 4.84 – 4.81 (m, 1H), 4.79 (s, 1H), 4.73 – 4.66 (m, 2H), 4.62 – 4.53 (m, 3H), 4.52 – 4.41 (m, 4H), 4.25 (br s, 1H), 4.19 (d, *J* = 2.9 Hz, 1H), 4.16 (d, *J* = 3.1 Hz, 1H), 4.13 – 4.08 (m, 2H), 4.02 – 3.40 (m, 72H), 2.98 (apparent t, *J* = 7.6 Hz, 2H), 2.08 (s, 3H), 2.07 – 1.98 (m, 15H), 1.67 (apparent quint, *J* = 7.7 Hz, 2H), 1.59 (apparent quint, *J* = 6.6 Hz, 2H), 1.43 – 1.36 (m, 2H), 1.15 (d, *J* = 6.5 Hz, 3H) ppm; ^{13}C NMR (151 MHz, D_2O) δ = 174.84, 174.77, 174.7, 174.6, 174.4, 102.9, 102.8, 102.7, 102.5, 101.7, 101.4, 101.0, 100.4, 99.5, 99.4, 98.7, 97.0, 82.1, 81.6, 80.4, 79.5, 79.3, 78.5, 78.2, 76.4, 76.3, 75.8, 75.3, 75.1, 74.8, 74.72, 74.69, 74.5, 74.4, 74.34, 74.29, 73.5, 73.3, 72.81, 72.76, 72.5, 72.4, 72.1, 71.9, 71.8, 70.9, 70.5, 70.2, 70.1, 69.93, 69.87, 69.43, 69.35, 69.2, 68.5, 68.2, 67.6, 67.32, 67.26, 66.7, 65.9, 65.7, 61.7, 61.6, 61.4, 61.0, 60.9, 60.6, 60.1, 59.94, 59.89, 59.8, 59.6, 55.3, 55.1, 55.0, 54.9, 54.8, 39.3, 28.0, 26.3, 22.304, 22.295, 22.22, 22.18, 22.13, 22.11, 22.07, 15.2 ppm. LRMS (ESI-MS) *m/z* calculated for $C_{95}H_{159}N_7O_{65}^{2-}$ [M-2H]$^{2-}$: 1219.5, found: 1219.4.

5-Aminopentyl 2-acetamido-2-deoxy-β-D-glucopyranosyl-(1→3)-β-D-galactopyranosyl-(1→4)-2-acetamido-2-deoxy-β-D-glucopyranosyl-(1→3)-β-D-galactopyranosyl-(1→4)-2-acetamido-2-deoxy-β-D-glucopyranosyl-(1→2)-α-D-mannopyranosyl-(1→3)-[β-D-galactopyranosyl-(1→4)-[α-L-fucopyranosyl-(1→3)]-2-acetamido-2-deoxy-β-D-glucopyranosyl-(1→3)-β-D-galactopyranosyl-(1→4)-2-acetamido-2-deoxy-β-D-glucopyranosyl-(1→2)-α-D-mannopyranosyl-(1→6)]-β-D-mannopyranosyl-(1→4)-2-acetamido-2-deoxy-β-D-glucopyranosyl-(1→4)-2-acetamido-2-deoxy-β-D-glucopyranoside (G5): asymmetric bi-antennary *N*-glycan **G5** was synthesized from the bi-antennary precursor **1** and *o*-alkynylbenzoates **Module E** and **Module D** following General Procedures VIII, III, VII, III, XI and XII sequentially. **G5** (4.5 mg, 63% overall from 17.0 mg pentadecasaccharide intermediate) was acquired from P2 purification and lyophilization as a fluffy white solid. **The pentadecasaccharide intermediate**: ^{1}H NMR (600 MHz, $CDCl_3$) δ = 7.82 – 6.64 (m, 202H), 6.56 (apparent t, *J* = 7.5 Hz, 2H), 6.51 (d, *J* = 6.7 Hz, 1H), 6.30 (apparent t, *J* = 7.1 Hz, 1H), 6.25 (apparent t, *J* = 7.4 Hz, 2H), 5.34 – 5.22 (m, 4H), 5.20 (d, *J* = 8.3 Hz, 1H), 5.15 – 5.09 (m, 3H), 5.08 – 5.03 (m, 1H), 5.01 (d, *J* = 11.7 Hz, 1H), 4.98 (d, *J* = 11.8 Hz, 1H), 4.94 (d, *J* = 7.4 Hz, 1H), 4.90 (br d, *J* = 6.9 Hz, 1H), 4.85 (d, *J* = 10.7 Hz, 1H), 4.82 – 4.64 (m, 10H), 4.63 – 4.19 (m, 50H), 4.18 – 3.87 (m, 30H), 3.86 – 3.73 (m, 10H), 3.73 – 3.04 (m, 49H), 3.01 (br d, *J* = 10.0 Hz, 1H), 2.92 (br d, *J* = 10.5 Hz, 1H), 2.90 – 2.81 (m, 2H), 2.81 – 2.71 (m, 2H), 2.71 – 2.59 (m, 4H), 2.52 – 2.44 (m, 1H), 2.43 – 2.35 (m, 1H), 2.14 (s, 3H), 2.10 (br d, *J* = 10.2 Hz, 1H), 2.06 – 2.00 (m, 4H), 1.40 – 1.25 (m, 4H), 1.12 – 0.99 (m, 5H) ppm; ^{13}C NMR (151 MHz, $CDCl_3$) δ = 206.3, 171.8, 169.0, 168.1, 167.5, 164.3, 164.2, 139.4, 139.3, 139.24, 139.19, 139.14, 139.07, 139.0, 138.9, 138.8, 138.7, 138.6, 138.51, 138.50, 138.4, 138.32, 138.29, 138.28, 138.24, 138.18, 138.1, 138.03, 137.99, 137.9, 137.8, 133.5, 133.2, 133.1, 132.9, 131.93, 131.87, 131.8, 131.7, 131.4, 131.0, 129.8, 129.54,

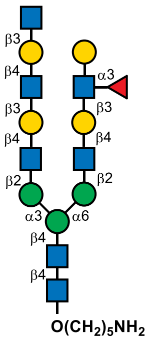

G5 (A19)

129.46, 128.9, 128.8, 128.7, 128.63, 128.61, 128.57, 128.55, 128.52, 128.48, 128.47, 128.45, 128.41, 128.40, 128.30, 128.29, 128.27, 128.24, 128.22, 128.21, 128.16, 128.14, 128.12, 128.10, 128.06, 128.03, 127.99, 127.97, 127.94, 127.91, 127.88, 127.84, 127.76, 127.75, 127.73, 127.68, 127.65, 127.63, 127.61, 127.56, 127.52, 127.50, 127.47, 127.42, 127.39, 127.36, 127.35, 127.30, 127.26, 127.24, 127.18, 127.12, 127.10, 127.07, 127.0, 126.9, 126.71, 126.68, 126.66, 125.4, 123.5, 123.3, 123.0, 101.8, 100.8, 100.63, 100.59, 100.2, 100.0, 99.9, 99.6, 99.5, 99.0, 98.1, 97.7, 97.2, 96.9, 96.8, 81.6, 80.9, 80.7, 80.5, 80.2, 79.9, 79.4, 78.7, 78.2, 77.7, 77.6, 76.8, 76.7, 76.65, 76.57, 76.5, 76.3, 76.2, 75.5, 75.3, 75.1, 75.0, 74.9, 74.8, 74.7, 74.63, 74.57, 74.54, 74.48, 74.43, 74.37, 74.2, 74.1, 73.95, 73.87, 73.64, 73.62, 73.59, 73.57, 73.52, 73.49, 73.44, 73.42, 73.38, 73.3, 73.01, 72.98, 72.89, 72.87, 72.74, 72.73, 72.6, 72.5, 72.4, 72.2, 72.11, 72.06, 72.0, 71.9, 71.7, 70.7, 70.6, 70.5, 70.0, 69.7, 69.6, 69.0, 68.71, 68.69, 68.6, 68.3, 67.7, 67.6, 66.6, 56.9, 56.6, 56.0, 55.8, 55.7, 55.6, 51.2, 37.8, 29.9, 28.8, 28.4, 28.0, 23.1, 21.1, 16.3 ppm. **G5**: ^{1}H NMR (600 MHz, D_2O) δ = 5.14 (d, *J* = 4.1 Hz, 1H), 5.12 (br s, 1H), 4.93 (br s, 1H), 4.84 (q, *J* = 6.8 Hz, 1H), 4.77 (br s, 1H), 4.73 – 4.67 (m, 3H), 4.63 – 4.54 (m, 3H), 4.53 – 4.42 (m, 5H), 4.25 (d, *J* = 2.8 Hz, 1H), 4.20 (d, *J* = 3.8 Hz, 1H), 4.18 – 4.13 (m, 3H), 4.11 (d, *J* = 3.4 Hz, 1H), 4.03 – 3.40 (m, 83H), 2.99 (apparent t, *J* = 7.6 Hz, 2H), 2.09 (s, 3H), 2.07 – 2.01 (m, 18H), 1.67 (apparent quint, *J* = 7.7 Hz, 2H), 1.60 (apparent quint, *J* = 6.6 Hz, 2H), 1.44 – 1.36 (m, 2H), 1.18 (d, *J* = 6.6 Hz, 3H) ppm; ^{13}C NMR (151 MHz, D_2O) δ = 174.94, 174.89, 174.8, 174.70, 174.68, 174.6, 174.4, 103.0, 102.94, 102.87, 102.8, 102.7, 102.6, 101.8, 101.4, 101.0, 100.4, 99.54, 99.45, 99.4, 98.6, 97.0, 82.3, 82.1, 82.04, 81.97, 79.4, 79.3, 78.5, 78.1, 76.4, 76.3, 75.6, 75.1, 74.90, 74.87, 74.8, 74.74, 74.70, 74.6, 74.52, 74.49, 74.4, 73.5, 73.0, 72.5, 72.4, 72.2, 72.15, 72.06, 72.0, 71.9, 71.0, 70.1, 70.0, 69.9, 69.7, 69.6, 69.43, 69.39, 69.2, 68.3, 67.7, 67.3, 66.7, 61.7, 61.6, 61.5, 60.9, 60.8, 60.4, 60.1, 59.9, 59.8, 59.6, 55.9, 55.6, 55.1, 55.0, 54.9, 54.8, 39.3, 28.0, 26.4, 22.3, 22.24, 22.21, 22.15, 22.14, 22.11, 22.07, 15.3 ppm. LRMS (ESI-MS) *m*/*z* calculated for $C_{109}H_{182}N_8O_{75}^{2-}$ $[M-2H]^{2-}$: 1402.0, found: 1401.9.

5-Aminopentyl 2-acetamido-2-deoxy-β-D-glucopyranosyl-(1→3)-β-D-galactopyranosyl-(1→4)-2-acetamido-2-deoxy-β-D-glucopyranosyl-(1→3)-β-D-galactopyranosyl-(1→4)-2-acetamido-2-deoxy-β-D-glucopyranosyl-(1→3)-β-D-galactopyranosyl-(1→4)-2-acetamido-2-deoxy-β-D-glucopyranosyl-(1→3)-β-D-galactopyranosyl-(1→4)-2-acetamido-2-deoxy-β-D-glucopyranosyl-(1→2)-α-D-mannopyranosyl-(1→3)-[2-acetamido-2-deoxy-β-D-glucopyranosyl-(1→3)-β-D-galactopyranosyl-(1→4)-2-acetamido-2-deoxy-β-D-glucopyranosyl-(1→3)-β-D-galactopyranosyl-(1→4)-2-acetamido-2-deoxy-β-D-glucopyranosyl-(1→2)-α-D-mannopyranosyl-(1→6)]-β-D-mannopyranosyl-(1→4)-2-acetamido-2-deoxy-β-D-glucopyranosyl-(1→4)-2-acetamido-2-deoxy-β-D-glucopyranoside (G6): asymmetric bi-antennary *N*-glycan **G6** was synthesized from the bi-antennary precursor **1** and *o*-alkynylbenzoates **Module E** and **Module I** following General Procedures VII, III, VIII, III, XI and XII sequentially. **G6** (9.6 mg, 59% overall from 39.0 mg nonadecasaccharide intermediate) was acquired from P2 purification and lyophilization as a fluffy white solid. **The nonadecasaccharide intermediate**:^{1}H NMR (600 MHz, $CDCl_3$) δ = 7.82 – 6.44 (m, 267H), 6.23 (apparent t,

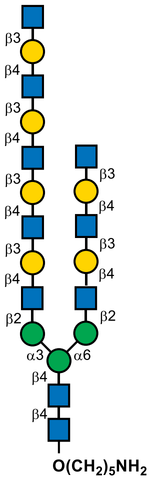

G6 (A2)

J = 7.7 Hz, 1H), 6.21 – 6.15 (m, 2H), 5.39 – 5.19 (m, 8H), 5.18 – 4.86 (m, 15H), 4.86 – 4.67 (m, 11H), 4.63 – 3.77 (m, 108H), 3.76 – 3.04 (m, 66H), 3.02 (br d, *J* = 9.8 Hz, 1H), 2.95 – 2.73 (m, 4H), 2.72 – 2.58 (m, 6H), 2.55 – 2.45 (m, 2H), 2.45 – 2.34 (m, 2H), 2.15 (s, 6H), 2.06 – 2.00 (m, 1H), 1.99 – 1.91 (m, 1H), 1.40 – 1.21 (m, 4H), 1.13 – 1.00 (m, 2H) ppm; ^{13}C NMR (151 MHz, $CDCl_3$) δ = 206.3, 171.8, 168.1, 167.8, 167.5, 167.1, 164.4, 164.3, 164.23, 164.20, 139.4, 139.32, 139.27, 139.2, 139.08, 139.07 139.0, 138.88, 138.86, 138.8, 138.6, 138.54, 138.50, 138.49, 138.38, 138.36, 138.33, 138.29, 138.2, 138.10, 138.07, 138.0, 137.9, 131.8, 131.5, 130.9, 129.8, 129.7, 129.5, 129.4, 128.8, 128.65, 128.63, 128.54, 128.52, 128.50, 128.43, 128.40, 128.37, 128.35, 128.32, 128.27, 128.25, 128.22, 128.20, 128.16, 128.14, 128.13, 128.10, 128.08, 128.06, 128.00, 127.98, 127.96, 127.93, 127.87, 127.85, 127.83, 127.79, 127.73, 127.72, 127.70, 127.67, 127.64, 127.60, 127.56, 127.55, 127.52, 127.50, 127.40, 127.38, 127.3, 127.2, 126.9, 126.68, 126.67, 126.6, 125.4, 123.4, 123.2, 122.7, 100.8, 100.7, 100.6, 99.6, 99.5, 98.1, 80.4, 80.2, 76.8, 76.6, 76.5, 76.2, 75.0, 74.9, 74.7, 74.5, 74.45, 74.41, 74.1, 73.90, 73.86, 73.6, 73.5, 73.42, 73.39, 73.3, 73.0, 72.9, 72.7, 72.4, 72.12, 72.09, 72.0, 71.9, 70.0, 69.0, 68.64, 68.58, 68.5, 67.8, 67.6, 55.9, 55.8, 51.2, 37.8, 29.9, 28.8, 28.4, 28.0, 23.1 ppm. **G6**: ^{1}H NMR (600 MHz, D_2O) δ = 5.12 (s, 1H), 4.93 (s, 1H), 4.78 (s, 1H), 4.72 – 4.65 (m, 6H), 4.63 – 4.54 (m, 3H), 4.53 – 4.40 (m, 7H), 4.28 – 4.07 (m, 9H), 4.04 – 3.39 (m, 107H), 2.99 (apparent t, *J* = 7.7 Hz, 2H), 2.11 – 1.98 (m, 30H), 1.67 (apparent quint, *J* = 7.7 Hz, 2H), 1.59 (apparent quint, *J* = 6.6 Hz, 2H), 1.45 – 1.35 (m, 2H) ppm; ^{13}C NMR (151 MHz, D_2O) δ = 174.93, 174.88, 174.7, 174.6, 174.4, 103.0, 102.9, 102.8, 102.7, 101.4, 101.0, 100.4, 99.6, 99.4, 97.0, 82.04, 81.96, 79.3, 78.5, 78.1, 76.4, 76.3, 75.8, 75.6, 74.9, 74.7, 74.52, 74.49, 74.4, 73.5, 72.8, 72.4, 72.2, 70.088, 70.087, 70.0, 69.7, 68.3, 67.3, 60.9, 60.5, 60.1, 59.9, 59.8, 55.6, 55.1, 39.3, 28.0, 26.3, 22.3, 22.21, 22.17, 22.15, 22.13, 22.08 ppm. LRMS (ESI-MS) *m*/*z* calculated for $C_{139}H_{230}N_{11}O_{96}^{3-}$ $[M-3H]^{3-}$: 1196.8, found: 1196.8.

Scheme S11. Assembly of Asymmetric Tri-antennary *N*-Glycans from Precursor 2 and *O*-Alkynylbenzoate Modules A-I[a]

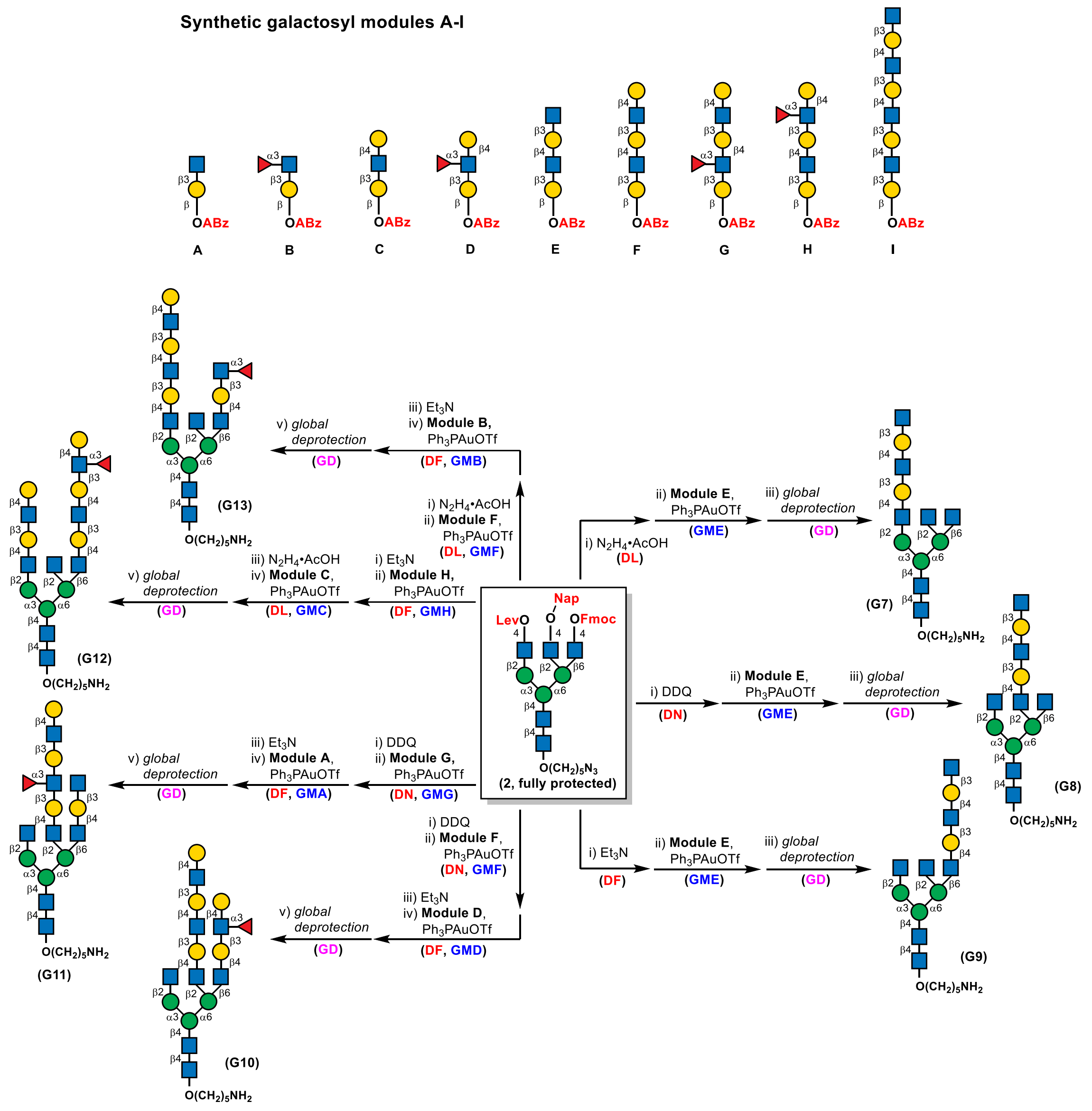

[a]Reagents and conditions: (**DL**) $N_2H_4{\cdot}AcOH$ (3.0 equiv.), CH_2Cl_2:MeOH (20:1, *v*/*v*), r.t., 3 hrs; (**DN**) DDQ (2.0 equiv.), CH_2Cl_2:H_2O (10:1, *v*/*v*), 0 °C, 3 hrs; (**DF**) CH_2Cl_2:Et_3N (10:1, *v*/*v*), r.t., 12 hrs; (**GMX**) **Module X** (X = A, B, C, D, E, F, G, H) (2.0 equiv.), $Ph_3PAuOTf$ (1.0 equiv.), 4Å MS, CH_2Cl_2, 0 °C, 30 min; (**GD**) i) EDA:*n*-BuOH (1:4, *v*/*v*), 90 °C, 18 hrs; ii) pyridine:Ac_2O (2:1, *v*/*v*), r.t., 24 hrs; iii) NaOMe (final concentration: 0.2 M), MeOH, r.t., 12 hrs; iv) $Pd(OH)_2/C$, H_2, THF:H_2O:*t*-BuOH (6:3:1, *v*/*v*/*v*), 3-12 hrs.

5-Aminopentyl 2-acetamido-2-deoxy-β-D-glucopyranosyl-(1→3)-β-D-galactopyranosyl-(1→4)-2-acetamido-2-deoxy-β-D-glucopyranosyl-(1→3)-β-D-galactopyranosyl-(1→4)-2-acetamido-2-deoxy-β-D-glucopyranosyl-(1→2)-α-D-mannopyranosyl-(1→3)-[2-acetamido-2-deoxy-β-D-glucopyranosyl-(1→2)-[2-acetamido-2-deoxy-β-D-glucopyranosyl-(1→6)]-α-D-mannopyranosyl-(1→6)]-β-D-mannopyranosyl-(1→4)-2-acetamido-2-deoxy-β-D-glucopyranosyl-(1→4)-2-acetamido-2-deoxy-β-D-glucopyranoside (G7): asymmetric tri-antennary *N*-glycan **G7** was synthesized from the tri-antennary precursor **2** and *o*-alkynylbenzoate **Module E** following General Procedures VII, III, XI and XII. An exemplified procedure was

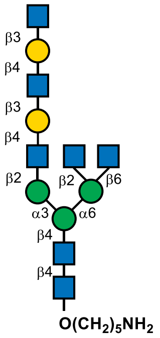

G7 (A1)

as follows: to a solution of fully protected octasaccharide **2** (300 mg, 77.7 μmol, 1.0 equiv.) in CH_2Cl_2 (10 mL) at room temperature was added a solution of $N_2H_4·AcOH$ in MeOH (0.466 mL, *ca.* 0.5 M, 0.233 mmol, 3.0 equiv.). The reaction was stirred at the same temperature for another 3 hrs before it was quenched by H_2O (10 mL) and diluted by CH_2Cl_2 (10 mL). The organic phase was separated and the aqueous phase was extracted by CH_2Cl_2 (10 mL × 3). The organic phases were combined, dried over Na_2SO_4, and concentrated under vacuum. The resulting residue was briefly purified by flash chromatography (silica gel, EtOAc:hexanes = 1:4 → 1:1, *v*/*v*) to afford secondary alcohol intermediate (263 mg, 90%) which was directly used for the pending glycosylation reactions. To a suspension of so-obtained acceptor (100 mg, 25.8 μmol, 1.0 equiv.), tetrasaccharide donor (**Module E**, 109 mg, 51.6 μmol, 2.0 equiv.) and 4Å MS 300 mg, flame dried) in CH_2Cl_2 (3.0 mL) at 0 °C was slowly added a solution of $Ph_3PAuOTf$ in CH_2Cl_2 (0.516 mL, *ca.* 0.05 M, 25.8 μmol, 1.0 equiv.). The reaction suspension was stirred at the same temperature for another 30 min before it was directly subjected to a flash column (silica gel, EtOAc:hexanes = 1:3 → 1:1, *v*/*v*), which provided the fully protected dodecasaccharide intermediate (108 mg, 72%) as a pale yellow foam. ^{1}H NMR (600 MHz, $CDCl_3$) δ = 7.92 – 6.50 (m, 173H), 5.36 – 5.27 (m, 2H), 5.24 (d, *J* = 8.3 Hz, 1H), 5.16 (d, *J* = 8.3 Hz, 1H), 5.09 (apparent t, *J* = 9.5 Hz, 1H), 5.06 – 4.96 (m, 4H), 4.96 – 4.66 (m, 12H), 4.66 – 4.21 (m, 41H), 4.21 – 3.90 (m, 27H), 3.89 – 3.79 (m, 4H), 3.79 – 3.21 (m, 35H), 3.21 – 3.11 (m, 4H), 3.06 (br t, *J* = 11.4 Hz, 2H), 2.96 (br d, *J* = 9.7 Hz, 1H), 2.93 – 2.78 (m, 3H), 2.73 – 2.47 (m, 6H), 2.46 – 2.37 (m, 2H), 2.16 (s, 3H), 1.44 – 1.20 (m, 4H), 1.13 – 0.99 (m, 2H) ppm; ^{13}C NMR (151 MHz, $CDCl_3$) δ = 206.2, 171.8, 168.0, 167.8, 167.6, 167.1, 166.9, 164.33, 164.26, 154.5, 143.5, 143.2, 141.45, 141.40, 139.3, 139.2, 139.1, 138.94, 138.87, 138.85, 138.79, 138.7, 138.5, 138.43, 138.36, 138.3, 138.23, 138.20, 138.14, 138.08, 138.01, 137.99, 137.96, 137.9, 137.7, 136.3, 134.0, 133.9, 133.8, 133.7, 133.5, 133.4, 133.3, 133.2, 133.0, 132.9, 132.8, 131.9, 131.8, 131.5, 131.4, 131.3, 130.9, 129.8, 129.6, 129.5, 129.3, 128.8, 128.6, 128.52, 128.50, 128.4, 128.35, 128.29, 128.25, 128.23, 128.19, 128.18, 128.15, 128.13, 128.10, 128.03, 127.98, 127.95, 127.91, 127.89, 127.87, 127.85, 127.81, 127.78, 127.76, 127.73, 127.68, 127.66, 127.63, 127.59, 127.58, 127.55, 127.52, 127.50, 127.48, 127.46, 127.42, 127.38, 127.35, 127.32, 127.29, 127.28, 127.20, 127.16, 127.04, 126.97, 126.9, 126.7, 126.6, 126.2, 126.1, 125.95, 125.93, 125.2, 125.0, 123.5, 123.3, 123.15, 123.08, 122.7, 120.2, 102.1, 100.62, 100.56, 99.6, 99.5, 98.64, 98.56, 98.1, 97.1, 96.93, 96.87, 96.4, 81.3, 80.5, 80.2, 80.1, 79.3, 79.1, 78.0, 77.9, 77.7, 77.4, 77.3, 77.1, 76.83, 76.77, 76.6, 76.5, 76.4, 76.2, 74.9, 74.85, 74.78, 74.7,

74.64, 74.61, 74.60, 74.54, 74.51, 74.48, 74.2, 74.1, 73.9, 73.8, 73.7, 73.61, 73.57, 73.55, 73.54, 73.49, 73.45, 73.4, 73.3, 73.2, 73.0, 72.72, 72.71, 72.6, 72.2, 72.1, 71.8, 70.6, 70.3, 70.1, 70.0, 69.9, 69.6, 69.5, 68.9, 68.7, 68.6, 68.2, 67.8, 67.72, 67.65, 66.0, 65.5, 56.8, 55.9, 55.85, 55.80, 55.6, 55.3, 51.2, 46.8, 37.8, 29.9, 28.7, 28.3, 28.0, 23.1 ppm. A solution of so-obtained fully protected dodecasaccharide (37.0 mg, 6.4 μmol) in a mixed solvent *n*-BuOH/EDA (5.0 mL, 4:1, *v*/*v*) was stirred at 90 °C for 18 hrs before it was cooled back to room temperature. All the volatiles were azeotropically removed under vacuum, and the residue was dissolved in pyridine/Ac_2O (3.0 mL, 2:1, *v*/*v*). The reaction mixture was stirred at room temperature for 24 hrs and was then dried azeotropically with PhMe (10 mL × 3). The residue was dissolved in MeOH (3.0 mL) and NaOMe (0.3 mL, *ca.* 5.4 M in MeOH, final concentration: 0.5 M) was added at room temperature. The reaction was stirred for another 24 hrs and was neutralized by Amberlite™ IR-120 (washed by MeOH). The suspension was filtered and concentrated under vacuum, and the residue was purified briefly by flash chromatography (silica gel, PhMe:acetone = 4:1 → 1:1, *v*/*v*). The resulting penta-ol (14.0 mg) was subjected to the final hydrogenolysis using H-Cube® Mini (*cf.* General Procedure XII) and P2 purification followed by lyophilization afforded undecasaccharide **G7** (9.0 mg, 56% overall from 40 mg fully protected dodecasaccharide intermediate) as a fluffy white solid. **G7**: ^{1}H NMR (600 MHz, D_2O) δ = 5.13 (s, 1H), 4.89 – 4.86 (m, 1H), 4.78 (s, 1H), 4.72 – 4.67 (m, 2H), 4.63 – 4.52 (m, 4H), 4.52 – 4.42 (m, 3H), 4.25 (d, *J* = 3.0 Hz, 1H), 4.22 – 4.18 (m, 2H), 4.16 (d, *J* = 3.3 Hz, 2H), 4.10 (d, *J* = 4.5 Hz, 1H), 4.02 – 3.39 (m, 68H), 2.98 (apparent t, *J* = 7.7 Hz, 2H), 2.11 – 2.00 (m, 21H), 1.67 (apparent quint, *J* = 7.7 Hz, 2H), 1.62 – 1.56 (m, 2H), 1.44 – 1.36 (m, 2H) ppm; ^{13}C NMR (151 MHz, D_2O) δ = 174.9, 174.7, 174.63, 174.59, 174.4, 174.2, 102.94, 102.86, 102.8, 102.7, 101.6, 101.4, 101.0, 100.4, 99.7, 99.52, 99.47, 97.2, 82.03, 81.96, 80.3, 79.4, 79.3, 78.5, 78.1, 76.5, 76.4, 75.80, 75.78, 75.6, 74.9, 74.8, 74.7, 74.52, 74.49, 74.44, 74.35, 73.7, 73.5, 73.3, 72.4, 72.2, 72.0, 71.9, 71.6, 70.1, 70.0, 69.9, 69.7, 69.4, 69.3, 68.3, 67.5, 67.3, 65.6, 65.5, 61.7, 60.93, 60.92, 60.8, 60.6, 60.4, 60.1, 59.9, 59.8, 55.63, 55.55, 55.4, 55.1, 55.0, 54.9, 54.8, 39.3, 28.0, 26.3, 22.5, 22.3, 22.20, 22.16, 22.13, 22.11, 22.1 ppm. LRMS (ESI-MS) *m*/*z* calculated for $C_{91}H_{152}N_8O_{61}^{2-}$ [M-2H]$^{2-}$: 1166.5, found: 1166.6.

5-Aminopentyl 2-acetamido-2-deoxy-β-D-glucopyranosyl-(1→2)-α-D-mannopyranosyl-(1→3)-[2-acetamido-2-deoxy-β-D-glucopyranosyl-(1→3)-β-D-galactopyranosyl-(1→4)-2-acetamido-2-deoxy-β-D-glucopyranosyl-(1→3)-β-D-galactopyranosyl-(1→4)-2-acetamido-2-deoxy-β-D-glucopyranosyl-(1→2)-[2-acetamido-2-deoxy-β-D-glucopyranosyl-(1→6)]-α-D-mannopyranosyl-(1→6)]-β-D-mannopyranosyl-(1→4)-2-acetamido-2-deoxy-β-D-glucopyranosyl-(1→4)-2-acetamido-2-deoxy-β-D-glucopyranoside (G8): similar to **G7**, asymmetric tri-antennary *N*-glycan **G8** was synthesized from the tri-antennary precursor **2** and *o*-alkynylbenzoate **Module E** following General Procedures IX, III, XI and XII sequentially. **G8** (12.3 mg, 61%

overall from 50.0 mg dodecasaccharide intermediate) was acquired from P2 purification and lyophilization as a fluffy white solid. **The dodecasaccharide intermediate**: ^{1}H NMR (600 MHz, $CDCl_3$) δ = 7.86 – 6.69 (m, 161H), 6.63 (d, *J* = 7.2 Hz, 2H), 6.38 – 6.29 (m, 3H), 5.37 (d, *J* = 8.3 Hz, 1H), 5.34 – 5.28 (m, 1H), 5.28 – 5.20 (m, 2H), 5.17 (apparent t, *J* = 9.6 Hz, 2H), 5.11 – 4.91 (m, 6H), 4.90 – 4.69 (m, 6H), 4.68 – 3.17 (m, 109H), 3.16 – 3.02 (m, 5H), 2.95 – 2.83 (m, 3H), 2.79 (br t, *J* = 8.9 Hz, 1H), 2.71 – 2.60 (m, 2H), 2.58 – 2.32 (m, 8H), 2.28 (br d, *J* = 9.5 Hz, 1H), 2.21 – 2.16 (m, 1H), 2.15 (s, 3H), 2.08 (s, 3H), 1.44 – 1.24 (m, 4H), 1.14 – 1.03 (m, 2H) ppm; ^{13}C NMR (151 MHz, $CDCl_3$) δ = 206.3, 206.2, 171.8, 168.2, 167.6, 167.5, 167.2, 164.35, 164.26, 154.4, 143.5, 143.2, 141.44, 141.39, 139.4, 139.15, 139.10, 139.07, 138.9, 138.8, 138.6, 138.5, 138.35, 138.33, 138.31, 138.2, 138.1, 138.04, 138.02, 137.99, 137.9, 137.7, 134.1, 134.0, 133.7, 133.6, 133.3, 133.1, 132.9, 131.9, 131.8, 131.7, 131.4, 131.1, 130.9, 129.8, 129.6, 129.5, 129.4, 128.8, 128.6, 128.53, 128.51, 128.48, 128.40, 128.37, 128.34, 128.28, 128.26, 128.23, 128.21, 128.13, 128.09, 128.05, 128.03, 128.01, 127.98, 127.93, 127.92, 127.89, 127.86, 127.83, 127.82, 127.77, 127.75, 127.72, 127.68, 127.65, 127.62, 127.60, 127.58, 127.52, 127.49, 127.46, 127.44, 127.40, 127.37, 127.34, 127.28, 127.26, 127.2, 127.1, 127.0, 126.68, 126.66, 125.2, 125.0, 123.8, 123.5, 123.2, 123.0, 122.9, 122.8, 120.2, 101.9, 100.6, 100.5, 99.6, 99.5, 98.8, 98.7, 98.1, 97.2, 96.6, 96.4, 96.1, 80.8, 80.7, 80.2, 79.8, 78.0, 77.9, 77.5, 77.45, 77.42, 77.3, 77.1, 76.8, 76.7, 76.6, 76.5, 76.3, 76.2, 75.0, 74.9, 74.8, 74.73, 74.67, 74.6, 74.5, 74.43, 74.40, 74.15, 74.11, 74.08, 74.0, 73.88, 73.85, 73.8, 73.7, 73.64, 73.57, 73.56, 73.51, 73.50, 73.4, 73.3, 73.24, 73.19, 72.9, 72.8, 72.7, 72.6, 72.3, 72.1, 71.9, 71.6, 70.7, 70.6, 70.3, 70.0, 69.93, 69.85, 69.7, 69.0, 68.63, 68.56, 68.3, 67.6, 65.9, 56.8, 56.0, 55.8, 55.53, 55.49, 55.3, 51.2, 46.8, 37.81, 37.77, 29.9, 29.8, 28.8, 28.4, 28.0, 23.1 ppm. **G8**: ^{1}H NMR (600 MHz, D_2O) δ = 5.13 (s, 1H), 4.87 (s, 1H), 4.78 (s, 1H), 4.70 (apparent t, *J* = 9.4 Hz, 2H), 4.60 (apparent d, *J* = 7.8 Hz, 2H), 4.56 (d, *J* = 8.4 Hz, 1H), 4.53 (d, *J* = 8.3 Hz, 1H), 4.51 – 4.44 (m, 3H), 4.26 (br d, *J* = 2.2 Hz, 1H), 4.23 – 4.18 (m, 2H), 4.18 – 4.14 (m, 2H), 4.09 (br d, *J* = 2.9 Hz, 1H), 4.02 – 3.37 (m, 68H), 2.99 (apparent t, *J* = 7.7 Hz, 2H), 2.12 – 2.00 (m, 21H), 1.67 (apparent quint, *J* = 7.8 Hz, 2H), 1.63 – 1.54 (m, 2H), 1.46 – 1.36 (m, 2H) ppm. LRMS (ESI-MS) *m/z* calculated for $C_{91}H_{152}N_8O_{61}^{2-}$ $[M-2H]^{2-}$: 1166.5, found: 1166.7.

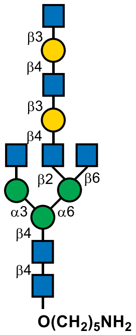

G8 (A5)

5-Aminopentyl 2-acetamido-2-deoxy-β-D-glucopyranosyl-(1→2)-α-D-mannopyranosyl-(1→3)-[2-acetamido-2-deoxy-β-D-glucopyranosyl-(1→2)-[2-acetamido-2-deoxy-β-D-glucopyranosyl-(1→3)-β-D-galactopyranosyl-(1→4)-2-acetamido-2-deoxy-β-D-glucopyranosyl-(1→3)-β-D-galactopyranosyl-(1→4)-2-acetamido-2-deoxy-β-D-glucopyranosyl-(1→6)]-α-D-mannopyranosyl-(1→6)]-β-D-mannopyranosyl-(1→4)-2-acetamido-2-deoxy-β-D-glucopyranosyl-(1→4)-2-acetamido-2-deoxy-β-D-glucopyranoside (G9): similar to **G7**, asymmetric tri-antennary *N*-glycan **G9** was synthesized from the tri-antennary precursor **2** and *o*-alkynylbenzoate **Module E** following General Procedures X, III, XI and XII sequentially. **G9** (11.5 mg, 54% overall from 52.0 mg dodecasaccharide intermediate) was acquired from P2 purification and lyophilization as a fluffy white solid. **The dodecasaccharide intermediate**: ^{1}H NMR (600 MHz, $CDCl_3$) δ = 7.93 – 6.46 (m,

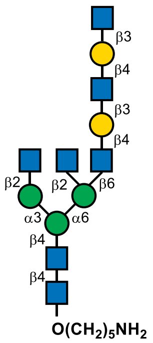

G9 (A6)

165H), 5.40 – 5.30 (m, 3H), 5.25 (d, J = 8.4 Hz, 1H), 5.22 – 5.14 (m, 2H), 5.13 – 5.00 (m, 4H), 4.97 – 4.86 (m, 4H), 4.85 – 4.66 (m, 8H), 4.65 – 3.94 (m, 59H), 3.92 – 3.78 (m, 8H), 3.78 – 3.21 (m, 33H), 3.18 (br t, J = 9.0 Hz, 2H), 3.12 – 2.95 (m, 4H), 2.95 – 2.80 (m, 3H), 2.74 – 2.47 (m, 8H), 2.47 – 2.25 (m, 5H), 2.23 – 2.11 (m, 5H), 2.09 (s, 3H), 1.99 (br d, J = 9.6 Hz, 1H), 1.46 – 1.24 (m, 4H), 1.17 – 1.02 (m, 2H) ppm; ^{13}C NMR (151 MHz, $CDCl_3$) δ = 206.2, 171.7, 171.6, 168.1, 167.8, 167.6, 167.5, 167.2, 167.1, 164.4, 164.3, 139.3, 139.1, 139.0, 138.9, 138.83, 138.79, 138.7, 138.4, 138.29, 138.25, 138.23, 138.20, 138.1, 138.03, 137.99, 137.9, 137.8, 136.2, 133.8, 133.7, 133.5, 133.3, 133.1, 132.94, 132.91, 131.9, 131.7, 131.6, 131.5, 131.4, 130.9, 129.8, 129.7, 129.5, 129.3, 128.8, 128.6, 128.50, 128.48, 128.38, 128.36, 128.35, 128.34, 128.30, 128.26, 128.24, 128.21, 128.19, 128.18, 128.10, 128.09, 128.05, 128.03, 128.02, 127.99, 127.97, 127.95, 127.94, 127.92, 127.89, 127.86, 127.83, 127.78, 127.695, 127.686, 127.62, 127.56, 127.53, 127.50, 127.47, 127.46, 127.40, 127.37, 127.33, 127.31, 127.2, 127.1, 127.0, 126.7, 126.6, 126.2, 126.1, 125.93, 125.89, 123.52, 123.47, 123.1, 123.0, 122.7, 102.1, 100.6, 100.4, 99.6, 99.5, 99.2, 98.7, 98.1, 97.0, 96.8, 96.5, 80.7, 80.3, 80.2, 80.0, 79.1, 79.0, 78.0, 77.8, 77.5, 77.0, 76.74, 76.69, 76.53, 76.46, 76.2, 76.0, 75.0, 74.8, 74.7, 74.63, 74.59, 74.56, 74.52, 74.49, 74.46, 74.4, 74.3, 74.2, 74.1, 73.9, 73.8, 73.69, 73.67, 73.54, 73.52, 73.50, 73.47, 73.4, 73.35, 73.34, 73.28, 73.1, 72.9, 72.7, 72.6, 72.3, 72.1, 72.0, 70.8, 70.6, 70.2, 70.0, 69.9, 69.8, 68.9, 68.7, 68.6, 68.4, 68.2, 67.7, 67.6, 67.1, 65.4, 65.2, 56.8, 55.9, 55.8, 55.53, 55.49, 55.4, 51.1, 37.8, 37.7, 29.8, 28.7, 28.3, 27.98, 27.95, 23.0 ppm. **G9**: ^{1}H NMR (600 MHz, D_2O) δ = 5.13 (s, 1H), 4.87 (s, 1H), 4.78 (s, 1H), 4.71 (d, J = 8.5 Hz, 1H), 4.69 (d, J = 8.6 Hz, 1H), 4.60 (d, J = 7.8 Hz, 1H), 4.58 – 4.54 (m, 3H), 4.50 (d, J = 7.8 Hz, 1H), 4.47 (d, J = 7.9 Hz, 2H), 4.26 (d, J = 3.0 Hz, 1H), 4.23 – 4.18 (m, 2H), 4.16 (d, J = 3.2 Hz, 2H), 4.10 (br d, J = 4.0 Hz, 1H), 4.03 – 3.39 (m, 68H), 3.03 – 2.96 (m, 2H), 2.11 – 1.99 (m, 21H), 1.68 (apparent quint, J = 7.7 Hz, 2H), 1.63 – 1.54 (m, 2H), 1.46 – 1.35 (m, 2H) ppm; ^{13}C NMR (151 MHz, D_2O) δ = 174.94, 174.89, 174.7, 174.63, 174.58, 174.4, 174.2, 102.89, 102.87, 102.8, 102.7, 101.6, 101.4, 101.0, 100.4, 99.73, 99.65, 99.6, 97.2, 82.01, 81.97, 80.3, 79.4, 79.3, 78.5, 78.2, 76.51, 76.47, 76.4, 75.80, 75.78, 75.6, 74.9, 74.7, 74.53, 74.49, 74.5, 74.4, 73.5, 73.3, 72.4, 72.3, 72.2, 71.9, 71.5, 70.3, 70.2, 70.1, 70.0, 69.95, 69.92, 69.88, 69.7, 69.4, 68.3, 67.5, 67.3, 65.6, 65.5, 61.7, 60.9, 60.6, 60.4, 60.1, 59.8, 55.6, 55.4, 55.3, 55.1, 55.02, 54.97, 54.9, 39.3, 28.0, 26.3, 22.5, 22.33, 22.31, 22.21, 22.16, 22.14, 22.11 ppm. LRMS (ESI-MS) m/z calculated for $C_{91}H_{152}N_8O_{61}^{2-}$ [M-2H]$^{2-}$: 1166.5, found: 1166.6.

5-Aminopentyl 2-acetamido-2-deoxy-β-D-glucopyranosyl-(1→2)-α-D-mannopyranosyl-(1→3)-[β-D-galactopyranosyl-(1→4)-2-acetamido-2-deoxy-β-D-glucopyranosyl-(1→3)-β-D-galactopyranosyl-(1→4)-2-acetamido-2-deoxy-β-D-glucopyranosyl-(1→3)-β-D-galactopyranosyl-(1→4)-2-acetamido-2-deoxy-β-D-glucopyranosyl-(1→2)-[β-D-galactopyranosyl-(1→4)-[α-L-fucopyranosyl-(1→3)]-2-acetamido-2-deoxy-β-D-glucopyranosyl-(1→3)-β-D-galactopyranosyl-(1→4)-2-acetamido-2-deoxy-β-D-glucopyranosyl-(1→6)]-α-D-mannopyranosyl-(1→6)]-β-D-mannopyranosyl-(1→4)-2-acetamido-2-deoxy-β-D-glucopyranosyl-(1→4)-2-acetamido-2-deoxy-β-D-glucopyranoside (G10): asymmetric tri-antennary *N*-glycan **G10** was

synthesized from the tri-antennary precursor **2** and *o*-alkynylbenzoates **Module F** and **Module D** following General Procedures IX, III, X, III, XI and XII sequentially. **G10** (4.1 mg, 58% overall from 17.0 mg heptadecasaccharide intermediate) was acquired from P2 purification and lyophilization as a fluffy white solid. **The heptadecasaccharide intermediate**: ^{1}H NMR (600 MHz, $CDCl_3$) δ = 7.81 – 6.67 (m, 222H), 6.64 – 6.54 (m, 2H), 6.33 – 6.11 (m, 3H), 5.40 – 5.09 (m, 11H), 5.06 (d, *J* = 11.6 Hz, 1H), 5.03 (d, *J* = 11.8 Hz, 1H), 4.96 – 4.74 (m, 10H), 4.73 – 3.16 (m, 155H), 3.16 – 3.07 (m, 5H), 3.02 (br d, *J* = 9.9 Hz, 2H), 2.98 – 2.76 (m, 5H), 2.65 (br d, *J* = 9.7 Hz, 1H), 2.59 – 2.46 (m, 2H), 2.40 – 2.28 (m, 3H), 2.07 (s, 3H), 2.04 (s, 3H), 2.01 (s, 3H), 1.99 – 1.89 (m, 2H), 1.44 – 1.25 (m, 4H), 1.12 – 1.02 (m, 5H) ppm; ^{13}C NMR (151 MHz, $CDCl_3$) δ = 206.3, 171.7, 169.4, 169.1, 168.22, 168.18, 167.8, 167.7, 167.61, 167.55, 167.2, 167.1, 164.5, 164.4, 164.2, 139.5, 139.39, 139.36, 139.3, 139.2, 139.1, 138.90, 138.88, 138.83, 138.79, 138.77, 138.6, 138.52, 138.51, 138.48, 138.42, 138.40, 138.37, 138.36, 138.3, 138.17, 138.15, 138.103, 138.095, 138.07, 138.03, 138.02, 138.01, 137.97, 137.9, 134.3, 134.0, 133.7, 133.6, 133.2, 132.9, 131.9, 131.7, 131.6, 131.5, 131.4, 129.8, 129.6, 129.5, 129.4, 129.3, 128.9, 128.8, 128.65, 128.63, 128.60, 128.59, 128.55, 128.53, 128.48, 128.43, 128.42, 128.41, 128.38, 128.31, 128.30, 128.28, 128.26, 128.25, 128.18, 128.16, 128.12, 128.08, 128.06, 128.04, 128.02, 127.99, 127.97, 127.95, 127.93, 127.90, 127.88, 127.86, 127.84, 127.82, 127.77, 127.75, 127.73, 127.68, 127.67, 127.63, 127.60, 127.56, 127.53, 127.48, 127.44, 127.42, 127.41, 127.38, 127.31, 127.26, 127.2, 127.13, 127.09, 127.06, 127.0, 126.9, 126.79, 126.75, 126.71, 126.67, 124.3, 123.6, 123.5, 123.3, 123.0, 122.8, 122.3, 102.1, 101.0, 100.8, 100.6, 100.5, 100.0, 99.9, 99.8, 99.6, 99.4, 98.8, 98.2, 97.2, 97.0, 96.9, 96.6, 96.3, 80.9, 80.8, 80.5, 80.4, 80.2, 79.4, 78.7, 78.1, 78.0, 77.7, 77.6, 77.1, 76.8, 76.7, 76.62, 76.56, 76.5, 76.3, 76.2, 76.1, 75.5, 75.4, 75.10, 75.07, 75.02, 74.97, 74.9, 74.8, 74.74, 74.71, 74.66, 74.62, 74.57, 74.6, 74.44, 74.37, 74.3, 74.2, 73.81, 73.77, 73.7, 73.6, 73.54, 73.51, 73.4, 73.3, 73.2, 73.02, 73.00, 72.9, 72.8, 72.74, 72.69, 72.6, 72.4, 72.2, 72.14, 72.08, 72.05, 71.9, 71.8, 71.7, 70.9, 70.8, 70.5, 70.3, 70.1, 69.9, 69.6, 69.0, 68.58, 68.57, 68.5, 68.34, 68.33, 68.1, 67.8, 67.64, 67.63, 67.1, 66.6, 65.5, 56.8, 56.1, 55.9, 55.6, 55.5, 51.2, 37.8, 29.9, 28.8, 28.4, 28.0, 23.1, 21.2, 21.1, 16.3 ppm. **G10**: ^{1}H NMR (600 MHz, D_2O) δ = 5.16 – 5.10 (m, 2H), 4.87 (s, 1H), 4.86 – 4.83 (m, 1H), 4.77 (s, 1H), 4.74 – 4.68 (m, 3H), 4.60 (d, *J* = 7.8 Hz, 2H), 4.58 – 4.53 (m, 2H), 4.52 – 4.43 (m, 6H), 4.26 (br s, 1H), 4.24 – 4.18 (m, 2H), 4.18 – 4.14 (m, 3H), 4.09 (d, *J* = 3.0 Hz, 1H), 4.04 – 3.35 (m, 94H), 2.99 (apparent t, *J* = 7.7 Hz, 2H), 2.11 – 1.99 (m, 24H), 1.68 (apparent quint, *J* = 7.7 Hz, 2H), 1.60 (apparent quint, *J* = 6.7 Hz, 2H), 1.45 – 1.37 (m, 2H), 1.18 (d, *J* = 6.6 Hz, 3H) ppm; ^{13}C NMR (151 MHz, D_2O) δ = 174.9, 174.75, 174.69, 174.6, 174.5, 174.4, 174.1, 102.89, 102.87, 102.85, 102.75, 102.74, 102.5, 101.8, 101.6, 101.4, 101.0, 100.3, 99.65, 99.60, 99.57, 98.6, 97.2, 82.1, 82.0, 79.3, 78.6, 78.5, 78.1, 76.5, 75.8, 75.3, 75.1, 74.90, 74.85, 74.7, 74.53, 74.49, 74.4, 73.54, 73.52, 73.3, 73.0, 72.5, 72.45, 72.37, 72.23, 72.16, 72.0, 71.9, 71.5, 71.0, 70.9, 70.2, 70.1, 69.94, 69.88, 69.4, 69.2, 68.5, 68.35, 68.33, 68.29, 67.7, 67.5, 67.3, 66.7, 65.6, 61.7, 61.5, 61.0, 60.94, 60.90, 60.6, 60.1, 60.0, 59.83, 59.81, 59.6, 55.9, 55.3, 55.2, 55.1, 55.00,

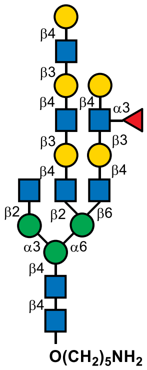

G10 (A10)

54.97, 54.9, 39.3, 28.0, 26.3, 22.5, 22.3, 22.24, 22.16, 22.15, 22.12, 22.07, 15.3 ppm. LRMS (ESI-MS) *m*/*z* calculated for $C_{123}H_{205}N_9O_{85}^{2-}$ $[M-2H]^{2-}$: 1584.6, found: 1584.9.

5-Aminopentyl 2-acetamido-2-deoxy-β-D-glucopyranosyl-(1→2)-α-D-mannopyranosyl-(1→3)-[β-D-galactopyranosyl-(1→4)-2-acetamido-2-deoxy-β-D-glucopyranosyl-(1→3)-β-D-galactopyranosyl-(1→4)-[α-L-fucopyranosyl-(1→3)]-2-acetamido-2-deoxy-β-D-glucopyranosyl-(1→3)-β-D-galactopyranosyl-(1→4)-2-acetamido-2-deoxy-β-D-glucopyranosyl-(1→2)-[2-acetamido-2-deoxy-β-D-glucopyranosyl-(1→3)-β-D-galactopyranosyl-(1→4)-2-acetamido-2-deoxy-β-D-glucopyranosyl-(1→6)]-α-D-mannopyranosyl-(1→6)]-β-D-mannopyranosyl-(1→4)-2-acetamido-2-deoxy-β-D-glucopyranosyl-(1→4)-2-acetamido-2-deoxy-β-D-glucopyranoside (G11): asymmetric tri-antennary *N*-glycan **G11** was synthesized from the tri-antennary precursor **2** and *o*-alkynylbenzoates **Module G** and **Module A** following General Procedures IX, III, X, III, XI and XII sequentially. **G11** (6.0 mg, 58% overall from 25.0 mg hexadecasaccharide intermediate) was acquired from P2 purification and lyophilization as a fluffy white solid. **The hexadecasaccharide intermediate**:

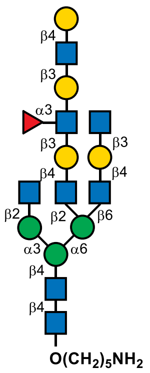

G11 (A11)

^{1}H NMR (600 MHz, $CDCl_3$) δ = 7.84 – 6.66 (m, 208H), 6.64 – 6.58 (m, 2H), 6.31 – 6.24 (m, 2H), 5.37 – 5.30 (m, 2H), 5.30 – 5.12 (m, 6H), 5.10 – 4.99 (m, 4H), 4.95 – 4.68 (m, 9H), 4.66 – 3.93 (m, 82H), 3.93 – 3.21 (m, 58H), 3.18 (br d, J = 10.0 Hz, 1H), 3.15 – 3.06 (m, 4H), 3.05 – 2.97 (m, 3H), 2.97 – 2.76 (m, 5H), 2.72 – 2.60 (m, 3H), 2.60 – 2.29 (m, 8H), 2.16 – 2.13 (m, 4H), 2.07 (s, 3H), 2.03 – 1.93 (m, 5H), 1.40 – 1.25 (m, 4H), 1.12 – 1.00 (m, 2H), 0.95 (br d, J = 6.3 Hz, 3H) ppm; ^{13}C NMR (151 MHz, $CDCl_3$) δ = 206.28, 206.27, 171.81, 171.77, 171.7, 169.4, 168.24, 168.18, 168.1, 168.0, 167.7, 167.61, 167.58, 167.0, 166.5, 164.5, 164.2, 163.8, 139.50, 139.48, 139.3, 139.2, 139.1, 138.9, 138.8, 138.7, 138.55, 138.52, 138.50, 138.4, 138.32, 138.30, 138.24, 138.15, 138.1, 138.05, 138.01, 137.97, 137.9, 134.3, 134.2, 134.0, 133.8, 133.7, 133.6, 133.3, 133.2, 133.0, 132.7, 132.1, 131.9, 131.7, 131.6, 131.5, 130.9, 130.0, 129.81, 129.76, 129.7, 129.43, 129.38, 129.3, 128.83, 128.75, 128.7, 128.54, 128.52, 128.49, 128.47, 128.44, 128.42, 128.40, 128.39, 128.31, 128.28, 128.25, 128.24, 128.13, 128.10, 128.07, 128.01, 127.98, 127.95, 127.93, 127.91, 127.88, 127.86, 127.83, 127.76, 127.74, 127.72, 127.63, 127.62, 127.60, 127.55, 127.53, 127.49, 127.47, 127.42, 127.41, 127.36, 127.3, 127.2, 127.1, 127.0, 126.9, 126.82, 126.78, 126.7, 124.4, 123.6, 123.3, 123.0, 122.8, 122.3, 102.2, 101.0, 100.6, 100.3, 100.05, 100.02, 99.53, 99.51, 99.46, 98.8, 98.2, 97.2, 97.0, 96.7, 96.4, 96.3, 81.2, 80.6, 80.5, 80.4, 80.2, 79.3, 78.9, 78.0, 77.6, 77.5, 77.1, 77.0, 76.80, 76.76, 76.7, 76.41, 76.37, 76.3, 76.2, 75.9, 75.1, 75.04, 74.99, 74.94, 74.92, 74.90, 74.85, 74.8, 74.7, 74.64, 74.61, 74.56, 74.4, 74.3, 74.2, 74.1, 73.90, 73.88, 73.80, 73.78, 73.70, 73.67, 73.63, 73.57, 73.55, 73.51, 73.49, 73.44, 73.37, 73.3, 73.23, 73.19, 73.1, 73.0, 72.9, 72.81, 72.77, 72.7, 72.5, 72.3, 72.1, 72.0, 71.83, 71.78, 71.6, 71.4, 70.8, 70.5, 70.3, 70.0, 69.93, 69.87, 69.6, 69.0, 68.6, 68.5, 68.3, 68.1, 67.8, 67.6, 67.0, 66.5, 65.5, 64.9, 56.83, 56.76, 56.1, 56.0, 55.9, 55.5, 55.45, 55.41, 51.2, 37.85, 37.80, 29.9, 28.8, 28.4, 28.1, 28.0, 23.1, 21.2, 16.1 ppm. **G11**: ^{1}H NMR (600 MHz, D_2O) δ = 5.16 – 5.10 (m, 2H), 4.87 (s, 1H), 4.84 – 4.81 (m, 1H), 4.77 (s, 1H), 4.73 – 4.67 (m, 3H), 4.60 (apparent d, J = 7.7 Hz, 2H), 4.58 – 4.53

(m, 2H), 4.52 – 4.43 (m, 5H), 4.27 – 4.24 (m, 1H), 4.24 – 4.18 (m, 2H), 4.18 – 4.13 (m, 2H), 4.12 – 4.06 (m, 2H), 4.03 – 3.38 (m, 88H), 2.99 (apparent t, *J* = 7.6 Hz, 2H), 2.11 – 1.99 (m, 24H), 1.67 (apparent quint, *J* = 7.7 Hz, 2H), 1.60 (apparent quint, *J* = 6.6 Hz, 2H), 1.43 – 1.36 (m, 2H), 1.16 (d, *J* = 6.5 Hz, 3H) ppm; ^{13}C NMR (151 MHz, D_2O) δ = 175.0, 174.84, 174.75, 174.7, 174.6, 174.5, 174.4, 174.1, 103.0, 102.9, 102.8, 102.7, 102.5, 101.7, 101.6, 101.4, 101.0, 100.3, 99.64, 99.59, 99.57, 98.7, 97.2, 82.1, 81.9, 81.6, 80.3, 79.3, 79.1, 79.0, 78.6, 78.5, 78.2, 76.54, 76.46, 75.8, 75.6, 75.3, 75.1, 75.0, 74.9, 74.74, 74.66, 74.6, 74.5, 74.43, 74.37, 73.5, 73.3, 72.8, 72.5, 72.4, 72.2, 72.1, 72.0, 71.9, 71.8, 71.5, 70.9, 70.5, 70.2, 70.1, 70.0, 69.94, 69.89, 69.74, 69.66, 69.4, 69.2, 68.5, 68.32, 68.28, 68.2, 67.6, 67.5, 67.3, 66.7, 65.6, 65.1, 61.7, 61.4, 61.2, 61.0, 60.95, 60.91, 60.6, 60.5, 60.1, 59.8, 59.6, 55.9, 55.6, 55.3, 55.1, 55.02, 54.98, 54.9, 54.8, 39.3, 28.0, 26.3, 22.5, 22.4, 22.3, 22.24, 22.15, 22.14, 22.12, 22.07, 15.3 ppm. LRMS (ESI-MS) *m/z* calculated for $C_{117}H_{195}N_9O_{80}^{2-}$ [M-2H]$^{2-}$: 1503.1, found: 1503.3.

5-Aminopentyl β-D-galactopyranosyl-(1→4)-2-acetamido-2-deoxy-β-D-glucopyranosyl-(1→3)-β-D-galactopyranosyl-(1→4)-2-acetamido-2-deoxy-β-D-glucopyranosyl-(1→2)-α-D-mannopyranosyl-(1→3)-[2-acetamido-2-deoxy-β-D-glucopyranosyl-(1→2)-[β-D-galactopyranosyl-(1→4)-[α-L-fucopyranosyl-(1→3)]-2-acetamido-2-deoxy-β-D-glucopyranosyl-(1→3)-β-D-galactopyranosyl-(1→4)-2-acetamido-2-deoxy-β-D-glucopyranosyl-(1→3)-β-D-galactopyranosyl-(1→4)-2-acetamido-2-deoxy-β-D-glucopyranosyl-(1→6)]-α-D-mannopyranosyl-(1→6)]-β-D-mannopyranosyl-(1→4)-2-acetamido-2-deoxy-β-D-glucopyranosyl-(1→4)-2-acetamido-2-deoxy-β-D-glucopyranoside (G12): asymmetric tri-antennary *N*-glycan **G12** was synthesized from the tri-antennary precursor **2** and *o*-alkynylbenzoates **Module H** and **Module C** following General Procedures X, III, VII, III, XI and XII sequentially. **G12** (3.5 mg, 61% overall from 14.0 mg heptadecasaccharide intermediate) was acquired from P2 purification and lyophilization as a fluffy white solid. **The heptasaccharide intermediate**: ^{1}H NMR (600 MHz, $CDCl_3$) δ = 7.93 – 6.65 (m, 238H), 6.58 (br t, *J* = 7.4 Hz, 2H), 6.51 (br t, *J* = 7.3 Hz, 1H), 5.66 (dd, *J* = 10.0, 7.8 Hz, 1H), 5.38 – 5.24 (m, 4H), 5.23 – 4.97 (m, 9H), 4.94 – 3.88 (m, 110H), 3.88 – 3.11 (m, 61H), 3.09 (br t, *J* = 8.8 Hz, 1H), 3.03 – 2.96 (m, 2H), 2.96 – 2.78 (m, 5H), 2.63 (br d, *J* = 7.2 Hz, 1H), 2.42 – 2.28 (m, 2H), 2.05 (s, 3H), 1.40 – 1.27 (m, 4H), 1.14 – 1.01 (m, 5H) ppm; ^{13}C NMR (151 MHz, $CDCl_3$) δ = 169.0, 168.1, 167.8, 167.6, 167.3, 167.2, 165.2, 164.5, 164.4, 164.3, 139.39, 139.36, 139.33, 139.29, 139.2, 138.98, 138.96, 138.90, 138.86, 138.84, 138.78, 138.76, 138.52, 138.47, 138.40, 138.38, 138.34, 138.32, 138.26, 138.24, 138.22, 138.15, 138.13, 138.10, 138.03, 138.01, 137.96, 137.8, 136.3, 135.3, 133.8, 133.7, 133.4, 133.3, 133.25, 133.21, 133.1, 133.0, 132.9, 131.6, 131.5, 131.0, 130.05, 129.98, 129.84, 129.82, 129.76, 129.7, 129.5, 129.4, 128.9, 128.8, 128.63, 128.62, 128.60, 128.58, 128.56, 128.52, 128.46, 128.444, 128.439, 128.40, 128.37, 128.36, 128.31, 128.30, 128.28, 128.25, 128.19, 128.17, 128.16, 128.15, 128.13, 128.10, 128.06, 128.04, 128.00, 127.95, 127.92, 127.86, 127.85, 127.81, 127.75, 127.74, 127.71, 127.68, 127.61, 127.56, 127.52, 127.51, 127.49, 127.45, 127.41, 127.37, 127.31, 127.28,

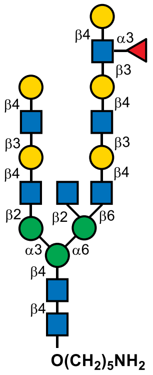

G12 (A20)

127.24, 127.18, 127.10, 127.06, 127.0, 126.8, 126.73, 126.71, 126.66, 126.5, 126.21, 126.17, 126.1, 126.05, 125.96, 125.9, 125.81, 125.78, 123.6, 123.3, 123.2, 122.8, 102.2, 101.01, 100.96, 100.6, 100.5, 100.00, 99.96, 99.72, 99.69, 99.3, 98.9, 98.2, 97.2, 96.9, 96.7, 96.6, 81.2, 80.9, 80.6, 80.4, 80.3, 79.8, 79.7, 79.5, 79.1, 79.0, 78.7, 78.0, 77.8, 77.6, 76.85, 76.78, 76.7, 76.65, 76.59, 76.57, 76.5, 76.4, 76.2, 75.5, 75.4, 75.1, 75.03, 74.95, 74.8, 74.7, 74.63, 74.59, 74.5, 74.4, 74.2, 73.8, 73.64, 73.62, 73.59, 73.56, 73.50, 73.45, 73.43, 73.39, 73.3, 73.0, 72.9, 72.8, 72.60, 72.56, 72.3, 72.2, 72.1, 72.0, 71.9, 71.7, 71.5, 71.43, 71.40, 70.8, 70.7, 70.6, 70.3, 70.0, 69.9, 69.0, 68.7, 68.6, 68.5, 68.30, 68.27, 68.2, 68.13, 68.09, 67.84, 67.82, 67.7, 67.6, 67.1, 66.6, 65.0, 56.9, 56.8, 56.1, 56.01, 55.95, 55.8, 55.5, 55.4, 51.2, 28.8, 28.4, 23.1, 21.1, 16.3 ppm. **G12**: ^{1}H NMR (600 MHz, D_2O) δ = 5.17 – 5.10 (m, 2H), 4.91 – 4.83 (m, 3H), 4.78 (s, 1H), 4.74 – 4.69 (m, 3H), 4.64 – 4.53 (m, 4H), 4.52 – 4.44 (m, 5H), 4.28 – 4.13 (m, 6H), 4.12 – 4.04 (m, 2H), 4.03 – 3.39 (m, 93H), 2.99 (apparent t, *J* = 7.6 Hz, 2H), 2.12 – 2.00 (m, 24H), 1.67 (apparent quint, *J* = 7.7 Hz, 2H), 1.59 (apparent hept, *J* = 6.5 Hz, 2H), 1.44 – 1.36 (m, 2H), 1.18 (d, *J* = 6.6 Hz, 3H) ppm; ^{13}C NMR (151 MHz, D_2O) δ = 174.89, 174.87, 174.7, 174.63, 174.58, 174.4, 174.2, 103.0, 102.9, 102.84, 102.76, 102.7, 102.5, 101.7, 101.6, 101.4, 101.0, 100.9, 100.4, 99.7, 99.5, 98.6, 97.2, 82.3, 82.1, 79.3, 78.5, 78.1, 76.5, 76.4, 75.8, 75.7, 75.3, 75.1, 74.90, 74.85, 74.73, 74.68, 74.52, 74.49, 74.3, 73.5, 73.3, 73.1, 73.0, 72.48, 72.45, 72.4, 72.25, 72.16, 71.9, 71.5, 71.0, 70.9, 70.2, 70.1, 69.94, 69.93, 69.4, 69.2, 68.5, 68.33, 68.27, 67.7, 67.5, 67.3, 66.7, 65.7, 61.7, 61.5, 61.0, 60.94, 60.90, 60.85, 60.6, 60.1, 59.8, 59.6, 55.9, 55.4, 55.2, 55.1, 55.02, 54.97, 54.9, 54.8, 39.3, 28.0, 26.3, 22.5, 22.34, 22.33, 22.23, 22.21, 22.17, 22.15, 22.11, 22.07, 15.3 ppm. LRMS (ESI-MS) *m/z* calculated for $C_{123}H_{205}N_9O_{85}^{2-}$ $[M-2H]^{2-}$: 1584.6, found: 1584.7.

5-Aminopentyl β-D-galactopyranosyl-(1→4)-2-acetamido-2-deoxy-β-D-glucopyranosyl-(1→3)-β-D-galactopyranosyl-(1→4)-2-acetamido-2-deoxy-β-D-glucopyranosyl-(1→3)-β-D-galactopyranosyl-(1→4)-2-acetamido-2-deoxy-β-D-glucopyranosyl-(1→2)-α-D-mannopyranosyl-(1→3)-[2-acetamido-2-deoxy-β-D-glucopyranosyl-(1→2)-[α-L-fucopyranosyl-(1→3)-2-acetamido-2-deoxy-β-D-glucopyranosyl-(1→3)-β-D-galactopyranosyl-(1→4)-2-acetamido-2-deoxy-β-D-glucopyranosyl-(1→6)]-α-D-mannopyranosyl-(1→6)]-β-D-mannopyranosyl-(1→4)-2-acetamido-2-deoxy-β-D-glucopyranosyl-(1→4)-2-acetamido-2-deoxy-β-D-glucopyranoside (G13): asymmetric tri-antennary *N*-glycan **G13** was synthesized from the tri-antennary precursor **2** and *o*-alkynylbenzoates **Module F** and **Module B** following General Procedures VII, III, X, III, XI and XII sequentially. **G13** (10.0 mg, 54% overall from 45.0 mg hexadecasaccharide intermediate) was acquired from P2 purification and lyophilization as a fluffy white solid. **The hexasaccharide intermediate**: ^{1}H NMR (600 MHz, $CDCl_3$) δ = 7.94 – 7.89 (m, 1H), 7.86 – 6.91 (m, 187H), 6.90 – 6.70 (m, 27H), 6.70 – 6.65 (m, 1H), 6.60 (apparent t, *J* = 7.4 Hz, 2H), 6.57 – 6.50 (m, 1H), 5.61 (s, 1H), 5.54 – 5.46 (m, 2H), 5.41 – 5.27 (m, 3H), 5.24 (d, *J* = 8.3 Hz, 1H), 5.16 (d, *J* = 7.9 Hz, 1H), 5.13 – 5.07 (m, 2H), 5.04 (d, *J* = 11.9 Hz, 2H), 5.00 (br d, *J* = 6.2 Hz, 1H), 4.96 – 4.63 (m, 21H), 4.63 – 3.57 (m, 102H), 3.57 – 3.20 (m, 30H), 3.20 – 3.08 (m, 3H), 3.06 – 2.98 (m, 2H), 2.99 – 2.78 (m, 5H), 2.72 (br d, *J* = 9.3 Hz, 1H), 2.46 – 2.33 (m, 2H), 2.25 (br s, 1H), 2.04

(s, 3H), 2.02 – 1.92 (m, 1H), 1.46 – 1.31 (m, 4H), 1.17 – 1.03 (m, 2H), 0.88 (d, J = 6.5 Hz, 3H) ppm; ^{13}C NMR (151 MHz, $CDCl_3$) δ = 169.4, 168.6, 168.2, 168.1, 167.9, 167.6, 167.5, 167.31, 167.25, 167.2, 164.6, 164.4,

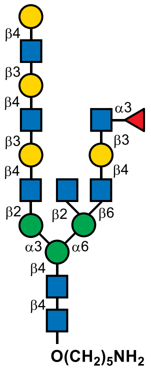

G13 (A8)

164.3, 139.4, 139.3, 139.2, 139.00, 138.96, 138.91, 138.90, 138.89, 138.86, 138.82, 138.77, 138.7, 138.51, 138.47, 138.40, 138.36, 138.32, 138.26, 138.23, 138.20, 138.17, 138.16, 138.1, 138.03, 137.97, 137.1, 136.3, 133.7, 133.5, 133.4, 133.3, 133.2, 133.0, 132.9, 131.9, 131.7, 131.6, 131.5, 130.9, 129.8, 129.7, 129.51, 129.46, 129.0, 128.8, 128.7, 128.63, 128.62, 128.58, 128.56, 128.54, 128.52, 128.48, 128.40, 128.37, 128.36, 128.32, 128.29, 128.25, 128.24, 128.19, 128.18, 128.15, 128.13, 128.12, 128.10, 128.06, 128.04, 128.03, 128.00, 127.99, 127.96, 127.92, 127.91, 127.87, 127.85, 127.80, 127.79, 127.75, 127.73, 127.70, 127.65, 127.60, 127.56, 127.52, 127.48, 127.412, 127.405, 127.31, 127.28, 127.24, 127.18, 127.0, 126.8, 126.74, 126.69, 126.6, 126.19, 126.17, 126.1, 126.0, 125.9, 123.5, 123.4, 123.3, 123.2, 123.1, 122.8, 102.2, 101.3, 101.0, 100.8, 100.6, 100.5, 100.2, 99.8, 99.7, 99.4, 99.3, 98.9, 98.2, 97.2, 96.8, 96.6, 82.1, 81.4, 80.54, 80.48, 80.4, 80.3, 79.6, 79.1, 79.0, 78.2, 78.1, 78.0, 77.8, 77.6, 77.4, 76.8, 76.71, 76.65, 76.5, 76.2, 75.4, 75.08, 75.05, 75.02, 74.95, 74.9, 74.8, 74.65, 74.57, 74.53, 74.49, 74.42, 74.38, 74.3, 73.8, 73.74, 73.67, 73.6, 73.54, 73.504, 73.496, 73.45, 73.42, 73.37, 73.3, 73.2, 73.0, 72.8, 72.7, 72.5, 72.3, 72.13, 72.06, 71.9, 71.8, 70.74, 70.67, 70.3, 70.0, 69.9, 69.0, 68.9, 68.7, 68.54, 68.49, 68.4, 68.3, 68.1, 67.8, 67.7, 67.3, 67.2, 66.3, 65.4, 65.0, 56.9, 56.1, 56.0, 55.8, 55.6, 55.4, 51.2, 28.8, 28.4, 23.1, 21.2, 16.5 ppm. **G13**: ^{1}H NMR (600 MHz, D_2O) δ = 5.13 (s, 1H), 5.01 (d, J = 4.1 Hz, 1H), 4.87 (s, 1H), 4.78 (s, 1H), 4.74 – 4.68 (m, 3H), 4.63 – 4.52 (m, 4H), 4.51 – 4.44 (m, 5H), 4.34 (q, J = 6.4 Hz, 1H), 4.28 – 4.18 (m, 3H), 4.18 – 4.13 (m, 3H), 4.11 – 4.07 (m, 1H), 4.03 – 3.39 (m, 88H), 2.99 (apparent t, J = 7.7 Hz, 2H), 2.11 – 1.98 (m, 24H), 1.67 (apparent quint, J = 7.7 Hz, 2H), 1.63 – 1.56 (m, 2H), 1.44 – 1.34 (m, 2H), 1.17 (d, J = 6.6 Hz, 3H) ppm; ^{13}C NMR (151 MHz, D_2O) δ = 174.95, 174.89, 174.7, 174.63, 174.58, 174.4, 174.2, 103.0, 102.9, 102.84, 102.77, 102.7, 102.6, 101.6, 101.4, 101.0, 100.4, 99.9, 99.7, 99.5, 97.2, 82.1, 81.9, 80.2, 80.1, 79.4, 79.3, 78.5, 78.4, 78.1, 76.5, 76.4, 75.8, 75.7, 75.3, 74.9, 74.7, 74.53, 74.50, 74.4, 73.5, 73.3, 72.5, 72.4, 72.24, 72.16, 71.9, 71.8, 71.5, 70.9, 70.3, 70.2, 70.1, 70.0, 69.9, 69.5, 69.39, 69.37, 68.5, 68.3, 68.0, 67.5, 67.3, 66.9, 65.7, 65.6, 61.7, 61.0, 60.9, 60.6, 60.5, 60.1, 59.9, 59.8, 55.40, 55.37, 55.2, 55.1, 55.01, 54.97, 54.9, 54.8, 39.3, 28.0, 26.3, 22.5, 22.35, 22.32, 22.22, 22.17, 22.16, 22.11, 22.08, 15.2 ppm. LRMS (ESI-MS) m/z calculated for $C_{117}H_{195}N_9O_{80}^{2-}$ $[M-2H]^{2-}$: 1503.6, found: 1503.4.

Figure S1. ***N*****-glycans used for microarray assays (sorted according to the terminal epitopes)**[a]

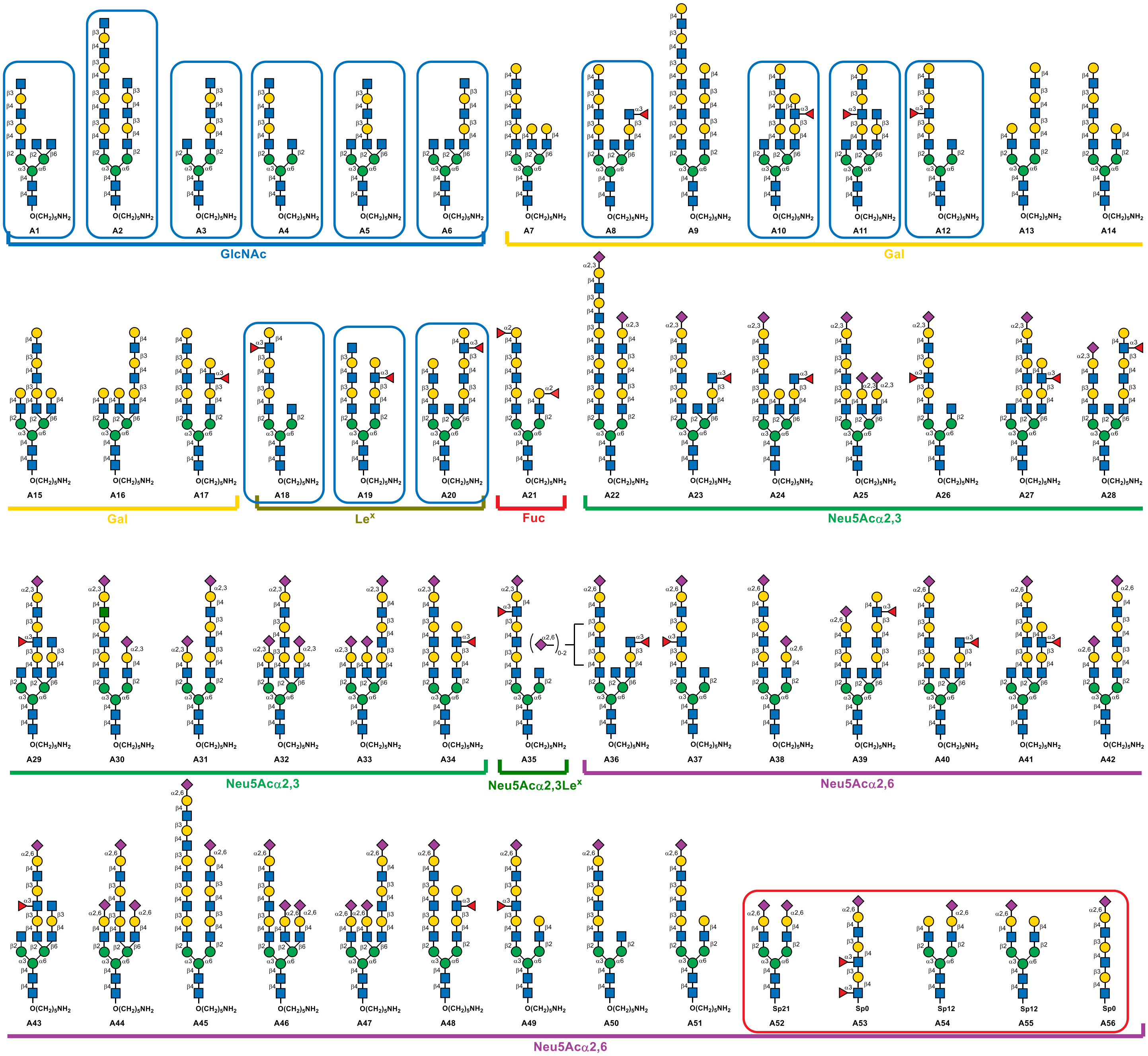

[a]*N*-glycans in the blue boxes (**A1–A6**, **A8**, **A10–A12** and **A18–A20**, or **G1–G13**) were synthesized chemically; *N*-glycans in the red box (**A52–A56**) were from the Consortium for Functional Glycomics (CFG) Database. All the other listed *N*-glycans were derivatized enzymatically from **G1–G13** (see Table S1 for details).

Table S1. Summary of preparation, ^{1}H NMR and LRMS data of A1–A51

Cpd No.	Structure	Parent cpd	General Procedure (Reaction Scale)	Yield (quantity)	^{1}H NMR and LRMS Data
**A1 (G7)**	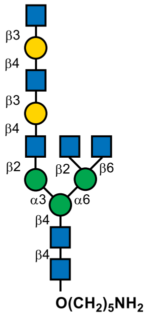	**2**	**VII** (300 mg **2**), **III** (100 mg acceptor + 109 mg **Module E**), **XI**, **XII** (37 mg fully protected dodecasaccharide)	39% from **2** (9.0 mg)	**^{1}H NMR (600 MHz, D_2O)** δ = 5.13 (s, 1H), 4.89 – 4.86 (m, 1H), 4.78 (s, 1H), 4.72 – 4.67 (m, 2H), 4.63 – 4.52 (m, 4H), 4.52 – 4.42 (m, 3H), 4.25 (d, *J* = 3.0 Hz, 1H), 4.22 – 4.18 (m, 2H), 4.16 (d, *J* = 3.3 Hz, 2H), 4.10 (d, *J* = 4.5 Hz, 1H), 4.02 – 3.39 (m, 68H), 2.98 (apparent t, *J* = 7.7 Hz, 2H), 2.11 – 2.00 (m, 21H), 1.67 (apparent quint, *J* = 7.7 Hz, 2H), 1.62 – 1.56 (m, 2H), 1.44 – 1.36 (m, 2H) ppm. <br>**LRMS (ESI-MS)** *m/z* calculated for $C_{91}H_{152}N_8O_{61}^{2-}$ $[M-2H]^{2-}$: 1166.5, found: 1166.6.
**A2 (G6)**	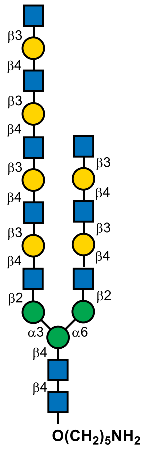	**1**	**VII** (80 mg **1**), **III** (50 mg acceptor + 66 mg **Module E**), **VIII** (50 mg product from the last step), **III** (40 mg acceptor + 60 mg **Module I**), **XI**, **XII** (39 mg fully protected nonadecasaccharide)	23% from **1** (9.6 mg)	**^{1}H NMR (600 MHz, D_2O)** δ = 5.12 (s, 1H), 4.93 (s, 1H), 4.78 (s, 1H), 4.72 – 4.65 (m, 6H), 4.63 – 4.54 (m, 3H), 4.53 – 4.40 (m, 7H), 4.28 – 4.07 (m, 9H), 4.04 – 3.39 (m, 107H), 2.99 (apparent t, *J* = 7.7 Hz, 2H), 2.11 – 1.98 (m, 30H), 1.67 (apparent quint, *J* = 7.7 Hz, 2H), 1.59 (apparent quint, *J* = 6.6 Hz, 2H), 1.45 – 1.35 (m, 2H) ppm. <br>**LRMS (ESI-MS)** *m/z* calculated for $C_{139}H_{230}N_{11}O_{96}^{3-}$ $[M-3H]^{3-}$: 1196.8, found: 1196.8.
**A3 (G1)**	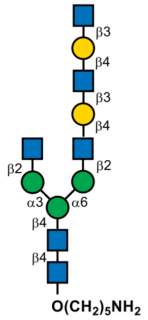	**1**	**VII** (80 mg **1**), **III** (28 mg acceptor + 37 mg **Module E**), **XI**, **XII** (31 mg fully protected undecasaccharide)	36% from **1** (7.5 mg)	**^{1}H NMR (600 MHz, D_2O)** δ = 5.12 (br s, 1H), 4.93 (br s, 1H), 4.77 (br s, 1H), 4.71 (d, *J* = 9.6 Hz, 1H), 4.69 (d, *J* = 8.5 Hz, 1H), 4.60 (d, *J* = 8.3 Hz, 1H), 4.59 (d, *J* = 8.3 Hz, 1H), 4.56 (d, *J* = 8.4 Hz, 1H), 4.50 (d, *J* = 7.7 Hz, 1H), 4.49 – 4.45 (m, 2H), 4.25 (d, *J* = 2.8 Hz, 1H), 4.20 (d, *J* = 3.4 Hz, 1H), 4.16 (apparent t, *J* = 3.7 Hz, 2H), 4.11 (d, *J* = 3.7 Hz, 1H), 4.02 – 3.40 (m, 63H), 2.99 (apparent t, *J* = 7.6 Hz, 2H), 2.09 (s, 3H), 2.06 (s, 3H), 2.05 (s, 3H), 2.05 – 2.04 (m, 6H), 2.04 (s, 3H), 1.67 (apparent quint, *J* = 7.7 Hz, 2H), 1.59 (apparent hept, *J* = 6.5 Hz, 2H), 1.44 – 1.37 (m, 2H) ppm.

					**LRMS (ESI-MS)** *m/z* calculated for $C_{83}H_{139}N_7O_{56}^{2-}$ [M-2H]$^{2-}$: 1064.9, found: 1065.0.
**A4 (G2)**	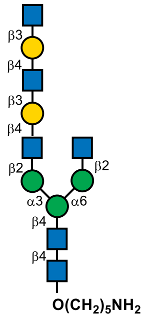	**1**	**VIII** (66 mg **1**), **III** (24 mg acceptor + 30 mg **Module E**), **XI**, **XII** (40 mg fully protected undecasaccharide)	34% from **1** (9.0 mg)	**^{1}H NMR (600 MHz, D_2O)** δ = 5.12 (br s, 1H), 4.93 (br s, 1H), 4.78 (s, 1H), 4.72 – 4.67 (m, 2H), 4.60 (d, J = 7.9 Hz, 1H), 4.58 (br d, J = 7.6 Hz, 1H), 4.56 (d, J = 8.5 Hz, 1H), 4.50 (d, J = 7.6 Hz, 1H), 4.49 – 4.44 (m, 2H), 4.25 (d, J = 2.6 Hz, 1H), 4.20 (d, J = 3.3 Hz, 1H), 4.18 – 4.14 (m, 2H), 4.13 – 4.10 (m, 1H), 4.02 – 3.40 (m, 63H), 2.99 (apparent t, J = 7.7 Hz, 2H), 2.09 (s, 3H), 2.08 – 2.05 (m, 6H), 2.05 – 2.02 (m, 9H), 1.67 (apparent quint, J = 7.7 Hz, 2H), 1.60 (apparent quint, J = 6.6 Hz, 2H), 1.44 – 1.36 (m, 2H) ppm.<br><br>**LRMS (ESI-MS)** *m/z* calculated for $C_{83}H_{139}N_7O_{56}^{2-}$ [M-2H]$^{2-}$: 1064.9, found: 1065.2.
**A5 (G8)**	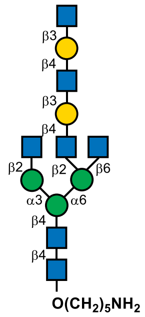	**2**	**IX** (300 mg **2**), **III** (50 mg acceptor + 50 mg **Module E**), **XI**, **XII** (50 mg fully protected dodecasaccharide)	32% from **2** (12.3 mg)	**^{1}H NMR (600 MHz, D_2O)** δ = 5.13 (s, 1H), 4.87 (s, 1H), 4.78 (s, 1H), 4.70 (apparent t, J = 9.4 Hz, 2H), 4.60 (apparent d, J = 7.8 Hz, 2H), 4.56 (d, J = 8.4 Hz, 1H), 4.53 (d, J = 8.3 Hz, 1H), 4.51 – 4.44 (m, 3H), 4.26 (br d, J = 2.2 Hz, 1H), 4.23 – 4.18 (m, 2H), 4.18 – 4.14 (m, 2H), 4.09 (br d, J = 2.9 Hz, 1H), 4.02 – 3.37 (m, 68H), 2.99 (apparent t, J = 7.7 Hz, 2H), 2.12 – 2.00 (m, 21H), 1.67 (apparent quint, J = 7.8 Hz, 2H), 1.63 – 1.54 (m, 2H), 1.46 – 1.36 (m, 2H) ppm.<br><br>**LRMS (ESI-MS)** *m/z* calculated for $C_{91}H_{152}N_8O_{61}^{2-}$ [M-2H]$^{2-}$: 1166.5, found: 1166.7.
**A6 (G9)**	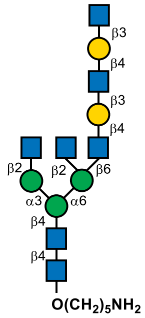	**2**	**X** (300 mg **2**), **III** (50 mg acceptor + 50 mg **Module E**), **XI**, **XII** (46 mg fully protected dodecasaccharide)	39% from **2** (11.5 mg)	**^{1}H NMR (600 MHz, D_2O)** δ = 5.13 (s, 1H), 4.87 (s, 1H), 4.78 (s, 1H), 4.71 (d, J = 8.5 Hz, 1H), 4.69 (d, J = 8.6 Hz, 1H), 4.60 (d, J = 7.8 Hz, 1H), 4.58 – 4.54 (m, 3H), 4.50 (d, J = 7.8 Hz, 1H), 4.47 (d, J = 7.9 Hz, 2H), 4.26 (d, J = 3.0 Hz, 1H), 4.23 – 4.18 (m, 2H), 4.16 (d, J = 3.2 Hz, 2H), 4.10 (br d, J = 4.0 Hz, 1H), 4.03 – 3.39 (m, 68H), 3.03 – 2.96 (m, 2H), 2.11 – 1.99 (m, 21H), 1.68 (apparent quint, J = 7.7 Hz, 2H), 1.63 – 1.54 (m, 2H), 1.46 – 1.35 (m, 2H) ppm.

					**LRMS (ESI-MS)** *m/z* calculated for $C_{91}H_{152}N_8O_{61}^{2-}$ [M-2H]$^{2-}$: 1166.5, found: 1166.6.
**A7**	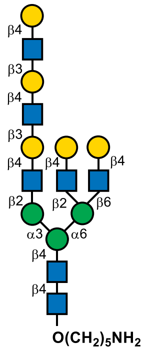	**A1**	**XIII** (UDP-Gal, Mn^{2+}, β4GalT1, 7.5 mg **A1**)	90-95% (8.5 mg)	**^{1}H NMR (600 MHz, D_2O)** δ = 5.13 (br s, 1H), 4.87 (br s, 1H), 4.77 (s, 1H), 4.74 – 4.67 (m, 2H), 4.64 – 4.52 (m, 4H), 4.52 – 4.42 (m, 6H), 4.28 – 4.13 (m, 5H), 4.12 – 4.07 (m, 1H), 4.05 – 3.37 (m, 86H), 2.99 (apparent t, *J* = 7.7 Hz, 2H), 2.11 – 2.00 (m, 21H), 1.68 (apparent hept, *J* = 8.1 Hz, 2H), 1.62 – 1.56 (m, 2H), 1.44 – 1.35 (m, 2H) ppm.<br><br>**LRMS (ESI-MS)** *m/z* calculated for $C_{109}H_{182}N_8O_{76}^{2-}$ [M-2H]$^{2-}$: 1410.0, found: 1410.0.
**A8 (G13)**	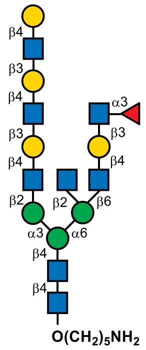	**2**	**VII** (300 mg **2**), **III** (50 mg acceptor + 60 mg **Module F**), **X** (50 mg product from the last step), **III** (46 mg acceptor + 25 mg **Module B**), **XI**, **XII** (45 mg fully protected hexasaccharide)	28% from **2** (10.0 mg)	**^{1}H NMR (600 MHz, D_2O)** δ = 5.13 (s, 1H), 5.01 (d, *J* = 4.1 Hz, 1H), 4.87 (s, 1H), 4.78 (s, 1H), 4.74 – 4.68 (m, 3H), 4.63 – 4.52 (m, 4H), 4.51 – 4.44 (m, 5H), 4.34 (q, *J* = 6.4 Hz, 1H), 4.28 – 4.18 (m, 3H), 4.18 – 4.13 (m, 3H), 4.11 – 4.07 (m, 1H), 4.03 – 3.39 (m, 88H), 2.99 (apparent t, *J* = 7.7 Hz, 2H), 2.11 – 1.98 (m, 24H), 1.67 (apparent quint, *J* = 7.7 Hz, 2H), 1.63 – 1.56 (m, 2H), 1.44 – 1.34 (m, 2H), 1.17 (d, *J* = 6.6 Hz, 3H) ppm.<br><br>**LRMS (ESI-MS)** *m/z* calculated for $C_{117}H_{195}N_9O_{80}^{2-}$ [M-2H]$^{2-}$: 1503.6, found: 1503.4.
**A9**	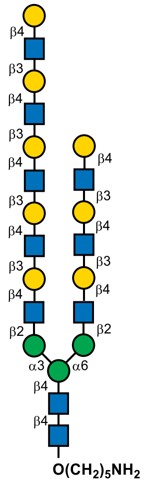	**A2**	**XIII** (UDP-Gal, Mn^{2+}, β4GalT1, 8.0 mg **A2**)	90-95% (8.0 mg)	**^{1}H NMR (600 MHz, D_2O)** δ = 5.12 (s, 1H), 4.93 (s, 1H), 4.75 (s, 1H), 4.71 (d, *J* = 8.3 Hz, 6H), 4.63 – 4.54 (m, 3H), 4.53 – 4.42 (m, 9H), 4.27 – 4.08 (m, 9H), 4.04 – 3.39 (m, 119H), 2.99 (apparent t, *J* = 7.7 Hz, 2H), 2.11 – 1.97 (m, 30H), 1.73 – 1.62 (m, 2H), 1.62 – 1.52 (m, 2H), 1.46 – 1.34 (m, 2H) ppm.<br><br>**LRMS (ESI-MS)** *m/z* calculated for $C_{151}H_{250}N_{11}O_{106}^{3-}$ [M-3H]$^{3-}$: 1304.8, found: 1304.9.

A10 (G10)	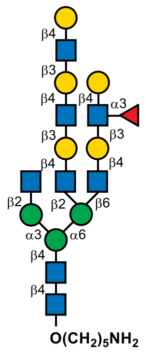	**2**	**IX** (300 mg **2**), **III** (50 mg acceptor + 60 mg **Module F**), **X** (54 mg product from the last step), **III** (30 mg acceptor + 25 mg **Module D**), **XI**, **XII** (17 mg fully protected heptadecasaccharide)	25% from **2** (4.1 mg)	**^{1}H NMR (600 MHz, D_2O)** δ = 5.16 – 5.10 (m, 2H), 4.87 (s, 1H), 4.86 – 4.83 (m, 1H), 4.77 (s, 1H), 4.74 – 4.68 (m, 3H), 4.60 (d, *J* = 7.8 Hz, 2H), 4.58 – 4.53 (m, 2H), 4.52 – 4.43 (m, 6H), 4.26 (br s, 1H), 4.24 – 4.18 (m, 2H), 4.18 – 4.14 (m, 3H), 4.09 (d, *J* = 3.0 Hz, 1H), 4.04 – 3.35 (m, 94H), 2.99 (apparent t, *J* = 7.7 Hz, 2H), 2.11 – 1.99 (m, 24H), 1.68 (apparent quint, *J* = 7.7 Hz, 2H), 1.60 (apparent quint, *J* = 6.7 Hz, 2H), 1.45 – 1.37 (m, 2H), 1.18 (d, *J* = 6.6 Hz, 3H) ppm. **LRMS (ESI-MS)** *m/z* calculated for $C_{123}H_{205}N_9O_{85}^{2-}$ $[M-2H]^{2-}$: 1584.6, found: 1584.9.
A11 (G11)	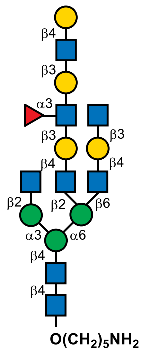	**2**	**IX** (300 mg **2**), **III** (50 mg acceptor + 60 mg **Module G**), **X** (56 mg product from the last step), **III** (30 mg acceptor + 15 mg **Module A**), **XI**, **XII** (25 mg fully protected hexasaccharide)	28% from **2** (6.0 mg)	**^{1}H NMR (600 MHz, D_2O)** δ = 5.16 – 5.10 (m, 2H), 4.87 (s, 1H), 4.84 – 4.81 (m, 1H), 4.77 (s, 1H), 4.73 – 4.67 (m, 3H), 4.60 (apparent d, *J* = 7.7 Hz, 2H), 4.58 – 4.53 (m, 2H), 4.52 – 4.43 (m, 5H), 4.27 – 4.24 (m, 1H), 4.24 – 4.18 (m, 2H), 4.18 – 4.13 (m, 2H), 4.12 – 4.06 (m, 2H), 4.03 – 3.38 (m, 88H), 2.99 (apparent t, *J* = 7.6 Hz, 2H), 2.11 – 1.99 (m, 24H), 1.67 (apparent quint, *J* = 7.7 Hz, 2H), 1.60 (apparent quint, *J* = 6.6 Hz, 2H), 1.43 – 1.36 (m, 2H), 1.16 (d, *J* = 6.5 Hz, 3H) ppm. **LRMS (ESI-MS)** *m/z* calculated for $C_{117}H_{195}N_9O_{80}^{2-}$ $[M-2H]^{2-}$: 1503.1, found: 1503.3.
A12 (G4)	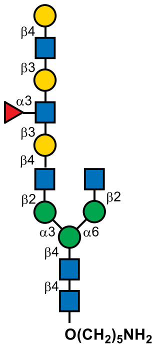	**1**	**VIII** (66 mg **1**), **III** (20 mg acceptor +36 mg **Module G**), **XI**, **XII** (19 mg fully protected tridecasaccharide)	36% from **1** (4.7 mg)	**^{1}H NMR (600 MHz, D_2O)** δ = 5.15 – 5.08 (m, 2H), 4.92 (s, 1H), 4.84 – 4.81 (m, 1H), 4.79 (s, 1H), 4.73 – 4.66 (m, 2H), 4.62 – 4.53 (m, 3H), 4.52 – 4.41 (m, 4H), 4.25 (br s, 1H), 4.19 (d, *J* = 2.9 Hz, 1H), 4.16 (d, *J* = 3.1 Hz, 1H), 4.13 – 4.08 (m, 2H), 4.02 – 3.40 (m, 72H), 2.98 (apparent t, *J* = 7.6 Hz, 2H), 2.08 (s, 3H), 2.07 – 1.98 (m, 15H), 1.67 (apparent quint, *J* = 7.7 Hz, 2H), 1.59 (apparent quint, *J* = 6.6 Hz, 2H), 1.43 – 1.36 (m, 2H), 1.15 (d, *J* = 6.5 Hz, 3H) ppm. **LRMS (ESI-MS)** *m/z* calculated for $C_{95}H_{159}N_7O_{65}^{2-}$ $[M-2H]^{2-}$: 1219.5, found: 1219.4.

**A13**	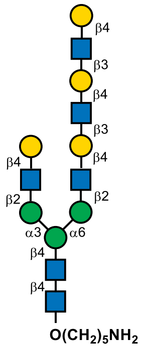	**A3**	**XIII** (UDP-Gal, Mn^{2+}, β4GalT1, 6.5 mg **A3**)	90-95% (7.2 mg)	**^{1}H NMR (600 MHz, D_2O)** δ = 5.11 (s, 1H), 4.91 (s, 1H), 4.76 (s, 1H), 4.71 – 4.67 (m, 2H), 4.61 – 4.53 (m, 3H), 4.51 – 4.42 (m, 5H), 4.27 – 4.02 (m, 7H), 4.02 – 3.40 (m, 73H), 2.97 (apparent t, *J* = 7.7 Hz, 2H), 2.14 – 1.93 (m, 18H), 1.66 (apparent quint, *J* = 7.1 Hz, 2H), 1.59 (apparent q, *J* = 6.6 Hz, 2H), 1.42 – 1.33 (m, 2H) ppm.<br><br>**LRMS (ESI-MS)** *m/z* calculated for $C_{95}H_{159}N_7O_{66}{}^{2-}$ $[M-2H]^{2-}$: 1227.5, found: 1227.6.
**A14**	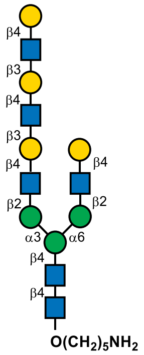	**A4**	**XIII** (UDP-Gal, Mn^{2+}, β4GalT1, 8.0 mg **A4**)	90-95% (8.5 mg)	**^{1}H NMR (600 MHz, D_2O)** δ = 5.12 (s, 1H), 4.93 (s, 1H), 4.77 (s, 1H), 4.72 – 4.67 (m, 2H), 4.63 – 4.55 (m, 3H), 4.52 – 4.42 (m, 5H), 4.27 – 4.09 (m, 5H), 4.04 – 3.44 (m, 75H), 2.99 (apparent t, *J* = 7.7 Hz, 2H), 2.11 – 1.98 (m, 18H), 1.67 (apparent quint, *J* = 7.7 Hz, 2H), 1.62 – 1.54 (m, 2H), 1.44 – 1.35 (m, 2H) ppm.<br><br>**LRMS (ESI-MS)** *m/z* calculated for $C_{95}H_{159}N_7O_{66}{}^{2-}$ $[M-2H]^{2-}$: 1227.5, found: 1227.7.
**A15**	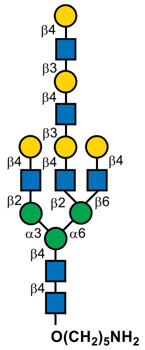	**A5**	**XIII** (UDP-Gal, Mn^{2+}, β4GalT1, 11.0 mg **A5**)	90-95% (11.9 mg)	**^{1}H NMR (600 MHz, D_2O)** δ = 5.14 (s, 1H), 4.88 (s, 1H), 4.77 (s, 1H), 4.71 (apparent d, *J* = 8.4 Hz, 2H), 4.64 – 4.57 (m, 3H), 4.55 (br d, *J* = 7.9 Hz, 1H), 4.52 – 4.44 (m, 6H), 4.25 (d, *J* = 2.9 Hz, 1H), 4.24 – 4.18 (m, 2H), 4.16 (apparent d, *J* = 2.8 Hz, 2H), 4.10 (br d, *J* = 3.5 Hz, 1H), 4.04 – 3.35 (m, 86H), 2.99 (apparent t, *J* = 7.8 Hz, 2H), 2.12 – 1.97 (m, 21H), 1.67 (apparent quint, *J* = 8.0 Hz, 2H), 1.63 – 1.54 (m, 2H), 1.48 – 1.34 (m, 2H) ppm.<br><br>**LRMS (ESI-MS)** *m/z* calculated for $C_{109}H_{182}N_8O_{76}{}^{2-}$ $[M-2H]^{2-}$: 1410.0, found: 1409.6.

**A16**	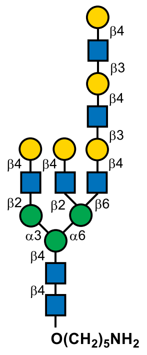	**A6**	**XIII**<br>(UDP-Gal, Mn^{2+},<br>β4GalT1, 9.6 mg **A6**)	90-95%<br>(10.6 mg)	**^{1}H NMR (600 MHz, D_2O)** δ = 5.13 (s, 1H), 4.87 (s, 1H), 4.77 (s, 1H), 4.71 (apparent d, J = 8.3 Hz, 2H), 4.63 – 4.57 (m, 3H), 4.55 (br d, J = 8.1 Hz, 1H), 4.52 – 4.42 (m, 6H), 4.25 (d, J = 2.9 Hz, 1H), 4.23 – 4.18 (m, 2H), 4.18 – 4.12 (m, 2H), 4.12 – 4.06 (m, 1H), 4.03 – 3.33 (m, 86H), 2.99 (apparent t, J = 7.9 Hz, 2H), 2.11 – 1.96 (m, 21H), 1.72 – 1.63 (m, 2H), 1.63 – 1.55 (m, 2H), 1.44 – 1.34 (m, 2H) ppm.<br><br>**LRMS (ESI-MS)** *m/z* calculated for $C_{109}H_{182}N_8O_{76}^{2-}$ $[M-2H]^{2-}$: 1410.0, found: 1409.8.
**A17**	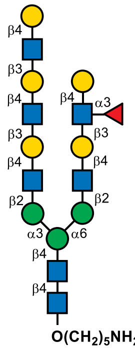	**A19**	**XIII**<br>(UDP-Gal, Mn^{2+},<br>β4GalT1, 3.0 mg **A19**)	90-95%<br>(2.8 mg)	**^{1}H NMR (600 MHz, D_2O)** δ = 5.13 (d, J = 4.1 Hz, 1H), 5.12 (br s, 1H), 4.93 (s, 1H), 4.84 (q, J = 6.7 Hz, 1H), 4.77 (s, 1H), 4.73 – 4.68 (m, 3H), 4.62 – 4.56 (m, 3H), 4.51 – 4.43 (m, 6H), 4.25 (d, J = 2.7 Hz, 1H), 4.19 (d, J = 3.5 Hz, 1H), 4.18 – 4.14 (m, 3H), 4.12 – 4.07 (m, 1H), 4.02 – 3.41 (m, 89H), 2.99 (apparent t, J = 7.7 Hz, 2H), 2.11 – 1.97 (m, 21H), 1.67 (apparent quint, J = 7.7 Hz, 2H), 1.59 (apparent quint, J = 6.5 Hz, 2H), 1.45 – 1.35 (m, 2H), 1.18 (d, J = 6.6 Hz, 3H) ppm.<br>**LRMS (ESI-MS)** *m/z* calculated for $C_{115}H_{192}N_8O_{80}^{2-}$ $[M-2H]^{2-}$: 1483.1, found: 1483.0.
**A18**<br>**(G3)**	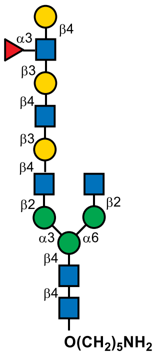	**1**	**VIII** (66 mg **1**),<br>**III** (24 mg acceptor +<br>40 mg **Module H**),<br>**XI**, **XII** (28 mg fully<br>protected<br>tridecasaccharide)	36% from **1**<br>(6.8 mg)	**^{1}H NMR (600 MHz, D_2O)** δ = 5.13 (d, J = 4.1 Hz, 1H), 5.12 (s, 1H), 4.92 (s, 1H), 4.85 (q, J = 6.6 Hz, 1H), 4.78 (s, 1H), 4.74 – 4.69 (m, 2H), 4.62 – 4.54 (m, 3H), 4.52 – 4.44 (m, 4H), 4.26 (d, J = 2.7 Hz, 1H), 4.19 (d, J = 3.9 Hz, 1H), 4.18 – 4.15 (m, 2H), 4.11 (dd, J = 3.4, 1.6 Hz, 1H), 4.02 – 3.40 (m, 72H), 2.99 (apparent t, J = 7.7 Hz, 2H), 2.09 (s, 3H), 2.07 – 2.05 (m, 6H), 2.05 – 2.01 (m, 9H), 1.67 (apparent quint, J = 7.7 Hz, 2H), 1.62 – 1.56 (m, 2H), 1.43 – 1.37 (m, 2H), 1.18 (d, J = 6.6 Hz, 3H) ppm.<br><br>**LRMS (ESI-MS)** *m/z* calculated for $C_{95}H_{159}N_7O_{65}^{2-}$ $[M-2H]^{2-}$: 1219.5, found: 1219.5.

**A19 (G5)**	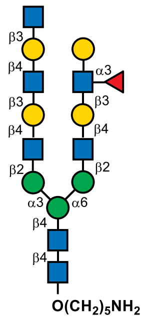	**1**	**VIII** (66 mg **1**), III (25 mg acceptor + 38 mg **Module E**), **VII** (30 mg product from last step), **III** (22 mg acceptor + 22 mg **Module D**), **XI, XII** (17 mg fully protected pentadecasaccharide)	20% from **1** (4.5 mg)	**^{1}H NMR (600 MHz, D$_2$O)** δ = 5.14 (d, *J* = 4.1 Hz, 1H), 5.12 (br s, 1H), 4.93 (br s, 1H), 4.84 (q, *J* = 6.8 Hz, 1H), 4.77 (br s, 1H), 4.73 – 4.67 (m, 3H), 4.63 – 4.54 (m, 3H), 4.53 – 4.42 (m, 5H), 4.25 (d, *J* = 2.8 Hz, 1H), 4.20 (d, *J* = 3.8 Hz, 1H), 4.18 – 4.13 (m, 3H), 4.11 (d, *J* = 3.4 Hz, 1H), 4.03 – 3.40 (m, 83H), 2.99 (apparent t, *J* = 7.6 Hz, 2H), 2.09 (s, 3H), 2.07 – 2.01 (m, 18H), 1.67 (apparent quint, *J* = 7.7 Hz, 2H), 1.60 (apparent quint, *J* = 6.6 Hz, 2H), 1.44 – 1.36 (m, 2H), 1.18 (d, *J* = 6.6 Hz, 3H) ppm.<br><br>**LRMS (ESI-MS)** *m/z* calculated for $C_{109}H_{182}N_8O_{75}^{2-}$ [M-2H]$^{2-}$: 1402.0, found: 1401.9.
**A20 (G12)**	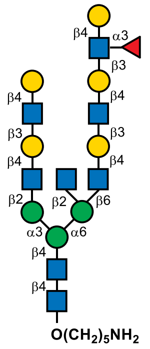	**2**	**X** (300 mg **2**), **III** (40 mg acceptor + 60 mg **Module H**), **VII** (51 mg product from the last step), **III** (35 mg acceptor + 20 mg **Module C**), **XI, XII** (14 mg fully protected heptasaccharide)	22% from **2** (3.5 mg)	**^{1}H NMR (600 MHz, D$_2$O)** δ = 5.17 – 5.10 (m, 2H), 4.91 – 4.83 (m, 3H), 4.78 (s, 1H), 4.74 – 4.69 (m, 3H), 4.64 – 4.53 (m, 4H), 4.52 – 4.44 (m, 5H), 4.28 – 4.13 (m, 6H), 4.12 – 4.04 (m, 2H), 4.03 – 3.39 (m, 93H), 2.99 (apparent t, *J* = 7.6 Hz, 2H), 2.12 – 2.00 (m, 24H), 1.67 (apparent quint, *J* = 7.7 Hz, 2H), 1.59 (apparent hept, *J* = 6.5 Hz, 2H), 1.44 – 1.36 (m, 2H), 1.18 (d, *J* = 6.6 Hz, 3H) ppm.<br><br>**LRMS (ESI-MS)** *m/z* calculated for $C_{123}H_{205}N_9O_{85}^{2-}$ [M-2H]$^{2-}$: 1584.6, found: 1584.7.
**A21**	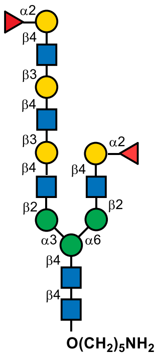	**A14**	**XVI** (GDP-Fuc, Mn^{2+}, *h*FuT2, 1.2 mg **A14**)	80% (1.1 mg)	**^{1}H NMR (600 MHz, D$_2$O)** δ = 5.31 (d, *J* = 3.0 Hz, 2H), 5.12 (s, 1H), 4.92 (s, 1H), 4.77 (s, 1H), 4.70 (apparent d, *J* = 8.4 Hz, 2H), 4.62 – 4.53 (m, 5H), 4.52 – 4.42 (m, 3H), 4.25 (br s, 1H), 4.22 (apparent t, *J* = 5.4 Hz, 2H), 4.19 (d, *J* = 3.3 Hz, 1H), 4.16 (apparent t, *J* = 3.7 Hz, 2H), 4.09 (d, *J* = 3.3 Hz, 1H), 4.01 – 3.42 (m, 81H), 2.99 (apparent t, *J* = 7.7 Hz, 2H), 2.11 – 2.00 (m, 18H), 1.67 (apparent quint, *J* = 7.7 Hz, 2H), 1.59 (apparent quint, *J* = 6.6 Hz, 2H), 1.44 – 1.35 (m, 2H), 1.26 – 1.21 (m, 6H) ppm.

					**LRMS (ESI-MS)** *m/z* calculated for $C_{107}H_{179}N_7O_{74}^{2-}$ $[M-2H]^{2-}$: 1373.5, found: 1373.4.
**A22**	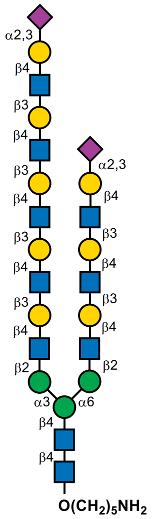	**A9**	**XIV** (CMP-Neu5Ac, Mg^{2+}, FAJ-JT-16, 2.0 mg **A9**)	83% (1.9 mg)	**^{1}H NMR (600 MHz, D_2O)** δ = 5.11 (s, 1H), 4.92 (s, 1H), 4.77 (s, 1H), 4.71 – 4.66 (m, 6H), 4.61 – 4.53 (m, 3H), 4.50 – 4.39 (m, 9H), 4.26 – 4.04 (m, 10H), 4.02 – 3.39 (m, 132H), 2.97 (apparent t, *J* = 7.7 Hz, 2H), 2.81 – 2.67 (m, 2H), 2.09 – 1.91 (m, 36H), 1.85 – 1.73 (m, 2H), 1.65 (apparent quint, *J* = 7.8 Hz, 2H), 1.61 – 1.53 (m, 2H), 1.42 – 1.33 (m, 2H) ppm.<br><br>**LRMS (ESI-MS)** *m/z* calculated for $C_{173}H_{284}N_{13}O_{122}^{3-}$ $[M-3H]^{3-}$: 1499.2, found: 1499.3.
**A23**	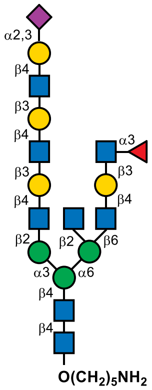	**A8**	**XIV** (CMP-Neu5Ac, Mg^{2+}, FAJ-JT-16, 3.0 mg **A8**)	90% (3.0 mg)	**^{1}H NMR (600 MHz, D_2O)** δ = 5.13 (s, 1H), 5.01 (d, *J* = 4.1 Hz, 1H), 4.87 (s, 1H), 4.77 (s, 1H), 4.70 (d, *J* = 7.8 Hz, 3H), 4.62 – 4.52 (m, 4H), 4.51 – 4.42 (m, 5H), 4.33 (q, *J* = 6.6 Hz, 1H), 4.29 – 4.06 (m, 8H), 4.04 – 3.37 (m, 94H), 2.97 (apparent t, *J* = 7.7 Hz, 2H), 2.76 (dd, *J* = 12.2, 4.6 Hz, 1H), 2.10 – 1.95 (m, 27H), 1.80 (apparent t, *J* = 12.1 Hz, 1H), 1.67 (apparent quint, *J* = 7.7 Hz, 2H), 1.59 (apparent quint, *J* = 6.3 Hz, 2H), 1.44 – 1.34 (m, 2H), 1.16 (d, *J* = 6.5 Hz, 3H) ppm.<br><br>**LRMS (ESI-MS)** *m/z* calculated for $C_{128}H_{212}N_{10}O_{88}^{2-}$ $[M-2H]^{2-}$: 1649.1, found: 1649.2.
**A24**	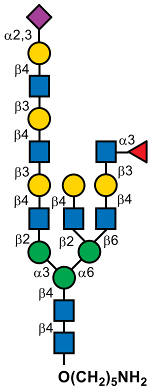	**A23**	**XIII** (UDP-Gal, Mn^{2+}, β4GalT1, 1.6 mg **A23**)	90-95% (1.6 mg)	**^{1}H NMR (600 MHz, D_2O)** δ = 5.13 (br s, 1H), 5.01 (d, *J* = 4.1 Hz, 1H), 4.88 (s, 1H), 4.77 (s, 1H), 4.73 – 4.67 (m, 3H), 4.63 – 4.52 (m, 5H), 4.52 – 4.43 (m, 5H), 4.34 (q, *J* = 6.6 Hz, 1H), 4.27 – 4.18 (m, 3H), 4.18 – 4.14 (m, 3H), 4.12 (dd, *J* = 9.9, 2.9 Hz, 1H), 4.10 – 4.07 (m, 1H), 4.04 – 3.36 (m, 100H), 2.99 (apparent t, *J* = 7.7 Hz, 2H), 2.76 (dd, *J* = 12.6, 4.6 Hz, 1H), 2.11 – 1.98 (m, 27H), 1.80 (apparent t, *J* = 12.2 Hz, 1H), 1.67 (apparent quint, *J* = 7.5 Hz, 2H), 1.62 – 1.54 (m, 2H), 1.44 – 1.35 (m, 2H), 1.17 (d, *J* = 6.5 Hz, 3H) ppm.

					**LRMS (ESI-MS)** *m/z* calculated for $C_{134}H_{221}N_{10}O_{93}^{3-}$ [M-3H]$^{3-}$: 1153.1, found: 1153.2.
**A25**	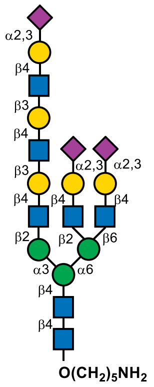	**A7**	**XIV** (CMP-Neu5Ac, Mg^{2+}, FAJ-JT-16, 1.4 mg **A7**)	85-90% (1.6 mg)	**^{1}H NMR (600 MHz, D_2O)** δ = 5.13 (s, 1H), 4.87 (s, 1H), 4.77 (s, 1H), 4.72 – 4.67 (m, 2H), 4.62 – 4.51 (m, 6H), 4.51 – 4.43 (m, 4H), 4.28 – 4.07 (m, 7H), 4.06 – 3.35 (m, 106H), 2.98 (apparent t, *J* = 7.7 Hz, 2H), 2.76 (br d, *J* = 12.1 Hz, 3H), 2.11 – 1.97 (m, 30H), 1.80 (apparent t, *J* = 12.4 Hz, 3H), 1.67 (apparent quint, *J* = 7.8 Hz, 2H), 1.62 – 1.54 (m, 2H), 1.45 – 1.34 (m, 2H) ppm.<br><br>**LRMS (ESI-MS)** *m/z* calculated for $C_{142}H_{232}N_{11}O_{100}^{3-}$ [M-3H]$^{3-}$: 1230.8, found: 1230.5.
**A26**	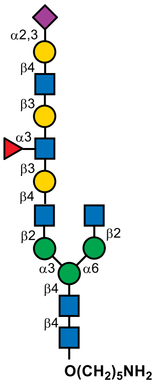	**A12**	**XIV** (CMP-Neu5Ac, Mg^{2+}, FAJ-JT-16, 1.2 mg **A12**)	85-90% (1.2 mg)	**^{1}H NMR (600 MHz, D_2O)** δ = 5.16 – 5.09 (s, 2H), 4.93 (s, 1H), 4.85 – 4.81 (m, 1H), 4.78 (s, 1H), 4.73 – 4.68 (m, 2H), 4.63 – 4.52 (m, 4H), 4.52 – 4.42 (m, 3H), 4.27 – 4.24 (m, 1H), 4.21 – 4.18 (m, 1H), 4.18 – 4.15 (m, 1H), 4.15 – 4.08 (m, 3H), 4.06 – 3.41 (m, 78H), 2.99 (apparent t, *J* = 7.7 Hz, 2H), 2.77 (dd, *J* = 12.7, 4.4 Hz, 1H), 2.07 – 1.99 (m, 21H), 1.81 (t, *J* = 12.0 Hz, 1H), 1.67 (apparent quint, *J* = 7.6 Hz, 2H), 1.62 – 1.55 (m, 2H), 1.44 – 1.36 (m, 2H), 1.16 (d, *J* = 6.5 Hz, 3H) ppm.<br><br>**LRMS (ESI-MS)** *m/z* calculated for $C_{106}H_{176}N_8O_{73}^{2-}$ [M-2H]$^{2-}$: 1365.0, found: 1364.9.
**A27**	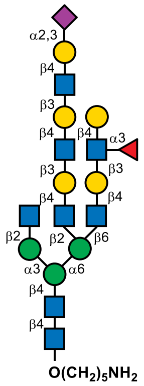	**A10**	**XIV** (CMP-Neu5Ac, Mg^{2+}, FAJ-JT-16, 1.6 mg **A10**)	85-90% (1.5 mg)	**^{1}H NMR (600 MHz, D_2O)** δ = 5.16 – 5.10 (m, 2H), 4.87 (s, 1H), 4.86 – 4.82 (m, 1H), 4.77 (s, 1H), 4.74 – 4.68 (m, 3H), 4.60 (br d, *J* = 7.6 Hz, 2H), 4.58 – 4.52 (m, 3H), 4.50 (d, *J* = 7.8 Hz, 1H), 4.48 – 4.41 (m, 4H), 4.28 – 4.24 (m, 1H), 4.23 – 4.18 (m, 2H), 4.18 – 4.14 (m, 3H), 4.12 (dd, *J* = 10.0, 2.7 Hz, 1H), 4.10 – 4.05 (m, 1H), 4.04 – 3.34 (m, 100H), 2.76 (dd, *J* = 12.6, 4.4 Hz, 1H), 2.66 (apparent t, *J* = 7.3 Hz, 2H), 2.11 – 1.98 (m, 27H), 1.80 (apparent t, *J* = 12.2 Hz, 1H), 1.60 – 1.51 (m, 2H), 1.51 – 1.43 (m, 2H), 1.37 – 1.29 (m, 2H), 1.18 (d, *J* = 6.5 Hz, 3H) ppm.

					**LRMS (ESI-MS)** *m/z* calculated for $C_{134}H_{221}N_{10}O_{93}^{3-}$ [M-3H]$^{3-}$: 1153.1, found: 1152.8.
**A28**	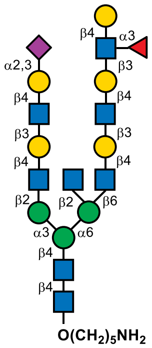	**A20**	**XIV** (CMP-Neu5Ac, Mg^{2+}, FAJ-JT-16, 1.6 mg **A20**)	75% (1.3 mg)	**^{1}H NMR (600 MHz, D_2O)** δ = 5.16 – 5.10 (m, 2H), 4.87 (s, 1H), 4.85 – 4.82 (m, 1H), 4.77 (s, 1H), 4.73 – 4.68 (m, 3H), 4.62 – 4.51 (m, 5H), 4.51 – 4.43 (m, 5H), 4.28 – 4.24 (m, 1H), 4.23 – 4.18 (m, 2H), 4.18 – 4.14 (m, 3H), 4.12 (dd, *J* = 9.7, 2.4 Hz, 1H), 4.10 – 4.07 (m, 1H), 4.03 – 3.37 (m, 100H), 2.84 (apparent t, *J* = 7.4 Hz, 2H), 2.76 (dd, *J* = 12.3, 4.3 Hz, 1H), 2.11 – 1.98 (m, 27H), 1.80 (apparent t, *J* = 12.2 Hz, 1H), 1.63 – 1.53 (m, 4H), 1.41 – 1.32 (m, 2H), 1.18 (d, *J* = 6.4 Hz, 3H) ppm.<br><br>**LRMS (ESI-MS)** *m/z* calculated for $C_{134}H_{221}N_{10}O_{93}^{3-}$ [M-3H]$^{3-}$: 1153.1, found: 1153.1.
**A29**	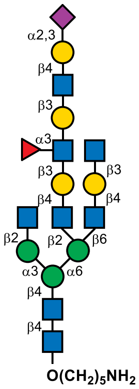	**A11**	**XIV** (CMP-Neu5Ac, Mg^{2+}, FAJ-JT-16, 1.5 mg **A11**)	80-90% (1.4 mg)	**^{1}H NMR (600 MHz, D_2O)** δ = 5.16 – 5.08 (m, 2H), 4.87 (s, 1H), 4.84 – 4.81 (m, 1H), 4.77 (s, 1H), 4.73 – 4.67 (m, 3H), 4.60 (d, *J* = 8.0 Hz, 2H), 4.58 – 4.52 (m, 3H), 4.49 (d, *J* = 7.4 Hz, 1H), 4.48 – 4.43 (m, 3H), 4.27 – 4.18 (m, 3H), 4.18 – 4.14 (m, 2H), 4.14 – 4.06 (m, 3H), 4.03 – 3.36 (m, 94H), 2.99 (apparent t, *J* = 7.8 Hz, 2H), 2.76 (dd, *J* = 12.4, 4.8 Hz, 1H), 2.11 – 1.97 (m, 27H), 1.80 (t, *J* = 12.2 Hz, 1H), 1.67 (apparent quint, *J* = 7.8 Hz, 2H), 1.62 – 1.56 (m, 2H), 1.43 – 1.36 (m, 2H), 1.16 (d, *J* = 6.4 Hz, 3H) ppm.<br><br>**LRMS (ESI-MS)** *m/z* calculated for $C_{128}H_{212}N_{10}O_{88}^{2-}$ [M-2H]$^{2-}$: 1649.1, found: 1649.3.

**A30**	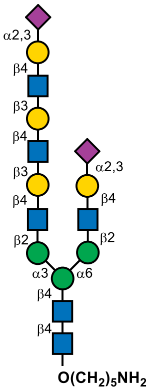	**A14**	**XIV** (CMP-Neu5Ac, Mg^{2+}, FAJ-JT-16, 1.2 mg **A14**)	80-90% (1.3 mg)	**^{1}H NMR (600 MHz, D_2O)** δ = 5.12 (s, 1H), 4.93 (s, 1H), 4.77 (s, 1H), 4.70 (d, *J* = 8.5 Hz, 2H), 4.62 – 4.53 (m, 5H), 4.51 – 4.43 (m, 3H), 4.28 – 4.23 (m, 1H), 4.22 – 4.18 (m, 1H), 4.17 – 4.15 (m, 2H), 4.14 – 4.09 (m, 3H), 4.03 – 3.45 (m, 87H), 2.99 (apparent t, *J* = 7.7 Hz, 2H), 2.76 (dd, *J* = 12.5, 4.6 Hz, 2H), 2.11 – 1.98 (m, 24H), 1.80 (apparent t, *J* = 12.1 Hz, 2H), 1.67 (apparent quint, *J* = 7.7 Hz, 2H), 1.63 – 1.56 (m, 2H), 1.47 – 1.34 (m, 2H) ppm.<br><br>**LRMS (ESI-MS)** *m/z* calculated for $C_{117}H_{193}N_9O_{82}^{2-}$ $[M-2H]^{2-}$: 1518.6, found: 1518.4.
**A31**	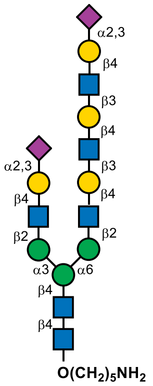	**A13**	**XIV** (CMP-Neu5Ac, Mg^{2+}, FAJ-JT-16, 1.2 mg **A13**)	80-90% (1.2 mg)	**^{1}H NMR (600 MHz, D_2O)** δ = 5.12 (s, 1H), 4.93 (s, 1H), 4.77 (s, 1H), 4.70 (br d, *J* = 8.1 Hz, 2H), 4.63 – 4.52 (m, 5H), 4.52 – 4.44 (m, 3H), 4.27 – 4.22 (m, 1H), 4.21 – 4.18 (m, 1H), 4.18 – 4.15 (m, 2H), 4.14 – 4.09 (m, 3H), 4.02 – 3.45 (m, 87H), 2.98 (apparent t, *J* = 7.7 Hz, 2H), 2.76 (dd, *J* = 12.3, 4.4 Hz, 2H), 2.11 – 1.99 (m, 24H), 1.80 (apparent t, *J* = 12.2 Hz, 2H), 1.67 (apparent quint, *J* = 7.7 Hz, 2H), 1.63 – 1.55 (m, 2H), 1.45 – 1.34 (m, 2H) ppm.<br><br>**LRMS (ESI-MS)** *m/z* calculated for $C_{117}H_{193}N_9O_{82}^{2-}$ $[M-2H]^{2-}$: 1518.6, found: 1518.5.
**A32**	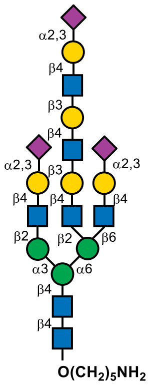	**A15**	**XIV** (CMP-Neu5Ac, Mg^{2+}, PmST1 (M144D), 1.4 mg **A15**)	63% (1.1 mg)	**^{1}H NMR (600 MHz, D_2O)** δ = 5.13 (s, 1H), 4.88 (s, 1H), 4.77 (s, 1H), 4.70 (br d, *J* = 8.7 Hz, 2H), 4.63 – 4.52 (m, 6H), 4.51 – 4.43 (m, 4H), 4.28 – 4.24 (m, 1H), 4.23 – 4.18 (m, 2H), 4.18 – 4.15 (m, 2H), 4.12 (d, *J* = 10.1 Hz, 2H), 4.09 (br s, 1H), 4.03 – 3.37 (m, 105H), 2.99 (apparent t, *J* = 7.7 Hz, 2H), 2.76 (dd, *J* = 12.7, 4.8 Hz, 3H), 2.10 – 1.96 (m, 30H), 1.81 (apparent t, *J* = 12.0 Hz, 3H), 1.67 (apparent quint, *J* = 7.6 Hz, 2H), 1.62 – 1.52 (m, 2H), 1.44 – 1.31 (m, 2H) ppm.<br><br>**LRMS (ESI-MS)** *m/z* calculated for $C_{142}H_{232}N_{11}O_{100}^{3-}$ $[M-3H]^{3-}$: 1230.8, found: 1230.9.

**A33**	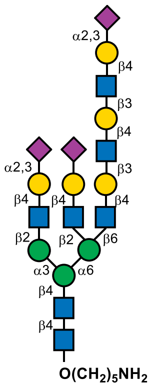	**A16**	**XIV** (CMP-Neu5Ac, Mg^{2+}, PmST1 (M144D), 1.4 mg **A16**)	81% (1.5 mg)	**^{1}H NMR (600 MHz, D_2O)** δ = 5.13 (s, 1H), 4.87 (s, 1H), 4.77 (s, 1H), 4.73 – 4.68 (m, 2H), 4.63 – 4.52 (m, 6H), 4.52 – 4.42 (m, 4H), 4.29 – 4.24 (m, 1H), 4.24 – 4.18 (m, 2H), 4.18 – 4.15 (m, 2H), 4.14 – 4.07 (m, 3H), 4.05 – 3.36 (m, 105H), 2.98 (apparent t, *J* = 7.6 Hz, 2H), 2.77 (dd, *J* = 12.5, 4.8 Hz, 3H), 2.12 – 1.95 (m, 30H), 1.81 (apparent t, *J* = 12.2 Hz, 3H), 1.72 – 1.62 (m, 2H), 1.62 – 1.55 (m, 2H), 1.44 – 1.33 (m, 2H) ppm.<br><br>**LRMS (ESI-MS)** *m/z* calculated for $C_{142}H_{232}N_{11}O_{100}^{3-}$ $[M-3H]^{3-}$: 1230.8, found: 1230.9.
**A34**	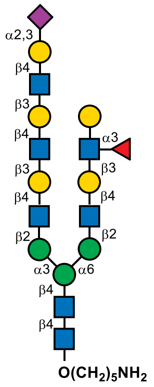	**A17**	**XIV** (CMP-Neu5Ac, Mg^{2+}, FAJ-JT-16, 1.5 mg **A17**)	90-100% (1.6 mg)	**^{1}H NMR (600 MHz, D_2O)** δ = 5.13 (d, *J* = 4.1 Hz, 1H), 5.12 (s, 1H), 4.93 (s, 1H), 4.86 – 4.82 (m, 1H), 4.77 (s, 1H), 4.73 – 4.68 (m, 3H), 4.62 – 4.53 (m, 4H), 4.51 – 4.43 (m, 5H), 4.27 – 4.23 (m, 1H), 4.21 – 4.15 (m, 4H), 4.14 – 4.09 (m, 2H), 4.02 – 3.43 (m, 95H), 2.98 (apparent t, *J* = 7.9 Hz, 2H), 2.76 (dd, *J* = 12.4, 4.6 Hz, 1H), 2.11 – 1.97 (m, 24H), 1.80 (apparent t, *J* = 12.2 Hz, 1H), 1.67 (apparent quint, *J* = 7.8 Hz, 2H), 1.63 – 1.56 (m, 2H), 1.45 – 1.34 (m, 2H), 1.18 (d, *J* = 6.5 Hz, 3H) ppm.<br><br>**LRMS (ESI-MS)** *m/z* calculated for $C_{126}H_{209}N_9O_{88}^{2-}$ $[M-2H]^{2-}$: 1628.6, found: 1628.6.
**A35**	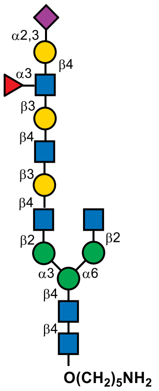	**A18**	**XIV** (CMP-Neu5Ac, Mg^{2+}, PmST1 (M144D), 1.2 mg **A18**)	57% (0.8 mg)	**^{1}H NMR (600 MHz, D_2O)** δ = 5.16 – 5.09 (m, 2H), 4.92 (s, 1H), 4.84 – 4.81 (m, 1H), 4.77 (s, 1H), 4.73 – 4.68 (m, 2H), 4.60 (d, *J* = 7.8 Hz, 1H), 4.58 (br d, *J* = 8.3 Hz, 1H), 4.56 (d, *J* = 8.6 Hz, 1H), 4.53 (d, *J* = 7.7 Hz, 1H), 4.49 (d, *J* = 7.0 Hz, 1H), 4.48 – 4.44 (m, 2H), 4.27 – 4.18 (m, 2H), 4.18 – 4.13 (m, 2H), 4.13 – 4.06 (m, 2H), 4.05 – 3.40 (m, 78H), 2.97 (apparent t, *J* = 7.7 Hz, 2H), 2.77 (dd, *J* = 12.6, 4.8 Hz, 1H), 2.10 – 1.98 (m, 21H), 1.80 (apparent t, *J* = 12.2 Hz, 1H), 1.69 – 1.62 (m, 2H), 1.62 – 1.55 (m, 2H), 1.43 – 1.33 (m, 2H), 1.17 (d, *J* = 6.3 Hz, 3H) ppm.

					**LRMS (ESI-MS)** *m/z* calculated for $C_{106}H_{176}N_8O_{73}^{2-}$ [M-2H]$^{2-}$: 1365.0, found: 1365.0.
**A36**	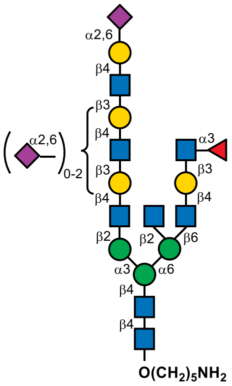	**A8**	**XV** (CMP-Neu5Ac, Mg^{2+}, JT-ISH-224, 1.5 mg **A8**)	80% (1.4 mg)	**^{1}H NMR (600 MHz, D$_2$O)** δ = 5.12 (s, 1H), 5.00 (s, 1H), 4.90 – 4.85 (m, 1H), 4.77 (s, 1H), 4.74 – 4.66 (m, 3H), 4.63 – 4.53 (m, 4H), 4.51 – 4.42 (m, 5H), 4.34 (q, *J* = 6.4 Hz, 1H), 4.26 (d, *J* = 7.0 Hz, 1H), 4.23 – 4.12 (m, 5H), 4.12 – 4.06 (m, 1H), 4.05 – 3.36 (m, 102H), 2.99 (apparent t, *J* = 7.7 Hz, 2H), 2.67 (br d, *J* = 12.4 Hz, 2H), 2.11 – 1.98 (m, 30H), 1.77 – 1.62 (m, 4H), 1.62 – 1.54 (m, 2H), 1.44 – 1.35 (m, 2H), 1.16 (d, *J* = 6.6 Hz, 3H) ppm.<br><br>**LRMS (ESI-MS)** *m/z* calculated for tri-sialoside $C_{150}H_{245}N_{12}O_{104}^{3-}$ [M-3H]$^{3-}$: 1293.1, found: 1293.2.
**A37**	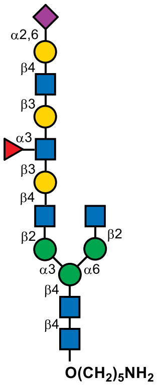	**A12**	**XV** (CMP-Neu5Ac, Mg^{2+}, *h*ST6Gal1, 2.4 mg **A12**, repeated for 2 rounds)	50% (1.4 mg)	**^{1}H NMR (600 MHz, D$_2$O)** δ = 5.17 – 5.08 (m, 2H), 4.92 (s, 1H), 4.84 – 4.81 (m, 1H), 4.77 (s, 1H), 4.75 – 4.67 (m, 2H), 4.60 (d, *J* = 7.8 Hz, 1H), 4.58 (br d, *J* = 7.8 Hz, 1H), 4.56 (d, *J* = 8.6 Hz, 1H), 4.49 (d, *J* = 7.4 Hz, 1H), 4.48 – 4.43 (m, 3H), 4.27 – 4.22 (m, 1H), 4.19 (d, *J* = 3.5 Hz, 1H), 4.16 (d, *J* = 2.5 Hz, 1H), 4.12 – 4.09 (m, 2H), 4.04 – 3.40 (m, 79H), 2.93 (apparent t, *J* = 7.5 Hz, 2H), 2.67 (dd, *J* = 12.4, 4.6 Hz, 1H), 2.10 – 2.03 (m, 21H), 1.73 (apparent t, *J* = 12.1 Hz, 1H), 1.64 (apparent quint, *J* = 7.8 Hz, 2H), 1.61 – 1.55 (m, 2H), 1.43 – 1.35 (m, 2H), 1.16 (d, *J* = 6.5 Hz, 3H) ppm.<br><br>**LRMS (ESI-MS)** *m/z* calculated for $C_{106}H_{176}N_8O_{73}^{2-}$ [M-2H]$^{2-}$: 1365.0, found: 1364.9.

**A38**	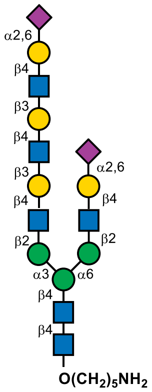	**A14**	**XV** (CMP-Neu5Ac, Mg^{2+}, Pd26ST (A200Y/S232Y), 1.2 mg **A14**)	70-80% (1.1 mg)	**^{1}H NMR (600 MHz, D_2O)** δ = 5.12 (s, 1H), 4.95 (s, 1H), 4.78 (s, 1H), 4.73 (d, *J* = 7.8 Hz, 1H), 4.70 (d, *J* = 8.3 Hz, 1H), 4.63 – 4.55 (m, 3H), 4.52 – 4.43 (m, 5H), 4.27 – 4.23 (m, 1H), 4.19 (d, *J* = 3.6 Hz, 1H), 4.18 – 4.14 (m, 2H), 4.12 (d, *J* = 3.6 Hz, 1H), 4.05 – 3.41 (m, 89H), 2.97 (apparent t, *J* = 7.7 Hz, 2H), 2.68 (dd, *J* = 12.5, 4.7 Hz, 2H), 2.11 – 1.98 (m, 24H), 1.72 (apparent t, *J* = 12.2 Hz, 2H), 1.66 (apparent quint, *J* = 7.9 Hz, 2H), 1.59 (apparent quint, *J* = 6.6 Hz, 2H), 1.45 – 1.33 (m, 2H) ppm.<br><br>**LRMS (ESI-MS)** *m/z* calculated for $C_{117}H_{193}N_9O_{82}^{2-}$ $[M\text{-}2H]^{2-}$: 1518.6, found: 1518.4.
**A39**	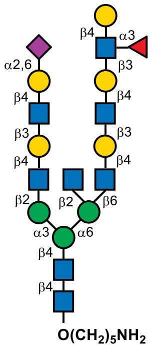	**A20**	**XV** (CMP-Neu5Ac, Mg^{2+}, Pd26ST (A200Y/S232Y), 1.6 mg **A20**)	80% (1.4 mg)	**^{1}H NMR (600 MHz, D_2O)** δ = 5.16 – 5.09 (m, 2H), 4.87 (s, 1H), 4.83 (q, *J* = 6.6 Hz, 1H), 4.77 (s, 1H), 4.74 – 4.68 (m, 3H), 4.62 – 4.53 (m, 4H), 4.51 – 4.41 (m, 6H), 4.28 – 4.24 (m, 1H), 4.24 – 4.18 (m, 2H), 4.18 – 4.13 (m, 3H), 4.12 – 4.06 (m, 1H), 4.04 – 3.37 (m, 101H), 2.67 (dd, *J* = 12.4, 4.6 Hz, 1H), 2.59 (apparent t, *J* = 7.1 Hz, 2H), 2.11 – 1.98 (m, 27H), 1.72 (apparent t, *J* = 12.2 Hz, 1H), 1.59 – 1.51 (m, 2H), 1.43 (apparent quint, *J* = 7.5 Hz, 2H), 1.37 – 1.27 (m, 2H), 1.18 (d, *J* = 6.6 Hz, 3H) ppm.<br><br>**LRMS (ESI-MS)** *m/z* calculated for $C_{134}H_{221}N_{10}O_{93}^{3-}$ $[M\text{-}3H]^{3-}$: 1153.1, found: 1153.2.
**A40**	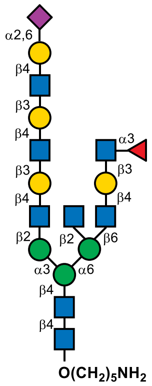	**A8**	**XV** (CMP-Neu5Ac, Mg^{2+}, Pd26ST (A200Y/S232Y), 1.5 mg **A8**)	86% (1.4 mg)	**^{1}H NMR (600 MHz, D_2O)** δ = 5.13 (s, 1H), 5.01 (d, *J* = 4.2 Hz, 1H), 4.87 (s, 1H), 4.76 (s, 1H), 4.75 – 4.68 (m, 3H), 4.62 – 4.52 (m, 4H), 4.52 – 4.42 (m, 5H), 4.34 (q, *J* = 6.8 Hz, 1H), 4.28 – 4.24 (m, 1H), 4.24 – 4.18 (m, 2H), 4.18 – 4.14 (m, 3H), 4.11 – 4.06 (m, 1H), 4.04 – 3.37 (m, 95H), 2.93 (apparent t, *J* = 7.7 Hz, 2H), 2.68 (dd, *J* = 12.3, 4.3 Hz, 1H), 2.11 – 1.96 (m, 27H), 1.72 (apparent t, *J* = 12.1 Hz, 1H), 1.68 – 1.60 (m, 2H), 1.60 – 1.51 (m, 2H), 1.44 – 1.32 (m, 2H), 1.17 (d, *J* = 6.6 Hz, 3H) ppm.

					**LRMS (ESI-MS)** *m/z* calculated for $C_{128}H_{212}N_{10}O_{88}^{2-}$ [M-2H]$^{2-}$: 1649.1, found: 1649.1.
**A41**	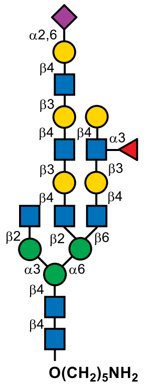	**A10**	**XV** (CMP-Neu5Ac, Mg^{2+}, Pd26ST (A200Y/S232Y), 1.6 mg **A10**)	70-80% (1.3 mg)	**^{1}H NMR (600 MHz, D_2O)** δ = 5.18 – 5.09 (m, 2H), 4.87 (s, 1H), 4.85 – 4.80 (m, 1H), 4.77 (s, 1H), 4.75 – 4.68 (m, 3H), 4.60 (d, *J* = 7.8 Hz, 2H), 4.58 – 4.52 (m, 2H), 4.52 – 4.42 (m, 6H), 4.28 – 4.24 (m, 1H), 4.24 – 4.18 (m, 2H), 4.18 – 4.14 (m, 3H), 4.11 – 4.07 (m, 1H), 4.04 – 3.35 (m, 101H), 2.93 (apparent t, *J* = 7.5 Hz, 2H), 2.68 (dd, *J* = 12.5, 4.7 Hz, 1H), 2.11 – 1.98 (m, 27H), 1.73 (apparent t, *J* = 12.2 Hz, 1H), 1.64 (apparent quint, *J* = 8.0 Hz, 2H), 1.61 – 1.55 (m, 2H), 1.43 – 1.34 (m, 2H), 1.18 (d, *J* = 6.5 Hz, 3H) ppm.<br><br>**LRMS (ESI-MS)** *m/z* calculated for $C_{134}H_{221}N_{10}O_{93}^{3-}$ [M-3H]$^{3-}$: 1153.1, found: 1153.1.
**A42**	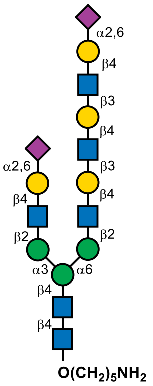	**A13**	**XV** (CMP-Neu5Ac, Mg^{2+}, Pd26ST (A200Y/S232Y), 1.2 mg **A13**)	70-80% (1.1 mg)	**^{1}H NMR (600 MHz, D_2O)** δ = 5.14 (s, 1H), 4.93 (s, 1H), 4.78 (s, 1H), 4.73 (d, *J* = 7.6 Hz, 1H), 4.71 (d, *J* = 8.4 Hz, 1H), 4.63 – 4.55 (m, 3H), 4.53 – 4.41 (m, 5H), 4.29 – 4.24 (m, 1H), 4.22 – 4.18 (m, 1H), 4.18 – 4.14 (m, 2H), 4.14 – 4.08 (m, 1H), 4.05 – 3.44 (m, 89H), 2.92 (apparent t, *J* = 7.6 Hz, 2H), 2.67 (br d, *J* = 12.2 Hz, 2H), 2.11 – 1.97 (m, 24H), 1.73 (apparent t, *J* = 12.2 Hz, 2H), 1.67 – 1.52 (m, 4H), 1.46 – 1.32 (m, 2H) ppm.<br><br>**LRMS (ESI-MS)** *m/z* calculated for $C_{117}H_{193}N_9O_{82}^{2-}$ [M-2H]$^{2-}$: 1518.6, found: 1518.6.
**A43**	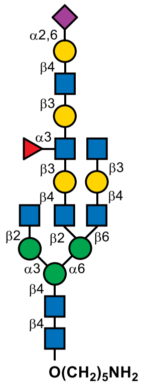	**A11**	**XV** (CMP-Neu5Ac, Mg^{2+}, Pd26ST (A200Y/S232Y), 1.5 mg **A11**)	66% (1.1 mg)	**^{1}H NMR (600 MHz, D_2O)** δ = 5.18 – 5.09 (m, 2H), 4.87 (s, 1H), 4.85 – 4.82 (m, 1H), 4.77 (s, 1H), 4.75 – 4.66 (m, 3H), 4.60 (d, *J* = 7.7 Hz, 2H), 4.58 – 4.52 (m, 2H), 4.52 – 4.42 (m, 5H), 4.27 – 4.18 (m, 3H), 4.18 – 4.14 (m, 2H), 4.12 – 4.06 (m, 2H), 4.05 – 3.37 (m, 95H), 2.97 (apparent t, *J* = 7.7 Hz, 2H), 2.67 (dd, *J* = 12.1, 4.4 Hz, 1H), 2.11 – 1.98 (m, 27H), 1.73 (apparent t, *J* = 12.1 Hz, 1H), 1.66 (apparent quint, *J* = 7.8 Hz, 2H), 1.63 – 1.53 (m, 2H),

					1.43 – 1.35 (m, 2H), 1.16 (d, *J* = 6.4 Hz, 3H) ppm.<br><br>**LRMS (ESI-MS)** *m/z* calculated for $C_{128}H_{212}N_{10}O_{88}^{2-}$ $[M-2H]^{2-}$: 1649.1, found: 1649.3.
**A44**	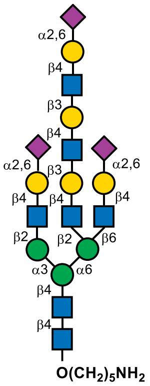	**A15**	**XV**<br>(CMP-Neu5Ac, Mg^{2+}, Pd26ST (A200Y/S232Y), 1.4 mg **A15**)	60%<br>(1.1 mg)	**^{1}H NMR (600 MHz, D_2O)** δ = 5.14 (s, 1H), 4.88 (s, 1H), 4.77 (s, 1H), 4.73 (d, *J* = 7.5 Hz, 1H), 4.71 (d, *J* = 8.1 Hz, 1H), 4.64 – 4.53 (m, 4H), 4.52 – 4.42 (m, 6H), 4.29 – 4.18 (m, 3H), 4.18 – 4.14 (m, 2H), 4.12 – 4.07 (m, 1H), 4.05 – 3.37 (m, 107H), 2.97 (apparent t, *J* = 7.8 Hz, 2H), 2.72 – 2.64 (m, 3H), 2.12 – 1.98 (m, 30H), 1.73 (apparent t, *J* = 12.2 Hz, 3H), 1.66 (apparent quint, *J* = 7.9 Hz, 2H), 1.62 – 1.53 (m, 2H), 1.45 – 1.34 (m, 2H) ppm.<br><br>**LRMS (ESI-MS)** *m/z* calculated for $C_{142}H_{232}N_{11}O_{100}^{3-}$ $[M-3H]^{3-}$: 1230.8, found: 1230.8.
**A45**	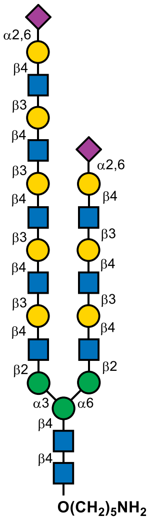	**A9**	**XV**<br>(CMP-Neu5Ac, Mg^{2+}, Pd26ST (A200Y/S232Y), 2.0 mg **A9**)	85%<br>(1.9 mg)	**^{1}H NMR (600 MHz, D_2O)** δ = 5.13 (s, 1H), 4.93 (s, 1H), 4.78 (s, 1H), 4.75 – 4.67 (m, 6H), 4.63 – 4.55 (m, 3H), 4.53 – 4.41 (m, 9H), 4.27 – 4.08 (m, 9H), 4.05 – 3.41 (m, 133H), 2.99 (apparent t, *J* = 7.6 Hz, 2H), 2.68 (dd, *J* = 12.3, 4.6 Hz, 2H), 2.11 – 1.98 (m, 36H), 1.73 (apparent t, *J* = 12.2 Hz, 2H), 1.67 (apparent quint, *J* = 7.7 Hz, 2H), 1.59 (apparent hept, *J* = 6.5 Hz, 2H), 1.43 – 1.35 (m, 2H) ppm.<br><br>**LRMS (ESI-MS)** *m/z* calculated for $C_{173}H_{284}N_{13}O_{122}^{3-}$ $[M-3H]^{3-}$: 1499.2, found: 1499.3.
**A46**	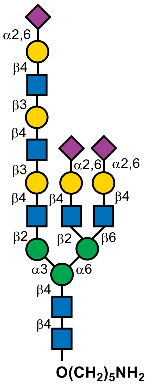	**A7**	**XV**<br>(CMP-Neu5Ac, Mg^{2+}, Pd26ST (A200Y/S232Y), 1.4 mg **A7**)	50-60%<br>(1.0 mg)	**^{1}H NMR (600 MHz, D_2O)** δ = 5.12 (s, 1H), 4.89 (s, 1H), 4.77 (s, 1H), 4.73 (d, *J* = 7.9 Hz, 1H), 4.70 (d, *J* = 8.1 Hz, 1H), 4.63 – 4.53 (m, 4H), 4.52 – 4.41 (m, 6H), 4.29 – 4.24 (m, 1H), 4.24 – 4.18 (m, 2H), 4.18 – 4.14 (m, 2H), 4.12 – 4.07 (m, 1H), 4.05 – 3.34 (m, 107H), 3.00 – 2.91 (m, 2H), 2.68 (dd, *J* = 12.3, 4.7 Hz, 3H), 2.11 – 1.98 (m, 30H), 1.72 (apparent t, *J* = 12.1 Hz, 3H), 1.67 – 1.62 (m, 2H), 1.62 – 1.54 (m, 2H), 1.43 – 1.34 (m, 2H) ppm.

					**LRMS (ESI-MS)** *m/z* calculated for $C_{142}H_{232}N_{11}O_{100}^{3-}$ [M-3H]$^{3-}$: 1230.8, found: 1230.8.
**A47**	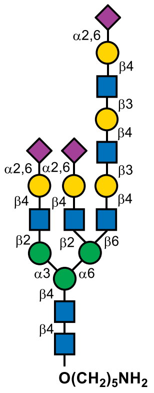	**A16**	**XV** (CMP-Neu5Ac, Mg^{2+}, Pd26ST (A200Y/S232Y), 1.4 mg **A16**)	70-80% (1.4 mg)	**^{1}H NMR (600 MHz, D_2O)** δ = 5.14 (s, 1H), 4.90 (s, 1H), 4.78 (s, 1H), 4.74 (d, *J* = 7.9 Hz, 1H), 4.71 (d, *J* = 8.3 Hz, 1H), 4.64 – 4.57 (m, 3H), 4.55 (d, *J* = 7.9 Hz, 1H), 4.52 – 4.42 (m, 6H), 4.28 – 4.25 (m, 1H), 4.25 – 4.19 (m, 2H), 4.16 (apparent d, *J* = 3.0 Hz, 2H), 4.11 – 4.07 (m, 1H), 4.04 – 3.37 (m, 107H), 2.99 (apparent t, *J* = 7.7 Hz, 2H), 2.68 (dd, *J* = 12.4, 4.9 Hz, 3H), 2.12 – 1.96 (m, 30H), 1.73 (apparent t, *J* = 12.1 Hz, 3H), 1.69 – 1.63 (m, 2H), 1.63 – 1.54 (m, 2H), 1.44 – 1.34 (m, 2H) ppm.<br><br>**LRMS (ESI-MS)** *m/z* calculated for $C_{142}H_{232}N_{11}O_{100}^{3-}$ [M-3H]$^{3-}$: 1230.8, found: 1230.8.
**A48**	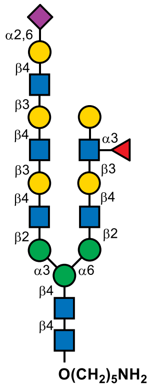	**A17**	**XV** (CMP-Neu5Ac, Mg^{2+}, Pd26ST (A200Y/S232Y), 1.5 mg **A17**)	80-90% (1.4 mg)	**^{1}H NMR (600 MHz, D_2O)** δ = 5.13 (d, *J* = 4.1 Hz, 1H), 5.12 (s, 1H), 4.93 (s, 1H), 4.84 (q, *J* = 6.8 Hz, 1H), 4.77 (s, 1H), 4.75 – 4.67 (m, 3H), 4.63 – 4.55 (m, 3H), 4.52 – 4.42 (m, 6H), 4.27 – 4.23 (m, 1H), 4.21 – 4.14 (m, 4H), 4.13 – 4.08 (m, 1H), 4.06 – 3.44 (m, 96H), 2.99 (apparent t, *J* = 7.6 Hz, 2H), 2.68 (dd, *J* = 12.6, 4.8 Hz, 1H), 2.11 – 1.96 (m, 24H), 1.73 (apparent t, *J* = 12.2 Hz, 1H), 1.67 (apparent quint, *J* = 7.7 Hz, 2H), 1.60 (apparent quint, *J* = 6.7 Hz, 2H), 1.44 – 1.36 (m, 2H), 1.18 (d, *J* = 6.6 Hz, 3H) ppm.<br><br>**LRMS (ESI-MS)** *m/z* calculated for $C_{126}H_{208}N_9O_{88}^{3-}$ [M-3H]$^{3-}$: 1085.4, found: 1085.5.

**A49**	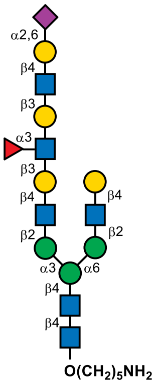	**A37**	**XIII** (UDP-Gal, Mn^{2+}, β4GalT1, 0.7 mg **A37**)	90-95% (0.7 mg)	**^{1}H NMR (600 MHz, D_2O)** δ = 5.16 – 5.09 (m, 2H), 4.93 (s, 1H), 4.84 – 4.81 (m, 1H), 4.77 (s, 1H), 4.75 – 4.68 (m, 2H), 4.63 – 4.55 (m, 3H), 4.52 – 4.42 (m, 5H), 4.25 (d, *J* = 2.2 Hz, 1H), 4.20 – 4.18 (m, 1H), 4.17 – 4.15 (m, 1H), 4.13 – 4.08 (m, 2H), 4.05 – 3.43 (m, 85H), 2.98 (apparent t, *J* = 7.6 Hz, 2H), 2.67 (dd, *J* = 12.5, 4.7 Hz, 1H), 2.11 – 1.98 (m, 21H), 1.73 (apparent t, *J* = 12.1 Hz, 1H), 1.67 (apparent quint, *J* = 7.7 Hz, 2H), 1.63 – 1.55 (m, 2H), 1.44 – 1.35 (m, 2H), 1.16 (d, *J* = 6.5 Hz, 3H) ppm.<br><br>**LRMS (ESI-MS)** *m/z* calculated for $C_{112}H_{186}N_8O_{78}^{2-}$ $[M-2H]^{2-}$: 1446.0, found: 1446.0.
**A50**	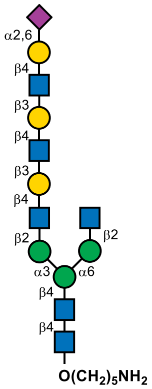	**A37**	**XVII** (Am0392, 0.7 mg **A37**)	Quant. (0.7 mg)	**^{1}H NMR (600 MHz, D_2O)** δ = 5.12 (s, 1H), 4.92 (s, 1H), 4.77 (s, 1H), 4.73 (d, *J* = 7.6 Hz, 1H), 4.70 (d, *J* = 8.0 Hz, 1H), 4.60 (d, *J* = 7.9 Hz, 1H), 4.58 (br d, *J* = 7.5 Hz, 1H), 4.56 (d, *J* = 8.5 Hz, 1H), 4.52 – 4.43 (m, 4H), 4.25 (d, *J* = 1.8 Hz, 1H), 4.19 (d, *J* = 3.1 Hz, 1H), 4.18 – 4.15 (m, 2H), 4.11 (d, *J* = 3.5 Hz, 1H), 4.04 – 3.39 (m, 76H), 2.99 (apparent t, *J* = 7.6 Hz, 2H), 2.68 (dd, *J* = 12.4, 4.6 Hz, 1H), 2.11 – 1.99 (m, 21H), 1.73 (apparent t, *J* = 12.2 Hz, 1H), 1.70 – 1.64 (m, 2H), 1.63 – 1.54 (m, 2H), 1.44 – 1.36 (m, 2H) ppm.<br><br>**LRMS (ESI-MS)** *m/z* calculated for $C_{100}H_{166}N_8O_{69}^{2-}$ $[M-2H]^{2-}$: 1292.0, found: 1292.0.
**A51**	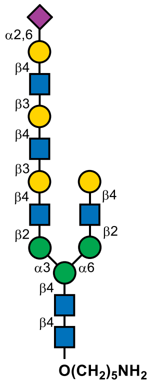	**A49**	**XVII** (Am0392, 0.4 mg **A49**)	Quant. (0.4 mg)	**^{1}H NMR (600 MHz, D_2O)** δ = 5.12 (s, 1H), 4.93 (s, 1H), 4.77 (s, 1H), 4.73 (d, *J* = 7.9 Hz, 1H), 4.70 (d, *J* = 8.4 Hz, 1H), 4.64 – 4.54 (m, 3H), 4.52 – 4.42 (m, 5H), 4.27 – 4.23 (m, 1H), 4.22 – 4.18 (m, 1H), 4.18 – 4.14 (m, 2H), 4.13 – 4.09 (m, 1H), 4.04 – 3.44 (m, 82H), 2.99 (apparent t, *J* = 7.6 Hz, 2H), 2.68 (dd, *J* = 12.2, 4.6 Hz, 1H), 2.11 – 2.00 (m, 21H), 1.73 (apparent t, *J* = 12.2 Hz, 1H), 1.70 – 1.64 (m, 2H), 1.63 – 1.56 (m, 2H), 1.44 – 1.35 (m, 2H) ppm.

					**LRMS (ESI-MS)** *m/z* calculated for $C_{106}H_{176}N_8O_{74}^{2-}$ $[M-2H]^{2-}$: 1373.0, found: 1372.9.

Microarray Analysis

Method

For microarray analysis purpose, 1.0 mM solutions were prepared for all the *N*-glycans (**A1**–**A56**, see Figure S1) and were then diluted to 100 µM using 50 mM PBS buffer. The solutions were then printed on SCHOTT NEXTERION® Slide H (75.6 mm × 25 mm × 1 mm) using MicroGridII (Digilab) equipped with StealthSMP4B microarray pins (Telechem). Each glycan was printed with six duplicates and the slide layout was as below.

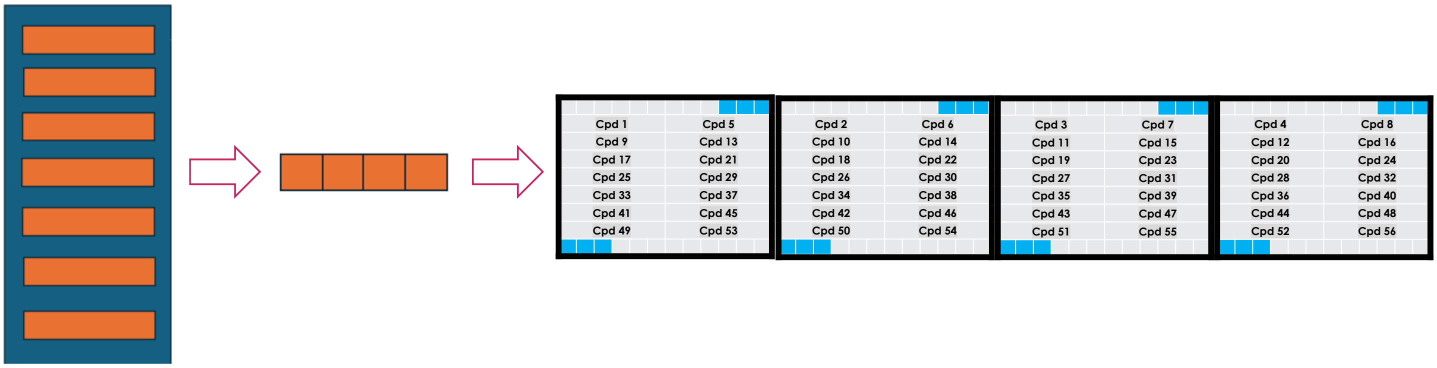

NHS-coated glass slide

All the proteins tested in this assay were purchased from commercial sources, which were summarized in Table S2.

Table S2. Summary of proteins used in microarray assay

Protein	Modification	Source (Catalog No.)
Sambucus Nigra Lectin (SNA)	Biotinylated	Vector Laboratories (B-1305-2)
Ricinus Communis Agglutinin I (RCA-I)	Biotinylated	Vector Laboratories (B-1085-1)
Erythrina Cristagalli Lectin (ECA)	Biotinylated	Vector Laboratories (B-1145-5)
Lycopersicon esculentum (LEL)	Biotinylated	Vector Laboratories (B-1175-1)
IVA hemagglutinin (H1N1, A/California/04/2009)	C-polyHis	Sino Biological (11055-V08H)<br>Accession#: ACP41105
IVA hemagglutinin (H1N1, A/Wisconsin/67/2022)	C-polyHis	Sino Biological (40940-V08H)<br>Accession#: WHO42457
IVA hemagglutinin (H3N2, A/Missouri/09/2014)	C-polyHis	Sino Biological (40494-V08B)<br>Accession#: ALH29486
IVB hemagglutinin (B/Victoria/02/1987)	C-polyHis	Sino Biological (40163-V08B)<br>Accession#: ABL77244
IVB hemagglutinin (B/Yamagata/16/1988)	C-polyHis	Sino Biological (40157-V08B)<br>Accession#: P18880 (for HA1)
Human Siglec-10	$hIgG_1$	R&D System (2130-SL-050)
Streptavidin	Alexa Fluor™ 488	Invitrogen (S32354)
Anti-His_6 mouse IgG_{2a}	–	BioLegend (362616)
Goat anti-mouse IgG	Alexa Fluor™ 488	Invitrogen (A28175)
Goat anti-human IgG Fc secondary antibody	R-phycoerythrin (R-PE)	Invitrogen (12-4998-82)

For analysis purposes, the biotinylated plant lectins (10 ug/mL) were pre-complexed with streptavidin-Alexa Fluor™ 488 (2 ug/mL) at 0 °C in PBS buffer containing 0.05% Tween-20 (PBST) for 15 min; the recombinant influenza hemagglutinins (50 ug/mL) were precomplexed with anti-His_6 mouse IgG_{2a} (25 ug/mL) and goat anti-mouse IgG (12.5 ug/mL) at 0 °C in PBST for 15 min; the recombinant human Siglec-10 (50 ug/mL) was pre-complexed with goat anti-human IgG Fc secondary antibody (25 ug/mL) on ice in PBST for 15 min. The resulting solution was incubated with the individual array for 1-4 hrs at room temperature. The slides were washed twice in PBS-T, PBS, then water, and dried under N_2 flow and the arrays were scanned on an Innoscan 1100AL microarray scanner (Innopsys). Of the six duplicates, the two with the highest and the lowest fluorescence intensities were removed and the remaining four duplicates were used to calculate the mean fluorescence and the standard error of the mean (SEM).

For data analysis purposes, we wish to understand more about how each structural characteristic impacts the binding to the protein. The structural characteristic includes, but not limited to, terminal GlcNAc, terminal Gal, terminal Le^x, terminal Neu5Acα2,3-, terminal Neu5Acα2,3Le^x, terminal Neu5Acα2,6-, terminal fucose, internal fucose, polyLacNAc on MGAT1/2/5 arm, number of antennae, number of LacNAc repeats and number of sialic acid residues. This was done by the following steps:

Step 1. Assign numeric values to each structural characteristic (such as number of terminal GlcNAcs, Gals, internal fucoses, sialic acids, LacNAc repeats, etc.).

New#	Terminal	Mean-Bg	SEM	T_GlcNAc	T_Gal	T_Lex1	T_Lex2	T_S23	T_Slex	T_S26	T_Fuc	Int_Fuc1	MGAT1	MGAT2	MGAT5	Bi	Tri	Antennae	Longest N	Total N	T_Sia/Ant	Total Sia
24	S23	-3.22	8.21619	0	1	0	0	1	0	0	1	0	1	0	0	0	1	3	3	5	0.33	1
25	S23	13831.7	1565.11	0	0	0	0	3	0	0	0	0	1	0	0	0	1	3	3	5	1.00	3
26	S23	-10.338	5.37737	1	0	0	0	1	0	0	0	1	1	0	0	1	0	2	3	3	0.50	1
27	S23	74.6825	50.0224	1	0	0	1	1	0	0	0	0	0	1	0	0	1	3	3	5	0.33	1
28	S23	28.845	22.7851	1	0	1	0	1	0	0	0	0	0	0	1	0	1	3	3	5	0.33	1
29	S23	-29.568	18.1161	2	0	0	0	1	0	0	0	1	0	1	0	0	1	3	3	4	0.33	1
30	S23	4788.81	692.713	0	0	0	0	2	0	0	0	0	1	0	0	1	0	2	3	4	1.00	2
31	S23	2767.67	477.423	0	0	0	0	2	0	0	0	0	0	1	0	1	0	2	3	4	1.00	2
32	S23	22873.5	1732.4	0	0	0	0	3	0	0	0	0	0	1	0	0	1	3	3	5	1.00	3
33	S23	35686.9	5322.94	0	0	0	0	3	0	0	0	0	0	0	1	0	1	3	3	5	1.00	3
34	S23	1174.7	292.492	0	0	0	1	1	0	0	0	0	1	0	0	1	0	2	3	5	0.50	1
35	S23Lex	253.855	81.6455	1	0	0	0	0	1	0	0	0	1	0	0	1	0	2	3	3	0.50	1
36	S26	46.8175	13.6227	1	0	0	0	0	0	1	1	0	1	0	0	0	1	3	3	4	0.33	1
37	S26	2677.89	433.286	1	0	0	0	0	0	1	0	1	1	0	0	1	0	2	3	3	0.50	1
38	S26	13822.4	1449.34	0	0	0	0	0	0	2	0	0	1	0	0	1	0	2	3	4	1.00	2
39	S26	-15.798	8.82314	1	0	1	0	0	0	1	0	0	0	0	1	0	1	3	3	5	0.33	1
40	S26	227.155	84.668	1	0	0	0	0	0	1	1	0	1	0	0	0	1	3	3	4	0.33	1
41	S26	222.68	58.3896	1	0	0	1	0	0	1	0	0	0	1	0	0	1	3	3	5	0.33	1
42	S26	20122	4490.44	0	0	0	0	0	0	2	0	0	0	1	0	1	0	2	3	4	1.00	2
43	S26	-26.583	47.0374	2	0	0	0	0	0	1	0	1	0	1	0	0	1	3	3	4	0.33	1
44	S26	26409.7	855.589	0	0	0	0	0	0	3	0	0	0	1	0	0	1	3	3	5	1.00	3
45	S26	26678.4	1625.98	0	0	0	0	0	0	2	0	0	1	0	0	1	0	2	5	8	1.00	2
46	S26	25617.9	4064.02	0	0	0	0	0	0	3	0	0	1	0	0	0	1	3	3	5	1.00	3
47	S26	39965.5	405.047	0	0	0	0	0	0	3	0	0	0	0	1	0	1	3	3	5	1.00	3
48	S26	1453.42	168.085	0	0	0	1	0	0	1	0	0	1	0	0	1	0	2	3	5	0.50	1
49	S26	1402.62	310.52	0	1	0	0	0	0	1	0	1	1	0	0	1	0	2	3	4	0.50	1
50	S26	1459.97	220.739	1	0	0	0	0	0	1	0	0	1	0	0	1	0	2	3	3	0.50	1
51	S26	1376.13	185.768	0	1	0	0	0	0	1	0	0	1	0	0	1	0	2	3	4	0.50	1
52	S26	-19.313	25.1777	0	0	0	0	0	0	2	0	0	1	1	0	1	0	2	1	2	1.00	2
53	S26	0.6375	12.8661	0	0	0	0	0	0	1	0	1	0	0	0	0	0	1	3	3	1.00	1
54	S26	-14.53	3.45132	0	1	0	0	0	0	1	0	0	0	1	0	1	0	2	1	2	0.50	1
55	S26	1187.9	75.0918	0	1	0	0	0	0	1	0	0	1	0	0	1	0	2	1	2	0.50	1
56	S26	2482.7	226.517	0	0	0	0	0	0	1	0	0	0	0	0	0	0	1	3	3	1.00	1

Step 2. Establish a Pearson correlation between the fluorescence vector (column 'Mean-Bg') and a vector of any given structural characteristic, and calculate the corresponding Pearson Correlation Coefficient *r*. In Excel, this could be easily done using the implemented function *r* = PEARSON (vector_1, vector_2).

Step 3. Perform the *t*-test for each Pearson correlation and calculate the *t* value by using the following equation:

$t = \frac{r\sqrt{n-2}}{\sqrt{1-r^2}}$, where r is the Pearson Correlation Coefficient, and n is the size of the samples (e.g., 56 total glycans, 35 sialosides, 21 α2,6-sialosides, 28 bi-antennary glycans, 26 tri-antennary glycans, etc.).

Step 4. Calculate the p-value using the Excel implemented function $p = T.DIST.2T(|t|, n-2)$, where t is the t value calculated in Step 3, and n is the sample size. The Pearson correlation is established if and only if the p-value is significantly small.

Results for the Microarray Assays

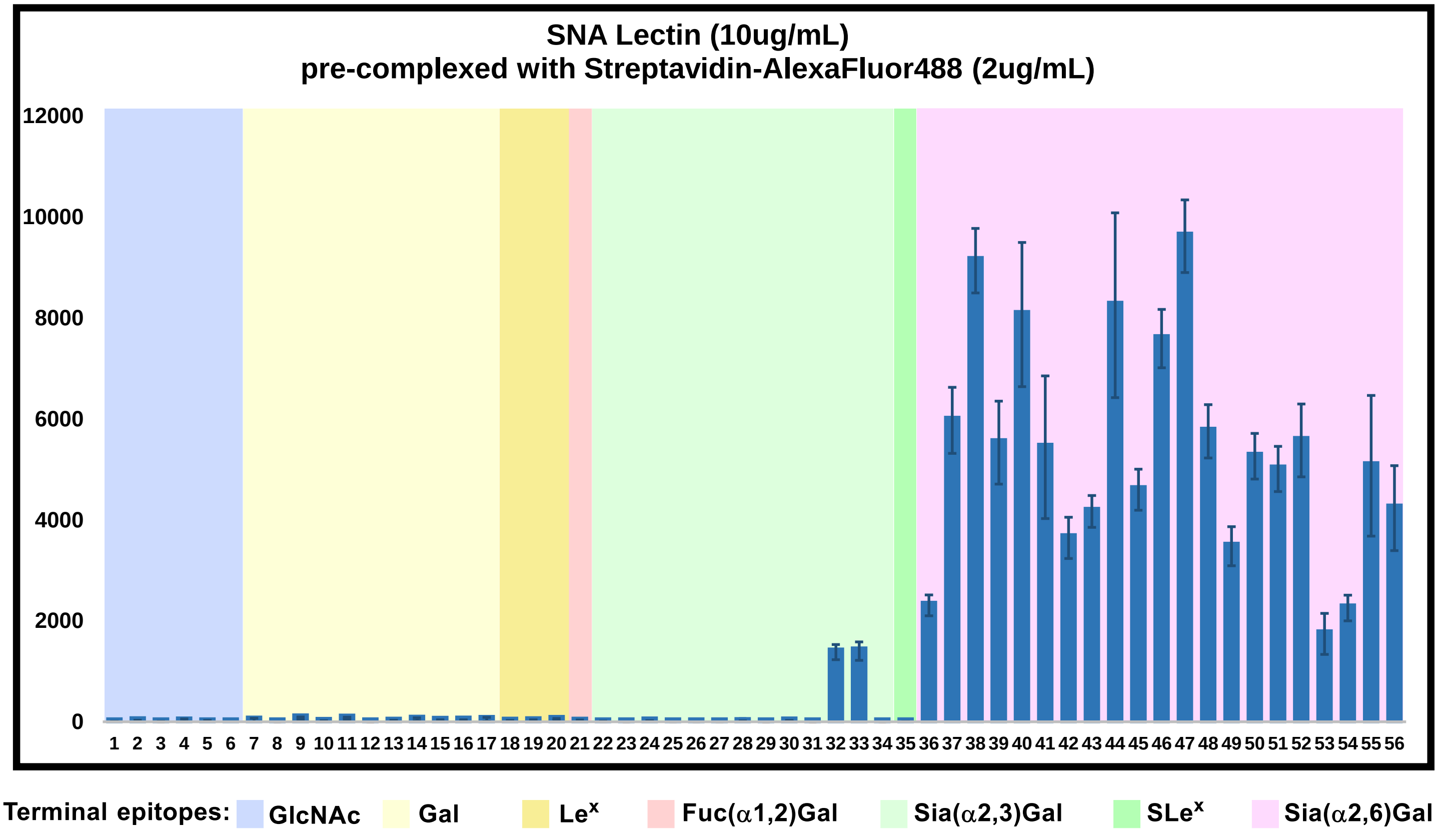

Figure S2. SNA binding to the glycan array.

Statistical results:

Mean-Bg	SEM	T_GlcNAc	T_Gal	T_Lex1	T_Lex2	T_S23	T_Slex	T_S26	T_Fuc	Int_Fuc1	MGAT1	MGAT2	MGAT5	Bi	Tri	Antennae	Longest N	Total N	T_Sia/Ant	Total Sia
S26 (21)	r	-0.0783	-0.32119	0.017119	0.034918	-	-	0.616185	-0.0273	-0.3468	0.153979	-0.14163	0.335581			0.454463	0.127307	0.291268	0.252352	0.616185
	T	-0.34237	-1.47834	0.074632	0.152296	-	-	3.410208	-0.11906	-1.61172	0.67928	-0.62366	1.55281			2.223885	0.559471	1.32715	1.136769	3.410208
	p	0.735828	0.155703	0.941287	0.880559	-	-	0.002936	0.906481	0.123511	0.505152	0.540268	0.136967			0.038479	0.582376	0.200188	0.269769	0.002936

S26-Biante (11)	r	0.596775	-0.45518
	T	2.23119	-1.53362
	p	0.052594	0.15949

S26-Triante (8)	r	-0.13052	-0.14253	0.305287
	T	-0.32248	-0.35273	0.785287
	p	0.758032	0.736349	0.462158

SNA lectin binds preferentially to multi-sialylated glycans (p = 0.00294) and recognizes MGAT1 arm better than MGAT2 arm on a bi-antennary structure (p = 0.0526, or compare **A38** vs **A42**).

*Glycans **A32** and **A33** also showed some weak binding to SNA due to a minor incorporation of α2,6-sialosides (~15% judged by ^{1}H NMR) by multifunctional sialyltransferase PmST1 (M144D) from *Pasteurella multocida*.

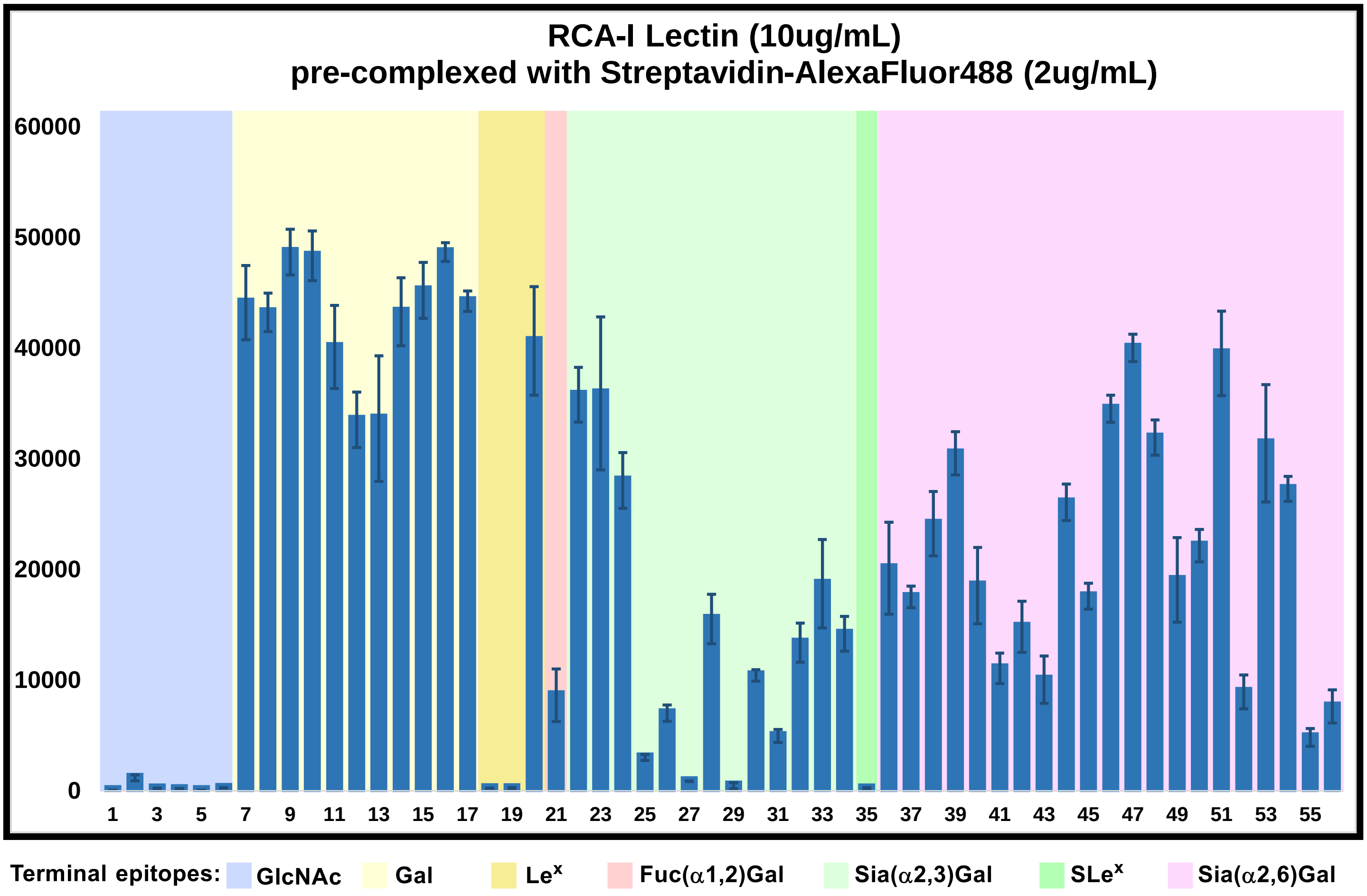

Figure S3. RCA-I binding to the glycan array

Statistical results:

		T_GlcNAc	T_Gal	T_Lex1	T_Lex2	T_S23	T_Slex	T_S26	T_Fuc	Int_Fuc1	MGAT1	MGAT2	MGAT5	Antennea	Longest N	Total N	T_Sia/Ant	Total Sia
All(56)	*r*	-0.47264	0.668912	0.014105	0.015067	-0.2574	-0.17323	0.100216	0.048753	-0.02579	-0.01815	-0.12049	0.160955	0.151241	0.340439	0.442583	-0.13248	-0.10917
	T	-3.94118	6.612673	0.103662	0.110734	-1.95743	-1.29249	0.74016	0.358685	-0.18957	-0.13341	-0.89195	1.198394	1.124321	2.66063	3.626867	-0.98216	-0.80702
	p	0.000235	1.75E-08	0.917822	0.912238	0.055474	0.201691	0.462409	0.721229	0.850354	0.894368	0.376377	0.235997	0.26585	0.010248	0.000637	0.330403	0.423196

RCA-I lectin recognizes terminal Gal (or LacNAc) much better than any other motifs. However, it also recognizes capped LacNAc to some extent, but this recognition is somehow compromised by terminal residues according to the following order: GlcNAc = Le^x (almost completely blocked in both cases) > Fucose(α1,2)- ≈ Neu5Ac(α2,3)- > Neu5Acα(2,6)-. In addition, RCA-I favors long polyLacNAc motifs ($p = 0.00064$).

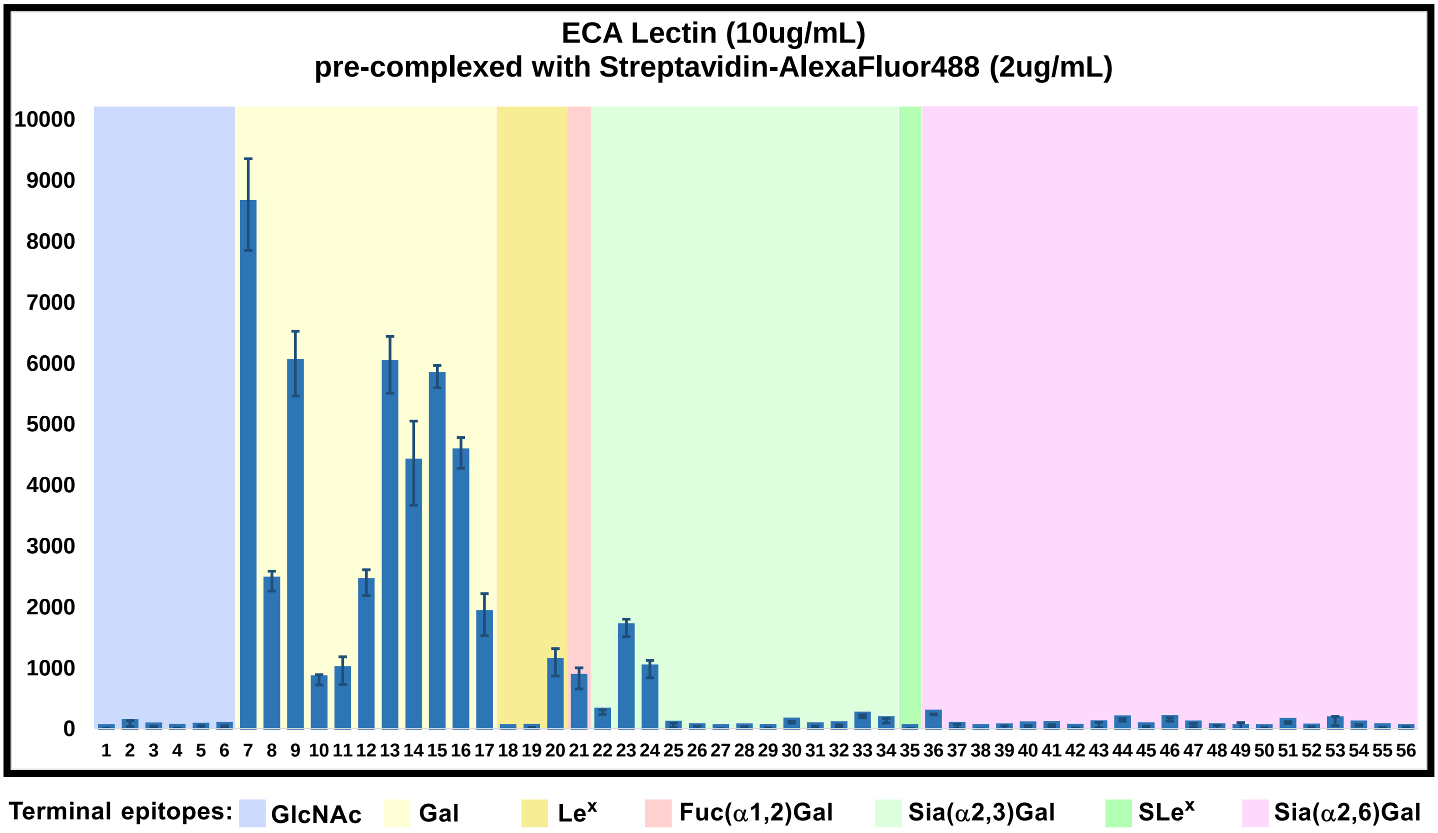

Figure S4. ECA binding to the glycan array

Statistical results:

		T_GlcNAc	T_Gal	T_Lex1	T_Lex2	T_S23	T_Slex	T_S26	T_Fuc	Int_Fuc1	MGAT1	MGAT2	MGAT5	Bi	Tri	Antennea	Longest N	Total N	T_Sia/Ant	Total Sia
T_Gal(17)	*r*	-0.29225	0.829501	-0.15616	-0.19295	-0.16703		-0.57103	-0.14174	-0.28423	-0.01271	0.003125	0.014995			0.161609	0.472239	0.49613	-0.61571	-0.62262
	T	-1.18355	5.752215	-0.61231	-0.76161	-0.65613		-2.69404	-0.55456	-1.14819	-0.04923	0.012104	0.058081			0.634244	2.074912	2.21308	-3.02632	-3.08158
	p	0.255008	3.82E-05	0.549501	0.4581	0.521677		0.016657	0.58737	0.268871	0.961387	0.990502	0.954451			0.535475	0.055615	0.042812	0.008504	0.007598

ECA only recognizes terminal Gal, but not terminal Le^x. The binding affinity is sensitive to both the number of terminal Gals ($p < 0.0001$) and the length of the LacNAc repeats ($p = 0.0428$).

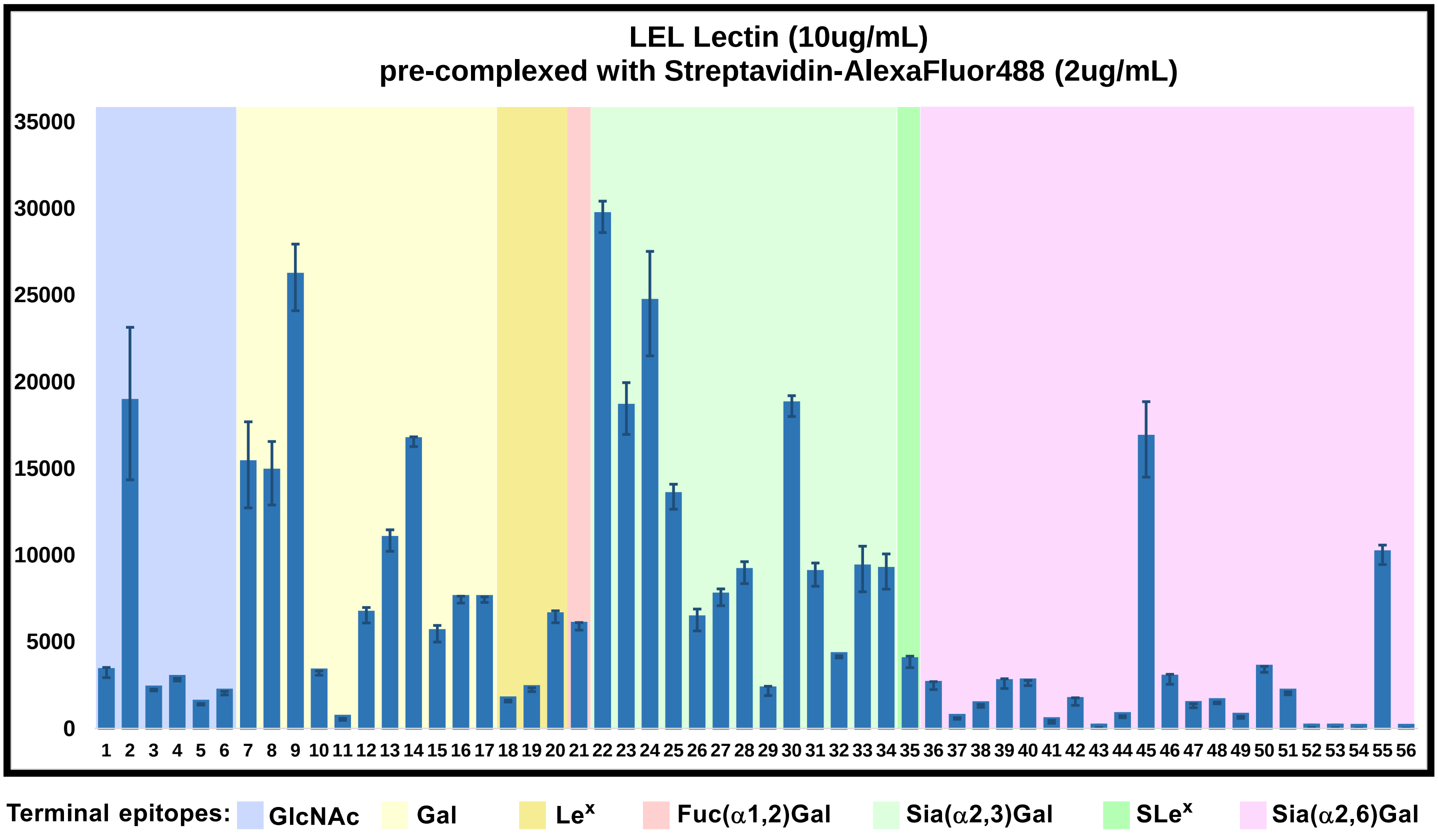

Figure S5. LEL binding to glycan array.

Statistical results:

		T_GlcNAc	T_Gal	T_Lex1	T_Lex2	T_S23	T_Slex	T_S26	T_Fuc	Int_Fuc1	MGAT1	MGAT2	MGAT5	Bi	Tri	Antennea	Longest N	Total N	T_Sia/Ant	Total Sia
All (56)	*r*	-0.24536	0.254948	-0.06991	-0.11738	0.264954	-0.05391	-0.3781	0.18047	-0.26277	0.372264	-0.32125	-0.06743			-0.14639	0.548322	0.590163	-0.07568	-0.12264
	T	-1.85983	1.937499	-0.51497	-0.8686	2.019173	-0.39676	-3.00125	1.348315	-2.00129	2.947413	-2.49284	-0.49667			-1.08743	4.818223	5.372075	-0.55773	-0.90808
	p	0.06836	0.057922	0.608677	0.388909	0.048445	0.693105	0.004064	0.183186	0.050398	0.004724	0.015771	0.621441			0.281678	1.22E-05	1.69E-06	0.579331	0.367873
									Bi-ante (28)	*r*	0.174959	-0.24727								
										T	0.906094	-1.30122								
										p	0.373207	0.204596								
									Tri-ante (26)	*r*	0.543778	-0.46369	-0.07465							
										T	3.174289	-2.56389	-0.36673							
										p	0.004087	0.017036	0.71703							

LEL lectin binds to all LacNAc motifs, no matter if it is capped or not. However, terminal capping weakens the binding according to the following order: Neu5Ac(α2,6)- > Le^x > GlcNAc, while the terminal Neu5Ac(α2,3)- seems to improve the binding moderately (p = 0.0484). LEL favors long LacNAc repeats ($p < 0.0001$) and recognizes MGAT1 better than the other two antennae on tri-antennary structures (p = 0.0041).

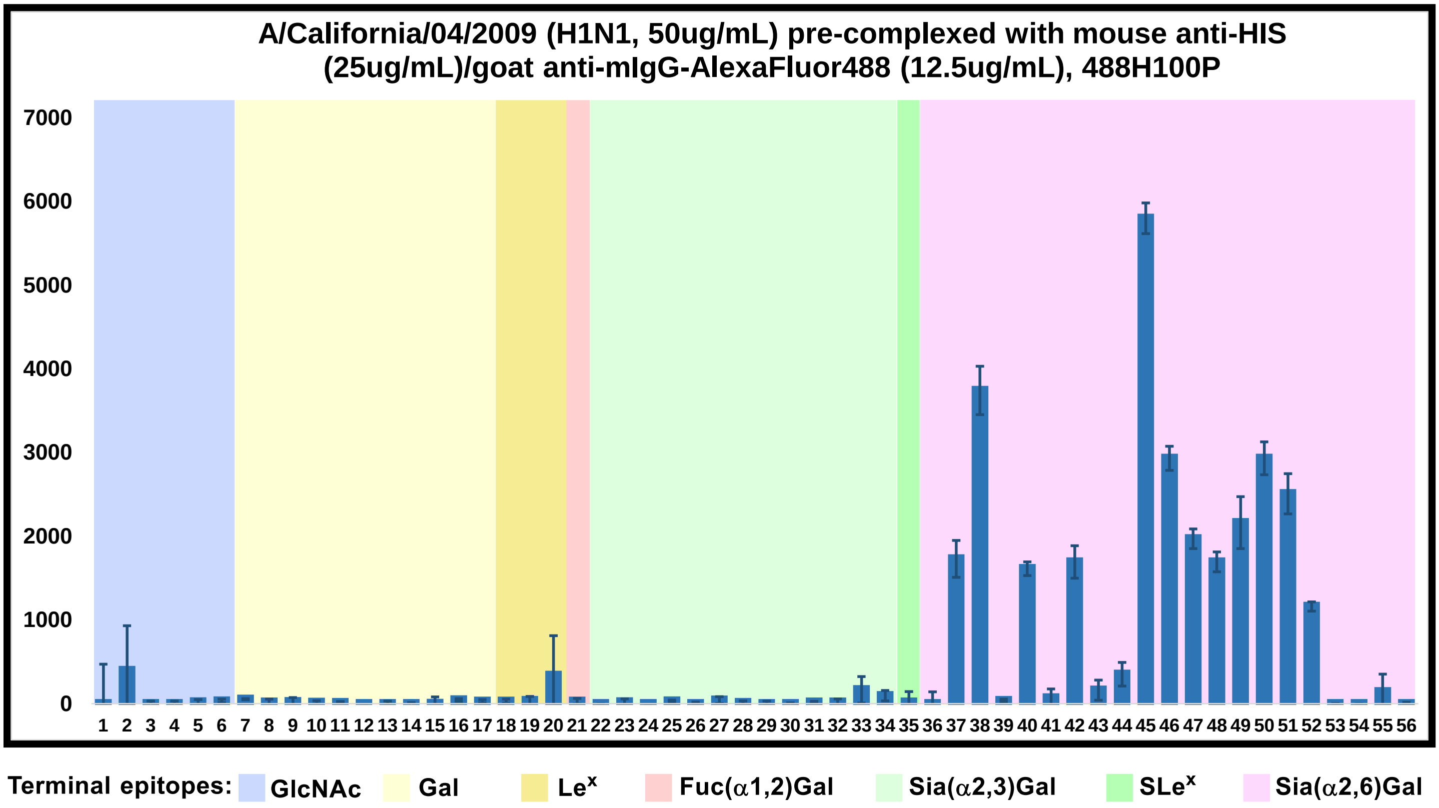

Figure S6. HA (H1N1, A/California/04/2009) binding to glycan array.

Statistical results:

		T_GlcNAc	T_Gal	T_Lex1	T_Lex2	T_S23	T_Slex	T_S26	T_Fuc	Int_Fuc1	MGAT1	MGAT2	MGAT5	Bi	Tri	Antennea	Longest N	Total N	T_Sia/Ant	Total Sia
S26 (21)	*r*	-0.27239	-0.08482	-0.21026	-0.12377			0.35871	-0.14117	-0.14428	0.567747	-0.3735	-0.09754			-0.07988	0.551597	0.541354	0.344242	0.35871
	T	-1.234	-0.37107	-0.93747	-0.54367			1.675058	-0.62157	-0.63557	3.006246	-1.75506	-0.42721			-0.34931	2.882536	2.806524	1.598197	1.675058
	p	0.232249	0.714688	0.360282	0.592993			0.110303	0.541609	0.532638	0.007261	0.095362	0.674032			0.730695	0.009538	0.011262	0.126497	0.110303

		MGAT1	MGAT2
Bi-ante S26 (11)	*r*	0.394912	-0.46943
	T	1.289554	-1.59496
	p	0.229357	0.145185

		MGAT1	MGAT2	MGAT5
Tri-ante S26 (8)	*r*	0.456094	-0.51167	0.062132
	T	1.255373	-1.45873	0.152485
	p	0.25602	0.194919	0.883803

Influenza A HA (H1N1, A/California/04/2009) favors long LacNAc repeats rather than short sequences (p = 0.0095).

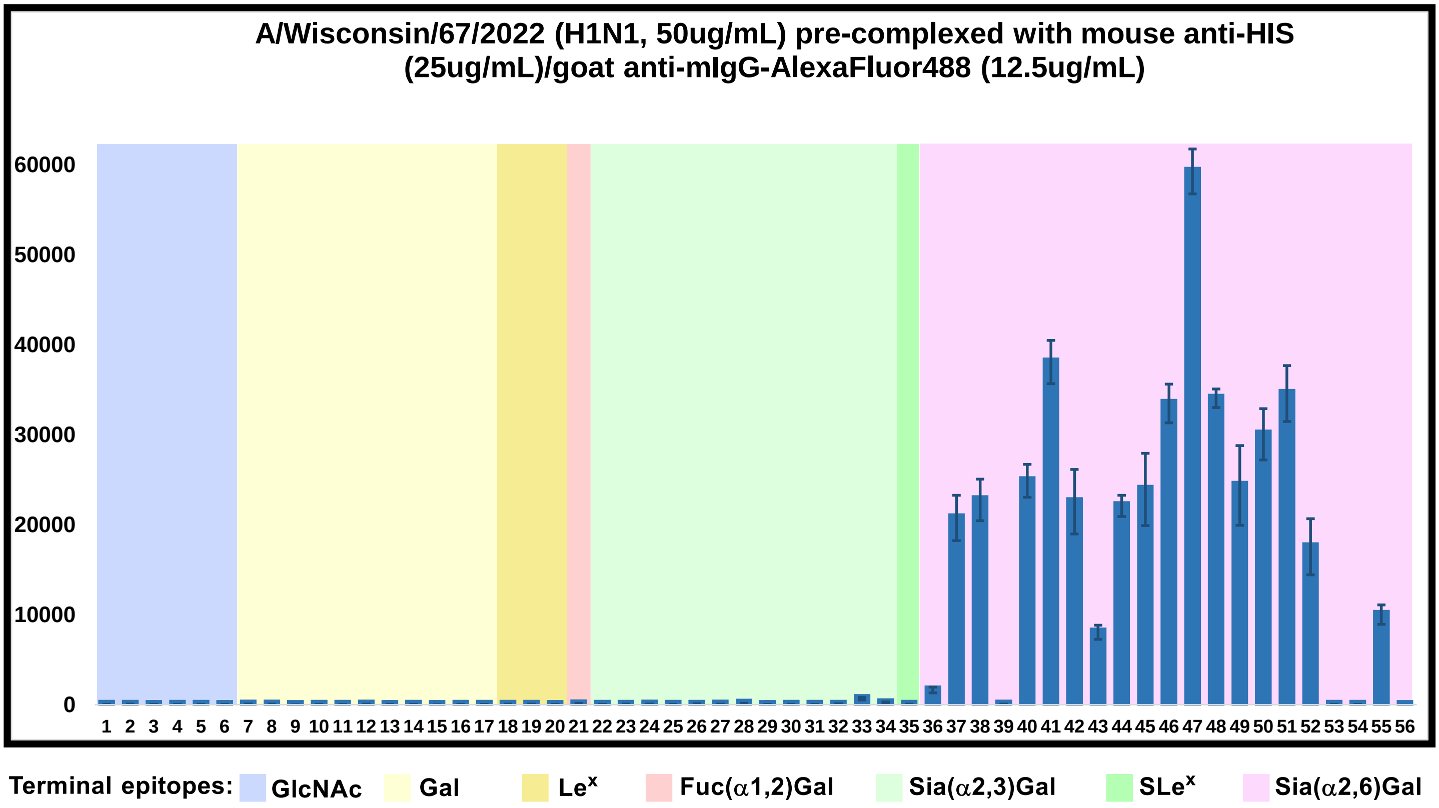

Figure S7. HA (H1N1, A/Wisconsin/67/2022) binding to glycan array.

Statistical results:

		T_GlcNAc	T_Gal	T_Lex1	T_Lex2	T_S23	T_Slex	T_S26	T_Fuc	Int_Fuc1	MGAT1	MGAT2	MGAT5	Bi	Tri	Antennae	Longest N	Total N	T_Sia/Ant	Total Sia
S26	*r*	-0.1722	-0.10044	-0.30007	0.335645	-	-	0.483766	-0.15264	-0.22689	0.212219	-0.0971	0.198596			0.326663	0.273711	0.412954	0.151518	0.483766
	T	-0.76197	-0.44003	-1.37117	1.553145	-	-	2.409382	-0.67324	-1.01549	0.946603	-0.42524	0.883254			1.506539	1.240449	1.976416	0.668165	2.409382
	p	0.455431	0.664882	0.1863	0.136887	-	-	0.026287	0.508901	0.322629	0.355726	0.675438	0.388138			0.148375	0.22991	0.062808	0.512064	0.026287

Bi-ante S26 (11)	*r*	0.516631	-0.5389	
	T	1.810183	-1.91925	
	p	0.103705	0.087161	
Tri-ante S26 (8)	*r*	-0.14111	-0.02869	0.189842
	T	-0.34914	-0.0703	0.473628
	p	0.738909	0.946238	0.652507

Influenza A HA (H1N1, A/Wisconsin/67/2022) favors multi-sialylated *N*-glycans ($p = 0.0263$). On the bi-antennary structures, it recognizes MGAT1 better than MGAT2 ($p \approx 0.1$).

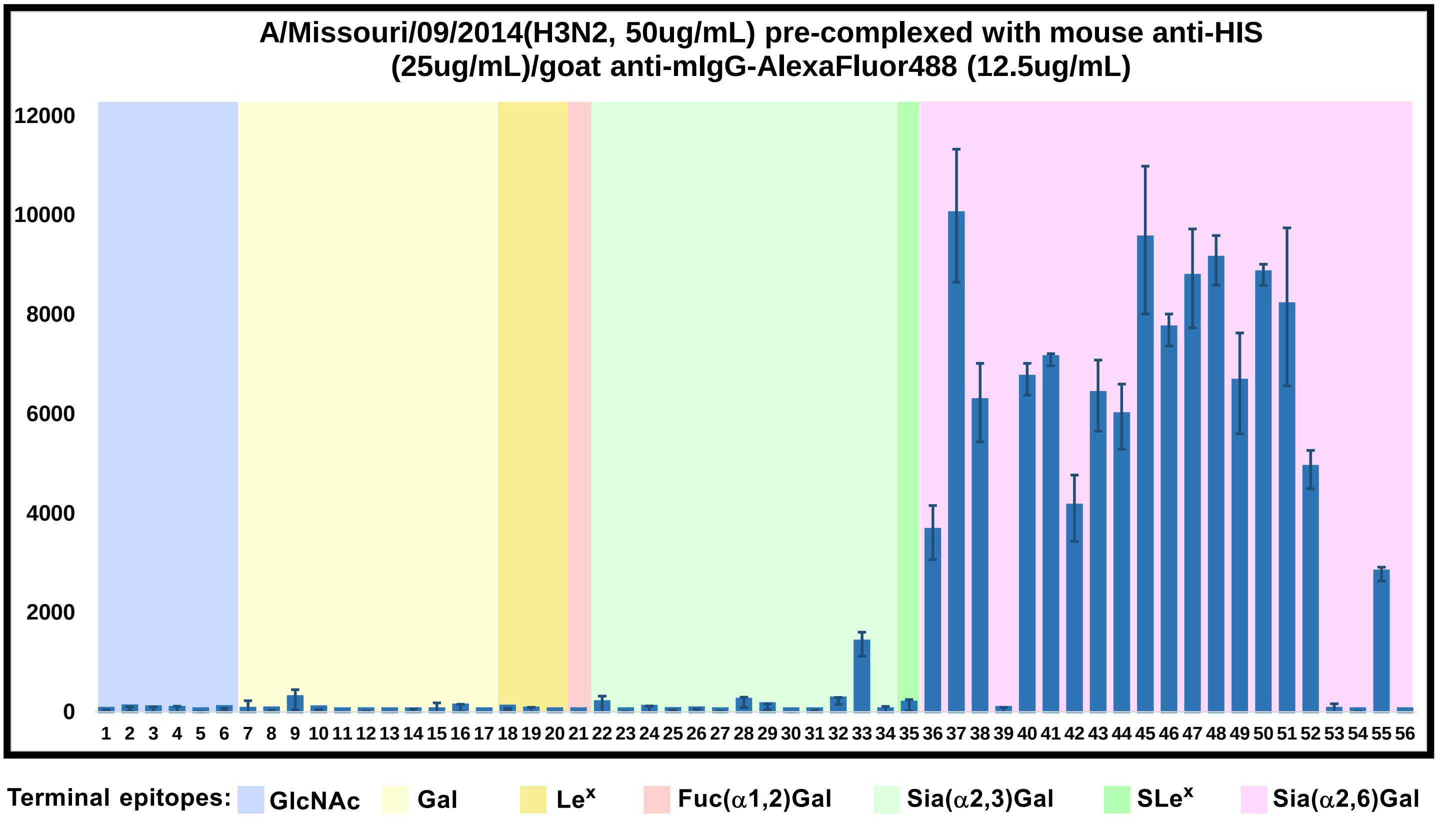

Figure S8. HA (H3N2, A/Missouri/09/2014) binding to glycan array.

Statistical results:

		Int_Fuc1	MGAT1	MGAT2	MGAT5	Bi	Tri	Antennea	Longest N	Total N	T_Sia/Ant	Total Sia
S26 (21)	*r*	0.031447	0.519595	-0.15592	-0.11475			0.302656	0.442556	0.461576	-0.03841	0.281016
	T	0.137144	2.650782	-0.68804	-0.50351			1.384166	2.151187	2.268022	-0.16753	1.276354
	p	0.89236	0.015774	0.499742	0.620394			0.182352	0.044539	0.035179	0.868724	0.217212

		MGAT1	MGAT2
Bi-ante S26 (11)	*r*	0.675288	-0.68576
	T	2.746734	-2.8266
	p	0.022597	0.019832

		MGAT1	MGAT2	MGAT5
Tri-ante S26 (8)	*r*	0.069159	0.209864	-0.31196
	T	0.169812	0.525768	-0.80427
	p	0.870739	0.617906	0.45192

Influenza A HA (H3N2, A/Missouri/09/2014) favors long polyLacNAc sequence ($p = 0.0445$) and recognizes MGAT1 arm better on bi-antennary structures ($p = 0.0226$).

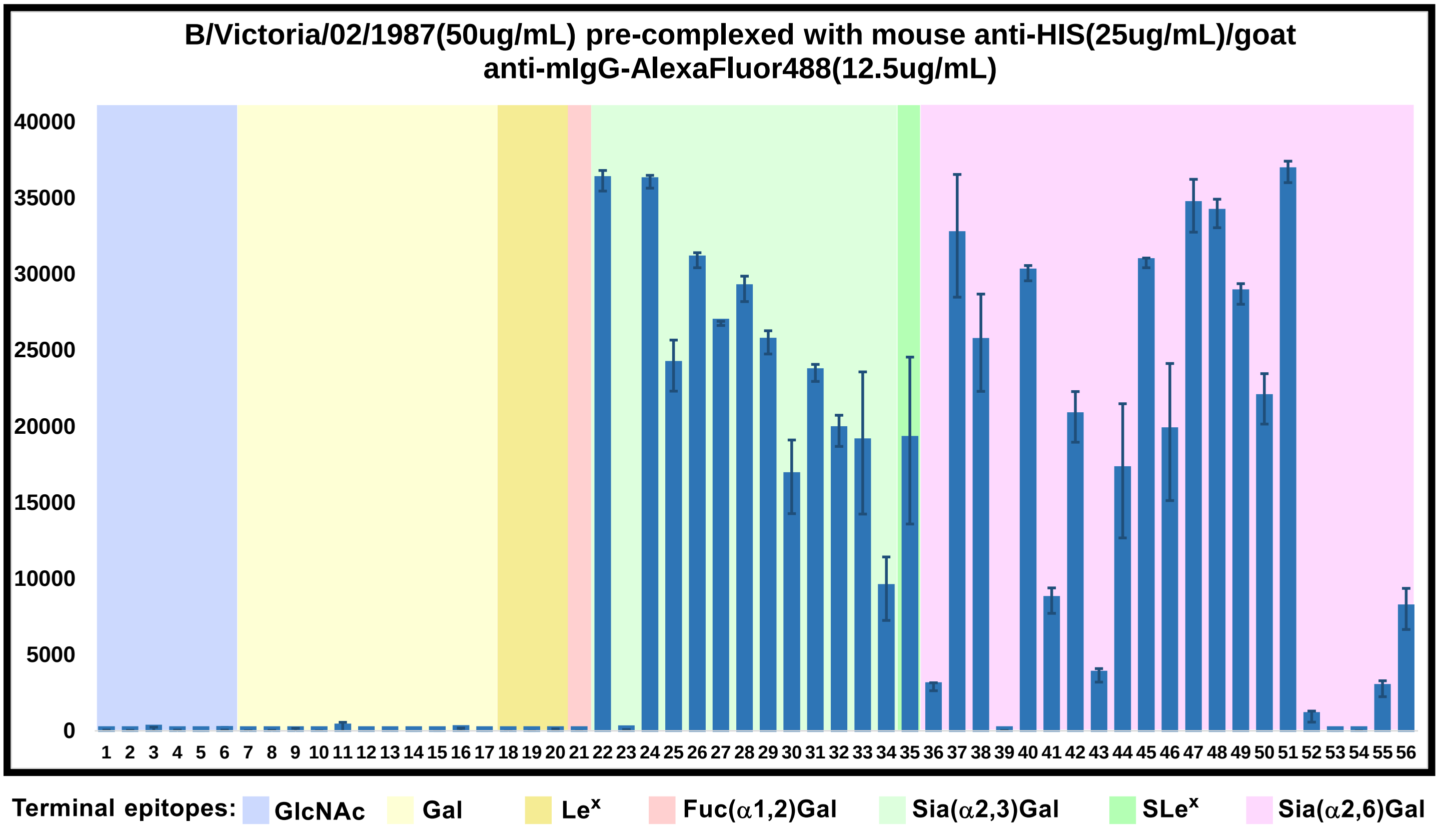

Figure S9. HA (B/Victoria/02/1987) binding to glycan array

Statistical results:

		T_GlcNAc	T_Gal	T_Lex1	T_Lex2	T_S23	T_Slex	T_S26	T_Fuc	Int_Fuc1	MGAT1	MGAT2	MGAT5	Bi	Tri	Antennea	Longest N	Total N	T_Sia/Ant	Total Sia
T_Sia (35)	r	-0.1128	0.05245	-0.0957	0.01189	0.18031	-0.0026	-0.0471	-0.0585	0.03449	0.25237	-0.2403	0.0397			0.15147	0.51273	0.44873	0.03568	0.15298
	T	-0.6523	0.3017	-0.5522	0.06829	1.05307	-0.0152	-0.2708	-0.3367	0.19826	1.49826	-1.4221	0.22824			0.88027	3.43066	2.88448	0.2051	0.88928
	p	0.5187	0.76478	0.58453	0.94597	0.29996	0.98798	0.7882	0.73846	0.84406	0.14357	0.16438	0.82087			0.38508	0.00164	0.00686	0.83876	0.38029
									Bi-ante	r	0.2767	-0.4923								
									T_Sia	T	1.11518	-2.1907								
									(17)	*p*	0.28232	0.04468								
									Tri-ante	r	0.0171	-0.1087	0.10238							
									T_Sia	T	0.06399	-0.409	0.38508							
									(16)	*p*	0.94989	0.68872	0.70597							

Influenza B HA (B/Victoria/02/1987) prefers long LacNAc repeats rather than short sequence ($p = 0.0016$).

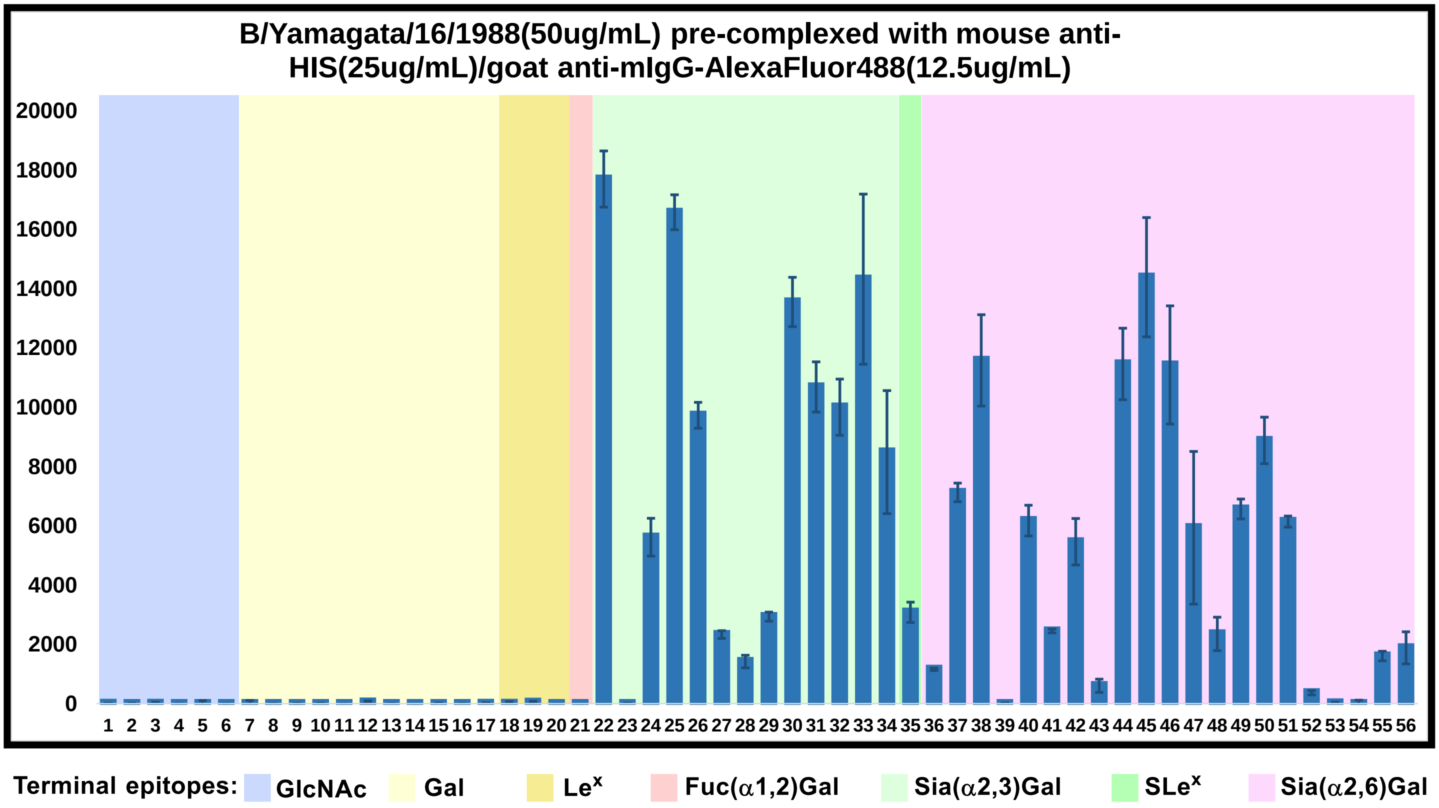

Figure S10. HA (B/Yamagata/16/1988) binding to glycan array.

Statistical results:

		T_GlcNAc	T_Gal	T_Lex1	T_Lex2	T_S23	T_Slex	T_S26	T_Fuc	Int_Fuc1	MGAT1	MGAT2	MGAT5	Bi	Tri	Antennea	Longest N	Total N	T_Sia/Ant	Total Sia
T_Sia (35)	r	-0.4199	-0.18565	-0.26716	-0.16863	0.547823	-0.10765	-0.00965	-0.21518	-0.16164	0.285268	-0.20892	-0.06395			0.075176	0.532765	0.539313	0.505054	0.595199
	T	-2.65783	-1.08532	-1.59258	-0.98276	3.761684	-0.62199	-0.05544	-1.26574	-0.94091	1.709787	-1.22723	-0.3681			0.43308	3.61649	3.679017	3.361548	4.25492
	p	0.01203	0.285646	0.120788	0.332877	0.000658	0.53822	0.956119	0.214468	0.35359	0.096694	0.228418	0.715153			0.667774	0.000985	0.000828	0.001972	0.000162
									Bi-ante (17)	r	0.199521	-0.37906								
										T	0.788598	-1.58651								
										p	0.442633	0.133476								
									Tri-ante (16)	r	0.155287	-0.12008	-0.03937							
										T	0.588165	-0.45256	-0.14741							
										p	0.56579	0.657802	0.884907							

Influenza B HA (B/Yamagata/16/1988) prefers long LacNAc repeats over the short ones ($p = 0.001$) and binds better to multi-sialylated glycans ($p = 0.0002$). More specifically, the binding affinity is more correlated to the number of Neu5Ac(α2,3)- residues ($p = 0.00066$).

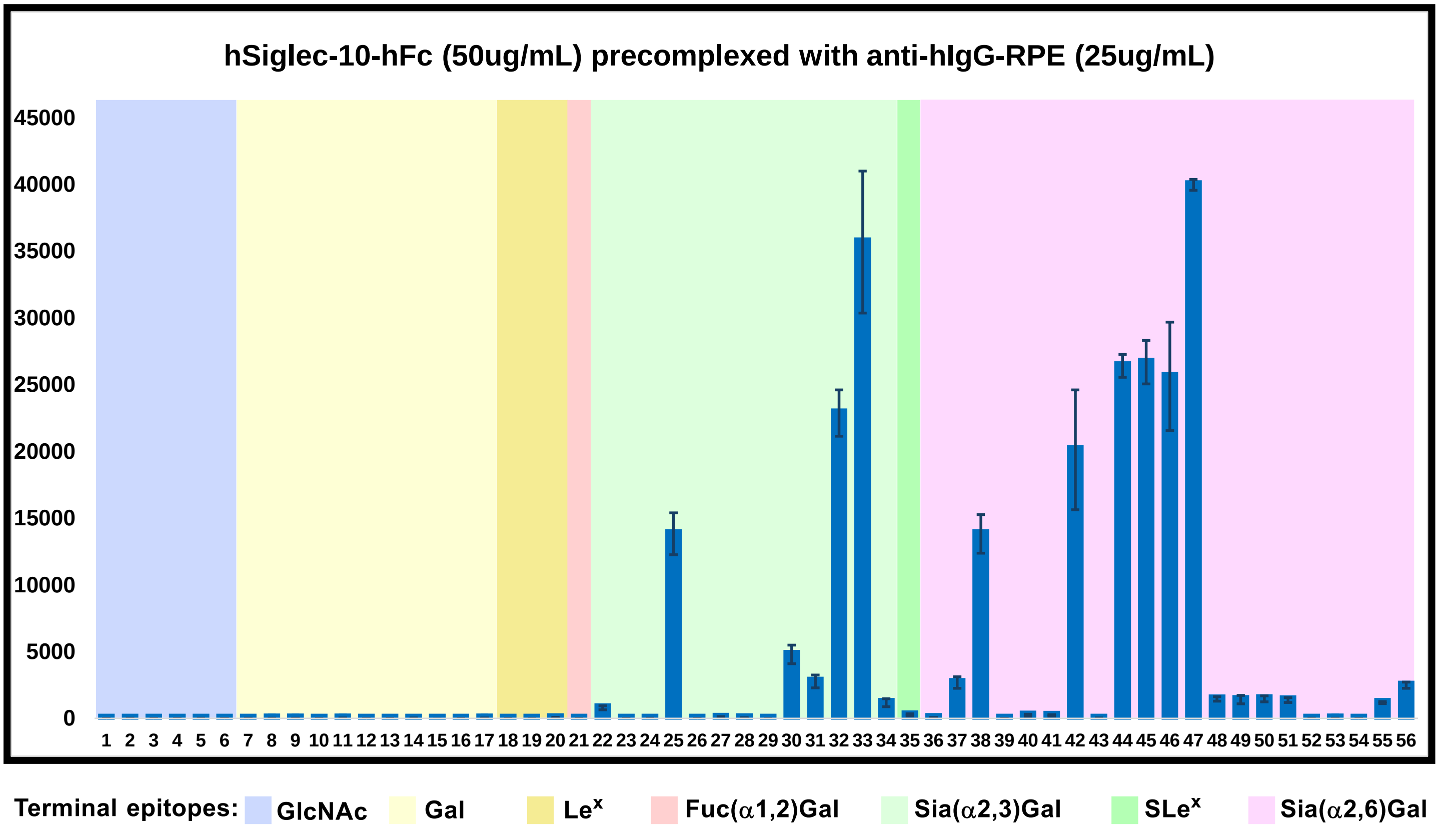

Figure S11. Human Siglec-10 binding to glycan array.

Statistical results:

		T_GlcNAc	T_Gal	T_Lex1	T_Lex2	T_S23	T_Slex	T_S26	T_Fuc	Int_Fuc1	MGAT1	MGAT2	MGAT5	Bi	Tri	Antennae	Longest N	Total N	T_Sia/Ant	Total Sia
S(35)	r	-0.42265	-0.22457	-0.15232	-0.19944	0.20805	-0.1024	0.519684	-0.22046	-0.25502	-0.22555	0.009575	0.373152			0.26717	0.221789	0.388798	0.649345	0.858323
	T	-2.679	-1.32388	-0.88537	-1.16916	1.221891	-0.59132	3.494266	-1.29836	-1.51509	-1.32998	0.055006	2.31048			1.592668	1.306623	2.424201	4.904978	9.609444
	p	0.011425	0.194641	0.382366	0.250716	0.230406	0.558332	0.001377	0.203163	0.139271	0.192646	0.956465	0.027254			0.12077	0.200374	0.020984	2.44E-05	4.35E-11

bi-ante (17)	r	-0.17818	0.07399
	T	-0.7013	0.287349
	p	0.493851	0.777777

tri-ante (16)	r	-0.20078	-0.11169	0.349355
	T	-0.76687	-0.42054	1.395071
	p	0.455897	0.680472	0.184733

Human Siglec-10 binds more strongly to multi-sialylated *N*-glycans ($p < 0.0001$).

Table S3. Summary of factors that affect the protein bindings towards *N*-glycans[a,b,c]

	Protein	Recognition Element	Antennary Favoritism		# of LacNAc	Sia Density	hampered by	Facilitated by
			Bi-antennary	Tri-antennary				
Plant Lectins	SNA	T_Neu5Acα2,6-	MGAT1*	–	–	Positive***	–	–
	RCA	T_Galactoseβ1,4-	–	–	Positive***	–	T_GlcNAc***<br>T_Neu5Acα2,3-*	–<br>–
	ECA	T_Galactoseβ1,4-	–	–	Positive**	Negative***	T_Neu5Acα2,6-**	–
	LEL	Type-II LacNAc	–	MGAT1***<br>MGAT2**<br>MGAT5 (–)	Positive***	–	T_Neu5Acα2,6-***<br>Int_Fuc**<br>T_GlcNAc*	T_Neu5Acα2,3-**
Influenza A/B HAs	H1N1(CA/04/2009)	T_Neu5Acα2,6-	–	–	Positive***	–	–	–
	H1N1(WI/67/2022)	T_Neu5Acα2,6-	MGAT1*	–	–	Positive**	–	–
	H3N2(MO/09/2014)	T_Neu5Acα2,6-	MGAT1**	–	Positive**	–	–	–
	B(Victoria/02/1987)	T_Neu5Acα2,3/6-	–	–	Positive***	–	–	–
	B(Yamagata/16/1988)	T_Neu5Acα2,3/6-	–	–	Positive***	Positive***	T_GlcNAc**	–
Siglec	hSiglec-10	T_Neu5Acα2,3/6-	–	MGAT5*	–	Positive***	T_GlcNAc**	–

[a]Pearson correlation coefficients (*r*-values) were calculated between each factor and fluorescence intensities. The significance was judged by *p*-values generated from the *t*-tests.
[b]Abbreviations: T_: terminal; Int_: internal;
[c]Red colored square: positive correlation; blue colored square: negative correlation. The darkness of color is proportionate to the observed Pearson correlation coefficient *r*.
$^{*}p < 0.1$
$^{**}p < 0.05$
$^{***}p < 0.01$

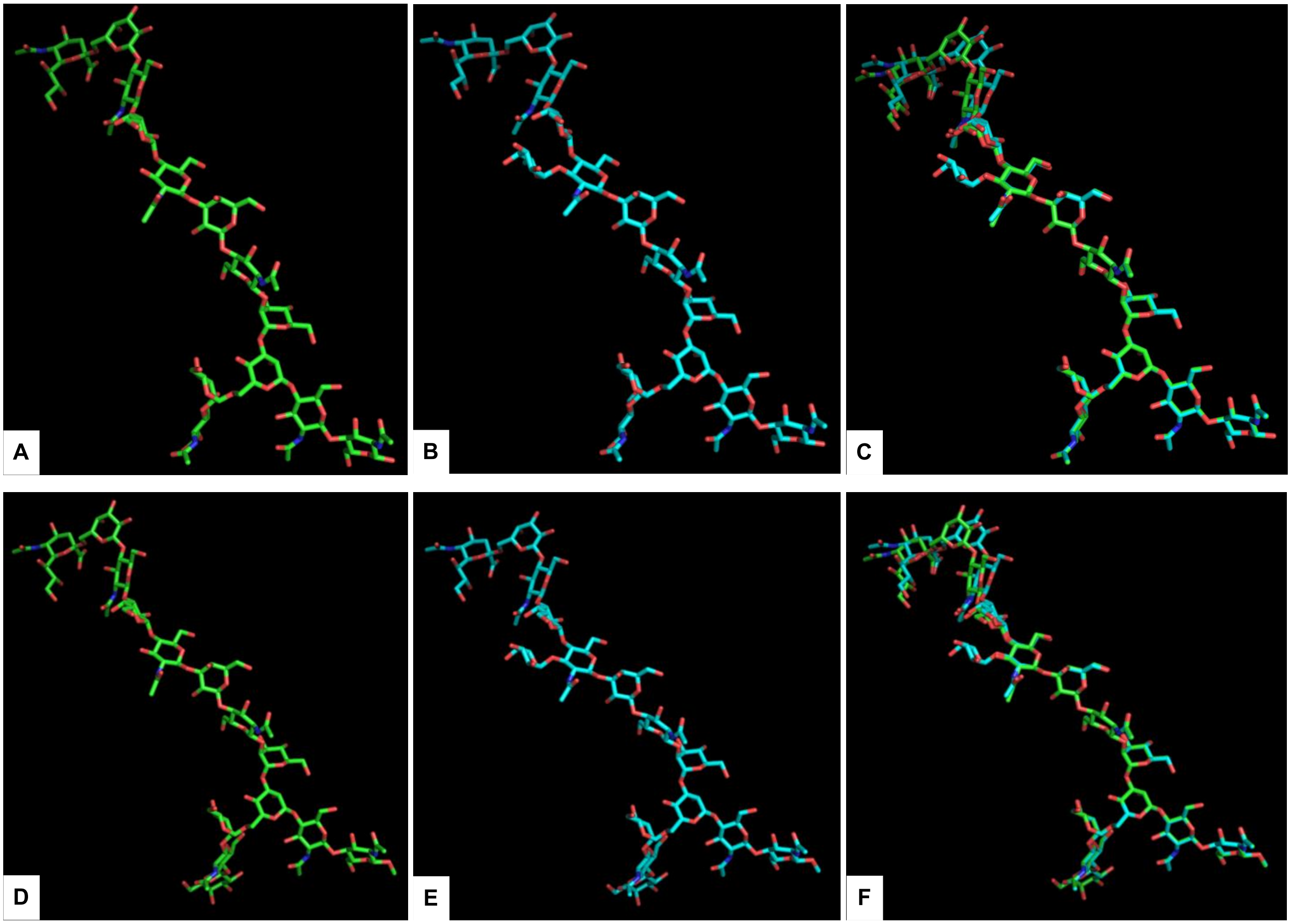

Figure S12. Comparison between conformations of fucosylated/afucosylated *N*-glycan pairs **A26**/**A50** and **A49**/**A51**. All the conformations were generated using GLYCAM06 force-field energy minimization based on a set of the following conformational selections: Man(α1,6)Man: ω_*gauche-gauche*; Neu5Ac(α2,6)Gal: φ_*trans*/ω_ *gauche-gauche*. The optimized conformers for afucosylated *N*-glycans **A50** (A) and **A51** (D), fucosylated *N*-glycans **A26** (B) and **A49** (E) and super-imposed conformers for **A26**/**A50** (C) and **A49**/**A51** (F) were displayed using PyMOL.

References

1. Boeggeman, E. E.; Balaji, P. V.; Sethi, N. Masibay, A. S.; Qasba, P. K. Expression of deletion constructs of bovine β-1,4-galactosyltransferase in *Escherichia coli*: importance of Cys134 for its activity. *Protein Eng. Des. Sel.* **1993**, *6*, 779.
2. Yu, H.; Yu, H.; Karpel, R.; Chen, X. Chemoenzymatic synthesis of CMP-sialic acid derivatives by a one-pot two-enzyme system: comparison of substrate flexibility of three microbial CMP-sialic acid synthetases. *Bioorg. Med. Chem.* **2004**, *12*, 6427.
3. Takakura, Y.; Tsukamoto, H.; Yamamoto, T. Molecular cloning, expression and properties of an alpha/beta-Galactoside alpha2,3-sialyltransferase from Vibrio sp. JT-FAJ-16. *J. Biochem.* **2007**, *142*, 403.
4. Sugiarto, G.; Lau, K.; Qu, J.; Li, Y.; Lim, S.; Mu, S.; Ames, J. B.; Fisher, A. J.; Chen, X. A sialyltransferase mutant with decreased donor hydrolysis and reduced sialidase activities for directly sialylating Lewis^x. *ACS Chem. Biol.* **2012**, *7*, 1232.
5. Tsukamoto, H.; Takakura, Y.; Mine, T.; Yamamoto, T. *Photobacterium* sp. JT-ISH-224 produces two sialyltransferases, α-/β-galactoside α2,3-sialyltransferase and β-galactoside α2,6-sialyltransferase. *J. Biochem.* **2008**, *143*, 187.
6. Ortiz-Soto, M. E.; Seibel, J. Expression of Functional Human Sialyltransferases ST3Gal1 and ST6Gal1 in *Escherichia coli*. *PLOS ONE* **2016**, *11*, e0155410.
7. Xu, Y.; Fan, Y.; Ye, J.; Wang, F.; Nie, Q.; Wang, L.; Wang, P. G.; Cao, H.; Cheng, J. Successfully engineering a bacterial sialyltransferase for regioselective α2,6-sialylation. *ACS Catal.* **2018**, *8*, 7222.
8. Kelly, R. J.;Rouquier, S.; Giorgi, D.; Lennon, G. G.; Lowe, J. B. Sequence and expression of a candidate for the human Secretor blood group alpha(1,2)fucosyltransferase gene (FUT2). Homozygosity for an enzyme-inactivating nonsense mutation commonly correlates with the non-secretor phenotype. *J. Biol. Chem.* **1995**, *270*, 4640.
9. Shuoker, B.; Pichler, M. J.; Jin, C.; Sakanaka, H.; Wu, H.; Gascueña, A. M.; Liu, J.; Nielsen, T. S.; Holgersson, J.; Karlsson, E. N.; Juge, N.; Meier, S.; Morth, J. P.; Karlsson, N. G.; Hachem, M. A. Sialidases and fucosidases of *Akkermansia muciniphila* are crucial for growth on mucin and nutrient sharing with mucus-associated gut bacteria. *Nat. Commun.* **2023**, *14*, 1833.
10. Crich, D.; Li, W.; Li, H. Direct chemical synthesis of the β-mannans: linear and block syntheses of the alternating β-(1→3)-β-(1→4)-mannan common to *Rhodotorula glutinis*, *Rhodotorula mucilaginosa*, and *Leptospira biflexa*. *J. Am. Chem. Soc.* **2004** *126*, 15081.
11. Shivatare, S. S.; Chang, S.-H.; Tsai, T.-I.; Ren, C.-T.; Chuang, H.-Y.; Hsu, L.; Lin, C.-W.; Li, S.-T.; Wu, C.-Y.; Wong, C.-H. Efficient convergent synthesis of bi-, tri-, and tetra-antennary complex type *N*-glycans and their HIV-1 antigenicity. *J. Am. Chem. Soc.* **2013**, *135*, 15832.
12. Valerio, S.; Pastore, A.; Adinolfi, M.; Iadonisi, A. Sequential one-pot glycosidations catalytically promoted: unprecedented strategy in oligosaccharide synthesis for the straightforward assemblage of the antitumor PI-88 pentasaccharide. *J. Org. Chem.* **2008**, *73*, 4496.
13. Shih, H.-W.; Chen, K.-T.; Cheng, T.-J.; Wong, C.-H.; Cheng, W.-C. A new synthetic approach toward bacterial transglycosylase substrates, lipid II and lipid IV. *Org. Lett.* **2011**, *13*, 4600.

14. Mondal, P. K.; Liao, G.; Mondal, M. A.; Guo, Z. Chemical synthesis of the repeating unit of Type Ia group B *Streptococcus* capsular polysaccharide. *Org. Lett.* **2015**, *17*, 1102.
15. Serna, S.; Kardak, B.; Reichardt, N.-C.; Martin-Lomas, M. Synthesis of a core trisaccharide building block for the assembly of *N*-glycan neoconjugates. *Tetrahedron: Asymmetry* **2009**, *20*, 851.
16. Bartetzko, M. P.; Schuhmacher, F.; Hahm, H. S.; Seeberger, P. H.; Pfrengle, F. Automated glycan assembly of oligosaccharides related to arabinogalactan proteins. *Org. Lett.* **2015**, *17*, 4344.
17. Zhang, Z.; Ollmann, I. R.; Ye, X.-S.; Wischnat, R.; Baasov, T.; Wong, C.-H. Programmable one-pot oligosaccharide synthesis. *J. Am. Chem. Soc.* **1999**, *121*, 734.
18. Niu, Y.; Wang, N.; Cao, X.; Ye, X.-S. Efficient formation and cleavage of benzylidene acetals by sodium hydrogen sulfate supported on silica gel. *Synlett* **2007**, *13*, 2116.
19. Verma, N.; Tu, Z.; Lu, M.-S.; Liu, S.-H.; Renata, S.; Phang, R.; Liu, P.-K.; Ghosh, B.; Lin, C.-H. Threshold of thioglycoside reactivity difference is critical for efficient synthesis of Type I oligosaccharides by chemoselective glycosylation. *J. Org. Chem.* **2021**, *86*, 892.

NMR Spectra

The anomeric signals of the oligosaccharides were assigned on the HSQC spectra according to chemical shifts, ^3J(HH) and ^1J(CH) coupling constant values.

Coupling constant values		α-linkage	β-linkage
^3J(HH) values	Galactosyl	–	8-9 Hz
	Glucosaminyl	–	7-9 Hz
	Fucosyl	3-4 Hz	–
	Mannosyl	1-1.5 Hz	0-1 Hz
^1J(CH) values	Galactosyl	–	158-163 Hz
	Glucosaminyl	–	162-168 Hz
	Fucosyl	168-170 Hz	–
	Mannosyl	168-172 Hz	155-158 Hz

For example, anomeric region of hexasaccharide **42** was assigned as follows:

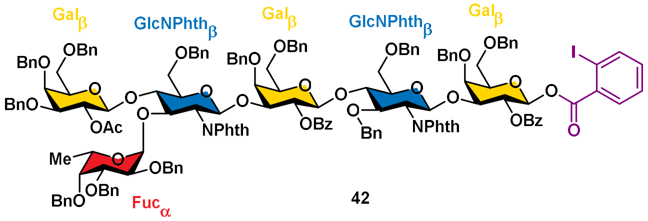

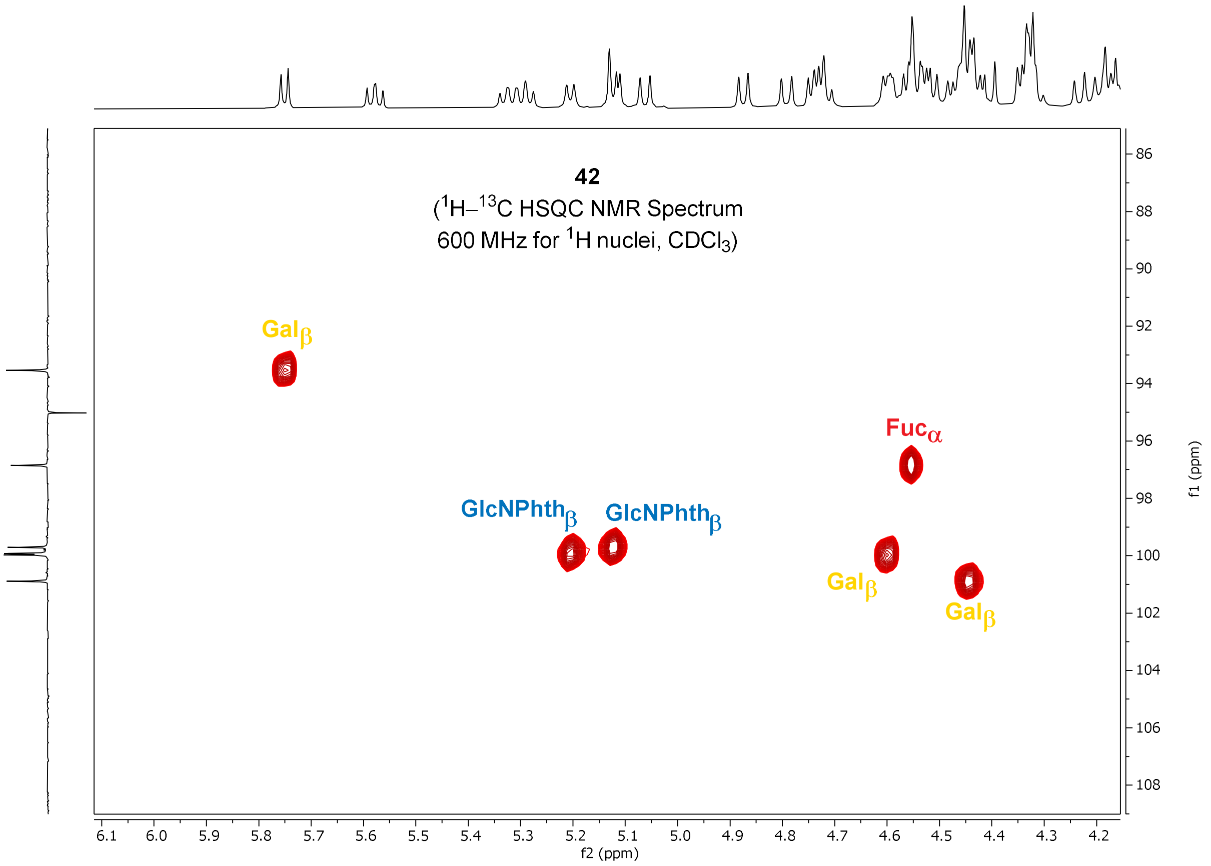

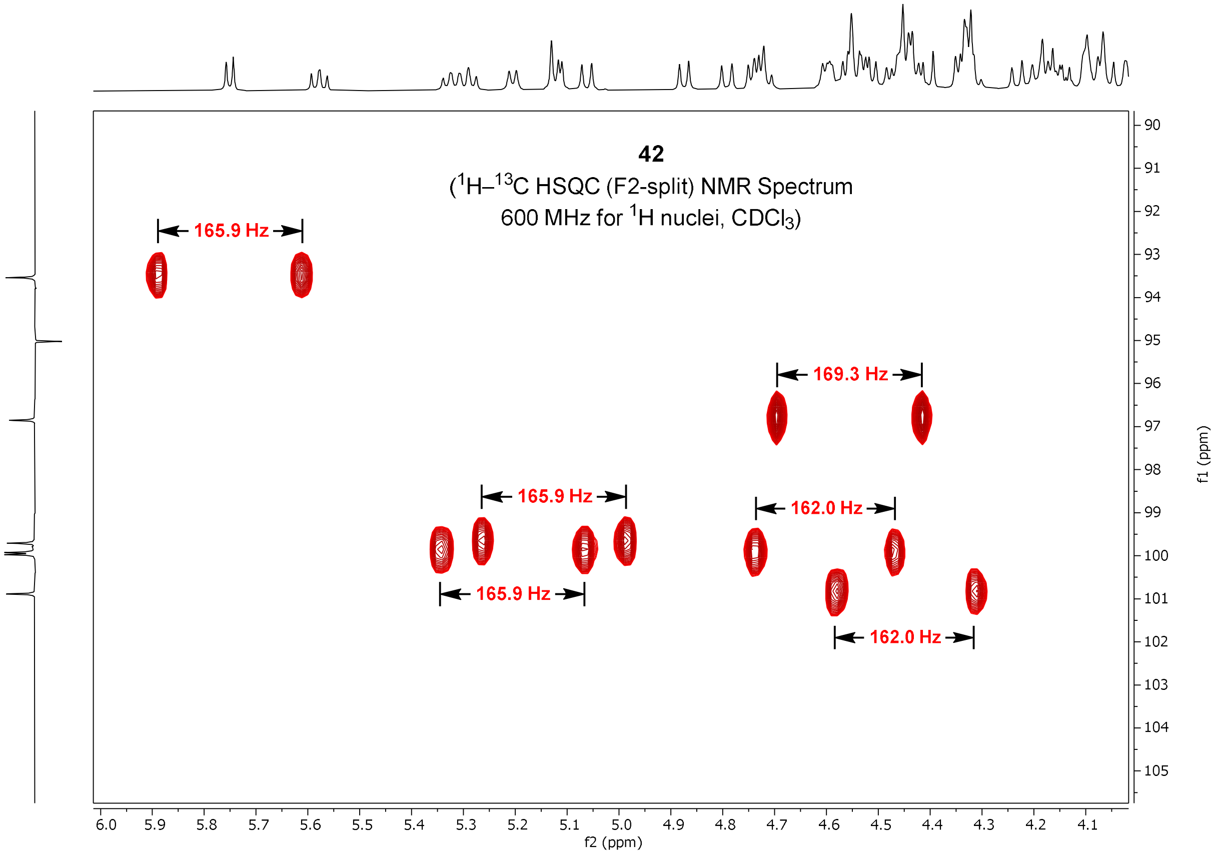

The most down-field (F2-dimension) anomeric signal ($^3J_{HH}$ = 8.2 Hz) corresponds to galactosyl benzoate, with $^1J_{CH}$ significantly enlarged to 165.9 Hz (larger than normal galactosyl anomeric $^1J_{CH}$ value due to inductive effect of an ester linkage) and ^{13}C chemical shift shrank below 95 ppm (indicating a glycosyl ester). Phth-protected β-linked glucosaminyl anomeric signal normally falls in the range of 5.0-5.2 ppm, with $^1J_{CH}$ value ranging from 162-168 Hz and $^3J_{HH}$ value around 8 Hz. An α-linked fucosyl anomeric signal has a $^1J_{CH}$ value between 168-170 Hz, and the signal seems more intensive than other anomeric signals. Galactosyl anomeric signals are usually located at high-field region (4.2-4.7 ppm), with relatively small $^1J_{CH}$ (158-163 Hz) values compared to those of other anomeric signals.

Unprotected oligosaccharides were assigned in a similar manner and for *N*-glycans, the locations of anomeric signals on HSQC spectra are rather conservative for a certain sugar type.

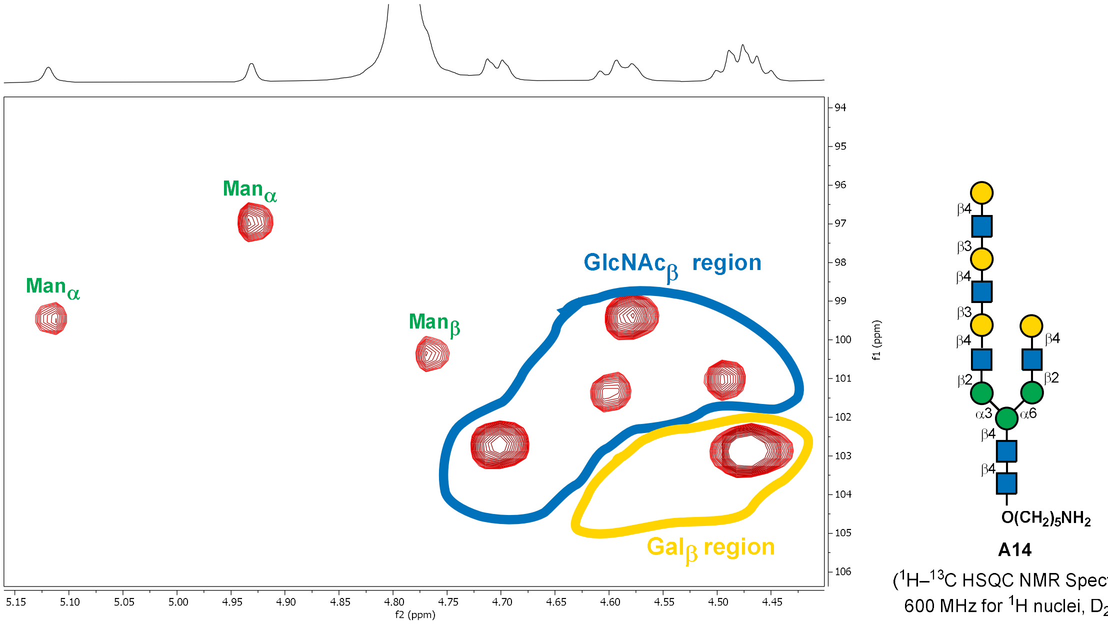

A14
(^{1}H–^{13}C HSQC NMR Spectrum
600 MHz for ^{1}H nuclei, D_2O)

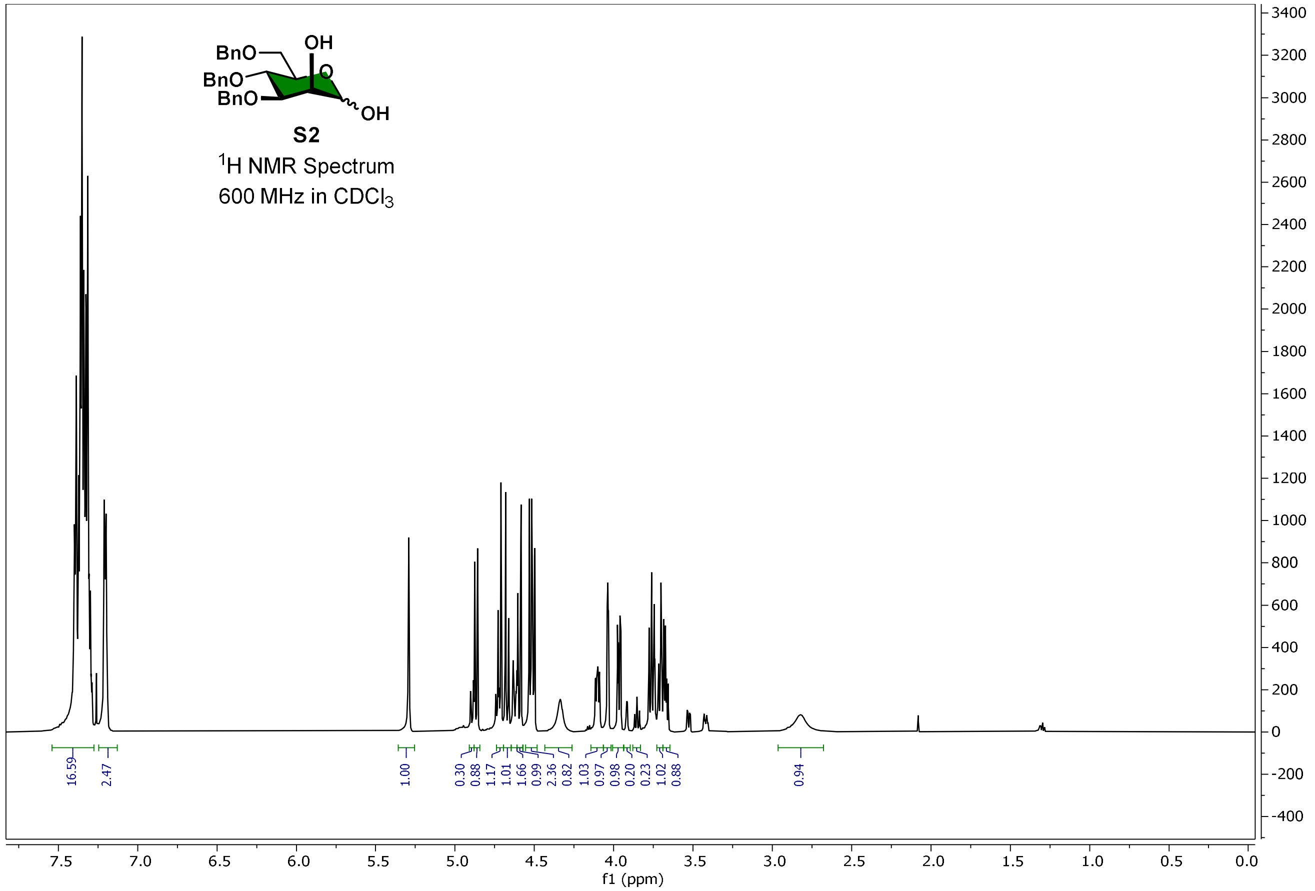
BnO
OH
BnO
BnO
O
OH
S2
1H NMR Spectrum
600 MHz in CDCl3
f1 (ppm)

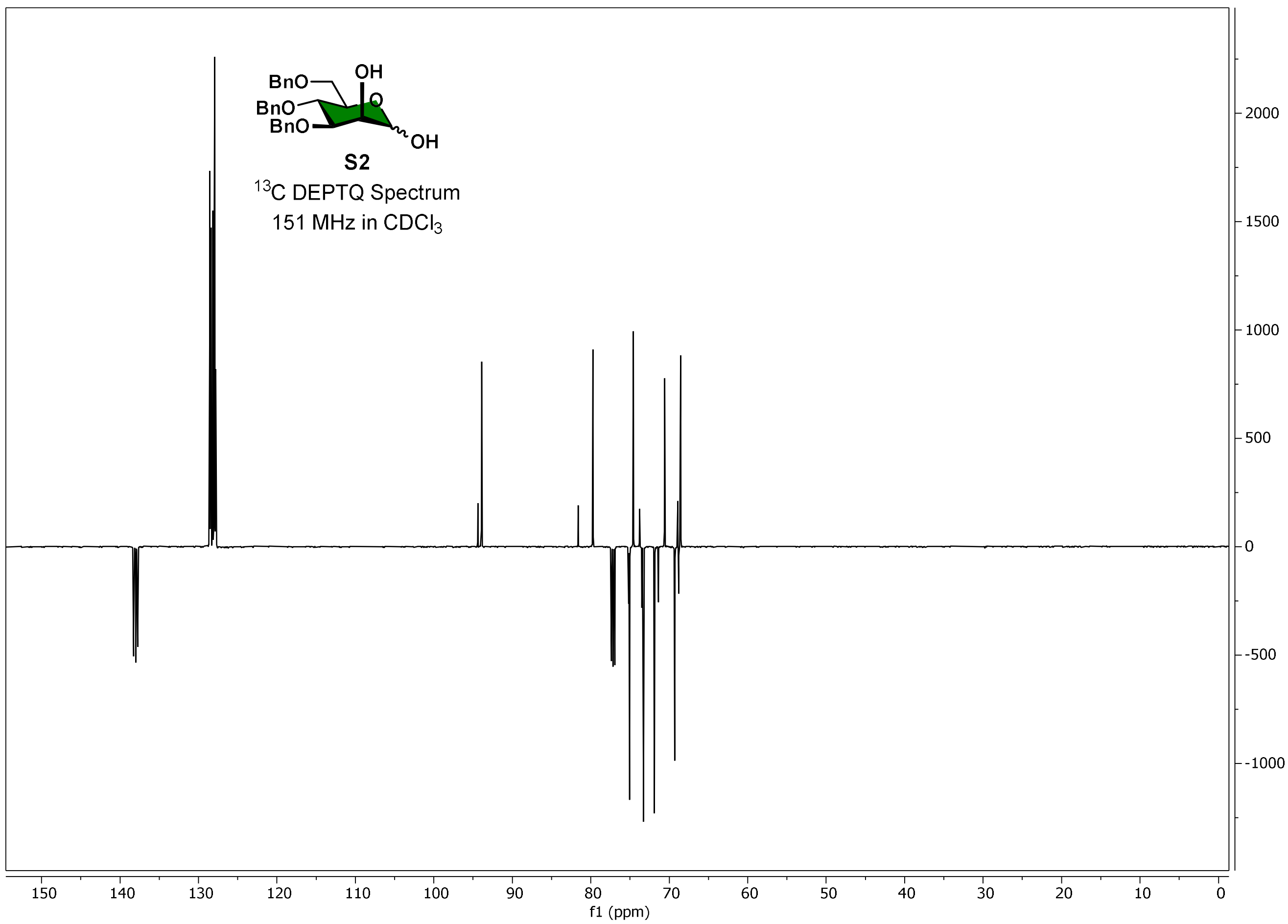
BnO
OH
BnO
BnO
O
OH
S2
13C DEPTQ Spectrum
151 MHz in CDCl3
f1 (ppm)

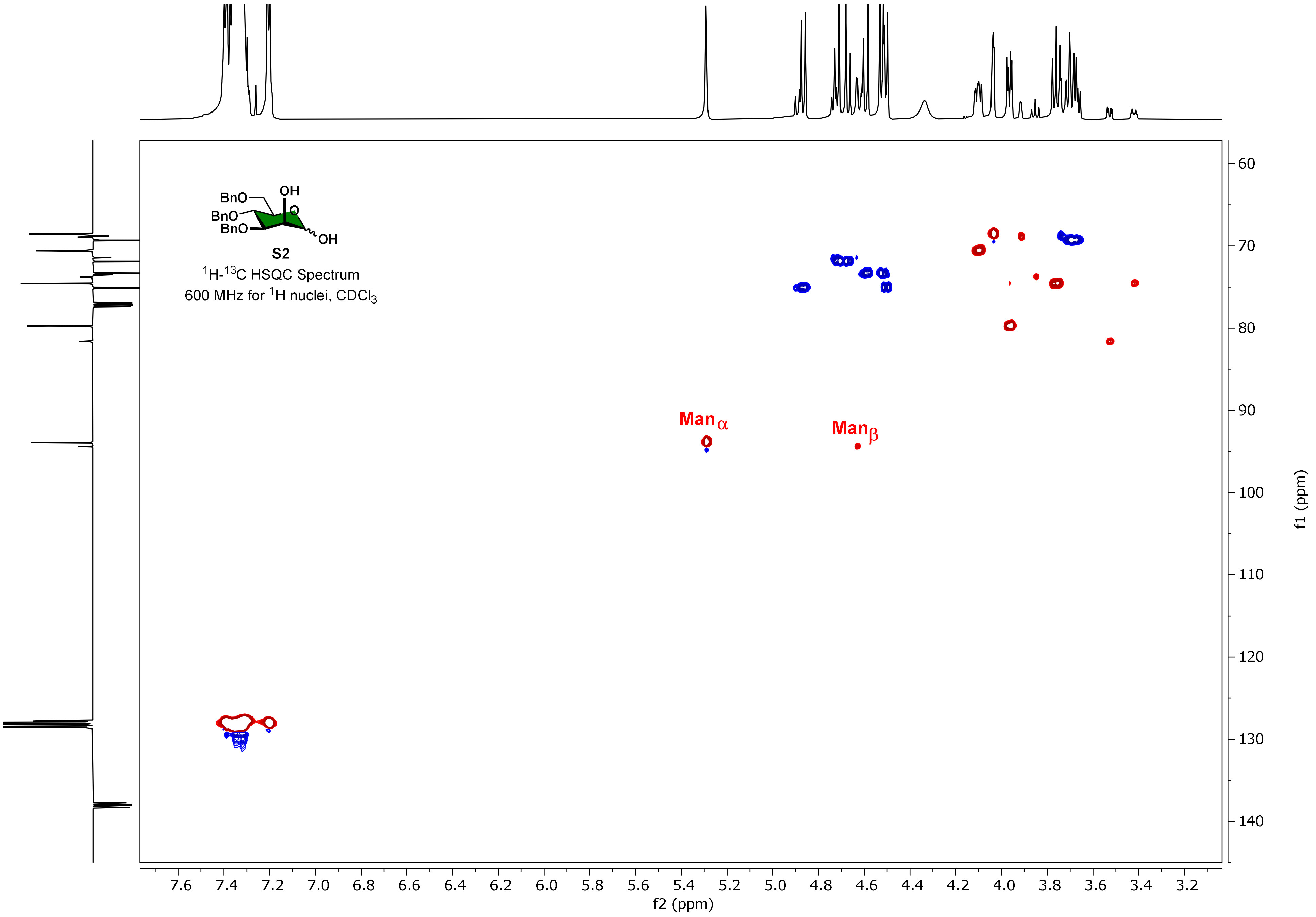
BnO
OH
BnO
BnO
O
OH
S2
1H-13C HSQC Spectrum
600 MHz for 1H nuclei, CDCl3
Manα
Manβ
f2 (ppm)
f1 (ppm)

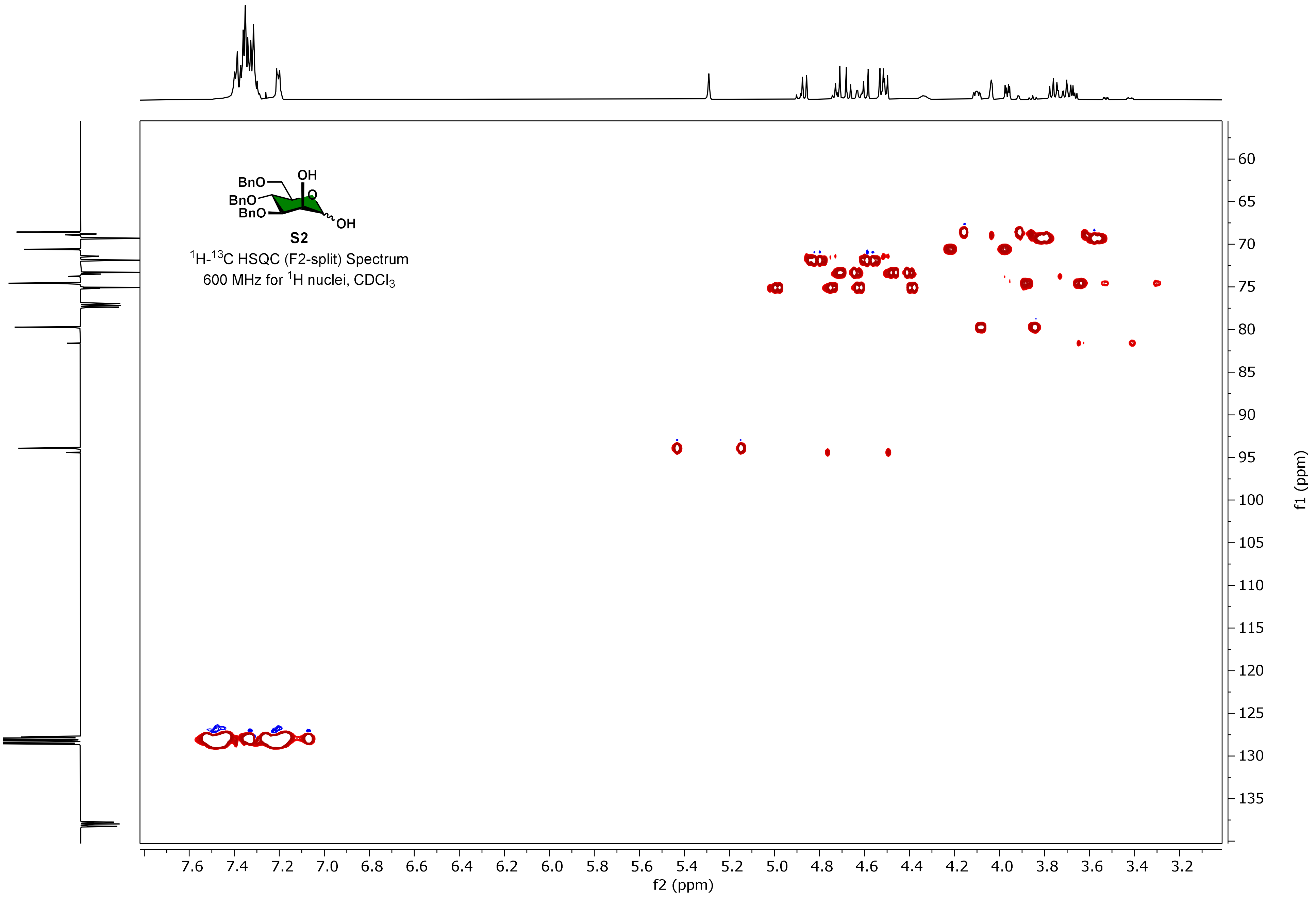
BnO
OH
O
BnO
BnO
OH
S2
^{1}H-^{13}C HSQC (F2-split) Spectrum
600 MHz for ^{1}H nuclei, CDCl$_3$
f2 (ppm)
f1 (ppm)

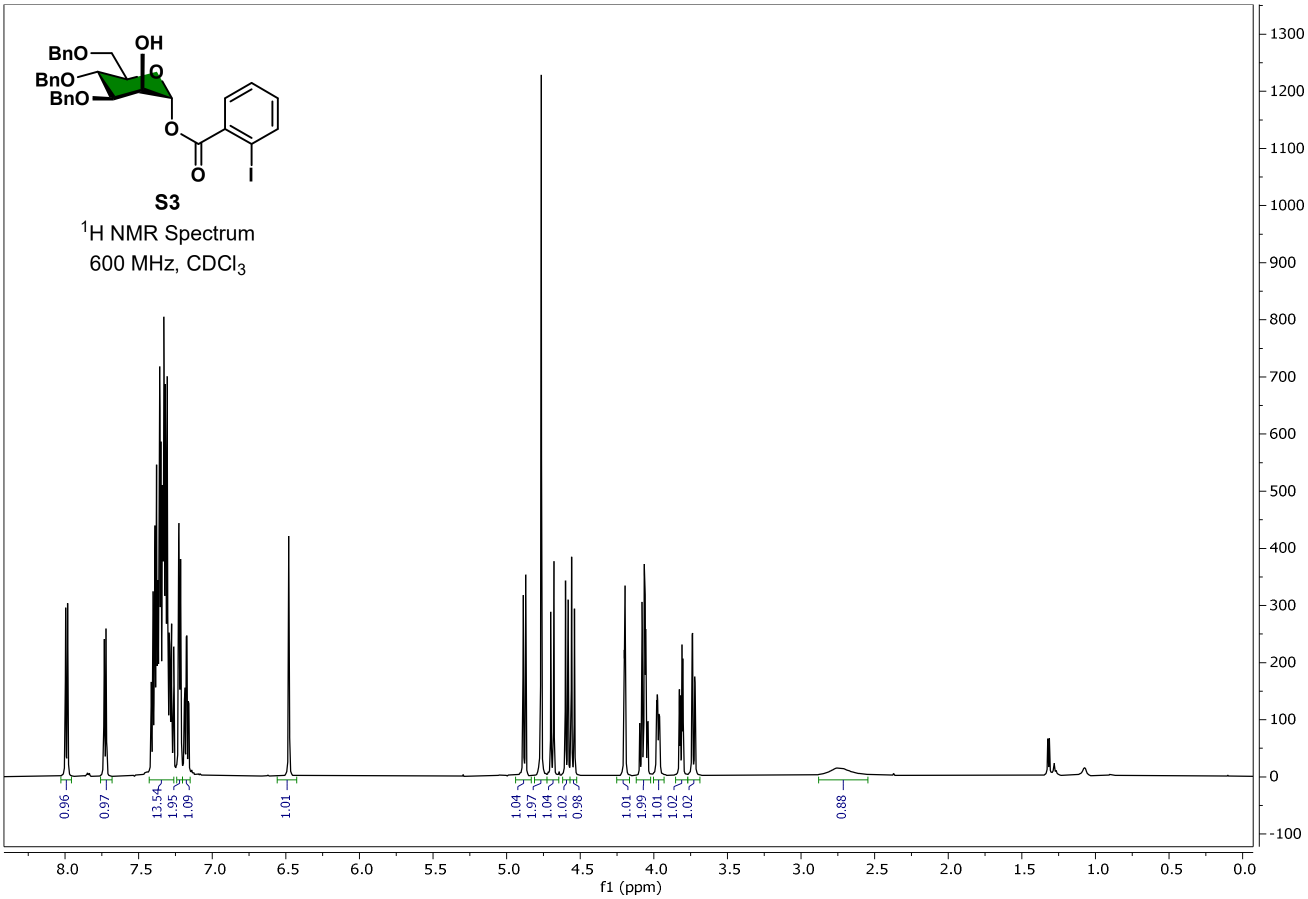
BnO
OH
O
BnO
BnO
O
O
I
S3
1H NMR Spectrum
600 MHz, CDCl3
0.96
0.97
13.54
1.95
1.09
1.01
1.04
1.97
1.04
1.02
0.98
1.01
1.99
1.01
1.02
1.02
0.88
f1 (ppm)

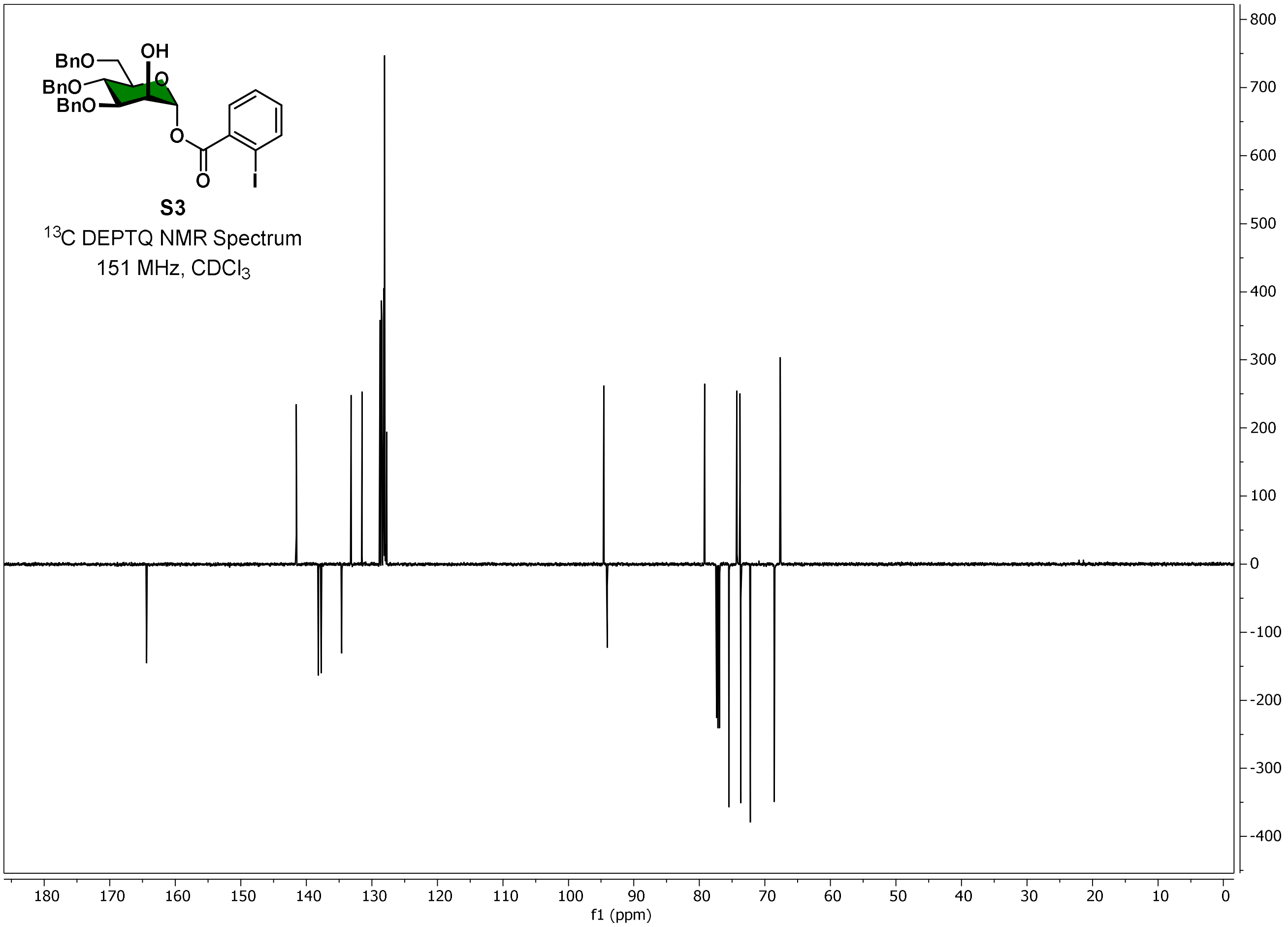
BnO
OH
O
BnO
BnO
O
O
I
S3
13C DEPTQ NMR Spectrum
151 MHz, CDCl3
f1 (ppm)

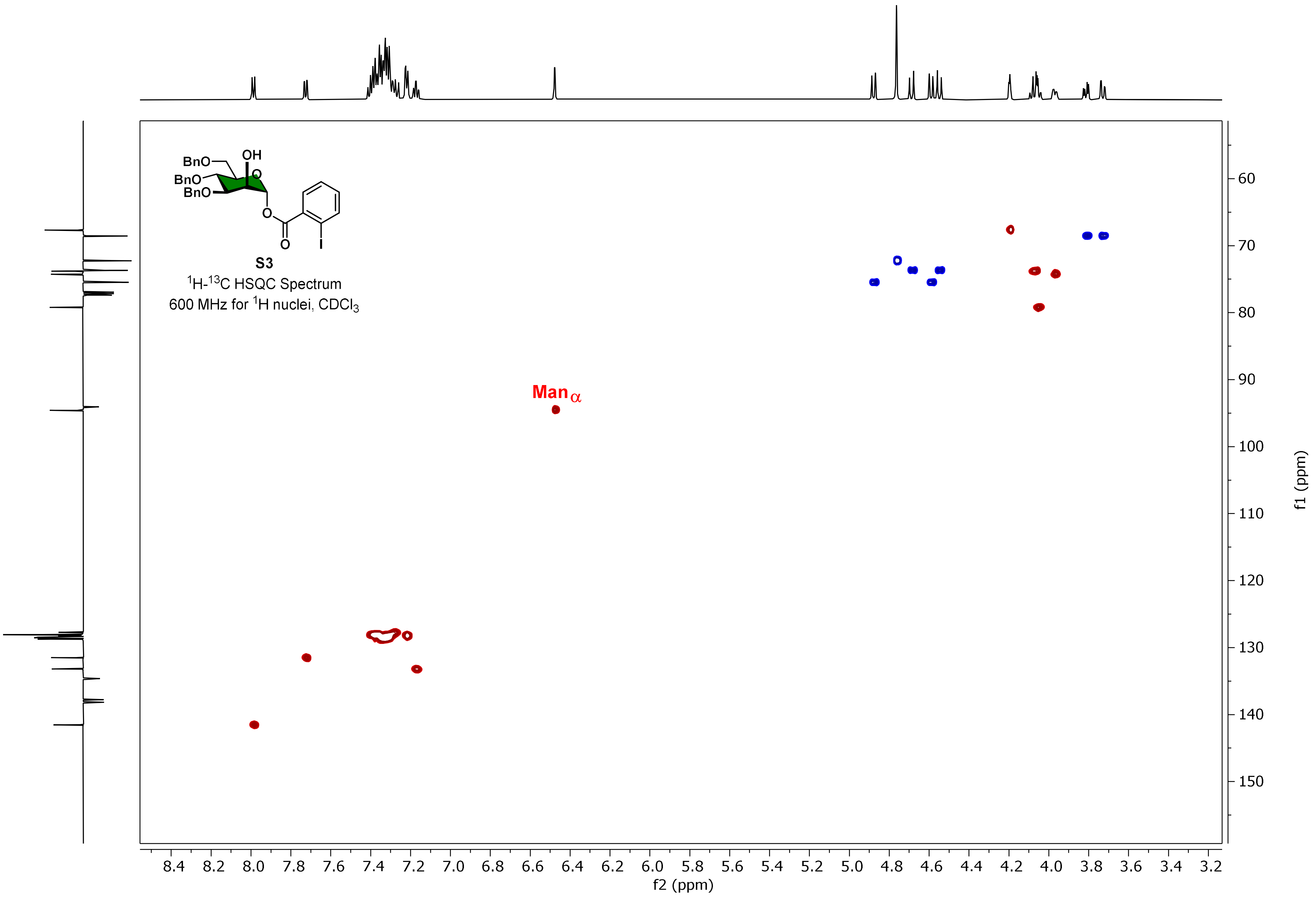
BnO
OH
BnO
BnO
O
O
O
I
S3
^1H-^{13}C HSQC Spectrum
600 MHz for ^1H nuclei, $CDCl_3$
Manα
f2 (ppm)
f1 (ppm)

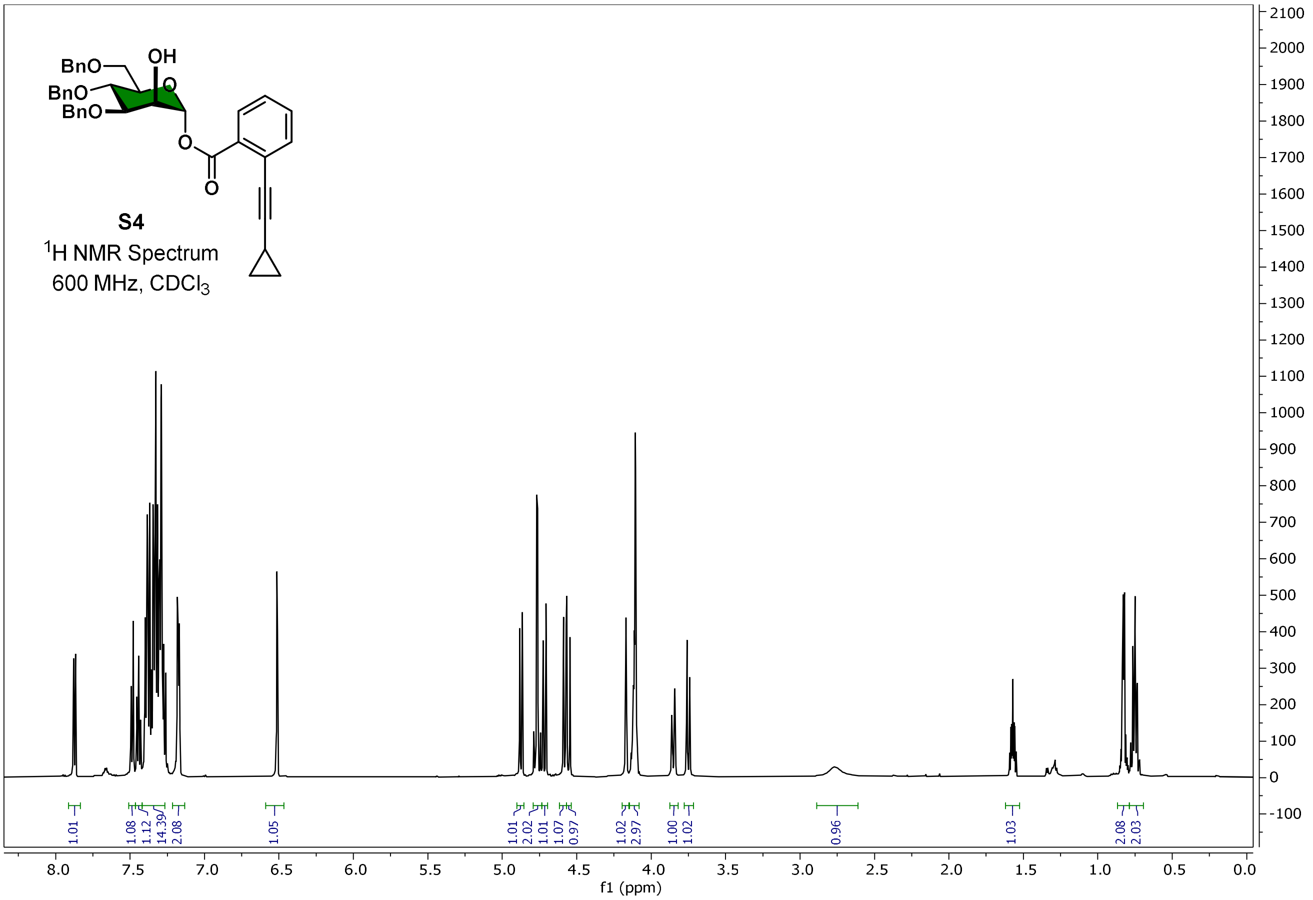

BnO
OH
BnO
BnO
O
O
O
S4
^{1}H NMR Spectrum
600 MHz, $CDCl_3$
1.01
1.08
1.12
14.39
2.08
1.05
1.01
2.02
1.01
1.07
0.97
1.02
2.97
1.00
1.02
0.96
1.03
2.08
2.03
f1 (ppm)

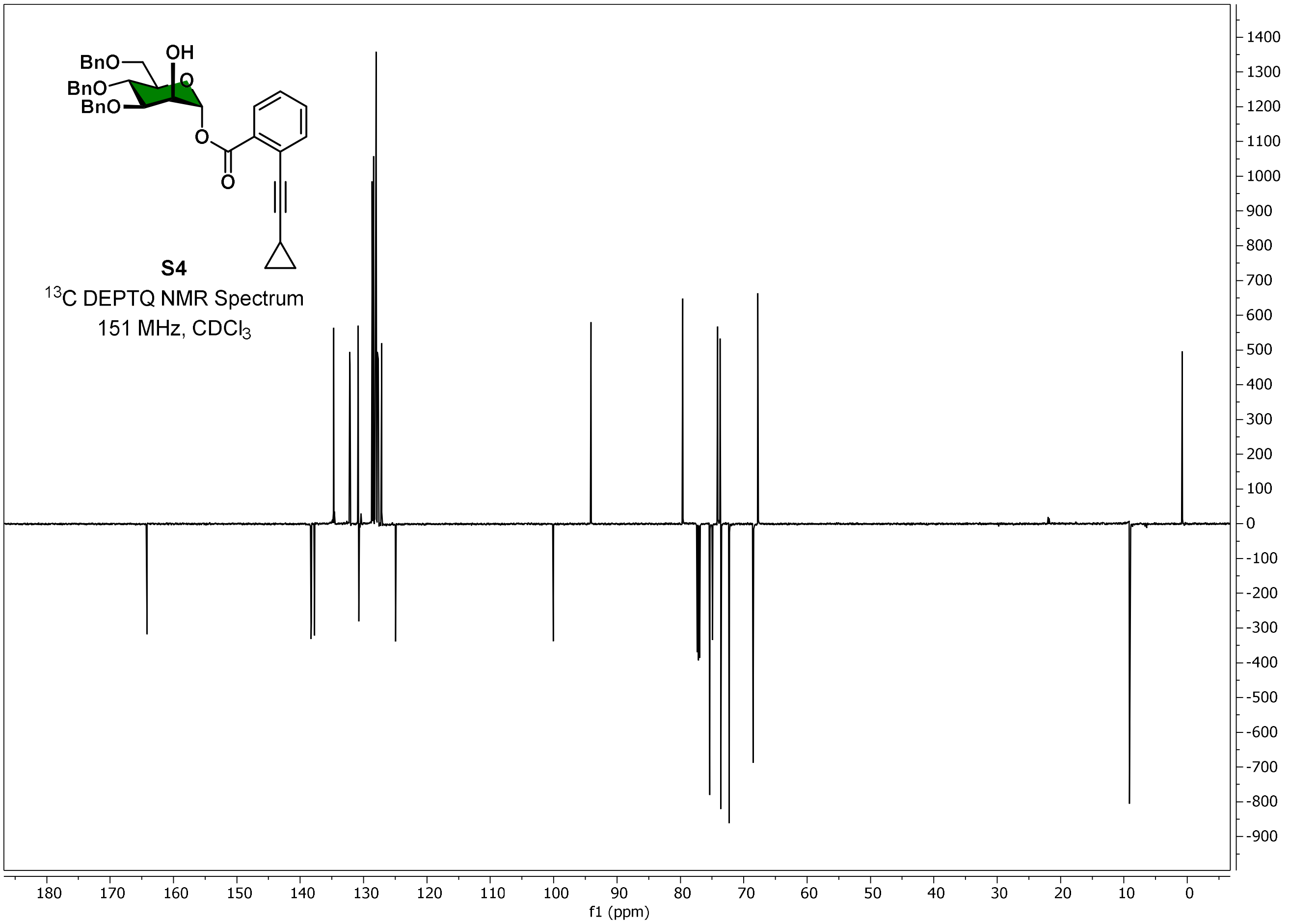

BnO
OH
BnO
BnO
O
O
O
S4
^{13}C DEPTQ NMR Spectrum
151 MHz, $CDCl_3$
f1 (ppm)

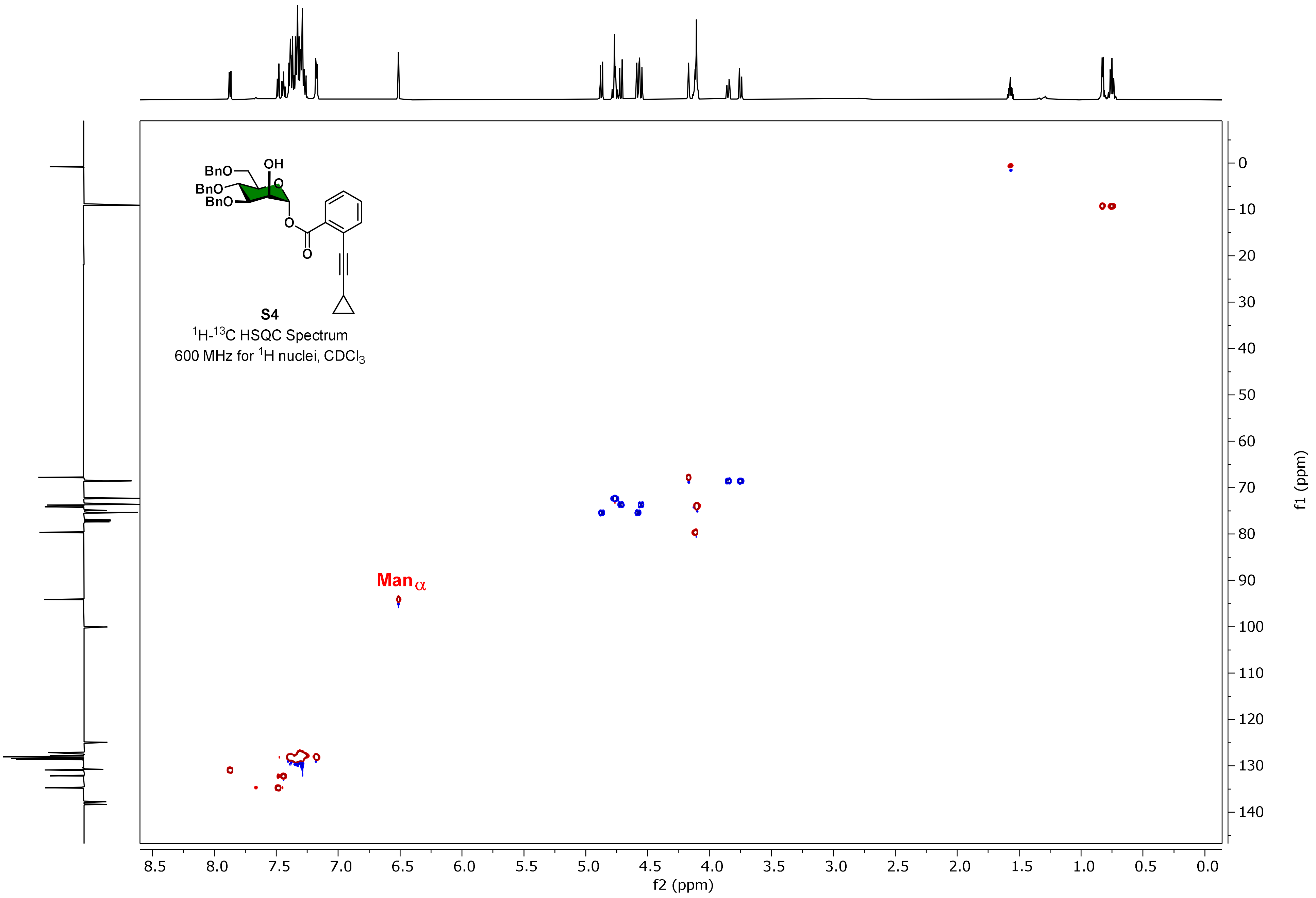
BnO
OH
O
BnO
BnO
O
O
S4
^{1}H-^{13}C HSQC Spectrum
600 MHz for ^{1}H nuclei, CDCl$_3$
Man$_\alpha$
f2 (ppm)
f1 (ppm)

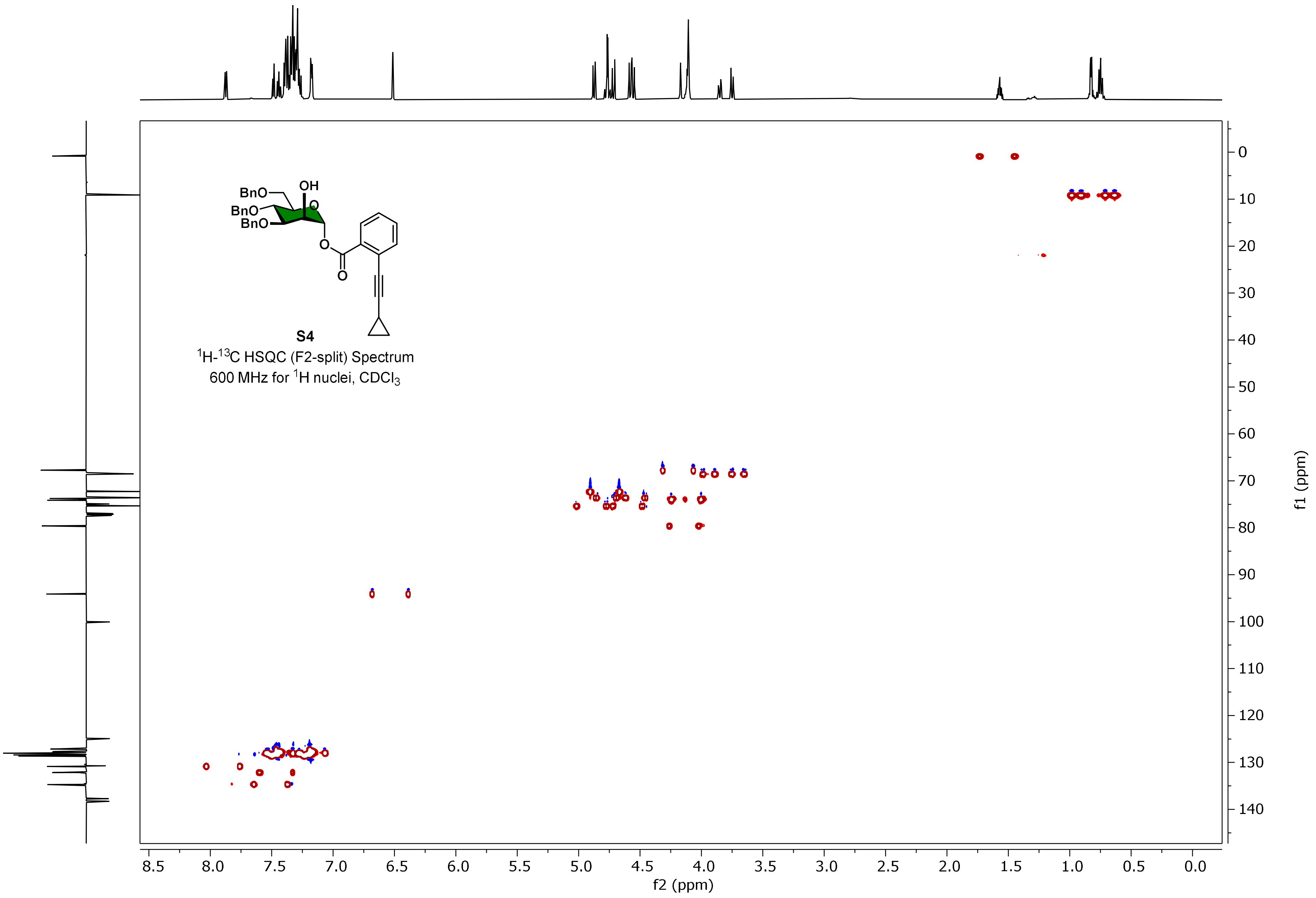

BnO
OH
BnO
BnO
O
O
O
S4
^{1}H-^{13}C HSQC (F2-split) Spectrum
600 MHz for ^{1}H nuclei, $CDCl_3$
f2 (ppm)
f1 (ppm)

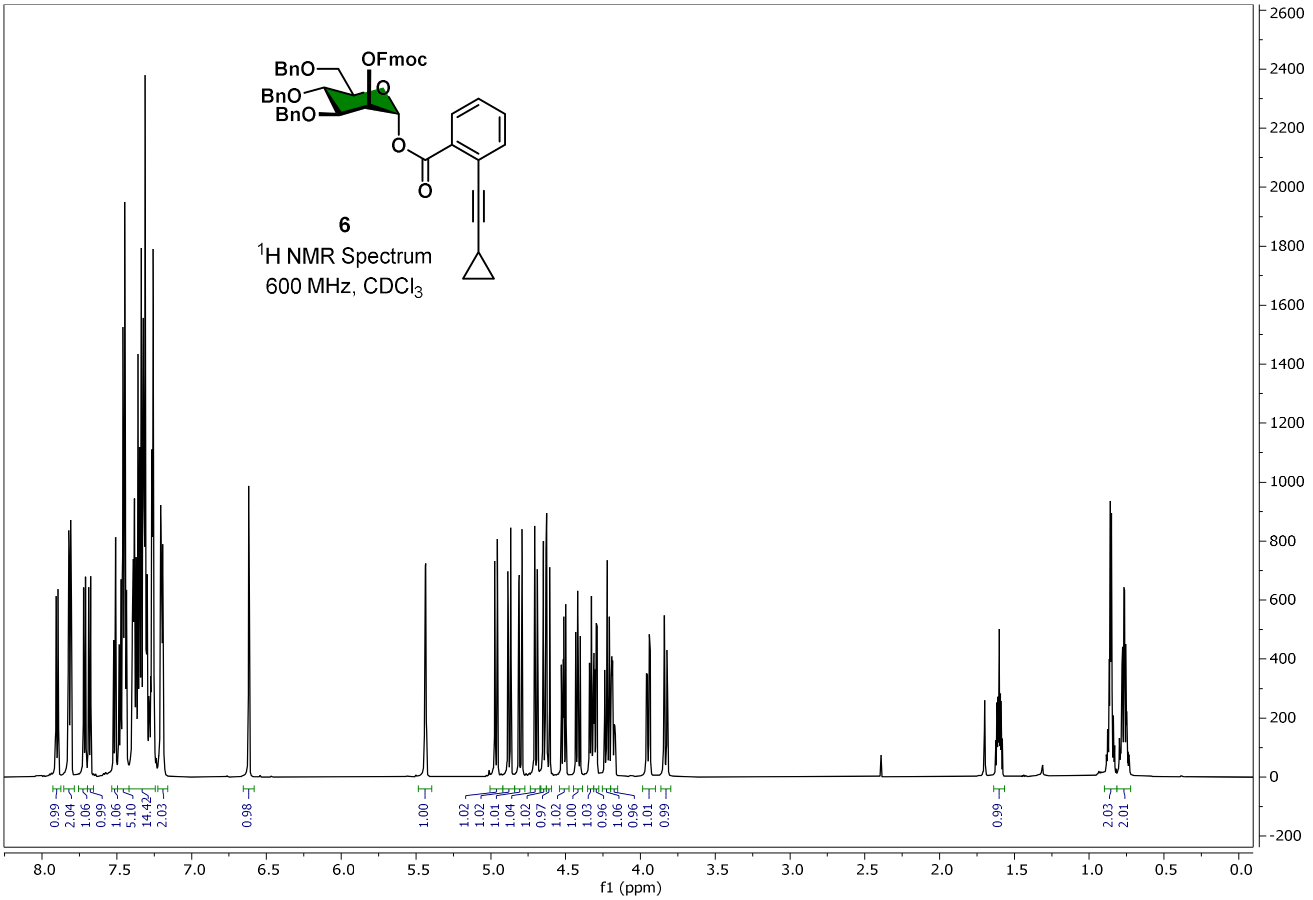
BnO
OFmoc
O
BnO
BnO
O
O
6
^{1}H NMR Spectrum
600 MHz, $CDCl_3$
f1 (ppm)

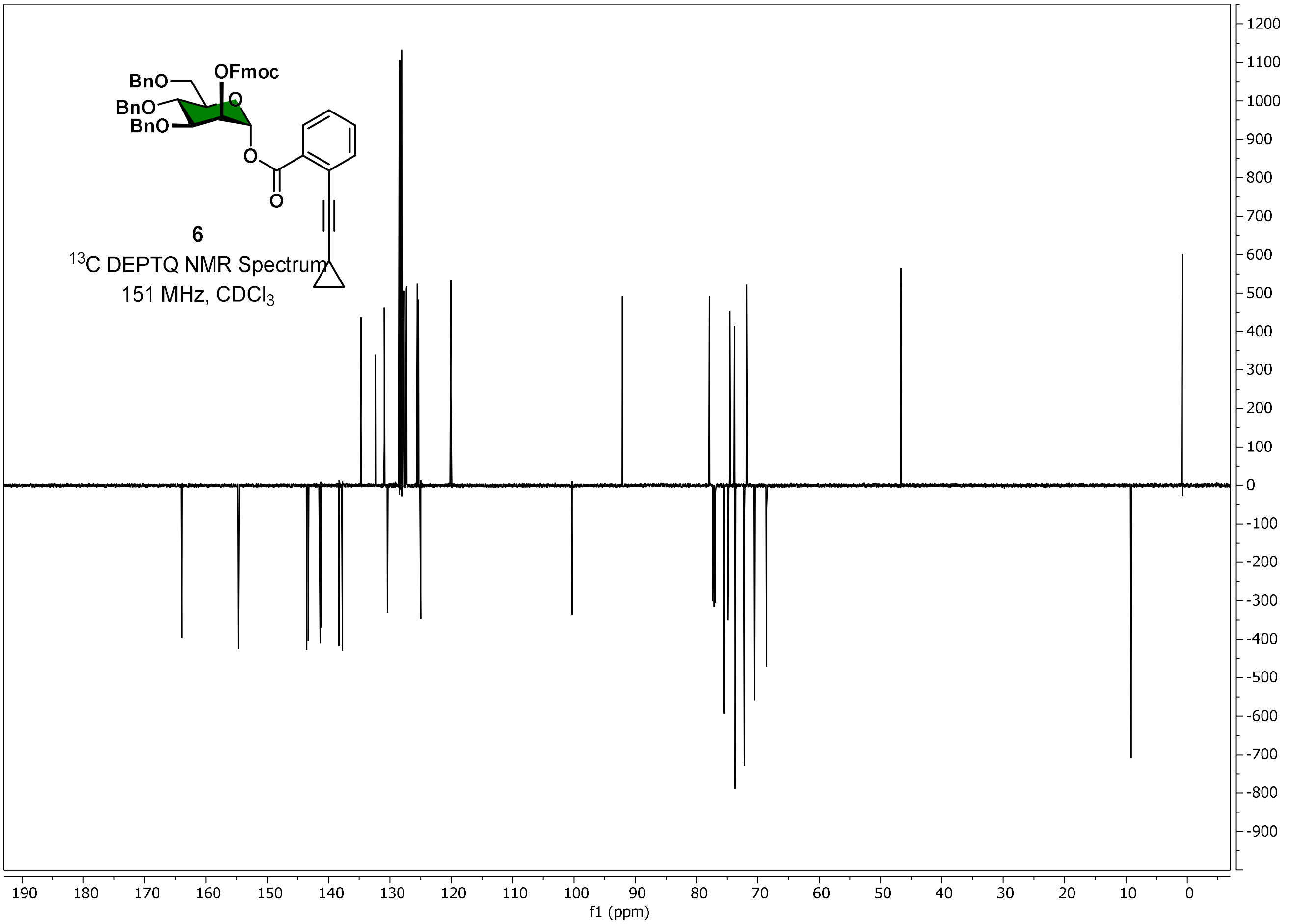
BnO
OFmoc
O
BnO
BnO
O
O
6
^{13}C DEPTQ NMR Spectrum
151 MHz, $CDCl_3$
f1 (ppm)

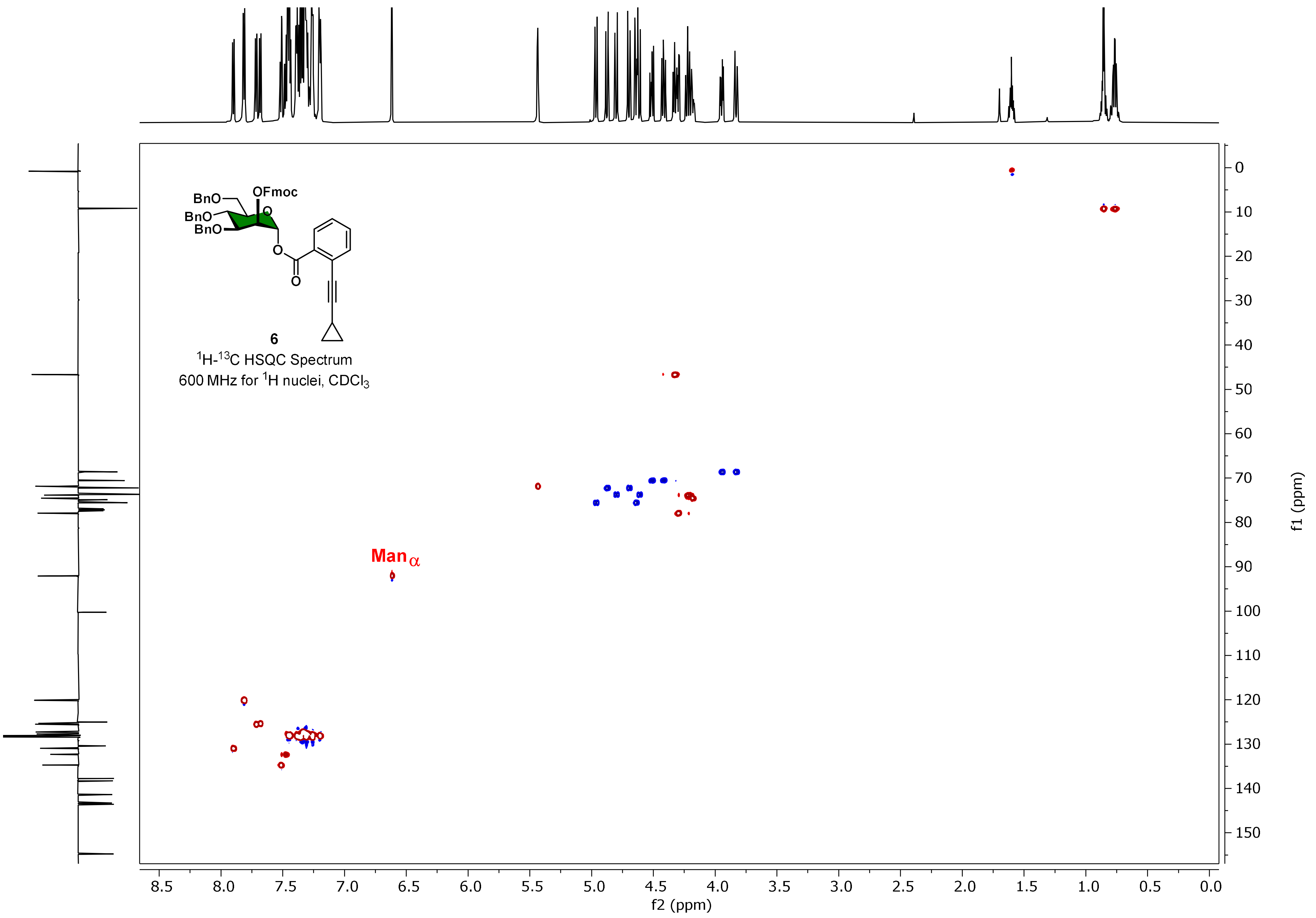
BnO
OFmoc
BnO
BnO
O
O
O
6
^{1}H-^{13}C HSQC Spectrum
600 MHz for ^{1}H nuclei, CDCl$_3$
Man$_\alpha$
f2 (ppm)
f1 (ppm)

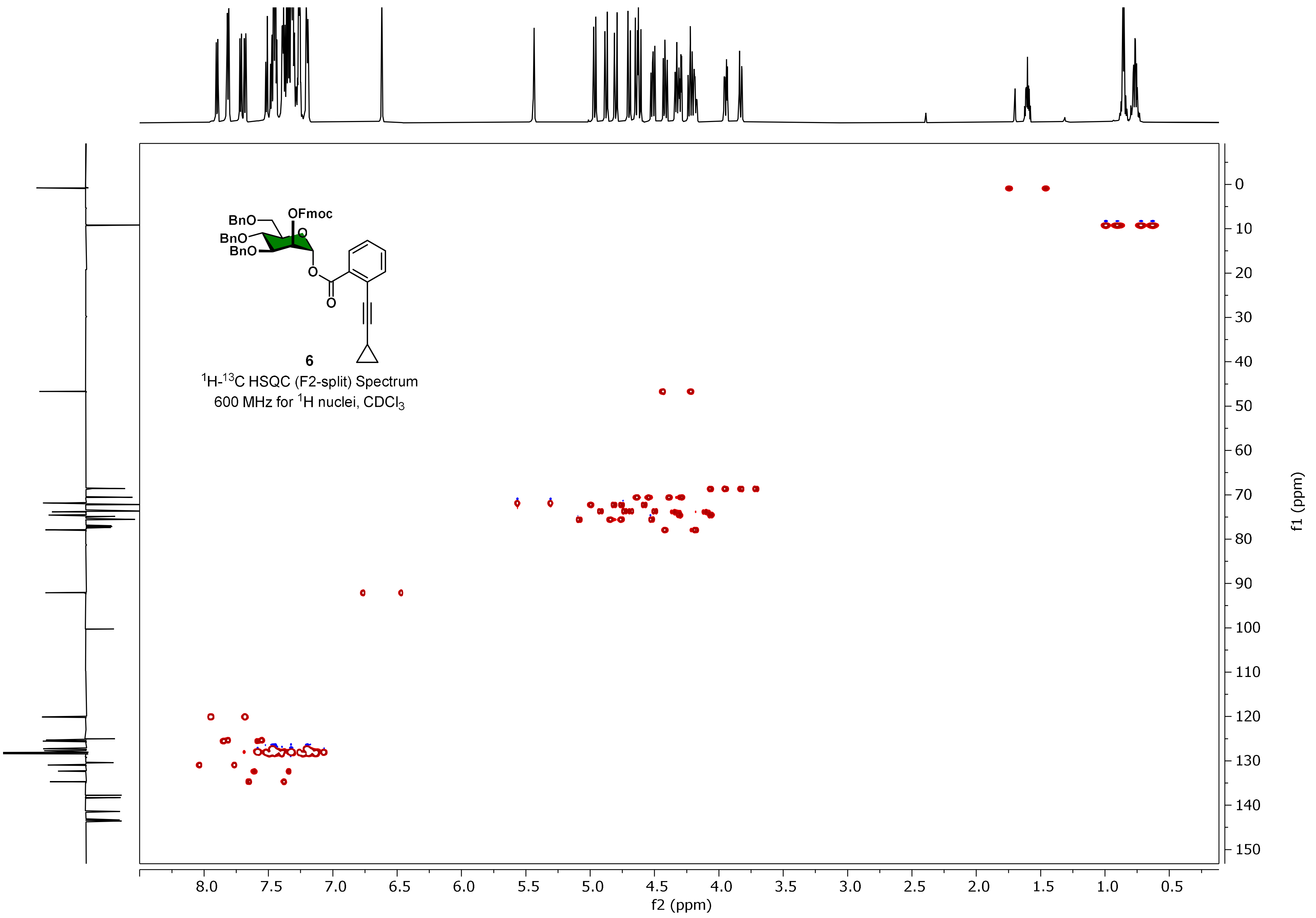
BnO
OFmoc
O
BnO
BnO
O
O
6
$^1H-^{13}C$ HSQC (F2-split) Spectrum
600 MHz for ^1H nuclei, $CDCl_3$
f2 (ppm)
f1 (ppm)
8.0 7.5 7.0 6.5 6.0 5.5 5.0 4.5 4.0 3.5 3.0 2.5 2.0 1.5 1.0 0.5
0 10 20 30 40 50 60 70 80 90 100 110 120 130 140 150

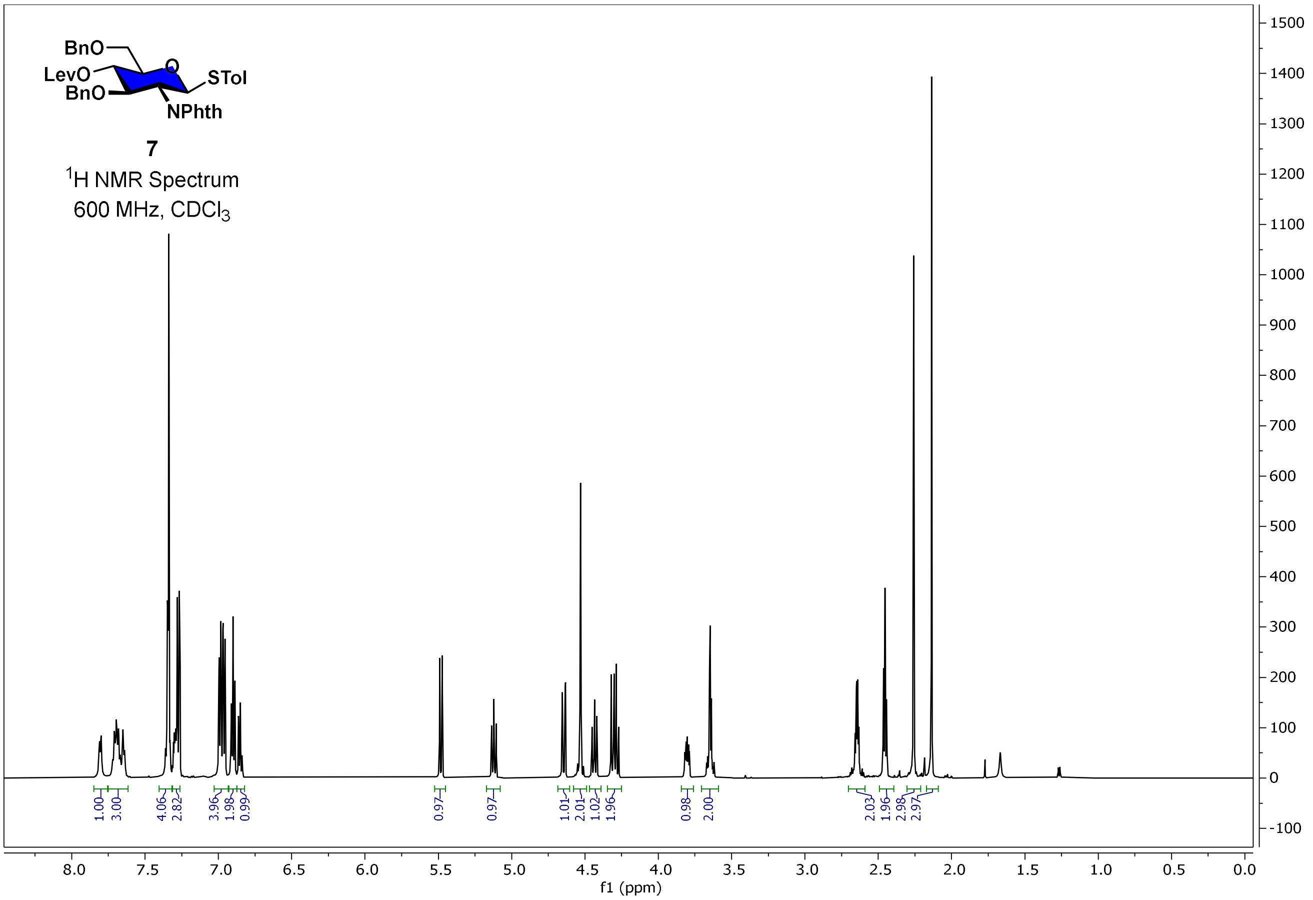
BnO
LevO
BnO
O
STol
NPhth
7
1H NMR Spectrum
600 MHz, CDCl3
f1 (ppm)

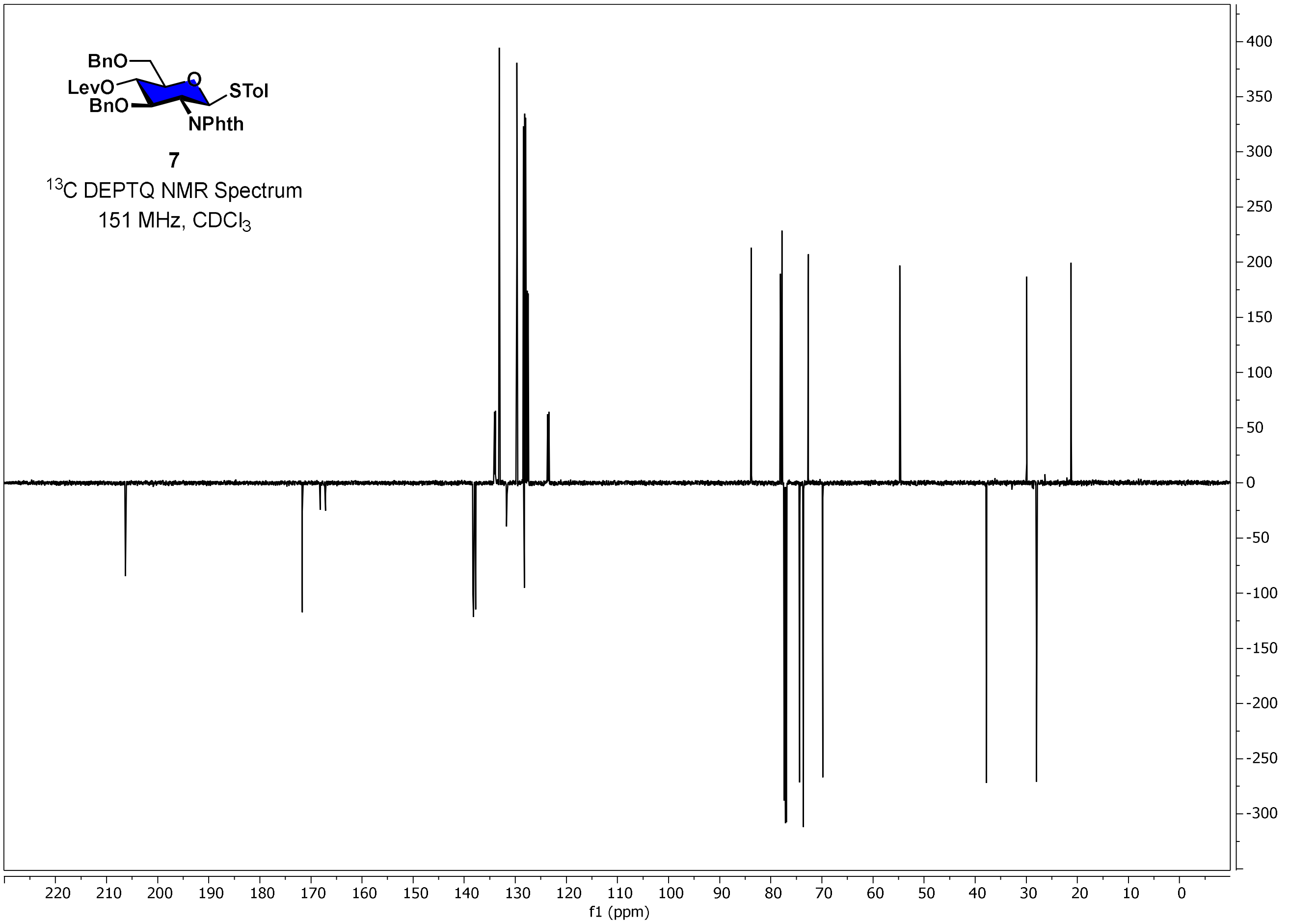
BnO
LevO
BnO
O
STol
NPhth
7
13C DEPTQ NMR Spectrum
151 MHz, CDCl3
f1 (ppm)

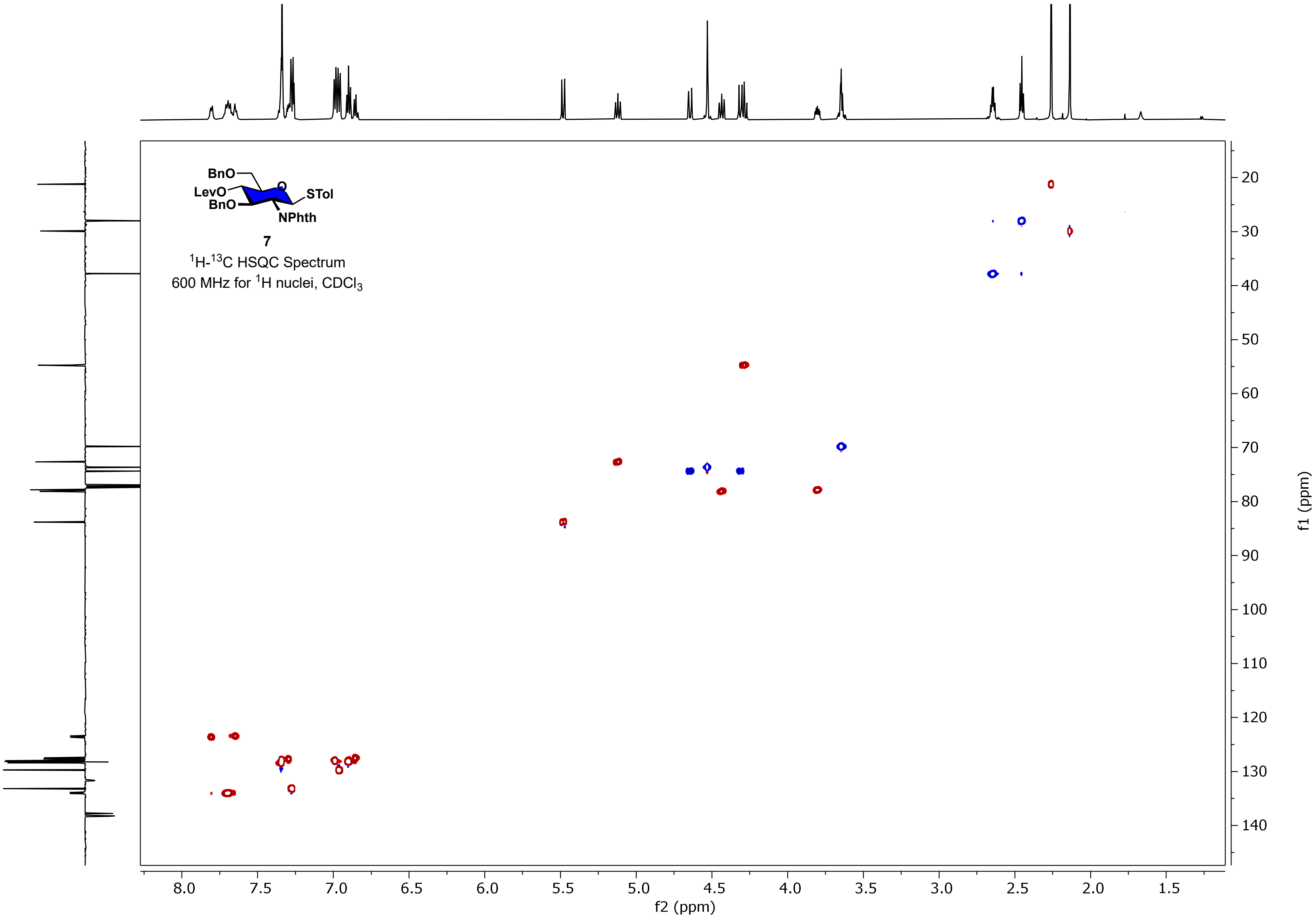
BnO
LevO
BnO
O
STol
NPhth
7
^{1}H-^{13}C HSQC Spectrum
600 MHz for ^{1}H nuclei, CDCl$_3$
f2 (ppm)
f1 (ppm)

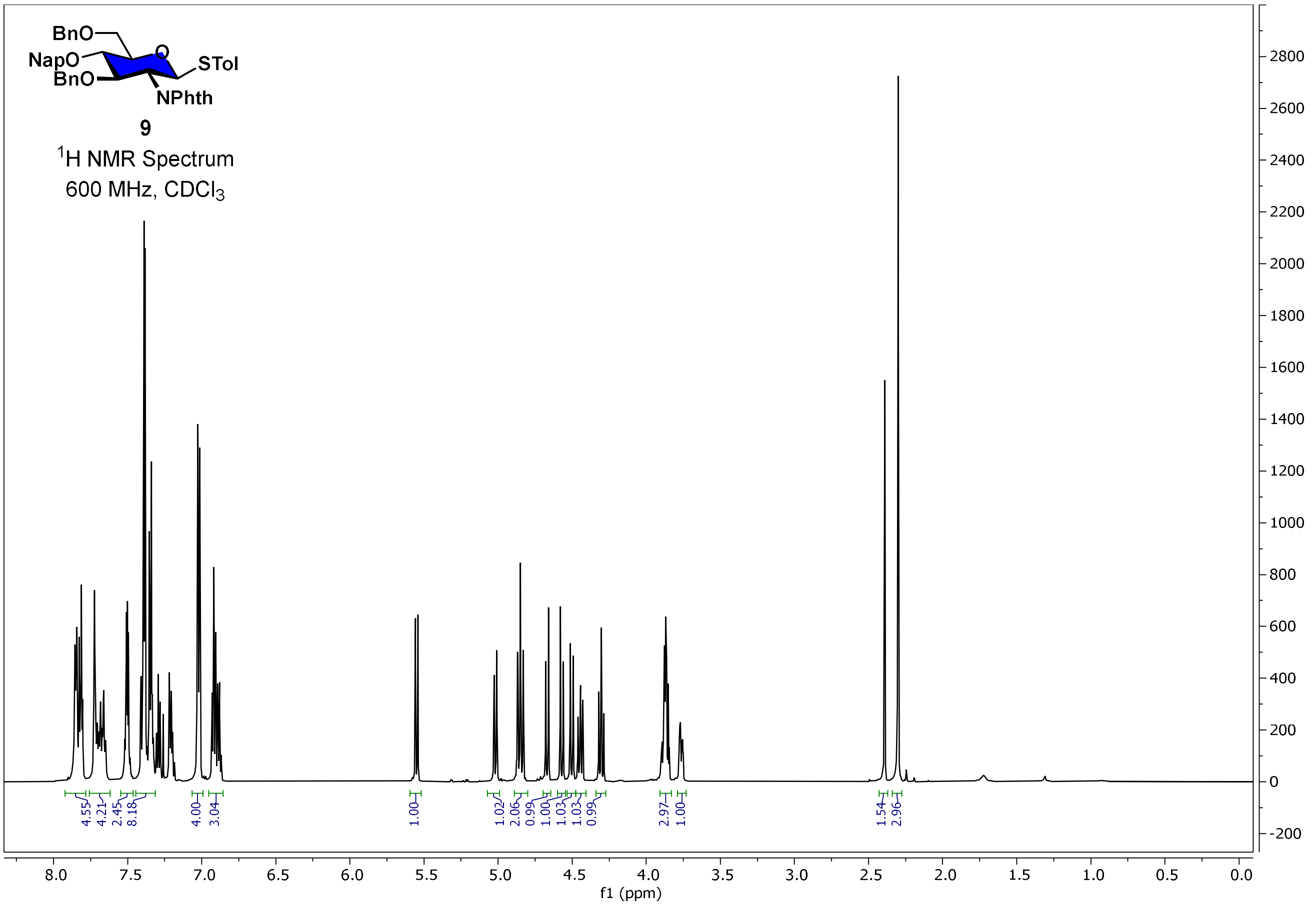
BnO
NapO
BnO
STol
NPhth
9
¹H NMR Spectrum
600 MHz, CDCl₃
f1 (ppm)

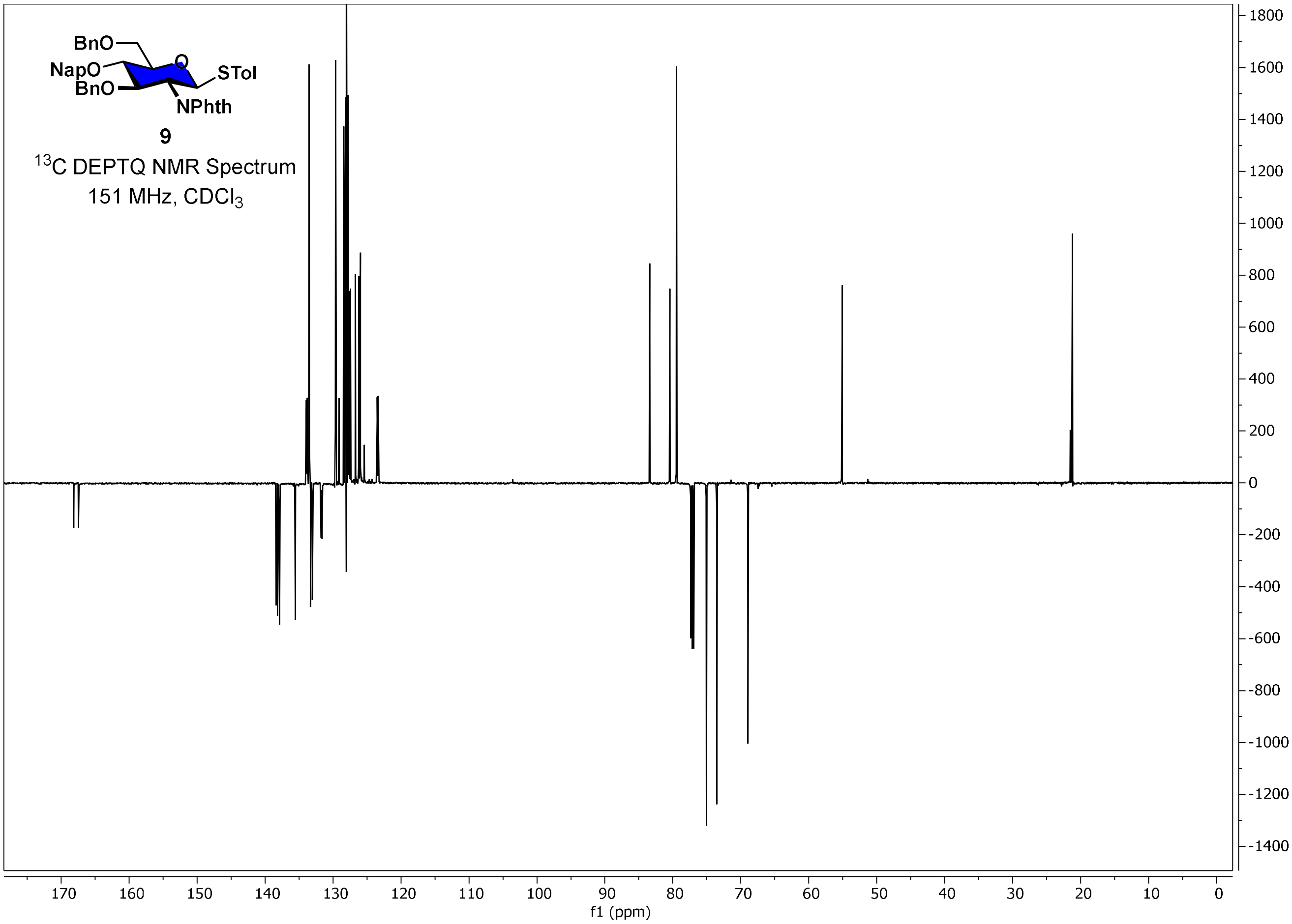
BnO
NapO
BnO
STol
NPhth
9
¹³C DEPTQ NMR Spectrum
151 MHz, CDCl₃
f1 (ppm)

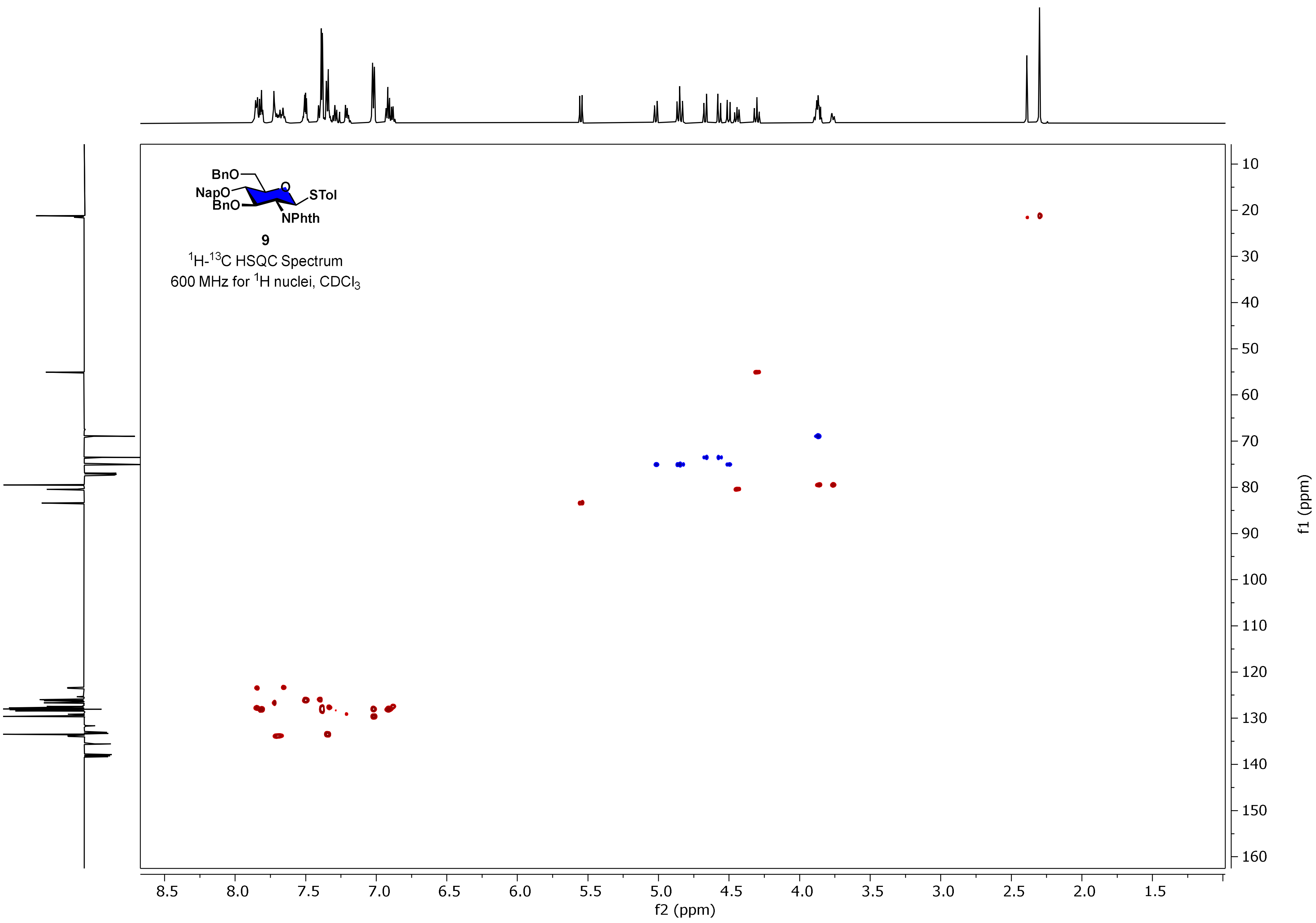
BnO
NapO
BnO
O
STol
NPhth
9
1H-13C HSQC Spectrum
600 MHz for 1H nuclei, CDCl3
f2 (ppm)
f1 (ppm)

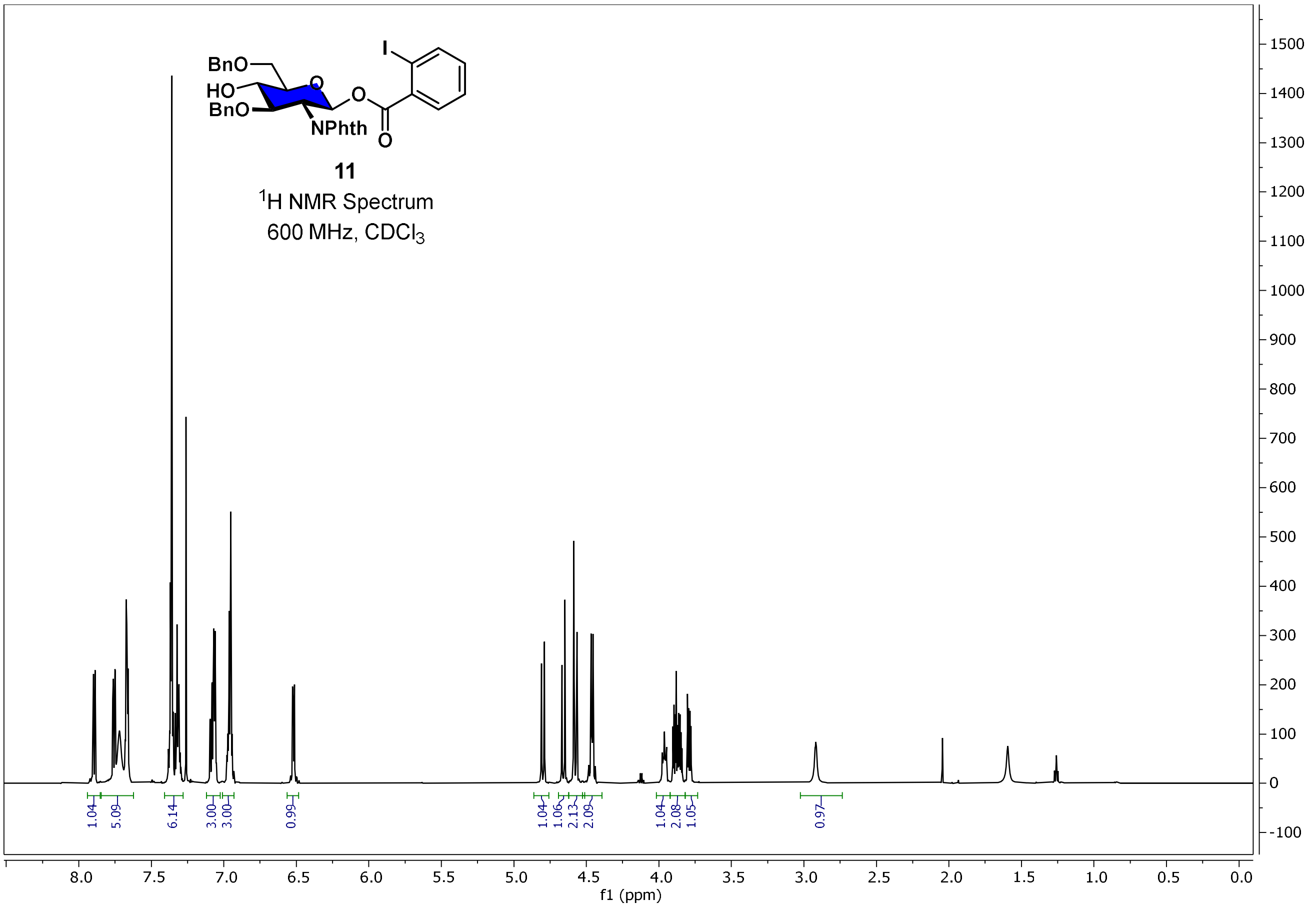
BnO
HO
BnO
O
O
I
NPhth
O
11
^{1}H NMR Spectrum
600 MHz, CDCl$_3$
1.04
5.09
6.14
3.00
3.00
0.99
1.04
1.06
2.13
2.09
1.04
2.08
1.05
0.97
f1 (ppm)

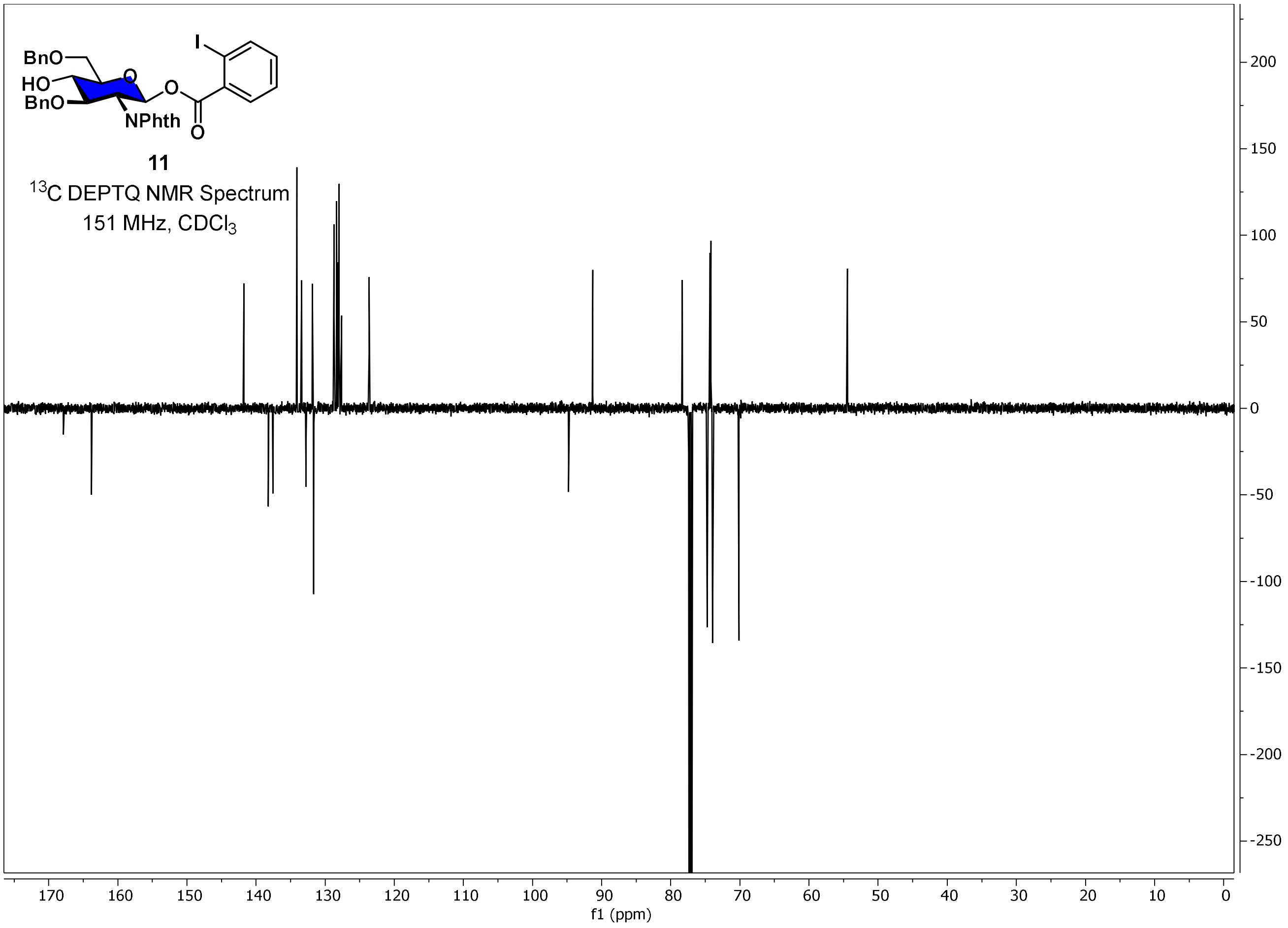
BnO
HO
BnO
O
O
I
NPhth
O
11
^{13}C DEPTQ NMR Spectrum
151 MHz, CDCl$_3$
f1 (ppm)

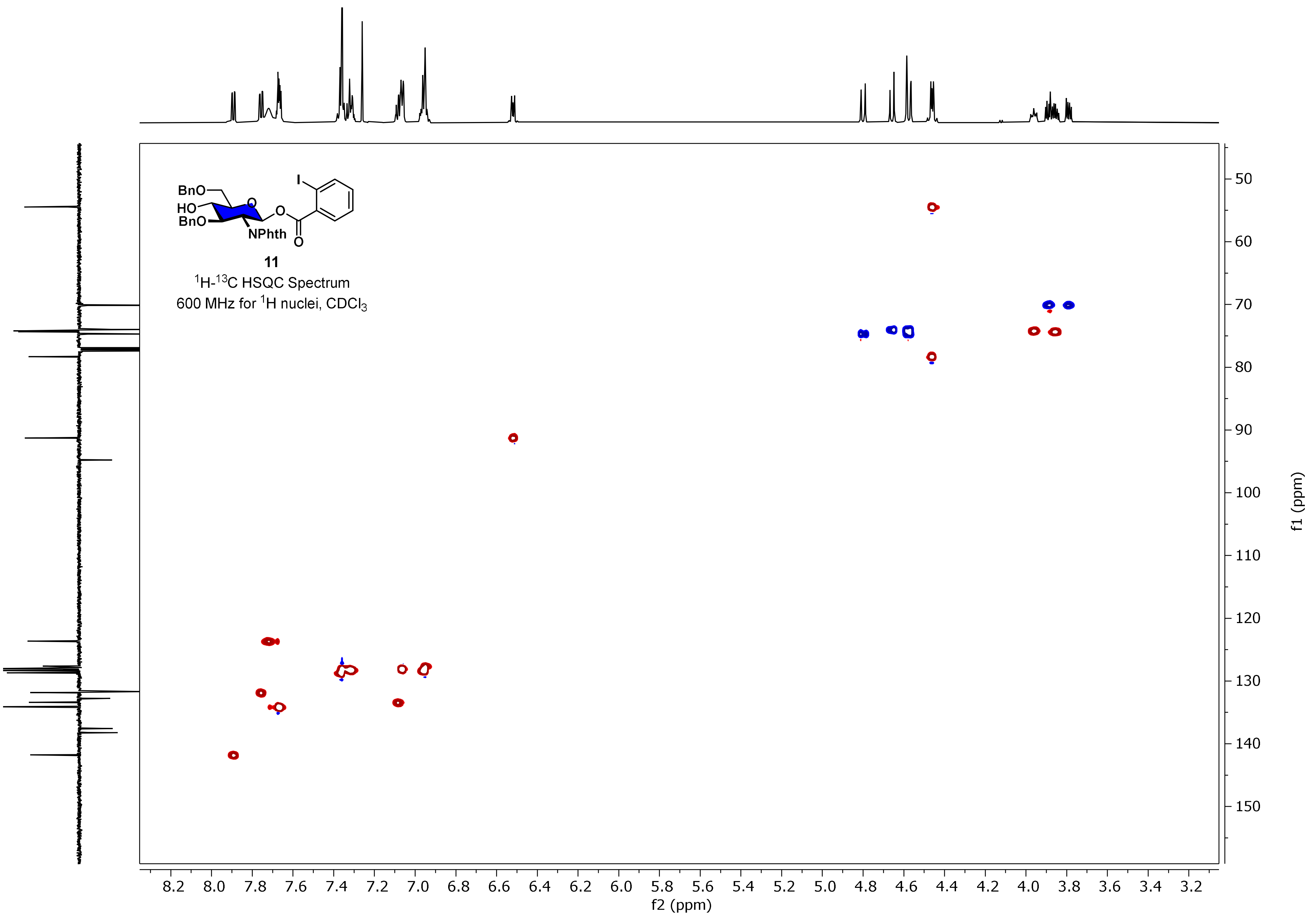
BnO
HO
BnO
O
O
I
NPhth
O
11
^{1}H-^{13}C HSQC Spectrum
600 MHz for ^{1}H nuclei, CDCl$_3$
f1 (ppm)
f2 (ppm)
8.2 8.0 7.8 7.6 7.4 7.2 7.0 6.8 6.6 6.4 6.2 6.0 5.8 5.6 5.4 5.2 5.0 4.8 4.6 4.4 4.2 4.0 3.8 3.6 3.4 3.2
50 60 70 80 90 100 110 120 130 140 150

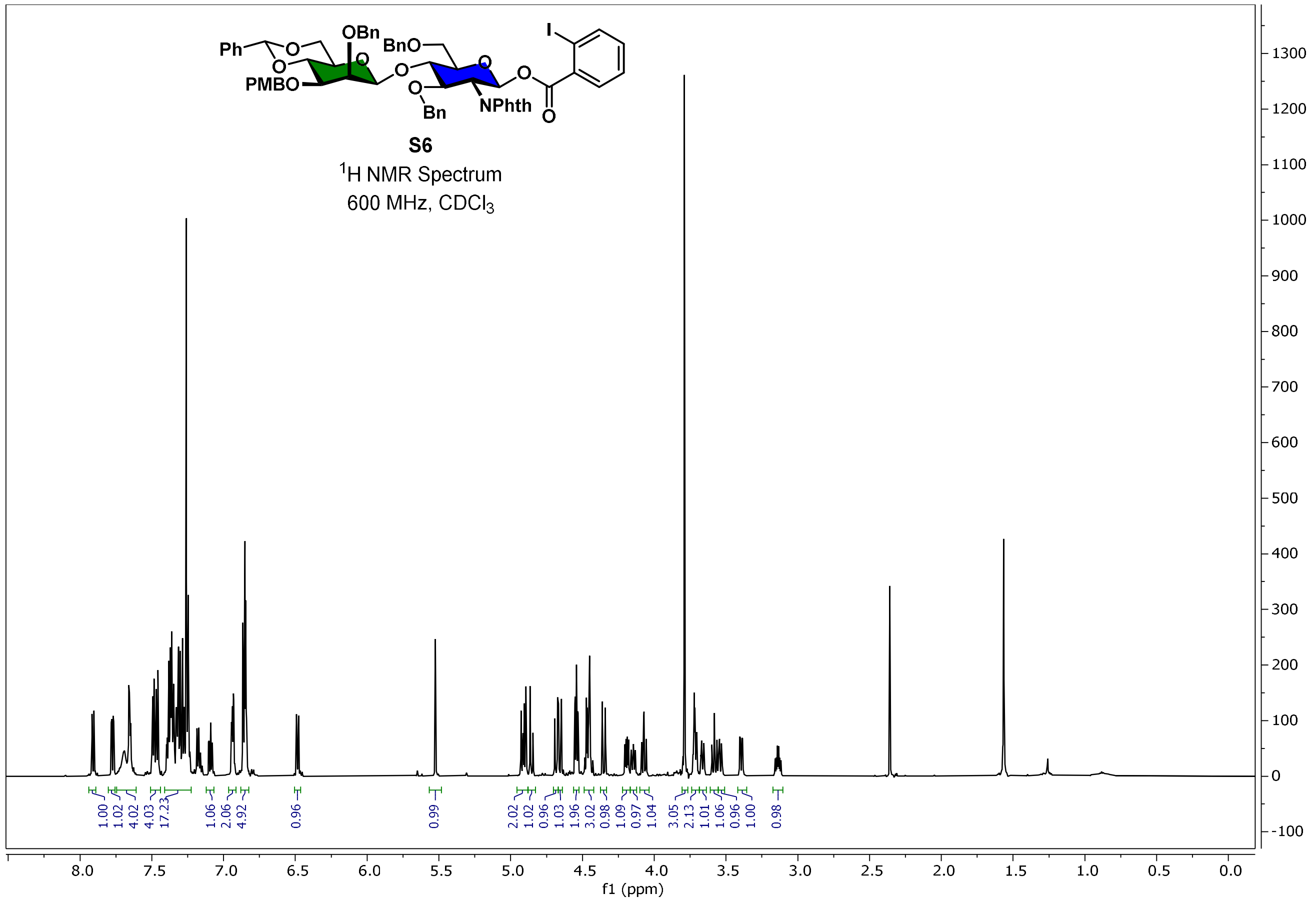
S6
^{1}H NMR Spectrum
600 MHz, $CDCl_3$
f1 (ppm)

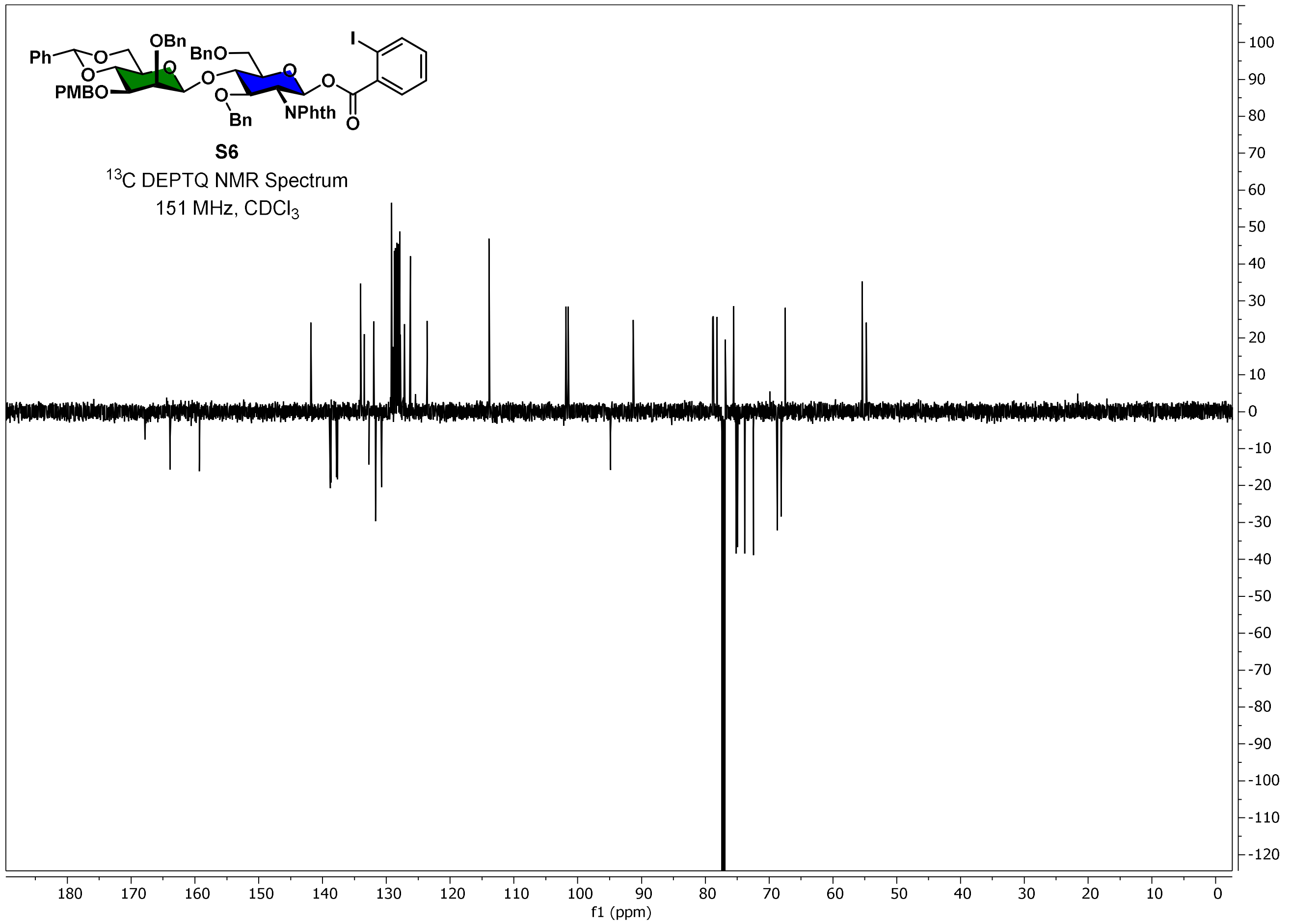
S6
^{13}C DEPTQ NMR Spectrum
151 MHz, $CDCl_3$
f1 (ppm)

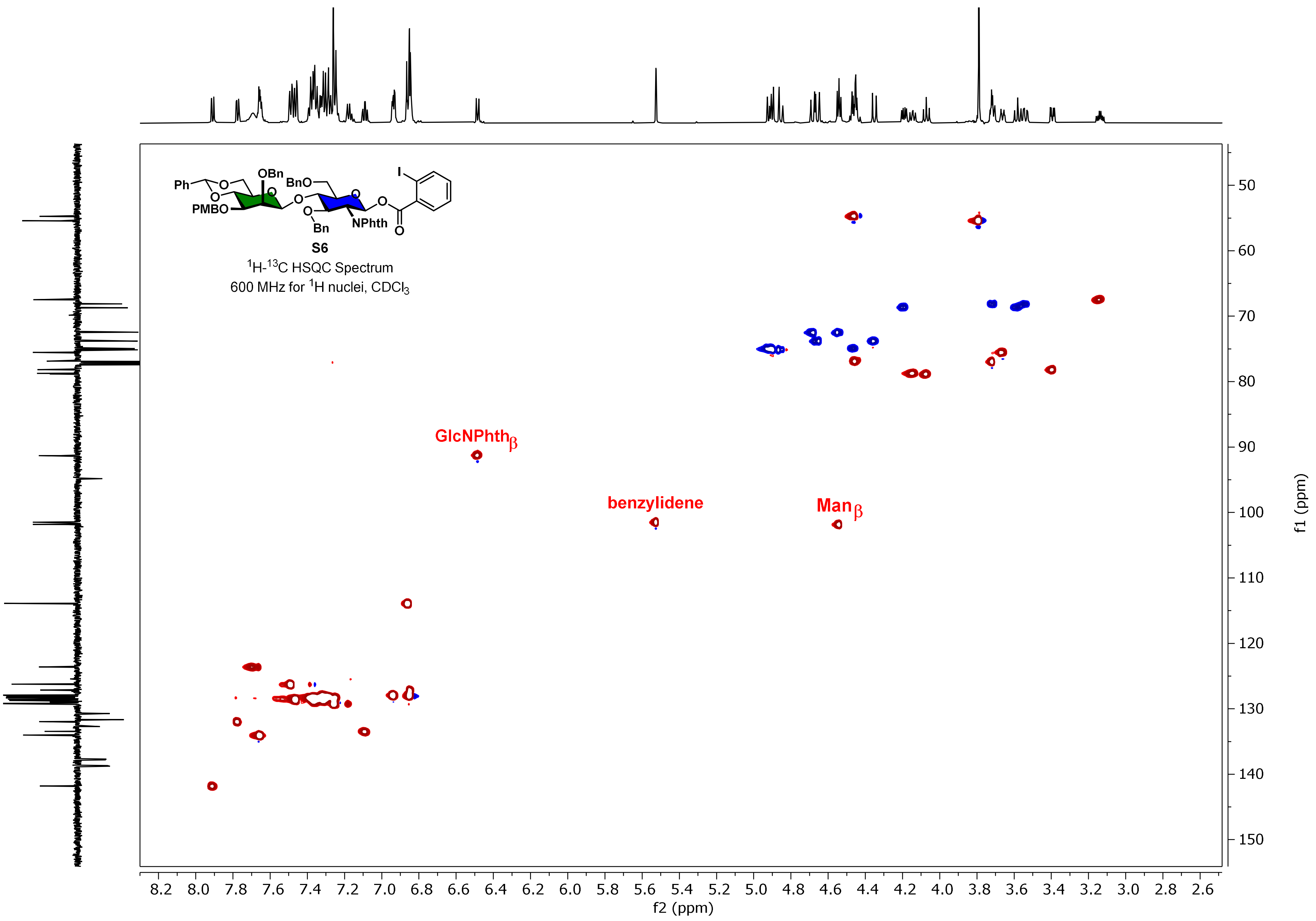

Ph
O
O
OBn
O
PMBO
BnO
O
O
O
Bn
NPhth
O
I
O
S6
1H-13C HSQC Spectrum
600 MHz for 1H nuclei, CDCl3
GlcNPhthβ
benzylidene
Manβ
f2 (ppm)
f1 (ppm)

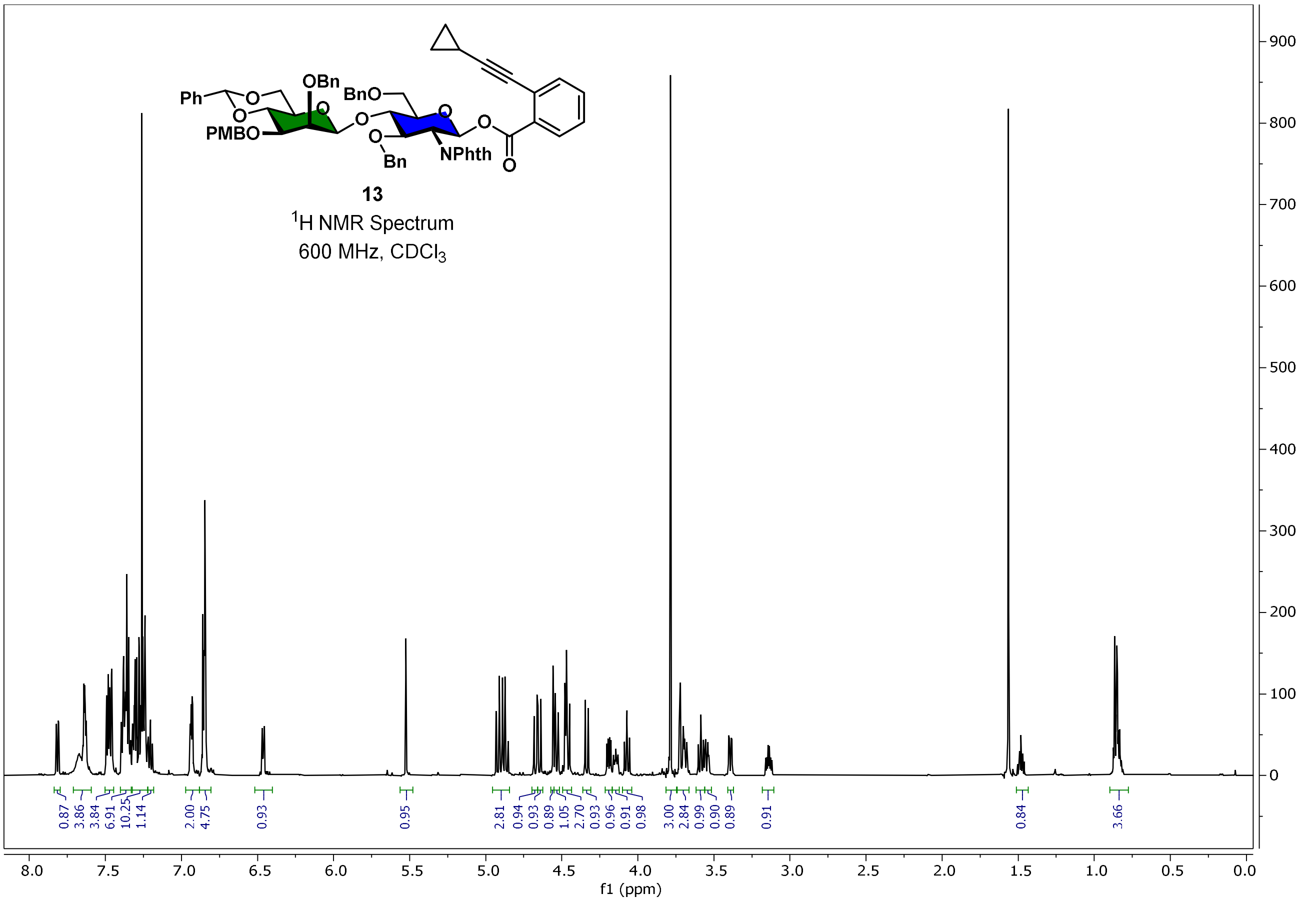

Ph
O
O
OBn
O
PMBO
BnO
O
O
O
Bn
NPhth
O
O
13
^{1}H NMR Spectrum
600 MHz, CDCl$_3$
0.87
3.86
3.84
6.91
10.25
1.14
2.00
4.75
0.93
0.95
2.81
0.94
0.93
0.89
1.05
2.70
0.93
0.96
0.91
0.98
3.00
2.84
0.99
0.90
0.89
0.91
0.84
3.66
f1 (ppm)

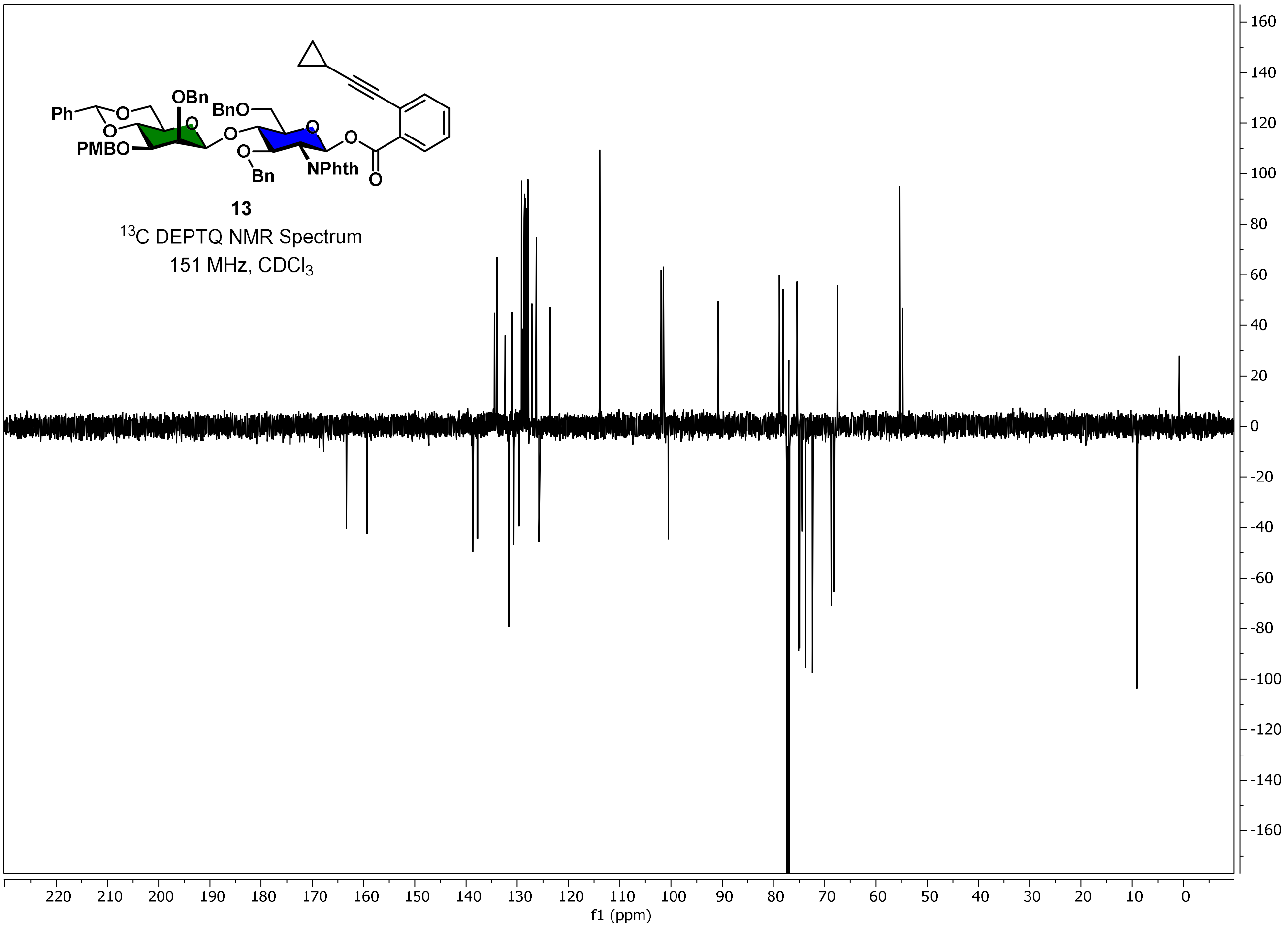

13
^{13}C DEPTQ NMR Spectrum
151 MHz, CDCl$_3$
f1 (ppm)

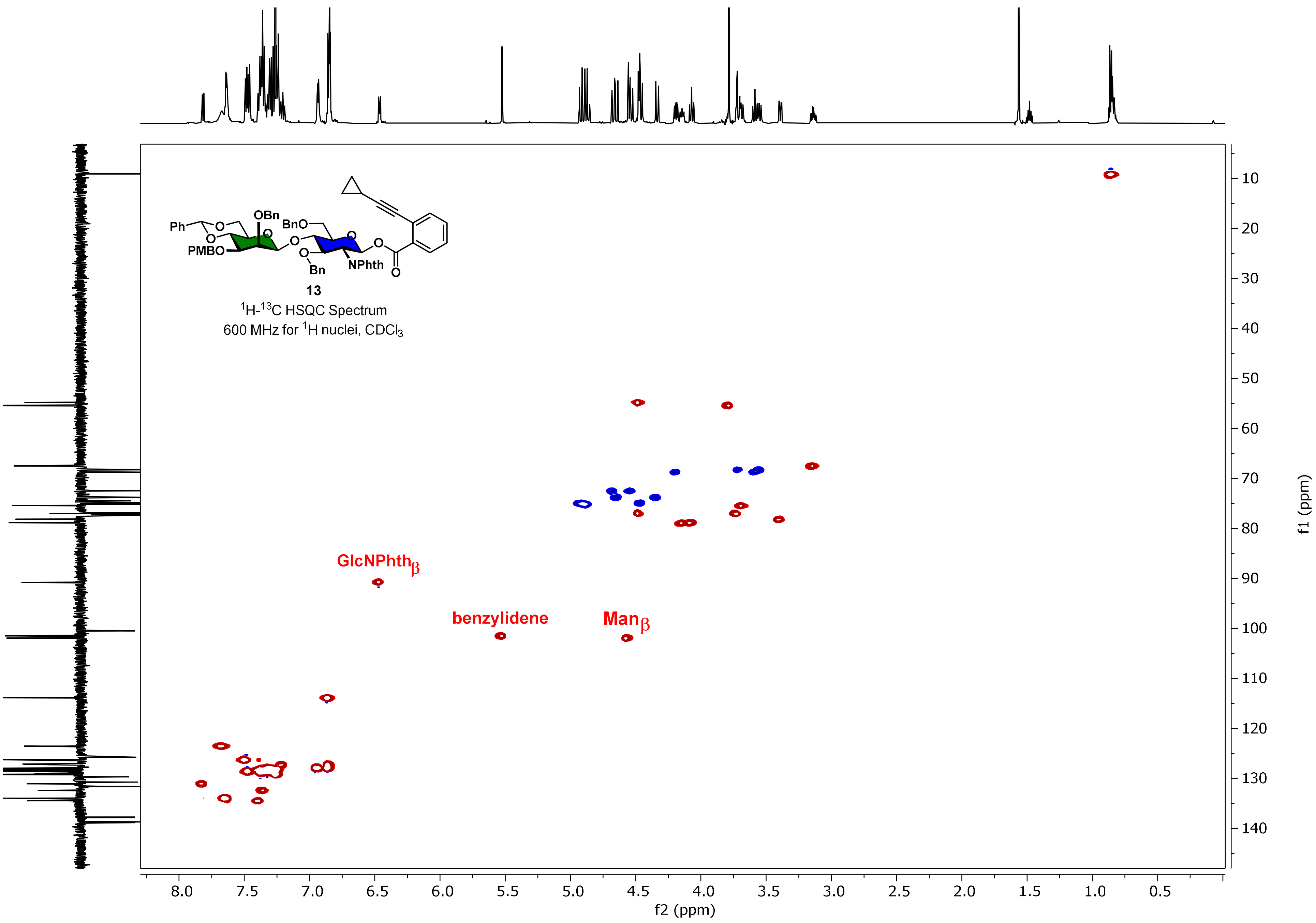

Ph
O
O
OBn
O
BnO
O
O
PMBO
O
Bn
NPhth
O
O
13
^{1}H-^{13}C HSQC Spectrum
600 MHz for ^{1}H nuclei, CDCl$_3$
GlcNPhth$_\beta$
benzylidene
Man$_\beta$
f2 (ppm)
f1 (ppm)

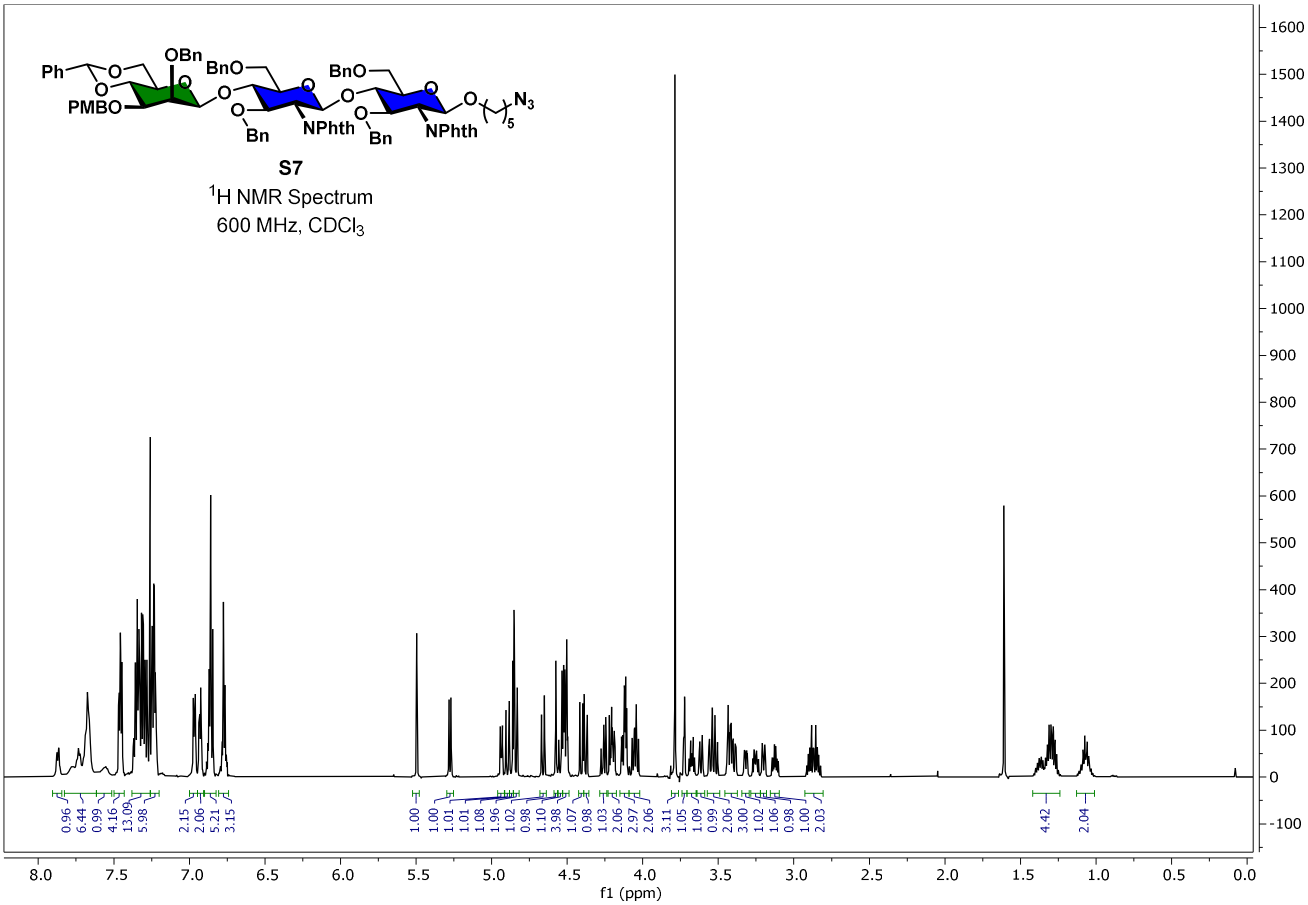
Ph
OBn
BnO
BnO
PMBO
Bn
NPhth
Bn
NPhth
N_3
S7
^{1}H NMR Spectrum
600 MHz, $CDCl_3$
f1 (ppm)

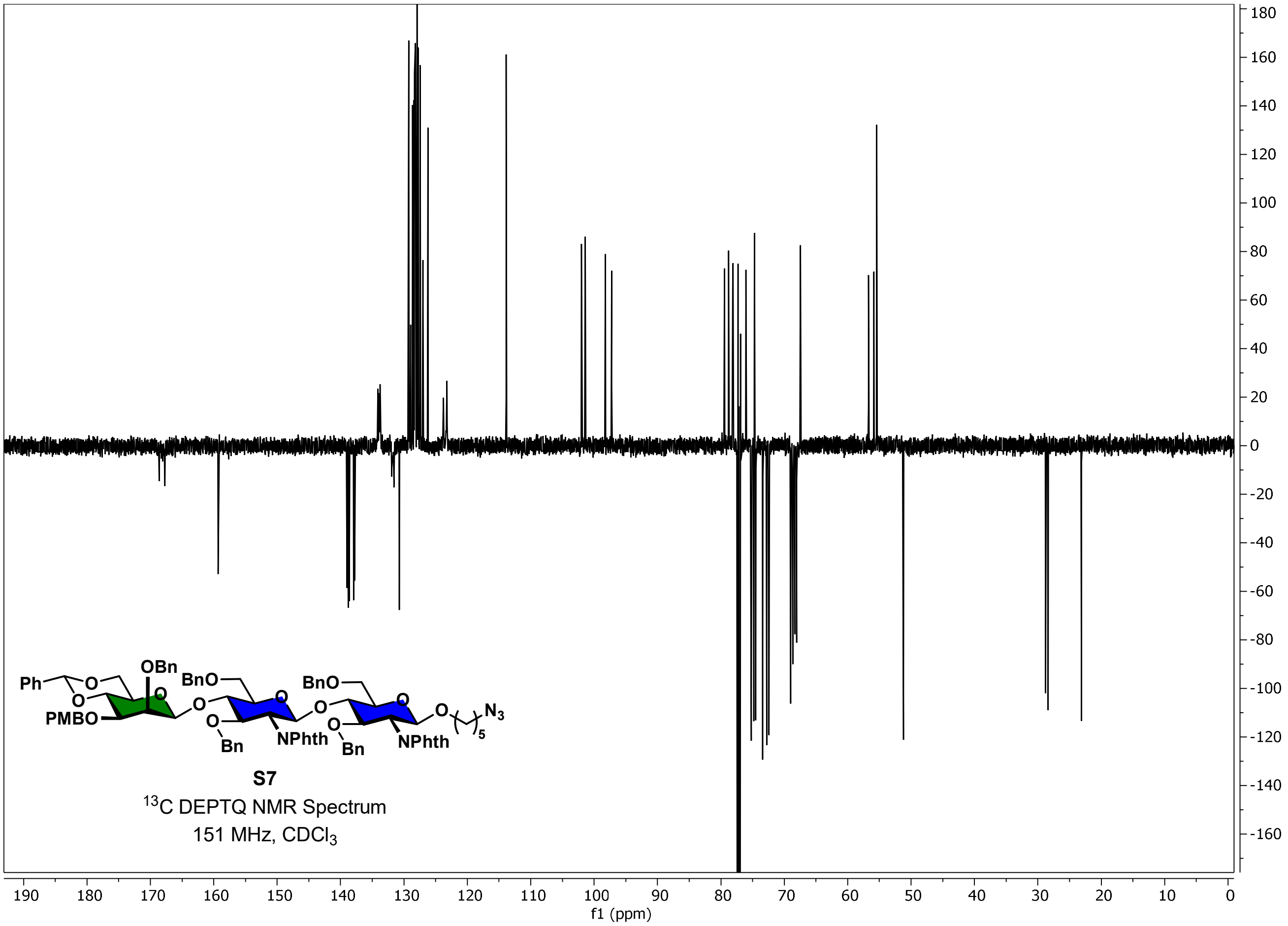
Ph
OBn
BnO
BnO
PMBO
Bn
NPhth
Bn
NPhth
N_3
S7
^{13}C DEPTQ NMR Spectrum
151 MHz, $CDCl_3$
f1 (ppm)

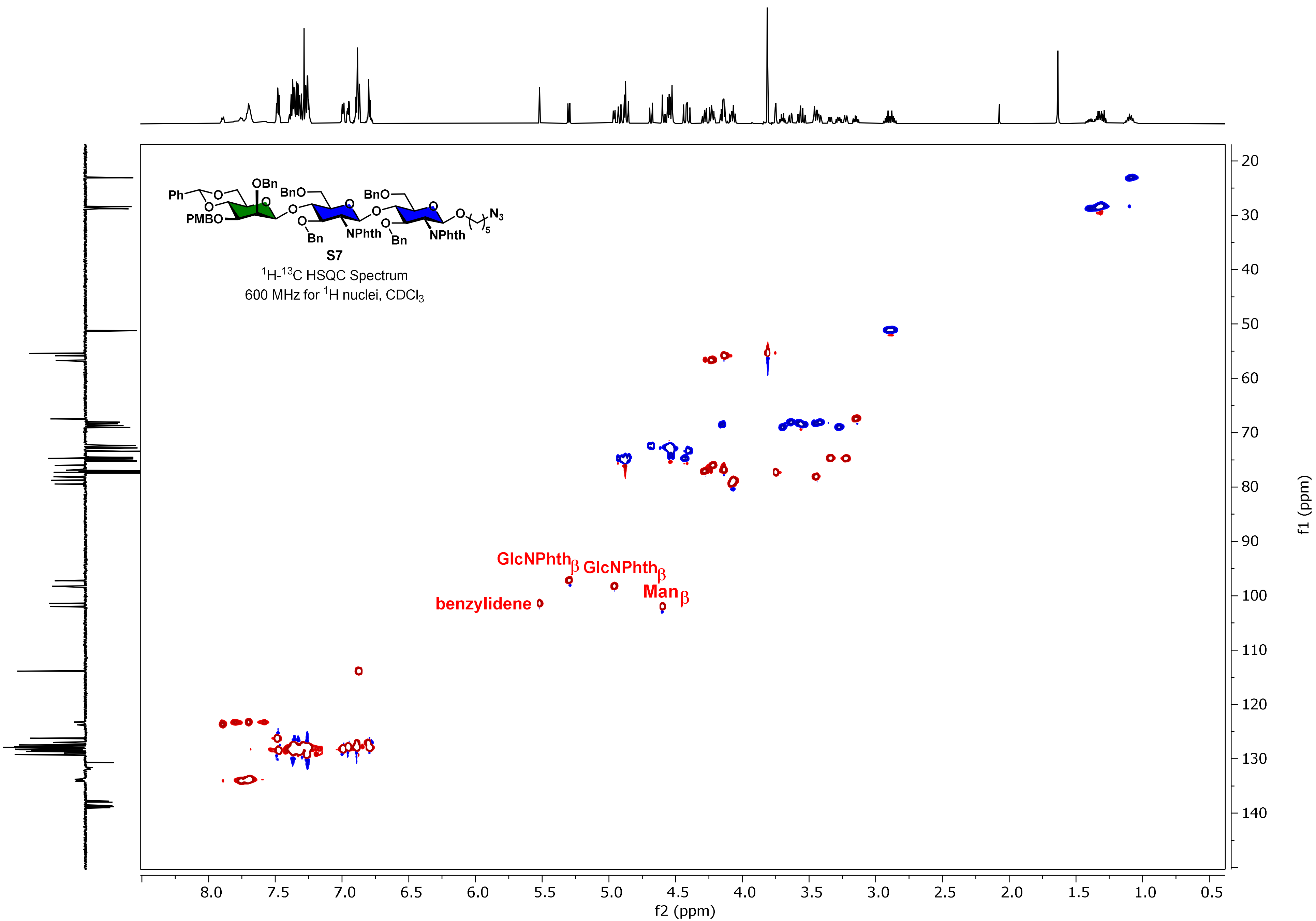

S7
^{1}H-^{13}C HSQC Spectrum
600 MHz for ^{1}H nuclei, CDCl$_3$
GlcNPhth$_β$
GlcNPhth$_β$
Man$_β$
benzylidene
f2 (ppm)
f1 (ppm)

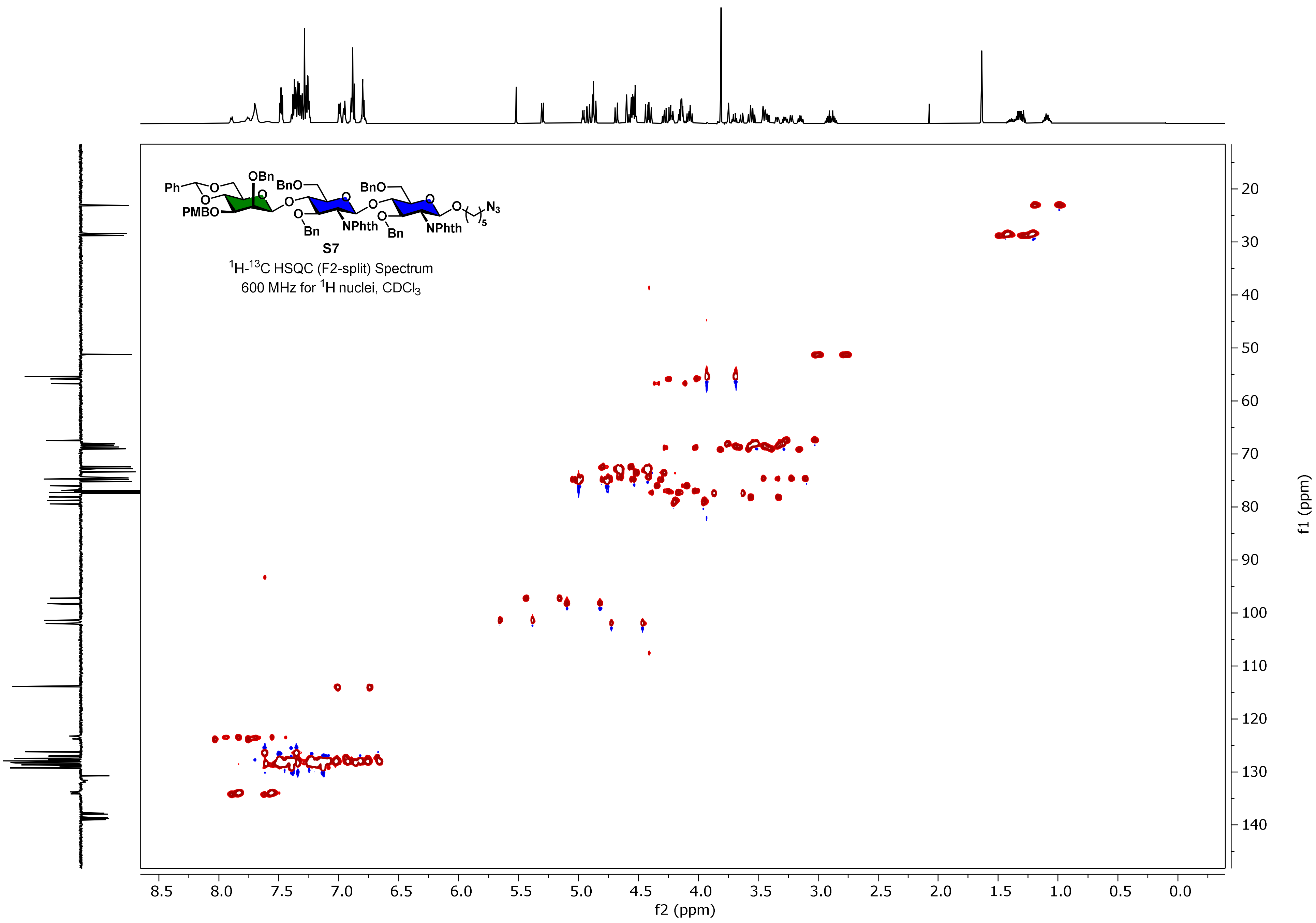

Ph
O
O
OBn
O
PMBO
BnO
O
O
Bn
NPhth
BnO
O
O
O
Bn
NPhth
O
N3
5
S7
1H-13C HSQC (F2-split) Spectrum
600 MHz for 1H nuclei, CDCl3
f2 (ppm)
f1 (ppm)

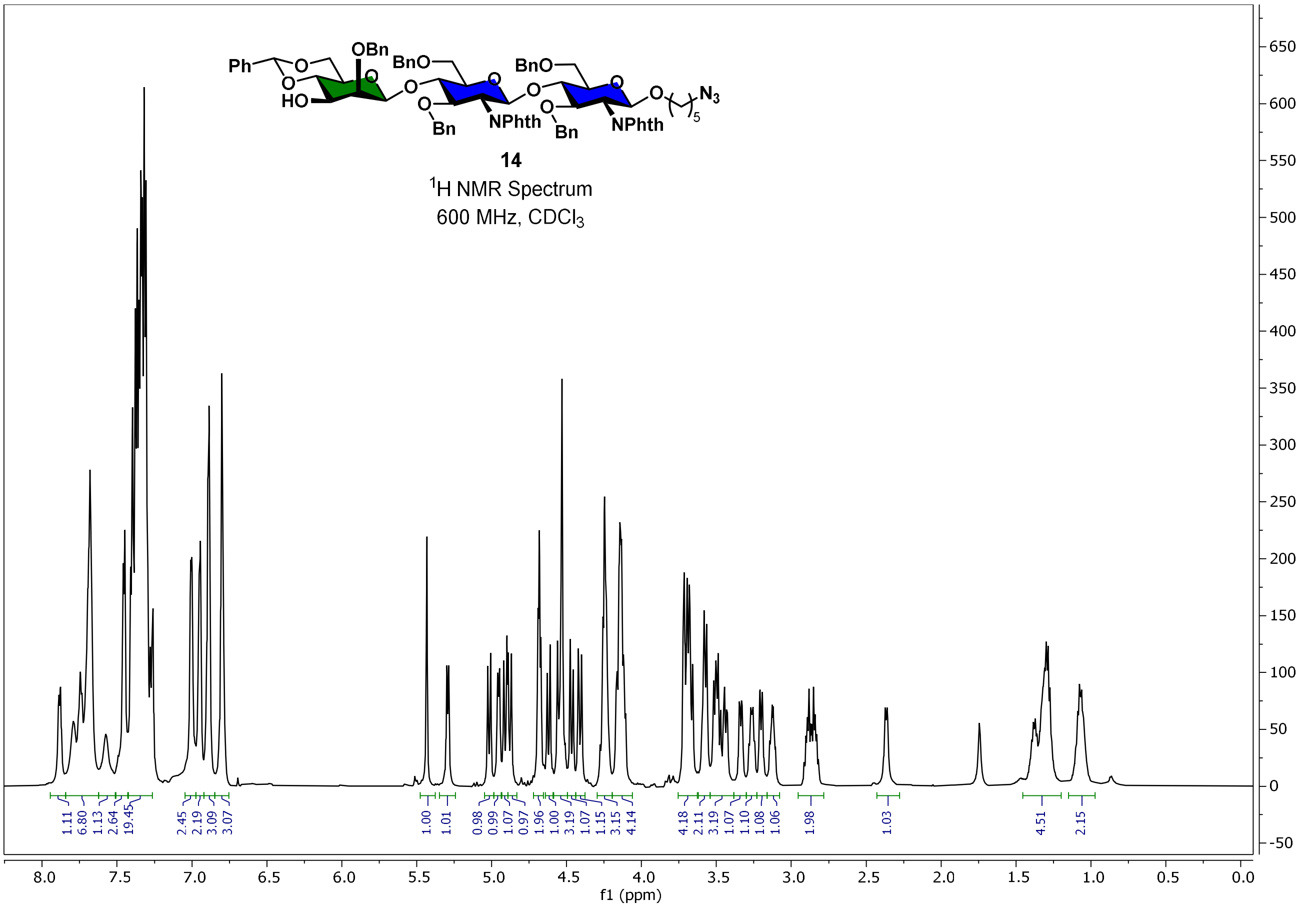
14
1H NMR Spectrum
600 MHz, CDCl3
f1 (ppm)

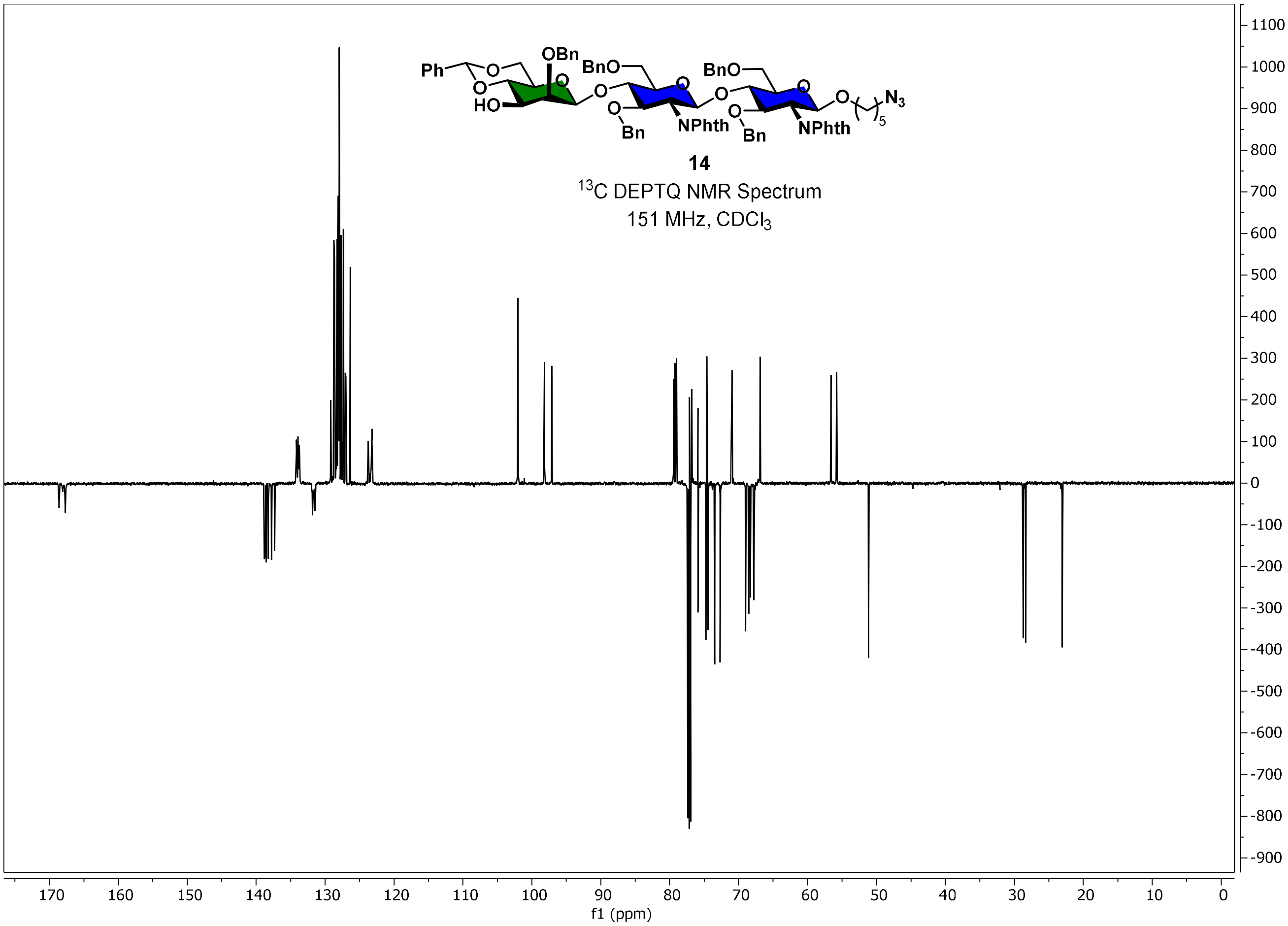
14
13C DEPTQ NMR Spectrum
151 MHz, CDCl3
f1 (ppm)

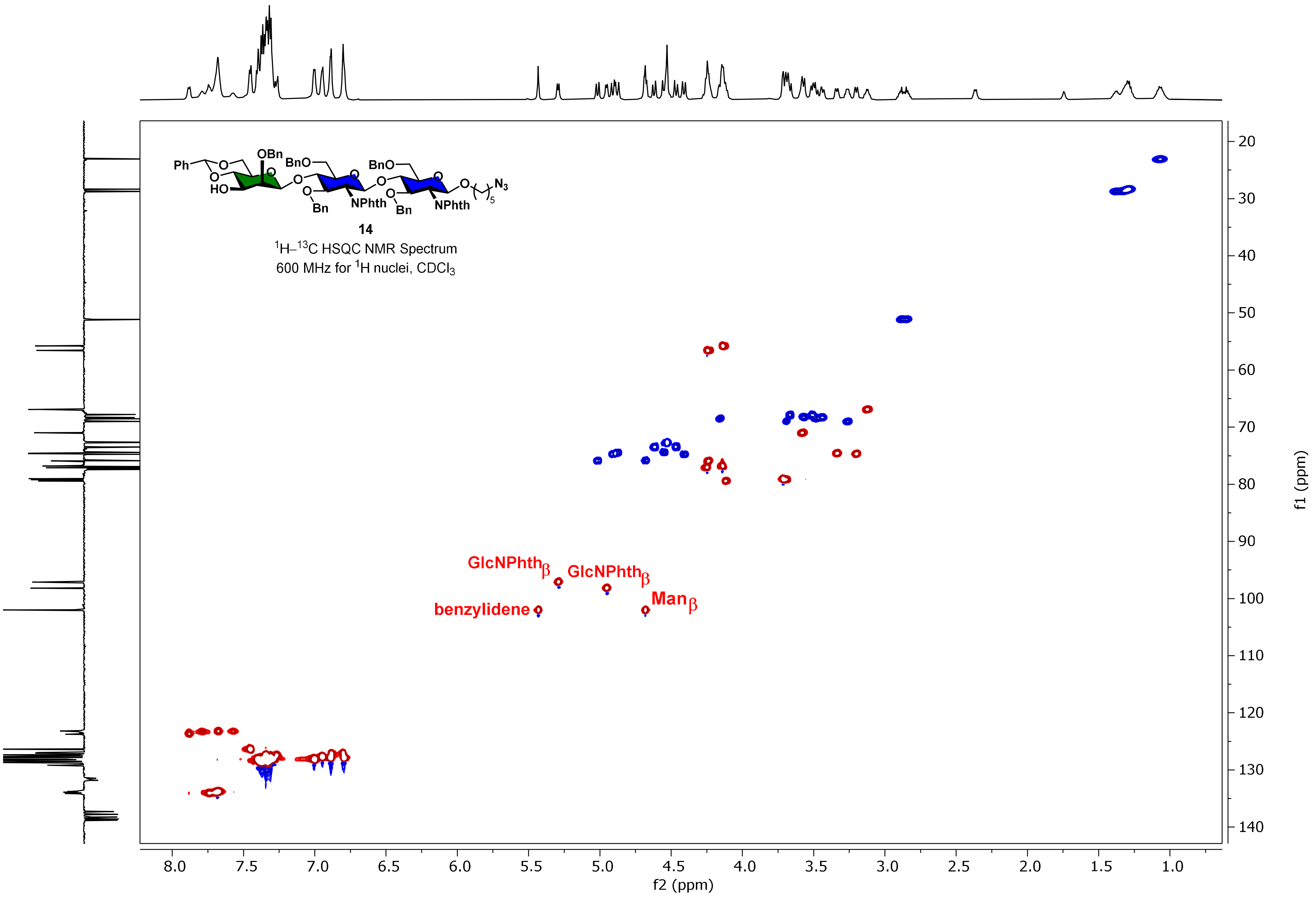
Ph
OBn
BnO
HO
Bn
NPhth
N3
14
1H–13C HSQC NMR Spectrum
600 MHz for 1H nuclei, CDCl3
GlcNPhthβ
GlcNPhthβ
Manβ
benzylidene
f2 (ppm)
f1 (ppm)

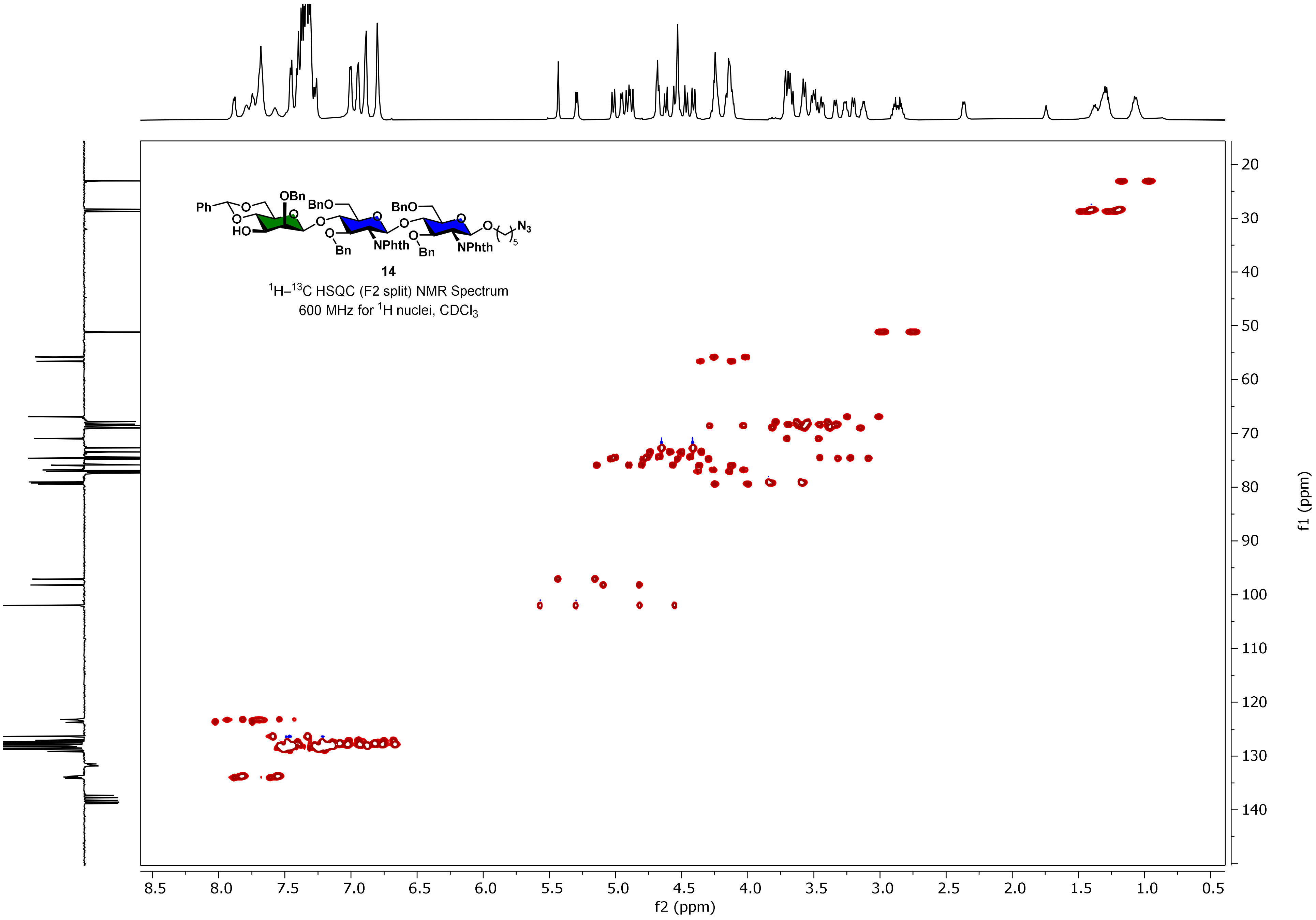
14
^{1}H–^{13}C HSQC (F2 split) NMR Spectrum
600 MHz for ^{1}H nuclei, $CDCl_3$
f2 (ppm)
f1 (ppm)

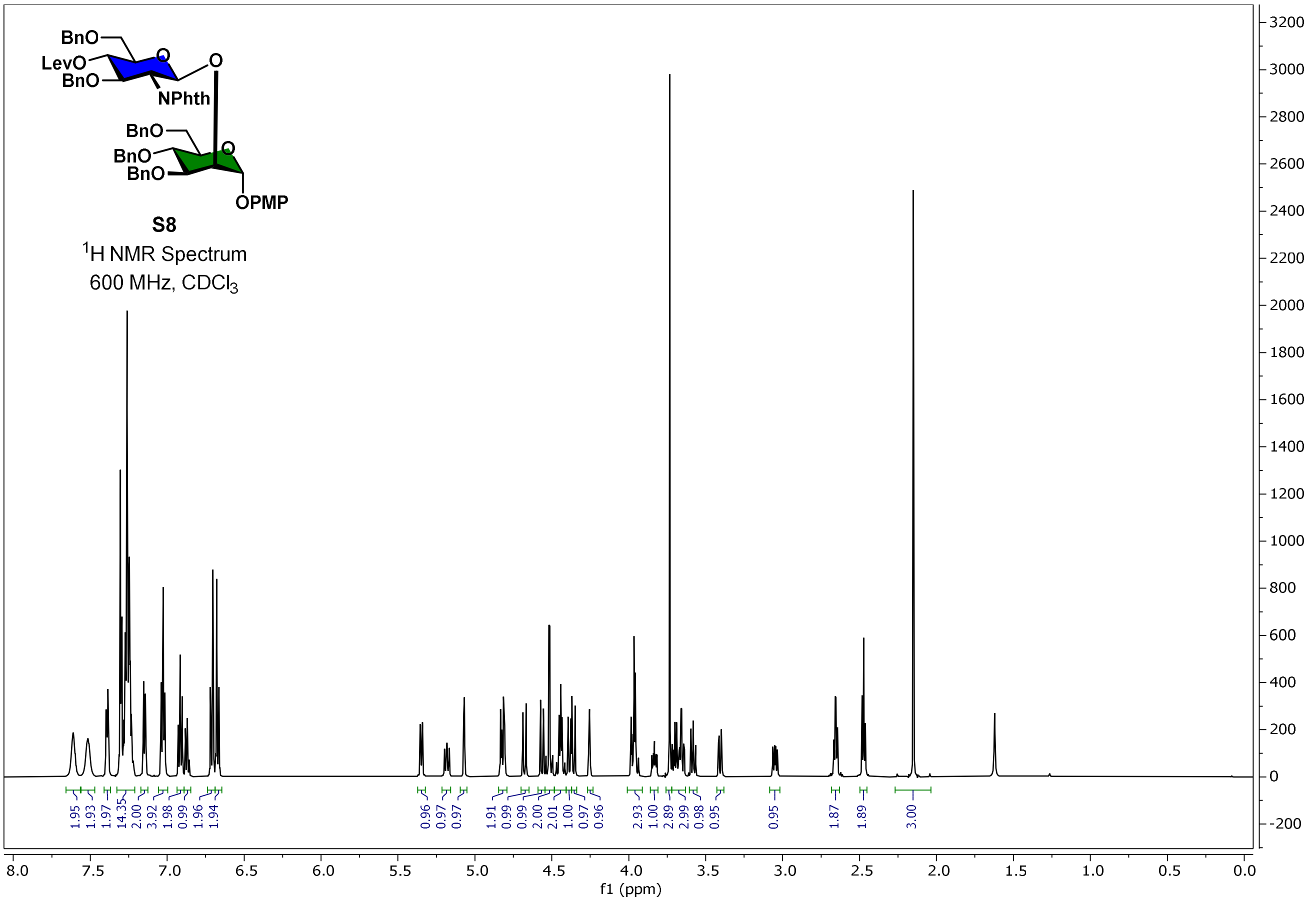
BnO
LevO
BnO
NPhth
BnO
BnO
BnO
OPMP
S8
^{1}H NMR Spectrum
600 MHz, $CDCl_3$
f1 (ppm)

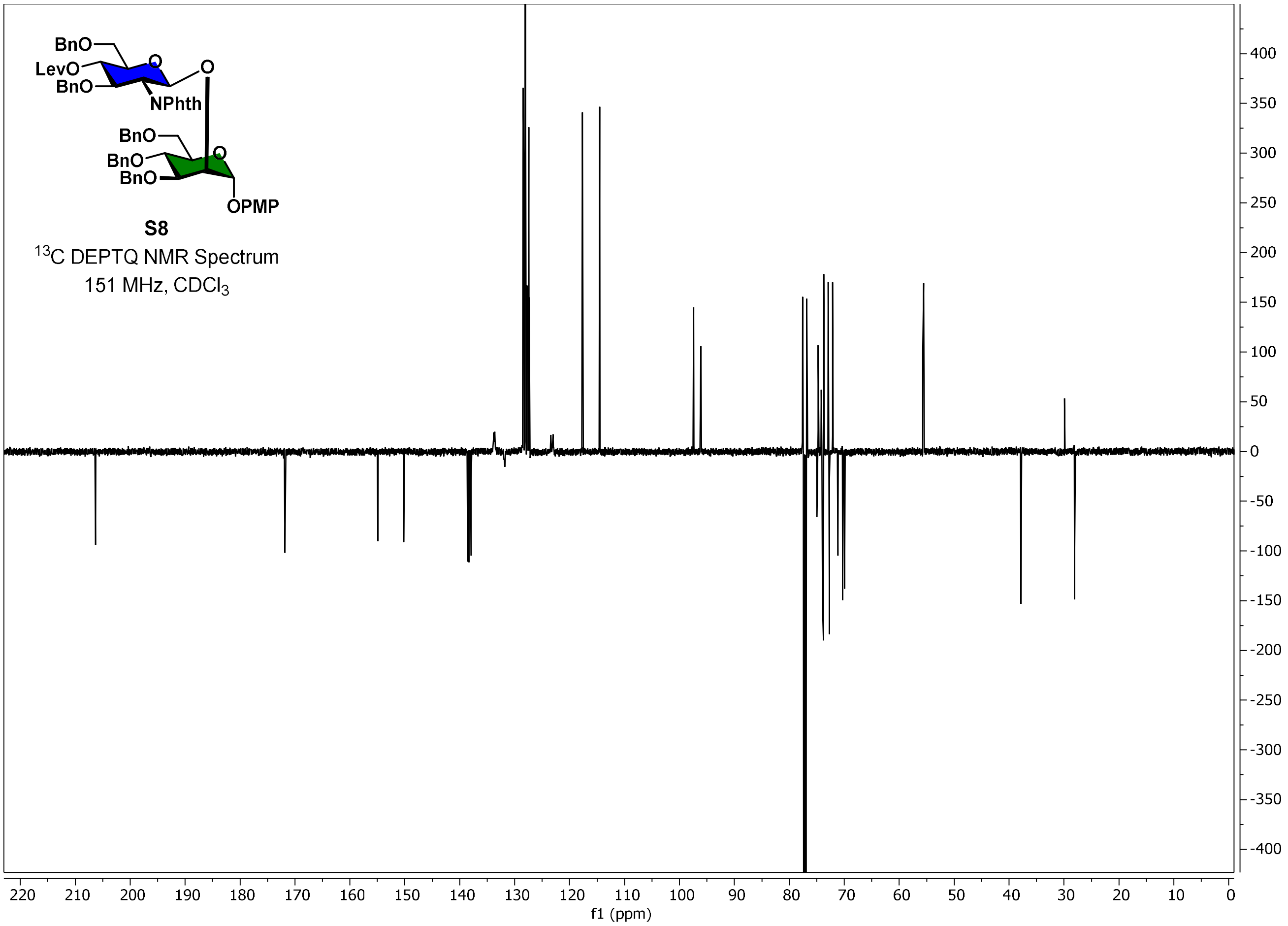
BnO
LevO
BnO
NPhth
BnO
BnO
BnO
OPMP
S8
^{13}C DEPTQ NMR Spectrum
151 MHz, $CDCl_3$
f1 (ppm)

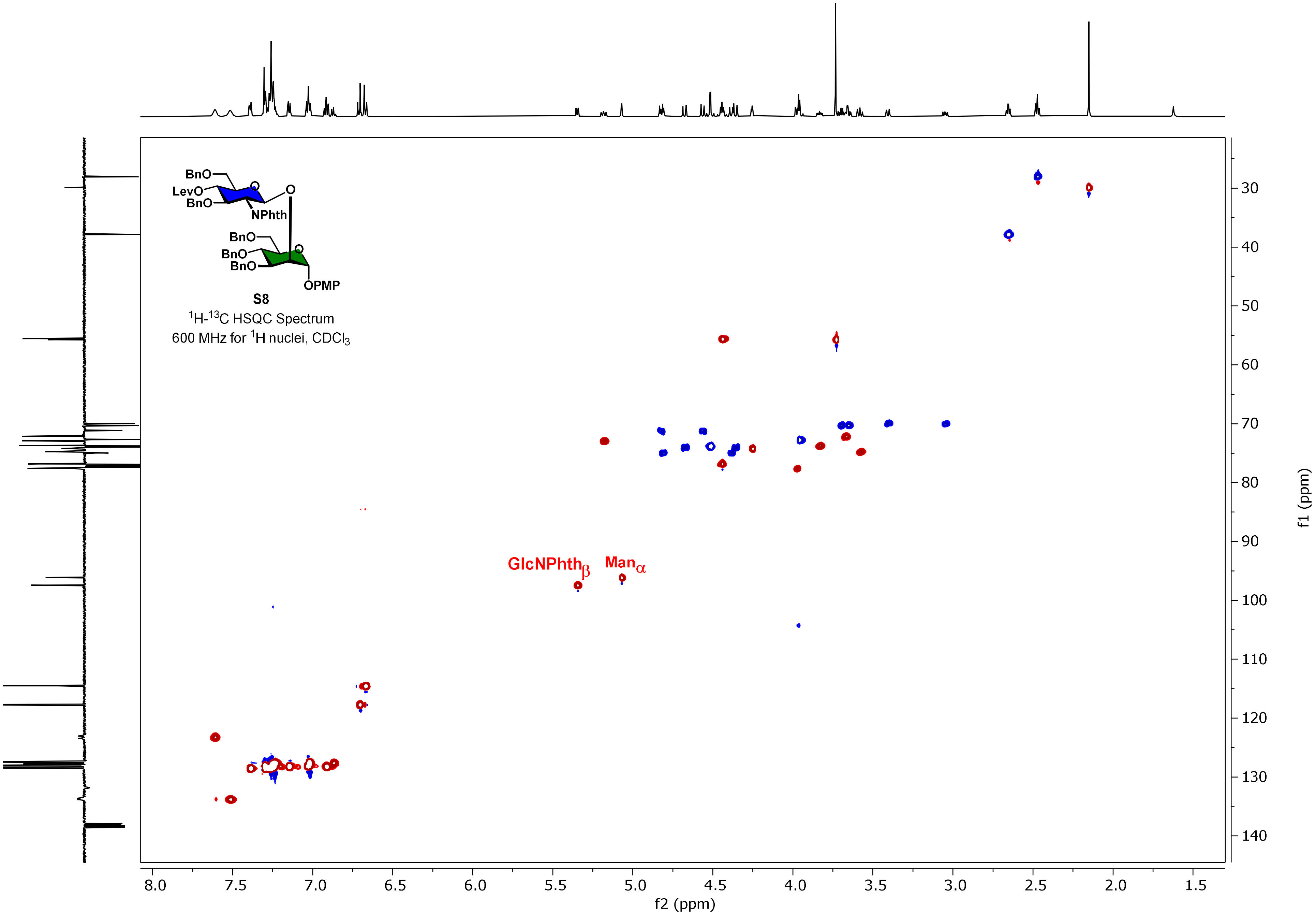
BnO
LevO
BnO
NPhth
BnO
BnO
BnO
OPMP
S8
1H-13C HSQC Spectrum
600 MHz for 1H nuclei, CDCl3
GlcNPhthβ
Manα
8.0 7.5 7.0 6.5 6.0 5.5 5.0 4.5 4.0 3.5 3.0 2.5 2.0 1.5
f2 (ppm)
30 40 50 60 70 80 90 100 110 120 130 140
f1 (ppm)

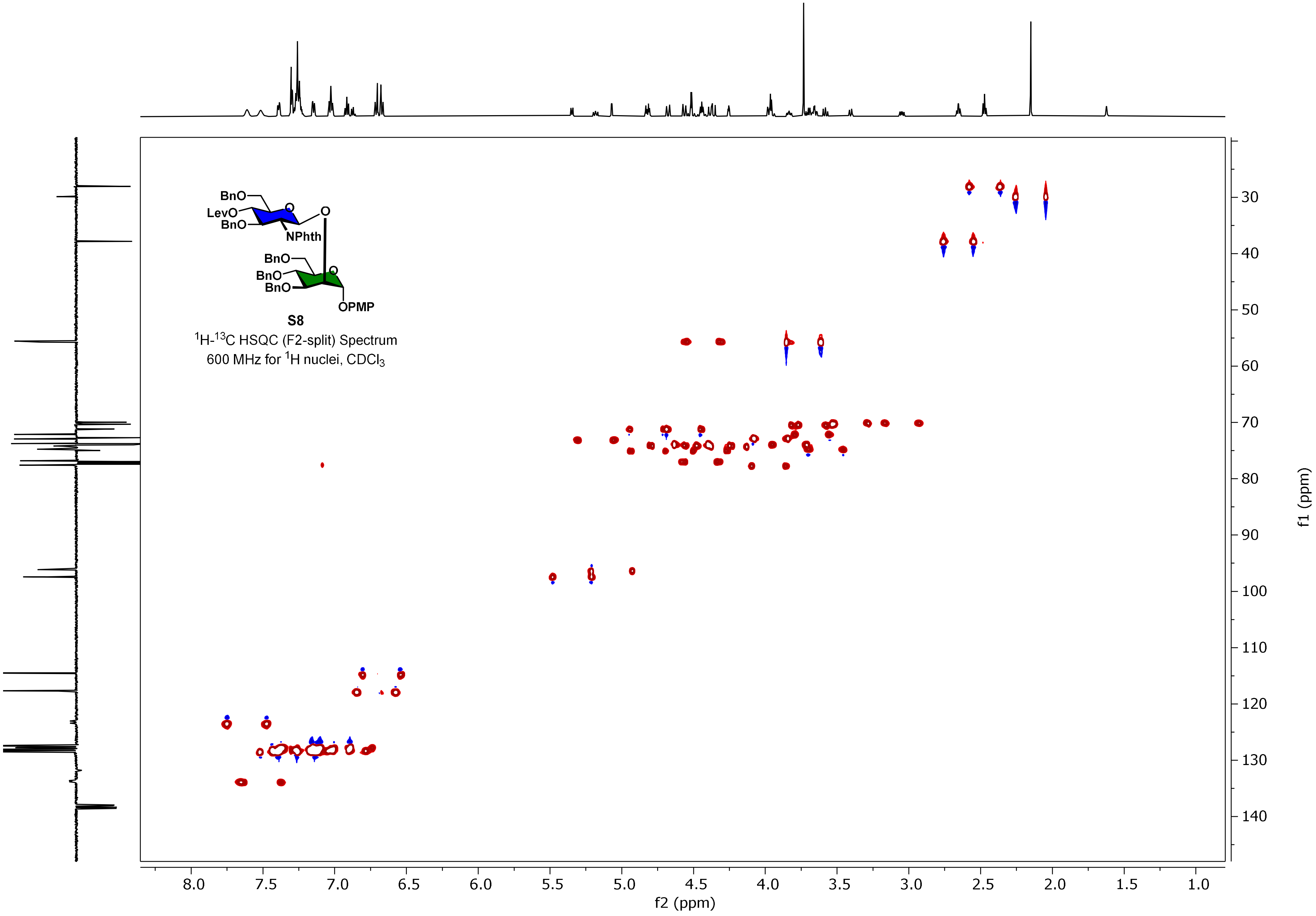

BnO
LevO
BnO
O
O
NPhth
BnO
BnO
BnO
O
OPMP
S8
^{1}H-^{13}C HSQC (F2-split) Spectrum
600 MHz for ^{1}H nuclei, $CDCl_3$
f2 (ppm)
f1 (ppm)

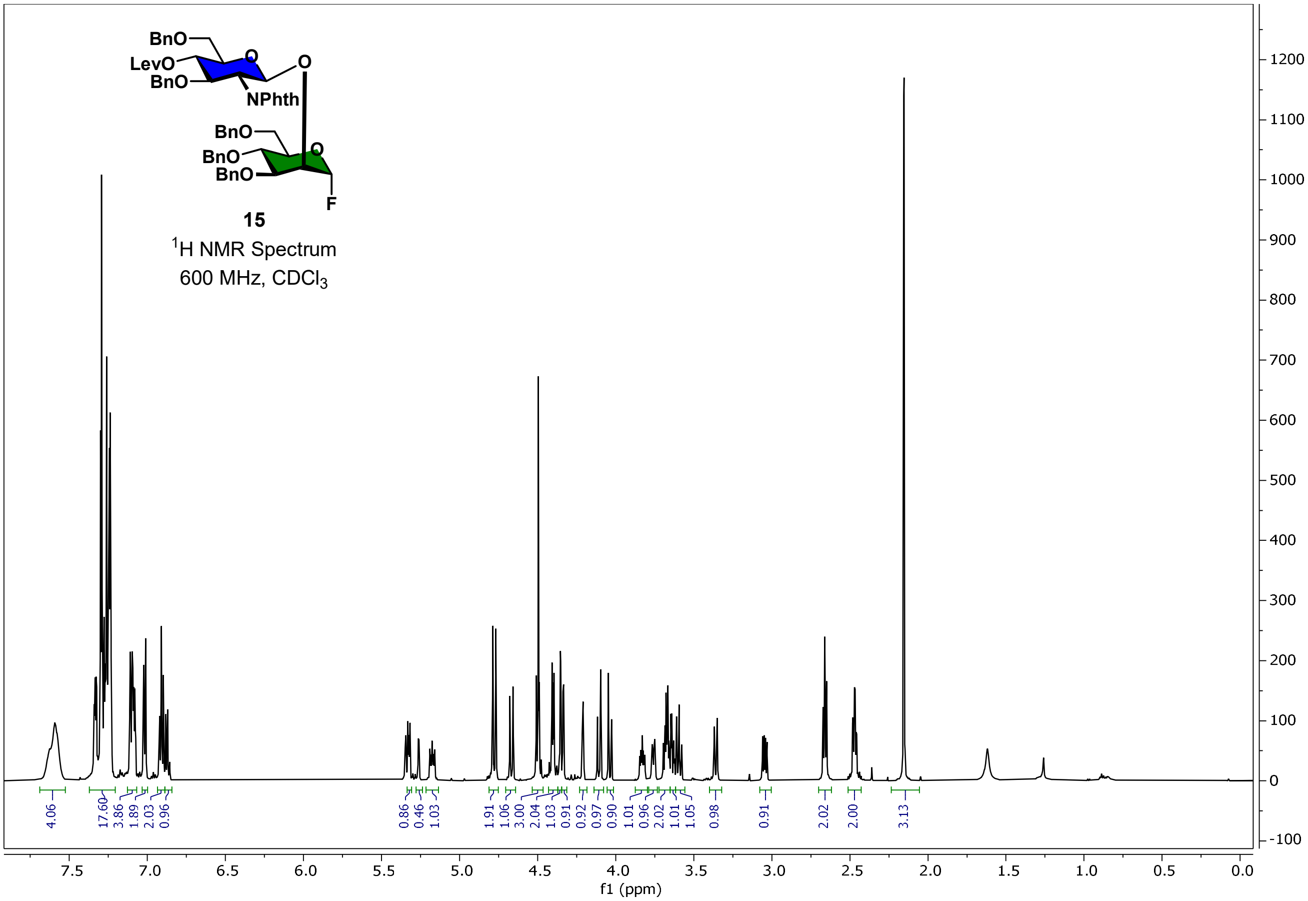
BnO
LevO
BnO
O
O
NPhth
BnO
BnO
BnO
O
F
15
^{1}H NMR Spectrum
600 MHz, $CDCl_3$
f1 (ppm)

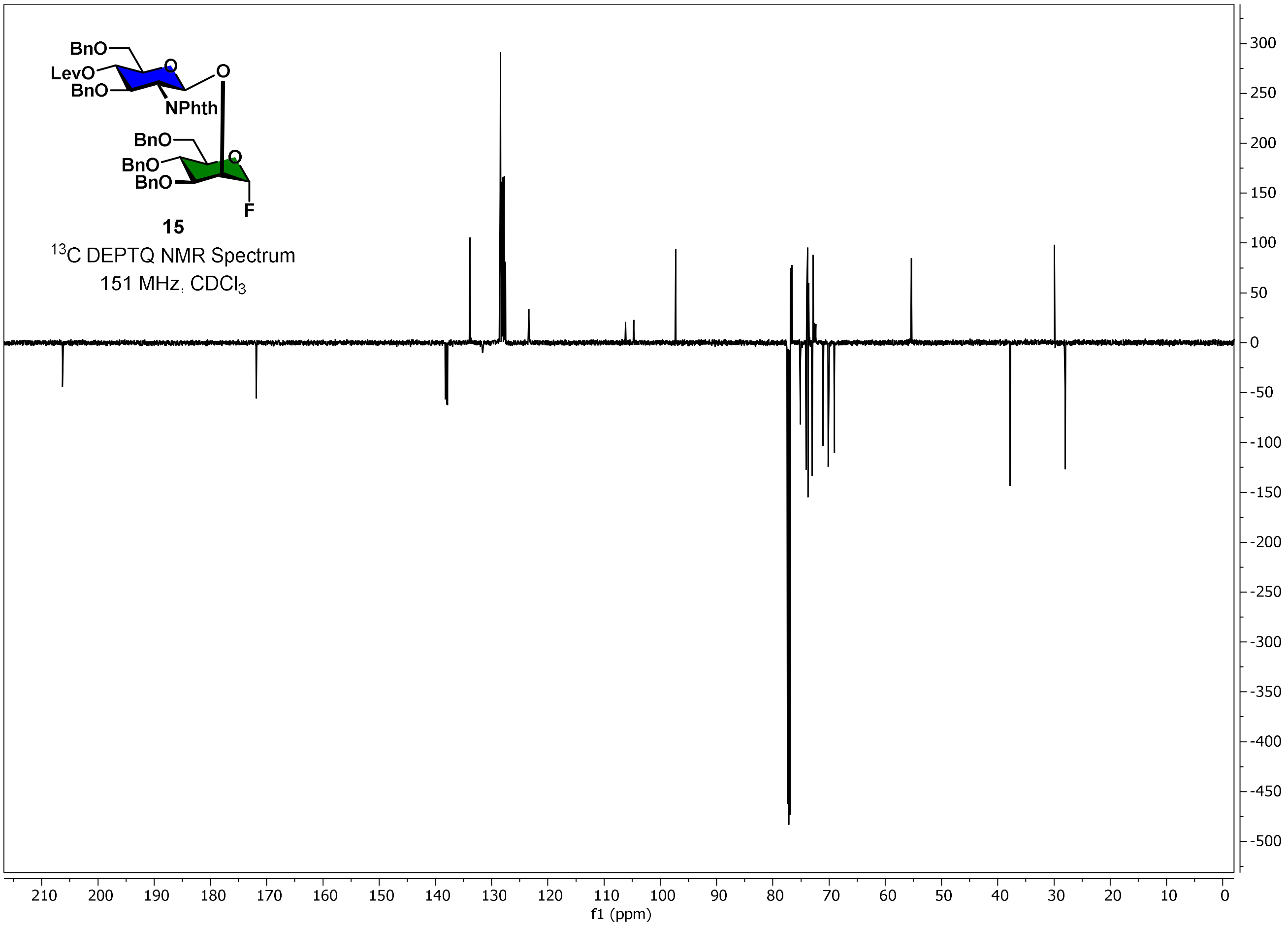
BnO
LevO
BnO
O
O
NPhth
BnO
BnO
BnO
O
F
15
^{13}C DEPTQ NMR Spectrum
151 MHz, $CDCl_3$
f1 (ppm)

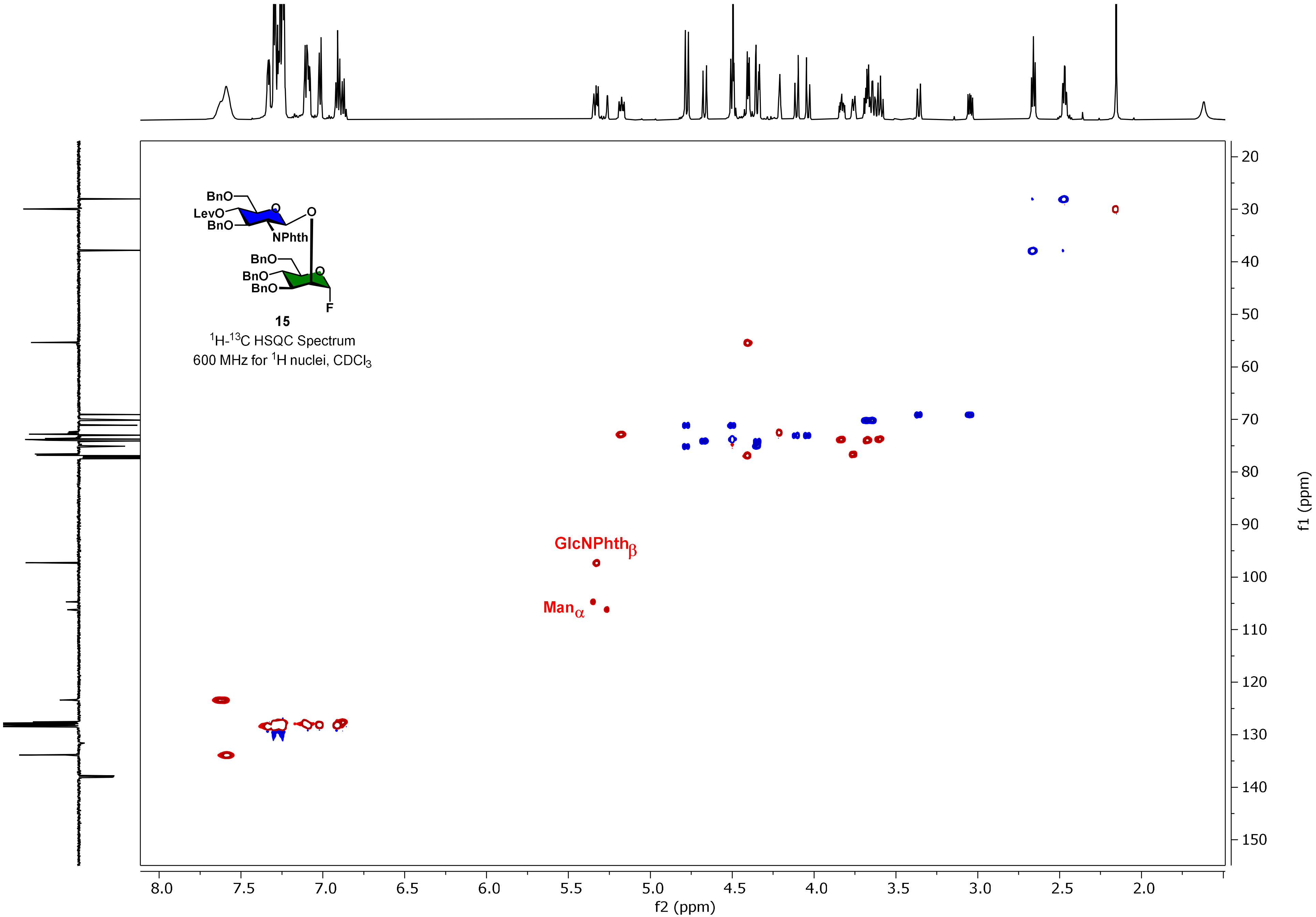
BnO
LevO
BnO
O
O
NPhth
BnO
BnO
BnO
O
F
15
^{1}H-^{13}C HSQC Spectrum
600 MHz for ^{1}H nuclei, CDCl$_3$
GlcNPhth$_\beta$
Man$_\alpha$
f2 (ppm)
f1 (ppm)

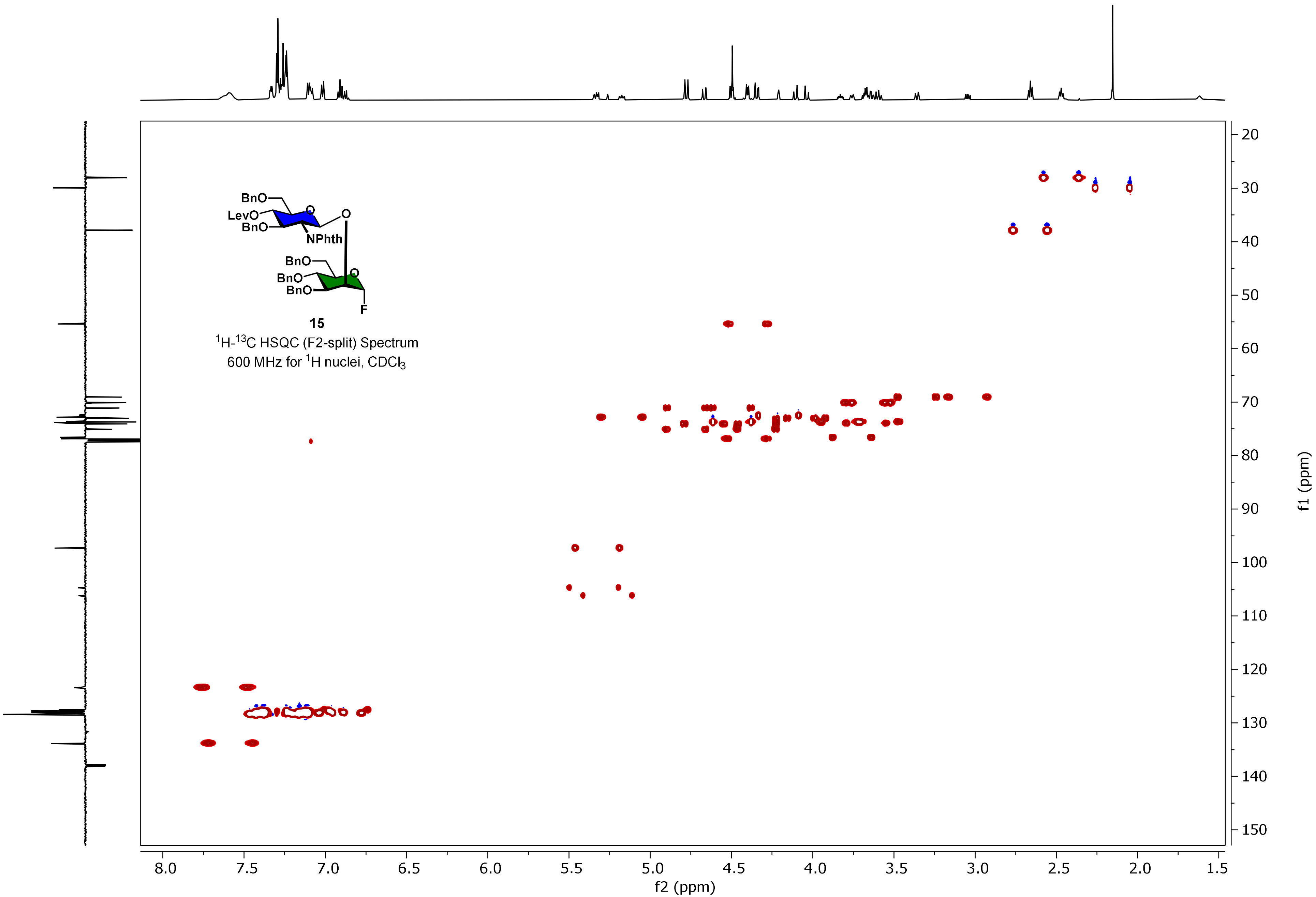
BnO
LevO
BnO
NPhth
BnO
BnO
BnO
O
F
15
1H-13C HSQC (F2-split) Spectrum
600 MHz for 1H nuclei, CDCl3
f2 (ppm)
f1 (ppm)

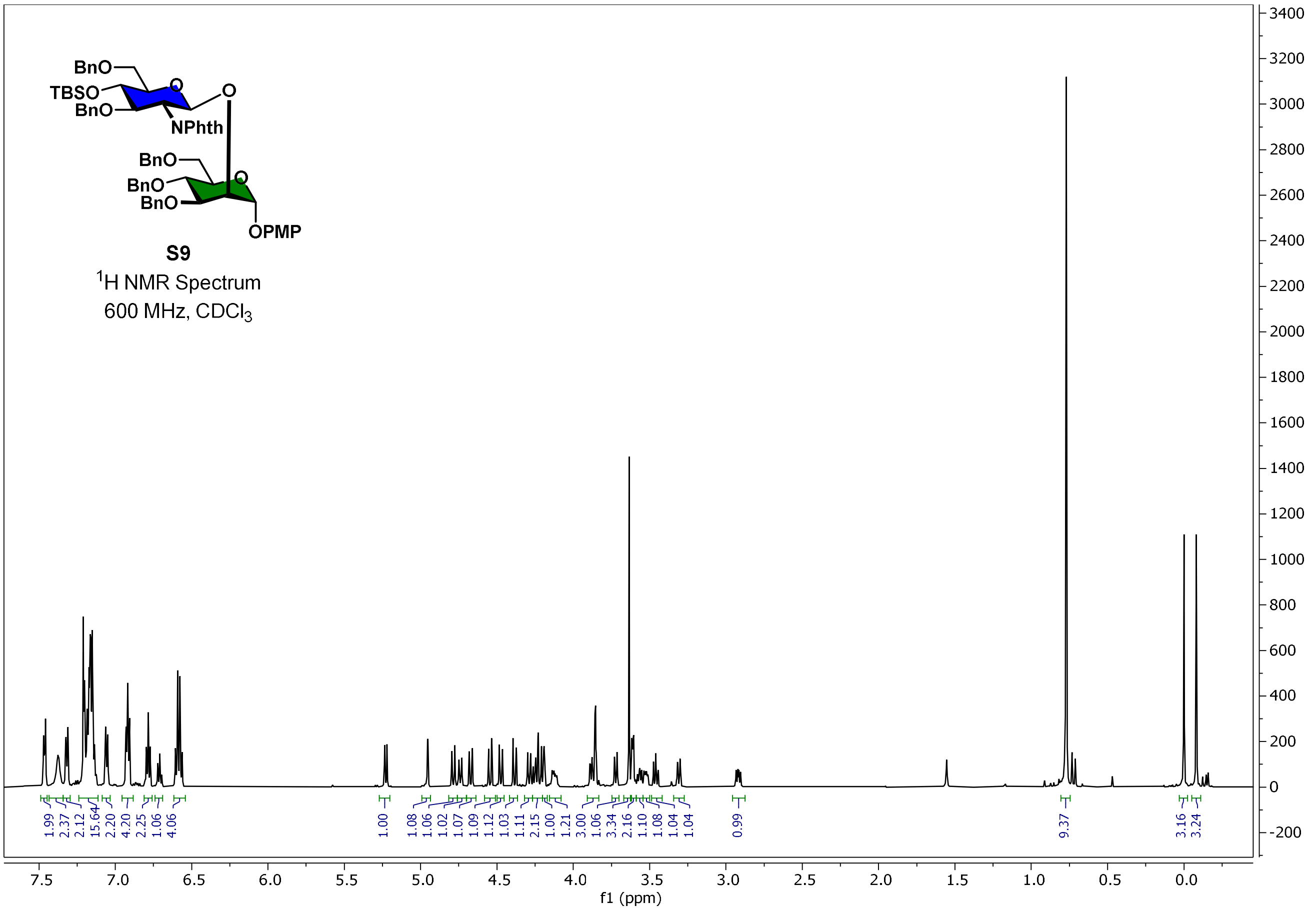
BnO
TBSO
BnO
O
O
NPhth
BnO
BnO
BnO
BnO
O
OPMP
S9
^{1}H NMR Spectrum
600 MHz, $CDCl_3$
f1 (ppm)

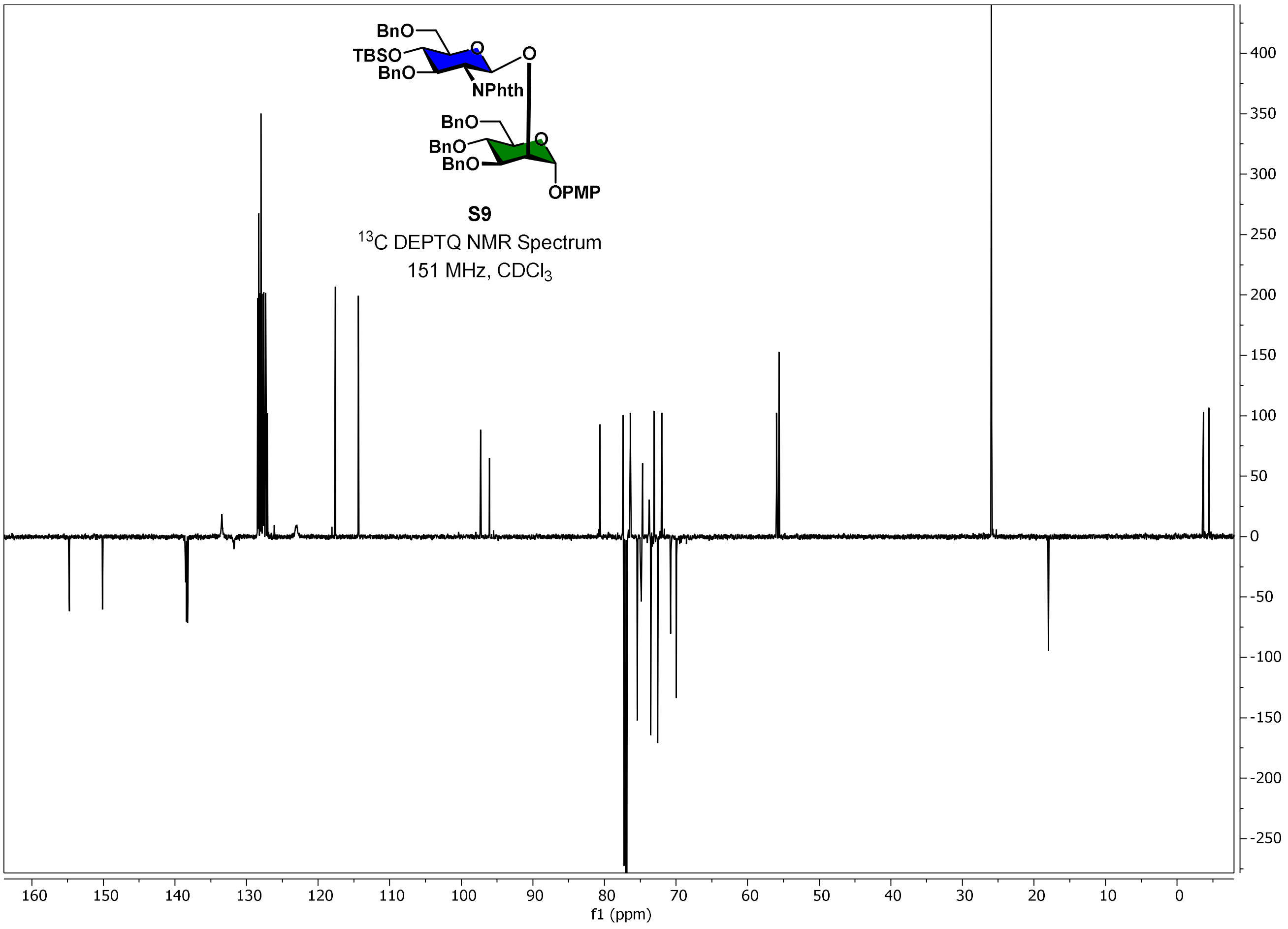
BnO
TBSO
BnO
O
O
NPhth
BnO
BnO
BnO
O
OPMP
S9
^{13}C DEPTQ NMR Spectrum
151 MHz, $CDCl_3$
f1 (ppm)

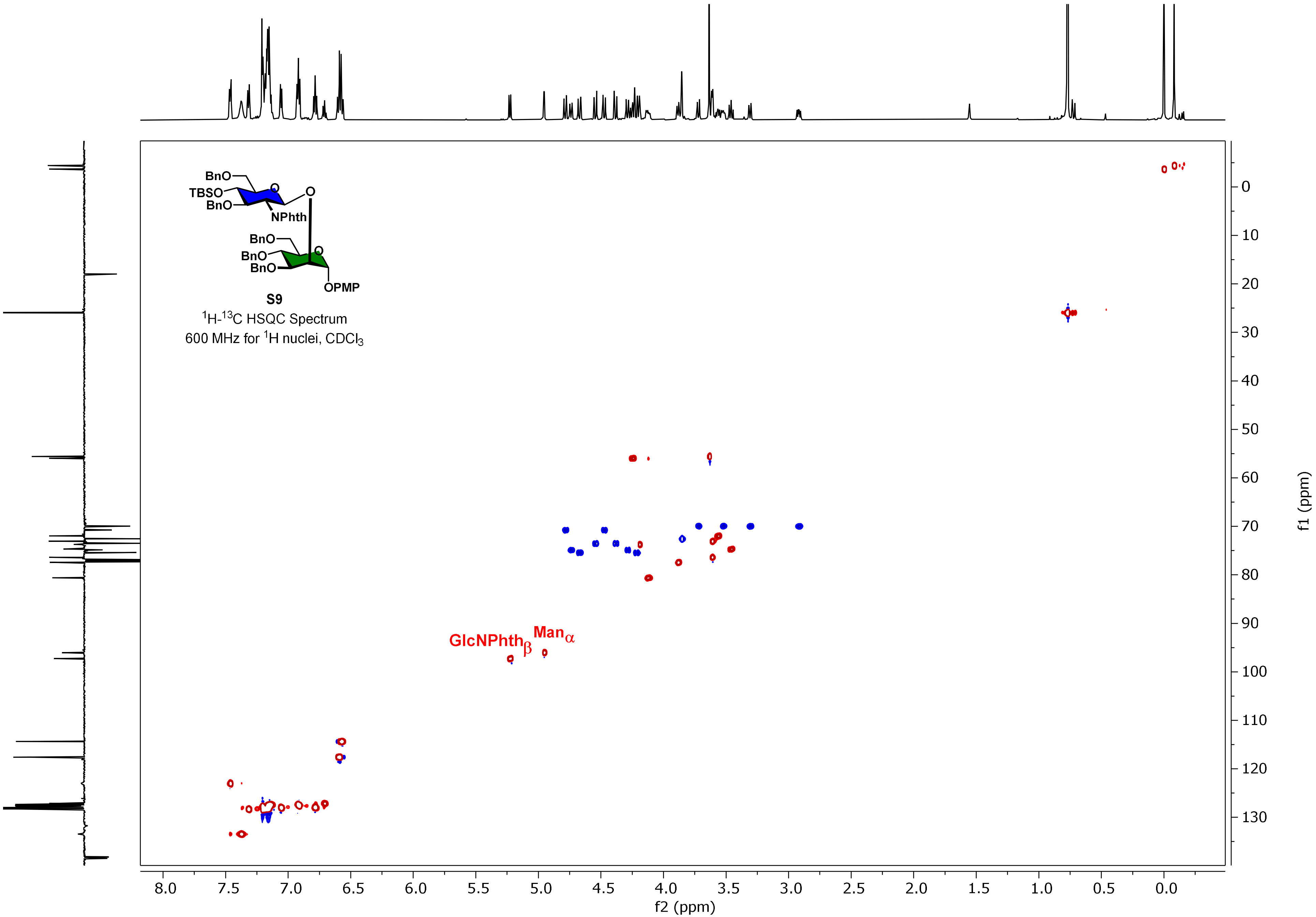
BnO
TBSO
BnO
NPhth
BnO
BnO
BnO
OPMP
S9
^{1}H-^{13}C HSQC Spectrum
600 MHz for ^{1}H nuclei, CDCl$_3$
GlcNPhth$_\beta$
Man$_\alpha$
f2 (ppm)
f1 (ppm)

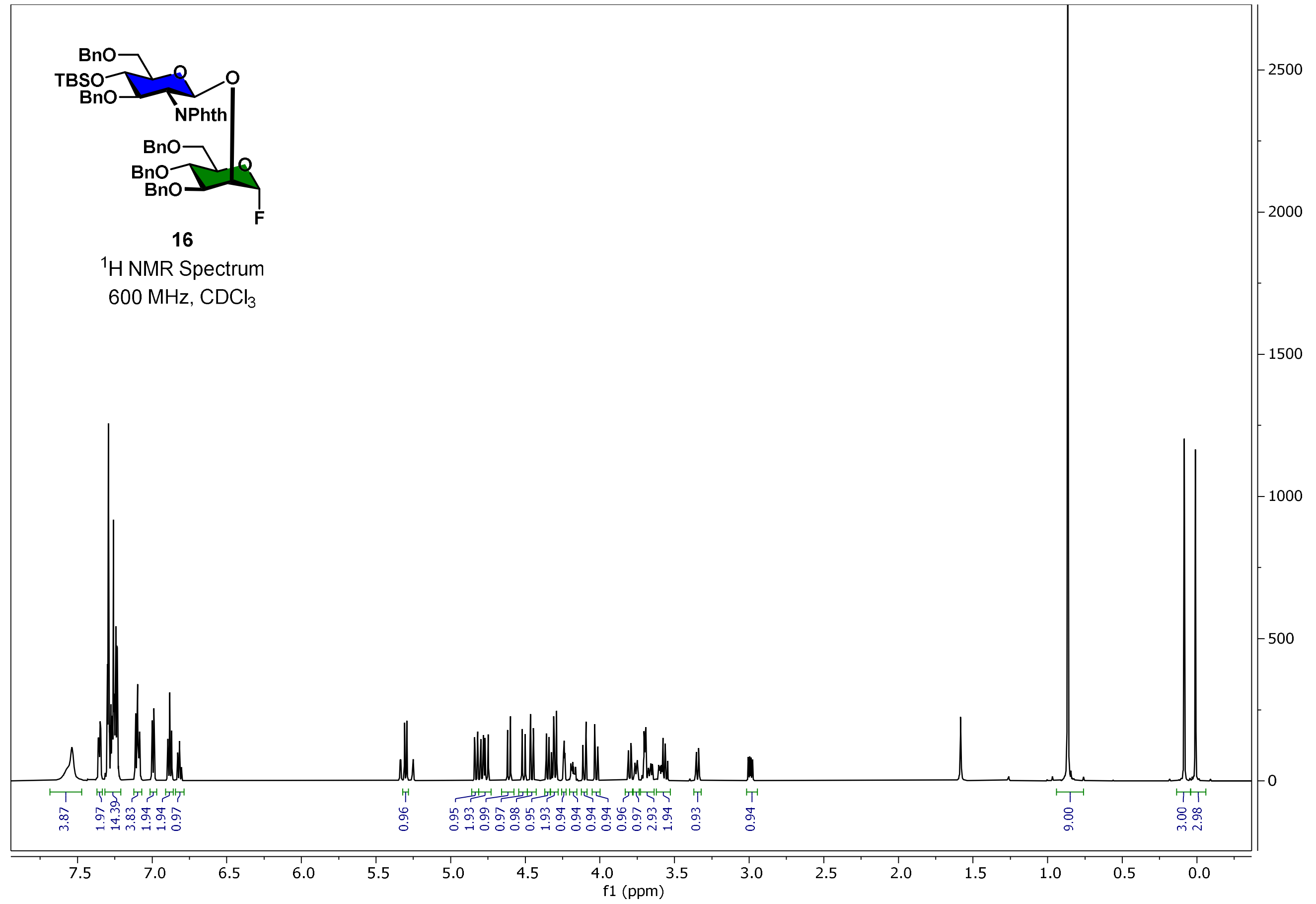
BnO
TBSO
BnO
NPhth
BnO
BnO
BnO
F
16
^{1}H NMR Spectrum
600 MHz, CDCl$_3$
f1 (ppm)
3.87 1.97 14.39 3.83 1.94 1.94 0.97 0.96 0.95 1.93 0.99 0.97 0.98 0.95 1.93 0.94 0.94 0.94 0.94 0.96 0.97 2.93 1.94 0.93 0.94 9.00 3.00 2.98

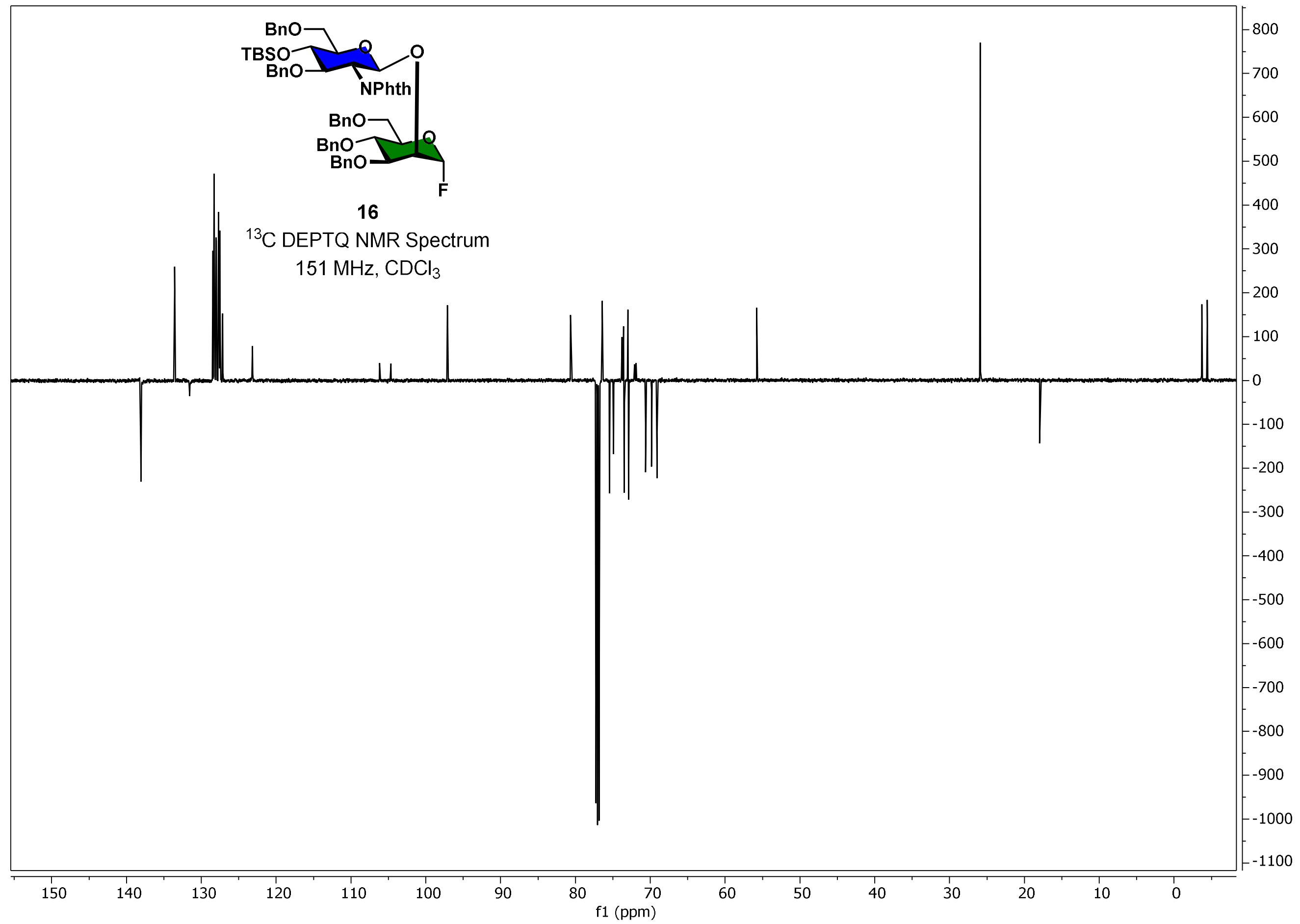
BnO
TBSO
BnO
NPhth
BnO
BnO
BnO
F
16
^{13}C DEPTQ NMR Spectrum
151 MHz, CDCl$_3$
f1 (ppm)

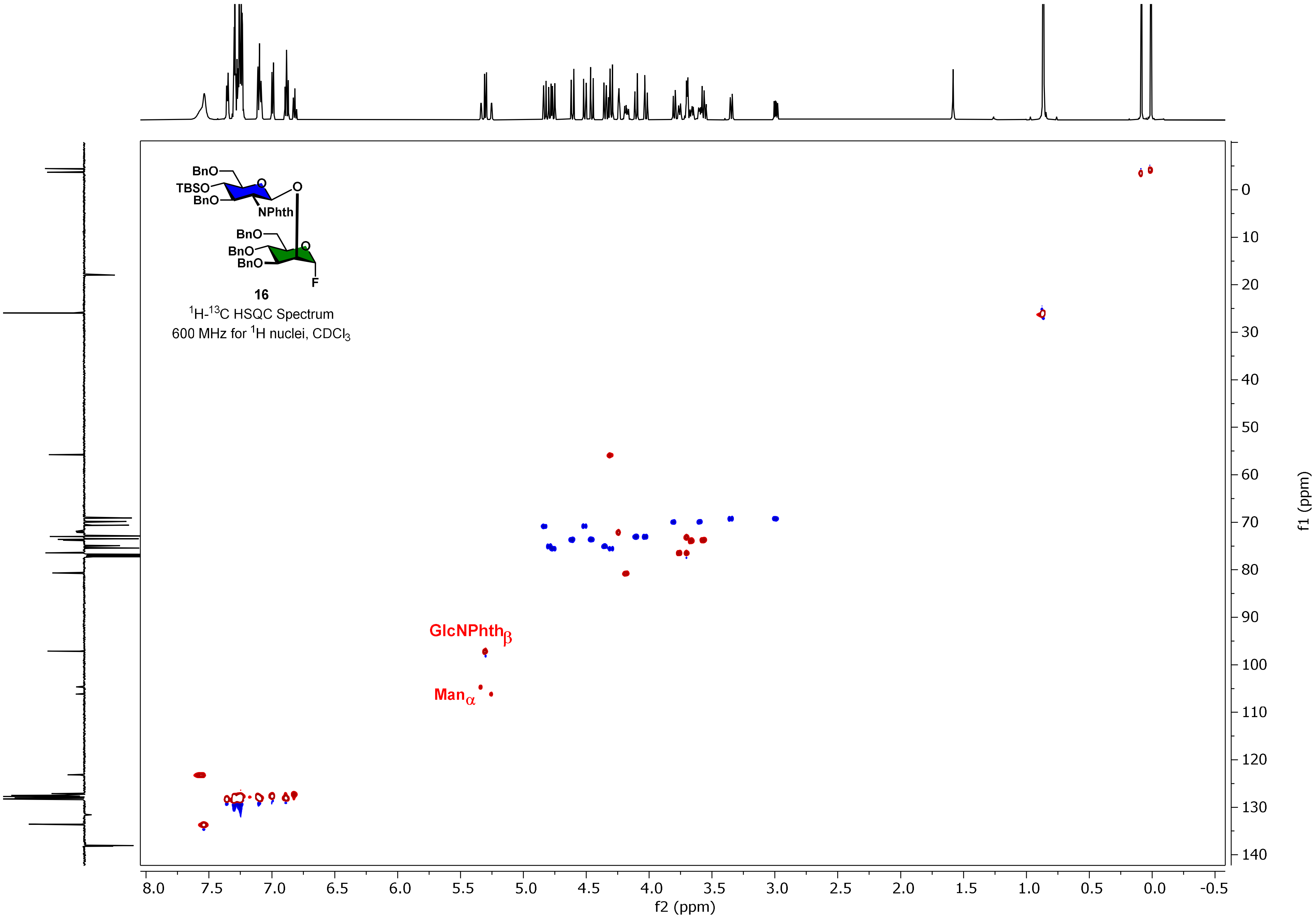
BnO
TBSO
BnO
NPhth
O
BnO
BnO
BnO
F
16
1H-13C HSQC Spectrum
600 MHz for 1H nuclei, CDCl3
GlcNPhthβ
Manα
f2 (ppm)
f1 (ppm)

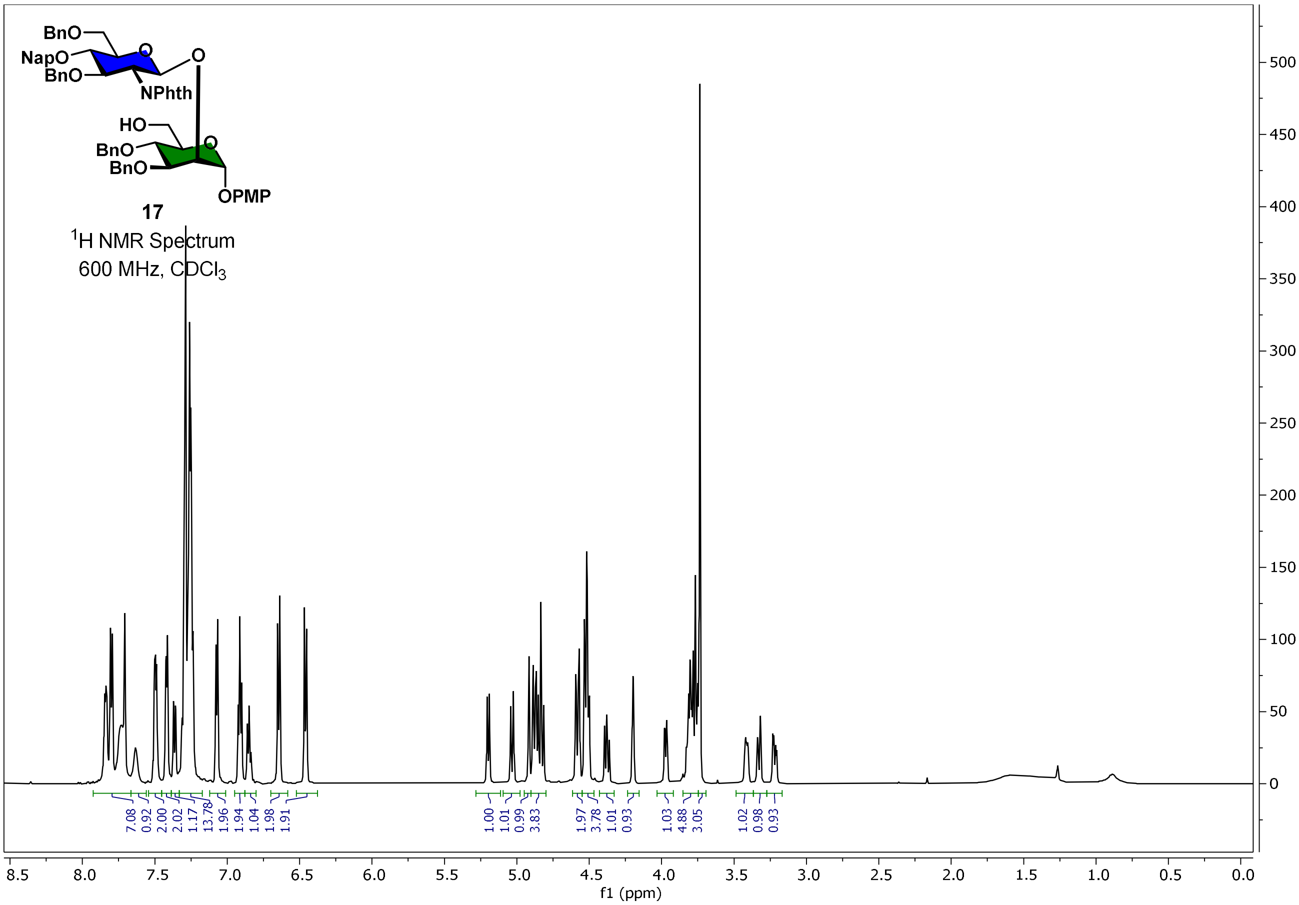
BnO
NapO
BnO
NPhth
HO
BnO
BnO
OPMP
17
^{1}H NMR Spectrum
600 MHz, CDCl$_3$
f1 (ppm)

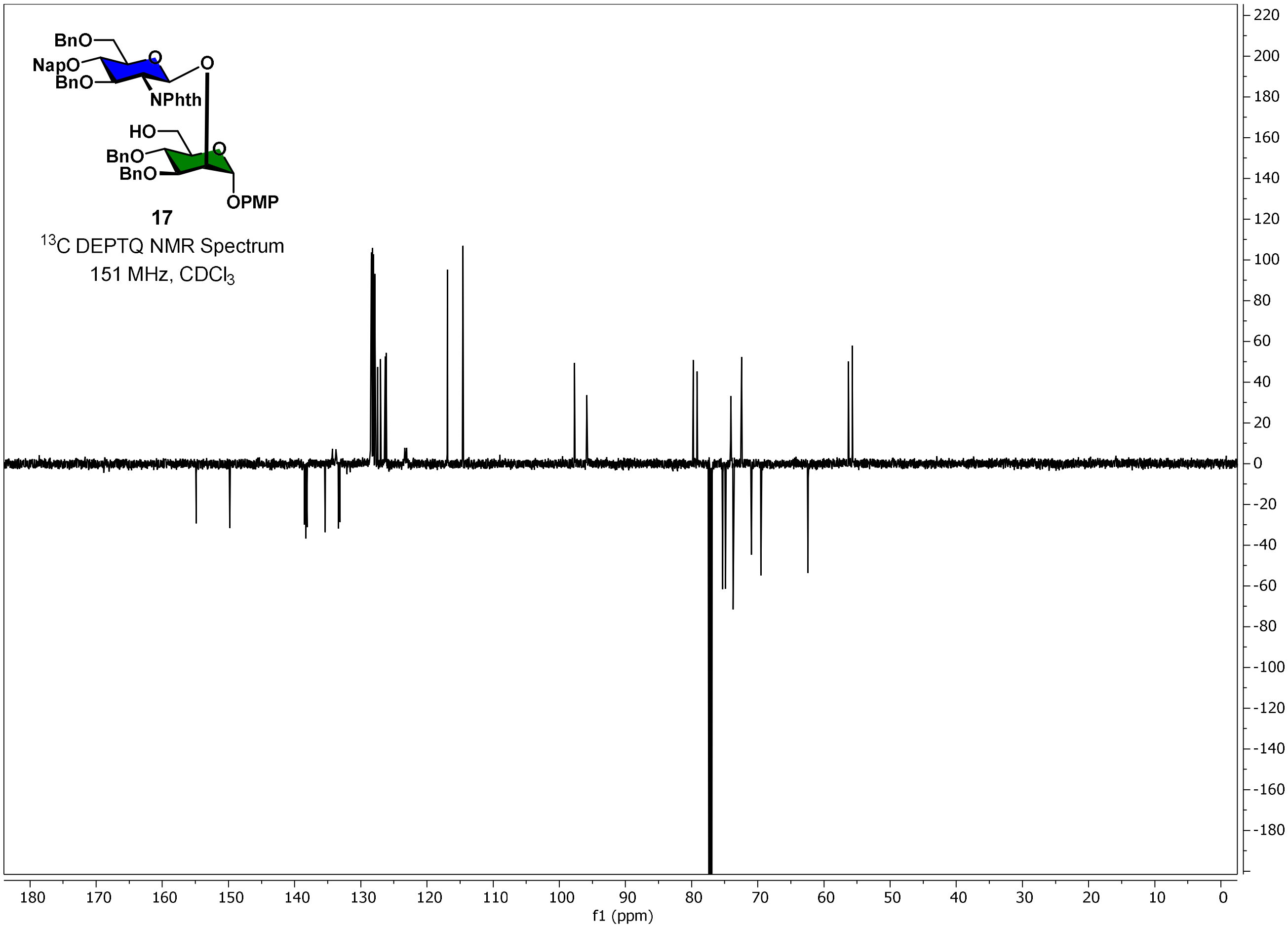
BnO
NapO
BnO
NPhth
HO
BnO
BnO
OPMP
17
^{13}C DEPTQ NMR Spectrum
151 MHz, CDCl$_3$
f1 (ppm)

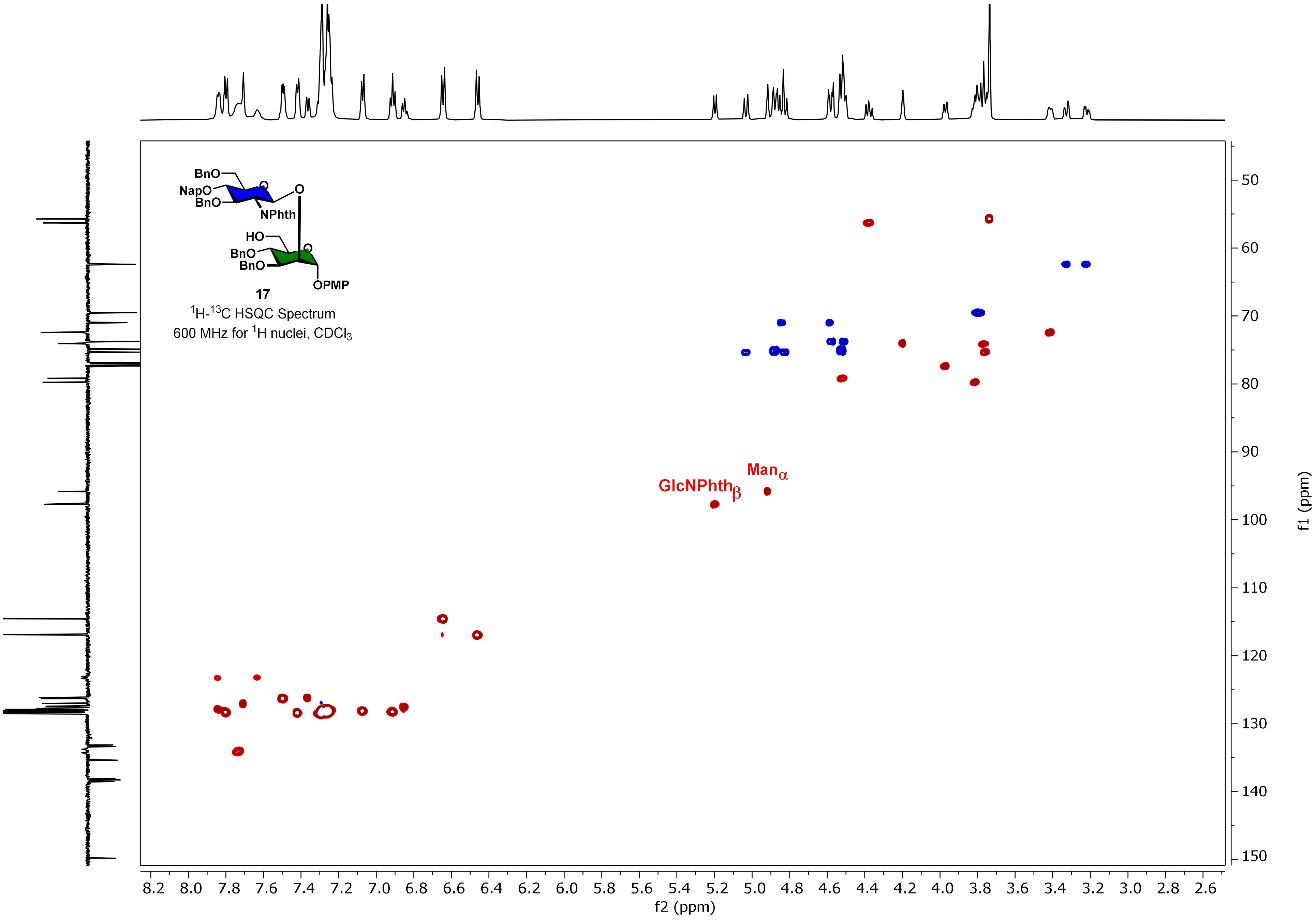
BnO
NapO
BnO
NPhth
O
HO
BnO
BnO
OPMP
17
^{1}H-^{13}C HSQC Spectrum
600 MHz for ^{1}H nuclei, $CDCl_3$
$GlcNPhth_{\beta}$
Man_{α}
f2 (ppm)
f1 (ppm)

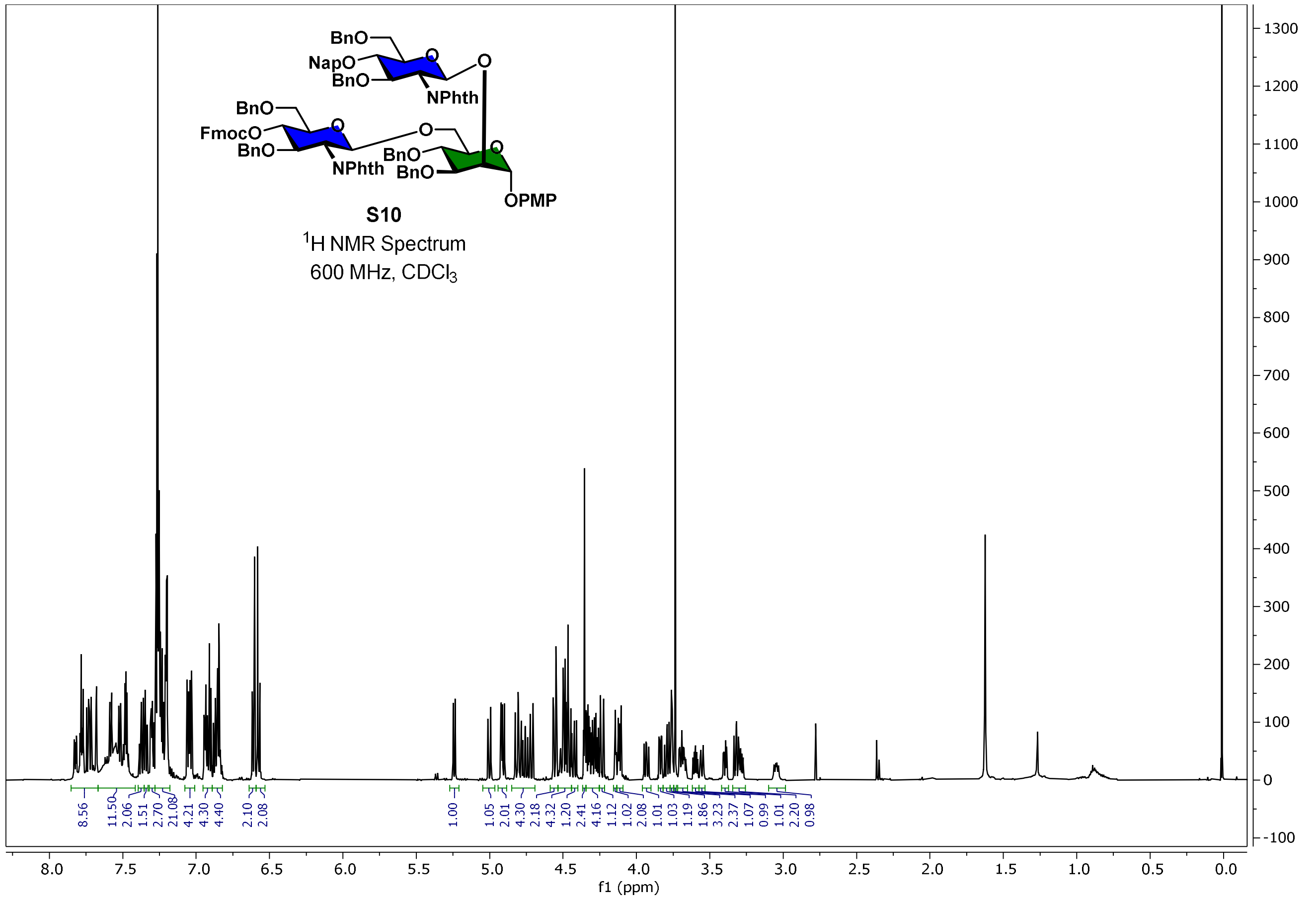
BnO
NapO
BnO
NPhth
BnO
FmocO
BnO
NPhth
BnO
BnO
OPMP
S10
1H NMR Spectrum
600 MHz, CDCl3
f1 (ppm)

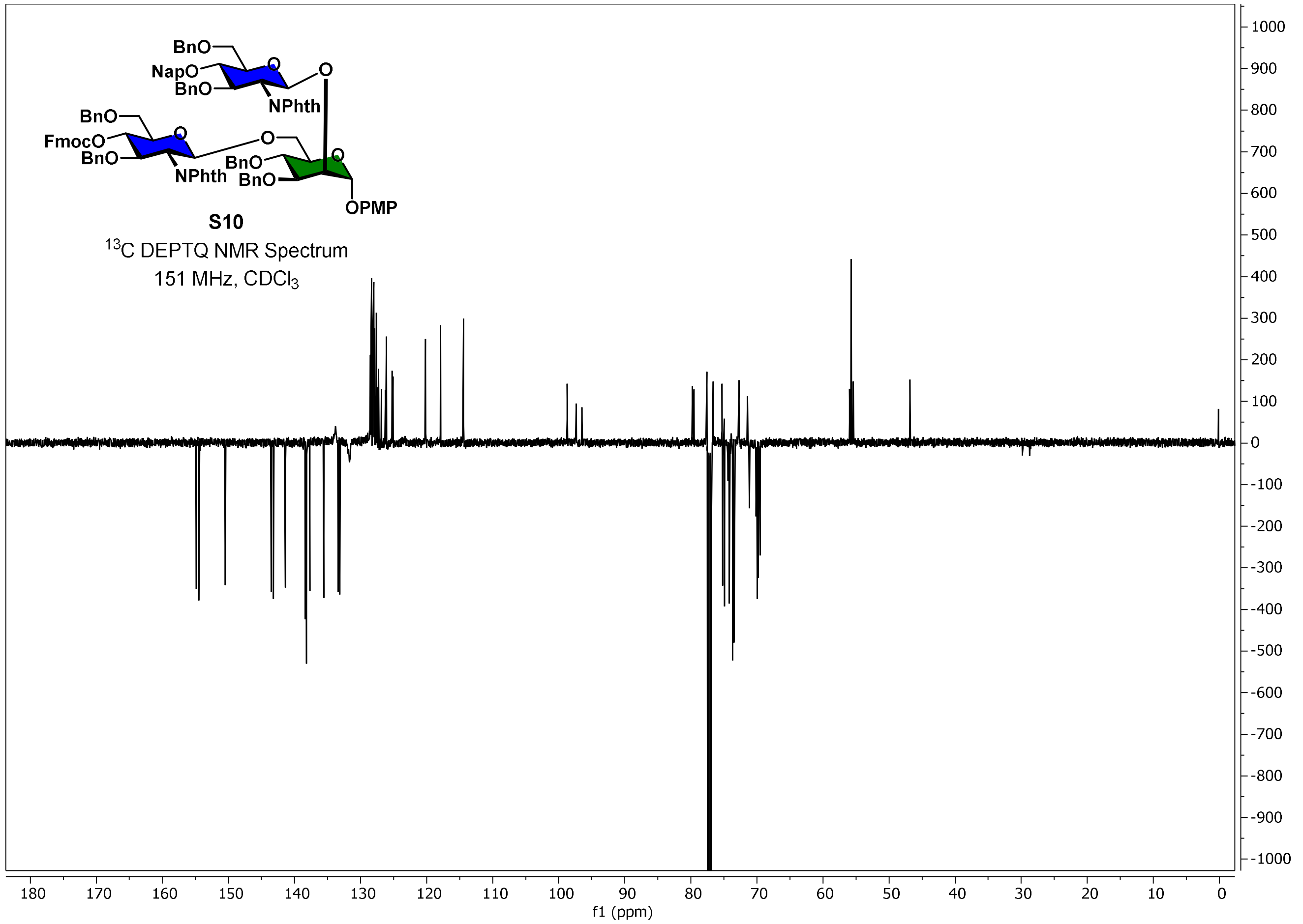
BnO
NapO
BnO
NPhth
BnO
FmocO
BnO
NPhth
BnO
BnO
OPMP
S10
13C DEPTQ NMR Spectrum
151 MHz, CDCl3
f1 (ppm)

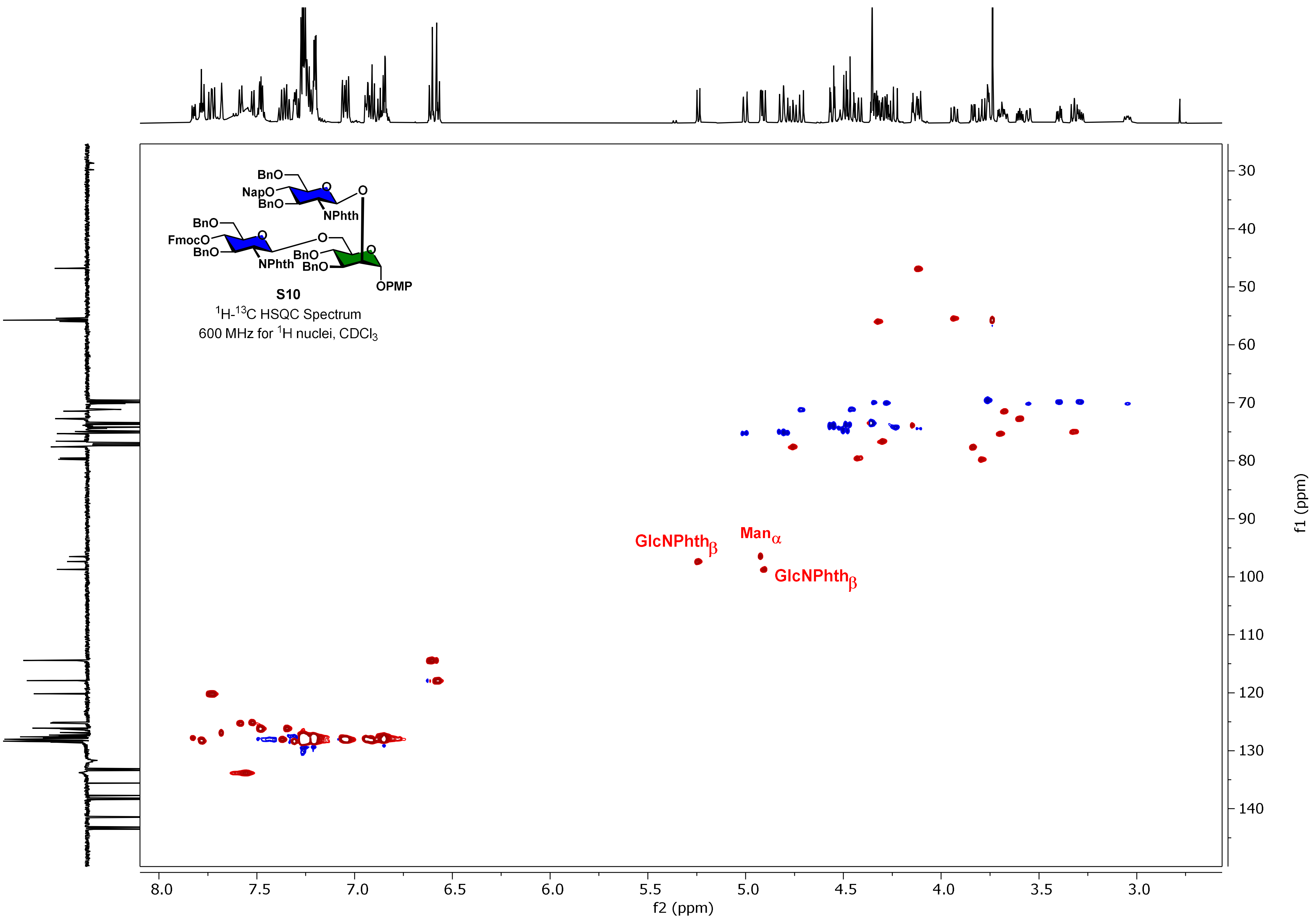

BnO
NapO
BnO
NPhth
O
BnO
FmocO
BnO
NPhth
O
BnO
BnO
OPMP
S10
^{1}H-^{13}C HSQC Spectrum
600 MHz for ^{1}H nuclei, $CDCl_3$
GlcNPhth$_\beta$
Man$_\alpha$
GlcNPhth$_\beta$
f2 (ppm)
f1 (ppm)

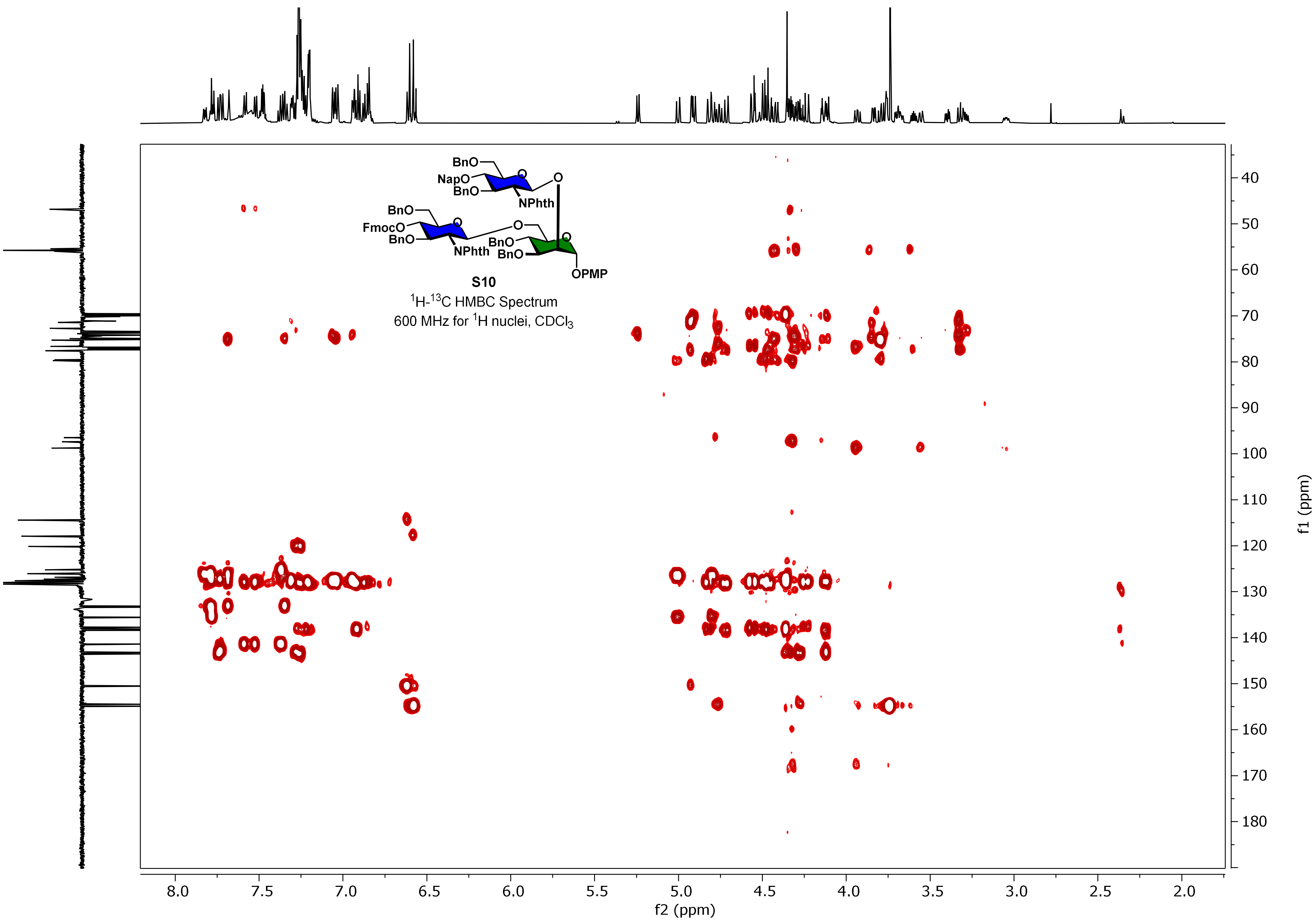
BnO
NapO
BnO
NPhth
O
BnO
FmocO
BnO
NPhth
BnO
BnO
OPMP
S10
¹H-¹³C HMBC Spectrum
600 MHz for ¹H nuclei, CDCl₃
f2 (ppm)
f1 (ppm)

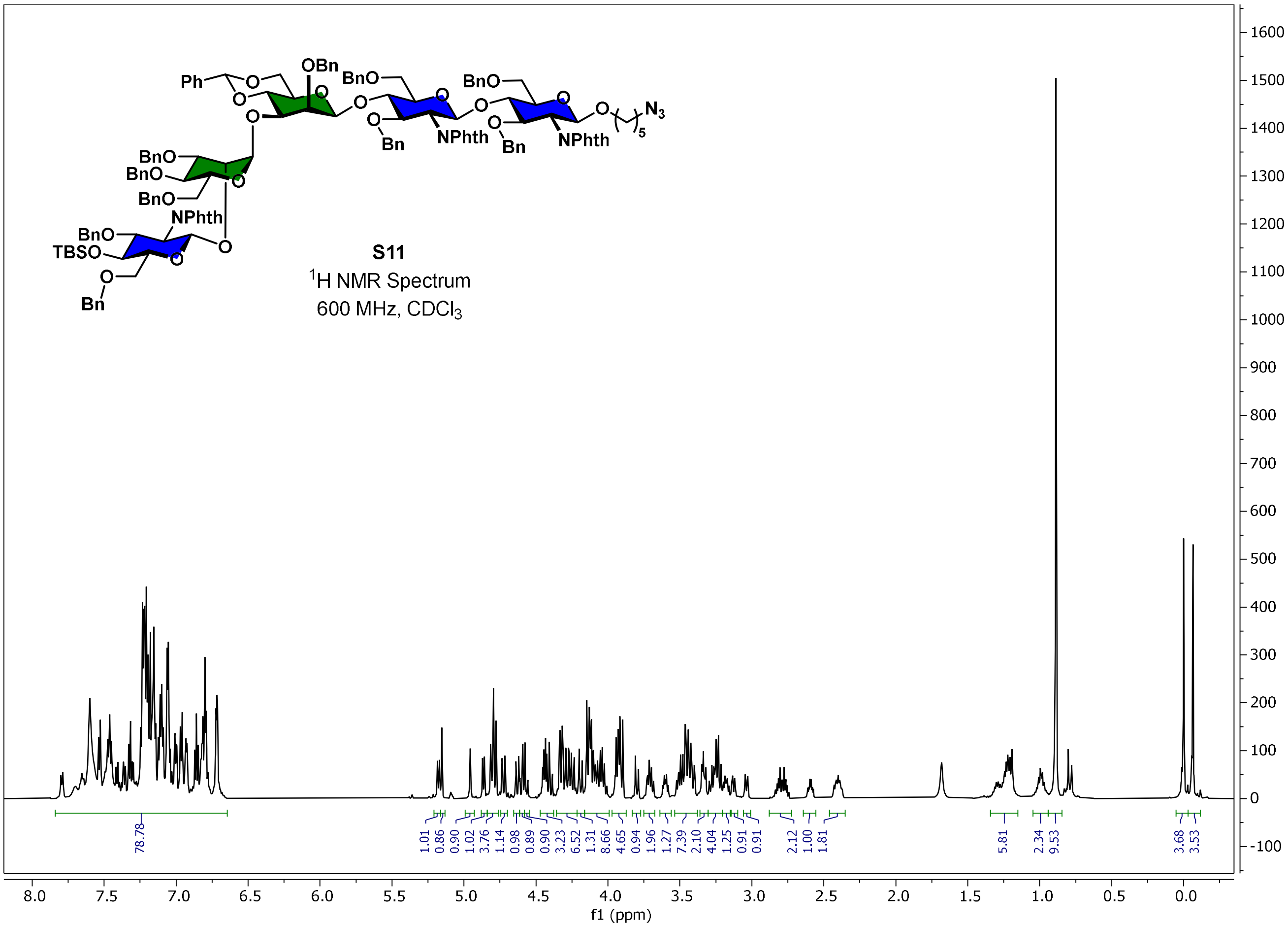
S11
1H NMR Spectrum
600 MHz, CDCl3
f1 (ppm)

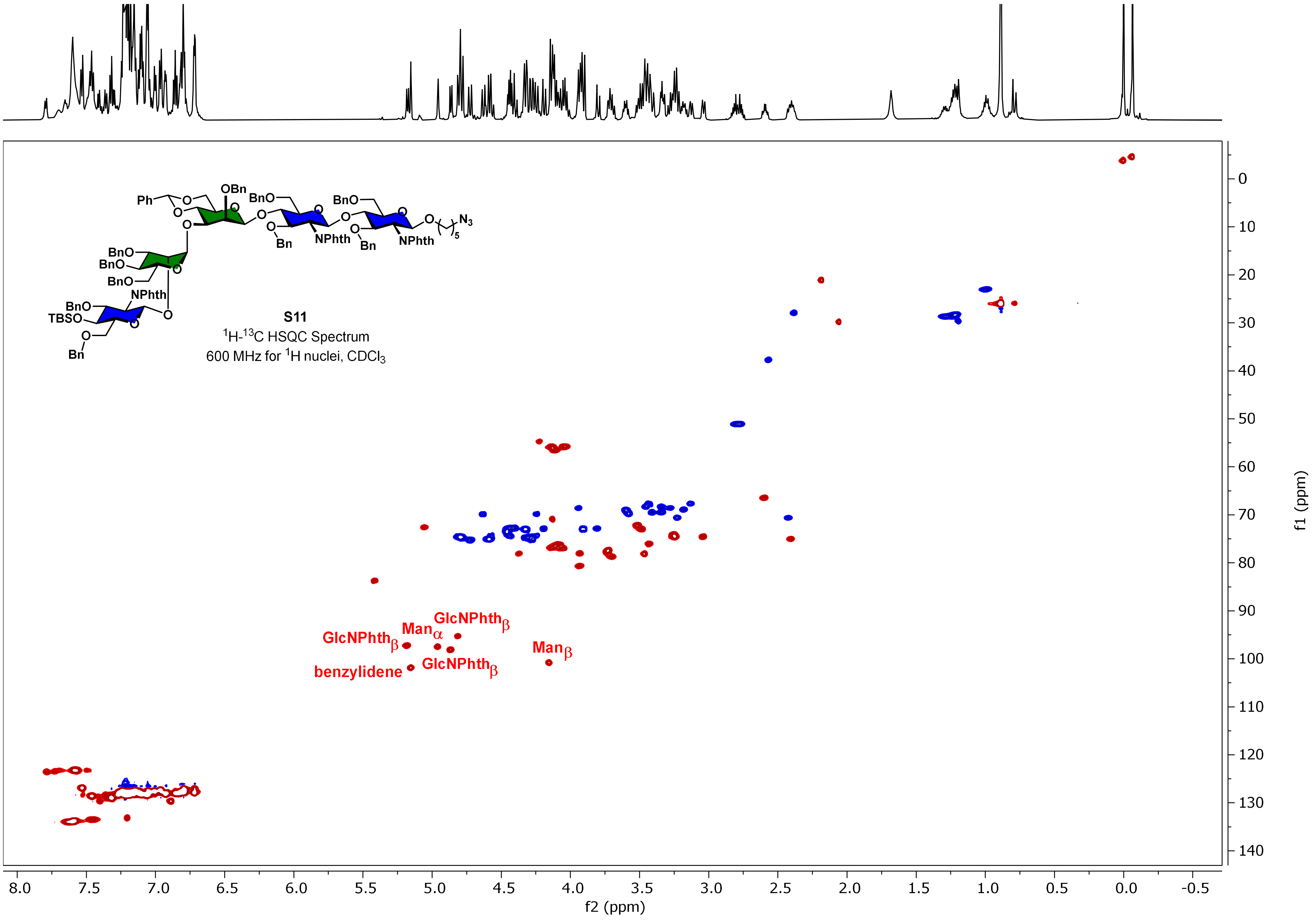

S11
1H-13C HSQC Spectrum
600 MHz for 1H nuclei, CDCl3
GlcNPhthβ
Manα
GlcNPhthβ
Manβ
benzylidene
GlcNPhthβ
f2 (ppm)
f1 (ppm)

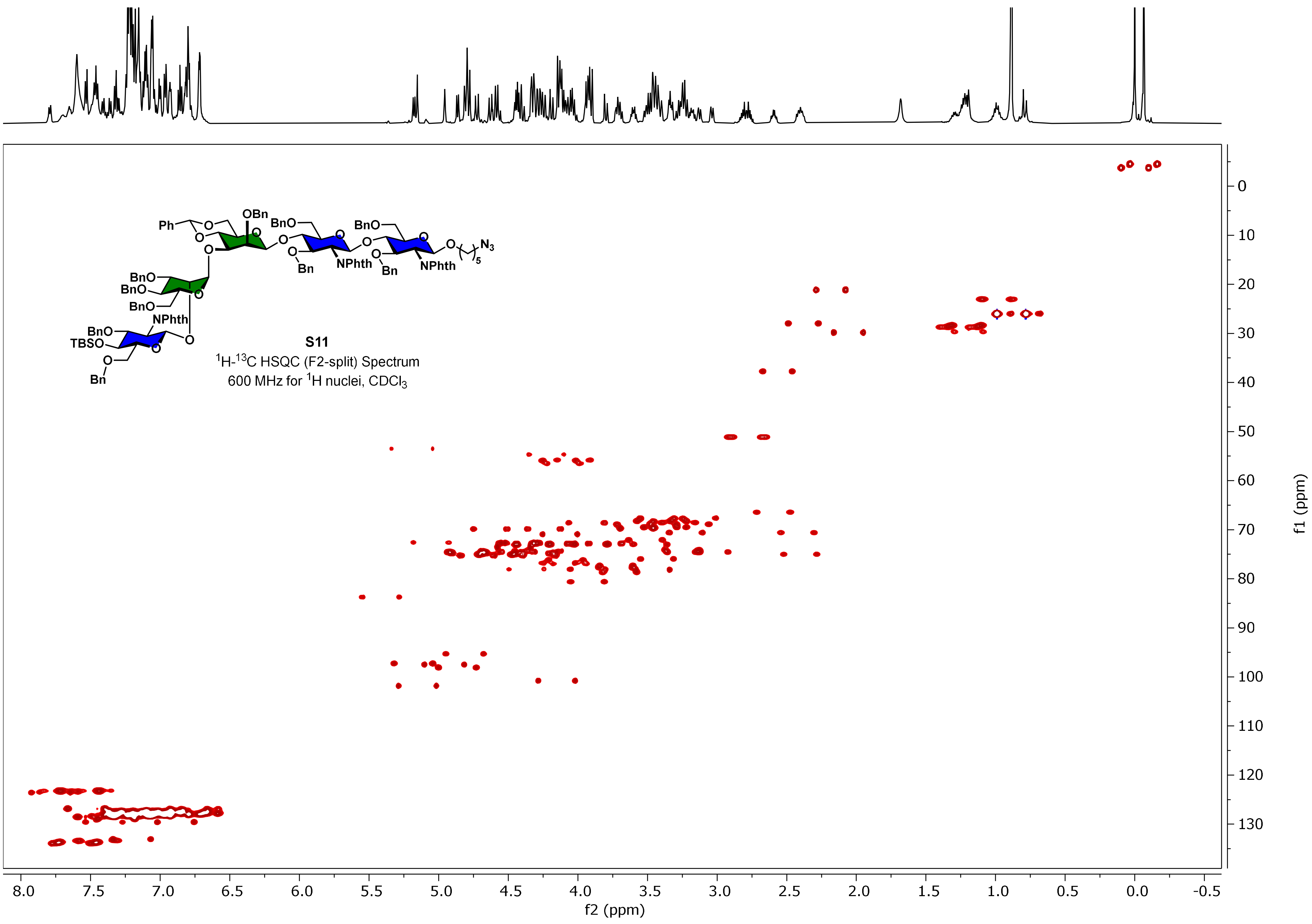

Ph
OBn
BnO
BnO
O
N3
5
NPhth
Bn
NPhth
Bn
BnO
BnO
BnO
NPhth
BnO
TBSO
Bn
S11
1H-13C HSQC (F2-split) Spectrum
600 MHz for 1H nuclei, CDCl3
f2 (ppm)
f1 (ppm)

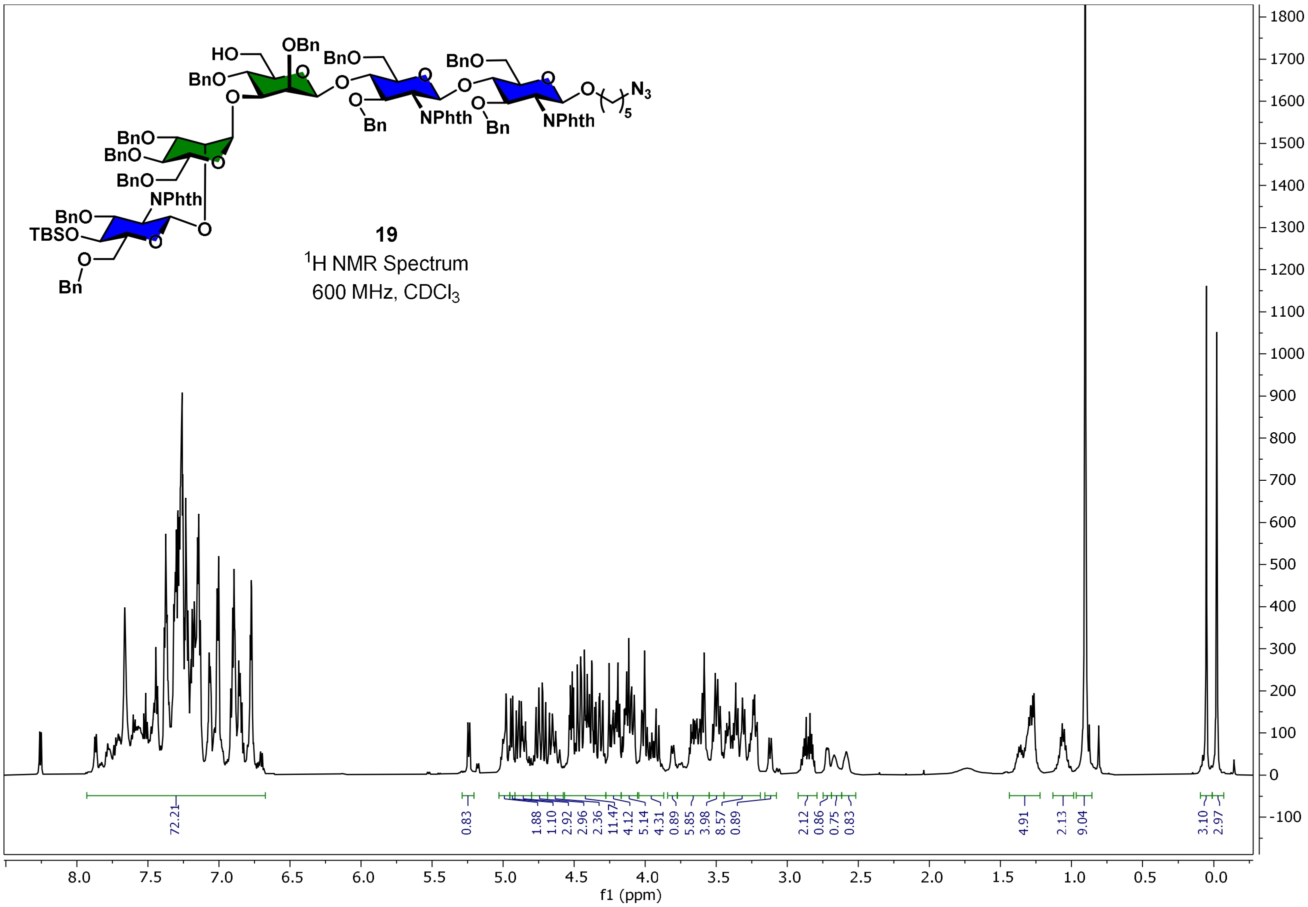
19
^{1}H NMR Spectrum
600 MHz, $CDCl_3$
f1 (ppm)

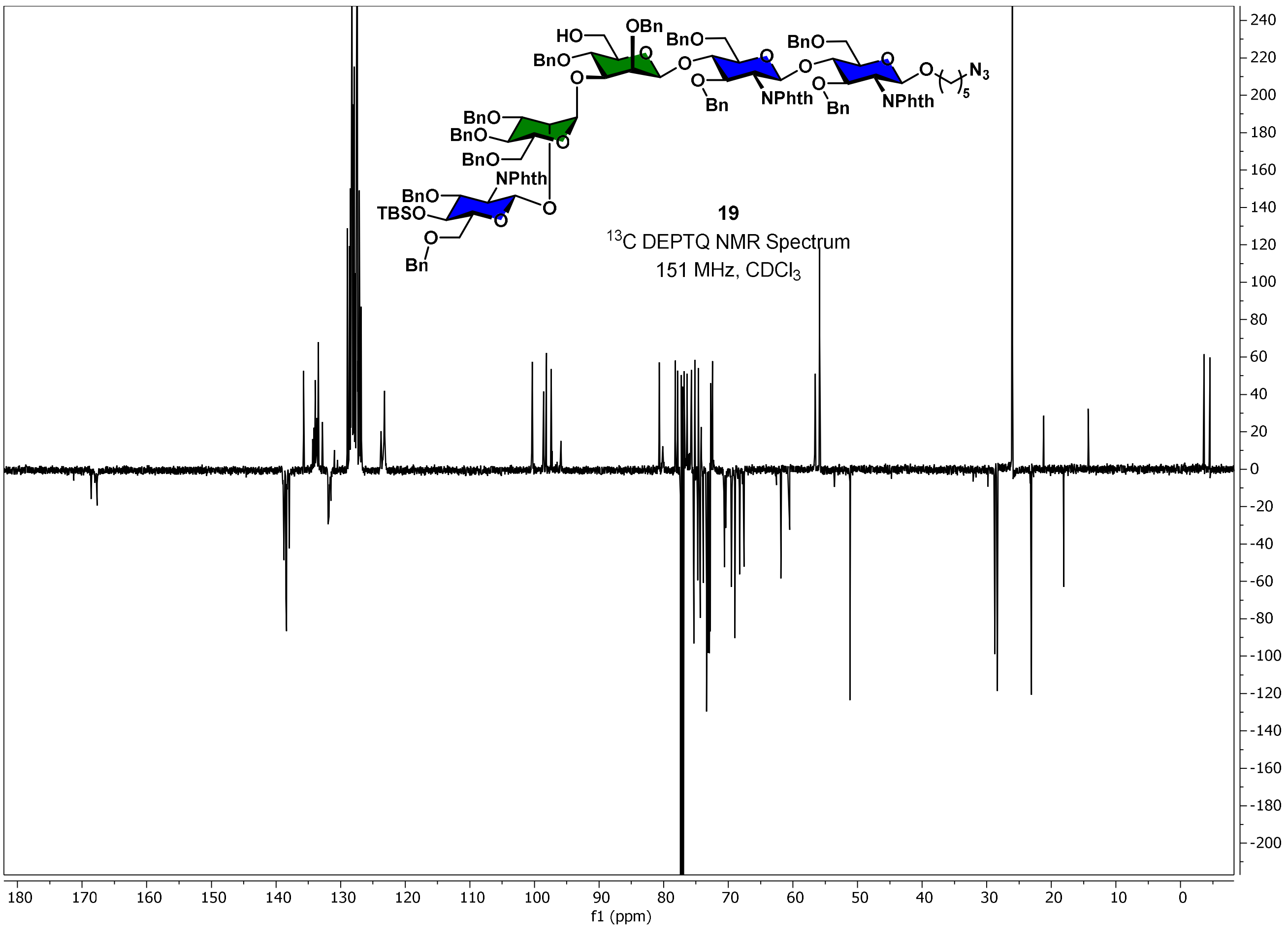
19
^{13}C DEPTQ NMR Spectrum
151 MHz, $CDCl_3$
f1 (ppm)

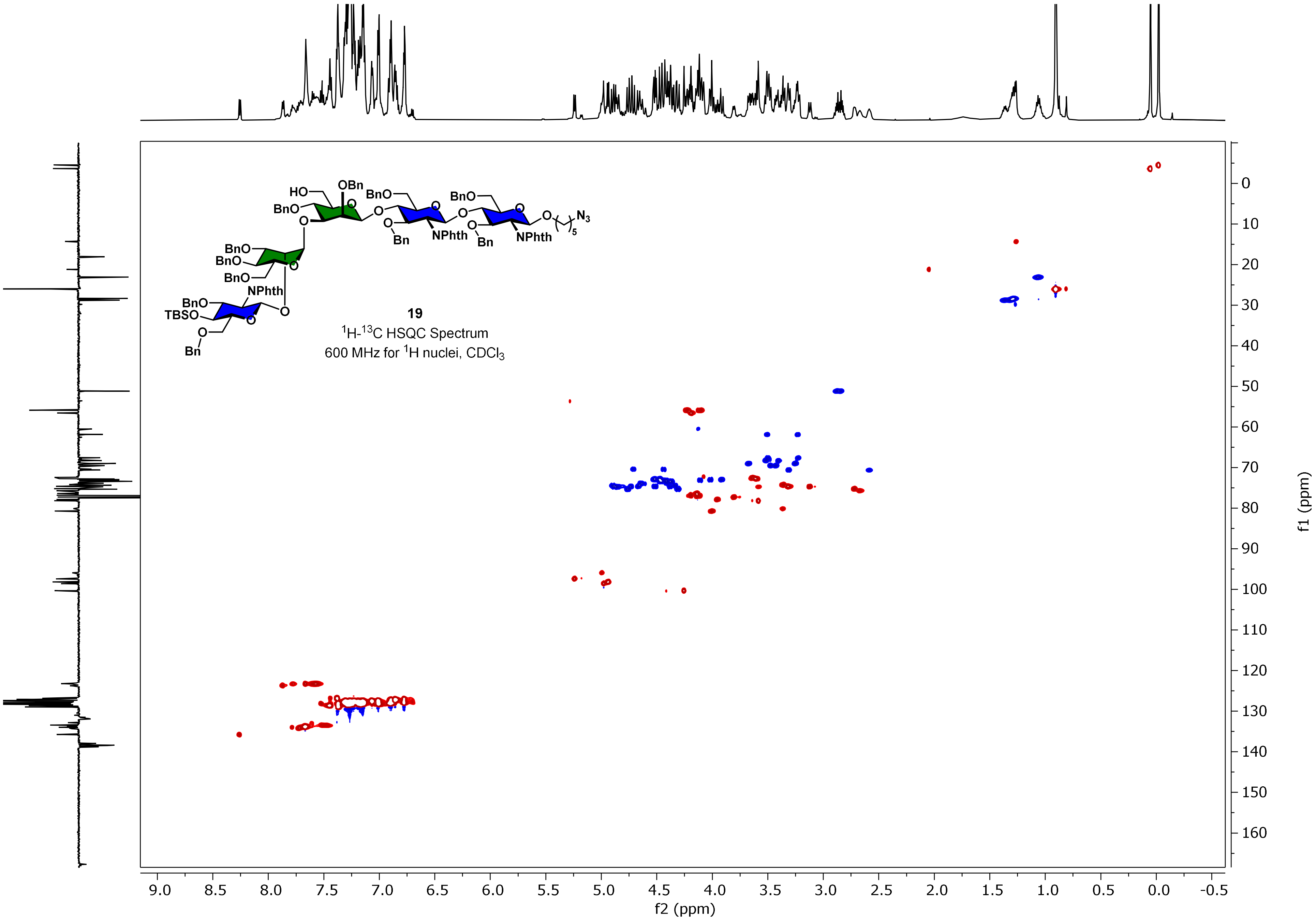
HO
OBn
BnO
BnO
BnO
BnO
BnO
BnO
BnO
NPhth
BnO
TBSO
O
Bn
Bn
NPhth
Bn
NPhth
N3
5
19
1H-13C HSQC Spectrum
600 MHz for 1H nuclei, CDCl3
f2 (ppm)
f1 (ppm)

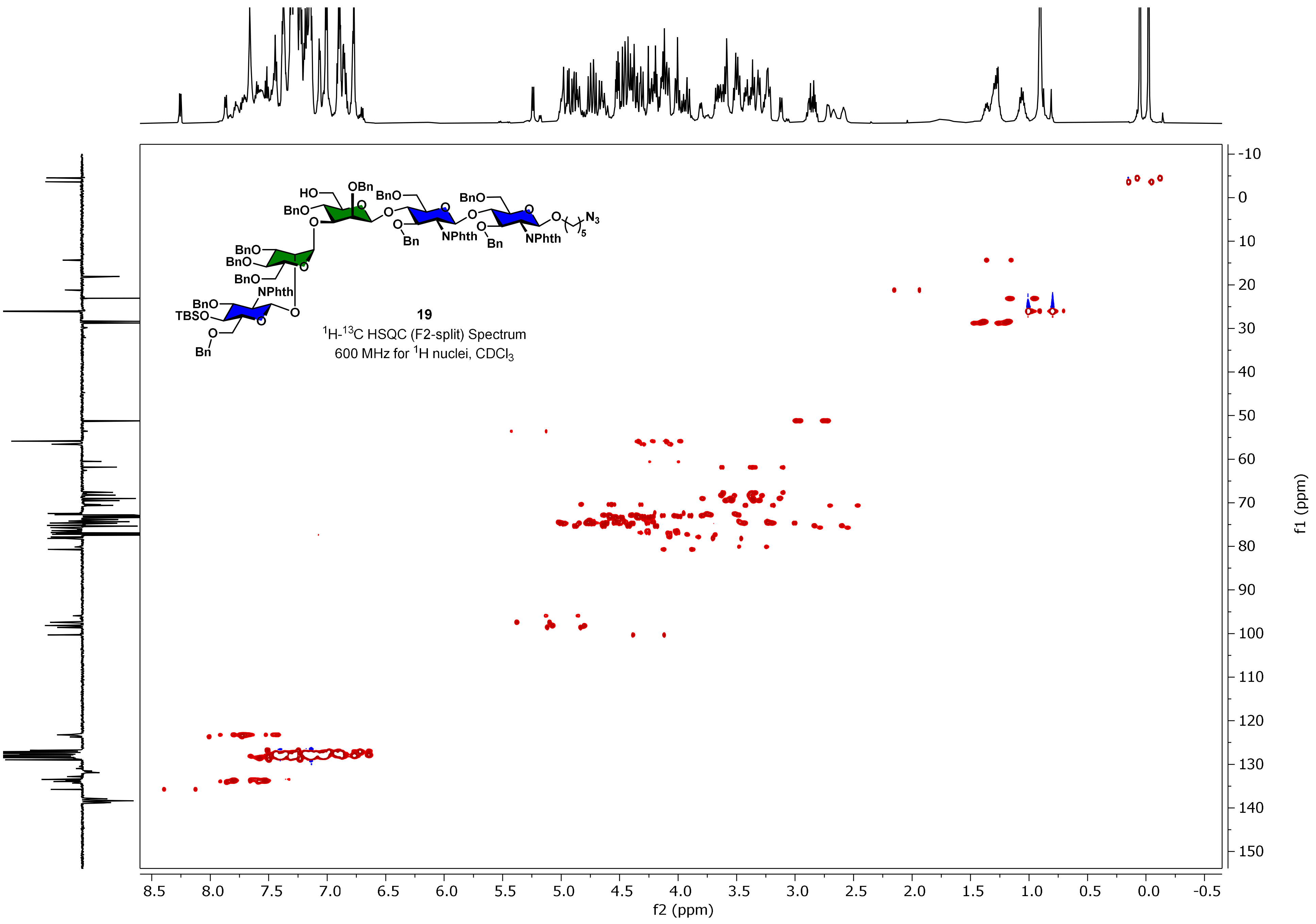
HO
OBn
BnO
BnO
BnO
O
N3
5
Bn
NPhth
Bn
NPhth
BnO
BnO
BnO
NPhth
BnO
TBSO
Bn
19
^{1}H-^{13}C HSQC (F2-split) Spectrum
600 MHz for ^{1}H nuclei, CDCl$_3$
f2 (ppm)
f1 (ppm)

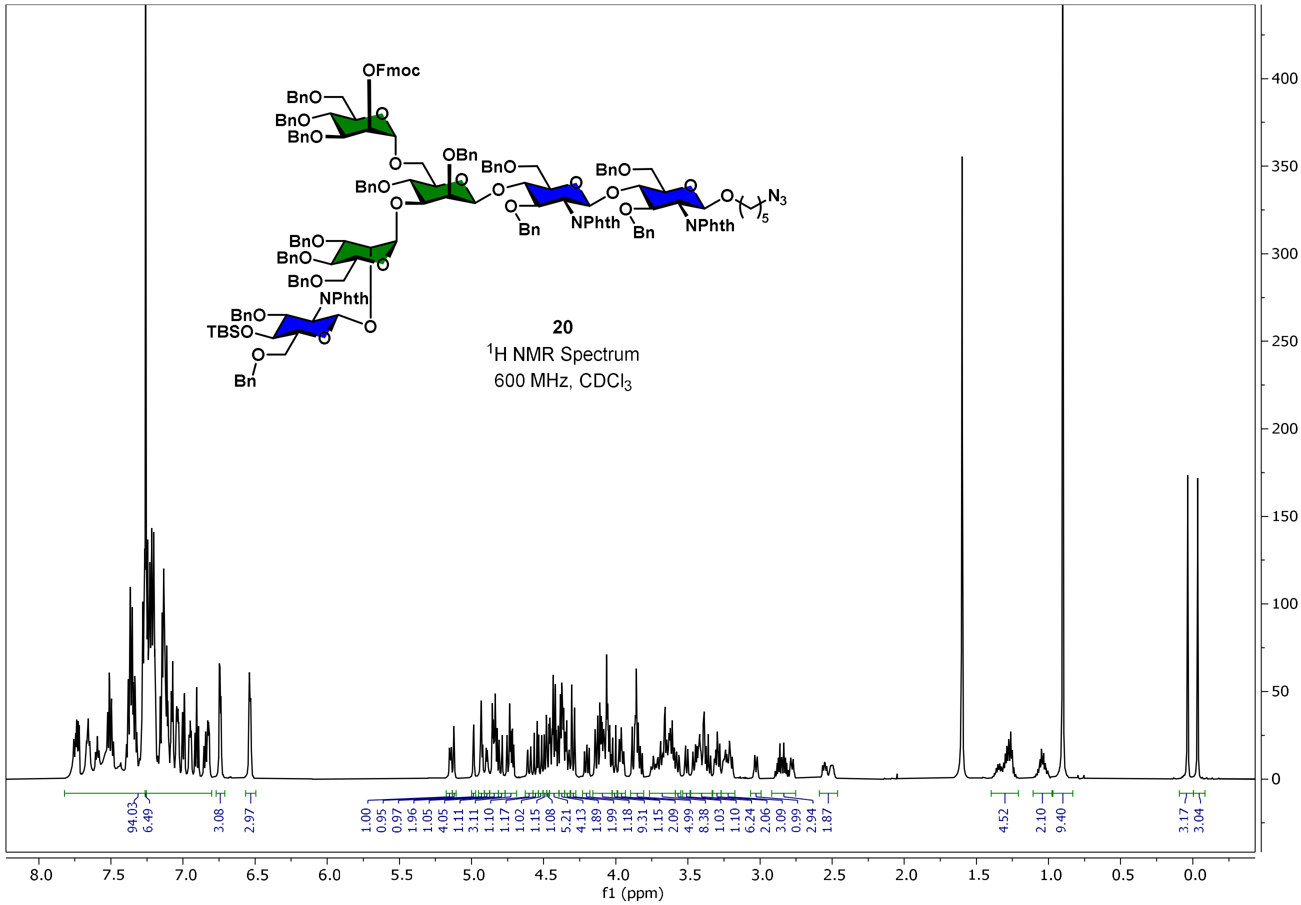
OFmoc
BnO
BnO
BnO
OBn
BnO
BnO
O
BnO
BnO
BnO
NPhth
NPhth
N3
Bn
Bn
BnO
TBSO
NPhth
Bn
20
1H NMR Spectrum
600 MHz, CDCl3
f1 (ppm)

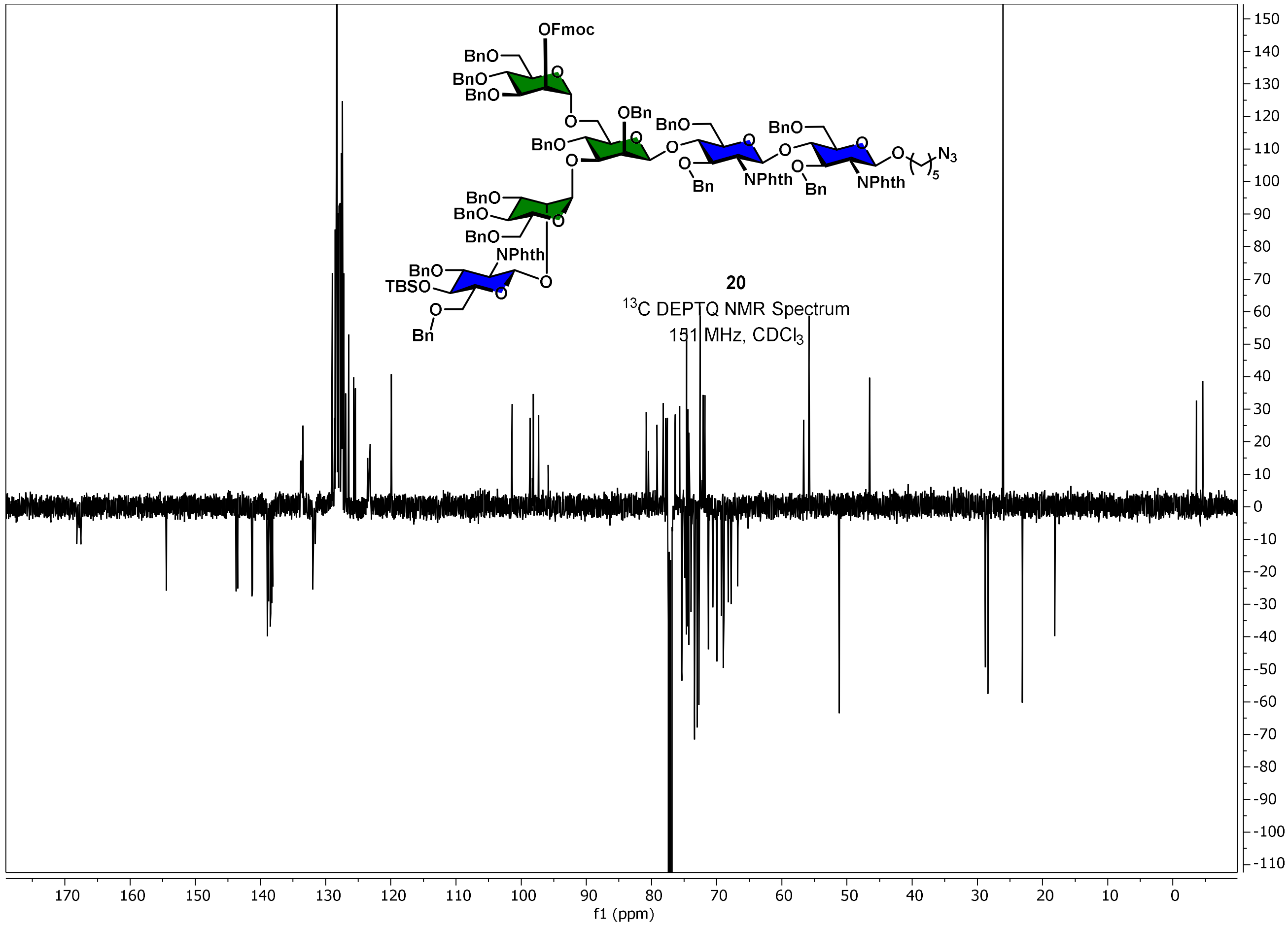
OFmoc
BnO
BnO
BnO
OBn
BnO
BnO
BnO
BnO
BnO
NPhth
NPhth
N3
Bn
Bn
BnO
TBSO
NPhth
Bn
20
13C DEPTQ NMR Spectrum
151 MHz, CDCl3
f1 (ppm)

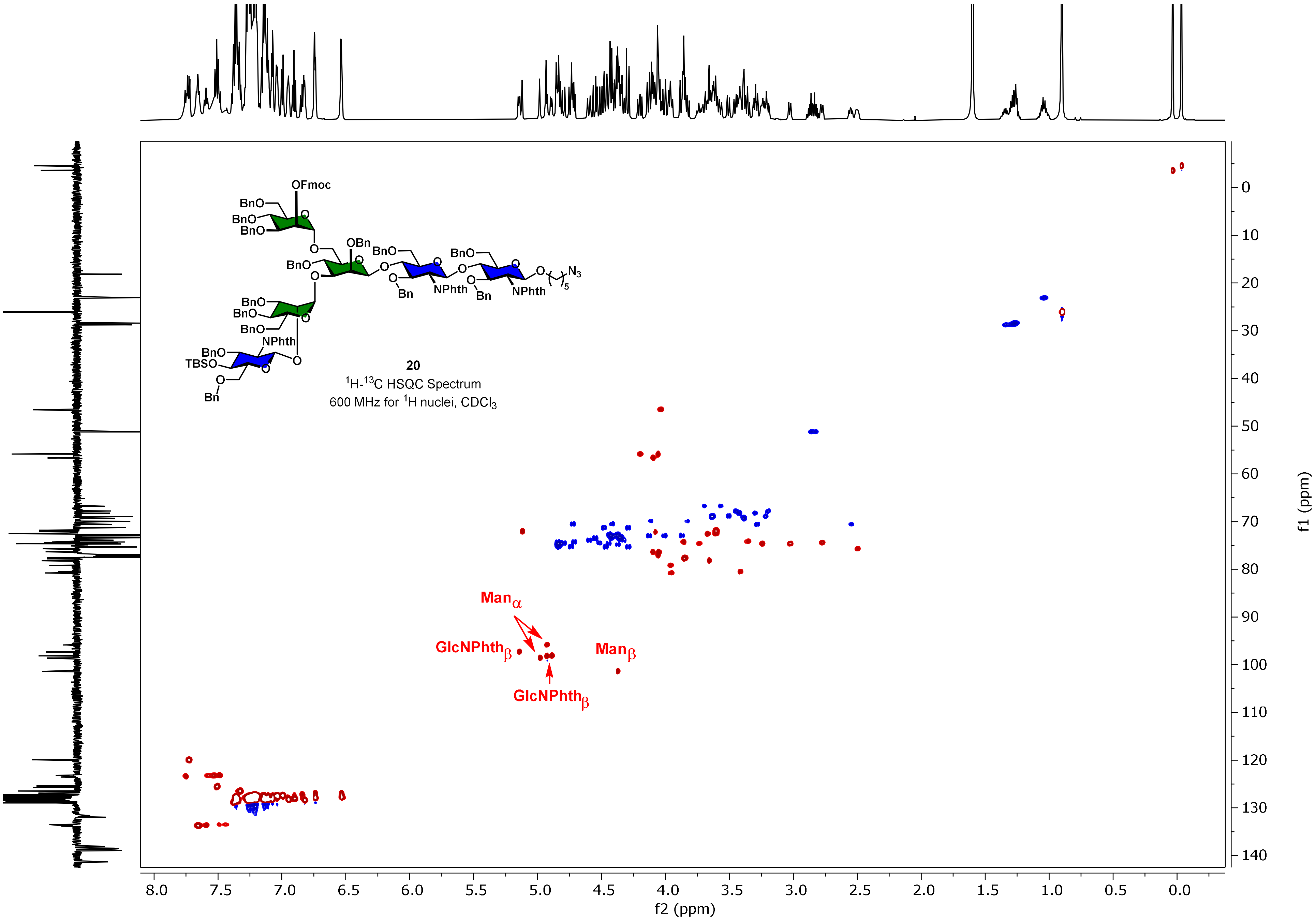

OFmoc
BnO
BnO
BnO
OBn
BnO
BnO
BnO
NPhth
Bn
Bn
NPhth
N_3
BnO
BnO
BnO
NPhth
BnO
TBSO
Bn
20
^{1}H-^{13}C HSQC Spectrum
600 MHz for ^{1}H nuclei, $CDCl_3$
Man$_\alpha$
GlcNPhth$_\beta$
GlcNPhth$_\beta$
Man$_\beta$
f1 (ppm)
f2 (ppm)

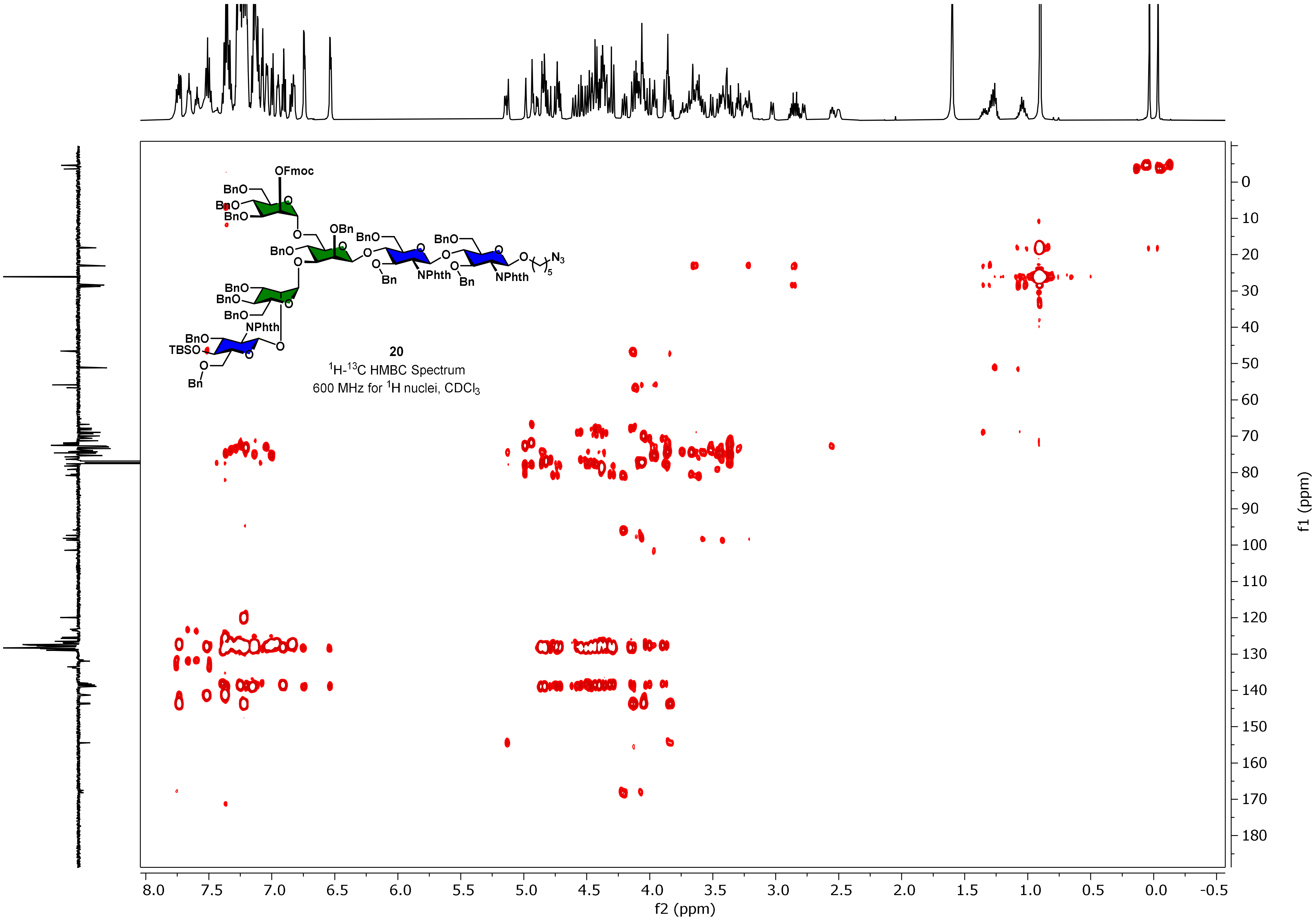
OFmoc
BnO
BnO
BnO
OBn
BnO
BnO
NPhth
Bn
BnO
NPhth
Bn
BnO
BnO
BnO
NPhth
BnO
TBSO
Bn
20
^1H-^{13}C HMBC Spectrum
600 MHz for ^1H nuclei, $CDCl_3$
f2 (ppm)
f1 (ppm)

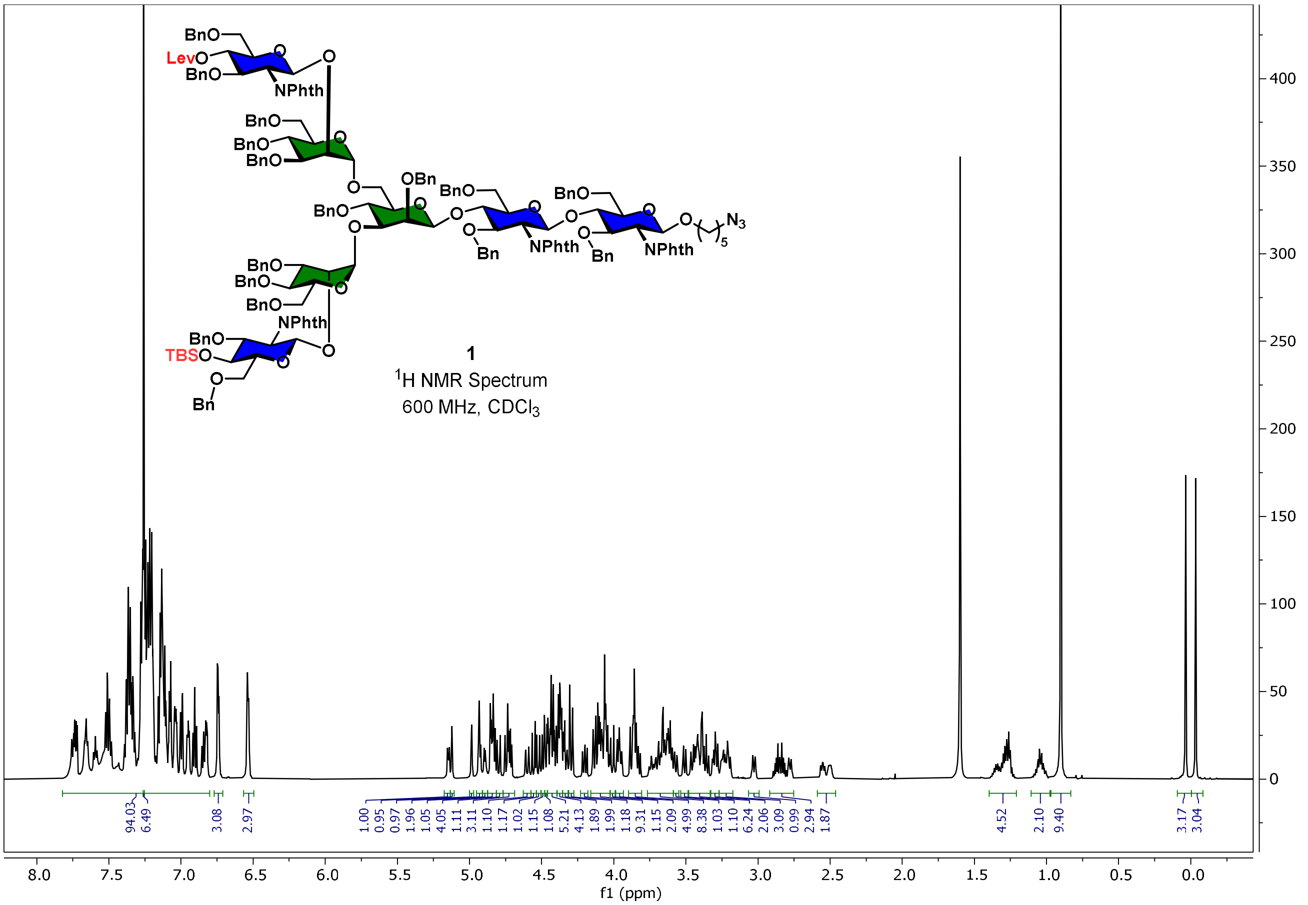
1
1H NMR Spectrum
600 MHz, CDCl3
BnO
LevO
NPhth
OBn
TBSO
Bn
N3
f1 (ppm)

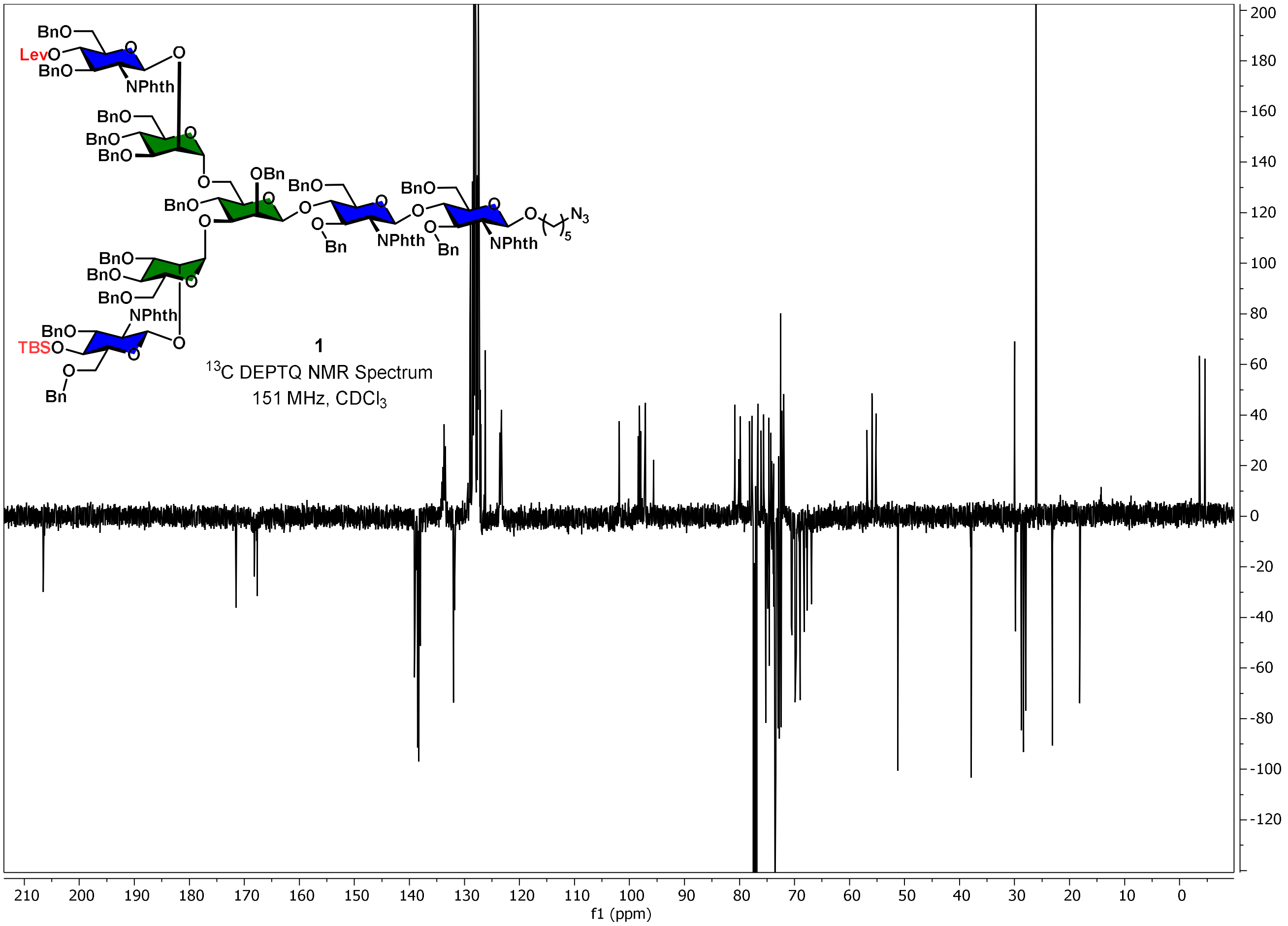
1
13C DEPTQ NMR Spectrum
151 MHz, CDCl3
BnO
LevO
NPhth
OBn
TBSO
Bn
N3
f1 (ppm)

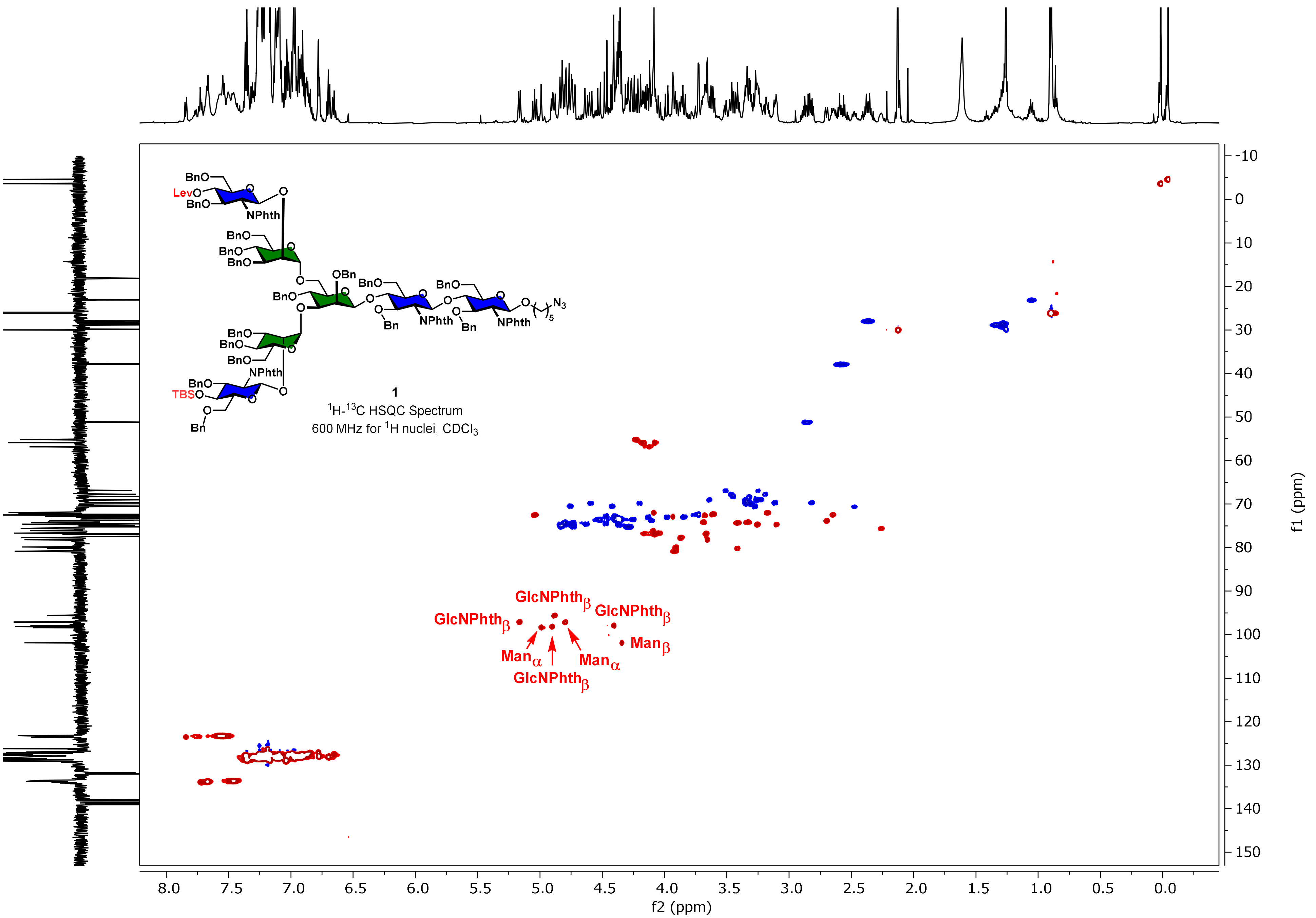

1
^{1}H-^{13}C HSQC Spectrum
600 MHz for ^{1}H nuclei, CDCl$_3$
GlcNPhth$_\beta$
GlcNPhth$_\beta$
GlcNPhth$_\beta$
Man$_\alpha$
Man$_\alpha$
Man$_\beta$
GlcNPhth$_\beta$
f2 (ppm)
f1 (ppm)

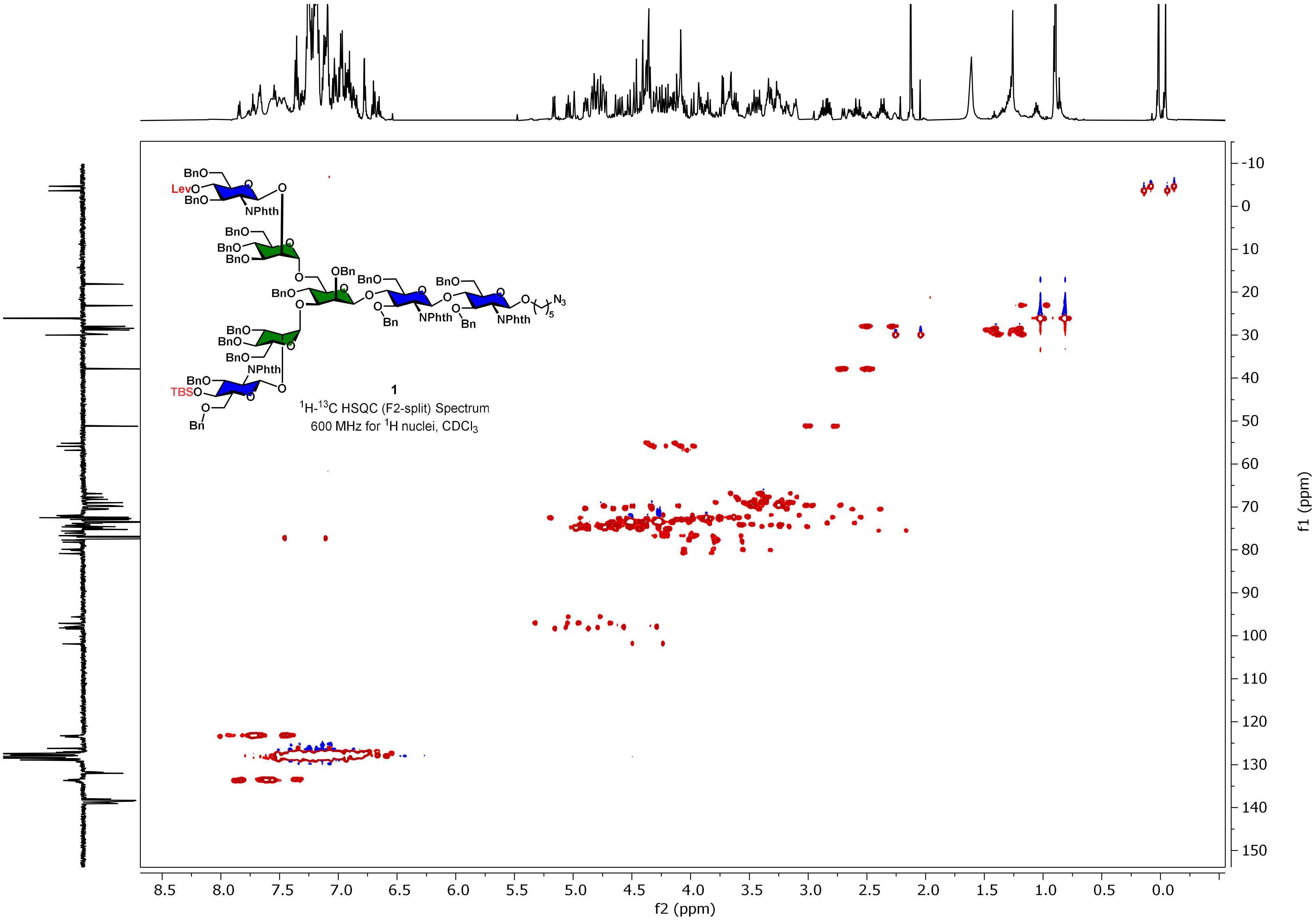
BnO
LevO
BnO
NPhth
O
BnO
BnO
BnO
OBn
BnO
BnO
BnO
O
N3
5
Bn
NPhth
Bn
NPhth
BnO
BnO
BnO
NPhth
BnO
TBSO
Bn
1
^{1}H-^{13}C HSQC (F2-split) Spectrum
600 MHz for ^{1}H nuclei, $CDCl_3$
f2 (ppm)
f1 (ppm)

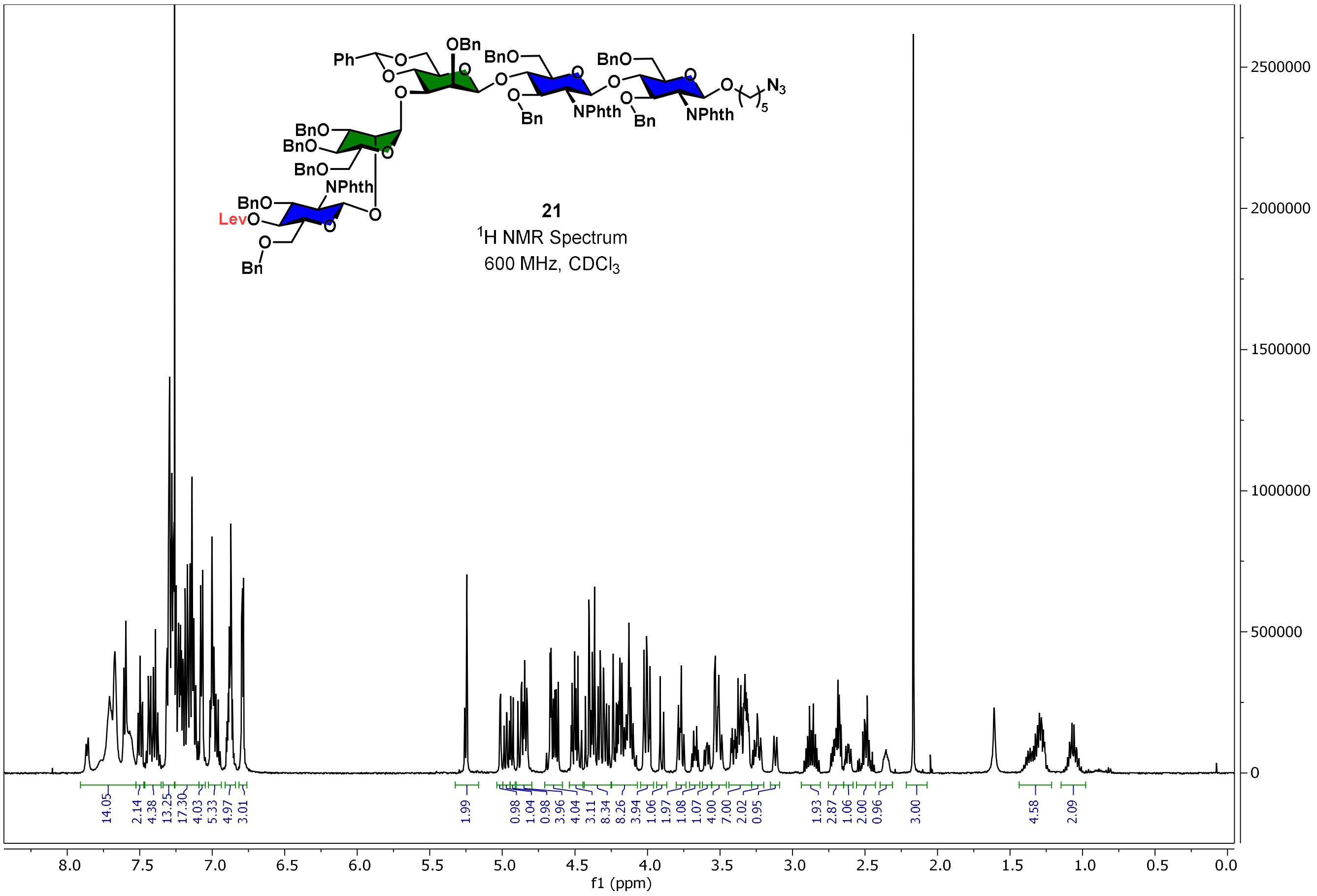
21
^{1}H NMR Spectrum
600 MHz, CDCl$_3$
f1 (ppm)

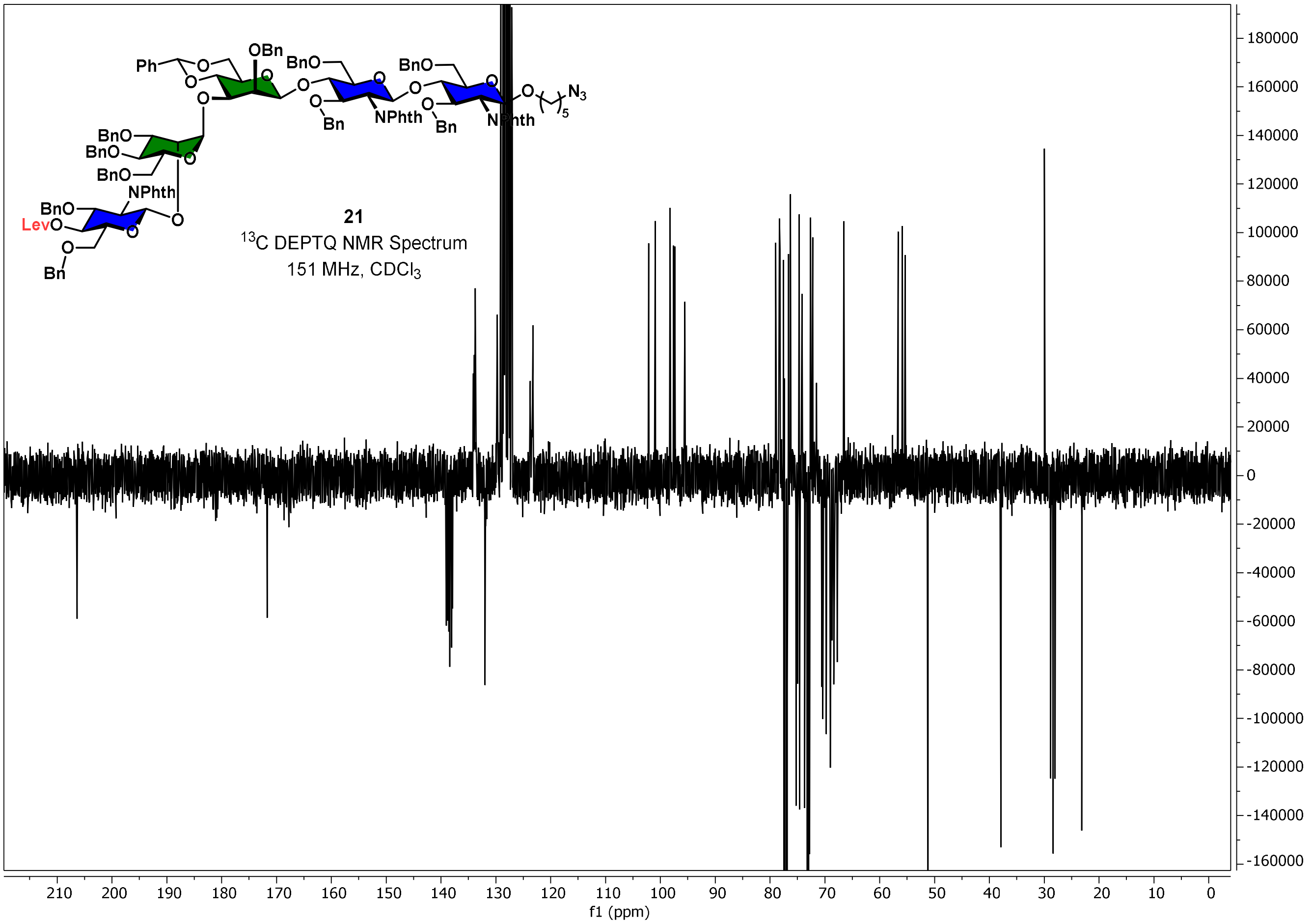
21
^{13}C DEPTQ NMR Spectrum
151 MHz, CDCl$_3$
f1 (ppm)

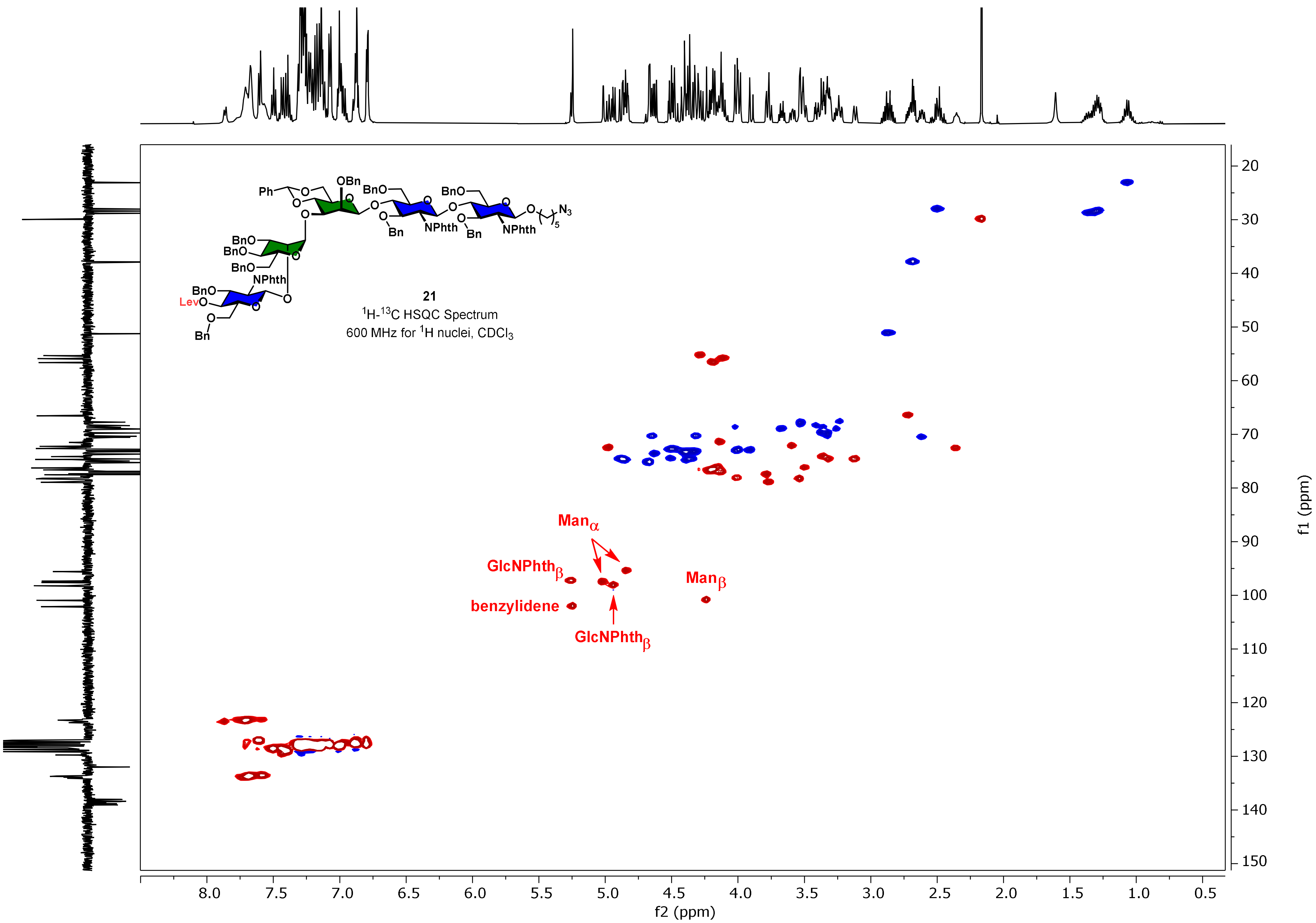
21
^{1}H-^{13}C HSQC Spectrum
600 MHz for ^{1}H nuclei, $CDCl_3$
Man$_\alpha$
GlcNPhth$_\beta$
Man$_\beta$
benzylidene
GlcNPhth$_\beta$
f2 (ppm)
f1 (ppm)

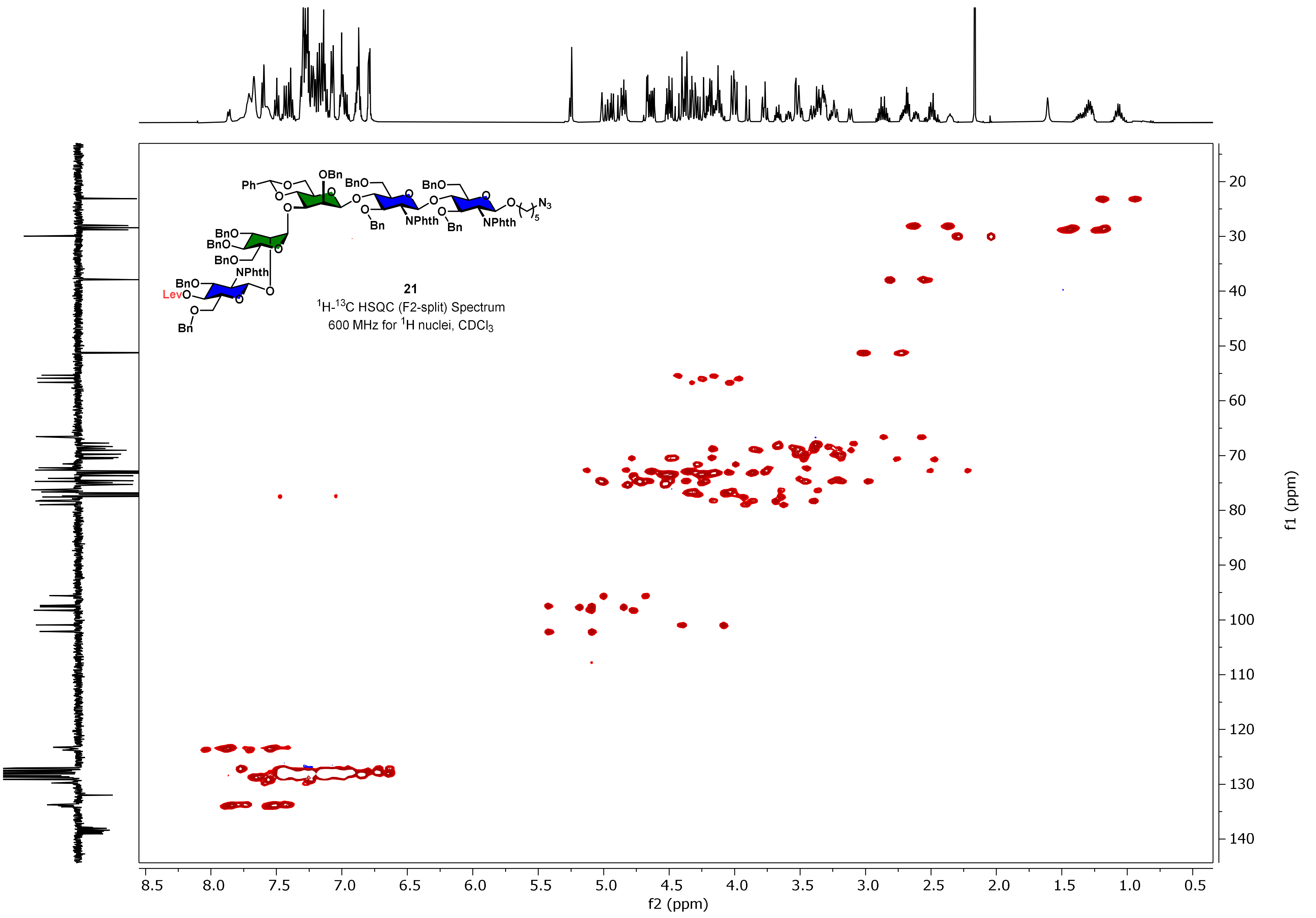

21
^{1}H-^{13}C HSQC (F2-split) Spectrum
600 MHz for ^{1}H nuclei, CDCl$_3$
f2 (ppm)
f1 (ppm)

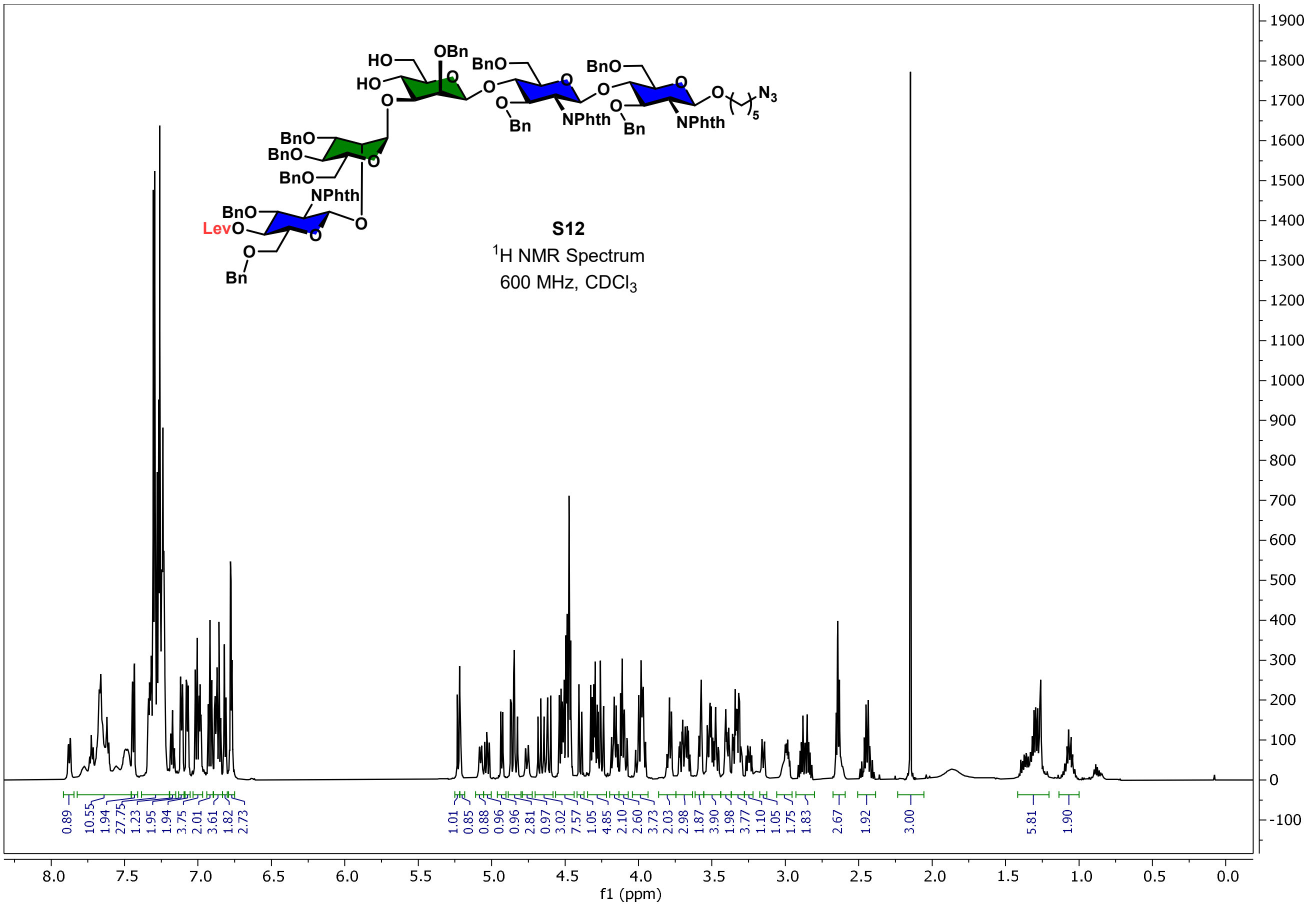
S12
^{1}H NMR Spectrum
600 MHz, $CDCl_3$
f1 (ppm)

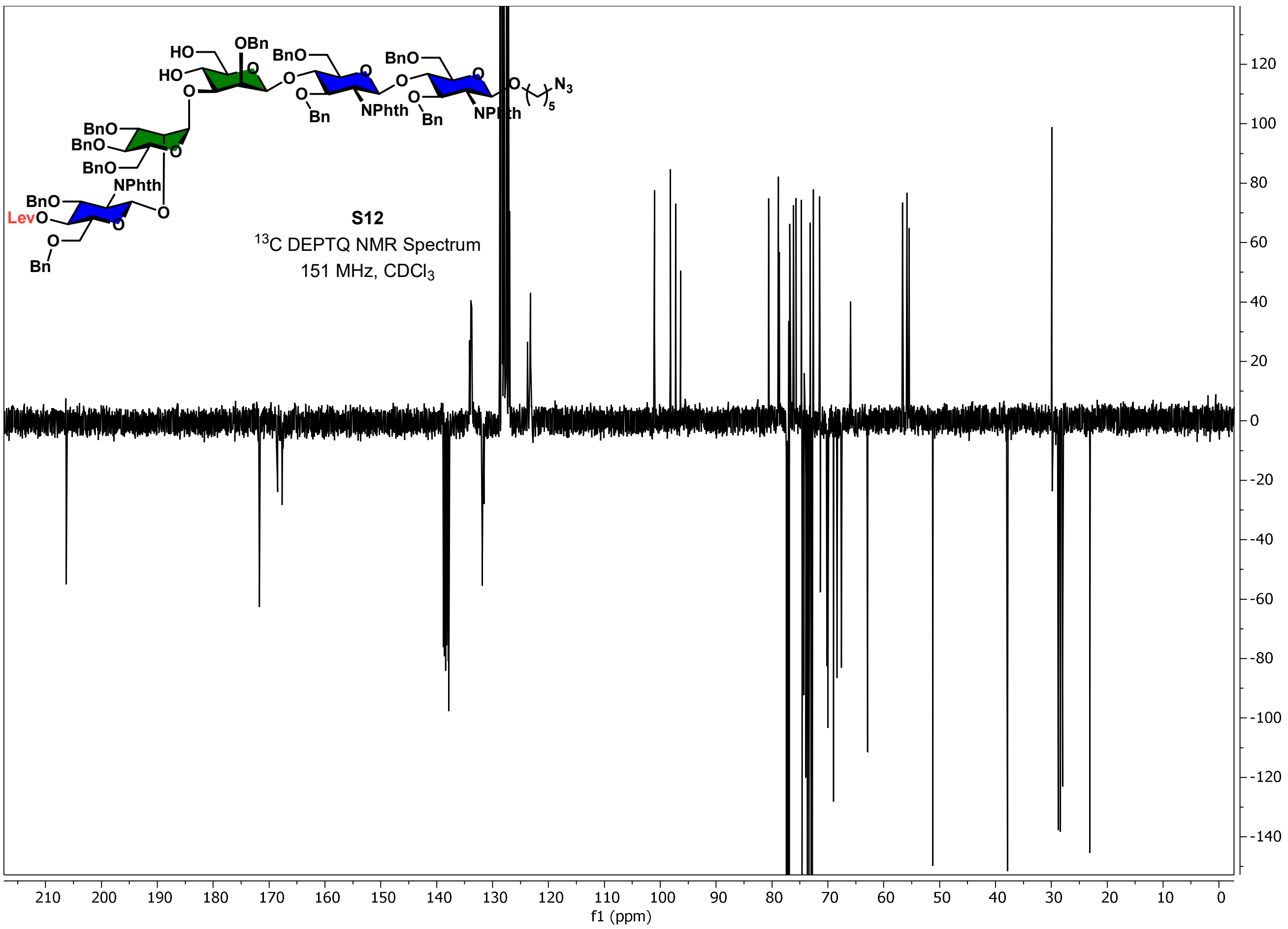
S12
^{13}C DEPTQ NMR Spectrum
151 MHz, $CDCl_3$
f1 (ppm)

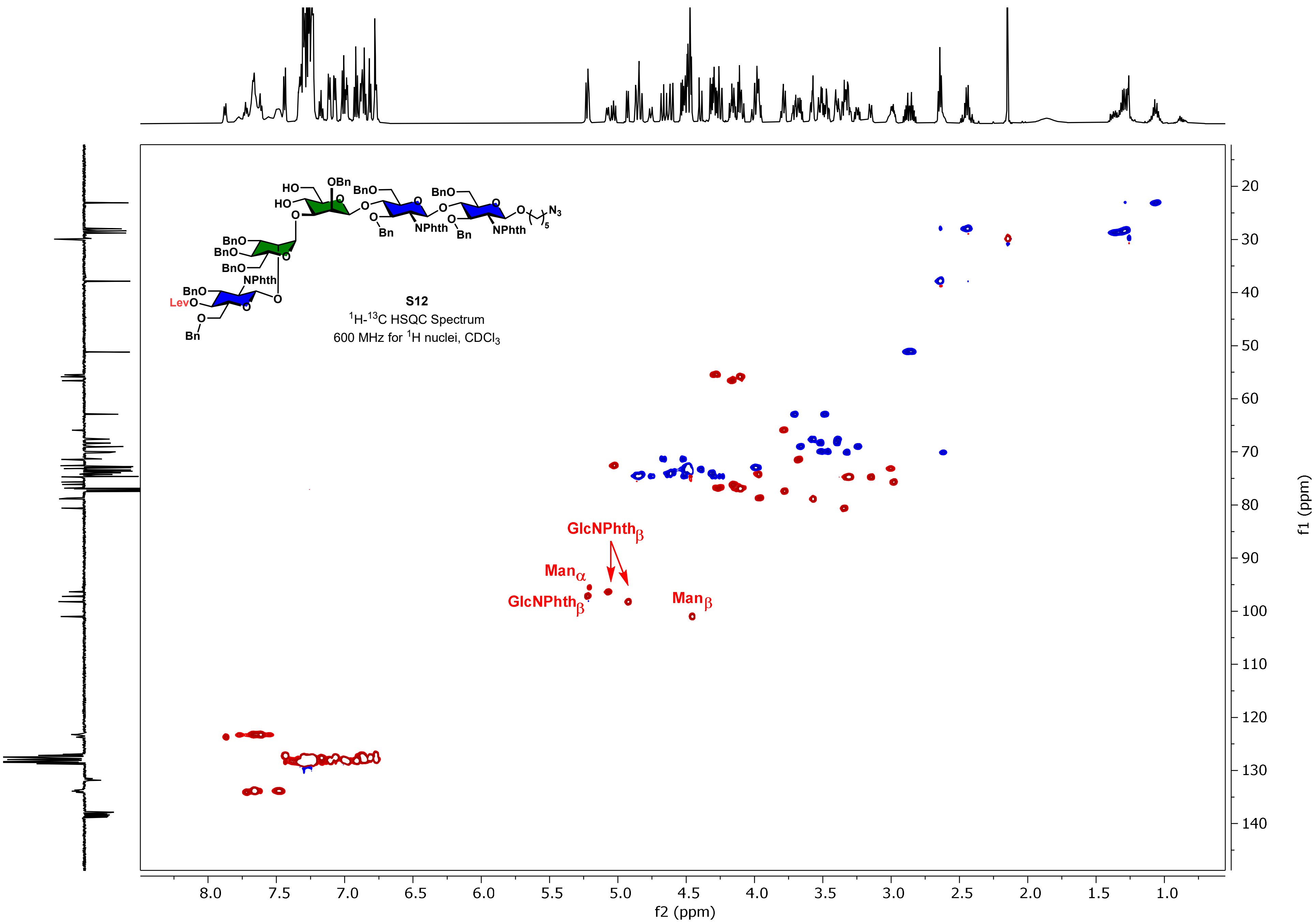
S12
^1H-^{13}C HSQC Spectrum
600 MHz for ^1H nuclei, $CDCl_3$
GlcNPhthβ
Manα
GlcNPhthβ
Manβ
f2 (ppm)
f1 (ppm)

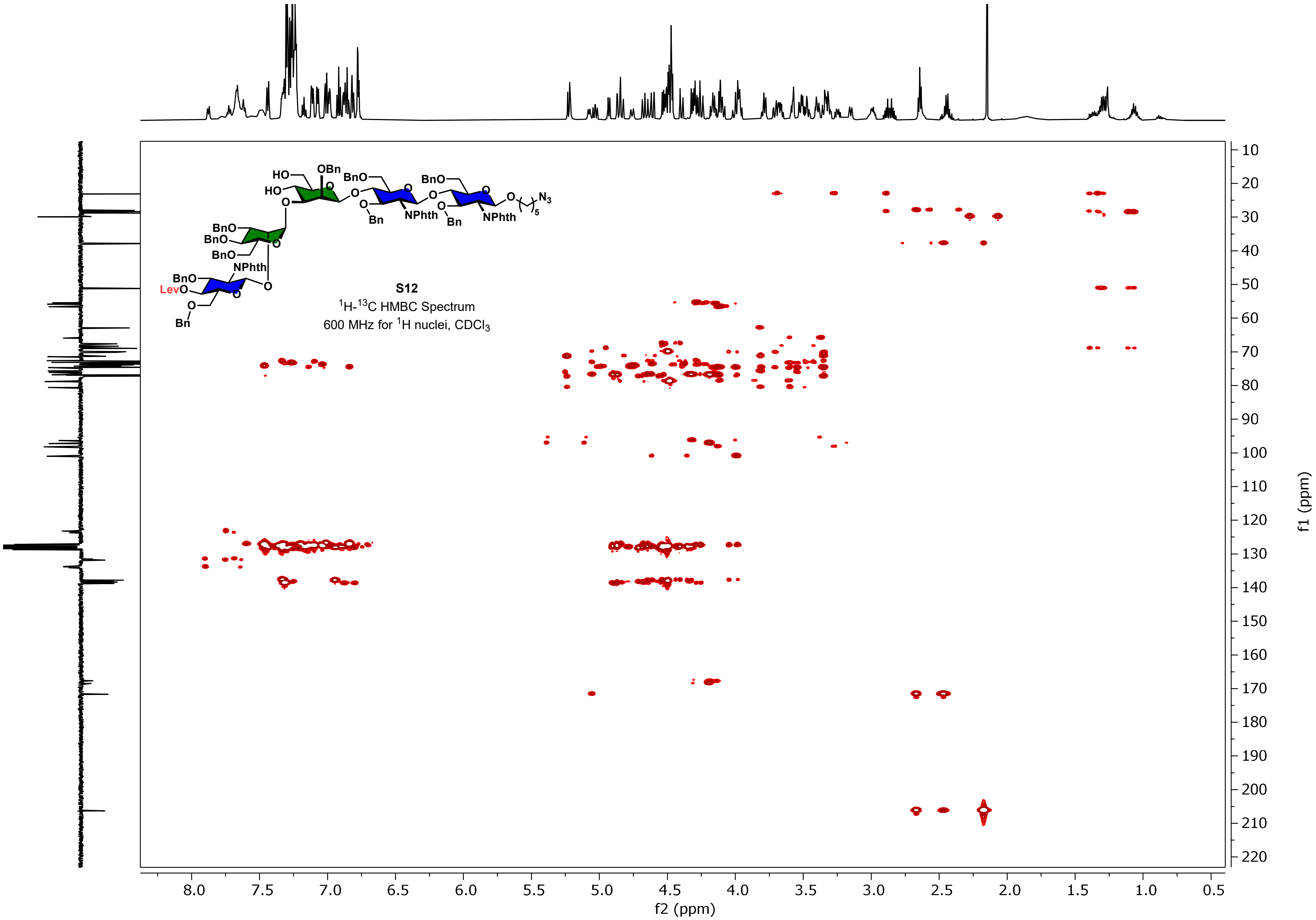
S12
^{1}H-^{13}C HMBC Spectrum
600 MHz for ^{1}H nuclei, $CDCl_3$
f2 (ppm)
f1 (ppm)

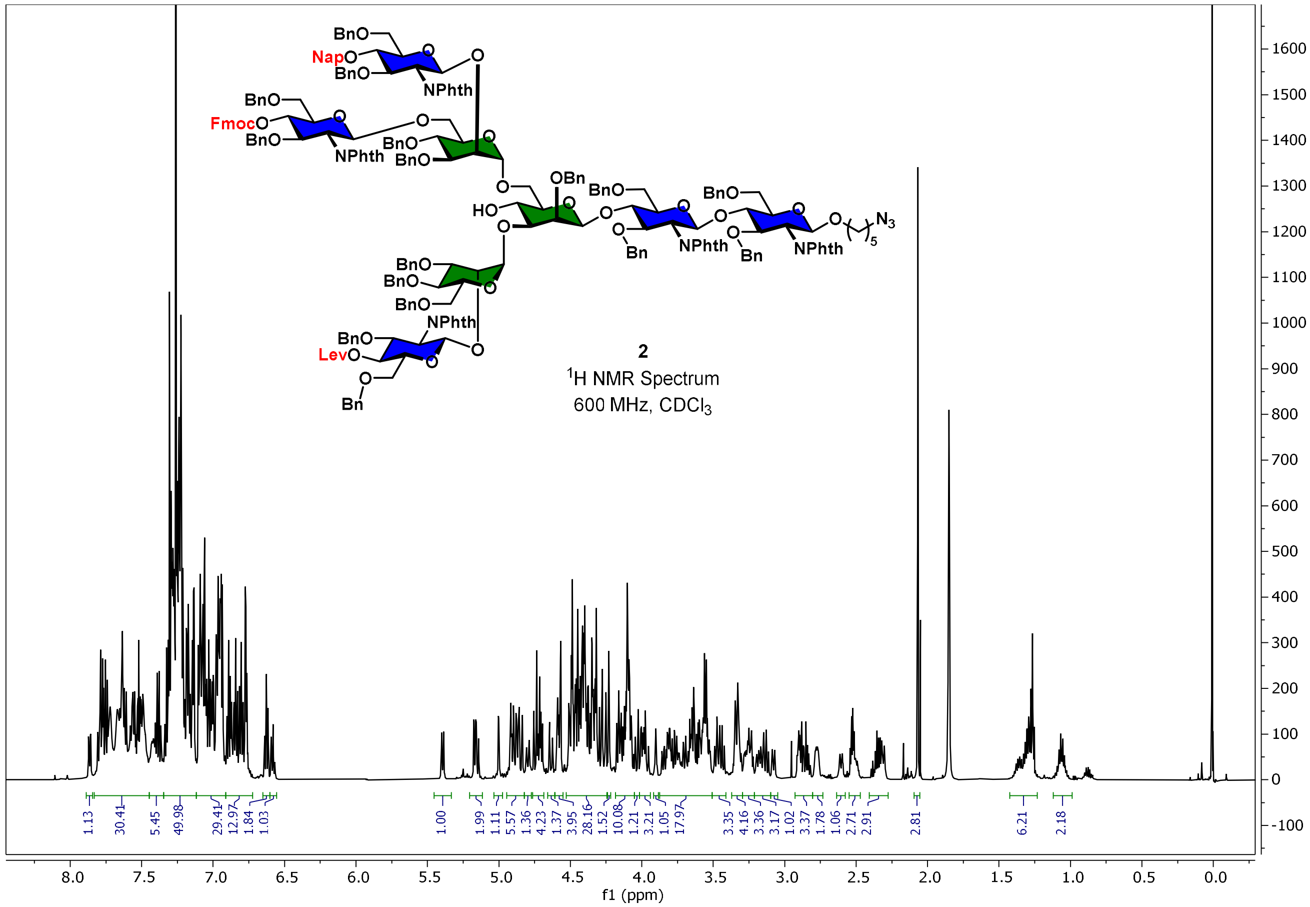
BnO
Nap
BnO
NPhth
BnO
Fmoc
BnO
NPhth
BnO
BnO
OBn
BnO
BnO
HO
Bn
NPhth
Bn
NPhth
N3
5
BnO
BnO
BnO
NPhth
BnO
Lev
Bn
2
1H NMR Spectrum
600 MHz, CDCl3
1.13
30.41
5.45
49.98
29.41
12.97
1.84
1.03
1.00
1.99
1.11
5.57
1.36
4.23
1.37
3.95
28.16
1.52
10.08
1.21
3.21
1.05
17.97
3.35
4.16
3.36
3.17
1.02
3.37
1.78
1.06
2.71
2.91
2.81
6.21
2.18
f1 (ppm)

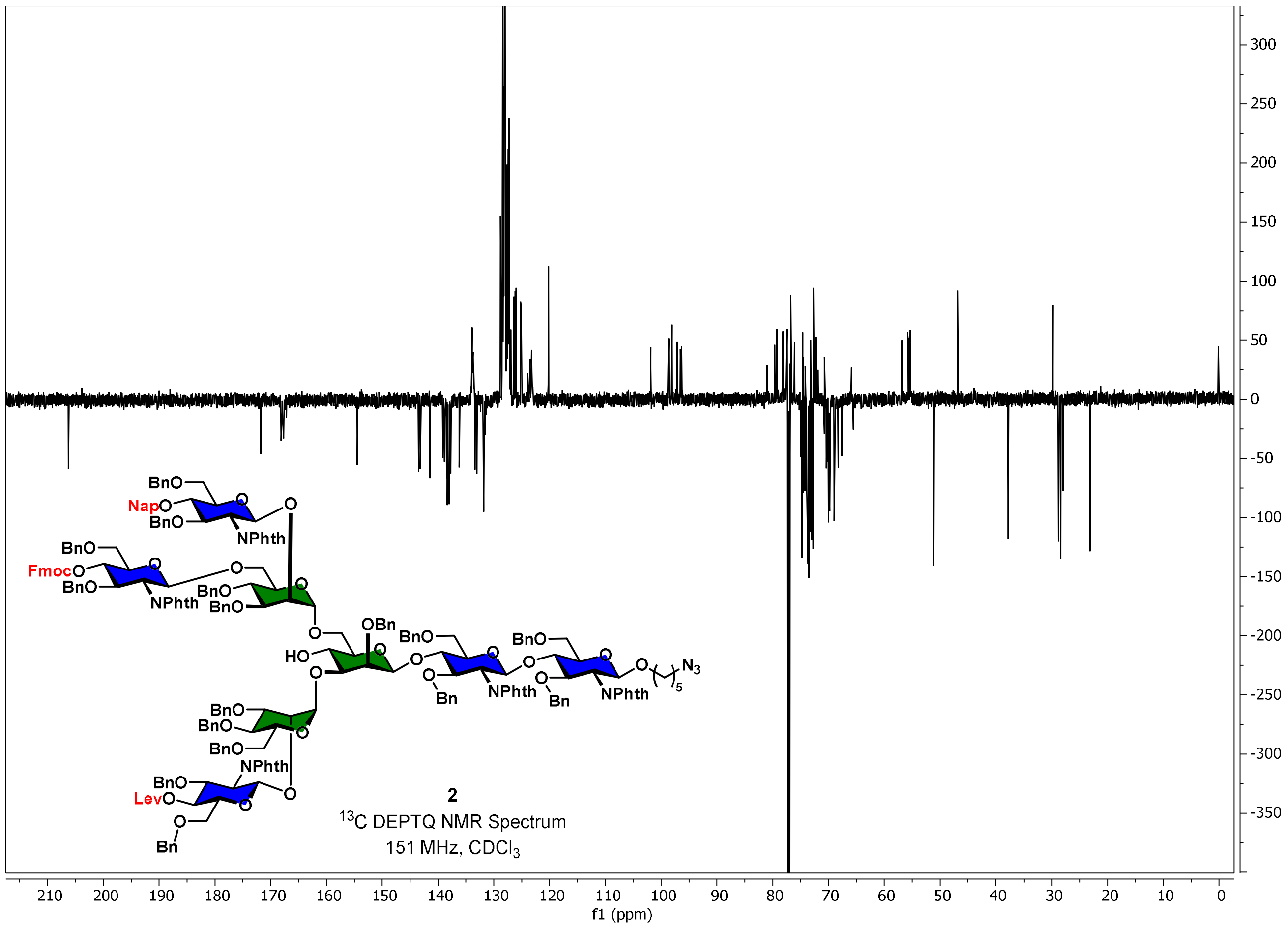
BnO
Nap
BnO
NPhth
BnO
Fmoc
BnO
NPhth
BnO
BnO
OBn
BnO
BnO
HO
Bn
NPhth
Bn
NPhth
N3
5
BnO
BnO
BnO
NPhth
BnO
Lev
Bn
2
13C DEPTQ NMR Spectrum
151 MHz, CDCl3
f1 (ppm)

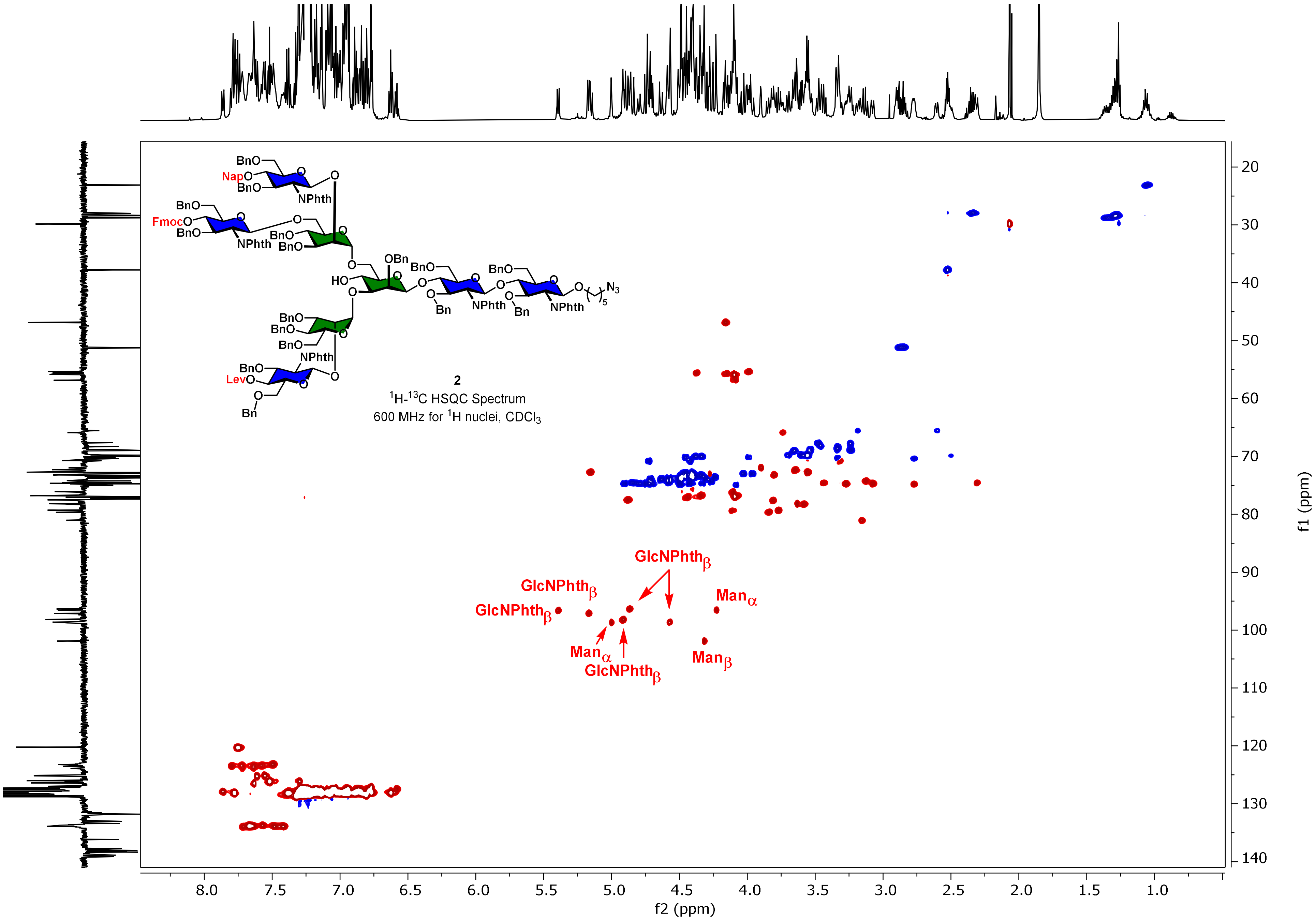
2
^{1}H-^{13}C HSQC Spectrum
600 MHz for ^{1}H nuclei, $CDCl_3$
GlcNPhthβ
GlcNPhthβ
GlcNPhthβ
Manα
Manα
GlcNPhthβ
Manβ
f2 (ppm)
f1 (ppm)

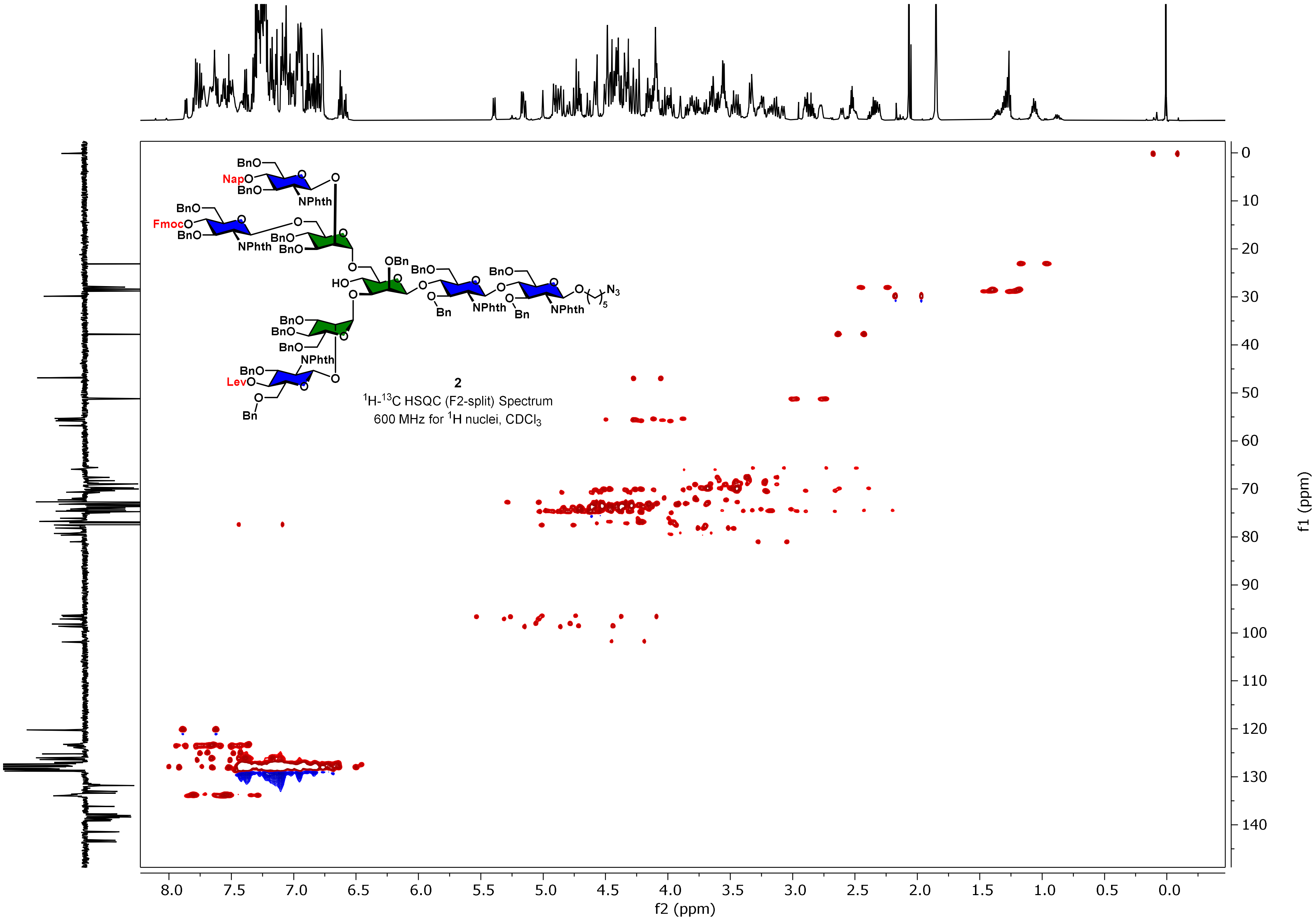
2
^{1}H-^{13}C HSQC (F2-split) Spectrum
600 MHz for ^{1}H nuclei, $CDCl_3$
f2 (ppm)
f1 (ppm)

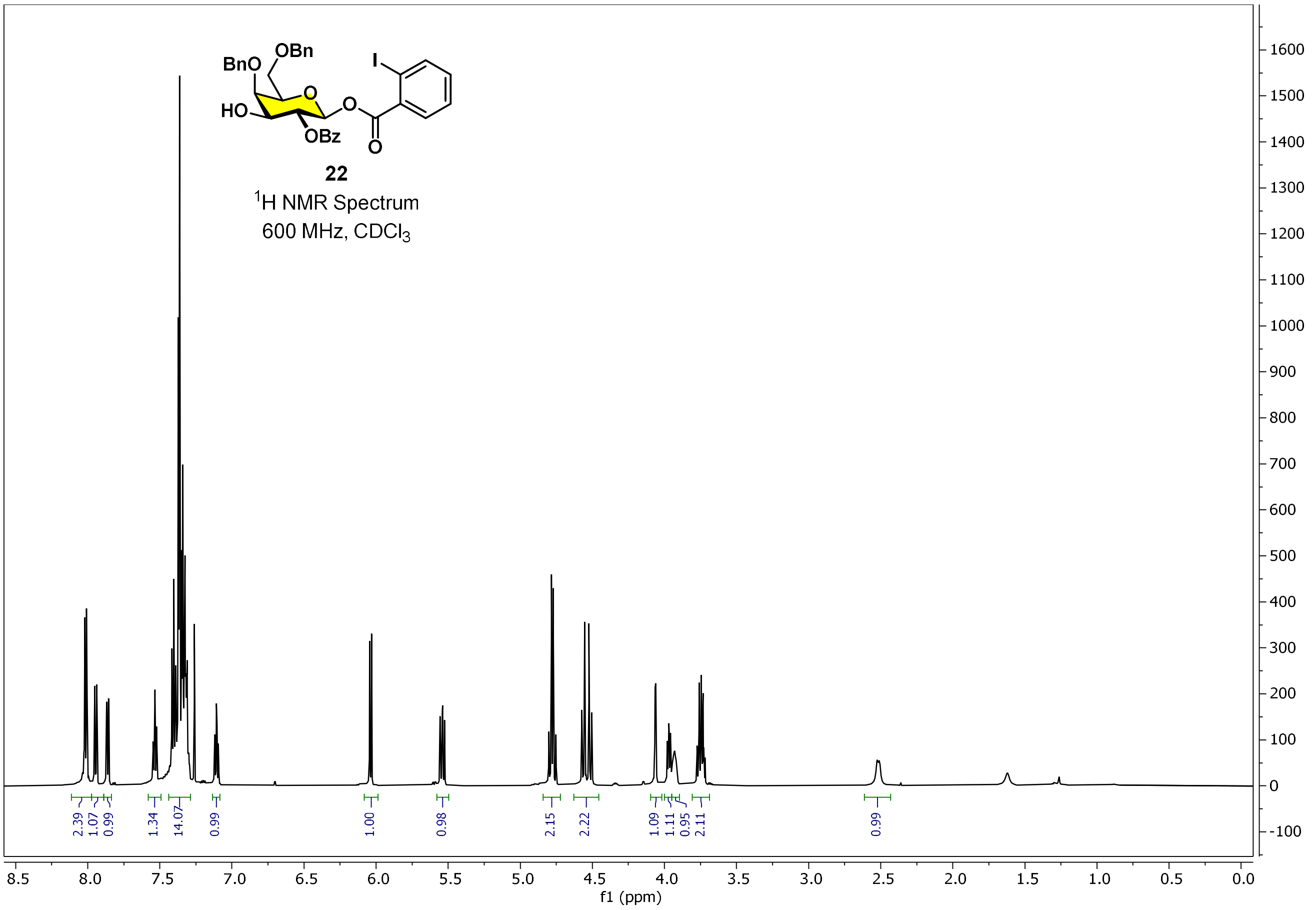
22
^{1}H NMR Spectrum
600 MHz, CDCl$_3$
f1 (ppm)

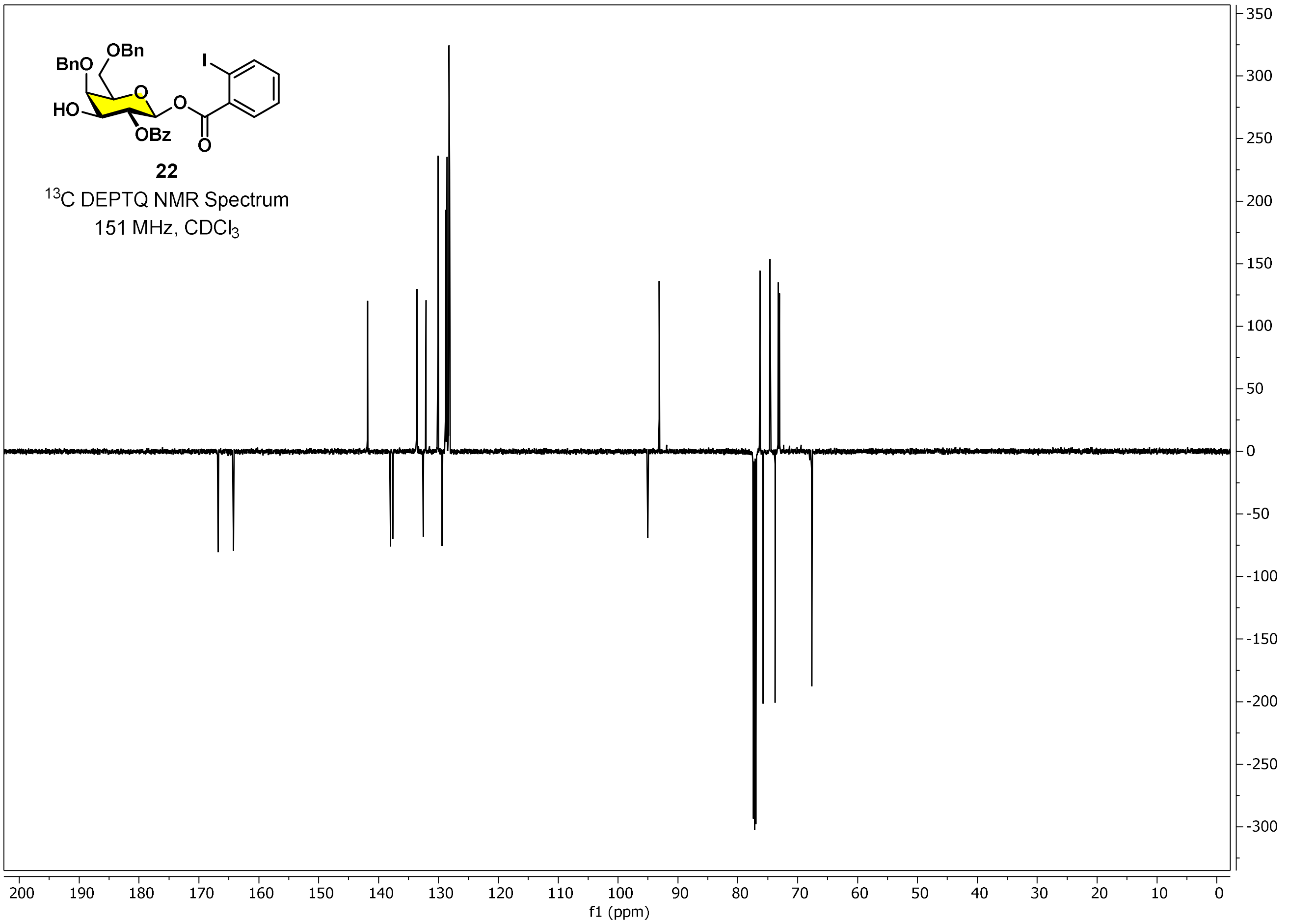
22
^{13}C DEPTQ NMR Spectrum
151 MHz, CDCl$_3$
f1 (ppm)

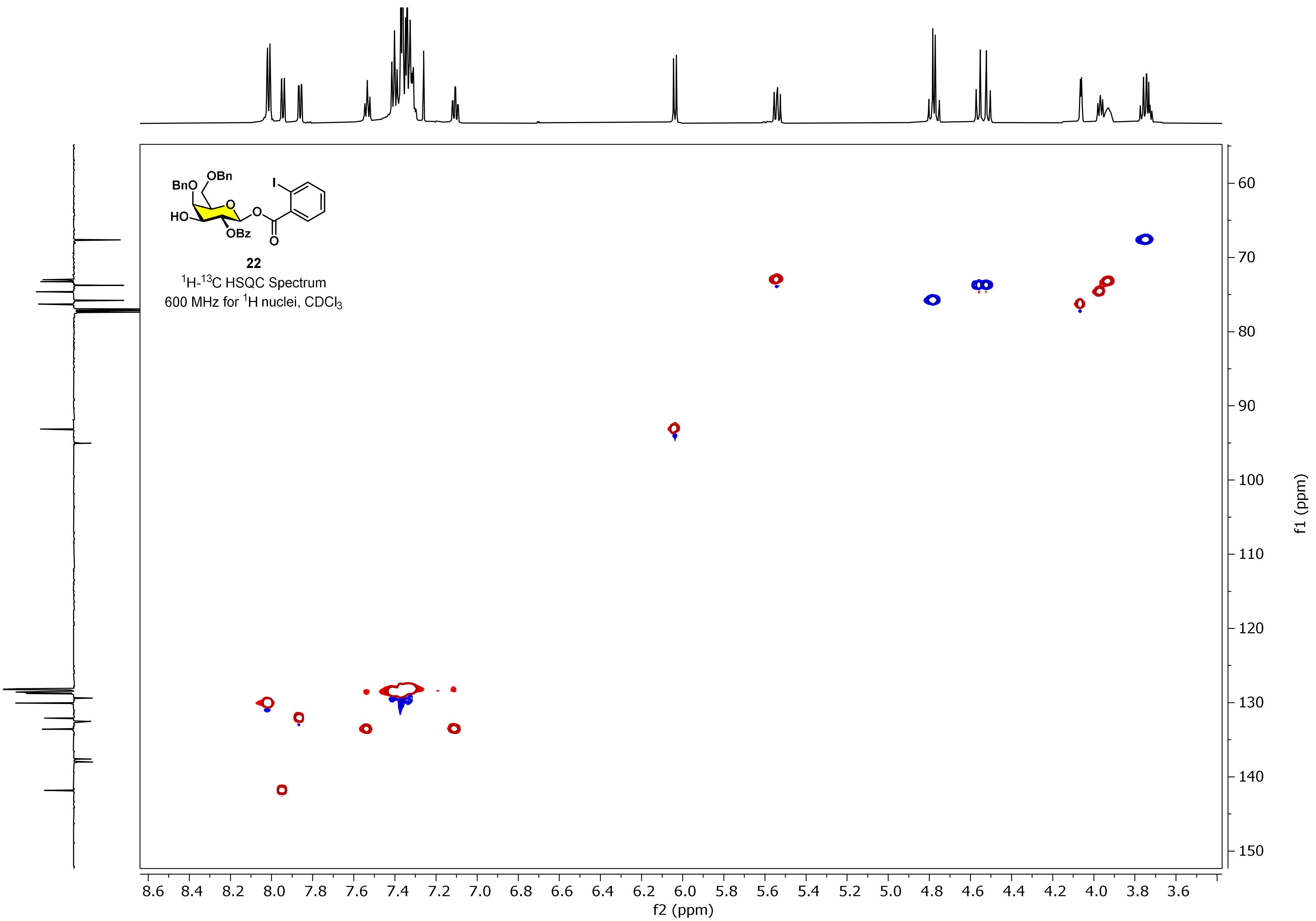
22
1H-13C HSQC Spectrum
600 MHz for 1H nuclei, CDCl3
f2 (ppm)
f1 (ppm)

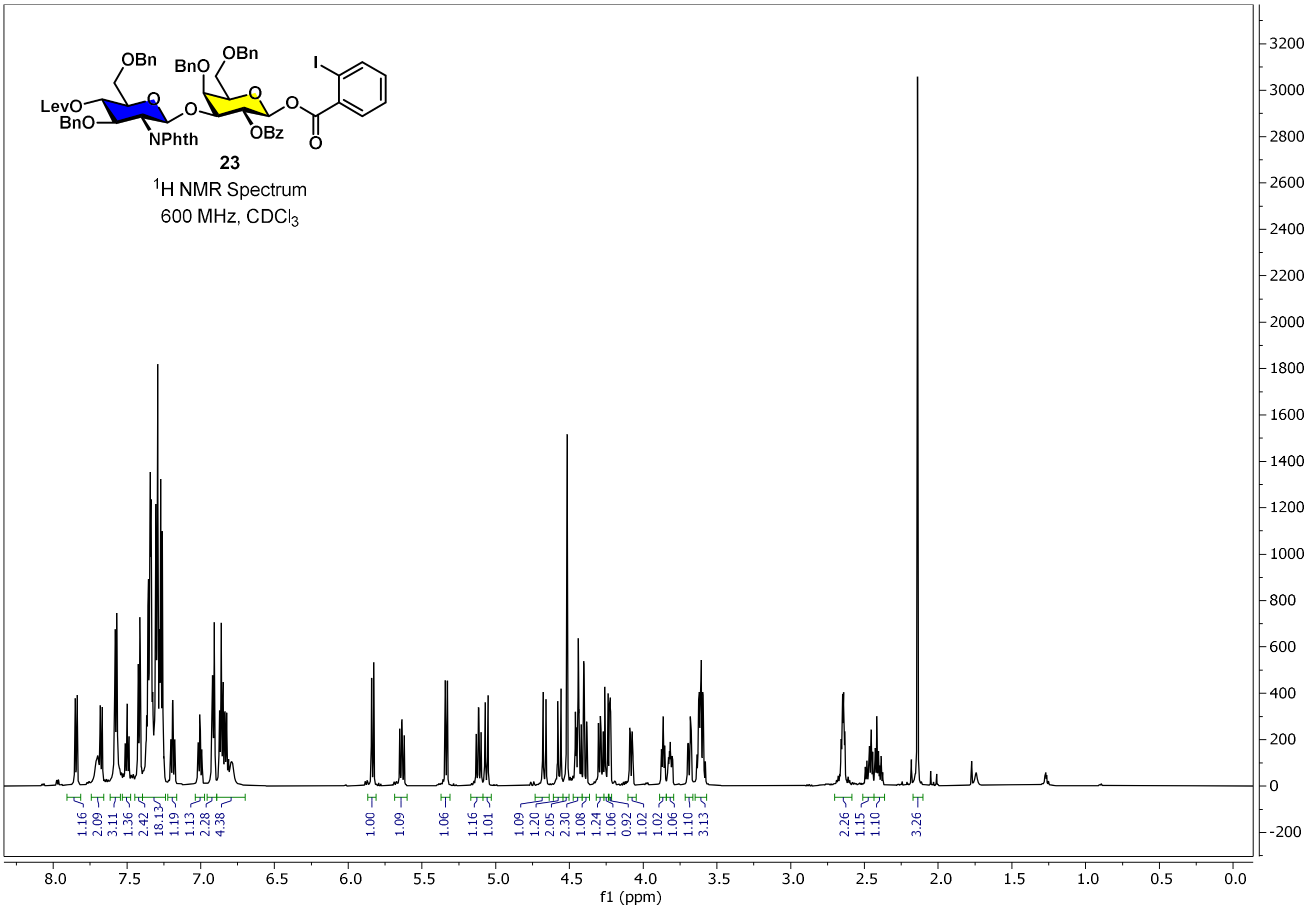

OBn
BnO
OBn
LevO
BnO
NPhth
OBz
I
O
23
^{1}H NMR Spectrum
600 MHz, $CDCl_3$
f1 (ppm)

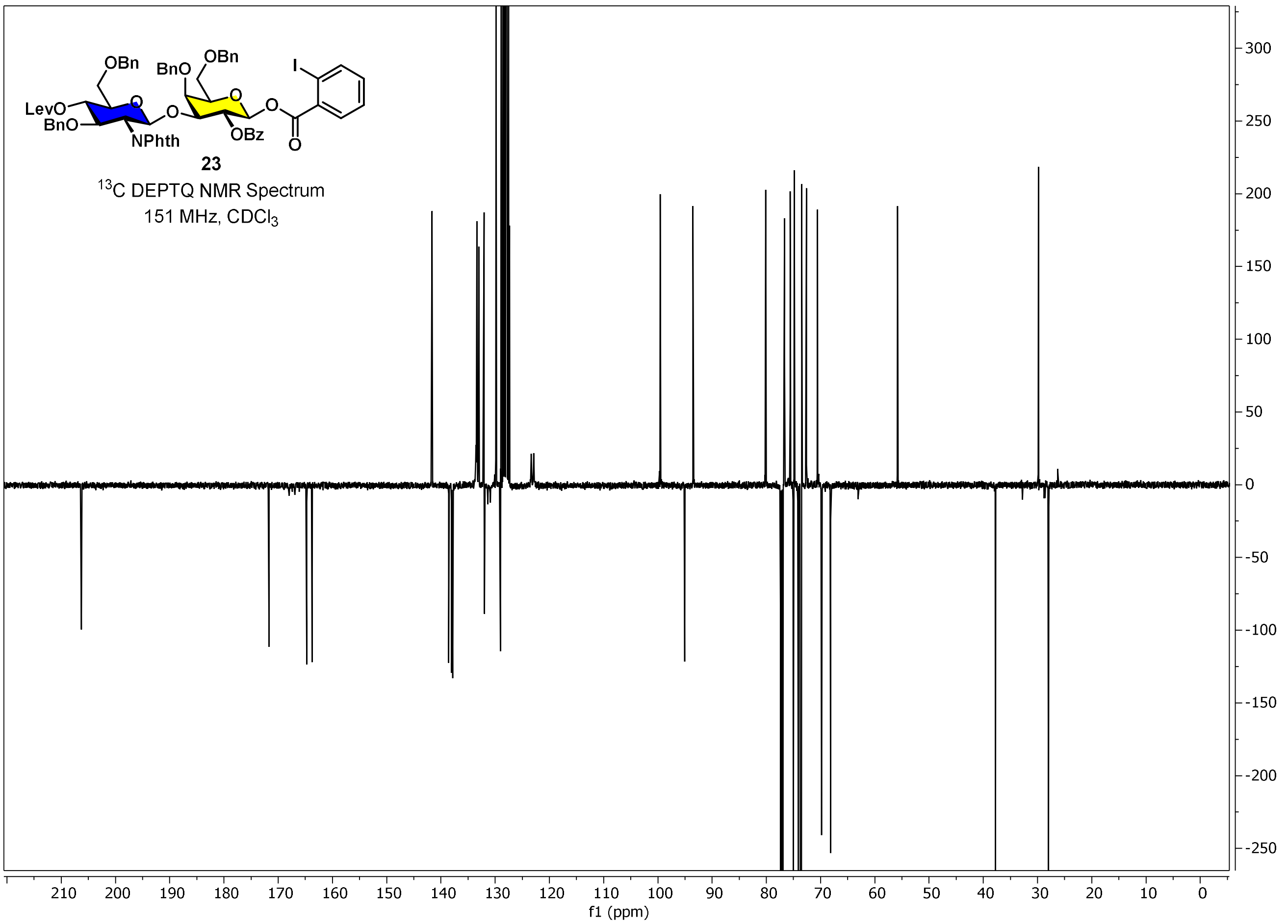

OBn
BnO
OBn
LevO
BnO
NPhth
OBz
I
O
23
^{13}C DEPTQ NMR Spectrum
151 MHz, $CDCl_3$
f1 (ppm)

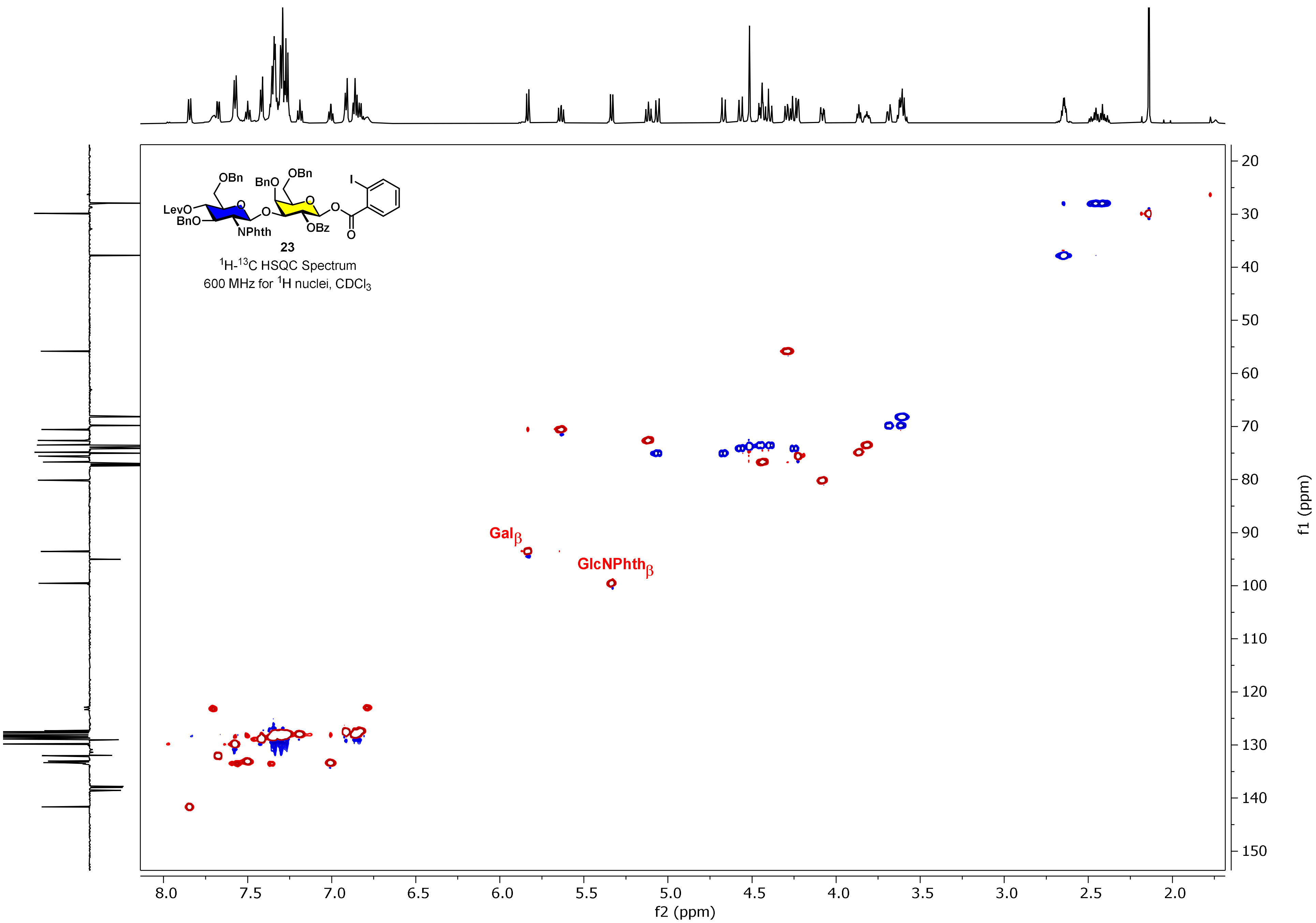
OBn
BnO
OBn
I
LevO
BnO
NPhth
OBz
23
^{1}H-^{13}C HSQC Spectrum
600 MHz for ^{1}H nuclei, CDCl$_3$
Gal$_\beta$
GlcNPhth$_\beta$
f2 (ppm)
f1 (ppm)

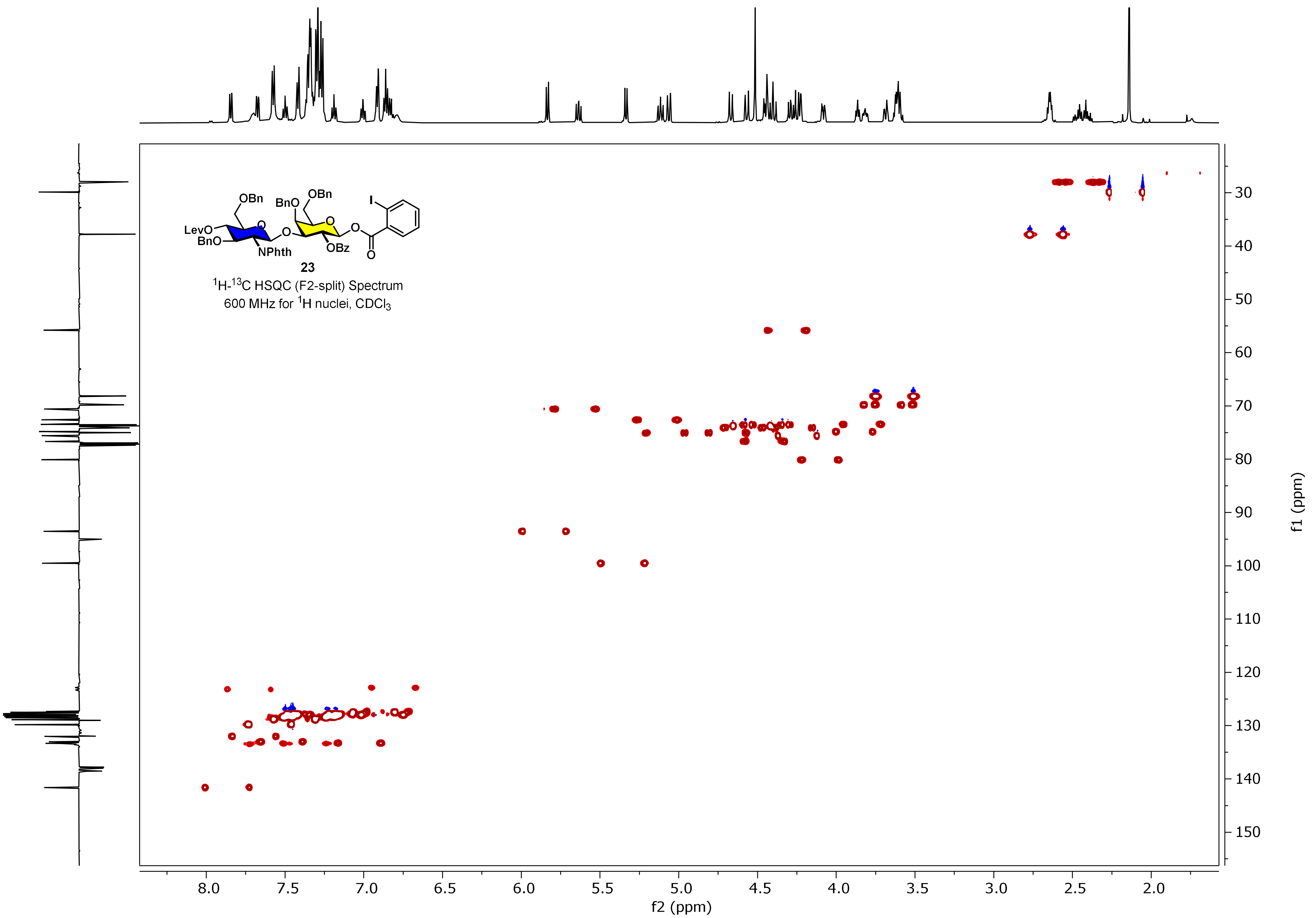

OBn
BnO
OBn
I
LevO
BnO
NPhth
OBz
O
23
1H-13C HSQC (F2-split) Spectrum
600 MHz for 1H nuclei, CDCl3
f2 (ppm)
f1 (ppm)

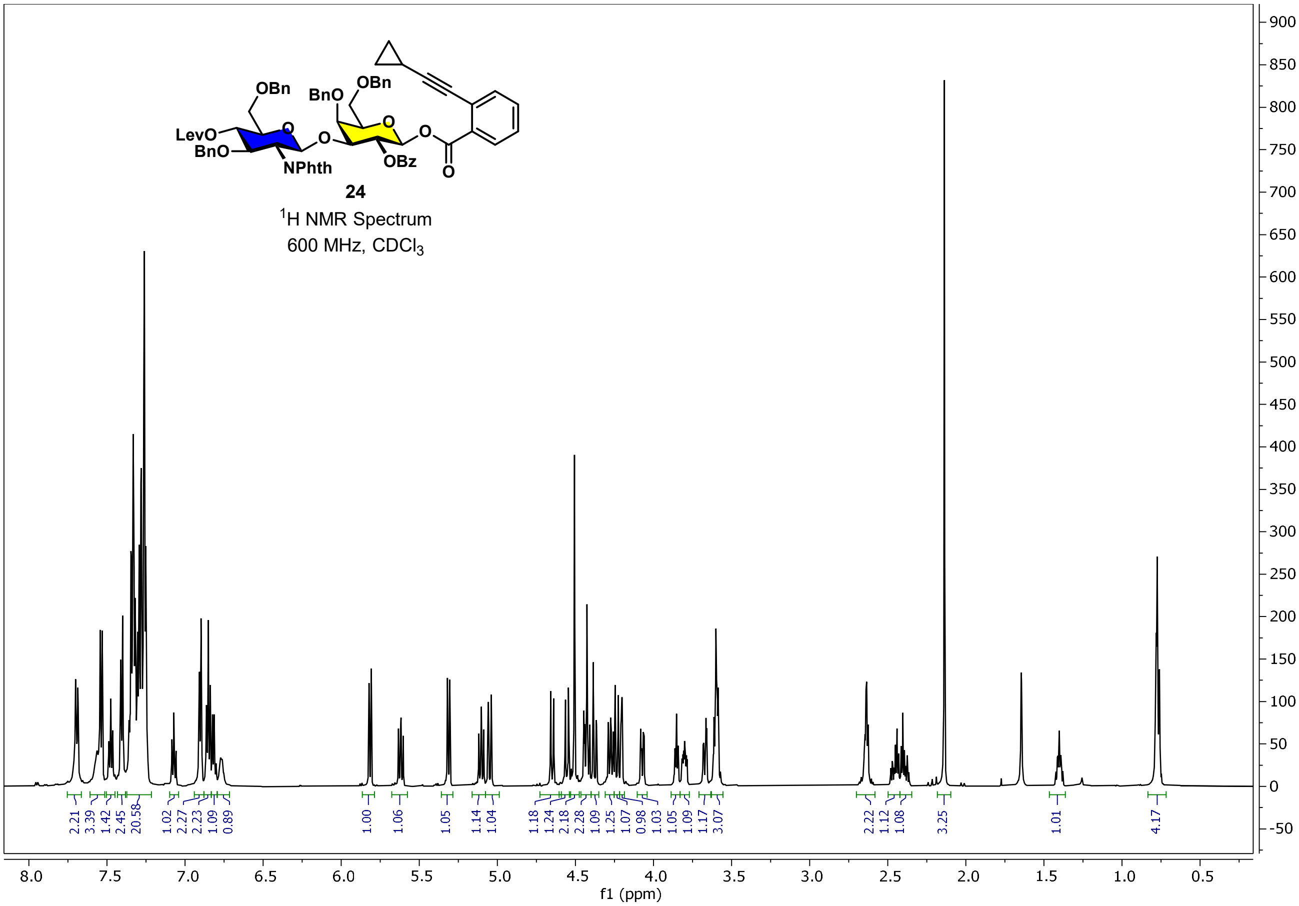
OBn
BnO
OBn
LevO
BnO
NPhth
OBz
O
24
1H NMR Spectrum
600 MHz, CDCl3
f1 (ppm)

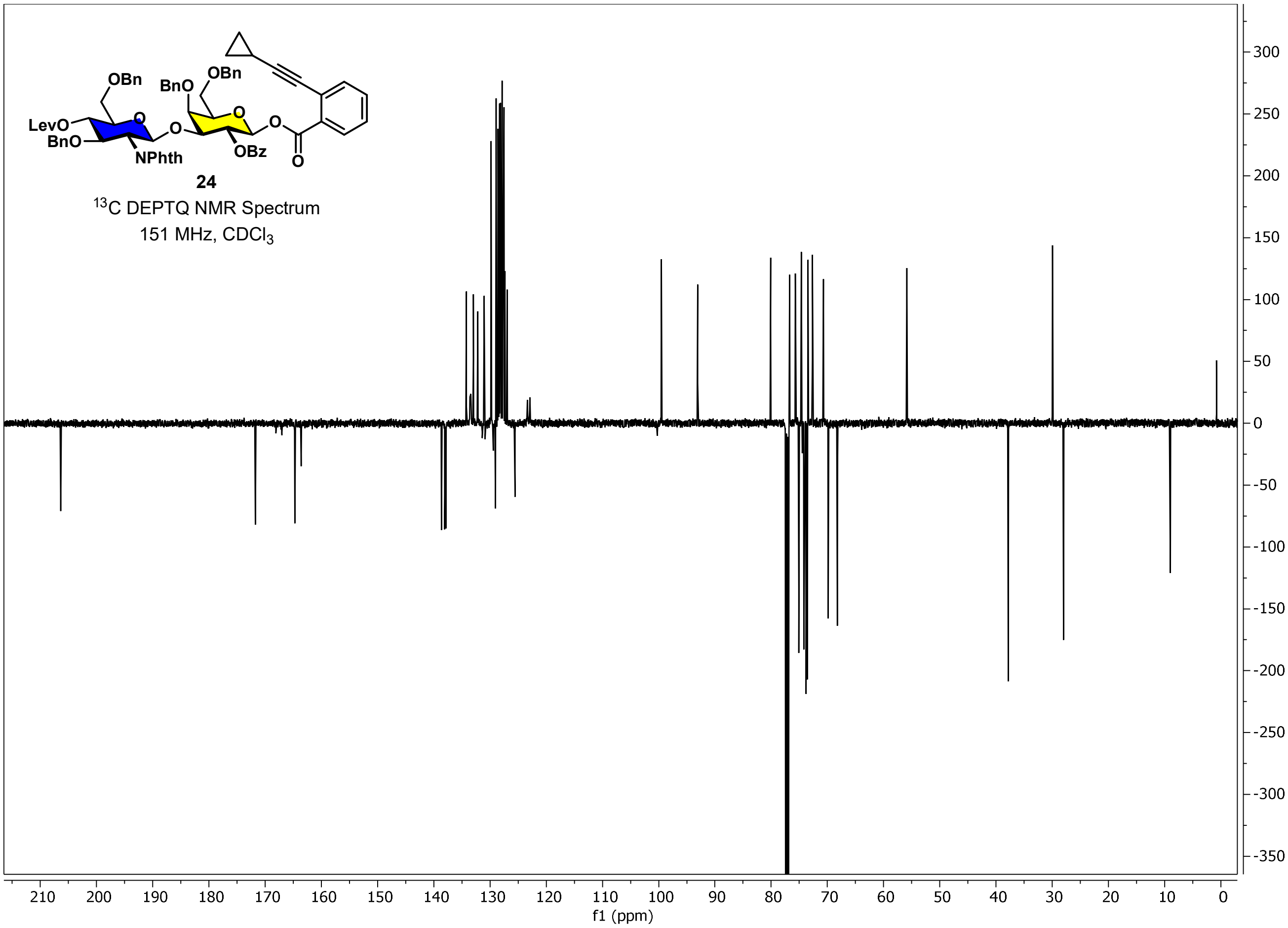
OBn
BnO
OBn
LevO
BnO
NPhth
OBz
O
24
13C DEPTQ NMR Spectrum
151 MHz, CDCl3
f1 (ppm)

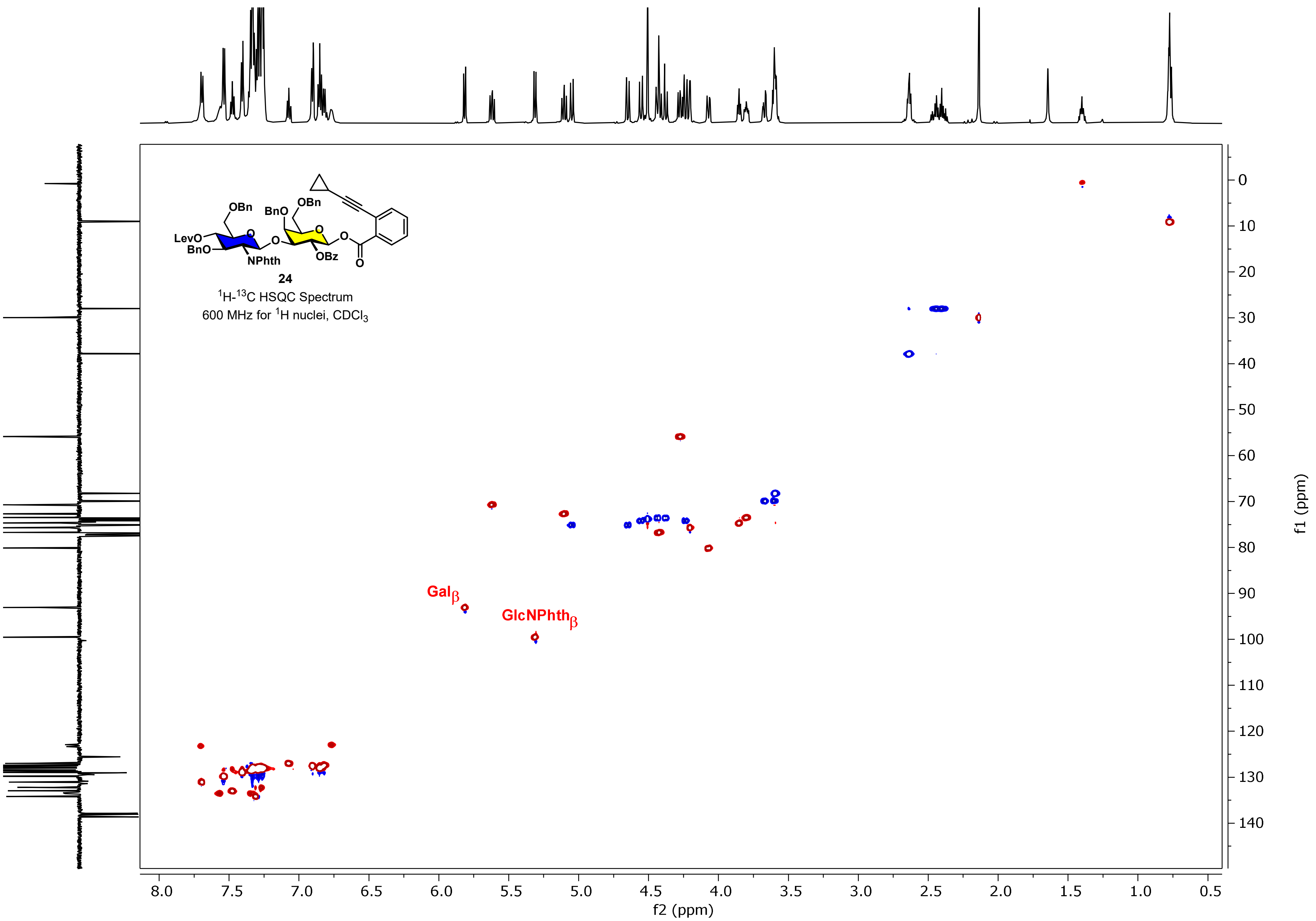

OBn
BnO
OBn
LevO
BnO
O
O
O
O
NPhth
OBz
O
24
^{1}H-^{13}C HSQC Spectrum
600 MHz for ^{1}H nuclei, CDCl$_3$
Gal$_\beta$
GlcNPhth$_\beta$
f2 (ppm)
f1 (ppm)

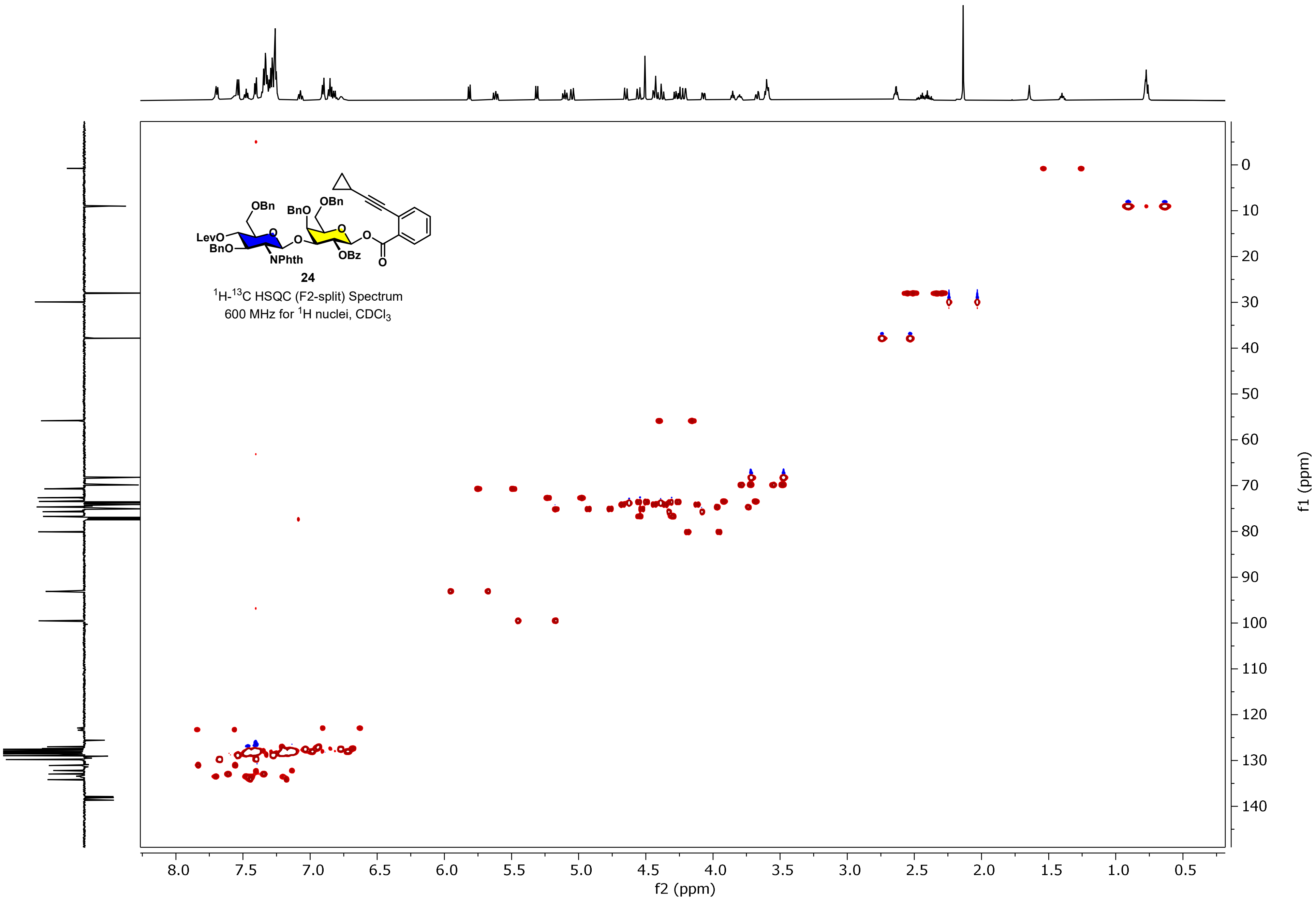
OBn
BnO
OBn
LevO
BnO
NPhth
OBz
O
24
1H-13C HSQC (F2-split) Spectrum
600 MHz for 1H nuclei, CDCl3
f2 (ppm)
f1 (ppm)

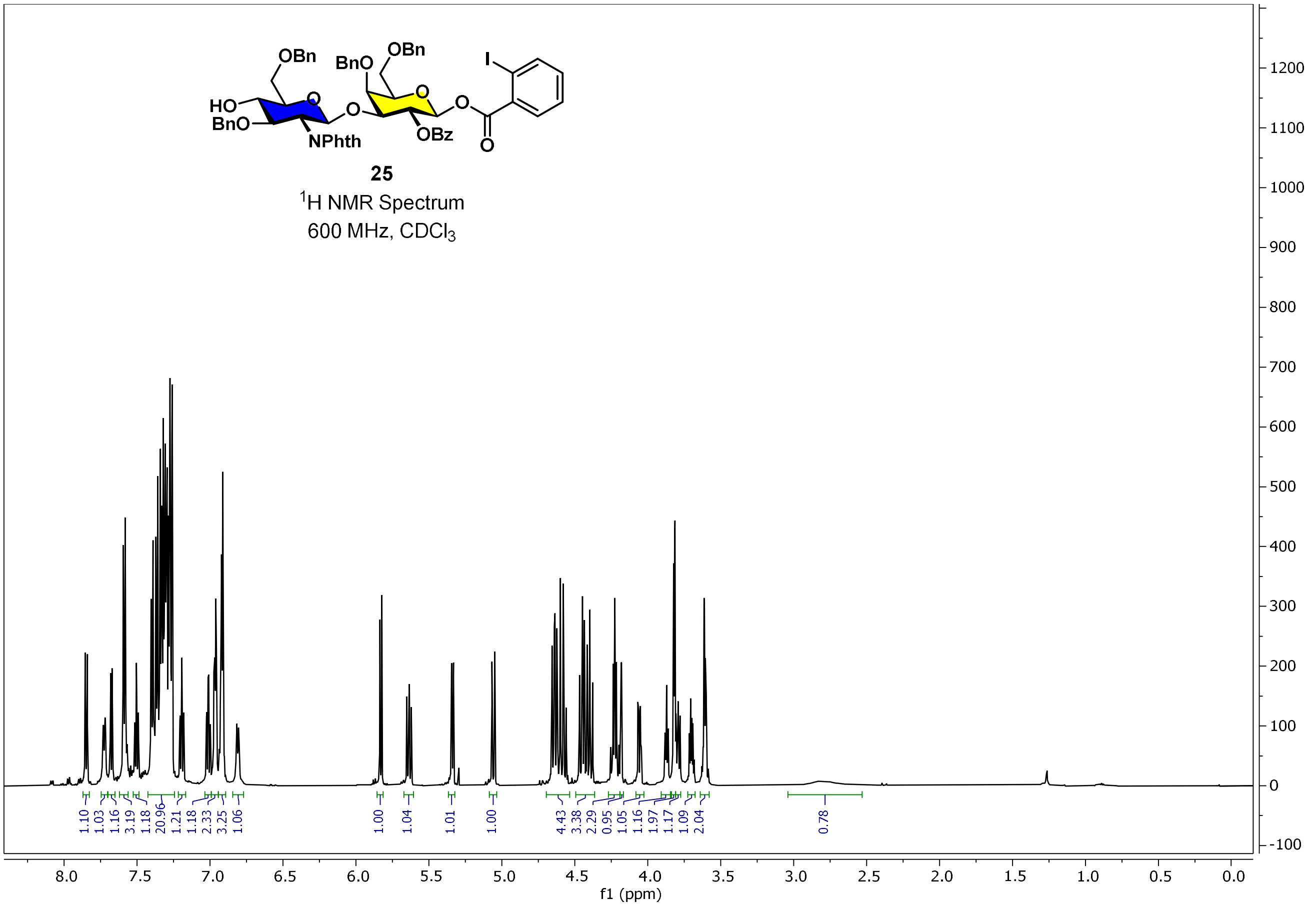
25
1H NMR Spectrum
600 MHz, CDCl3
f1 (ppm)

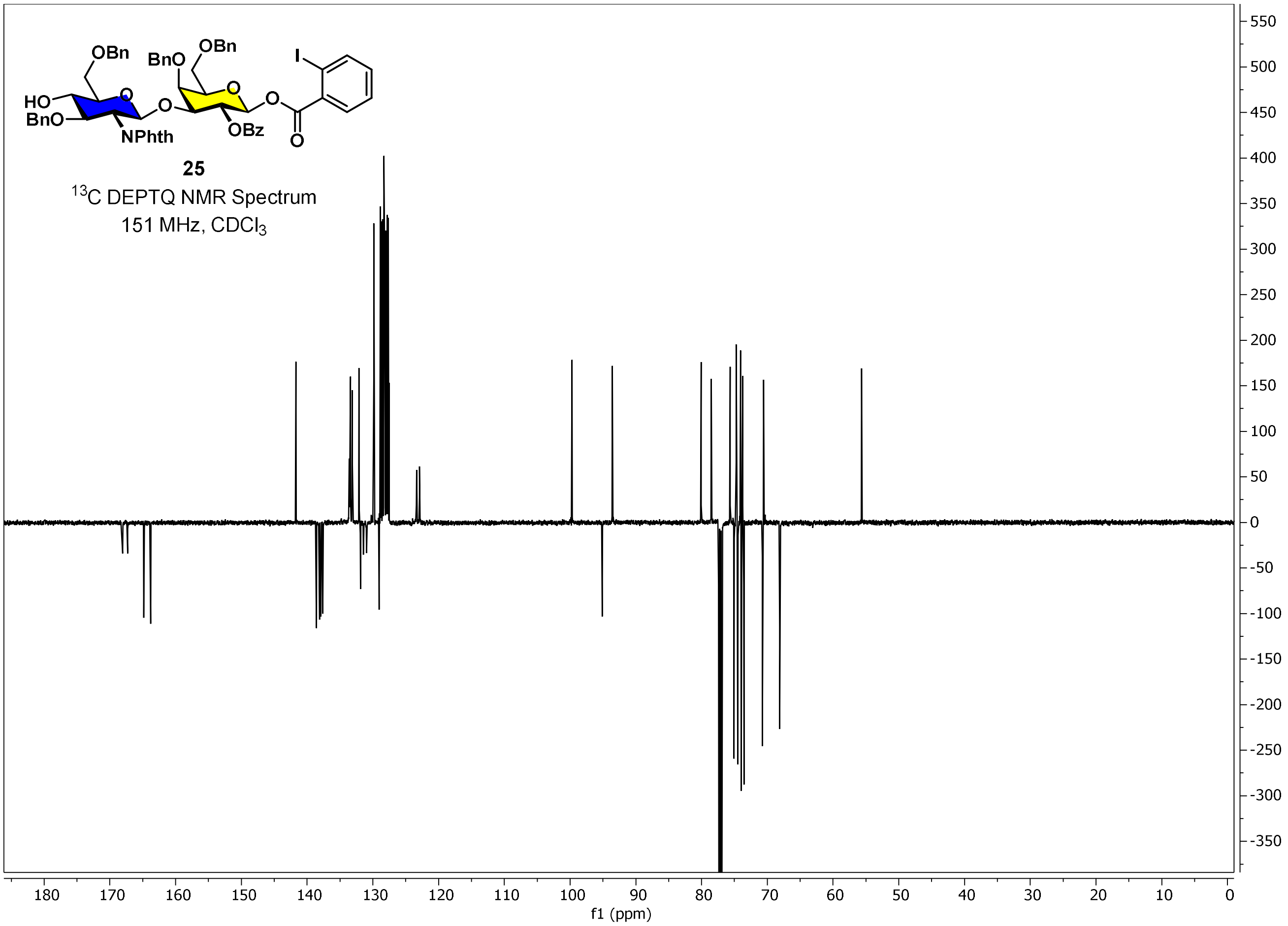
25
13C DEPTQ NMR Spectrum
151 MHz, CDCl3
f1 (ppm)

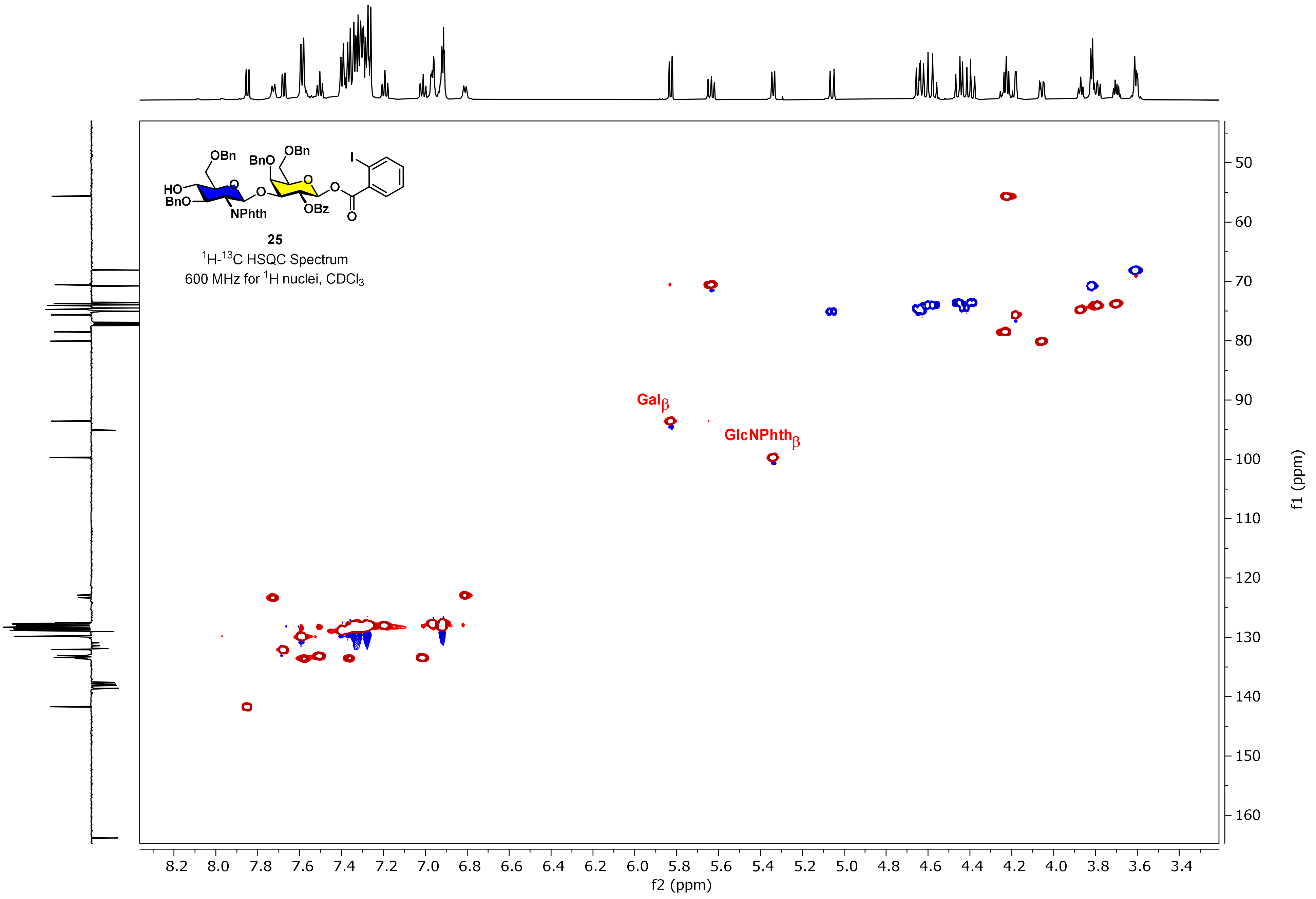

OBn
BnO
OBn
I
HO
BnO
O
O
O
O
NPhth
OBz
O
25
^{1}H-^{13}C HSQC Spectrum
600 MHz for ^{1}H nuclei, CDCl$_3$
Gal$_\beta$
GlcNPhth$_\beta$
f2 (ppm)
f1 (ppm)

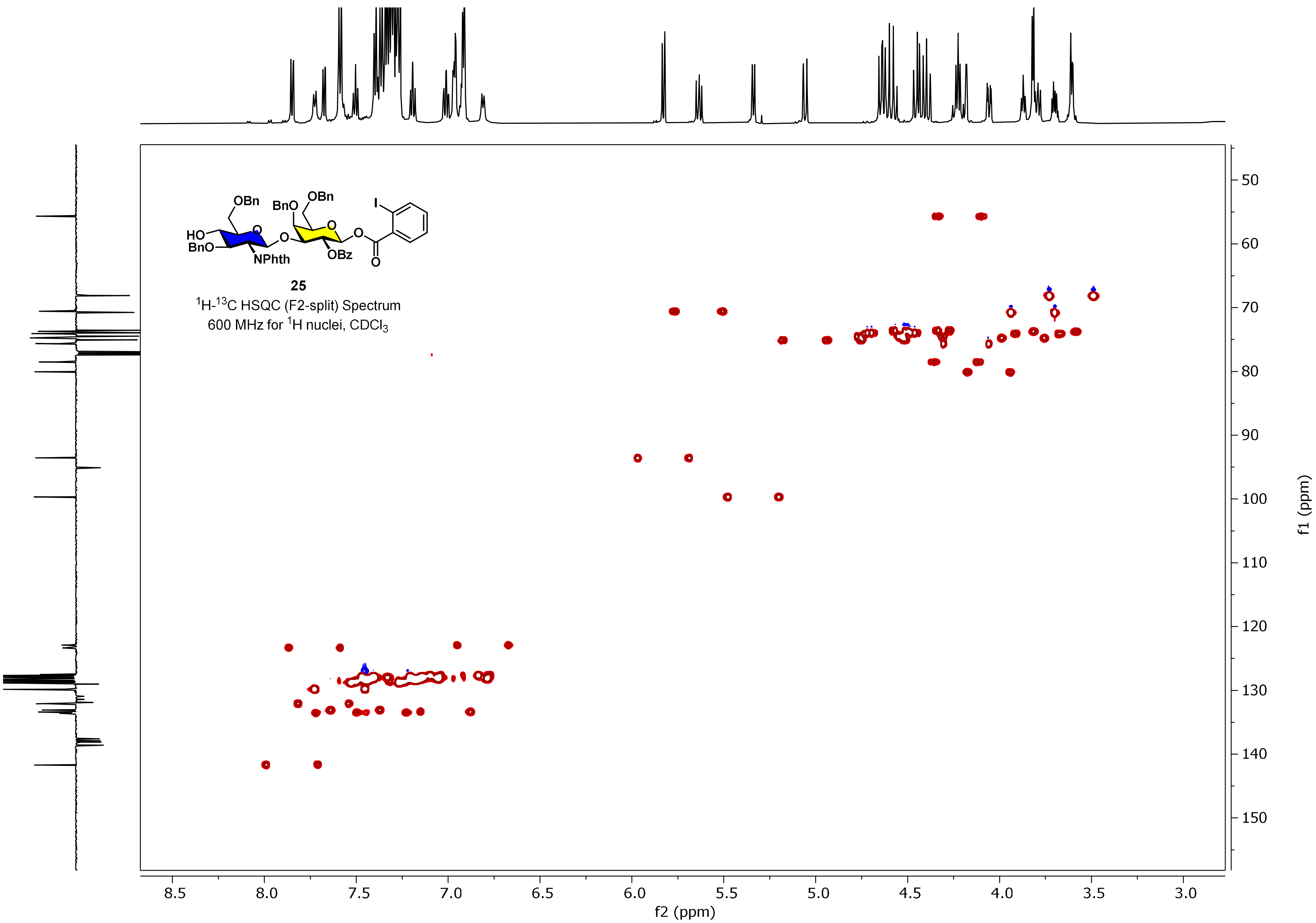

OBn
BnO
OBn
I
HO
BnO
O
O
O
O
NPhth
OBz
O
25
^1H-^{13}C HSQC (F2-split) Spectrum
600 MHz for ^1H nuclei, $CDCl_3$
f2 (ppm)
f1 (ppm)
8.5
8.0
7.5
7.0
6.5
6.0
5.5
5.0
4.5
4.0
3.5
3.0
50
60
70
80
90
100
110
120
130
140
150

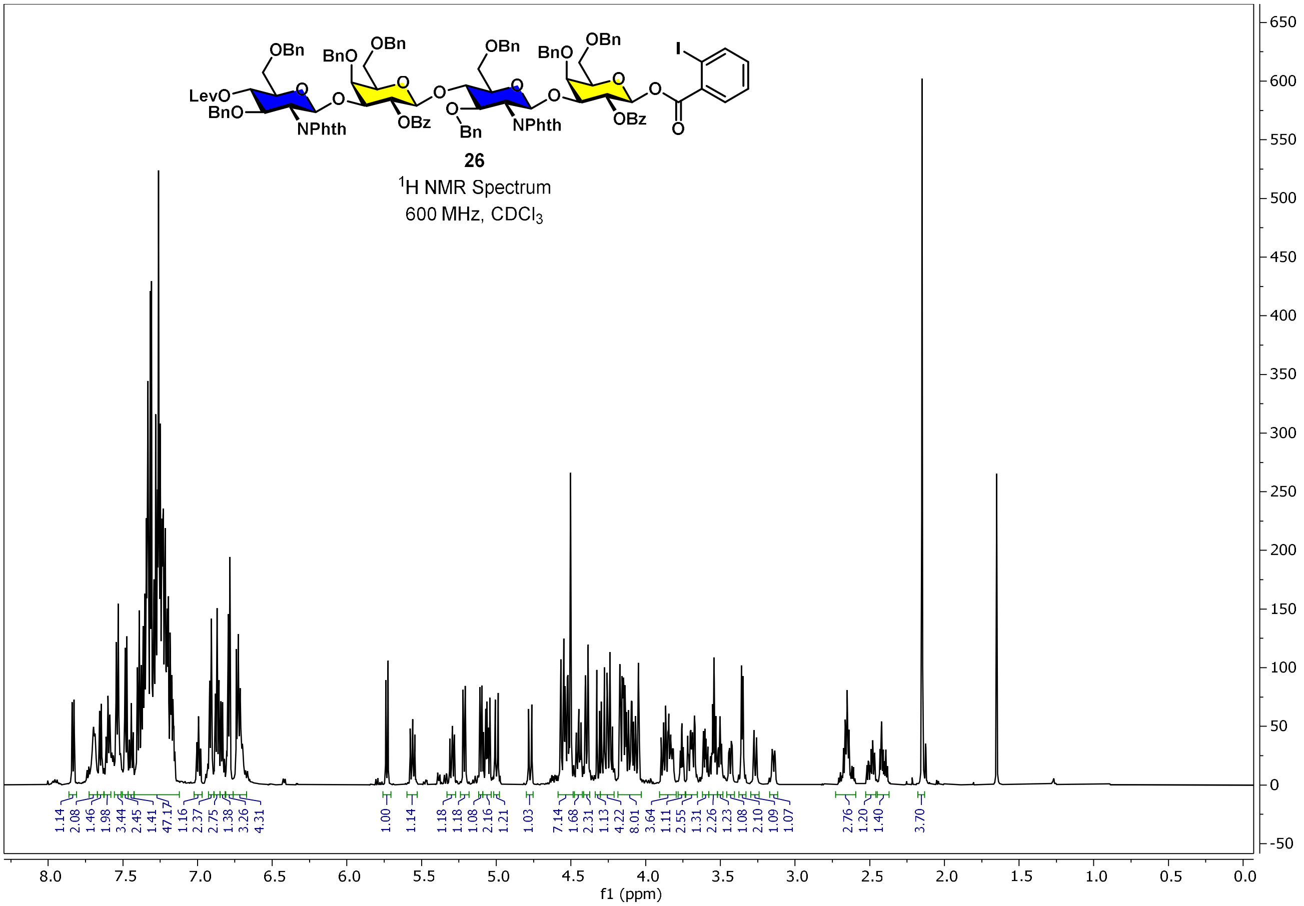

26
^{1}H NMR Spectrum
600 MHz, CDCl$_3$
f1 (ppm)

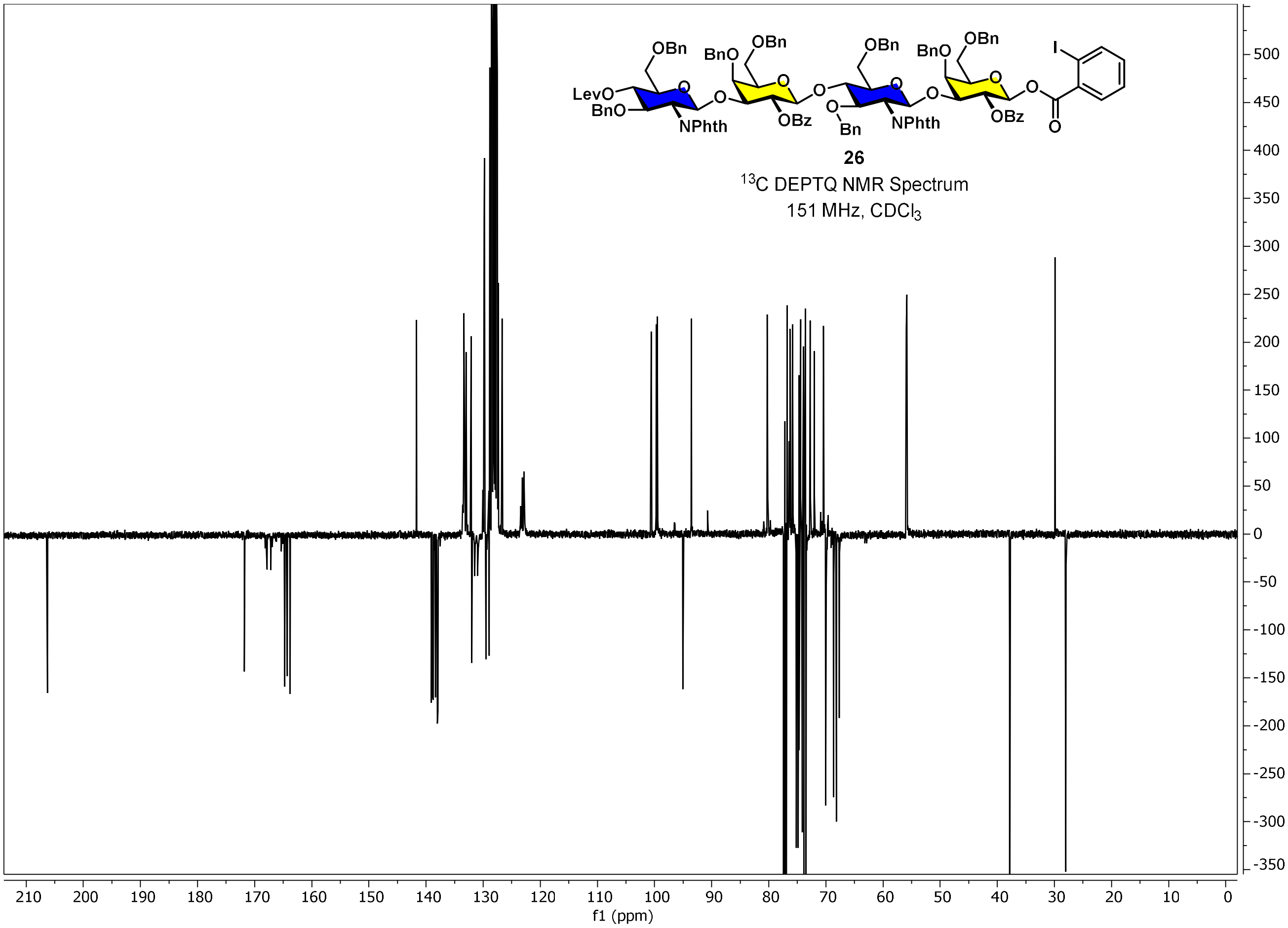

26
^{13}C DEPTQ NMR Spectrum
151 MHz, CDCl$_3$
f1 (ppm)

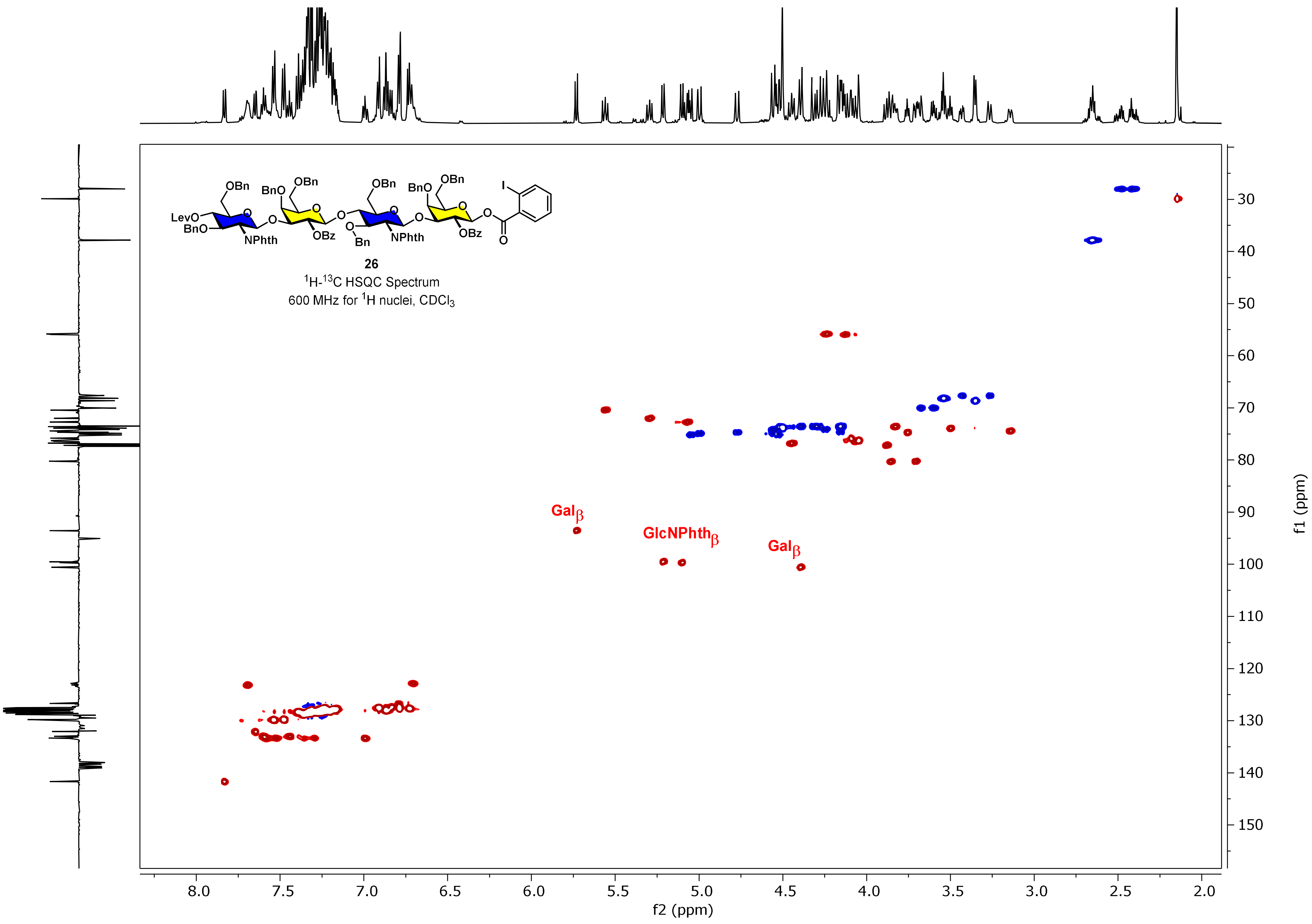

26
^{1}H-^{13}C HSQC Spectrum
600 MHz for ^{1}H nuclei, CDCl$_3$
Gal$_\beta$
GlcNPhth$_\beta$
Gal$_\beta$
f2 (ppm)
f1 (ppm)

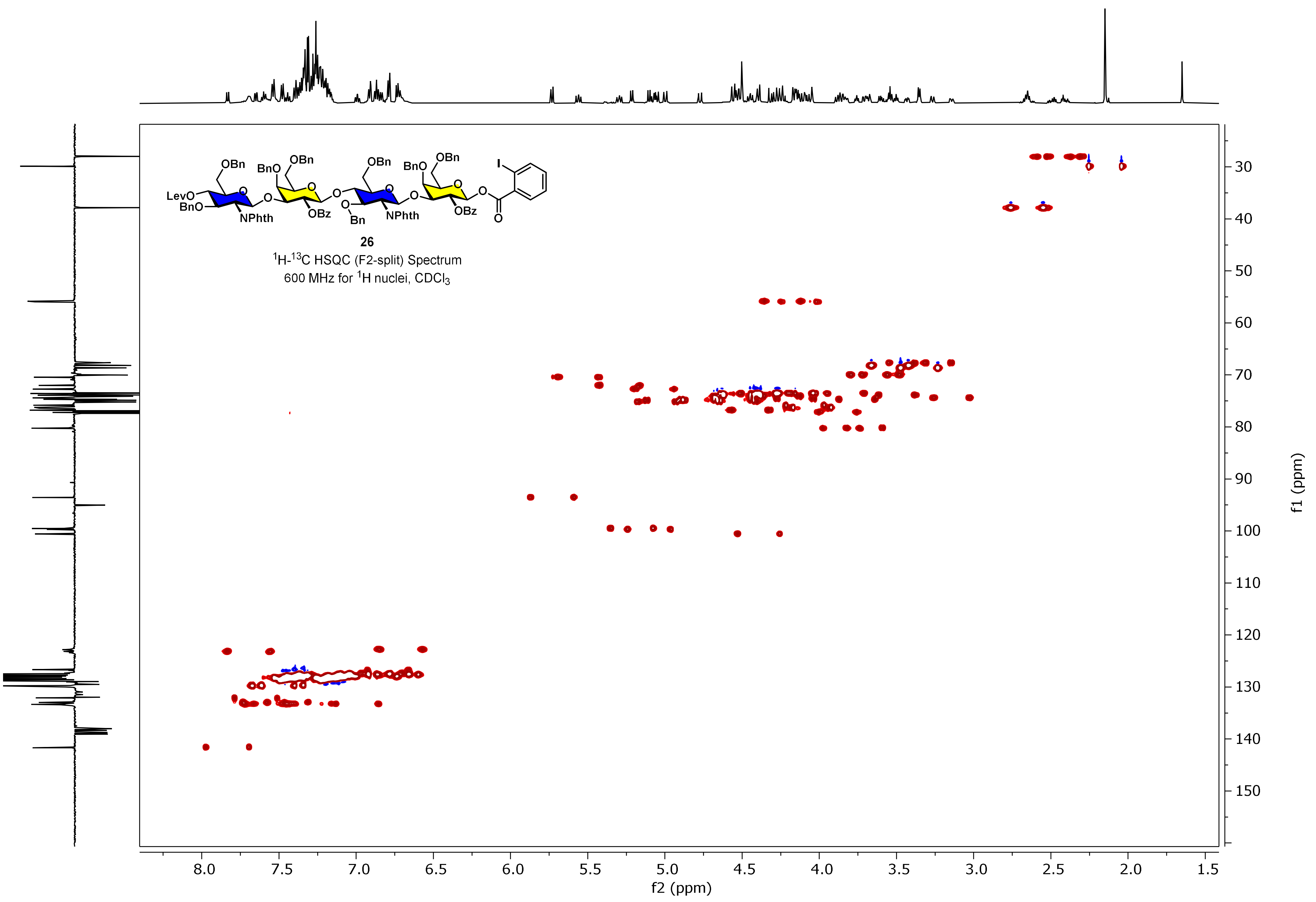
LevO
BnO
OBn
BnO
OBn
NPhth
OBz
O
Bn
NPhth
OBz
I
26
1H-13C HSQC (F2-split) Spectrum
600 MHz for 1H nuclei, CDCl3
f2 (ppm)
f1 (ppm)

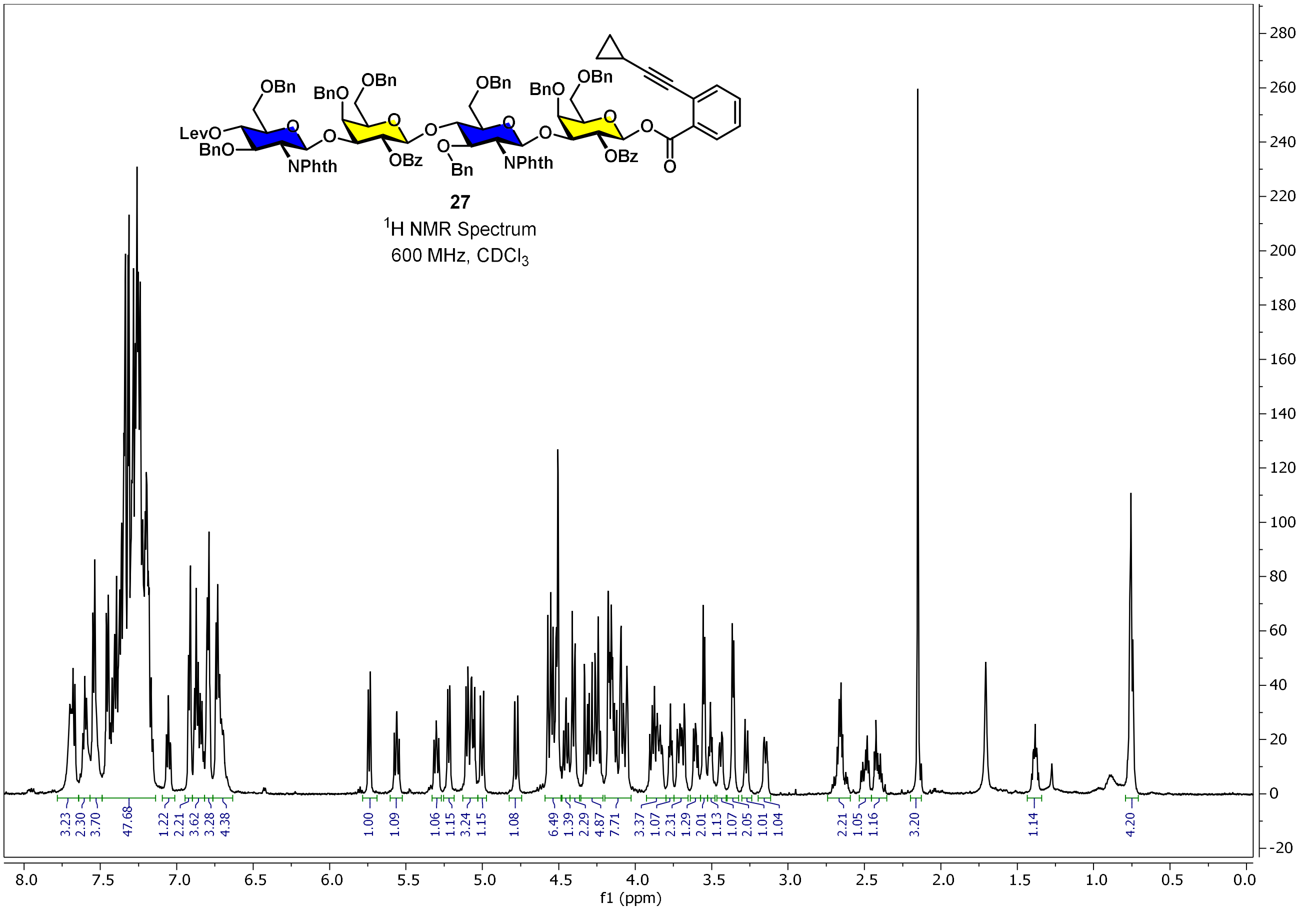
OBn
BnO
OBn
OBn
BnO
OBn
LevO
BnO
NPhth
OBz
O
Bn
NPhth
OBz
O
27
^{1}H NMR Spectrum
600 MHz, $CDCl_3$
3.23
2.30
3.70
47.68
1.22
2.21
3.62
3.28
4.38
1.00
1.09
1.06
1.15
3.24
1.15
1.08
6.49
1.39
2.29
4.87
7.71
3.37
1.07
2.31
1.29
2.01
1.13
1.07
2.05
1.01
1.04
2.21
1.05
1.16
3.20
1.14
4.20
f1 (ppm)

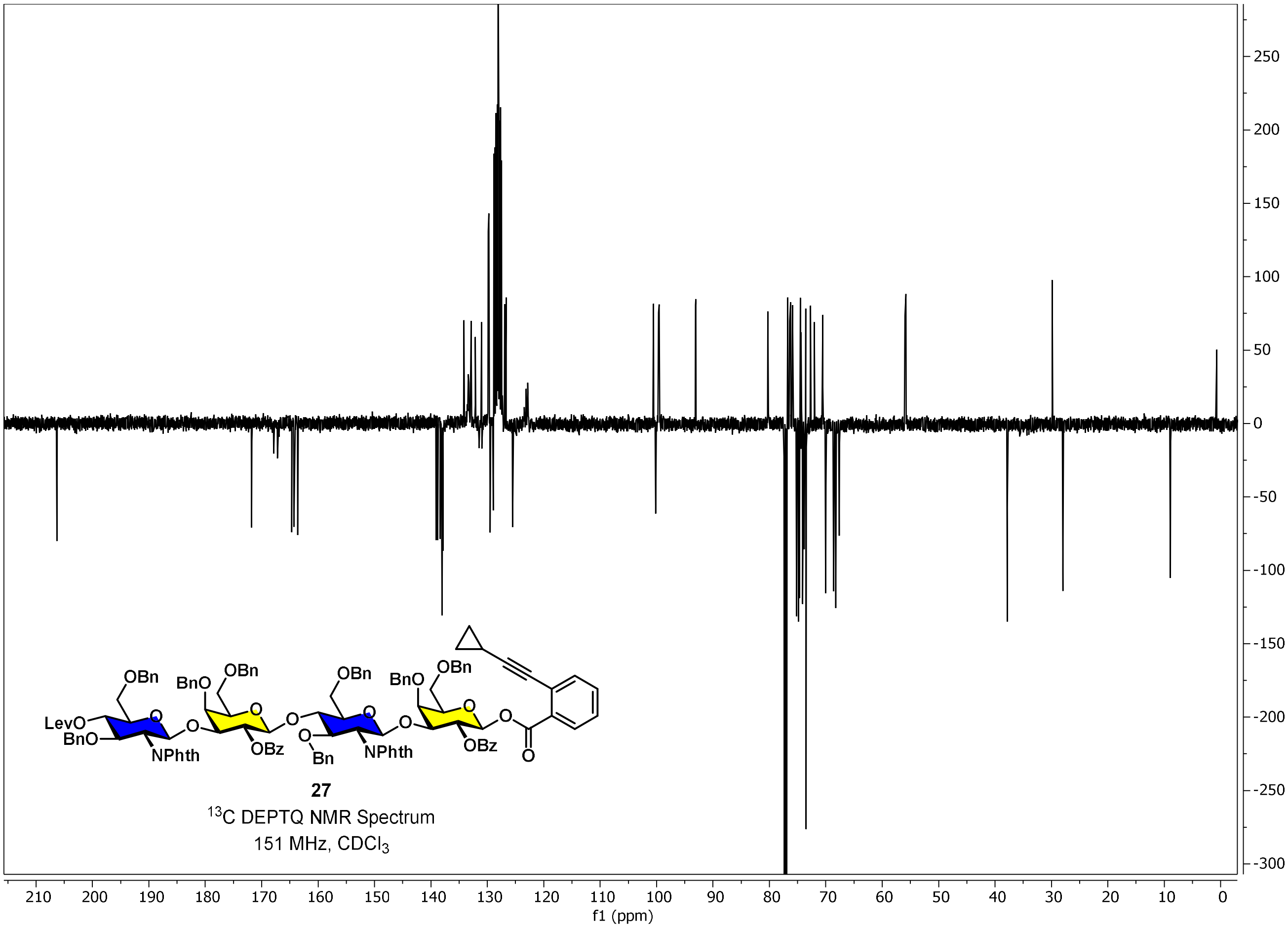
OBn
BnO
OBn
OBn
BnO
OBn
LevO
BnO
NPhth
OBz
O
Bn
NPhth
OBz
O
27
^{13}C DEPTQ NMR Spectrum
151 MHz, $CDCl_3$
f1 (ppm)

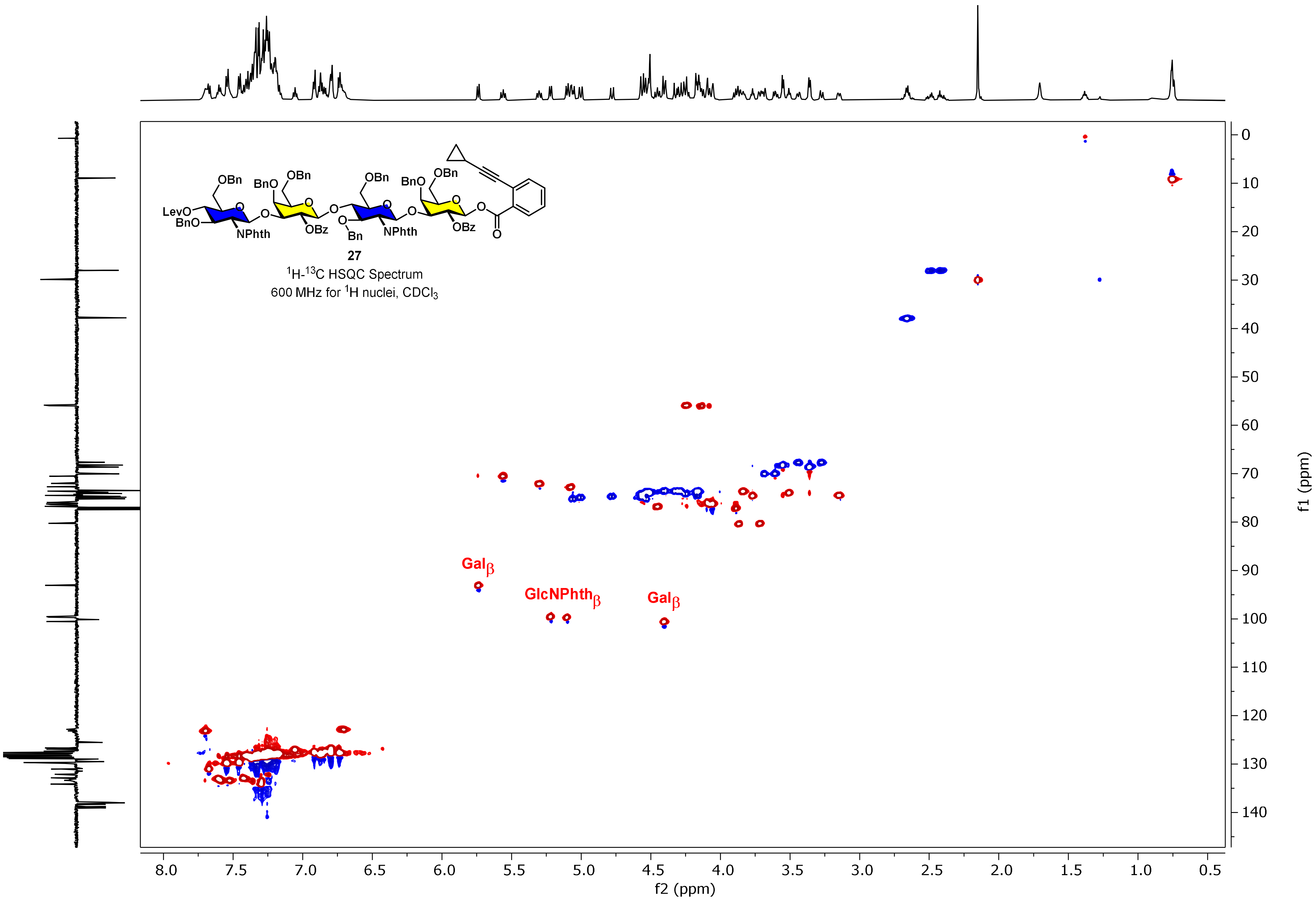

OBn
BnO
OBn
OBn
BnO
OBn
LevO
BnO
O
NPhth
OBz
Bn
NPhth
OBz
27
^{1}H-^{13}C HSQC Spectrum
600 MHz for ^{1}H nuclei, CDCl$_3$
Gal$_\beta$
GlcNPhth$_\beta$
Gal$_\beta$
f2 (ppm)
f1 (ppm)

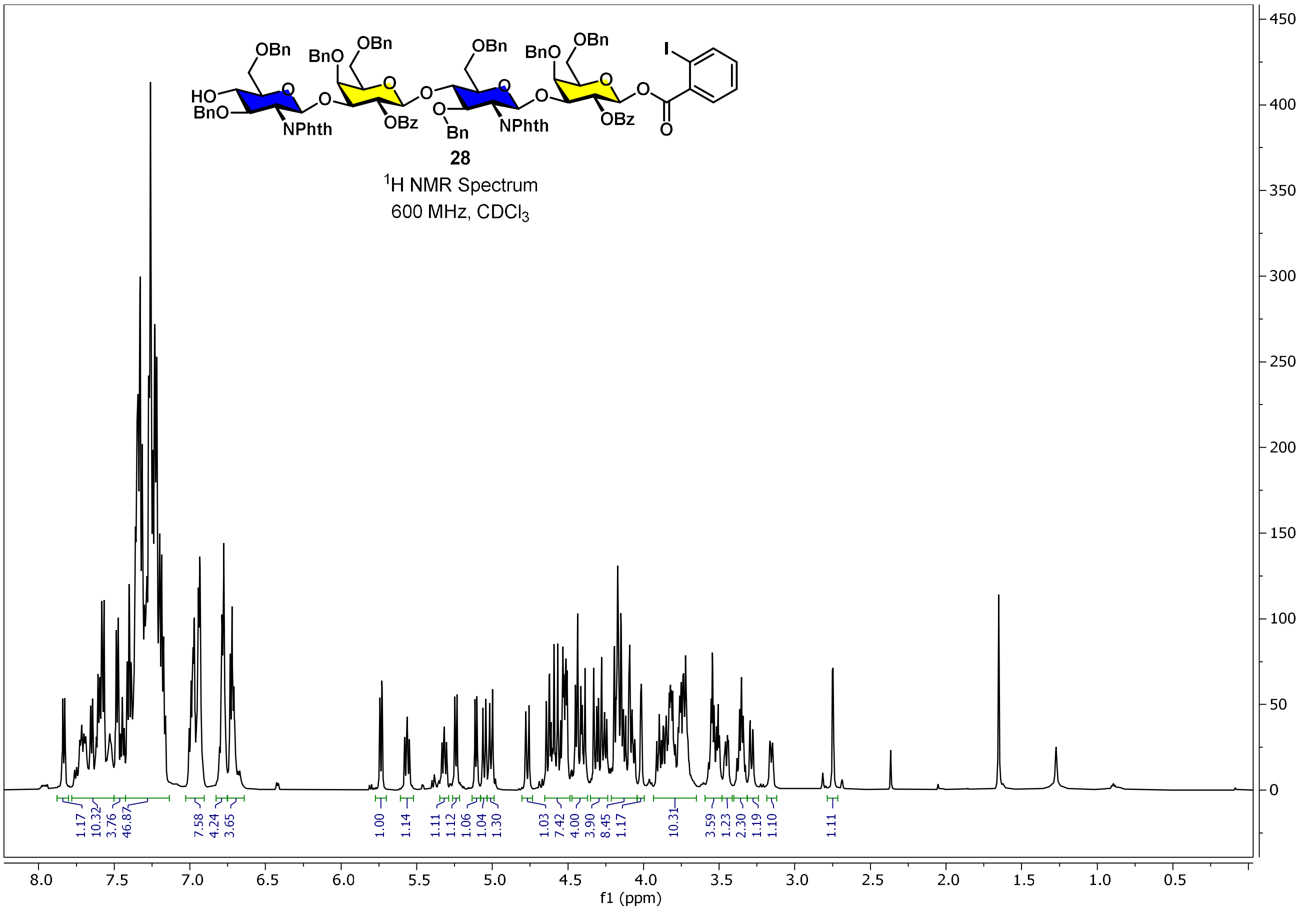
28
^{1}H NMR Spectrum
600 MHz, $CDCl_3$
f1 (ppm)

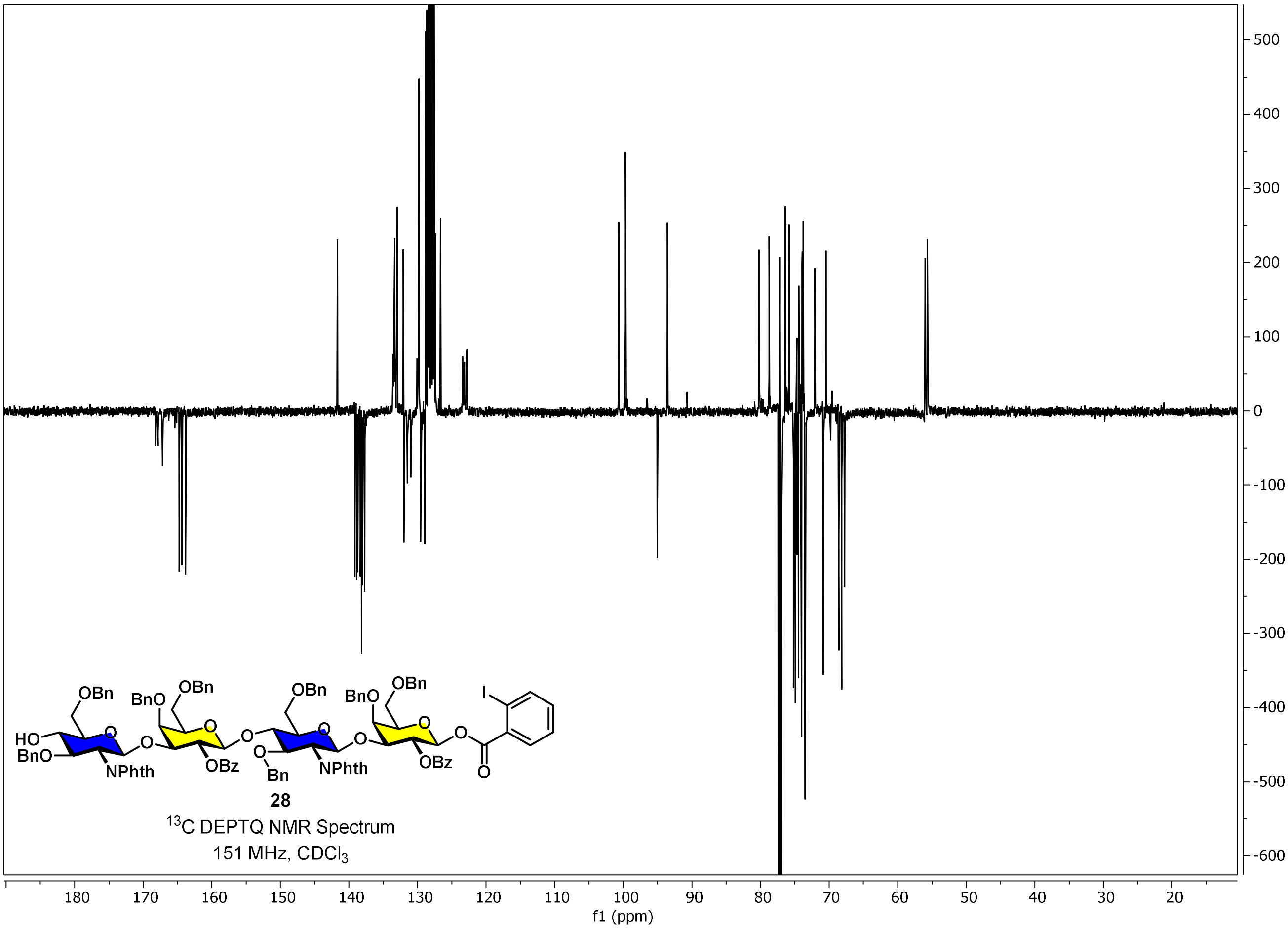
28
^{13}C DEPTQ NMR Spectrum
151 MHz, $CDCl_3$
f1 (ppm)

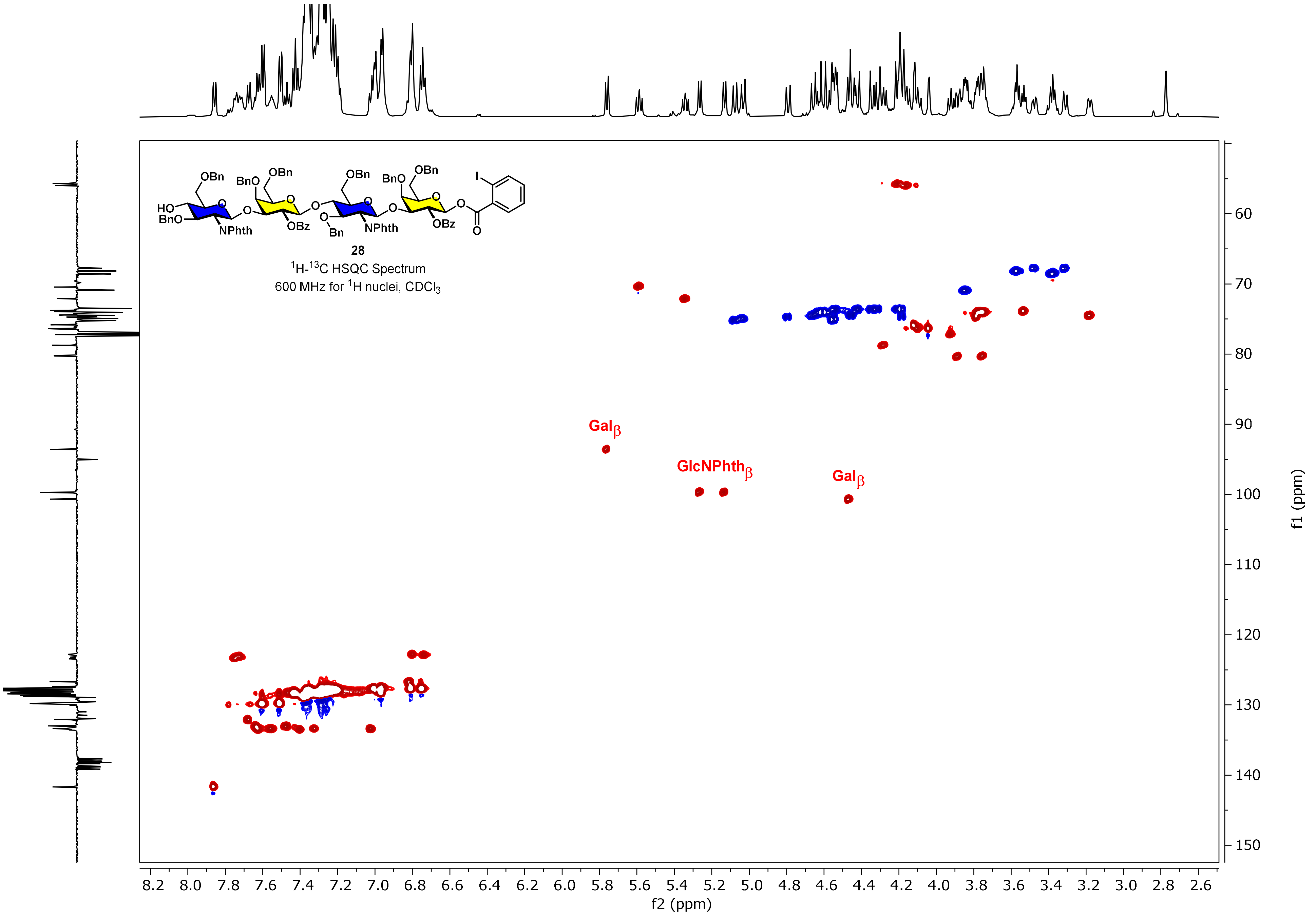

OBn
BnO
OBn
OBn
BnO
OBn
I
HO
BnO
O
O
O
O
NPhth
OBz
Bn
NPhth
OBz
O
28
1H-13C HSQC Spectrum
600 MHz for 1H nuclei, CDCl3
Galβ
GlcNPhthβ
Galβ
f2 (ppm)
f1 (ppm)

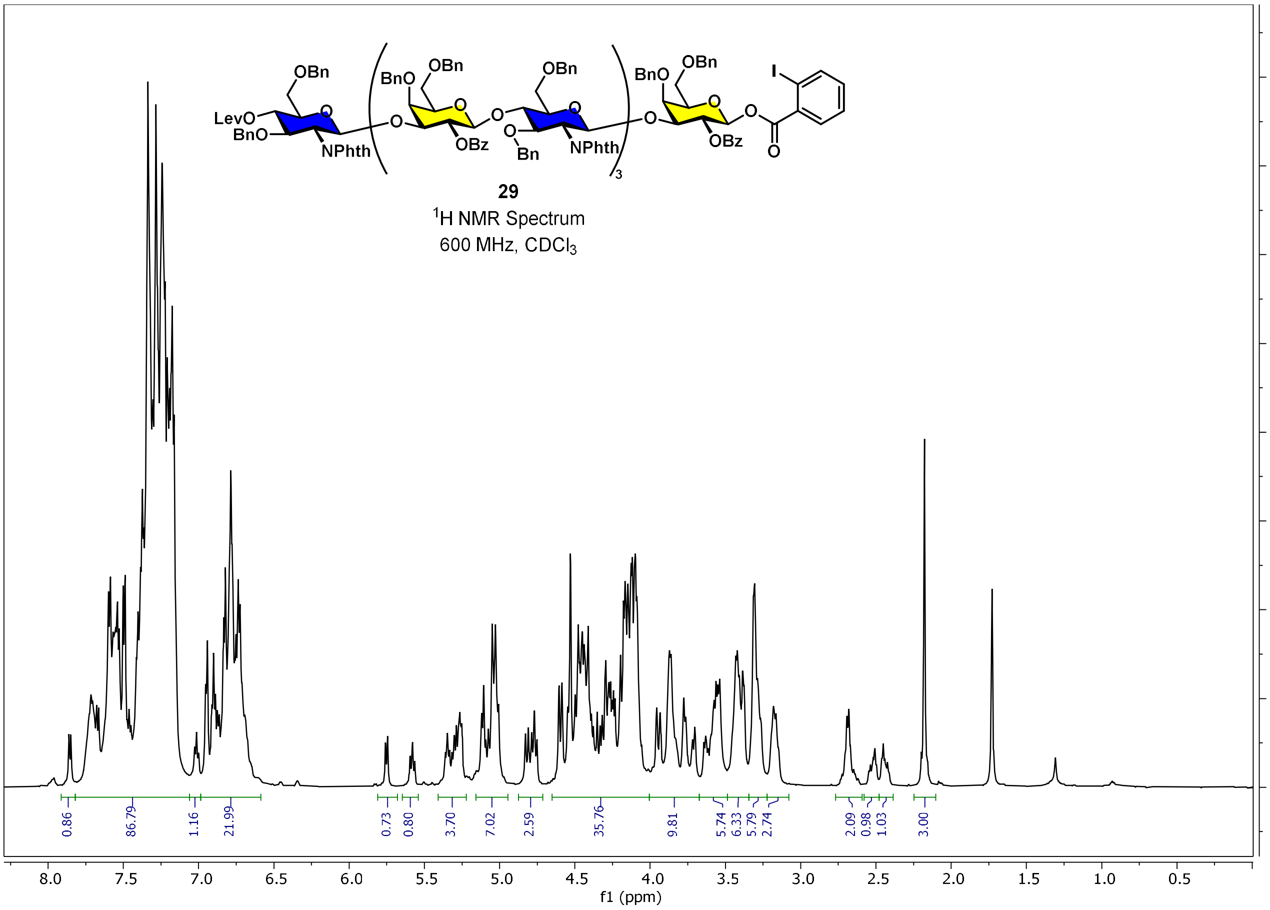

LevO
BnO
OBn
NPhth
BnO
OBn
OBz
O
Bn
OBn
NPhth
3
BnO
OBn
OBz
I
O
29
1H NMR Spectrum
600 MHz, CDCl3
0.86
86.79
1.16
21.99
0.73
0.80
3.70
7.02
2.59
35.76
9.81
5.74
6.33
5.79
2.74
2.09
0.98
1.03
3.00
f1 (ppm)

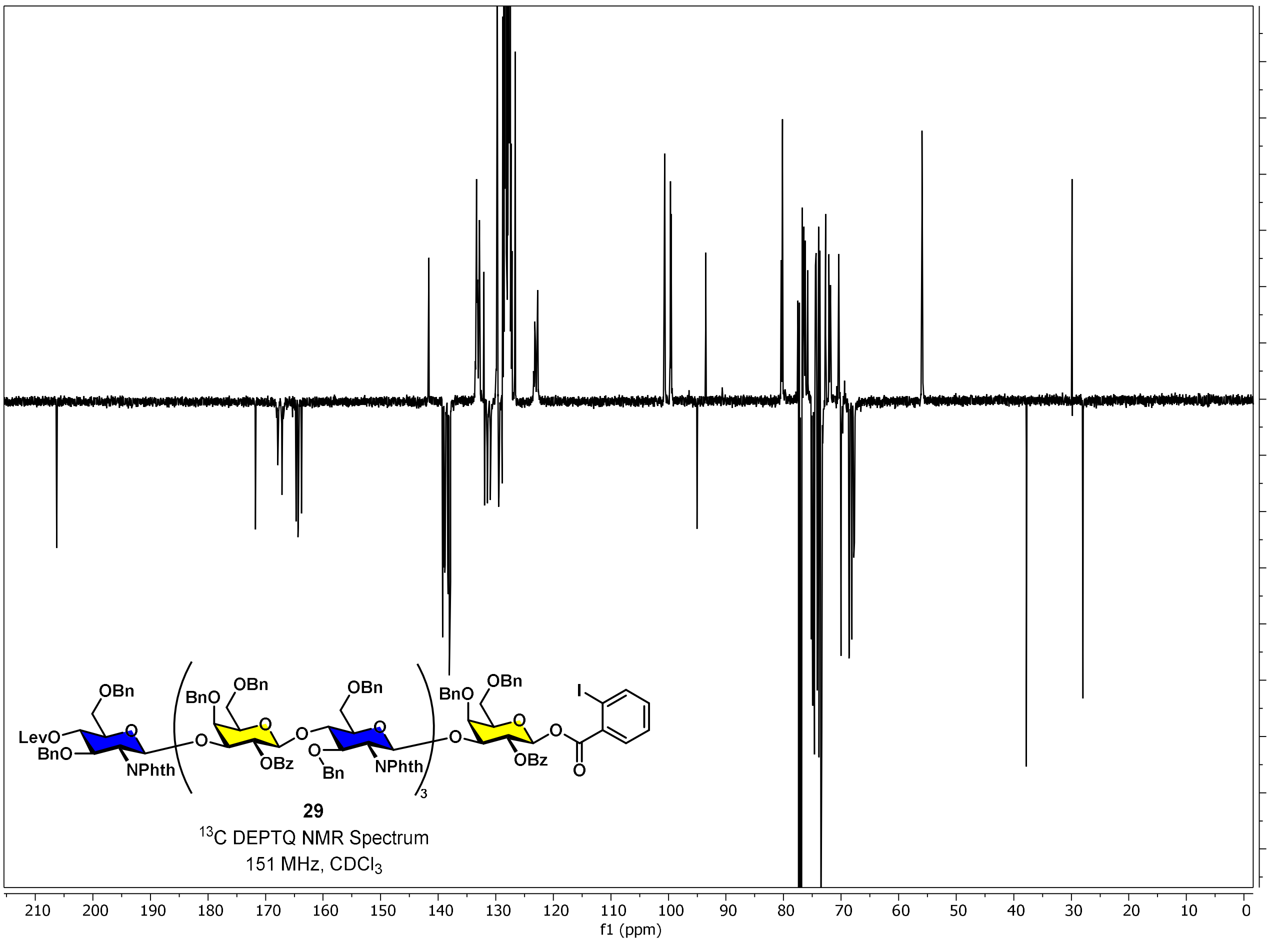

LevO
BnO
OBn
NPhth
BnO
OBn
OBz
O
Bn
OBn
NPhth
3
BnO
OBn
OBz
I
O
29
13C DEPTQ NMR Spectrum
151 MHz, CDCl3
f1 (ppm)

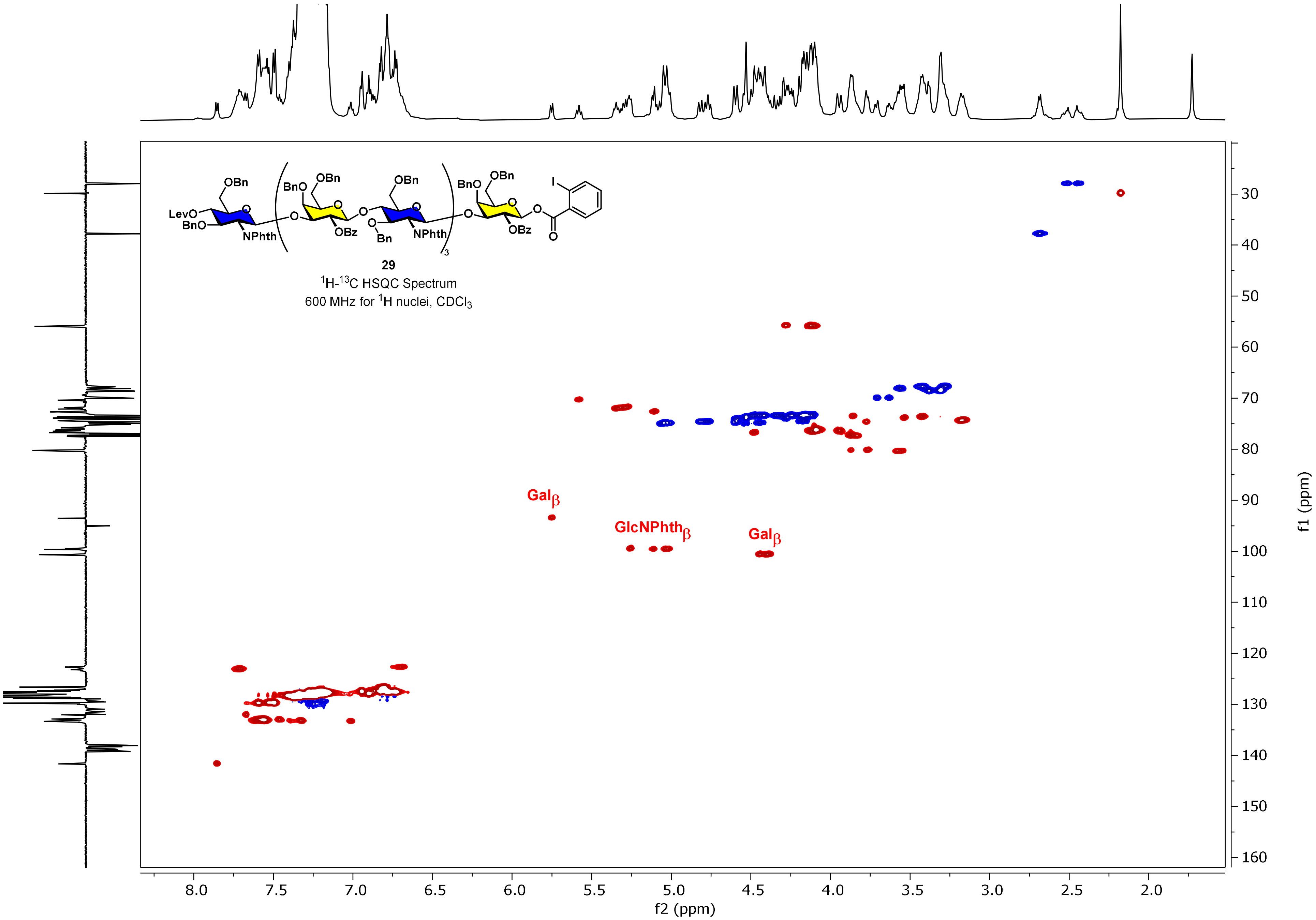
LevO
BnO
OBn
NPhth
BnO
OBn
OBz
O
Bn
OBn
NPhth
3
BnO
OBn
OBz
I
29
^{1}H-^{13}C HSQC Spectrum
600 MHz for ^{1}H nuclei, CDCl$_3$
Gal$_\beta$
GlcNPhth$_\beta$
Gal$_\beta$
f2 (ppm)
f1 (ppm)

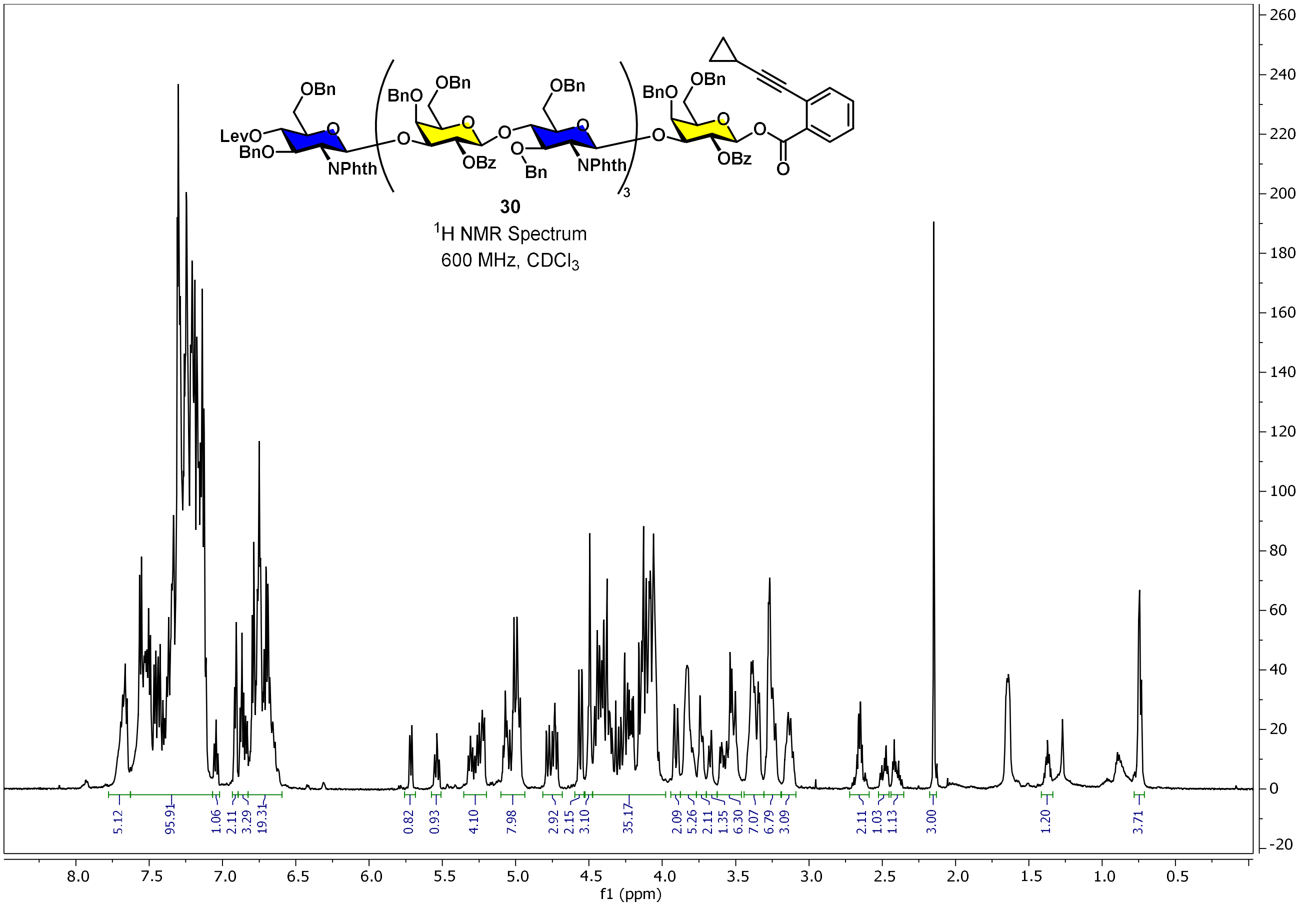

OBn
LevO
BnO
NPhth
BnO
OBn
OBz
O
Bn
OBn
NPhth
3
BnO
OBn
OBz
30
^{1}H NMR Spectrum
600 MHz, $CDCl_3$
5.12
95.91
1.06
2.11
3.29
19.31
0.82
0.93
4.10
7.98
2.92
2.15
3.10
35.17
2.09
5.26
2.11
1.35
6.30
7.07
6.79
3.09
2.11
1.03
1.13
3.00
1.20
3.71
f1 (ppm)

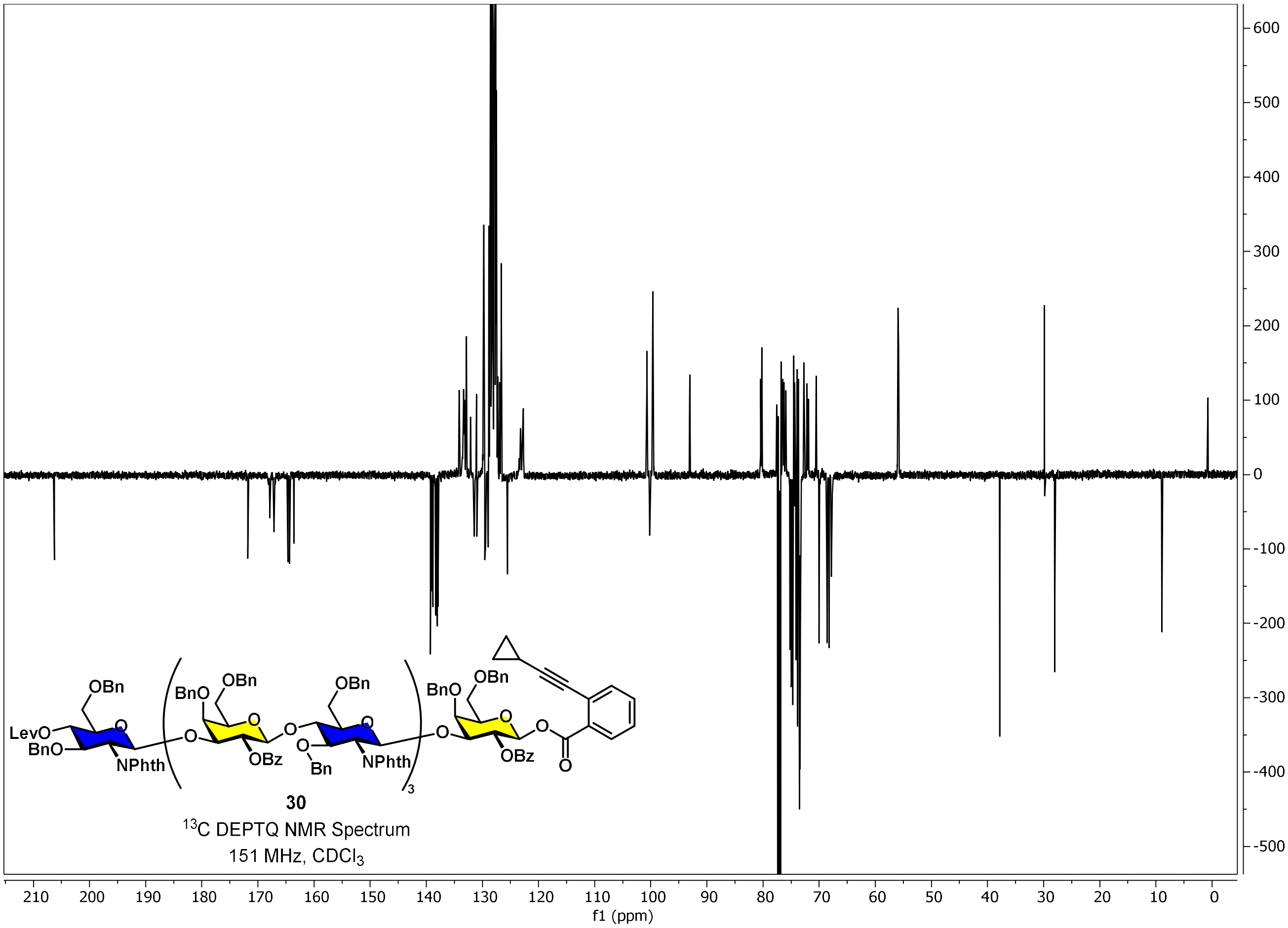

OBn
LevO
BnO
NPhth
BnO
OBn
OBz
O
Bn
OBn
NPhth
3
BnO
OBn
OBz
30
^{13}C DEPTQ NMR Spectrum
151 MHz, $CDCl_3$
f1 (ppm)

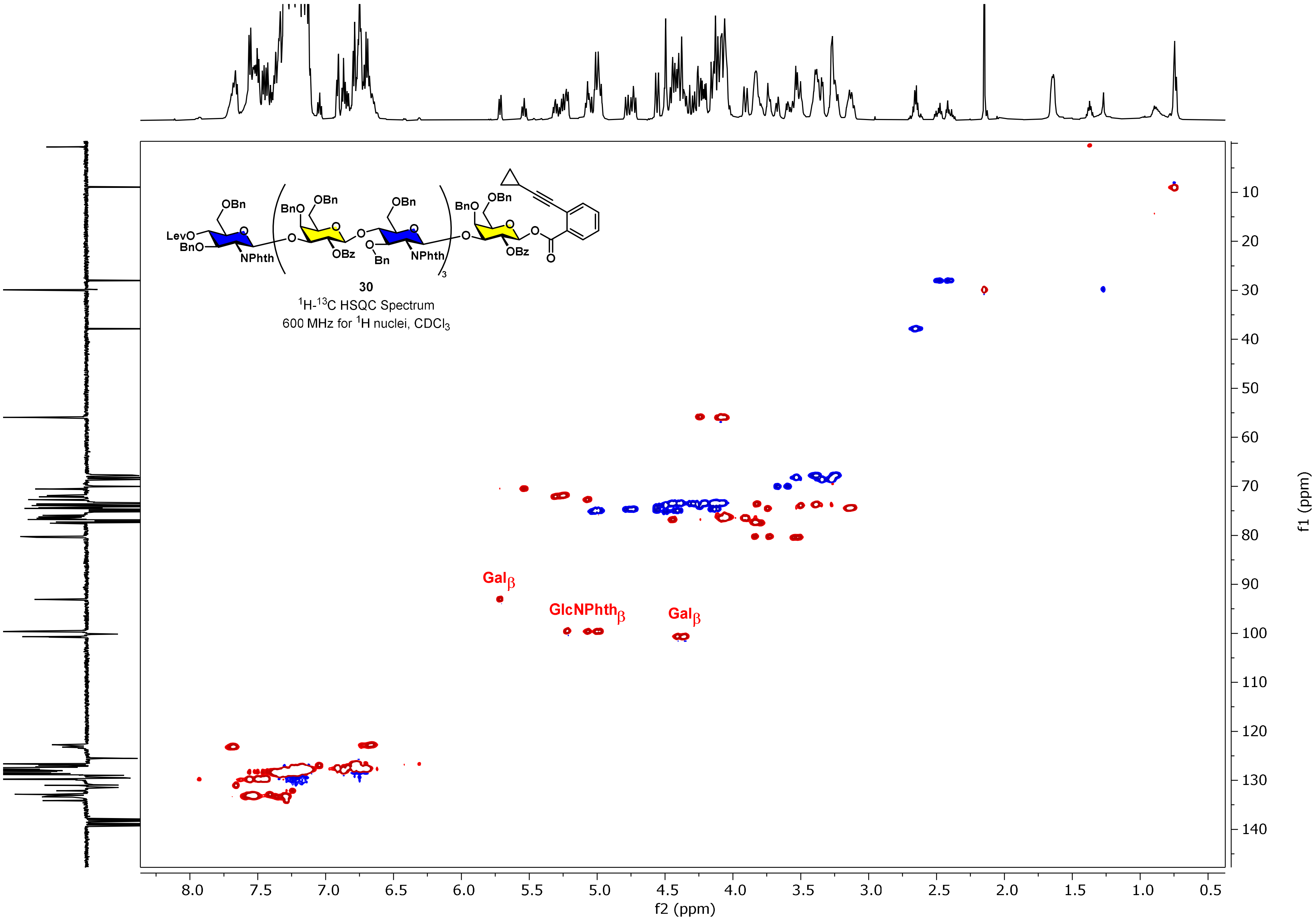

LevO
BnO
OBn
NPhth
BnO
OBn
OBz
O
Bn
OBn
NPhth
3
BnO
OBn
OBz
O
30
^{1}H-^{13}C HSQC Spectrum
600 MHz for ^{1}H nuclei, $CDCl_3$
Gal$_\beta$
GlcNPhth$_\beta$
Gal$_\beta$
f2 (ppm)
f1 (ppm)

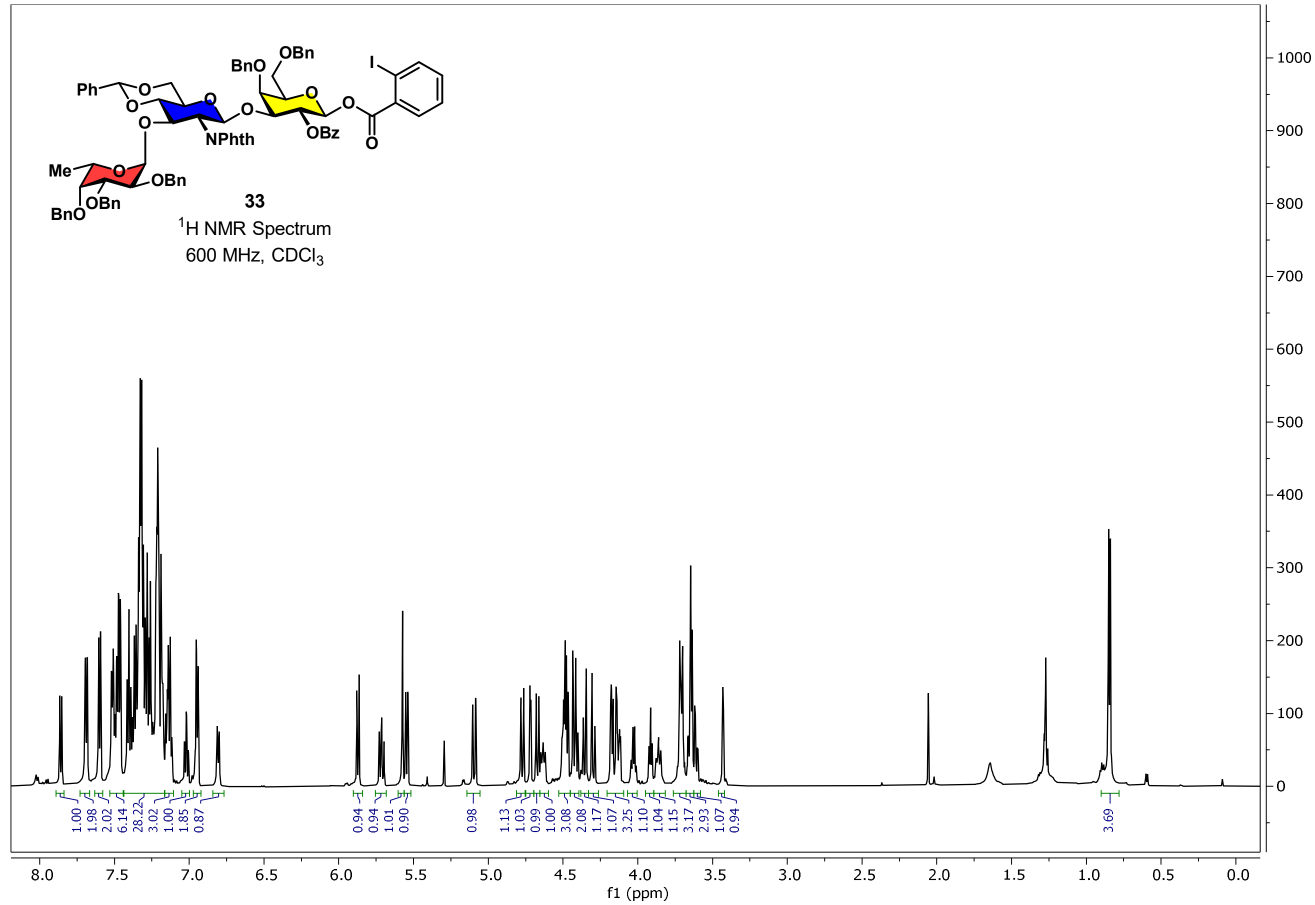

33
^{1}H NMR Spectrum
600 MHz, CDCl$_3$
f1 (ppm)

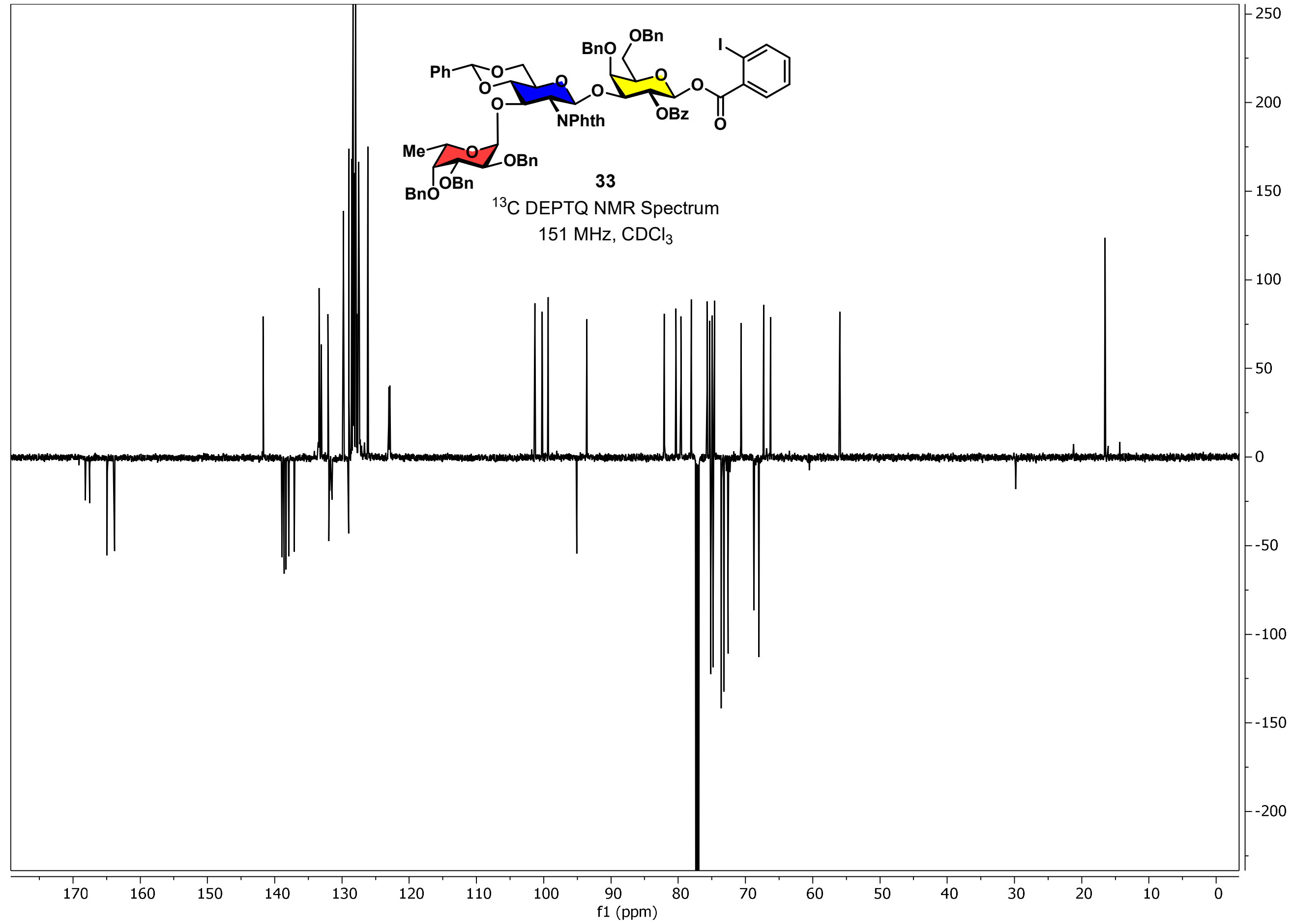

33
^{13}C DEPTQ NMR Spectrum
151 MHz, CDCl$_3$
f1 (ppm)

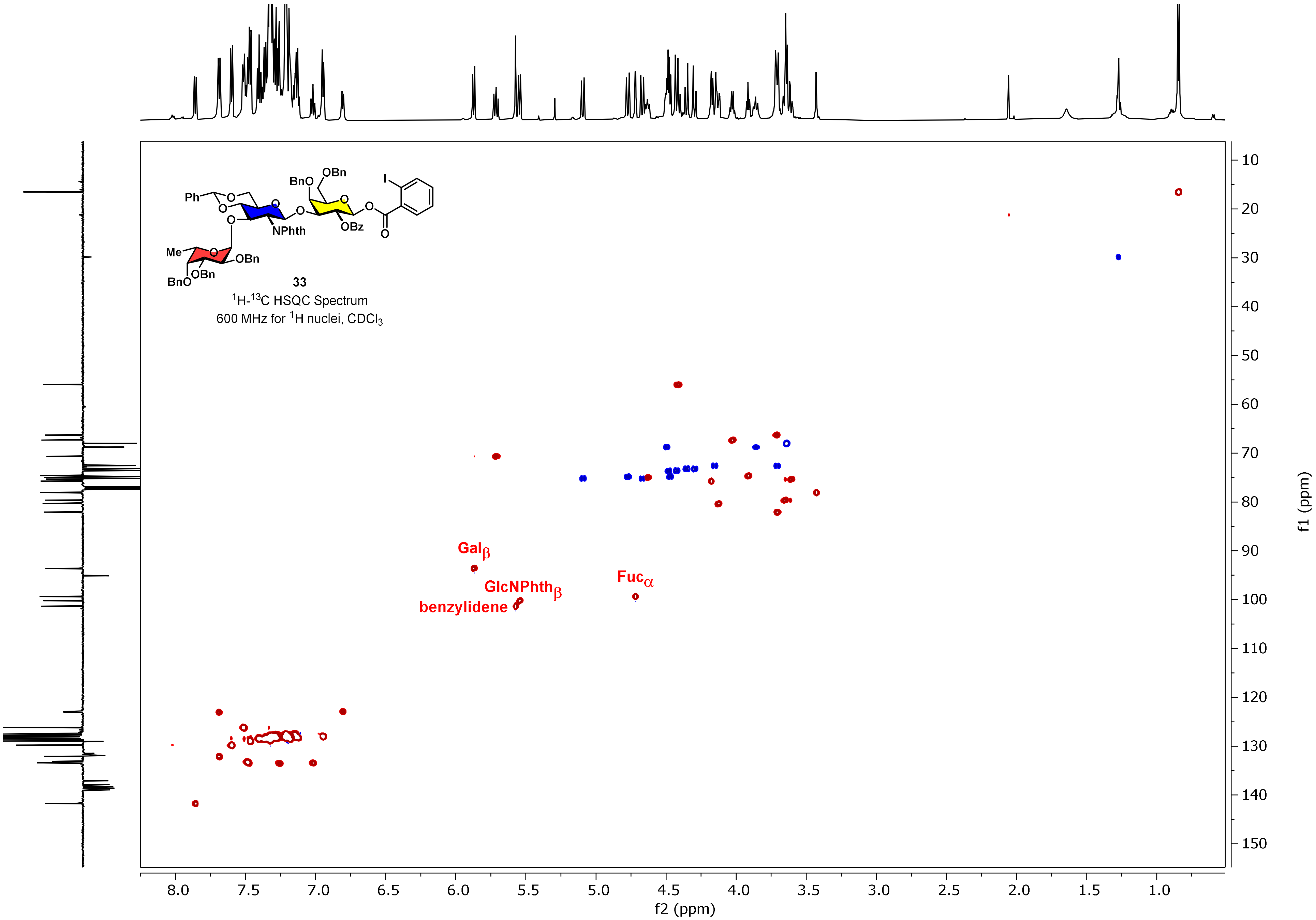

33
^{1}H-^{13}C HSQC Spectrum
600 MHz for ^{1}H nuclei, CDCl$_3$
Gal$_\beta$
GlcNPhth$_\beta$
benzylidene
Fuc$_\alpha$
f2 (ppm)
f1 (ppm)

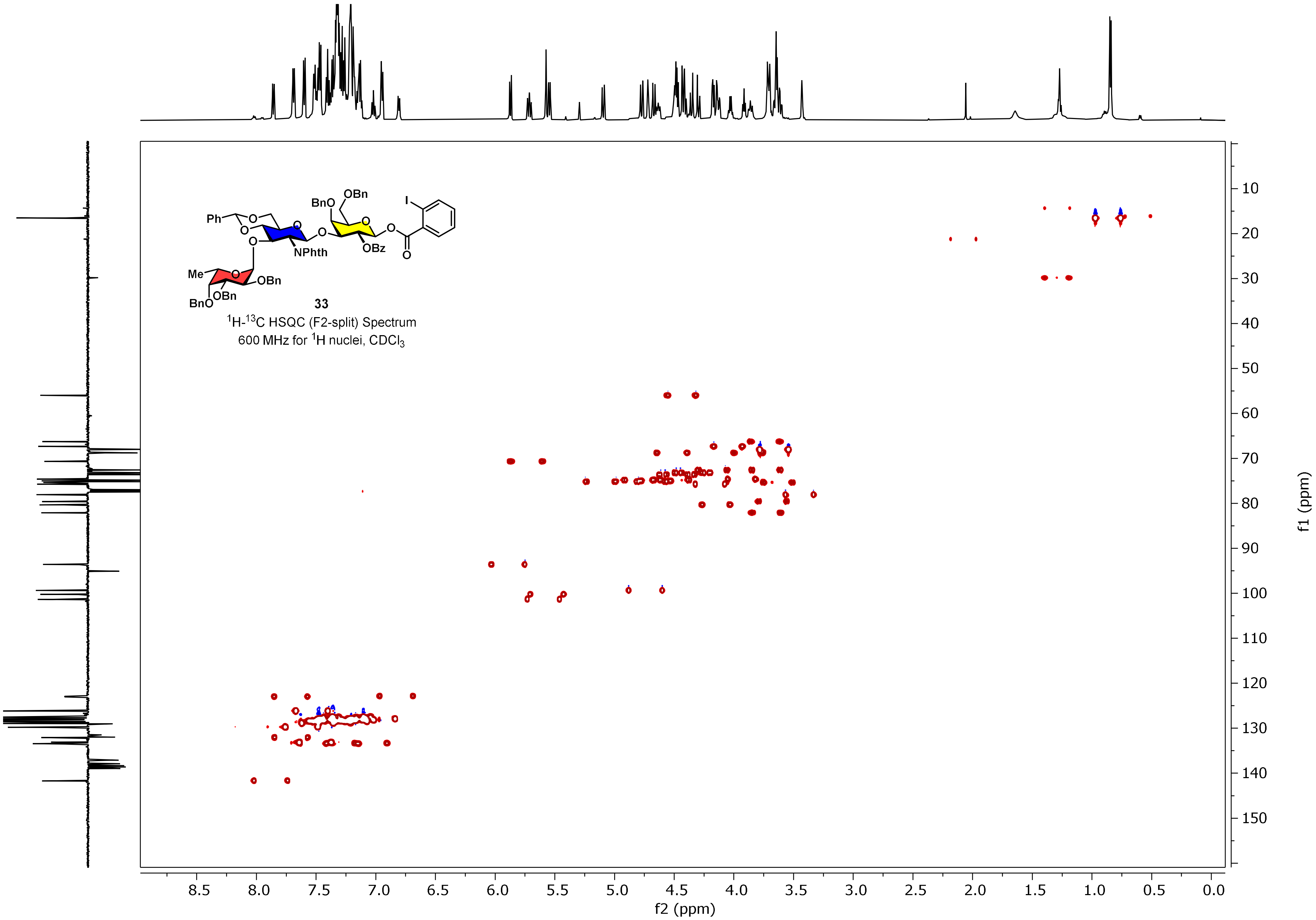

33
^{1}H-^{13}C HSQC (F2-split) Spectrum
600 MHz for ^{1}H nuclei, $CDCl_3$
f2 (ppm)
f1 (ppm)

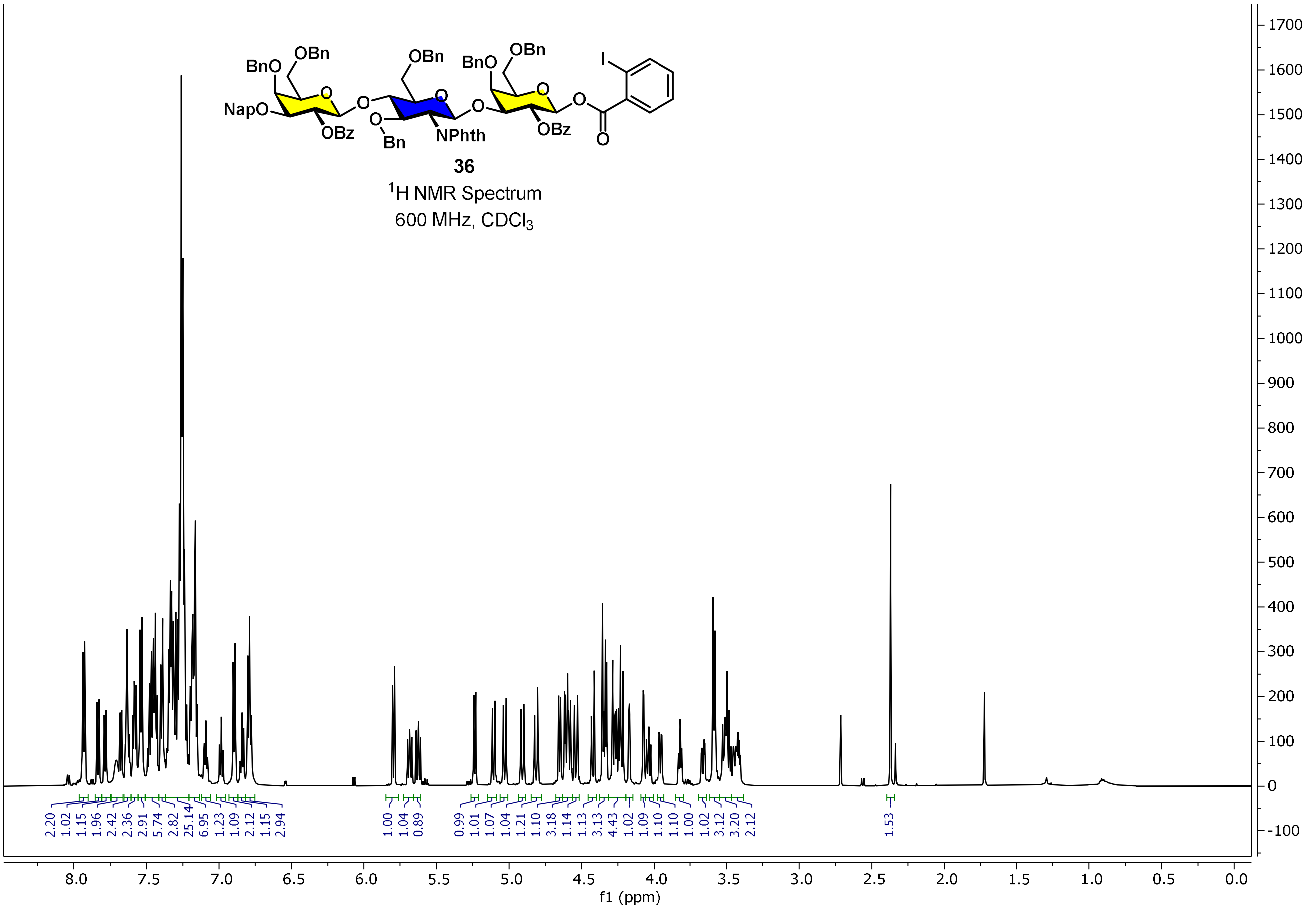
36
^{1}H NMR Spectrum
600 MHz, CDCl$_3$
f1 (ppm)

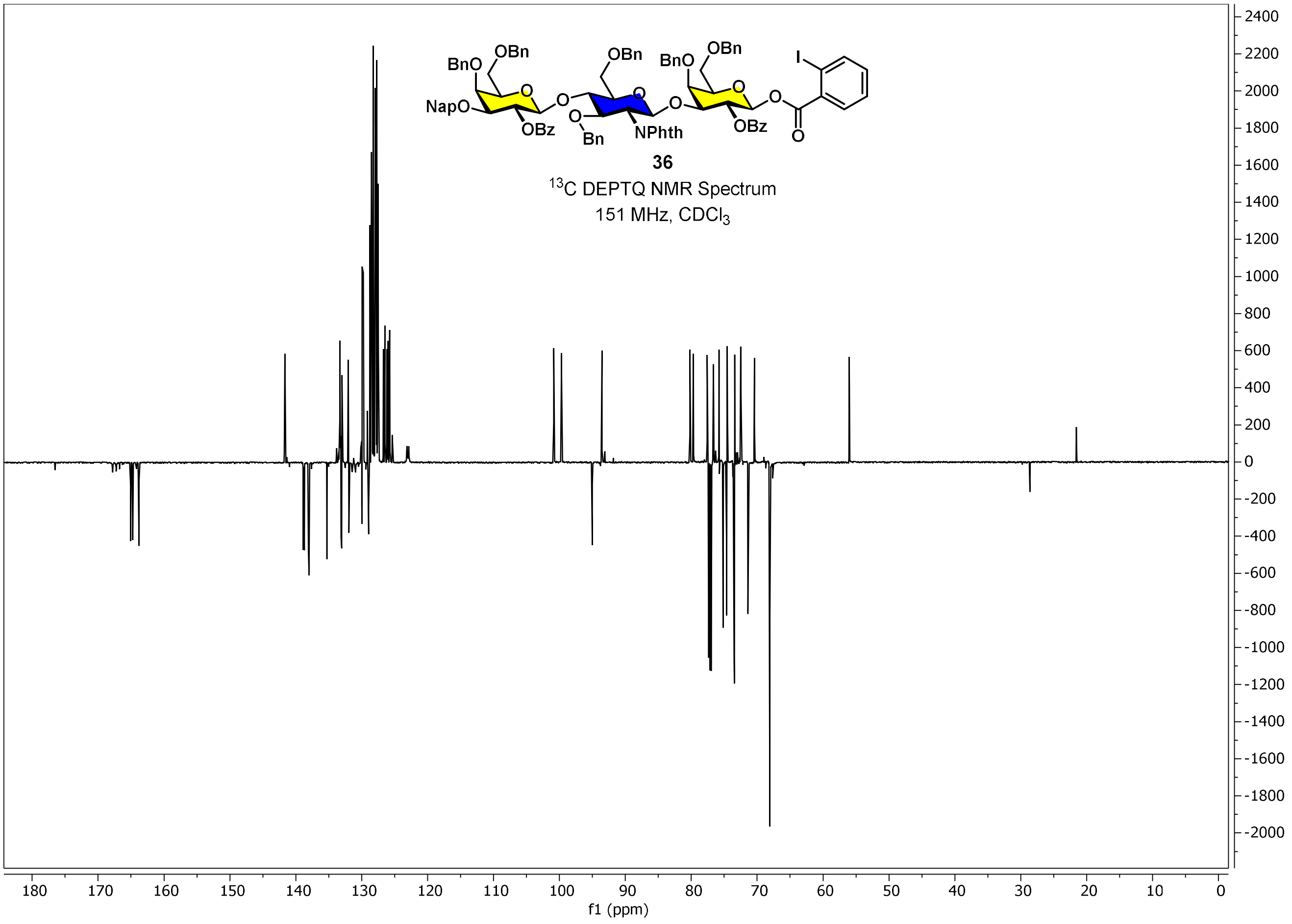
36
^{13}C DEPTQ NMR Spectrum
151 MHz, CDCl$_3$
f1 (ppm)

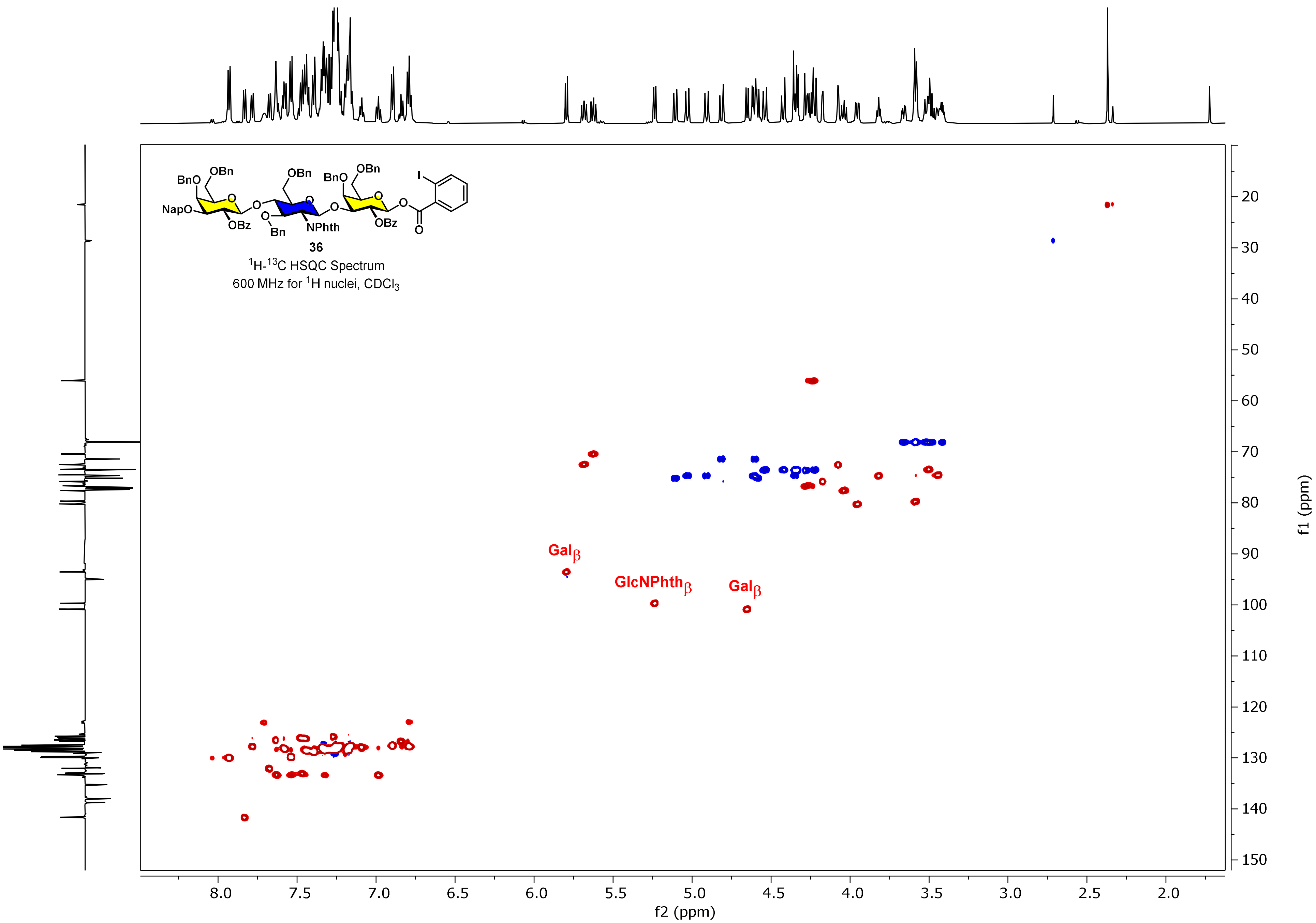
BnO
OBn
NapO
OBz
O
Bn
NPhth
OBz
I
36
^{1}H-^{13}C HSQC Spectrum
600 MHz for ^{1}H nuclei, CDCl$_3$
Gal$_\beta$
GlcNPhth$_\beta$
Gal$_\beta$
f1 (ppm)
f2 (ppm)

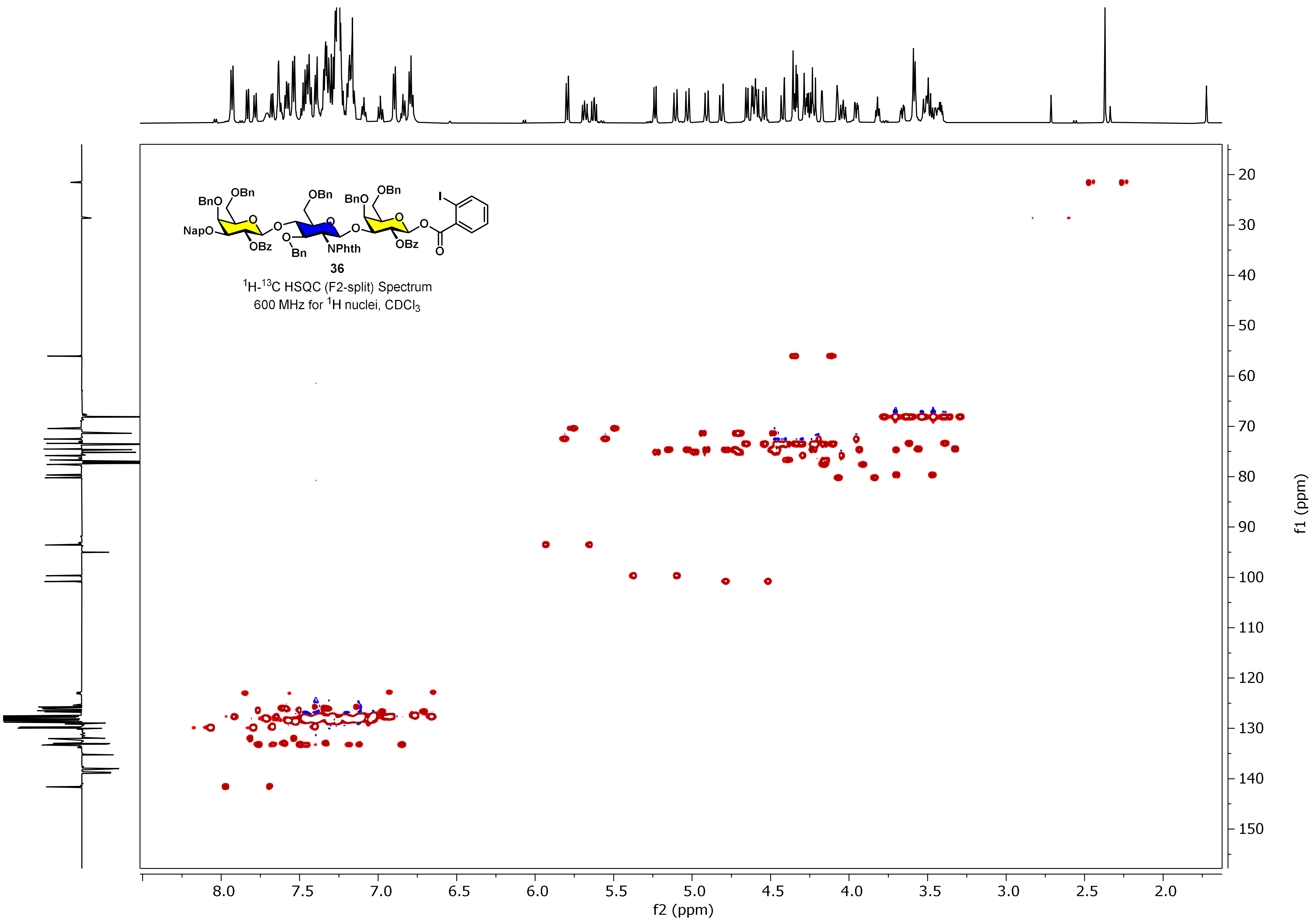

BnO
OBn
NapO
OBz
O
OBn
NPhth
Bn
BnO
OBn
OBz
I
36
^{1}H-^{13}C HSQC (F2-split) Spectrum
600 MHz for ^{1}H nuclei, $CDCl_3$
f2 (ppm)
f1 (ppm)

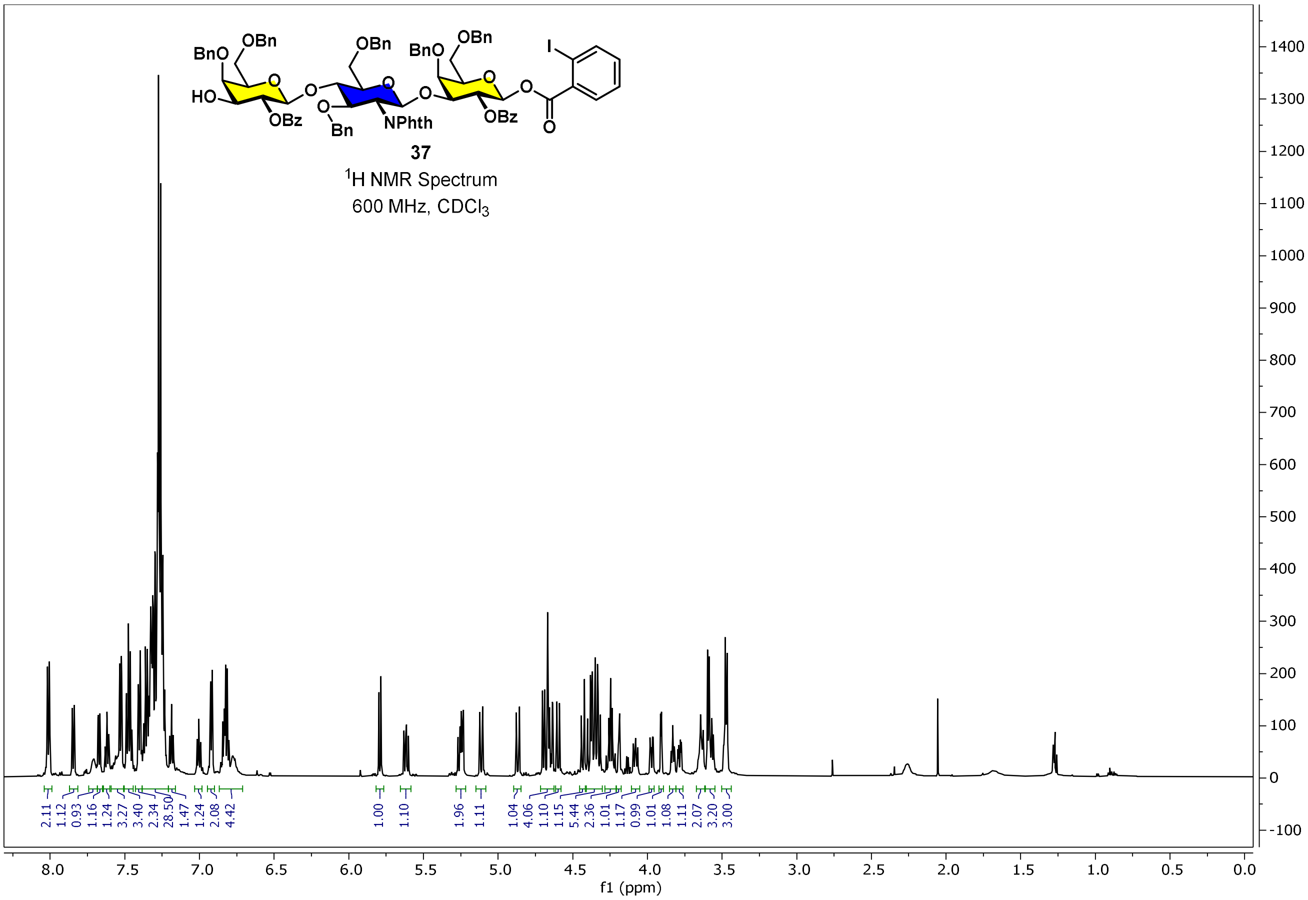

37
^{1}H NMR Spectrum
600 MHz, CDCl$_3$
f1 (ppm)

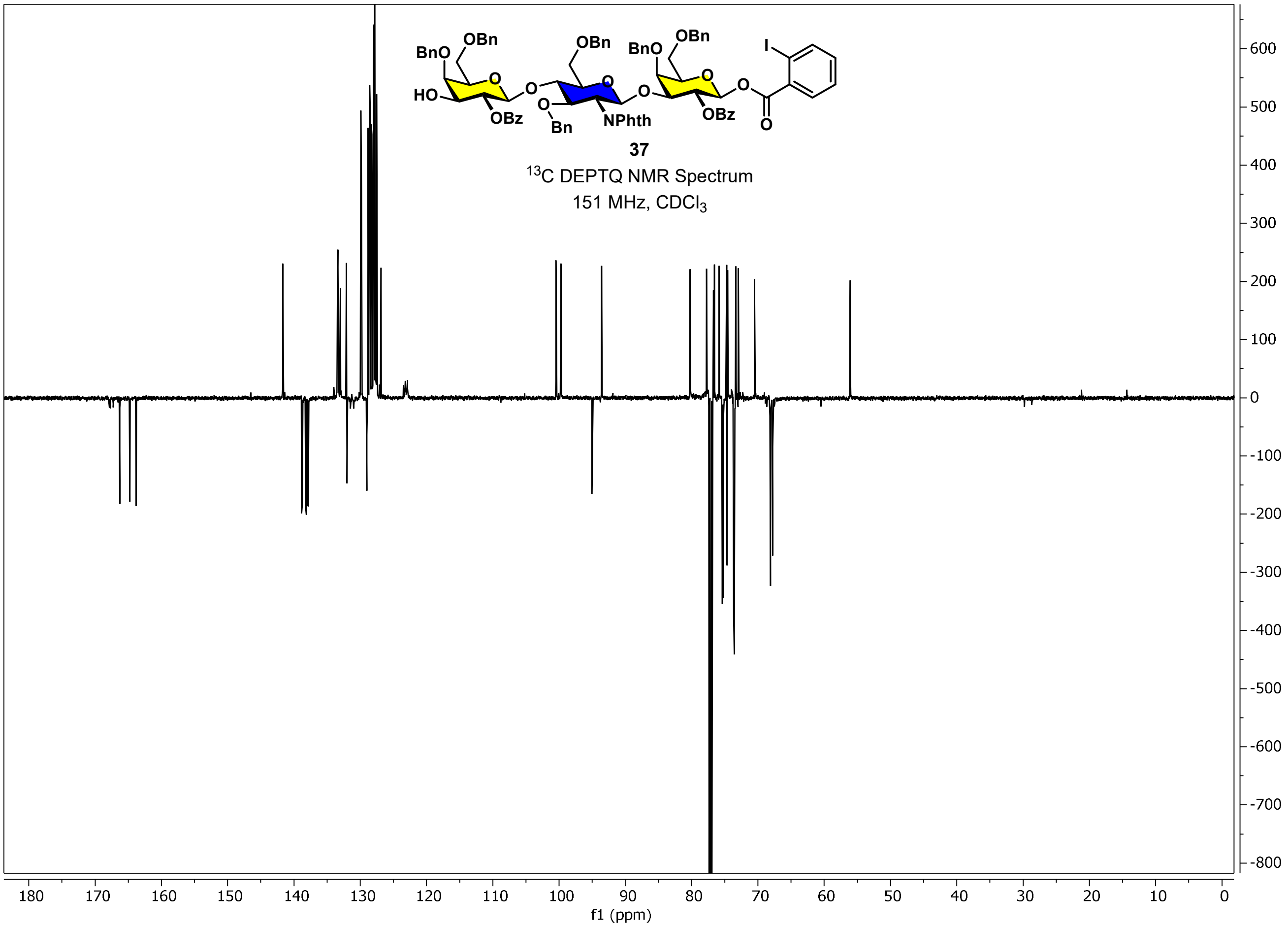

37
^{13}C DEPTQ NMR Spectrum
151 MHz, CDCl$_3$
f1 (ppm)

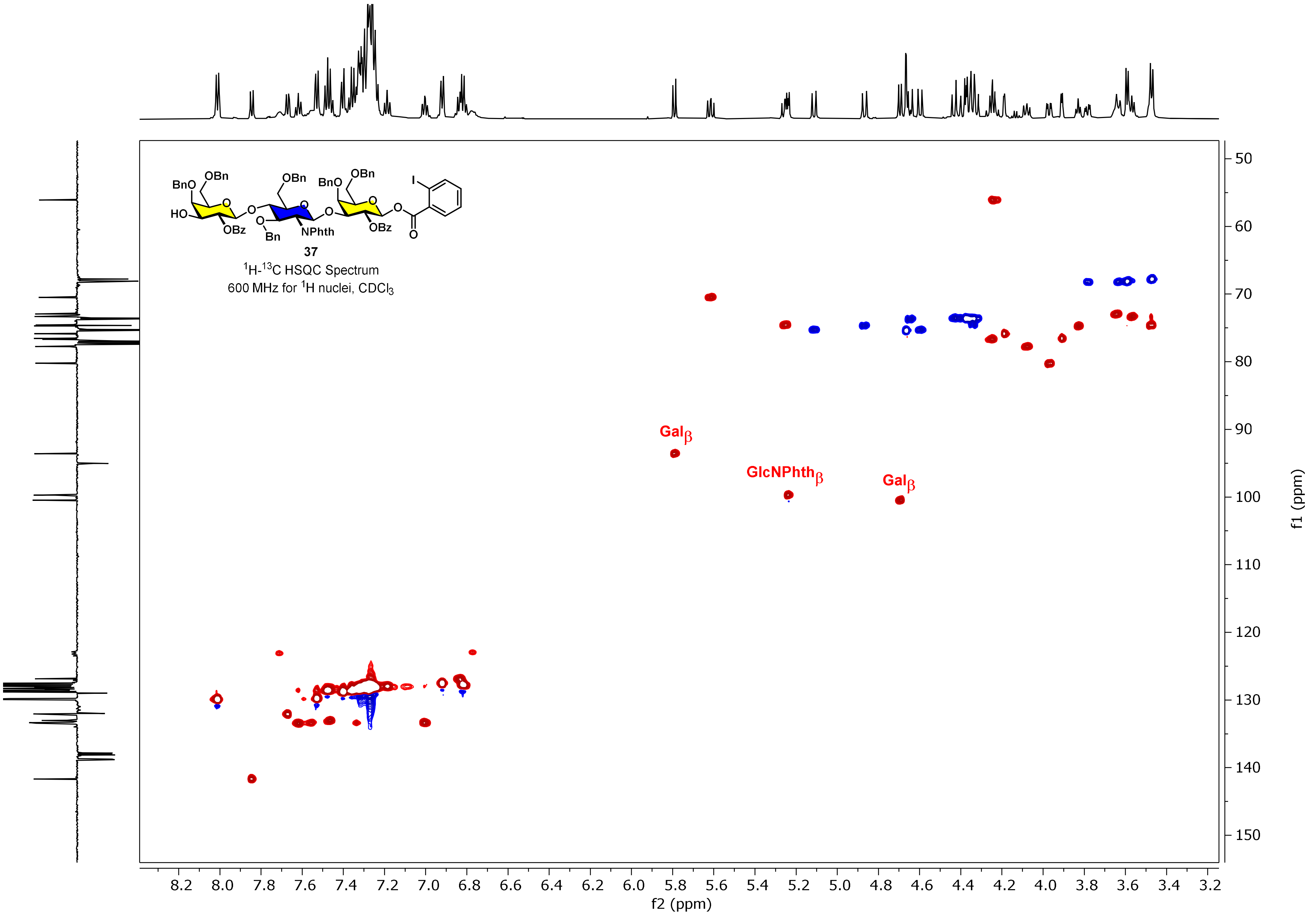

37
^{1}H-^{13}C HSQC Spectrum
600 MHz for ^{1}H nuclei, $CDCl_3$
Galβ
GlcNPhthβ
Galβ
f2 (ppm)
f1 (ppm)

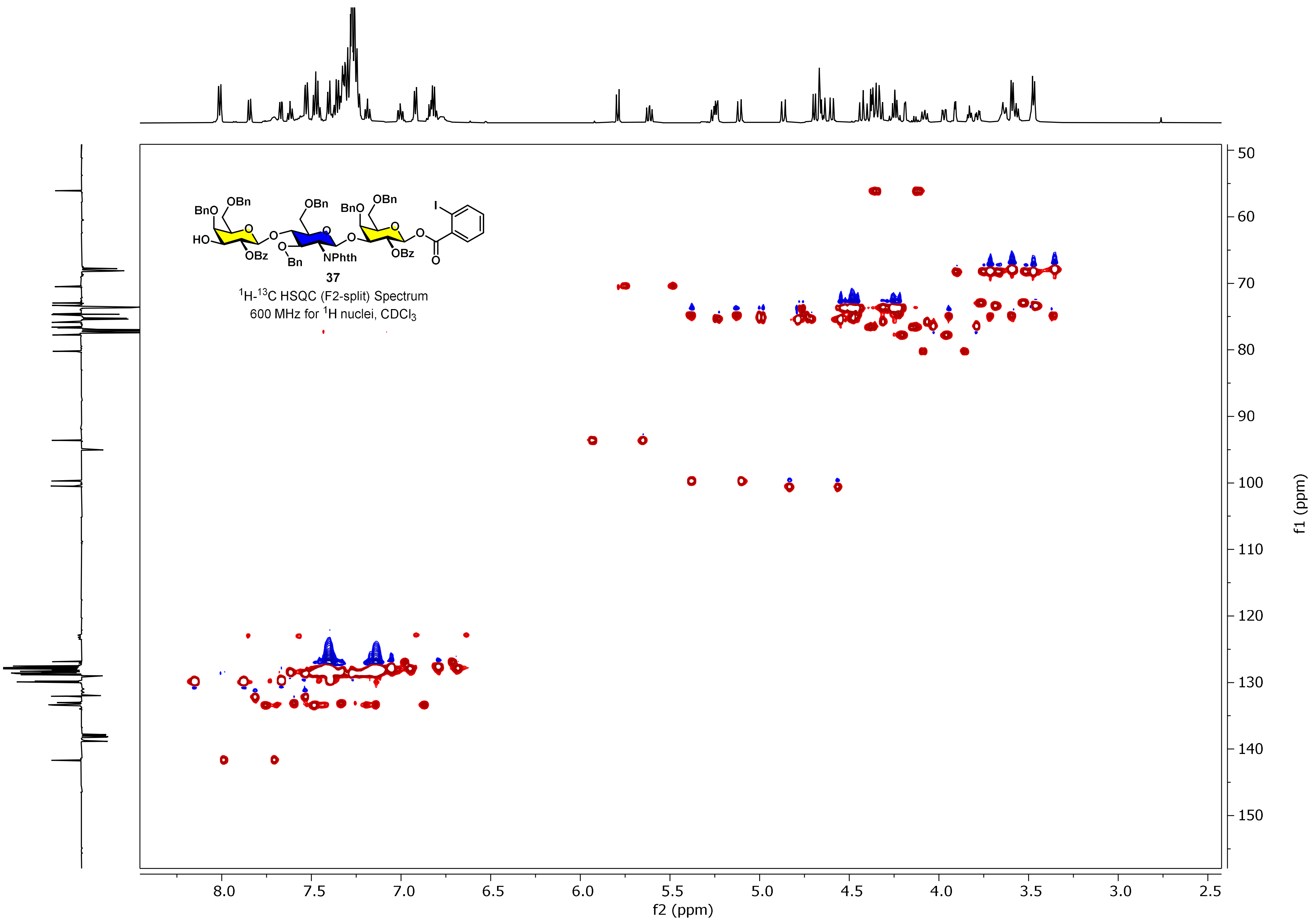

37
^{1}H-^{13}C HSQC (F2-split) Spectrum
600 MHz for ^{1}H nuclei, $CDCl_3$
f2 (ppm)
f1 (ppm)

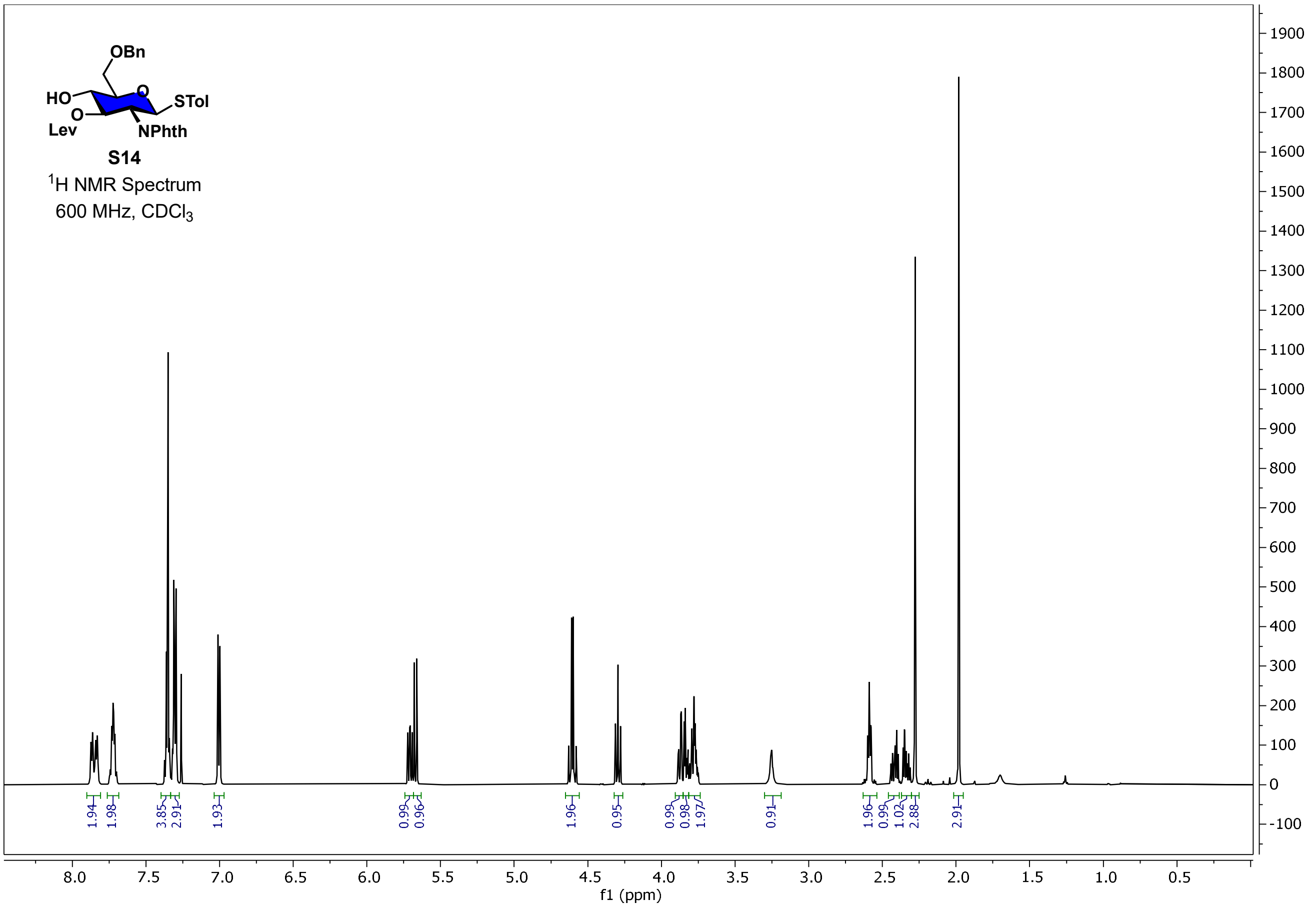
OBn
HO
O
STol
O
Lev
NPhth
S14
^{1}H NMR Spectrum
600 MHz, $CDCl_3$
f1 (ppm)

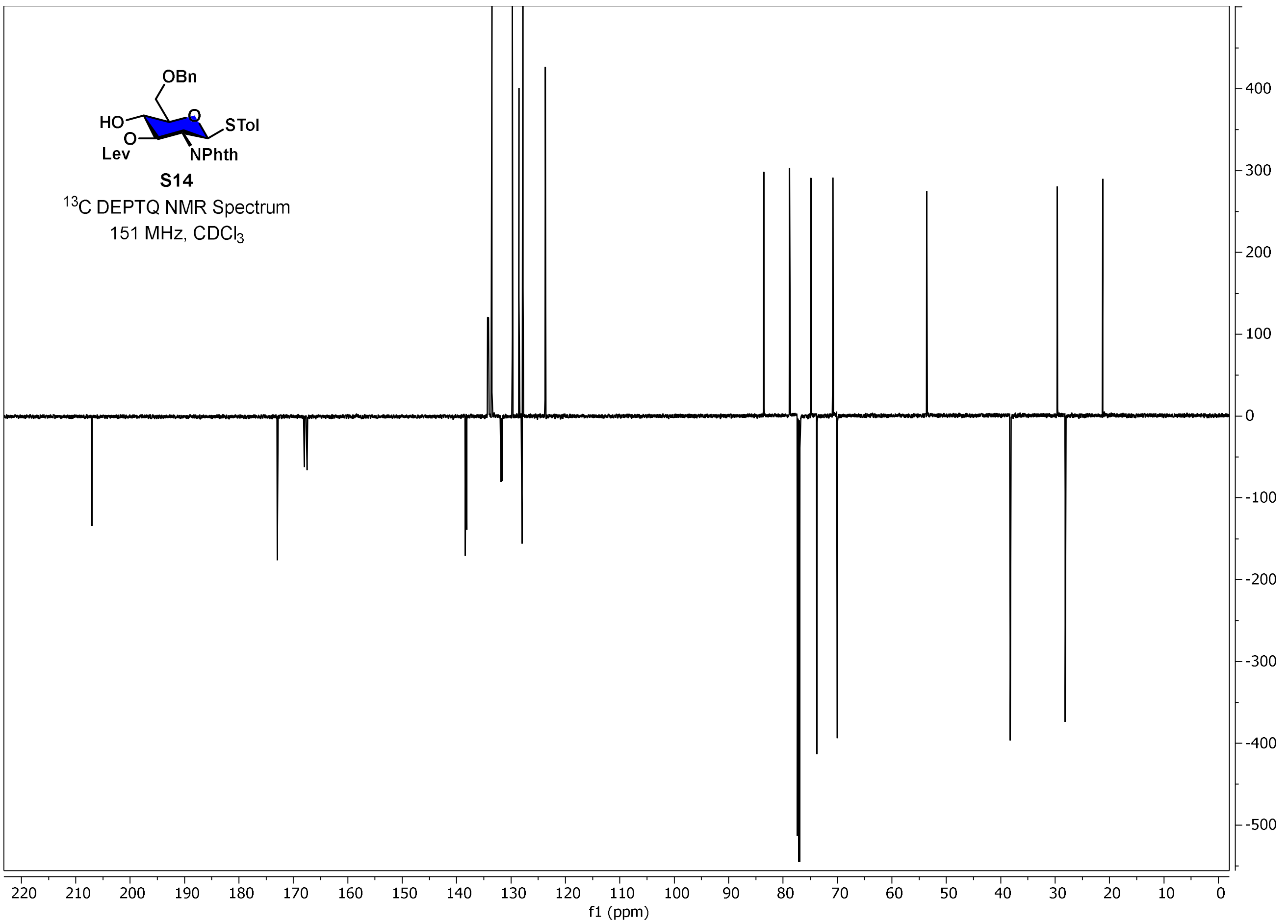
OBn
HO
O
STol
O
Lev
NPhth
S14
^{13}C DEPTQ NMR Spectrum
151 MHz, $CDCl_3$
f1 (ppm)

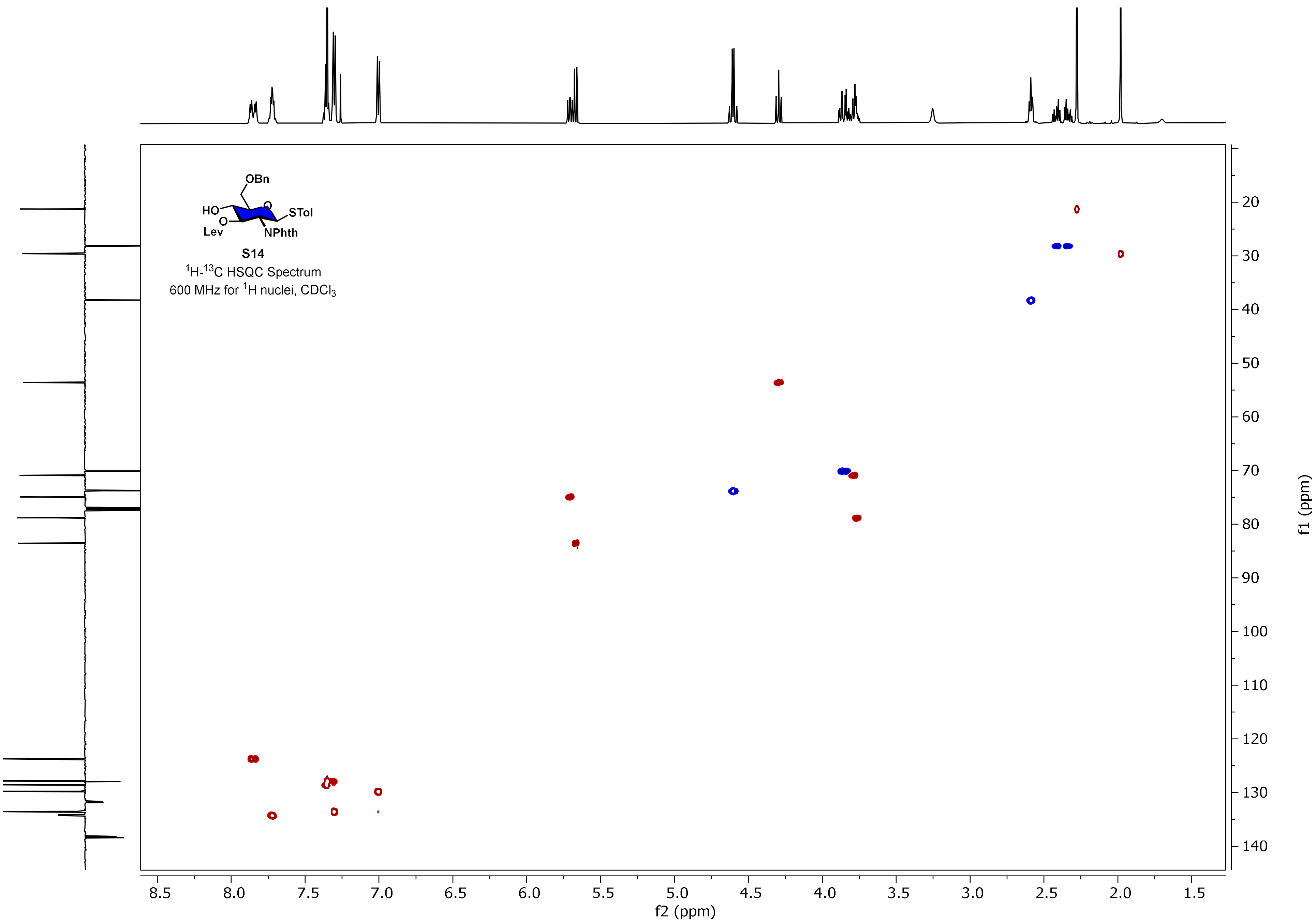

OBn
HO
O
Lev
O
STol
NPhth
S14
^{1}H-^{13}C HSQC Spectrum
600 MHz for ^{1}H nuclei, CDCl$_3$
f2 (ppm)
f1 (ppm)

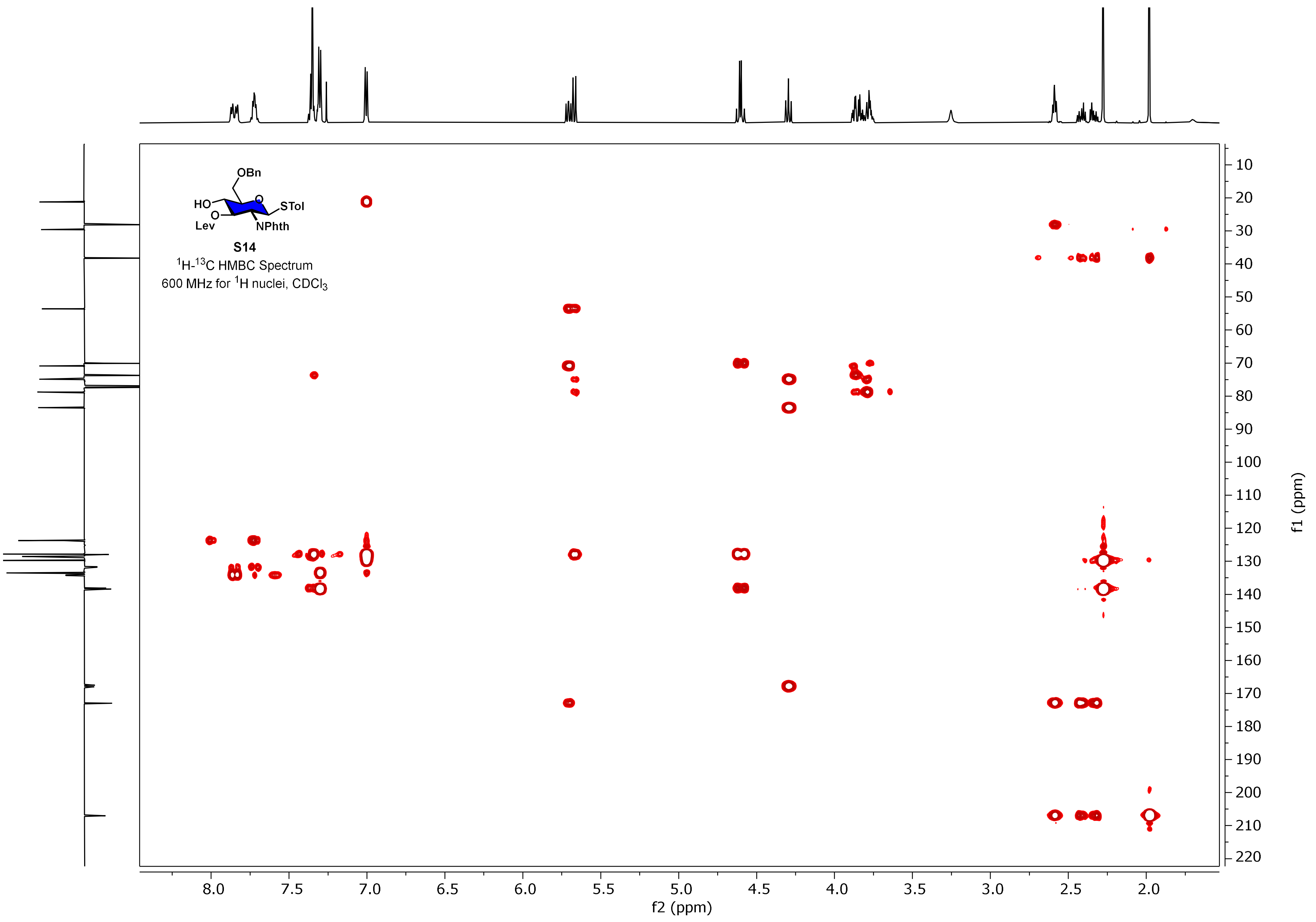
OBn
HO
O
Lev
STol
NPhth
S14
^{1}H-^{13}C HMBC Spectrum
600 MHz for ^{1}H nuclei, $CDCl_3$
f2 (ppm)
f1 (ppm)

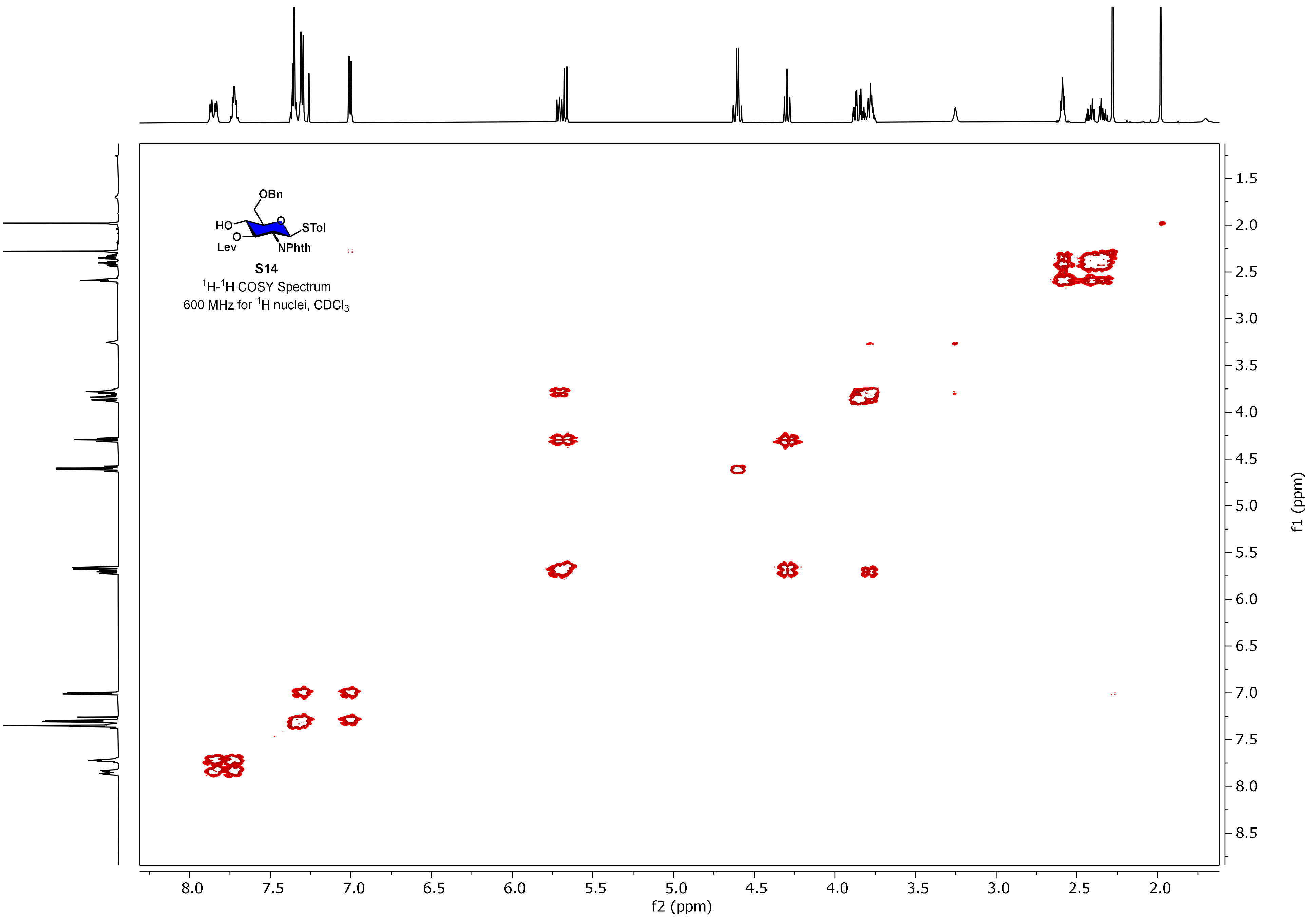
OBn
HO
O
Lev
O
STol
NPhth
S14
1H-1H COSY Spectrum
600 MHz for 1H nuclei, CDCl3
f2 (ppm)
f1 (ppm)

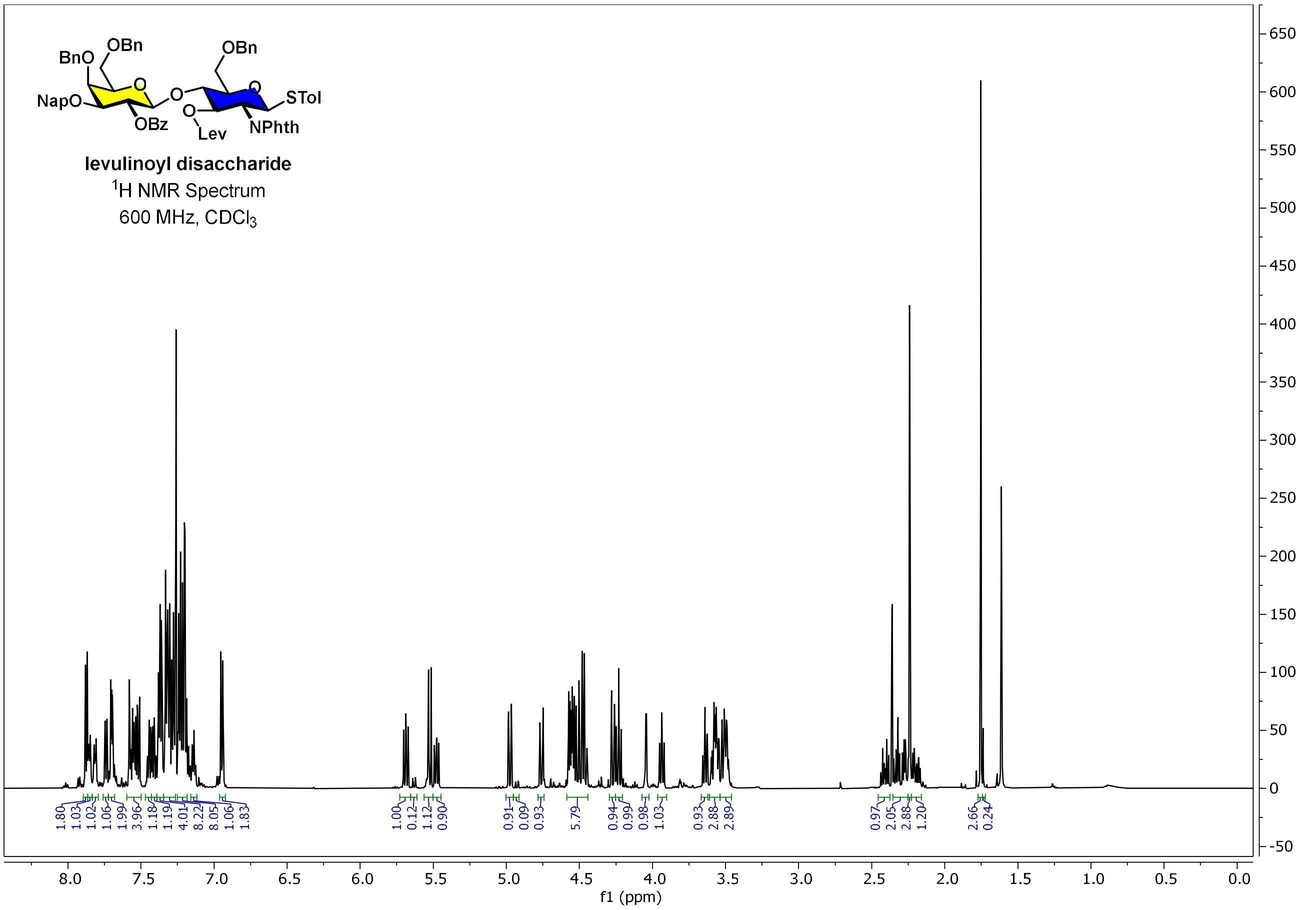
levulinoyl disaccharide
^{1}H NMR Spectrum
600 MHz, CDCl$_3$
f1 (ppm)

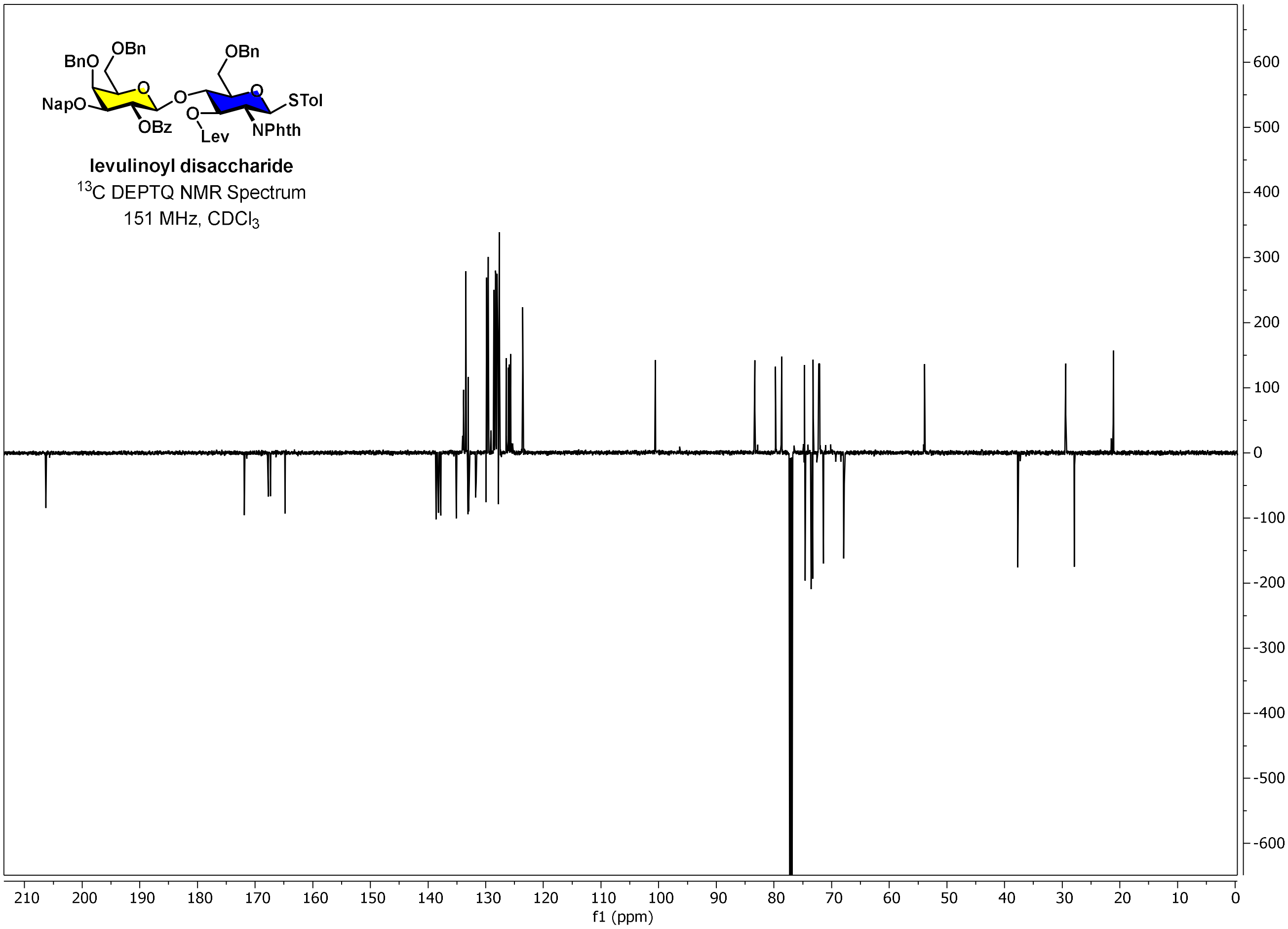
levulinoyl disaccharide
^{13}C DEPTQ NMR Spectrum
151 MHz, CDCl$_3$
f1 (ppm)

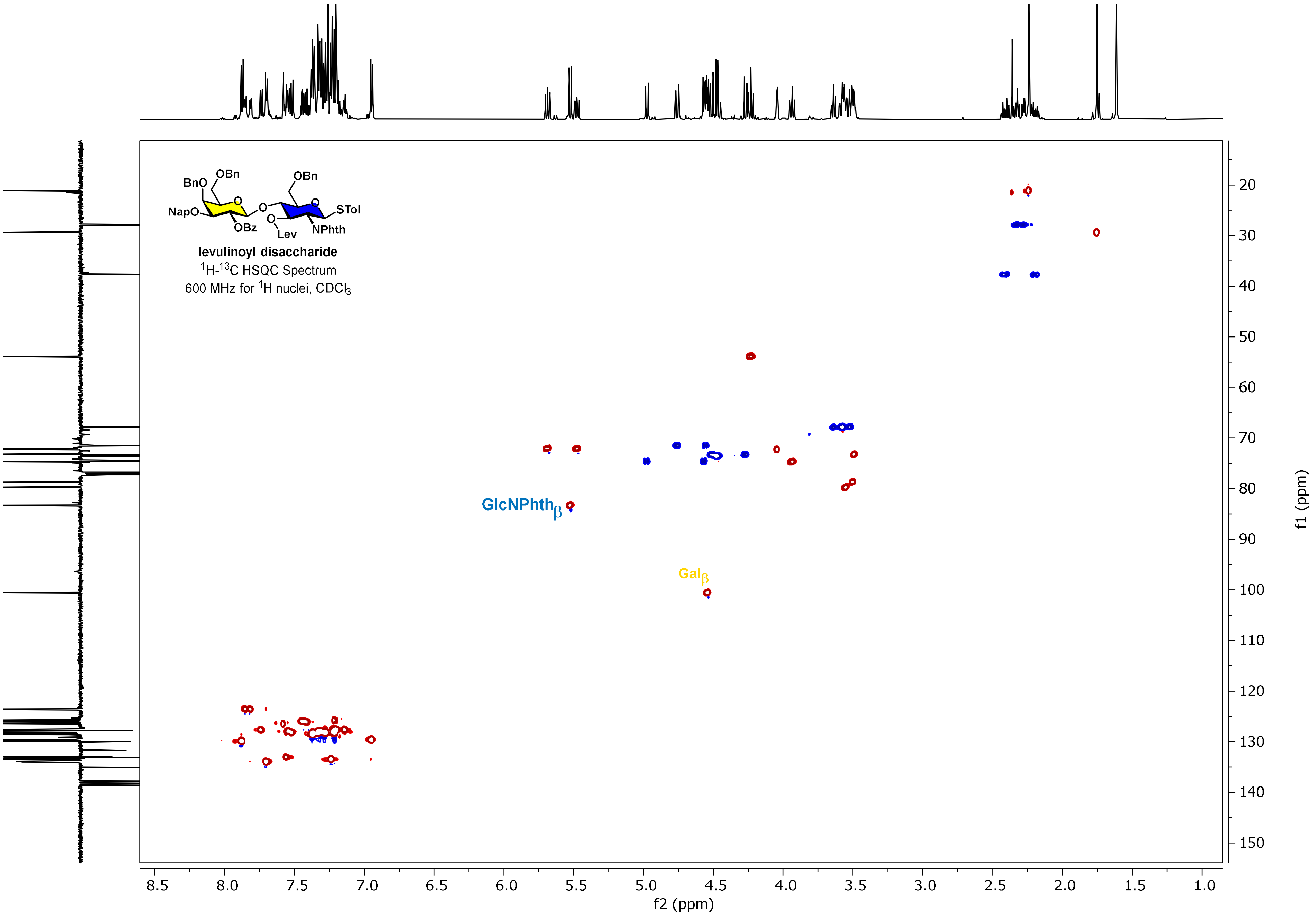

BnO
OBn
NapO
OBz
O
OBn
STol
Lev
NPhth
levulinoyl disaccharide
^{1}H-^{13}C HSQC Spectrum
600 MHz for ^{1}H nuclei, $CDCl_3$
GlcNPhth$_{\beta}$
Gal$_{\beta}$
f2 (ppm)
f1 (ppm)

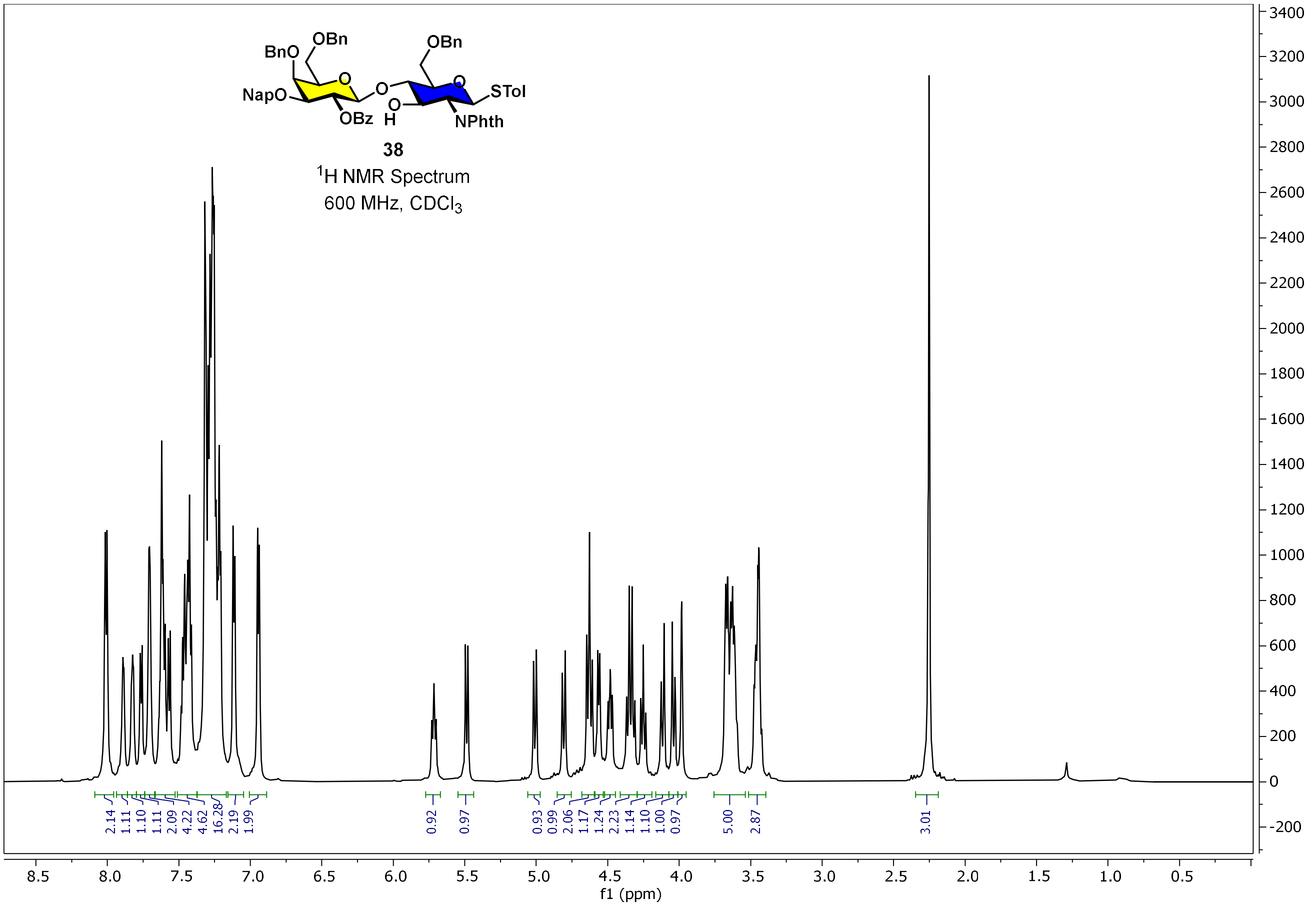
BnO
OBn
OBn
NapO
O
STol
OBz
H
NPhth
38
1H NMR Spectrum
600 MHz, CDCl3
f1 (ppm)

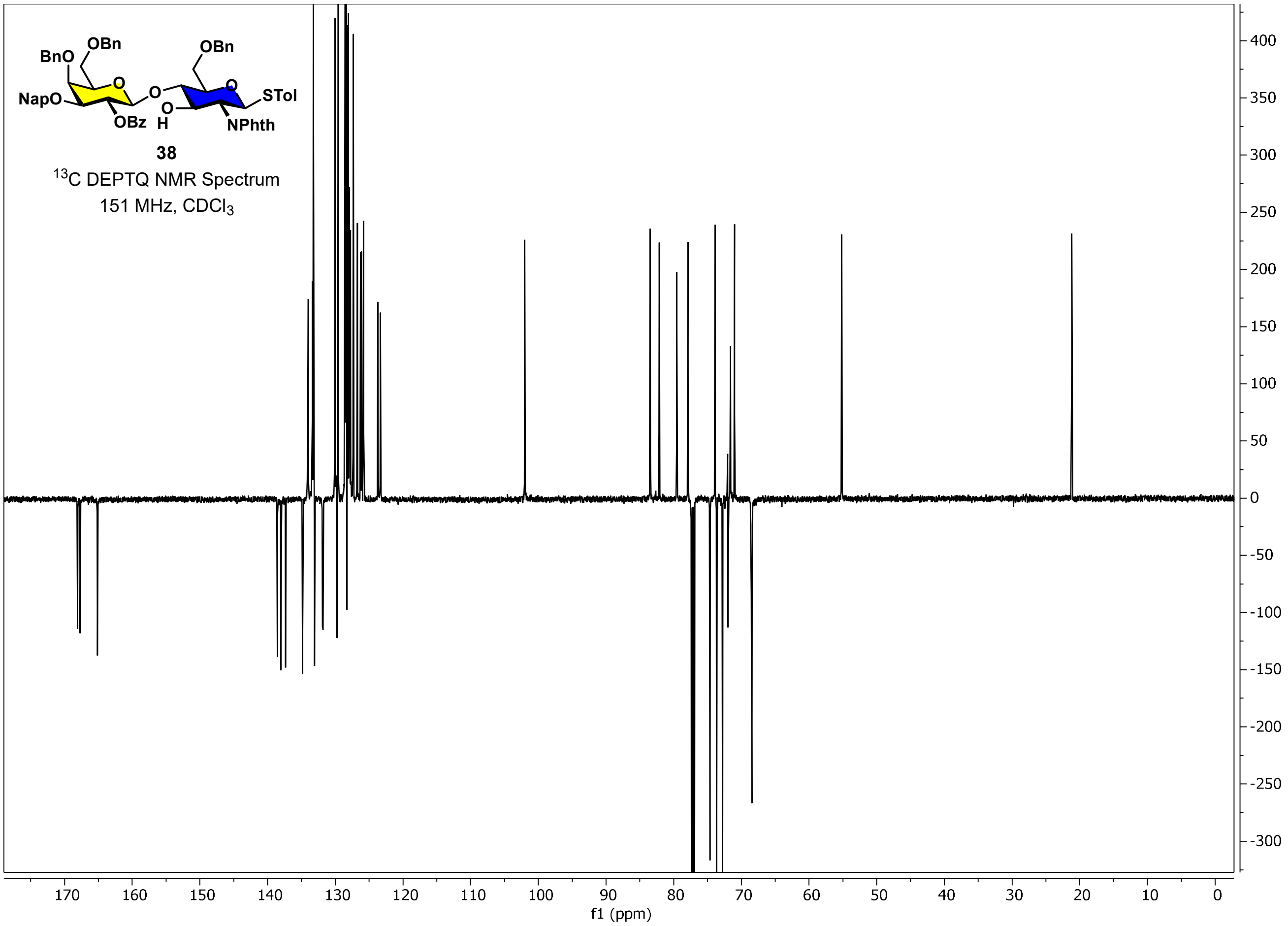
BnO
OBn
OBn
NapO
O
STol
OBz
H
NPhth
38
13C DEPTQ NMR Spectrum
151 MHz, CDCl3
f1 (ppm)

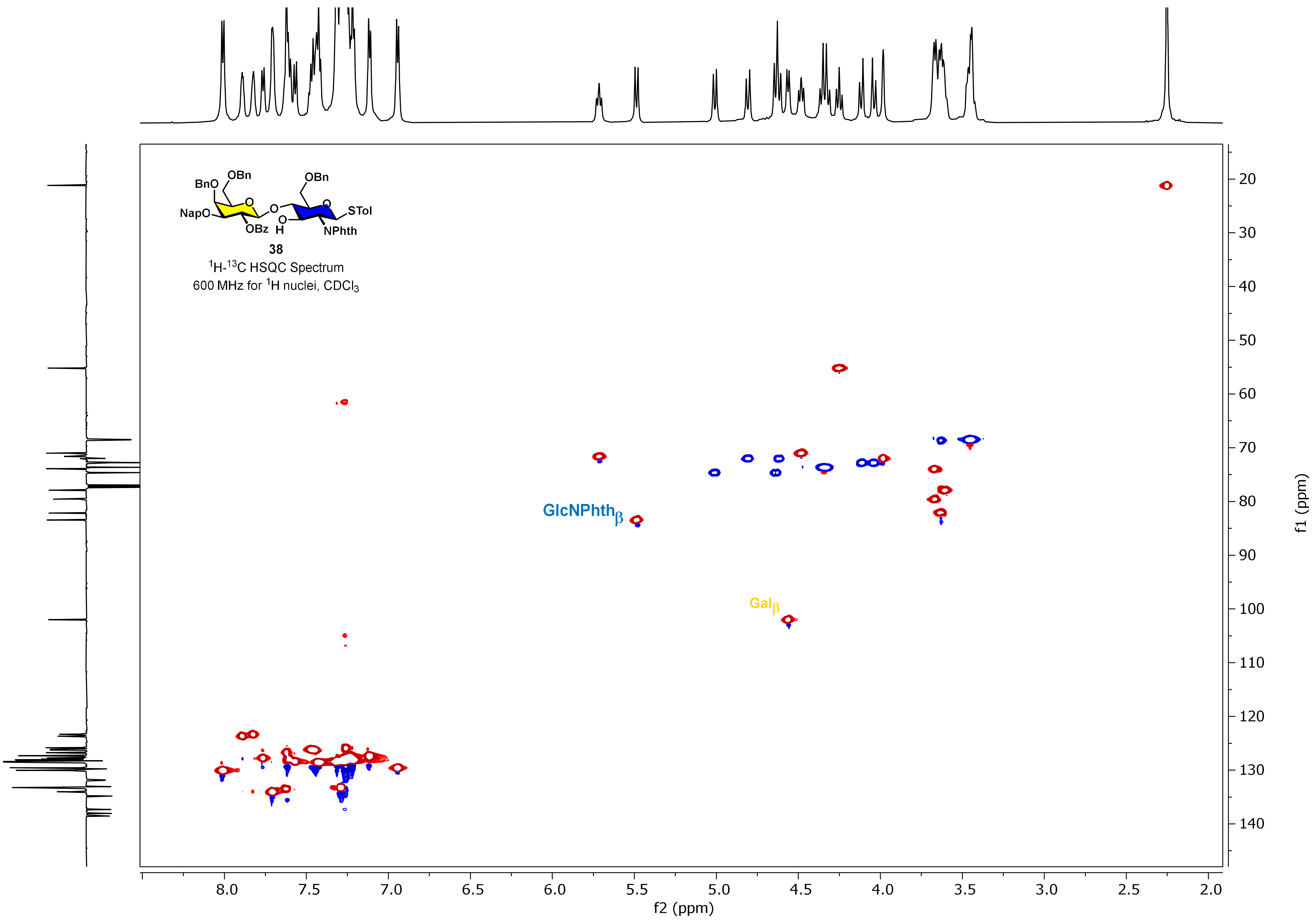

BnO
OBn
NapO
O
OBz
O
H
OBn
O
STol
NPhth
38
^{1}H-^{13}C HSQC Spectrum
600 MHz for ^{1}H nuclei, CDCl$_3$
GlcNPhth$_\beta$
Gal$_\beta$
f1 (ppm)
f2 (ppm)

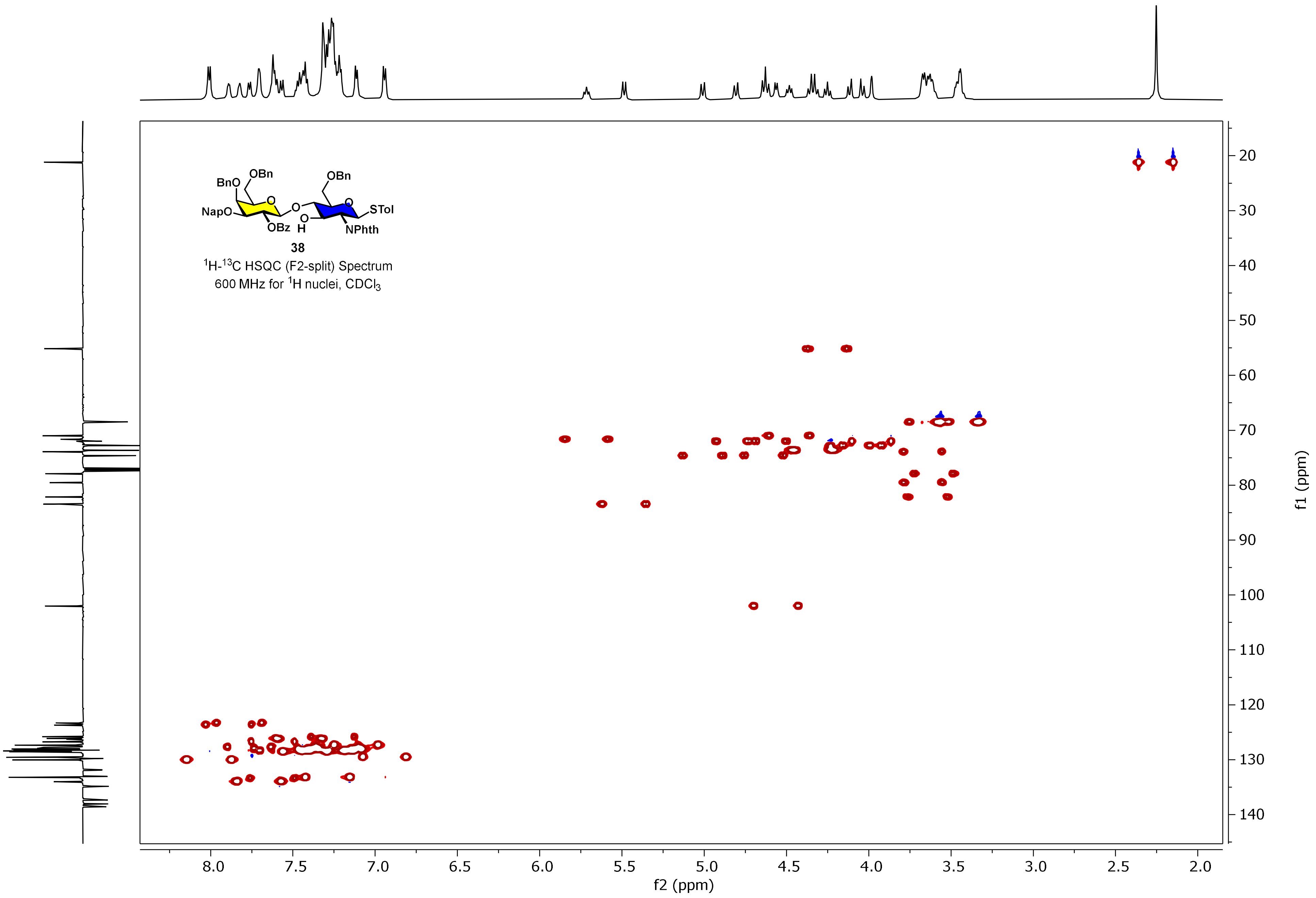

BnO
OBn
NapO
OBz
H
OBn
STol
NPhth
38
1H-13C HSQC (F2-split) Spectrum
600 MHz for 1H nuclei, CDCl3
f2 (ppm)
f1 (ppm)

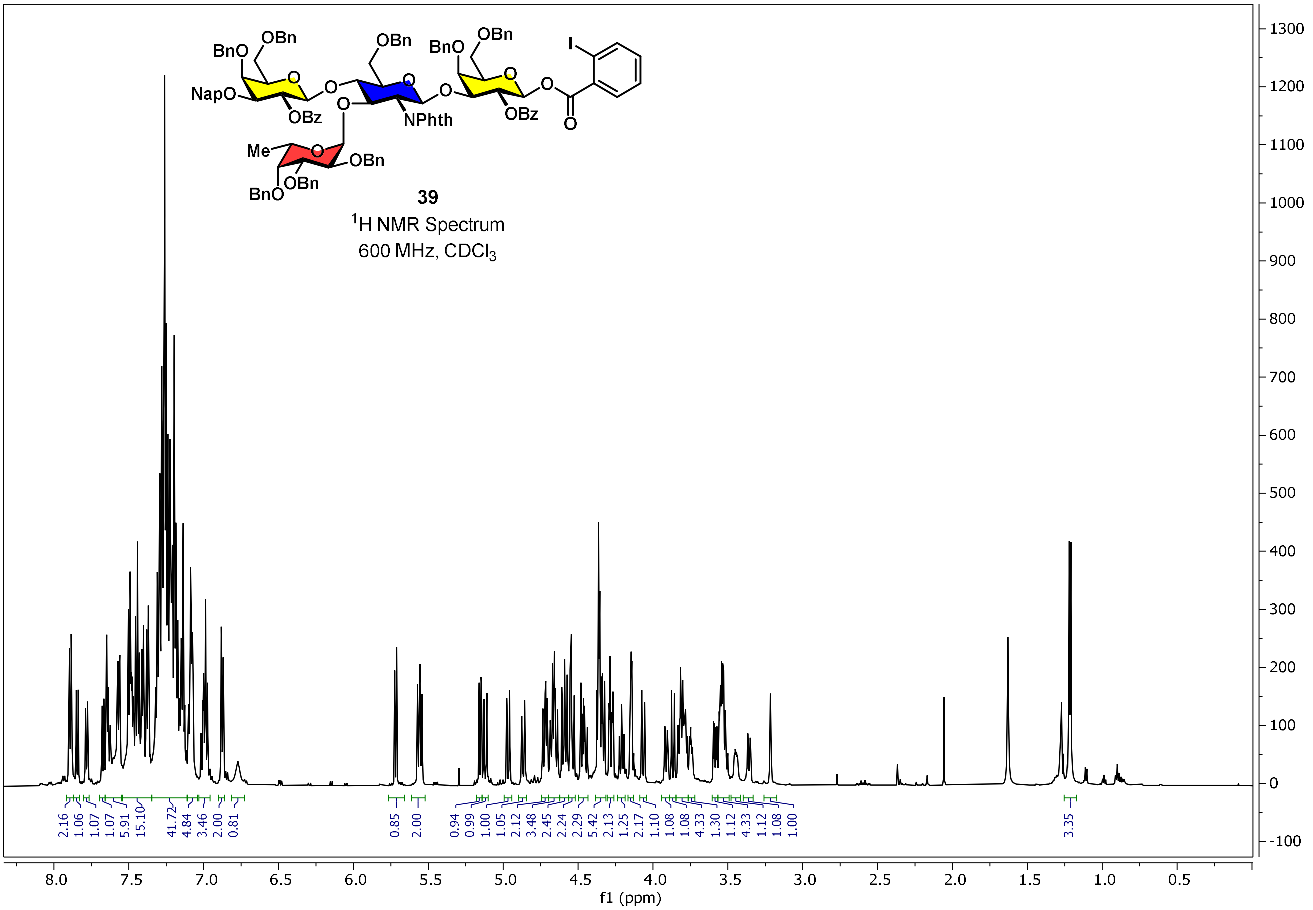
BnO
OBn
NapO
OBz
O
OBn
NPhth
BnO
OBn
OBz
I
Me
OBn
BnO
OBn
39
1H NMR Spectrum
600 MHz, CDCl3
2.16
1.06
1.07
1.07
5.91
15.10
41.72
4.84
3.46
2.00
0.81
0.85
2.00
0.94
0.99
1.00
1.05
2.12
3.48
2.45
2.24
2.29
5.42
2.13
1.25
2.17
1.10
1.08
1.08
4.33
1.30
1.12
4.33
1.12
1.08
1.00
3.35
f1 (ppm)

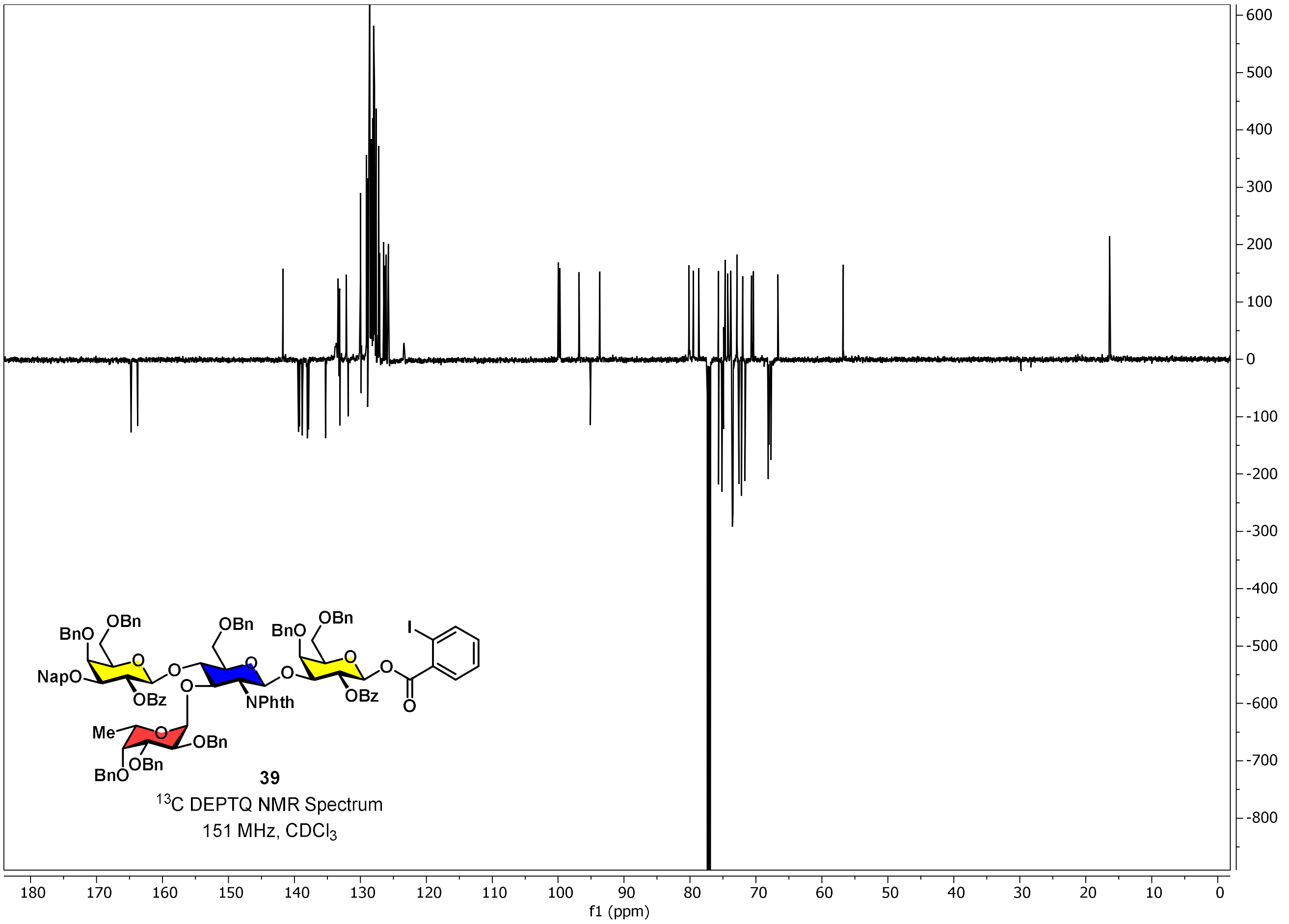
BnO
OBn
NapO
OBz
OBn
NPhth
BnO
OBn
OBz
I
Me
OBn
BnO
OBn
39
13C DEPTQ NMR Spectrum
151 MHz, CDCl3
f1 (ppm)

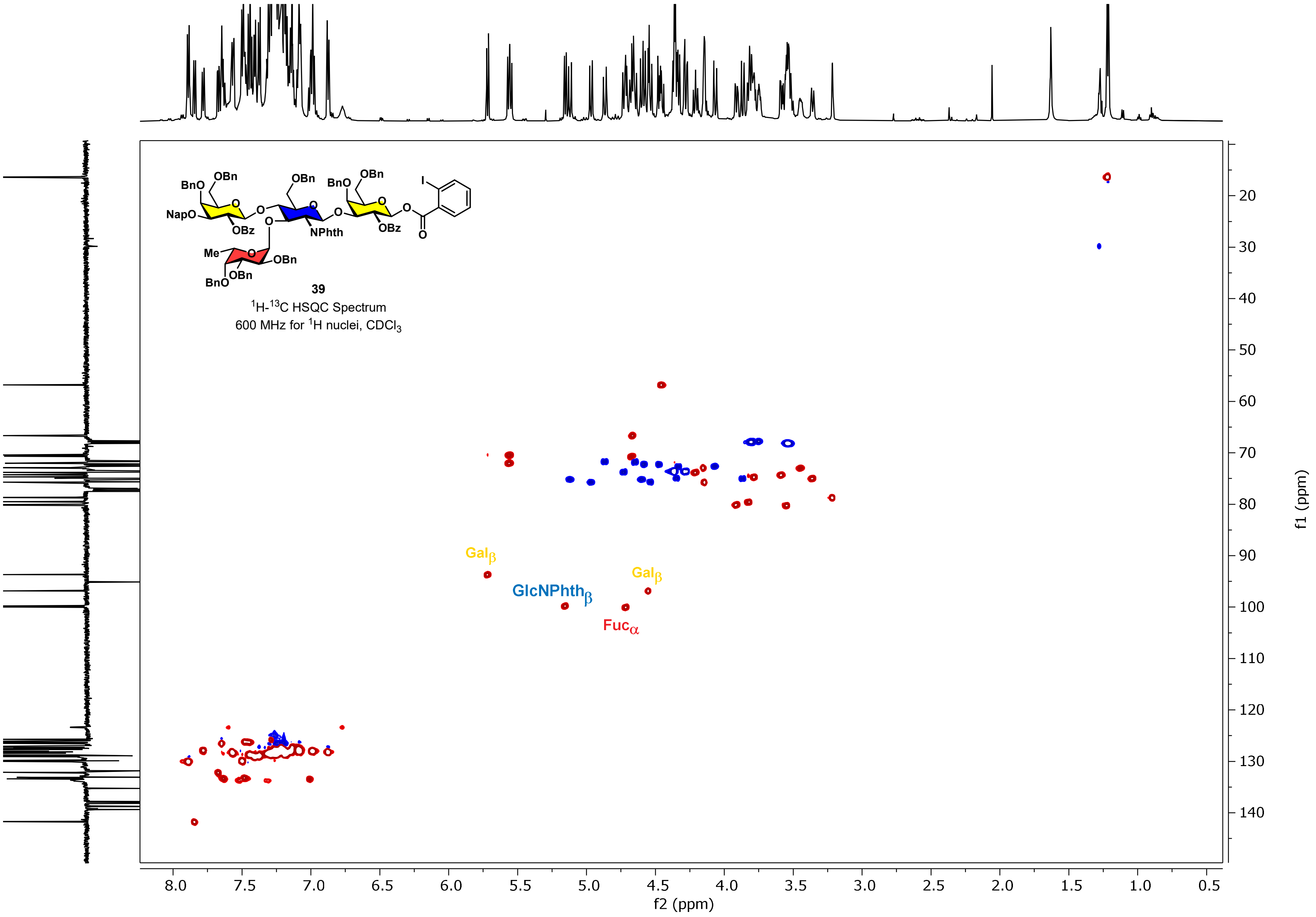

BnO
OBn
NapO
OBz
OBn
NPhth
BnO
OBn
OBz
I
O
Me
OBn
BnO
OBn
39
^{1}H-^{13}C HSQC Spectrum
600 MHz for ^{1}H nuclei, $CDCl_3$
Gal$_\beta$
GlcNPhth$_\beta$
Gal$_\beta$
Fuc$_\alpha$
f2 (ppm)
f1 (ppm)

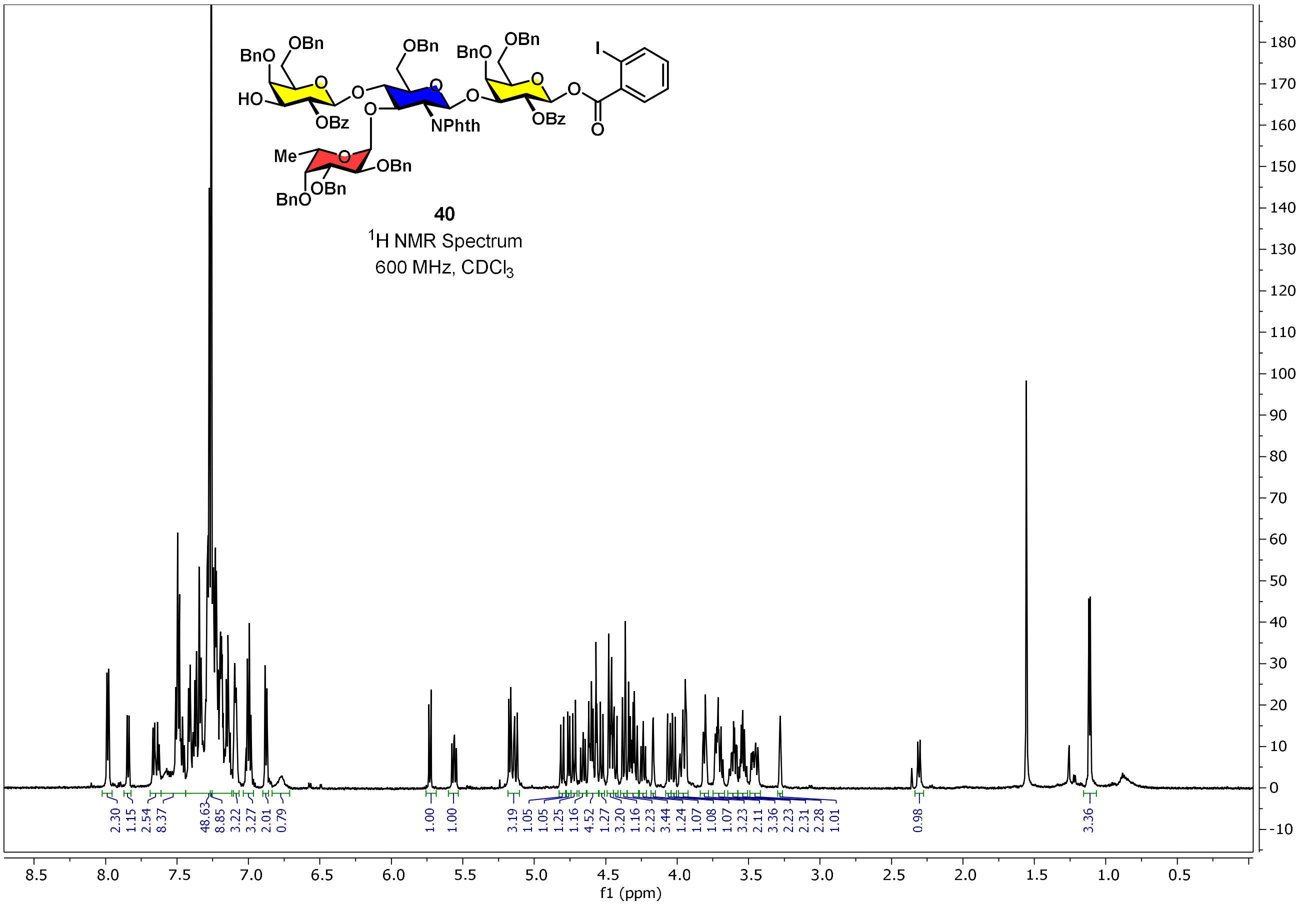

BnO
OBn
HO
OBz
OBn
NPhth
BnO
OBn
OBz
I
O
Me
OBn
BnO
OBn
40
1H NMR Spectrum
600 MHz, CDCl3
f1 (ppm)

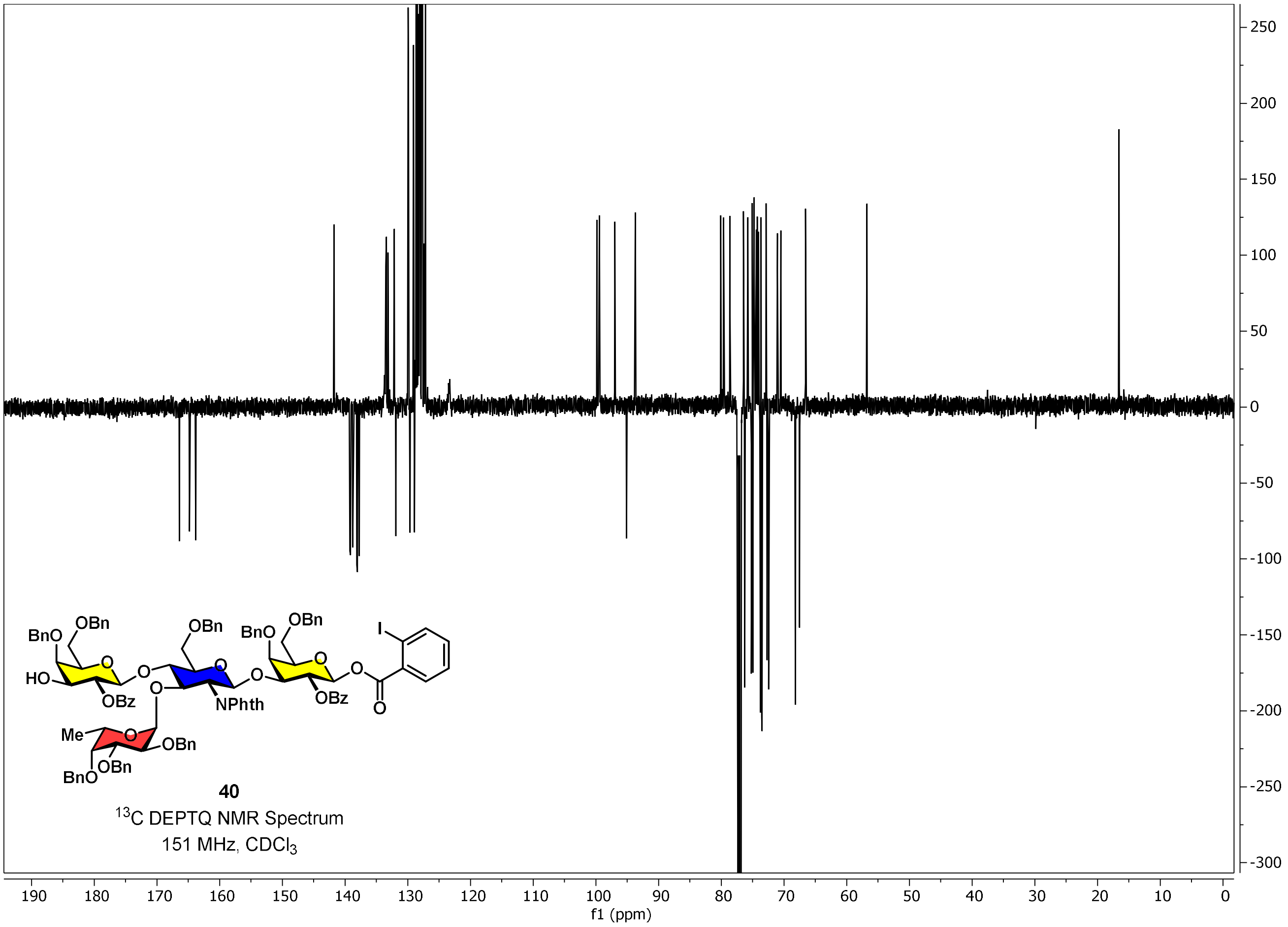

BnO
OBn
HO
OBz
OBn
NPhth
BnO
OBn
OBz
I
O
Me
OBn
BnO
OBn
40
13C DEPTQ NMR Spectrum
151 MHz, CDCl3
f1 (ppm)

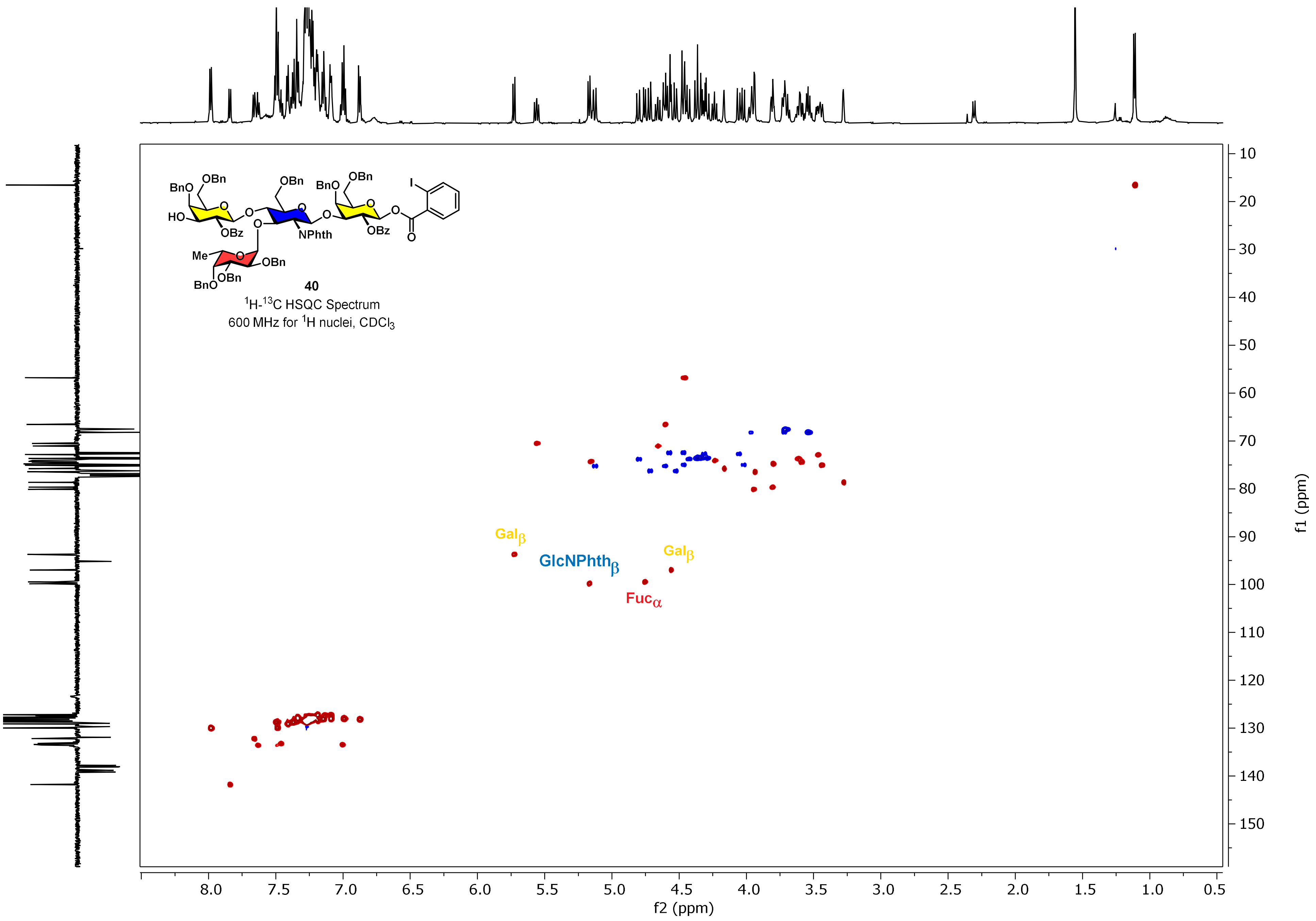
BnO
OBn
HO
OBz
O
NPhth
BnO
OBn
OBz
I
Me
OBn
BnO
OBn
40
^{1}H-^{13}C HSQC Spectrum
600 MHz for ^{1}H nuclei, $CDCl_3$
Gal$_\beta$
GlcNPhth$_\beta$
Gal$_\beta$
Fuc$_\alpha$
f2 (ppm)
f1 (ppm)

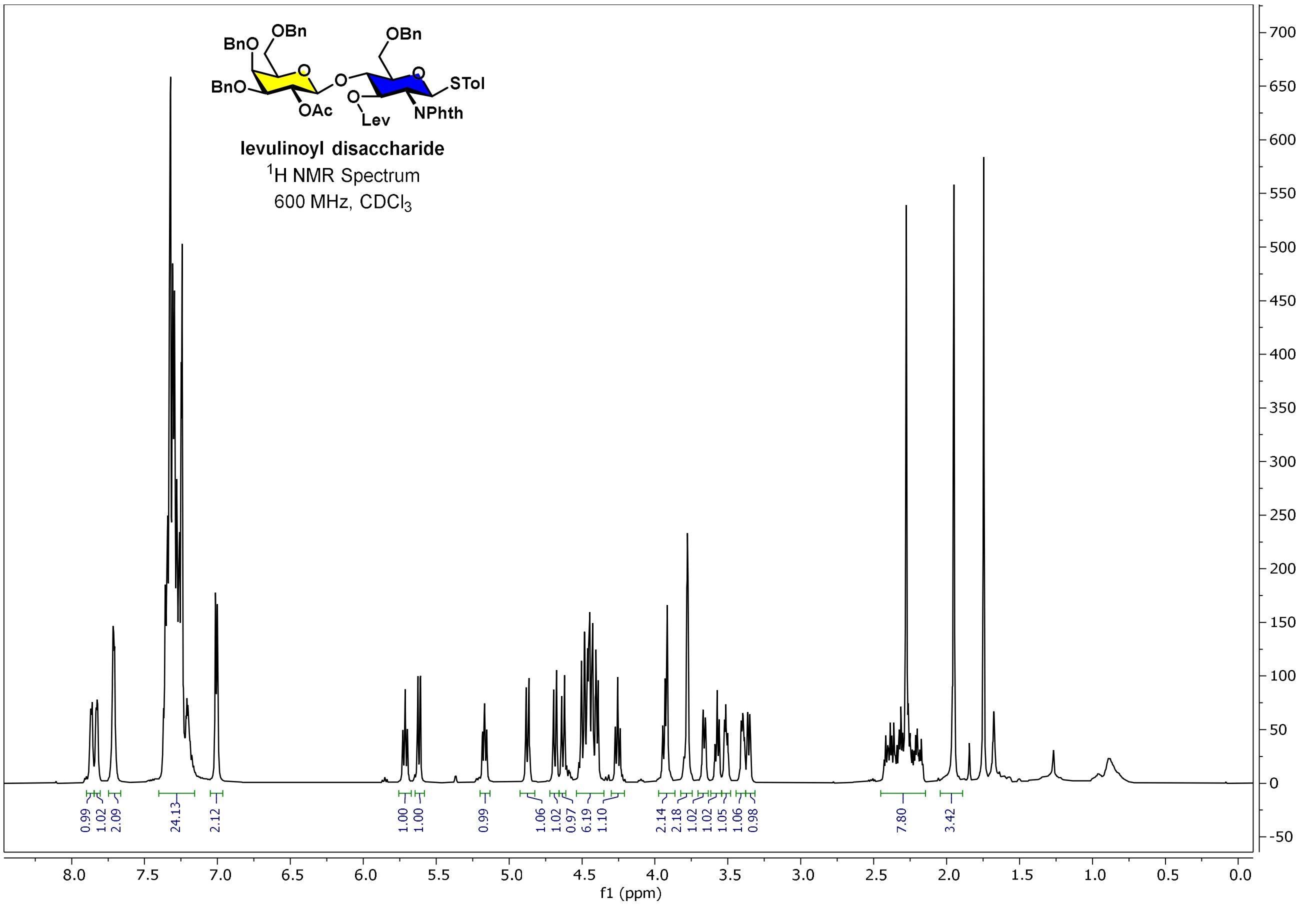
BnO
OBn
BnO
OAc
OBn
STol
Lev
NPhth
levulinoyl disaccharide
1H NMR Spectrum
600 MHz, CDCl3
f1 (ppm)

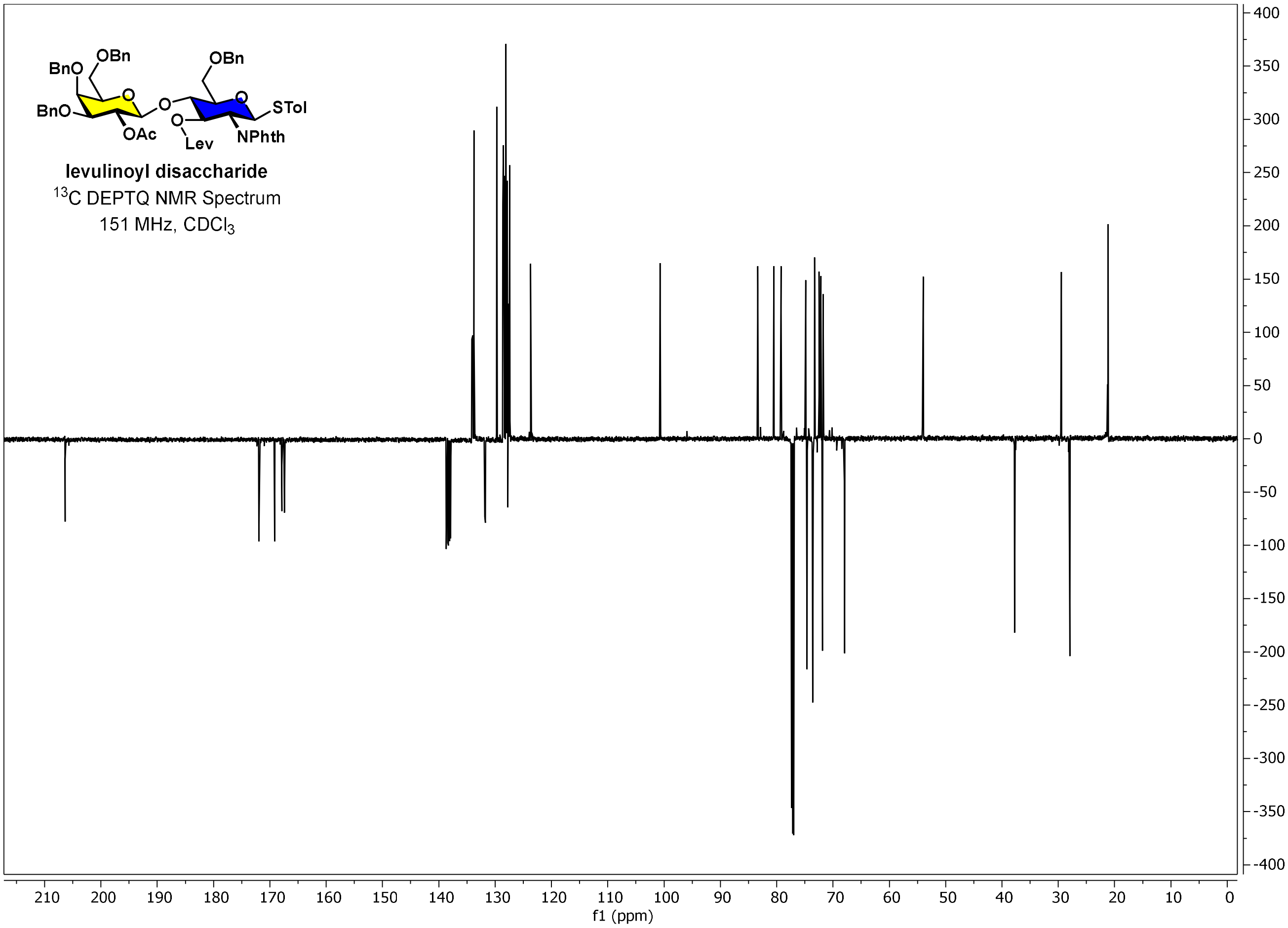
BnO
OBn
BnO
OAc
OBn
STol
Lev
NPhth
levulinoyl disaccharide
13C DEPTQ NMR Spectrum
151 MHz, CDCl3
f1 (ppm)

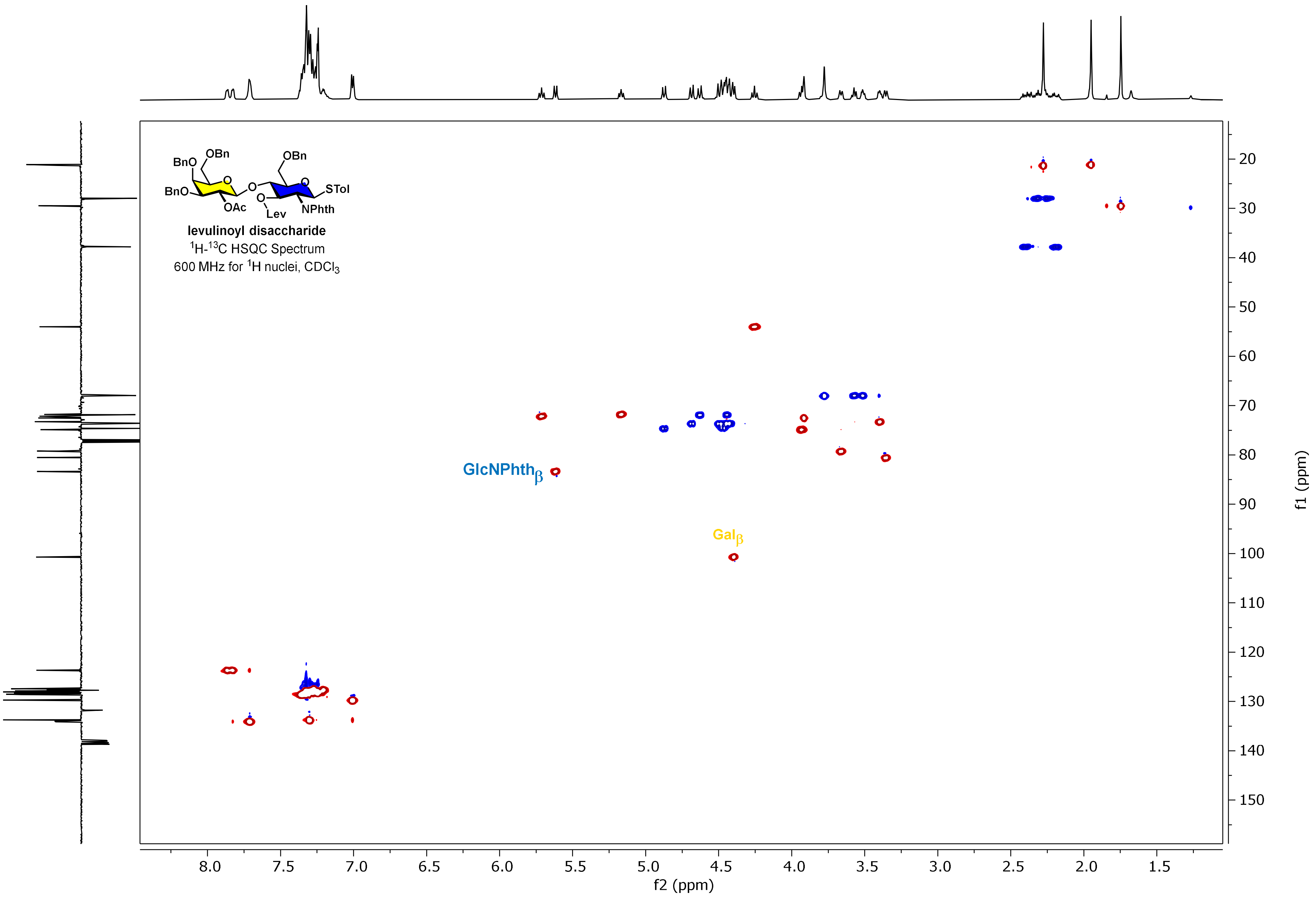

BnO
OBn
BnO
OAc
O
Lev
OBn
STol
NPhth
levulinoyl disaccharide
1H-13C HSQC Spectrum
600 MHz for 1H nuclei, CDCl3
GlcNPhthβ
Galβ
f2 (ppm)
f1 (ppm)

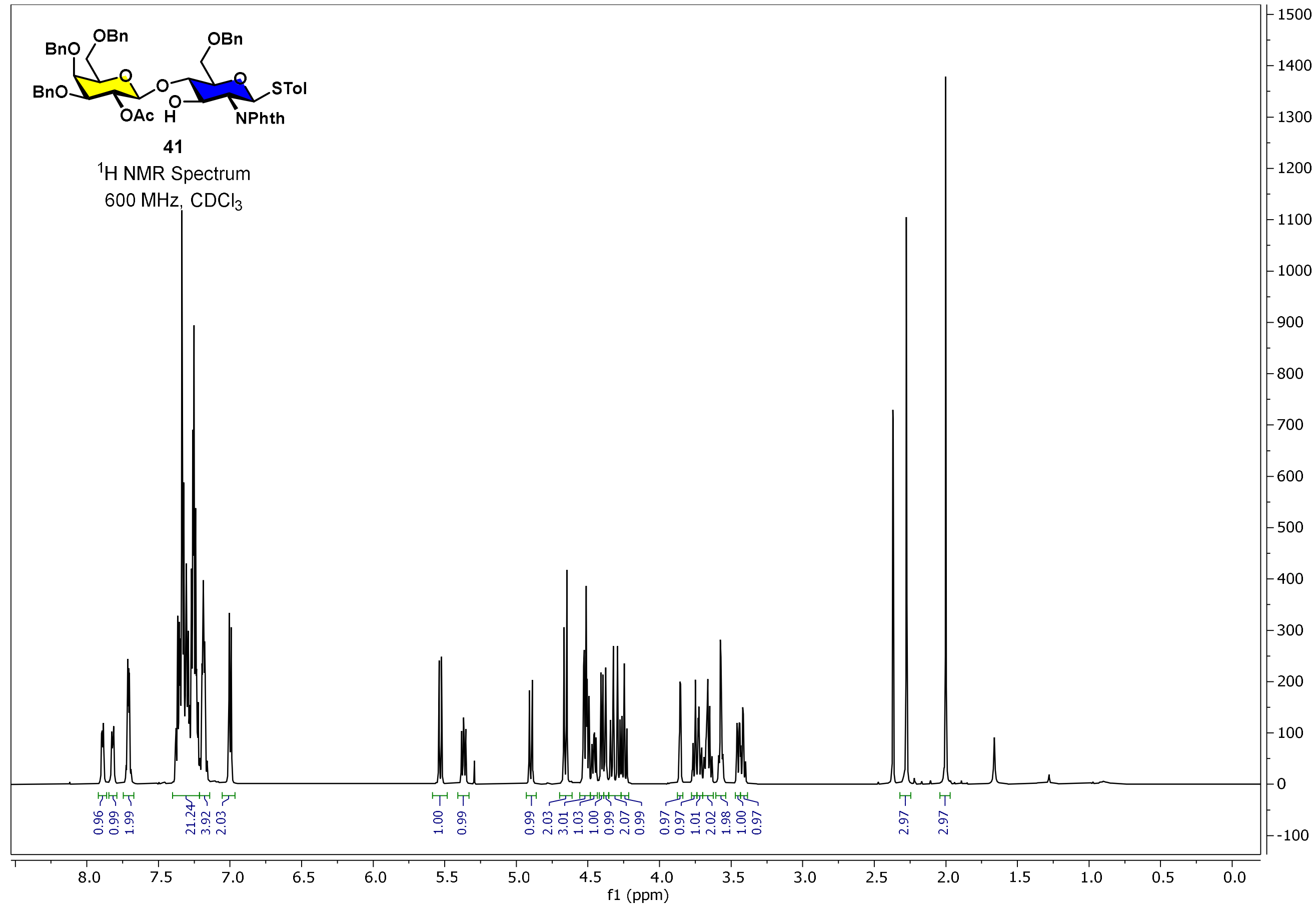
BnO
OBn
BnO
OAc
O
H
OBn
STol
NPhth
41
¹H NMR Spectrum
600 MHz, CDCl3
f1 (ppm)

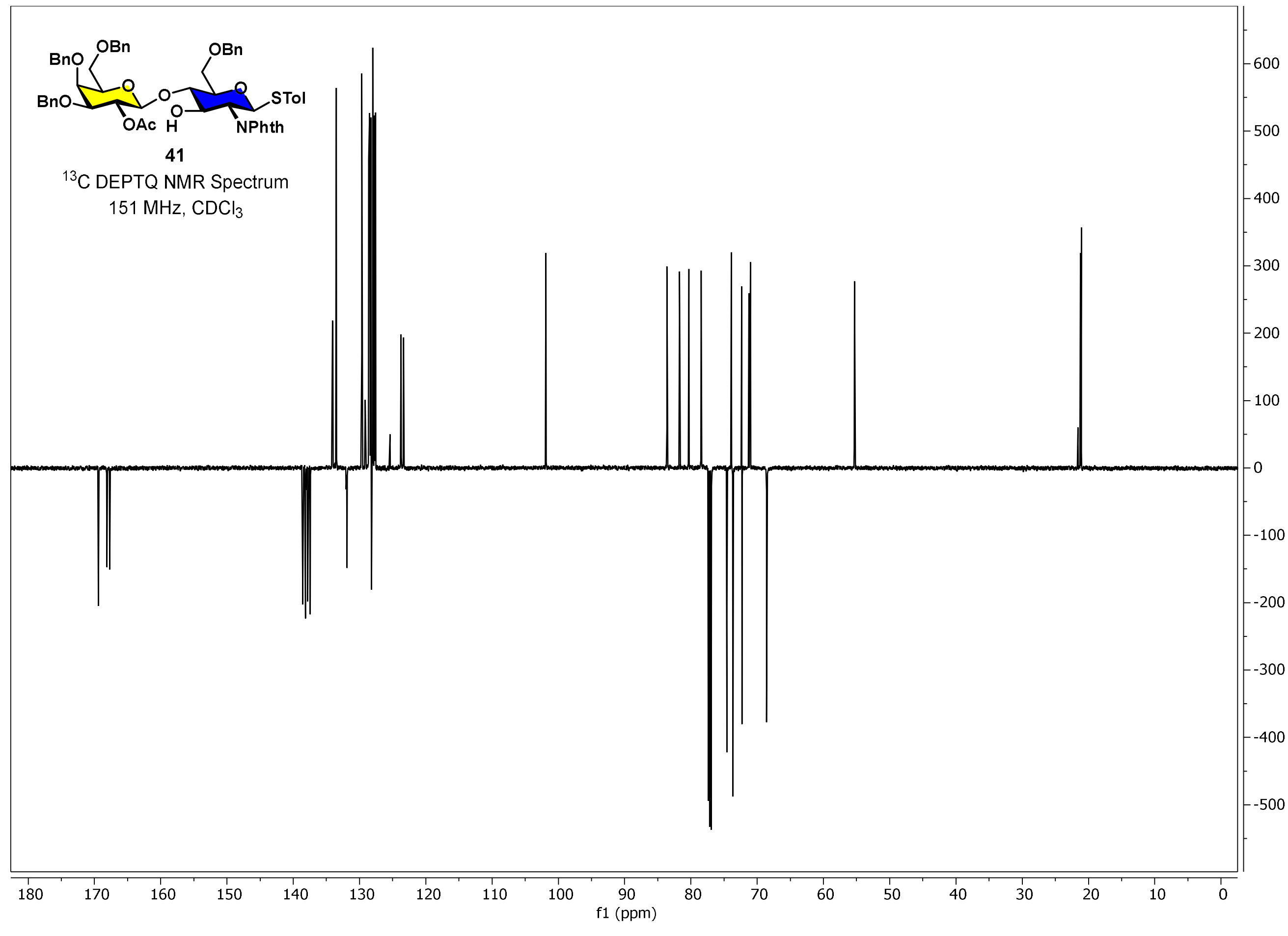
BnO
OBn
BnO
OAc
O
H
OBn
STol
NPhth
41
¹³C DEPTQ NMR Spectrum
151 MHz, CDCl3
f1 (ppm)

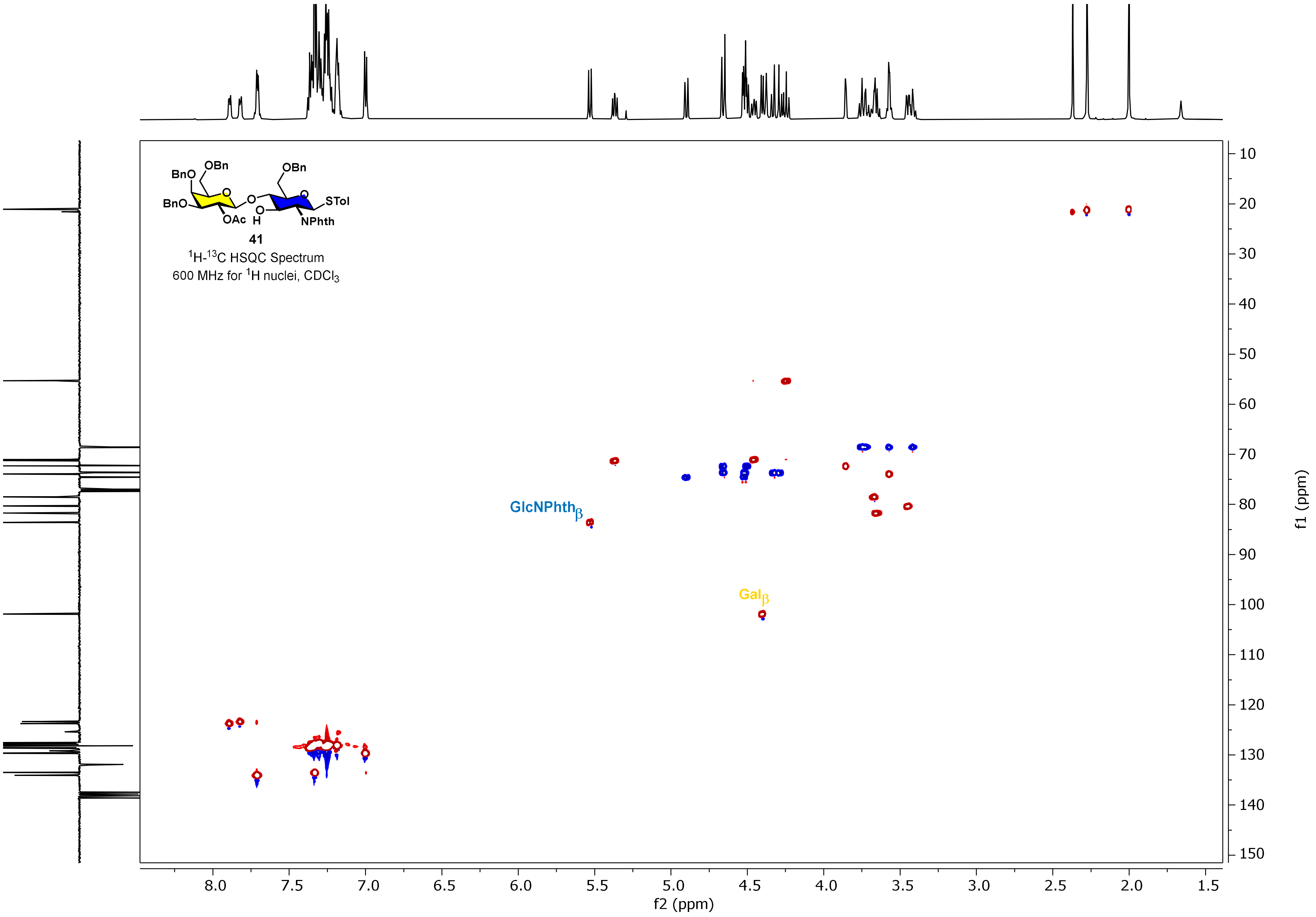
OBn
BnO
BnO
OAc
O
OBn
STol
NPhth
41
^{1}H-^{13}C HSQC Spectrum
600 MHz for ^{1}H nuclei, CDCl$_3$
GlcNPhth$_\beta$
Gal$_\beta$
f1 (ppm)
f2 (ppm)

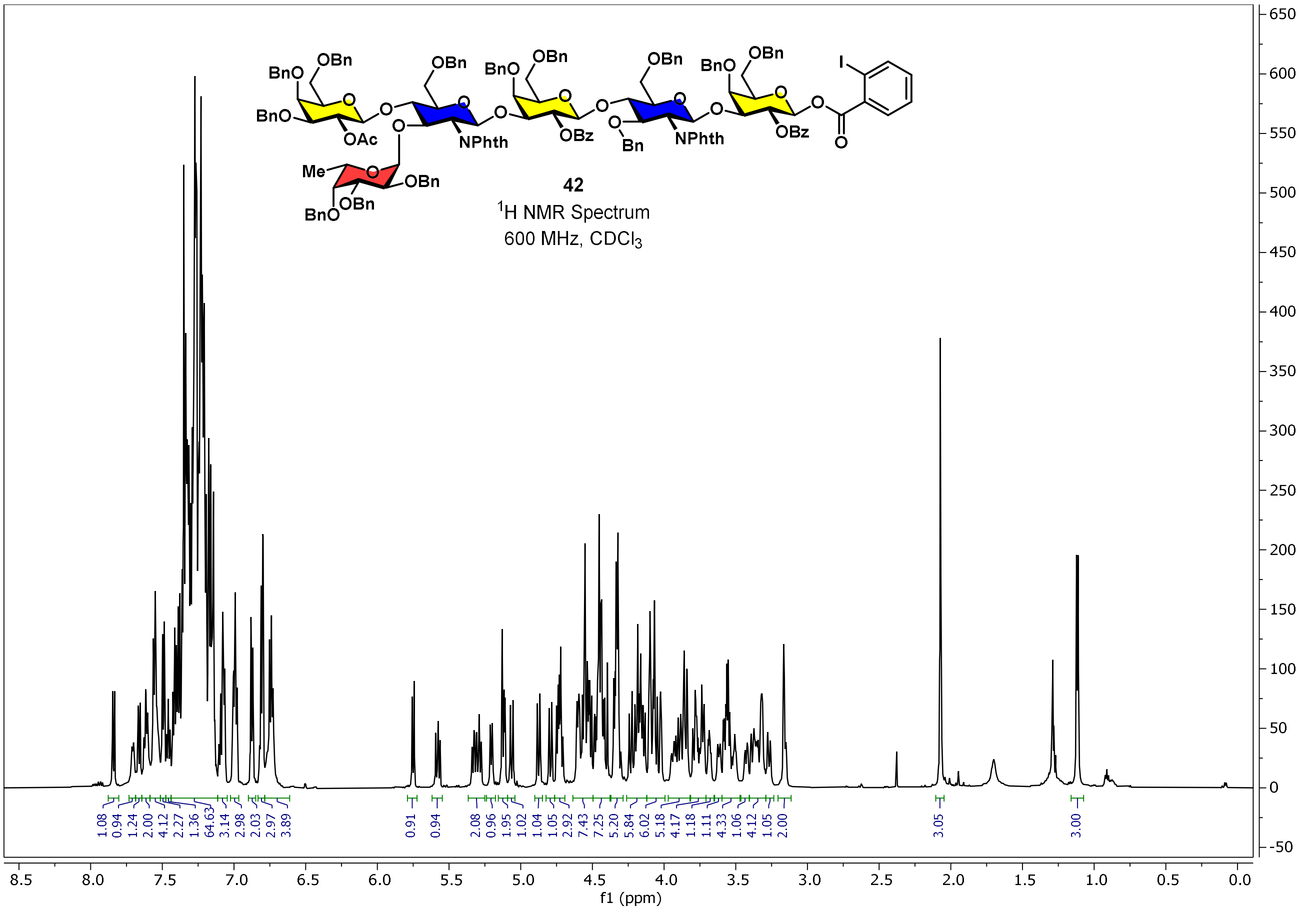
42
^{1}H NMR Spectrum
600 MHz, $CDCl_3$
f1 (ppm)

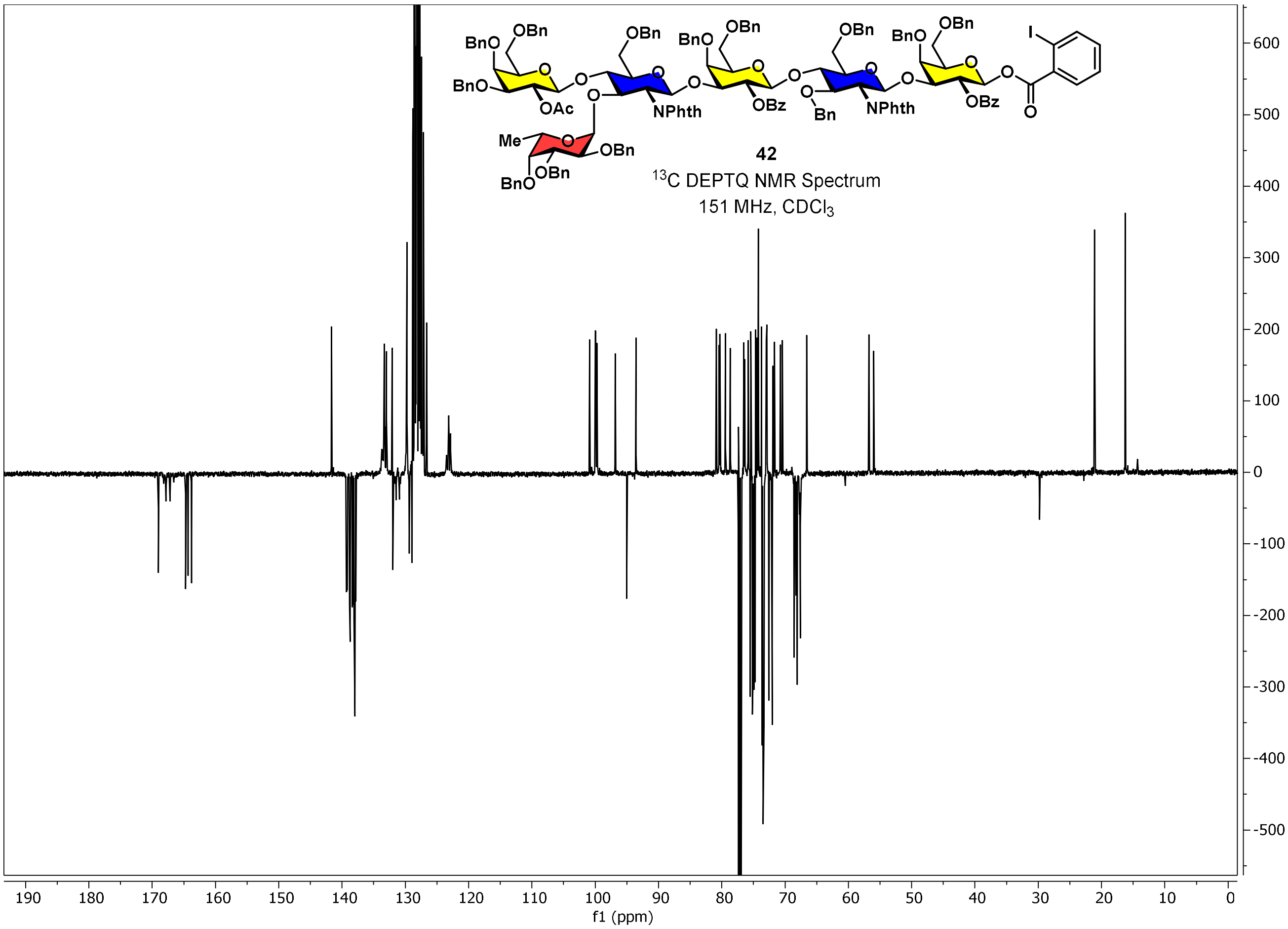
42
^{13}C DEPTQ NMR Spectrum
151 MHz, $CDCl_3$
f1 (ppm)

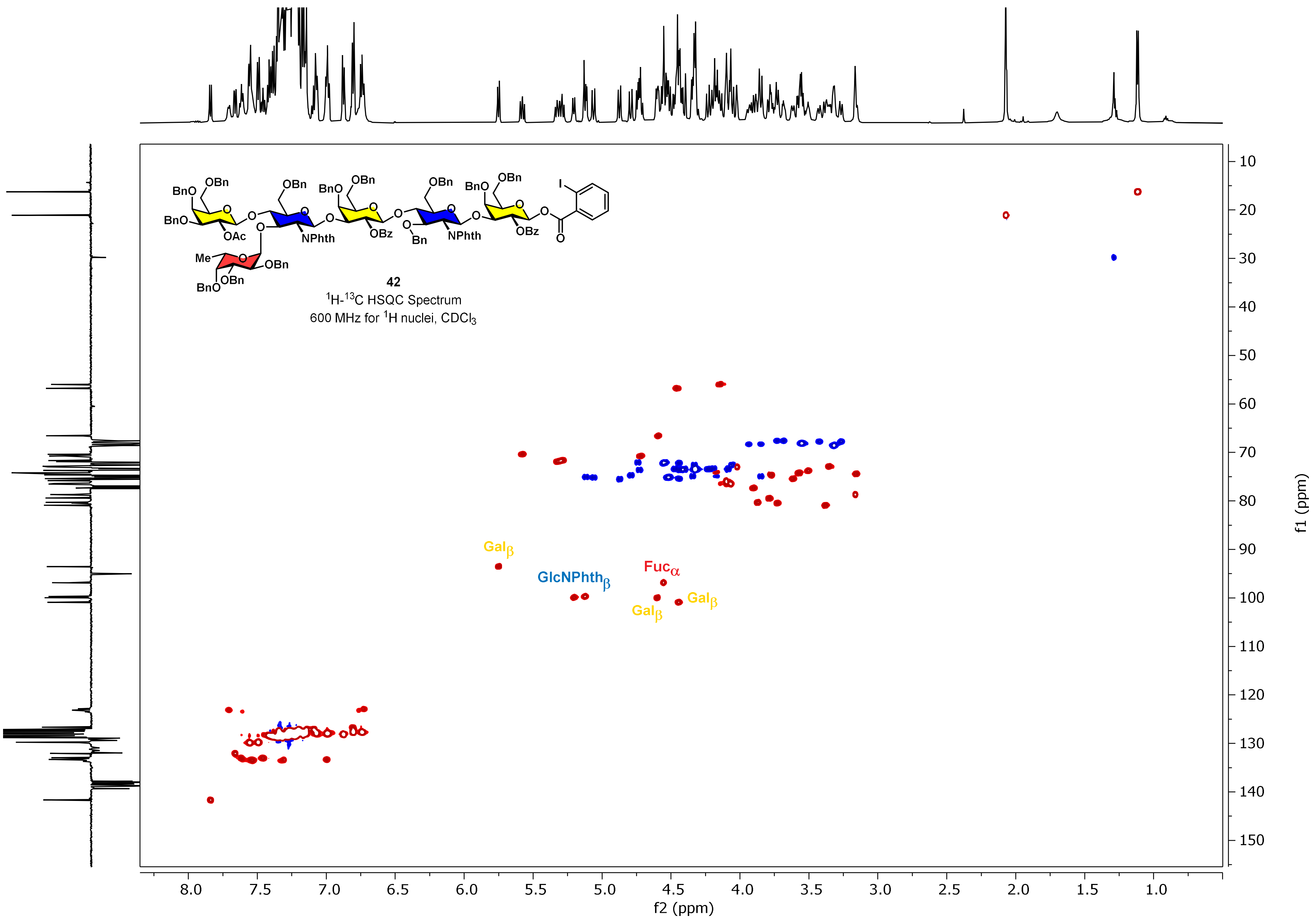

42
^{1}H-^{13}C HSQC Spectrum
600 MHz for ^{1}H nuclei, CDCl$_3$
BnO
OBn
OAc
NPhth
OBz
Bn
Me
Gal$_\beta$
GlcNPhth$_\beta$
Fuc$_\alpha$
f2 (ppm)
f1 (ppm)

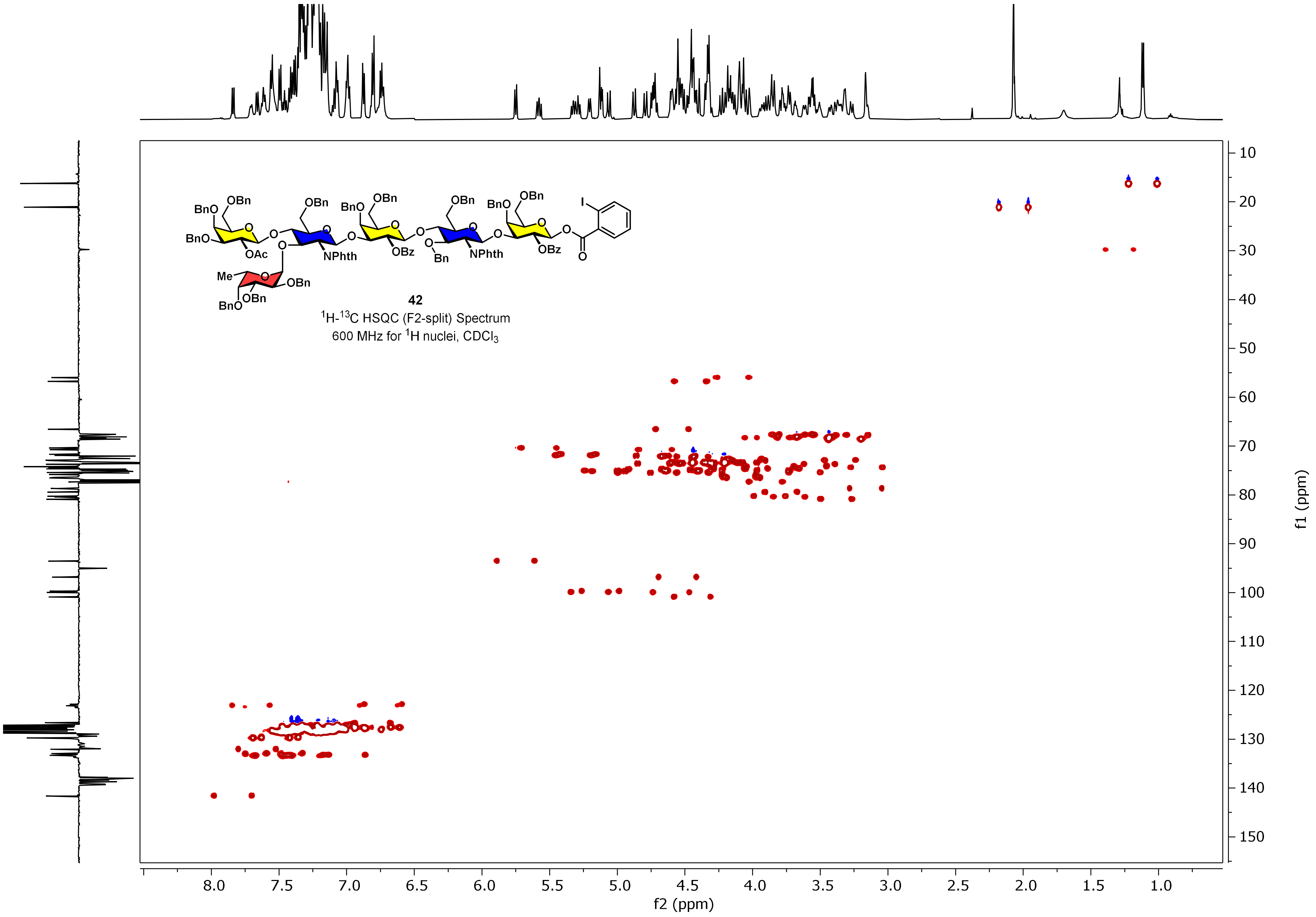
42
^{1}H-^{13}C HSQC (F2-split) Spectrum
600 MHz for ^{1}H nuclei, CDCl$_3$
f2 (ppm)
f1 (ppm)

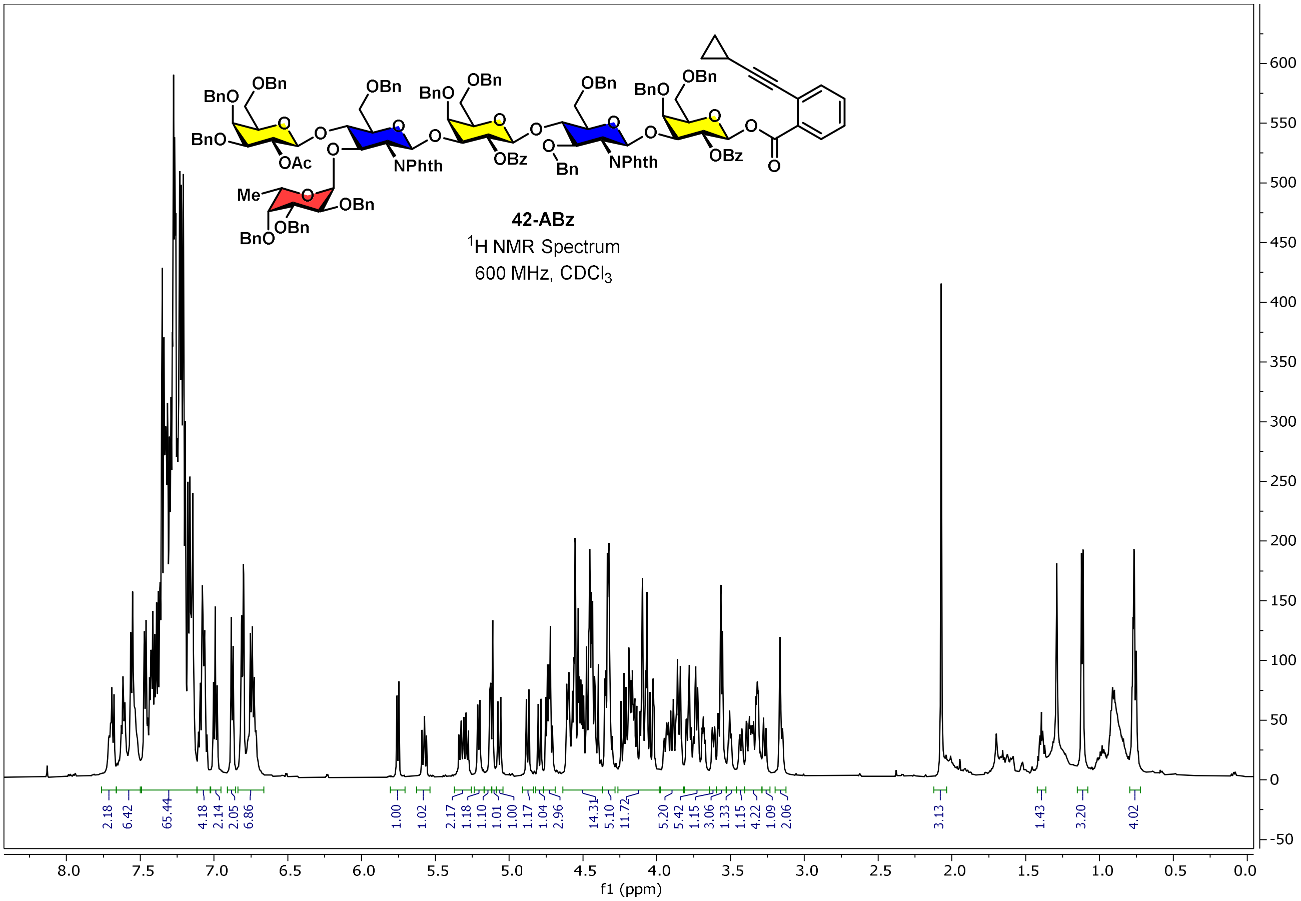
42-ABz
1H NMR Spectrum
600 MHz, CDCl3
f1 (ppm)

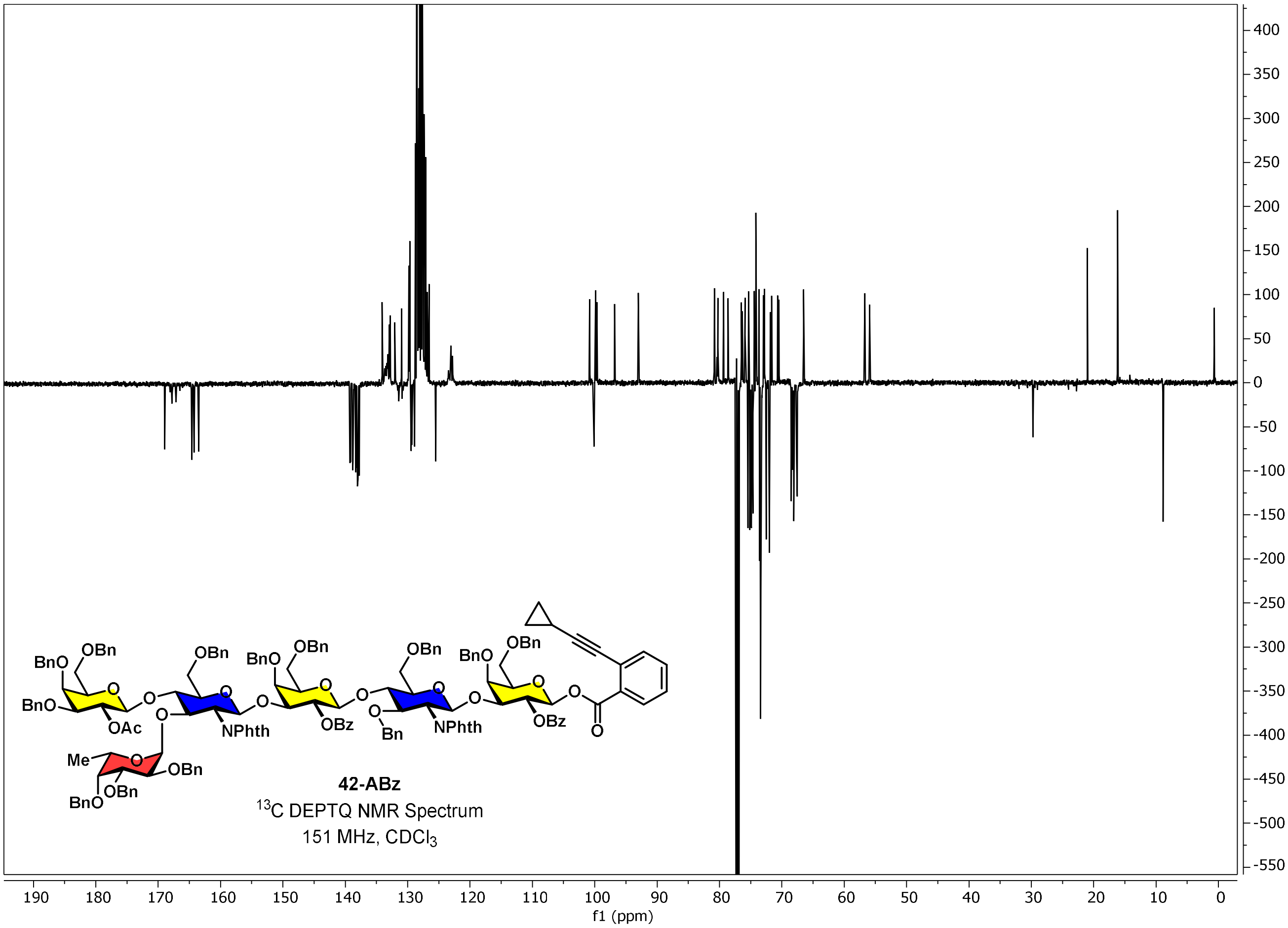
42-ABz
13C DEPTQ NMR Spectrum
151 MHz, CDCl3
f1 (ppm)

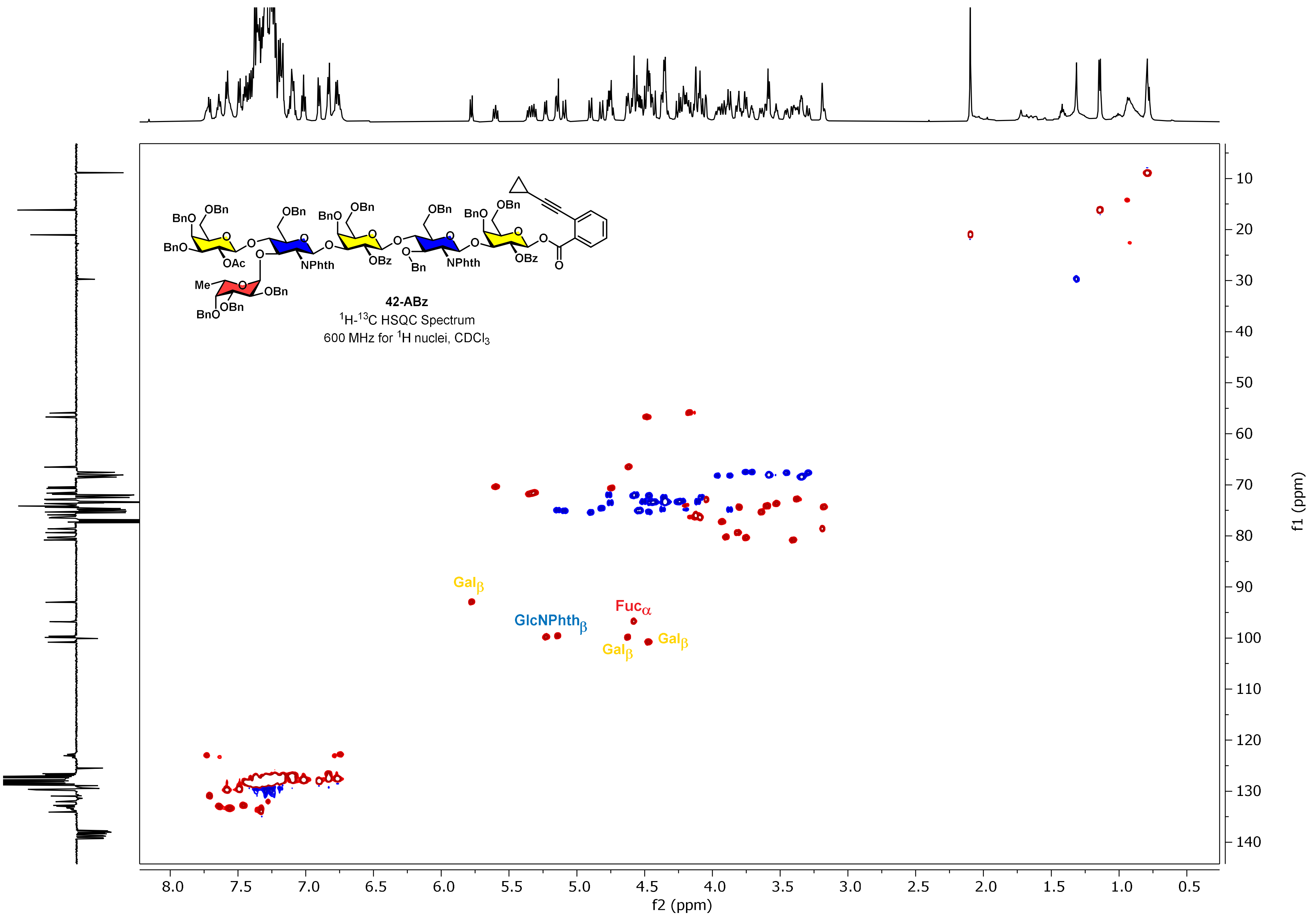

42-ABz
^{1}H-^{13}C HSQC Spectrum
600 MHz for ^{1}H nuclei, CDCl$_3$
Gal$_\beta$
GlcNPhth$_\beta$
Fuc$_\alpha$
Gal$_\beta$
Gal$_\beta$
f2 (ppm)
f1 (ppm)

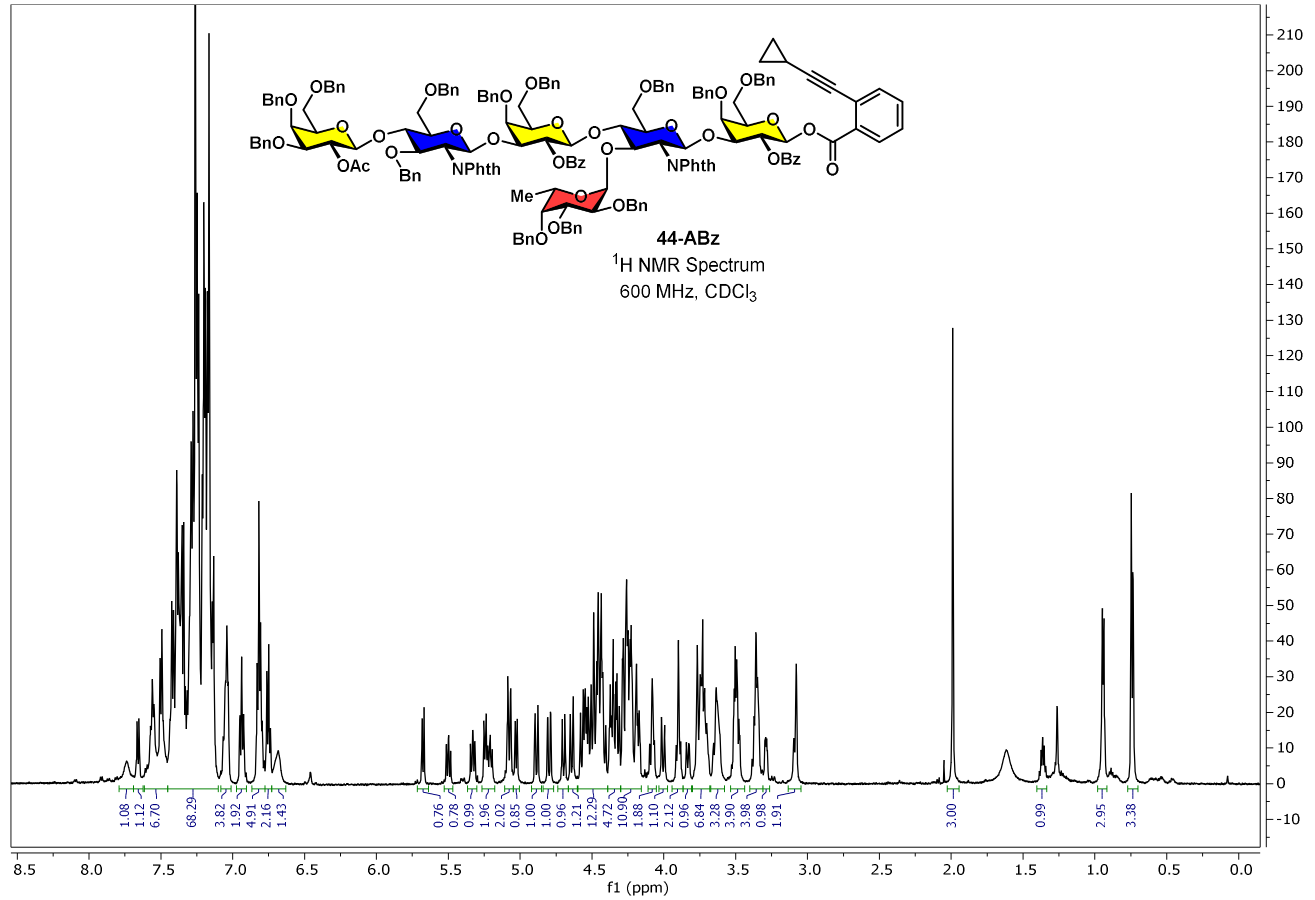
BnO
OBn
BnO
OAc
O
OBn
O
Bn
NPhth
BnO
OBn
OBz
OBn
NPhth
OBn
OBz
Me
OBn
BnO
OBn
44-ABz
^{1}H NMR Spectrum
600 MHz, $CDCl_3$
f1 (ppm)

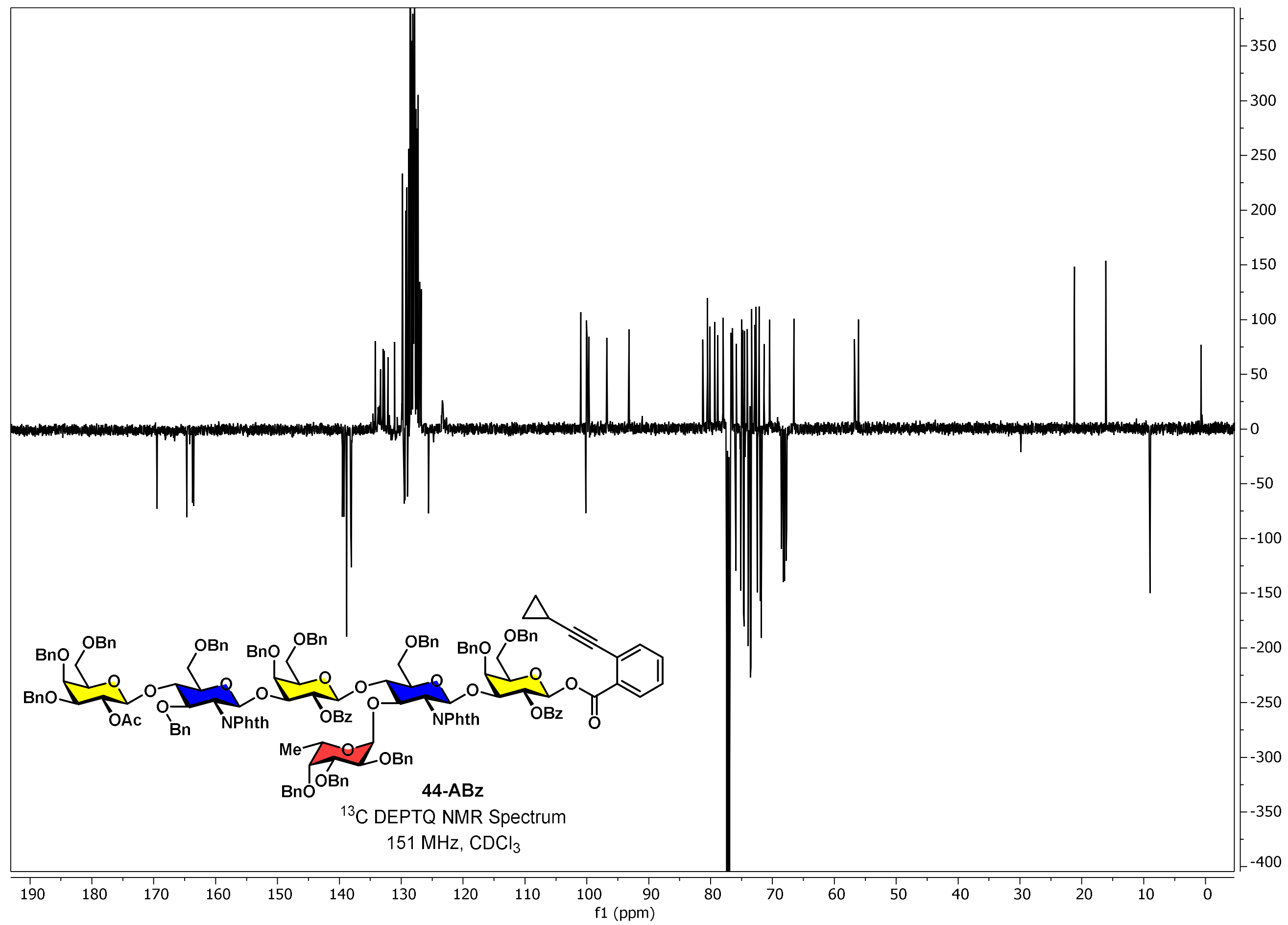
BnO
OBn
BnO
OAc
OBn
Bn
NPhth
BnO
OBn
OBz
OBn
NPhth
OBn
OBz
Me
OBn
BnO
OBn
44-ABz
^{13}C DEPTQ NMR Spectrum
151 MHz, $CDCl_3$
f1 (ppm)

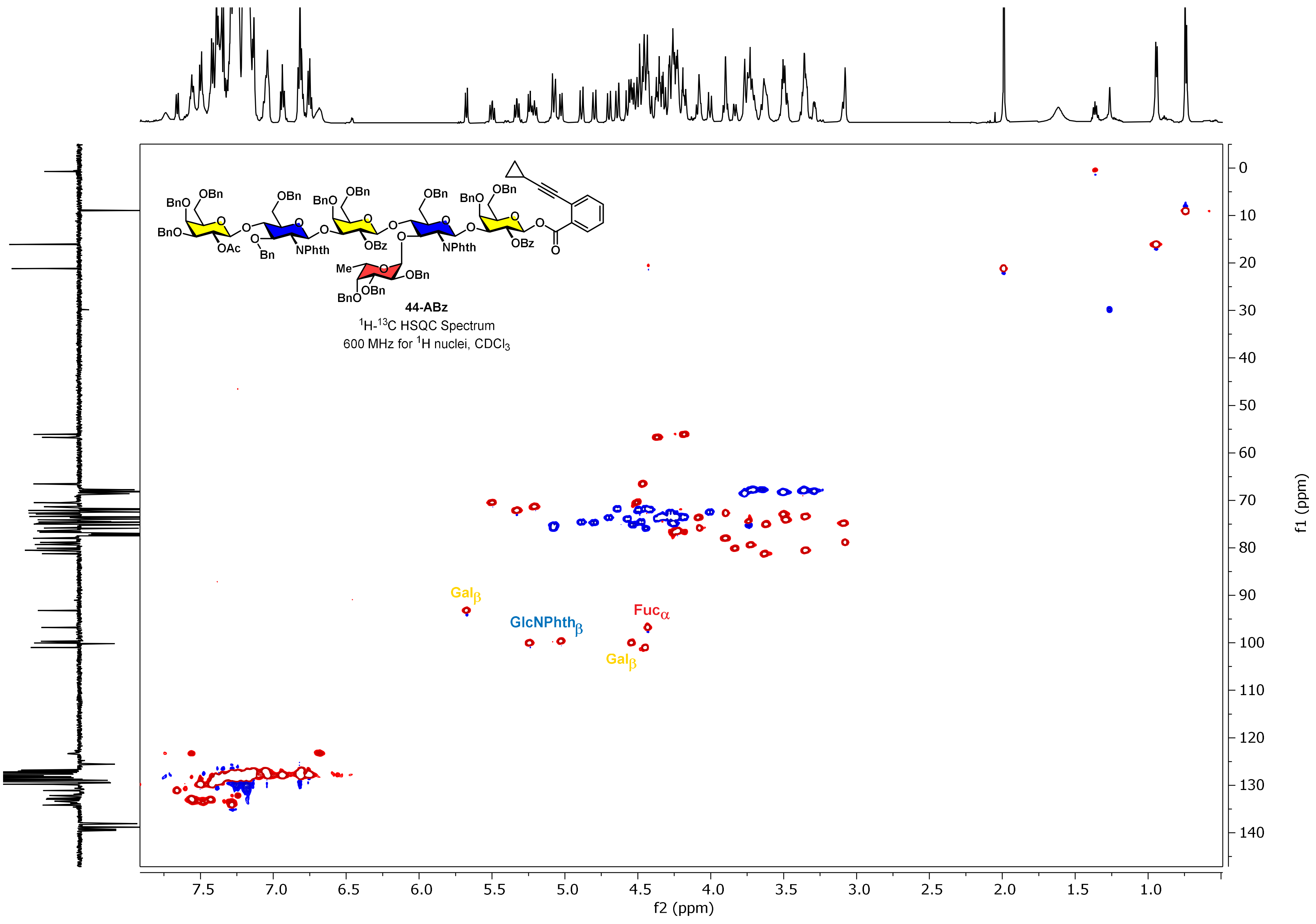
44-ABz
1H-13C HSQC Spectrum
600 MHz for 1H nuclei, CDCl3
Galβ
GlcNPhthβ
Fucα
Galβ
f2 (ppm)
f1 (ppm)

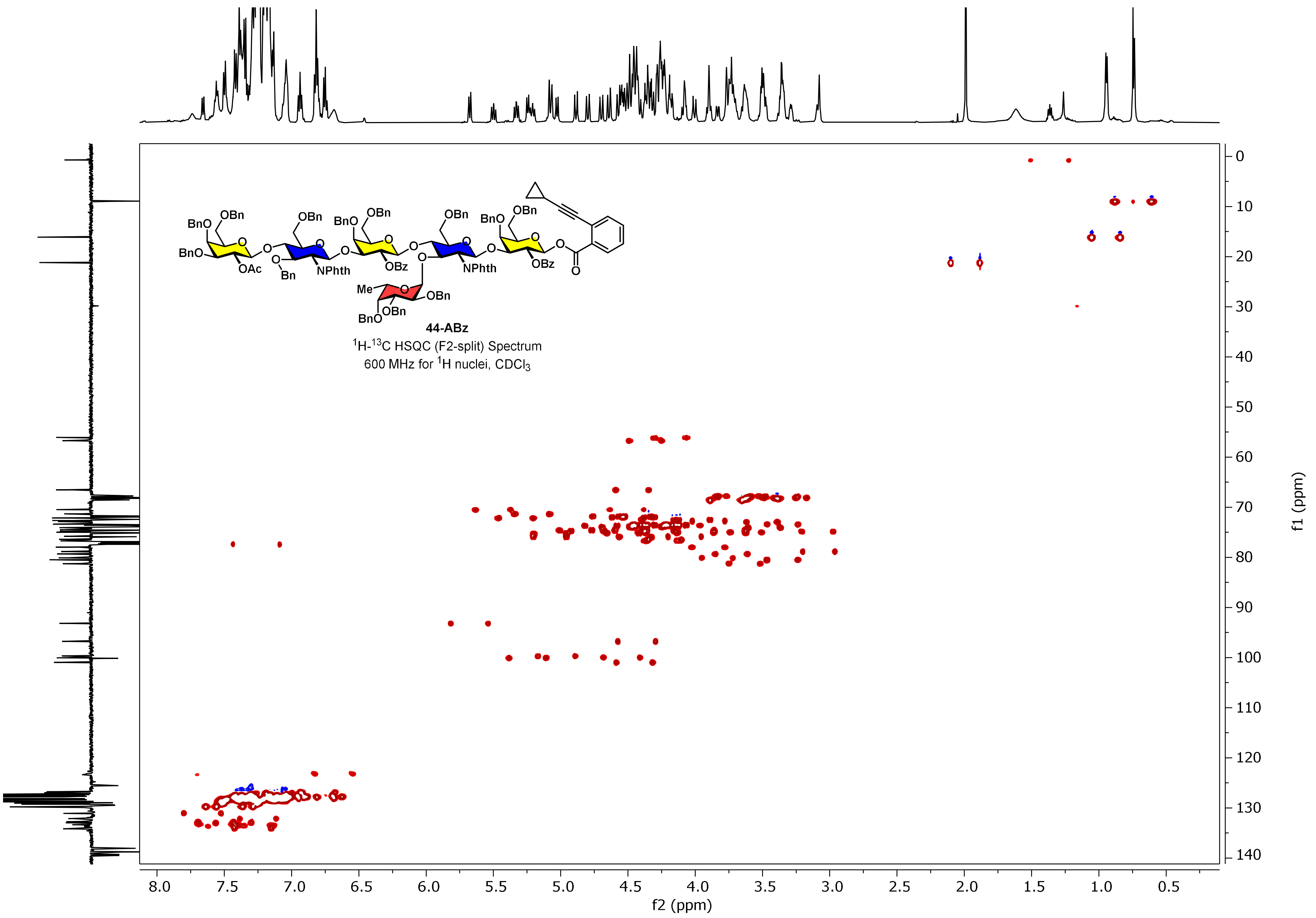
44-ABz
1H-13C HSQC (F2-split) Spectrum
600 MHz for 1H nuclei, CDCl3
f2 (ppm)
f1 (ppm)

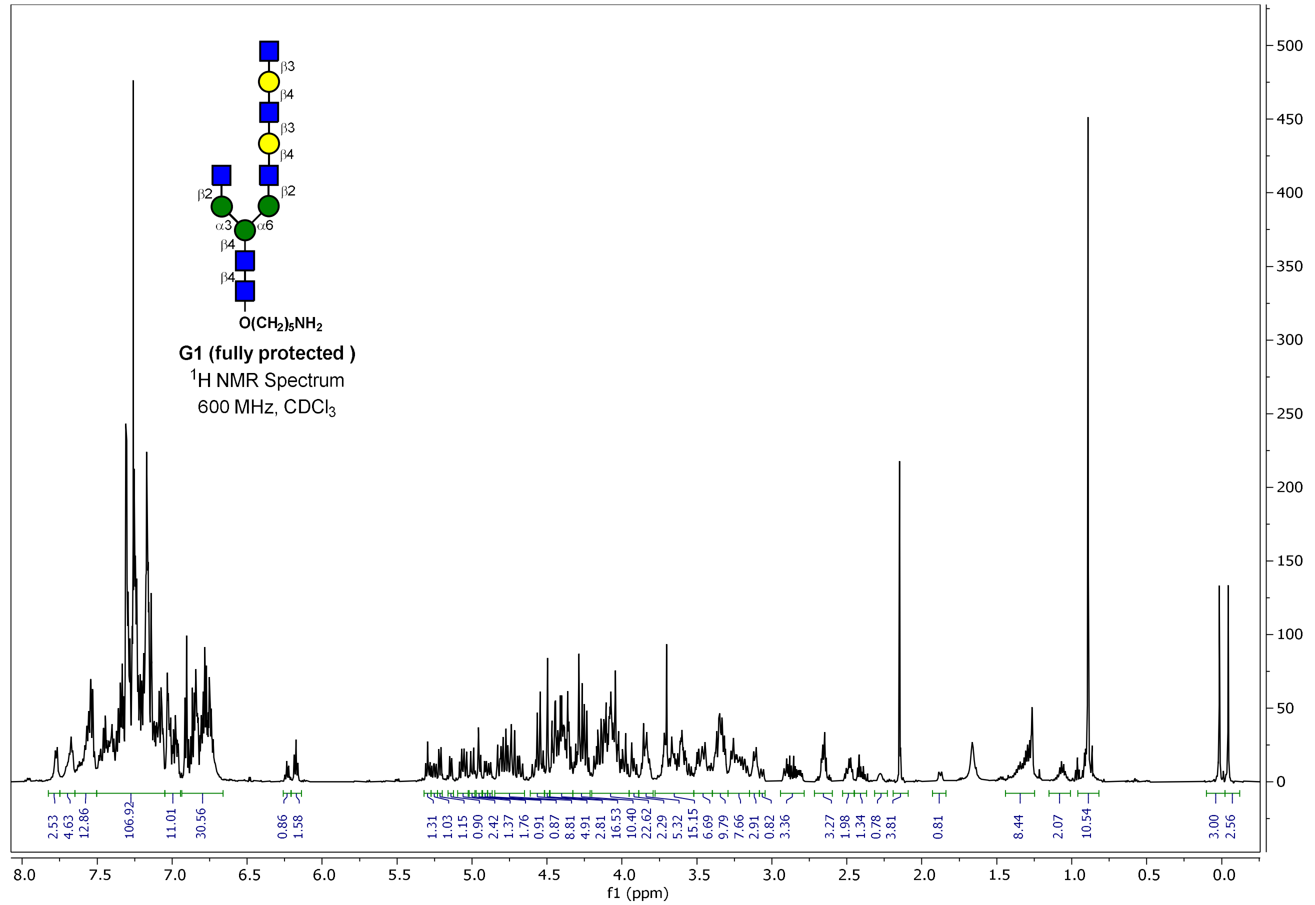
O(CH2)5NH2
G1 (fully protected)
1H NMR Spectrum
600 MHz, CDCl3
f1 (ppm)

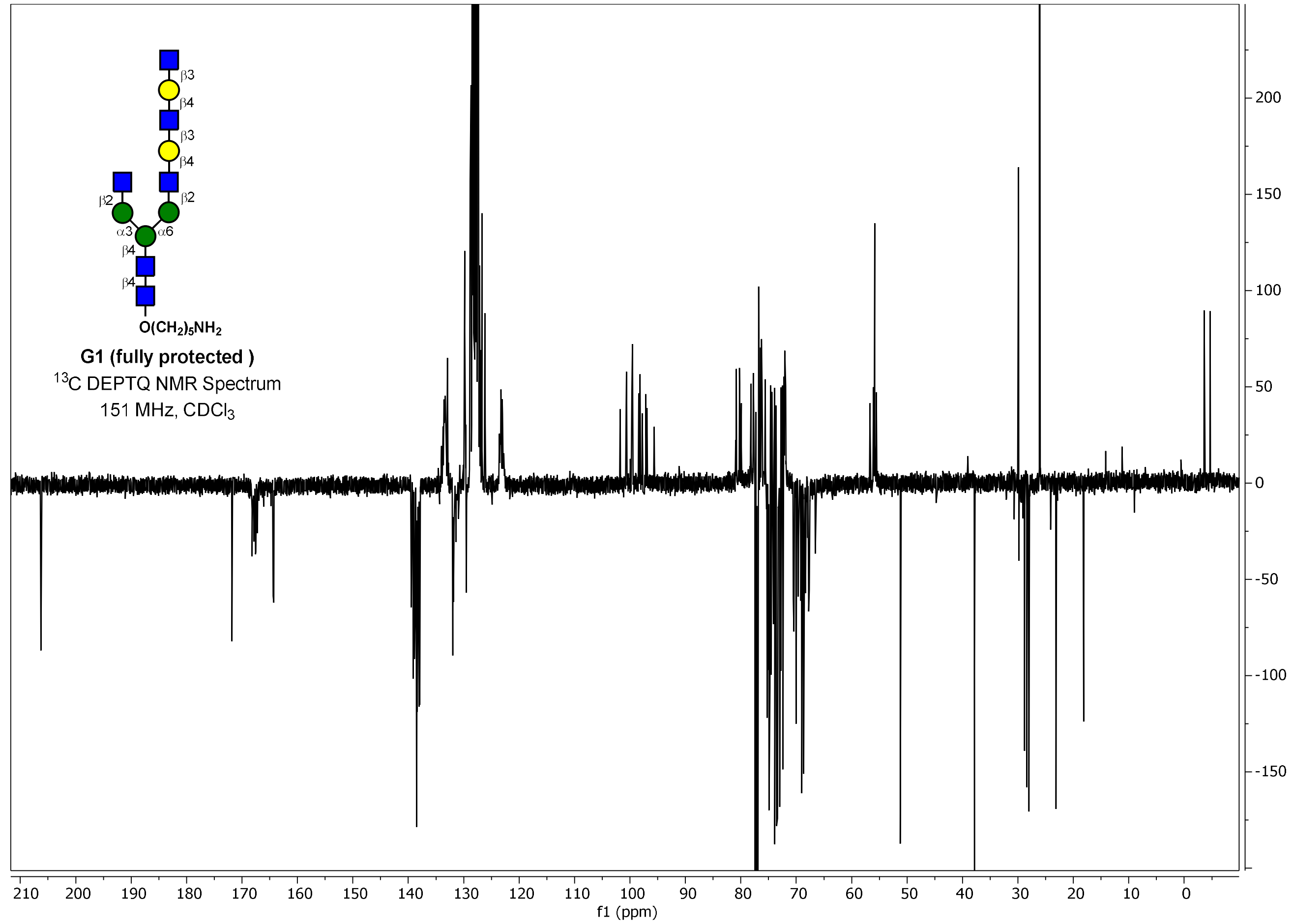
O(CH2)5NH2
G1 (fully protected)
13C DEPTQ NMR Spectrum
151 MHz, CDCl3
f1 (ppm)

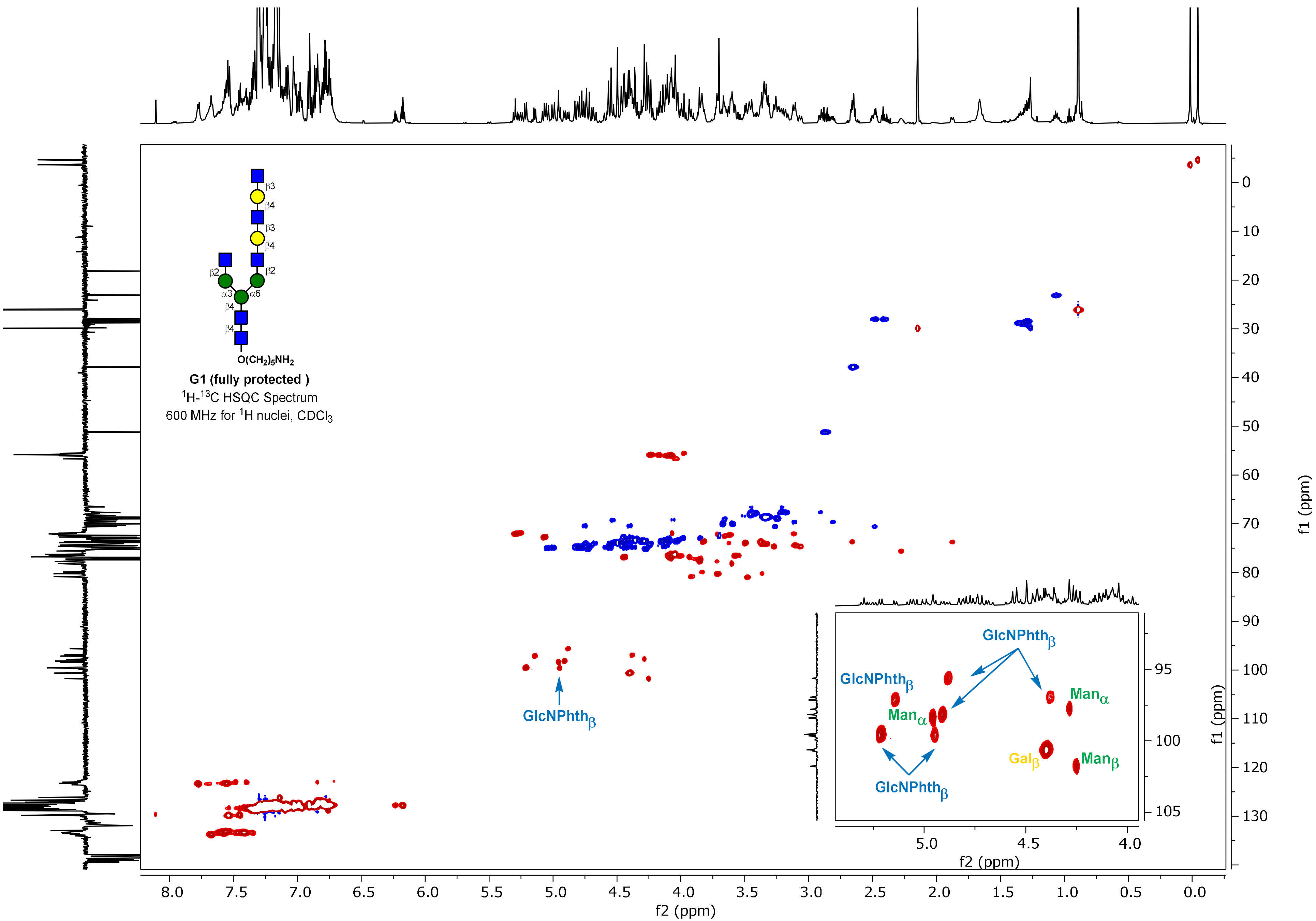

O(CH2)5NH2
G1 (fully protected)
1H-13C HSQC Spectrum
600 MHz for 1H nuclei, CDCl3
GlcNPhthβ
GlcNPhthβ
GlcNPhthβ
Manα
Manα
GlcNPhthβ
Galβ
Manβ
f2 (ppm)
f1 (ppm)

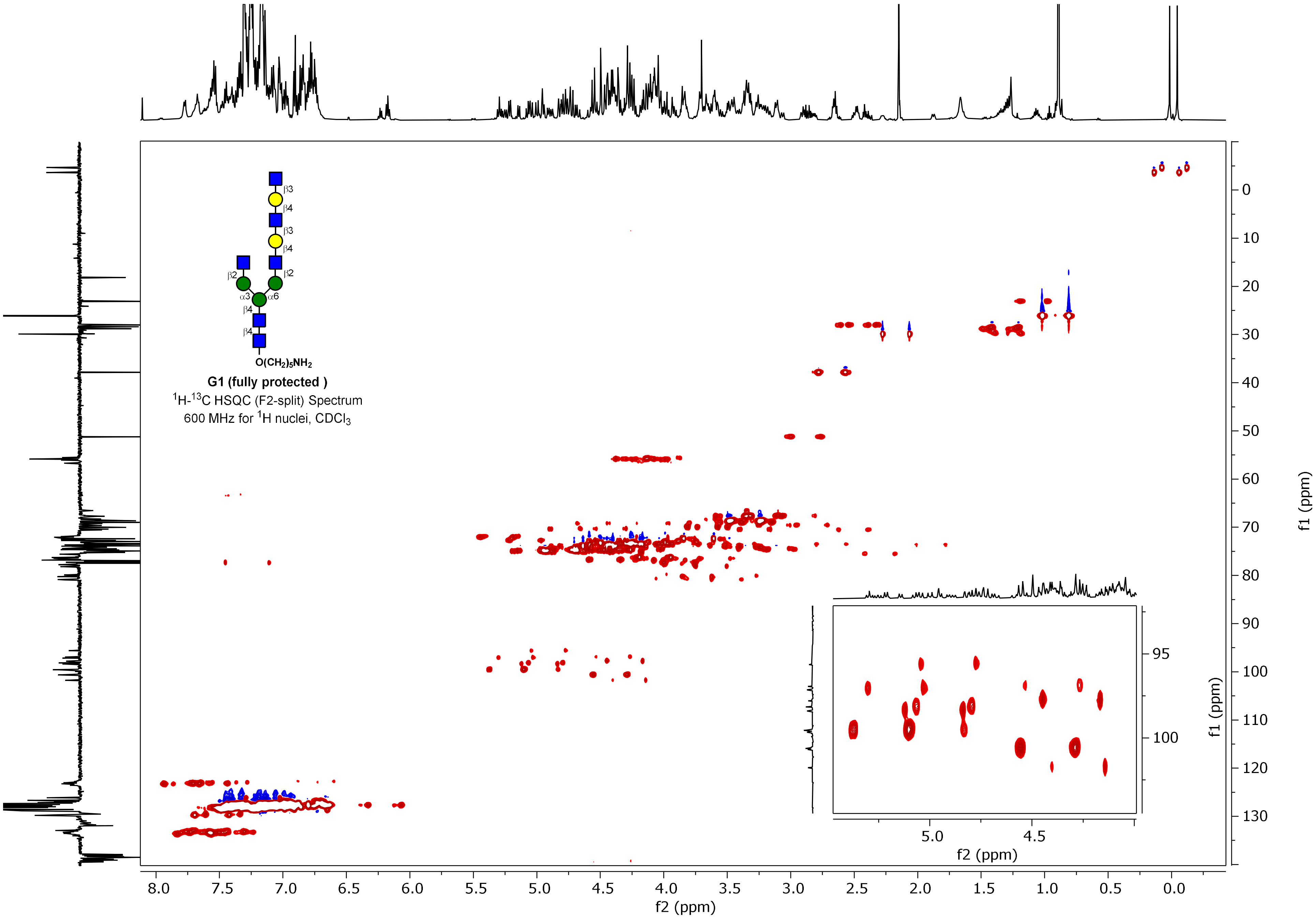
β3
β4
β3
β4
β2
β2
α3
α6
β4
β4
O(CH2)5NH2
G1 (fully protected)
1H-13C HSQC (F2-split) Spectrum
600 MHz for 1H nuclei, CDCl3
f2 (ppm)
f1 (ppm)

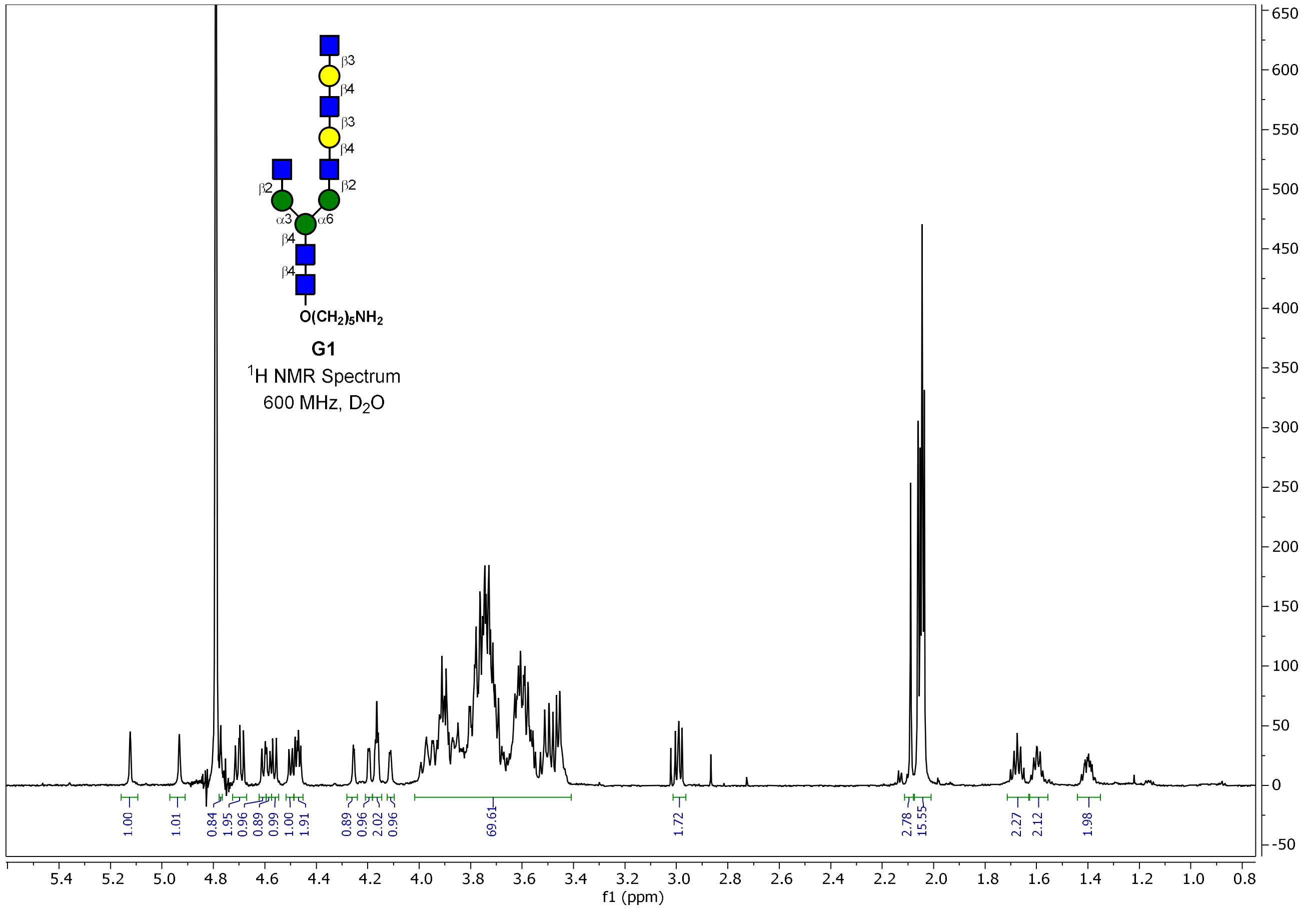
β3
β4
β3
β4
β2
β2
α3
α6
β4
β4
O(CH2)5NH2
G1
1H NMR Spectrum
600 MHz, D2O
1.00
1.01
0.84
1.95
0.96
0.89
0.99
1.00
1.91
0.89
0.96
2.02
0.96
69.61
1.72
2.78
15.55
2.27
2.12
1.98
f1 (ppm)

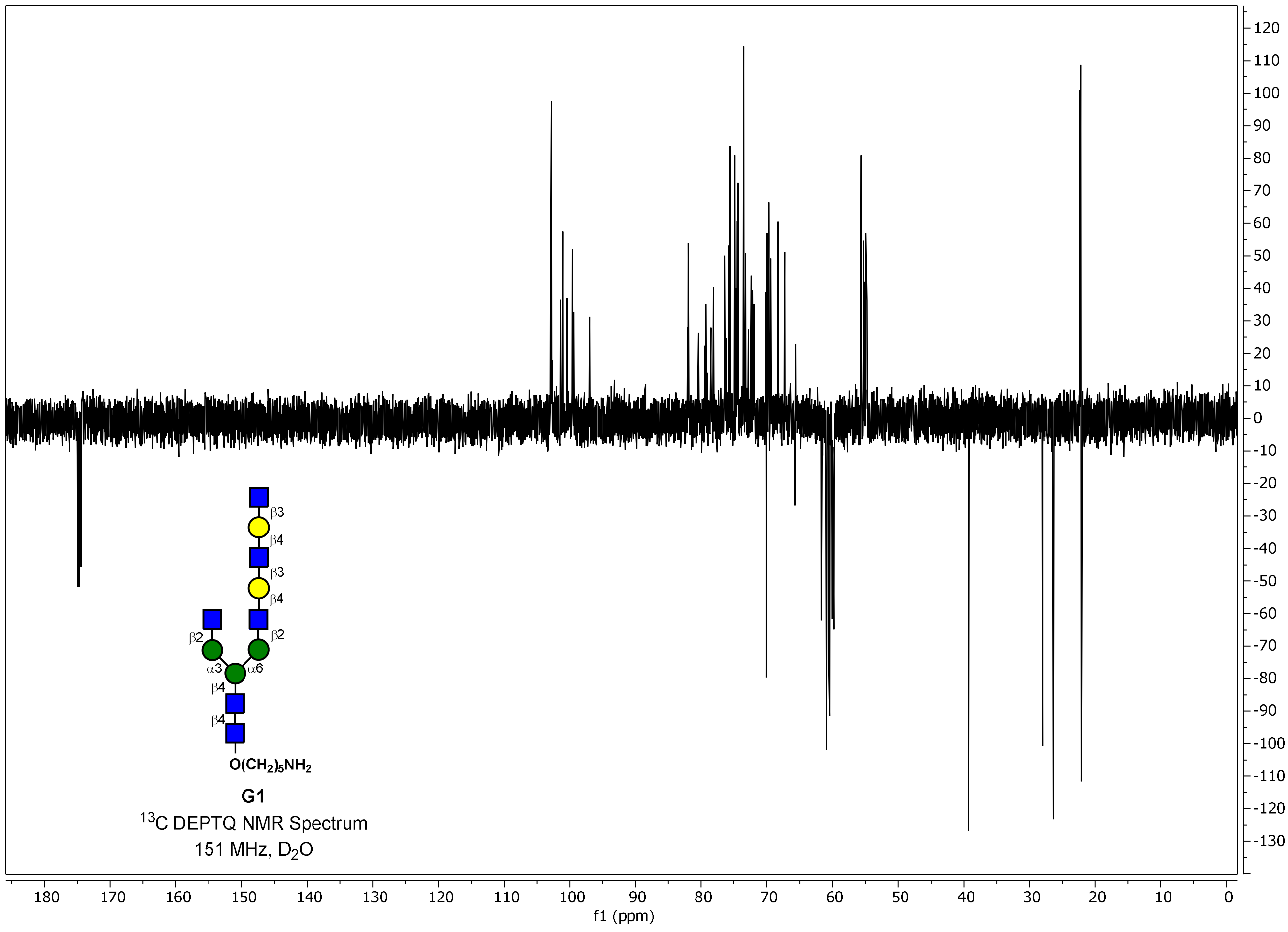
β3
β4
β3
β4
β2
β2
α3
α6
β4
β4
O(CH2)5NH2
G1
13C DEPTQ NMR Spectrum
151 MHz, D2O
f1 (ppm)

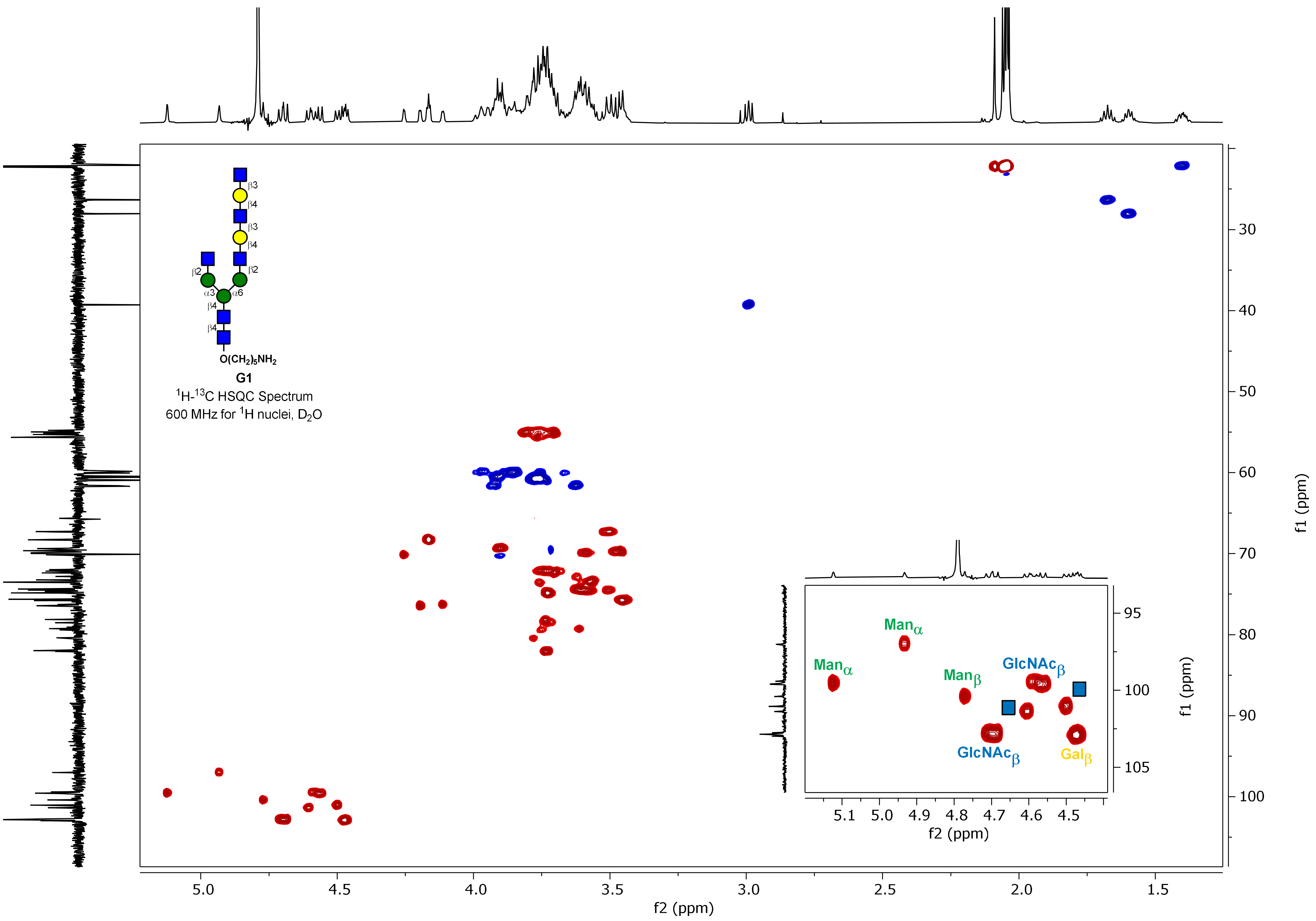
β3
β4
β3
β4
β2
β2
α3
α6
β4
β4
O(CH2)5NH2
G1
1H-13C HSQC Spectrum
600 MHz for 1H nuclei, D2O
Manα
Manα
Manβ
GlcNAcβ
GlcNAcβ
Galβ
f2 (ppm)
f1 (ppm)
f2 (ppm)
f1 (ppm)

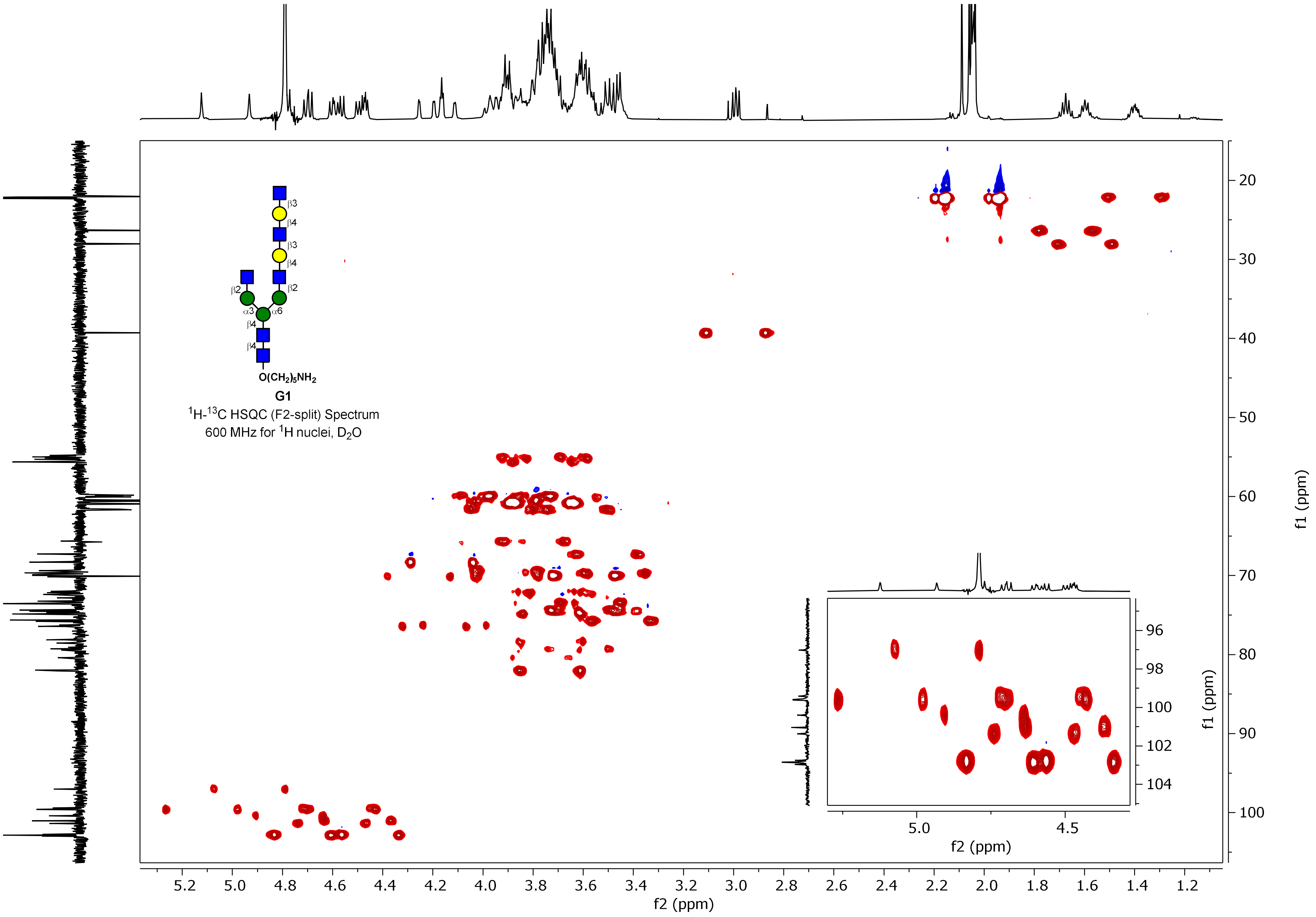

β3
β4
β3
β4
β2
β2
α3
α6
β4
β4
O(CH2)5NH2
G1
1H-13C HSQC (F2-split) Spectrum
600 MHz for 1H nuclei, D2O
f2 (ppm)
f1 (ppm)

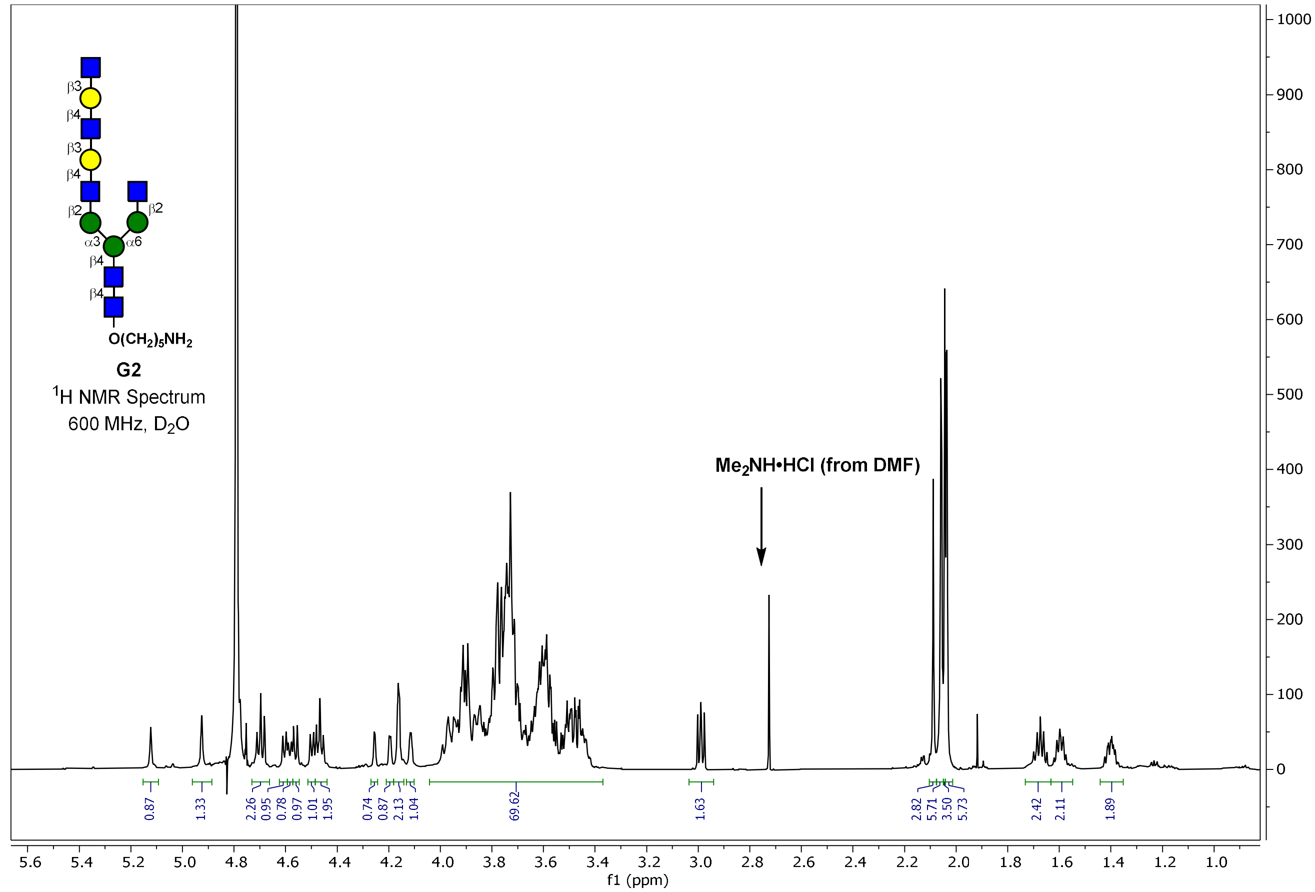
O(CH2)5NH2
G2
1H NMR Spectrum
600 MHz, D2O
Me2NH•HCl (from DMF)
0.87
1.33
2.26
0.95
0.78
0.97
1.01
1.95
0.74
0.87
2.13
1.04
69.62
1.63
2.82
5.71
3.50
5.73
2.42
2.11
1.89
f1 (ppm)

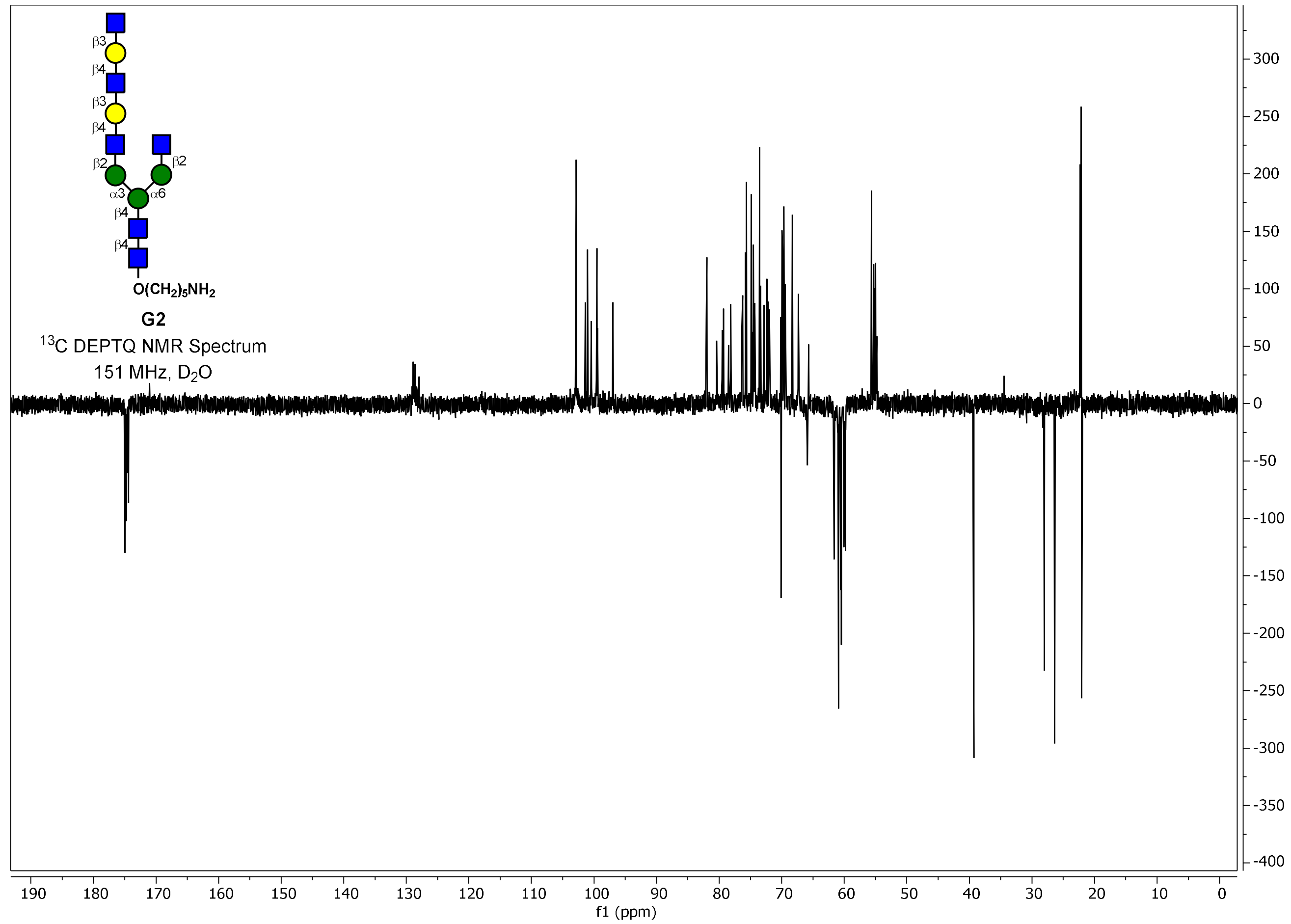
O(CH2)5NH2
G2
13C DEPTQ NMR Spectrum
151 MHz, D2O
f1 (ppm)

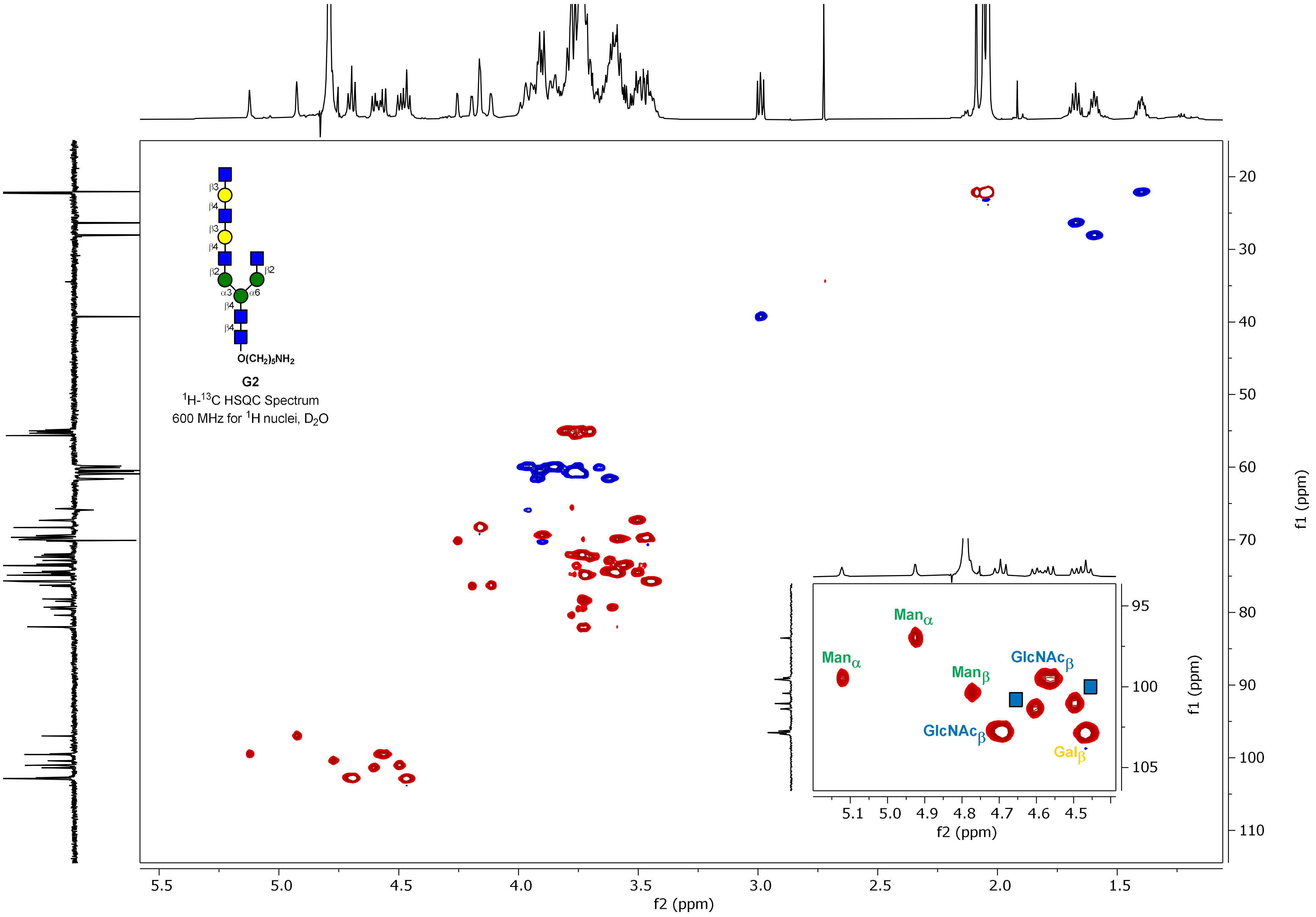

β3
β4
β3
β4
β2
β2
α3
α6
β4
β4
O(CH2)5NH2
G2
1H-13C HSQC Spectrum
600 MHz for 1H nuclei, D2O
Manα
Manα
Manβ
GlcNAcβ
GlcNAcβ
Galβ
5.1 5.0 4.9 4.8 4.7 4.6 4.5
f2 (ppm)
95
100
105
f1 (ppm)
5.5 5.0 4.5 4.0 3.5 3.0 2.5 2.0 1.5
f2 (ppm)
20 30 40 50 60 70 80 90 100 110
f1 (ppm)

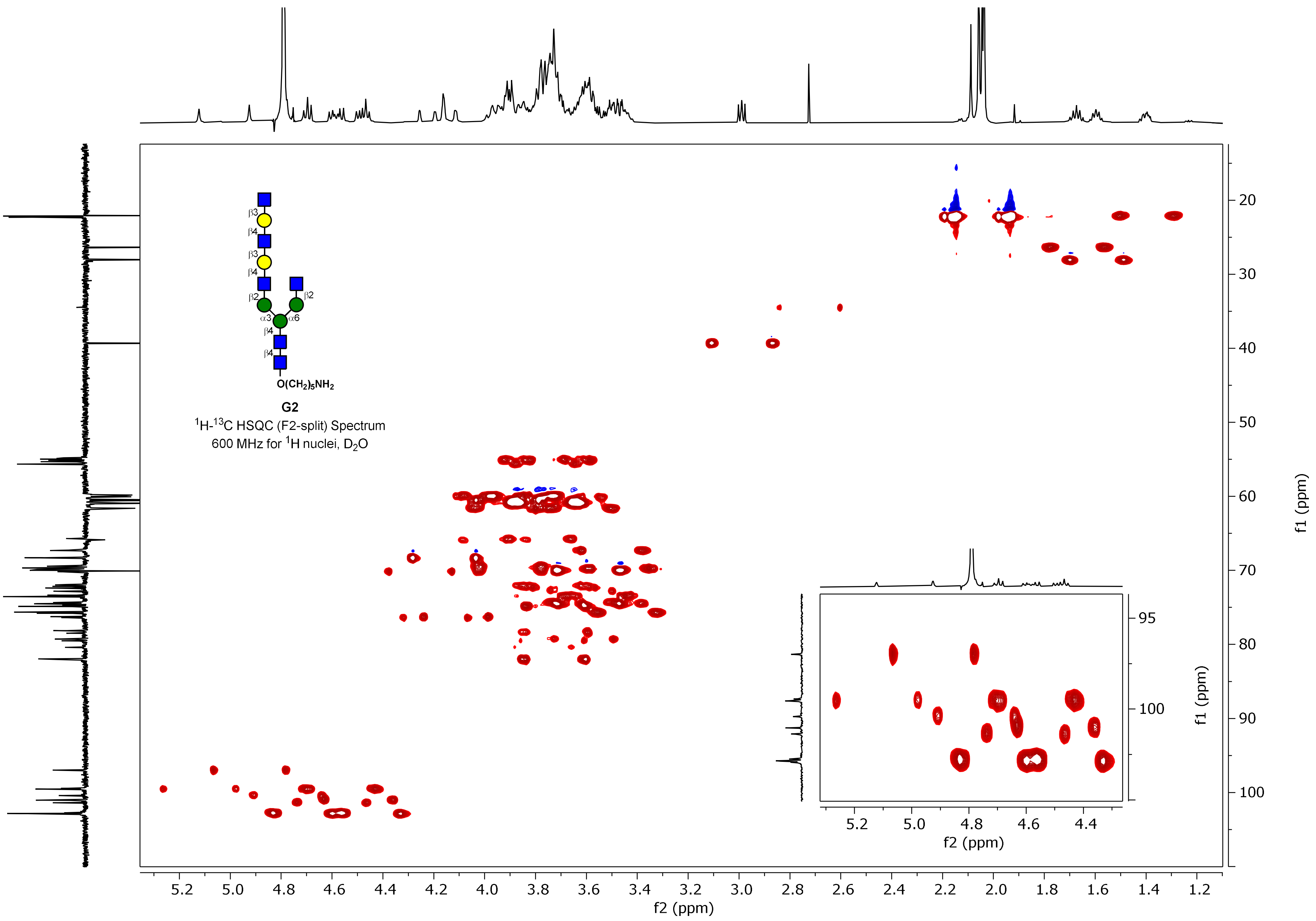
O(CH2)5NH2
G2
1H-13C HSQC (F2-split) Spectrum
600 MHz for 1H nuclei, D2O
f2 (ppm)
f1 (ppm)

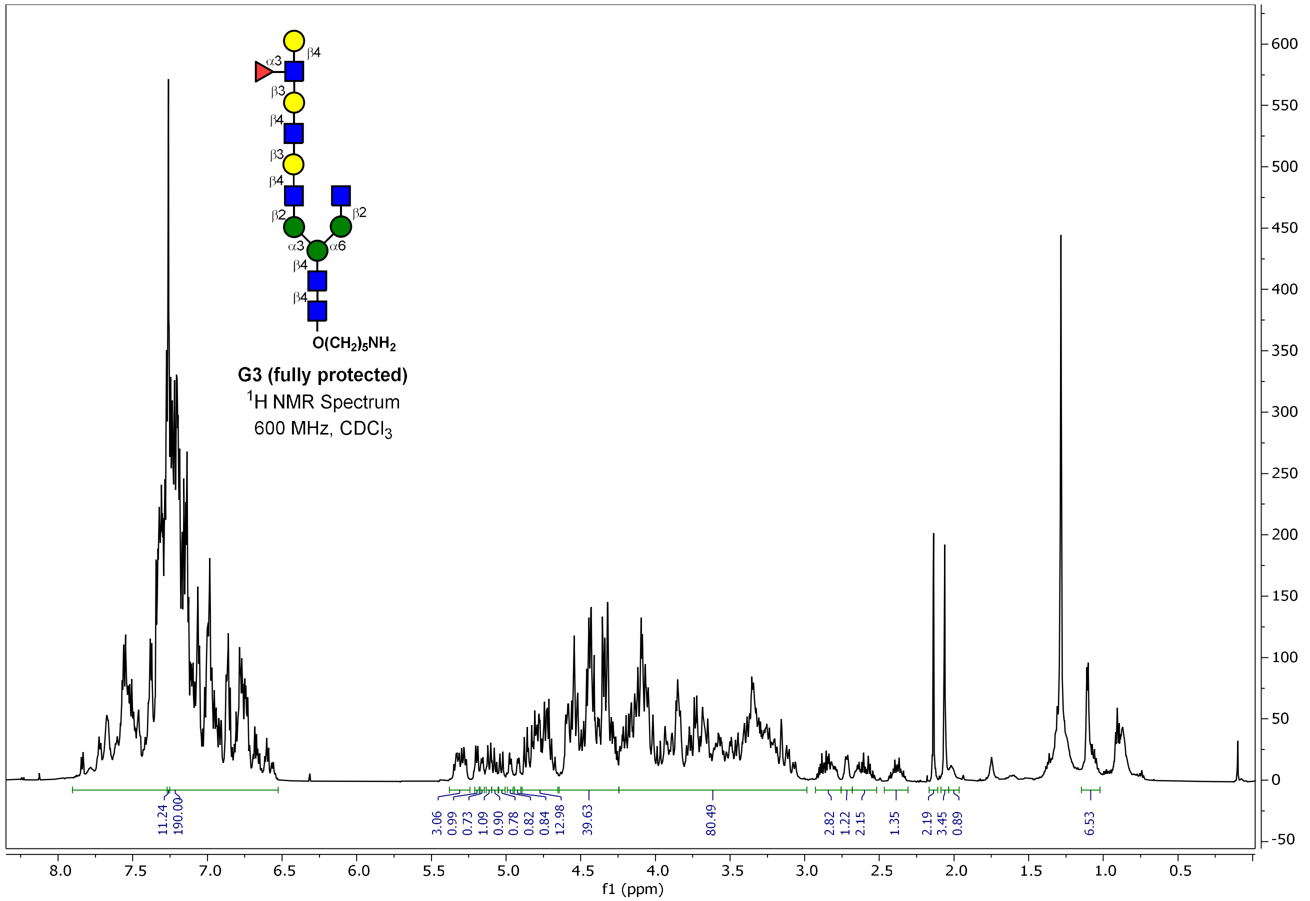
O(CH2)5NH2
G3 (fully protected)
1H NMR Spectrum
600 MHz, CDCl3
f1 (ppm)

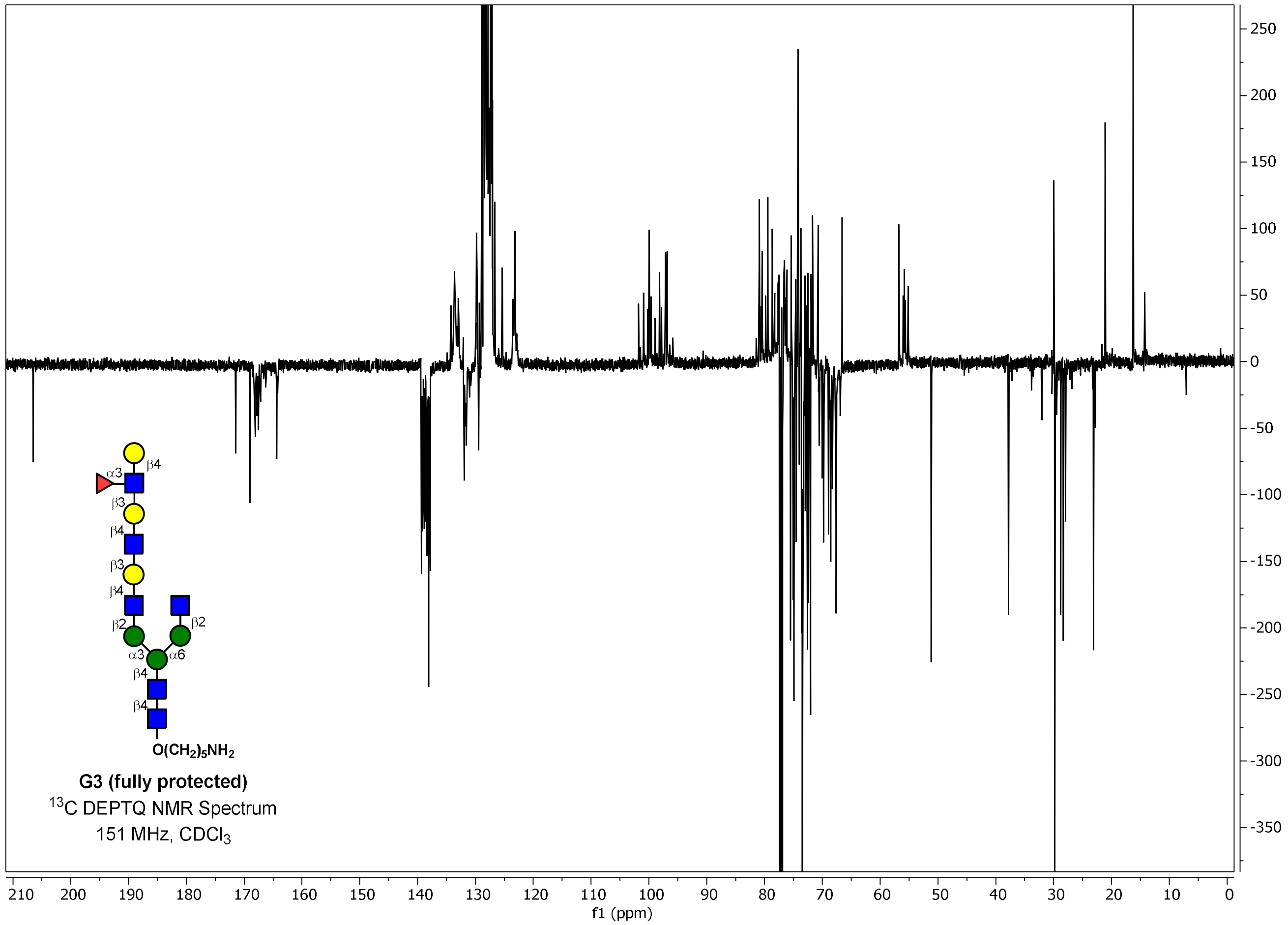
O(CH2)5NH2
G3 (fully protected)
13C DEPTQ NMR Spectrum
151 MHz, CDCl3
f1 (ppm)

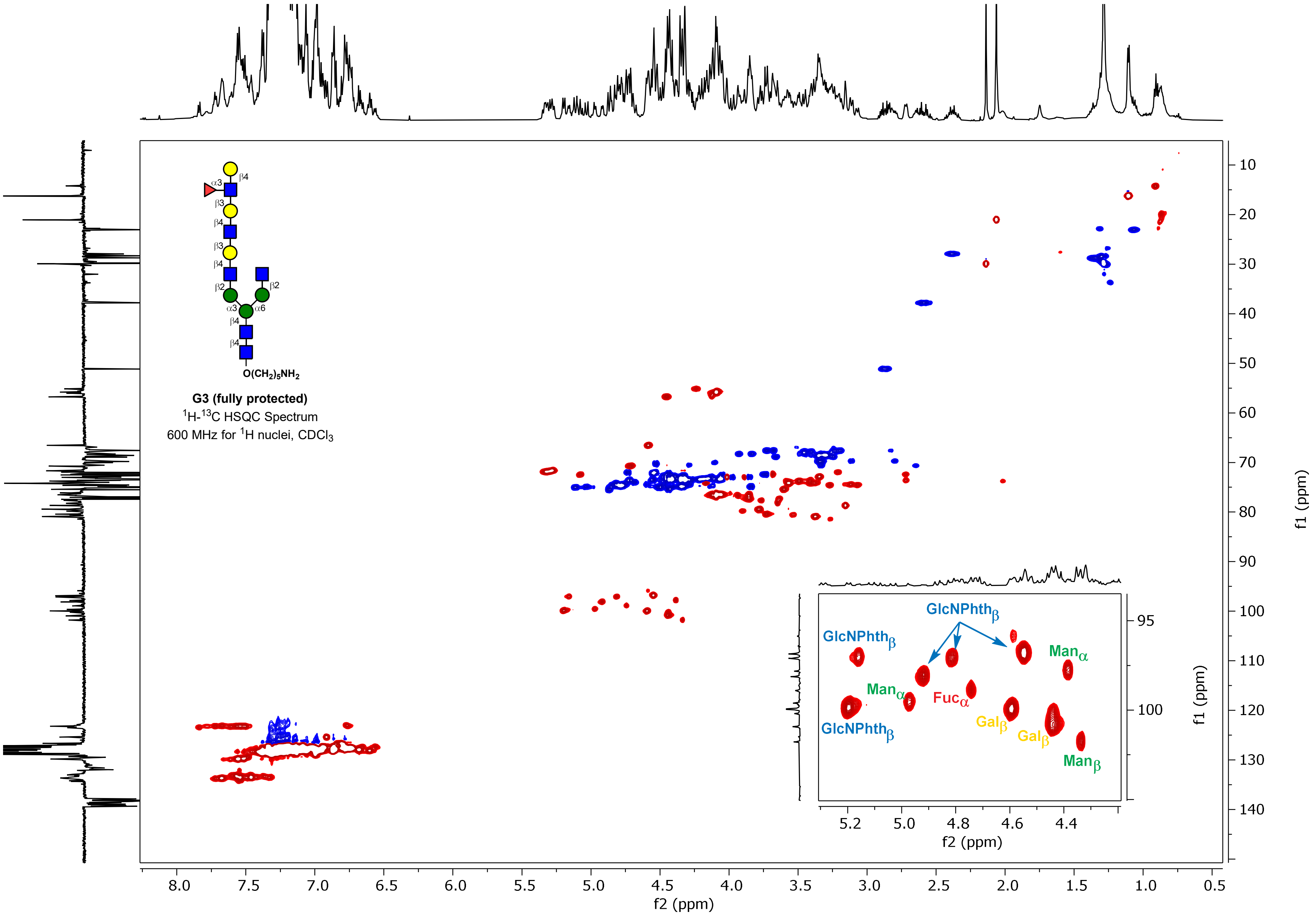

G3 (fully protected)
^{1}H-^{13}C HSQC Spectrum
600 MHz for ^{1}H nuclei, $CDCl_3$
$O(CH_2)_5NH_2$
GlcNPhthβ
GlcNPhthβ
Manα
Manα
Fucα
Galβ
Galβ
Manβ
GlcNPhthβ
f2 (ppm)
f1 (ppm)

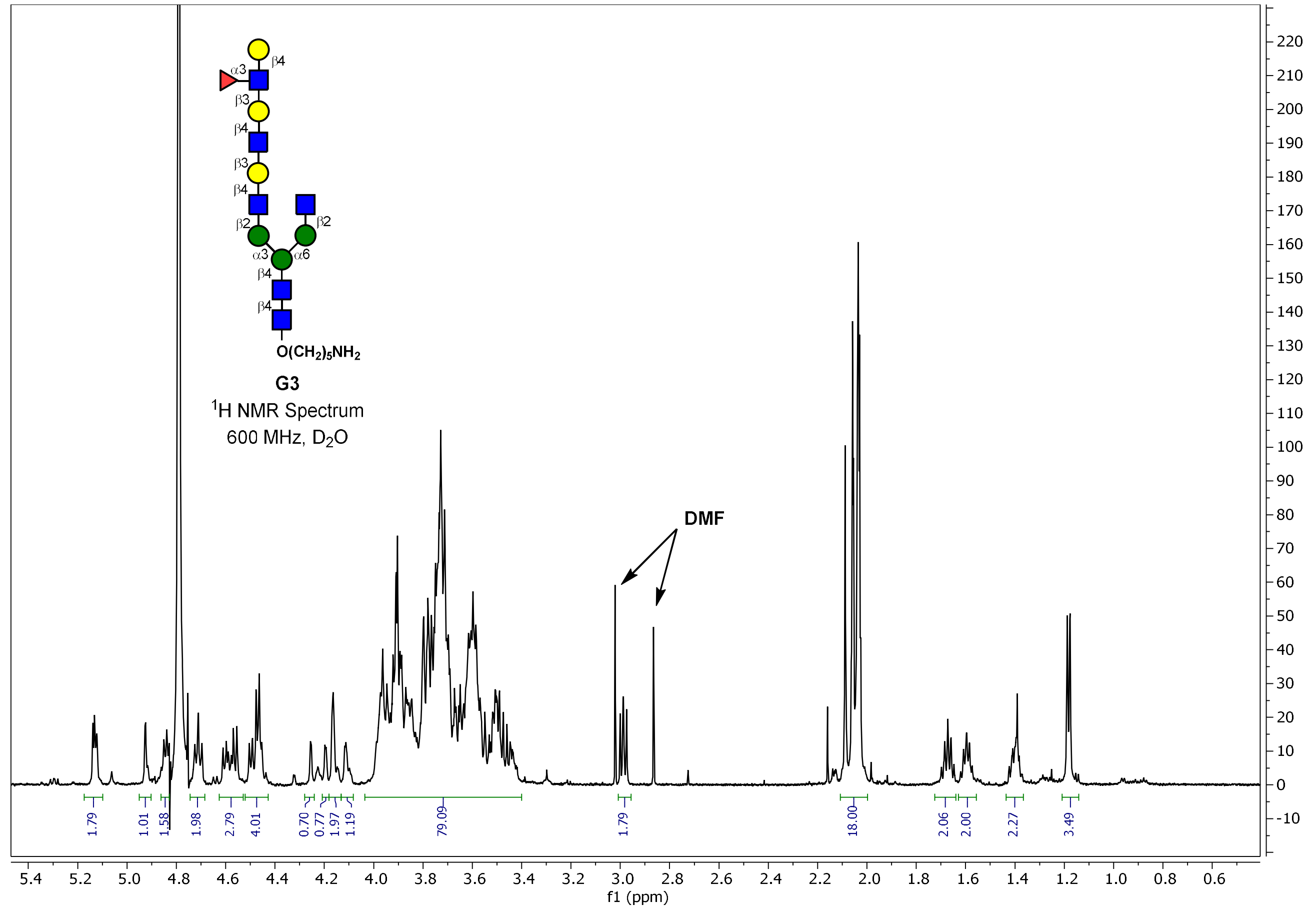

O(CH2)5NH2
G3
1H NMR Spectrum
600 MHz, D2O
DMF
f1 (ppm)

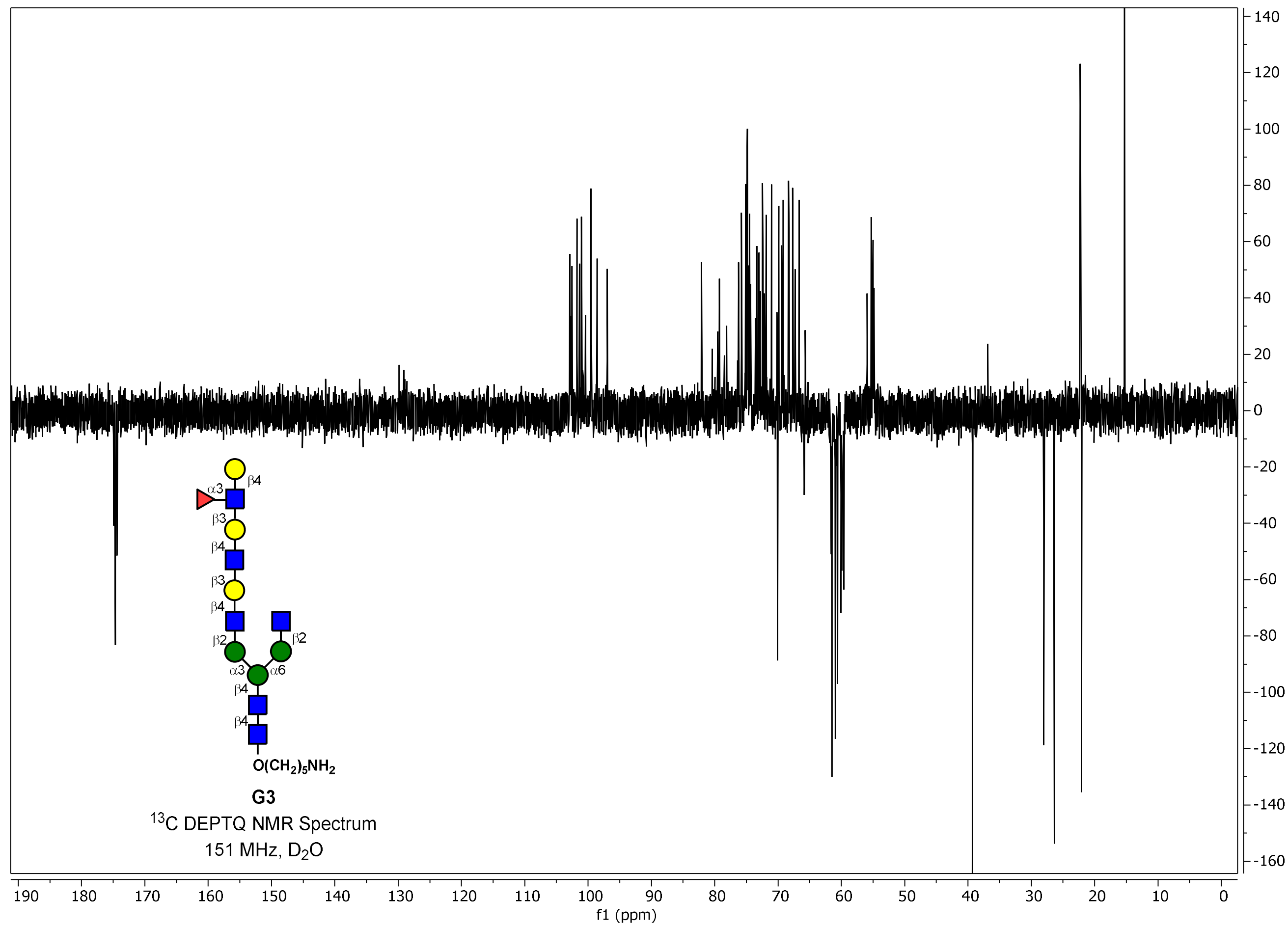

O(CH2)5NH2
G3
13C DEPTQ NMR Spectrum
151 MHz, D2O
f1 (ppm)

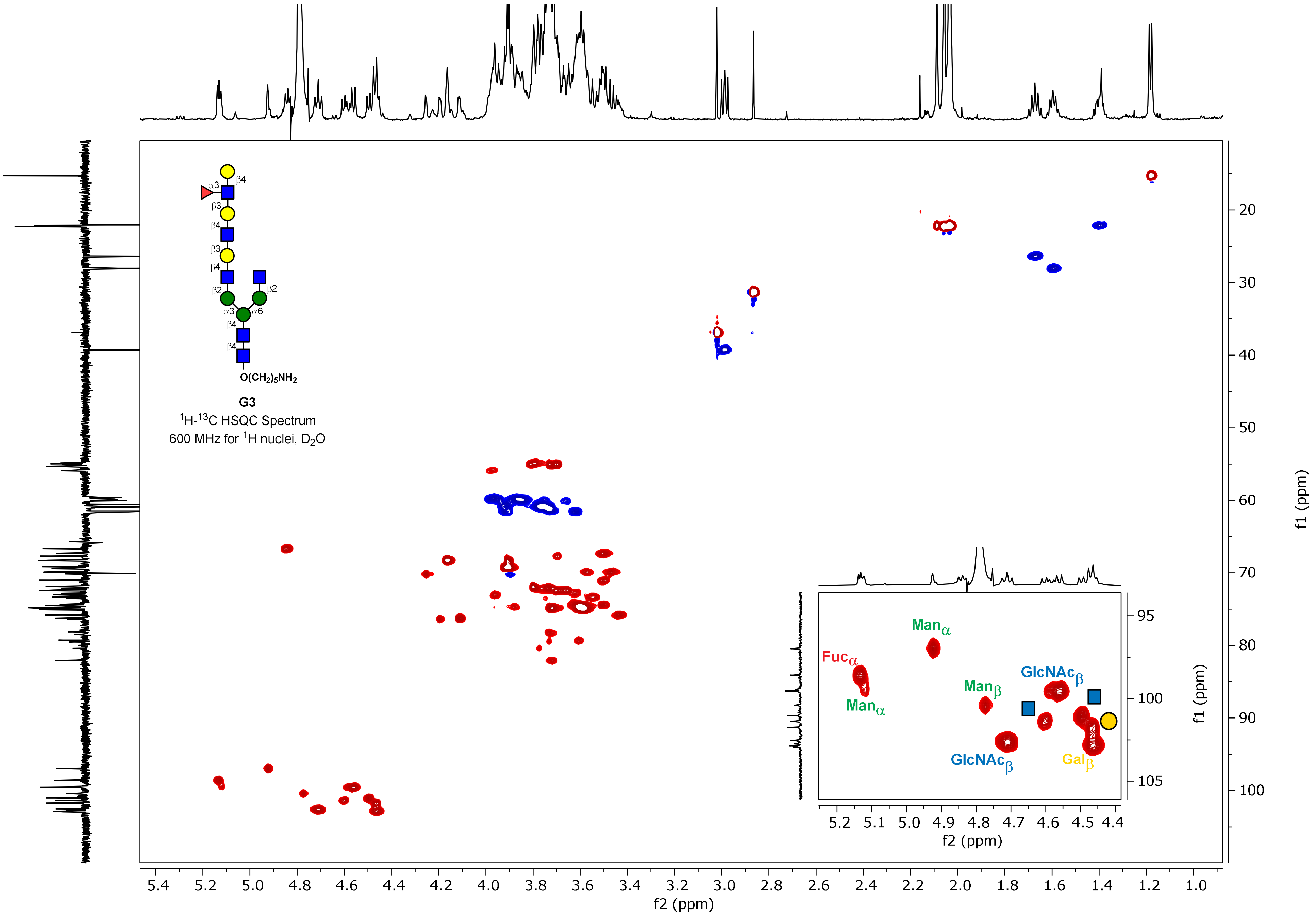

G3
1H-13C HSQC Spectrum
600 MHz for 1H nuclei, D2O
O(CH2)5NH2
Manα
Fucα
Manα
Manβ
GlcNAcβ
GlcNAcβ
Galβ
f2 (ppm)
f1 (ppm)

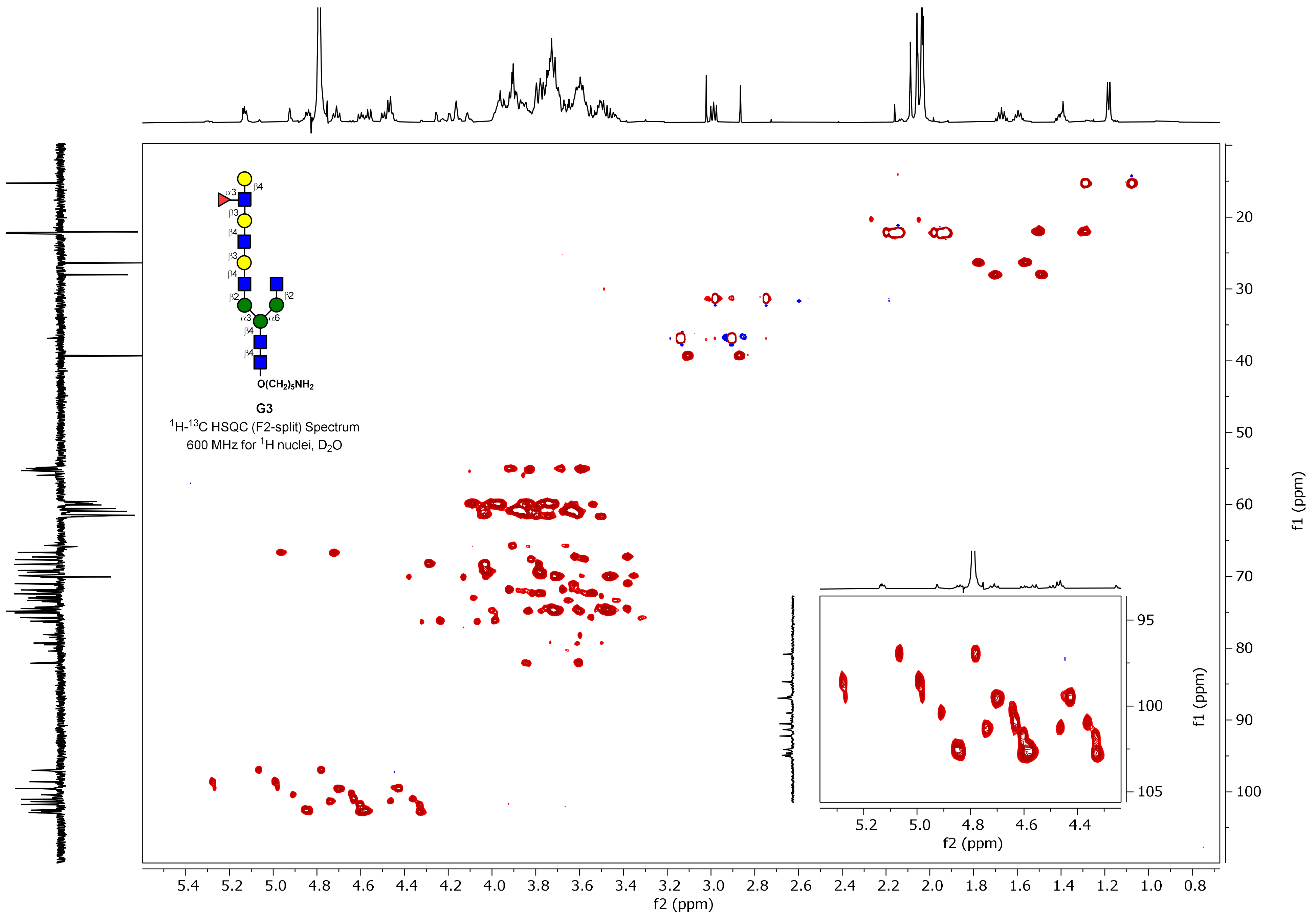

α3
β4
β3
β4
β3
β4
β2
β2
α3
α6
β4
β4
O(CH2)5NH2
G3
1H-13C HSQC (F2-split) Spectrum
600 MHz for 1H nuclei, D2O
f2 (ppm)
f1 (ppm)

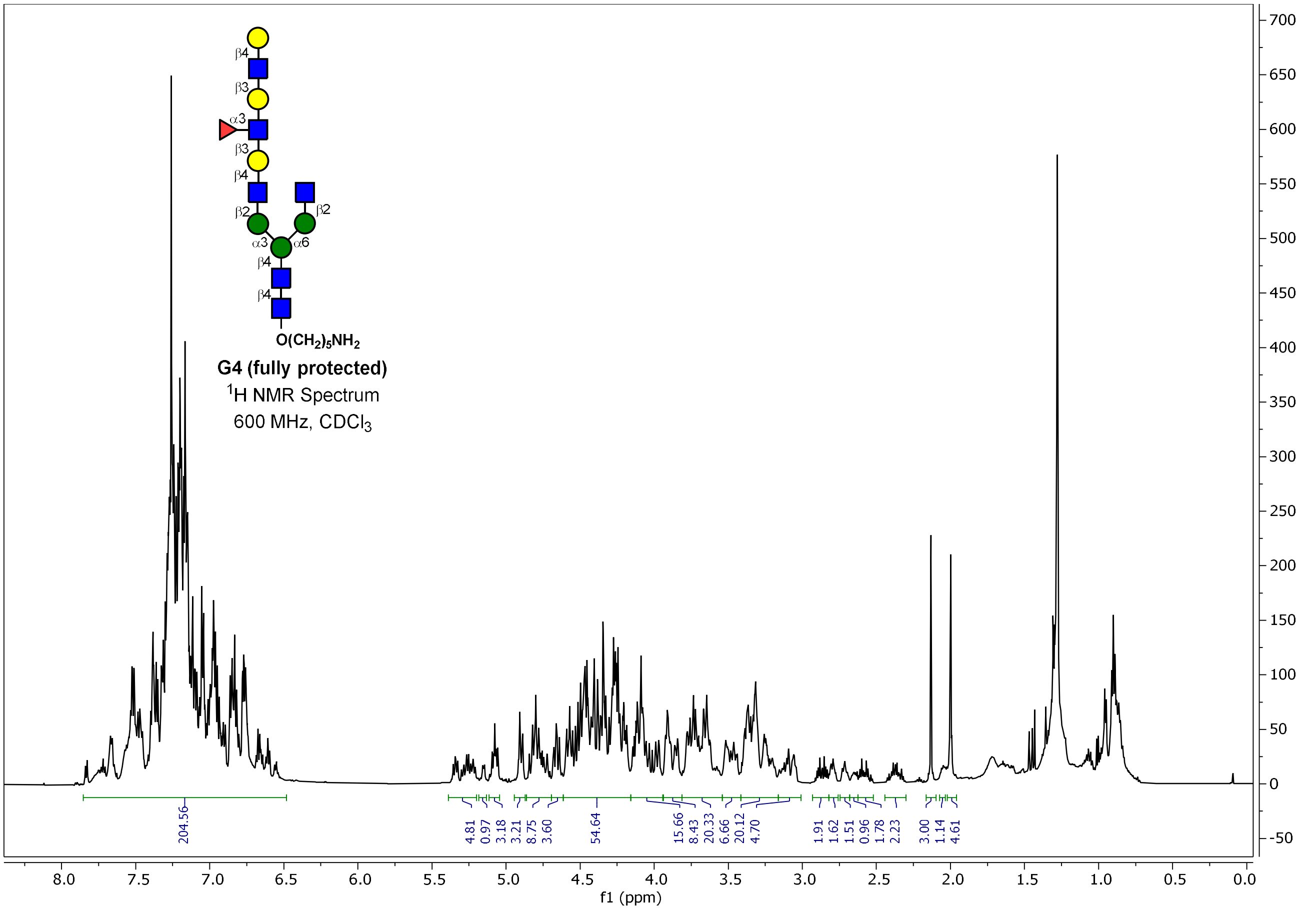
β4
β3
α3
β3
β4
β2
β2
α3
α6
β4
β4
$O(CH_2)_5NH_2$
G4 (fully protected)
^1H NMR Spectrum
600 MHz, $CDCl_3$
204.56
4.81
0.97
3.18
3.21
8.75
3.60
54.64
15.66
8.43
20.33
6.66
20.12
4.70
1.91
1.62
1.51
0.96
1.78
2.23
3.00
1.14
4.61
f1 (ppm)

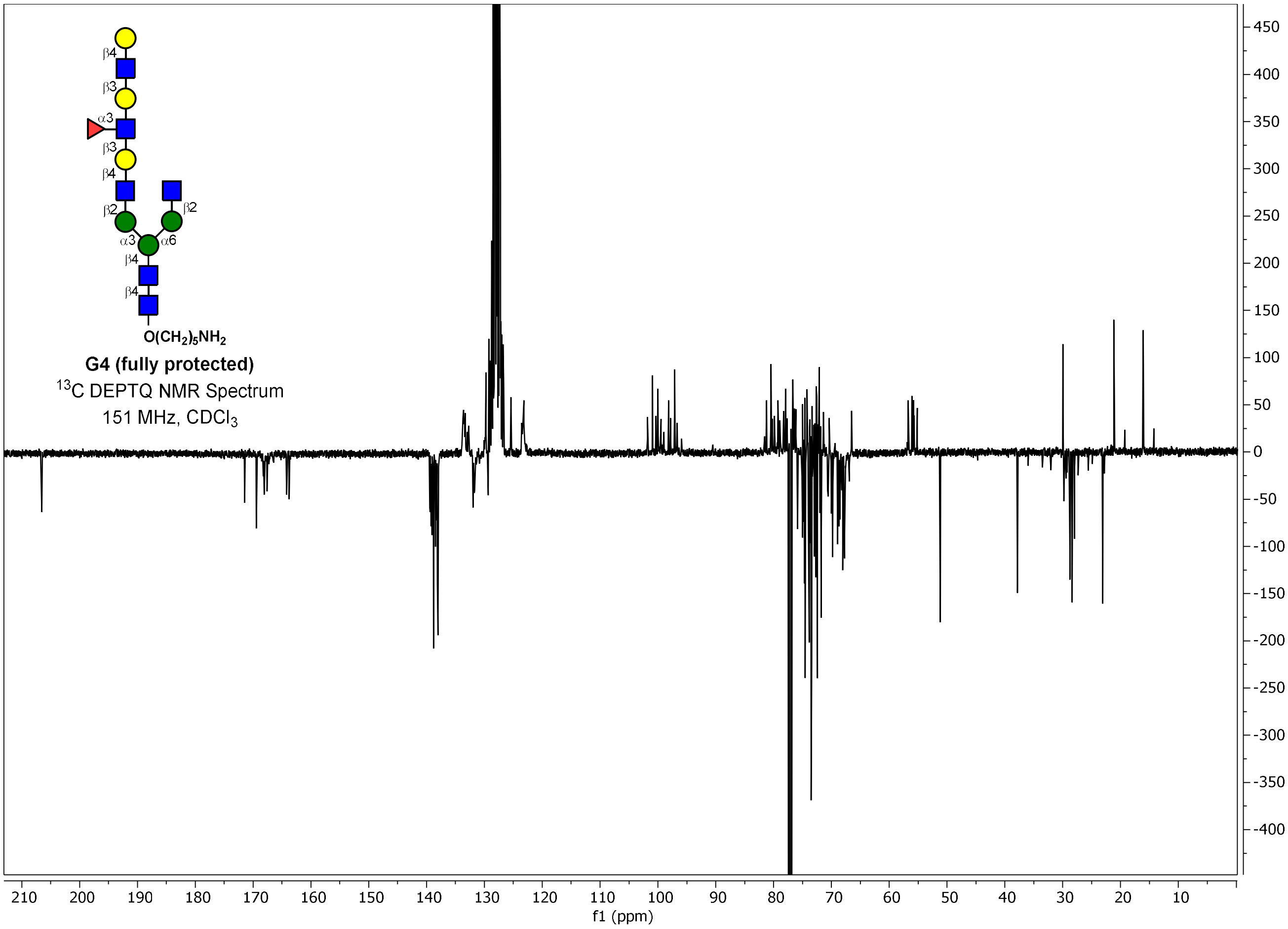
β4
β3
α3
β3
β4
β2
β2
α3
α6
β4
β4
$O(CH_2)_5NH_2$
G4 (fully protected)
^{13}C DEPTQ NMR Spectrum
151 MHz, $CDCl_3$
f1 (ppm)

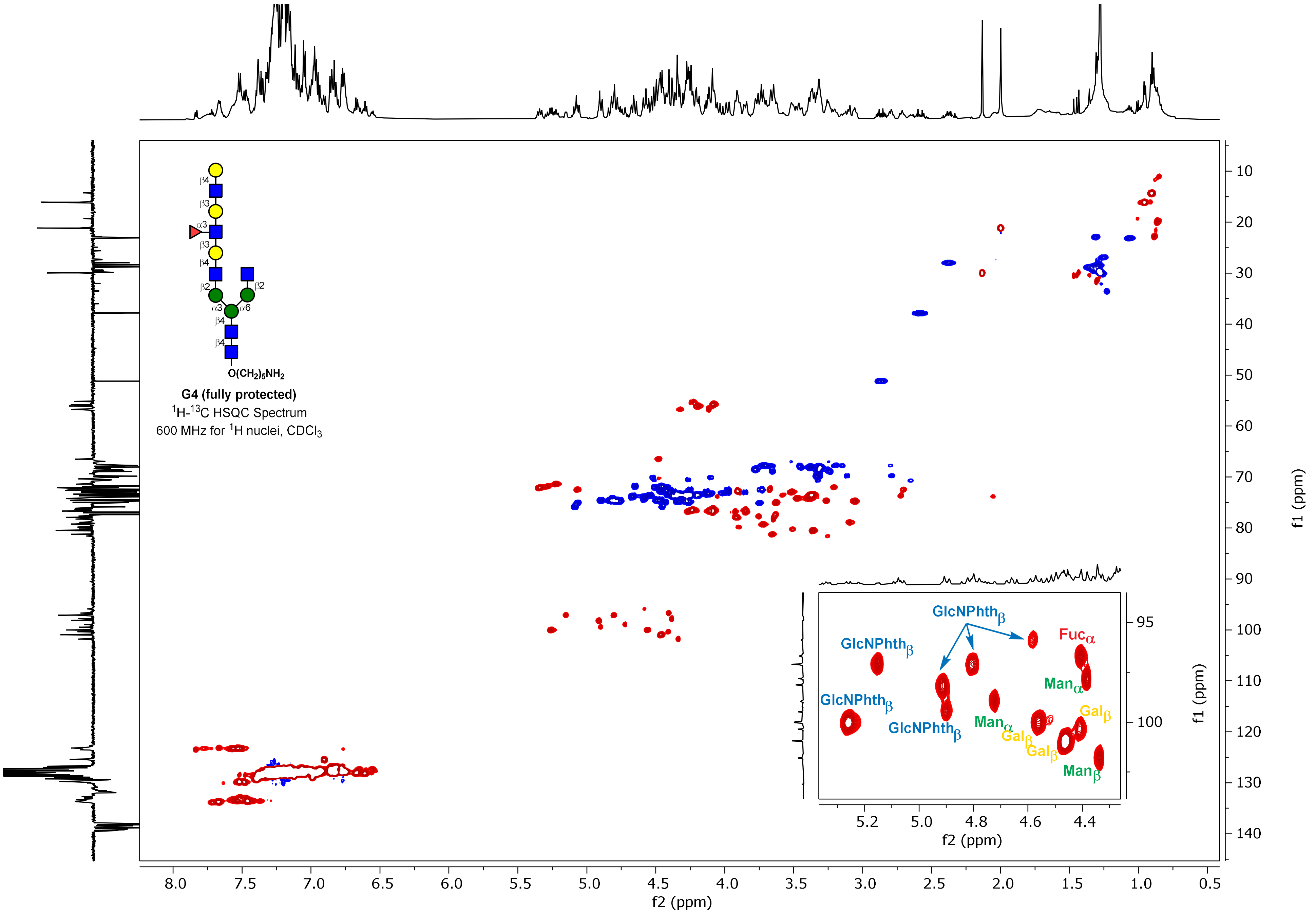

O(CH2)5NH2
G4 (fully protected)
1H-13C HSQC Spectrum
600 MHz for 1H nuclei, CDCl3
GlcNPhthβ
Fucα
Manα
Galβ
Manβ
f1 (ppm)
f2 (ppm)

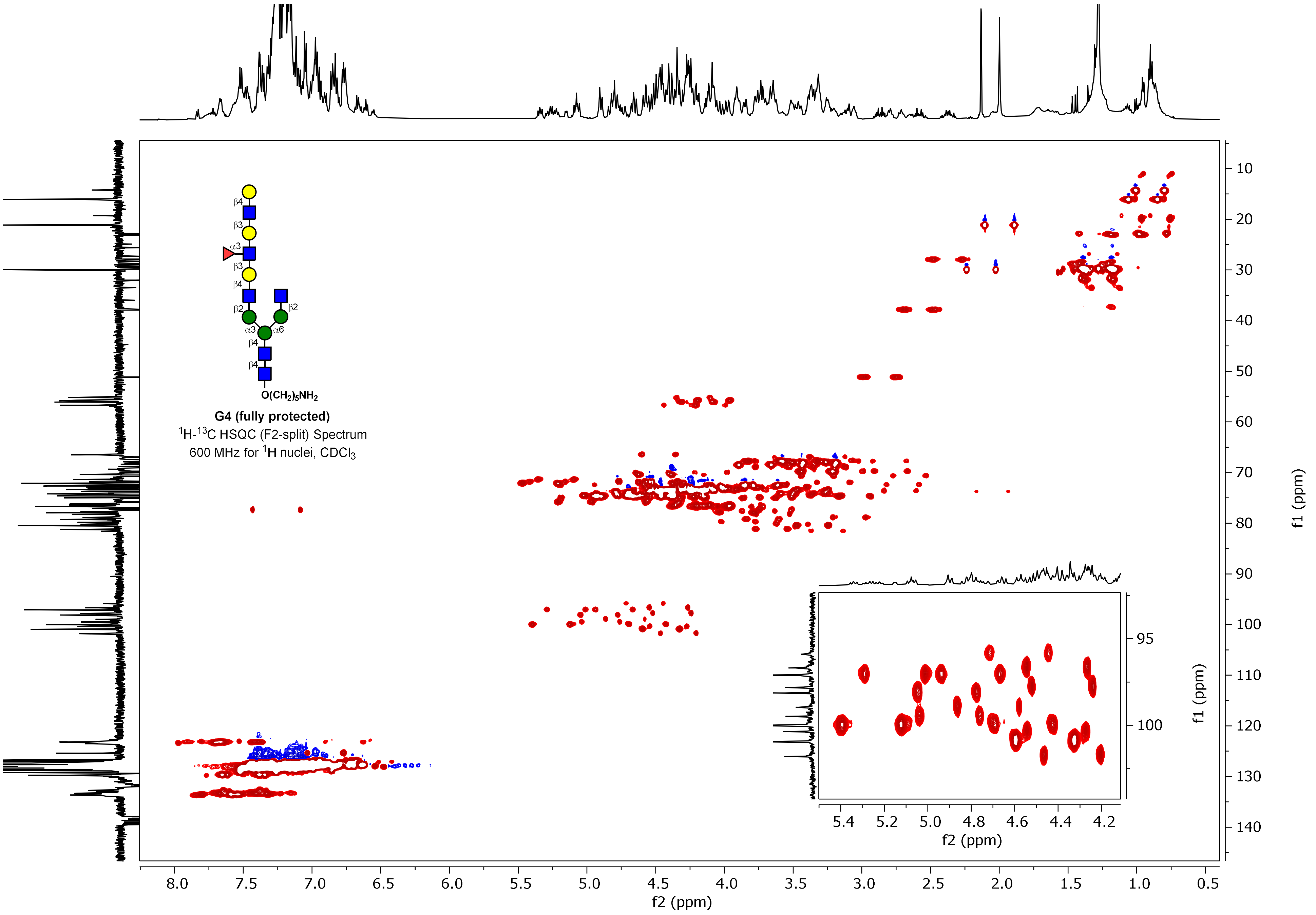

β4
β3
α3
β3
β4
β2
β2
α3
α6
β4
β4
O(CH2)5NH2
G4 (fully protected)
1H-13C HSQC (F2-split) Spectrum
600 MHz for 1H nuclei, CDCl3
f2 (ppm)
f1 (ppm)

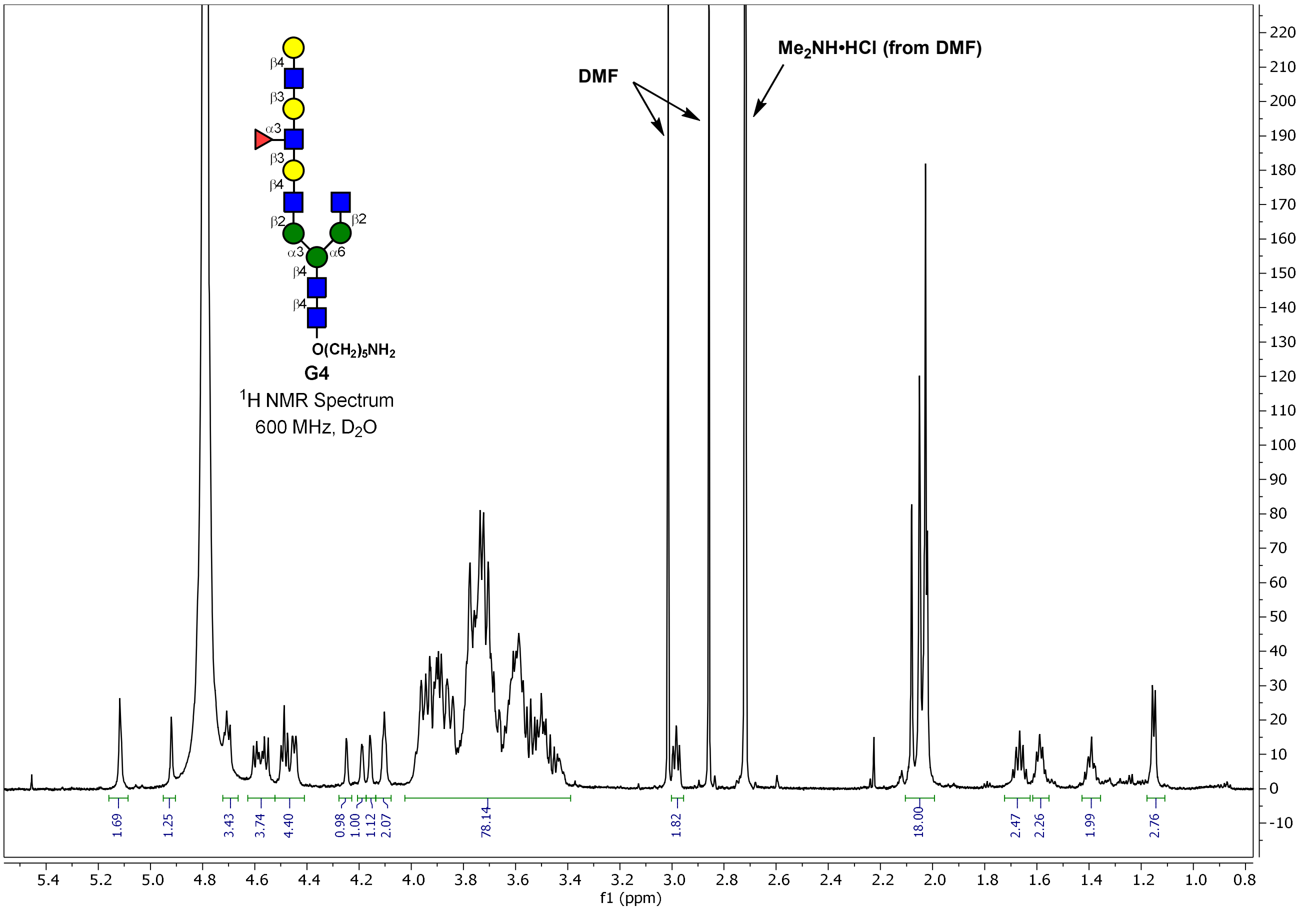

DMF
Me$_2$NH•HCl (from DMF)
O(CH$_2$)$_5$NH$_2$
G4
^{1}H NMR Spectrum
600 MHz, D$_2$O
f1 (ppm)

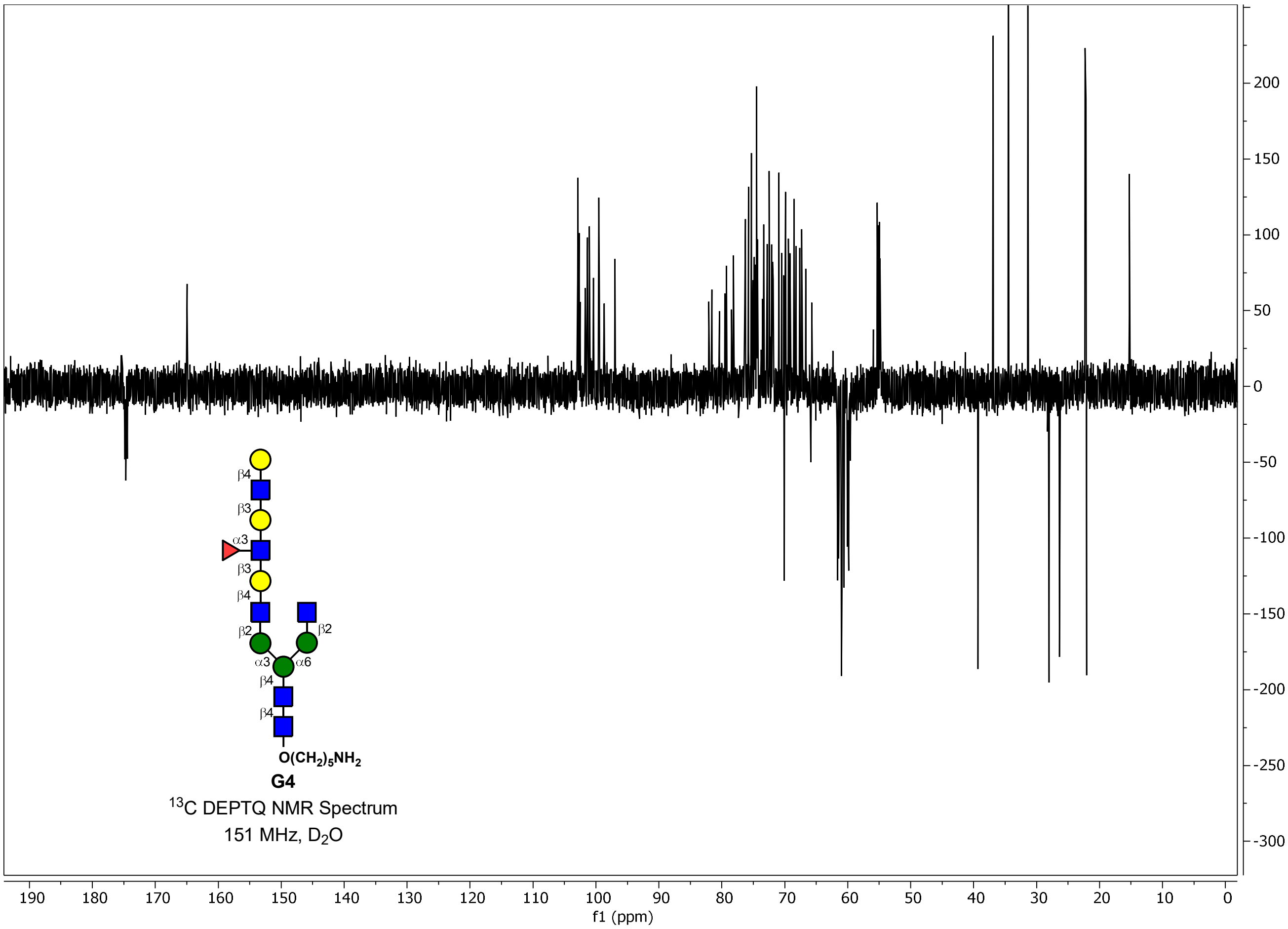

O(CH$_2$)$_5$NH$_2$
G4
^{13}C DEPTQ NMR Spectrum
151 MHz, D$_2$O
f1 (ppm)

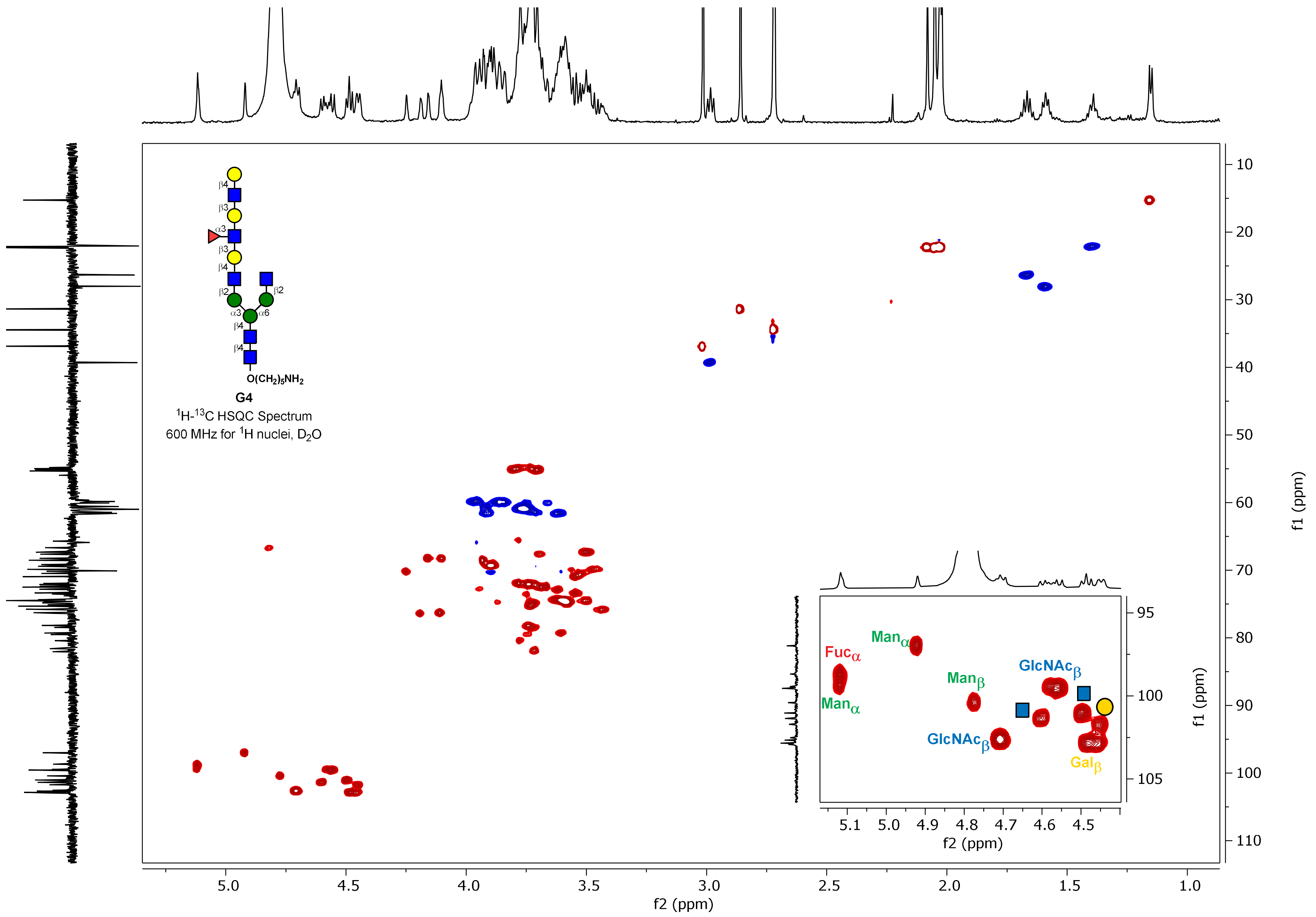
β4
β3
α3
β3
β4
β2
β2
α3
α6
β4
β4
O(CH2)5NH2
G4
1H-13C HSQC Spectrum
600 MHz for 1H nuclei, D2O
Fucα
Manα
Manα
Manβ
GlcNAcβ
GlcNAcβ
Galβ
f2 (ppm)
f1 (ppm)

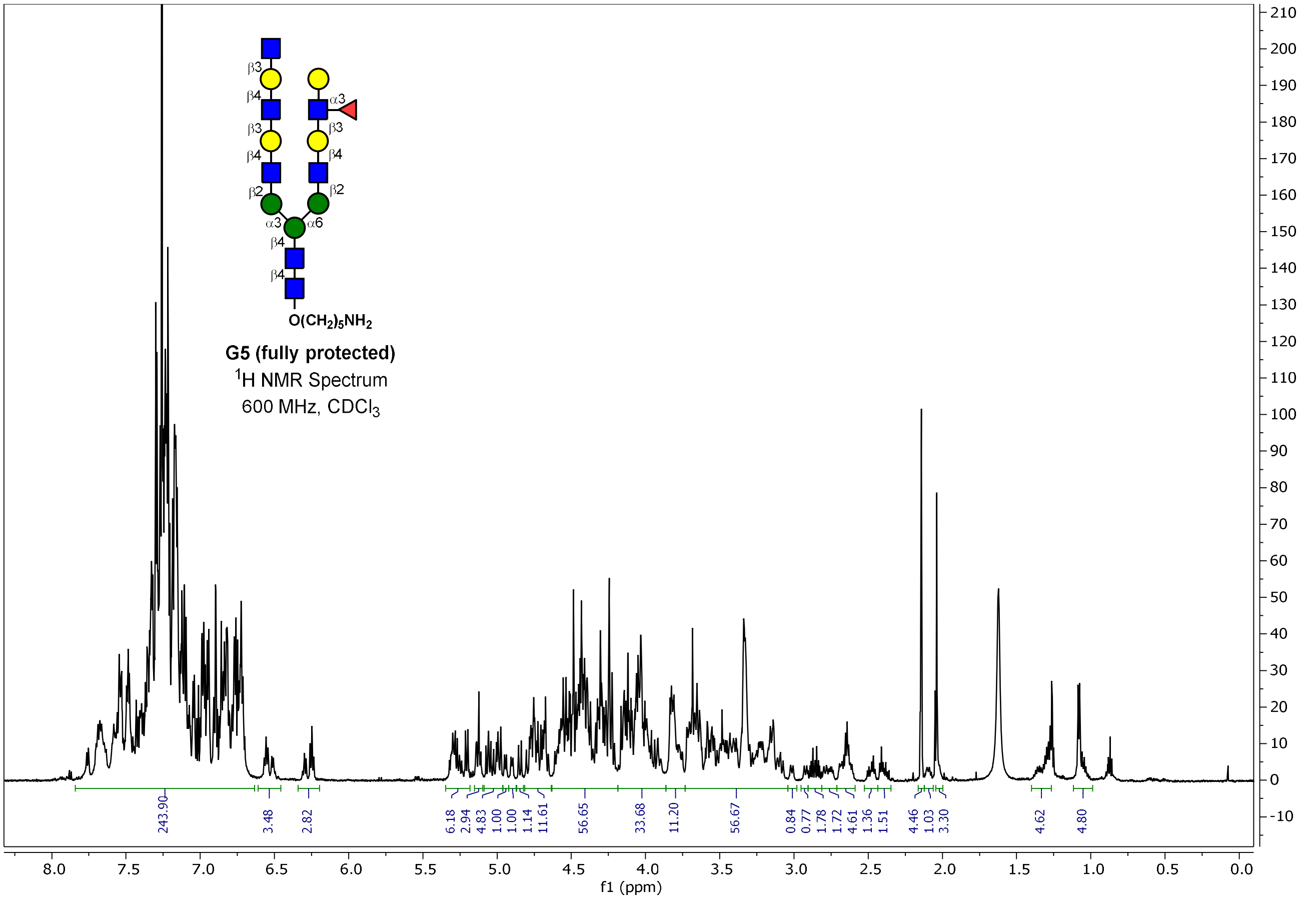
O(CH2)5NH2
G5 (fully protected)
1H NMR Spectrum
600 MHz, CDCl3
f1 (ppm)

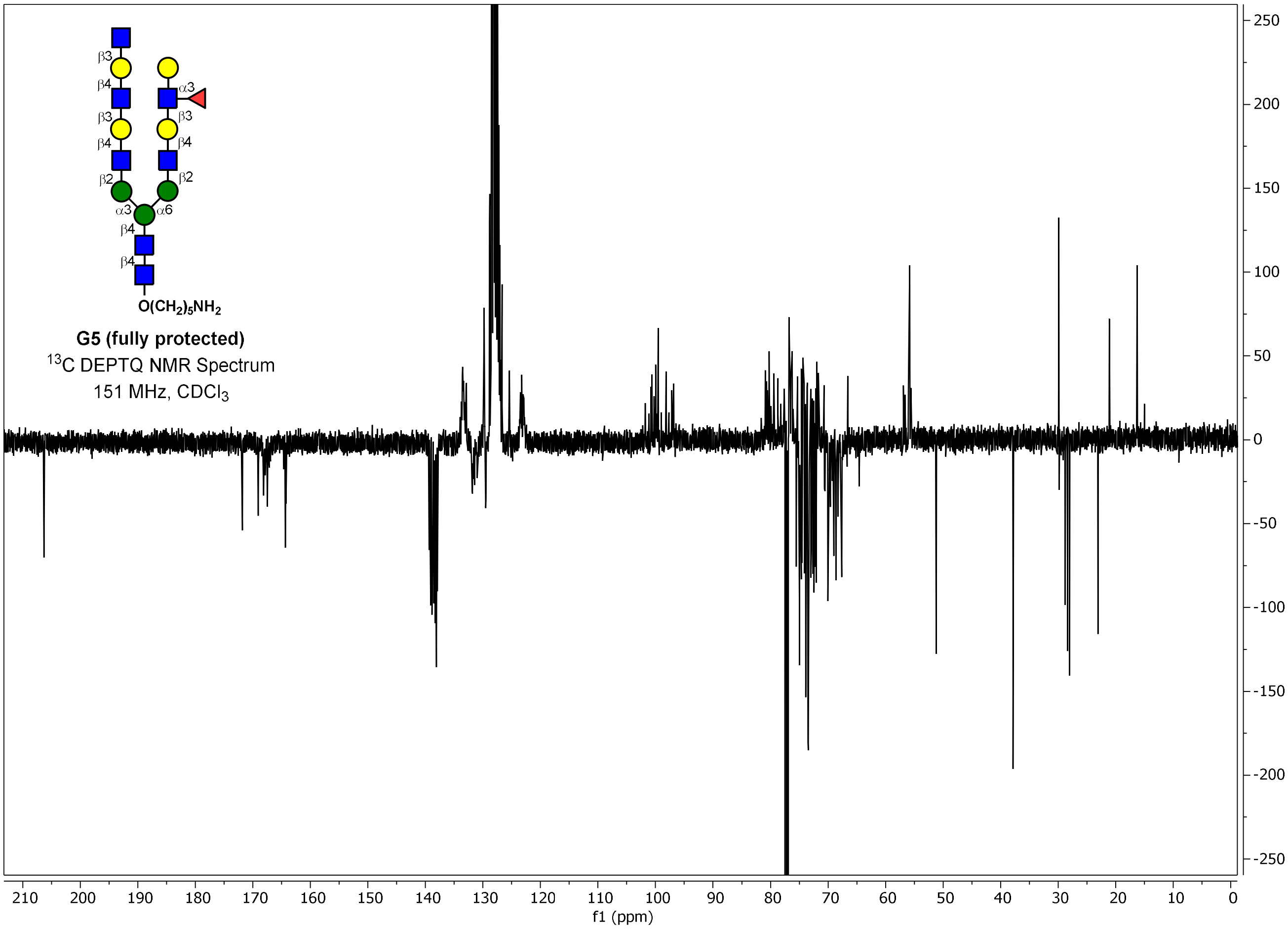
O(CH2)5NH2
G5 (fully protected)
13C DEPTQ NMR Spectrum
151 MHz, CDCl3
f1 (ppm)

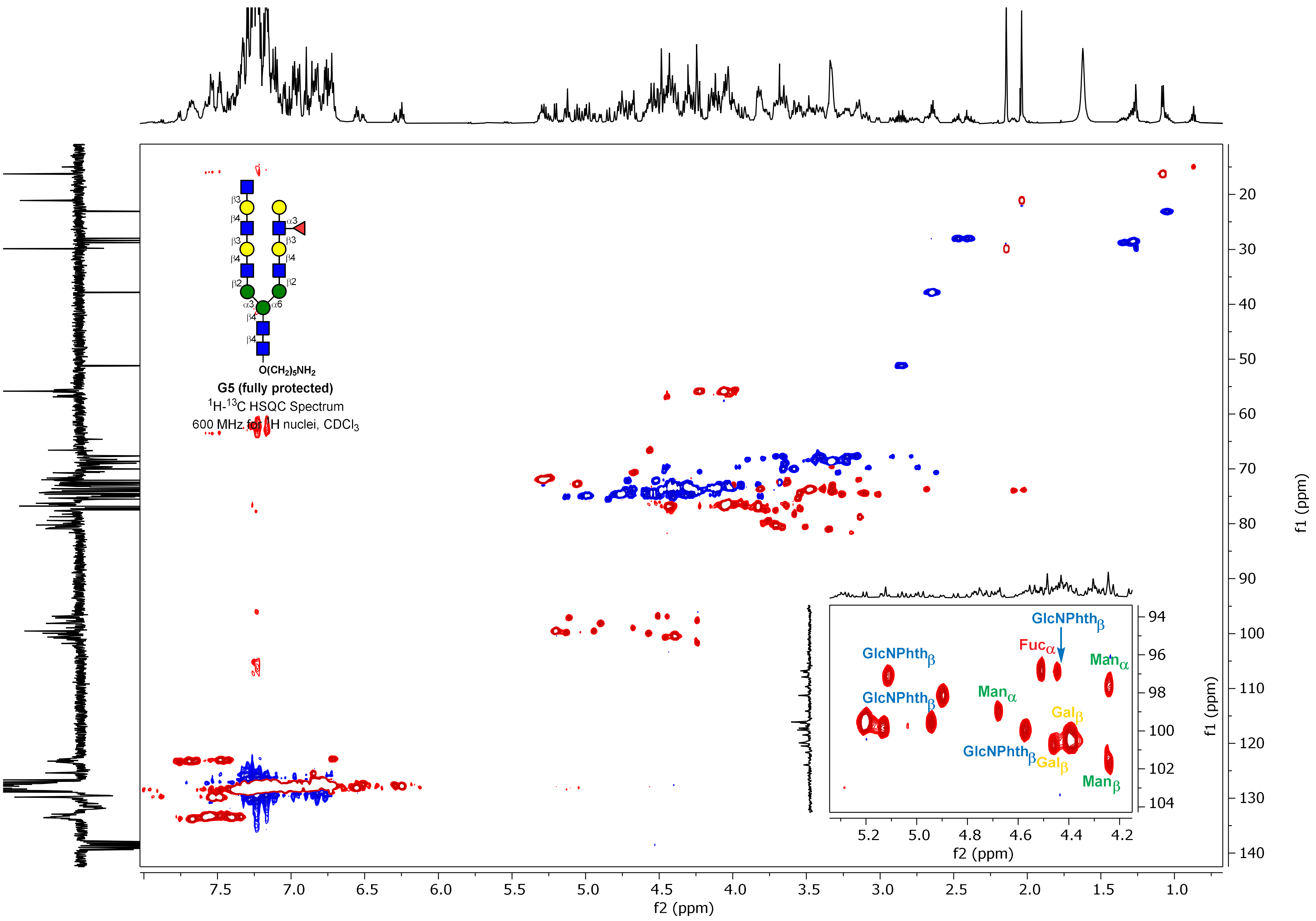
O(CH2)5NH2
G5 (fully protected)
1H-13C HSQC Spectrum
600 MHz for 1H nuclei, CDCl3
GlcNPhthβ
Fucα
Manα
Galβ
Manβ
f2 (ppm)
f1 (ppm)

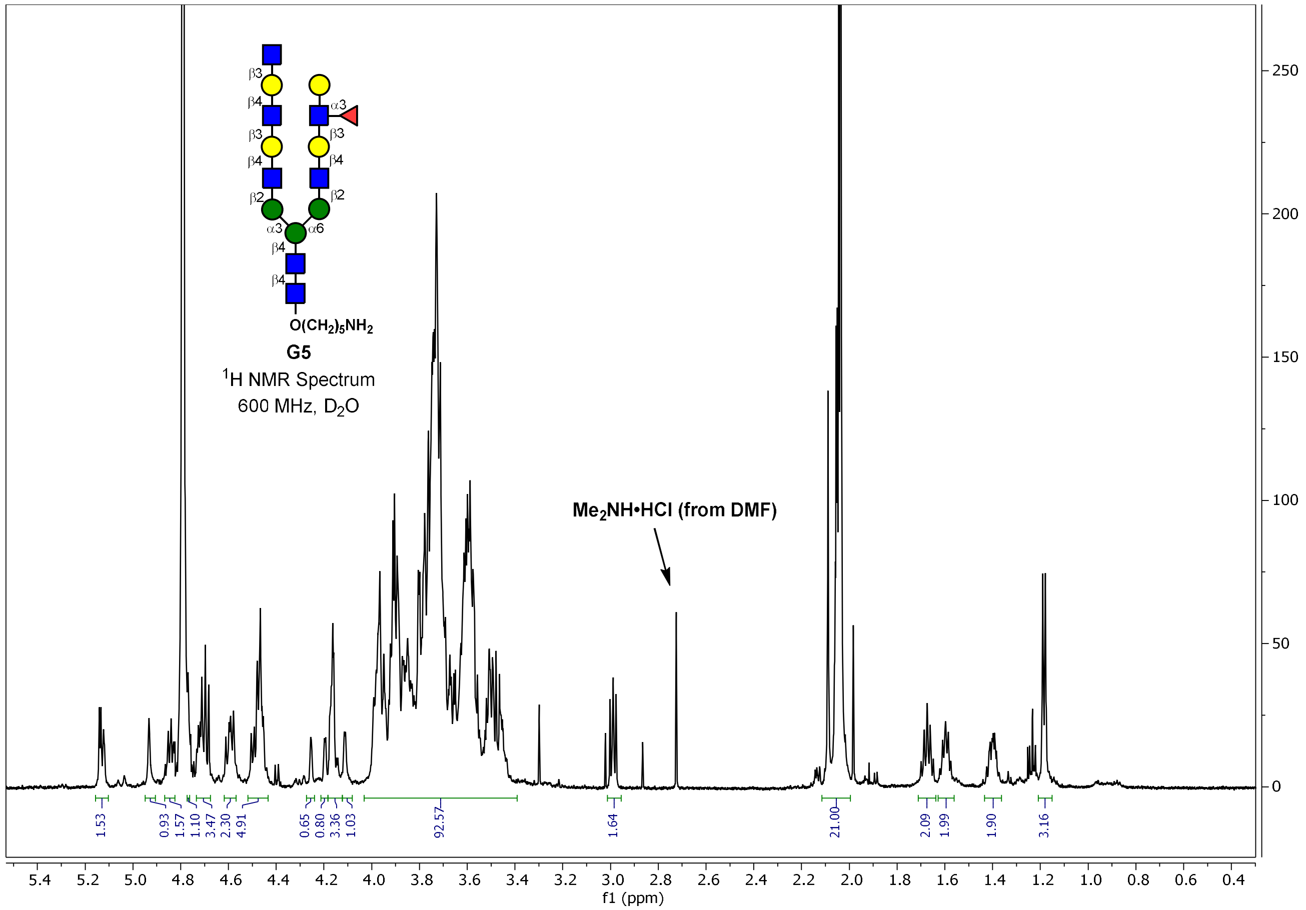
O(CH2)5NH2
G5
1H NMR Spectrum
600 MHz, D2O
Me2NH•HCl (from DMF)
f1 (ppm)

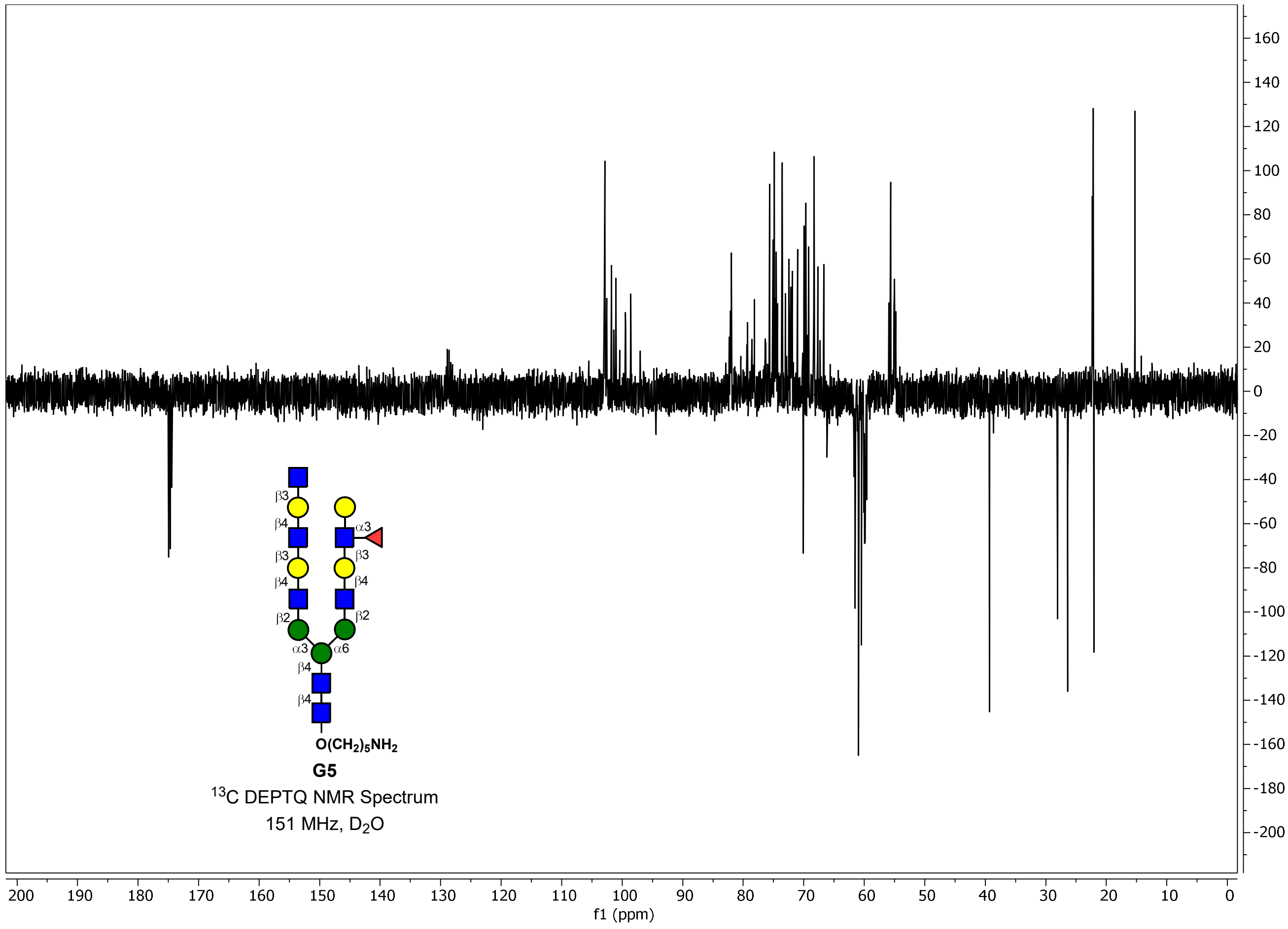
O(CH2)5NH2
G5
13C DEPTQ NMR Spectrum
151 MHz, D2O
f1 (ppm)

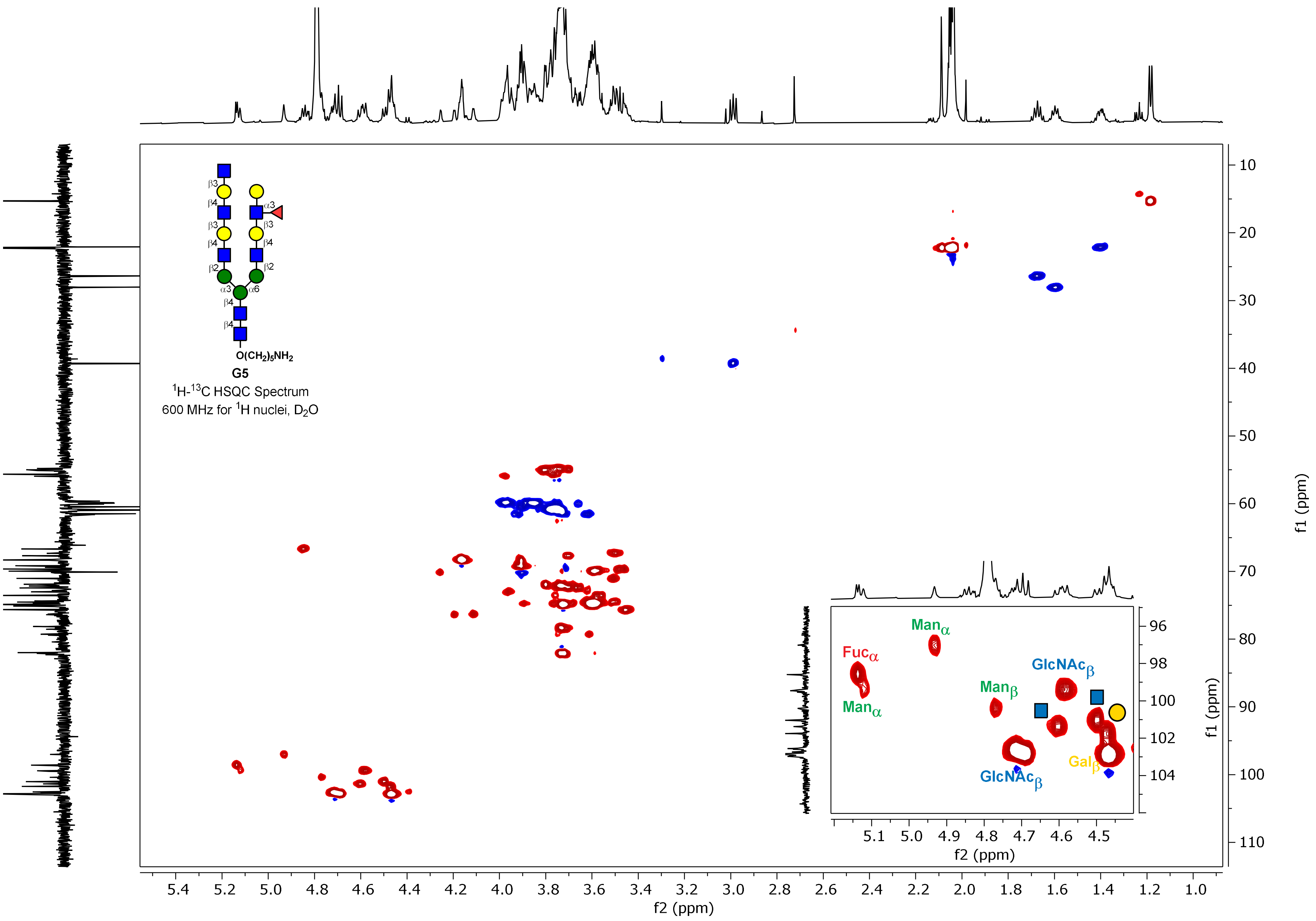
O(CH2)5NH2
G5
1H-13C HSQC Spectrum
600 MHz for 1H nuclei, D2O
Manα
Fucα
GlcNAcβ
Manβ
Manα
Galβ
GlcNAcβ
f2 (ppm)
f1 (ppm)
f2 (ppm)
f1 (ppm)

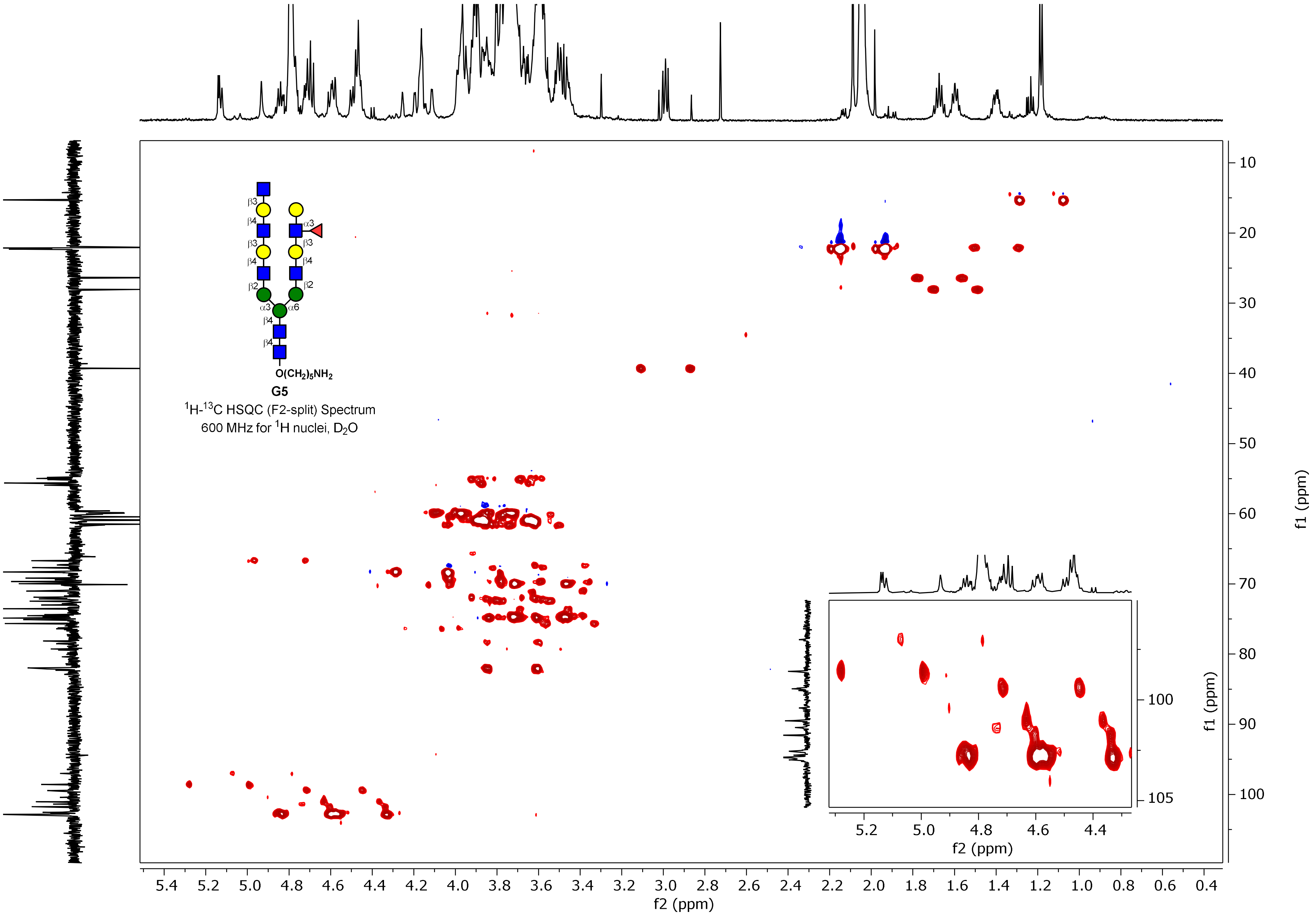
O(CH2)5NH2
G5
1H-13C HSQC (F2-split) Spectrum
600 MHz for 1H nuclei, D2O
f2 (ppm)
f1 (ppm)

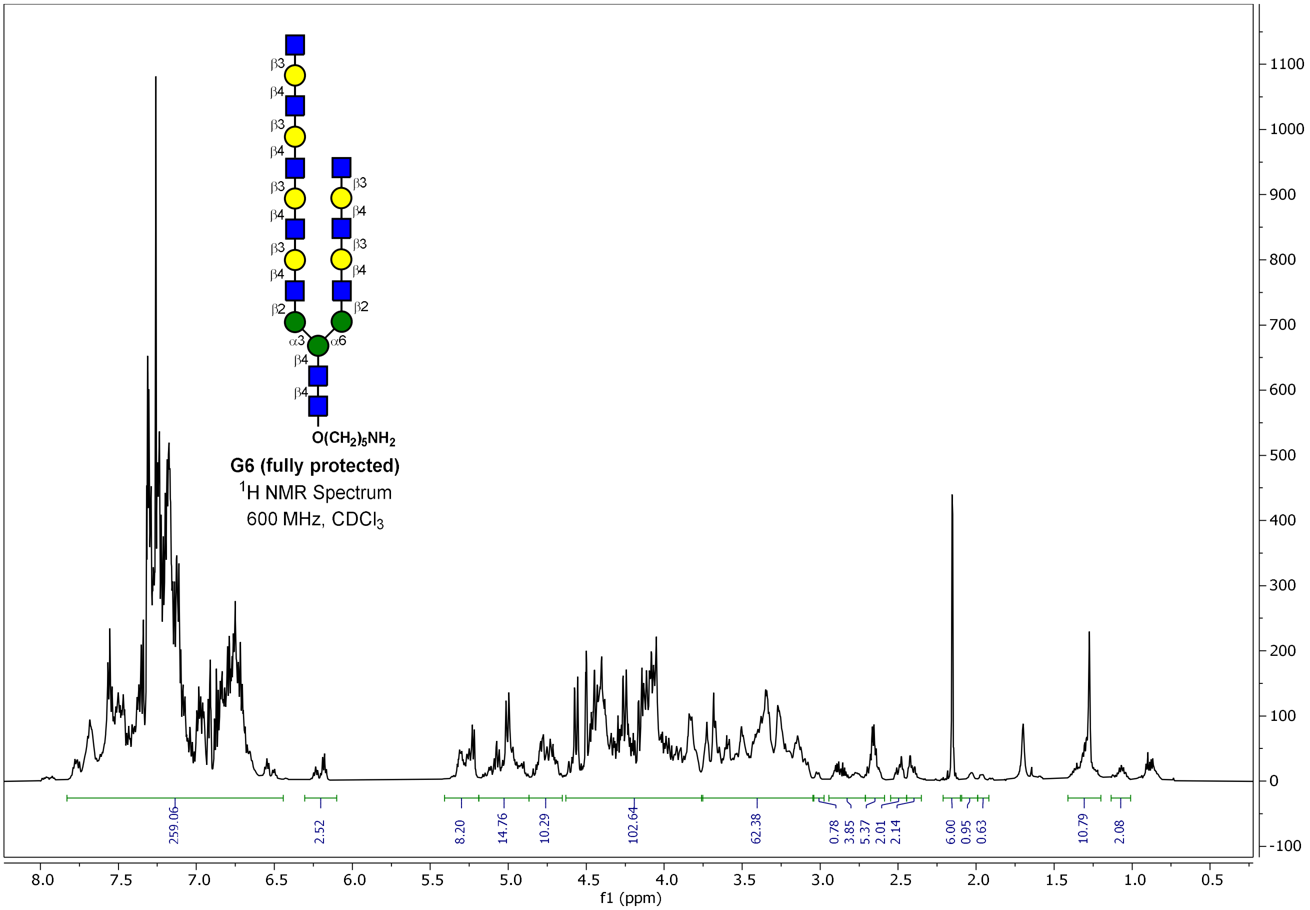

O(CH2)5NH2
G6 (fully protected)
1H NMR Spectrum
600 MHz, CDCl3
259.06
2.52
8.20
14.76
10.29
102.64
62.38
0.78
3.85
5.37
2.01
2.14
6.00
0.95
0.63
10.79
2.08
f1 (ppm)

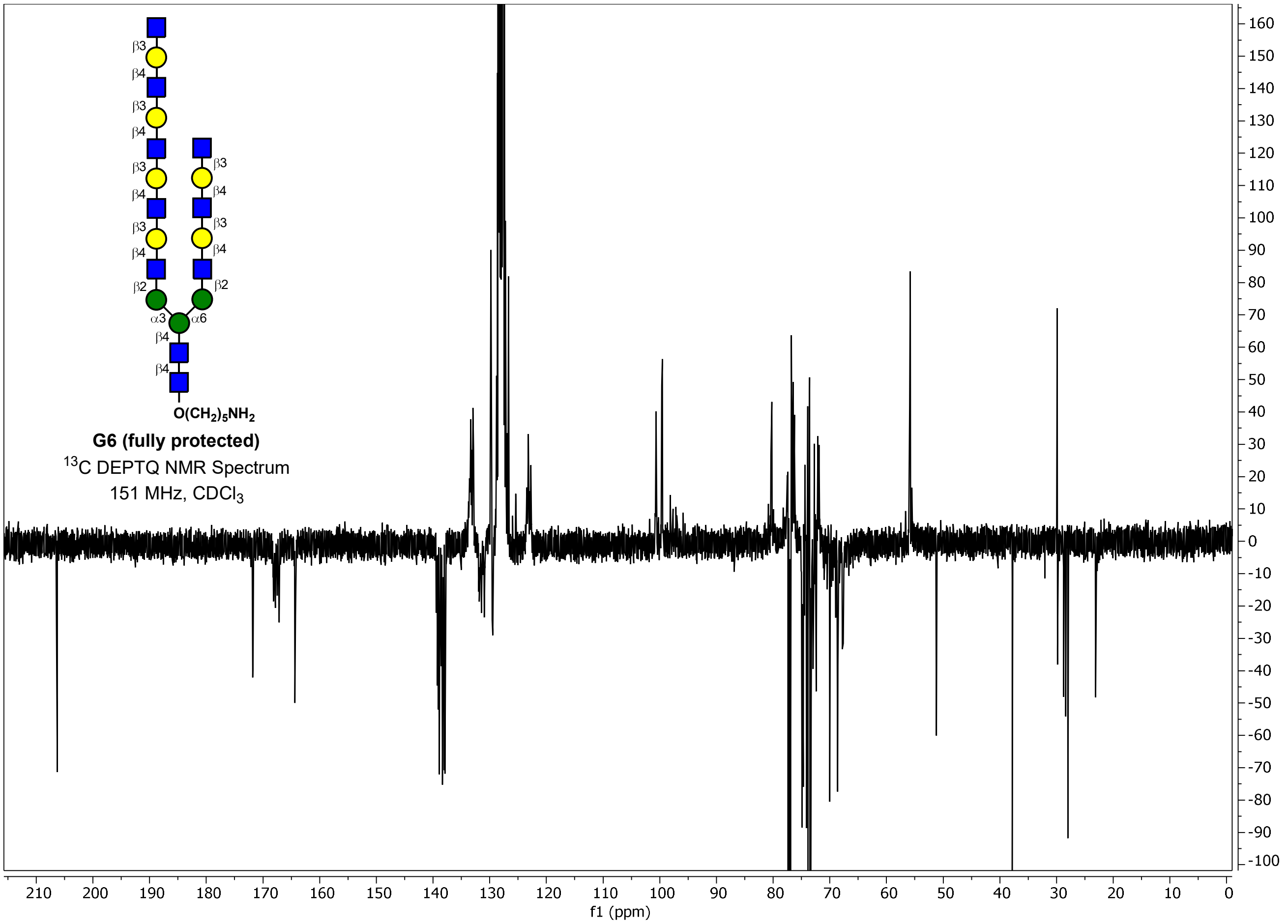

O(CH2)5NH2
G6 (fully protected)
13C DEPTQ NMR Spectrum
151 MHz, CDCl3
f1 (ppm)

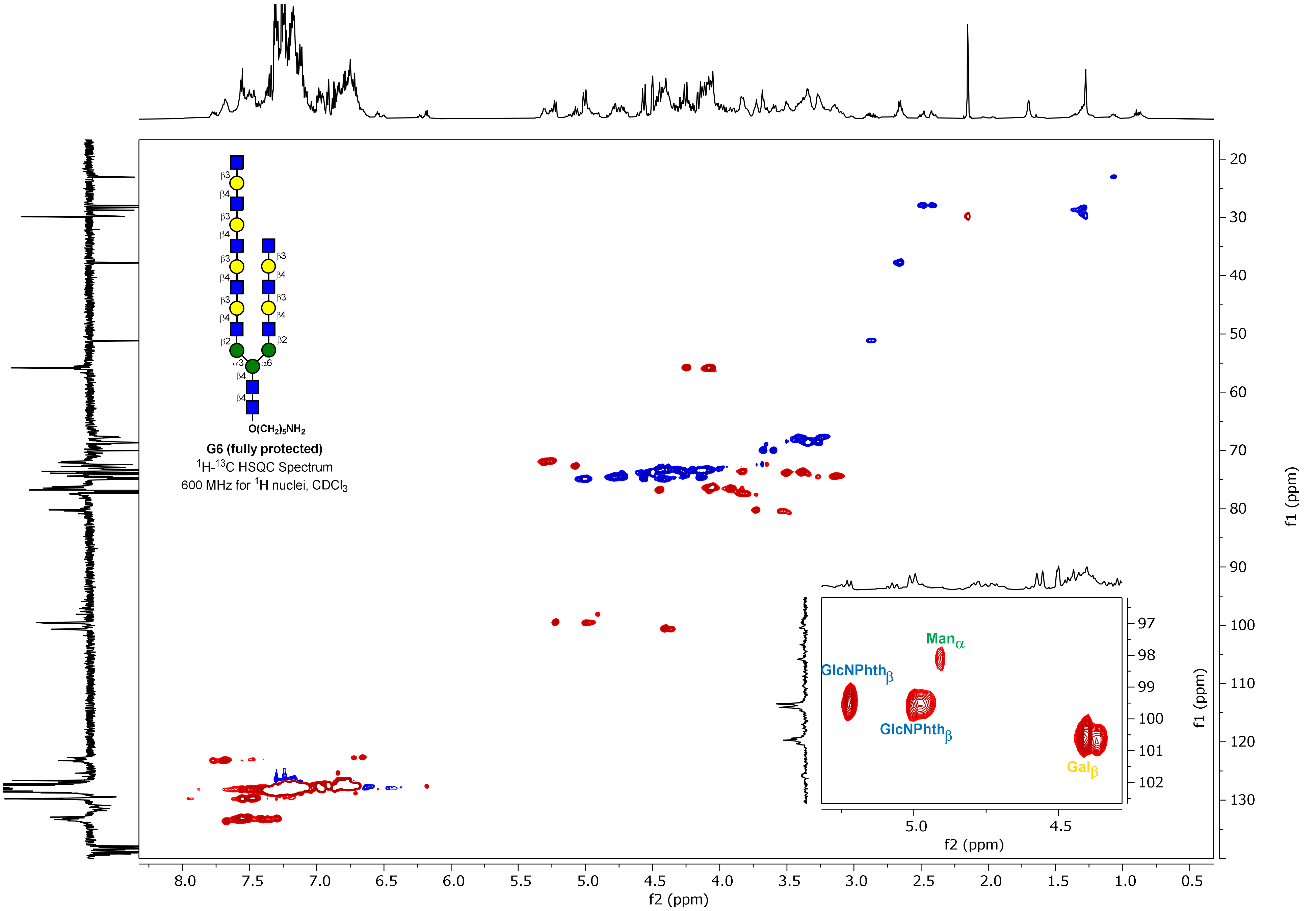

O(CH2)5NH2
G6 (fully protected)
1H-13C HSQC Spectrum
600 MHz for 1H nuclei, CDCl3
Manα
GlcNPhthβ
GlcNPhthβ
Galβ
f2 (ppm)
f1 (ppm)

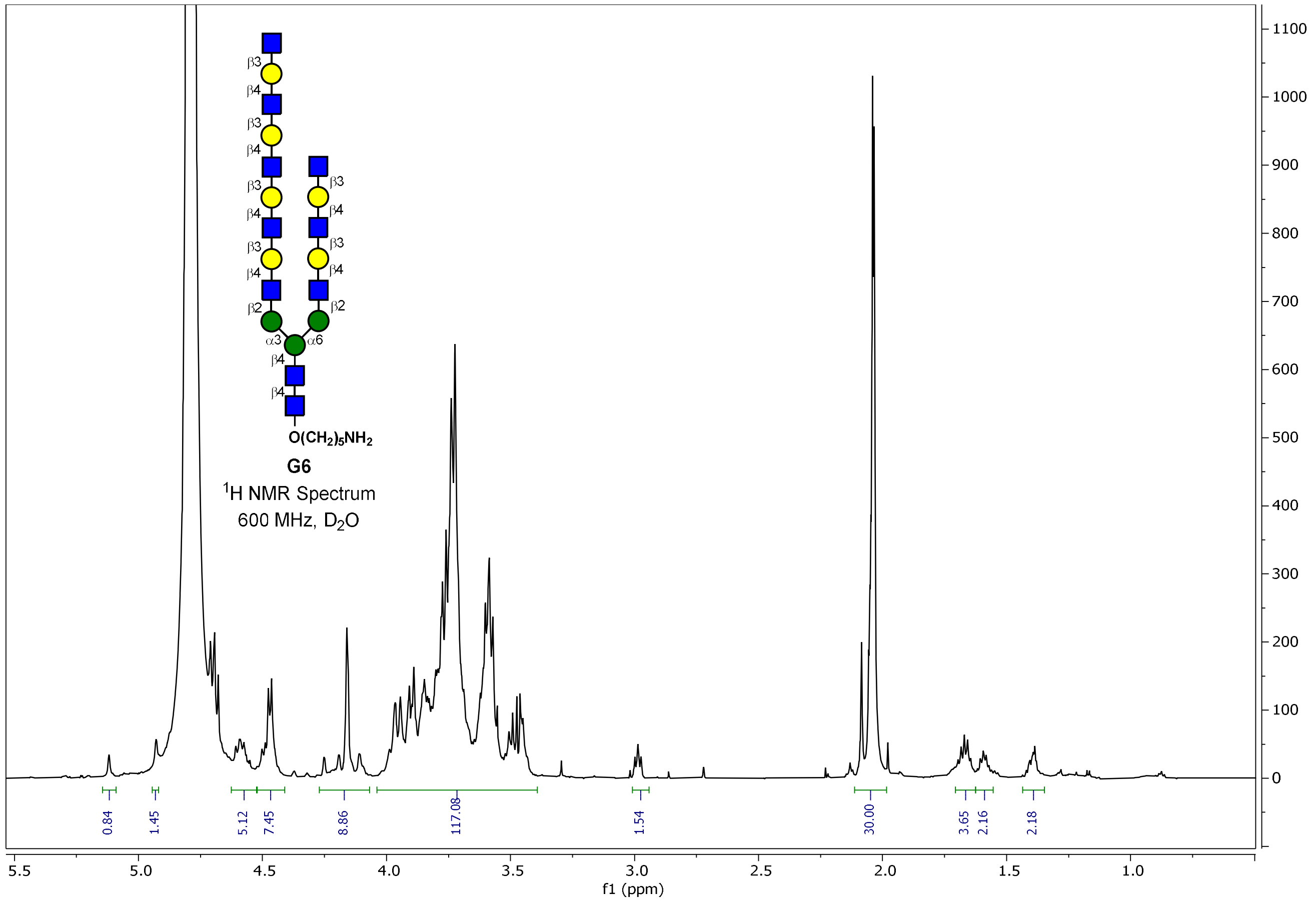
O(CH2)5NH2
G6
1H NMR Spectrum
600 MHz, D2O
f1 (ppm)

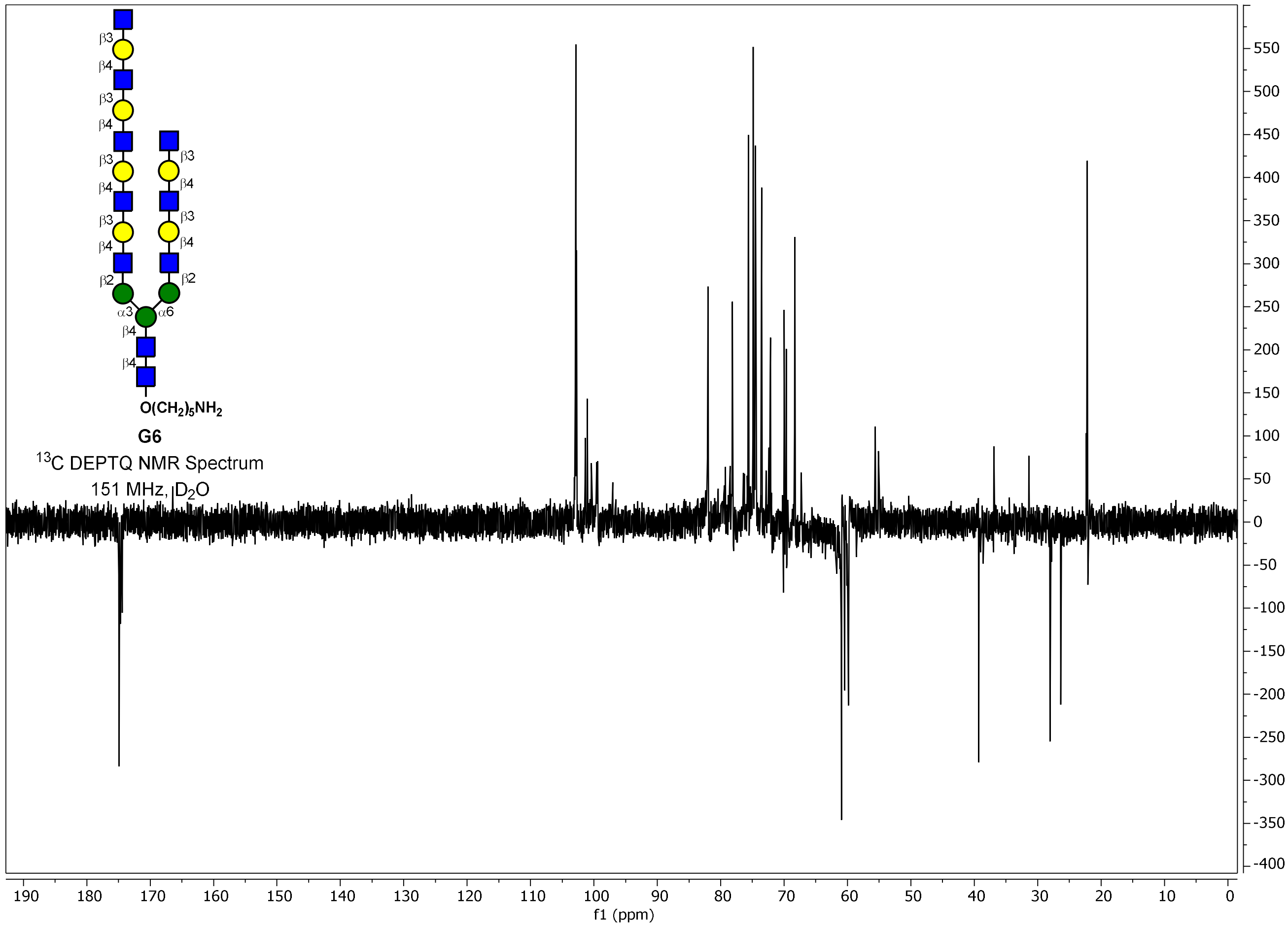
O(CH2)5NH2
G6
13C DEPTQ NMR Spectrum
151 MHz, D2O
f1 (ppm)

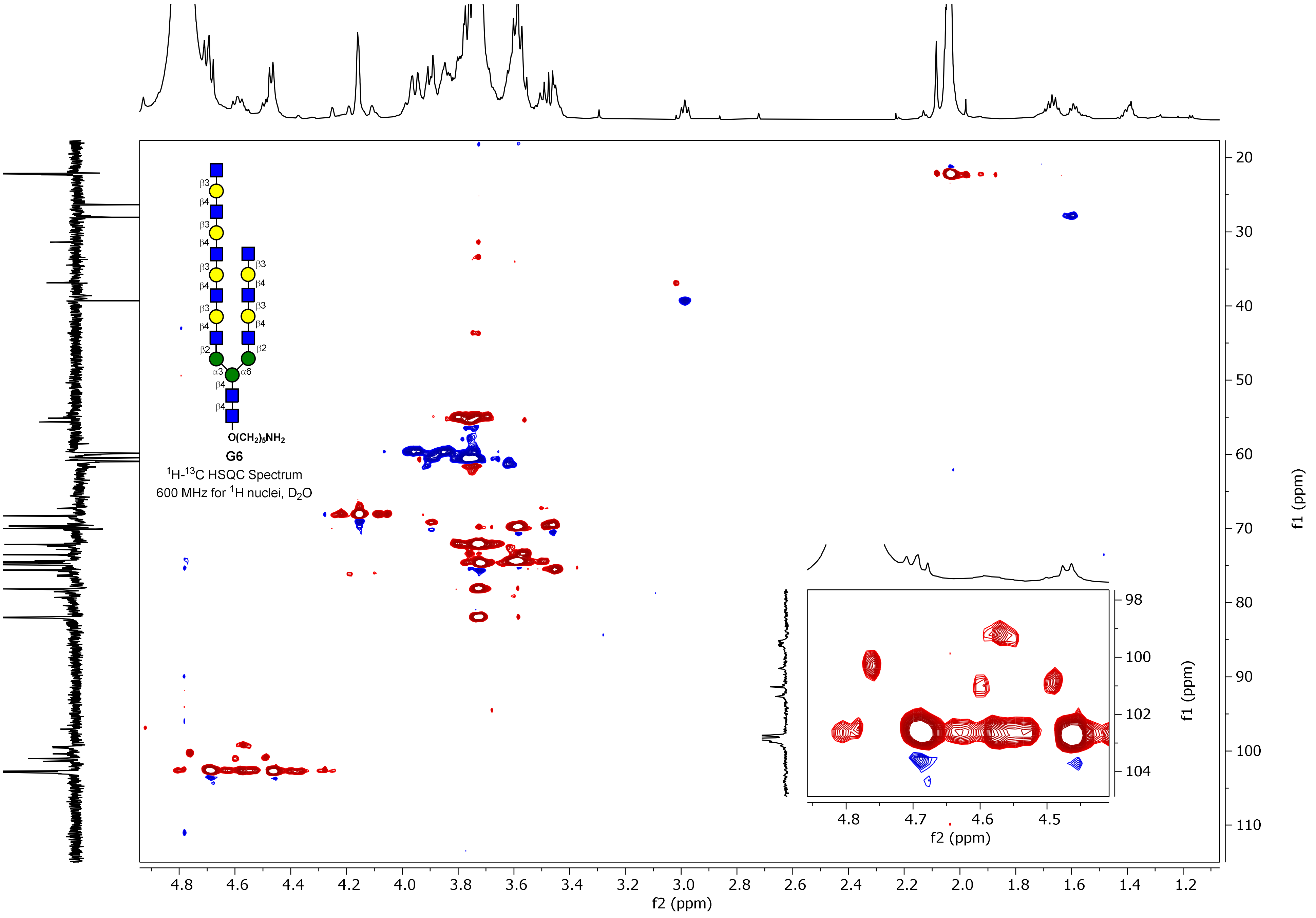

G6
^{1}H-^{13}C HSQC Spectrum
600 MHz for ^{1}H nuclei, D_2O
O(CH2)5NH2
f2 (ppm)
f1 (ppm)

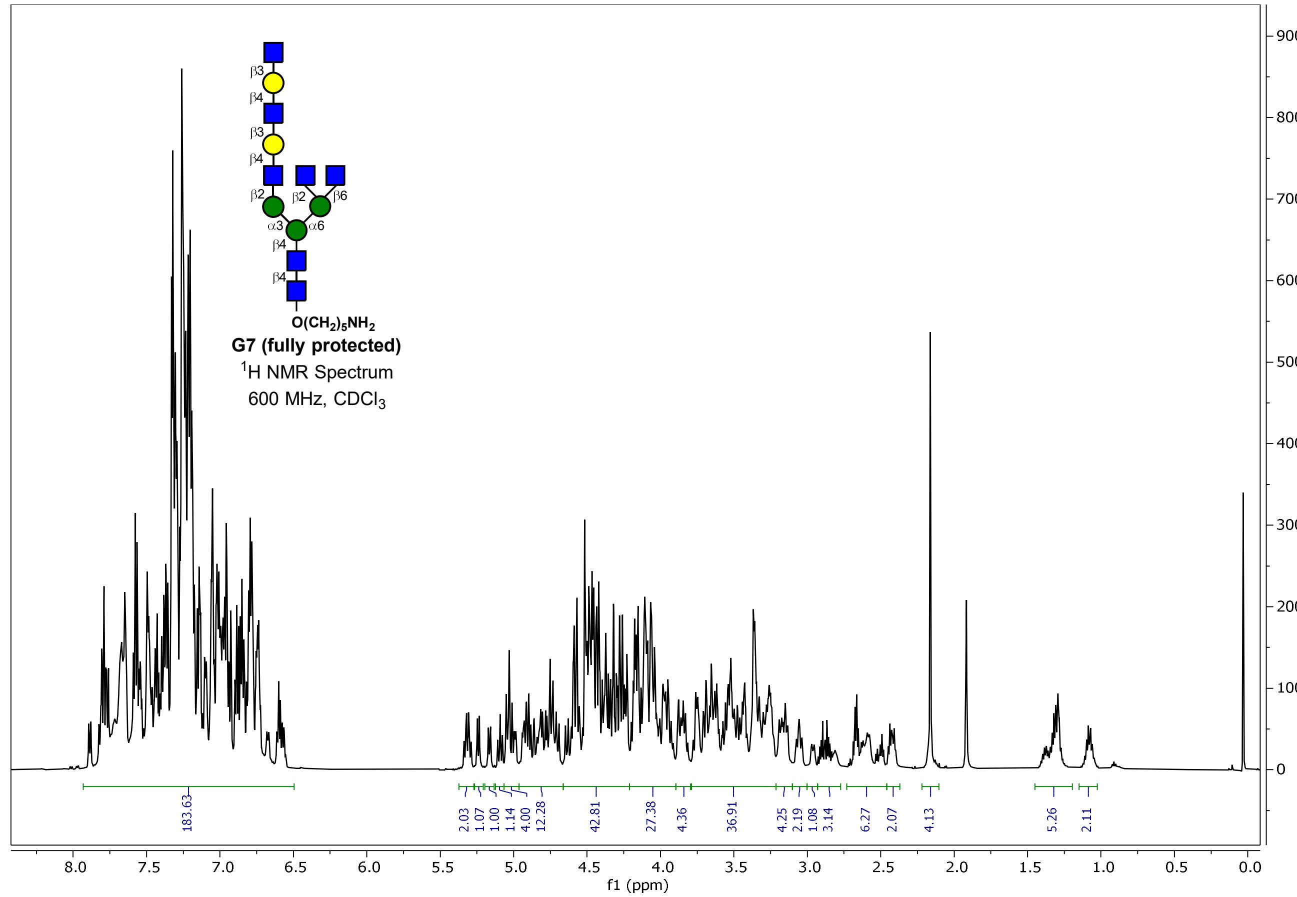
O(CH2)5NH2
G7 (fully protected)
1H NMR Spectrum
600 MHz, CDCl3
f1 (ppm)

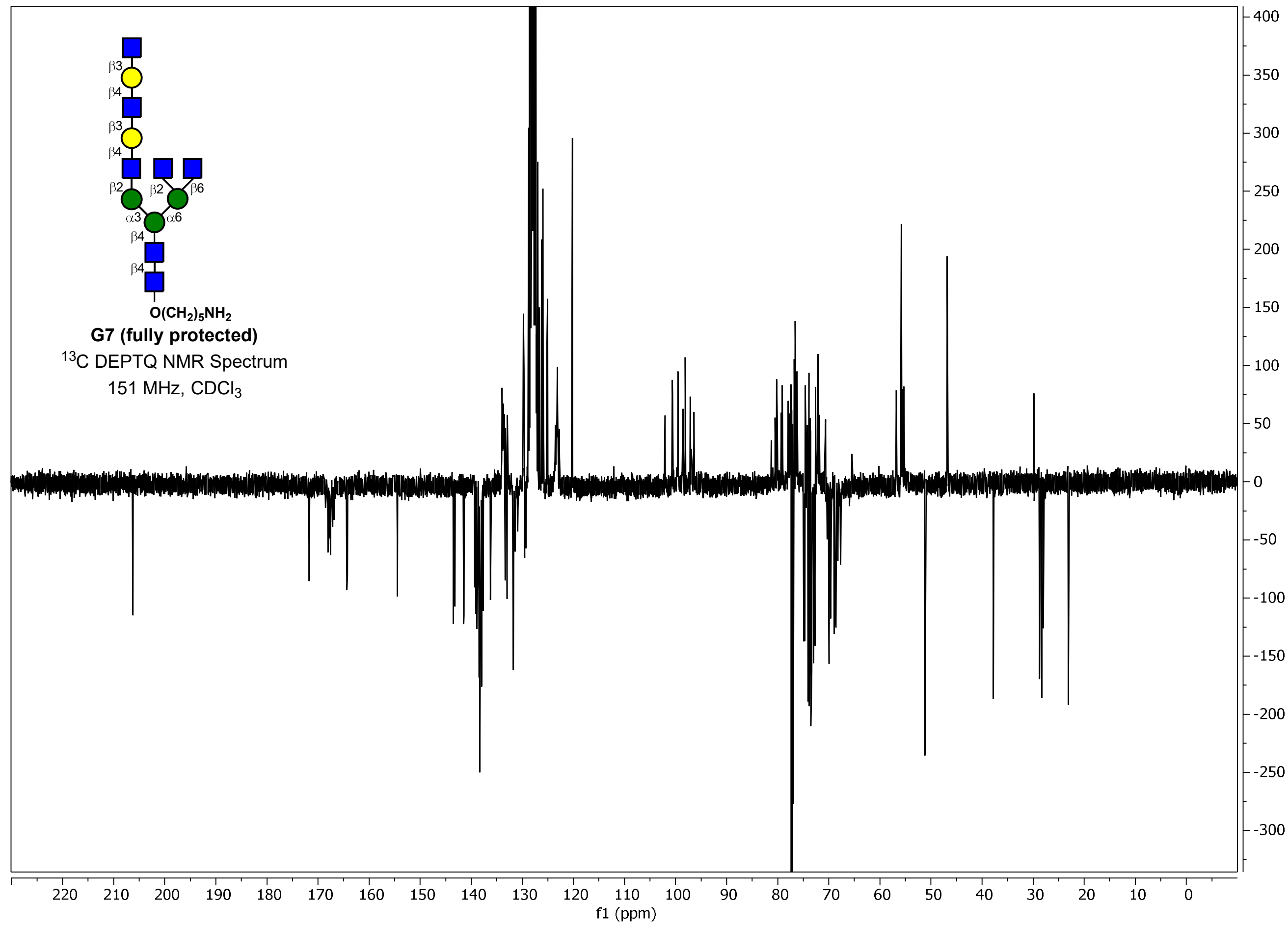
O(CH2)5NH2
G7 (fully protected)
13C DEPTQ NMR Spectrum
151 MHz, CDCl3
f1 (ppm)

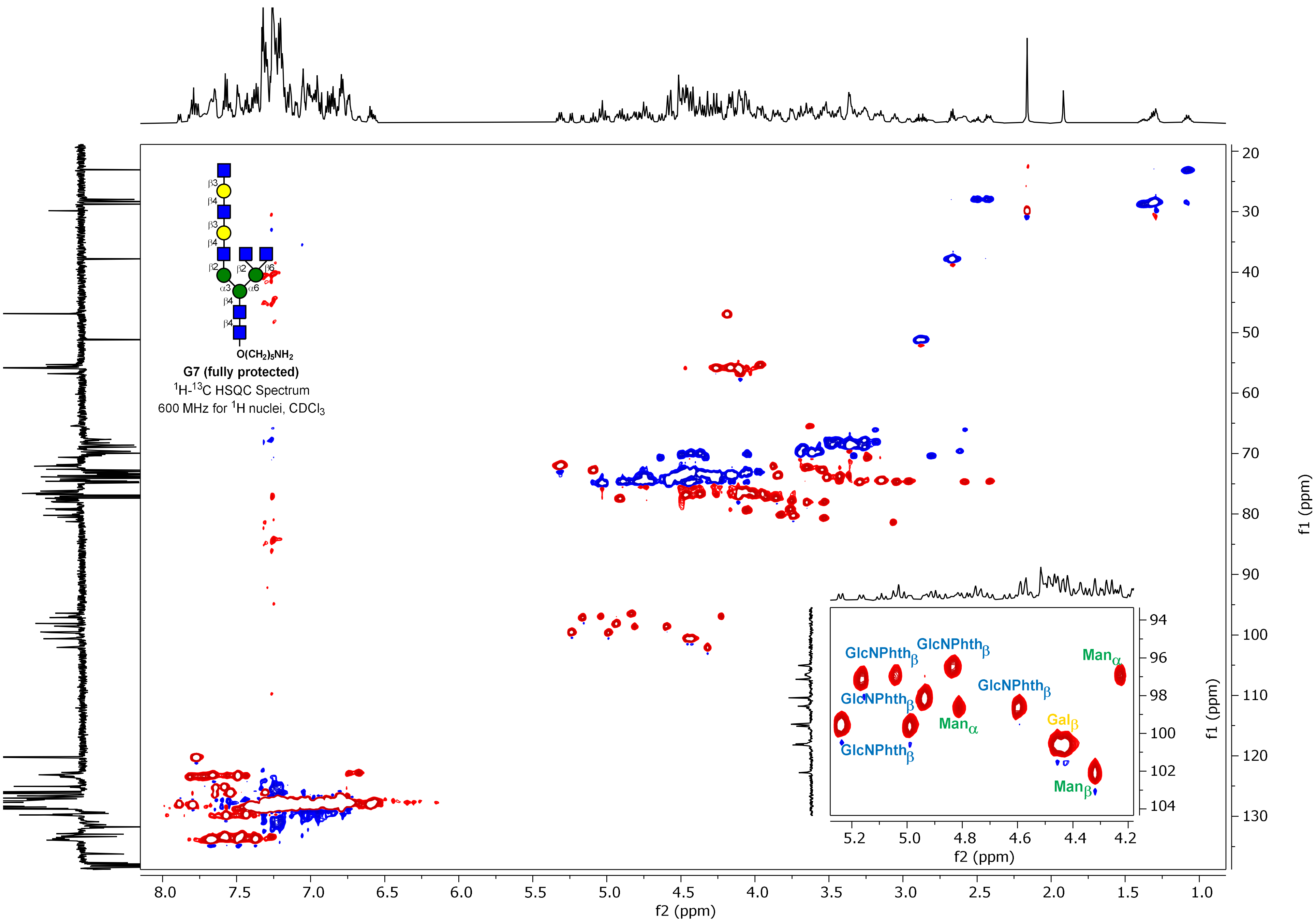

O(CH2)5NH2
G7 (fully protected)
1H-13C HSQC Spectrum
600 MHz for 1H nuclei, CDCl3
f2 (ppm)
f1 (ppm)
GlcNPhthβ
GlcNPhthβ
GlcNPhthβ
GlcNPhthβ
GlcNPhthβ
Manα
Manα
Galβ
Manβ

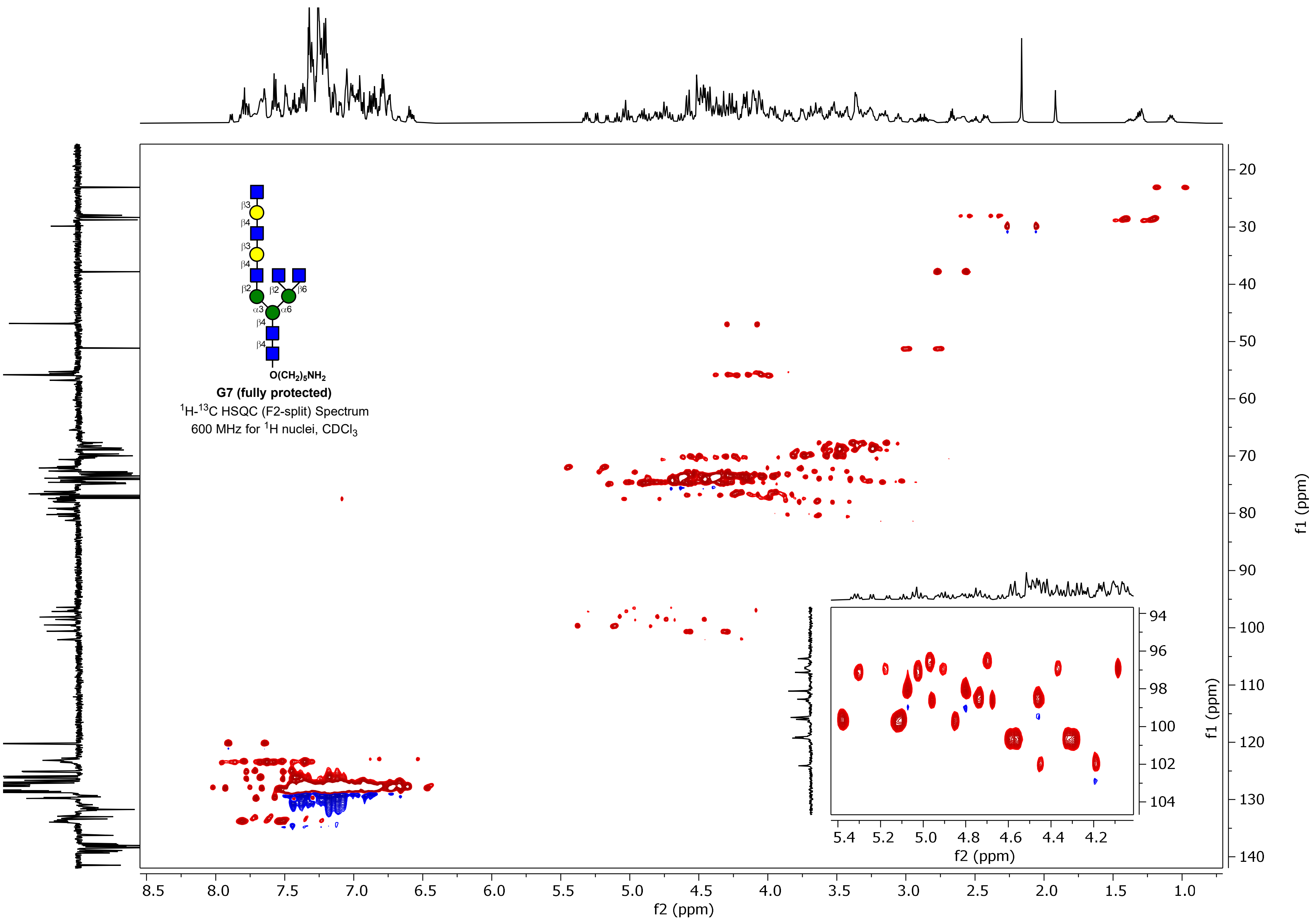

β3
β4
β3
β4
β2
β2
β6
α3
α6
β4
β4
O(CH2)5NH2
G7 (fully protected)
1H-13C HSQC (F2-split) Spectrum
600 MHz for 1H nuclei, CDCl3
f2 (ppm)
f1 (ppm)

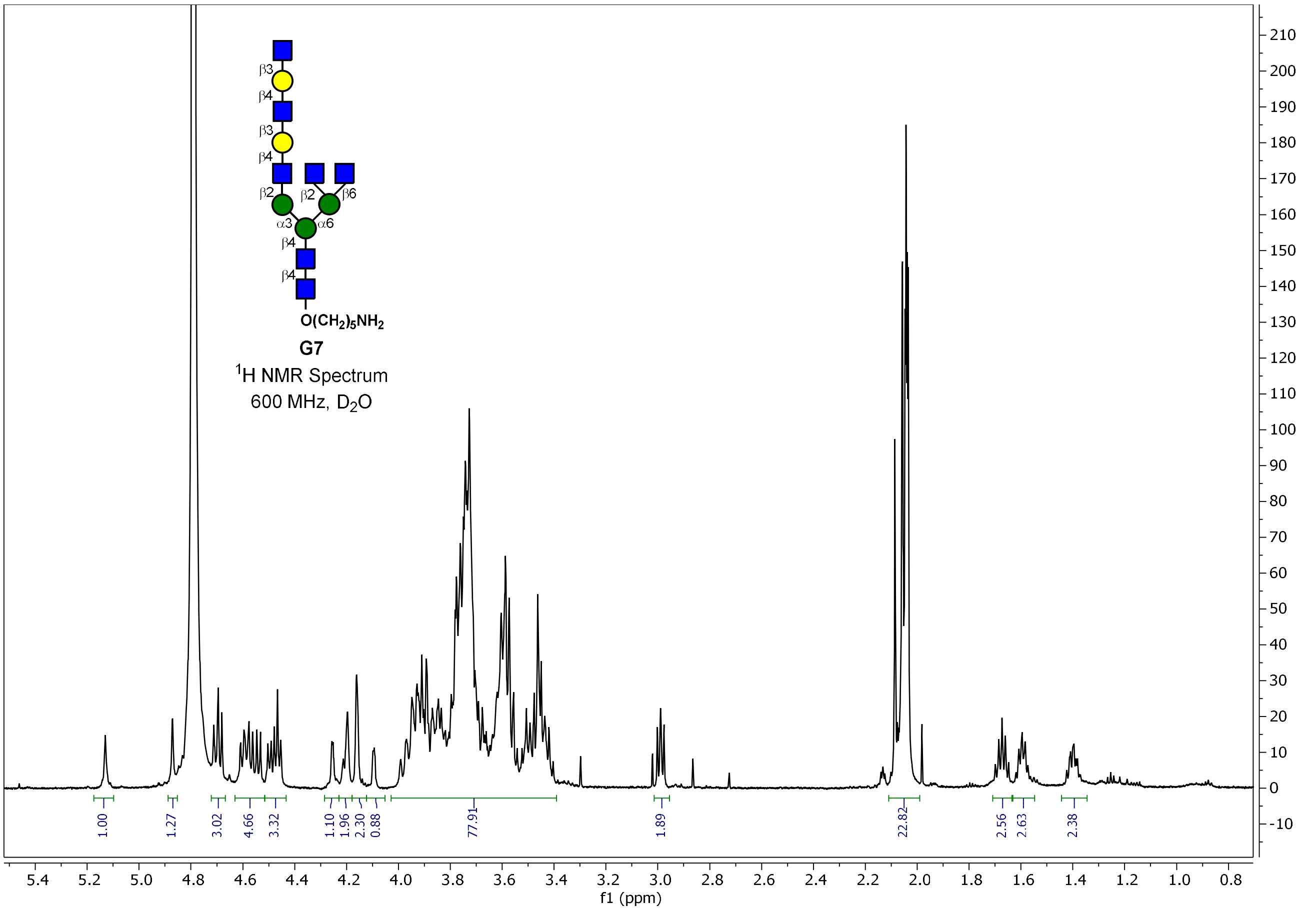
β3
β4
β3
β4
β2
β2
β6
α3
α6
β4
β4
O(CH2)5NH2
G7
1H NMR Spectrum
600 MHz, D2O
1.00
1.27
3.02
4.66
3.32
1.10
1.96
2.30
0.88
77.91
1.89
22.82
2.56
2.63
2.38
f1 (ppm)

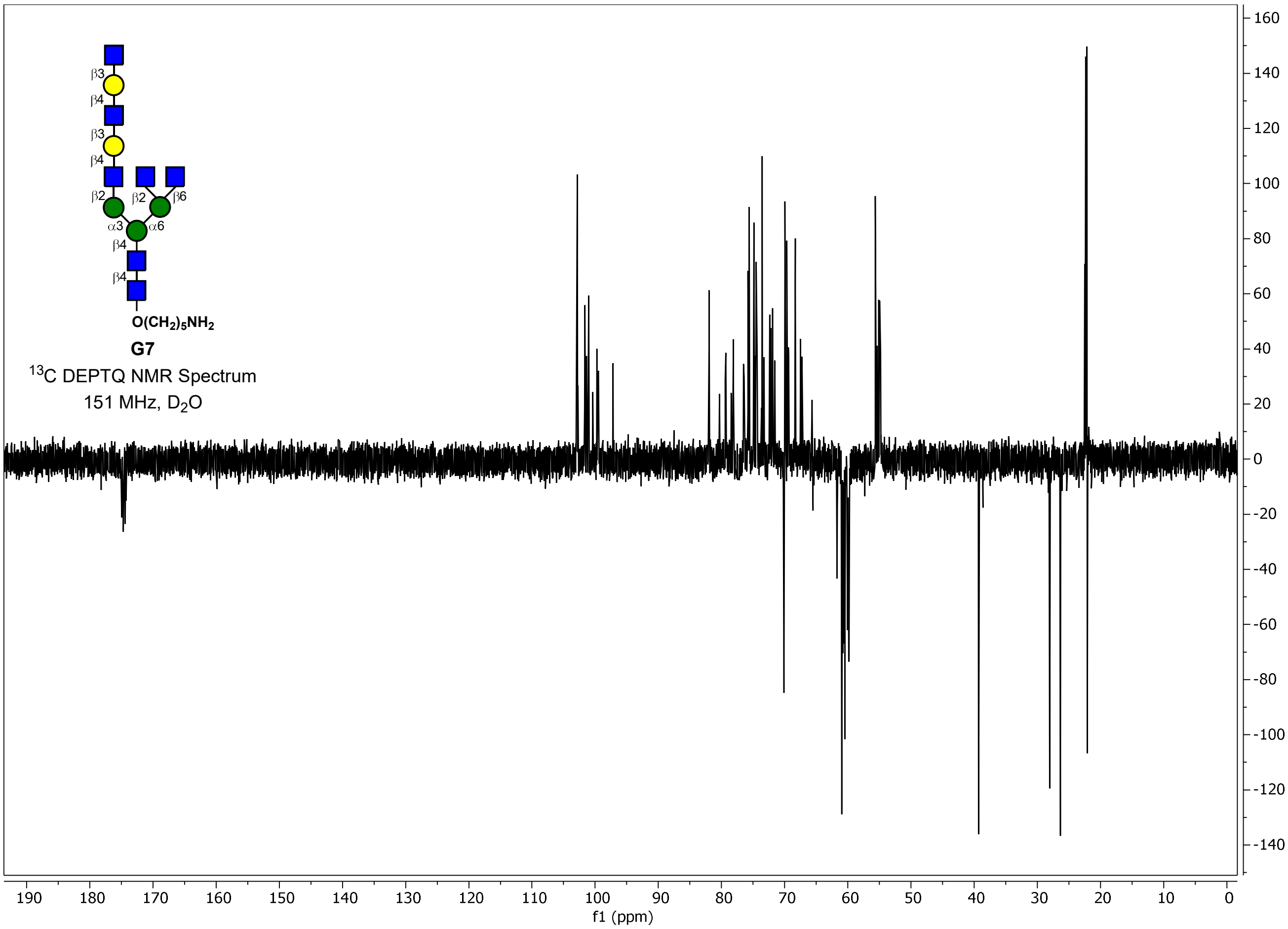
β3
β4
β3
β4
β2
β2
β6
α3
α6
β4
β4
O(CH2)5NH2
G7
13C DEPTQ NMR Spectrum
151 MHz, D2O
f1 (ppm)

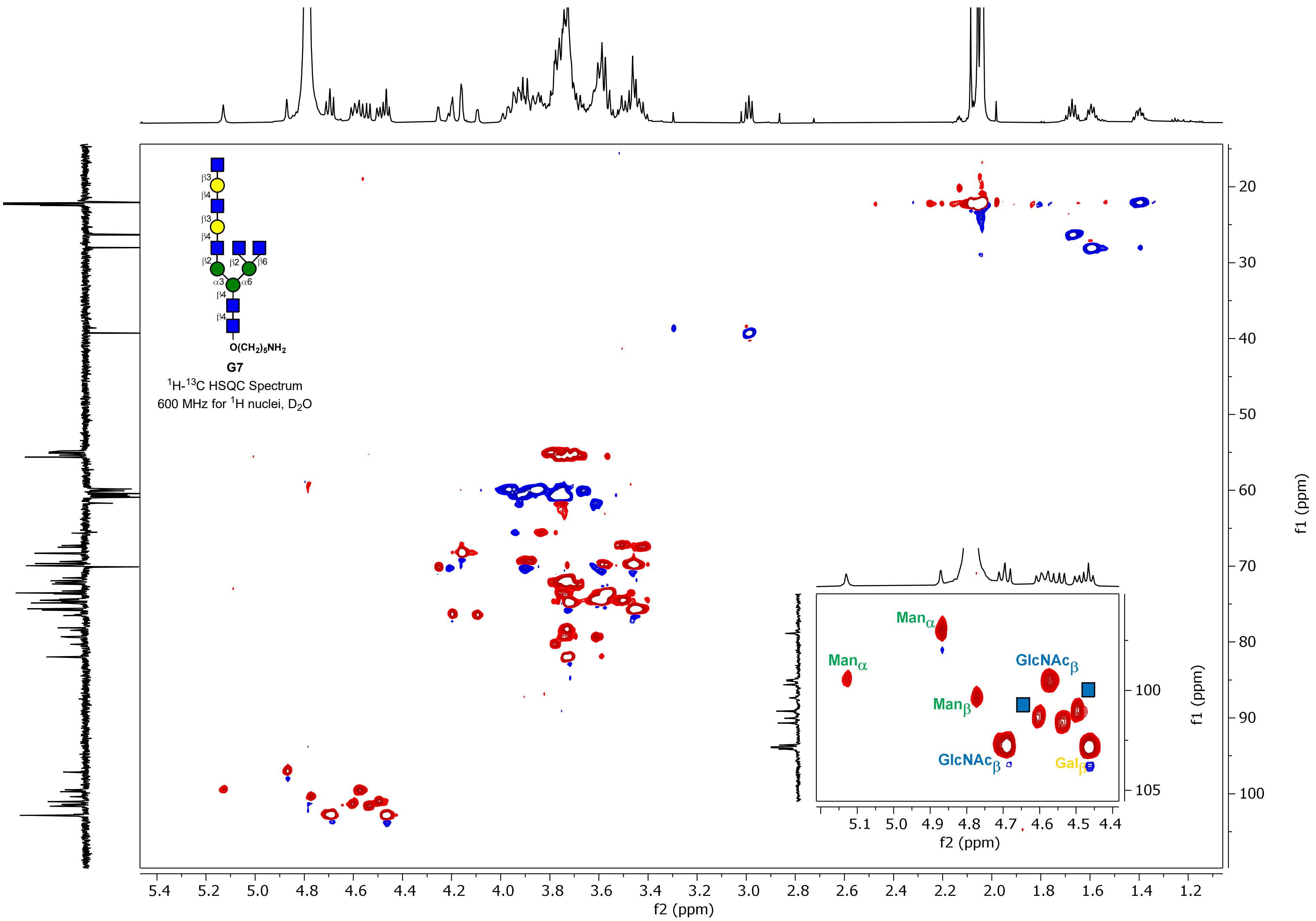

β3
β4
β3
β4
β2
β2
β6
α3
α6
β4
β4
O(CH2)5NH2
G7
1H-13C HSQC Spectrum
600 MHz for 1H nuclei, D2O
Manα
Manα
GlcNAcβ
Manβ
GlcNAcβ
Galβ
f2 (ppm)
f1 (ppm)

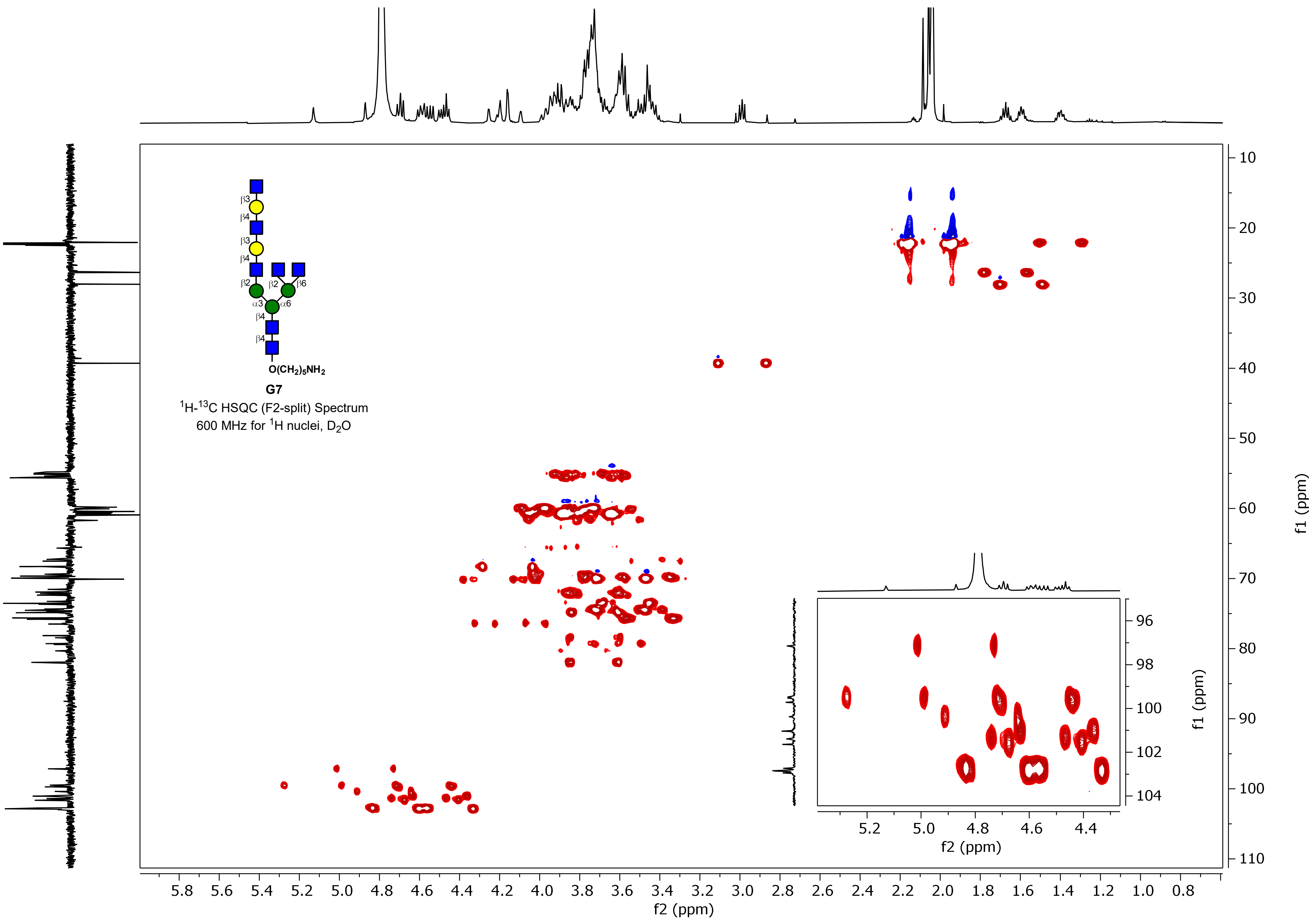
O(CH2)5NH2
G7
1H-13C HSQC (F2-split) Spectrum
600 MHz for 1H nuclei, D2O
f2 (ppm)
f1 (ppm)

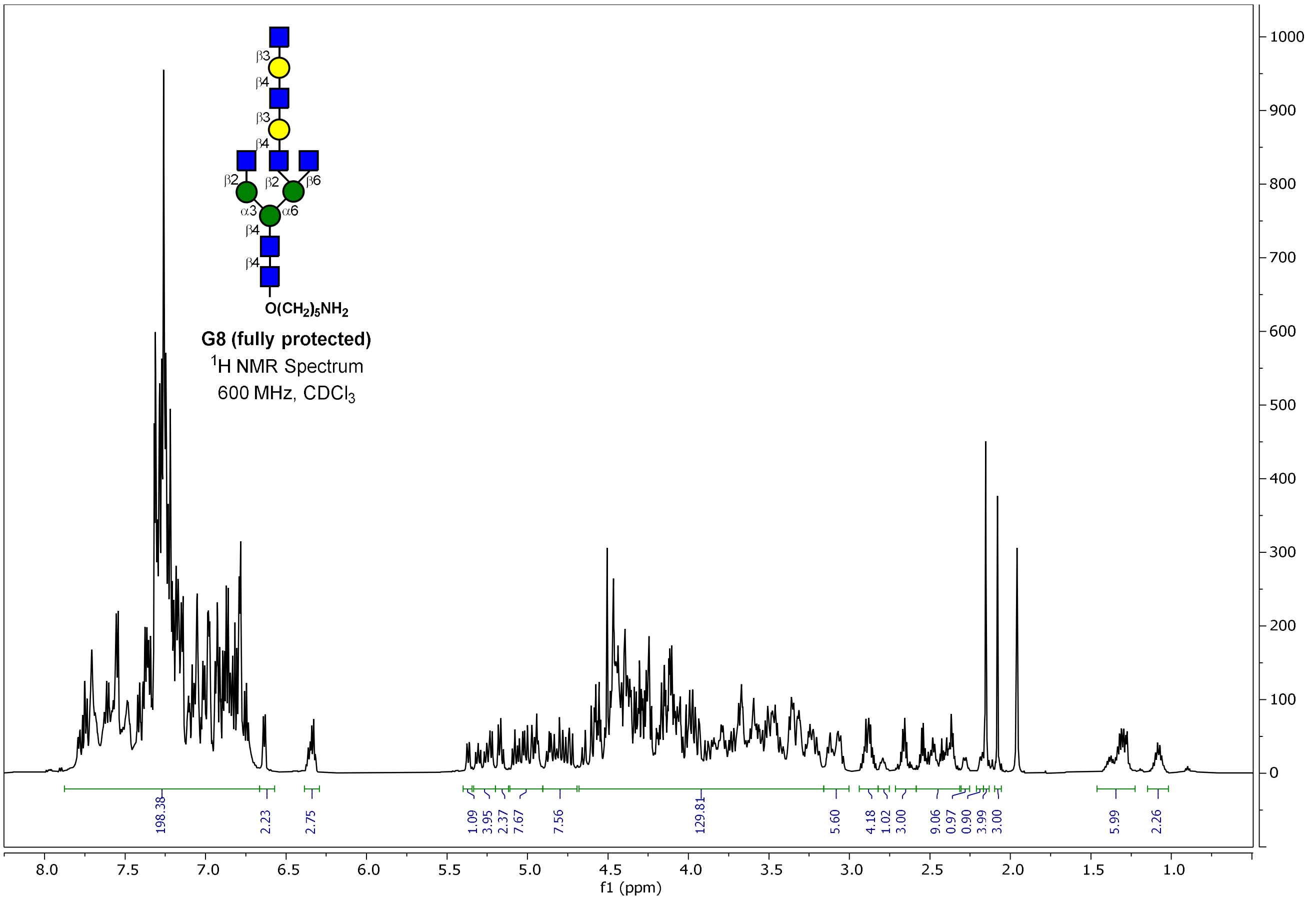
O(CH2)5NH2
G8 (fully protected)
1H NMR Spectrum
600 MHz, CDCl3
f1 (ppm)

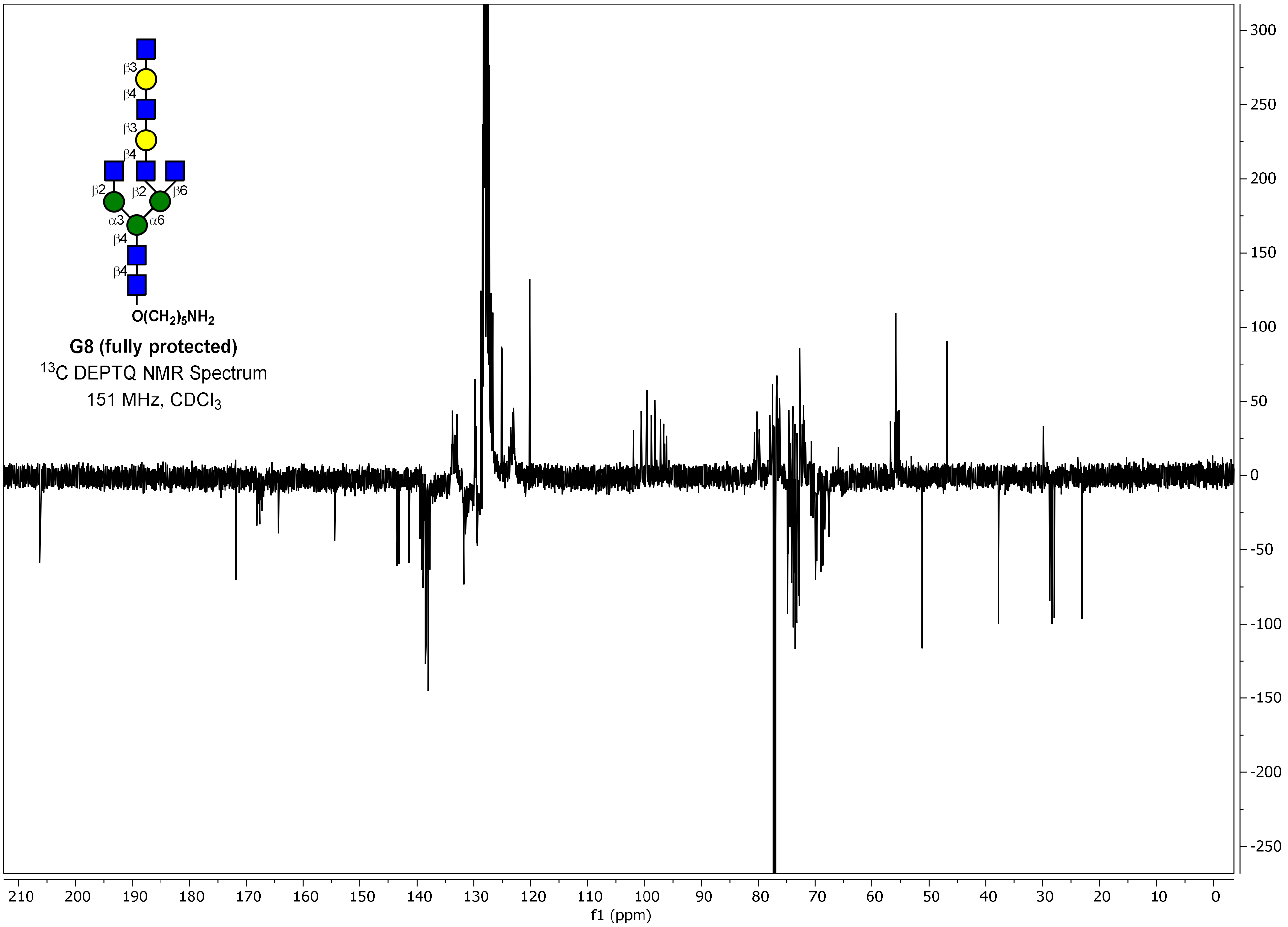
O(CH2)5NH2
G8 (fully protected)
13C DEPTQ NMR Spectrum
151 MHz, CDCl3
f1 (ppm)

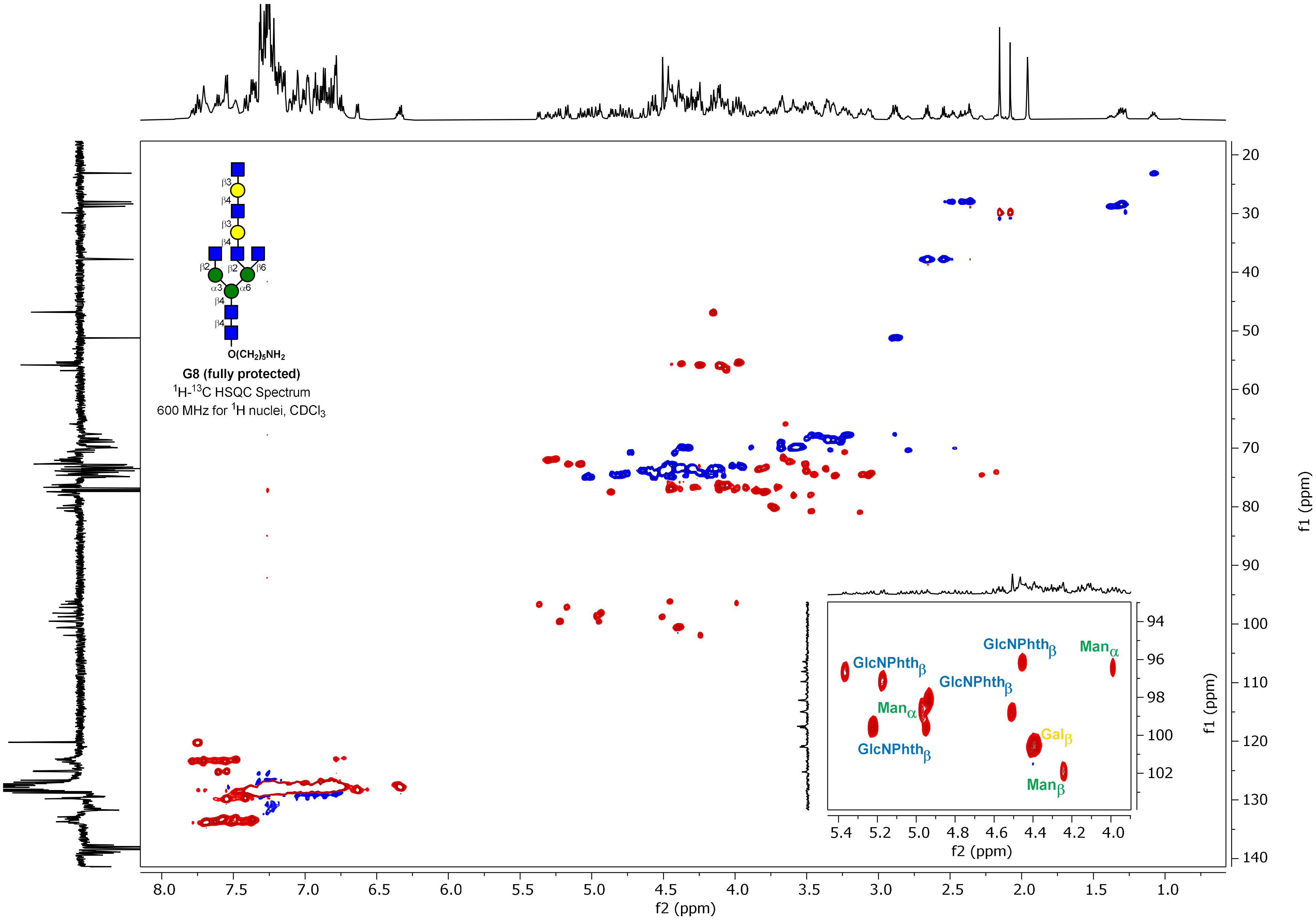

β3
β4
β3
β4
β2
β2
β6
α3
α6
β4
β4
O(CH2)5NH2
G8 (fully protected)
1H-13C HSQC Spectrum
600 MHz for 1H nuclei, CDCl3
GlcNPhthβ
GlcNPhthβ
Manα
GlcNPhthβ
Manα
Galβ
GlcNPhthβ
Manβ
f2 (ppm)
f1 (ppm)

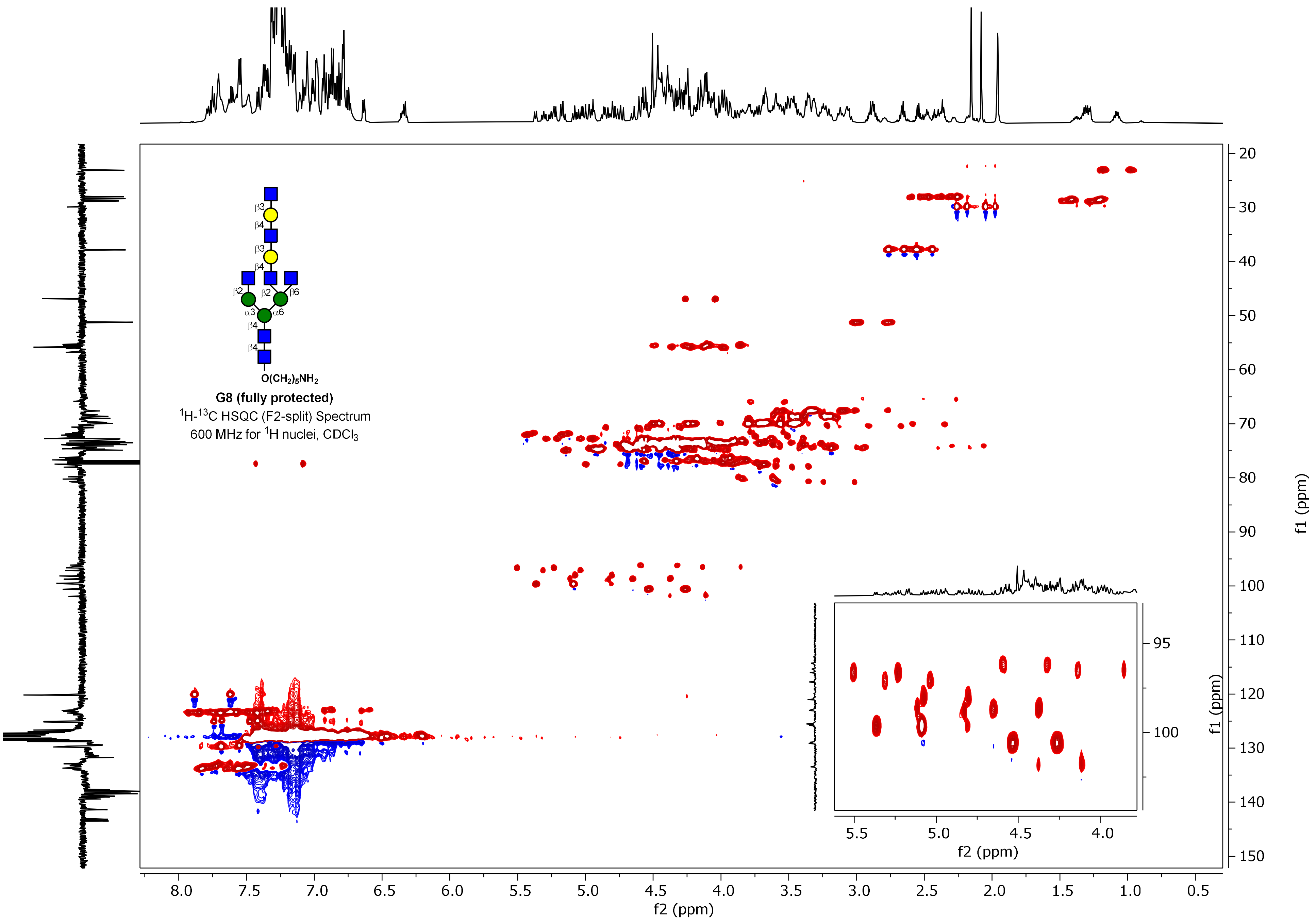
O(CH2)5NH2
G8 (fully protected)
1H-13C HSQC (F2-split) Spectrum
600 MHz for 1H nuclei, CDCl3
f2 (ppm)
f1 (ppm)

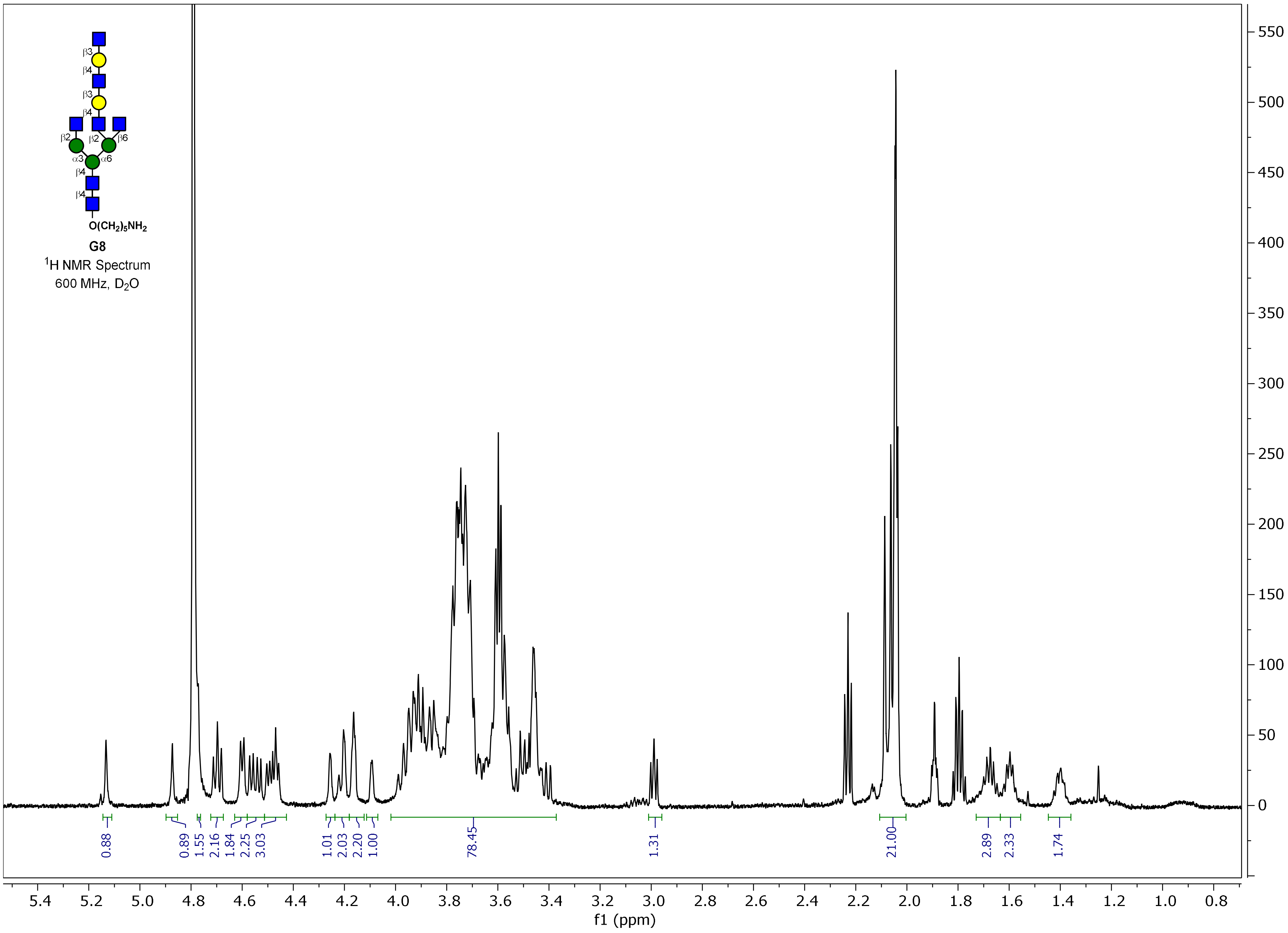

O(CH2)5NH2
G8
1H NMR Spectrum
600 MHz, D2O
f1 (ppm)
0.88
0.89
1.55
2.16
1.84
2.25
3.03
1.01
2.03
2.20
1.00
78.45
1.31
21.00
2.89
2.33
1.74

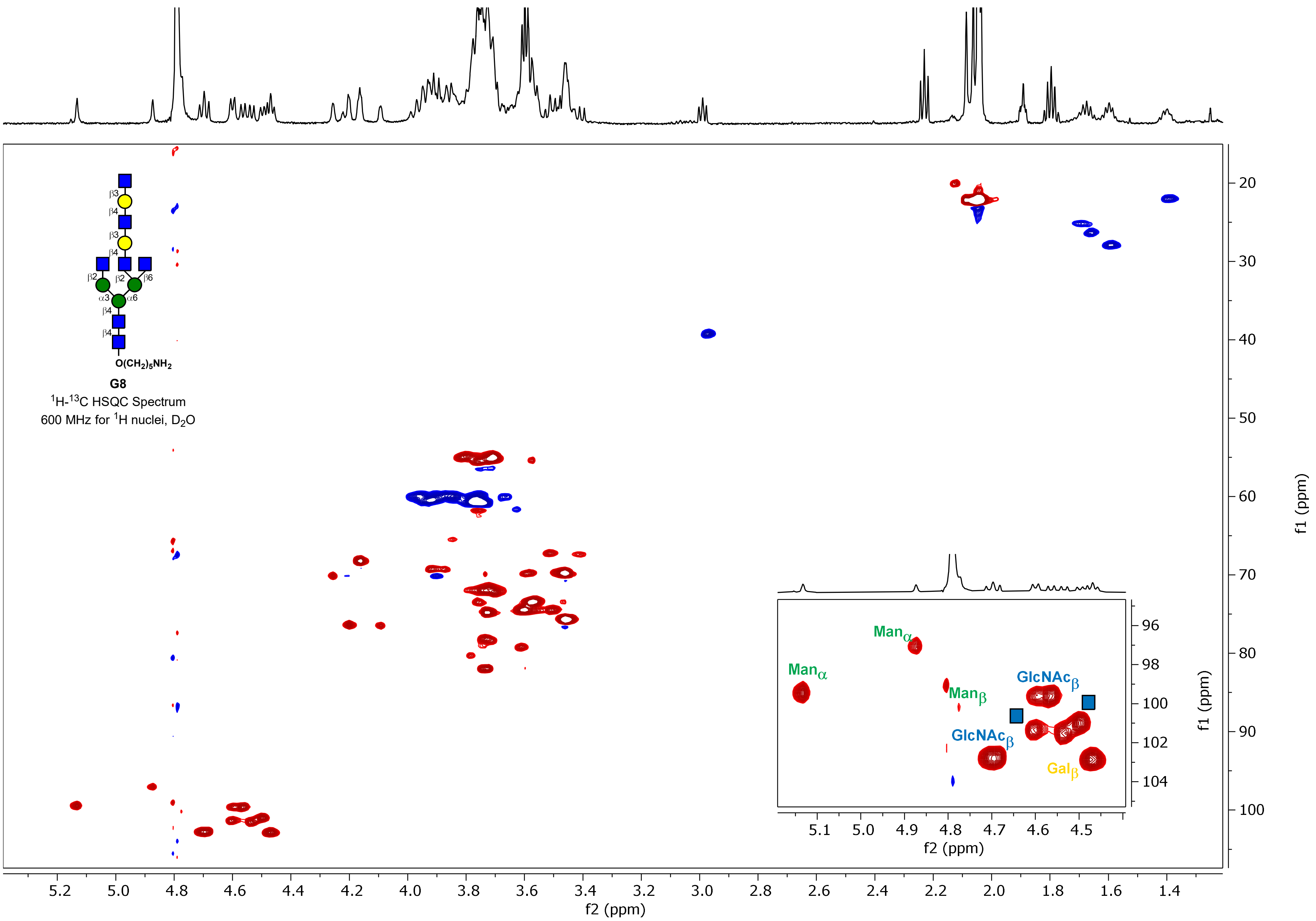

O(CH2)5NH2
G8
1H-13C HSQC Spectrum
600 MHz for 1H nuclei, D2O
Manα
Manα
Manβ
GlcNAcβ
GlcNAcβ
Galβ
f2 (ppm)
f1 (ppm)

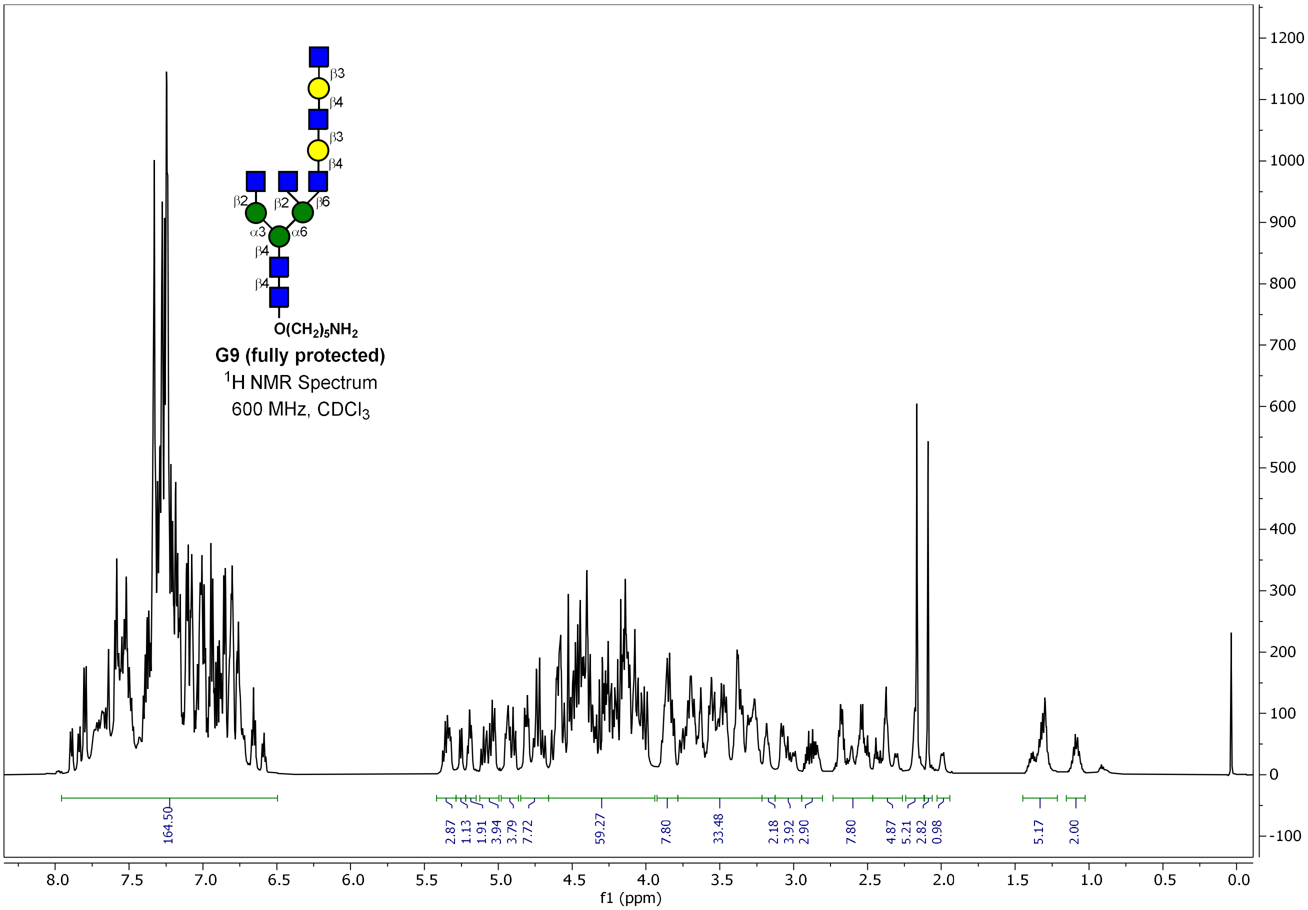
β3
β4
β3
β4
β2
β2
β6
α3
α6
β4
β4
O(CH2)5NH2
G9 (fully protected)
1H NMR Spectrum
600 MHz, CDCl3
164.50
2.87
1.13
1.91
3.94
3.79
7.72
59.27
7.80
33.48
2.18
3.92
2.90
7.80
4.87
5.21
2.82
0.98
5.17
2.00
f1 (ppm)

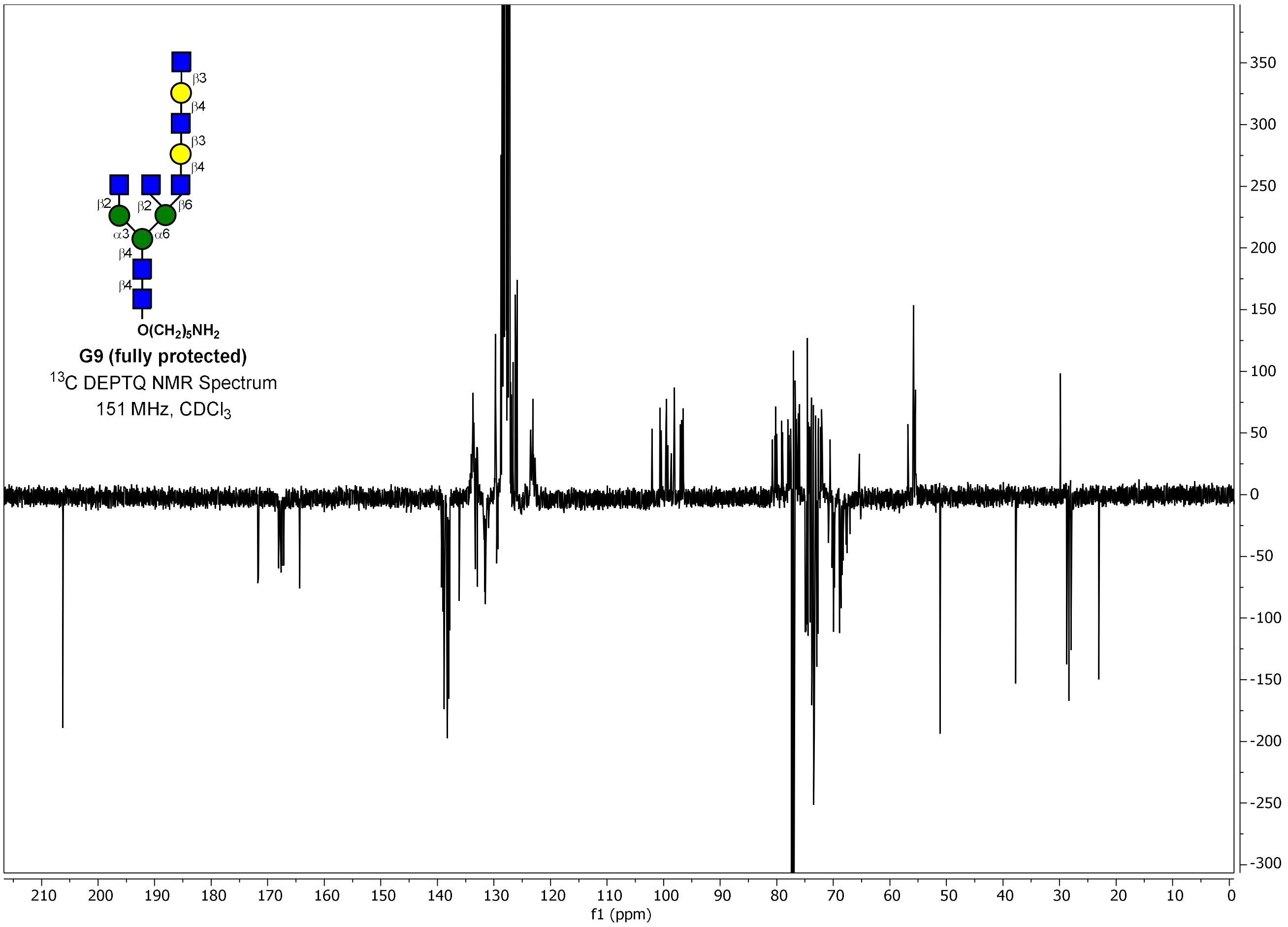
β3
β4
β3
β4
β2
β2
β6
α3
α6
β4
β4
O(CH2)5NH2
G9 (fully protected)
13C DEPTQ NMR Spectrum
151 MHz, CDCl3
f1 (ppm)

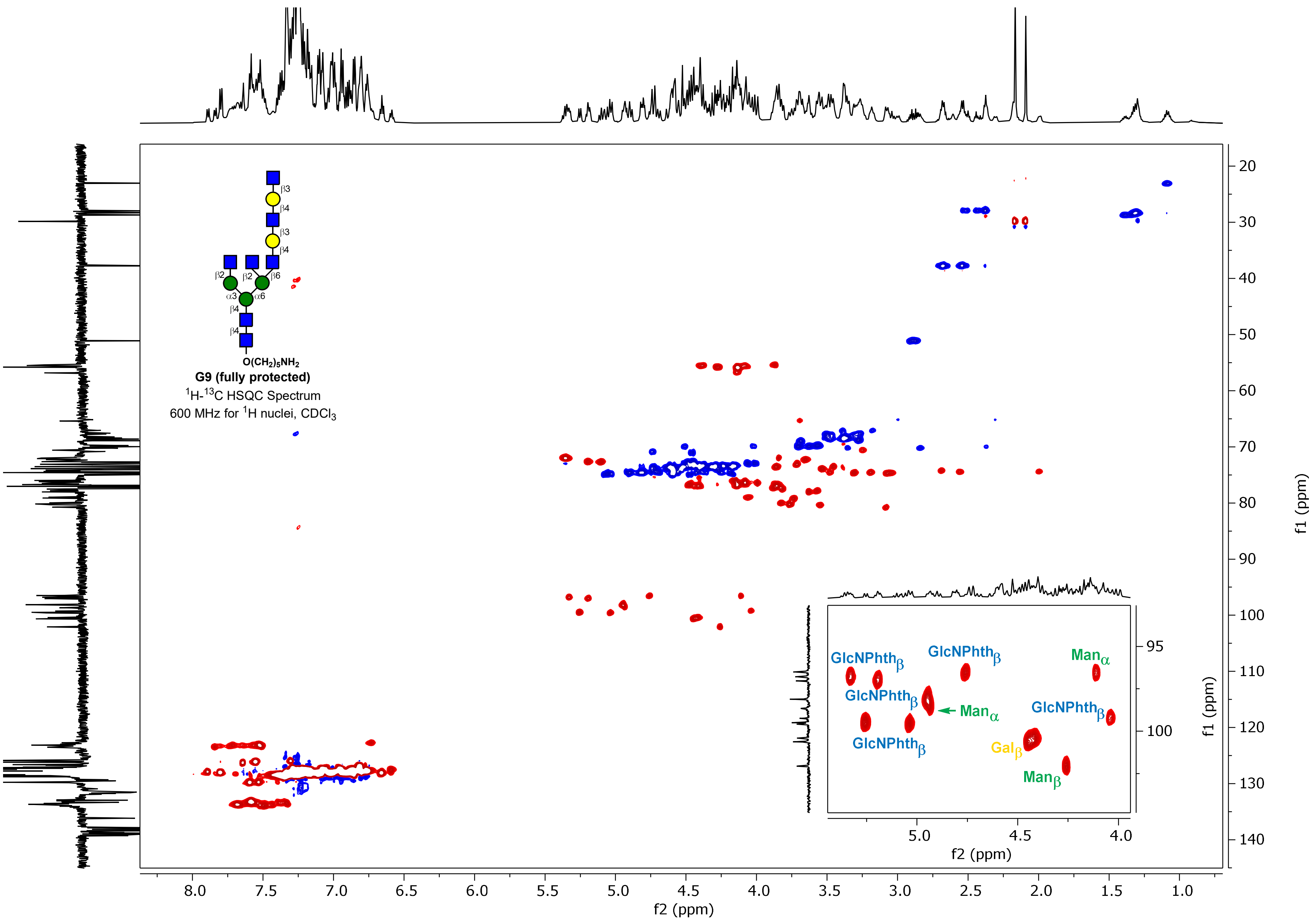

O(CH2)5NH2
G9 (fully protected)
1H-13C HSQC Spectrum
600 MHz for 1H nuclei, CDCl3
GlcNPhthβ
GlcNPhthβ
Manα
GlcNPhthβ
Manα
GlcNPhthβ
GlcNPhthβ
Galβ
Manβ
f2 (ppm)
f1 (ppm)

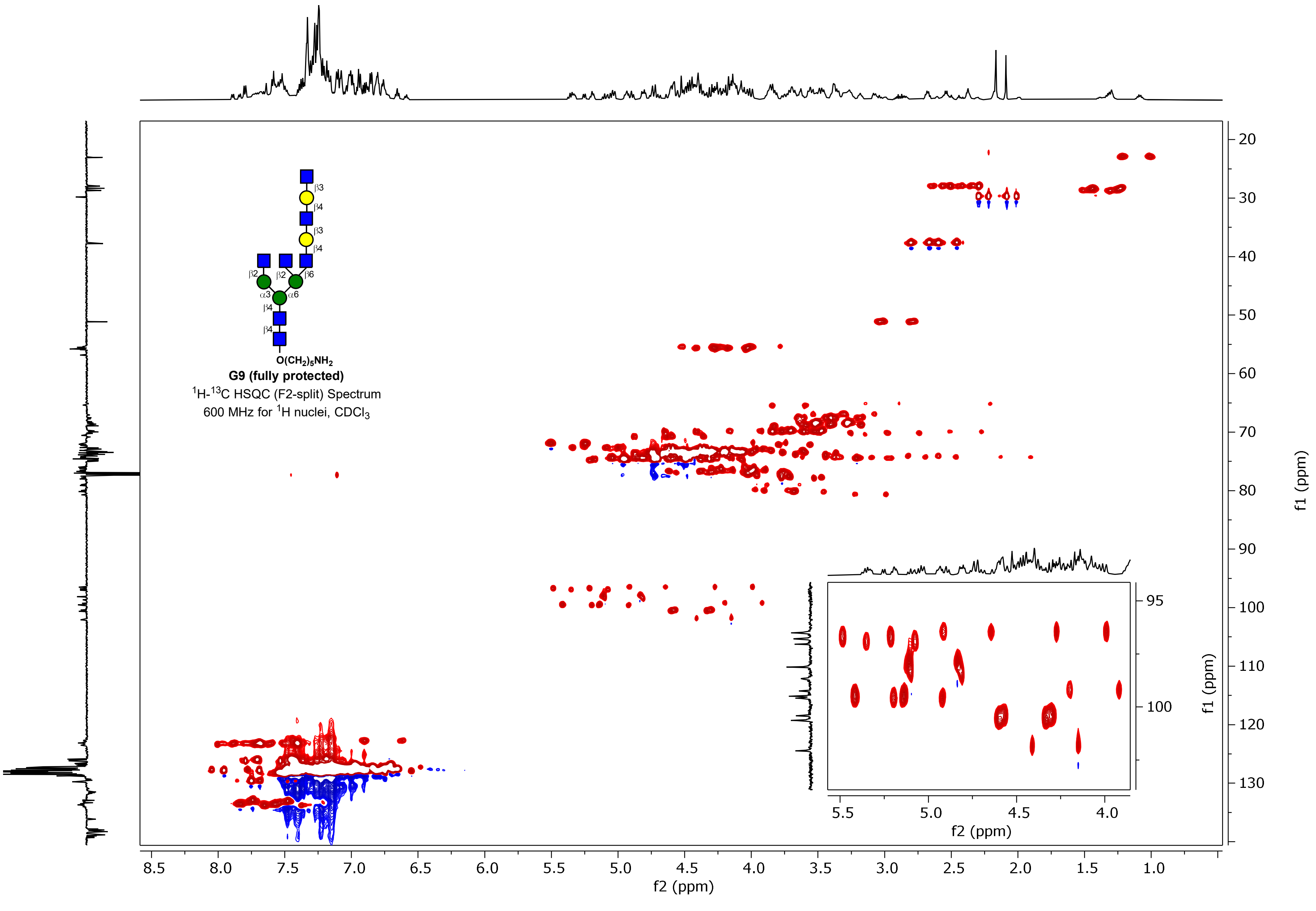

β3
β4
β3
β4
β2
β2
β6
α3
α6
β4
β4
O(CH2)5NH2
G9 (fully protected)
1H-13C HSQC (F2-split) Spectrum
600 MHz for 1H nuclei, CDCl3
f2 (ppm)
f1 (ppm)

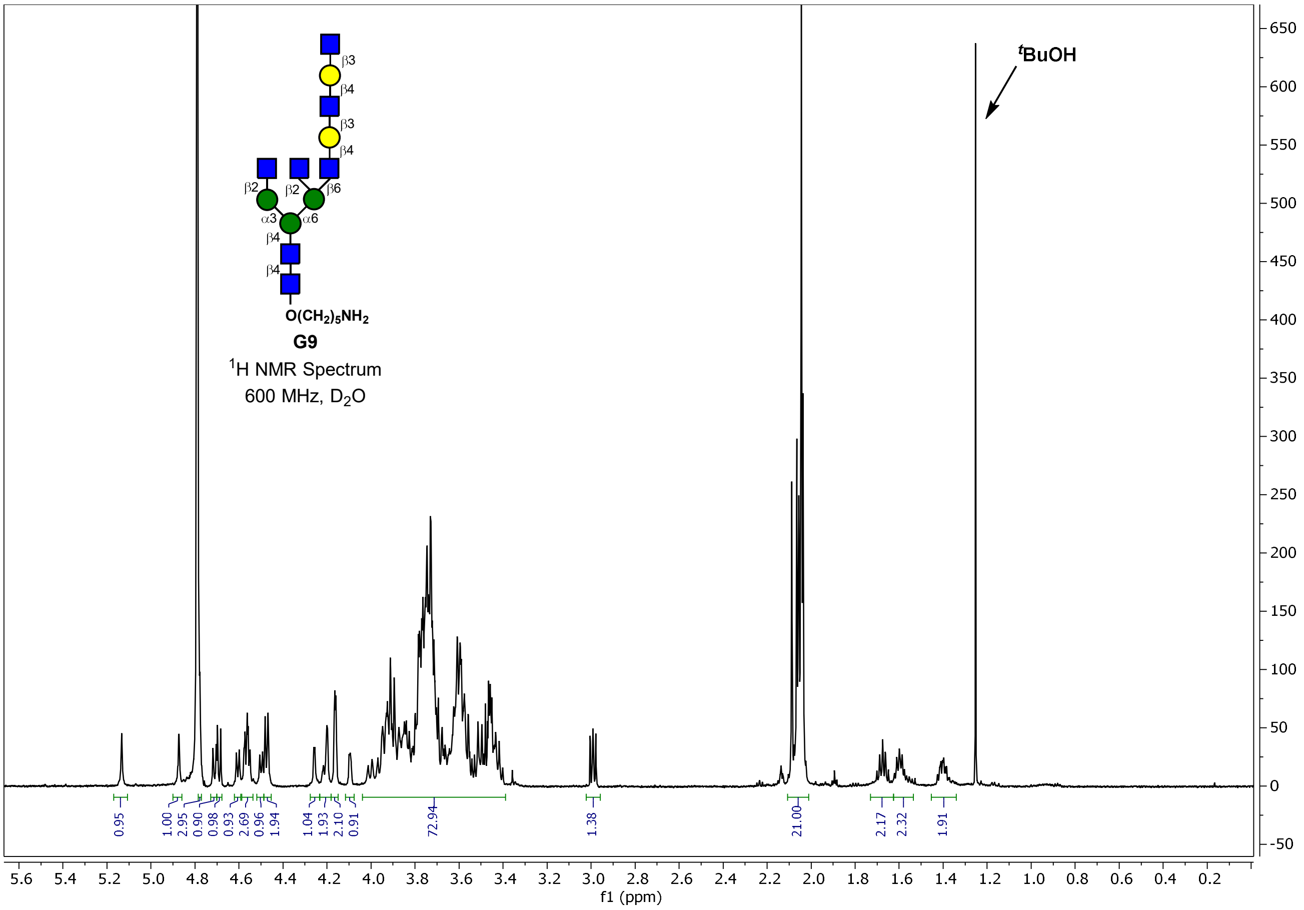
tBuOH
O(CH2)5NH2
G9
1H NMR Spectrum
600 MHz, D2O
f1 (ppm)

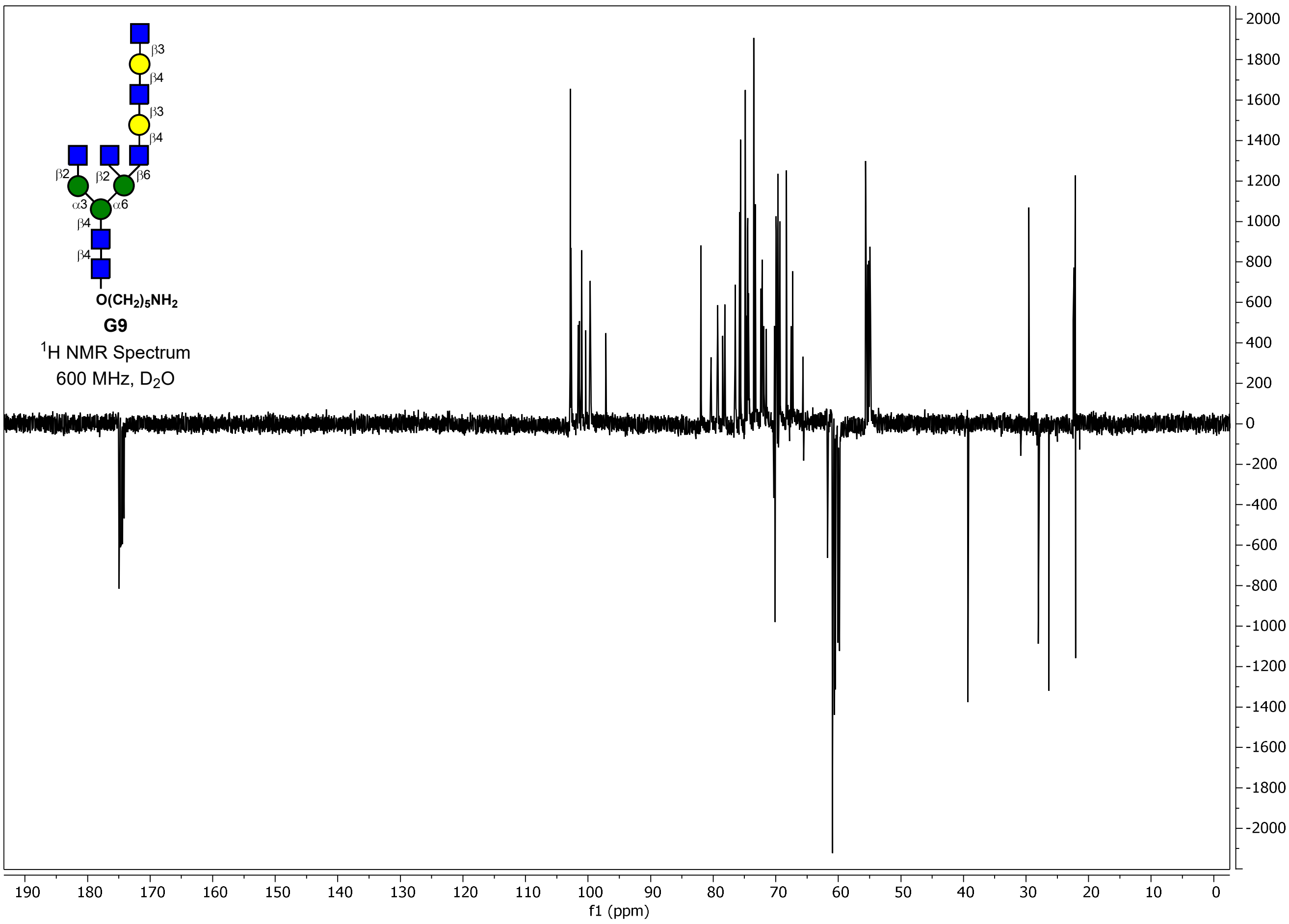
O(CH2)5NH2
G9
1H NMR Spectrum
600 MHz, D2O
f1 (ppm)

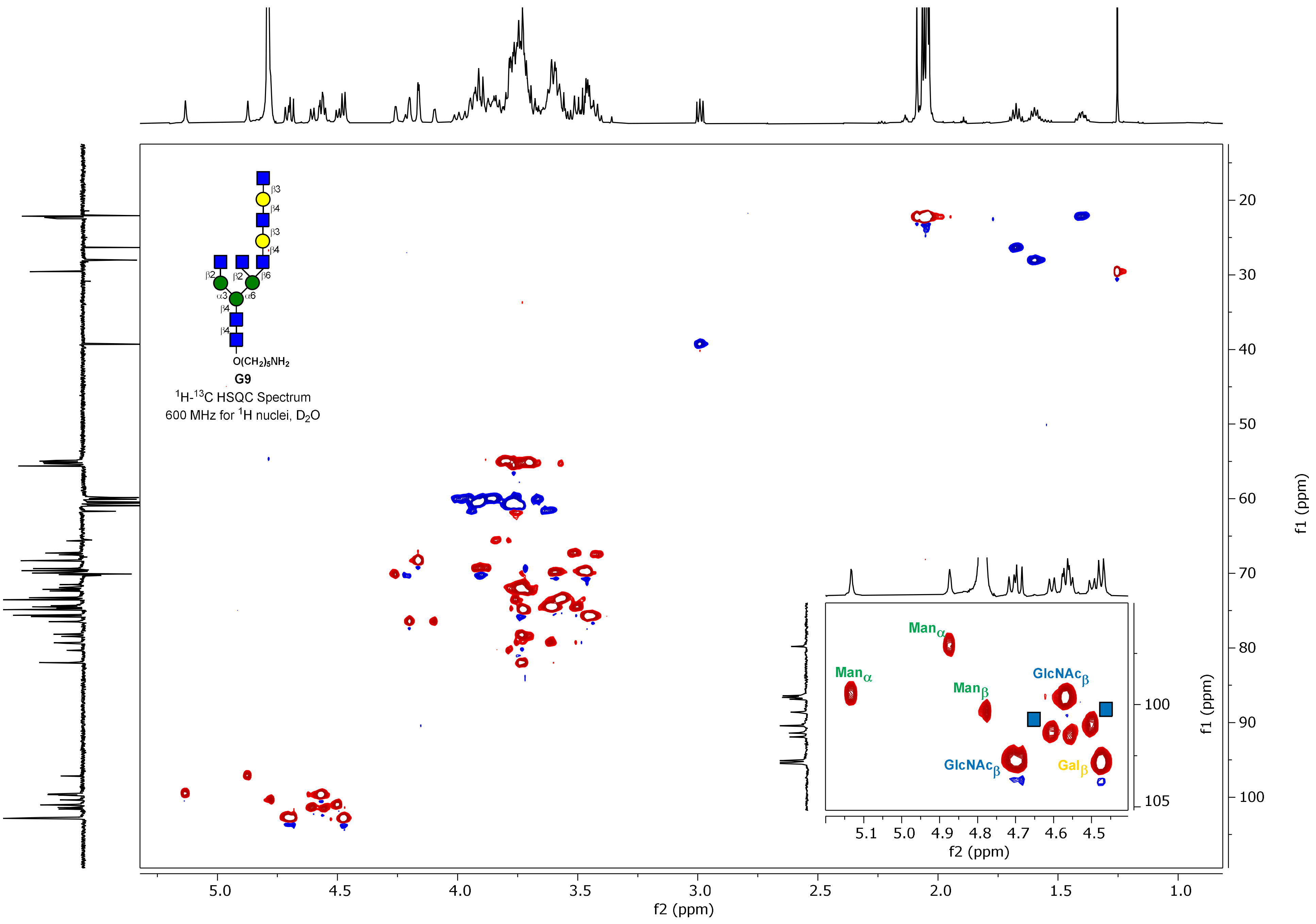

β3
β4
β3
β4
β2
β2
β6
α3
α6
β4
β4
O(CH2)5NH2
G9
1H-13C HSQC Spectrum
600 MHz for 1H nuclei, D2O
Manα
Manα
Manβ
GlcNAcβ
GlcNAcβ
Galβ
f2 (ppm)
f1 (ppm)

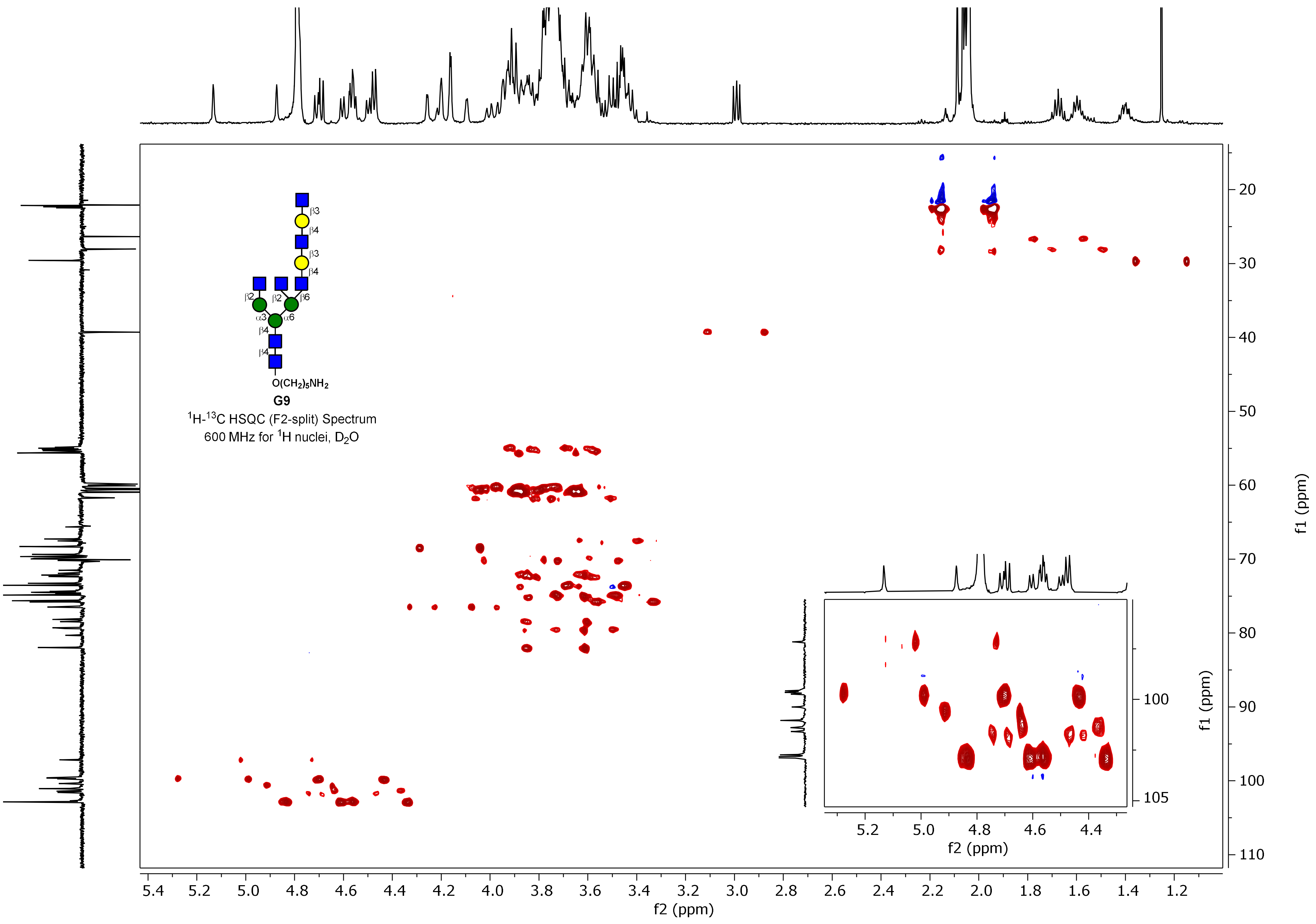

β3
β4
β3
β4
β2
β2
β6
α3
α6
β4
β4
O(CH2)5NH2
G9
1H-13C HSQC (F2-split) Spectrum
600 MHz for 1H nuclei, D2O
f2 (ppm)
f1 (ppm)

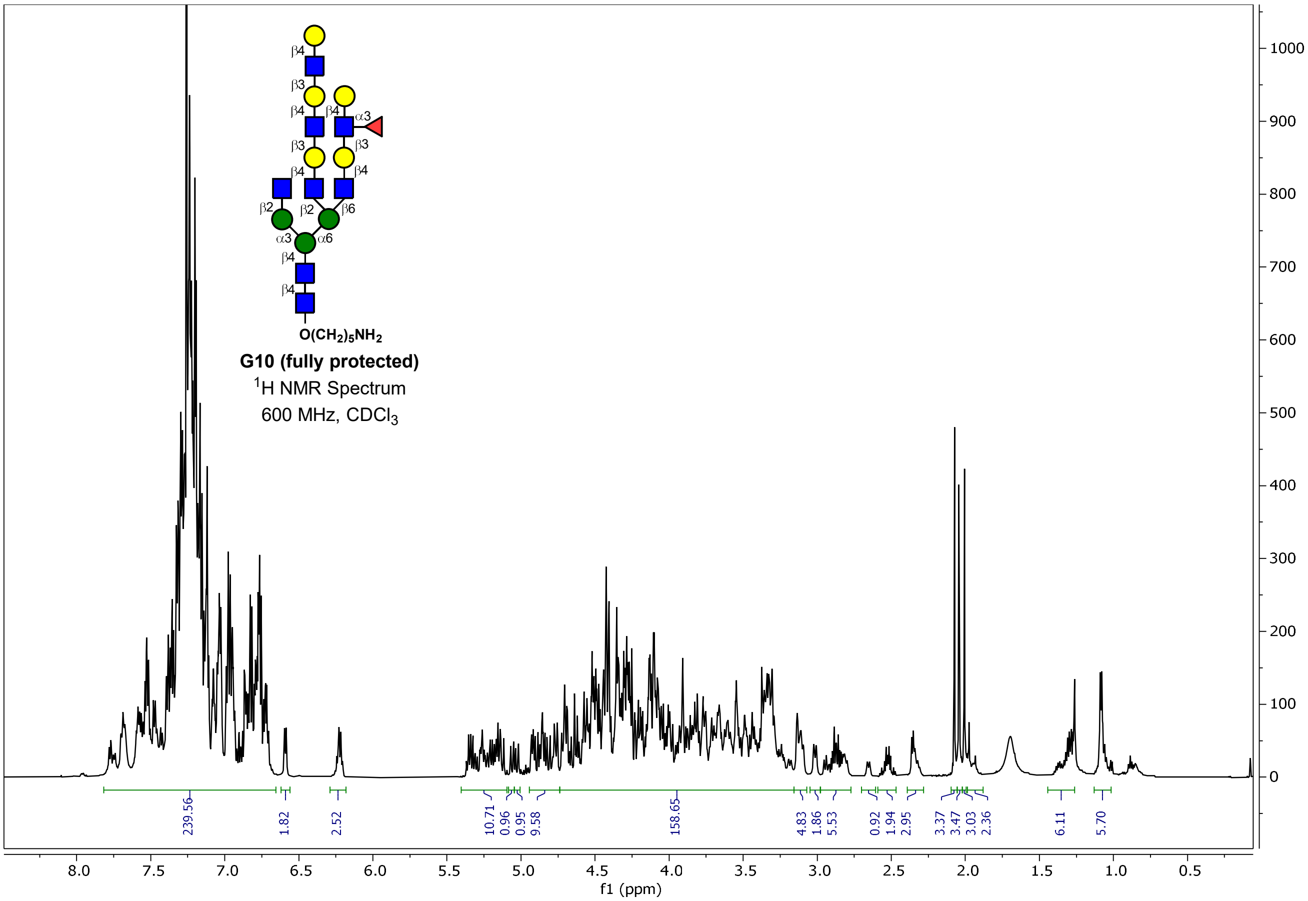

O(CH2)5NH2
G10 (fully protected)
1H NMR Spectrum
600 MHz, CDCl3
f1 (ppm)

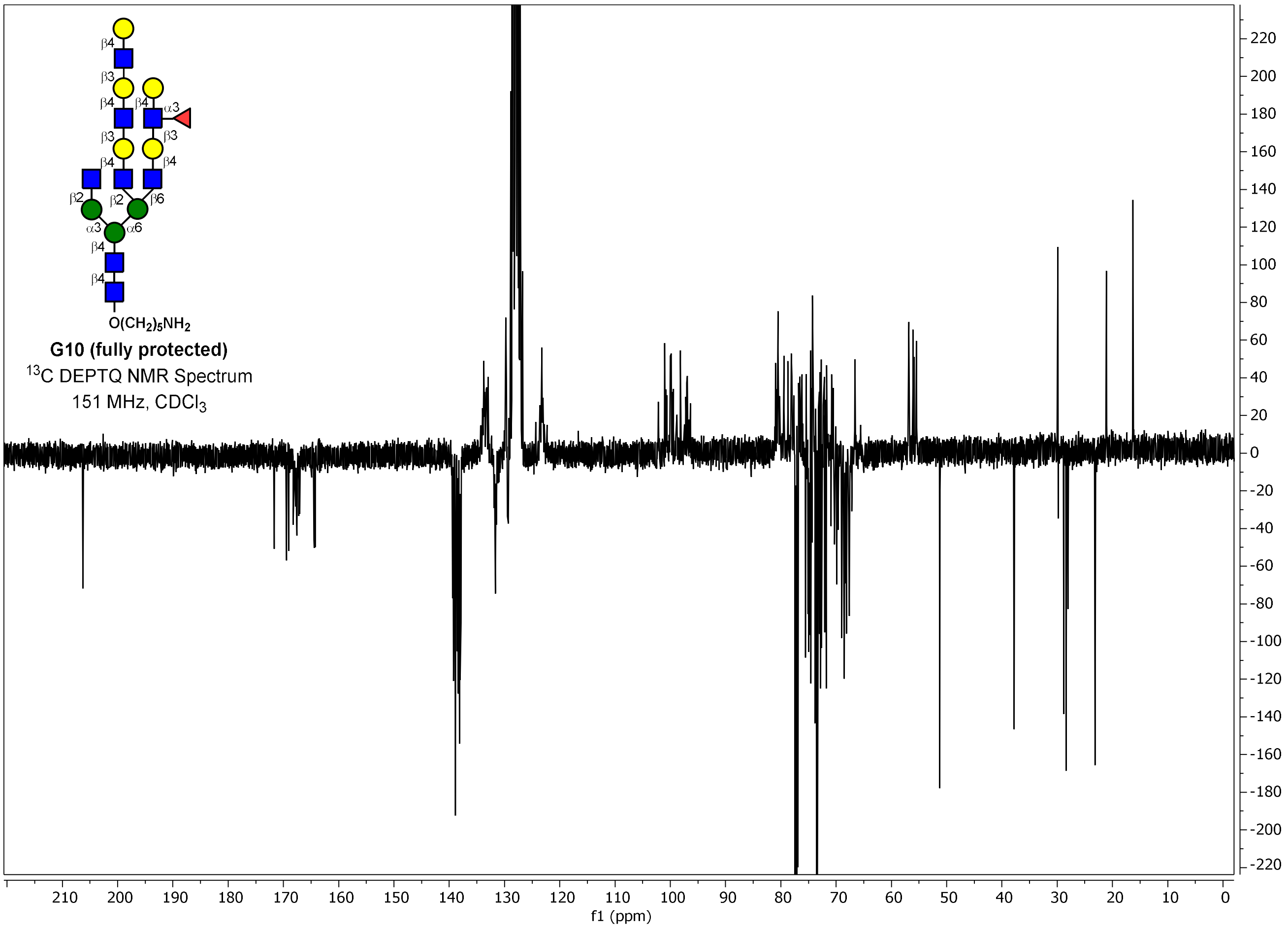

O(CH2)5NH2
G10 (fully protected)
13C DEPTQ NMR Spectrum
151 MHz, CDCl3
f1 (ppm)

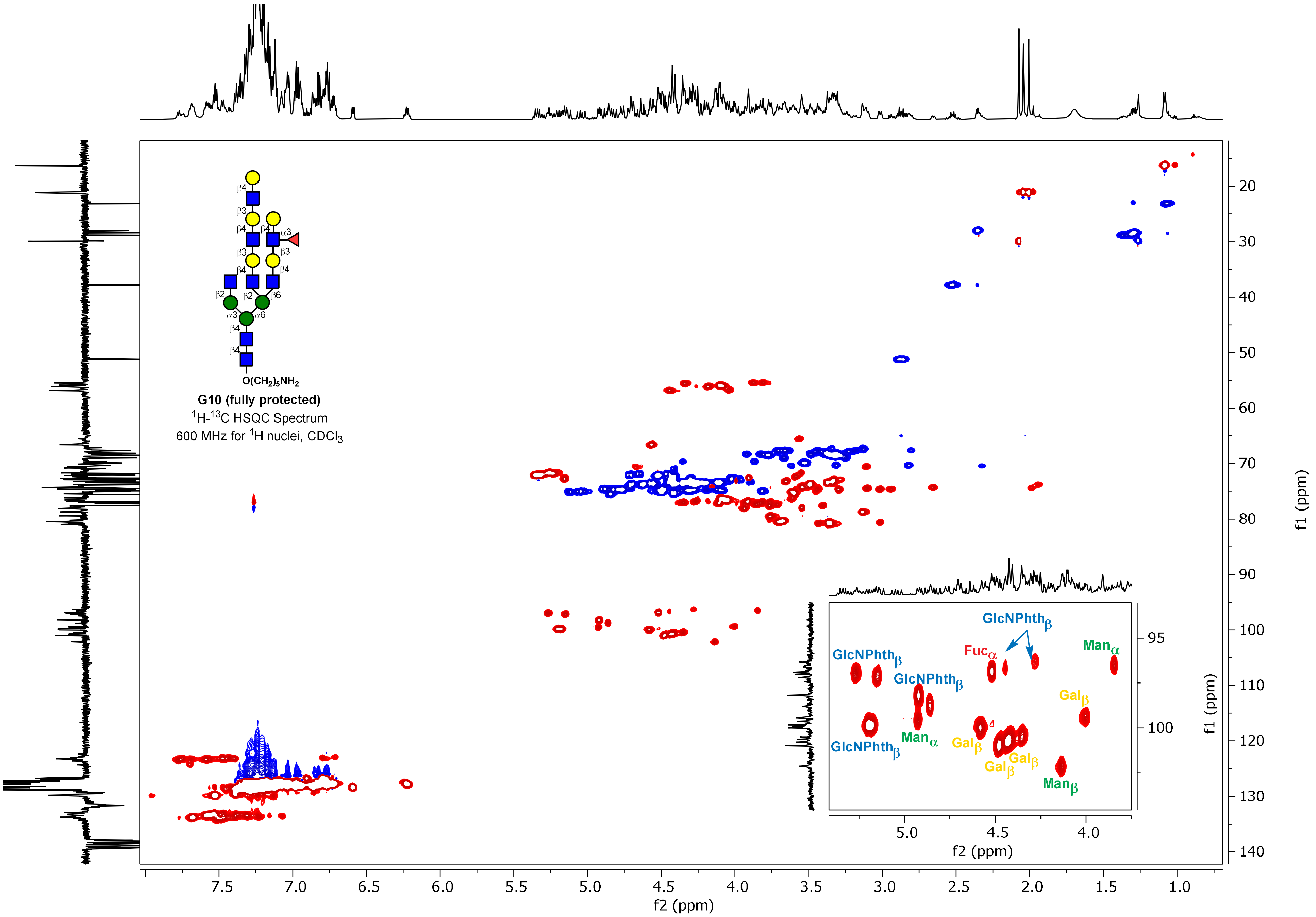
O(CH2)5NH2
G10 (fully protected)
1H-13C HSQC Spectrum
600 MHz for 1H nuclei, CDCl3
GlcNPhthβ
Fucα
Manα
GlcNPhthβ
GlcNPhthβ
Galβ
GlcNPhthβ
Manα
Galβ
Galβ
Galβ
Manβ
f2 (ppm)
f1 (ppm)

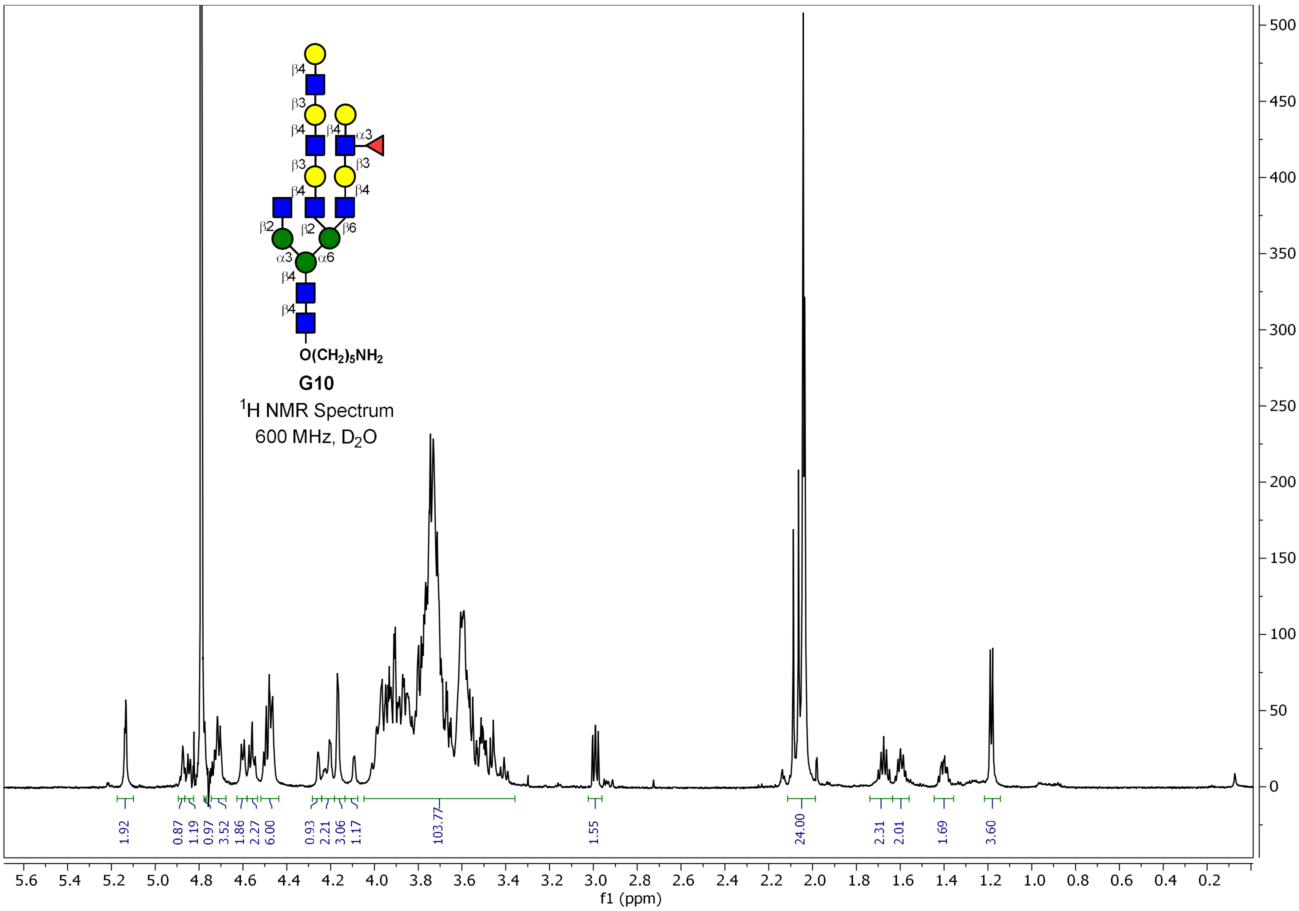

O(CH2)5NH2
G10
1H NMR Spectrum
600 MHz, D2O
f1 (ppm)

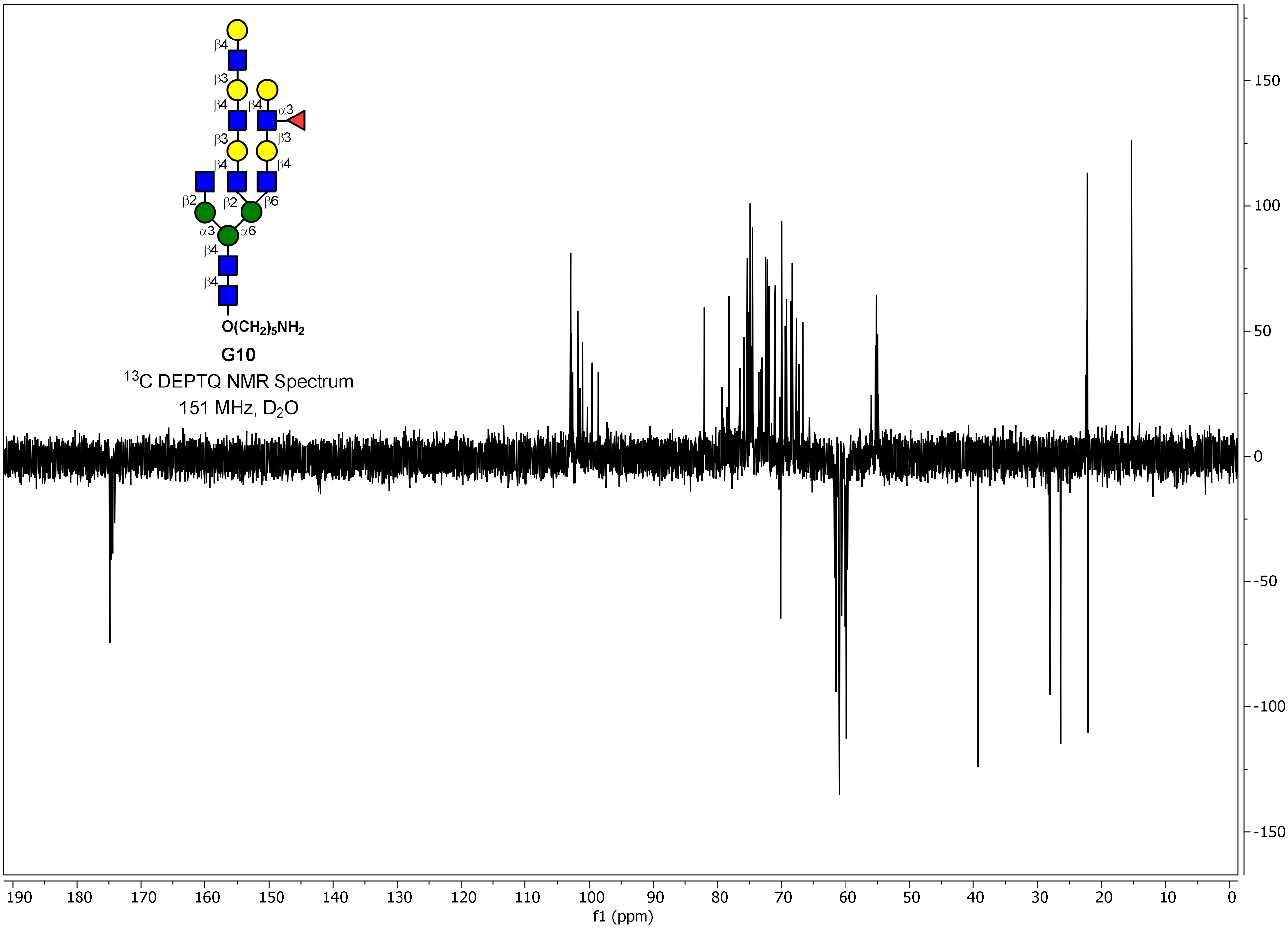

O(CH2)5NH2
G10
13C DEPTQ NMR Spectrum
151 MHz, D2O
f1 (ppm)

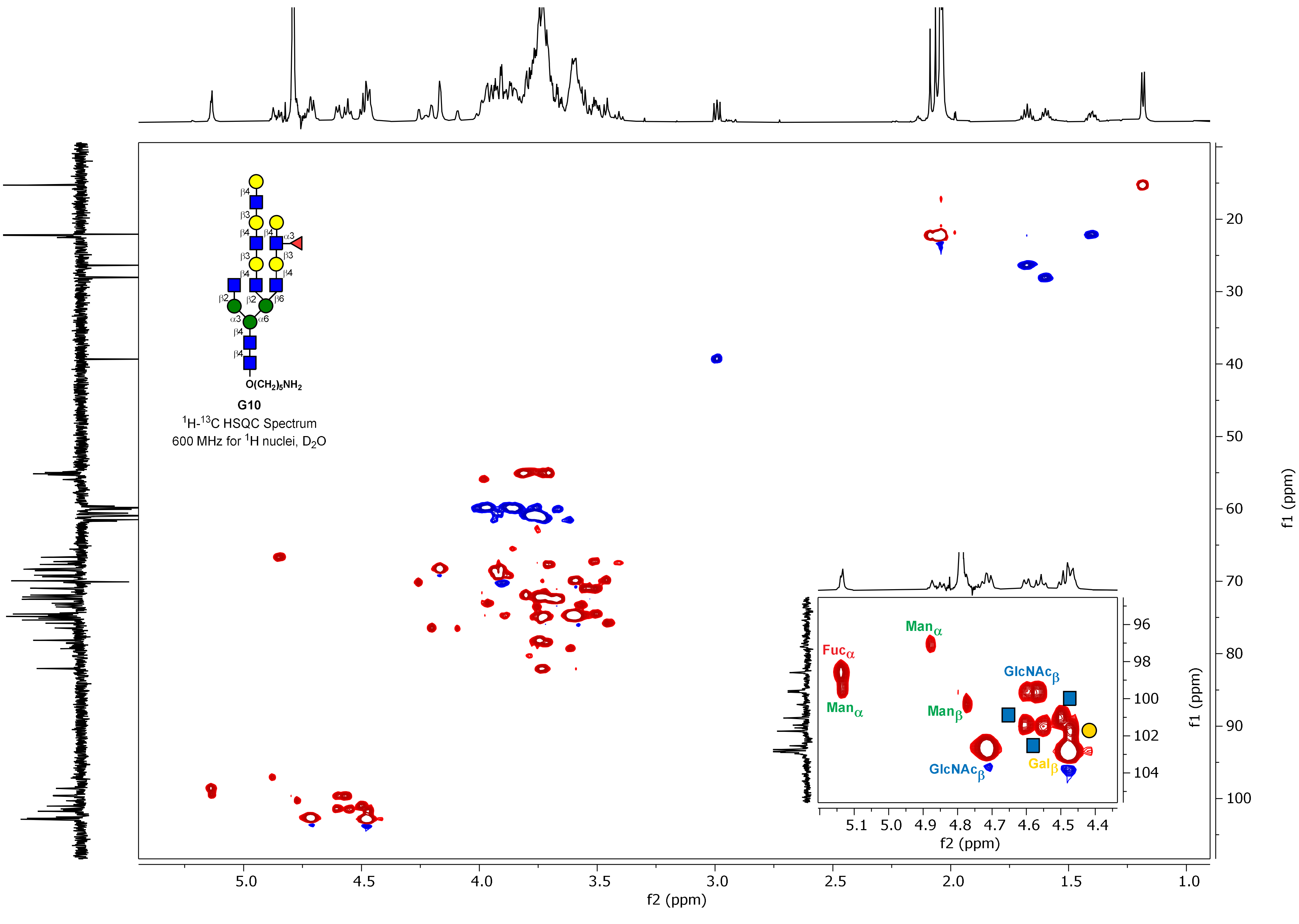

O(CH2)5NH2
G10
1H-13C HSQC Spectrum
600 MHz for 1H nuclei, D2O
Fucα
Manα
Manα
Manβ
GlcNAcβ
GlcNAcβ
Galβ
f2 (ppm)
f1 (ppm)
f2 (ppm)
f1 (ppm)

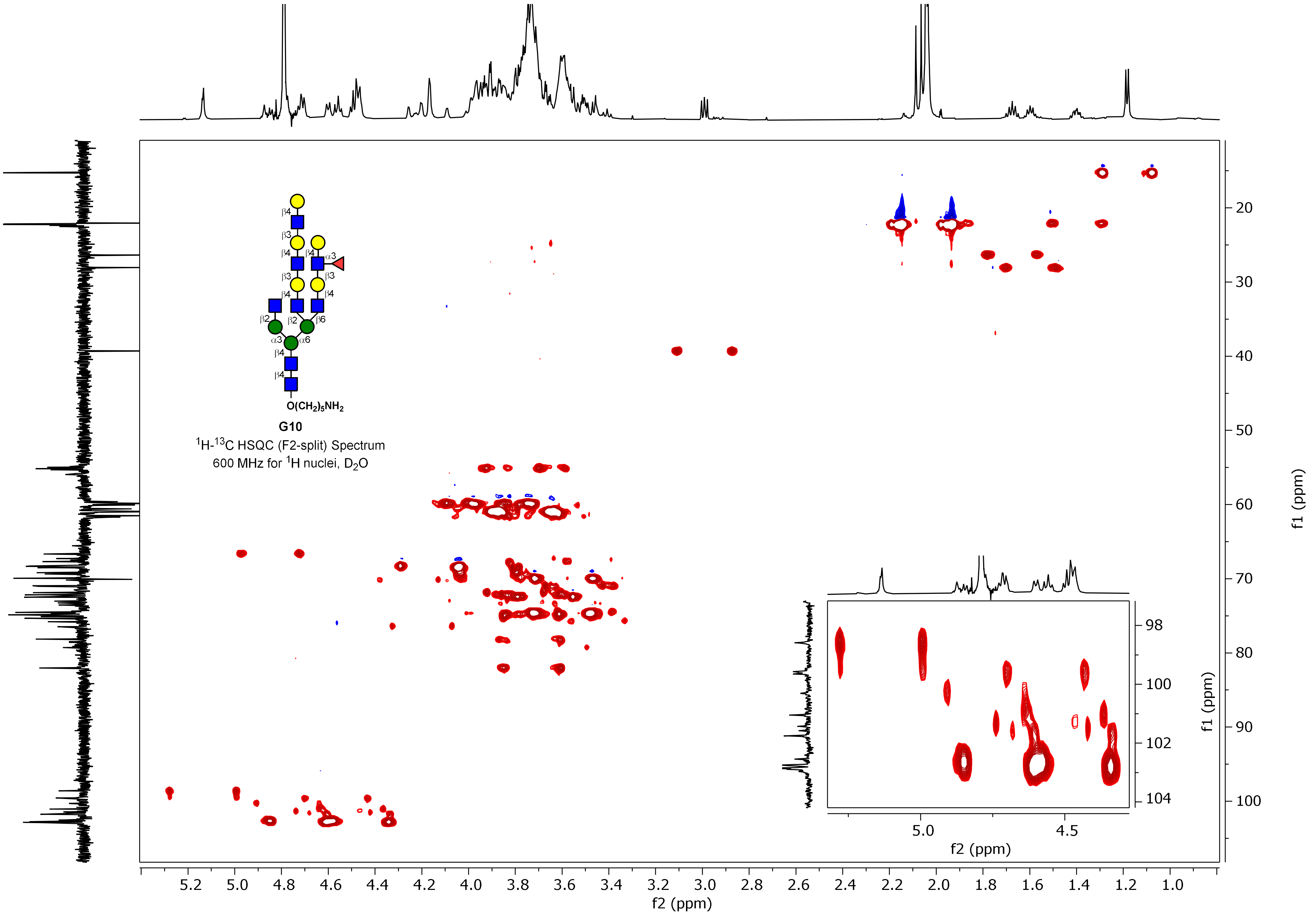

β4
β3
β4
β4
α3
β3
β3
β4
β4
β4
β2
β2
β6
α3
α6
β4
β4
O(CH2)5NH2
G10
1H-13C HSQC (F2-split) Spectrum
600 MHz for 1H nuclei, D2O
f2 (ppm)
f1 (ppm)

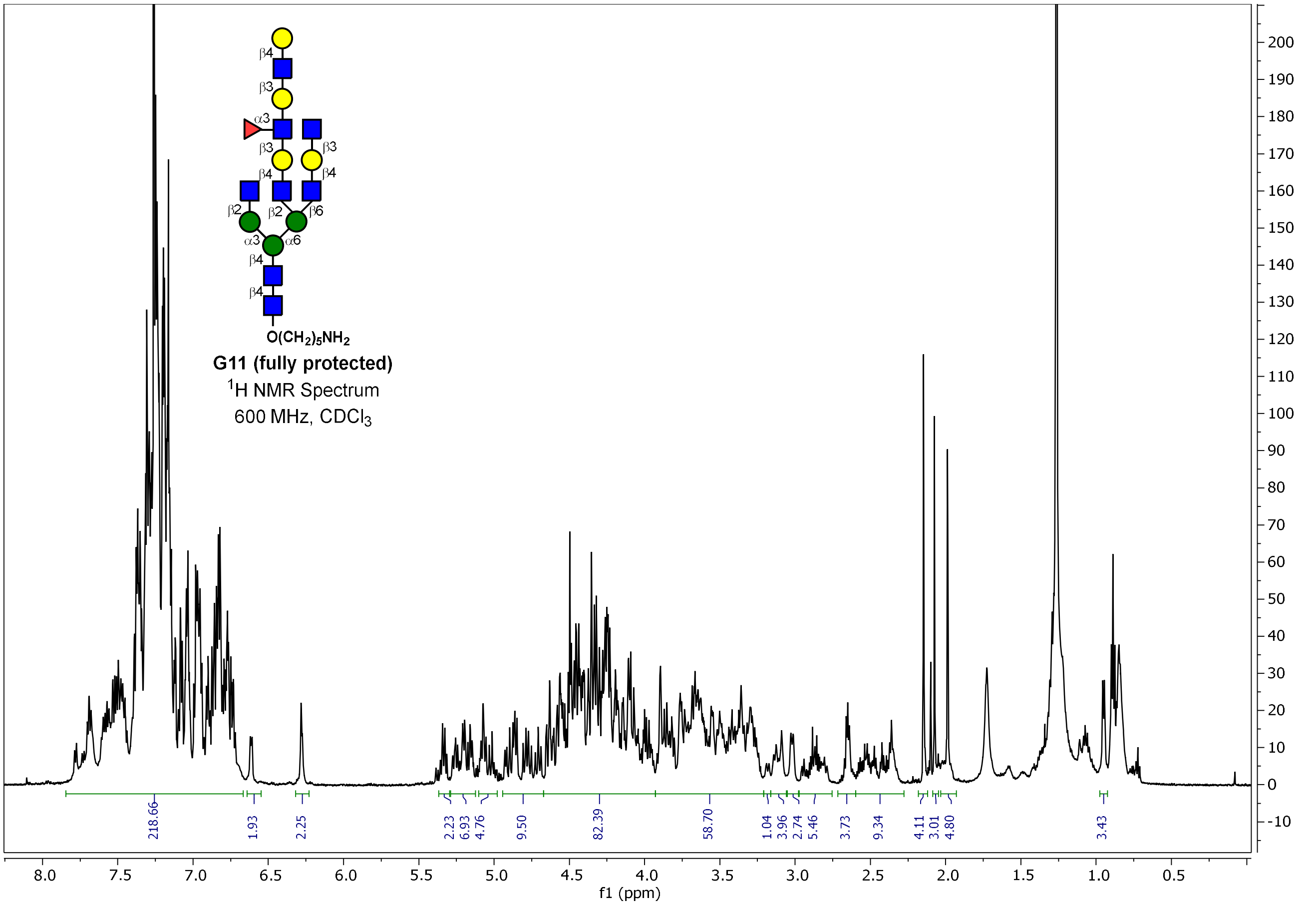

O(CH2)5NH2
G11 (fully protected)
1H NMR Spectrum
600 MHz, CDCl3
f1 (ppm)

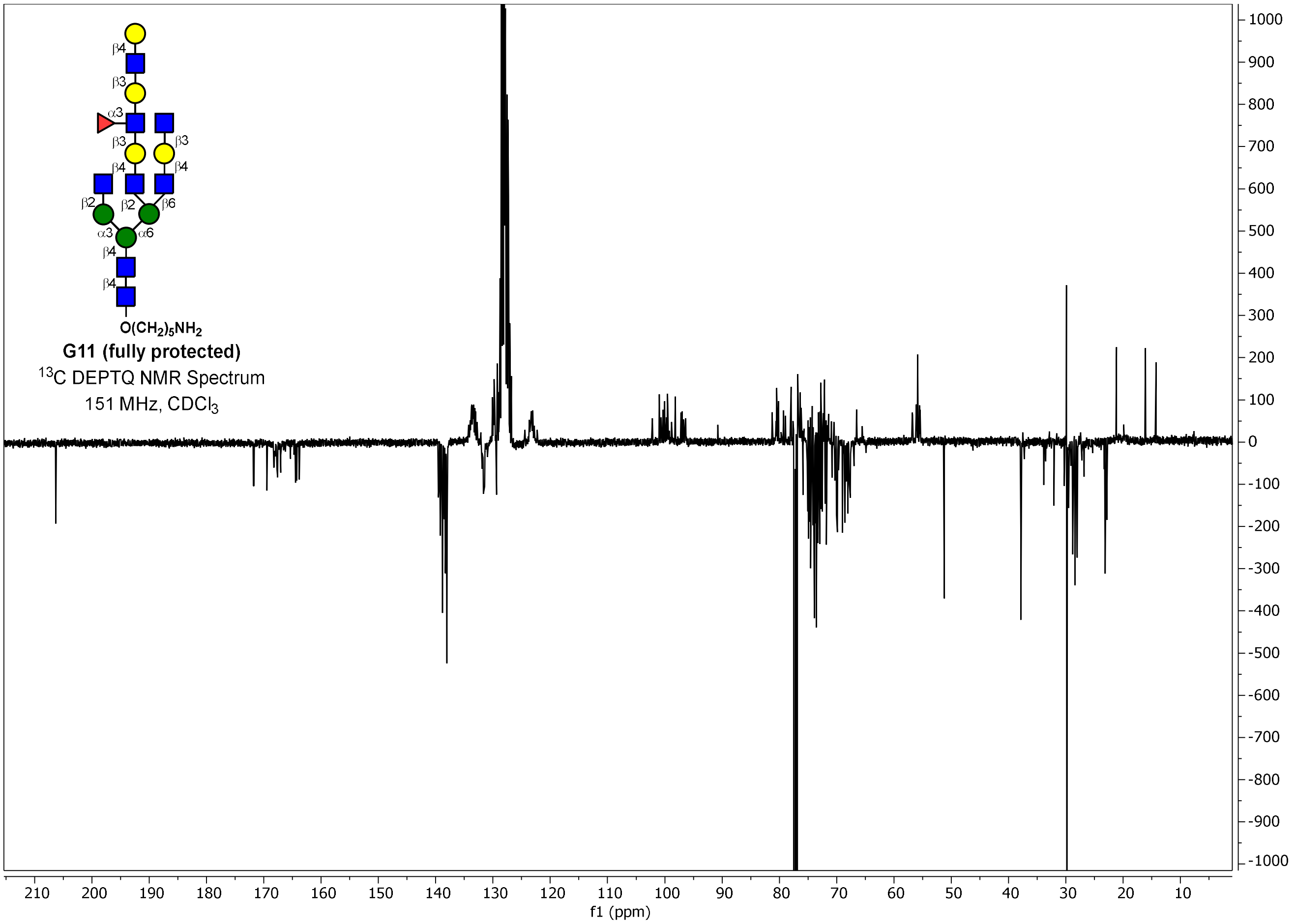

O(CH2)5NH2
G11 (fully protected)
13C DEPTQ NMR Spectrum
151 MHz, CDCl3
f1 (ppm)

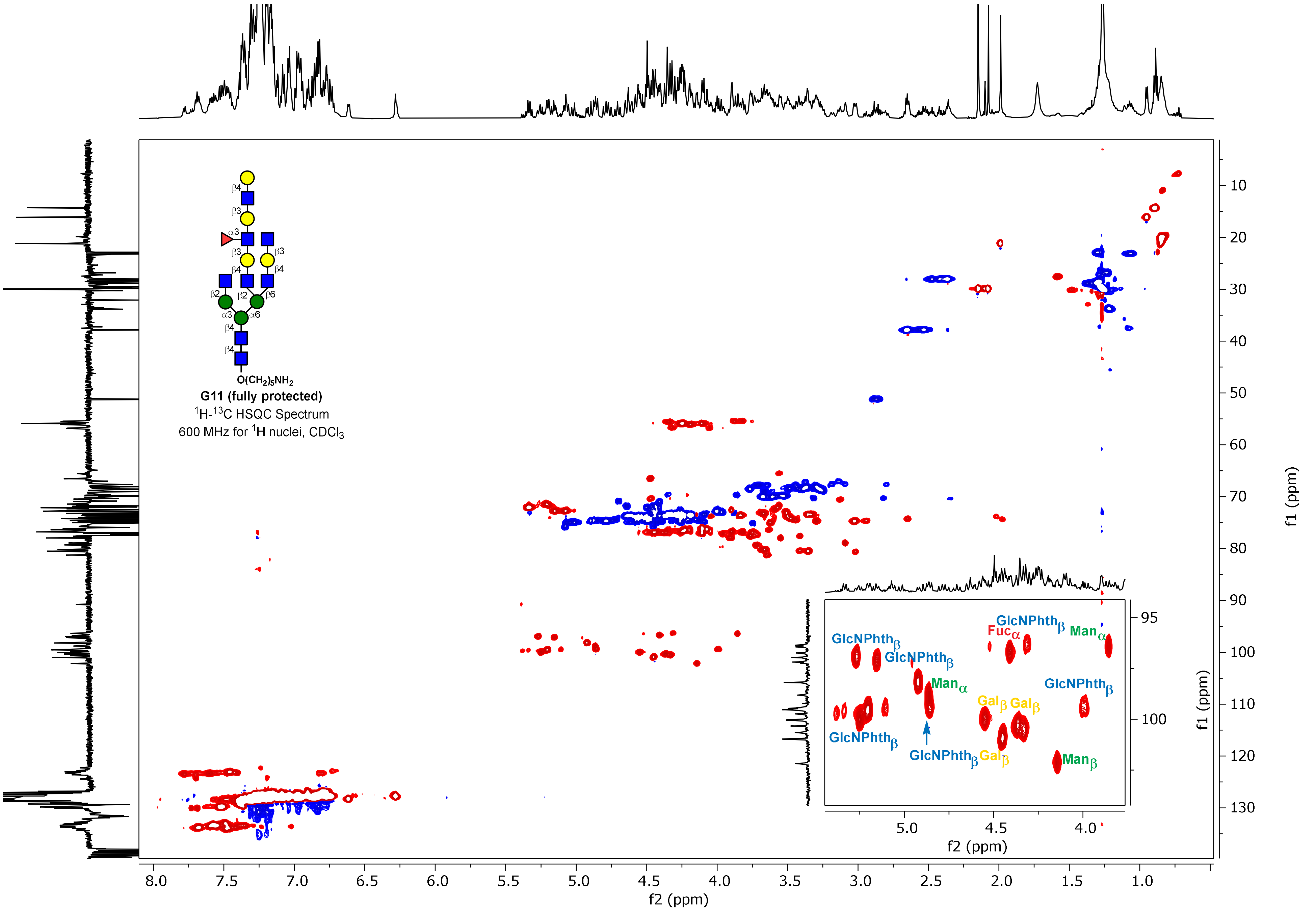
O(CH2)5NH2
G11 (fully protected)
1H-13C HSQC Spectrum
600 MHz for 1H nuclei, CDCl3
GlcNPhthβ
Fucα
Manα
Galβ
Manβ
f1 (ppm)
f2 (ppm)

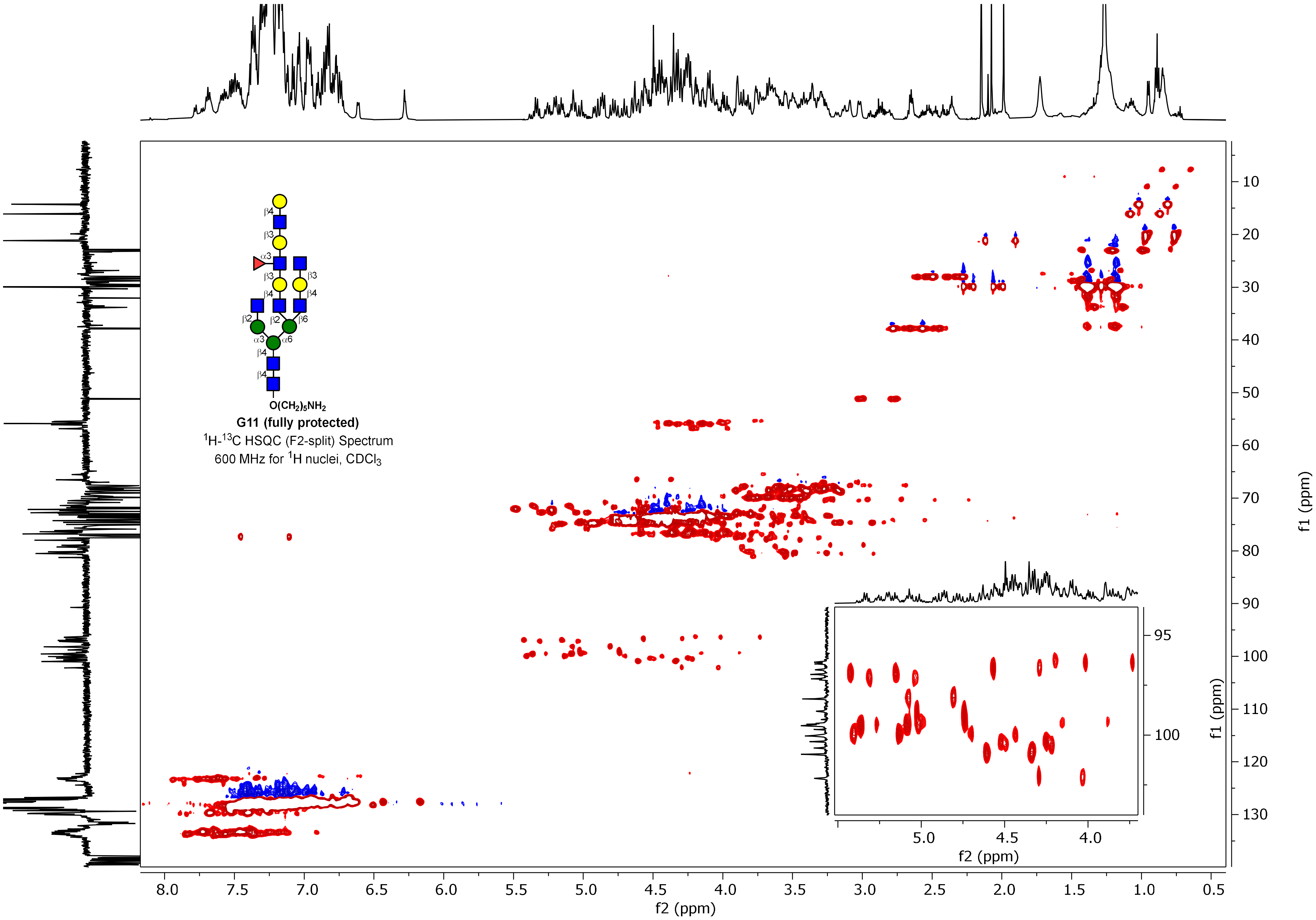

O(CH2)5NH2
G11 (fully protected)
1H-13C HSQC (F2-split) Spectrum
600 MHz for 1H nuclei, CDCl3
f2 (ppm)
f1 (ppm)

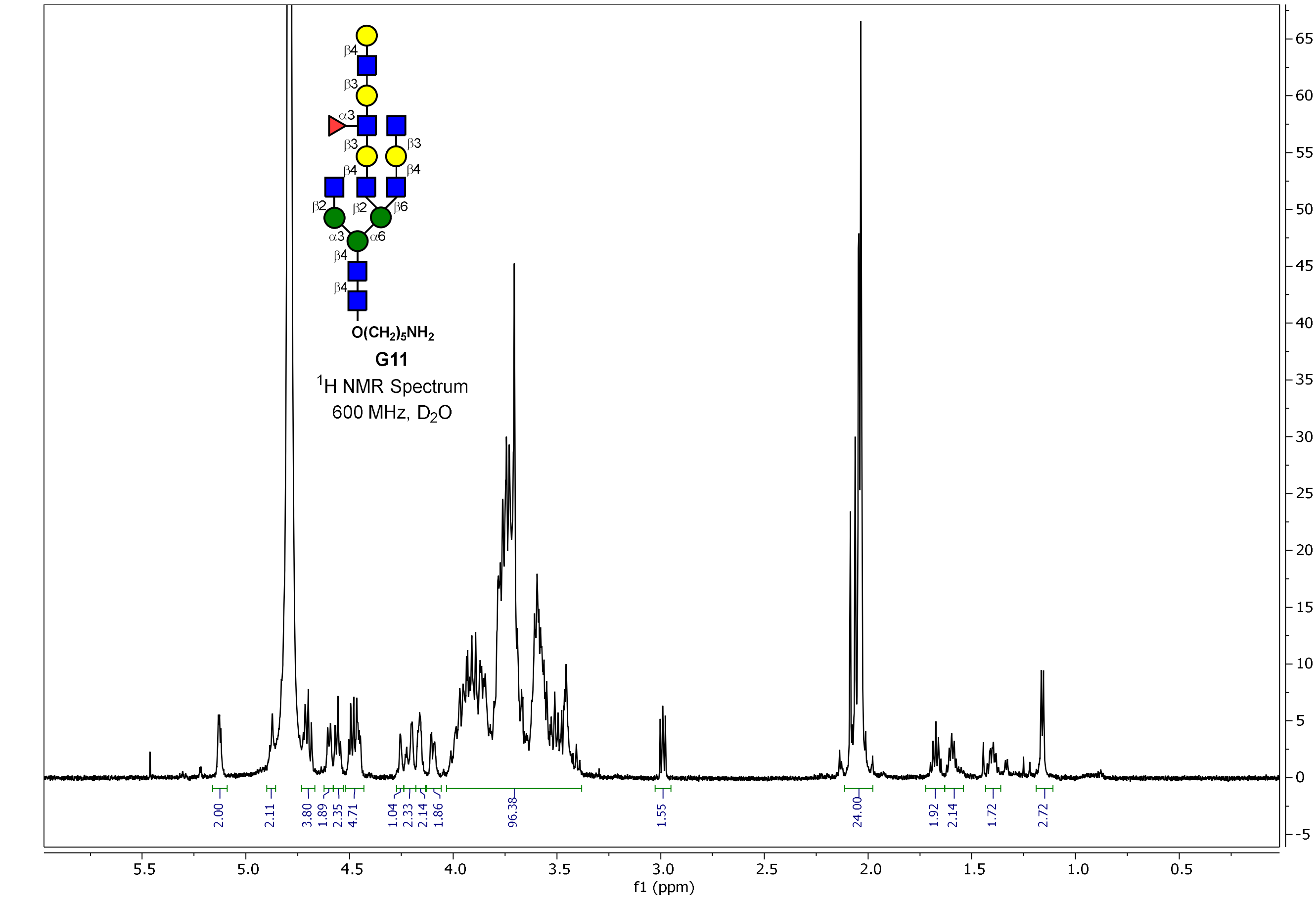
O(CH2)5NH2
G11
1H NMR Spectrum
600 MHz, D2O
f1 (ppm)

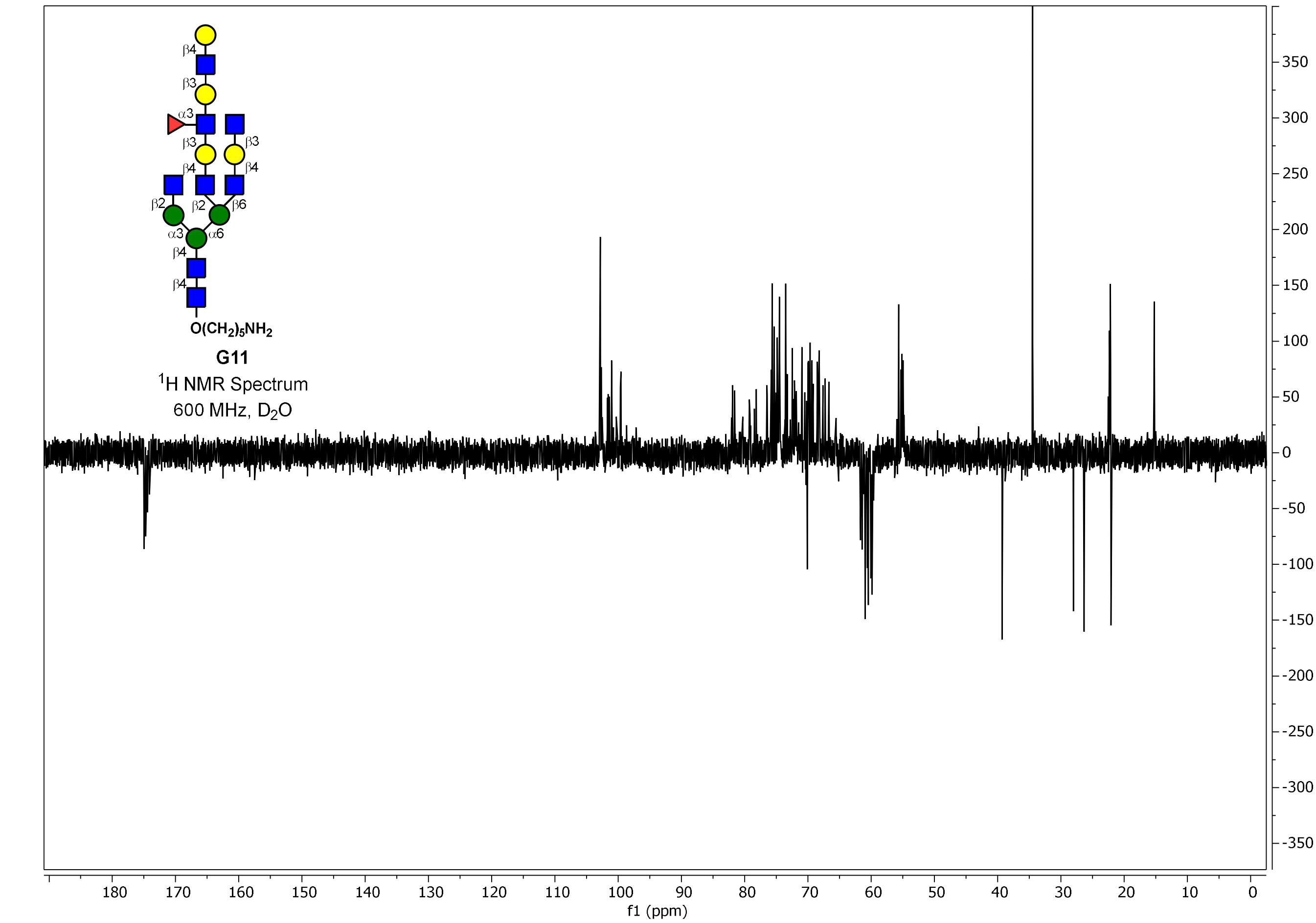
O(CH2)5NH2
G11
1H NMR Spectrum
600 MHz, D2O
f1 (ppm)

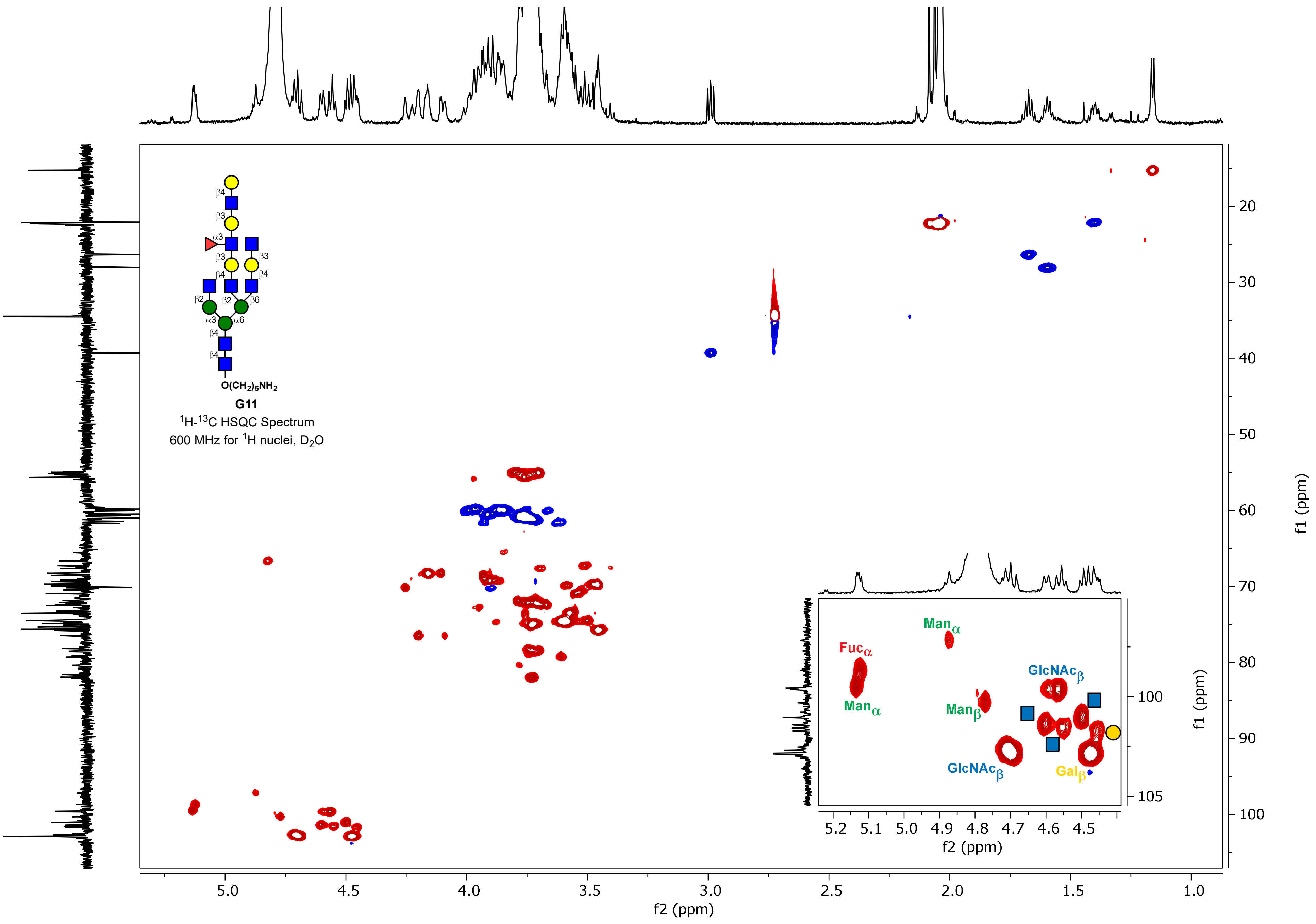

O(CH2)5NH2
G11
1H-13C HSQC Spectrum
600 MHz for 1H nuclei, D2O
Fucα
Manα
Manα
Manβ
GlcNAcβ
GlcNAcβ
Galβ
f2 (ppm)
f1 (ppm)

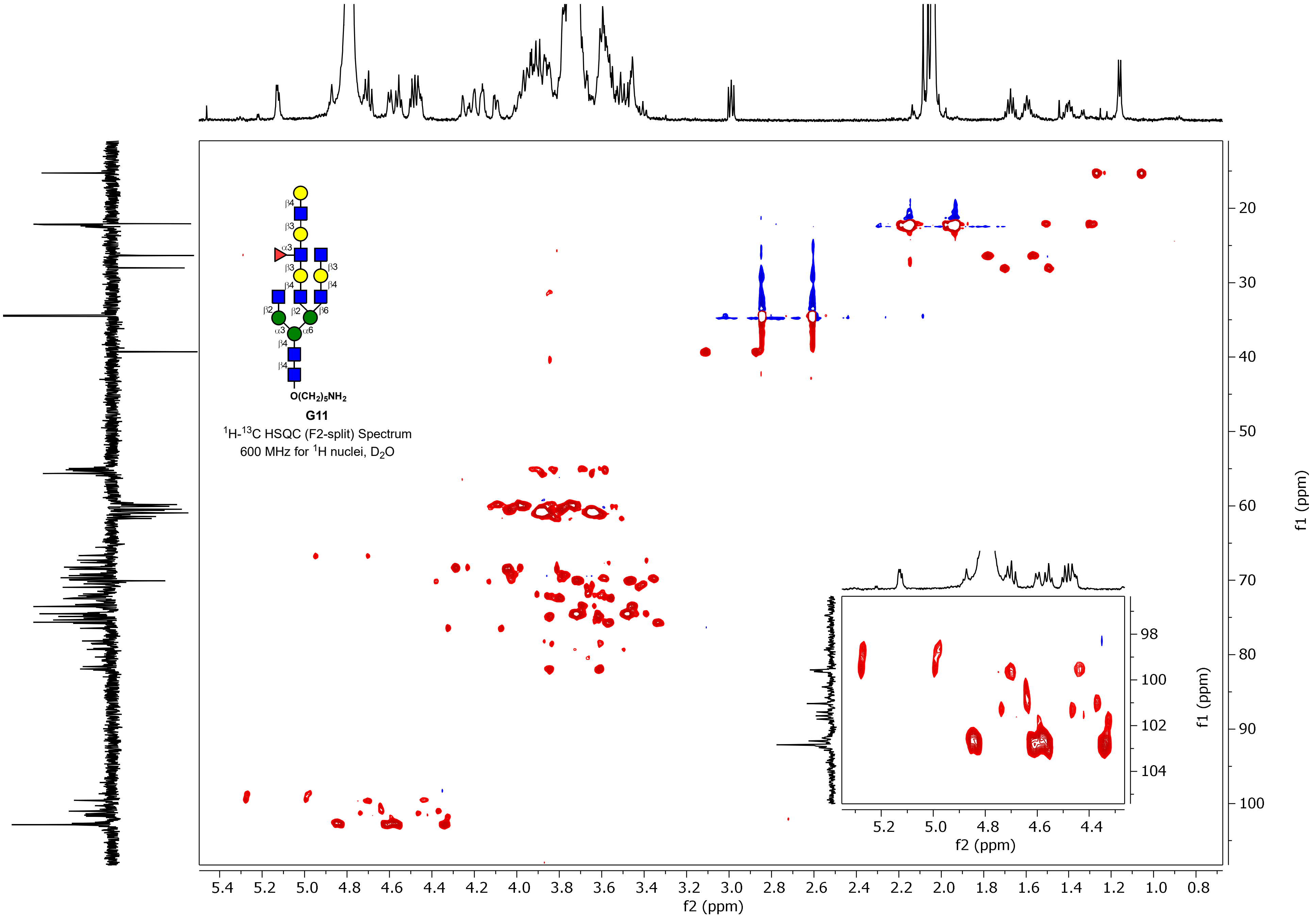

β4
β3
α3
β3
β3
β4
β4
β2
β2
β6
α3
α6
β4
β4
O(CH2)5NH2
G11
1H-13C HSQC (F2-split) Spectrum
600 MHz for 1H nuclei, D2O
f2 (ppm)
f1 (ppm)

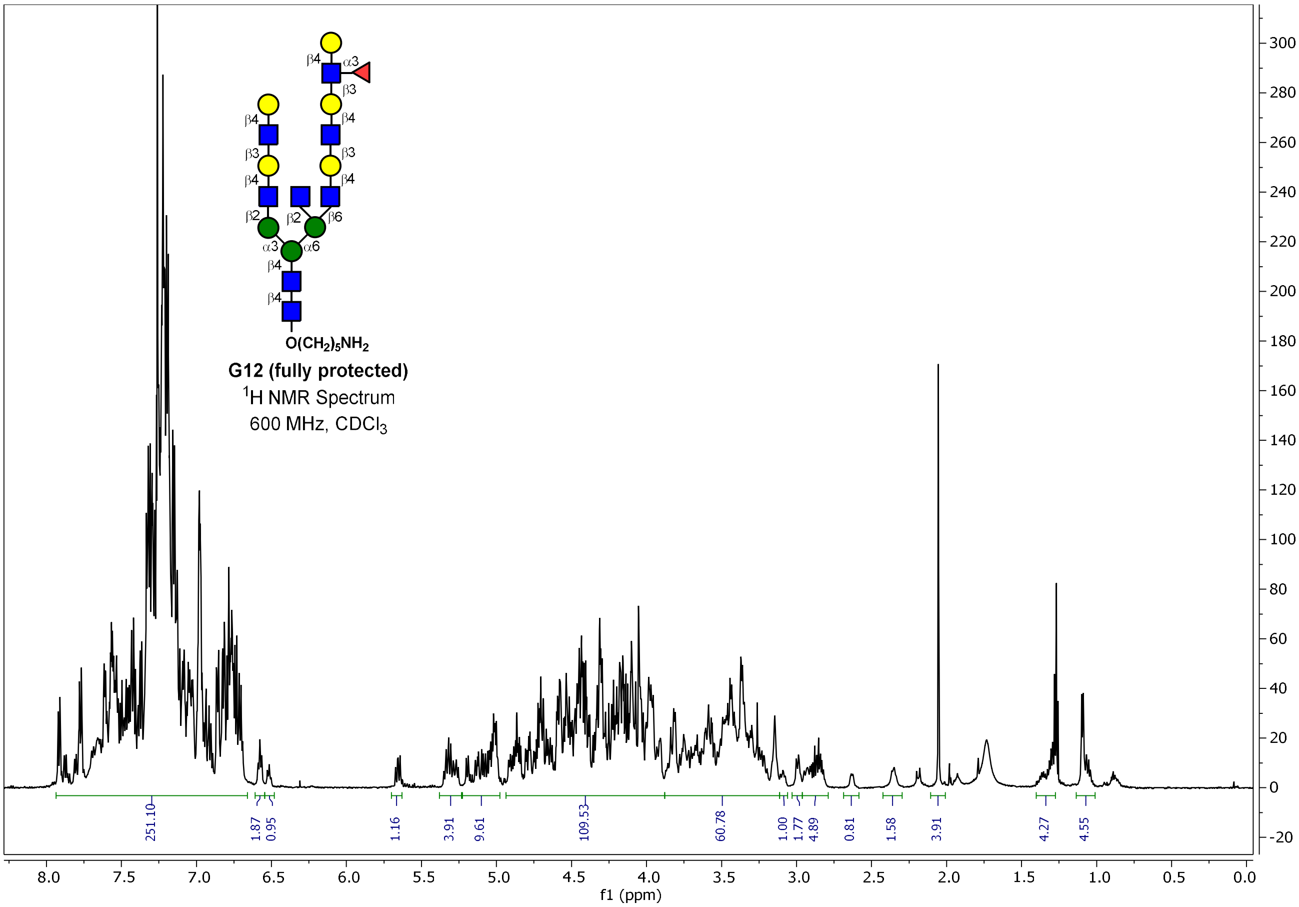
O(CH2)5NH2
G12 (fully protected)
1H NMR Spectrum
600 MHz, CDCl3
f1 (ppm)

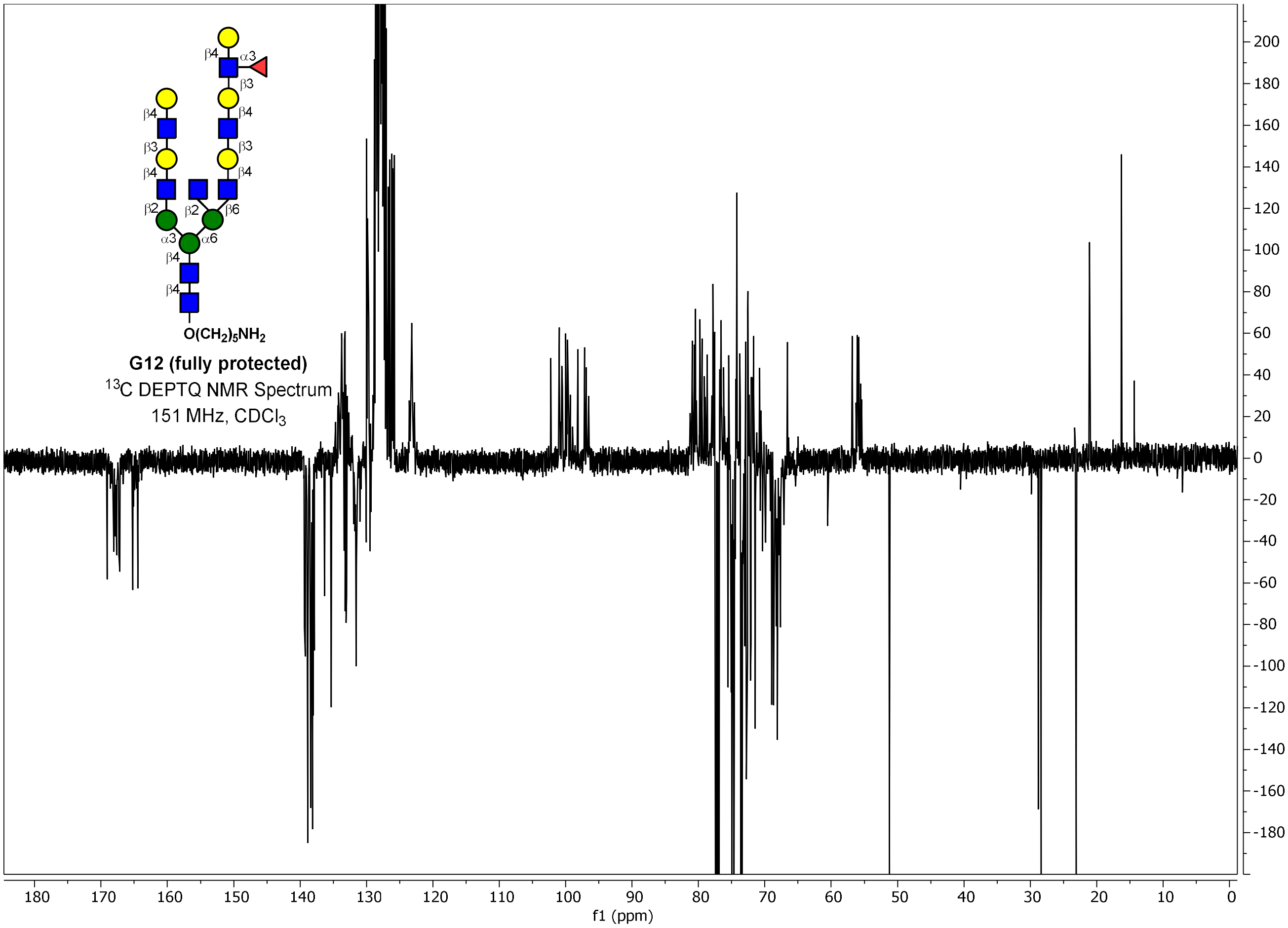
O(CH2)5NH2
G12 (fully protected)
13C DEPTQ NMR Spectrum
151 MHz, CDCl3
f1 (ppm)

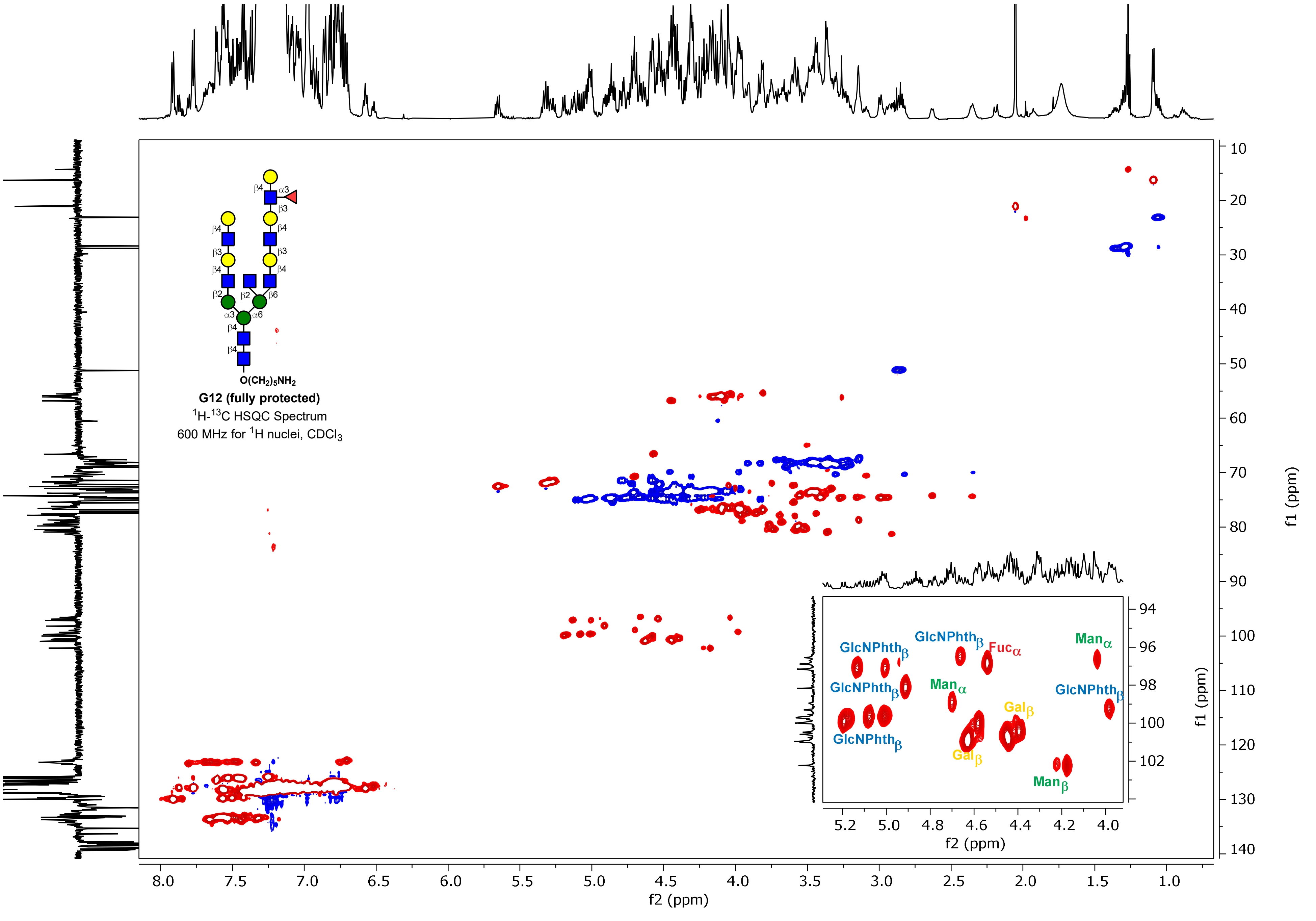

O(CH2)5NH2
G12 (fully protected)
1H-13C HSQC Spectrum
600 MHz for 1H nuclei, CDCl3
f2 (ppm)
f1 (ppm)
GlcNPhthβ
Fucα
Manα
Galβ
Manβ

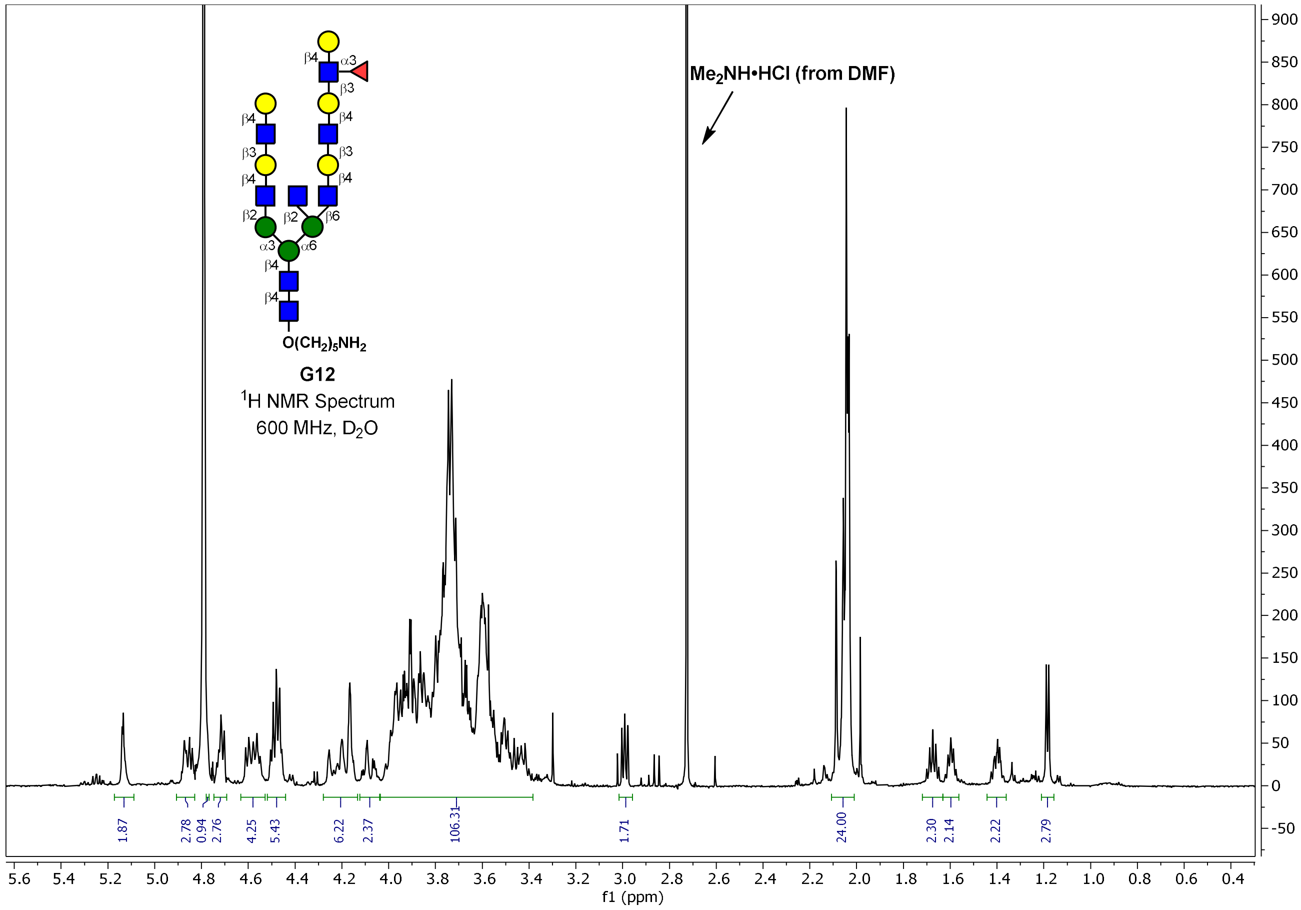
Me₂NH•HCl (from DMF)
O(CH₂)₅NH₂
G12
¹H NMR Spectrum
600 MHz, D₂O
f1 (ppm)

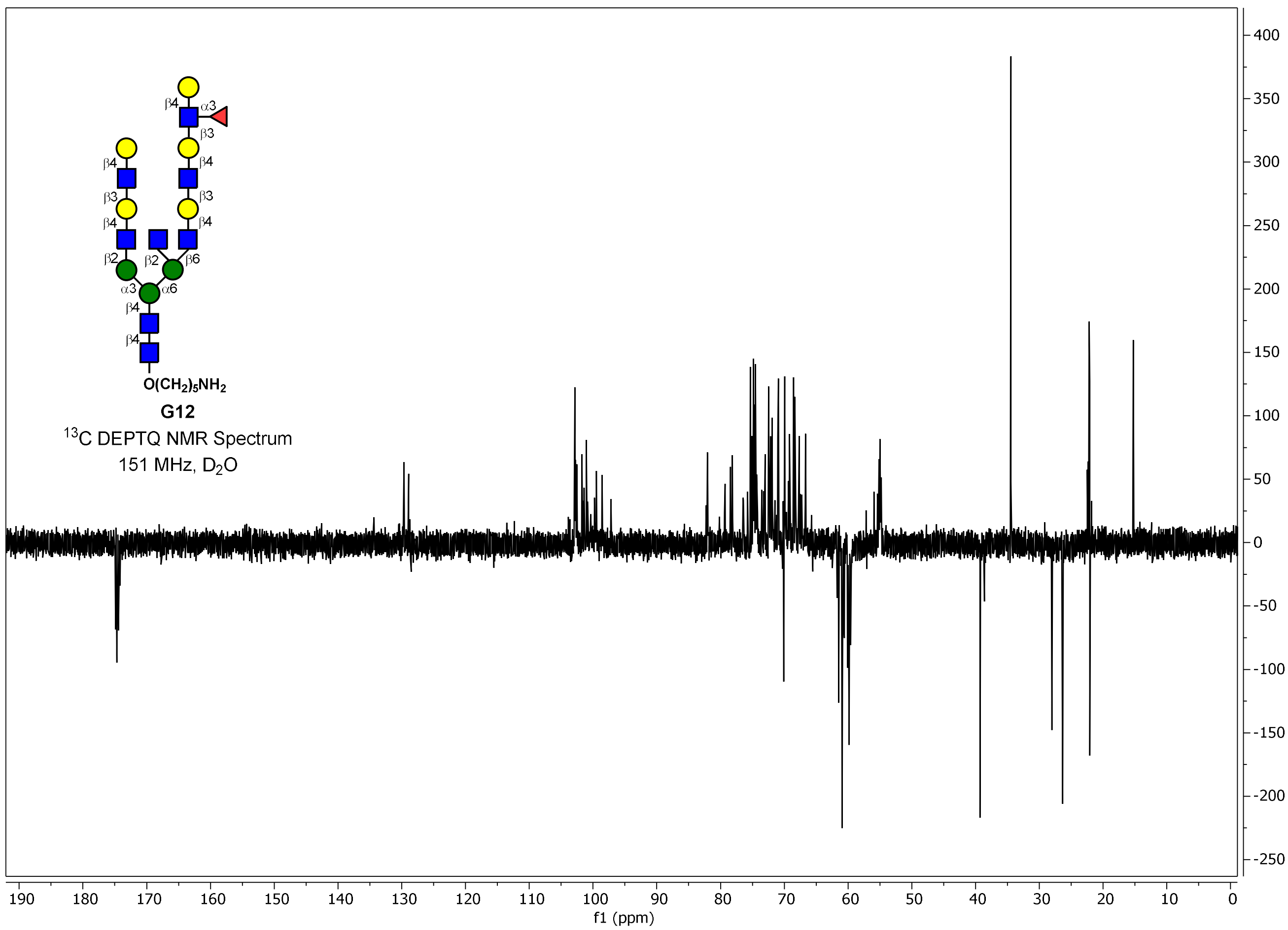
O(CH₂)₅NH₂
G12
¹³C DEPTQ NMR Spectrum
151 MHz, D₂O
f1 (ppm)

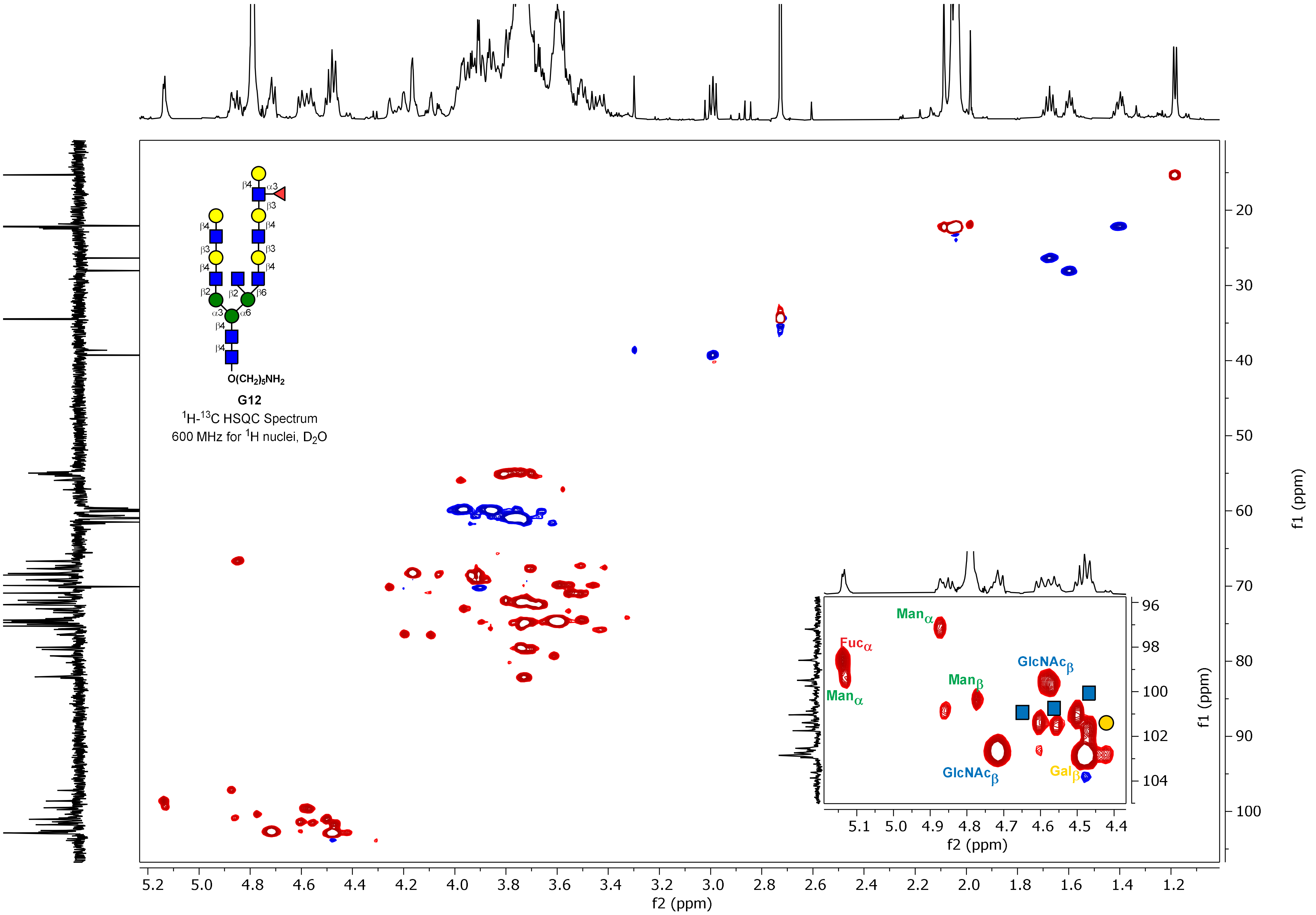

O(CH2)5NH2
G12
1H-13C HSQC Spectrum
600 MHz for 1H nuclei, D2O
Manα
Fucα
Manα
Manβ
GlcNAcβ
GlcNAcβ
Galβ
f2 (ppm)
f1 (ppm)

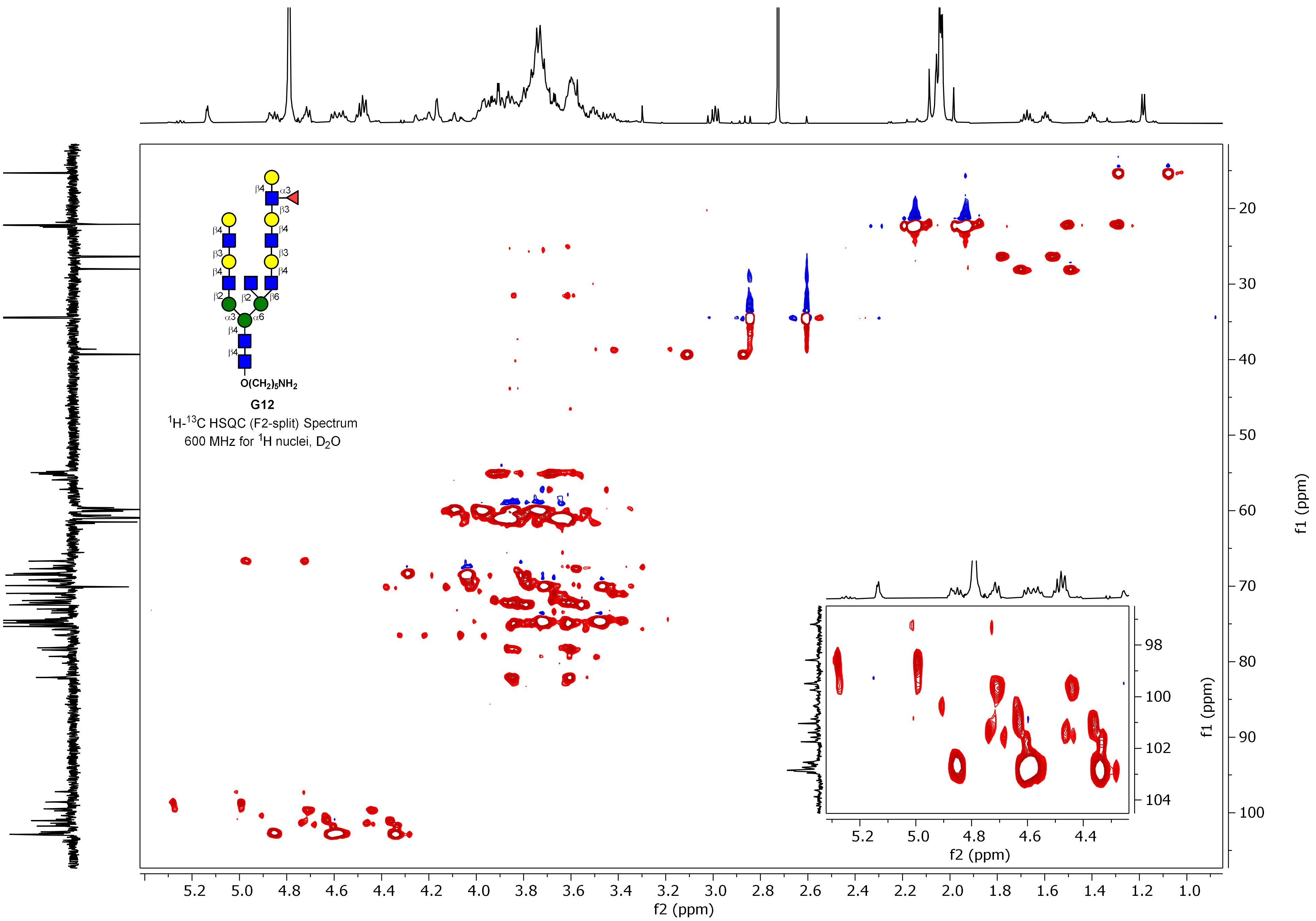

G12
^{1}H-^{13}C HSQC (F2-split) Spectrum
600 MHz for ^{1}H nuclei, D_2O
O(CH2)5NH2
f2 (ppm)
f1 (ppm)

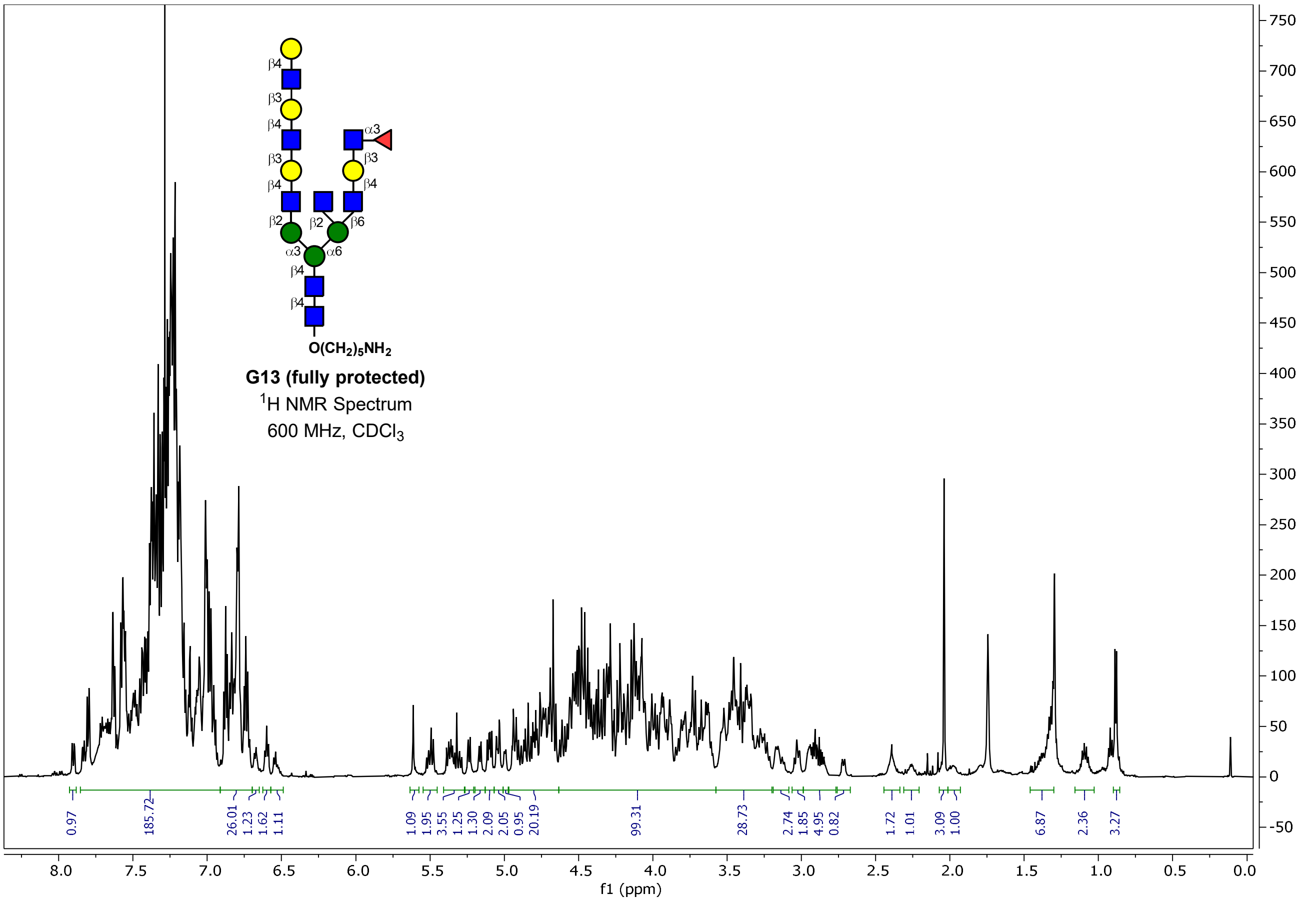

β4
β3
β4
β3
β4
β2
α3
β4
β4
β2
β6
α6
α3
β3
β4
O(CH2)5NH2
G13 (fully protected)
1H NMR Spectrum
600 MHz, CDCl3
f1 (ppm)

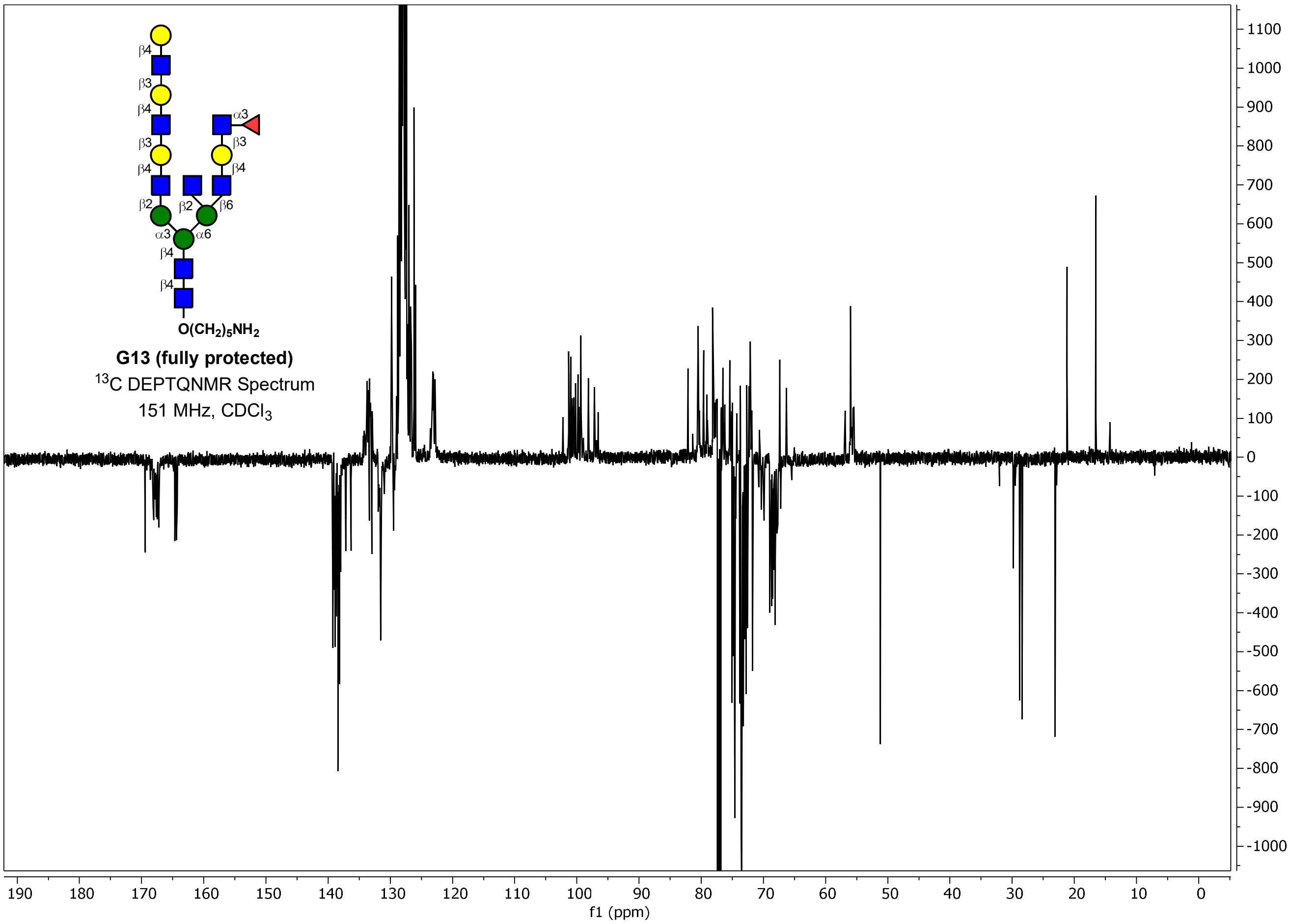

O(CH2)5NH2
G13 (fully protected)
13C DEPTQNMR Spectrum
151 MHz, CDCl3
f1 (ppm)

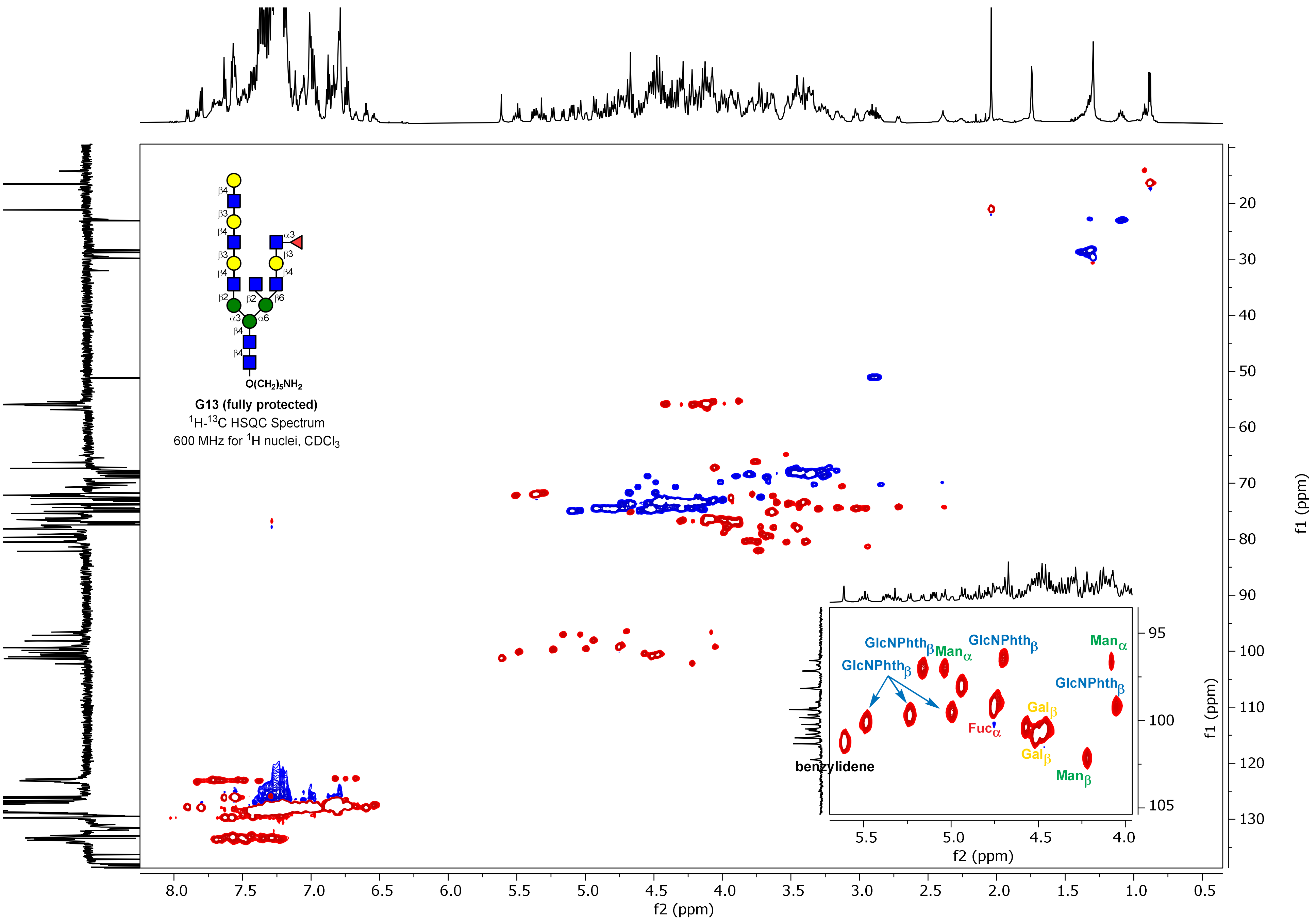

O(CH2)5NH2
G13 (fully protected)
1H-13C HSQC Spectrum
600 MHz for 1H nuclei, CDCl3
GlcNPhthβ
Manα
GlcNPhthβ
Manα
GlcNPhthβ
GlcNPhthβ
Galβ
Fucα
Galβ
Manβ
benzylidene
f2 (ppm)
f1 (ppm)

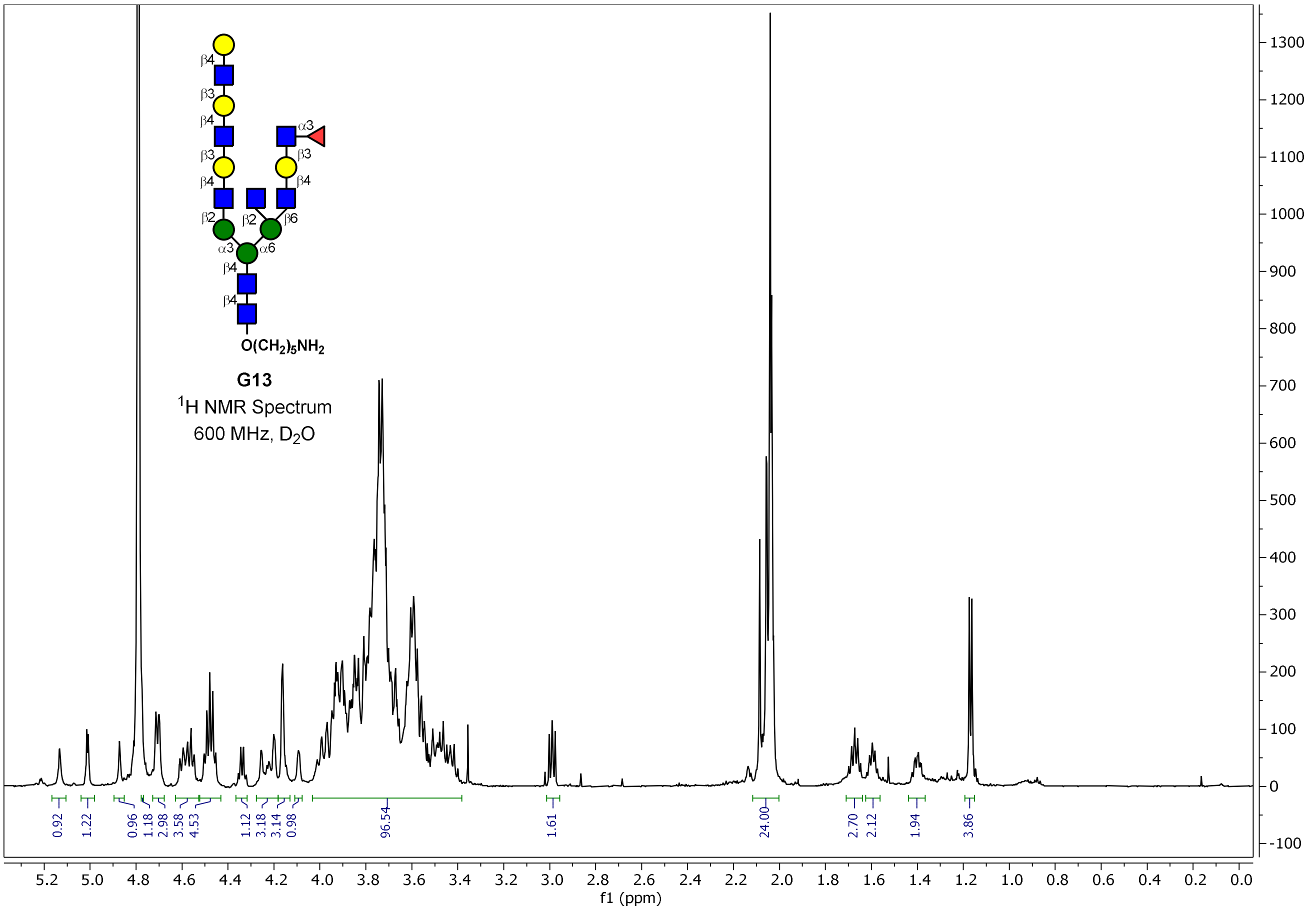
O(CH2)5NH2
G13
1H NMR Spectrum
600 MHz, D2O
f1 (ppm)

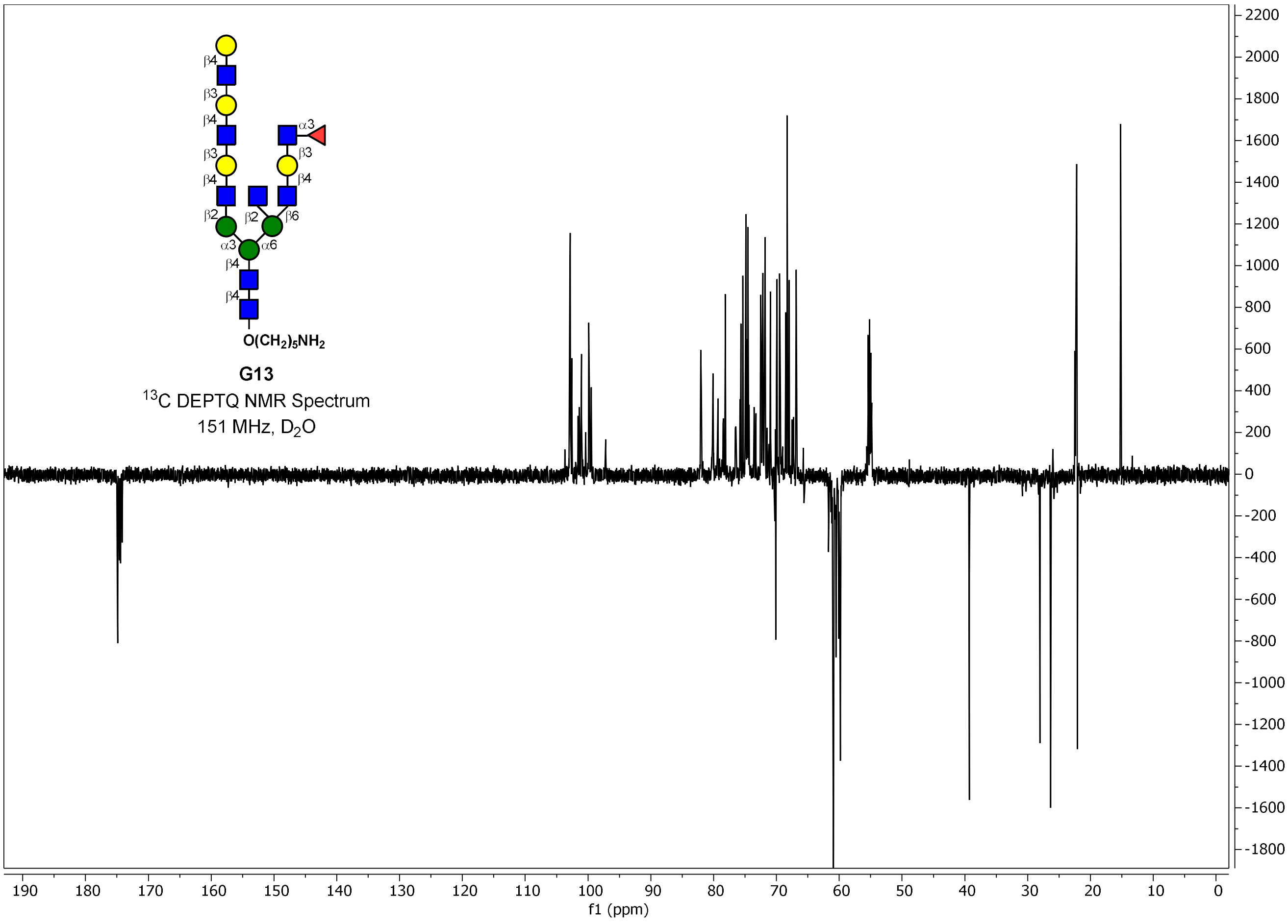
O(CH2)5NH2
G13
13C DEPTQ NMR Spectrum
151 MHz, D2O
f1 (ppm)

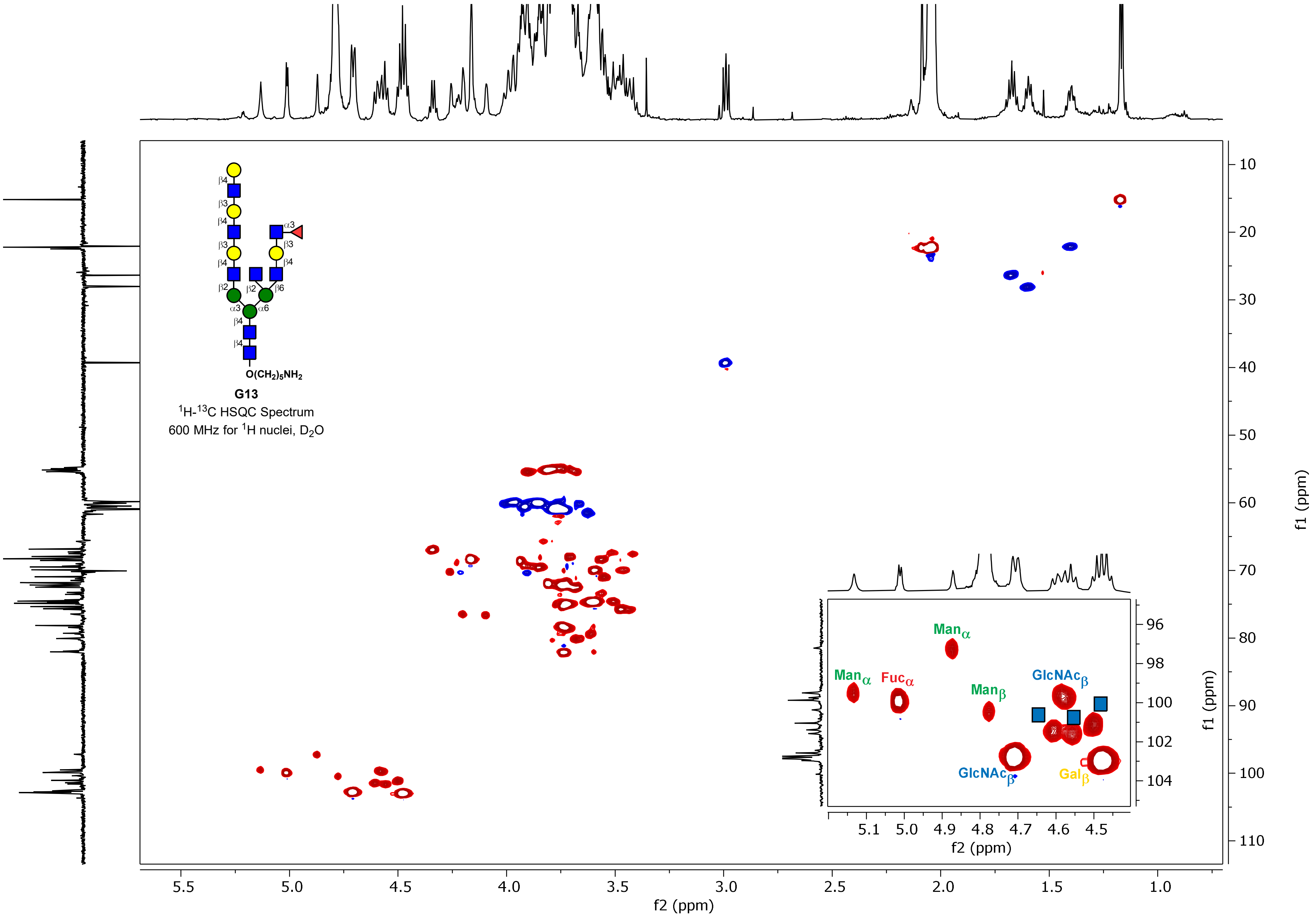

G13
^{1}H-^{13}C HSQC Spectrum
600 MHz for ^{1}H nuclei, D_2O
O(CH2)5NH2
Manα
Fucα
Manβ
GlcNAcβ
Galβ
f2 (ppm)
f1 (ppm)

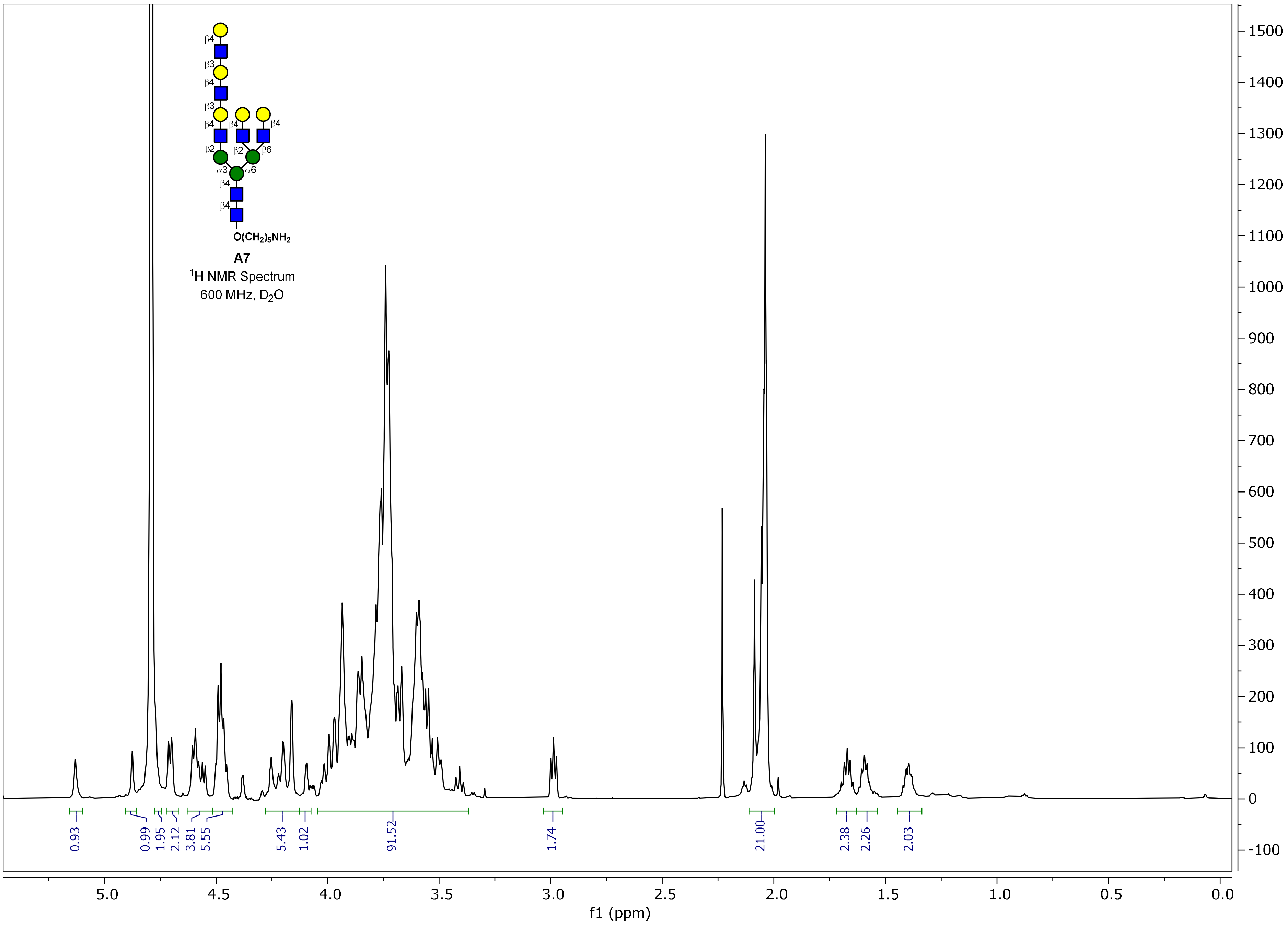

O(CH2)5NH2
A7
1H NMR Spectrum
600 MHz, D2O
f1 (ppm)

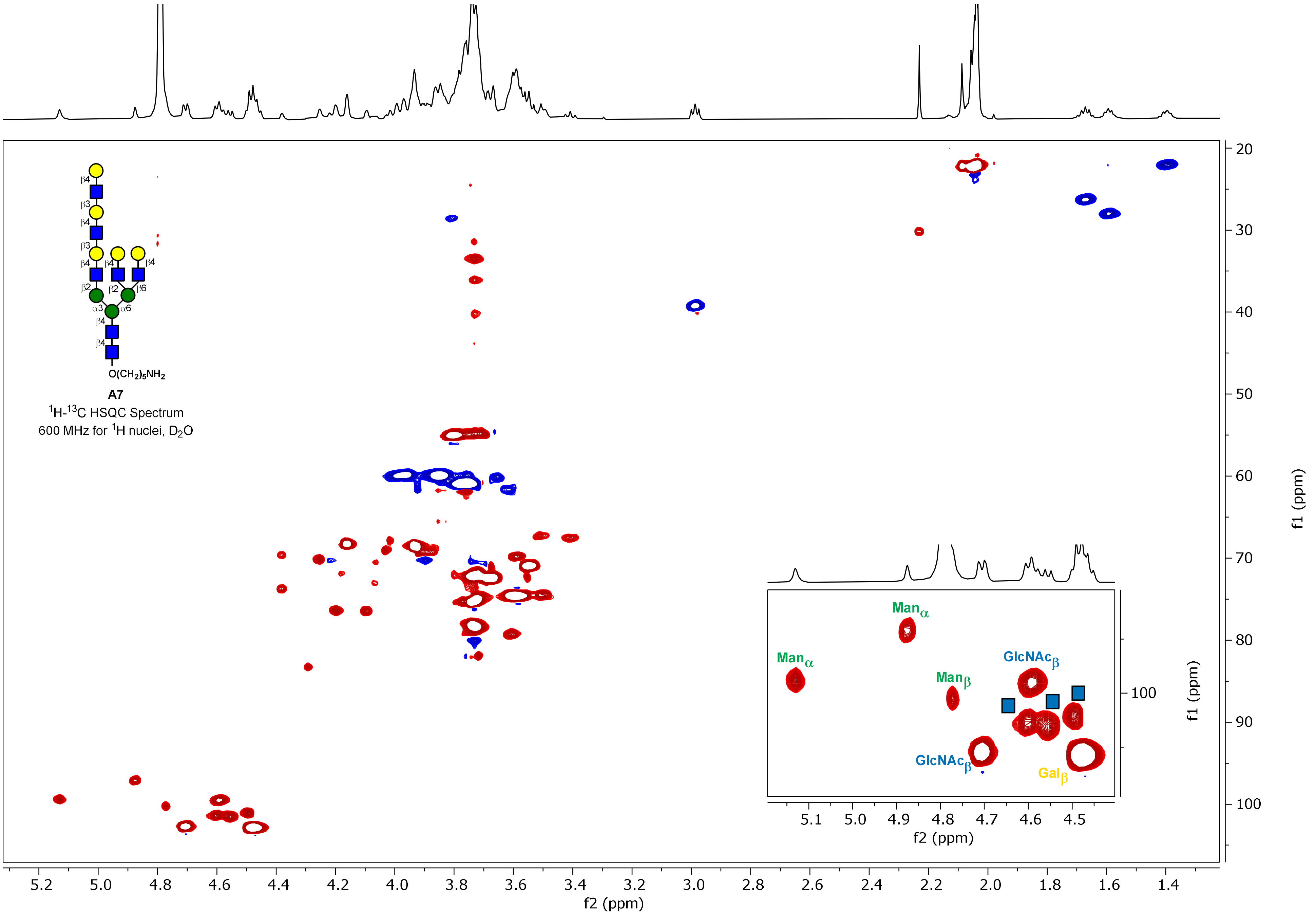
O(CH2)5NH2
A7
1H-13C HSQC Spectrum
600 MHz for 1H nuclei, D2O
Manα
Manα
Manβ
GlcNAcβ
GlcNAcβ
Galβ
f2 (ppm)
f1 (ppm)
f2 (ppm)
f1 (ppm)

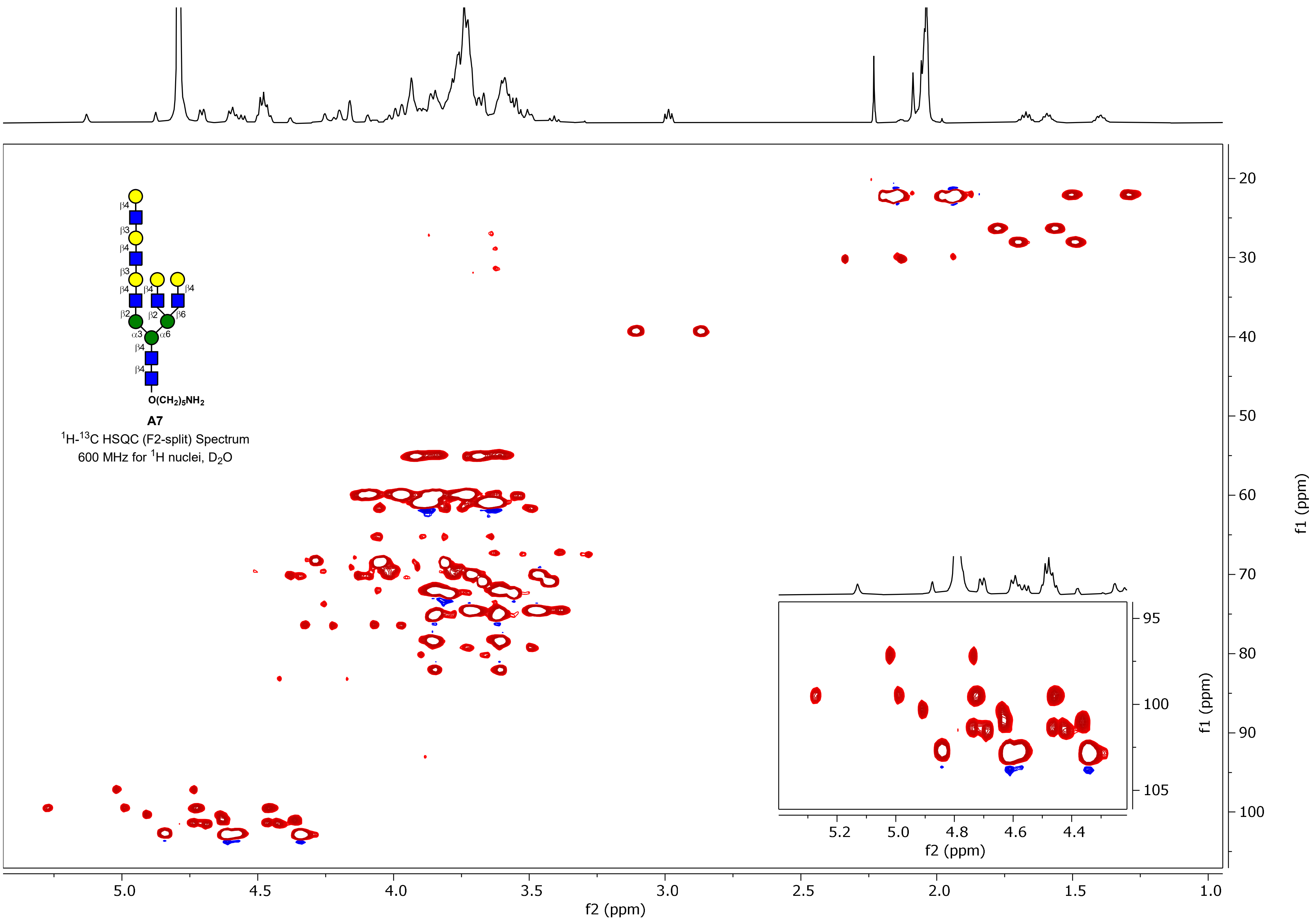

O(CH2)5NH2
A7
1H-13C HSQC (F2-split) Spectrum
600 MHz for 1H nuclei, D2O
f2 (ppm)
f1 (ppm)

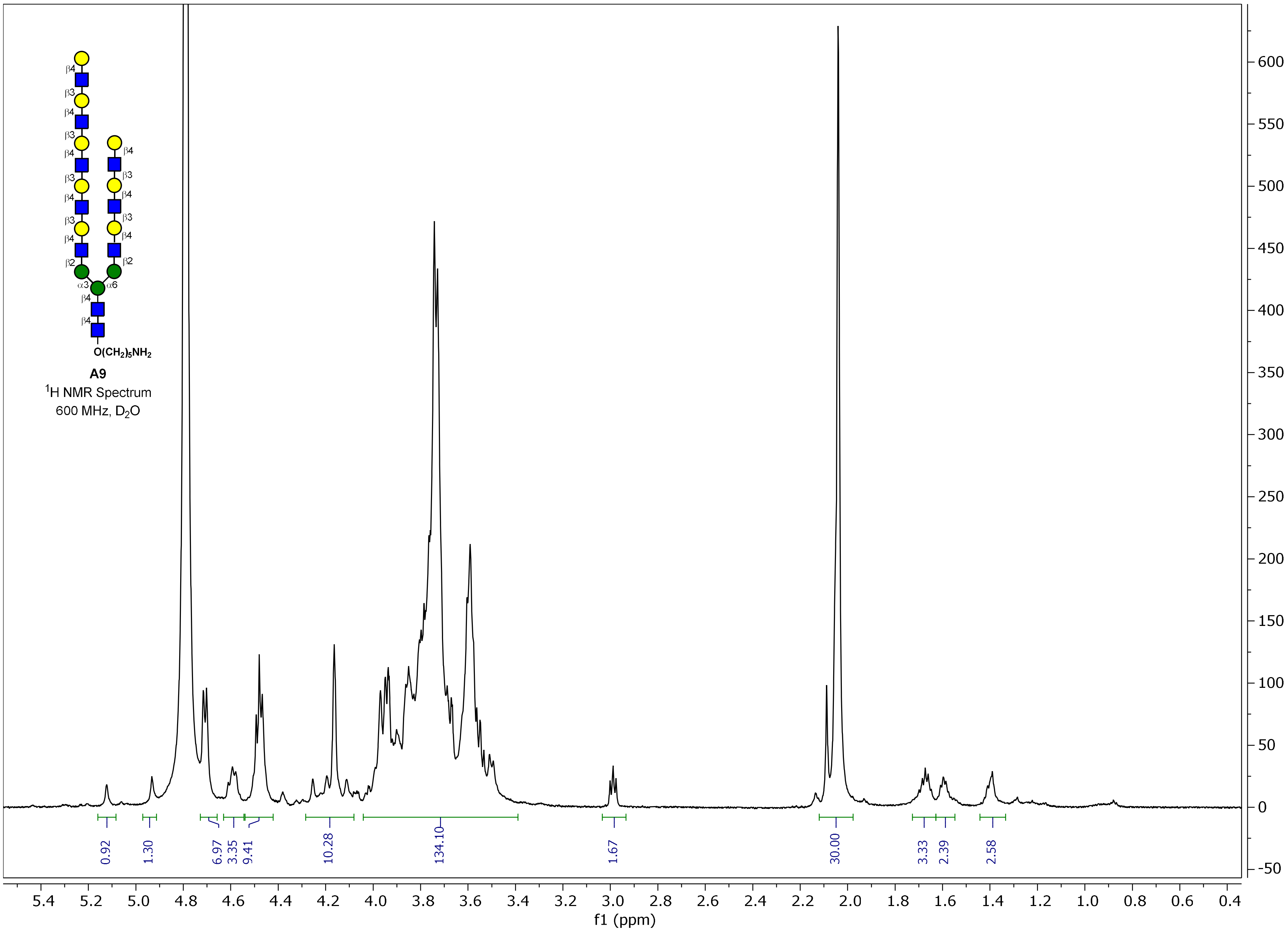

O(CH2)5NH2
A9
1H NMR Spectrum
600 MHz, D2O
f1 (ppm)
0.92
1.30
6.97
3.35
9.41
10.28
134.10
1.67
30.00
3.33
2.39
2.58

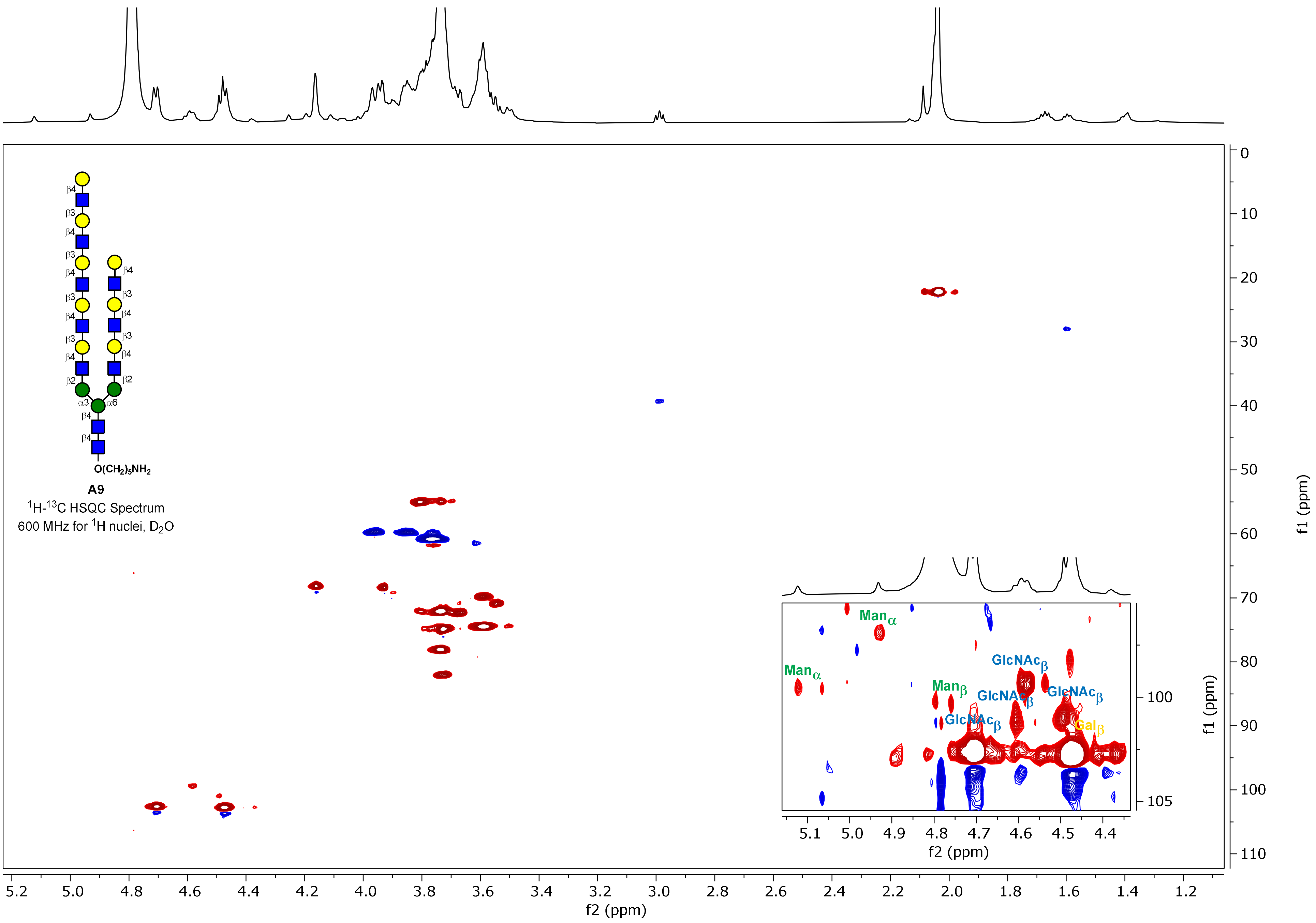
O(CH2)5NH2
A9
1H-13C HSQC Spectrum
600 MHz for 1H nuclei, D2O
β4
β3
β2
α3
α6
Manα
Manβ
GlcNAcβ
Galβ
f2 (ppm)
f1 (ppm)

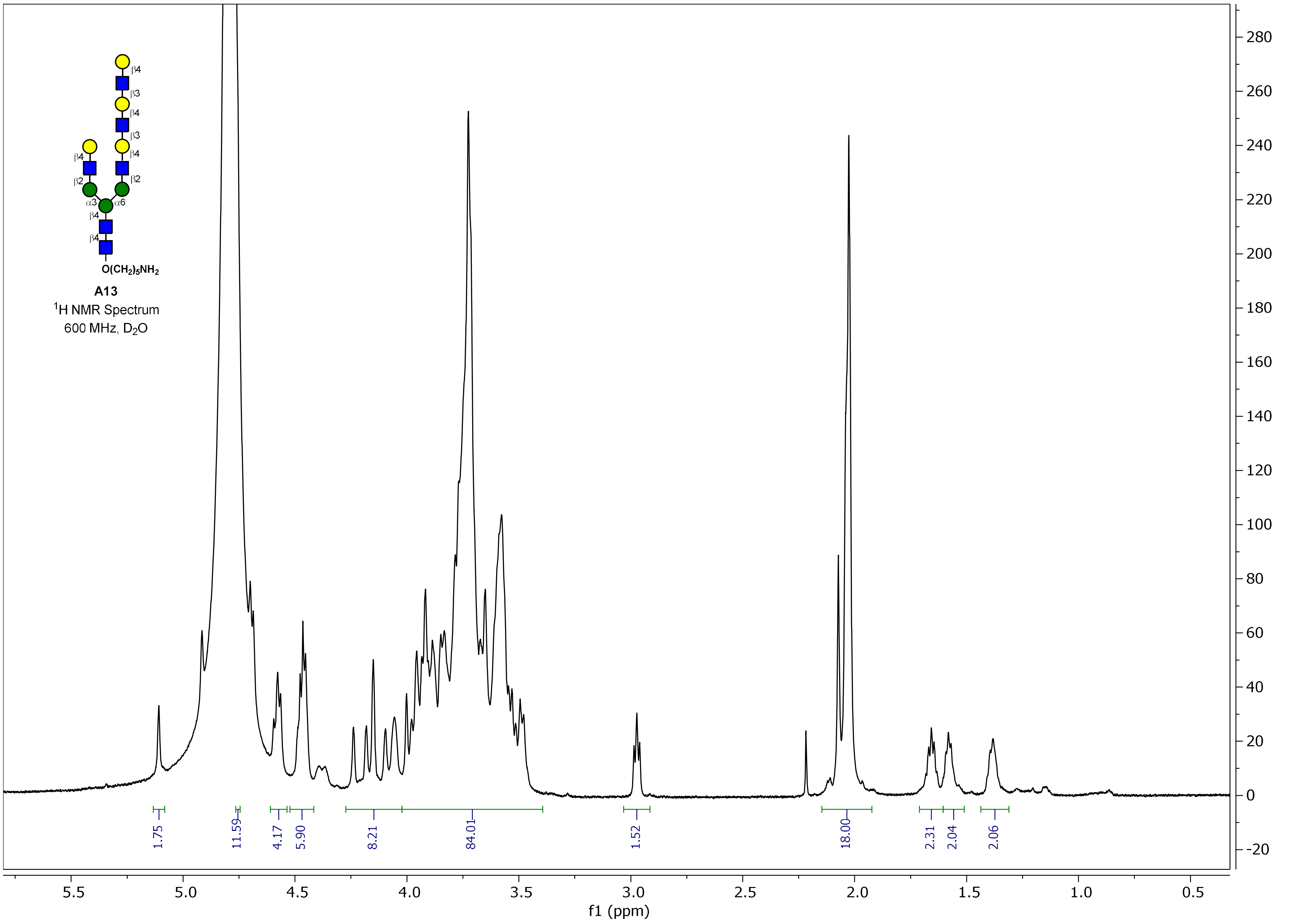
O(CH2)5NH2
A13
1H NMR Spectrum
600 MHz, D2O
β4
β3
β2
α3
α6
1.75
11.59
4.17
5.90
8.21
84.01
1.52
18.00
2.31
2.04
2.06
5.5
5.0
4.5
4.0
3.5
3.0
2.5
2.0
1.5
1.0
0.5
f1 (ppm)
280
260
240
220
200
180
160
140
120
100
80
60
40
20
0
-20

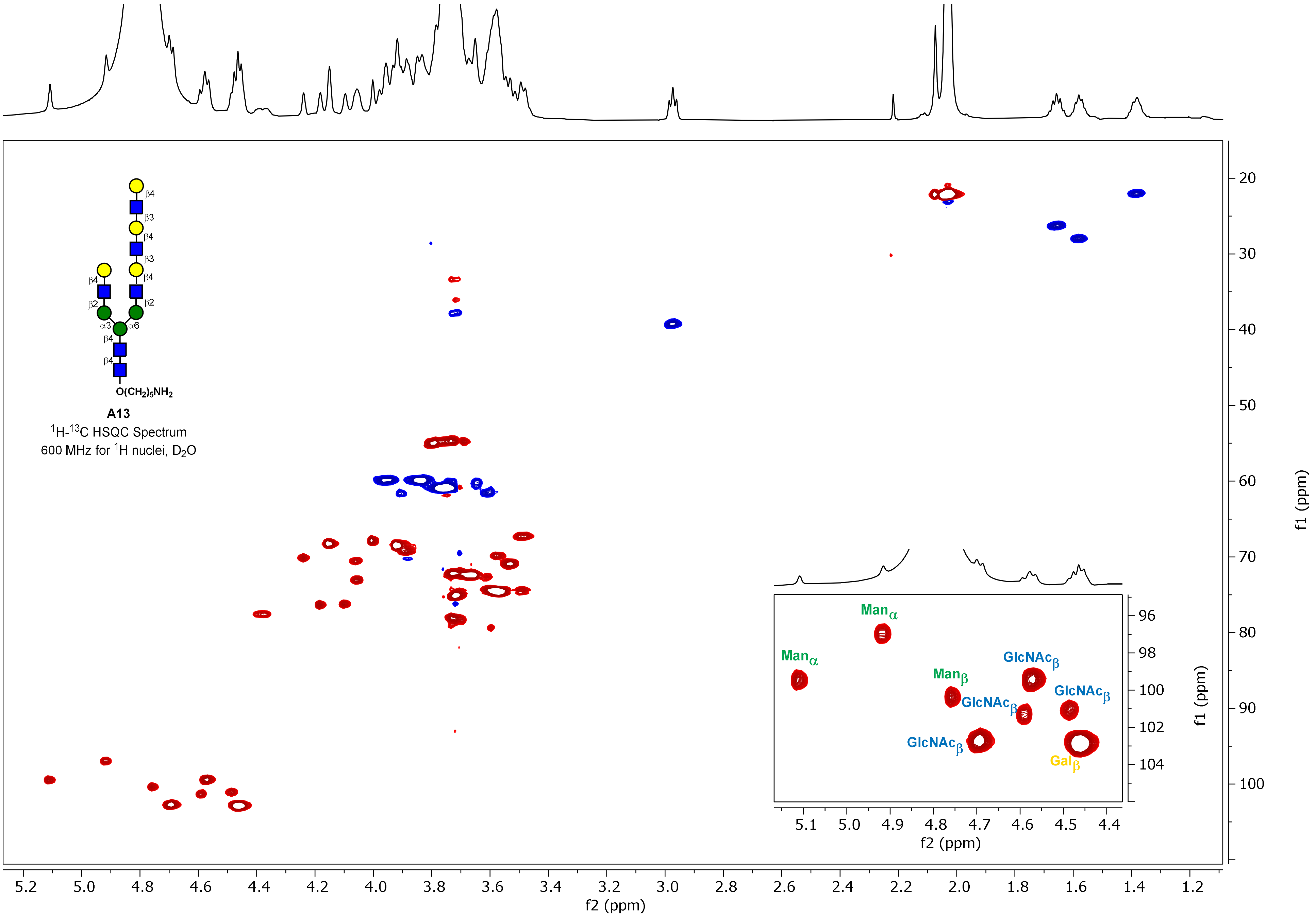

β4
β3
β4
β3
β4
β2
β4
β2
α3
α6
β4
β4
O(CH2)5NH2
A13
1H-13C HSQC Spectrum
600 MHz for 1H nuclei, D2O
Manα
Manα
Manβ
GlcNAcβ
GlcNAcβ
GlcNAcβ
GlcNAcβ
Galβ
f2 (ppm)
f1 (ppm)

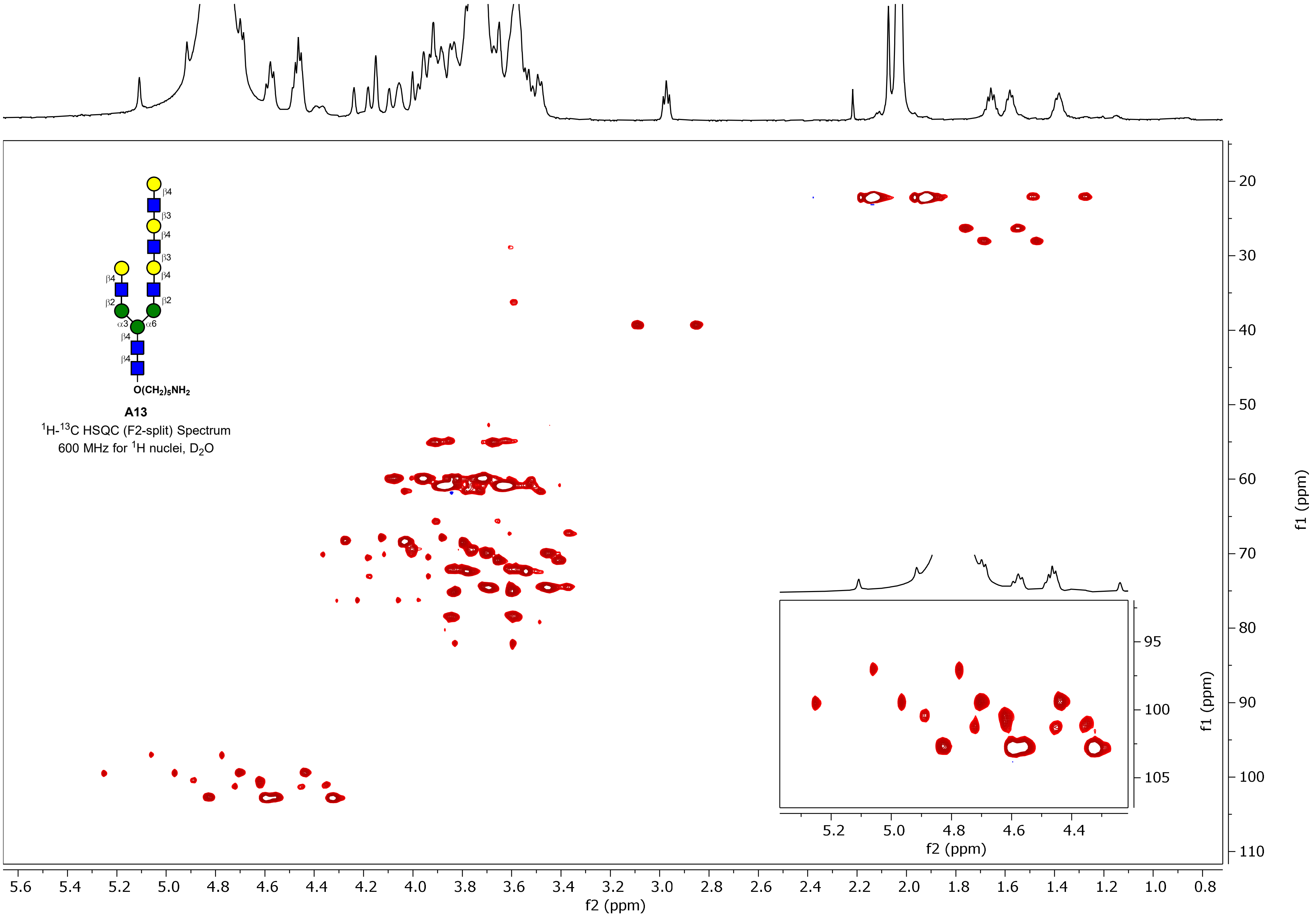
β4
β3
β4
β3
β4
β4
β2
β2
α3
α6
β4
β4
O(CH2)5NH2
A13
1H-13C HSQC (F2-split) Spectrum
600 MHz for 1H nuclei, D2O
f2 (ppm)
f1 (ppm)

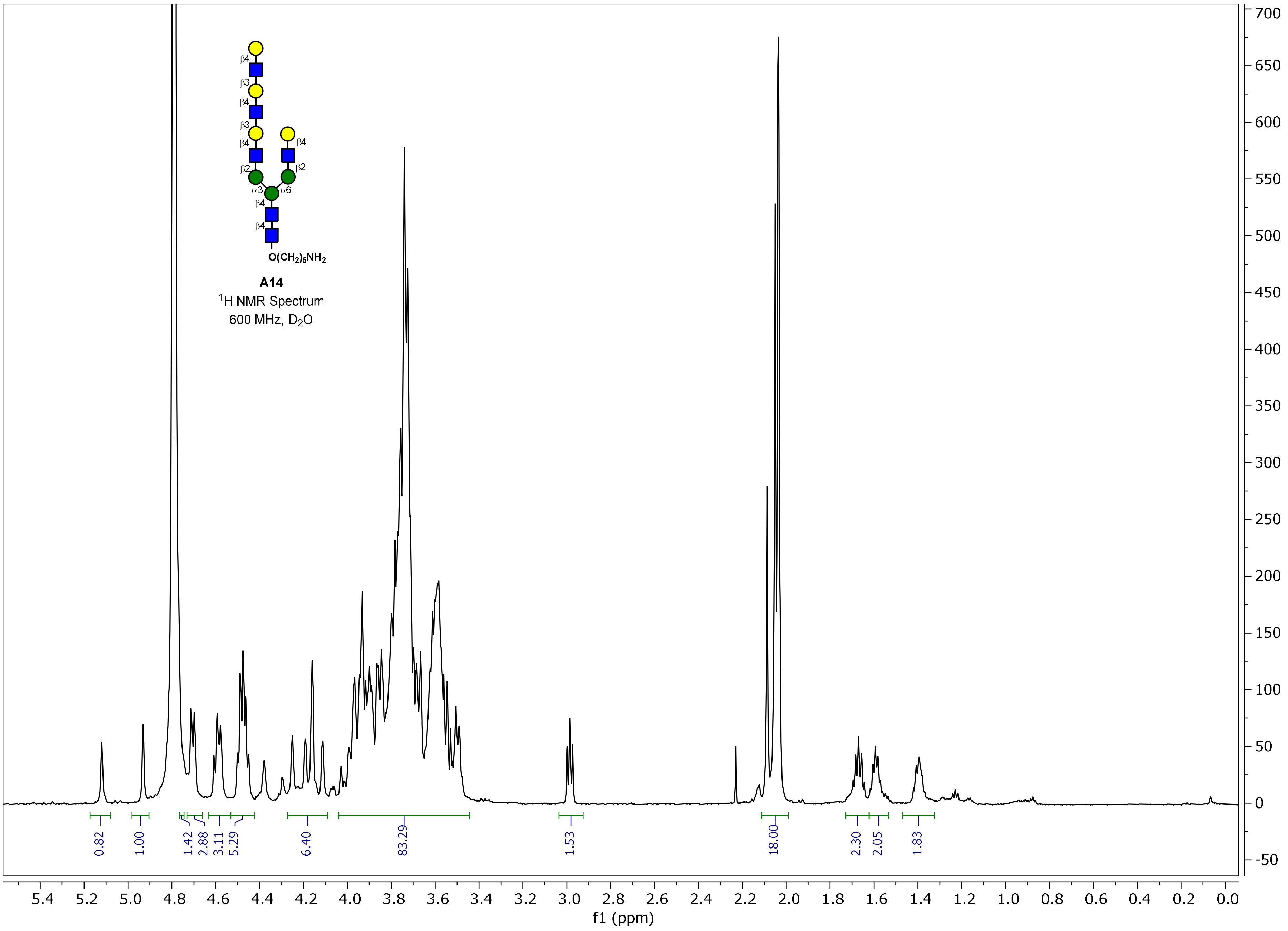

O(CH2)5NH2
A14
1H NMR Spectrum
600 MHz, D2O
β4
β3
β2
α3
α6
0.82
1.00
1.42
2.88
3.11
5.29
6.40
83.29
1.53
18.00
2.30
2.05
1.83
f1 (ppm)

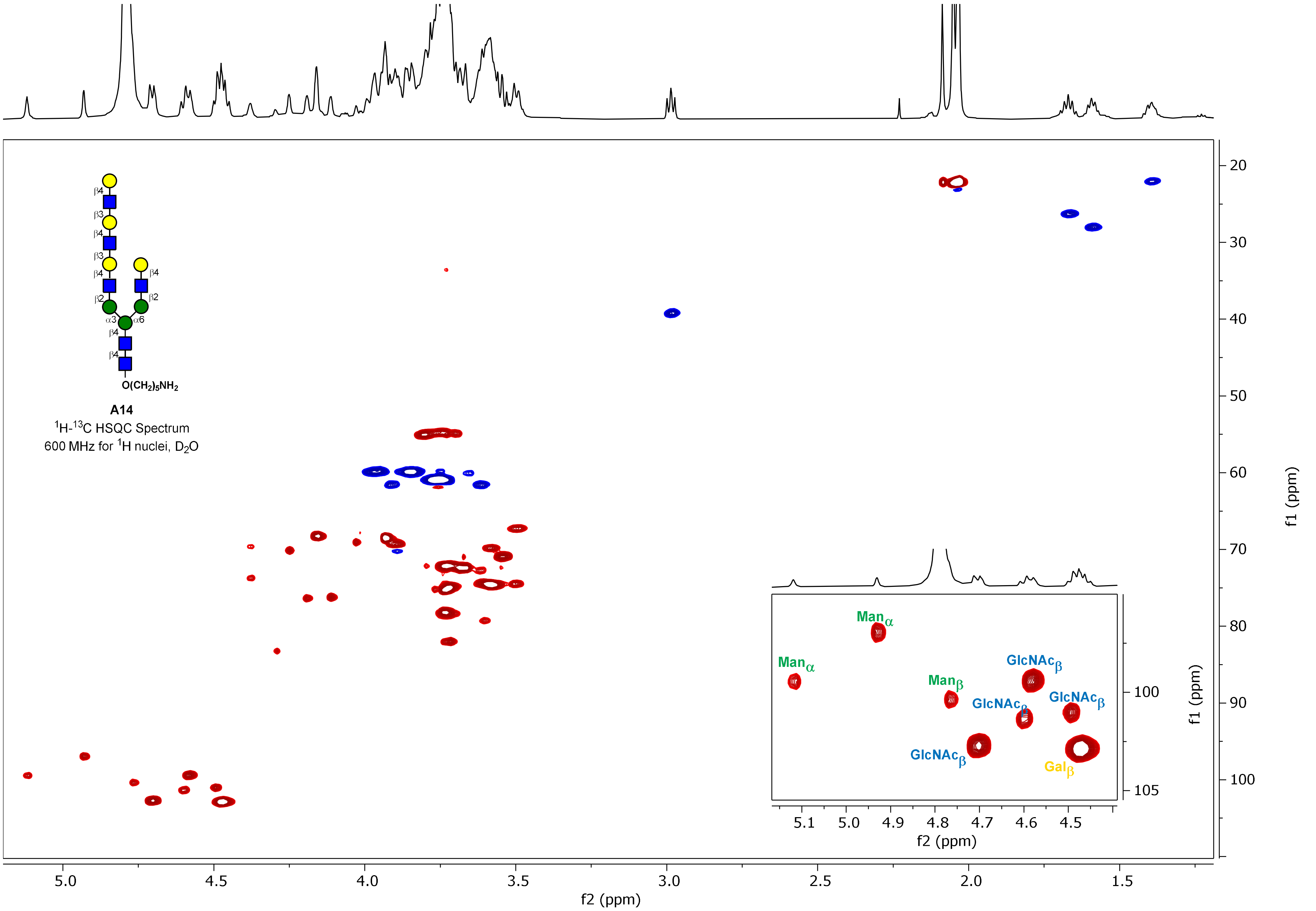

O(CH2)5NH2
A14
1H-13C HSQC Spectrum
600 MHz for 1H nuclei, D2O
Manα
Manα
Manβ
GlcNAcβ
GlcNAcβ
GlcNAcβ
GlcNAcβ
Galβ
f2 (ppm)
f1 (ppm)
f2 (ppm)
f1 (ppm)

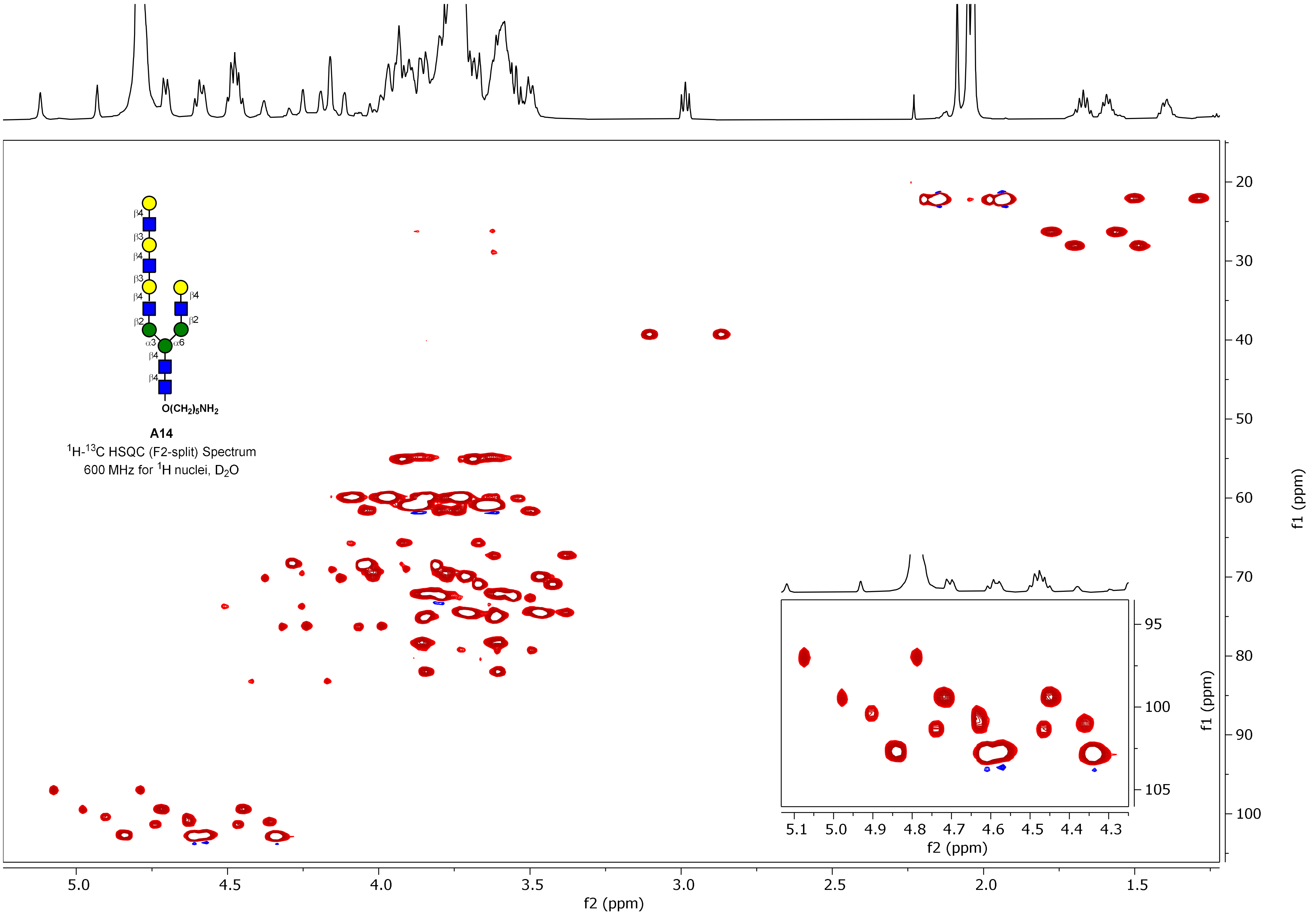
β4
β3
β4
β3
β4
β4
β2
β2
α3
α6
β4
β4
O(CH2)5NH2
A14
1H-13C HSQC (F2-split) Spectrum
600 MHz for 1H nuclei, D2O
f2 (ppm)
f1 (ppm)

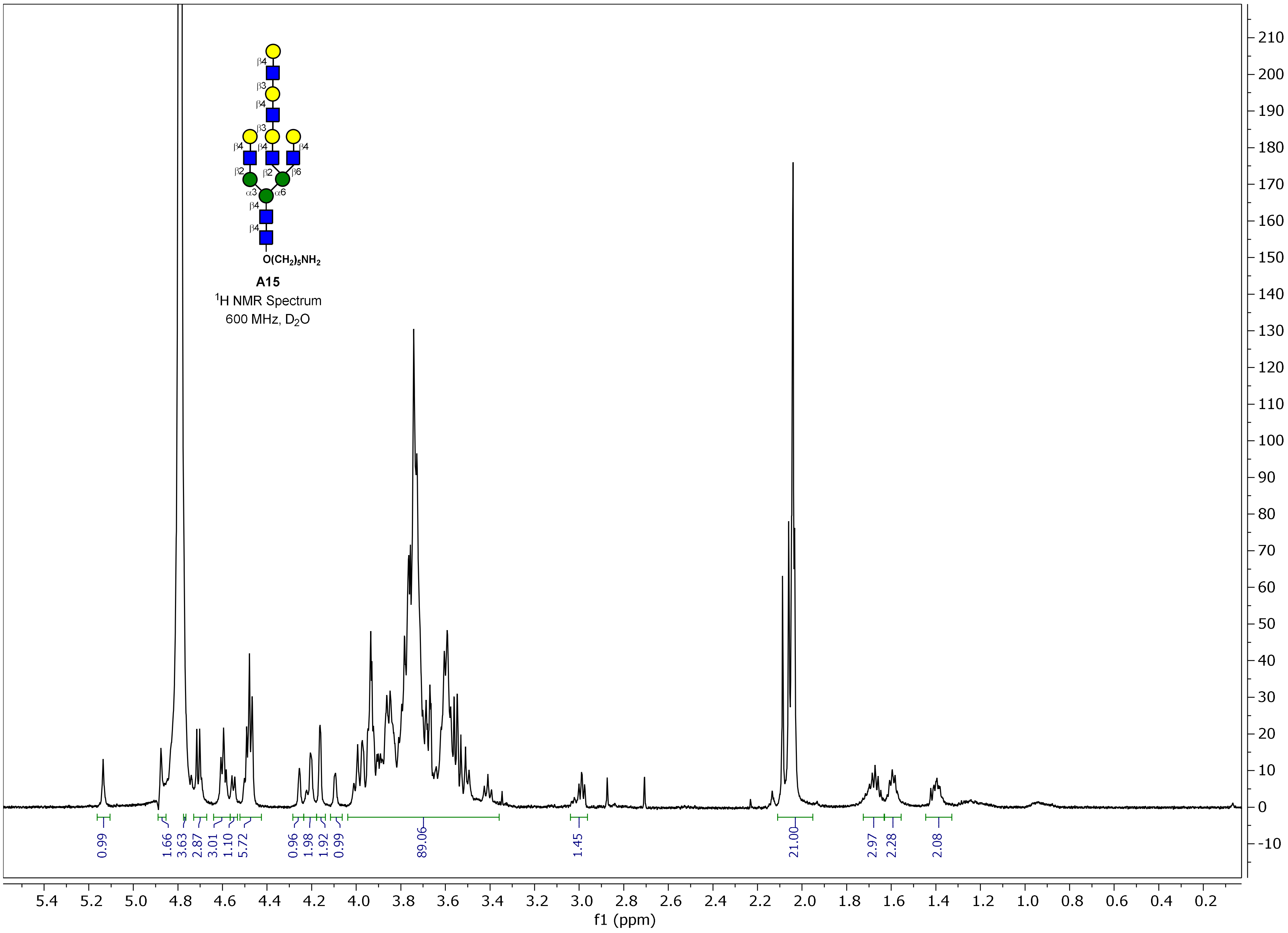
O(CH2)5NH2
A15
1H NMR Spectrum
600 MHz, D2O
f1 (ppm)

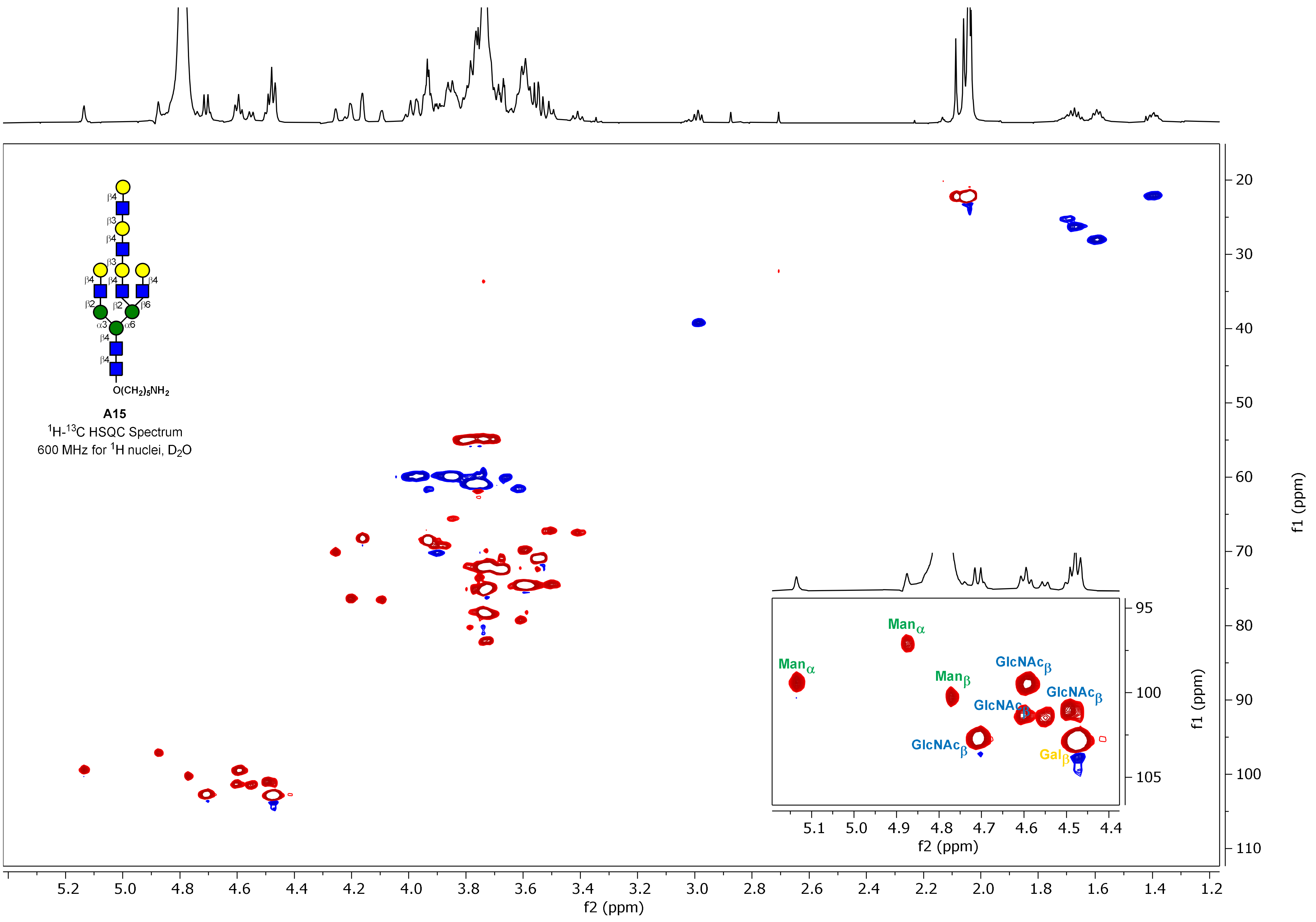

O(CH2)5NH2
A15
1H-13C HSQC Spectrum
600 MHz for 1H nuclei, D2O
Manα
Manα
Manβ
GlcNAcβ
GlcNAcβ
GlcNAcβ
GlcNAcβ
Galβ
f2 (ppm)
f1 (ppm)

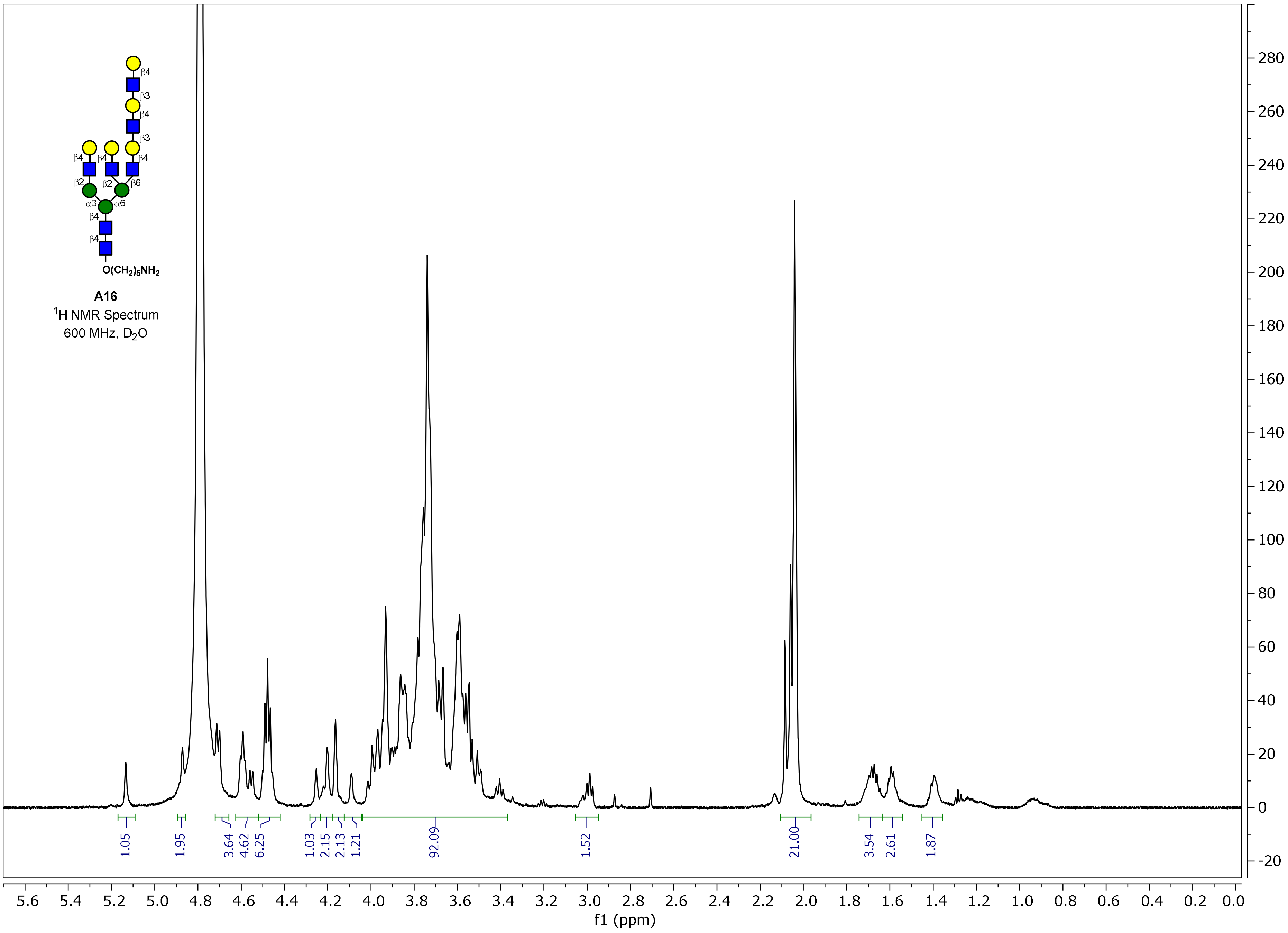

O(CH2)5NH2
A16
1H NMR Spectrum
600 MHz, D2O
f1 (ppm)

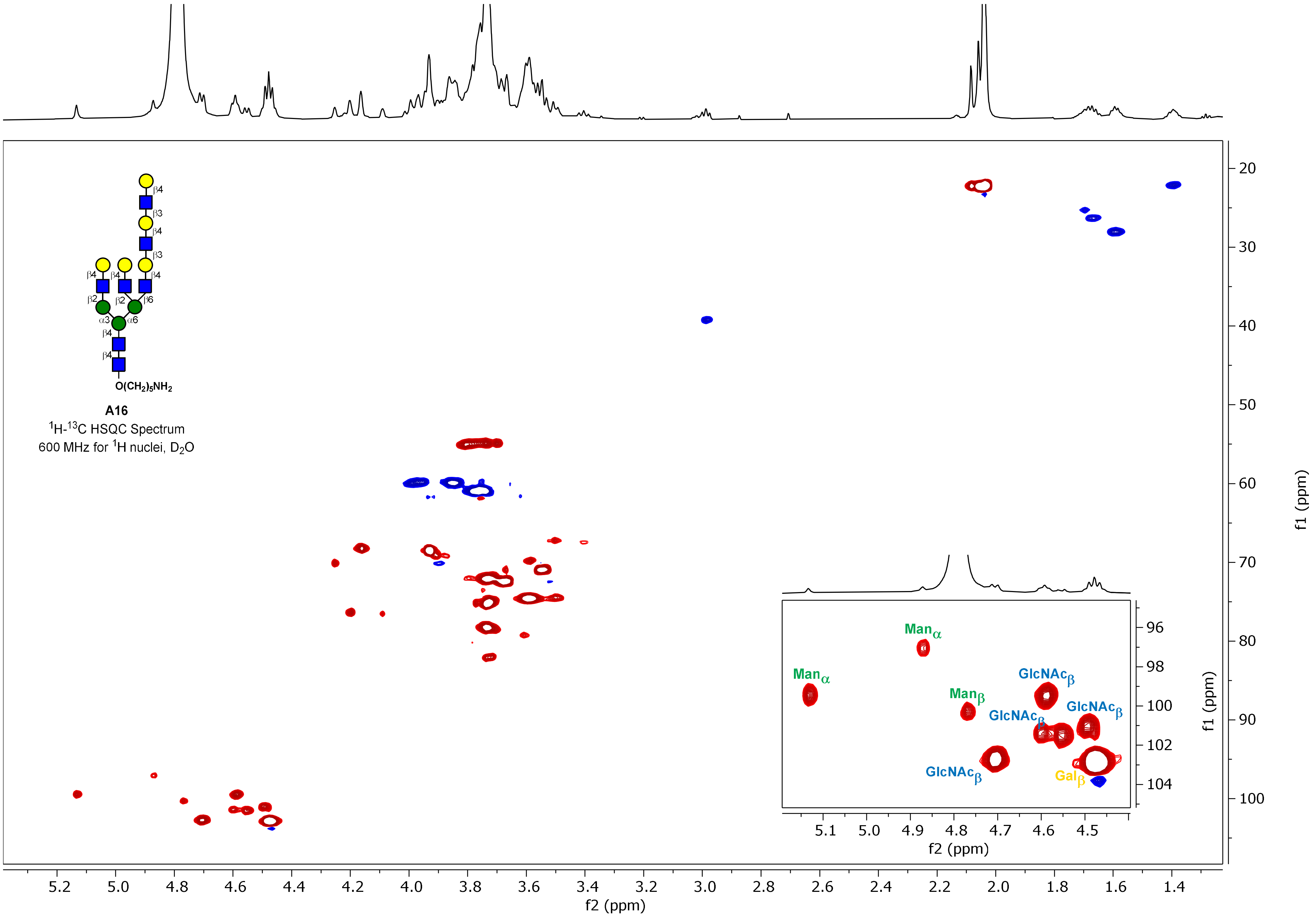

β4
β3
β4
β3
β4
β4
β4
β2
β2
β6
α3
α6
β4
β4
O(CH2)5NH2
A16
1H-13C HSQC Spectrum
600 MHz for 1H nuclei, D2O
Manα
Manα
Manβ
GlcNAcβ
GlcNAcβ
GlcNAcβ
GlcNAcβ
Galβ
f1 (ppm)
f2 (ppm)
f1 (ppm)
f2 (ppm)

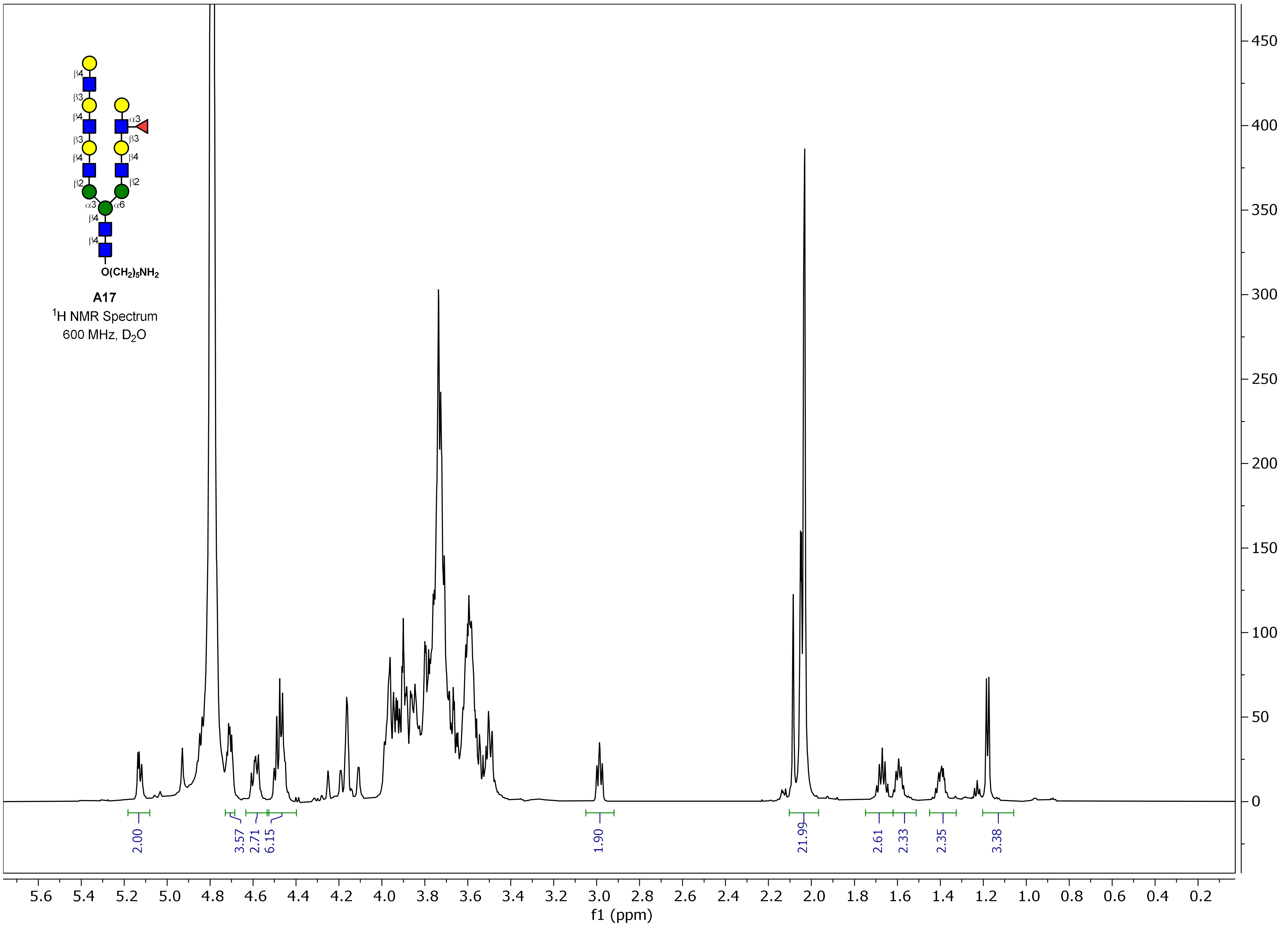

O(CH2)5NH2
A17
1H NMR Spectrum
600 MHz, D2O
f1 (ppm)

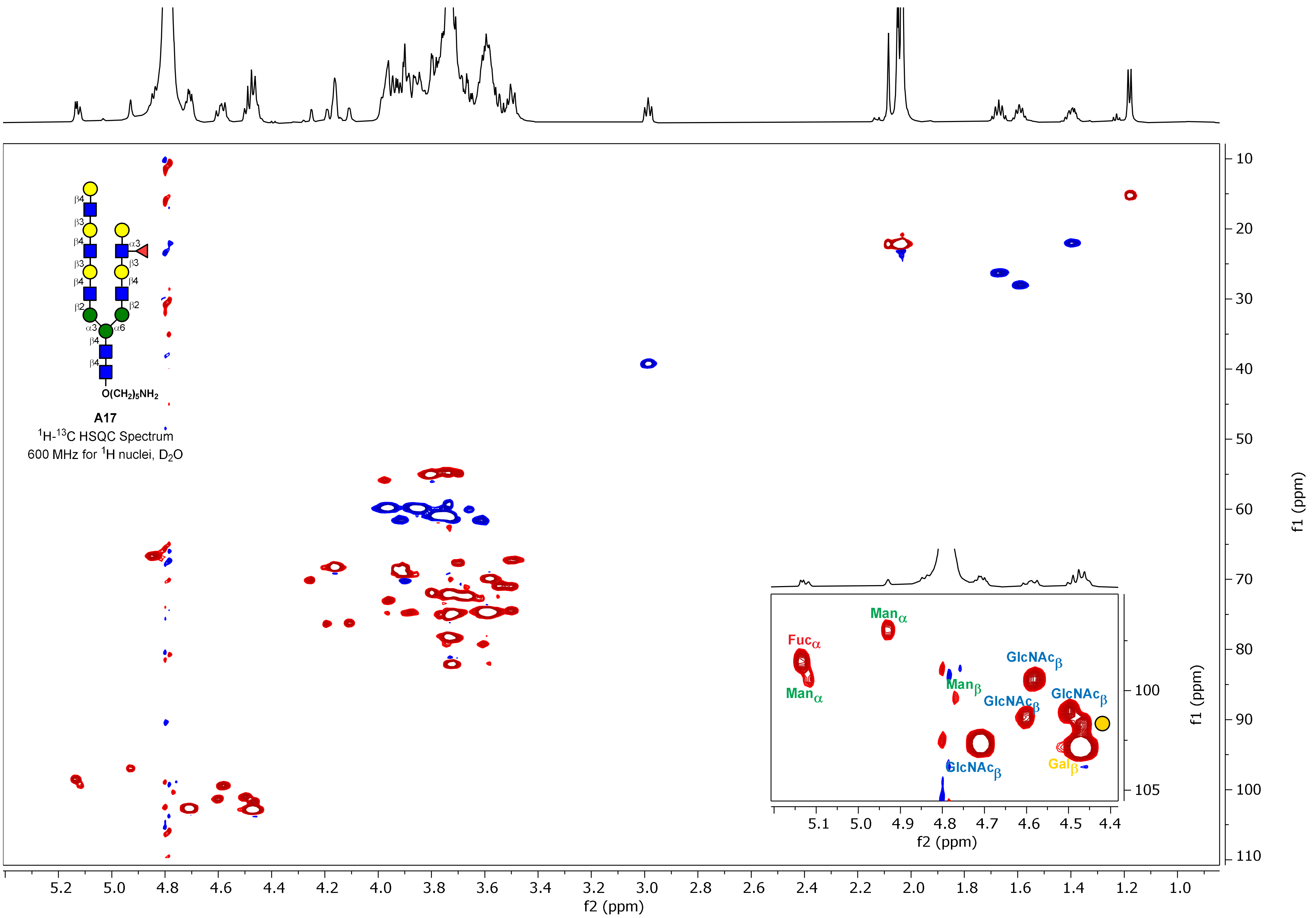

O(CH2)5NH2
A17
1H-13C HSQC Spectrum
600 MHz for 1H nuclei, D2O
Manα
Fucα
Manα
GlcNAcβ
Manβ
GlcNAcβ
GlcNAcβ
GlcNAcβ
Galβ
f2 (ppm)
f1 (ppm)

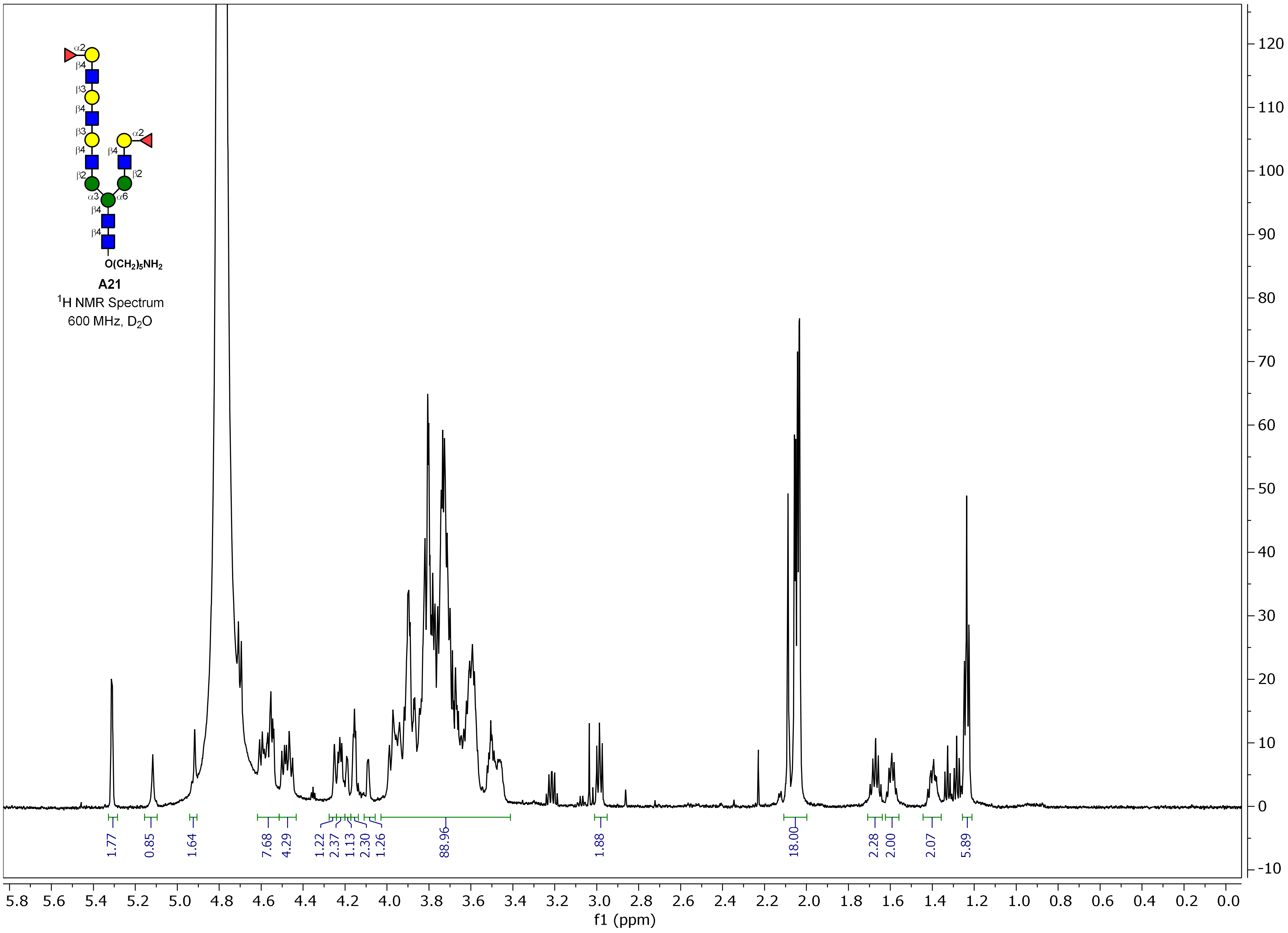

O(CH2)5NH2
A21
1H NMR Spectrum
600 MHz, D2O
f1 (ppm)

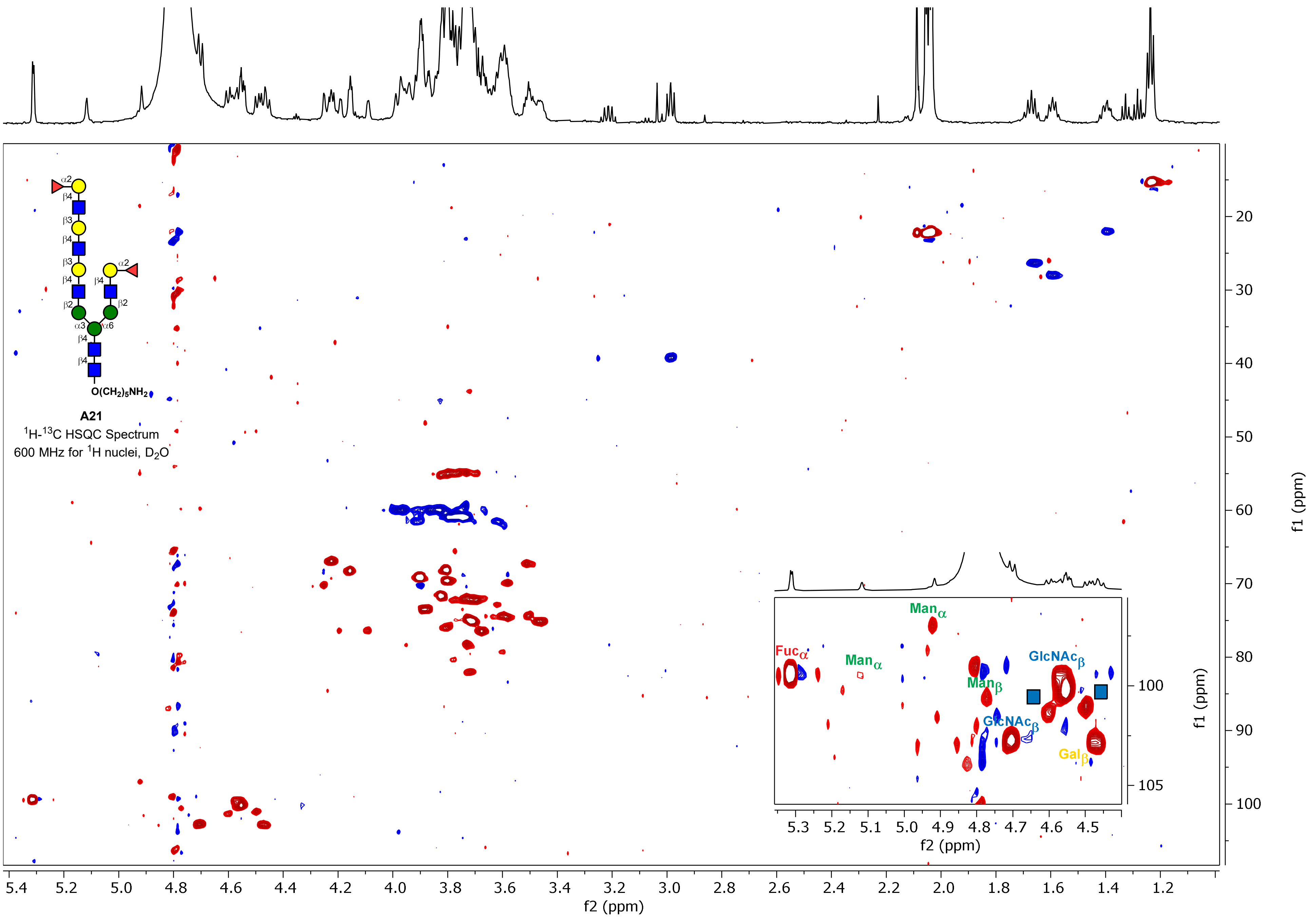

O(CH2)5NH2
A21
1H-13C HSQC Spectrum
600 MHz for 1H nuclei, D2O
Fucα
Manα
Manα
Manβ
GlcNAcβ
GlcNAcβ
Galβ
f2 (ppm)
f1 (ppm)
f2 (ppm)
f1 (ppm)

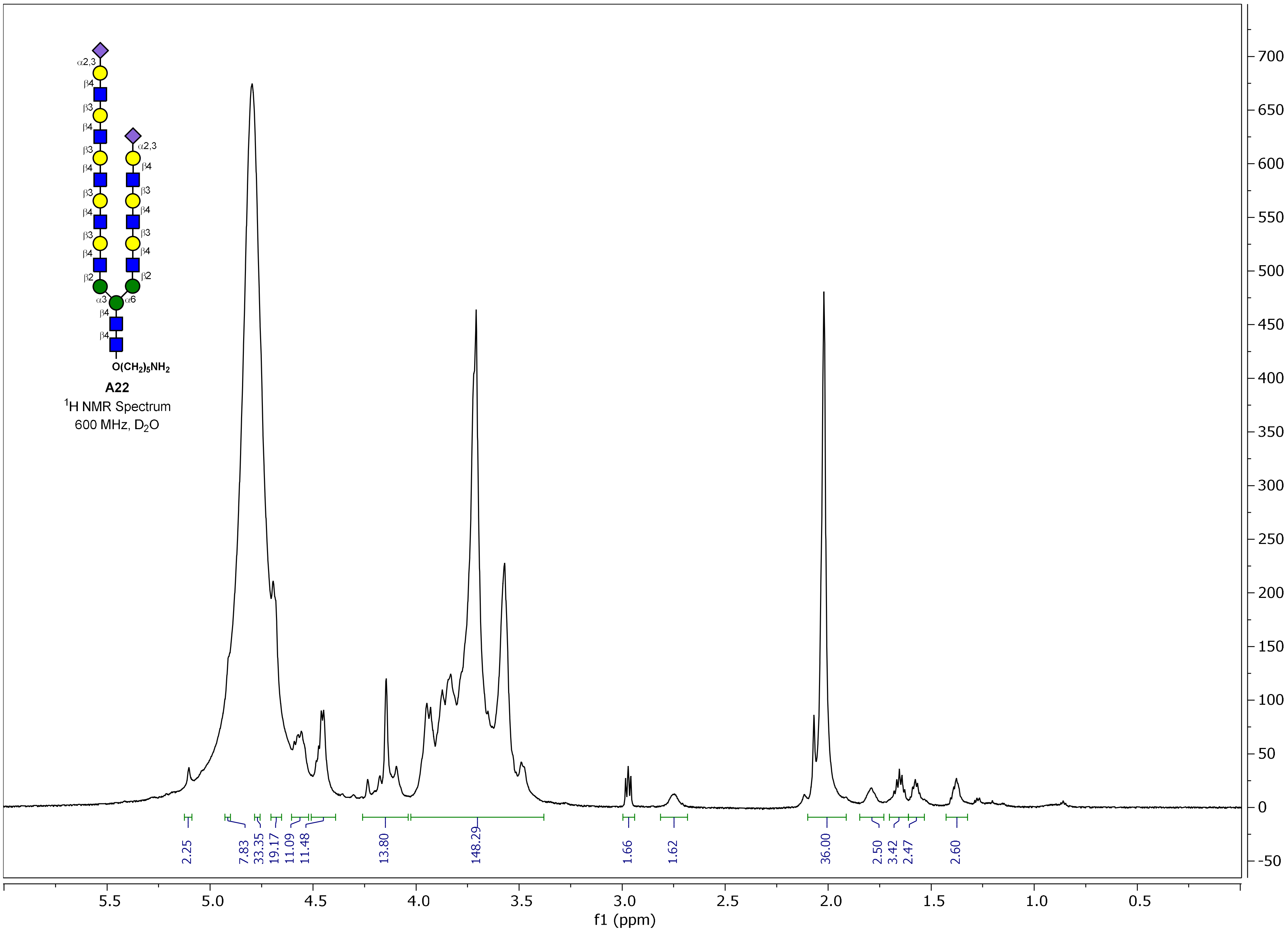

A22
^{1}H NMR Spectrum
600 MHz, D_2O
O(CH2)5NH2
f1 (ppm)

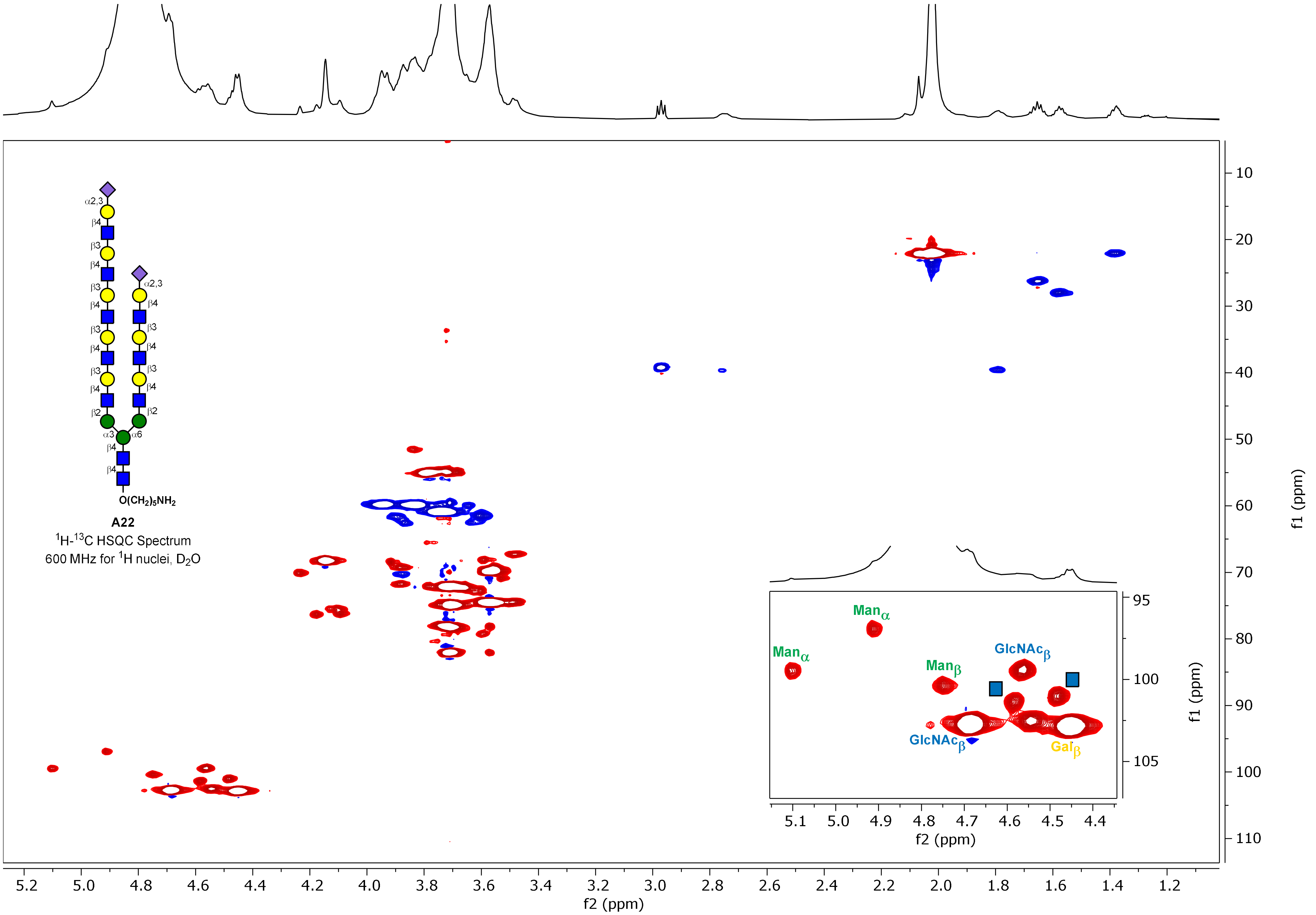
α2,3
β4
β3
β2
α3
α6
O(CH2)5NH2
A22
1H-13C HSQC Spectrum
600 MHz for 1H nuclei, D2O
Manα
Manβ
GlcNAcβ
Galβ
f2 (ppm)
f1 (ppm)

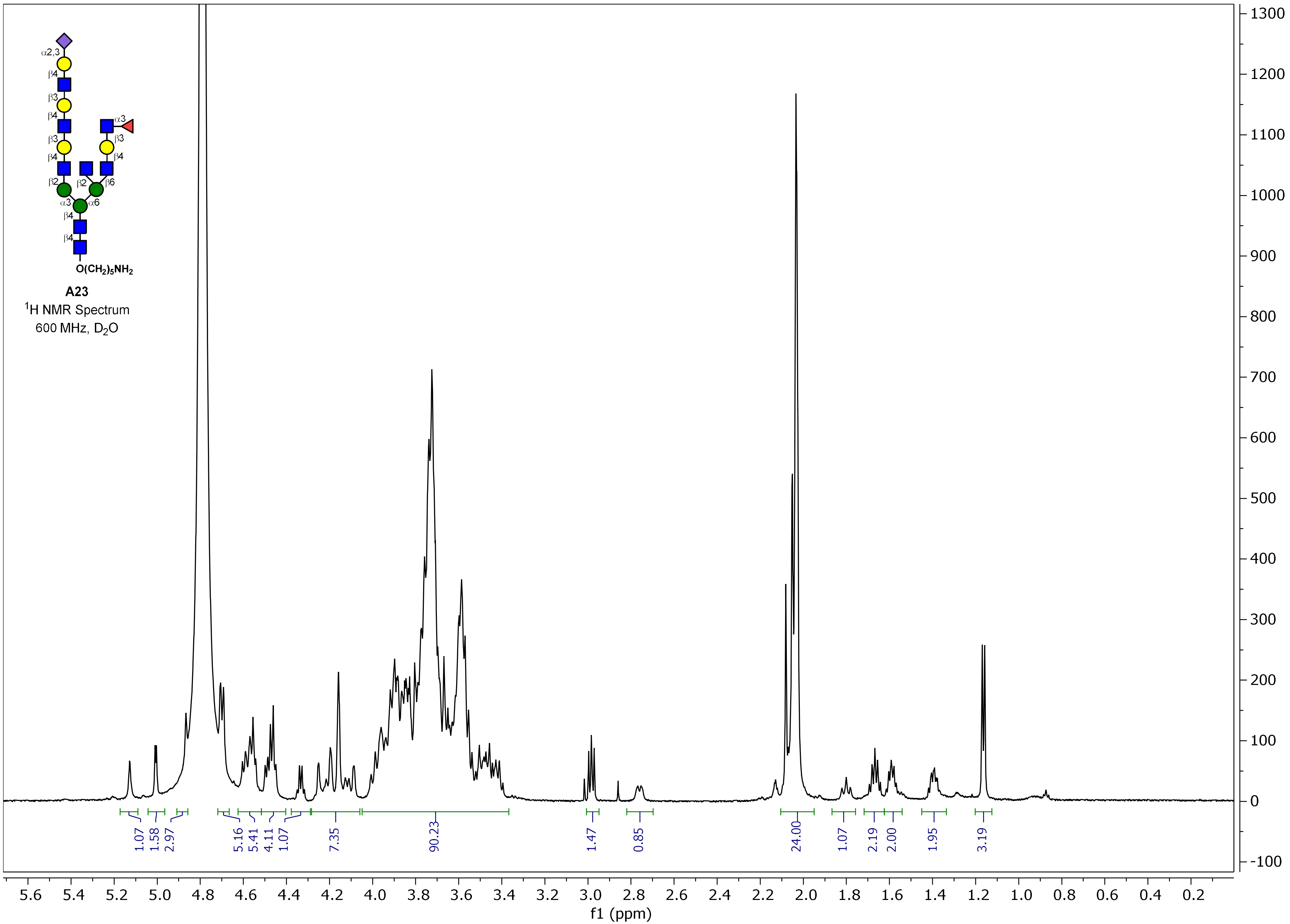
A23
¹H NMR Spectrum
600 MHz, D₂O
O(CH₂)₅NH₂
1.07
1.58
2.97
5.16
5.41
4.11
1.07
7.35
90.23
1.47
0.85
24.00
1.07
2.19
2.00
1.95
3.19
f1 (ppm)

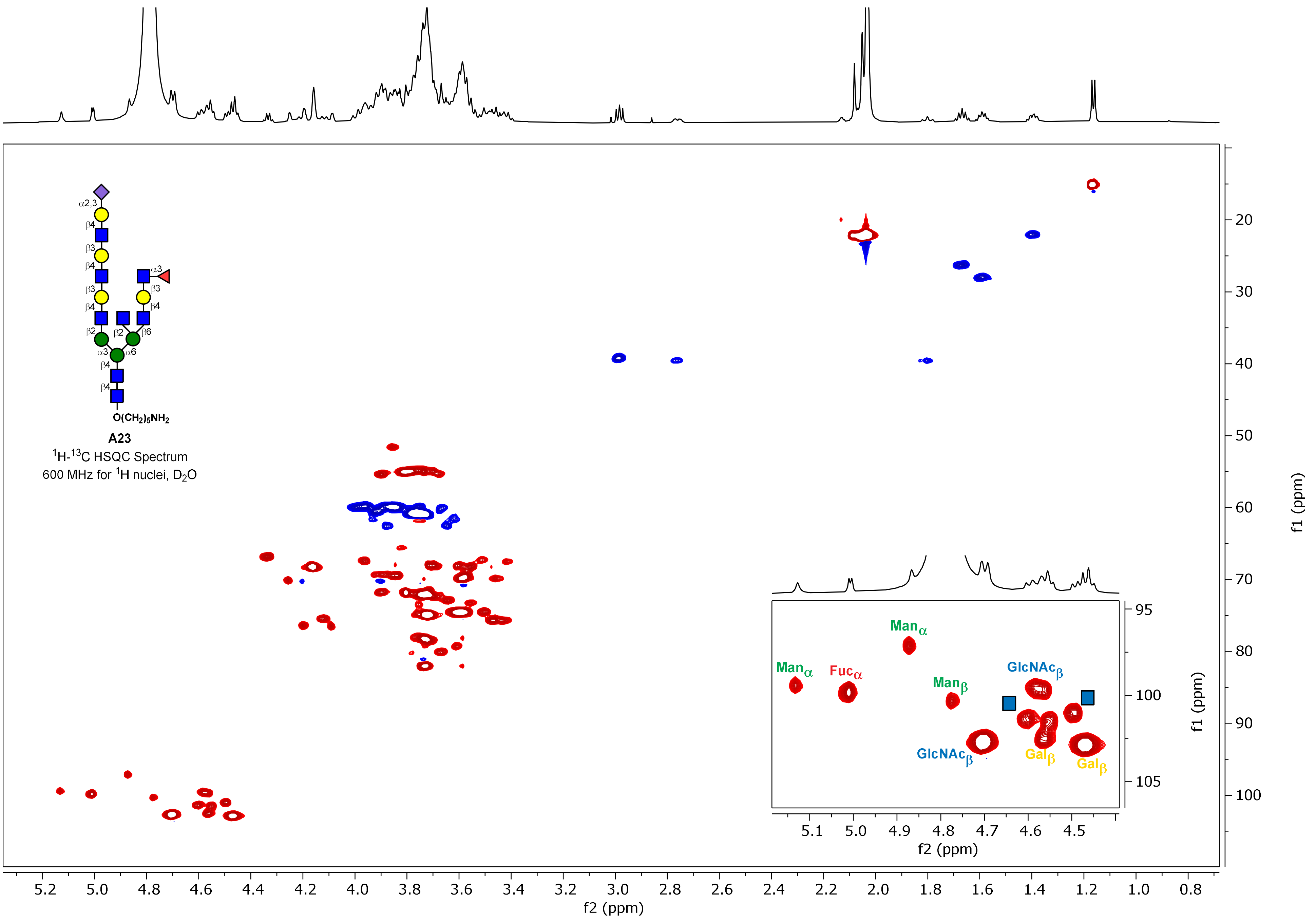
α2,3
β4
β3
β4
β3
β4
β2
α3
β3
β4
β2
β6
α3
α6
β4
β4
O(CH2)5NH2
A23
1H-13C HSQC Spectrum
600 MHz for 1H nuclei, D2O
Manα
Manα
Fucα
Manβ
GlcNAcβ
GlcNAcβ
Galβ
Galβ
f1 (ppm)
f2 (ppm)

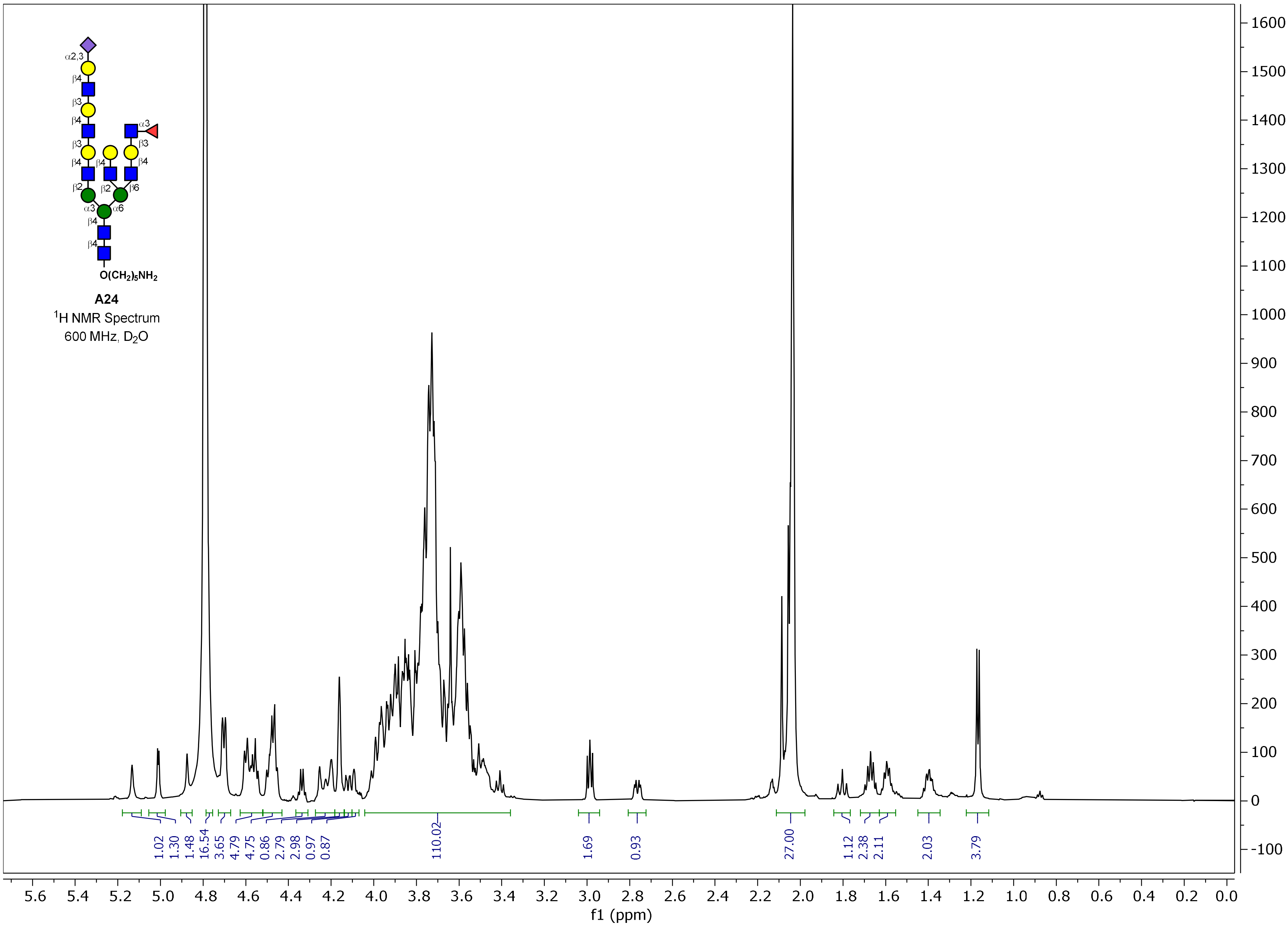

A24
^{1}H NMR Spectrum
600 MHz, D_2O
O(CH2)5NH2
f1 (ppm)

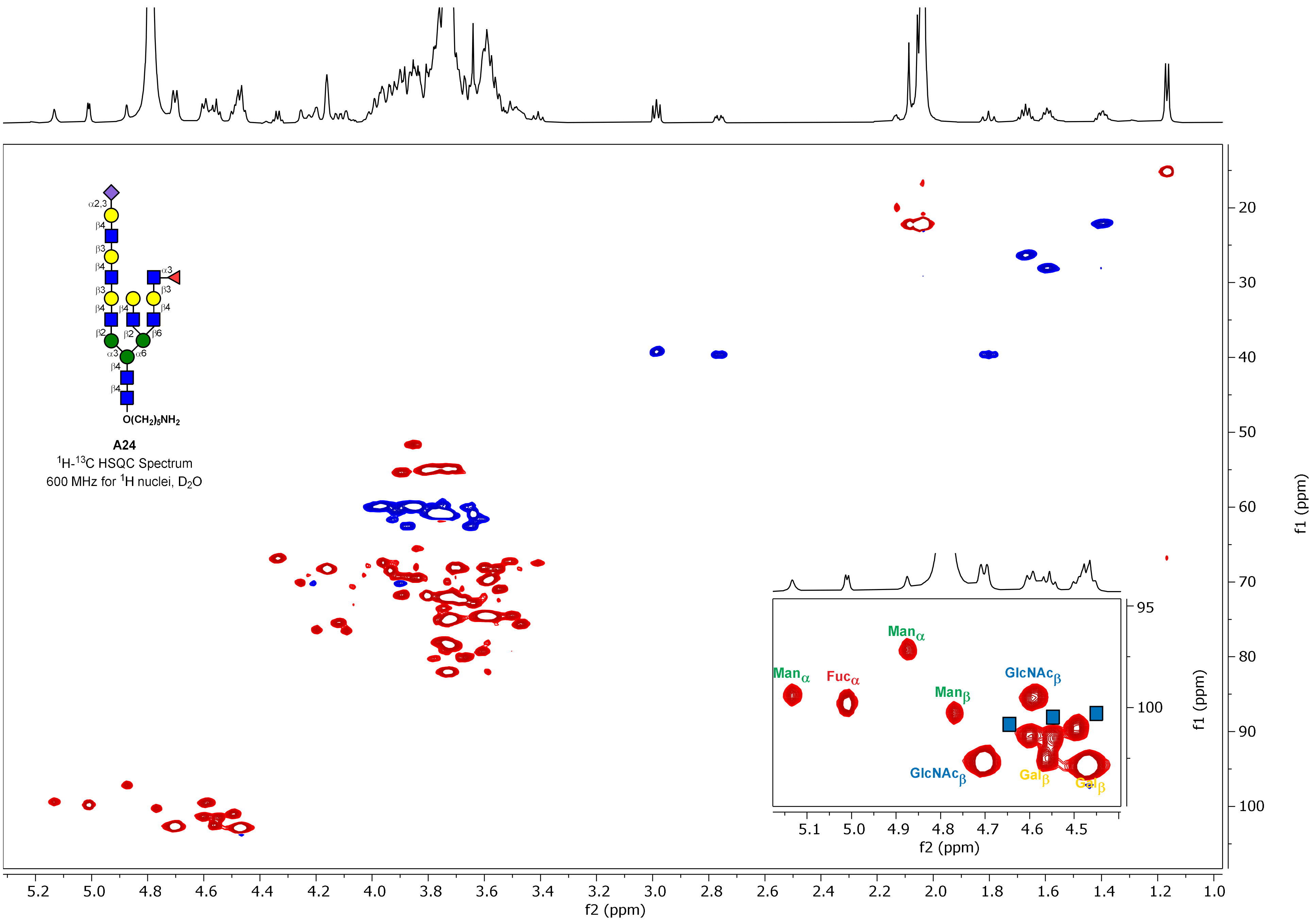
α2,3
β4
β3
β4
α3
β3
β4
β4
β4
β2
β2
β6
α3
α6
β4
β4
O(CH2)5NH2
A24
1H-13C HSQC Spectrum
600 MHz for 1H nuclei, D2O
Manα
Fucα
Manα
Manβ
GlcNAcβ
GlcNAcβ
Galβ
Galβ
f1 (ppm)
f2 (ppm)
f1 (ppm)
f2 (ppm)

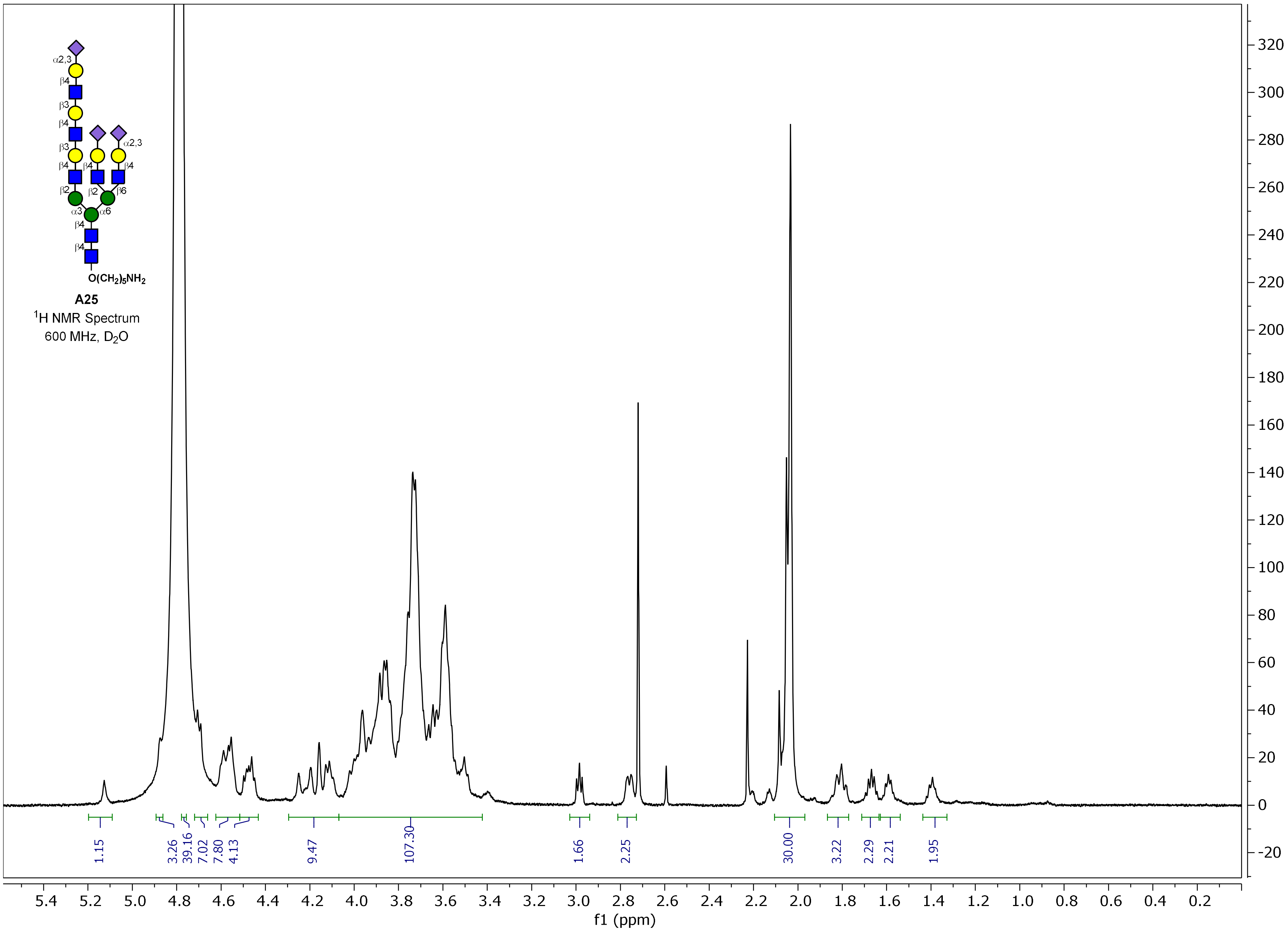

A25
1H NMR Spectrum
600 MHz, D2O
O(CH2)5NH2
f1 (ppm)
1.15
3.26
39.16
7.02
7.80
4.13
9.47
107.30
1.66
2.25
30.00
3.22
2.29
2.21
1.95

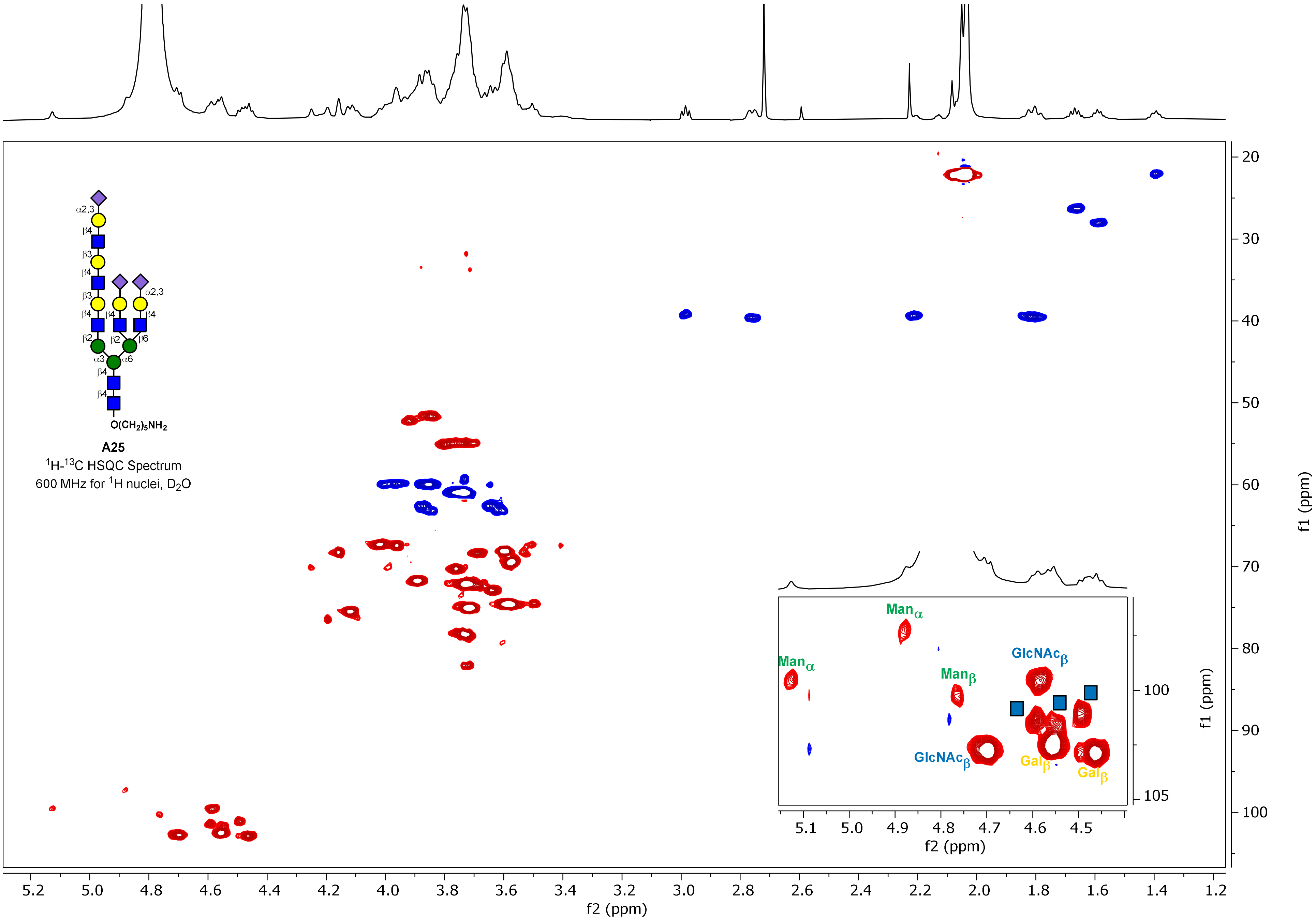

α2,3
β4
β3
β4
β3
β4
β4
α2,3
β4
β2
β2
β6
α3
α6
β4
β4
O(CH2)5NH2
A25
1H-13C HSQC Spectrum
600 MHz for 1H nuclei, D2O
Manα
Manα
GlcNAcβ
Manβ
GlcNAcβ
Galβ
Galβ
f2 (ppm)
f1 (ppm)
f2 (ppm)
f1 (ppm)

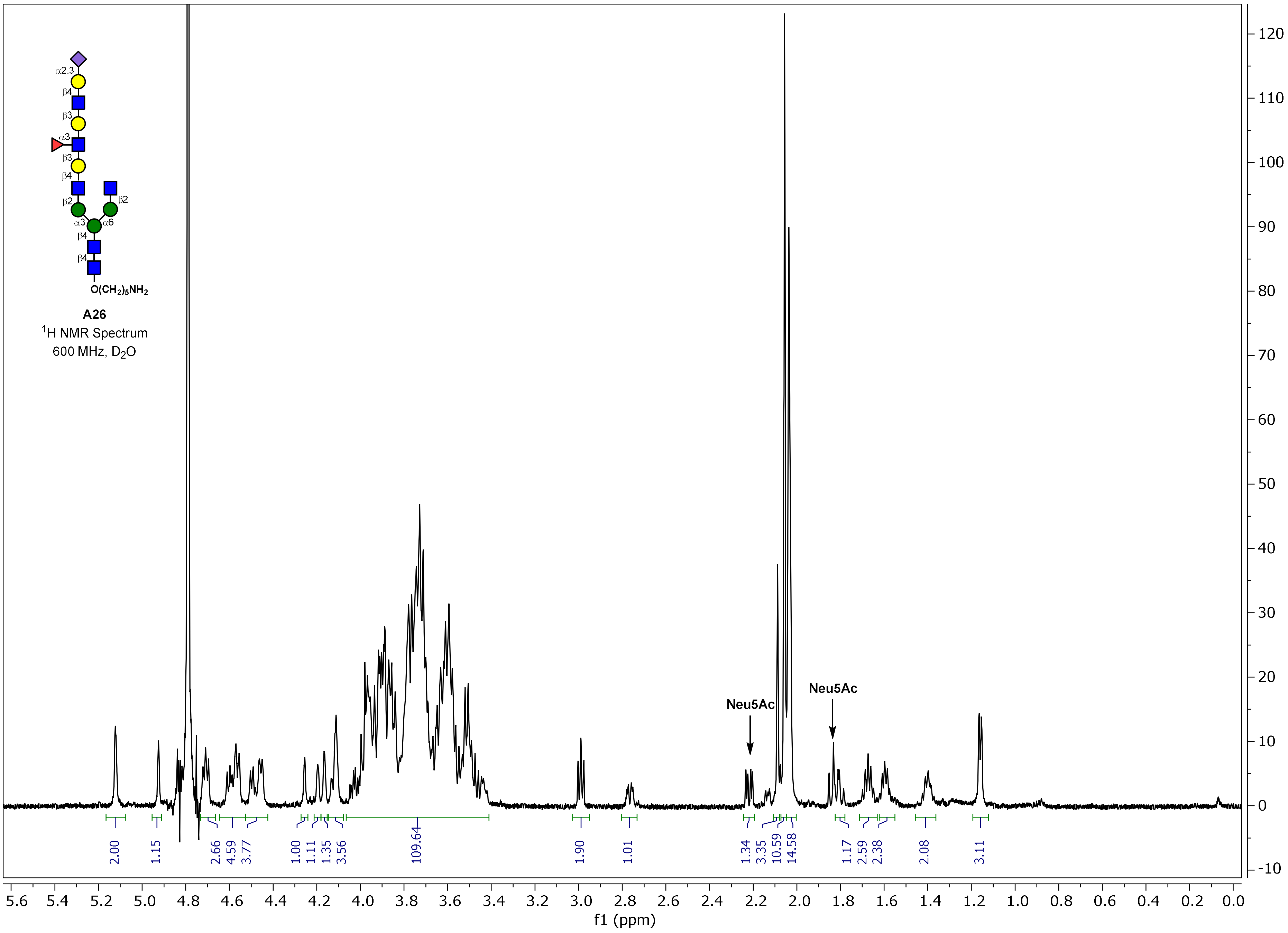
O(CH2)5NH2
A26
1H NMR Spectrum
600 MHz, D2O
Neu5Ac
Neu5Ac
f1 (ppm)

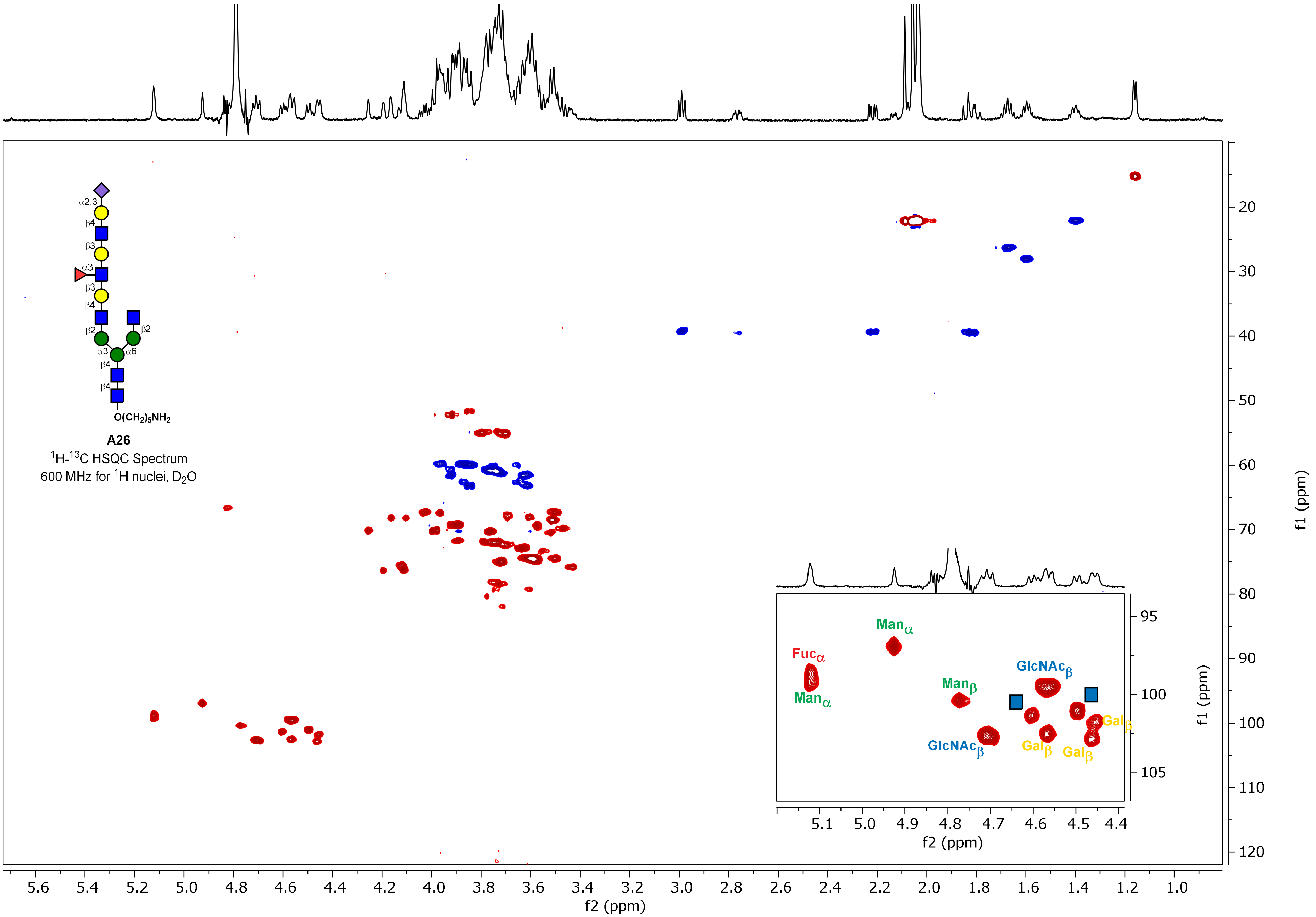

α2,3
β4
β3
α3
β3
β4
β2
β2
α3
α6
β4
β4
O(CH2)5NH2
A26
1H-13C HSQC Spectrum
600 MHz for 1H nuclei, D2O
Fucα
Manα
Manα
Manβ
GlcNAcβ
GlcNAcβ
Galβ
Galβ
Galβ
f2 (ppm)
f1 (ppm)
f2 (ppm)
f1 (ppm)

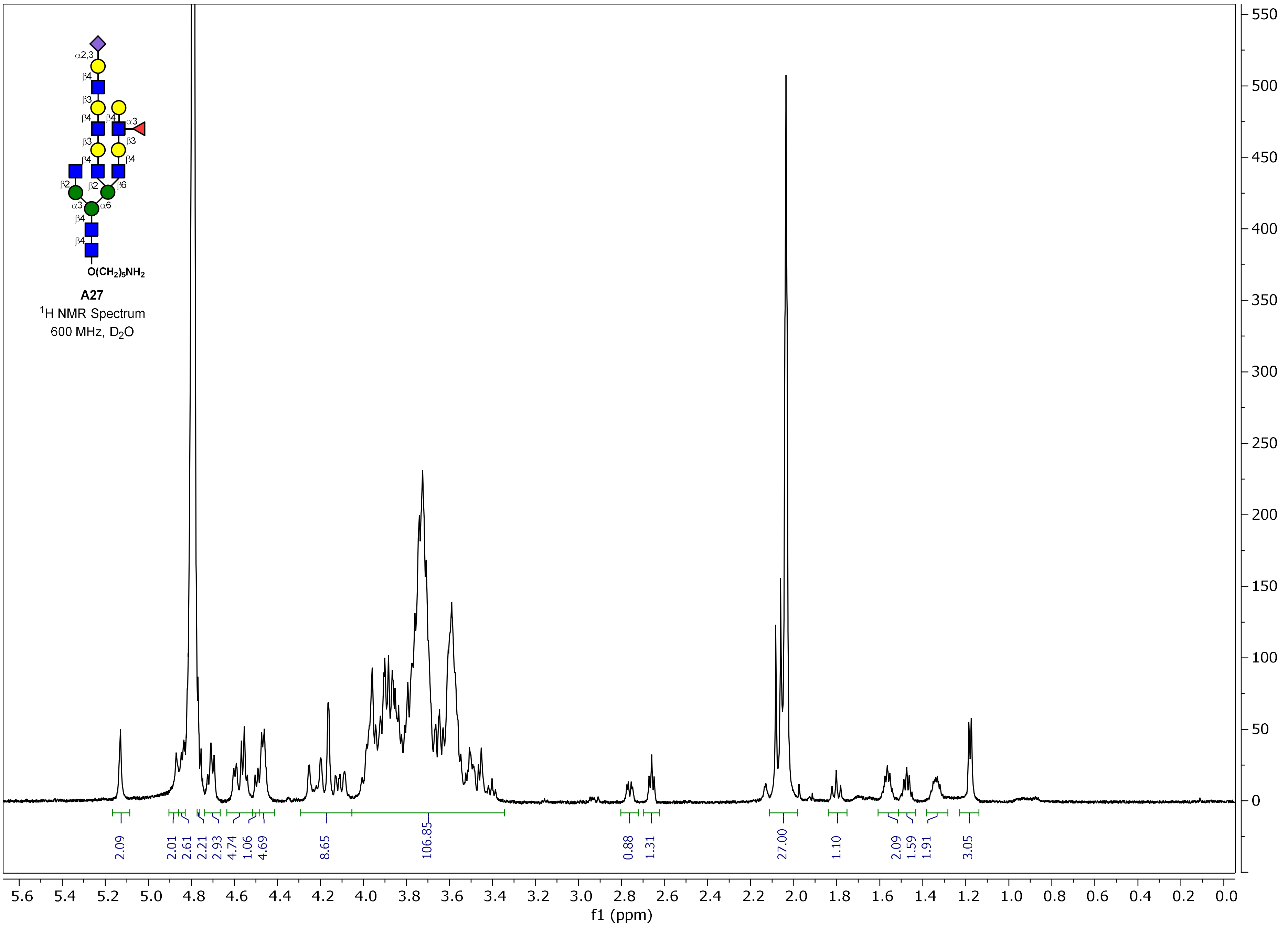
α2,3
β4
β3
β4
β4
α3
β3
β3
β4
β4
β2
β2
β6
α3
α6
β4
β4
O(CH2)5NH2
A27
1H NMR Spectrum
600 MHz, D2O
2.09
2.01
2.61
2.21
2.93
4.74
1.06
4.69
8.65
106.85
0.88
1.31
27.00
1.10
2.09
1.59
1.91
3.05
f1 (ppm)

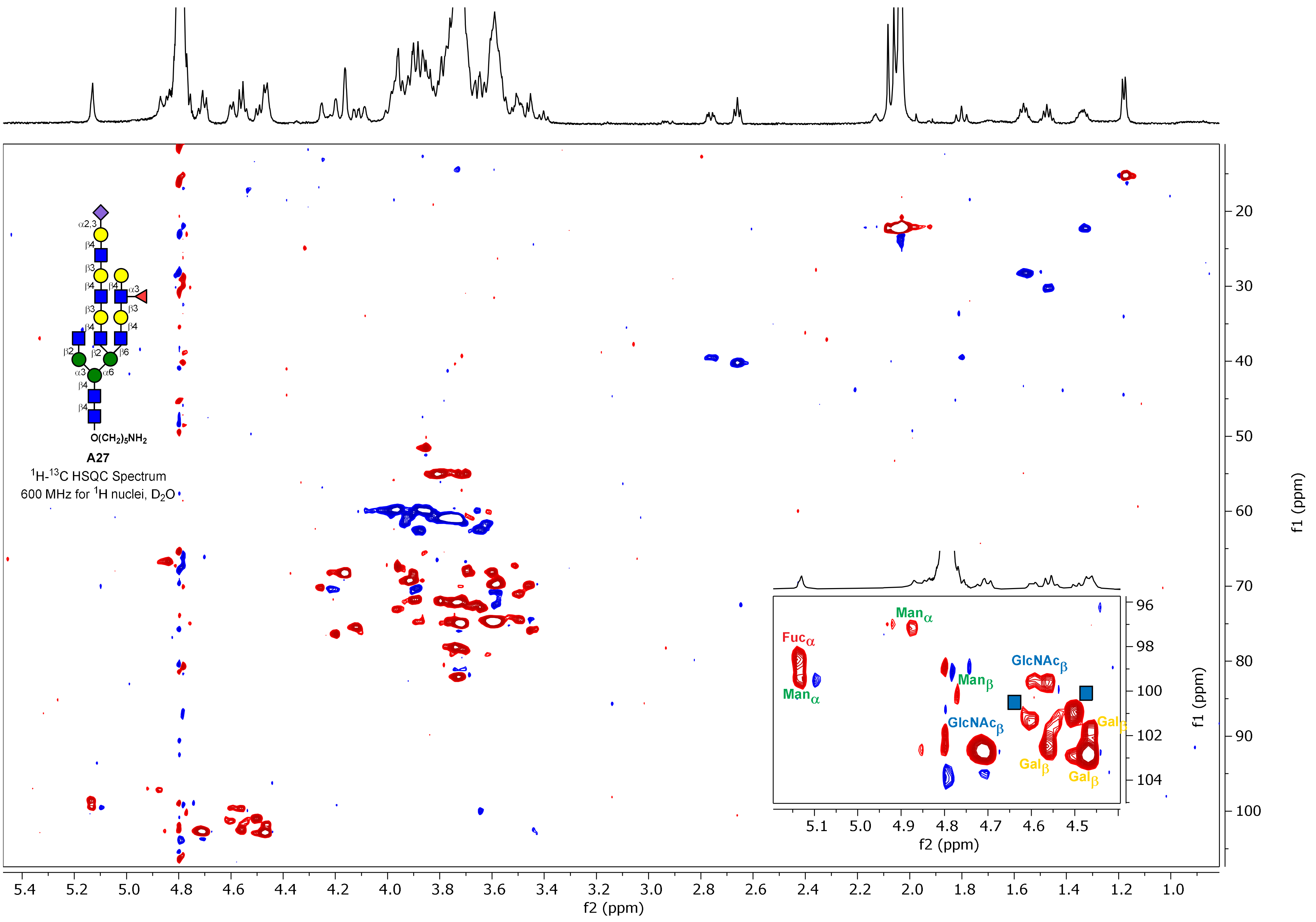

A27
^1H-^{13}C HSQC Spectrum
600 MHz for ^1H nuclei, D_2O
O(CH2)5NH2
α2,3
β4
β3
α3
β2
β6
α6
Fucα
Manα
Manβ
GlcNAcβ
Galβ
f2 (ppm)
f1 (ppm)

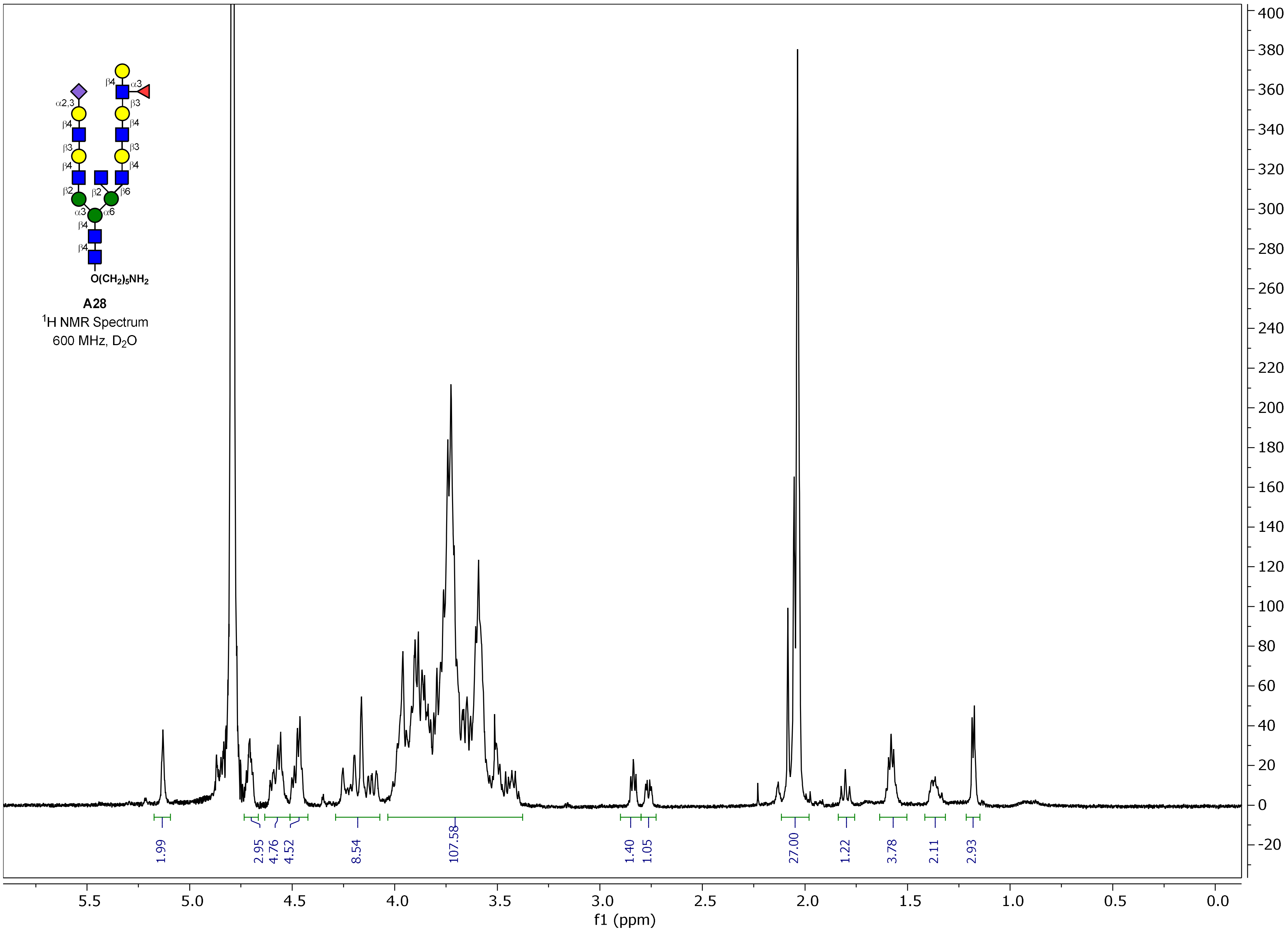

O(CH2)5NH2
A28
1H NMR Spectrum
600 MHz, D2O
1.99
2.95
4.76
4.52
8.54
107.58
1.40
1.05
27.00
1.22
3.78
2.11
2.93
f1 (ppm)

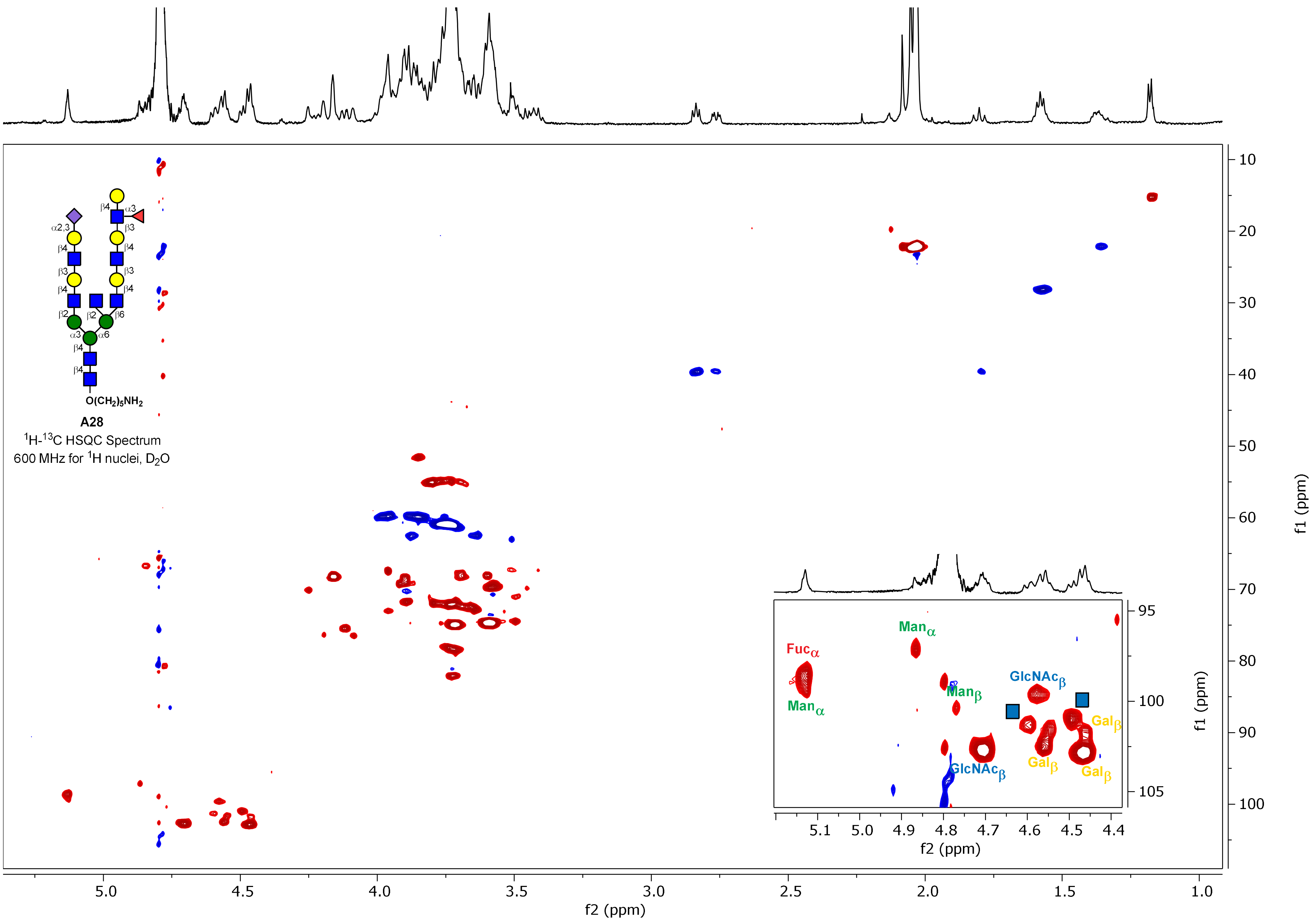

O(CH2)5NH2
A28
1H-13C HSQC Spectrum
600 MHz for 1H nuclei, D2O
Fucα
Manα
Manα
Manβ
GlcNAcβ
GlcNAcβ
Galβ
Galβ
Galβ
f2 (ppm)
f1 (ppm)

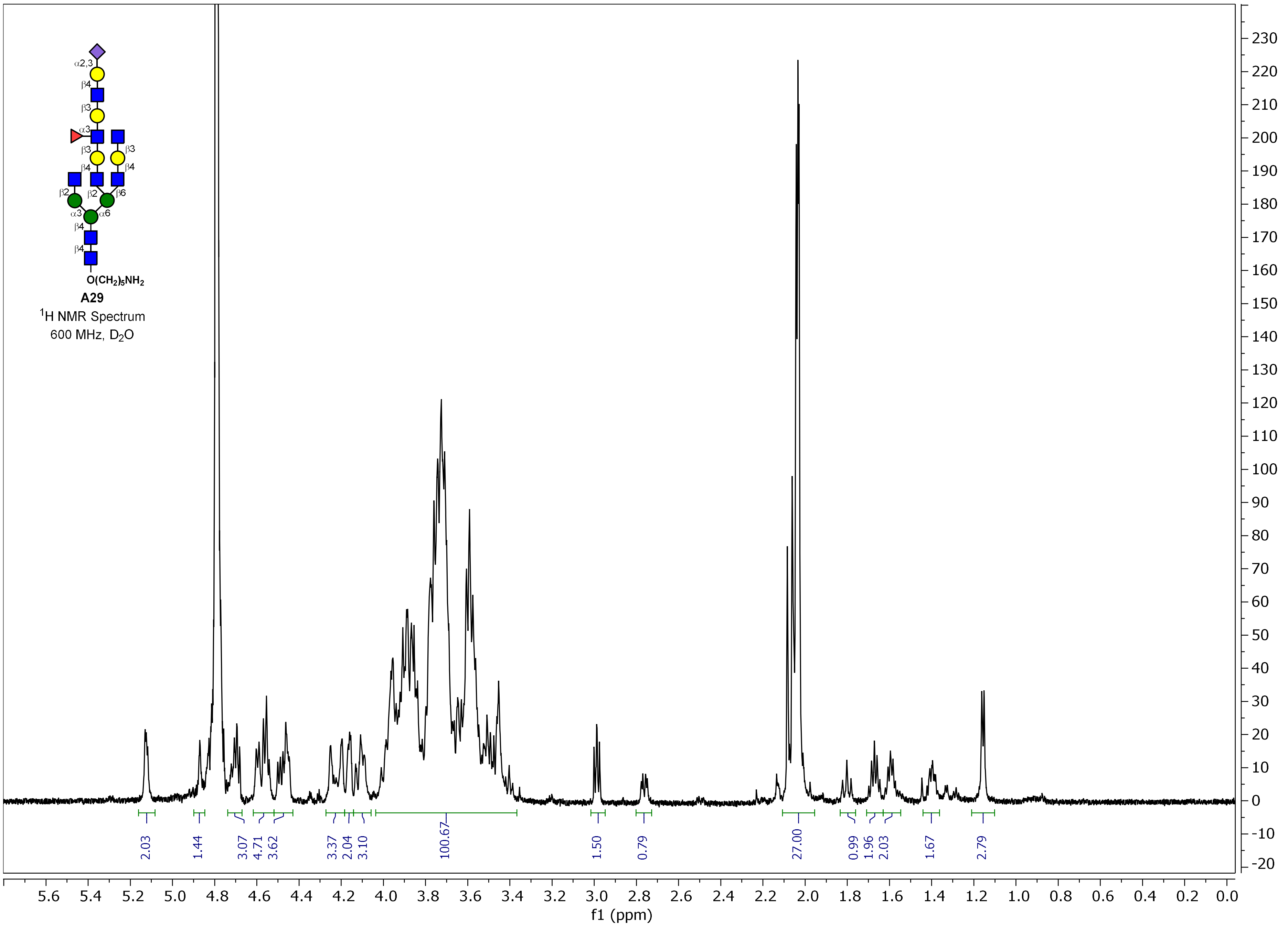

α2,3
β4
β3
α3
β3
β3
β4
β4
β2
β2
β6
α3
α6
β4
β4
O(CH2)5NH2
A29
1H NMR Spectrum
600 MHz, D2O
2.03
1.44
3.07
4.71
3.62
3.37
2.04
3.10
100.67
1.50
0.79
27.00
0.99
1.96
2.03
1.67
2.79
f1 (ppm)

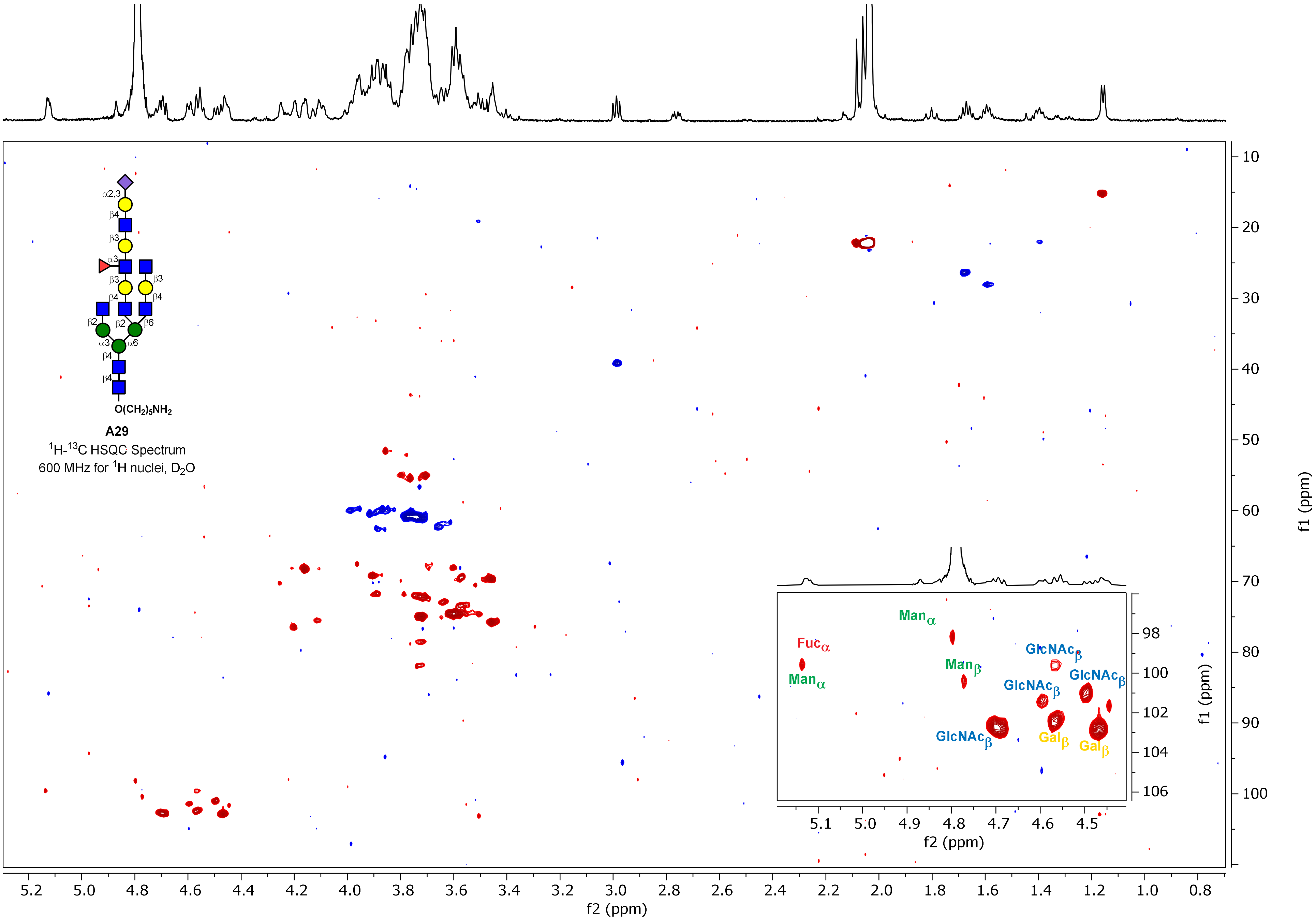

α2,3
β4
β3
α3
β3
β4
β3
β4
β2
β2
β6
α3
α6
β4
β4
O(CH2)5NH2
A29
1H-13C HSQC Spectrum
600 MHz for 1H nuclei, D2O
Manα
Fucα
Manα
Manβ
GlcNAcβ
GlcNAcβ
GlcNAcβ
GlcNAcβ
Galβ
Galβ
f2 (ppm)
f1 (ppm)
f2 (ppm)
f1 (ppm)

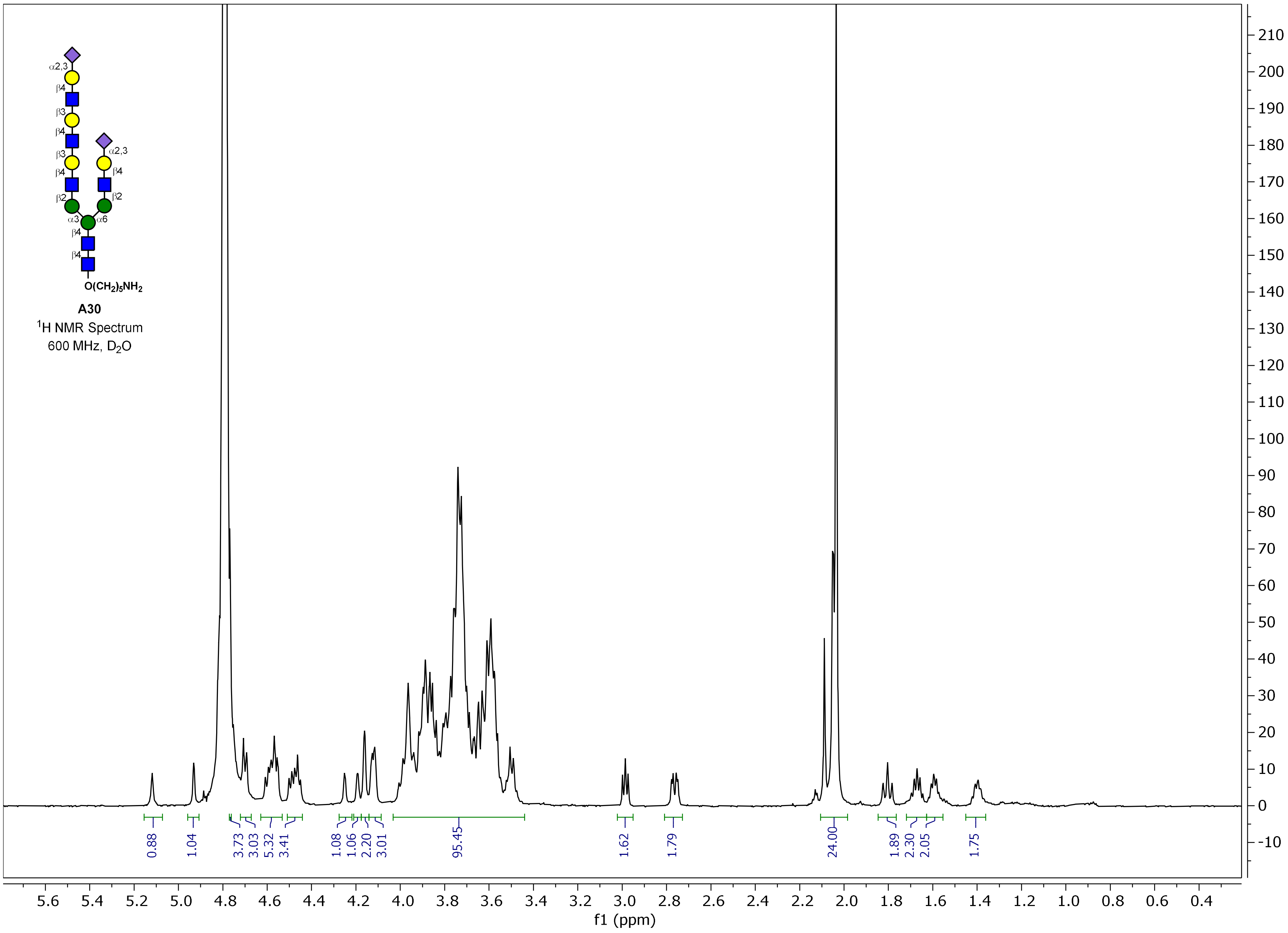

α2,3
β4
β3
β4
β3
β4
β2
α2,3
β4
β2
α3
α6
β4
β4
O(CH2)5NH2
A30
1H NMR Spectrum
600 MHz, D2O
0.88
1.04
3.73
3.03
5.32
3.41
1.08
1.06
2.20
3.01
95.45
1.62
1.79
24.00
1.89
2.30
2.05
1.75
f1 (ppm)

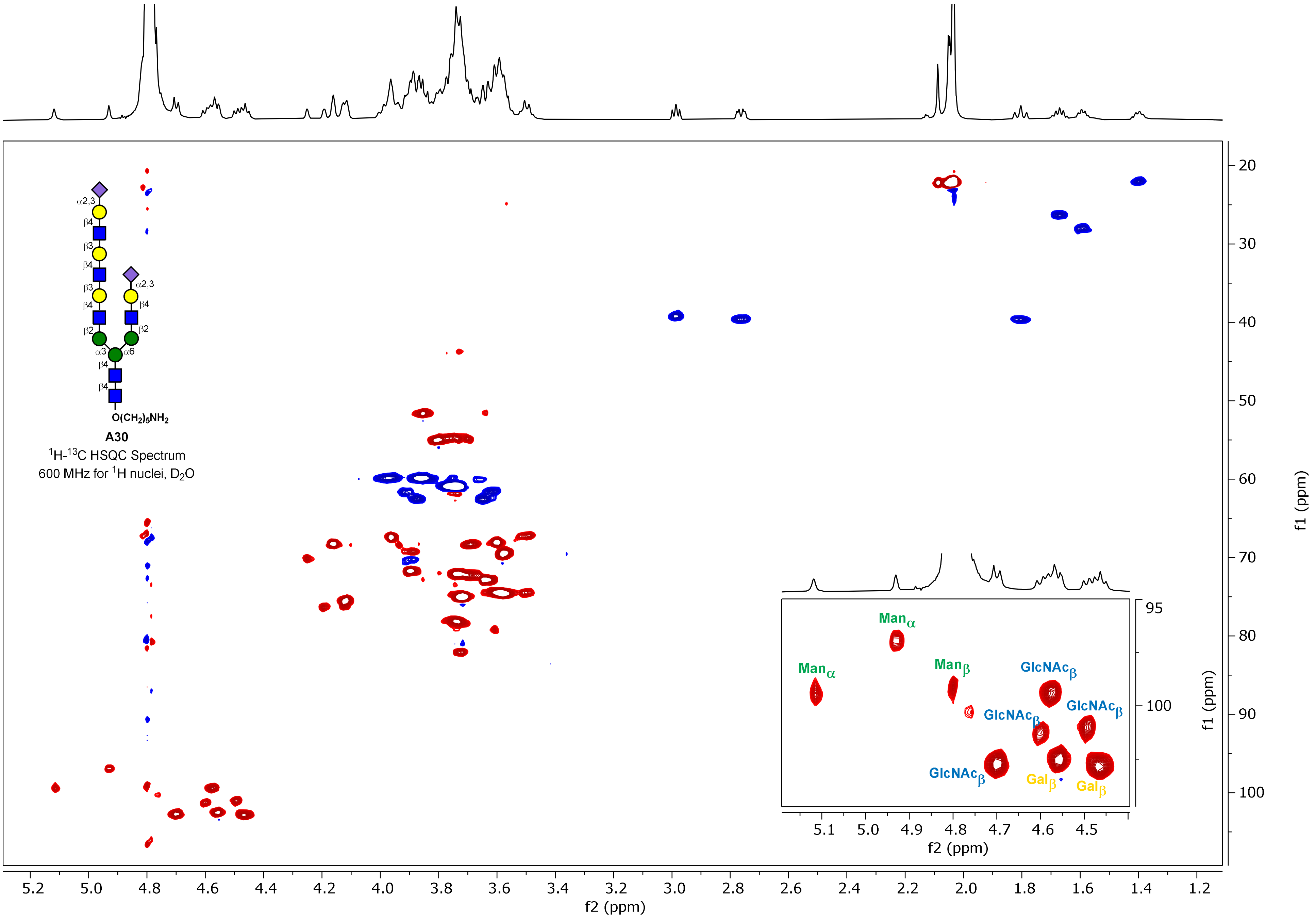
α2,3
β4
β3
β4
β3
β4
β2
α2,3
β4
β2
α3
α6
β4
β4
O(CH2)5NH2
A30
1H-13C HSQC Spectrum
600 MHz for 1H nuclei, D2O
Manα
Manα
Manβ
GlcNAcβ
GlcNAcβ
GlcNAcβ
GlcNAcβ
Galβ
Galβ
f2 (ppm)
f1 (ppm)
f2 (ppm)
f1 (ppm)

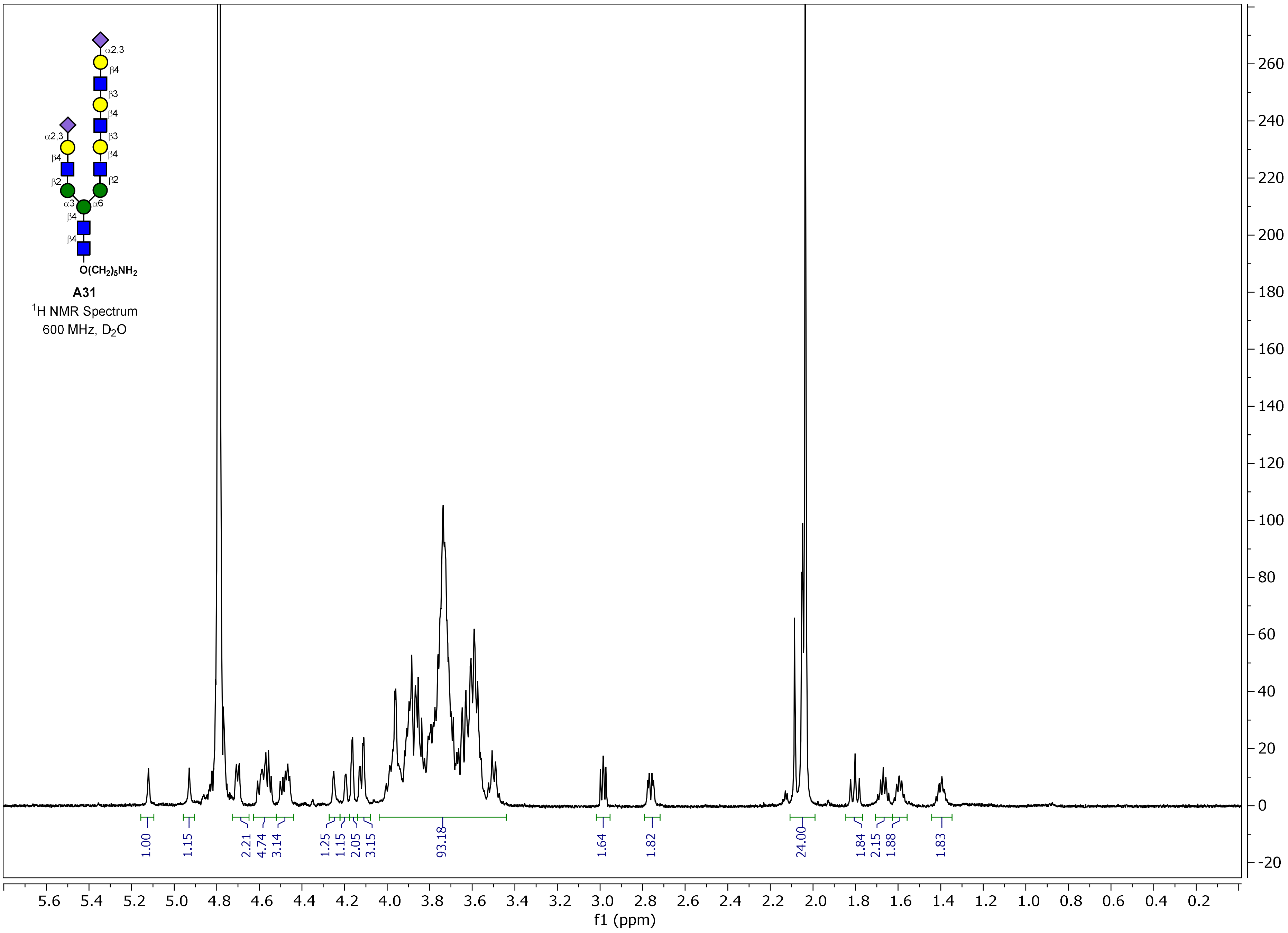

α2,3
β4
β3
β4
β3
β4
β2
α2,3
β4
β2
α3
α6
β4
β4
O(CH2)5NH2
A31
1H NMR Spectrum
600 MHz, D2O
1.00
1.15
2.21
4.74
3.14
1.25
1.15
2.05
3.15
93.18
1.64
1.82
24.00
1.84
2.15
1.88
1.83
f1 (ppm)

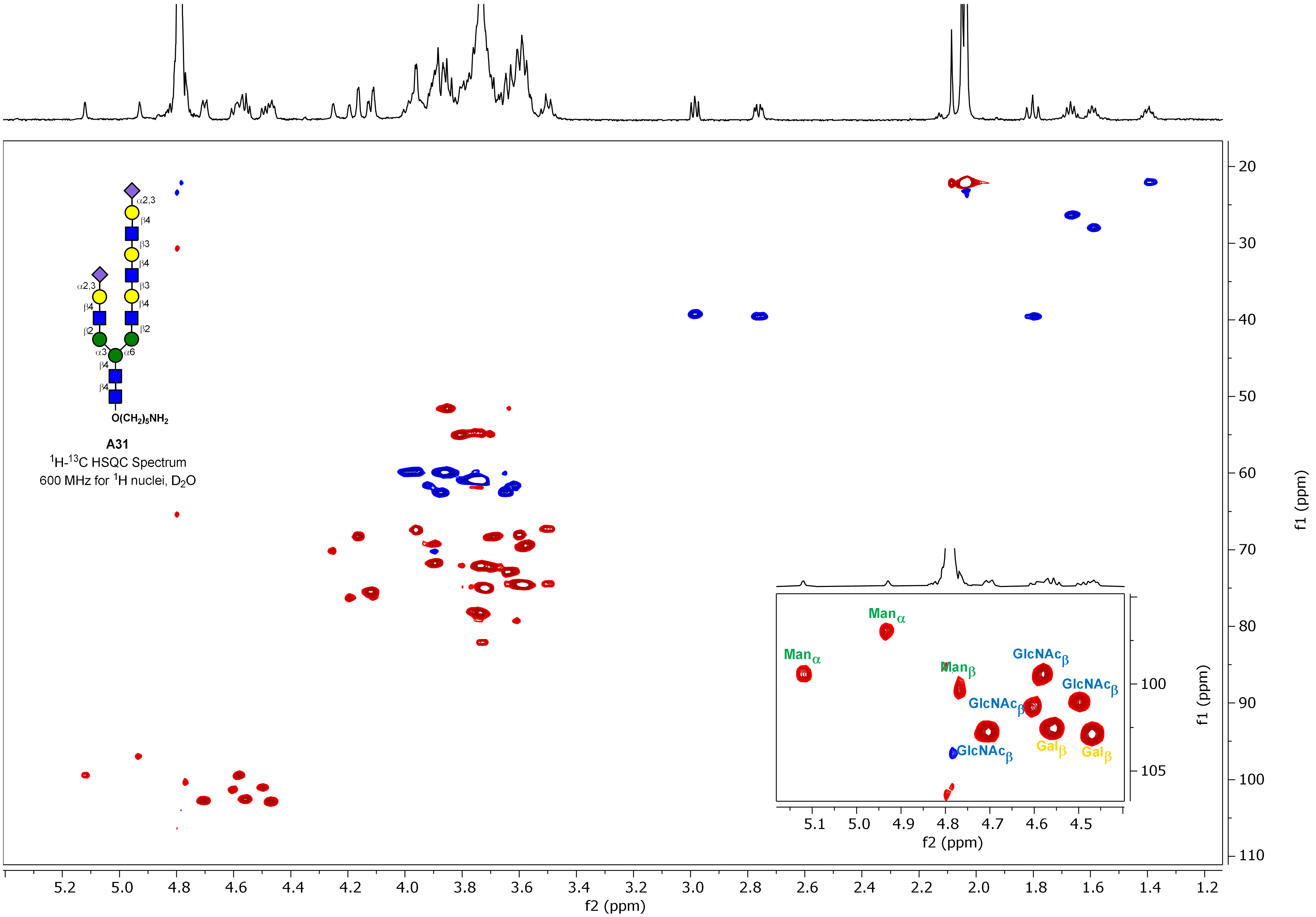
α2,3
β4
β3
β2
α3
α6
O(CH2)5NH2
A31
1H-13C HSQC Spectrum
600 MHz for 1H nuclei, D2O
Manα
Manβ
GlcNAcβ
Galβ
f2 (ppm)
f1 (ppm)

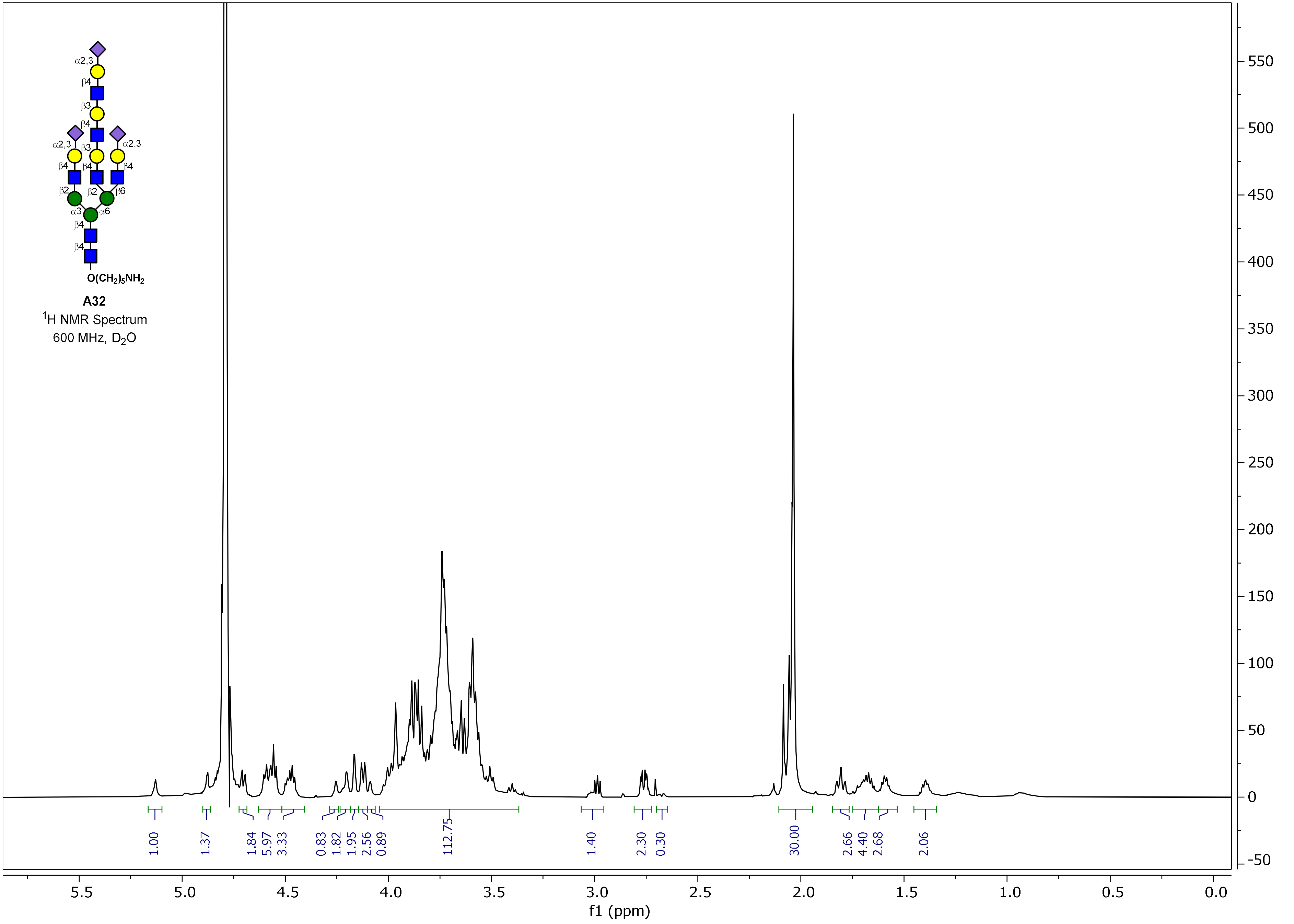
O(CH2)5NH2
A32
1H NMR Spectrum
600 MHz, D2O
α2,3
β4
β3
β2
β6
α3
α6
1.00
1.37
1.84
5.97
3.33
0.83
1.82
1.95
2.56
0.89
112.75
1.40
2.30
0.30
30.00
2.66
4.40
2.68
2.06
f1 (ppm)

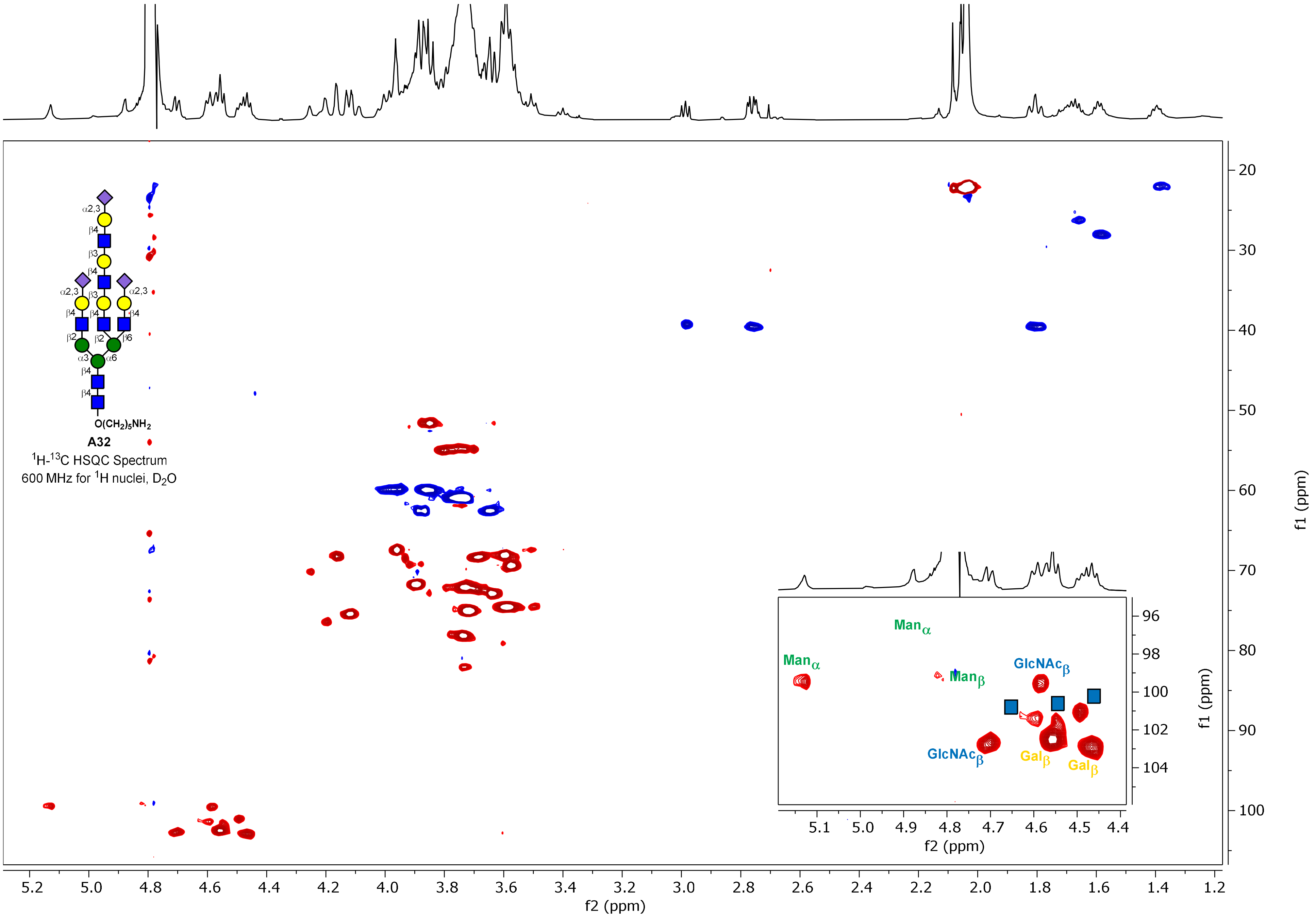
α2,3
β4
β3
β4
α2,3
β3
α2,3
β4
β4
β4
β2
β2
β6
α3
α6
β4
β4
O(CH2)5NH2
A32
1H-13C HSQC Spectrum
600 MHz for 1H nuclei, D2O
Manα
Manα
Manβ
GlcNAcβ
GlcNAcβ
Galβ
Galβ
f2 (ppm)
f1 (ppm)
f2 (ppm)
f1 (ppm)

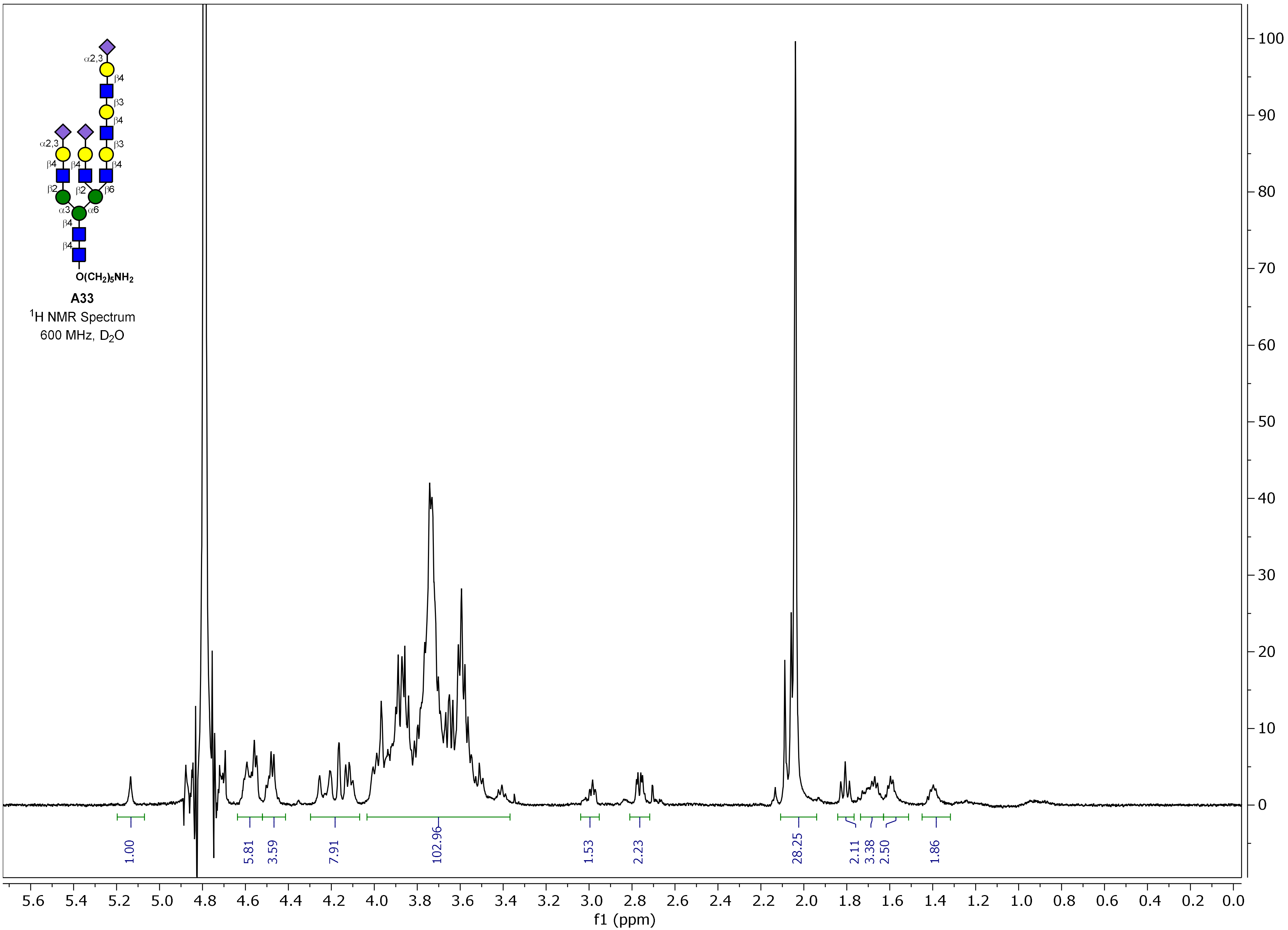

α2,3
β4
β3
β4
β3
β4
α2,3
β4
β4
β2
β2
β6
α3
α6
β4
β4
$O(CH_2)_5NH_2$
A33
^1H NMR Spectrum
600 MHz, D_2O
1.00
5.81
3.59
7.91
102.96
1.53
2.23
28.25
2.11
3.38
2.50
1.86
f1 (ppm)

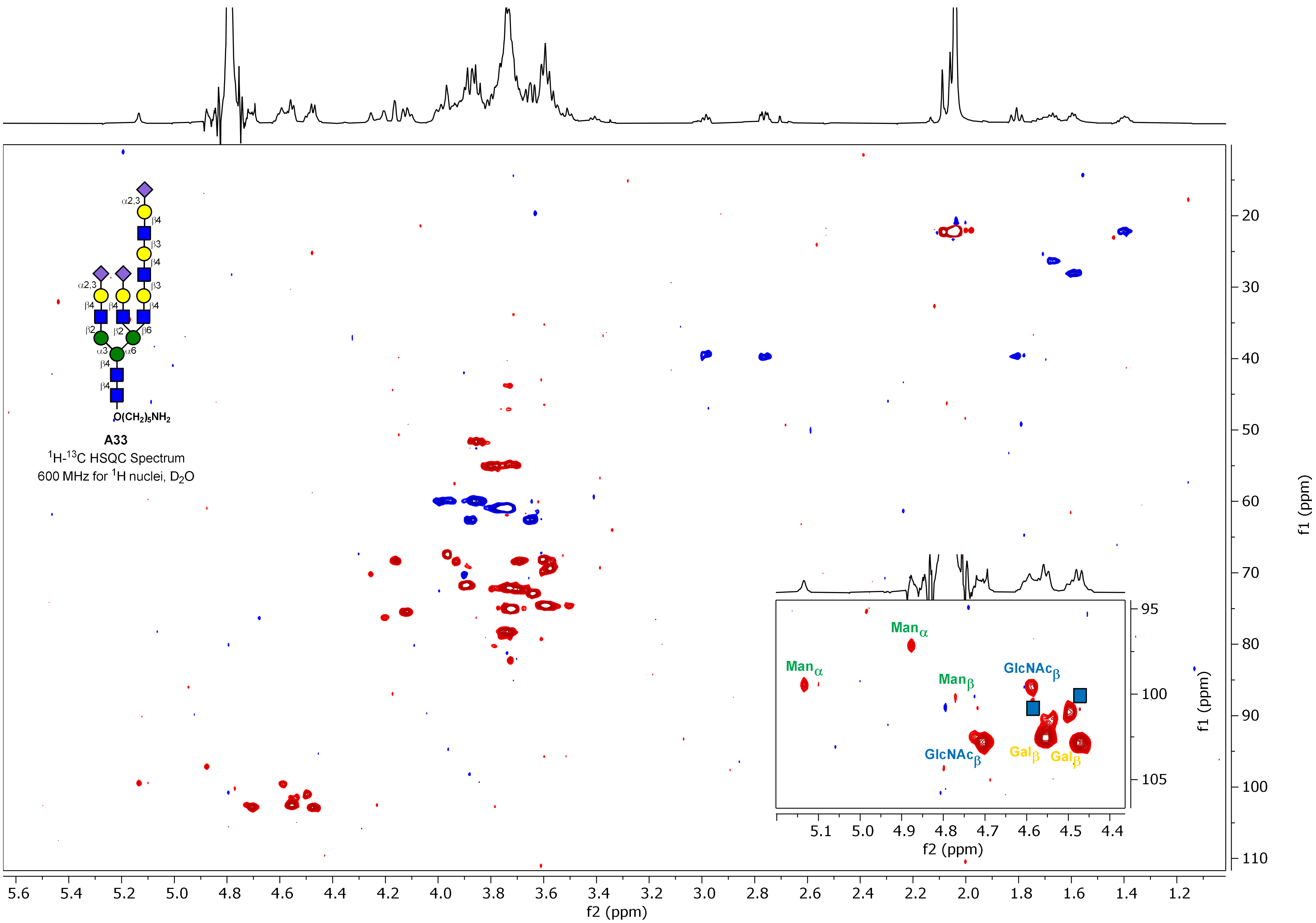

A33
^{1}H-^{13}C HSQC Spectrum
600 MHz for ^{1}H nuclei, D_2O
O(CH2)5NH2
α2,3
β4
β3
β2
β6
α3
α6
Manα
Manβ
GlcNAcβ
Galβ
f2 (ppm)
f1 (ppm)

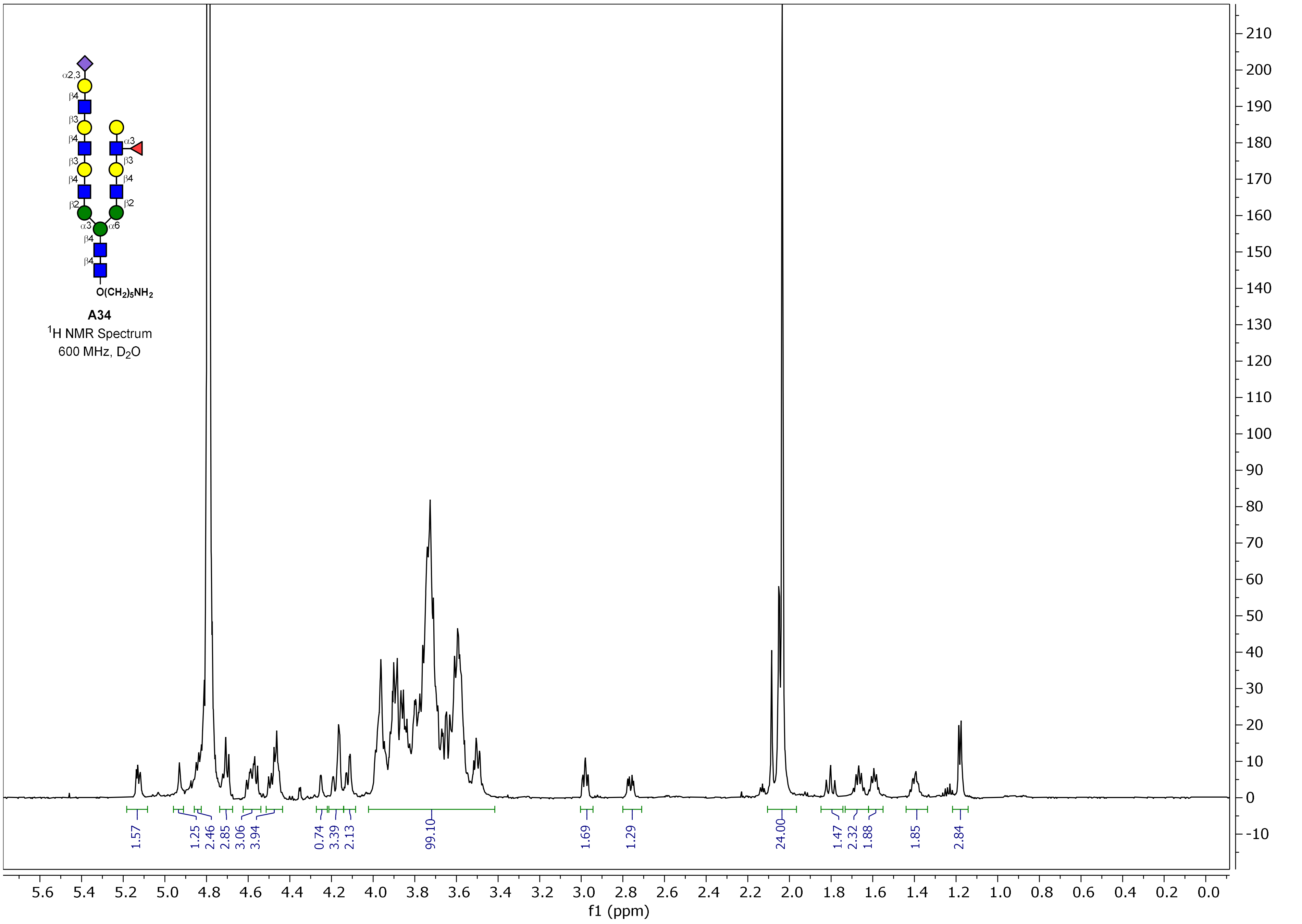
O(CH2)5NH2
A34
1H NMR Spectrum
600 MHz, D2O
f1 (ppm)

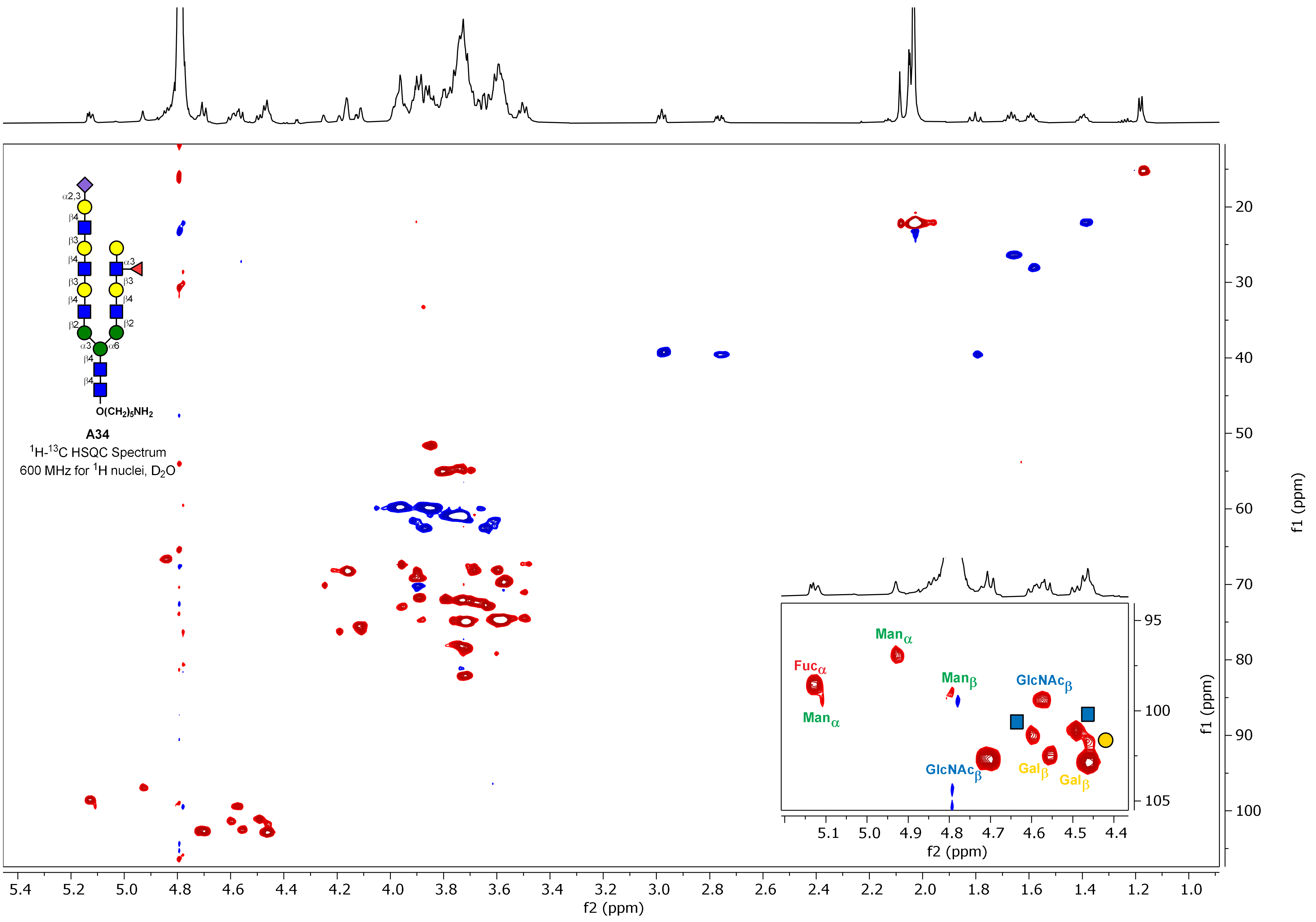
A34
$^{1}H-^{13}C$ HSQC Spectrum
600 MHz for ^{1}H nuclei, D_2O
O(CH2)5NH2
α2,3
β4
β3
α3
β2
α6
Fucα
Manα
Manβ
GlcNAcβ
Galβ
f2 (ppm)
f1 (ppm)

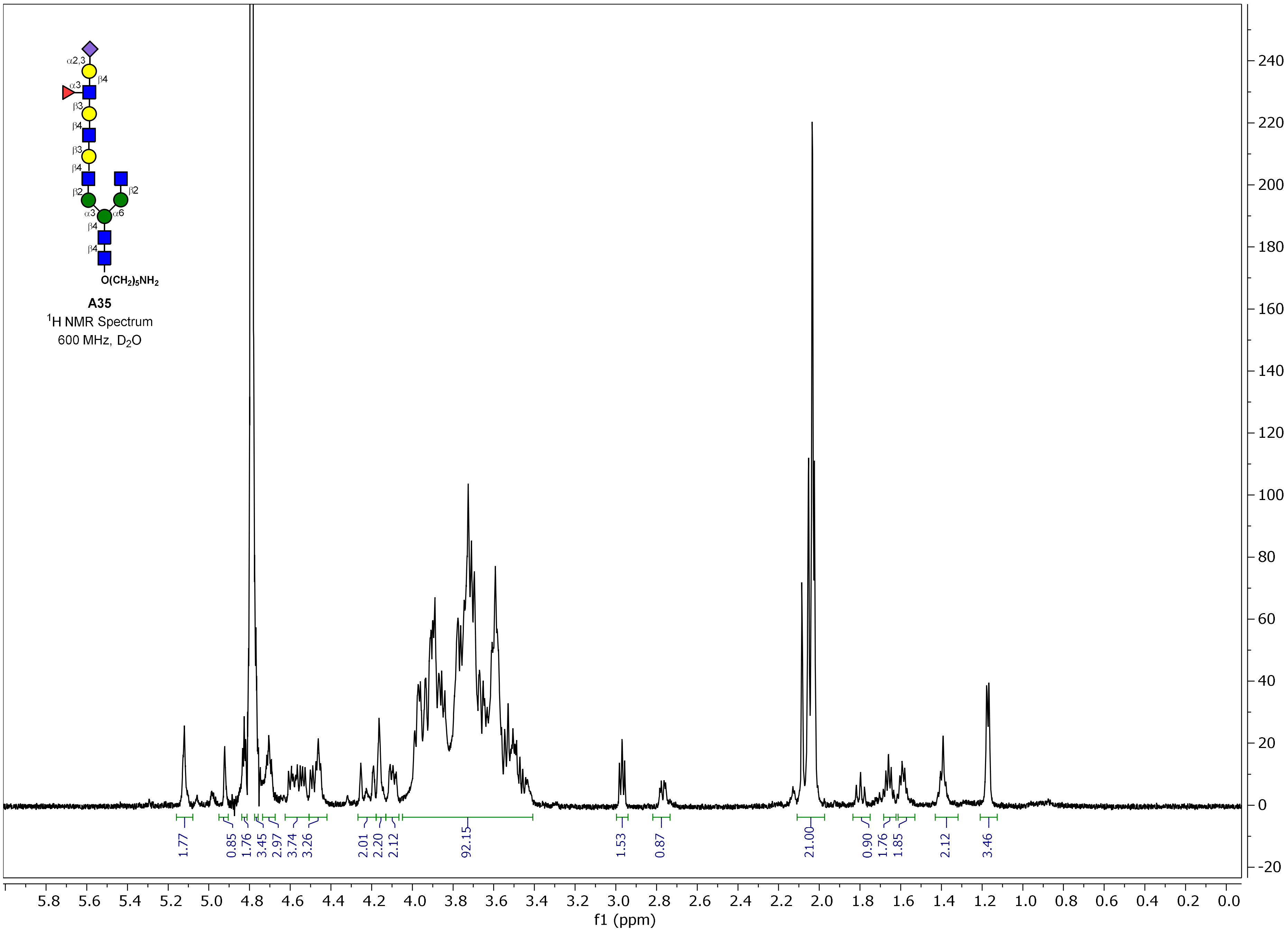

α2,3
β4
α3
β3
β4
β3
β4
β2
β2
α3
α6
β4
β4
O(CH2)5NH2
A35
1H NMR Spectrum
600 MHz, D2O
1.77
0.85
1.76
3.45
2.97
3.74
3.26
2.01
2.20
2.12
92.15
1.53
0.87
21.00
0.90
1.76
1.85
2.12
3.46
f1 (ppm)

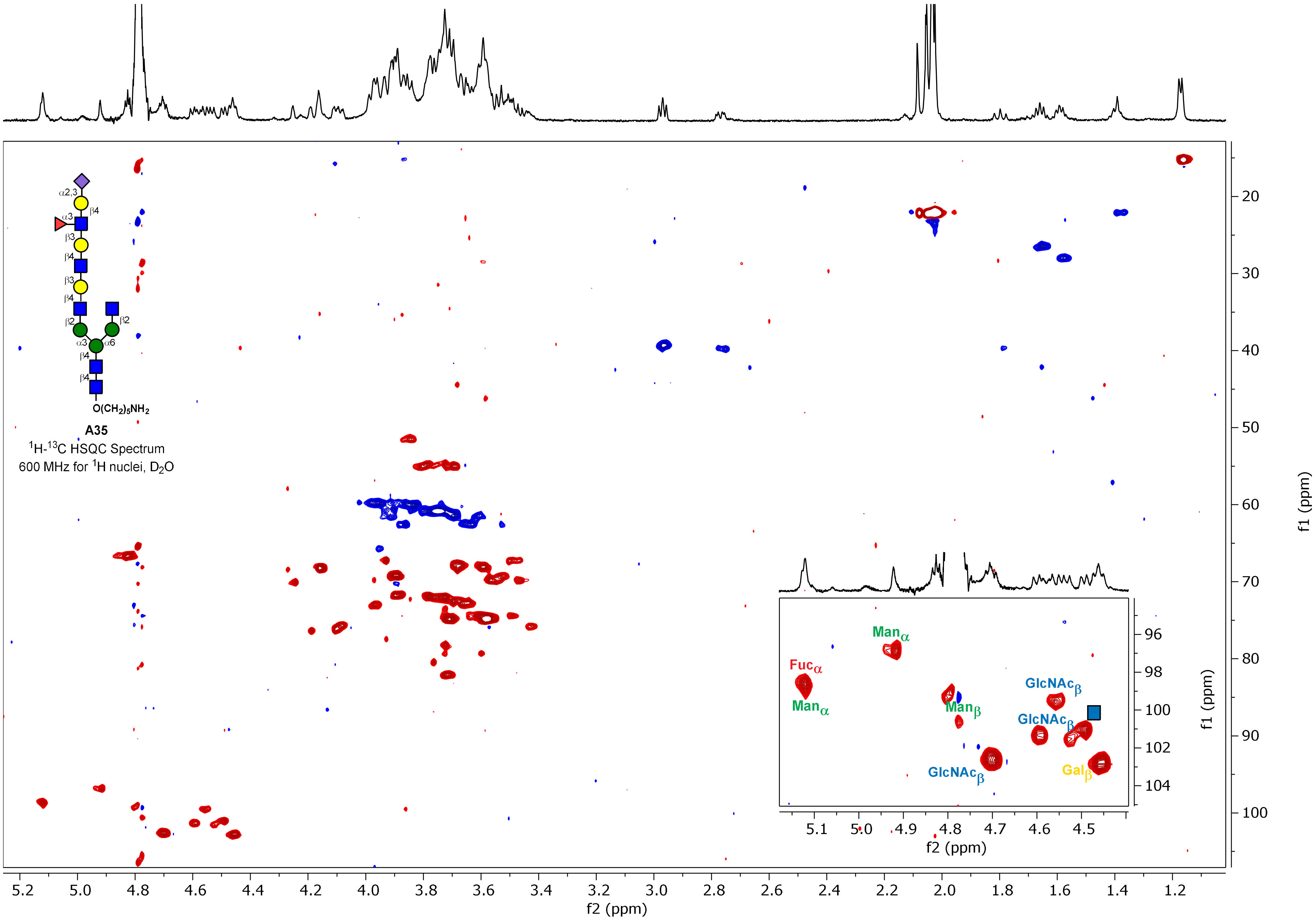

A35
^{1}H-^{13}C HSQC Spectrum
600 MHz for ^{1}H nuclei, D_2O
O(CH2)5NH2
Fucα
Manα
Manα
Manβ
GlcNAcβ
GlcNAcβ
GlcNAcβ
Galβ
f2 (ppm)
f1 (ppm)

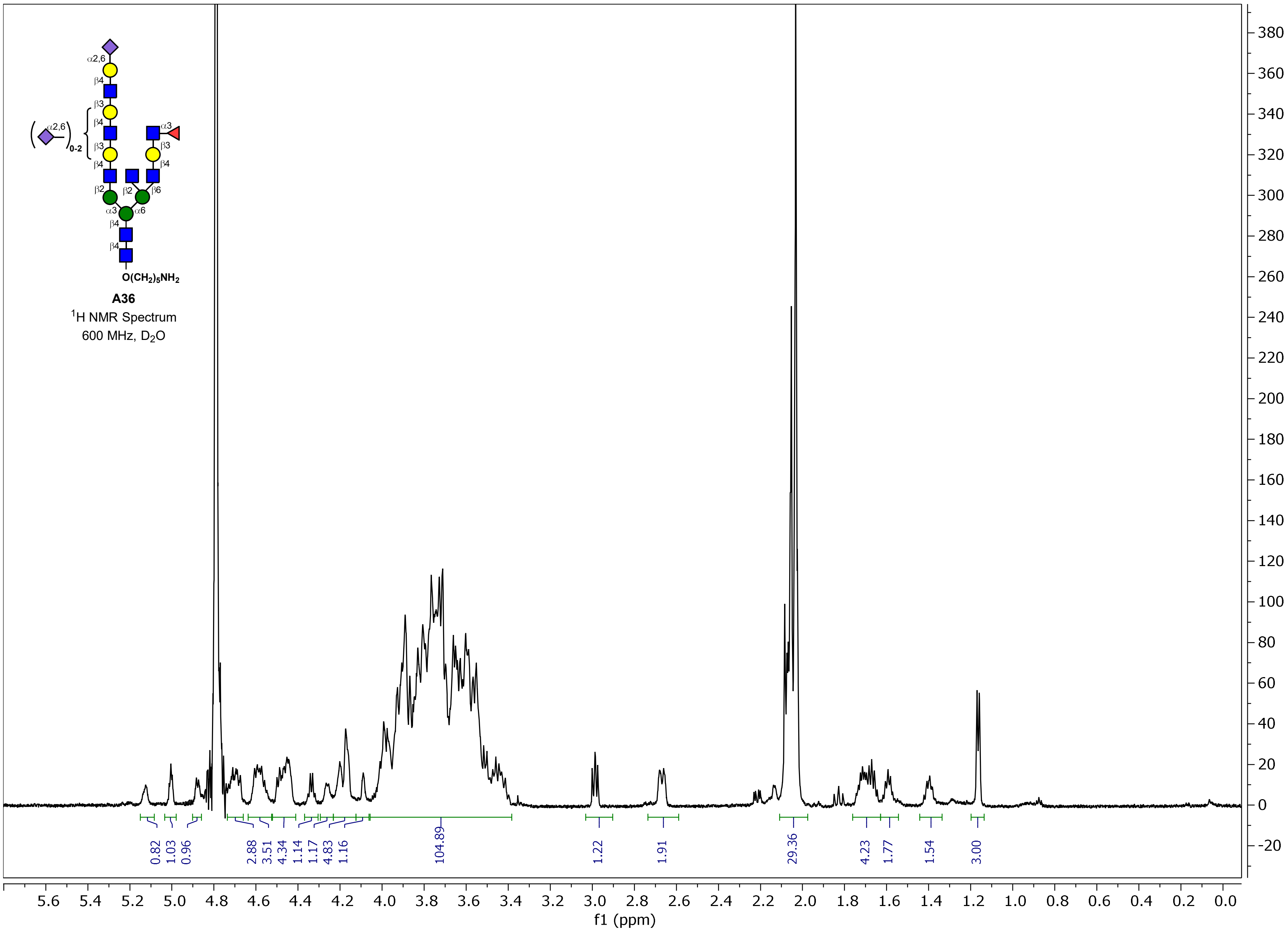
α2,6
β4
β3
β4
β3
β4
β2
α3
β4
β4
α2,6
0-2
α3
β3
β4
β2
β6
α6
O(CH2)5NH2
A36
1H NMR Spectrum
600 MHz, D2O
0.82
1.03
0.96
2.88
3.51
4.34
1.14
1.17
4.83
1.16
104.89
1.22
1.91
29.36
4.23
1.77
1.54
3.00
f1 (ppm)

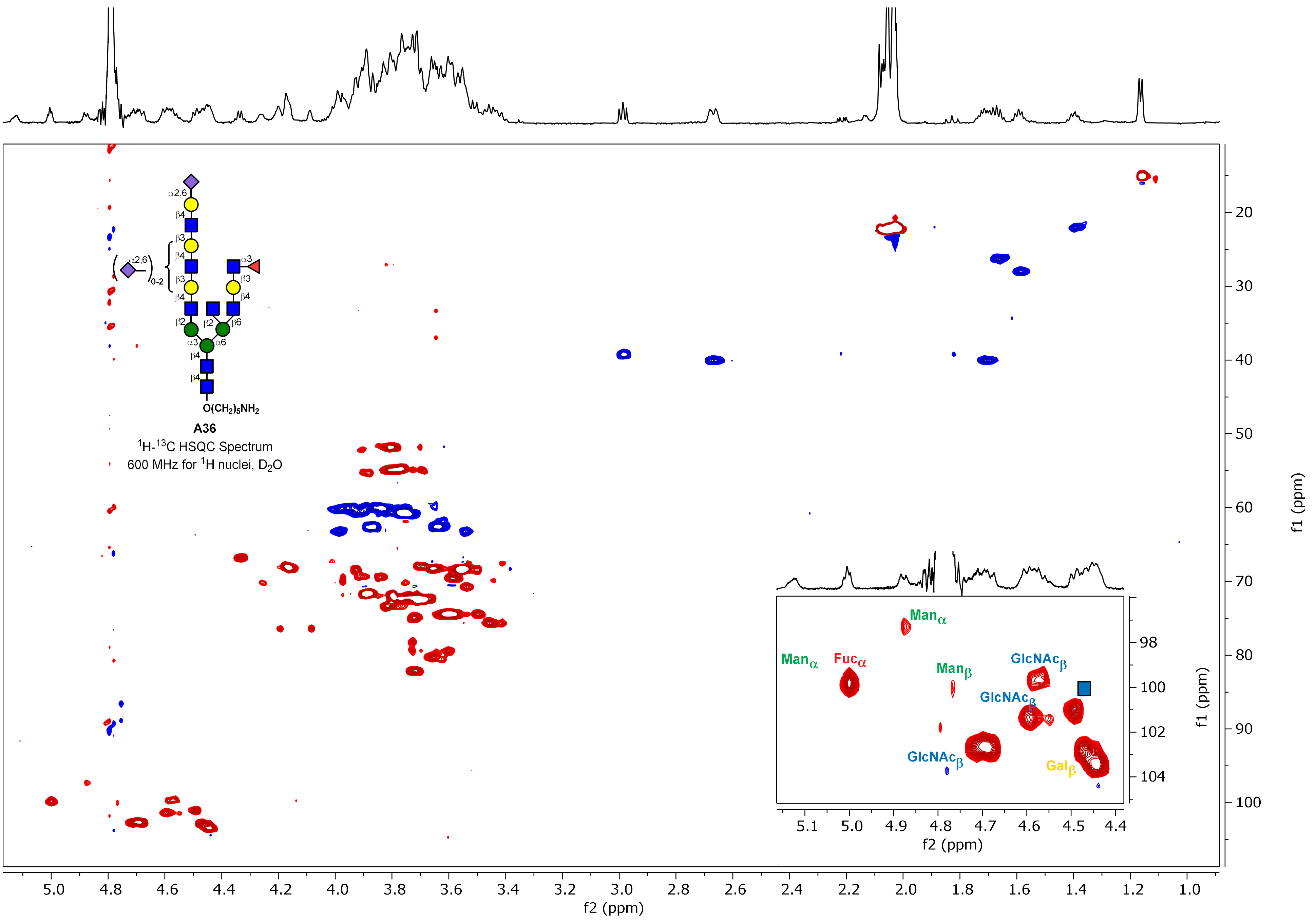

α2,6
β4
β3
β4
β3
β4
β2
α3
α6
β4
β4
β6
β2
(α2,6)0-2
α3
β3
β4
O(CH2)5NH2
A36
1H-13C HSQC Spectrum
600 MHz for 1H nuclei, D2O
Manα
Manα
Fucα
Manβ
GlcNAcβ
GlcNAcβ
GlcNAcβ
Galβ
f2 (ppm)
f1 (ppm)

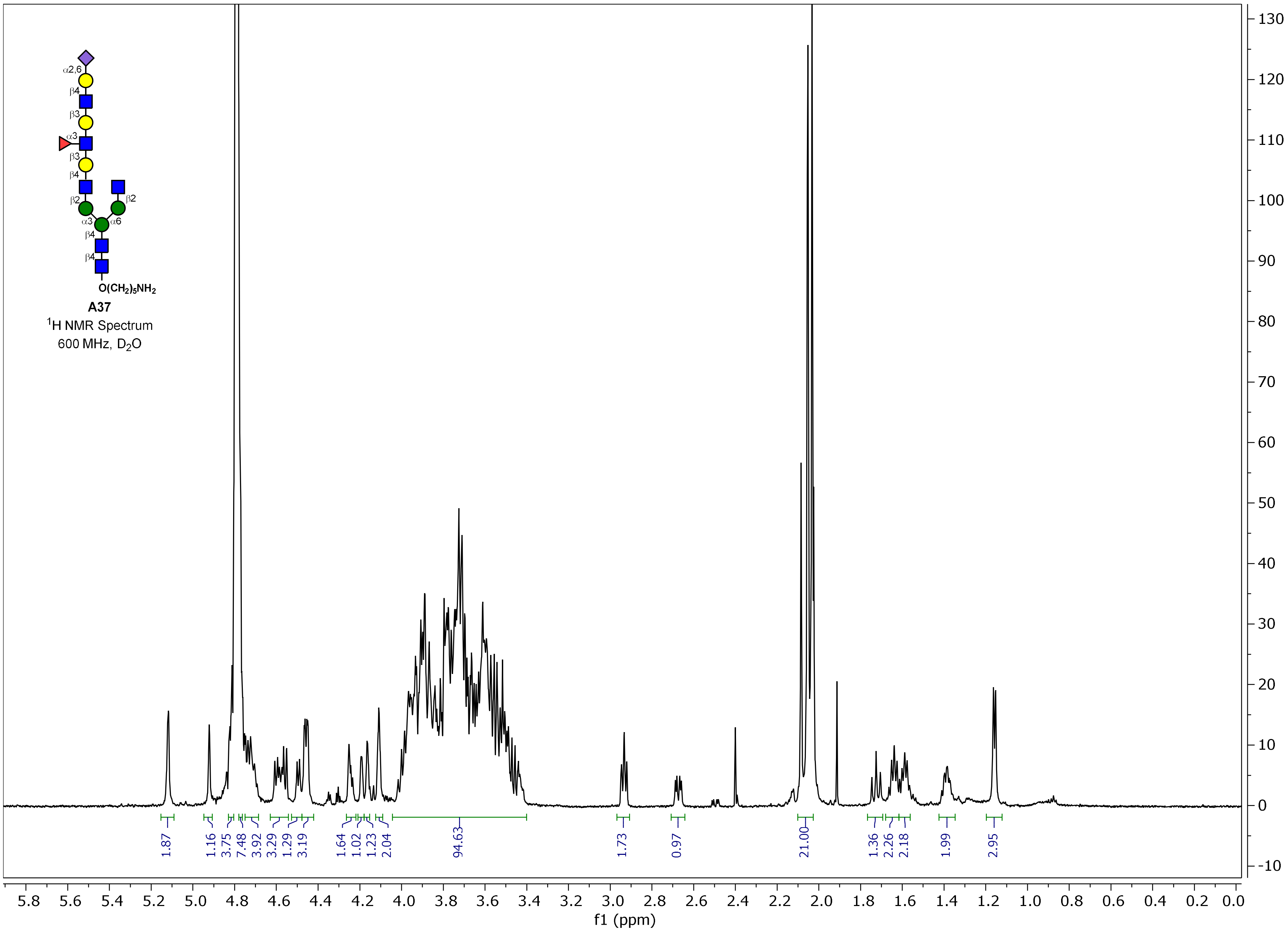

α2,6
β4
β3
α3
β3
β4
β2
β2
α3
α6
β4
β4
O(CH2)5NH2
A37
1H NMR Spectrum
600 MHz, D2O
1.87
1.16
3.75
7.48
3.92
3.29
1.29
3.19
1.64
1.02
1.23
2.04
94.63
1.73
0.97
21.00
1.36
2.26
2.18
1.99
2.95
f1 (ppm)

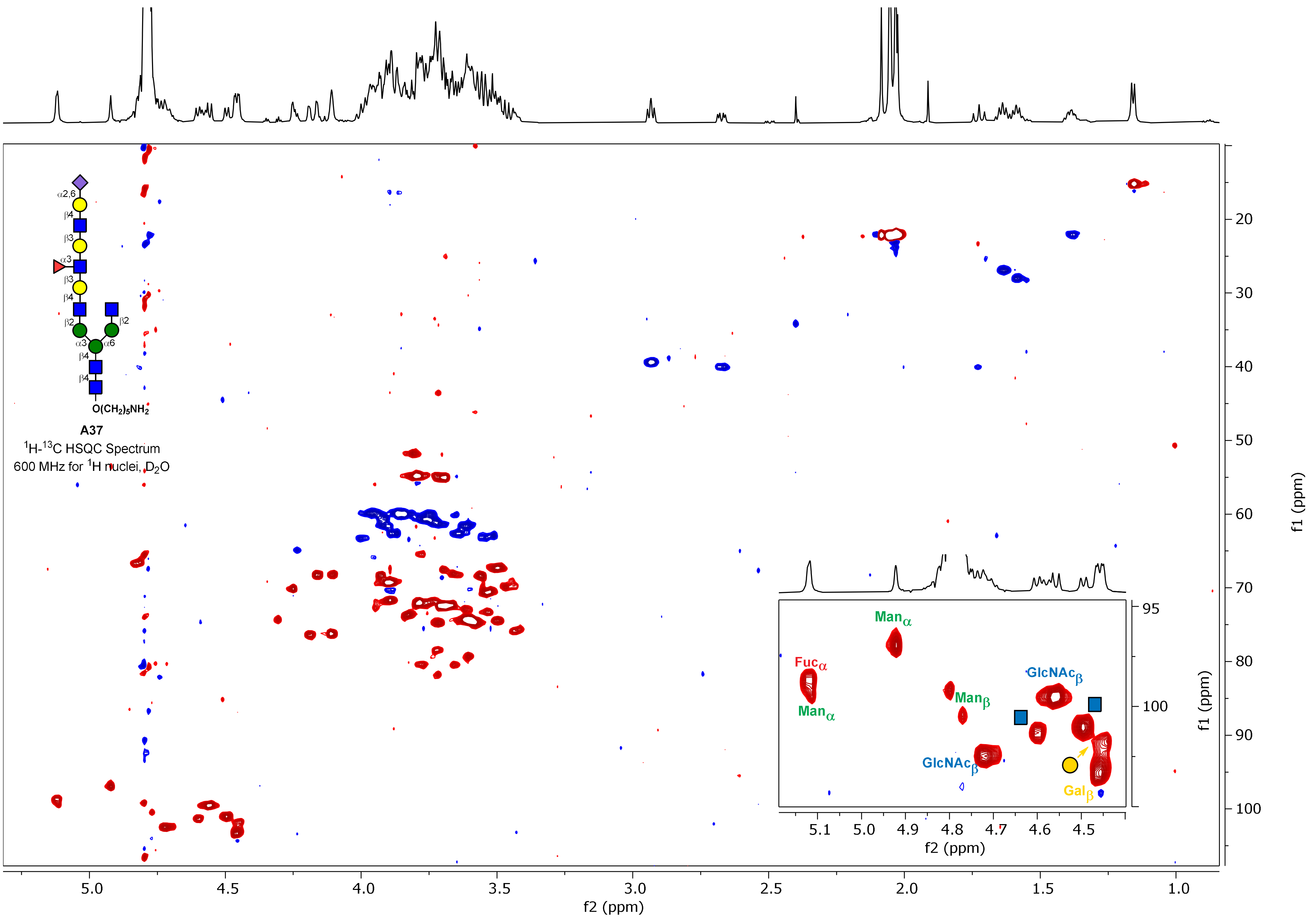

α2,6
β4
β3
α3
β3
β4
β2
β2
α3
α6
β4
β4
O(CH2)5NH2
A37
1H-13C HSQC Spectrum
600 MHz for 1H nuclei, D2O
Manα
Fucα
Manα
GlcNAcβ
Manβ
GlcNAcβ
Galβ
f2 (ppm)
f1 (ppm)

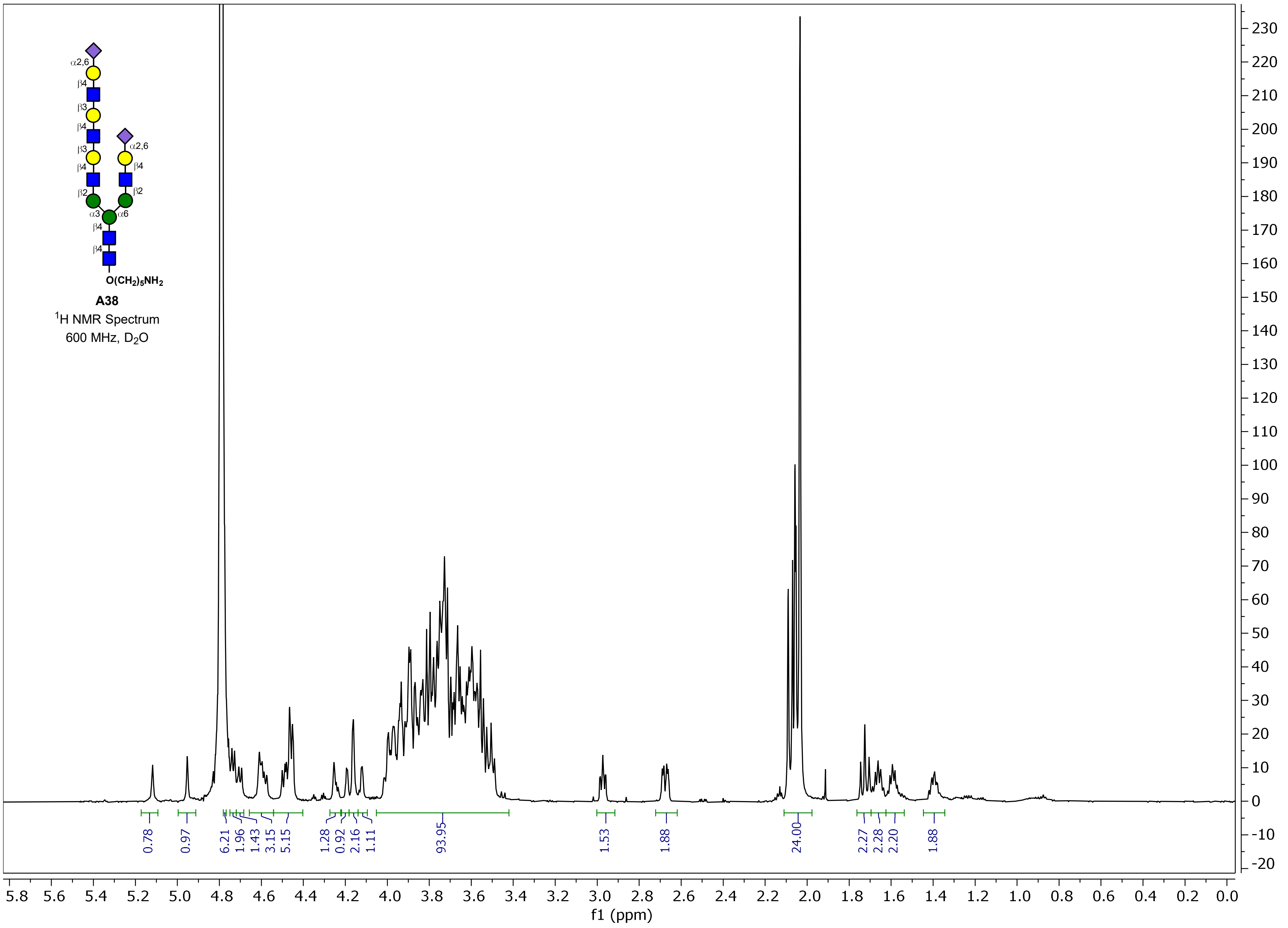

α2,6
β4
β3
β4
β3
β4
β2
α3
α6
β4
β4
α2,6
β4
β2
O(CH2)5NH2
A38
1H NMR Spectrum
600 MHz, D2O
0.78
0.97
6.21
1.96
1.43
3.15
5.15
1.28
0.92
2.16
1.11
93.95
1.53
1.88
24.00
2.27
2.28
2.20
1.88
f1 (ppm)

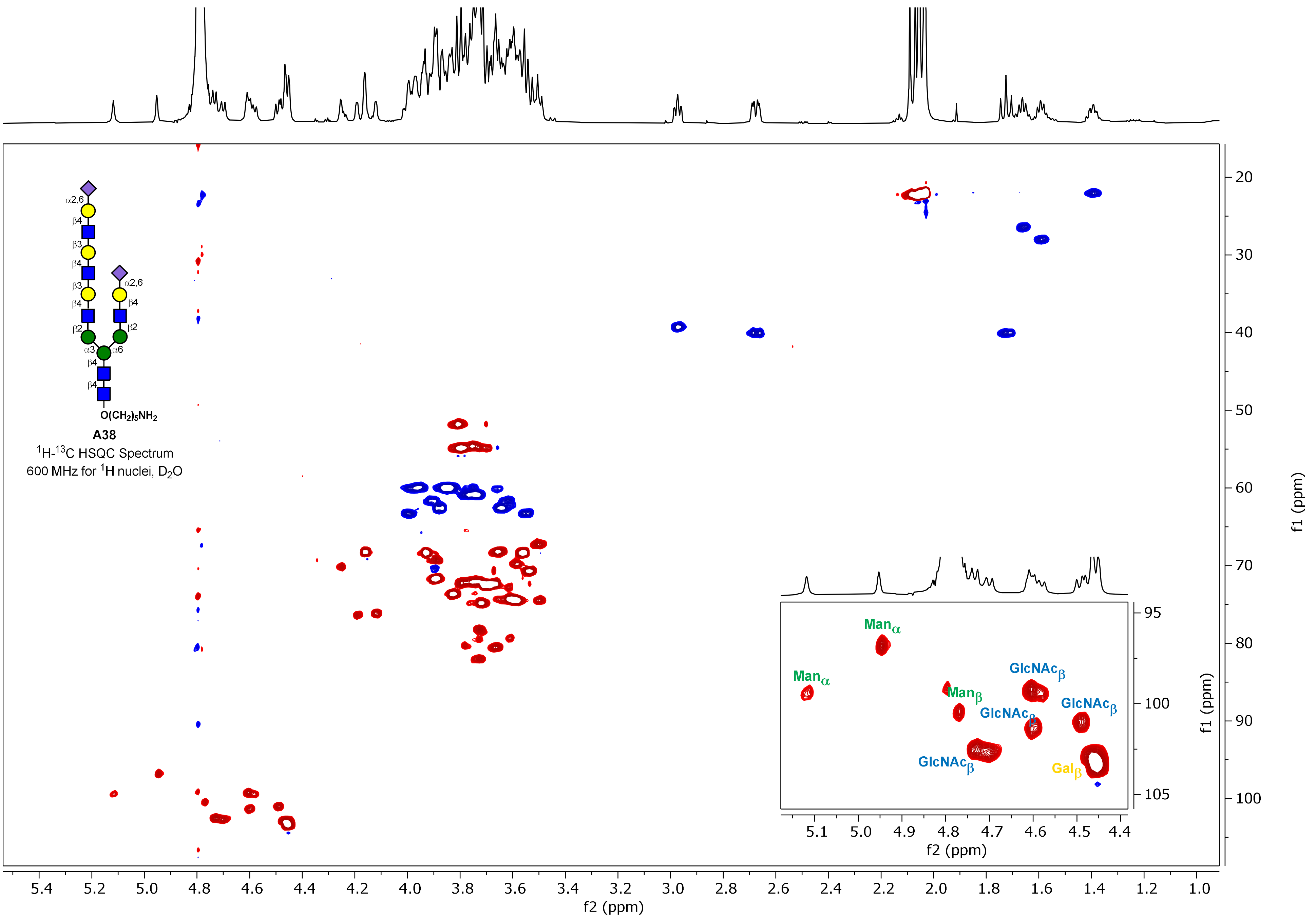
O(CH2)5NH2
A38
1H-13C HSQC Spectrum
600 MHz for 1H nuclei, D2O
Manα
Manα
Manβ
GlcNAcβ
GlcNAcβ
GlcNAcβ
GlcNAcβ
Galβ
f2 (ppm)
f1 (ppm)

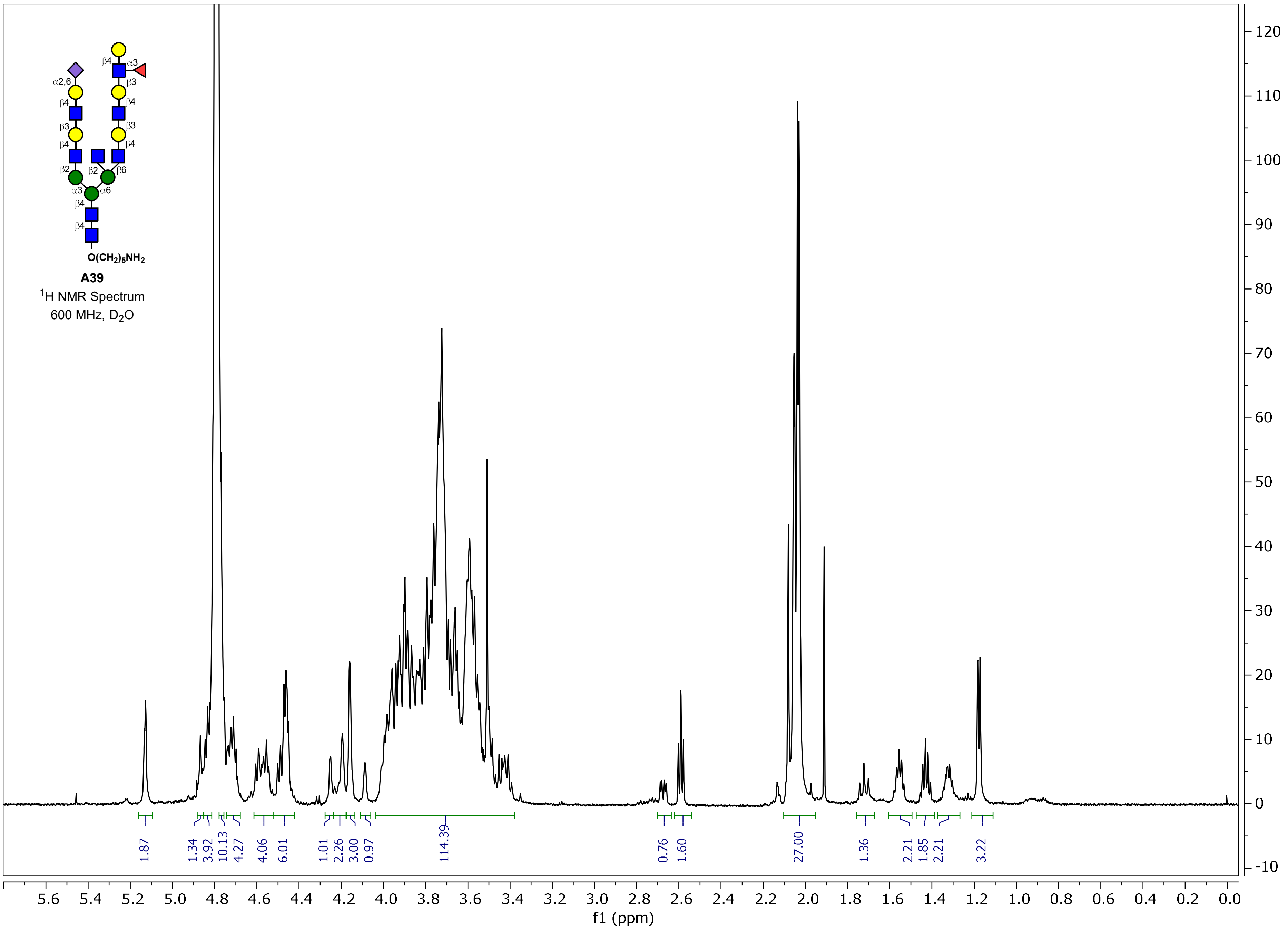
A39
^{1}H NMR Spectrum
600 MHz, D_2O
O(CH2)5NH2
f1 (ppm)

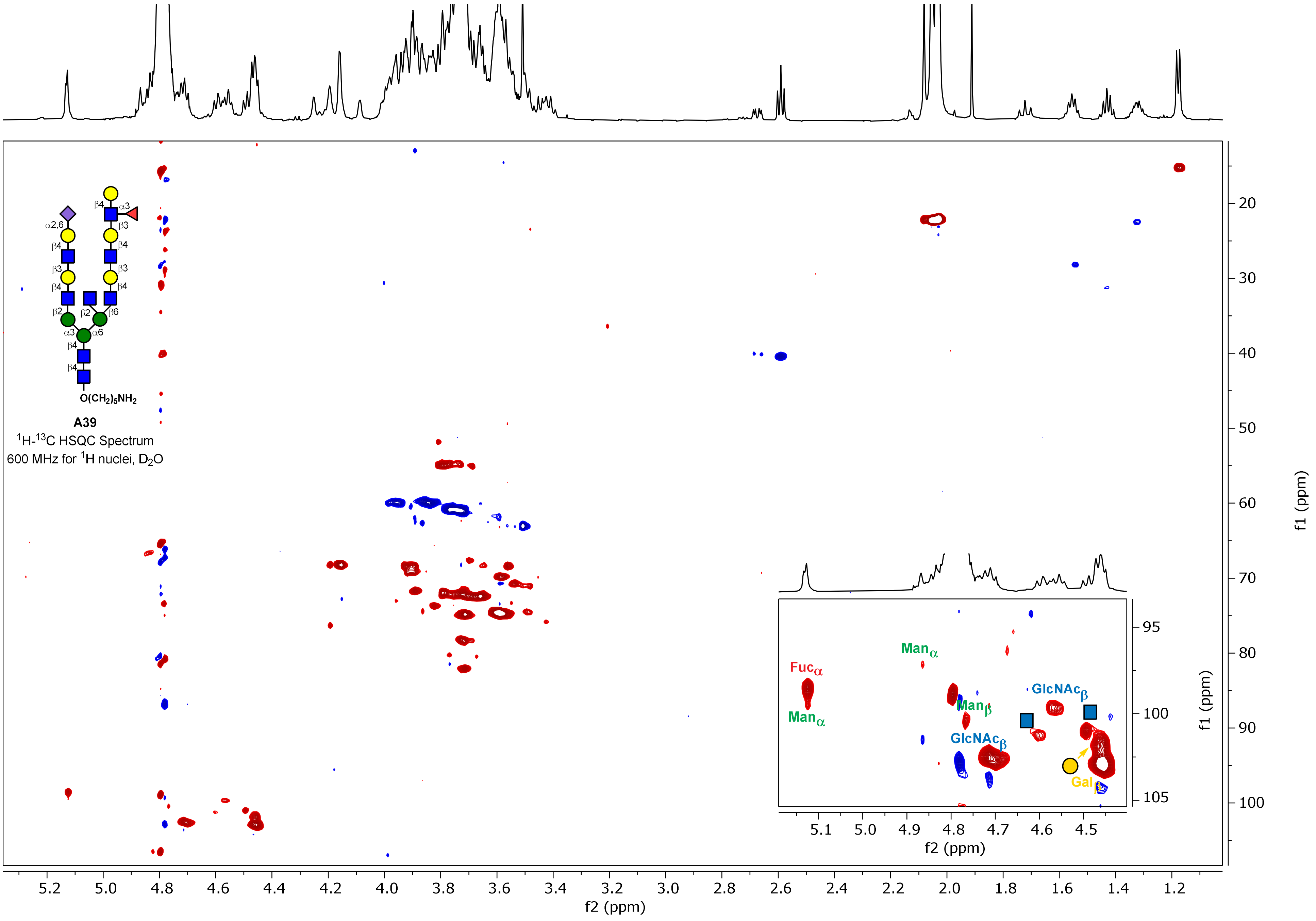
α2,6
β4
β3
β4
β2
β2
β6
α3
α6
β4
β4
α3
O(CH2)5NH2
A39
1H-13C HSQC Spectrum
600 MHz for 1H nuclei, D2O
Fucα
Manα
Manα
Manβ
GlcNAcβ
GlcNAcβ
Galβ
f2 (ppm)
f1 (ppm)

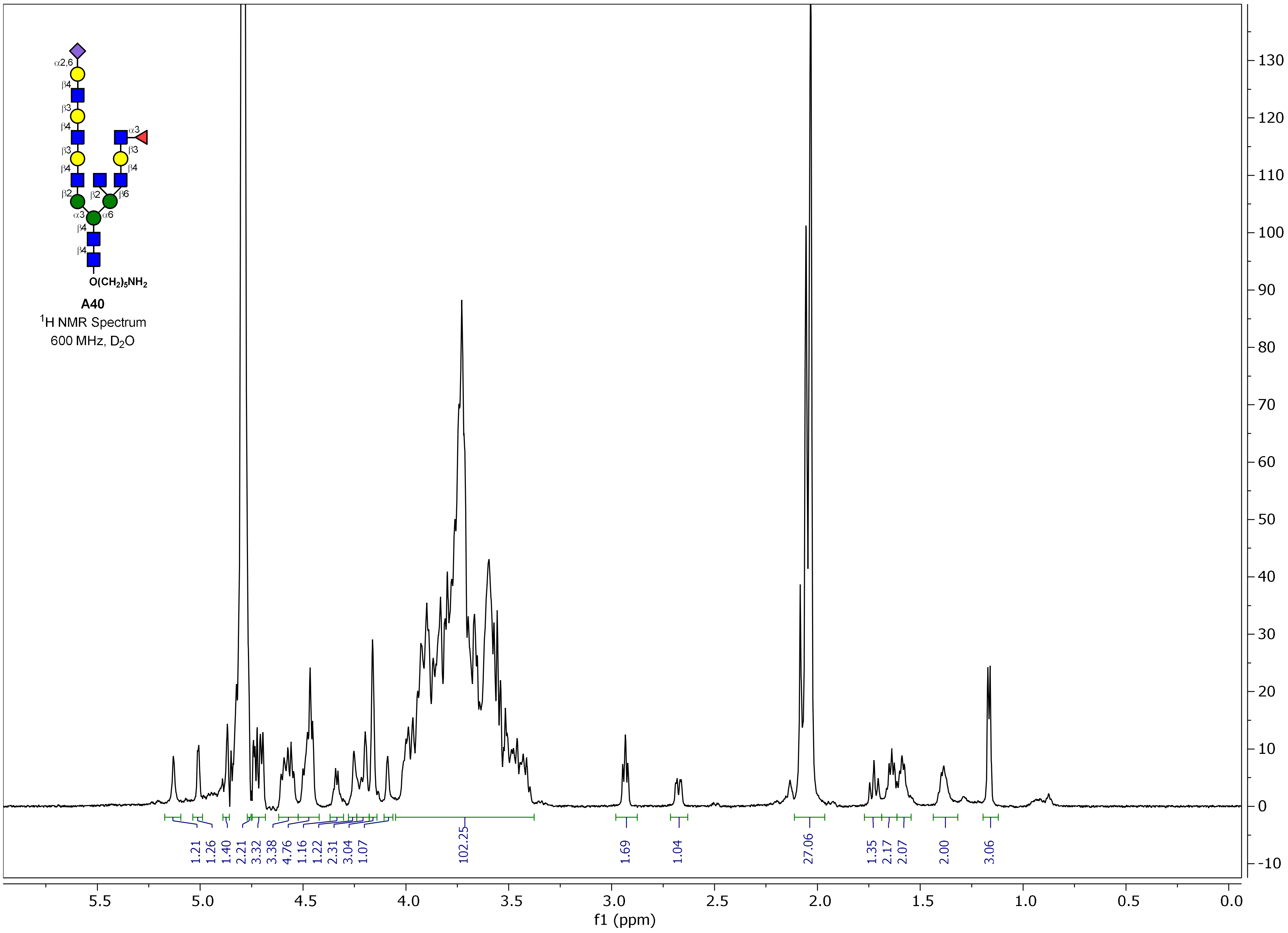
O(CH2)5NH2
A40
1H NMR Spectrum
600 MHz, D2O
f1 (ppm)

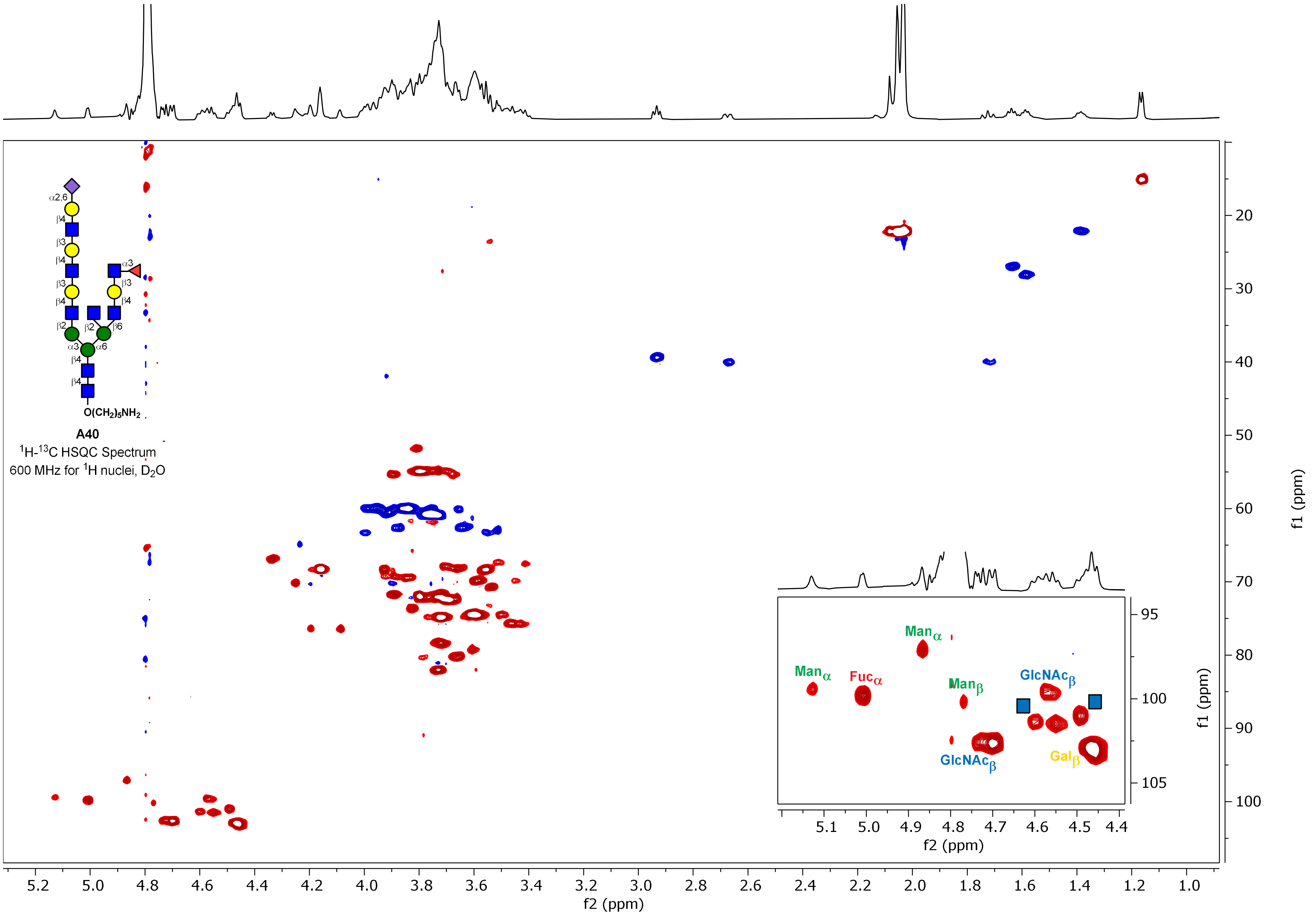

α2,6
β4
β3
β4
β3
β4
β2
β2
α3
β3
β4
β6
α3
α6
β4
β4
O(CH2)5NH2
A40
1H-13C HSQC Spectrum
600 MHz for 1H nuclei, D2O
Manα
Manα
Fucα
Manβ
GlcNAcβ
GlcNAcβ
Galβ
f2 (ppm)
f1 (ppm)
f2 (ppm)
f1 (ppm)

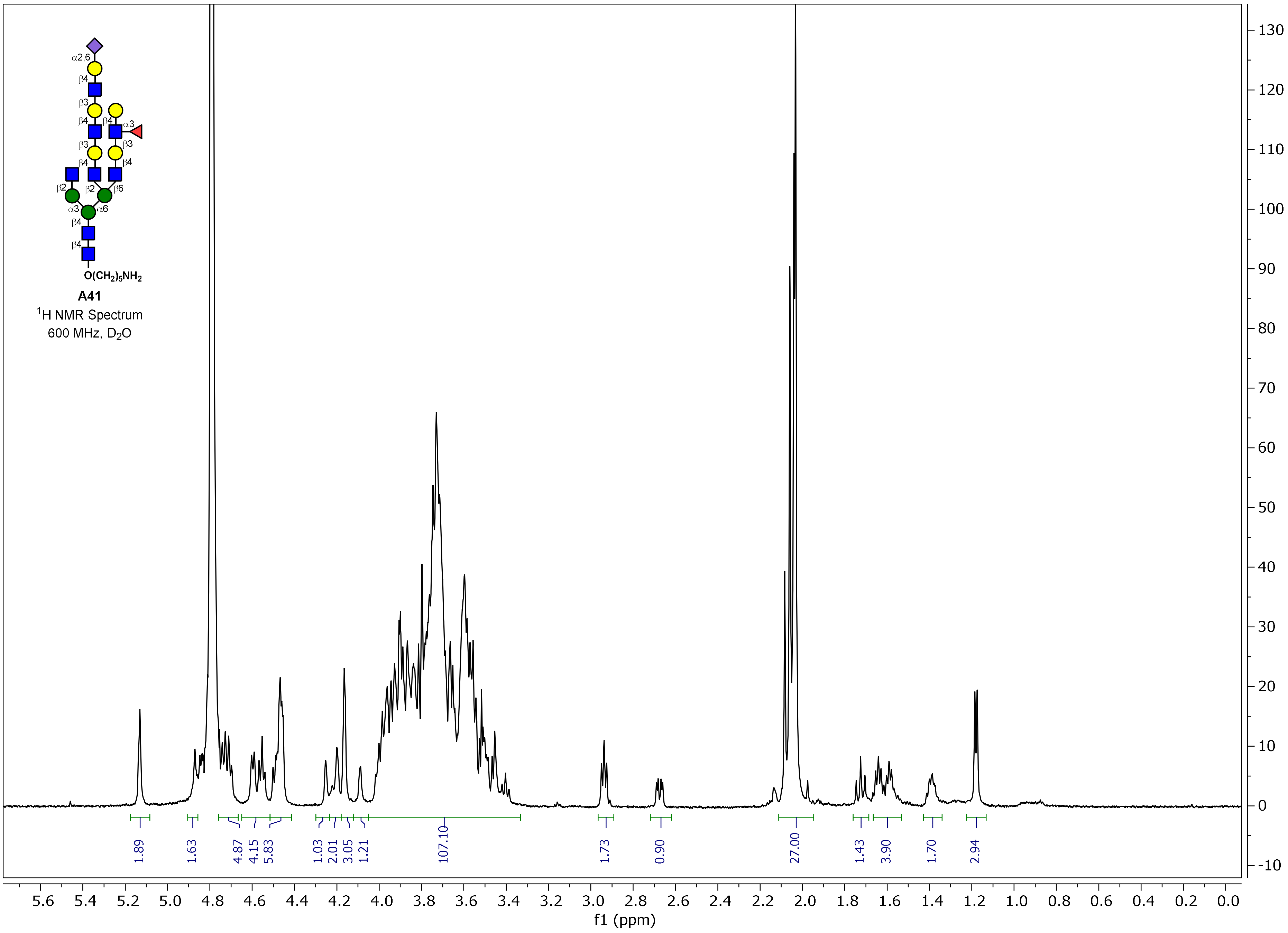

α2,6
β4
β3
β4
β4
α3
β3
β3
β4
β4
β2
β2
β6
α3
α6
β4
β4
O(CH2)5NH2
A41
1H NMR Spectrum
600 MHz, D2O
1.89
1.63
4.87
4.15
5.83
1.03
2.01
3.05
1.21
107.10
1.73
0.90
27.00
1.43
3.90
1.70
2.94
f1 (ppm)

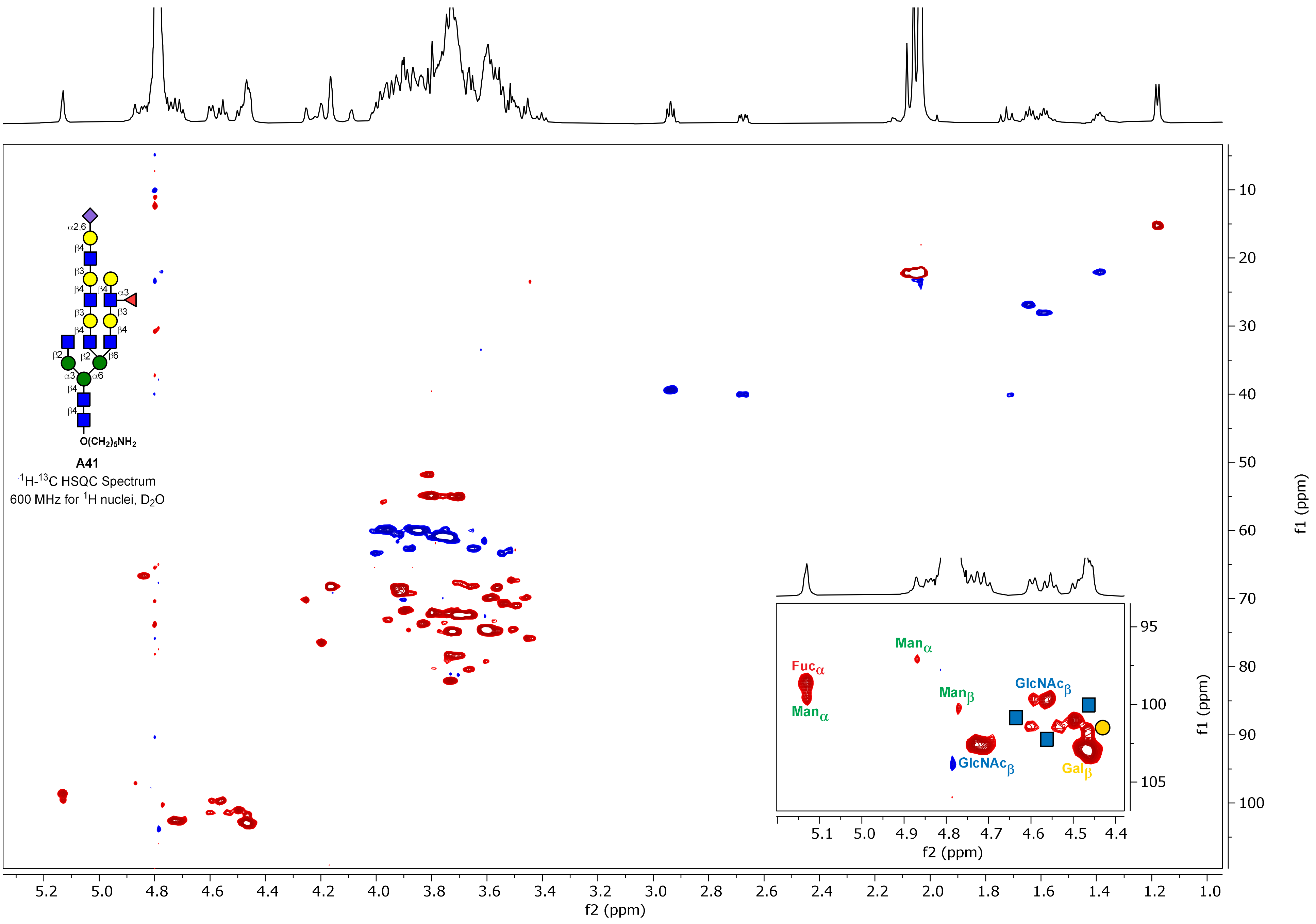

α2,6
β4
β3
β4
β4
α3
β3
β3
β4
β4
β2
β2
β6
α3
α6
β4
β4
O(CH2)5NH2
A41
1H-13C HSQC Spectrum
600 MHz for 1H nuclei, D2O
Fucα
Manα
Manα
Manβ
GlcNAcβ
GlcNAcβ
Galβ
f2 (ppm)
f1 (ppm)
f2 (ppm)
f1 (ppm)

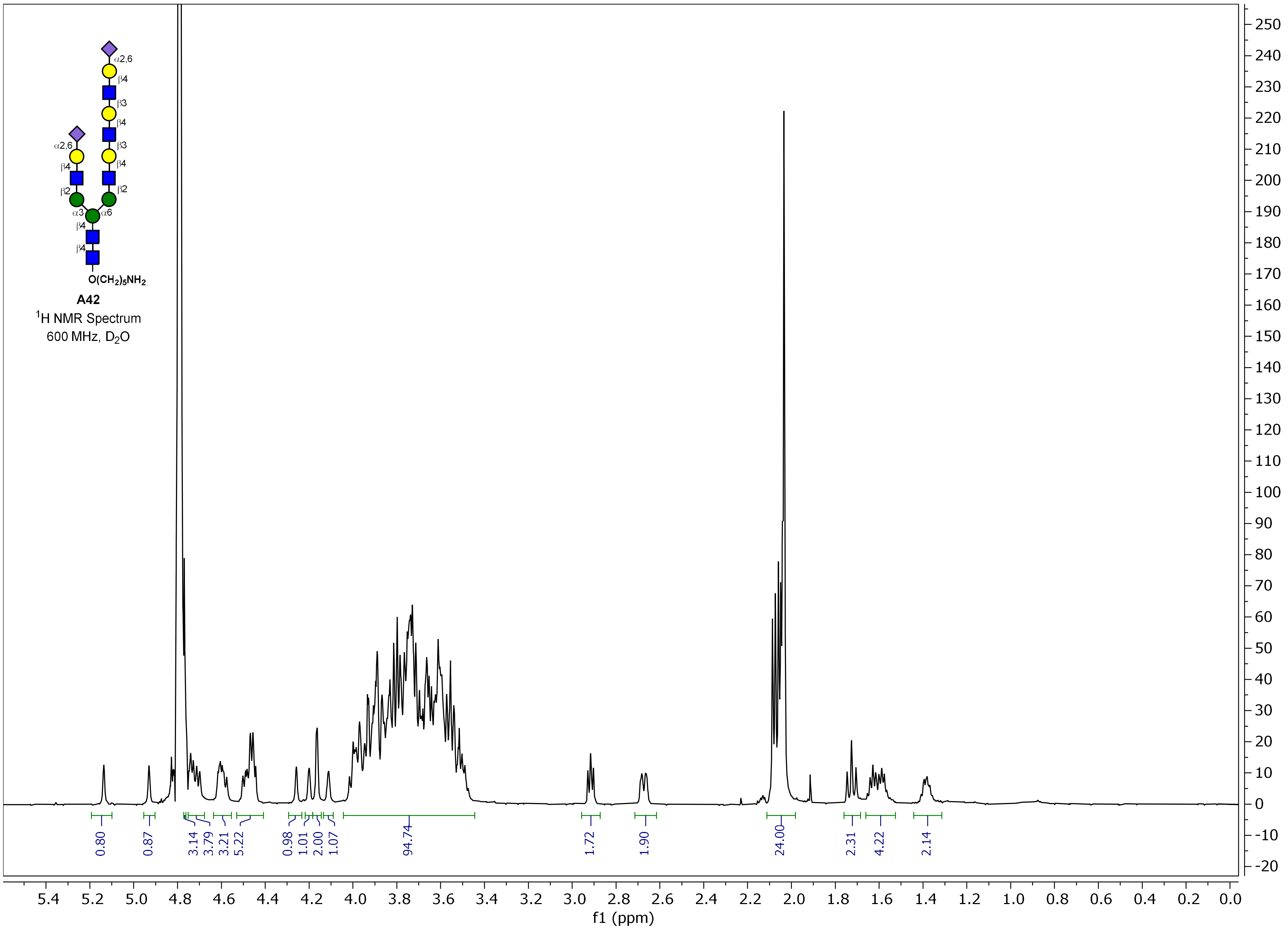
α2,6
β4
β3
β2
α3
α6
O(CH2)5NH2
A42
1H NMR Spectrum
600 MHz, D2O
0.80
0.87
3.14
3.79
3.21
5.22
0.98
1.01
2.00
1.07
94.74
1.72
1.90
24.00
2.31
4.22
2.14
f1 (ppm)

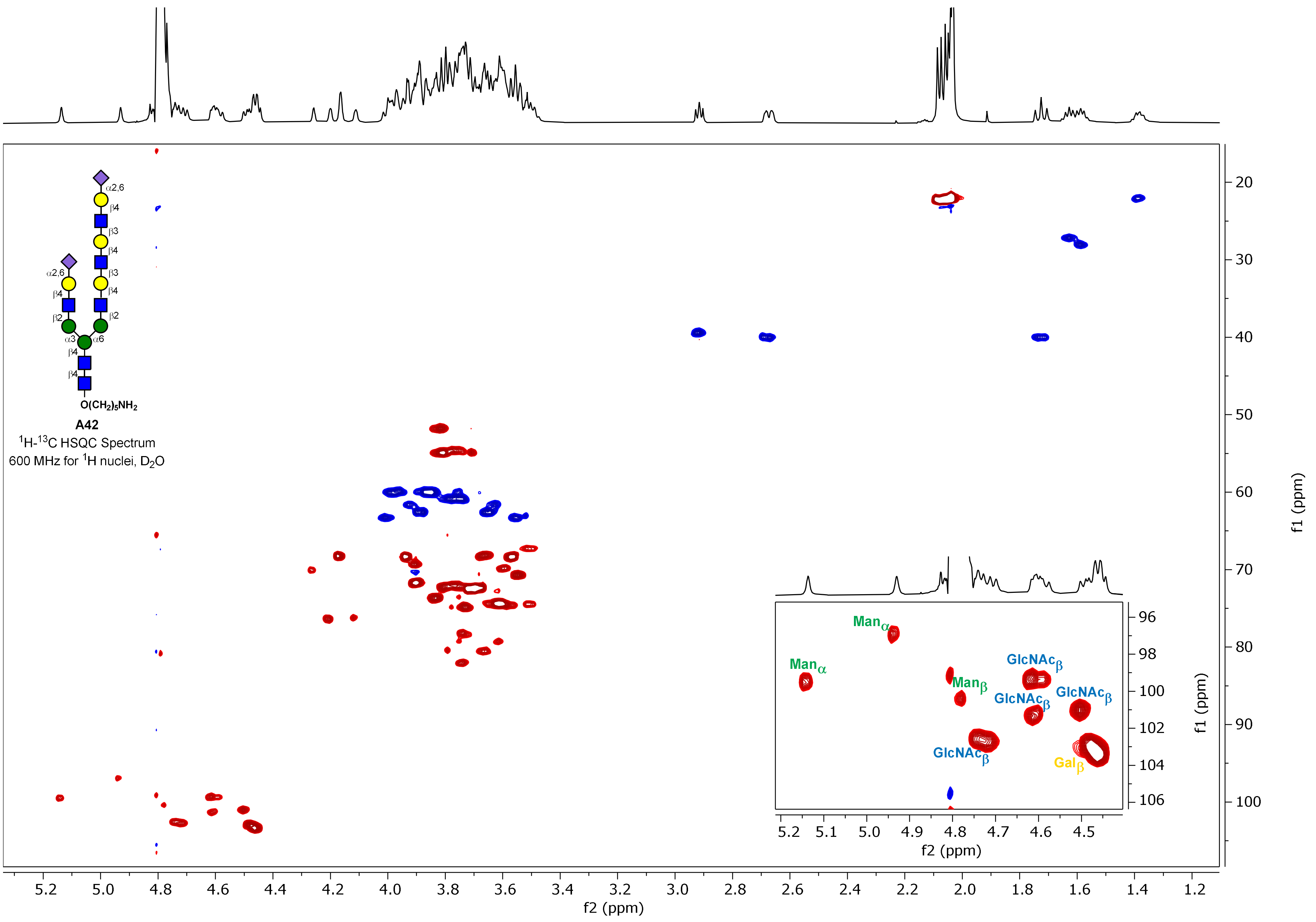
α2,6
β4
β3
β4
β3
β4
β2
α2,6
β4
β2
α3
α6
β4
β4
O(CH2)5NH2
A42
1H-13C HSQC Spectrum
600 MHz for 1H nuclei, D2O
Manα
Manα
GlcNAcβ
Manβ
GlcNAcβ
GlcNAcβ
GlcNAcβ
Galβ
f2 (ppm)
f1 (ppm)
f2 (ppm)
f1 (ppm)

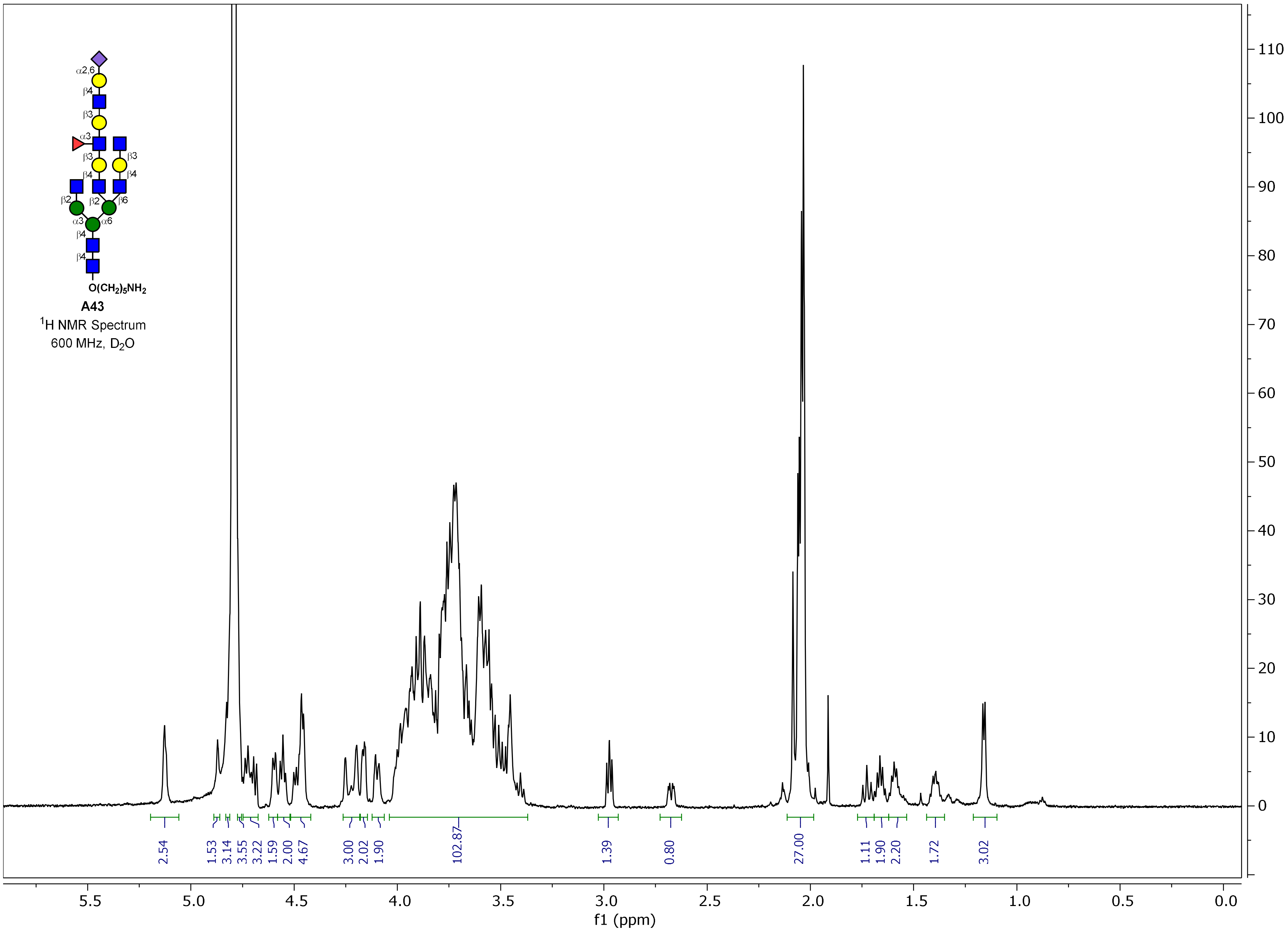

α2,6
β4
β3
α3
β3
β3
β4
β4
β2
β2
β6
α3
α6
β4
β4
O(CH2)5NH2
A43
1H NMR Spectrum
600 MHz, D2O
2.54
1.53
3.14
3.55
3.22
1.59
2.00
4.67
3.00
2.02
1.90
102.87
1.39
0.80
27.00
1.11
1.90
2.20
1.72
3.02
f1 (ppm)

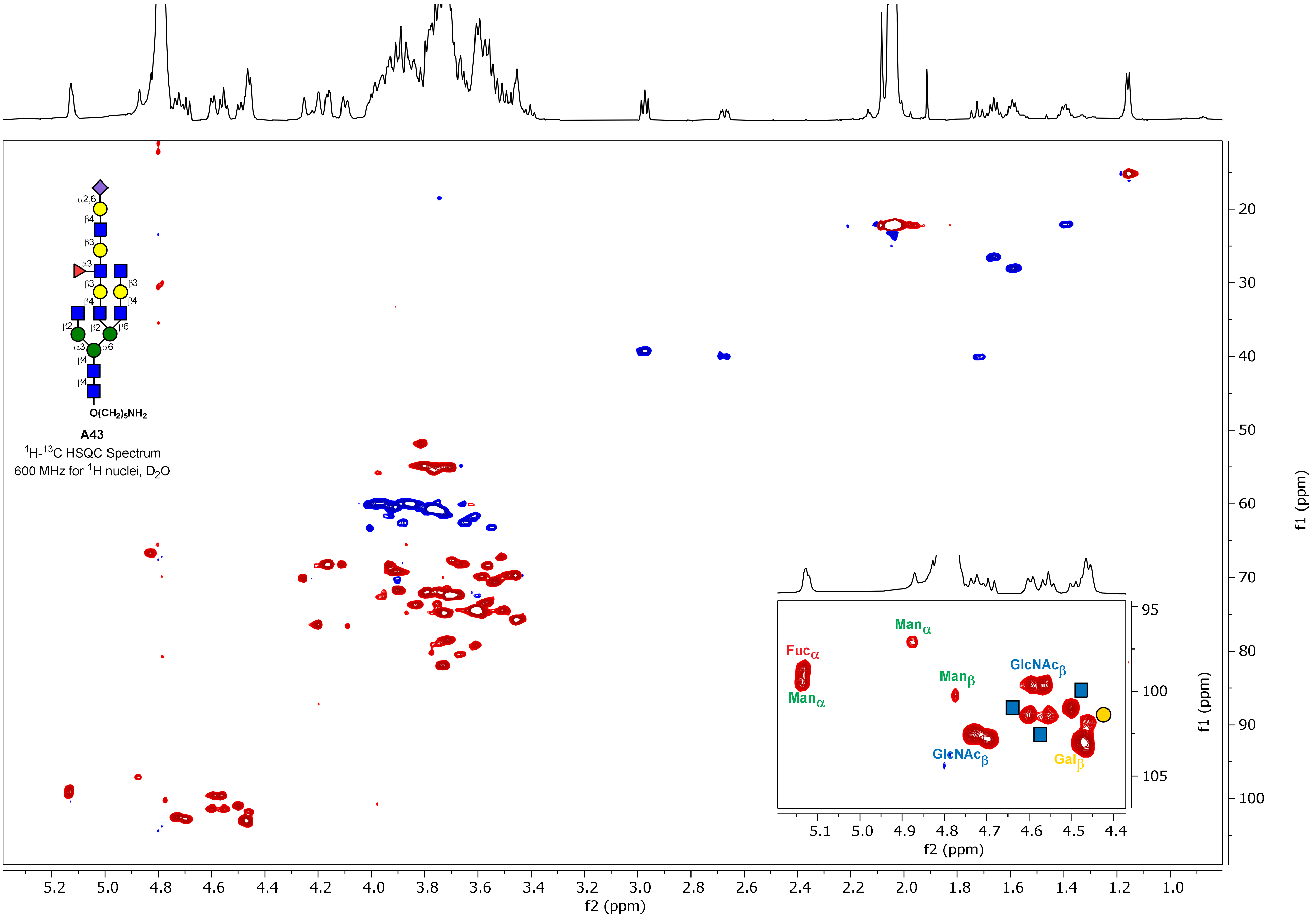

A43
^{1}H-^{13}C HSQC Spectrum
600 MHz for ^{1}H nuclei, D_2O
O(CH2)5NH2
Fucα
Manα
Manα
Manβ
GlcNAcβ
GlcNAcβ
Galβ
f2 (ppm)
f1 (ppm)

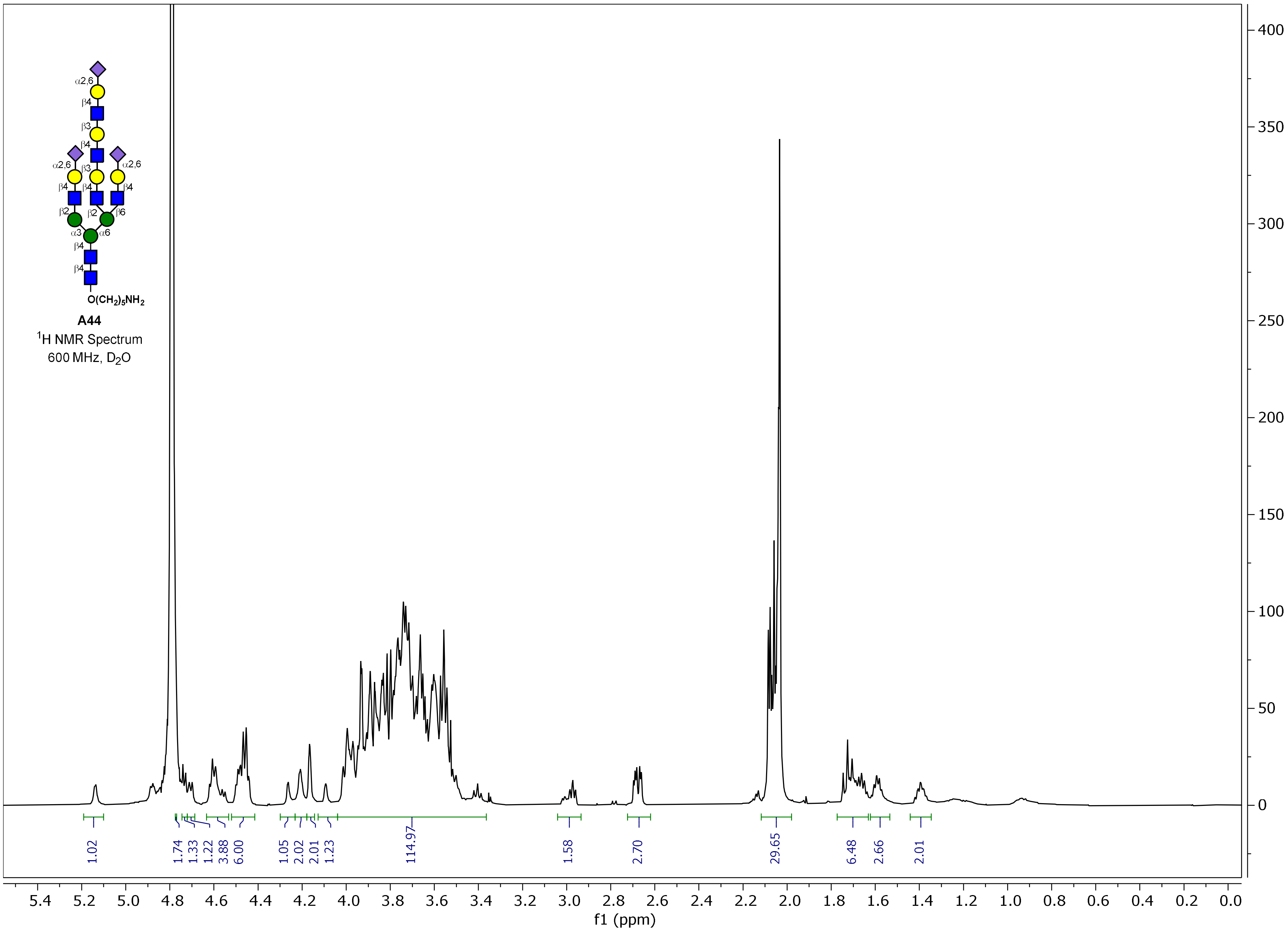
O(CH2)5NH2
A44
1H NMR Spectrum
600 MHz, D2O
1.02
1.74
1.33
1.22
3.88
6.00
1.05
2.02
2.01
1.23
114.97
1.58
2.70
29.65
6.48
2.66
2.01
f1 (ppm)

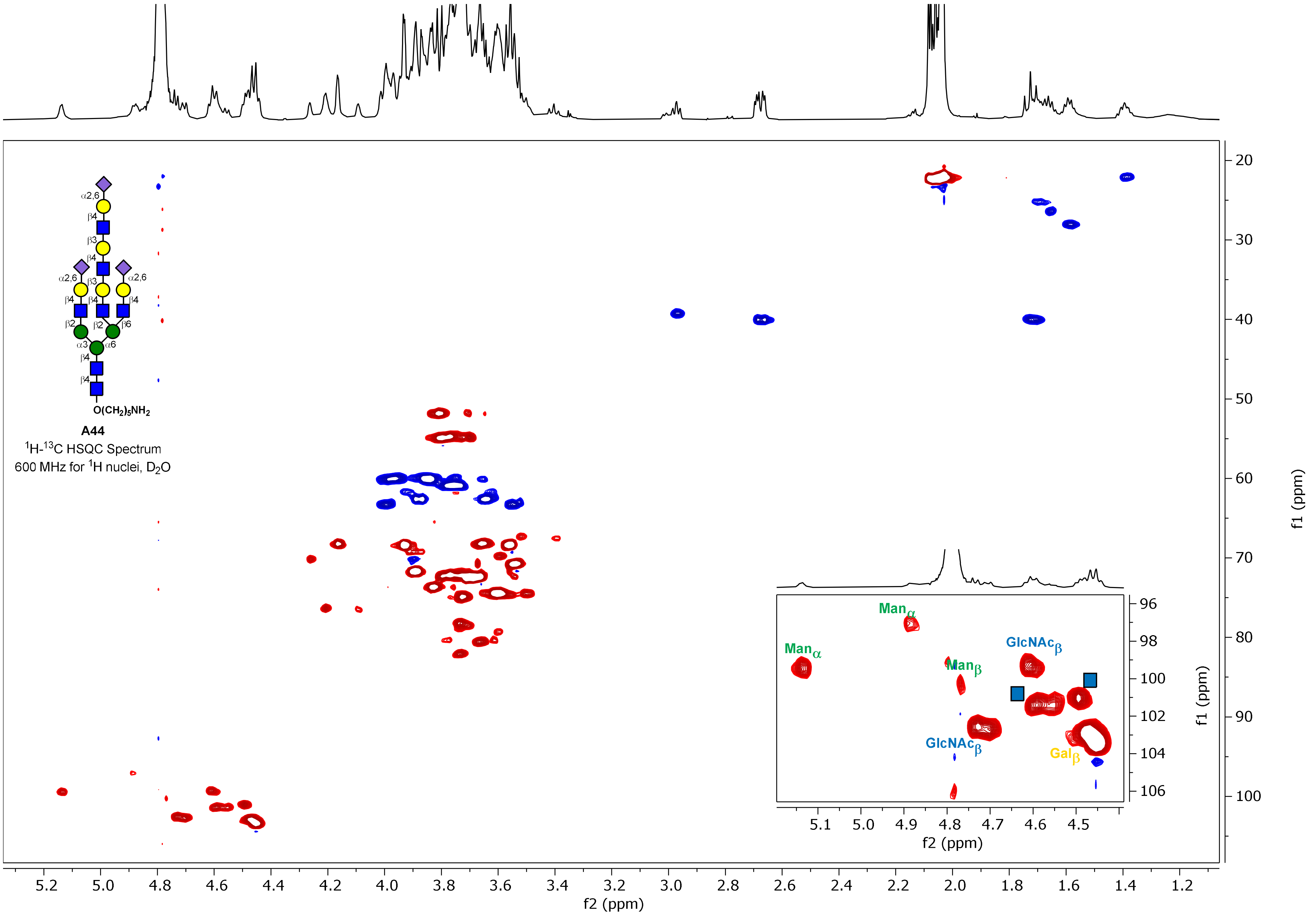

O(CH2)5NH2
A44
1H-13C HSQC Spectrum
600 MHz for 1H nuclei, D2O
Manα
Manα
Manβ
GlcNAcβ
GlcNAcβ
Galβ
f2 (ppm)
f1 (ppm)

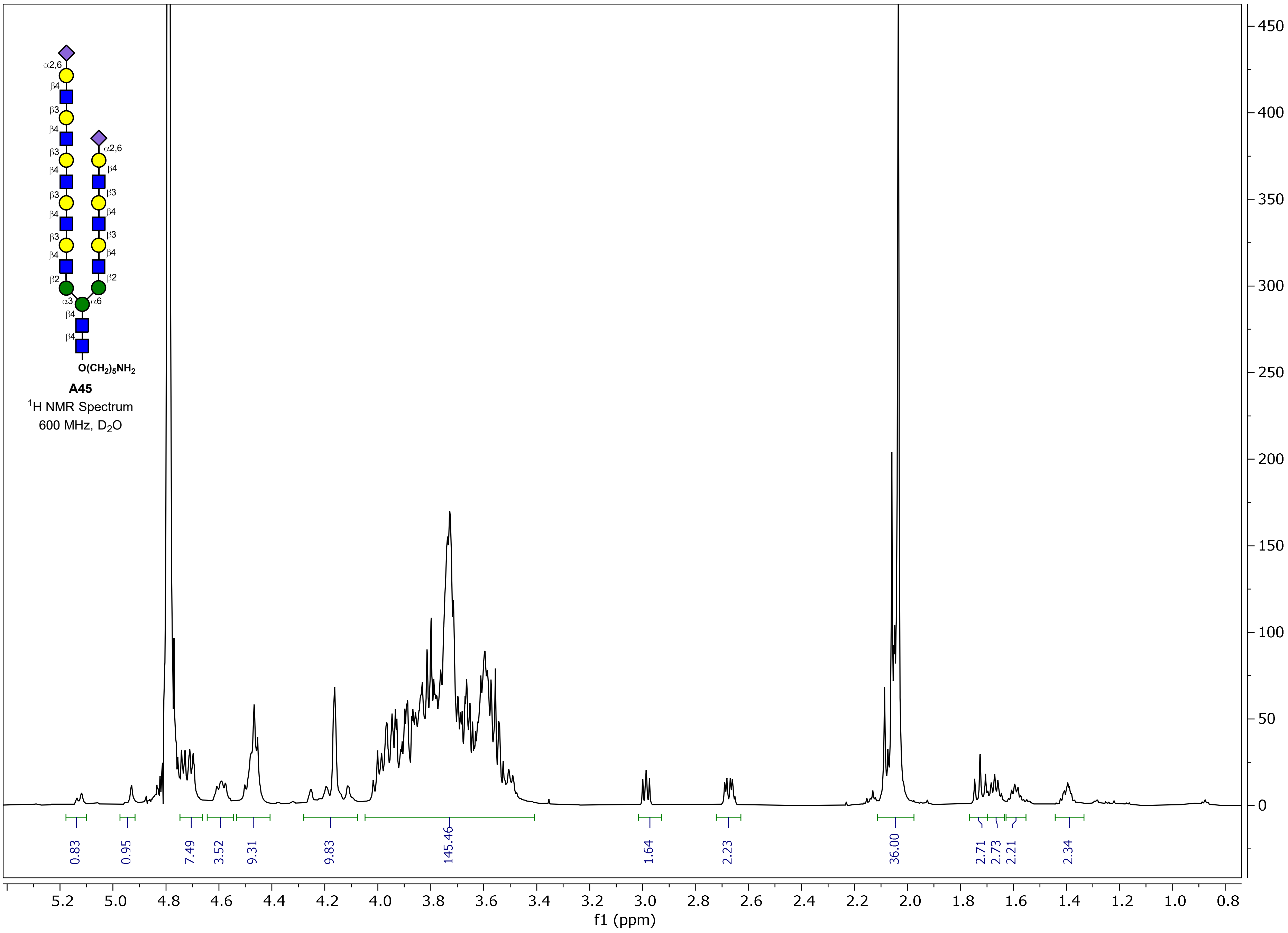
O(CH2)5NH2
A45
1H NMR Spectrum
600 MHz, D2O
f1 (ppm)

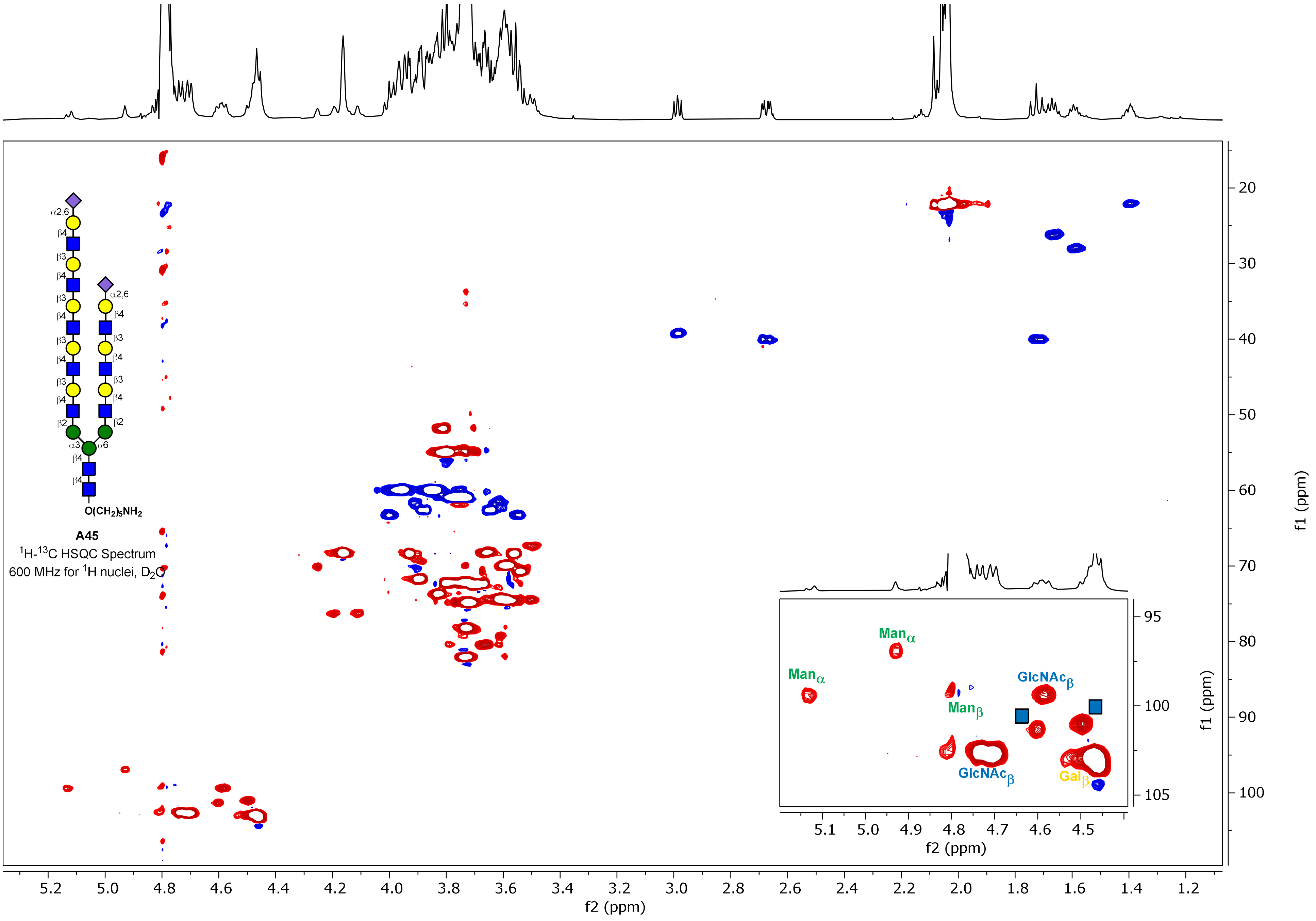
A45
1H-13C HSQC Spectrum
600 MHz for 1H nuclei, D2O
O(CH2)5NH2
Manα
Manα
GlcNAcβ
Manβ
GlcNAcβ
Galβ
f2 (ppm)
f1 (ppm)

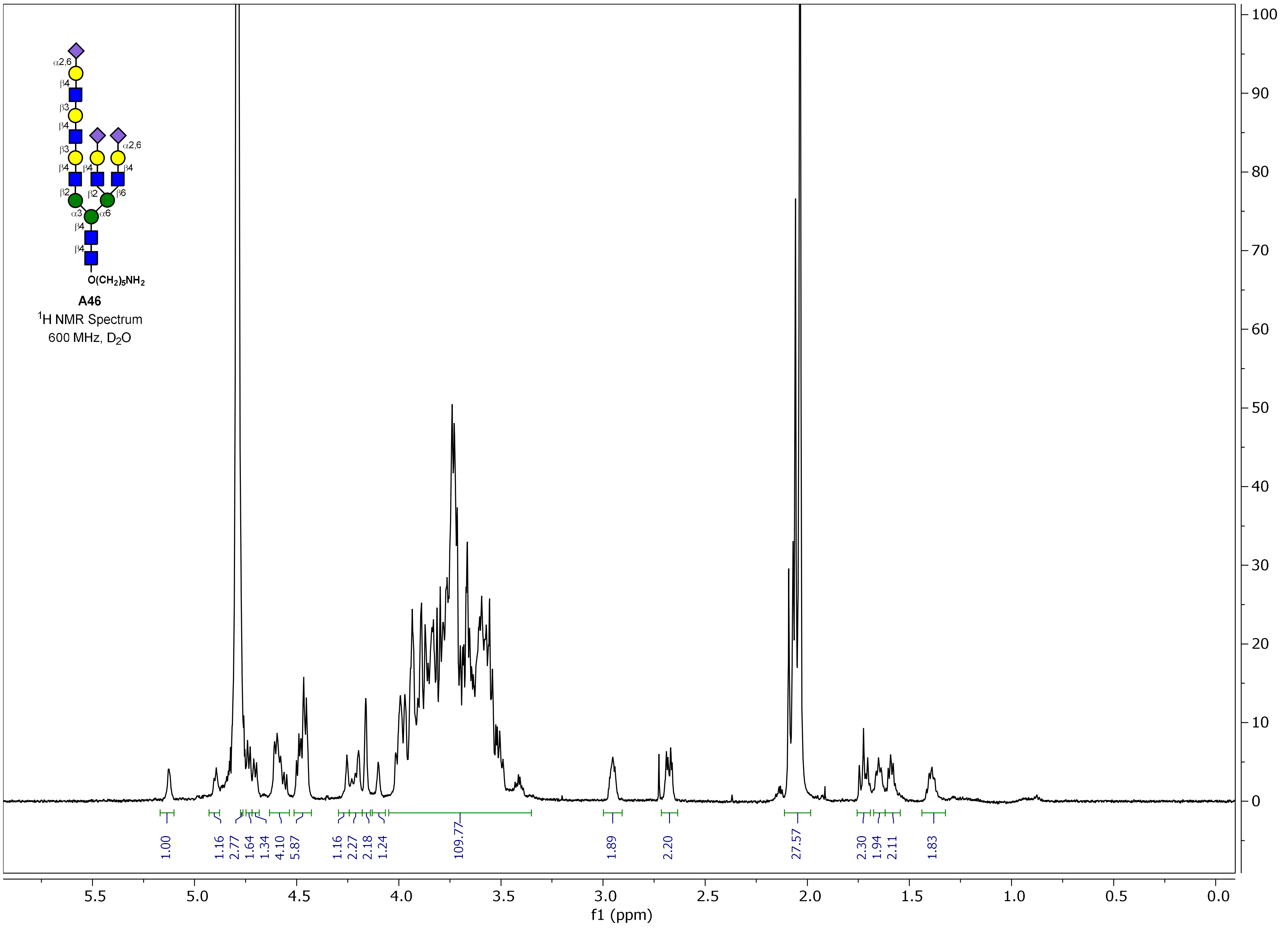
α2,6
β4
β3
β4
β3
β4
β2
α3
α6
β6
O(CH2)5NH2
A46
1H NMR Spectrum
600 MHz, D2O
1.00
1.16
2.77
1.64
1.34
4.10
5.87
1.16
2.27
2.18
1.24
109.77
1.89
2.20
27.57
2.30
1.94
2.11
1.83
f1 (ppm)

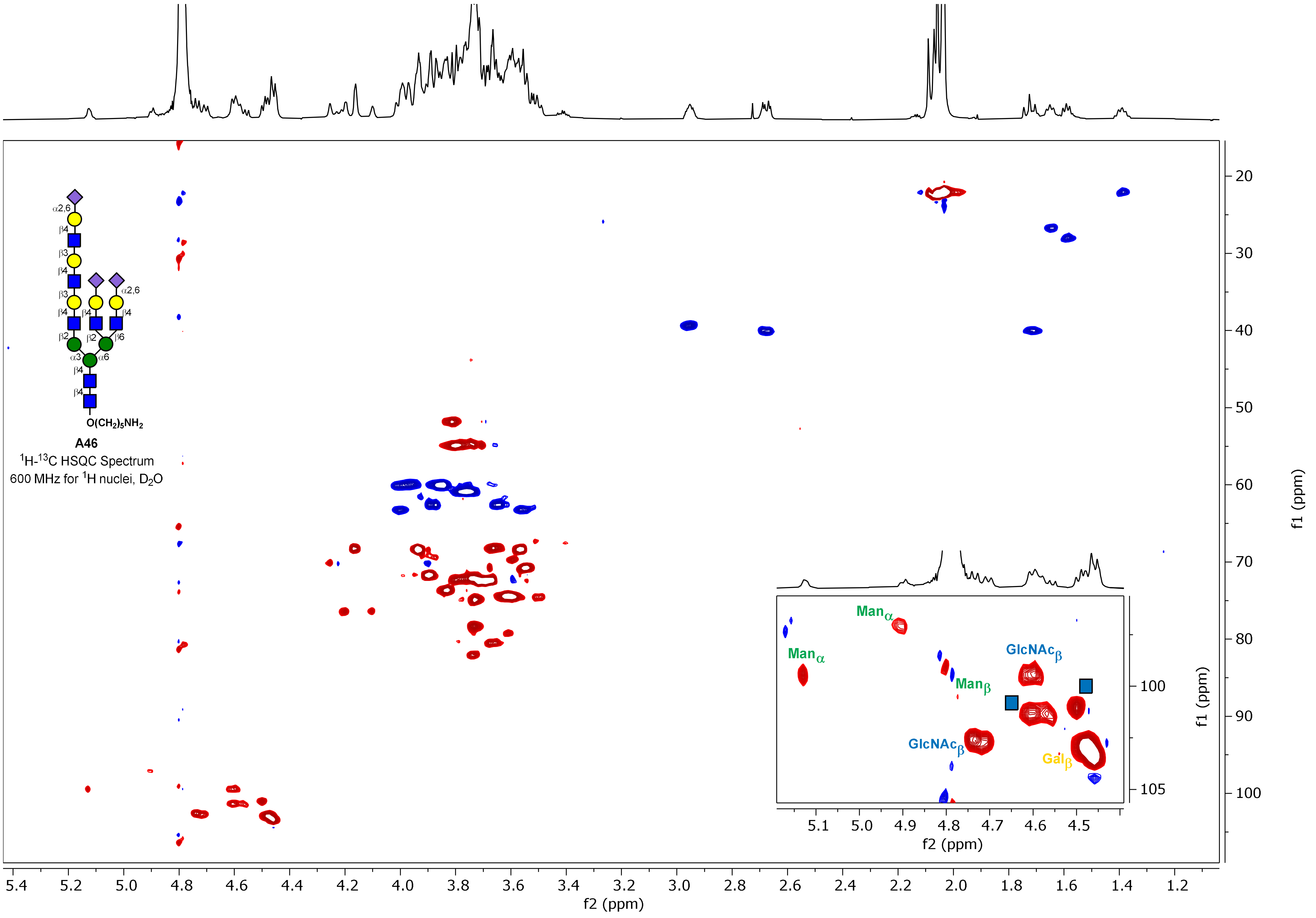
α2,6
β4
β3
β4
β3
β4
β2
α3
α6
β6
O(CH2)5NH2
A46
1H-13C HSQC Spectrum
600 MHz for 1H nuclei, D2O
Manα
Manα
Manβ
GlcNAcβ
GlcNAcβ
Galβ
f2 (ppm)
f1 (ppm)

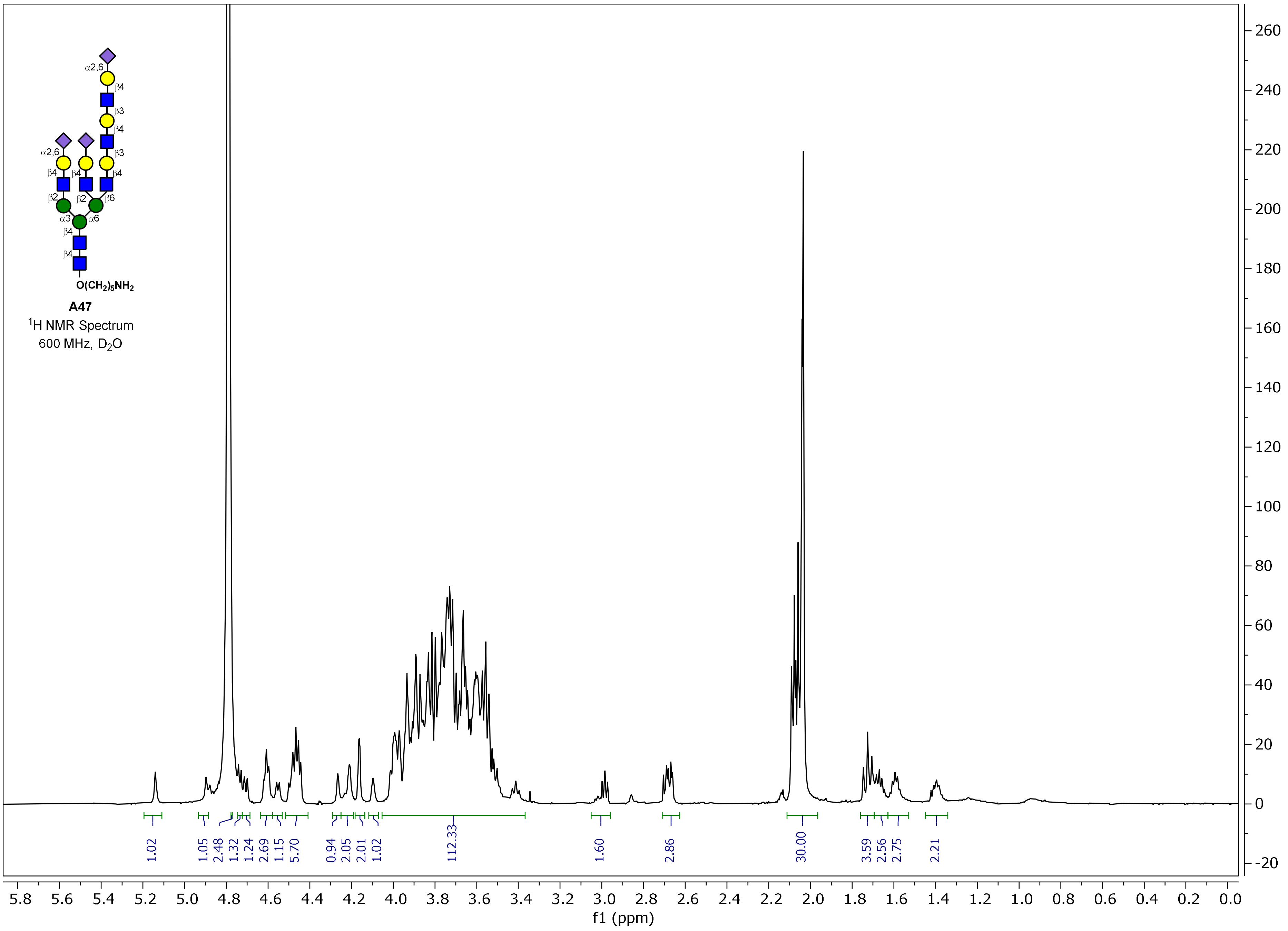
O(CH2)5NH2
A47
1H NMR Spectrum
600 MHz, D2O
f1 (ppm)

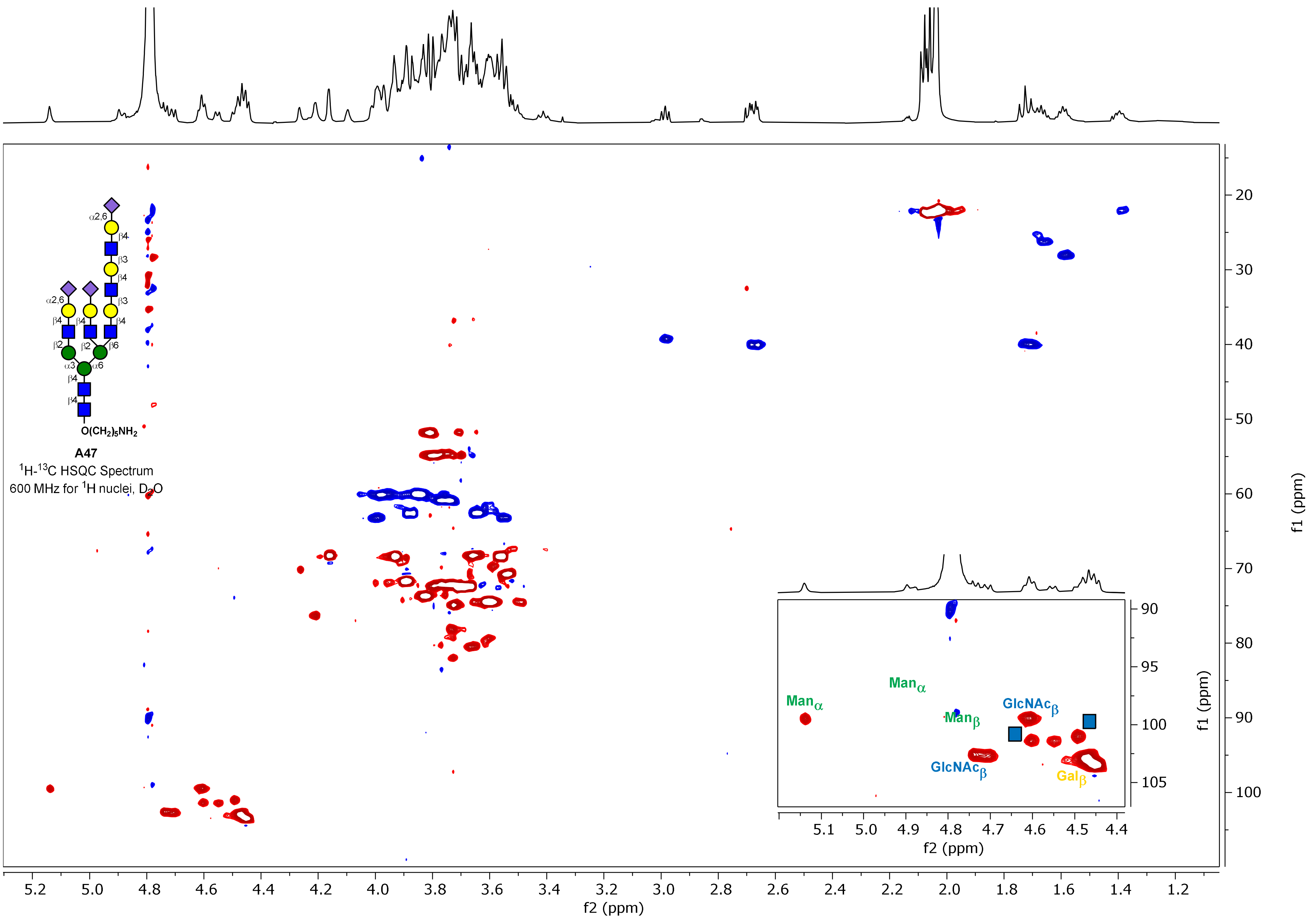
A47
^{1}H-^{13}C HSQC Spectrum
600 MHz for ^{1}H nuclei, D$_2$O
O(CH$_2$)$_5$NH$_2$
Man$_\alpha$
Man$_\alpha$
Man$_\beta$
GlcNAc$_\beta$
GlcNAc$_\beta$
Gal$_\beta$
f2 (ppm)
f1 (ppm)

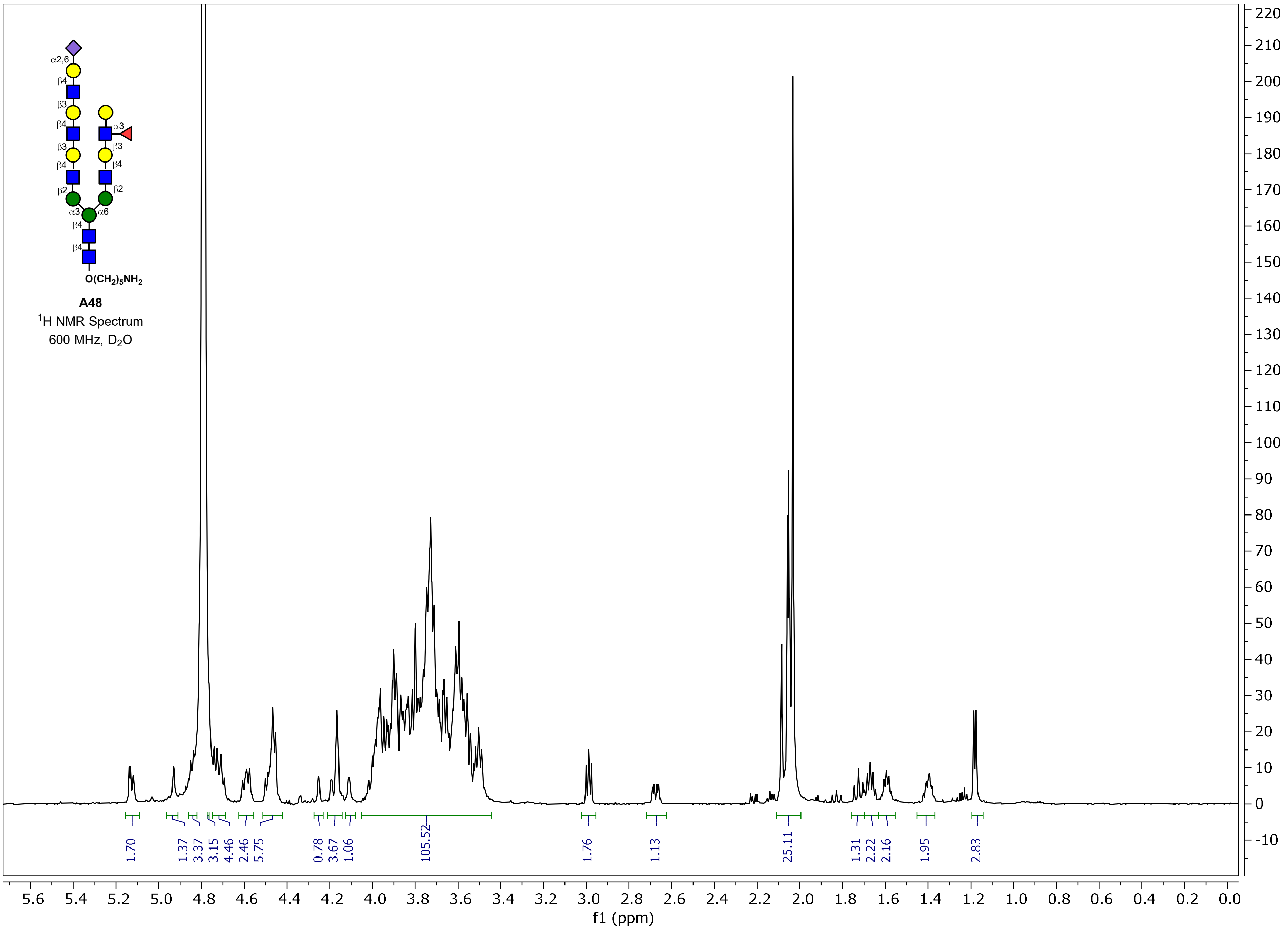

α2,6
β4
β3
β4
β3
β4
β2
α3
α6
β4
β4
α3
β3
β4
β2
$O(CH_2)_5NH_2$
A48
^{1}H NMR Spectrum
600 MHz, D_2O
1.70
1.37
3.37
3.15
4.46
2.46
5.75
0.78
3.67
1.06
105.52
1.76
1.13
25.11
1.31
2.22
2.16
1.95
2.83
f1 (ppm)

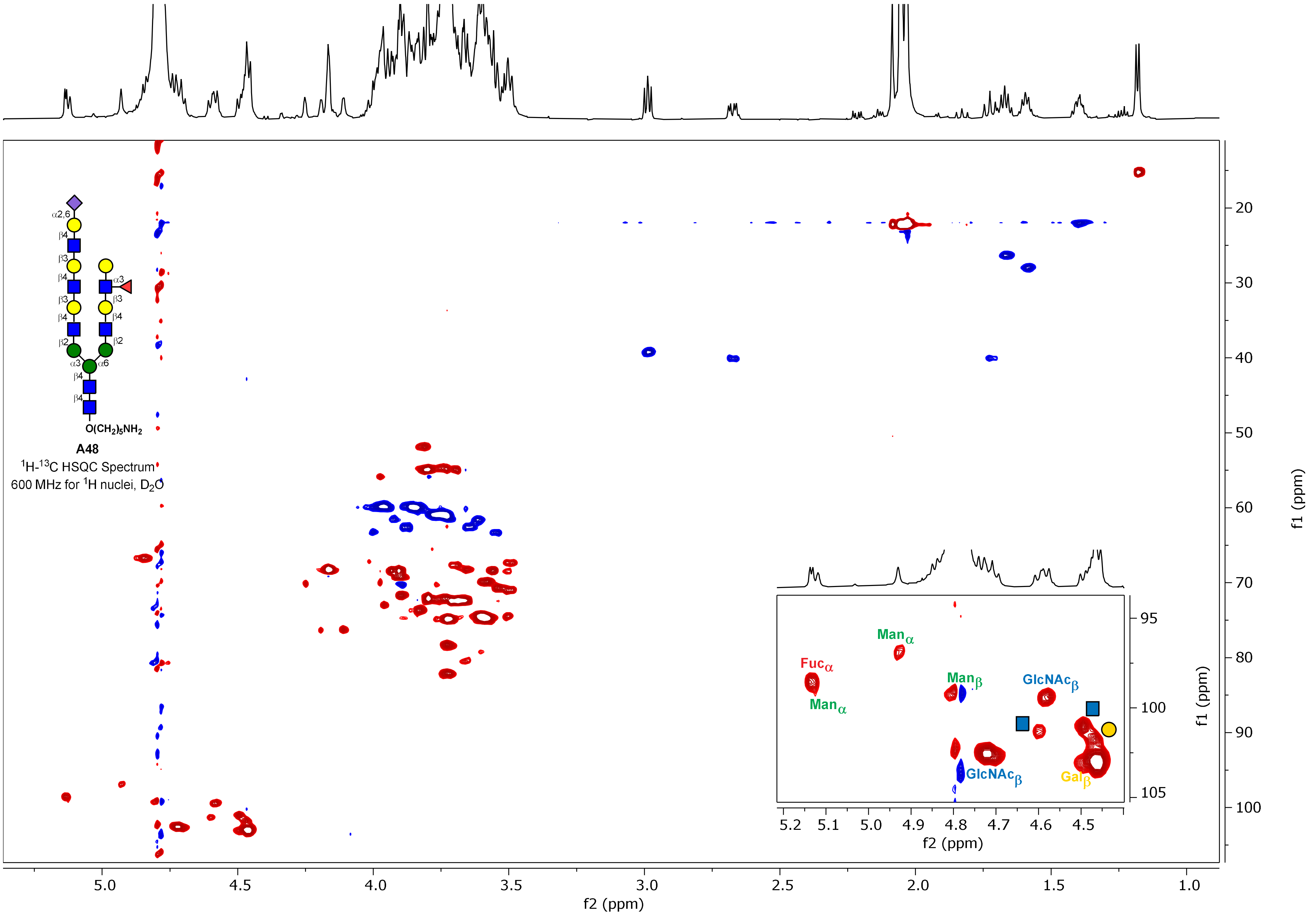

α2,6
β4
β3
β4
α3
β3
β4
β2
α6
O(CH2)5NH2
A48
1H-13C HSQC Spectrum
600 MHz for 1H nuclei, D2O
Manα
Fucα
Manα
Manβ
GlcNAcβ
GlcNAcβ
Galβ
f2 (ppm)
f1 (ppm)

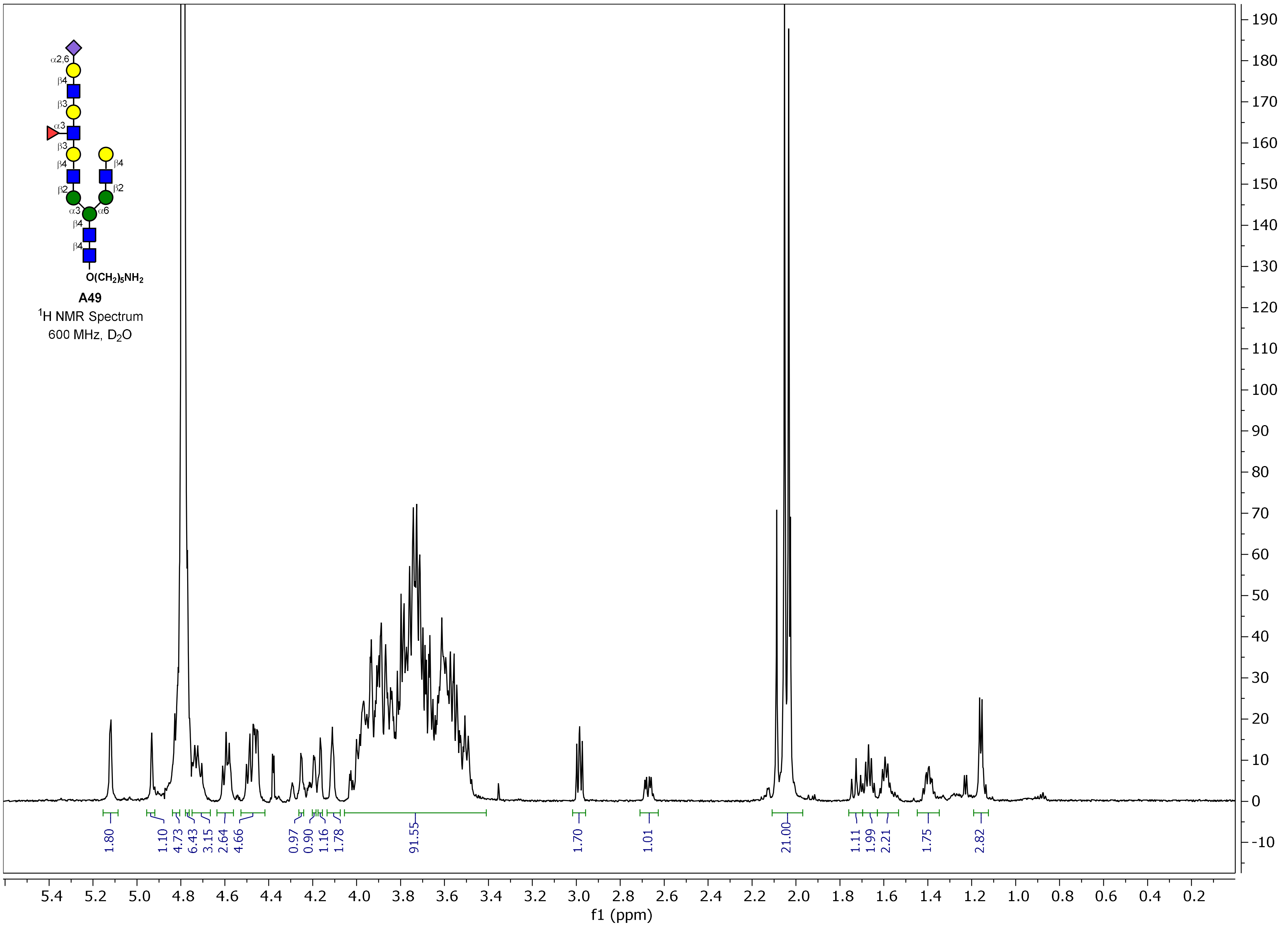

α2,6
β4
β3
α3
β3
β4
β4
β2
β2
α3
α6
β4
β4
O(CH2)5NH2
A49
1H NMR Spectrum
600 MHz, D2O
1.80
1.10
4.73
6.43
3.15
2.64
4.66
0.97
0.90
1.16
1.78
91.55
1.70
1.01
21.00
1.11
1.99
2.21
1.75
2.82
f1 (ppm)

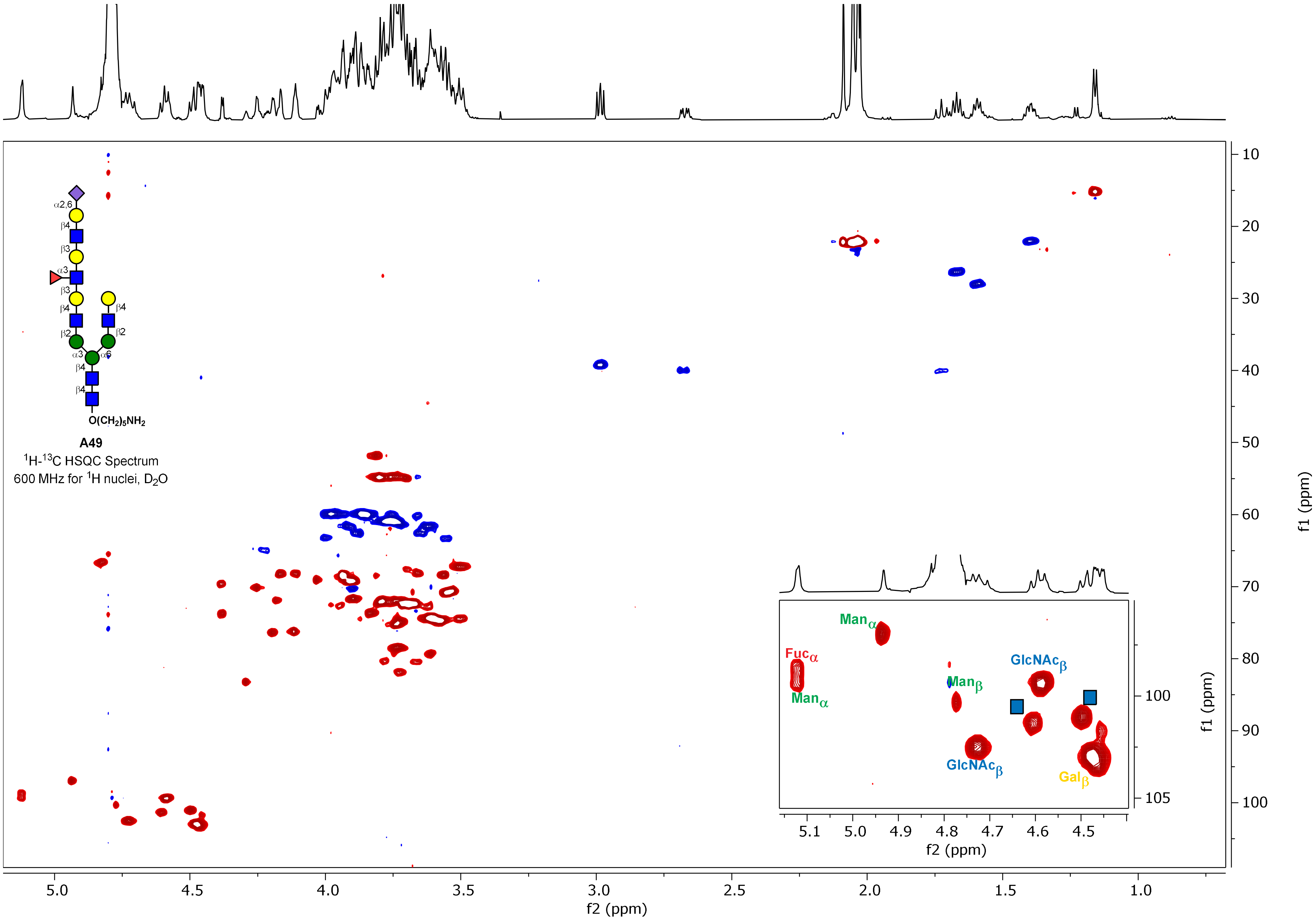
A49
^{1}H-^{13}C HSQC Spectrum
600 MHz for ^{1}H nuclei, D_2O
O(CH2)5NH2
Man α
Fuc α
Man α
GlcNAc β
Man β
GlcNAc β
Gal β
f2 (ppm)
f1 (ppm)

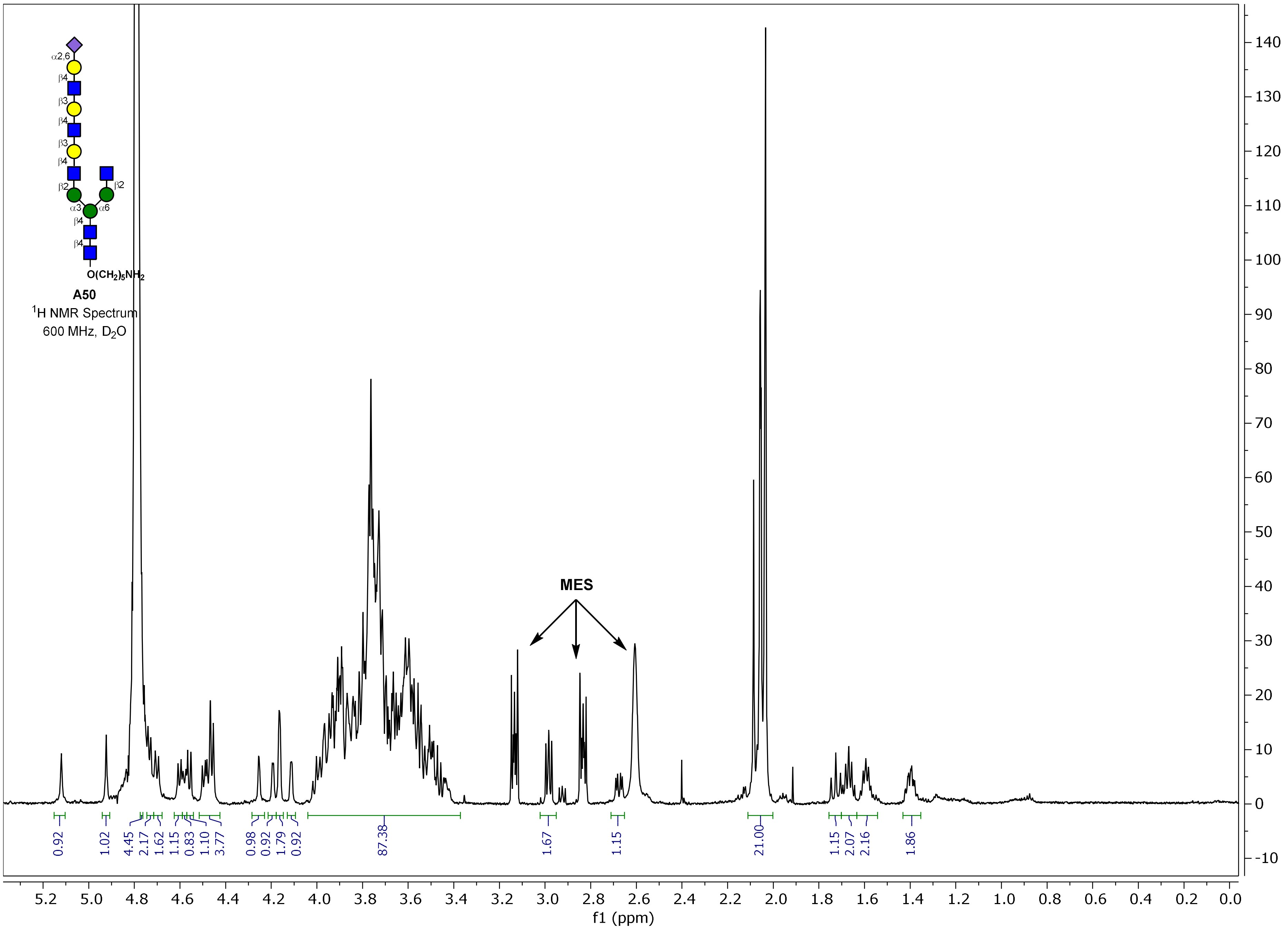

α2,6
β4
β3
β4
β3
β4
β2
β2
α3
α6
β4
β4
O(CH2)5NH2
A50
1H NMR Spectrum
600 MHz, D2O
MES
f1 (ppm)

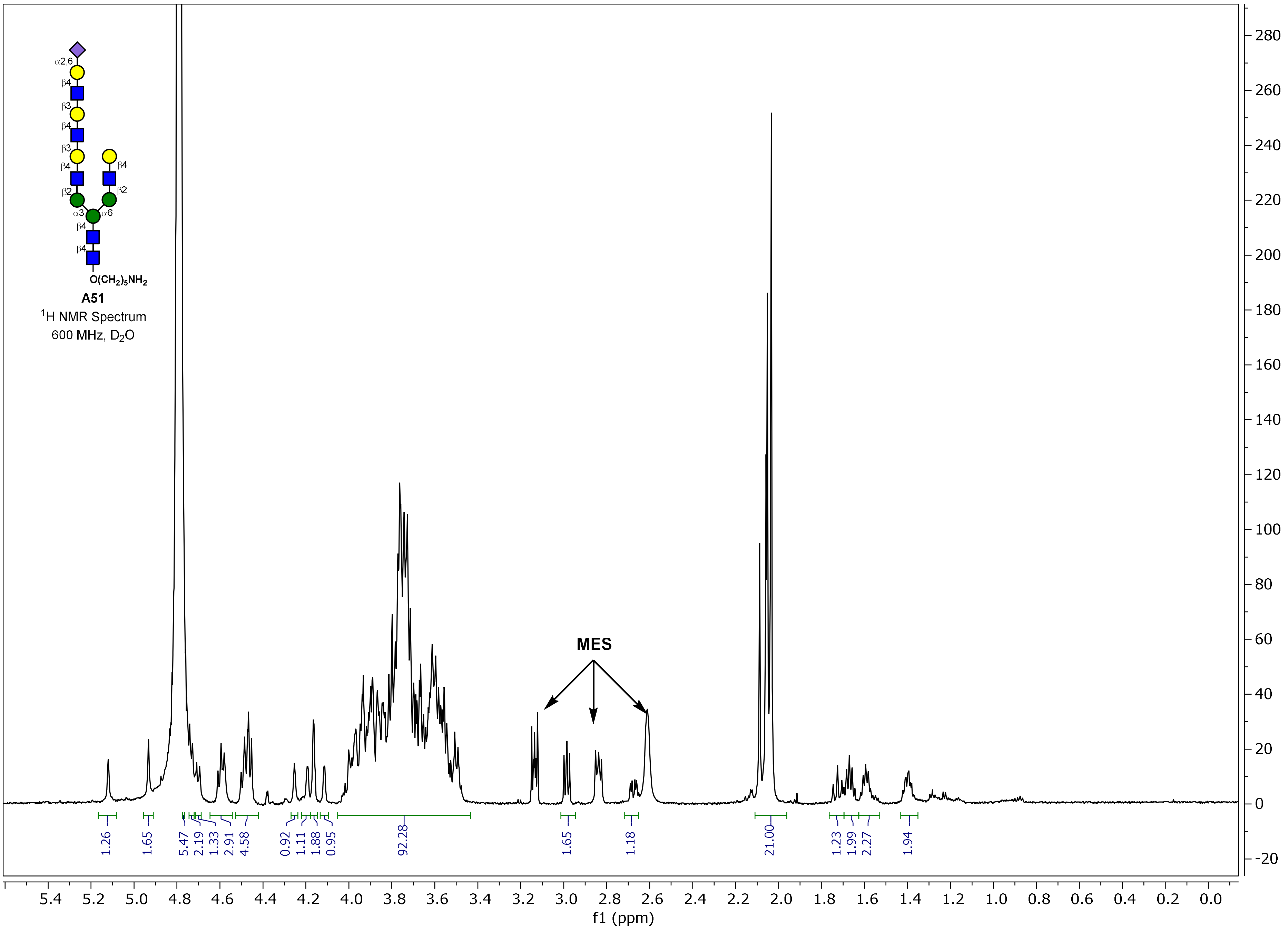
α2,6
β4
β3
β4
β3
β4
β4
β2
β2
α3
α6
β4
β4
O(CH2)5NH2
A51
1H NMR Spectrum
600 MHz, D2O
MES
1.26
1.65
5.47
2.19
1.33
2.91
4.58
0.92
1.11
1.88
0.95
92.28
1.65
1.18
21.00
1.23
1.99
2.27
1.94
5.4 5.2 5.0 4.8 4.6 4.4 4.2 4.0 3.8 3.6 3.4 3.2 3.0 2.8 2.6 2.4 2.2 2.0 1.8 1.6 1.4 1.2 1.0 0.8 0.6 0.4 0.2 0.0
f1 (ppm)
280
260
240
220
200
180
160
140
120
100
80
60
40
20
0
-20
